# Pathway analysis of genetic variants in folate‐mediated one‐carbon metabolism‐related genes and survival in a prospectively followed cohort of colorectal cancer patients

**DOI:** 10.1002/cam4.1407

**Published:** 2018-05-29

**Authors:** Jennifer Ose, Akke Botma, Yesilda Balavarca, Katharina Buck, Dominique Scherer, Nina Habermann, Jolantha Beyerle, Katrin Pfütze, Petra Seibold, Elisabeth J. Kap, Axel Benner, Lina Jansen, Katja Butterbach, Michael Hoffmeister, Hermann Brenner, Alexis Ulrich, Martin Schneider, Jenny Chang‐Claude, Barbara Burwinkel, Cornelia M. Ulrich

**Affiliations:** ^1^ Department of Population Health Sciences Huntsman Cancer Institute University of Utah Salt Lake City Utah; ^2^ Division of Preventive Oncology National Center for Tumor Diseases and German Cancer Research Center Heidelberg Germany; ^3^ Institute of Medical Biometry and Informatics University of Heidelberg Heidelberg Germany; ^4^ Genome Biology, European Molecular Biology Laboratory German Cancer Research Center and National Center for Tumor Diseases Heidelberg Germany; ^5^ Division of Molecular Epidemiology German Cancer Research Center Heidelberg Germany; ^6^ Division Molecular Biology of Breast Cancer Department of Gynecology and Obstetrics University of Heidelberg Heidelberg Germany; ^7^ Division of Cancer Epidemiology German Cancer Research Center Heidelberg Germany; ^8^ Division of Biostatistics German Cancer Research Center Heidelberg Germany; ^9^ Division of Clinical Epidemiology and Aging Research German Cancer Research Center Heidelberg Germany; ^10^ Clinic for General, Visceral and Transplantation Surgery Heidelberg University Hospital Heidelberg Germany

**Keywords:** Colorectal cancer, one‐carbon metabolism, polymorphisms, survival

## Abstract

Folate‐mediated one‐carbon metabolism (FOCM) is a key pathway essential for nucleotide synthesis, DNA methylation, and repair. This pathway is a critical target for 5‐fluorouracil (5‐FU), which is predominantly used for colorectal cancer (CRC) treatment. A comprehensive assessment of polymorphisms in FOCM‐related genes and their association with prognosis has not yet been performed. Within 1,739 CRC cases aged ≥30 years diagnosed from 2003 to 2007 (DACHS study), we investigated 397 single nucleotide polymorphisms (SNPs) and 50 candidates in 48 FOCM‐related genes for associations with overall‐ (OS) and disease‐free survival (DFS) using multiple Cox regression (adjusted for age, sex, stage, grade, BMI, and alcohol). We investigated effect modification by 5‐FU‐based chemotherapy and assessed pathway‐specific effects. Correction for multiple testing was performed using false discovery rates (FDR). After a median follow‐up time of 5.0 years, 585 patients were deceased. For one candidate SNP in *MTHFR* and two in *TYMS,* we observed significant inverse associations with OS (*MTHFR*: rs1801133, C677T: HR_het_ = 0.81, 95% CI: 0.67–0.97; *TYMS*: rs1001761: HR_het_ = 0.82, 95% CI: 0.68–0.99 and rs2847149: HR_het_ = 0.82, 95% CI: 0.68–0.99). After FDR correction, one polymorphism in paraoxonase 1 (*PON1*; rs3917538) was significantly associated with OS (HR_het_ = 1.28, 95% CI: 1.07–1.53; HR_hzv_ = 2.02, 95% CI:1.46–2.80; HR_logAdd_ = 1.31, p_FDR_ = 0.01). Adjusted pathway analyses showed significant associations for pyrimidine biosynthesis (*P *= 0.04) and fluorouracil drug metabolism (*P* < 0.01) with significant gene–chemotherapy interactions, including *PON1* rs3917538. This study supports the concept that FOCM‐related genes could be associated with CRC survival and may modify effects of 5‐FU‐based chemotherapy in genes in pyrimidine and fluorouracil metabolism, which are relevant targets for therapeutic response and prognosis in CRC. These results require confirmation in additional clinical studies.

## Introduction

Colorectal cancer (CRC) incidence and mortality rates have been decreasing during the past decade; yet it remains the second leading cause of cancer deaths in the United States 1.

**Figure 1:**
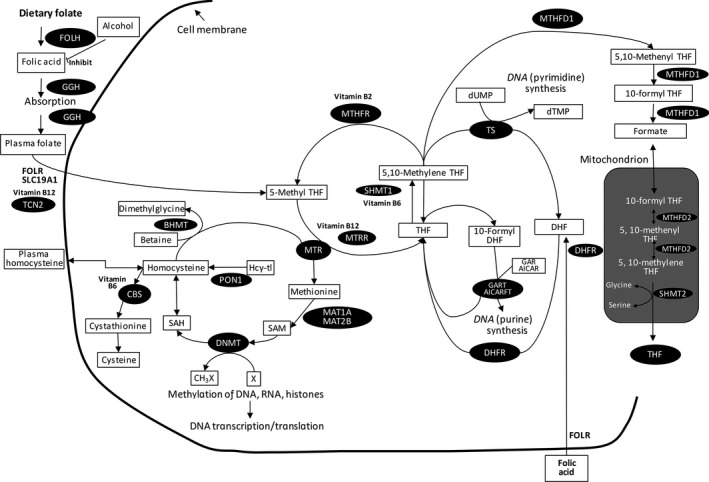
Overview of folate‐mediated one‐carbon metabolism, links to methylation reactions and nucleotide synthesis (by Cheng TY et al. 2015). THF = tetrahydrofolate; DHF = dihydrofolate; DNMT = DNA methyltransferases; GGH = gamma‐glutamyl‐hydrolase; RFC = reduced folate carrier; Hcy‐tl = homocysteine thiolactone; hFR = human folate receptor; MTHFR = 5,10‐methylenetetrahydrofolate reductase; DHFR = dihydrofolate reductase; GART = glycinamide ribonucleotide transformylase; AICARFT = 5‐amino‐imidazole‐4‐carboxamide ribonucleotide transformylase; AICAR = 5‐aminoimidazole‐4‐carboxamide ribonucleotide; GAR = glycinamide ribonucleotide; SAM (AdoMet) = S‐adenosylmethionine; SAH (AdoHcy) = S‐adenosylhomocysteine; dUMP = deoxyuridine monophosphate; dTMP = deoxythymidine monophosphate; MS = methionine synthase; TS = thymidylate synthase; DNMT = DNA methyltransferases; MTRR = methionine synthase reductase; X = a variety of substrates for methylation

The antimetabolite 5‐fluorouracil (5‐FU) is the most frequently used chemotherapy in CRC treatment, targeting thymidylate synthase (*TYMS*) in folate‐mediated one‐carbon metabolism (FOCM) 2. 5‐FU directly inhibits purine synthesis by inhibiting *TYMS,* resulting in decreased DNA replication and repair 3. There are large interindividual differences, however, in the effectiveness and tolerability of 5‐FU, which may be due to genetic variation in FOCM‐related genes 4, 5, 6. An overview of folate‐mediated one‐carbon metabolism and the genes and metabolites involved in that pathway is presented in Fig. 1 6.

Prior studies investigating FOCM‐related genes and response to 5‐FU‐based chemotherapy in CRC patients were largely limited to *TYMS* and *MTHFR* variants yielding inconsistent results 7, 8, 9, 10, 11, 12, 13, 14, 15, 16, 17, 18, 19. While earlier data linked low expression of *TYMS* to worse response to 5‐FU‐based chemotherapy 17, subsequent studies have related low *TYMS* expression to improved response rates in patients with CRC 13, 18. *In vitro* data have linked 5‐FU sensitivity to *MTHFR* A1298C but not *MTHFR* C677T. Yet, clinical studies 9, 10, 11, 16, 19 associated 5‐FU sensitivity with *MTHFR* C677T but not with *MTHFR* A1298C. Understanding the interaction of *TYMS* and *MTHFR* genotypes with 5‐FU‐based chemotherapy could help identify patients who are more likely to respond to 5‐FU‐based chemotherapy, using personalized information to tailor chemotherapy.

To date, no comprehensive assessment of genetic variability in FOCM and association with CRC prognosis has been performed. Therefore, we aimed to assess whether single genetic variants as well as *a priori* defined pathways in FOCM (e.g., folate, pyrimidine synthesis, and fluorouracil pathway) were associated with overall‐ and disease‐free survival in patients from a large cohort of prospectively followed CRC patients. Finally, we evaluated interactions between genetic variants and 5‐FU‐based chemotherapy on overall‐ and disease‐free survival.

## Materials and Methods

### Study population

Our study population comprised 1,739 CRC patients who participated in an ongoing population‐based study “Darmkrebs: Chancen der Verhütung durch Screening” (DACHS) from Germany with long‐term follow‐up of patients 20. CRC patients with a primary, confirmed diagnosis of CRC, recruited from hospitals in the Rhein‐Neckar‐Odenwald region between 1 January 2003 and December 2007 were included. Patients were eligible if they were ≥30 years of age, resident in the study region, and able to complete an in‐person interview. Extensive information on sociodemographic characteristics, medical history and lifestyle factors was collected by trained interviewers using standardized questionnaires to collect information on established and suggested CRC risk and prognostic factors. A blood sample (>99% of the analyzed patients) or mouthwash for DNA extraction was taken. Clinical and histological data were extracted from medical and pathological records.

Follow‐up information on overall survival (OS) and disease‐free survival (DFS; defined as cancer recurrence) was collected 3 and 5 years after diagnosis. For all patients, vital status, date, and cause of death through the end of 2012 were ascertained via local population registries. Causes of death were verified by death certificates and coded based on ICD‐10 classifications. Information on therapy (at 3‐year follow‐up) and recurrences (at 3‐ and 5‐year follow‐up) was collected from clinical providers.

The study was approved by the ethics committee of the University of Heidelberg and conducted in agreement with the Helsinki Declaration. Written informed consent was provided from all participants for future use of research purposes.

### SNPs and functional non‐SNP polymorphisms

Altogether, 1,754 cases were genotyped. Based on functional data and literature, we selected 48 genes in the FOCM pathway: *AARS, ABCC4, ADH1B, ADH1C, BHMT, BHMT2, CBS, DHFR, DNMT1, DNMT3A, DNMT3B, DPYD, DPYS, DUT, EHMT1, EHMT2, FDXR, FOLH1, FOLR1, FPGS, GGH, GNMT, MAT1A, MAT2B, MTHFD1, MTHFD2, MTHFR, MTR, MTRR, NFKB1, NME1, NME2, PON1, PRDM2, RRM1, RRM2, SHMT1, SHMT2, SLC19A1, SLC29A1, TK1, TCN2, TYMP, TYMS, UMPH2, UMPK, UMPS, and UNG* (Table S1).

Polymorphisms that may affect protein levels and/or function are referred to as candidate polymorphisms. We selected 50 candidates (Table S2), including, among others, five polymorphisms in *TYMS* (rs1001761, rs10502289, rs503296 including two intronic variants (rs2847149, rs2853533)), five *TCN* candidates (rs1131603, rs1801198, rs4820889, rs9606756, and rs9621049), and two *MTHFR* candidates (rs1801131(C677T), rs1801133(A1298C)). Additionally, two non‐SNP variants in the *TYMS* gene were selected: an insertion/deletion (indel) of 6 bp at position 1494 (3′ UTR indel) and a variable number of tandem repeats of a 28‐bp sequence (*TSER*) 21.

We used a comprehensive approach to investigate 397 tagSNPs, which represent genetic variation across the selected genes (Table S1). The tagging approach exploits the linkage disequilibrium (LD; nonrandom correlation between SNPs) across the human genome by selecting tagSNPs, which serve as proxies for correlated SNPs in specific regions. Hence, a subset of SNPs may be sufficient to cover most of the genetic variation within a specific region. Data from the HapMap Project were used with a pairwise tagging approach applying *r* ^2^ = 0.80 as cutoff 22.

### Genotyping

Genomic DNA was extracted from EDTA blood or mouthwash samples using the FlexiGene DNA kit (Qiagen GmbH, Hilden, Germany) and quantified using Quant‐iT Pico Green dsDNA reagent kit (Invitrogen/Life Technologies, Darmstadt, Germany). Of 492 selected SNPs, 447 passed quality control after genotyping (for 45, success rate was below 95%, seven were not in Hardy–Weinberg equilibrium (HWE) and were selected to be genotyped on the customized GoldenGate assay (Illumina, San Diego, CA) 23. The iPLEX assay (Sequenom, Hamburg, Germany) for the MassArray system was used to genotype five SNPs that failed genotyping on the Illumina GoldenGate platform 24. Quality of genotyping was high with concordance of duplicates from Centre d'Etude du Polymorphisme Humain (Paris, France) and control samples above 98%. The two selected non‐SNP polymorphisms in the *TYMS* gene were genotyped using fragment analysis and single‐strand conformation polymorphism in the laboratory of Dr. Ulrich at the National Center for Tumor Diseases in Heidelberg, Germany.

### Statistical analysis

Differences in baseline characteristics between deceased and nondeceased patients and deviation from HWE were evaluated using chi‐square statistic. Imputation of environmental factors was made by single imputation due to only few missing values (<1%) except for grade (11%). Imputation of grade was compared with best case (all missings set to grade 1) and worst case (all missings set to grade 4), resulting in similar effect estimates (<3% difference).

Genotype imputation was performed using IMPUTE2 and the 1000 Genomes reference panel 25. Follow‐up time was calculated as time from diagnosis to the event of interest or censoring (date of last information).

Cox proportional hazards models were used to estimate hazard ratios (HR) for OS and DFS, and their 95% confidence intervals (CIs) were associated with the genetic variants. We considered three types of inheritance models: codominant (if each of the three genotypes as derived from the biallelic SNPs had frequencies ≥5), dominant (if at least one genotype as derived from the biallelic SNPs had frequencies <5), and log‐additive model.

Multivariable models were determined using a backward elimination procedure on the interaction terms based on Akaike's information criterion, forcing clinical variables and all main effects into the model. Analyses were adjusted for age (<60, 60–70, 70–80, 80+), sex, stage (I, II, III, IV), grade (1/2 vs. 3/4), BMI (<18.5, 18.5–25, 25–30, 30+ kg/m^2^) and alcohol intake (0, 0–6.1, 6.1–15.6, 15.6–32.6, >32.6 g/day).

We investigated effect modification by 5‐FU‐based chemotherapy in the associations of all investigated SNPs with OS and DFS. The interaction terms between SNPs and 5‐FU‐based chemotherapy were derived from a comparison of the model with and without interaction terms using the likelihood ratio test. For pathway analysis, polymorphisms in high LD (*r* ^2^ > 0.5) within each gene were summarized to discard redundant information. Remaining polymorphisms were standardized 26, and genewise principal component analysis was applied explaining 95% of the variance in the data. The SNPs and the two non‐SNP polymorphisms were entered into a multivariable global test using Cox regression modeling 27. The Molecular Signatures Database v3.1 of the Broad Institute was used to identify subpathways (i.e., gene sets) searching for one‐carbon, folate, and 5‐FU‐based chemotherapy. YY KEGG and YY GO pathways were extracted 28 (Table S3). Candidate variants were not adjusted for multiple testing as they were selected based on functional data and independent hypotheses. TagSNPs or pathway analyses were corrected using the false discovery rates (FDR) for main effects and interaction tests.

All statistical analyses were two‐sided (significance level: *P* < 0.05) and performed using SAS (v9.4, SAS Institute, Cary, NC) and R (v3.1, R Foundation for Statistical Computing, Vienna, Austria).

## Results

During a median follow‐up time of 5.0 years (range: 0.01–6.4 years), 585 of the 1,739 patients died, 420 due to CRC. Patients were on average 68.2 ± 10.4 years old at diagnosis (Table 1). Deceased patients were more likely to be older, have a higher tumor stage and grade compared to nondeceased patients, and also more likely to have received adjuvant chemotherapy (especially 5‐FU and folic acid therapy). Almost two thirds of the patients had a colon carcinoma compared to one third of patients diagnosed with rectal cancer.

**Table 1:** Selected characteristics of deceased and nondeceased patients.^a^

	Deceased (*n *= 585)	Nondeceased (*n *= 1,154)	*P*‐Value
Age (%)			
<60	84 (14.4)	249 (21.6)	**<0.01**
60–70	173 (29.6)	428 (37.1)	
70–80	189 (32.3)	363 (31.5)	
80+	139 (23.8)	114 (9.9)	
Sex (%)			
Female	259 (44.3)	465 (40.3)	0.11
Male	326 (55.7)	689 (59.7)	
Site (%)			
Colon	359 (61.4)	700 (60.7)	0.77
Rectum	226 (38.6)	454 (39.3)	
CRC first‐degree family history (%)			
No	502 (85.8)	979 (84.8)	0.59
Yes	83 (14.2)	175 (15.2)	
Stage (%)			
I	61 (10.4)	366 (31.7)	**<0.01**
II	115 (19.7)	413 (35.8)	
III	201 (34.4)	344 (29.8)	
IV	208 (35.6)	31 (2.7)	
Grade (%)			
1,2	369 (63.1)	877 (76.0)	**<0.01**
3,4	216 (36.9)	277 (24.0)	
Smoking (%)			
Never	304 (52.0)	533 (46.2)	0.08
Former	200 (34.2)	442 (38.3)	
Current	81 (13.8)	179 (15.5)	
BMI [kg/m^2^] (%)			
<18	22 (3.8)	18 (1.6)	**<0.01**
18–25	235 (40.2)	384 (33.3)	
25–30	231 (39.5)	513 (44.5)	
>30	97 (16.6)	239 (20.7)	
Alcohol intake, [g/day] (%)			
0	213 (36.4)	310 (26.9)	**<0.01**
>0–6.1	94 (16.1)	217 (18.8)	
>6.1–15.6	94 (16.1)	204 (17.7)	
>15.6–32.6	91 (15.6)	214 (18.5)	
>32.6	93 (15.9)	209 (18.1)	
Radiotherapy (%)			
No	468 (80)	942 (81.6)	**0.03**
Adjuvant	54 (9.2)	90 (7.8)	
Neo‐adjuvant	55 (9.4)	119 (10.3)	
Chemotherapy (%)			
No	230 (39.3)	697 (60.4)	**<0.01**
Adjuvant	312 (53.3)	379 (32.8)	
Neo‐adjuvant	33 (5.6)	75 (6.5)	
5‐FU‐based chemotherapy (%)			
No	40 (6.8)	52 (4.5)	0.91
Yes	283 (48.4)	359 (31.1)	
Not available	253 (43.2)	742 (64.3)	

a Percentages may not add up to 100.

*P*‐values in bold are statistically significant.

Selected results are presented in Tables 2 and 3. All results for the two non‐SNP variants in the TYMS gene are presented in Table S4a,b,c. The pathway analyses are presented in Table S5. Sensitivity analyses restricting the dataset to patients who received 5‐FU / 5‐FU + FA are presented in Table S6. The results of all survival analyses are presented in Table S7 and Table S8 (overall survival and disease‐free survival) and Table S9 and Table S10 (overall survival and disease‐free survival stratified by 5‐FU based chemotherapy). We have clearly defined hypotheses for each of the selected *n *= 50 candidate SNPs and consider the unadjusted *P*‐values as the relevant ones for this study. However, we have decided to present the FDR‐adjusted *P*‐values for the candidate SNPs as well. We observed significant inverse associations with OS for three candidate SNPs: one SNP in *MTHFR* (rs1801133, C677T: HR_het_ = 0.81, 95% CI: 0.67–0.97) and two candidates in *TYMS (*rs1001761: HR_het_ = 0.82, 95% CI: 0.68–0.99 and rs2847149: HR_het_ = 0.82, 95% CI: 0.68–0.99). A polymorphism in the paraoxonase 1 (*PON1*) gene (tag SNP rs3917538) was significantly associated with OS after FDR adjustment: HR_hzv_ = 2.02, 95% CI: 1.46–2.80; HR_het_ = 1.28, 95% CI: 1.07–1.53; HR_logAdd_ = 1.31, p_FDR_ < 0.01). Nominally significant associations were observed for two SNPs in *PON1* (rs3917527, rs757158) and one in *TYMS* (rs2244500). Significant inverse associations were observed for one candidate SNP in *EHMT2* with DFS: rs2736428 (HR_het_ = 0.80, 95% CI: 0.66–0.98). However, in the more recent HapMap database 22, this SNP is located on *SLC44A4*. Thus, we decided not to consider it further. Nominally significant associations were observed for 19 tagSNPs, but diminished after FDR adjustment.

**Table 2:** Associations between selected polymorphisms in FOCM‐related genes and overall‐ and disease‐free survival

	Gene	SNP	Genotype	HR(95%‐CI)^h^	*P* a	p_FDR_ b	p_Trend_ c	p_Trend‐FDR_ d	p_Genewide‐FDR_ e
Overall Survival	*PON1*	rs3917538	C/C	ref			<0.01	<0.01	<0.01
			C/T	1.18 (0.97–1.43)	0.09				
			T/T	2.02 (1.46–2.80)	**<0.01**				
			C/T or T/T^f^	1.28 (1.07–1.53)	**<0.01**	0.59			**0.04**
	*TYMS*	rs1001761^g^	C/C	ref			**0.04**	0.73	0.11
			C/T	0.84 (0.68–1.02)	0.08				
			T/T	0.77 (0.59–1.00)	0.05				
			C/T or T/T^f^	0.82 (0.68–0.99)	**0.04**	**0.59**			0.09
	*TYMS*	rs2847149^g^	G/G	ref			**0.04**	0.73	0.11
			G/A	0.84 (0.68–1.02)	0.08				
			A/A	0.77 (0.59–1.00)	**0.02**				
			G/A or A/A^f^	0.82 (0.68–0.99)	**0.04**	**0.59**			0.09
	*TYMS*	rs495139	C/C	ref			0.07	0.79	0.11
			C/G	1.48 (1.20–1.82)	**<0.01**				
			G/G	1.17 (0.89–1.53)	0.27				
			C/G or G/G^f^	1.39 (1.14–1.69)	**<0.01**	0.45			**<0.01**
Disease‐free survival	*MAT2B*	rs6882306	T/T	ref			**<0.01**	0.45	**0.01**
			T/C	1.31 (1.05–1.62)	**0.01**				
			C/C	1.91 (1.15–3.16)	**0.01**				
			T/C or C/C^f^	1.35 (1.10–1.66)	**<0.01**	0.84			**0.02**
	*UMPS*	rs1162	A/A	ref			**<0.01**	0.60	**0.02**
			A/G	1.22 (0.99–1.50)	0.06				
			G/G	1.57 (1.15–2.13)	**<0.01**				
			A/G or G/G^f^	1.29 (1.06–1.57)	**0.01**	0.84			0.07

a p:*P*‐value for log‐additive and dominant model.

b p_FDR_:FDR‐adjusted.

c p_Trend_:*P*‐value trend.

d p_Trend‐FDR_:FDR‐adjusted trend.

e p_Genewide‐FDR_:FDR‐adjusted genewide effect.

f Dominant model, (HR_het_).

g Candidate, FDR‐adjusted cutoff for significance of *P*‐value = 0.01.

h Adjusted for age, sex, stage, grade, BMI, alcohol intake.

*P*‐values in bold are statistically significant.

**Table 3:** Associations between selected polymorphisms in FOCM‐related genes and overall‐ and disease‐free survival stratified by 5‐FU‐based chemotherapy.^a^

	Gene	SNP	Genotype	No 5‐FU‐based chemotherapy			Received 5‐FU‐based chemotherapy			*P*‐values Interaction		
				Alive	Deceased	HR(95% CI)	Alive	Deceased	HR(95%‐CI)			
				*n*	*n*		*n*	*n*		p_trend_ b	p_trend‐FDR_ c	p_FDR‐Genewide_ d
Overall survival	*PON1*	rs3917538	C/C	37	20	ref	231	132	1.21 (0.71–2.06)	0.36	0.59	**0.04**
			C/T	18	12	1.55 (0.69–3.47)	137	112	1.54 (0.89–2.64)			
			T/T	3	2	0.55 (0.07–4.30)	9	26	**2.97 (1.51**–**5.85)**			
			C/T or T/T^e^	21	14	1.30 (0.59–2.83)	146	138	1.65 (0.97–2.83)			
Disease‐free survival	*MAT2B*	rs12655857	G/G	24	21	ref	189	170	0.80 (0.47–1.35)	**0.01**	0.99	**0.04**
			G/T	31	9	**0.36 (0.15**–**0.88)**	128	119	0.91 (0.53–1.55)			
			T/T	5	2	0.22 (0.03–1.67)	24	17	0.78 (0.38–1.60)			
			G/T or T/T^e^	36	11	**0.33 (0.14**–**0.79)**	152	136	0.89 (0.52–1.52)	**0.01**	0.85	**0.03**
	*TCN2*	rs9621049^f^	C/C	54	25	ref	264	256	**1.65 (1.03**–**2.67)**	**0.02**	0.99	0.15
			C/T	6	6	3.33 (1.22–9.10)	73	47	1.41 (0.80–2.45)			
			T/T	0	1	0.63 (0.09–4.60)	4	3	1.05 (0.14–7.98)			
			C/T or T/T^e^	6	7	3.34 (1.22–9.11)	77	50	1.39 (0.80–2.43)	**0.02**	0.85	0.08

a Adjusted for age, sex, stage, grade, BMI, alcohol intake.

b p_trend_: *P*‐value for trend.

c p_trend‐FDR:_FDR‐adjusted trend.

d p_FDR‐Genewide:_FDR‐adjusted genewide effect.

e Dominant model (HR_het_).

f Candidate, FDR‐adjusted cutoff for significance of *P*‐value = 0.02.

*P*‐values in bold are statistically significant.

Selected results of effect modification analyses are presented in Table 3. We observed nominally significant interactions between 5‐FU‐based chemotherapy and 12 tagSNPs with OS (p_Inter_ < 0.05; data not shown). Significant interactions in relation to DFS were observed for three candidate SNPs, two on *TCN2* (rs1801198: p_Inter_ = 0.02 and rs9621049: p_Inter_ = 0.02), and one on *SHMT1* (rs9909104:p_Inter_ = 0.04). For DFS, we identified 17 nominally significant interactions between tagSNPs and 5‐FU‐based chemotherapy (*P* < 0.05). There was no significant association with OS or DFS (Table S4a) or effect modification for *TYMS* 3′ UTR 1494 del with OS or DFS (Table S4b,c). The *TSER* 2R/2R genotype was associated with a marginal nearly threefold increase in risk of death in patients receiving 5‐FU‐based chemotherapy (HR_hzv_ = 2.97, 95% CI: 0.96–9.26) compared to chemonaïve patients (HR_hzv_ = 2.17, 95% CI: 0.99–4.73p_Inter_ = 0.06; Table S4c). FDR‐adjusted analyses showed genewide effects on OS for *PON1* and *TYMS* (both p_FDRGene_ < 0.01) and significant interaction with 5‐FU‐based chemotherapy (e.g., *PON1* p_FDRGeneInter_ = 0.04 and *TYMS* p_FDRGeneInter_ = 0.01).

Genewide effects on DFS and significant interaction with 5‐FU‐based chemotherapy were observed for *MAT2B* (p_FDRGene_ = 0.01, p_FDRGeneInter_ = 0.04) and *UMPS* (p_FDRGene_ = 0.02, p_FDRGeneInter_ = 0.02; data not shown). 5‐FU‐based chemotherapy is often combined with other drugs that do not target the folate pathway. Yet, it is possible that drugs such as oxaliplatin or irinotecan may affect 5‐FU‐based–SNP interactions. To address this question, we performed sensitivity analyses restricting the dataset to patients who received 5‐FU / 5‐FU + FA (Table S6). Due to the limited statistical power, results need to be interpreted with caution. For the dominant genotype of rs3917538 (*PON1*), we have observed similar associations with overall survival between patients receiving 5‐FU‐based chemotherapy compared to patients who have received 5‐FU / 5‐FU + FA: (5‐FU‐based chemotherapy: HR_hzv_ = 2.97, 95% CI: 0.96–9.26; 5‐FU / 5‐FU + FA: HR_hzv_ = 2.84, 95% CI: 1.31–6.16). The same was observed for the associations for rs12655857 (MAT2B) and DFS. For rs9621049 (*TCN2*) restricting the dataset to patients who have received 5‐FU / 5‐FU + FA revealed a statistically significant reduced risk of death among patients with the CT/TT genotype: (5‐FU / 5‐FU + FA: HR_het/hzv_ = 0.55, 95% CI: 0.32–0.96)p_interaction_ = 0.03.

In global pathway analyses, we observed global significance for OS in the “fluorouracil” (*P* = 0.01) and pyrimidine pathway (*P* = 0.04), but not in “folate,” “methionine,” or “purine” pathways (Table S5).

## Discussion

Our study provides, for the first time, a comprehensive pathway analysis of genetic variants in FOCM and their role in overall‐ and disease‐free survival in patients with CRC. Data from our interaction analyses support the importance of genetic variants as modifiers of response to 5‐FU‐based chemotherapy and the prognostic impact in patients with CRC. Pathway effects were observed for genes in pyrimidine biosynthesis and fluorouracil drug metabolism, which are relevant targets for therapeutic response and CRC prognosis.

Prior studies primarily investigated *TYMS* and *MTHFR* candidate gene variants; however, with inconsistent and limited results in that, only a few FOCM‐related genes were evaluated 7, 8, 9, 10, 11, 12, 13, 14, 15, 16, 17, 18, 19. In agreement with prior research, we have shown an inverse association of rs1801133 (*MTHFR*, C677T) 29, 30, 31, rs1001761, and rs2847149^12^, ^17^, ^18^ with OS in CRC patients. Numerous tagSNPs in FOCM‐related genes were nominally associated with OS (e.g., *DPYD, DPYS*). After FDR correction, only *PON1* (rs3917538; intronic, C/T) remained significant. Notably, prior studies have shown increased serum *PON1* activity in patients with CRC compared to healthy controls 32, 33. Genetic variation in *PON1* has also been linked to prostate 34 and ovarian cancer 35. There are no prior studies on rs3917538. This SNP, however, is highly correlated with rs662 (LD *r* ^2^ = 0.70), a missense mutation within 450‐kb distance of rs3917538. Rs662 has been linked to prognosis in metastatic gastric cancer 36.

For *PON1* and *TYMS,* we observed genewide significance (*PON1*: p_FDRGene_ = 0.04; *TYMS*: p_FDRGene_ < 0.01).

We did not observe significant associations of SNPs with DFS after FDR adjustment. Genewide significance after FDR adjustment was observed for *MAT2B* (p_FDRGene_ = 0.02) and *UMPS* (p_FDRGene_ < 0.01). *MAT2B* belongs to the methionine adenosyltransferase family and catalyzes the biosynthesis of *S*‐adenosylmethionine (*SAM*). *SAM* is essential in FOCM and has been linked to induced growth of human colon cancer cells *in vitro* 37. The gene *UMPS* encodes uridine 5′‐monophosphate synthase, an enzyme that catalyzes the final steps of *de novo* pyrimidine biosynthetic pathway. While the activity of this pathway is low in resting cells, it is indispensable in proliferating cells and is invariably upregulated in neoplastic cells and tumors 38.

In stratified analyses by 5‐FU‐based chemotherapy, we did not observe significant interactions with *a priori* selected candidate SNPs and OS. Significant genewide interactions with 5‐FU‐based chemotherapy were observed for *PON1* and *TYMS* (p_FDR‐INT_ = 0.04, p_FDR‐INT_ < 0.01, respectively). Prior research has linked the response rate and toxicity of 5‐FU‐based chemotherapy to thymidylate synthase 18. In fact, higher expression of *TYMS* in tumors has been associated with poor prognosis and worse response to 5‐FU‐based chemotherapy regimens 39. While there is strong evidence for the role of *TYMS* in response to 5‐FU‐based chemotherapy 39, this is the first study linking *PON1* to chemotherapy response in CRC. Prior data in metastatic gastric cancer show poor OS in patients with *PON1* rs662 AA/AG genotype that have received a combined regimen of 5‐FU‐based chemotherapy, epirubicin, and oxaliplatin 36. This is consistent with our findings.

Significant interaction with 5‐FU‐based chemotherapy and two *TCN2* candidates—rs9621049 and rs1801198—was observed for DFS. Prior research has linked rs1801198 to CpG island methylator phenotype high status 40, which is increasingly being recognized as an independent predictor of response to 5‐FU‐based chemotherapy 41, 42.

After FDR adjustment, we observed genewide significant interaction between 5‐FU‐based chemotherapy and DFS for *MAT2B* that catalyzes *SAM* biosynthesis. *SAM* modulates the anticancer effect of 5‐FU, but not other cytotoxic agents such as cisplatin 43. We did observe genewide significance for the association of *UMPS* with DFS, without effect modification by 5‐FU‐based chemotherapy. This is surprising as mutations of *UMPS* have been linked to 5‐FU resistance in CRC 44.

This is the most comprehensive study to date investigating the role of FOCM in relation to CRC survival. In addition, we evaluated interactions between FOCM genes and 5‐FU‐based chemotherapy and their impact on CRC prognosis. The pathway analysis approach covered all genetic variants simultaneously; thus, it accounts for interactions between genes assessing the association between a pathway and disease prognosis. All events of interest were ascertained actively and verified using death certificates, medical records, and information from attending physicians. Therefore, misclassification in the outcome variable is highly unlikely. The majority of patients were residents of Central Europe, which is indicative for a homogeneous study population.

Several limitations should be noted. False‐positive results might have occurred when we investigated the gene‐5‐FU interactions although we used FDR to minimize this possibility. The generalizability of our discoveries from a population free of folic acid fortification to populations where fortification is mandatory may be limited as folic acids can impact several aspects of FOCM 45. Further investigations in clinical populations are warranted to replicate findings and validate the clinical importance of the present results.

In conclusion, genetic variation in FOCM appears to be, to some extent, associated with CRC prognosis. Notably, effects were observed for genes in pyrimidine biosynthesis and fluorouracil drug metabolism, which are relevant therapeutic targets. Further investigations in clinical populations are warranted to replicate findings and validate the clinical importance of the present results.

## Conflict of Interest

There are no conflict of interest disclosures from the authors.

## Supporting information


**Table S1.** Polymorphisms in Folate‐mediated One‐Carbon Metabolism by Gene.
**Table S2.** Candidate SNPs.
**Table S3.** Selected subpathways and genes included.

**Table S4.** (a) Associations between non‐SNP *TYMS* polymorphisms with overall and disease‐free survival. (b) Associations between non‐SNP *TYMS* polymorphisms with overall survival stratified by 5‐FU chemotherapy. (c) Associations between non‐SNP *TYMS* polymorphisms with disease‐free survival stratified by 5‐FU chemotherapy.

**Table S5.** Global test on different pathways.

**Table S6.** Associations between selected polymorphisms in FOCM‐related genes and overall‐ and disease‐free survival stratified by 5‐FU‐based chemotherapy.

**Table S7.** Associations between polymorphisms in FOCM‐related genes and overall survival.

**Table S8.** Associations between selected polymorphisms in FOCM‐related genes and disease‐free survival.

**Table S9.** Associations between polymorphisms in FOCM‐related genes and overall survival stratified by 5‐FU‐based chemotherapy

**Table S10.** Associations between polymorphisms in FOCM‐related genes and disease‐free survival stratified by 5‐FU‐based chemotherapy*.
