# Supplementary material for: Pathway analysis of genetic variants in folate‐mediated one‐carbon metabolism‐related genes and survival in a prospectively followed cohort of colorectal cancer patients
Source: Cancer Med. 2018 May 29;7(7):2797–807. doi: 10.1002/cam4.1407 (PMC6051204; doi:10.1002/cam4.1407)
Supplement: Supplementary file 1 — Table S1. Polymorphisms in Folate‐mediated One‐Carbon Metabolism by Gene. Table S2. Candidate SNPs. Table S3. Selected subpathways and genes included. [file CAM4-7-2797-s001.docx]

| **Supplementary Table 1. Polymorphisms in Folate-mediated One-Carbon Metabolism by Gene.*** | | |
| --- | --- | --- |
| **Gene** |  | **Polymorphism** |
| **AARS** | **Alanyl-tRNA synthetase** |  |
|  | The human alanyl-tRNA synthetase (AARS) belongs to a family of tRNA synthases, of the class II enzymes. Class II tRNA synthases evolved early in evolution and are highly conserved | rs2070203, rs34087264 |
| **ABCC4** | **ATP binding cassette subfamily C member 4** |  |
|  | The protein encoded by this gene is a member of the superfamily of ATP-binding cassette (ABC) transporters. ABC proteins transport various molecules across extra- and intra-cellular membranes. | rs10508023, rs1059751, rs11568643, rs12864049, rs1628382, rs1678354, rs1678383, rs1678395, rs1678405, rs17189540, rs17235152, rs17268122, rs17268170, rs1729764, rs1729767, rs17300935, rs1750996, rs1751025, rs1751051, rs1764416, rs2274401, rs2892716, rs3782964, rs3818494, rs3864997, rs4148421, rs4148446, rs4148455, rs4148540, rs4148542, rs4148544, rs4636781, rs4771910, rs4773850, rs7981095, rs8001444, rs931111, rs943288, rs943290, rs9516530, rs9516551, rs9524822, rs9524861, rs9524902, rs9556455, rs9561778, rs9561811, rs9590183, rs997777 |
| **ADH1B** | **alcohol dehydrogenase 1B** |  |
|  | The protein encoded by this gene is a member of the alcohol dehydrogenase family. Members of this enzyme family metabolize a wide variety of substrates, including ethanol, retinol, other aliphatic alcohols, hydroxysteroids, and lipid peroxidation products | rs1159918, rs1229984, rs12507573, rs1693457, rs2066701 |
| **ADH1C** | **alcohol dehydrogenase 1C** |  |
|  | This gene encodes class I alcohol dehydrogenase, gamma subunit, which is a member of the alcohol dehydrogenase family. Members of this enzyme family metabolize a wide variety of substrates, including ethanol, retinol, other aliphatic alcohols, hydroxysteroids, and lipid peroxidation products. | rs11936869, rs1229849, rs1229863, rs1229980, rs1693482, rs2173201, rs2298753, rs2866152, rs904096 |
| **BHMT** | **betaine--homocysteine S-methyltransferase** |  |
|  | This gene encodes a cytosolic enzyme that catalyzes the conversion of betaine and homocysteine to dimethylglycine and methionine, respectively. | rs12655567, rs1291041, rs16876500, rs492842, rs9637824 |
| **BHMT2** | **betaine--homocysteine S-methyltransferase 2** |  |
|  | The protein encoded by this gene is one of two methyl transferases that can catalyze the transfer of the methyl group from betaine to homocysteine. Anomalies in homocysteine metabolism have been implicated in disorders ranging from vascular disease to neural tube birth defects such as spina bifida. | rs16876512, rs2909856, rs476620, rs626105, rs631305 |
| **CBS** | **cystathionine-beta-synthase** |  |
|  | The protein encoded by this gene acts as a homotetramer to catalyze the conversion of homocysteine to cystathionine, the first step in the transsulfuration pathway. The encoded protein is allosterically activated by adenosyl-methionine and uses pyridoxal phosphate as a cofactor. Defects in this gene can cause cystathionine beta-synthase deficiency (CBSD), which can lead to homocystinuria. | rs11701048, rs234706, rs234711, rs234713, rs2839623, rs2839626, rs422791, rs706209, rs719037, rs719038 |
| **DHFR** | **dihydrofolate reductase** |  |
|  | Dihydrofolate reductase converts dihydrofolate into tetrahydrofolate, a methyl group shuttle required for the de novo synthesis of purines, thymidylic acid, and certain amino acids. | rs10474632, rs11951910, rs1643665, rs1650717, rs1805355, rs6151617, rs6864493, rs836788, rs836790, rs836817 |
| **DNMT1** | **DNA methyltransferase 1** |  |
|  | This gene encodes an enzyme that transfers methyl groups to cytosine nucleotides of genomic DNA. | rs2228612 |
| **DNMT3A** | **DNA methyltransferase 3 alpha** |  |
|  | This gene encodes a DNA methyltransferase that is thought to function in de novo methylation, rather than maintenance methylation. The protein localizes to the cytoplasm and nucleus and its expression is developmentally regulated. | rs10460566,rs11695471, rs11887120, rs12991495, rs13401241, rs13420827, rs13428812, rs4665287 |
| **DNMT3B** | **DNA methyltransferase 3 beta** |  |
|  | This gene encodes a DNA methyltransferase which is thought to function in de novo methylation, rather than maintenance methylation. The protein localizes primarily to the nucleus and its expression is developmentally regulated. | rs13045669, rs17123673, rs183603, rs2235760, rs2424908, rs2424909, rs4911108, rs6058896, rs6119954, rs6579038 |
| **DPYD** | **dihydropyrimidine dehydrogenase** |  |
|  | The protein encoded by this gene is a pyrimidine catabolic enzyme and the initial and rate-limiting factor in the pathway of uracil and thymidine catabolism. Mutations in this gene result in dihydropyrimidine dehydrogenase deficiency, an error in pyrimidine metabolism associated with thymine-uraciluria and an increased risk of toxicity in cancer patients receiving 5-fluorouracil chemotherapy. | rs1034215, rs10783058, rs10783070, rs10875048, rs10875055, rs10875079, rs10875085, rs10875097, rs11165781, rs11165783, rs11165873, rs11165875, rs11165881, rs11587873, rs12030174, rs12046744, rs12047910, rs12073044, rs12126093, rs12134028, rs12740796, rs1333717, rs1413228, rs1415681, rs1514495, rs1520658, rs17116806, rs17431828, rs17471640, rs17702702, rs1801265, rs2039447, rs2151567, rs2152878, rs2786505, rs2786512, rs2786519, rs2811170, rs2811199, rs2811219, rs4300257, rs4379706, rs4950021, rs4950033, rs495257, rs552926, rs628959, rs6656660, rs6663670, rs6683883, rs6686861, rs7414210, rs7530858, rs7544128, rs7545340, rs828054, rs885622, rs9437663 |
| **DPYS** | **dihydropyrimidinase** |  |
|  | Dihydropyrimidinase catalyzes the conversion of 5,6-dihydrouracil to 3-ureidopropionate in pyrimidine metabolism. Dihydropyrimidinase is expressed at a high level in liver and kidney as a major 2.5-kb transcript and a minor 3.8-kb transcript. | rs13249169, rs13263121, rs16871361, rs17245950, rs2253336, rs2280010, rs2333874, rs2669429, rs2669434, rs2853142, rs2853145, rs2853149, rs2853154, rs2853161, rs2959024, rs2959025, rs2959026, rs3133278, rs3750187, rs3793357, rs3793358, rs6468924 |
| **DUT** | **deoxyuridine triphosphatase** |  |
|  | This gene encodes an essential enzyme of nucleotide metabolism. The encoded protein forms a ubiquitous, homotetrameric enzyme that hydrolyzes dUTP to dUMP and pyrophosphate. | rs8025164 |
| **EHMT1** | **euchromatic histone lysine methyltransferase 1** |  |
|  | The protein encoded by this gene is a histone methyltransferase that is part of the E2F6 complex, which represses transcription. The encoded protein methylates the Lys-9 position of histone H3, which tags it for transcriptional repression. | rs10780190, rs10867083, rs11137190, rs3123510, rs3125795, rs4573359, rs4634736, rs4876902, rs4876904, rs9314635 |
| **EHMT2** | **euchromatic histone lysine methyltransferase 2** |  |
|  | This gene encodes a methyltransferase that methylates lysine residues of histone H3. Methylation of H3 at lysine 9 by this protein results in recruitment of additional epigenetic regulators and repression of transcription. | rs2736428, rs9267649 |
| **FOLH1** | **folate hydrolase 1** |  |
|  | This gene encodes a type II transmembrane glycoprotein belonging to the M28 peptidase family. The protein acts as a glutamate carboxypeptidase on different alternative substrates, including the nutrient folate and the neuropeptide N-acetyl-l-aspartyl-l-glutamate. | rs10839236, rs16906190, rs202676, rs202680, rs202720, rs2299650, rs617528, rs663877, rs670776, rs7124497 |
| **FOLR1** | **folate receptor 1** |  |
|  | The protein encoded by this gene is a member of the folate receptor family. Members of this gene family bind folic acid and its reduced derivatives, and transport 5-methyltetrahydrofolate into cells. | rs651646 |
| **FDXR** | **ferredoxin reductase** |  |
|  | This gene encodes a mitochondrial flavoprotein that initiates electron transport for cytochromes P450 receiving electrons from NADPH. Multiple alternatively spliced transcript variants have been found for this gene | rs2070918, rs509911, rs689882, rs689895 |
| **FPGS** | **folylpolyglutamate synthase** |  |
|  | This gene encodes the folylpolyglutamate synthetase enzyme. This enzyme has a central role in establishing and maintaining both cytosolic and mitochondrial folylpolyglutamate concentrations and, therefore, is essential for folate homeostasis and the survival of proliferating cells. | rs10987746, rs7033913, rs7039798 |
| **GGH** | **gamma-glutamyl hydrolase** |  |
|  | This gene catalyzes the hydrolysis of folylpoly-gamma-glutamates and antifolylpoly-gamma-glutamates by the removal of gamma-linked polyglutamates and glutamate. | rs10957264, rs11545076, rs11545077, rs11545078, rs11995525, rs16930073, rs17194931, rs1800909, rs3758149, rs3780130, rs4446729, rs6472067, rs7010484 |
| **GNMT** | **glycine N-methyltransferase** |  |
|  | The protein encoded by this gene is an enzyme that catalyzes the conversion of S-adenosyl-L-methionine (along with glycine) to S-adenosyl-L-homocysteine and sarcosine. This protein is found in the cytoplasm and acts as a homotetramer. | rs1053538, rs2296805, rs6901782, rs6927188 |
| **MAT1A** | **methionine adenosyltransferase 1A** |  |
|  | This gene catalyzes a two-step reaction that involves the transfer of the adenosyl moiety of ATP to methionine to form S-adenosylmethionine and tripolyphosphate, which is subsequently cleaved to PPi and Pi. S-adenosylmethionine is the source of methyl groups for most biological methylations | rs10887708, rs10887718, rs1832683, rs2236568, rs2236569, rs9421467, rs998765, rs998766 |
| **MAT2B** | **methionine adenosyltransferase 2B** |  |
|  | The protein encoded by this gene belongs to the methionine adenosyltransferase (MAT) family. MAT catalyzes the biosynthesis of S-adenosylmethionine from methionine and ATP. | rs12655857, rs6869277, rs6874065, rs6882306, rs7721639 |
| **MTHFD1** | **methylenetetrahydrofolate dehydrogenase, cyclohydrolase and formyltetrahydrofolate synthetase 1** |  |
|  | This gene encodes a protein that possesses three distinct enzymatic activities, 5,10-methylenetetrahydrofolate dehydrogenase, 5,10-methenyltetrahydrofolate cyclohydrolase and 10-formyltetrahydrofolate synthetase. Each of these activities catalyzes one of three sequential reactions in the interconversion of 1-carbon derivatives of tetrahydrofolate, which are substrates for methionine, thymidylate, and de novo purine syntheses. | rs1256148, rs13329053, rs2236224, rs2236225, rs2281603, rs8003379 |
| **MTHFD2** | **methylenetetrahydrofolate dehydrogenase, cyclohydrolase and formyltetrahydrofolate synthetase 2** |  |
|  | This gene encodes a nuclear-encoded mitochondrial bifunctional enzyme with methylenetetrahydrofolate dehydrogenase and methenyltetrahydrofolate cyclohydrolase activities. The enzyme functions as a homodimer and is unique in its absolute requirement for magnesium and inorganic phosphate. | rs10177833, rs702462, rs702465, rs7571842, rs7587117, rs828861, rs828863 |
| **MTHFR** | **methylenetetrahydrofolate reductase** |  |
|  | The protein encoded by this gene catalyzes the conversion of 5,10-methylenetetrahydrofolate to 5-methyltetrahydrofolate, a co-substrate for homocysteine remethylation to methionine. Genetic variation in this gene influences susceptibility to occlusive vascular disease, neural tube defects, colon cancer and acute leukemia, and mutations in this gene are associated with methylenetetrahydrofolate reductase deficiency.[ | rs1476413, rs17376328, rs17421462, rs1801131, rs1801133 (singleton), rs2066471, rs4846047, rs4846049, rs7538516, |
| **MTR** | **5-methyltetrahydrofolate-homocysteine methyltransferase** |  |
|  | This gene encodes the 5-methyltetrahydrofolate-homocysteine methyltransferase. This enzyme, also known as cobalamin-dependent methionine synthase, catalyzes the final step in methionine biosynthesis. Mutations in MTR have been identified as the underlying cause of methylcobalamin deficiency complementation group G | rs10733117, rs12129440, rs1805087, rs3890786, rs4659727 |
| **MTRR** | **5-methyltetrahydrofolate-homocysteine methyltransferase reductase** |  |
|  | This gene encodes a member of the ferredoxin-NADP(+) reductase (FNR) family of electron transferases. This protein functions in the synthesis of methionine by regenerating methionine synthase to a functional state. Because methionine synthesis requires methyl-group transfer by a folate donor, activity of the encoded enzyme is important for folate metabolism and cellular methylation | rs10380, rs10475399, rs11134265, rs13181011, rs161869, rs162036 (tagged by rs162039), rs162039,rs162270, rs16879334, rs1801394 (singleton), rs1802059, rs2077744, rs2287780, rs2303080, rs7715062, rs9282787, rs9332 |
| **NFKB1** | **nuclear factor kappa B subunit 1** |  |
|  | This gene encodes a 105 kD protein which can undergo cotranslational processing by the 26S proteasome to produce a 50 kD protein. The 105 kD protein is a Rel protein-specific transcription inhibitor and the 50 kD protein is a DNA binding subunit of the NF-kappa-B (NFKB) protein complex | rs1609798, rs230540, rs230541, rs230547, rs3774934, rs3774968, rs4648022, rs4648090, rs4648110, rs4648141, rs4698863, rs7674640  rs909332, rs997476 |
| **NME1** | **NME/NM23 nucleoside diphosphate kinase 1** |  |
|  | This gene (NME1) was identified because of its reduced mRNA transcript levels in highly metastatic cells. Nucleoside diphosphate kinase (NDK) exists as a hexamer composed of 'A' (encoded by this gene) and 'B' (encoded by NME2) isoforms | rs10514981, rs11651252, rs11652793, rs11868380, rs1558252, rs1558253, rs16949683, rs2318784, rs2318785, rs3760469, rs4605213, rs7207090, rs7222463, rs7226059, rs880178 |
| **NME2** | **NME/NM23 nucleoside diphosphate kinase 2** |  |
|  | Nucleoside diphosphate kinase (NDK) exists as a hexamer composed of 'A' (encoded by NME1) and 'B' (encoded by this gene) isoforms. | rs2269829, rs3917527, rs3917538, rs757158  rs854560 |
| **PON1** | **paraoxonase 1** |  |
|  | The enzyme encoded by this gene is an arylesterase that mainly hydrolyzes paroxon to produce p-nitrophenol. Paroxon is an organophosphorus anticholinesterase compound that is produced in vivo by oxidation of the insecticide parathion. Polymorphisms in this gene are a risk factor in coronary artery disease. | rs2269829, rs3917527, rs3917538, rs757158, rs854560 |
| **PRDM2** | **PR/SET domain 2** |  |
|  | This tumor suppressor gene is a member of a nuclear histone/protein methyltransferase superfamily. It encodes a zinc finger protein that can bind to retinoblastoma protein, estrogen receptor, and the TPA-responsive element (MTE) of the heme-oxygenase-1 gene. | rs1015370, rs1203634, rs1203645, rs17350795, rs1980472, rs2235515, rs2244634, rs2245213, rs2294484, rs2744689, rs6690270 |
| **RRM1** | **ribonucleotide reductase catalytic subunit M1** |  |
|  | This gene encodes the large and catalytic subunit of ribonucleotide reductase, an enzyme essential for the conversion of ribonucleotides into deoxyribonucleotides. A pool of available deoxyribonucleotides is important for DNA replication during S phase of the cell cycle as well as multiple DNA repair processes. | rs10835601, rs10835613, rs10835677, rs10835678, rs12288551, rs12806698, rs1465952, rs4910904, rs7103860, rs7115496 |
| **RRM2** | **ribonucleotide reductase regulatory subunit M2** |  |
|  | This gene encodes one of two non-identical subunits for ribonucleotide reductase. This reductase catalyzes the formation of deoxyribonucleotides from ribonucleotides. Synthesis of the encoded protein (M2) is regulated in a cell-cycle dependent fashion. Transcription from this gene can initiate from alternative promoters, which results in two isoforms that differ in the lengths of their N-termini | rs1138729, rs4668664, rs6741290, rs7574663 |
| **SHMT1** | **serine hydroxymethyltransferase 1** |  |
|  | This gene encodes the cytosolic form of serine hydroxymethyltransferase, a pyridoxal phosphate-containing enzyme that catalyzes the reversible conversion of serine and tetrahydrofolate to glycine and 5,10-methylene tetrahydrofolate. This reaction provides one-carbon units for synthesis of methionine, thymidylate, and purines in the cytoplasm | rs1979277, rs2168781, rs4924849, rs9909104 |
| **SHMT2** | **serine hydroxymethyltransferase 2** |  |
|  | This gene encodes the mitochondrial form of a pyridoxal phosphate-dependent enzyme that catalyzes the reversible reaction of serine and tetrahydrofolate to glycine and 5,10-methylene tetrahydrofolate. The encoded product is primarily responsible for glycine synthesis. | rs10876968, rs1800165, rs7133939, rs7485577, rs7489231 |
| **SLC19A1** | **solute carrier family 19 member 1** |  |
|  | The membrane protein encoded by this gene is a transporter of folate and is involved in the regulation of intracellular concentrations of folate. Three transcript variants encoding different isoforms have been found for this gene. | rs1051266, rs1131596, rs12483553, rs12659, rs3788190, rs3788205, rs7279664 |
| **SLC29A1** | **solute carrier family 29 member 1** |  |
|  | This gene is a member of the equilibrative nucleoside transporter family. The gene encodes a transmembrane glycoprotein that localizes to the plasma and mitochondrial membranes and mediates the cellular uptake of nucleosides from the surrounding medium. | rs1057985, rs6458375, rs666462, rs6905285, rs693955, rs747199, rs9357436 |
| **TCN2** | **transcobalamin 2** |  |
|  | This gene encodes a member of the vitamin B12-binding protein family. This family of proteins, alternatively referred to as R binders, is expressed in various tissues and secretions. This plasma protein binds cobalamin and mediates the transport of cobalamin into cells. | rs10418, rs1131603 (singleton), rs1544468, rs1801198, rs4820874, rs4820886, rs4820889, rs5997711, rs740234, rs740235, rs9606756 , rs9621049 |
| **TYMS** | **thymidylate synthetase** |  |
|  | Thymidylate synthase catalyzes the methylation of deoxyuridylate to deoxythymidylate using 5,10-methylenetetrahydrofolate (methylene-THF) as a cofactor. This function maintains the dTMP (thymidine-5-prime monophosphate) pool critical for DNA replication and repair. The enzyme has been of interest as a target for cancer chemotherapeutic agents. It is considered to be the primary site of action for 5-fluorouracil, 5-fluoro-2-prime-deoxyuridine, and some folate analogs. | rs1001761, rs10502289, rs2244500, rs2741182, rs2847149, rs2853533, rs502396, rs495139 |
| **TK1** | **thymidine kinase 1** |  |
|  | The protein encoded by this gene is a cytosolic enzyme that catalyzes the addition of a gamma-phosphate group to thymidine. This creates dTMP and is the first step in the biosynthesis of dTTP, which is one component required for DNA replication. The encoded protein, whose levels fluctuate depending on the cell cycle stage, can act as a low activity dimer or a high activity tetramer. | rs1065769, rs12232476, rs16970907, rs1811086  rs2292235, rs2854701, rs2854702, rs9897765 |
| **TYMP** | **thymidine phosphorylase** |  |
|  | This gene encodes an angiogenic factor which promotes angiogenesis in vivo and stimulates the in vitro growth of a variety of endothelial cells. It has a highly restricted target cell specificity acting only on endothelial cells. Mutations in this gene have been associated with mitochondrial neurogastrointestinal encephalomyopathy | rs131815, rs131816, rs131817, rs140521  rs140522, rs140524 |
| **UMPH2** | **NT5C 5', 3'-nucleotidase, cytosolic** |  |
|  | This gene encodes a nucleotidase that catalyzes the dephosphorylation of the 5' deoxyribonucleotides (dNTP) and 2'(3')-dNTP and ribonucleotides, but not 5' ribonucleotides. Of the different forms of nucleotidases characterized, this enzyme is unique in its preference for 5'-dNTP | rs2291028, rs4789143, rs750844 |
| **UMPK** | **cytidine/uridine monophosphate kinase 1** |  |
|  | This gene encodes one of the enzymes required for cellular nucleic acid biosynthesis. This enzyme catalyzes the transfer of a phosphate group from ATP to CMP, UMP, or dCMP, to form the corresponding diphosphate nucleotide. | rs11582877, rs2622903, rs2820989, rs6660321  rs6690084 |
| **UMPS** | **uridine monophosphate synthetase** |  |
|  | This gene encodes a uridine 5'-monophosphate synthase. The encoded protein is a bifunctional enzyme that catalyzes the final two steps of the de novo pyrimidine biosynthetic pathway. The first reaction is carried out by the N-terminal enzyme orotate phosphoribosyltransferase which converts orotic acid to orotidine-5'-monophosphate | rs1162, rs13146, rs16835902, rs17282057, rs606552, rs694897 |
| **UNG** | **uracil DNA glycosylase** |  |
|  | This gene encodes one of several uracil-DNA glycosylases. One important function of uracil-DNA glycosylases is to prevent mutagenesis by eliminating uracil from DNA molecules by cleaving the N-glycosylic bond and initiating the base-excision repair (BER) pathway. Uracil bases occur from cytosine deamination or misincorporation of dUMP residues. | rs1059262, rs2160603, rs246079, rs246085  rs2569987, rs3219243 |
| *Candidate polymorphisms are marked by underlining | | |

| **Supplementary Table 2. Candidate SNPs** | | | |
| --- | --- | --- | --- |
| **Gene** | **SNP** | **Nucleotide**  **Changes** | **Amino Acid**  **Changes** |
| ***ADH1B*** | rs1229984 | A>G | His-Arg |
| ***ADH1C*** | rs1693482 | C>T | Arg-Gln |
| ***BHMT2*** | rs626105 | A>G |  |
| ***CDS*** | rs234713 | A>G | Gly-Gly |
| ***DNMT1*** | rs2228612 | A>G | Ile-Val |
| ***DNMT3A*** | rs11695471 | A>T |  |
| ***DNMT3A*** | rs13420827 | C>G |  |
| ***DNMT3B*** | rs2424909 | C>T |  |
| ***EHMT1*** | rs3125795 | G>T |  |
| ***EHMT1*** | rs4634736 | A>G |  |
| ***EHMT2*** | rs2736428 | A>G |  |
| ***FOLH1*** | rs10839236 | C>T |  |
| ***FOLH1*** | rs202676 | C>T | Tyr-His |
| ***FOLH1*** | rs202720 | C>G |  |
| ***GGH*** | rs11545076 | G>T |  |
| ***GGH*** | rs11545077 | A>G | Ala-Thr |
| ***GGH*** | rs11545078 | C>T | Thr-Ile |
| ***GGH*** | rs1800909 | C>T | Cys-Arg |
| ***GGH*** | rs3758149 | C>T |  |
| ***MTHFD1*** | rs2236224 | C>T |  |
| ***MTHFD1*** | rs2236225 | C>T | Arg-Gln |
| ***MTHFD1*** | rs8003379 | A>C |  |
| ***MTHFD2*** | rs702465 | A>T |  |
| ***MTHFD2*** | rs7571842 | A>G |  |
| ***MTHFR*** | rs1801131 | A>C | Glu-Ala |
| ***MTHFR*** | rs1801133 | C>T | Ala-Val |
| ***MTR*** | rs1805087 | A>G | Asp-Gly |
| ***MTRR*** | rs10380 | C>T | His-Tyr |
| ***MTRR*** | rs16879334 | C>G | Pro-Arg |
| ***MTRR*** | rs1801394 | A>G | Ile-Met |
| ***MTRR*** | rs2287780 | C>T | Arg-Cys |
| ***MTRR*** | rs2303080 | A>T | Ser-Thr |
| ***MTRR*** | rs9332 | C>T |  |
| ***PON1*** | rs854560 | A>C>G>N>T | Leu-Met |
| ***PRDM2*** | rs17350795 | A>G | Ser-Asn |
| ***SHMT1*** | rs1979277 | A>G | Leu-Phe |
| ***SHMT1*** | rs9909104 | C>T |  |
| ***SLC19A1*** | rs1051266 | A>G | His-Arg |
| ***SLC19A1*** | rs1131596 | C>T |  |
| ***SLC19A1*** | rs12659 | C>T |  |
| ***TCN2*** | rs1131603 | C>T | Leu-Ser |
| ***TCN2*** | rs1801198 | C>G | Arg-Pro |
| ***TCN2*** | rs4820889 | A>G | Arg-Gln |
| ***TCN2*** | rs9606756 | A>G | Ile-Val |
| ***TCN2*** | rs9621049 | C>T | Ser-Phe |
| ***TYMS*** | rs1001761 | C>T |  |
| ***TYMS*** | rs10502289 | A>T |  |
| ***TYMS*** | rs2847149 | A>G |  |
| ***TYMS*** | rs2853533 | C>G | Arg-Gly |
| ***TYMS*** | rs502396 | C>T |  |

| **Supplementary Table 3. Selected subpathways and genes included^1^** | |
| --- | --- |
| **Subpathways** | **Genes per pathways** |
| **Folate** | MTR_PC3, DNMT3A_PC4, TCN2_PC2, EHMT1_PC1, MTRR_PC1, TYMS_PC2, DNMT3B_PC5, DHFR_PC1, EHMT1_PC2, TCN2_PC5, MAT2B_PC4, MAT2B_PC2, DNMT1_PC0, MTRR_PC6, MTHFR_PC1, MTHFD2_PC3, MTHFR_PC5, SLC19A1_PC4, DNMT3A_PC3, EHMT1_PC3, MTR_PC1, BHMT_PC4, BHMT_PC3, BHMT2_PC3, BHMT_PC1, BHMT2_PC1, DHFR_PC5, BHMT2_PC2, BHMT_PC2, MTRR_PC3, SHMT1_PC1, TCN2_PC3, MTHFD2_PC2, MTRR_PC5, DNMT3B_PC6  DHFR_PC3, MTR_PC2, MTRR_PC2, DNMT3A_PC2, TCN2_PC4, EHMT1_PC6, DNMT3B_PC4, SLC19A1_PC1, FPGS_PC2, SHMT1_PC2, TYMS_PC5, MTHFD1_PC2, DNMT3B_PC3, TCN2_PC6, GGH_PC2, TYMS_PC4, EHMT1_PC4, NFKB1_PC5  MAT2B_PC1, DHFR_PC2, NFKB1_PC2, GGH_PC5, MTHFD1_PC3, DNMT3A_PC5, DNMT3B_PC1, SHMT2_PC3, SLC19A1_PC3, MTHFR_PC4, TCN2_PC1, TYMS_PC3, SHMT2_PC2, MTHFD2_PC1, NFKB1_PC4, TCN2_PC7, GGH_PC1, DNMT3B_PC2, NFKB1_PC6, SHMT1_PC3, GGH_PC4, DHFR_PC4, SLC19A1_PC2, MTHFR_PC2, DNMT3A_PC1, MTHFD1_PC1, GGH_PC3, MTRR_PC4, TYMS_PC1, EHMT1_PC5, NFKB1_PC1, FOLR1_PC0, MAT2B_PC3, FPGS_PC1, NFKB1_PC3, MTHFR_PC3, SHMT2_PC1 |
| **Methionine** | MTR_PC3, MAT1A_PC3, DNMT3A_PC2, CBS_PC3, MTRR_PC5, MAT1A_PC2, DNMT3B_PC6, MAT1A_PC1, CBS_PC2, EHMT1_PC6, DNMT3B_PC4, MAT2B_PC1, MTRR_PC1, DNMT3A_PC5, CBS_PC6, DNMT1_PC0, MTRR_PC6, MTRR_PC4  MAT2B_PC2, CBS_PC1, DNMT3B_PC2, MTR_PC1, EHMT1_PC2, MAT1A_PC4, CBS_PC5, EHMT1_PC3, DNMT3B_PC3, BHMT_PC4, BHMT_PC3, BHMT2_PC3, BHMT_PC1, BHMT2_PC1, BHMT2_PC2, BHMT_PC2, DNMT3A_PC1, CBS_PC4  MAT2B_PC3, EHMT1_PC5, MTRR_PC3, DNMT3A_PC4, EHMT1_PC4, MAT2B_PC4, DNMT3B_PC5, EHMT1_PC1, DNMT3A_PC3, MTR_PC2, MTRR_PC2, DNMT3B_PC1 |
| **Pyrimidine** | DPYD_PC5, DPYS_PC6, DPYD_PC10, DPYS_PC13, DPYD_PC24, TK1_PC2, TYMS_PC1, DPYD_PC15, DPYD_PC19, RRM2_PC1, TK1_PC5, DPYD_PC16, DPYD_PC26, TYMP_PC3, RRM1_PC5, DPYS_PC12, DPYD_PC23, RRM1_PC4  UMPS_PC3, NME1_PC3, DPYD_PC20, DPYD_PC28, DPYS_PC9, DPYD_PC12, DPYS_PC5, RRM1_PC2, RRM2_PC2, TYMP_PC1, UMPS_PC1, DPYD_PC25, DPYD_PC14, NME1_PC2, DPYS_PC10, TYMS_PC4, DPYS_PC3, NME1_PC10  TYMP_PC4, DPYD_PC13, RRM1_PC3, DPYD_PC17, DPYS_PC1, DUT_PC0, DPYD_PC11, NME1_PC8, TK1_PC3, NME1_PC5, NME1_PC1, NME2_PC0, NME1_PC6, TYMS_PC2, DPYD_PC2, TK1_PC1, NME1_PC7, DPYD_PC9, TYMS_PC5, DPYD_PC22, TYMP_PC2, DPYD_PC4, UMPS_PC2, TK1_PC4, DPYD_PC18, RRM1_PC1, DPYS_PC4, NME1_PC4, TYMS_PC3, DPYS_PC8  DPYD_PC27, DPYS_PC11, DPYD_PC8, DPYS_PC7, DPYD_PC6, DPYS_PC2, DPYD_PC7, TYMP_PC5, DPYD_PC3, NME1_PC9, DPYD_PC1, DPYD_PC21 |
| **Purine** | NME1_PC3, MTRR_PC3, RRM2_PC2, NME1_PC4, RRM2_PC1, MTRR_PC6, NME1_PC7, NME1_PC9, RRM1_PC1, MTRR_PC1, RRM1_PC2, MTRR_PC5, RRM1_PC3, RRM1_PC4, NME1_PC2, NME1_PC5, NME1_PC1, NME2_PC0  NME1_PC6, NME1_PC8, MTRR_PC4, RRM1_PC5, MTRR_PC2, NME1_PC10 |
| **Fluorouracil** | DPYD_PC5, DPYS_PC6, UMPS_PC1, DPYD_PC25, DPYD_PC26, TYMP_PC3, DPYS_PC4, DPYD_PC10, DPYS_PC13, DPYD_PC24, TK1_PC2, DPYS_PC7, DPYD_PC6, DPYD_PC21, DPYS_PC12, DPYD_PC23, DPYD_PC28, DPYS_PC9  DPYD_PC18, UMPS_PC3, DPYD_PC3, TK1_PC5, DPYD_PC16, DPYS_PC3, DPYD_PC22, TYMP_PC2, DPYD_PC4, UMPS_PC2, DPYD_PC14, DPYS_PC10, TK1_PC4, DPYD_PC20, DPYD_PC12, DPYS_PC5, DPYD_PC11, K1_PC3  TYMP_PC5, DPYD_PC27, DPYS_PC8, DPYD_PC15, DPYD_PC9, TYMP_PC4, DPYS_PC2, DPYD_PC7, TYMP_PC1, DPYD_PC8, TK1_PC1, DPYD_PC2, DPYD_PC17, DPYS_PC11, DPYS_PC1, DPYD_PC13, DPYD_PC19, DPYD_PC1 |
| **Glycine** | CBS_PC6, SHMT1_PC1, GNMT_PC1, CBS_PC4, BHMT_PC4, BHMT_PC3, BHMT2_PC3, BHMT_PC1, BHMT2_PC1, BHMT2_PC2, BHMT_PC2, CBS_PC1, SHMT2_PC3, GNMT_PC3, CBS_PC3, SHMT2_PC1, CBS_PC5, SHMT1_PC3  SHMT2_PC2, GNMT_PC2, SHMT1_PC2, CBS_PC2 |

^1^The Molecular Signatures Database v3.1 of the Broad Institute was used to identify subpathways through searching for one-carbon, folate, and 5-FU-based search terms and YY KEGG and YY GO pathways were extracted.
