# Supplementary material for: Pathway analysis of genetic variants in folate‐mediated one‐carbon metabolism‐related genes and survival in a prospectively followed cohort of colorectal cancer patients
Source: Cancer Med. 2018 May 29;7(7):2797–807. doi: 10.1002/cam4.1407 (PMC6051204; doi:10.1002/cam4.1407)
Supplement: Supplementary file 2 — Table S4. (a) Associations between non‐SNP TYMS polymorphisms with overall and disease‐free survival. (b) Associations between non‐SNP TYMS polymorphisms with overall survival stratified by 5‐FU chemotherapy. (c) Associations between non‐SNP TYMS polymorphisms with disease‐free survival stratified by 5‐FU chemotherapy. [file CAM4-7-2797-s002.docx]

| **Supplementary Table 4a. Associations between non-SNP *TYMS* polymorphisms with overall and disease-free survival** | | | | | | | | | | | |
| --- | --- | --- | --- | --- | --- | --- | --- | --- | --- | --- | --- |
|  |  |  | **Overall Survival** | | | |  | **Disease-free Survival** | | | |
|  | **Genotype** | **n** | **Hazard Ratio (95% CI)** | | | **p-Value** | **n** | **Hazard Ratio (95% CI)** | | | **p-Value** |
| **TS 3'-UTR 1494 del** | **+6/+6** | 269 | **Ref** | | |  | 263 | **Ref** | | | |
|  | **+6/-6** | 237 | 0.87 (0.72 - 1.05) | | | 0.14 | 216 | 0.85 (0.69 - 1.05) | | | 0.13 |
|  | **-6/-6** | 50 | 0.80 (0.57 - 1.11) | | | 0.19 | 55 | 1.04 (0.75 - 1.46) | | | 0.81 |
|  |  |  |  |  |  |  |  |  |  |  |  |
|  | **+6/-6 or -6/-6** | 287 | 0.86 (0.72 - 1.02) | | | 0.09 | 271 | 0.89 (0.73 - 1.08) | | | 0.23 |
|  |  |  |  |  |  |  |  |  |  |  |  |
|  | **per -6 allele** | 50 | 0.88 (0.77 - 1.02) | | | 0.09 | 55 | 0.95 (0.82 - 1.11) | | | 0.54 |
|  |  |  |  |  |  |  |  |  |  |  |  |
| **TSER** | **3R/3R** | 138 | **Ref** | | |  | 137 | **Ref** | | |  |
|  | **2R/3R** | 286 | 1.18 (0.95 - 1.48) | | | 0.13 | 267 | 1.15 (0.91 - 1.46) | | | 0.24 |
|  | **2R/2R** | 114 | 1.08 (0.83 - 1.42) | | | 0.57 | 114 | 1.05 (0.79 - 1.41) | | | 0.73 |
|  |  |  |  |  |  |  |  |  |  |  |  |
|  | **2R/3R or 2R/2R** | 400 | 1.15 (0.94 - 1.19) | | | 0.18 | 381 | 1.12 (0.89 - 1.41) | | | 0.32 |
|  |  |  |  |  |  |  |  |  |  |  |  |
|  | **per 2R allele** | 114 | 1.05 (0.92 - 1.42) | | | 0.18 | 114 | 1.03 (0.89 - 1.19) | | | 0.66 |

| **Supplementary Table 4b. Associations between non-SNP *TYMS* polymorphisms with overall survival stratified by 5-FU chemotherapy** | | | | | | | | | | | | |
| --- | --- | --- | --- | --- | --- | --- | --- | --- | --- | --- | --- | --- |
|  |  |  | **Received 5-FU Chemotherapy** | | | |  | **Did not receive 5-FU chemotherapy** | | | |  |
| **Overall Survival** | **Genotype** | **n** | **Hazard Ratio (95% CI)** | | | **p-Value** | **n** | **Hazard Ratio (95% CI)** | | | **p-Value** | **p-Value_interact_** |
| **TS 3'-UTR 1494 del** | **+6/+6** | 135 | **Ref** | | |  | 17 | **Ref** | | | | 0.41 |
|  | **+6/-6** | 106 | 0.98 (0.54 - 1.79) | | | 0.95 | 16 | 0.82 (0.38 - 1.74) | | | 0.61 |  |
|  | **-6/-6** | 29 | 0.90 (0.45 - 1.77) | | | 0.76 | 1 | NA | | | NA |  |
|  |  |  |  |  |  |  |  |  |  |  |  |  |
|  | **+6/-6 or -6/-6** | 135 | 0.96 (0.53 - 1.73) | | | 0.88 | 17 | 0.77 (0.36 - 1.62) | | | 0.48 | 0.91 |
|  |  |  |  |  |  |  |  |  |  |  |  |  |
|  | **per -6 allele** | 29 | 0.81 (0.44 - 1.50) | | | 0.51 | 1 | 0.50 (0.13 - 1.99) | | | 0.32 | 0.63 |
|  |  |  |  |  |  |  |  |  |  |  |  |  |
| **TSER** | **3R/3R** | 71 | **Ref** | | |  | 6 | **Ref** | | |  | 0.49 |
|  | **2R/3R** | 134 | 2.18 (0.88 – 5.40) | | | 0.09 | 18 | 1.84 (0.66 – 5.15) | | | 0.24 |  |
|  | **2R/2R** | 56 | 2.01 (0.79 – 5.12) | | | 0.14 | 10 | 2.15 (0.70 – 6.64) | | | 0.18 |  |
|  |  |  |  |  |  |  |  |  |  |  |  |  |
|  | **2R/3R or 2R/2R** | 190 | 2.13 (0.86 - 5.25) | | | 0.10 | 28 | 1.95 (0.73 - 5.18) | | | 0.18 | 0.27 |
|  |  |  |  |  |  |  |  |  |  |  |  |  |
|  | **per 2R allele** | 56 | 1.85 (0.88 - 3.89) | | | 0.10 | 17 | 2.04 (0.71 - 5.84) | | | 0.18 | 0.26 |

| **Supplementary Table 4c. Associations between non-SNP *TYMS* polymorphisms with disease-free survival stratified by 5-FU chemotherapy** | | | | | | | | | | | | |  |
| --- | --- | --- | --- | --- | --- | --- | --- | --- | --- | --- | --- | --- | --- |
|  |  | **Received 5-FU Chemotherapy** | | | | | **Did not receive 5-FU chemotherapy** | | | | |  | |
| **Disease-free survival** | **Genotype** | **n** | **Hazard Ratio (95% CI)** | | | **p-Value** | **n** | **Hazard Ratio (95% CI)** | | | **p-Value** | **p-Value_interact_** | |
| **TS 3'-UTR 1494 del** | **+6/+6** | 153 | **Ref** | | |  | 18 | **Ref** | | | | 0.63 | |
|  | **+6/-6** | 119 | 0.94 (0.50 - 1.76) | | | 0.86 | 12 | 0.58 (0.26 - 1.31) | | | 0.19 |  | |
|  | **-6/-6** | 34 | 1.06 (0.53 - 2.12) | | | 0.86 | 2 | 0.98 (0.13 - 7.60) | | | 0.98 |  | |
|  |  |  |  |  |  |  |  |  |  |  |  |  | |
|  | **+6/-6 or -6/-6** | 153 | 0.97 (0.53 - 1.80) | | | 0.93 | 14 | 0.60 (0.27 - 1.33) | | | 0.19 | 0.34 | |
|  |  |  |  |  |  |  |  |  |  |  |  |  | |
|  | **per -6 allele** | 34 | 1.01 (0.52 - 1.96) | | | 0.97 | 2 | 0.45 (0.10 - 2.01) | | | 0.29 | 0.37 | |
|  |  |  |  |  |  |  |  |  |  |  |  |  | |
| **TSER** | **3R/3R** | 85 | **Ref** | | |  | 7 | **Ref** | | |  |  | |
|  | **2R/3R** | 145 | 2.03 (0.88 - 4.68) | | | 0.10 | 14 | 1.30 (0.47 - 3.57) | | | 0.60 | 0.12 | |
|  | **2R/2R** | 65 | 1.80 (0.76 - 4.30) | | | 0.18 | 11 | 2.85 (0.97 - 8.38) | | | 0.06 |  | |
|  |  |  |  |  |  |  |  |  |  |  |  |  | |
|  | **2R/3R or 2R/2R** | 210 | 1.95 (0.85 - 4.49) | | | 0.11 | 25 | 1.69 (0.66 – 4.32) | | | 0.27 | 0.27 | |
|  |  |  |  |  |  |  |  |  |  |  |  |  | |
|  | **per 2R allele** | 65 | 2.17 (0.99 - 4.73) | | | 0.05 | 11 | 2.97 (0.96 - 9.26) | | | 0.06 | 0.06 | |
