# Supplementary material for: Pathway analysis of genetic variants in folate‐mediated one‐carbon metabolism‐related genes and survival in a prospectively followed cohort of colorectal cancer patients
Source: Cancer Med. 2018 May 29;7(7):2797–807. doi: 10.1002/cam4.1407 (PMC6051204; doi:10.1002/cam4.1407)
Supplement: Supplementary file 3 — Table S5. Global test on different pathways. [file CAM4-7-2797-s003.docx]

| **Supplementary Table 5. Global test on different pathways^1,2^** | | | | | |
| --- | --- | --- | --- | --- | --- |
| **Pathway** | **p-value** | **Statistic** | **Expected** | **Standard deviation** | **EigenSNPs (n)** |
| **Folate** | 0.710 | 0.051 | 0.056 | ±0.009 | 86 |
| **Methionine** | 0.833 | 0.044 | 0.056 | ±0.012 | 46 |
| **Pyrimidine** | **0.045** | 0.078 | 0.056 | ±0.013 | 73 |
| **Purine** | 0.957 | 0.030 | 0.056 | ±0.015 | 22 |
| **Fluorouracil** | **0.011** | 0.093 | 0.056 | ±0.014 | 51 |
| **Glycine** | 0.945 | 0.028 | 0.056 | ±0.018 | 21 |
| **Total** | 0.214 | 0.061 | 0.056 | ±0.006 | 236 |
| **^1^**Unadjusted, non-imputed genotypes.  ^2^ The Molecular Signatures Database v3.1 of the Broad Institute was used to identify subpathways through searching for one-carbon, folate, and 5-FU based search terms and YY KEGG and YY GO pathways were extracted. | | | | | |
