# Supplementary material for: Pathway analysis of genetic variants in folate‐mediated one‐carbon metabolism‐related genes and survival in a prospectively followed cohort of colorectal cancer patients
Source: Cancer Med. 2018 May 29;7(7):2797–807. doi: 10.1002/cam4.1407 (PMC6051204; doi:10.1002/cam4.1407)
Supplement: Supplementary file 4 — Table S6. Associations between selected polymorphisms in FOCM‐related genes and overall‐ and disease‐free survival stratified by 5‐FU‐based chemotherapy. [file CAM4-7-2797-s004.docx]

| **Supplementary Table 6. Associations between selected polymorphisms in FOCM-related genes and overall- and disease-free survival stratified by 5-FU-based chemotherapy*** | | | | | | | | | | | | | |
| --- | --- | --- | --- | --- | --- | --- | --- | --- | --- | --- | --- | --- | --- |
|  |  |  |  | **No 5-FU-based-chemotherapy** | | | **Received 5-FU-based-chemotherapy** | | | **Restricted to 5-FU or 5-FU + FA** | | | |
|  |  |  |  | **Alive** | **Deceased** | **HR(95%-CI)** | **Alive** | **Deceased** | **HR(95%-CI)** | **Alive Deceased HR(95%-CI)** | | | |
| **Gene** | | **SNP** | **Genotype** | **n** | **n** |  | **n** | **n** |  | **n n** | | |  |
| ***Overall survival PON1*** | | **rs3917538** | C/C | 37 | 20 | ref | 231 | 132 | 1.21(0.71-2.06) | 135 | 58 | 0.89(0.65-1.21) | |
|  |  |  | C/T | 18 | 12 | 1.55(0.69-3.47) | 137 | 112 | 1.54(0.89-2.64) | 78 | 36 | 0.83(0.55-1.25) | |
|  |  |  | T/T | 3 | 2 | 0.55(0.07-4.30) | 9 | 26 | **2.97(1.51-5.85)** | 6 | 7 | **2.84(1.31-6.16)** | |
|  |  |  | C/T or T/T^ǂ^ | 21 | 14 | 1.30(0.59-2.83) | 146 | 138 | 1.65(0.97-2.83) | 84 | 43 | 0.98(0.67-1.42) | |
| ***Disease-free survival MAT2B*** | | **rs12655857** | G/G | 24 | 21 | ref | 189 | 170 | 0.80(0.47-1.35) | 112 | 61 | 0.82(0.58-1.17) | |
|  |  |  | G/T | 31 | 9 | **0.36(0.15-0.88)** | 128 | 119 | 0.91(0.53-1.55) | 79 | 47 | 1.00(0.69-1.45) | |
|  |  |  | T/T | 5 | 2 | 0.22(0.03-1.67) | 24 | 17 | 0.78(0.38-1.60) | 16 | 5 | 0.82(0.33-2.02) | |
|  |  |  | G/T or T/T^ǂ^ | 36 | 11 | **0.33(0.14-0.79)** | 152 | 136 | 0.89(0.52-1.52) | 95 | 42 | 0.98(0.68-1.39) | |
| ***TCN2*** | | **rs9621049**** | C/C | 54 | 25 | ref | 264 | 256 | **1.65(1.03-2.67)** | 154 | 93 | 1.10(0.83-1.46) | |
|  |  |  | C/T | 6 | 6 | 3.33(1.22-9.10) | 73 | 47 | 1.41(0.80-2.45) | 50 | 19 | **0.54(0.30-0.95)** | |
|  |  |  | T/T | 0 | 1 | 0.63(0.09-4.60) | 4 | 3 | 1.05(0.14-7.98) | 3 | 1 | 0.93(0.13-6.72) | |
|  |  |  | C/T or T/T^ǂ^ | 6 | 7 | 3.34(1.22-9.11) | 77 | 50 | 1.39(0.80-2.43) | 53 | 20 | **0.55(0.32-0.96)** | |
| *adjusted for age, sex, stage, grade, BMI, alcohol intake  **candidate, FDR-adjusted cut-off for significance of p-value = 0.02  ^ǂ^dominant model (HR_het_)  ^1^p_trend_:p-Value for trend  ^2^p_trendFDR_:FDR adjusted trend  ^3^p_FDRGenwide_:FDR adjusted genewide effect | | | | | | | | | | | | | |
