# Supplementary material for: Pathway analysis of genetic variants in folate‐mediated one‐carbon metabolism‐related genes and survival in a prospectively followed cohort of colorectal cancer patients
Source: Cancer Med. 2018 May 29;7(7):2797–807. doi: 10.1002/cam4.1407 (PMC6051204; doi:10.1002/cam4.1407)
Supplement: Supplementary file 5 — Table S7. Associations between polymorphisms in FOCM‐related genes and overall survival. [file CAM4-7-2797-s005.docx]

| **Supplementary Table 7.**  **Associations between polymorphisms in FOCM-related genes and overall survival** | | | | | | | | | | | | | |
| --- | --- | --- | --- | --- | --- | --- | --- | --- | --- | --- | --- | --- | --- |
|  | | | | | | | **Ctrl** | | **Cases** | |  | | |
| **Gene** | **SNP** | **Genotype** | **HR (95%-CI)** | **p** | **FDR_p** | **FDR_(byGene)_p_** | **N** | **%** | **N** | **%** | **NObsUsed** | **Events** | **LowCount** |
| AARS ---- tag | rs2070203 | T/T | 1.00 (.-.) | . | . | . | 313 | 26,32 | 163 | 29,26 | 1516 | 489 |  |
| AARS ---- tag |  | T/C or C/C | 0.99 (0.81-1.20) | 0.89 | 0.99 | 0.89 | 876 | 73,68 | 394 | 70,74 | 1516 | 489 |  |
| AARS ---- tag | rs34087264 | G/G | 1.00 (.-.) | . | . | . | 356 | 29,94 | 167 | 29,98 | 1516 | 489 |  |
| AARS ---- tag |  | G/A or A/A | 0.83 (0.68-1.00) | 0.05 | 0.64 | 0.11 | 833 | 70,06 | 390 | 70,02 | 1516 | 489 |  |
| ABCC4 ---- tag | rs10508023 | G/G | 1.00 (.-.) | . | . | . | 940 | 79,06 | 429 | 77,02 | 1516 | 489 |  |
| ABCC4 ---- tag |  | G/C or C/C | 1.10 (0.89-1.35) | 0.39 | 0.93 | 0.81 | 249 | 20,94 | 128 | 22,98 | 1516 | 489 |  |
| ABCC4 ---- tag | rs1059751 | T/T | 1.00 (.-.) | . | . | . | 340 | 28,60 | 158 | 28,37 | 1516 | 489 |  |
| ABCC4 ---- tag |  | T/C or C/C | 1.05 (0.86-1.28) | 0.62 | 0.99 | 0.95 | 849 | 71,40 | 399 | 71,63 | 1516 | 489 |  |
| ABCC4 ---- tag | rs11568643 | A/A | 1.00 (.-.) | . | . | . | 1000 | 84,10 | 455 | 81,69 | 1516 | 489 |  |
| ABCC4 ---- tag |  | A/G or G/G | 1.24 (0.99-1.56) | 0.06 | 0.66 | 0.60 | 189 | 15,90 | 102 | 18,31 | 1516 | 489 |  |
| ABCC4 ---- NA | rs11568658 | G/G | 1.00 (.-.) | . | . | . | 1127 | 94,79 | 527 | 94,61 | 1516 | 489 |  |
| ABCC4 ---- NA |  | G/T or T/T | 1.13 (0.75-1.71) | 0.56 | 0.99 | 0.95 | 62 | 5,21 | 30 | 5,39 | 1516 | 489 |  |
| ABCC4 ---- tag | rs12864049 | T/T | 1.00 (.-.) | . | . | . | 890 | 74,85 | 416 | 74,69 | 1516 | 489 |  |
| ABCC4 ---- tag |  | T/C or C/C | 1.00 (0.82-1.24) | 0.96 | 0.99 | 0.98 | 299 | 25,15 | 141 | 25,31 | 1516 | 489 |  |
| ABCC4 ---- tag | rs1628382 | G/G | 1.00 (.-.) | . | . | . | 741 | 62,32 | 347 | 62,30 | 1516 | 489 |  |
| ABCC4 ---- tag |  | G/A or A/A | 1.01 (0.84-1.21) | 0.95 | 0.99 | 0.98 | 448 | 37,68 | 210 | 37,70 | 1516 | 489 |  |
| ABCC4 ---- tag | rs1678354 | C/C | 1.00 (.-.) | . | . | . | 500 | 42,05 | 237 | 42,55 | 1516 | 489 |  |
| ABCC4 ---- tag |  | C/G or G/G | 1.01 (0.84-1.21) | 0.92 | 0.99 | 0.98 | 689 | 57,95 | 320 | 57,45 | 1516 | 489 |  |
| ABCC4 ---- tag | rs1678383 | T/T | 1.00 (.-.) | . | . | . | 964 | 81,08 | 450 | 80,79 | 1516 | 489 |  |
| ABCC4 ---- tag |  | T/G or G/G | 0.89 (0.70-1.12) | 0.31 | 0.90 | 0.72 | 225 | 18,92 | 107 | 19,21 | 1516 | 489 |  |
| ABCC4 ---- tag | rs1678395 | G/G | 1.00 (.-.) | . | . | . | 1031 | 86,71 | 476 | 85,46 | 1516 | 489 |  |
| ABCC4 ---- tag |  | G/A or A/A | 0.87 (0.67-1.12) | 0.28 | 0.88 | 0.71 | 158 | 13,29 | 81 | 14,54 | 1516 | 489 |  |
| ABCC4 ---- tag | rs1678405 | T/T | 1.00 (.-.) | . | . | . | 562 | 47,27 | 255 | 45,78 | 1516 | 489 |  |
| ABCC4 ---- tag |  | T/C or C/C | 1.11 (0.93-1.33) | 0.25 | 0.86 | 0.71 | 627 | 52,73 | 302 | 54,22 | 1516 | 489 |  |
| ABCC4 ---- tag | rs17189540 | A/A | 1.00 (.-.) | . | . | . | 1042 | 87,64 | 477 | 85,64 | 1516 | 489 |  |
| ABCC4 ---- tag |  | A/G or G/G | 1.18 (0.92-1.52) | 0.20 | 0.85 | 0.70 | 147 | 12,36 | 80 | 14,36 | 1516 | 489 |  |
| ABCC4 ---- tag | rs17235152 | T/T | 1.00 (.-.) | . | . | . | 864 | 72,67 | 409 | 73,43 | 1516 | 489 |  |
| ABCC4 ---- tag |  | T/C or C/C | 1.02 (0.83-1.25) | 0.86 | 0.99 | 0.98 | 325 | 27,33 | 148 | 26,57 | 1516 | 489 |  |
| ABCC4 ---- tag | rs17268122 | G/G | 1.00 (.-.) | . | . | . | 721 | 60,64 | 347 | 62,30 | 1516 | 489 |  |
| ABCC4 ---- tag |  | G/T or T/T | 1.00 (0.83-1.21) | 0.99 | 1.00 | 0.99 | 468 | 39,36 | 210 | 37,70 | 1516 | 489 |  |
| ABCC4 ---- tag | rs17268170 | C/C | 1.00 (.-.) | . | . | . | 980 | 82,42 | 450 | 80,79 | 1516 | 489 |  |
| ABCC4 ---- tag |  | C/T or T/T | 1.01 (0.81-1.27) | 0.93 | 0.99 | 0.98 | 209 | 17,58 | 107 | 19,21 | 1516 | 489 |  |
| ABCC4 ---- tag | rs1729764 | A/A | 1.00 (.-.) | . | . | . | 937 | 78,81 | 453 | 81,33 | 1516 | 489 |  |
| ABCC4 ---- tag |  | A/G or G/G | 0.86 (0.68-1.09) | 0.21 | 0.85 | 0.70 | 252 | 21,19 | 104 | 18,67 | 1516 | 489 |  |
| ABCC4 ---- tag | rs1729767 | T/T | 1.00 (.-.) | . | . | . | 641 | 53,91 | 285 | 51,17 | 1516 | 489 |  |
| ABCC4 ---- tag |  | T/C or C/C | 1.07 (0.89-1.28) | 0.49 | 0.95 | 0.91 | 548 | 46,09 | 272 | 48,83 | 1516 | 489 |  |
| ABCC4 ---- tag | rs17300935 | C/C | 1.00 (.-.) | . | . | . | 876 | 73,68 | 407 | 73,07 | 1516 | 489 |  |
| ABCC4 ---- tag |  | C/G or G/G | 1.06 (0.86-1.29) | 0.60 | 0.99 | 0.95 | 313 | 26,32 | 150 | 26,93 | 1516 | 489 |  |
| ABCC4 ---- tag | rs1750190 | G/G | 1.00 (.-.) | . | . | . | 321 | 27,00 | 132 | 23,70 | 1516 | 489 |  |
| ABCC4 ---- tag |  | G/A or A/A | 1.17 (0.94-1.44) | 0.16 | 0.83 | 0.70 | 868 | 73,00 | 425 | 76,30 | 1516 | 489 |  |
| ABCC4 ---- tag | rs1750996 | A/A | 1.00 (.-.) | . | . | . | 798 | 67,12 | 378 | 67,86 | 1516 | 489 |  |
| ABCC4 ---- tag |  | A/G or G/G | 0.91 (0.75-1.10) | 0.33 | 0.92 | 0.72 | 391 | 32,88 | 179 | 32,14 | 1516 | 489 |  |
| ABCC4 ---- tag | rs1751025 | C/C | 1.00 (.-.) | . | . | . | 571 | 48,02 | 255 | 45,78 | 1516 | 489 |  |
| ABCC4 ---- tag |  | C/G or G/G | 1.18 (0.99-1.42) | 0.07 | 0.66 | 0.60 | 618 | 51,98 | 302 | 54,22 | 1516 | 489 |  |
| ABCC4 ---- tag | rs1751051 | T/T | 1.00 (.-.) | . | . | . | 509 | 42,81 | 232 | 41,65 | 1516 | 489 |  |
| ABCC4 ---- tag |  | T/A or A/A | 0.96 (0.80-1.16) | 0.69 | 0.99 | 0.98 | 680 | 57,19 | 325 | 58,35 | 1516 | 489 |  |
| ABCC4 ---- tag | rs1764416 | G/G | 1.00 (.-.) | . | . | . | 1016 | 85,45 | 486 | 87,25 | 1516 | 489 |  |
| ABCC4 ---- tag |  | G/A or A/A | 0.87 (0.66-1.15) | 0.32 | 0.90 | 0.72 | 173 | 14,55 | 71 | 12,75 | 1516 | 489 |  |
| ABCC4 ---- tag | rs2274401 | T/T | 1.00 (.-.) | . | . | . | 730 | 61,40 | 358 | 64,27 | 1516 | 489 |  |
| ABCC4 ---- tag |  | T/C or C/C | 0.90 (0.74-1.08) | 0.26 | 0.87 | 0.71 | 459 | 38,60 | 199 | 35,73 | 1516 | 489 |  |
| ABCC4 ---- tag | rs2892716 | C/C | 1.00 (.-.) | . | . | . | 443 | 37,26 | 235 | 42,19 | 1516 | 489 |  |
| ABCC4 ---- tag |  | C/T or T/T | 1.04 (0.86-1.25) | 0.71 | 0.99 | 0.98 | 746 | 62,74 | 322 | 57,81 | 1516 | 489 |  |
| ABCC4 ---- tag | rs3782964 | C/C | 1.00 (.-.) | . | . | . | 782 | 65,77 | 397 | 71,27 | 1516 | 489 |  |
| ABCC4 ---- tag |  | C/T or T/T | 0.87 (0.71-1.06) | 0.17 | 0.85 | 0.70 | 407 | 34,23 | 160 | 28,73 | 1516 | 489 |  |
| ABCC4 ---- tag | rs3818494 | C/C | 1.00 (.-.) | . | . | . | 535 | 45,00 | 244 | 43,81 | 1516 | 489 |  |
| ABCC4 ---- tag |  | C/G or G/G | 1.24 (1.03-1.49) | 0.02 | 0.59 | 0.60 | 654 | 55,00 | 313 | 56,19 | 1516 | 489 |  |
| ABCC4 ---- tag | rs3864997 | G/G | 1.00 (.-.) | . | . | . | 303 | 25,48 | 147 | 26,39 | 1516 | 489 |  |
| ABCC4 ---- tag |  | G/T or T/T | 1.14 (0.93-1.40) | 0.20 | 0.85 | 0.70 | 886 | 74,52 | 410 | 73,61 | 1516 | 489 |  |
| ABCC4 ---- tag | rs4148421 | G/G | 1.00 (.-.) | . | . | . | 352 | 29,60 | 157 | 28,19 | 1516 | 489 |  |
| ABCC4 ---- tag |  | G/A or A/A | 0.99 (0.81-1.21) | 0.92 | 0.99 | 0.98 | 837 | 70,40 | 400 | 71,81 | 1516 | 489 |  |
| ABCC4 ---- tag | rs4148446 | G/G | 1.00 (.-.) | . | . | . | 388 | 32,63 | 206 | 36,98 | 1516 | 489 |  |
| ABCC4 ---- tag |  | G/A or A/A | 1.02 (0.85-1.23) | 0.83 | 0.99 | 0.98 | 801 | 67,37 | 351 | 63,02 | 1516 | 489 |  |
| ABCC4 ---- tag | rs4148455 | G/G | 1.00 (.-.) | . | . | . | 904 | 76,03 | 421 | 75,58 | 1516 | 489 |  |
| ABCC4 ---- tag |  | G/A or A/A | 1.06 (0.86-1.31) | 0.57 | 0.99 | 0.95 | 285 | 23,97 | 136 | 24,42 | 1516 | 489 |  |
| ABCC4 ---- tag | rs4148540 | C/C | 1.00 (.-.) | . | . | . | 1066 | 89,66 | 482 | 86,54 | 1516 | 489 |  |
| ABCC4 ---- tag |  | C/T or T/T | 1.07 (0.82-1.39) | 0.62 | 0.99 | 0.95 | 123 | 10,34 | 75 | 13,46 | 1516 | 489 |  |
| ABCC4 ---- tag | rs4148542 | G/G | 1.00 (.-.) | . | . | . | 333 | 28,01 | 146 | 26,21 | 1516 | 489 |  |
| ABCC4 ---- tag |  | G/A or A/A | 0.98 (0.80-1.20) | 0.85 | 0.99 | 0.98 | 856 | 71,99 | 411 | 73,79 | 1516 | 489 |  |
| ABCC4 ---- tag | rs4148544 | G/G | 1.00 (.-.) | . | . | . | 510 | 42,89 | 239 | 42,91 | 1516 | 489 |  |
| ABCC4 ---- tag |  | G/A or A/A | 0.98 (0.82-1.18) | 0.85 | 0.99 | 0.98 | 679 | 57,11 | 318 | 57,09 | 1516 | 489 |  |
| ABCC4 ---- tag | rs4283094 | C/C | 1.00 (.-.) | . | . | . | 309 | 25,99 | 126 | 22,62 | 1516 | 489 |  |
| ABCC4 ---- tag |  | C/G or G/G | 1.04 (0.84-1.29) | 0.70 | 0.99 | 0.98 | 880 | 74,01 | 431 | 77,38 | 1516 | 489 |  |
| ABCC4 ---- tag | rs4636781 | A/A | 1.00 (.-.) | . | . | . | 855 | 71,91 | 389 | 69,84 | 1516 | 489 |  |
| ABCC4 ---- tag |  | A/G or G/G | 1.16 (0.96-1.42) | 0.13 | 0.83 | 0.70 | 334 | 28,09 | 168 | 30,16 | 1516 | 489 |  |
| ABCC4 ---- tag | rs4771910 | T/T | 1.00 (.-.) | . | . | . | 576 | 48,44 | 281 | 50,45 | 1516 | 489 |  |
| ABCC4 ---- tag |  | T/C or C/C | 0.87 (0.72-1.04) | 0.12 | 0.83 | 0.70 | 613 | 51,56 | 276 | 49,55 | 1516 | 489 |  |
| ABCC4 ---- tag | rs4773850 | T/T | 1.00 (.-.) | . | . | . | 531 | 44,66 | 289 | 51,89 | 1516 | 489 |  |
| ABCC4 ---- tag |  | T/G or G/G | 0.81 (0.68-0.97) | 0.02 | 0.59 | 0.60 | 658 | 55,34 | 268 | 48,11 | 1516 | 489 |  |
| ABCC4 ---- tag | rs7981095 | A/A | 1.00 (.-.) | . | . | . | 772 | 64,93 | 353 | 63,38 | 1516 | 489 |  |
| ABCC4 ---- tag |  | A/T or T/T | 0.95 (0.78-1.15) | 0.58 | 0.99 | 0.95 | 417 | 35,07 | 204 | 36,62 | 1516 | 489 |  |
| ABCC4 ---- tag | rs8001444 | C/C | 1.00 (.-.) | . | . | . | 410 | 34,48 | 187 | 33,57 | 1516 | 489 |  |
| ABCC4 ---- tag |  | C/T or T/T | 0.92 (0.76-1.12) | 0.40 | 0.93 | 0.81 | 779 | 65,52 | 370 | 66,43 | 1516 | 489 |  |
| ABCC4 ---- tag | rs931111 | T/T | 1.00 (.-.) | . | . | . | 799 | 67,20 | 373 | 66,97 | 1516 | 489 |  |
| ABCC4 ---- tag |  | T/C or C/C | 1.01 (0.83-1.22) | 0.93 | 0.99 | 0.98 | 390 | 32,80 | 184 | 33,03 | 1516 | 489 |  |
| ABCC4 ---- tag | rs943288 | T/T | 1.00 (.-.) | . | . | . | 910 | 76,53 | 424 | 76,12 | 1516 | 489 |  |
| ABCC4 ---- tag |  | T/A or A/A | 1.04 (0.84-1.29) | 0.71 | 0.99 | 0.98 | 279 | 23,47 | 133 | 23,88 | 1516 | 489 |  |
| ABCC4 ---- tag | rs943290 | A/A | 1.00 (.-.) | . | . | . | 643 | 54,08 | 305 | 54,76 | 1516 | 489 |  |
| ABCC4 ---- tag |  | A/G or G/G | 0.87 (0.73-1.05) | 0.14 | 0.83 | 0.70 | 546 | 45,92 | 252 | 45,24 | 1516 | 489 |  |
| ABCC4 ---- tag | rs9516530 | C/C | 1.00 (.-.) | . | . | . | 648 | 54,50 | 310 | 55,66 | 1516 | 489 |  |
| ABCC4 ---- tag |  | C/T or T/T | 0.90 (0.76-1.08) | 0.28 | 0.88 | 0.71 | 541 | 45,50 | 247 | 44,34 | 1516 | 489 |  |
| ABCC4 ---- tag | rs9516551 | C/C | 1.00 (.-.) | . | . | . | 947 | 79,65 | 440 | 78,99 | 1516 | 489 |  |
| ABCC4 ---- tag |  | C/A or A/A | 0.81 (0.65-1.01) | 0.06 | 0.66 | 0.60 | 242 | 20,35 | 117 | 21,01 | 1516 | 489 |  |
| ABCC4 ---- tag | rs9524822 | T/T | 1.00 (.-.) | . | . | . | 764 | 64,26 | 367 | 65,89 | 1516 | 489 |  |
| ABCC4 ---- tag |  | T/C or C/C | 0.89 (0.73-1.07) | 0.21 | 0.85 | 0.70 | 425 | 35,74 | 190 | 34,11 | 1516 | 489 |  |
| ABCC4 ---- tag | rs9524861 | G/G | 1.00 (.-.) | . | . | . | 619 | 52,06 | 270 | 48,47 | 1516 | 489 |  |
| ABCC4 ---- tag |  | G/C or C/C | 0.97 (0.81-1.17) | 0.77 | 0.99 | 0.98 | 570 | 47,94 | 287 | 51,53 | 1516 | 489 |  |
| ABCC4 ---- tag | rs9524902 | T/T | 1.00 (.-.) | . | . | . | 324 | 27,25 | 163 | 29,26 | 1516 | 489 |  |
| ABCC4 ---- tag |  | T/C or C/C | 0.83 (0.68-1.01) | 0.06 | 0.66 | 0.60 | 865 | 72,75 | 394 | 70,74 | 1516 | 489 |  |
| ABCC4 ---- tag | rs9556455 | G/G | 1.00 (.-.) | . | . | . | 905 | 76,11 | 413 | 74,15 | 1516 | 489 |  |
| ABCC4 ---- tag |  | G/A or A/A | 1.15 (0.94-1.41) | 0.16 | 0.84 | 0.70 | 284 | 23,89 | 144 | 25,85 | 1516 | 489 |  |
| ABCC4 ---- NA | rs9561778 | G/G | 1.00 (.-.) | . | . | . | 772 | 64,93 | 370 | 66,43 | 1516 | 489 |  |
| ABCC4 ---- NA |  | G/T or T/T | 1.01 (0.83-1.22) | 0.93 | 0.99 | 0.98 | 417 | 35,07 | 187 | 33,57 | 1516 | 489 |  |
| ABCC4 ---- tag | rs9561811 | C/C | 1.00 (.-.) | . | . | . | 787 | 66,19 | 379 | 68,04 | 1516 | 489 |  |
| ABCC4 ---- tag |  | C/T or T/T | 1.11 (0.92-1.34) | 0.29 | 0.88 | 0.71 | 402 | 33,81 | 178 | 31,96 | 1516 | 489 |  |
| ABCC4 ---- tag | rs9590183 | T/T | 1.00 (.-.) | . | . | . | 1038 | 87,30 | 494 | 88,69 | 1516 | 489 |  |
| ABCC4 ---- tag |  | T/A or A/A | 0.90 (0.67-1.19) | 0.45 | 0.93 | 0.87 | 151 | 12,70 | 63 | 11,31 | 1516 | 489 |  |
| ABCC4 ---- tag | rs997777 | T/T | 1.00 (.-.) | . | . | . | 600 | 50,46 | 271 | 48,65 | 1516 | 489 |  |
| ABCC4 ---- tag |  | T/A or A/A | 1.01 (0.84-1.21) | 0.94 | 0.99 | 0.98 | 589 | 49,54 | 286 | 51,35 | 1516 | 489 |  |
| ADH1B ---- tag | rs1159918 | G/G | 1.00 (.-.) | . | . | . | 527 | 44,32 | 255 | 45,78 | 1516 | 489 |  |
| ADH1B ---- tag |  | G/T or T/T | 0.92 (0.77-1.10) | 0.37 | 0.93 | 0.64 | 662 | 55,68 | 302 | 54,22 | 1516 | 489 |  |
| ADH1B ---- candidate literature | rs1229984 | G/G | 1.00 (.-.) | . | . | . | 1076 | 90,50 | 505 | 90,66 | 1516 | 489 |  |
| ADH1B ---- candidate literature |  | G/A or A/A | 0.90 (0.66-1.22) | 0.51 | 0.96 | 0.64 | 113 | 9,50 | 52 | 9,34 | 1516 | 489 |  |
| ADH1B ---- tag | rs12507573 | C/C | 1.00 (.-.) | . | . | . | 381 | 32,04 | 159 | 28,55 | 1516 | 489 |  |
| ADH1B ---- tag |  | C/A or A/A | 1.02 (0.84-1.24) | 0.85 | 0.99 | 0.85 | 808 | 67,96 | 398 | 71,45 | 1516 | 489 |  |
| ADH1B ---- tag | rs1693457 | T/T | 1.00 (.-.) | . | . | . | 820 | 68,97 | 401 | 71,99 | 1516 | 489 |  |
| ADH1B ---- tag |  | T/C or C/C | 0.83 (0.68-1.02) | 0.07 | 0.66 | 0.37 | 369 | 31,03 | 156 | 28,01 | 1516 | 489 |  |
| ADH1B ---- tag | rs2066701 | C/C | 1.00 (.-.) | . | . | . | 542 | 45,58 | 243 | 43,63 | 1516 | 489 |  |
| ADH1B ---- tag |  | C/T or T/T | 1.07 (0.90-1.28) | 0.45 | 0.93 | 0.64 | 647 | 54,42 | 314 | 56,37 | 1516 | 489 |  |
| ADH1C ---- tag | rs11936869 | C/C | 1.00 (.-.) | . | . | . | 606 | 50,97 | 301 | 54,04 | 1516 | 489 |  |
| ADH1C ---- tag |  | C/G or G/G | 0.89 (0.74-1.06) | 0.19 | 0.85 | 0.63 | 583 | 49,03 | 256 | 45,96 | 1516 | 489 |  |
| ADH1C ---- tag | rs1229849 | T/T | 1.00 (.-.) | . | . | . | 637 | 53,57 | 285 | 51,17 | 1516 | 489 |  |
| ADH1C ---- tag |  | T/A or A/A | 1.02 (0.85-1.22) | 0.87 | 0.99 | 0.95 | 552 | 46,43 | 272 | 48,83 | 1516 | 489 |  |
| ADH1C ---- tag | rs1229863 | A/A | 1.00 (.-.) | . | . | . | 873 | 73,42 | 406 | 72,89 | 1516 | 489 |  |
| ADH1C ---- tag |  | A/T or T/T | 0.94 (0.76-1.15) | 0.54 | 0.97 | 0.69 | 316 | 26,58 | 151 | 27,11 | 1516 | 489 |  |
| ADH1C ---- tag | rs1229980 | C/C | 1.00 (.-.) | . | . | . | 1073 | 90,24 | 490 | 87,97 | 1516 | 489 |  |
| ADH1C ---- tag |  | C/G or G/G | 1.14 (0.86-1.50) | 0.36 | 0.93 | 0.63 | 116 | 9,76 | 67 | 12,03 | 1516 | 489 |  |
| ADH1C ---- candidate | rs1693482 | C/C | 1.00 (.-.) | . | . | . | 491 | 41,30 | 229 | 41,11 | 1516 | 489 |  |
| ADH1C ---- candidate |  | C/T or T/T | 0.93 (0.77-1.11) | 0.42 | 0.93 | 0.63 | 698 | 58,70 | 328 | 58,89 | 1516 | 489 |  |
| ADH1C ---- tag | rs2173201 | C/C | 1.00 (.-.) | . | . | . | 676 | 56,85 | 329 | 59,07 | 1516 | 489 |  |
| ADH1C ---- tag |  | C/A or A/A | 0.92 (0.77-1.11) | 0.39 | 0.93 | 0.63 | 513 | 43,15 | 228 | 40,93 | 1516 | 489 |  |
| ADH1C ---- tag | rs2298753 | T/T | 1.00 (.-.) | . | . | . | 977 | 82,17 | 465 | 83,48 | 1516 | 489 |  |
| ADH1C ---- tag |  | T/C or C/C | 0.86 (0.68-1.10) | 0.24 | 0.86 | 0.63 | 212 | 17,83 | 92 | 16,52 | 1516 | 489 |  |
| ADH1C ---- tag | rs2866152 | G/G | 1.00 (.-.) | . | . | . | 727 | 61,14 | 331 | 59,43 | 1516 | 489 |  |
| ADH1C ---- tag |  | G/C or C/C | 0.99 (0.83-1.19) | 0.95 | 0.99 | 0.95 | 462 | 38,86 | 226 | 40,57 | 1516 | 489 |  |
| ADH1C ---- tag | rs904096 | T/T | 1.00 (.-.) | . | . | . | 484 | 40,71 | 227 | 40,75 | 1516 | 489 |  |
| ADH1C ---- tag |  | T/G or G/G | 0.92 (0.76-1.10) | 0.37 | 0.93 | 0.63 | 705 | 59,29 | 330 | 59,25 | 1516 | 489 |  |
| BHMT ---- tag | rs10944 | A/A | 1.00 (.-.) | . | . | . | 283 | 23,80 | 136 | 24,42 | 1516 | 489 |  |
| BHMT ---- tag |  | A/C or C/C | 0.92 (0.74-1.13) | 0.41 | 0.93 | 0.72 | 906 | 76,20 | 421 | 75,58 | 1516 | 489 |  |
| BHMT ---- tag | rs12655567 | C/C | 1.00 (.-.) | . | . | . | 434 | 36,50 | 218 | 39,14 | 1516 | 489 |  |
| BHMT ---- tag |  | C/G or G/G | 0.84 (0.70-1.01) | 0.06 | 0.66 | 0.21 | 755 | 63,50 | 339 | 60,86 | 1516 | 489 |  |
| BHMT ---- tag | rs1291041 | G/G | 1.00 (.-.) | . | . | . | 498 | 41,88 | 247 | 44,34 | 1516 | 489 |  |
| BHMT ---- tag |  | G/T or T/T | 0.82 (0.69-0.99) | 0.04 | 0.59 | 0.21 | 691 | 58,12 | 310 | 55,66 | 1516 | 489 |  |
| BHMT ---- tag | rs16876500 | C/C | 1.00 (.-.) | . | . | . | 944 | 79,39 | 444 | 79,71 | 1516 | 489 |  |
| BHMT ---- tag |  | C/T or T/T | 1.05 (0.84-1.31) | 0.69 | 0.99 | 0.72 | 245 | 20,61 | 113 | 20,29 | 1516 | 489 |  |
| BHMT ---- tag | rs492842 | A/A | 1.00 (.-.) | . | . | . | 437 | 36,75 | 215 | 38,60 | 1516 | 489 |  |
| BHMT ---- tag |  | A/G or G/G | 0.96 (0.80-1.15) | 0.63 | 0.99 | 0.72 | 752 | 63,25 | 342 | 61,40 | 1516 | 489 |  |
| BHMT ---- tag | rs558133 | T/T | 1.00 (.-.) | . | . | . | 566 | 47,60 | 270 | 48,47 | 1516 | 489 |  |
| BHMT ---- tag |  | T/G or G/G | 0.92 (0.77-1.10) | 0.36 | 0.93 | 0.72 | 623 | 52,40 | 287 | 51,53 | 1516 | 489 |  |
| BHMT ---- tag | rs9637824 | A/A | 1.00 (.-.) | . | . | . | 438 | 36,84 | 209 | 37,52 | 1516 | 489 |  |
| BHMT ---- tag |  | A/G or G/G | 0.97 (0.80-1.16) | 0.72 | 0.99 | 0.72 | 751 | 63,16 | 348 | 62,48 | 1516 | 489 |  |
| BHMT2 ---- tag | rs16876512 | C/C | 1.00 (.-.) | . | . | . | 939 | 78,97 | 441 | 79,17 | 1516 | 489 |  |
| BHMT2 ---- tag |  | C/T or T/T | 1.06 (0.85-1.32) | 0.59 | 0.99 | 0.71 | 250 | 21,03 | 116 | 20,83 | 1516 | 489 |  |
| BHMT2 ---- tag | rs2461248 | A/A | 1.00 (.-.) | . | . | . | 279 | 23,47 | 134 | 24,06 | 1516 | 489 |  |
| BHMT2 ---- tag |  | A/T or T/T | 0.92 (0.75-1.14) | 0.47 | 0.94 | 0.70 | 910 | 76,53 | 423 | 75,94 | 1516 | 489 |  |
| BHMT2 ---- tag | rs2909856 | T/T | 1.00 (.-.) | . | . | . | 478 | 40,20 | 233 | 41,83 | 1516 | 489 |  |
| BHMT2 ---- tag |  | T/C or C/C | 0.92 (0.77-1.10) | 0.36 | 0.93 | 0.70 | 711 | 59,80 | 324 | 58,17 | 1516 | 489 |  |
| BHMT2 ---- tag | rs476620 | A/A | 1.00 (.-.) | . | . | . | 438 | 36,84 | 209 | 37,52 | 1516 | 489 |  |
| BHMT2 ---- tag |  | A/G or G/G | 0.97 (0.80-1.16) | 0.71 | 0.99 | 0.71 | 751 | 63,16 | 348 | 62,48 | 1516 | 489 |  |
| BHMT2 ---- candidate literature | rs626105 | G/G | 1.00 (.-.) | . | . | . | 745 | 62,66 | 349 | 62,66 | 1516 | 489 |  |
| BHMT2 ---- candidate literature |  | G/A or A/A | 0.91 (0.75-1.09) | 0.30 | 0.89 | 0.70 | 444 | 37,34 | 208 | 37,34 | 1516 | 489 |  |
| BHMT2 ---- tag | rs631305 | G/G | 1.00 (.-.) | . | . | . | 816 | 68,63 | 388 | 69,66 | 1516 | 489 |  |
| BHMT2 ---- tag |  | G/A or A/A | 0.91 (0.75-1.11) | 0.37 | 0.93 | 0.70 | 373 | 31,37 | 169 | 30,34 | 1516 | 489 |  |
| CBS ---- tag | rs11701048 | C/C | 1.00 (.-.) | . | . | . | 1008 | 84,78 | 482 | 86,54 | 1516 | 489 |  |
| CBS ---- tag |  | C/T or T/T | 0.96 (0.74-1.25) | 0.78 | 0.99 | 0.79 | 181 | 15,22 | 75 | 13,46 | 1516 | 489 |  |
| CBS ---- tag | rs234706 | G/G | 1.00 (.-.) | . | . | . | 524 | 44,07 | 240 | 43,09 | 1516 | 489 |  |
| CBS ---- tag |  | G/A or A/A | 1.03 (0.86-1.23) | 0.74 | 0.99 | 0.79 | 665 | 55,93 | 317 | 56,91 | 1516 | 489 |  |
| CBS ---- tag | rs234711 | C/C | 1.00 (.-.) | . | . | . | 714 | 60,05 | 320 | 57,45 | 1516 | 489 |  |
| CBS ---- tag |  | C/A or A/A | 1.09 (0.91-1.31) | 0.35 | 0.93 | 0.79 | 475 | 39,95 | 237 | 42,55 | 1516 | 489 |  |
| CBS ---- candidate literature | rs234713 | G/G | 1.00 (.-.) | . | . | . | 593 | 49,87 | 273 | 49,01 | 1516 | 489 |  |
| CBS ---- candidate literature |  | G/A or A/A | 1.04 (0.87-1.24) | 0.68 | 0.99 | 0.79 | 596 | 50,13 | 284 | 50,99 | 1516 | 489 |  |
| CBS ---- tag | rs2839623 | T/T | 1.00 (.-.) | . | . | . | 990 | 83,26 | 451 | 80,97 | 1516 | 489 |  |
| CBS ---- tag |  | T/A or A/A | 1.16 (0.92-1.46) | 0.21 | 0.85 | 0.71 | 199 | 16,74 | 106 | 19,03 | 1516 | 489 |  |
| CBS ---- tag | rs2839626 | C/C | 1.00 (.-.) | . | . | . | 545 | 45,84 | 249 | 44,70 | 1516 | 489 |  |
| CBS ---- tag |  | C/T or T/T | 1.02 (0.86-1.23) | 0.79 | 0.99 | 0.79 | 644 | 54,16 | 308 | 55,30 | 1516 | 489 |  |
| CBS ---- tag | rs422791 | T/T | 1.00 (.-.) | . | . | . | 606 | 50,97 | 274 | 49,19 | 1516 | 489 |  |
| CBS ---- tag |  | T/C or C/C | 1.23 (1.02-1.47) | 0.03 | 0.59 | 0.14 | 583 | 49,03 | 283 | 50,81 | 1516 | 489 |  |
| CBS ---- tag | rs706209 | C/C | 1.00 (.-.) | . | . | . | 371 | 31,20 | 161 | 28,90 | 1516 | 489 |  |
| CBS ---- tag |  | C/T or T/T | 0.95 (0.78-1.17) | 0.65 | 0.99 | 0.79 | 818 | 68,80 | 396 | 71,10 | 1516 | 489 |  |
| CBS ---- tag | rs719037 | A/A | 1.00 (.-.) | . | . | . | 416 | 34,99 | 175 | 31,42 | 1516 | 489 |  |
| CBS ---- tag |  | A/G or G/G | 1.25 (1.03-1.51) | 0.03 | 0.59 | 0.14 | 773 | 65,01 | 382 | 68,58 | 1516 | 489 |  |
| CBS ---- tag | rs719038 | T/T | 1.00 (.-.) | . | . | . | 515 | 43,31 | 222 | 39,86 | 1516 | 489 |  |
| CBS ---- tag |  | T/C or C/C | 0.97 (0.81-1.16) | 0.75 | 0.99 | 0.79 | 674 | 56,69 | 335 | 60,14 | 1516 | 489 |  |
| DHFR ---- tag | rs10474632 | G/G | 1.00 (.-.) | . | . | . | 996 | 83,77 | 478 | 85,82 | 1516 | 489 |  |
| DHFR ---- tag |  | G/A or A/A | 0.74 (0.57-0.95) | 0.02 | 0.59 | 0.20 | 193 | 16,23 | 79 | 14,18 | 1516 | 489 |  |
| DHFR ---- tag | rs11951910 | T/T | 1.00 (.-.) | . | . | . | 961 | 80,82 | 445 | 79,89 | 1516 | 489 |  |
| DHFR ---- tag |  | T/C or C/C | 1.05 (0.83-1.31) | 0.70 | 0.99 | 0.89 | 228 | 19,18 | 112 | 20,11 | 1516 | 489 |  |
| DHFR ---- tag | rs1643665 | T/T | 1.00 (.-.) | . | . | . | 564 | 47,43 | 256 | 45,96 | 1516 | 489 |  |
| DHFR ---- tag |  | T/C or C/C | 1.02 (0.86-1.22) | 0.80 | 0.99 | 0.89 | 625 | 52,57 | 301 | 54,04 | 1516 | 489 |  |
| DHFR ---- tag | rs1650717 | T/T | 1.00 (.-.) | . | . | . | 648 | 54,50 | 291 | 52,24 | 1516 | 489 |  |
| DHFR ---- tag |  | T/G or G/G | 1.12 (0.94-1.34) | 0.22 | 0.85 | 0.59 | 541 | 45,50 | 266 | 47,76 | 1516 | 489 |  |
| DHFR ---- tag | rs1805355 | G/G | 1.00 (.-.) | . | . | . | 1039 | 87,38 | 485 | 87,07 | 1516 | 489 |  |
| DHFR ---- tag |  | G/A or A/A | 0.98 (0.75-1.29) | 0.91 | 0.99 | 0.91 | 150 | 12,62 | 72 | 12,93 | 1516 | 489 |  |
| DHFR ---- tag | rs6151617 | A/A | 1.00 (.-.) | . | . | . | 437 | 36,75 | 205 | 36,80 | 1516 | 489 |  |
| DHFR ---- tag |  | A/G or G/G | 0.94 (0.78-1.13) | 0.49 | 0.95 | 0.89 | 752 | 63,25 | 352 | 63,20 | 1516 | 489 |  |
| DHFR ---- tag | rs6864493 | T/T | 1.00 (.-.) | . | . | . | 677 | 56,94 | 317 | 56,91 | 1516 | 489 |  |
| DHFR ---- tag |  | T/C or C/C | 0.97 (0.81-1.16) | 0.75 | 0.99 | 0.89 | 512 | 43,06 | 240 | 43,09 | 1516 | 489 |  |
| DHFR ---- tag | rs836788 | G/G | 1.00 (.-.) | . | . | . | 507 | 42,64 | 239 | 42,91 | 1516 | 489 |  |
| DHFR ---- tag |  | G/A or A/A | 0.97 (0.81-1.16) | 0.74 | 0.99 | 0.89 | 682 | 57,36 | 318 | 57,09 | 1516 | 489 |  |
| DHFR ---- tag | rs836790 | A/A | 1.00 (.-.) | . | . | . | 839 | 70,56 | 377 | 67,68 | 1516 | 489 |  |
| DHFR ---- tag |  | A/G or G/G | 1.12 (0.93-1.36) | 0.24 | 0.85 | 0.59 | 350 | 29,44 | 180 | 32,32 | 1516 | 489 |  |
| DHFR ---- tag | rs836817 | G/G | 1.00 (.-.) | . | . | . | 560 | 47,10 | 251 | 45,06 | 1516 | 489 |  |
| DHFR ---- tag |  | G/T or T/T | 1.12 (0.93-1.34) | 0.23 | 0.85 | 0.59 | 629 | 52,90 | 306 | 54,94 | 1516 | 489 |  |
| DNMT1 ---- candidate | rs2228612 | A/A | 1.00 (.-.) | . | . | . | 1028 | 86,46 | 482 | 86,54 | 1516 | 489 |  |
| DNMT1 ---- candidate |  | A/G or G/G | 1.11 (0.85-1.45) | 0.43 | 0.93 | 0.43 | 161 | 13,54 | 75 | 13,46 | 1516 | 489 |  |
| DNMT3A ---- tag | rs10460566 | A/A | 1.00 (.-.) | . | . | . | 671 | 56,43 | 335 | 60,14 | 1516 | 489 |  |
| DNMT3A ---- tag |  | A/G or G/G | 0.97 (0.81-1.17) | 0.76 | 0.99 | 0.92 | 518 | 43,57 | 222 | 39,86 | 1516 | 489 |  |
| DNMT3A ---- candidate literature | rs11695471 | T/T | 1.00 (.-.) | . | . | . | 536 | 45,08 | 236 | 42,37 | 1516 | 489 |  |
| DNMT3A ---- candidate literature |  | T/A or A/A | 1.10 (0.92-1.32) | 0.30 | 0.89 | 0.89 | 653 | 54,92 | 321 | 57,63 | 1516 | 489 |  |
| DNMT3A ---- tag | rs11887120 | C/C | 1.00 (.-.) | . | . | . | 416 | 34,99 | 195 | 35,01 | 1516 | 489 |  |
| DNMT3A ---- tag |  | C/T or T/T | 0.98 (0.81-1.18) | 0.82 | 0.99 | 0.92 | 773 | 65,01 | 362 | 64,99 | 1516 | 489 |  |
| DNMT3A ---- tag | rs12991495 | T/T | 1.00 (.-.) | . | . | . | 585 | 49,20 | 248 | 44,52 | 1516 | 489 |  |
| DNMT3A ---- tag |  | T/C or C/C | 1.11 (0.93-1.33) | 0.25 | 0.86 | 0.89 | 604 | 50,80 | 309 | 55,48 | 1516 | 489 |  |
| DNMT3A ---- tag | rs13401241 | A/A | 1.00 (.-.) | . | . | . | 342 | 28,76 | 149 | 26,75 | 1516 | 489 |  |
| DNMT3A ---- tag |  | A/C or C/C | 1.01 (0.82-1.24) | 0.92 | 0.99 | 0.92 | 847 | 71,24 | 408 | 73,25 | 1516 | 489 |  |
| DNMT3A ---- candidate literature | rs13420827 | C/C | 1.00 (.-.) | . | . | . | 784 | 65,94 | 374 | 67,15 | 1516 | 489 |  |
| DNMT3A ---- candidate literature |  | C/G or G/G | 0.94 (0.78-1.14) | 0.56 | 0.99 | 0.89 | 405 | 34,06 | 183 | 32,85 | 1516 | 489 |  |
| DNMT3A ---- tag | rs13428812 | A/A | 1.00 (.-.) | . | . | . | 550 | 46,26 | 277 | 49,73 | 1516 | 489 |  |
| DNMT3A ---- tag |  | A/G or G/G | 1.07 (0.89-1.29) | 0.45 | 0.93 | 0.89 | 639 | 53,74 | 280 | 50,27 | 1516 | 489 |  |
| DNMT3A ---- tag | rs4665287 | C/C | 1.00 (.-.) | . | . | . | 796 | 66,95 | 380 | 68,22 | 1516 | 489 |  |
| DNMT3A ---- tag |  | C/T or T/T | 0.93 (0.77-1.13) | 0.48 | 0.95 | 0.89 | 393 | 33,05 | 177 | 31,78 | 1516 | 489 |  |
| DNMT3B ---- tag | rs13045669 | A/A | 1.00 (.-.) | . | . | . | 1099 | 92,43 | 519 | 93,18 | 1516 | 489 |  |
| DNMT3B ---- tag |  | A/G or G/G | 0.85 (0.58-1.26) | 0.43 | 0.93 | 0.97 | 90 | 7,57 | 38 | 6,82 | 1516 | 489 |  |
| DNMT3B ---- tag | rs17123673 | A/A | 1.00 (.-.) | . | . | . | 1092 | 91,84 | 507 | 91,02 | 1516 | 489 |  |
| DNMT3B ---- tag |  | A/G or G/G | 0.81 (0.59-1.11) | 0.19 | 0.85 | 0.97 | 97 | 8,16 | 50 | 8,98 | 1516 | 489 |  |
| DNMT3B ---- tag | rs183603 | A/A | 1.00 (.-.) | . | . | . | 652 | 54,84 | 313 | 56,19 | 1516 | 489 |  |
| DNMT3B ---- tag |  | A/G or G/G | 0.90 (0.75-1.08) | 0.27 | 0.88 | 0.97 | 537 | 45,16 | 244 | 43,81 | 1516 | 489 |  |
| DNMT3B ---- tag | rs2235760 | C/C | 1.00 (.-.) | . | . | . | 845 | 71,07 | 391 | 70,20 | 1516 | 489 |  |
| DNMT3B ---- tag |  | C/T or T/T | 1.03 (0.85-1.26) | 0.74 | 0.99 | 0.97 | 344 | 28,93 | 166 | 29,80 | 1516 | 489 |  |
| DNMT3B ---- tag | rs2424908 | C/C | 1.00 (.-.) | . | . | . | 761 | 64,00 | 371 | 66,61 | 1516 | 489 |  |
| DNMT3B ---- tag |  | C/T or T/T | 0.97 (0.80-1.17) | 0.74 | 0.99 | 0.97 | 428 | 36,00 | 186 | 33,39 | 1516 | 489 |  |
| DNMT3B ---- candidate literature | rs2424909 | T/T | 1.00 (.-.) | . | . | . | 450 | 37,85 | 225 | 40,39 | 1516 | 489 |  |
| DNMT3B ---- candidate literature |  | T/C or C/C | 1.02 (0.85-1.22) | 0.87 | 0.99 | 0.97 | 739 | 62,15 | 332 | 59,61 | 1516 | 489 |  |
| DNMT3B ---- tag | rs4911108 | A/A | 1.00 (.-.) | . | . | . | 485 | 40,79 | 237 | 42,55 | 1516 | 489 |  |
| DNMT3B ---- tag |  | A/G or G/G | 1.00 (0.84-1.20) | 0.97 | 0.99 | 0.97 | 704 | 59,21 | 320 | 57,45 | 1516 | 489 |  |
| DNMT3B ---- tag | rs6058896 | C/C | 1.00 (.-.) | . | . | . | 1059 | 89,07 | 494 | 88,69 | 1516 | 489 |  |
| DNMT3B ---- tag |  | C/T or T/T | 1.04 (0.78-1.38) | 0.80 | 0.99 | 0.97 | 130 | 10,93 | 63 | 11,31 | 1516 | 489 |  |
| DNMT3B ---- tag | rs6119954 | G/G | 1.00 (.-.) | . | . | . | 818 | 68,80 | 390 | 70,02 | 1516 | 489 |  |
| DNMT3B ---- tag |  | G/A or A/A | 0.99 (0.81-1.20) | 0.91 | 0.99 | 0.97 | 371 | 31,20 | 167 | 29,98 | 1516 | 489 |  |
| DNMT3B ---- tag | rs6579038 | A/A | 1.00 (.-.) | . | . | . | 1051 | 88,39 | 493 | 88,51 | 1516 | 489 |  |
| DNMT3B ---- tag |  | A/G or G/G | 1.02 (0.77-1.35) | 0.90 | 0.99 | 0.97 | 138 | 11,61 | 64 | 11,49 | 1516 | 489 |  |
| DPYD ---- tag | rs1034215 | C/C | 1.00 (.-.) | . | . | . | 717 | 60,30 | 358 | 64,27 | 1516 | 489 |  |
| DPYD ---- tag |  | C/T or T/T | 0.90 (0.74-1.08) | 0.26 | 0.87 | 0.53 | 472 | 39,70 | 199 | 35,73 | 1516 | 489 |  |
| DPYD ---- tag | rs10783058 | T/T | 1.00 (.-.) | . | . | . | 519 | 43,65 | 212 | 38,06 | 1516 | 489 |  |
| DPYD ---- tag |  | T/C or C/C | 1.28 (1.06-1.54) | 0.01 | 0.59 | 0.29 | 670 | 56,35 | 345 | 61,94 | 1516 | 489 |  |
| DPYD ---- tag | rs10783070 | C/C | 1.00 (.-.) | . | . | . | 828 | 69,64 | 394 | 70,74 | 1516 | 489 |  |
| DPYD ---- tag |  | C/T or T/T | 1.16 (0.96-1.41) | 0.13 | 0.83 | 0.51 | 361 | 30,36 | 163 | 29,26 | 1516 | 489 |  |
| DPYD ---- tag | rs10875048 | G/G | 1.00 (.-.) | . | . | . | 790 | 66,44 | 370 | 66,43 | 1516 | 489 |  |
| DPYD ---- tag |  | G/A or A/A | 0.92 (0.76-1.11) | 0.38 | 0.93 | 0.63 | 399 | 33,56 | 187 | 33,57 | 1516 | 489 |  |
| DPYD ---- tag | rs10875055 | C/C | 1.00 (.-.) | . | . | . | 355 | 29,86 | 129 | 23,16 | 1516 | 489 |  |
| DPYD ---- tag |  | C/T or T/T | 1.32 (1.07-1.63) | 0.01 | 0.59 | 0.29 | 834 | 70,14 | 428 | 76,84 | 1516 | 489 |  |
| DPYD ---- tag | rs10875079 | A/A | 1.00 (.-.) | . | . | . | 313 | 26,32 | 148 | 26,57 | 1516 | 489 |  |
| DPYD ---- tag |  | A/G or G/G | 0.95 (0.77-1.16) | 0.59 | 0.99 | 0.81 | 876 | 73,68 | 409 | 73,43 | 1516 | 489 |  |
| DPYD ---- tag | rs10875085 | A/A | 1.00 (.-.) | . | . | . | 805 | 67,70 | 378 | 67,86 | 1516 | 489 |  |
| DPYD ---- tag |  | A/T or T/T | 0.93 (0.77-1.12) | 0.44 | 0.93 | 0.69 | 384 | 32,30 | 179 | 32,14 | 1516 | 489 |  |
| DPYD ---- tag | rs10875097 | G/G | 1.00 (.-.) | . | . | . | 833 | 70,06 | 365 | 65,53 | 1516 | 489 |  |
| DPYD ---- tag |  | G/A or A/A | 1.22 (1.00-1.47) | 0.05 | 0.59 | 0.51 | 356 | 29,94 | 192 | 34,47 | 1516 | 489 |  |
| DPYD ---- tag | rs11165781 | T/T | 1.00 (.-.) | . | . | . | 802 | 67,45 | 382 | 68,58 | 1516 | 489 |  |
| DPYD ---- tag |  | T/C or C/C | 0.95 (0.78-1.15) | 0.60 | 0.99 | 0.81 | 387 | 32,55 | 175 | 31,42 | 1516 | 489 |  |
| DPYD ---- tag | rs11165783 | T/T | 1.00 (.-.) | . | . | . | 658 | 55,34 | 303 | 54,40 | 1516 | 489 |  |
| DPYD ---- tag |  | T/C or C/C | 1.14 (0.95-1.37) | 0.15 | 0.83 | 0.51 | 531 | 44,66 | 254 | 45,60 | 1516 | 489 |  |
| DPYD ---- tag | rs11165873 | A/A | 1.00 (.-.) | . | . | . | 316 | 26,58 | 169 | 30,34 | 1516 | 489 |  |
| DPYD ---- tag |  | A/T or T/T | 0.87 (0.71-1.05) | 0.15 | 0.83 | 0.51 | 873 | 73,42 | 388 | 69,66 | 1516 | 489 |  |
| DPYD ---- tag | rs11165875 | T/T | 1.00 (.-.) | . | . | . | 489 | 41,13 | 209 | 37,52 | 1516 | 489 |  |
| DPYD ---- tag |  | T/C or C/C | 1.13 (0.94-1.36) | 0.19 | 0.85 | 0.51 | 700 | 58,87 | 348 | 62,48 | 1516 | 489 |  |
| DPYD ---- tag | rs11165881 | T/T | 1.00 (.-.) | . | . | . | 417 | 35,07 | 204 | 36,62 | 1516 | 489 |  |
| DPYD ---- tag |  | T/C or C/C | 1.07 (0.89-1.29) | 0.46 | 0.94 | 0.71 | 772 | 64,93 | 353 | 63,38 | 1516 | 489 |  |
| DPYD ---- tag | rs11587873 | C/C | 1.00 (.-.) | . | . | . | 659 | 55,42 | 340 | 61,04 | 1516 | 489 |  |
| DPYD ---- tag |  | C/T or T/T | 0.88 (0.73-1.06) | 0.18 | 0.85 | 0.51 | 530 | 44,58 | 217 | 38,96 | 1516 | 489 |  |
| DPYD ---- tag | rs12030174 | C/C | 1.00 (.-.) | . | . | . | 861 | 72,41 | 401 | 71,99 | 1516 | 489 |  |
| DPYD ---- tag |  | C/T or T/T | 0.91 (0.74-1.11) | 0.34 | 0.92 | 0.61 | 328 | 27,59 | 156 | 28,01 | 1516 | 489 |  |
| DPYD ---- tag | rs12046744 | A/A | 1.00 (.-.) | . | . | . | 638 | 53,66 | 305 | 54,76 | 1516 | 489 |  |
| DPYD ---- tag |  | A/C or C/C | 0.97 (0.81-1.17) | 0.78 | 0.99 | 0.92 | 551 | 46,34 | 252 | 45,24 | 1516 | 489 |  |
| DPYD ---- tag | rs12047910 | G/G | 1.00 (.-.) | . | . | . | 853 | 71,74 | 413 | 74,15 | 1516 | 489 |  |
| DPYD ---- tag |  | G/A or A/A | 0.88 (0.72-1.07) | 0.20 | 0.85 | 0.51 | 336 | 28,26 | 144 | 25,85 | 1516 | 489 |  |
| DPYD ---- tag | rs12073044 | T/T | 1.00 (.-.) | . | . | . | 936 | 78,72 | 453 | 81,33 | 1516 | 489 |  |
| DPYD ---- tag |  | T/A or A/A | 0.85 (0.68-1.08) | 0.19 | 0.85 | 0.51 | 253 | 21,28 | 104 | 18,67 | 1516 | 489 |  |
| DPYD ---- tag | rs12126093 | T/T | 1.00 (.-.) | . | . | . | 596 | 50,13 | 286 | 51,35 | 1516 | 489 |  |
| DPYD ---- tag |  | T/C or C/C | 0.92 (0.77-1.10) | 0.37 | 0.93 | 0.62 | 593 | 49,87 | 271 | 48,65 | 1516 | 489 |  |
| DPYD ---- tag | rs12134028 | C/C | 1.00 (.-.) | . | . | . | 1070 | 89,99 | 496 | 89,05 | 1516 | 489 |  |
| DPYD ---- tag |  | C/T or T/T | 0.80 (0.59-1.07) | 0.14 | 0.83 | 0.51 | 119 | 10,01 | 61 | 10,95 | 1516 | 489 |  |
| DPYD ---- tag | rs12740796 | T/T | 1.00 (.-.) | . | . | . | 890 | 74,85 | 417 | 74,87 | 1516 | 489 |  |
| DPYD ---- tag |  | T/C or C/C | 1.05 (0.85-1.29) | 0.64 | 0.99 | 0.85 | 299 | 25,15 | 140 | 25,13 | 1516 | 489 |  |
| DPYD ---- tag | rs1333717 | A/A | 1.00 (.-.) | . | . | . | 681 | 57,28 | 341 | 61,22 | 1516 | 489 |  |
| DPYD ---- tag |  | A/G or G/G | 0.90 (0.75-1.08) | 0.26 | 0.87 | 0.53 | 508 | 42,72 | 216 | 38,78 | 1516 | 489 |  |
| DPYD ---- tag | rs1413228 | A/A | 1.00 (.-.) | . | . | . | 952 | 80,07 | 443 | 79,53 | 1516 | 489 |  |
| DPYD ---- tag |  | A/G or G/G | 1.03 (0.82-1.29) | 0.80 | 0.99 | 0.93 | 237 | 19,93 | 114 | 20,47 | 1516 | 489 |  |
| DPYD ---- tag | rs1415681 | G/G | 1.00 (.-.) | . | . | . | 884 | 74,35 | 424 | 76,12 | 1516 | 489 |  |
| DPYD ---- tag |  | G/T or T/T | 1.07 (0.87-1.32) | 0.53 | 0.97 | 0.78 | 305 | 25,65 | 133 | 23,88 | 1516 | 489 |  |
| DPYD ---- tag | rs1514495 | C/C | 1.00 (.-.) | . | . | . | 690 | 58,03 | 329 | 59,07 | 1516 | 489 |  |
| DPYD ---- tag |  | C/T or T/T | 0.96 (0.80-1.16) | 0.70 | 0.99 | 0.89 | 499 | 41,97 | 228 | 40,93 | 1516 | 489 |  |
| DPYD ---- tag | rs1520658 | A/A | 1.00 (.-.) | . | . | . | 943 | 79,31 | 437 | 78,46 | 1516 | 489 |  |
| DPYD ---- tag |  | A/G or G/G | 1.14 (0.92-1.42) | 0.24 | 0.86 | 0.53 | 246 | 20,69 | 120 | 21,54 | 1516 | 489 |  |
| DPYD ---- NA | rs17116806 | C/C | 1.00 (.-.) | . | . | . | 788 | 66,27 | 346 | 62,12 | 1516 | 489 |  |
| DPYD ---- NA |  | C/A or A/A | 1.16 (0.96-1.40) | 0.12 | 0.83 | 0.51 | 401 | 33,73 | 211 | 37,88 | 1516 | 489 |  |
| DPYD ---- tag | rs17431828 | G/G | 1.00 (.-.) | . | . | . | 490 | 41,21 | 240 | 43,09 | 1516 | 489 |  |
| DPYD ---- tag |  | G/C or C/C | 1.01 (0.84-1.21) | 0.91 | 0.99 | 0.95 | 699 | 58,79 | 317 | 56,91 | 1516 | 489 |  |
| DPYD ---- tag | rs17471640 | T/T | 1.00 (.-.) | . | . | . | 543 | 45,67 | 269 | 48,29 | 1516 | 489 |  |
| DPYD ---- tag |  | T/C or C/C | 0.89 (0.74-1.06) | 0.20 | 0.85 | 0.51 | 646 | 54,33 | 288 | 51,71 | 1516 | 489 |  |
| DPYD ---- tag | rs17702702 | G/G | 1.00 (.-.) | . | . | . | 818 | 68,80 | 393 | 70,56 | 1516 | 489 |  |
| DPYD ---- tag |  | G/C or C/C | 0.95 (0.78-1.15) | 0.58 | 0.99 | 0.81 | 371 | 31,20 | 164 | 29,44 | 1516 | 489 |  |
| DPYD ---- NA | rs1801265 | T/T | 1.00 (.-.) | . | . | . | 683 | 57,44 | 331 | 59,43 | 1516 | 489 |  |
| DPYD ---- NA |  | T/C or C/C | 1.01 (0.84-1.21) | 0.91 | 0.99 | 0.95 | 506 | 42,56 | 226 | 40,57 | 1516 | 489 |  |
| DPYD ---- tag | rs2039447 | T/T | 1.00 (.-.) | . | . | . | 553 | 46,51 | 257 | 46,14 | 1516 | 489 |  |
| DPYD ---- tag |  | T/C or C/C | 1.10 (0.92-1.32) | 0.29 | 0.88 | 0.56 | 636 | 53,49 | 300 | 53,86 | 1516 | 489 |  |
| DPYD ---- tag | rs2151567 | G/G | 1.00 (.-.) | . | . | . | 1074 | 90,33 | 499 | 89,59 | 1516 | 489 |  |
| DPYD ---- tag |  | G/A or A/A | 1.21 (0.90-1.62) | 0.20 | 0.85 | 0.51 | 115 | 9,67 | 58 | 10,41 | 1516 | 489 |  |
| DPYD ---- tag | rs2152878 | A/A | 1.00 (.-.) | . | . | . | 687 | 57,78 | 330 | 59,25 | 1516 | 489 |  |
| DPYD ---- tag |  | A/G or G/G | 1.02 (0.85-1.22) | 0.84 | 0.99 | 0.94 | 502 | 42,22 | 227 | 40,75 | 1516 | 489 |  |
| DPYD ---- tag | rs2786505 | G/G | 1.00 (.-.) | . | . | . | 885 | 74,43 | 417 | 74,87 | 1516 | 489 |  |
| DPYD ---- tag |  | G/T or T/T | 1.18 (0.96-1.44) | 0.12 | 0.82 | 0.51 | 304 | 25,57 | 140 | 25,13 | 1516 | 489 |  |
| DPYD ---- tag | rs2786512 | G/G | 1.00 (.-.) | . | . | . | 442 | 37,17 | 206 | 36,98 | 1516 | 489 |  |
| DPYD ---- tag |  | G/A or A/A | 0.98 (0.82-1.18) | 0.87 | 0.99 | 0.94 | 747 | 62,83 | 351 | 63,02 | 1516 | 489 |  |
| DPYD ---- tag | rs2786519 | A/A | 1.00 (.-.) | . | . | . | 736 | 61,90 | 317 | 56,91 | 1516 | 489 |  |
| DPYD ---- tag |  | A/G or G/G | 1.14 (0.95-1.37) | 0.15 | 0.83 | 0.51 | 453 | 38,10 | 240 | 43,09 | 1516 | 489 |  |
| DPYD ---- tag | rs2811170 | A/A | 1.00 (.-.) | . | . | . | 879 | 73,93 | 441 | 79,17 | 1516 | 489 |  |
| DPYD ---- tag |  | A/T or T/T | 0.76 (0.61-0.95) | 0.02 | 0.59 | 0.29 | 310 | 26,07 | 116 | 20,83 | 1516 | 489 |  |
| DPYD ---- tag | rs2811199 | G/G | 1.00 (.-.) | . | . | . | 847 | 71,24 | 403 | 72,35 | 1516 | 489 |  |
| DPYD ---- tag |  | G/A or A/A | 1.17 (0.96-1.42) | 0.12 | 0.83 | 0.51 | 342 | 28,76 | 154 | 27,65 | 1516 | 489 |  |
| DPYD ---- tag | rs2811219 | T/T | 1.00 (.-.) | . | . | . | 672 | 56,52 | 336 | 60,32 | 1516 | 489 |  |
| DPYD ---- tag |  | T/C or C/C | 0.91 (0.76-1.10) | 0.34 | 0.92 | 0.61 | 517 | 43,48 | 221 | 39,68 | 1516 | 489 |  |
| DPYD ---- tag | rs4300257 | A/A | 1.00 (.-.) | . | . | . | 762 | 64,09 | 351 | 63,02 | 1516 | 489 |  |
| DPYD ---- tag |  | A/C or C/C | 0.98 (0.81-1.18) | 0.82 | 0.99 | 0.93 | 427 | 35,91 | 206 | 36,98 | 1516 | 489 |  |
| DPYD ---- tag | rs4379706 | T/T | 1.00 (.-.) | . | . | . | 668 | 56,18 | 331 | 59,43 | 1516 | 489 |  |
| DPYD ---- tag |  | T/C or C/C | 1.00 (0.84-1.21) | 0.96 | 0.99 | 0.97 | 521 | 43,82 | 226 | 40,57 | 1516 | 489 |  |
| DPYD ---- tag | rs4950021 | T/T | 1.00 (.-.) | . | . | . | 363 | 30,53 | 170 | 30,52 | 1516 | 489 |  |
| DPYD ---- tag |  | T/G or G/G | 1.09 (0.89-1.32) | 0.41 | 0.93 | 0.66 | 826 | 69,47 | 387 | 69,48 | 1516 | 489 |  |
| DPYD ---- tag | rs4950033 | T/T | 1.00 (.-.) | . | . | . | 341 | 28,68 | 165 | 29,62 | 1516 | 489 |  |
| DPYD ---- tag |  | T/C or C/C | 1.04 (0.85-1.27) | 0.70 | 0.99 | 0.89 | 848 | 71,32 | 392 | 70,38 | 1516 | 489 |  |
| DPYD ---- tag | rs495257 | T/T | 1.00 (.-.) | . | . | . | 423 | 35,58 | 185 | 33,21 | 1516 | 489 |  |
| DPYD ---- tag |  | T/C or C/C | 1.14 (0.94-1.38) | 0.17 | 0.85 | 0.51 | 766 | 64,42 | 372 | 66,79 | 1516 | 489 |  |
| DPYD ---- tag | rs552926 | A/A | 1.00 (.-.) | . | . | . | 445 | 37,43 | 180 | 32,32 | 1516 | 489 |  |
| DPYD ---- tag |  | A/G or G/G | 1.26 (1.04-1.53) | 0.02 | 0.59 | 0.29 | 744 | 62,57 | 377 | 67,68 | 1516 | 489 |  |
| DPYD ---- tag | rs628959 | A/A | 1.00 (.-.) | . | . | . | 565 | 47,52 | 299 | 53,68 | 1516 | 489 |  |
| DPYD ---- tag |  | A/G or G/G | 0.92 (0.77-1.10) | 0.37 | 0.93 | 0.62 | 624 | 52,48 | 258 | 46,32 | 1516 | 489 |  |
| DPYD ---- tag | rs6656660 | G/G | 1.00 (.-.) | . | . | . | 902 | 75,86 | 421 | 75,58 | 1516 | 489 |  |
| DPYD ---- tag |  | G/T or T/T | 1.04 (0.84-1.28) | 0.72 | 0.99 | 0.89 | 287 | 24,14 | 136 | 24,42 | 1516 | 489 |  |
| DPYD ---- tag | rs6663670 | A/A | 1.00 (.-.) | . | . | . | 847 | 71,24 | 407 | 73,07 | 1516 | 489 |  |
| DPYD ---- tag |  | A/C or C/C | 1.13 (0.92-1.37) | 0.24 | 0.86 | 0.53 | 342 | 28,76 | 150 | 26,93 | 1516 | 489 |  |
| DPYD ---- tag | rs6683883 | T/T | 1.00 (.-.) | . | . | . | 474 | 39,87 | 193 | 34,65 | 1516 | 489 |  |
| DPYD ---- tag |  | T/C or C/C | 1.13 (0.94-1.36) | 0.20 | 0.85 | 0.51 | 715 | 60,13 | 364 | 65,35 | 1516 | 489 |  |
| DPYD ---- tag | rs6686861 | C/C | 1.00 (.-.) | . | . | . | 1042 | 87,64 | 464 | 83,30 | 1516 | 489 |  |
| DPYD ---- tag |  | C/T or T/T | 1.14 (0.90-1.46) | 0.27 | 0.88 | 0.55 | 147 | 12,36 | 93 | 16,70 | 1516 | 489 |  |
| DPYD ---- tag | rs7414210 | A/A | 1.00 (.-.) | . | . | . | 839 | 70,56 | 408 | 73,25 | 1516 | 489 |  |
| DPYD ---- tag |  | A/C or C/C | 0.97 (0.79-1.18) | 0.74 | 0.99 | 0.90 | 350 | 29,44 | 149 | 26,75 | 1516 | 489 |  |
| DPYD ---- tag | rs7530858 | A/A | 1.00 (.-.) | . | . | . | 933 | 78,47 | 429 | 77,02 | 1516 | 489 |  |
| DPYD ---- tag |  | A/G or G/G | 1.15 (0.93-1.42) | 0.20 | 0.85 | 0.51 | 256 | 21,53 | 128 | 22,98 | 1516 | 489 |  |
| DPYD ---- tag | rs7544128 | C/C | 1.00 (.-.) | . | . | . | 640 | 53,83 | 298 | 53,50 | 1516 | 489 |  |
| DPYD ---- tag |  | C/G or G/G | 1.01 (0.85-1.22) | 0.87 | 0.99 | 0.94 | 549 | 46,17 | 259 | 46,50 | 1516 | 489 |  |
| DPYD ---- tag | rs7545340 | G/G | 1.00 (.-.) | . | . | . | 653 | 54,92 | 295 | 52,96 | 1516 | 489 |  |
| DPYD ---- tag |  | G/A or A/A | 1.12 (0.94-1.34) | 0.22 | 0.85 | 0.52 | 536 | 45,08 | 262 | 47,04 | 1516 | 489 |  |
| DPYD ---- tag | rs828054 | A/A | 1.00 (.-.) | . | . | . | 277 | 23,30 | 149 | 26,75 | 1516 | 489 |  |
| DPYD ---- tag |  | A/C or C/C | 0.94 (0.76-1.15) | 0.54 | 0.97 | 0.78 | 912 | 76,70 | 408 | 73,25 | 1516 | 489 |  |
| DPYD ---- tag | rs885622 | G/G | 1.00 (.-.) | . | . | . | 494 | 41,55 | 201 | 36,09 | 1516 | 489 |  |
| DPYD ---- tag |  | G/A or A/A | 1.19 (0.99-1.43) | 0.07 | 0.66 | 0.51 | 695 | 58,45 | 356 | 63,91 | 1516 | 489 |  |
| DPYD ---- tag | rs9437663 | G/G | 1.00 (.-.) | . | . | . | 743 | 62,49 | 358 | 64,27 | 1516 | 489 |  |
| DPYD ---- tag |  | G/A or A/A | 1.00 (0.83-1.21) | 0.97 | 0.99 | 0.97 | 446 | 37,51 | 199 | 35,73 | 1516 | 489 |  |
| DPYS ---- tag | rs13249169 | A/A | 1.00 (.-.) | . | . | . | 947 | 79,65 | 433 | 77,74 | 1516 | 489 |  |
| DPYS ---- tag |  | A/T or T/T | 1.09 (0.88-1.36) | 0.44 | 0.93 | 0.61 | 242 | 20,35 | 124 | 22,26 | 1516 | 489 |  |
| DPYS ---- NA | rs13263121 | T/T | 1.00 (.-.) | . | . | . | 488 | 41,04 | 256 | 45,96 | 1516 | 489 |  |
| DPYS ---- NA |  | T/A or A/A | 0.82 (0.69-0.99) | 0.04 | 0.59 | 0.19 | 701 | 58,96 | 301 | 54,04 | 1516 | 489 |  |
| DPYS ---- tag | rs16871361 | T/T | 1.00 (.-.) | . | . | . | 1068 | 89,82 | 496 | 89,05 | 1516 | 489 |  |
| DPYS ---- tag |  | T/C or C/C | 1.03 (0.78-1.37) | 0.82 | 0.99 | 0.82 | 121 | 10,18 | 61 | 10,95 | 1516 | 489 |  |
| DPYS ---- NA | rs17245950 | T/T | 1.00 (.-.) | . | . | . | 918 | 77,21 | 433 | 77,74 | 1516 | 489 |  |
| DPYS ---- NA |  | T/A or A/A | 0.91 (0.73-1.14) | 0.42 | 0.93 | 0.61 | 271 | 22,79 | 124 | 22,26 | 1516 | 489 |  |
| DPYS ---- NA | rs2253336 | A/A | 1.00 (.-.) | . | . | . | 955 | 80,32 | 449 | 80,61 | 1516 | 489 |  |
| DPYS ---- NA |  | A/G or G/G | 0.94 (0.74-1.18) | 0.58 | 0.99 | 0.67 | 234 | 19,68 | 108 | 19,39 | 1516 | 489 |  |
| DPYS ---- tag | rs2280010 | C/C | 1.00 (.-.) | . | . | . | 700 | 58,87 | 288 | 51,71 | 1516 | 489 |  |
| DPYS ---- tag |  | C/T or T/T | 1.23 (1.02-1.47) | 0.03 | 0.59 | 0.19 | 489 | 41,13 | 269 | 48,29 | 1516 | 489 |  |
| DPYS ---- tag | rs2333874 | T/T | 1.00 (.-.) | . | . | . | 549 | 46,17 | 248 | 44,52 | 1516 | 489 |  |
| DPYS ---- tag |  | T/G or G/G | 1.03 (0.86-1.24) | 0.73 | 0.99 | 0.80 | 640 | 53,83 | 309 | 55,48 | 1516 | 489 |  |
| DPYS ---- NA | rs2669429 | C/C | 1.00 (.-.) | . | . | . | 379 | 31,88 | 156 | 28,01 | 1516 | 489 |  |
| DPYS ---- NA |  | C/T or T/T | 1.14 (0.93-1.40) | 0.19 | 0.85 | 0.40 | 810 | 68,12 | 401 | 71,99 | 1516 | 489 |  |
| DPYS ---- tag | rs2669434 | C/C | 1.00 (.-.) | . | . | . | 602 | 50,63 | 312 | 56,01 | 1516 | 489 |  |
| DPYS ---- tag |  | C/A or A/A | 0.83 (0.69-0.99) | 0.04 | 0.59 | 0.19 | 587 | 49,37 | 245 | 43,99 | 1516 | 489 |  |
| DPYS ---- tag | rs2853142 | T/T | 1.00 (.-.) | . | . | . | 441 | 37,09 | 226 | 40,57 | 1516 | 489 |  |
| DPYS ---- tag |  | T/C or C/C | 0.79 (0.66-0.95) | 0.01 | 0.59 | 0.19 | 748 | 62,91 | 331 | 59,43 | 1516 | 489 |  |
| DPYS ---- NA | rs2853145 | A/A | 1.00 (.-.) | . | . | . | 754 | 63,41 | 372 | 66,79 | 1516 | 489 |  |
| DPYS ---- NA |  | A/C or C/C | 0.90 (0.74-1.09) | 0.29 | 0.89 | 0.54 | 435 | 36,59 | 185 | 33,21 | 1516 | 489 |  |
| DPYS ---- tag | rs2853149 | G/G | 1.00 (.-.) | . | . | . | 338 | 28,43 | 154 | 27,65 | 1516 | 489 |  |
| DPYS ---- tag |  | G/A or A/A | 1.07 (0.87-1.31) | 0.53 | 0.97 | 0.67 | 851 | 71,57 | 403 | 72,35 | 1516 | 489 |  |
| DPYS ---- tag | rs2853154 | T/T | 1.00 (.-.) | . | . | . | 635 | 53,41 | 328 | 58,89 | 1516 | 489 |  |
| DPYS ---- tag |  | T/C or C/C | 0.83 (0.69-1.00) | 0.04 | 0.59 | 0.19 | 554 | 46,59 | 229 | 41,11 | 1516 | 489 |  |
| DPYS ---- tag | rs2853161 | A/A | 1.00 (.-.) | . | . | . | 301 | 25,32 | 139 | 24,96 | 1516 | 489 |  |
| DPYS ---- tag |  | A/G or G/G | 0.92 (0.75-1.13) | 0.43 | 0.93 | 0.61 | 888 | 74,68 | 418 | 75,04 | 1516 | 489 |  |
| DPYS ---- NA | rs2959024 | T/T | 1.00 (.-.) | . | . | . | 620 | 52,14 | 270 | 48,47 | 1516 | 489 |  |
| DPYS ---- NA |  | T/G or G/G | 1.06 (0.88-1.27) | 0.55 | 0.98 | 0.67 | 569 | 47,86 | 287 | 51,53 | 1516 | 489 |  |
| DPYS ---- NA | rs2959025 | A/A | 1.00 (.-.) | . | . | . | 507 | 42,64 | 208 | 37,34 | 1516 | 489 |  |
| DPYS ---- NA |  | A/G or G/G | 1.08 (0.90-1.29) | 0.43 | 0.93 | 0.61 | 682 | 57,36 | 349 | 62,66 | 1516 | 489 |  |
| DPYS ---- tag | rs2959026 | G/G | 1.00 (.-.) | . | . | . | 443 | 37,26 | 198 | 35,55 | 1516 | 489 |  |
| DPYS ---- tag |  | G/A or A/A | 1.13 (0.94-1.37) | 0.20 | 0.85 | 0.40 | 746 | 62,74 | 359 | 64,45 | 1516 | 489 |  |
| DPYS ---- NA | rs3133278 | T/T | 1.00 (.-.) | . | . | . | 578 | 48,61 | 286 | 51,35 | 1516 | 489 |  |
| DPYS ---- NA |  | T/C or C/C | 0.88 (0.73-1.05) | 0.15 | 0.83 | 0.37 | 611 | 51,39 | 271 | 48,65 | 1516 | 489 |  |
| DPYS ---- tag | rs3750187 | G/G | 1.00 (.-.) | . | . | . | 719 | 60,47 | 358 | 64,27 | 1516 | 489 |  |
| DPYS ---- tag |  | G/A or A/A | 0.84 (0.70-1.02) | 0.08 | 0.66 | 0.24 | 470 | 39,53 | 199 | 35,73 | 1516 | 489 |  |
| DPYS ---- tag | rs3793357 | T/T | 1.00 (.-.) | . | . | . | 1055 | 88,73 | 499 | 89,59 | 1516 | 489 |  |
| DPYS ---- tag |  | T/G or G/G | 1.05 (0.78-1.40) | 0.76 | 0.99 | 0.80 | 134 | 11,27 | 58 | 10,41 | 1516 | 489 |  |
| DPYS ---- tag | rs3793358 | G/G | 1.00 (.-.) | . | . | . | 877 | 73,76 | 434 | 77,92 | 1516 | 489 |  |
| DPYS ---- tag |  | G/A or A/A | 0.81 (0.65-1.01) | 0.06 | 0.66 | 0.22 | 312 | 26,24 | 123 | 22,08 | 1516 | 489 |  |
| DPYS ---- tag | rs6468924 | C/C | 1.00 (.-.) | . | . | . | 713 | 59,97 | 358 | 64,27 | 1516 | 489 |  |
| DPYS ---- tag |  | C/T or T/T | 0.87 (0.72-1.05) | 0.15 | 0.83 | 0.37 | 476 | 40,03 | 199 | 35,73 | 1516 | 489 |  |
| DUT ---- tag | rs8025164 | G/G | 1.00 (.-.) | . | . | . | 855 | 71,91 | 396 | 71,10 | 1516 | 489 |  |
| DUT ---- tag |  | G/A or A/A | 1.01 (0.83-1.24) | 0.89 | 0.99 | 0.89 | 334 | 28,09 | 161 | 28,90 | 1516 | 489 |  |
| EHMT1 ---- tag | rs10780190 | C/C | 1.00 (.-.) | . | . | . | 1057 | 88,90 | 499 | 89,59 | 1516 | 489 |  |
| EHMT1 ---- tag |  | C/T or T/T | 1.08 (0.80-1.45) | 0.63 | 0.99 | 0.97 | 132 | 11,10 | 58 | 10,41 | 1516 | 489 |  |
| EHMT1 ---- tag | rs10867083 | G/G | 1.00 (.-.) | . | . | . | 536 | 45,08 | 252 | 45,24 | 1516 | 489 |  |
| EHMT1 ---- tag |  | G/A or A/A | 0.99 (0.82-1.18) | 0.88 | 0.99 | 0.97 | 653 | 54,92 | 305 | 54,76 | 1516 | 489 |  |
| EHMT1 ---- tag | rs11137190 | C/C | 1.00 (.-.) | . | . | . | 609 | 51,22 | 279 | 50,09 | 1516 | 489 |  |
| EHMT1 ---- tag |  | C/G or G/G | 1.12 (0.93-1.34) | 0.22 | 0.85 | 0.81 | 580 | 48,78 | 278 | 49,91 | 1516 | 489 |  |
| EHMT1 ---- tag | rs3123510 | G/G | 1.00 (.-.) | . | . | . | 415 | 34,90 | 193 | 34,65 | 1516 | 489 |  |
| EHMT1 ---- tag |  | G/A or A/A | 1.10 (0.91-1.33) | 0.33 | 0.91 | 0.90 | 774 | 65,10 | 364 | 65,35 | 1516 | 489 |  |
| EHMT1 ---- candidate literature | rs3125795 | G/G | 1.00 (.-.) | . | . | . | 1054 | 88,65 | 497 | 89,23 | 1516 | 489 |  |
| EHMT1 ---- candidate literature |  | G/T or T/T | 1.11 (0.83-1.49) | 0.47 | 0.94 | 0.97 | 135 | 11,35 | 60 | 10,77 | 1516 | 489 |  |
| EHMT1 ---- tag | rs4573359 | G/G | 1.00 (.-.) | . | . | . | 986 | 82,93 | 461 | 82,76 | 1516 | 489 |  |
| EHMT1 ---- tag |  | G/T or T/T | 1.01 (0.80-1.28) | 0.94 | 0.99 | 0.97 | 203 | 17,07 | 96 | 17,24 | 1516 | 489 |  |
| EHMT1 ---- candidate literature | rs4634736 | G/G | 1.00 (.-.) | . | . | . | 985 | 82,84 | 462 | 82,94 | 1516 | 489 |  |
| EHMT1 ---- candidate literature |  | G/A or A/A | 1.00 (0.78-1.26) | 0.97 | 0.99 | 0.97 | 204 | 17,16 | 95 | 17,06 | 1516 | 489 |  |
| EHMT1 ---- tag | rs4876902 | C/C | 1.00 (.-.) | . | . | . | 739 | 62,15 | 331 | 59,43 | 1516 | 489 |  |
| EHMT1 ---- tag |  | C/T or T/T | 1.16 (0.97-1.40) | 0.11 | 0.81 | 0.58 | 450 | 37,85 | 226 | 40,57 | 1516 | 489 |  |
| EHMT1 ---- tag | rs4876904 | T/T | 1.00 (.-.) | . | . | . | 351 | 29,52 | 173 | 31,06 | 1516 | 489 |  |
| EHMT1 ---- tag |  | T/G or G/G | 0.98 (0.81-1.19) | 0.83 | 0.99 | 0.97 | 838 | 70,48 | 384 | 68,94 | 1516 | 489 |  |
| EHMT1 ---- tag | rs7390244 | G/G | 1.00 (.-.) | . | . | . | 283 | 23,80 | 152 | 27,29 | 1516 | 489 |  |
| EHMT1 ---- tag |  | G/A or A/A | 1.00 (0.82-1.23) | 0.97 | 0.99 | 0.97 | 906 | 76,20 | 405 | 72,71 | 1516 | 489 |  |
| EHMT1 ---- tag | rs9314635 | G/G | 1.00 (.-.) | . | . | . | 483 | 40,62 | 207 | 37,16 | 1516 | 489 |  |
| EHMT1 ---- tag |  | G/T or T/T | 1.18 (0.98-1.42) | 0.08 | 0.68 | 0.58 | 706 | 59,38 | 350 | 62,84 | 1516 | 489 |  |
| EHMT2 ---- candidate/tag | rs2736428 | G/G | 1.00 (.-.) | . | . | . | 478 | 40,20 | 238 | 42,73 | 1516 | 489 |  |
| EHMT2 ---- candidate/tag |  | G/A or A/A | 0.91 (0.76-1.09) | 0.31 | 0.89 | 0.31 | 711 | 59,80 | 319 | 57,27 | 1516 | 489 |  |
| EHMT2 ---- tag | rs9267649 | G/G | 1.00 (.-.) | . | . | . | 842 | 70,82 | 402 | 72,17 | 1516 | 489 |  |
| EHMT2 ---- tag |  | G/A or A/A | 0.88 (0.72-1.07) | 0.20 | 0.85 | 0.31 | 347 | 29,18 | 155 | 27,83 | 1516 | 489 |  |
| FDXR ---- NA | rs2070918 | T/T | 1.00 (.-.) | . | . | . | 548 | 46,09 | 260 | 46,68 | 1516 | 489 |  |
| FDXR ---- NA |  | T/C or C/C | 1.12 (0.94-1.34) | 0.22 | 0.85 | 0.68 | 641 | 53,91 | 297 | 53,32 | 1516 | 489 |  |
| FDXR ---- tag | rs509911 | A/A | 1.00 (.-.) | . | . | . | 725 | 60,98 | 350 | 62,84 | 1516 | 489 |  |
| FDXR ---- tag |  | A/G or G/G | 1.07 (0.89-1.29) | 0.48 | 0.95 | 0.68 | 464 | 39,02 | 207 | 37,16 | 1516 | 489 |  |
| FDXR ---- NA | rs689882 | G/G | 1.00 (.-.) | . | . | . | 602 | 50,63 | 301 | 54,04 | 1516 | 489 |  |
| FDXR ---- NA |  | G/A or A/A | 0.96 (0.80-1.15) | 0.65 | 0.99 | 0.68 | 587 | 49,37 | 256 | 45,96 | 1516 | 489 |  |
| FDXR ---- NA | rs689895 | G/G | 1.00 (.-.) | . | . | . | 583 | 49,03 | 283 | 50,81 | 1516 | 489 |  |
| FDXR ---- NA |  | G/C or C/C | 0.96 (0.80-1.15) | 0.68 | 0.99 | 0.68 | 606 | 50,97 | 274 | 49,19 | 1516 | 489 |  |
| FOLH1 ---- candidate literature | rs10839236 | T/T | 1.00 (.-.) | . | . | . | 471 | 39,61 | 213 | 38,24 | 1516 | 489 |  |
| FOLH1 ---- candidate literature |  | T/C or C/C | 1.08 (0.89-1.30) | 0.44 | 0.93 | 0.79 | 718 | 60,39 | 344 | 61,76 | 1516 | 489 |  |
| FOLH1 ---- tag | rs16906190 | A/A | 1.00 (.-.) | . | . | . | 985 | 82,84 | 471 | 84,56 | 1516 | 489 |  |
| FOLH1 ---- tag |  | A/G or G/G | 1.05 (0.81-1.35) | 0.71 | 0.99 | 0.79 | 204 | 17,16 | 86 | 15,44 | 1516 | 489 |  |
| FOLH1 ---- candidate | rs202676 | T/T | 1.00 (.-.) | . | . | . | 737 | 61,98 | 349 | 62,66 | 1516 | 489 |  |
| FOLH1 ---- candidate |  | T/C or C/C | 0.96 (0.80-1.16) | 0.67 | 0.99 | 0.79 | 452 | 38,02 | 208 | 37,34 | 1516 | 489 |  |
| FOLH1 ---- tag | rs202680 | A/A | 1.00 (.-.) | . | . | . | 650 | 54,67 | 314 | 56,37 | 1516 | 489 |  |
| FOLH1 ---- tag |  | A/T or T/T | 0.96 (0.80-1.15) | 0.66 | 0.99 | 0.79 | 539 | 45,33 | 243 | 43,63 | 1516 | 489 |  |
| FOLH1 ---- candidate literature | rs202720 | G/G | 1.00 (.-.) | . | . | . | 735 | 61,82 | 349 | 62,66 | 1516 | 489 |  |
| FOLH1 ---- candidate literature |  | G/C or C/C | 0.95 (0.79-1.15) | 0.61 | 0.99 | 0.79 | 454 | 38,18 | 208 | 37,34 | 1516 | 489 |  |
| FOLH1 ---- tag | rs2299650 | G/G | 1.00 (.-.) | . | . | . | 464 | 39,02 | 213 | 38,24 | 1516 | 489 |  |
| FOLH1 ---- tag |  | G/T or T/T | 1.06 (0.88-1.28) | 0.51 | 0.96 | 0.79 | 725 | 60,98 | 344 | 61,76 | 1516 | 489 |  |
| FOLH1 ---- tag | rs617528 | G/G | 1.00 (.-.) | . | . | . | 933 | 78,47 | 430 | 77,20 | 1516 | 489 |  |
| FOLH1 ---- tag |  | G/A or A/A | 1.10 (0.89-1.35) | 0.37 | 0.93 | 0.79 | 256 | 21,53 | 127 | 22,80 | 1516 | 489 |  |
| FOLH1 ---- tag | rs663877 | T/T | 1.00 (.-.) | . | . | . | 914 | 76,87 | 426 | 76,48 | 1516 | 489 |  |
| FOLH1 ---- tag |  | T/G or G/G | 0.91 (0.74-1.12) | 0.37 | 0.93 | 0.79 | 275 | 23,13 | 131 | 23,52 | 1516 | 489 |  |
| FOLH1 ---- tag | rs670776 | A/A | 1.00 (.-.) | . | . | . | 737 | 61,98 | 349 | 62,66 | 1516 | 489 |  |
| FOLH1 ---- tag |  | A/T or T/T | 0.96 (0.80-1.16) | 0.67 | 0.99 | 0.79 | 452 | 38,02 | 208 | 37,34 | 1516 | 489 |  |
| FOLH1 ---- tag | rs7124497 | G/G | 1.00 (.-.) | . | . | . | 1090 | 91,67 | 512 | 91,92 | 1516 | 489 |  |
| FOLH1 ---- tag |  | G/A or A/A | 0.96 (0.68-1.34) | 0.80 | 0.99 | 0.80 | 99 | 8,33 | 45 | 8,08 | 1516 | 489 |  |
| FOLR1 ---- tag | rs651646 | T/T | 1.00 (.-.) | . | . | . | 362 | 30,45 | 180 | 32,32 | 1516 | 489 |  |
| FOLR1 ---- tag |  | T/A or A/A | 0.96 (0.79-1.16) | 0.68 | 0.99 | 0.68 | 827 | 69,55 | 377 | 67,68 | 1516 | 489 |  |
| FPGS ---- tag | rs10987746 | T/T | 1.00 (.-.) | . | . | . | 344 | 28,93 | 153 | 27,47 | 1516 | 489 |  |
| FPGS ---- tag |  | T/C or C/C | 0.93 (0.77-1.14) | 0.51 | 0.96 | 0.63 | 845 | 71,07 | 404 | 72,53 | 1516 | 489 |  |
| FPGS ---- tag | rs7033913 | T/T | 1.00 (.-.) | . | . | . | 371 | 31,20 | 181 | 32,50 | 1516 | 489 |  |
| FPGS ---- tag |  | T/C or C/C | 0.95 (0.79-1.16) | 0.63 | 0.99 | 0.63 | 818 | 68,80 | 376 | 67,50 | 1516 | 489 |  |
| FPGS ---- tag | rs7039798 | G/G | 1.00 (.-.) | . | . | . | 390 | 32,80 | 167 | 29,98 | 1516 | 489 |  |
| FPGS ---- tag |  | G/A or A/A | 0.94 (0.77-1.14) | 0.54 | 0.97 | 0.63 | 799 | 67,20 | 390 | 70,02 | 1516 | 489 |  |
| GGH ---- tag | rs10957264 | G/G | 1.00 (.-.) | . | . | . | 849 | 71,40 | 384 | 68,94 | 1516 | 489 |  |
| GGH ---- tag |  | G/T or T/T | 1.10 (0.91-1.34) | 0.33 | 0.92 | 0.42 | 340 | 28,60 | 173 | 31,06 | 1516 | 489 |  |
| GGH ---- candidate literature | rs11545076 | T/T | 1.00 (.-.) | . | . | . | 597 | 50,21 | 270 | 48,47 | 1516 | 489 |  |
| GGH ---- candidate literature |  | T/G or G/G | 0.92 (0.77-1.10) | 0.36 | 0.93 | 0.42 | 592 | 49,79 | 287 | 51,53 | 1516 | 489 |  |
| GGH ---- candidate | rs11545077 | G/G | 1.00 (.-.) | . | . | . | 646 | 54,33 | 295 | 52,96 | 1516 | 489 |  |
| GGH ---- candidate |  | G/A or A/A | 0.99 (0.83-1.19) | 0.93 | 0.99 | 0.93 | 543 | 45,67 | 262 | 47,04 | 1516 | 489 |  |
| GGH ---- candidate | rs11545078 | C/C | 1.00 (.-.) | . | . | . | 991 | 83,35 | 447 | 80,25 | 1516 | 489 |  |
| GGH ---- candidate |  | C/T or T/T | 1.13 (0.90-1.42) | 0.31 | 0.89 | 0.42 | 198 | 16,65 | 110 | 19,75 | 1516 | 489 |  |
| GGH ---- tag | rs11995525 | G/G | 1.00 (.-.) | . | . | . | 650 | 54,67 | 282 | 50,63 | 1516 | 489 |  |
| GGH ---- tag |  | G/A or A/A | 1.20 (1.00-1.44) | 0.05 | 0.59 | 0.29 | 539 | 45,33 | 275 | 49,37 | 1516 | 489 |  |
| GGH ---- tag | rs16930073 | G/G | 1.00 (.-.) | . | . | . | 940 | 79,06 | 456 | 81,87 | 1516 | 489 |  |
| GGH ---- tag |  | G/A or A/A | 0.82 (0.65-1.04) | 0.10 | 0.80 | 0.33 | 249 | 20,94 | 101 | 18,13 | 1516 | 489 |  |
| GGH ---- tag | rs17194931 | G/G | 1.00 (.-.) | . | . | . | 991 | 83,35 | 447 | 80,25 | 1516 | 489 |  |
| GGH ---- tag |  | G/A or A/A | 1.13 (0.90-1.42) | 0.31 | 0.89 | 0.42 | 198 | 16,65 | 110 | 19,75 | 1516 | 489 |  |
| GGH ---- candidate literature | rs1800909 | T/T | 1.00 (.-.) | . | . | . | 593 | 49,87 | 269 | 48,29 | 1516 | 489 |  |
| GGH ---- candidate literature |  | T/C or C/C | 0.90 (0.76-1.08) | 0.27 | 0.88 | 0.42 | 596 | 50,13 | 288 | 51,71 | 1516 | 489 |  |
| GGH ---- candidate literature | rs3758149 | C/C | 1.00 (.-.) | . | . | . | 597 | 50,21 | 270 | 48,47 | 1516 | 489 |  |
| GGH ---- candidate literature |  | C/T or T/T | 0.92 (0.77-1.10) | 0.36 | 0.93 | 0.42 | 592 | 49,79 | 287 | 51,53 | 1516 | 489 |  |
| GGH ---- tag | rs3780130 | A/A | 1.00 (.-.) | . | . | . | 730 | 61,40 | 369 | 66,25 | 1516 | 489 |  |
| GGH ---- tag |  | A/T or T/T | 0.77 (0.63-0.93) | 0.01 | 0.59 | 0.09 | 459 | 38,60 | 188 | 33,75 | 1516 | 489 |  |
| GGH ---- tag | rs4446729 | C/C | 1.00 (.-.) | . | . | . | 625 | 52,57 | 295 | 52,96 | 1516 | 489 |  |
| GGH ---- tag |  | C/T or T/T | 1.02 (0.85-1.22) | 0.85 | 0.99 | 0.92 | 564 | 47,43 | 262 | 47,04 | 1516 | 489 |  |
| GGH ---- tag | rs6472067 | C/C | 1.00 (.-.) | . | . | . | 482 | 40,54 | 213 | 38,24 | 1516 | 489 |  |
| GGH ---- tag |  | C/G or G/G | 1.17 (0.97-1.41) | 0.10 | 0.79 | 0.33 | 707 | 59,46 | 344 | 61,76 | 1516 | 489 |  |
| GGH ---- tag | rs7010484 | T/T | 1.00 (.-.) | . | . | . | 539 | 45,33 | 248 | 44,52 | 1516 | 489 |  |
| GGH ---- tag |  | T/C or C/C | 1.15 (0.96-1.37) | 0.14 | 0.83 | 0.36 | 650 | 54,67 | 309 | 55,48 | 1516 | 489 |  |
| GNMT ---- tag | rs1053538 | C/C | 1.00 (.-.) | . | . | . | 326 | 27,42 | 156 | 28,01 | 1516 | 489 |  |
| GNMT ---- tag |  | C/G or G/G | 1.03 (0.85-1.26) | 0.75 | 0.99 | 0.75 | 863 | 72,58 | 401 | 71,99 | 1516 | 489 |  |
| GNMT ---- tag | rs2296805 | G/G | 1.00 (.-.) | . | . | . | 356 | 29,94 | 190 | 34,11 | 1516 | 489 |  |
| GNMT ---- tag |  | G/T or T/T | 0.84 (0.69-1.01) | 0.07 | 0.66 | 0.28 | 833 | 70,06 | 367 | 65,89 | 1516 | 489 |  |
| GNMT ---- tag | rs6901782 | T/T | 1.00 (.-.) | . | . | . | 902 | 75,86 | 432 | 77,56 | 1516 | 489 |  |
| GNMT ---- tag |  | T/C or C/C | 1.08 (0.87-1.34) | 0.47 | 0.94 | 0.70 | 287 | 24,14 | 125 | 22,44 | 1516 | 489 |  |
| GNMT ---- tag | rs6927188 | A/A | 1.00 (.-.) | . | . | . | 695 | 58,45 | 310 | 55,66 | 1516 | 489 |  |
| GNMT ---- tag |  | A/G or G/G | 1.06 (0.88-1.27) | 0.53 | 0.97 | 0.70 | 494 | 41,55 | 247 | 44,34 | 1516 | 489 |  |
| MAT1A ---- tag | rs10887708 | G/G | 1.00 (.-.) | . | . | . | 582 | 48,95 | 295 | 52,96 | 1516 | 489 |  |
| MAT1A ---- tag |  | G/A or A/A | 0.93 (0.78-1.12) | 0.45 | 0.93 | 0.82 | 607 | 51,05 | 262 | 47,04 | 1516 | 489 |  |
| MAT1A ---- tag | rs10887718 | T/T | 1.00 (.-.) | . | . | . | 340 | 28,60 | 151 | 27,11 | 1516 | 489 |  |
| MAT1A ---- tag |  | T/C or C/C | 1.23 (1.00-1.51) | 0.05 | 0.59 | 0.20 | 849 | 71,40 | 406 | 72,89 | 1516 | 489 |  |
| MAT1A ---- tag | rs11202403 | C/C | 1.00 (.-.) | . | . | . | 774 | 65,10 | 360 | 64,63 | 1516 | 489 |  |
| MAT1A ---- tag |  | C/T or T/T | 1.01 (0.84-1.22) | 0.89 | 0.99 | 0.98 | 415 | 34,90 | 197 | 35,37 | 1516 | 489 |  |
| MAT1A ---- tag | rs1832683 | C/C | 1.00 (.-.) | . | . | . | 826 | 69,47 | 382 | 68,58 | 1516 | 489 |  |
| MAT1A ---- tag |  | C/T or T/T | 0.93 (0.76-1.13) | 0.45 | 0.93 | 0.82 | 363 | 30,53 | 175 | 31,42 | 1516 | 489 |  |
| MAT1A ---- tag | rs2236568 | C/C | 1.00 (.-.) | . | . | . | 374 | 31,46 | 187 | 33,57 | 1516 | 489 |  |
| MAT1A ---- tag |  | C/A or A/A | 0.90 (0.74-1.09) | 0.27 | 0.88 | 0.80 | 815 | 68,54 | 370 | 66,43 | 1516 | 489 |  |
| MAT1A ---- tag | rs2236569 | A/A | 1.00 (.-.) | . | . | . | 540 | 45,42 | 227 | 40,75 | 1516 | 489 |  |
| MAT1A ---- tag |  | A/G or G/G | 1.30 (1.08-1.56) | 0.01 | 0.59 | 0.05 | 649 | 54,58 | 330 | 59,25 | 1516 | 489 |  |
| MAT1A ---- tag | rs9421467 | G/G | 1.00 (.-.) | . | . | . | 1060 | 89,15 | 504 | 90,48 | 1516 | 489 |  |
| MAT1A ---- tag |  | G/C or C/C | 0.97 (0.73-1.30) | 0.86 | 0.99 | 0.98 | 129 | 10,85 | 53 | 9,52 | 1516 | 489 |  |
| MAT1A ---- tag | rs998765 | A/A | 1.00 (.-.) | . | . | . | 301 | 25,32 | 149 | 26,75 | 1516 | 489 |  |
| MAT1A ---- tag |  | A/T or T/T | 1.00 (0.81-1.22) | 0.97 | 0.99 | 0.98 | 888 | 74,68 | 408 | 73,25 | 1516 | 489 |  |
| MAT1A ---- tag | rs998766 | C/C | 1.00 (.-.) | . | . | . | 370 | 31,12 | 179 | 32,14 | 1516 | 489 |  |
| MAT1A ---- tag |  | C/G or G/G | 1.00 (0.82-1.21) | 0.98 | 1.00 | 0.98 | 819 | 68,88 | 378 | 67,86 | 1516 | 489 |  |
| MAT2B ---- tag | rs12655857 | G/G | 1.00 (.-.) | . | . | . | 672 | 56,52 | 305 | 54,76 | 1516 | 489 |  |
| MAT2B ---- tag |  | G/T or T/T | 1.05 (0.87-1.26) | 0.63 | 0.99 | 0.93 | 517 | 43,48 | 252 | 45,24 | 1516 | 489 |  |
| MAT2B ---- tag | rs6869277 | C/C | 1.00 (.-.) | . | . | . | 933 | 78,47 | 436 | 78,28 | 1516 | 489 |  |
| MAT2B ---- tag |  | C/T or T/T | 0.99 (0.79-1.23) | 0.93 | 0.99 | 0.93 | 256 | 21,53 | 121 | 21,72 | 1516 | 489 |  |
| MAT2B ---- tag | rs6874065 | A/A | 1.00 (.-.) | . | . | . | 328 | 27,59 | 153 | 27,47 | 1516 | 489 |  |
| MAT2B ---- tag |  | A/G or G/G | 1.03 (0.84-1.26) | 0.76 | 0.99 | 0.93 | 861 | 72,41 | 404 | 72,53 | 1516 | 489 |  |
| MAT2B ---- tag | rs6882306 | T/T | 1.00 (.-.) | . | . | . | 793 | 66,69 | 369 | 66,25 | 1516 | 489 |  |
| MAT2B ---- tag |  | T/C or C/C | 1.25 (1.04-1.52) | 0.02 | 0.59 | 0.10 | 396 | 33,31 | 188 | 33,75 | 1516 | 489 |  |
| MAT2B ---- tag | rs7721639 | T/T | 1.00 (.-.) | . | . | . | 832 | 69,97 | 397 | 71,27 | 1516 | 489 |  |
| MAT2B ---- tag |  | T/G or G/G | 1.04 (0.85-1.27) | 0.70 | 0.99 | 0.93 | 357 | 30,03 | 160 | 28,73 | 1516 | 489 |  |
| MTHFD1 ---- tag | rs1256148 | G/G | 1.00 (.-.) | . | . | . | 708 | 59,55 | 324 | 58,17 | 1516 | 489 |  |
| MTHFD1 ---- tag |  | G/A or A/A | 1.07 (0.89-1.29) | 0.45 | 0.93 | 0.88 | 481 | 40,45 | 233 | 41,83 | 1516 | 489 |  |
| MTHFD1 ---- tag | rs13329053 | T/T | 1.00 (.-.) | . | . | . | 366 | 30,78 | 182 | 32,68 | 1516 | 489 |  |
| MTHFD1 ---- tag |  | T/C or C/C | 1.01 (0.83-1.22) | 0.95 | 0.99 | 0.95 | 823 | 69,22 | 375 | 67,32 | 1516 | 489 |  |
| MTHFD1 ---- candidate literature | rs2236224 | C/C | 1.00 (.-.) | . | . | . | 458 | 38,52 | 221 | 39,68 | 1516 | 489 |  |
| MTHFD1 ---- candidate literature |  | C/T or T/T | 1.06 (0.88-1.28) | 0.51 | 0.96 | 0.88 | 731 | 61,48 | 336 | 60,32 | 1516 | 489 |  |
| MTHFD1 ---- candidate | rs2236225 | C/C | 1.00 (.-.) | . | . | . | 370 | 31,12 | 188 | 33,75 | 1516 | 489 |  |
| MTHFD1 ---- candidate |  | C/T or T/T | 0.99 (0.82-1.20) | 0.94 | 0.99 | 0.95 | 819 | 68,88 | 369 | 66,25 | 1516 | 489 |  |
| MTHFD1 ---- tag | rs2281603 | A/A | 1.00 (.-.) | . | . | . | 699 | 58,79 | 322 | 57,81 | 1516 | 489 |  |
| MTHFD1 ---- tag |  | A/G or G/G | 0.95 (0.79-1.14) | 0.59 | 0.99 | 0.88 | 490 | 41,21 | 235 | 42,19 | 1516 | 489 |  |
| MTHFD1 ---- candidate literature | rs8003379 | A/A | 1.00 (.-.) | . | . | . | 667 | 56,10 | 316 | 56,73 | 1516 | 489 |  |
| MTHFD1 ---- candidate literature |  | A/C or C/C | 1.17 (0.98-1.41) | 0.08 | 0.69 | 0.49 | 522 | 43,90 | 241 | 43,27 | 1516 | 489 |  |
| MTHFD2 ---- tag | rs10177833 | A/A | 1.00 (.-.) | . | . | . | 379 | 31,88 | 150 | 26,93 | 1516 | 489 |  |
| MTHFD2 ---- tag |  | A/C or C/C | 1.20 (0.98-1.47) | 0.07 | 0.66 | 0.26 | 810 | 68,12 | 407 | 73,07 | 1516 | 489 |  |
| MTHFD2 ---- tag | rs702462 | T/T | 1.00 (.-.) | . | . | . | 393 | 33,05 | 179 | 32,14 | 1516 | 489 |  |
| MTHFD2 ---- tag |  | T/A or A/A | 0.98 (0.81-1.19) | 0.83 | 0.99 | 0.83 | 796 | 66,95 | 378 | 67,86 | 1516 | 489 |  |
| MTHFD2 ---- candidate literature | rs702465 | A/A | 1.00 (.-.) | . | . | . | 338 | 28,43 | 151 | 27,11 | 1516 | 489 |  |
| MTHFD2 ---- candidate literature |  | A/T or T/T | 1.07 (0.88-1.31) | 0.49 | 0.95 | 0.69 | 851 | 71,57 | 406 | 72,89 | 1516 | 489 |  |
| MTHFD2 ---- candidate literature | rs7571842 | A/A | 1.00 (.-.) | . | . | . | 354 | 29,77 | 132 | 23,70 | 1516 | 489 |  |
| MTHFD2 ---- candidate literature |  | A/G or G/G | 1.18 (0.96-1.46) | 0.11 | 0.82 | 0.26 | 835 | 70,23 | 425 | 76,30 | 1516 | 489 |  |
| MTHFD2 ---- tag | rs7587117 | T/T | 1.00 (.-.) | . | . | . | 506 | 42,56 | 211 | 37,88 | 1516 | 489 |  |
| MTHFD2 ---- tag |  | T/C or C/C | 1.13 (0.94-1.36) | 0.20 | 0.85 | 0.35 | 683 | 57,44 | 346 | 62,12 | 1516 | 489 |  |
| MTHFD2 ---- tag | rs828861 | C/C | 1.00 (.-.) | . | . | . | 340 | 28,60 | 155 | 27,83 | 1516 | 489 |  |
| MTHFD2 ---- tag |  | C/G or G/G | 1.04 (0.86-1.27) | 0.67 | 0.99 | 0.78 | 849 | 71,40 | 402 | 72,17 | 1516 | 489 |  |
| MTHFD2 ---- tag | rs828863 | G/G | 1.00 (.-.) | . | . | . | 947 | 79,65 | 475 | 85,28 | 1516 | 489 |  |
| MTHFD2 ---- tag |  | G/A or A/A | 0.72 (0.56-0.94) | 0.01 | 0.59 | 0.10 | 242 | 20,35 | 82 | 14,72 | 1516 | 489 |  |
| MTHFR ---- tag | rs1476413 | G/G | 1.00 (.-.) | . | . | . | 634 | 53,32 | 292 | 52,42 | 1516 | 489 |  |
| MTHFR ---- tag |  | G/A or A/A | 0.98 (0.82-1.18) | 0.86 | 0.99 | 0.90 | 555 | 46,68 | 265 | 47,58 | 1516 | 489 |  |
| MTHFR ---- tag | rs17376328 | G/G | 1.00 (.-.) | . | . | . | 1062 | 89,32 | 474 | 85,10 | 1516 | 489 |  |
| MTHFR ---- tag |  | G/A or A/A | 1.19 (0.92-1.54) | 0.18 | 0.85 | 0.69 | 127 | 10,68 | 83 | 14,90 | 1516 | 489 |  |
| MTHFR ---- tag | rs17421462 | G/G | 1.00 (.-.) | . | . | . | 1006 | 84,61 | 478 | 85,82 | 1516 | 489 |  |
| MTHFR ---- tag |  | G/A or A/A | 0.92 (0.71-1.19) | 0.51 | 0.96 | 0.84 | 183 | 15,39 | 79 | 14,18 | 1516 | 489 |  |
| MTHFR ---- candidate | rs1801131 | A/A | 1.00 (.-.) | . | . | . | 550 | 46,26 | 251 | 45,06 | 1516 | 489 |  |
| MTHFR ---- candidate |  | A/C or C/C | 0.99 (0.83-1.18) | 0.90 | 0.99 | 0.90 | 639 | 53,74 | 306 | 54,94 | 1516 | 489 |  |
| MTHFR ---- candidate | rs1801133 | C/C | 1.00 (.-.) | . | . | . | 479 | 40,29 | 244 | 43,81 | 1516 | 489 |  |
| MTHFR ---- candidate |  | C/T or T/T | 0.81 (0.67-0.97) | 0.02 | 0.59 | 0.18 | 710 | 59,71 | 313 | 56,19 | 1516 | 489 |  |
| MTHFR ---- tag | rs2066471 | G/G | 1.00 (.-.) | . | . | . | 826 | 69,47 | 396 | 71,10 | 1516 | 489 |  |
| MTHFR ---- tag |  | G/A or A/A | 0.94 (0.77-1.15) | 0.56 | 0.99 | 0.84 | 363 | 30,53 | 161 | 28,90 | 1516 | 489 |  |
| MTHFR ---- tag | rs4846047 | G/G | 1.00 (.-.) | . | . | . | 588 | 49,45 | 292 | 52,42 | 1516 | 489 |  |
| MTHFR ---- tag |  | G/C or C/C | 0.90 (0.75-1.07) | 0.23 | 0.85 | 0.69 | 601 | 50,55 | 265 | 47,58 | 1516 | 489 |  |
| MTHFR ---- tag | rs4846049 | G/G | 1.00 (.-.) | . | . | . | 542 | 45,58 | 246 | 44,17 | 1516 | 489 |  |
| MTHFR ---- tag |  | G/T or T/T | 0.98 (0.82-1.17) | 0.83 | 0.99 | 0.90 | 647 | 54,42 | 311 | 55,83 | 1516 | 489 |  |
| MTHFR ---- tag | rs7538516 | T/T | 1.00 (.-.) | . | . | . | 438 | 36,84 | 203 | 36,45 | 1516 | 489 |  |
| MTHFR ---- tag |  | T/C or C/C | 0.93 (0.77-1.12) | 0.44 | 0.93 | 0.84 | 751 | 63,16 | 354 | 63,55 | 1516 | 489 |  |
| MTR ---- tag | rs10733117 | A/A | 1.00 (.-.) | . | . | . | 426 | 35,83 | 195 | 35,01 | 1516 | 489 |  |
| MTR ---- tag |  | A/G or G/G | 0.95 (0.79-1.15) | 0.60 | 0.99 | 0.83 | 763 | 64,17 | 362 | 64,99 | 1516 | 489 |  |
| MTR ---- tag | rs12129440 | G/G | 1.00 (.-.) | . | . | . | 640 | 53,83 | 318 | 57,09 | 1516 | 489 |  |
| MTR ---- tag |  | G/A or A/A | 0.94 (0.78-1.12) | 0.49 | 0.95 | 0.83 | 549 | 46,17 | 239 | 42,91 | 1516 | 489 |  |
| MTR ---- candidate | rs1805087 | A/A | 1.00 (.-.) | . | . | . | 789 | 66,36 | 384 | 68,94 | 1516 | 489 |  |
| MTR ---- candidate |  | A/G or G/G | 0.98 (0.81-1.19) | 0.83 | 0.99 | 0.83 | 400 | 33,64 | 173 | 31,06 | 1516 | 489 |  |
| MTR ---- tag | rs3890786 | C/C | 1.00 (.-.) | . | . | . | 405 | 34,06 | 185 | 33,21 | 1516 | 489 |  |
| MTR ---- tag |  | C/T or T/T | 1.05 (0.86-1.27) | 0.65 | 0.99 | 0.83 | 784 | 65,94 | 372 | 66,79 | 1516 | 489 |  |
| MTR ---- tag | rs4659727 | A/A | 1.00 (.-.) | . | . | . | 784 | 65,94 | 384 | 68,94 | 1516 | 489 |  |
| MTR ---- tag |  | A/G or G/G | 0.97 (0.80-1.18) | 0.76 | 0.99 | 0.83 | 405 | 34,06 | 173 | 31,06 | 1516 | 489 |  |
| MTRR ---- candidate literature/tag | rs10380 | C/C | 1.00 (.-.) | . | . | . | 1000 | 84,10 | 465 | 83,48 | 1516 | 489 |  |
| MTRR ---- candidate literature/tag |  | C/T or T/T | 1.03 (0.81-1.31) | 0.81 | 0.99 | 0.90 | 189 | 15,90 | 92 | 16,52 | 1516 | 489 |  |
| MTRR ---- tag | rs10475399 | G/G | 1.00 (.-.) | . | . | . | 515 | 43,31 | 241 | 43,27 | 1516 | 489 |  |
| MTRR ---- tag |  | G/A or A/A | 1.03 (0.86-1.24) | 0.73 | 0.99 | 0.90 | 674 | 56,69 | 316 | 56,73 | 1516 | 489 |  |
| MTRR ---- tag | rs11134265 | C/C | 1.00 (.-.) | . | . | . | 519 | 43,65 | 250 | 44,88 | 1516 | 489 |  |
| MTRR ---- tag |  | C/T or T/T | 1.05 (0.87-1.25) | 0.63 | 0.99 | 0.90 | 670 | 56,35 | 307 | 55,12 | 1516 | 489 |  |
| MTRR ---- tag | rs13181011 | T/T | 1.00 (.-.) | . | . | . | 786 | 66,11 | 338 | 60,68 | 1516 | 489 |  |
| MTRR ---- tag |  | T/C or C/C | 1.04 (0.86-1.25) | 0.71 | 0.99 | 0.90 | 403 | 33,89 | 219 | 39,32 | 1516 | 489 |  |
| MTRR ---- tag | rs161869 | C/C | 1.00 (.-.) | . | . | . | 410 | 34,48 | 198 | 35,55 | 1516 | 489 |  |
| MTRR ---- tag |  | C/T or T/T | 1.05 (0.87-1.26) | 0.63 | 0.99 | 0.90 | 779 | 65,52 | 359 | 64,45 | 1516 | 489 |  |
| MTRR ---- tagged by rs162039 | rs162036 | A/A | 1.00 (.-.) | . | . | . | 958 | 80,57 | 444 | 79,71 | 1516 | 489 |  |
| MTRR ---- tagged by rs162039 |  | A/G or G/G | 1.03 (0.83-1.29) | 0.78 | 0.99 | 0.90 | 231 | 19,43 | 113 | 20,29 | 1516 | 489 |  |
| MTRR ---- tag | rs162039 | C/C | 1.00 (.-.) | . | . | . | 959 | 80,66 | 444 | 79,71 | 1516 | 489 |  |
| MTRR ---- tag |  | C/T or T/T | 1.03 (0.83-1.29) | 0.78 | 0.99 | 0.90 | 230 | 19,34 | 113 | 20,29 | 1516 | 489 |  |
| MTRR ---- tag | rs162270 | G/G | 1.00 (.-.) | . | . | . | 842 | 70,82 | 388 | 69,66 | 1516 | 489 |  |
| MTRR ---- tag |  | G/T or T/T | 1.08 (0.89-1.32) | 0.42 | 0.93 | 0.90 | 347 | 29,18 | 169 | 30,34 | 1516 | 489 |  |
| MTRR ---- candidate | rs16879334 | C/C | 1.00 (.-.) | . | . | . | 1113 | 93,61 | 530 | 95,15 | 1516 | 489 |  |
| MTRR ---- candidate |  | C/G or G/G | 0.88 (0.58-1.34) | 0.54 | 0.97 | 0.90 | 76 | 6,39 | 27 | 4,85 | 1516 | 489 |  |
| MTRR ----candidate | rs1801394 | G/G | 1.00 (.-.) | . | . | . | 343 | 28,85 | 172 | 30,88 | 1516 | 489 |  |
| MTRR ----candidate |  | G/A or A/A | 0.96 (0.79-1.17) | 0.69 | 0.99 | 0.90 | 846 | 71,15 | 385 | 69,12 | 1516 | 489 |  |
| MTRR ---- tag | rs1802059 | G/G | 1.00 (.-.) | . | . | . | 462 | 38,86 | 207 | 37,16 | 1516 | 489 |  |
| MTRR ---- tag |  | G/A or A/A | 0.98 (0.82-1.18) | 0.84 | 0.99 | 0.90 | 727 | 61,14 | 350 | 62,84 | 1516 | 489 |  |
| MTRR ---- tag | rs2077744 | T/T | 1.00 (.-.) | . | . | . | 866 | 72,83 | 403 | 72,35 | 1516 | 489 |  |
| MTRR ---- tag |  | T/C or C/C | 1.06 (0.87-1.29) | 0.58 | 0.99 | 0.90 | 323 | 27,17 | 154 | 27,65 | 1516 | 489 |  |
| MTRR ---- candidate | rs2287780 | C/C | 1.00 (.-.) | . | . | . | 1113 | 93,61 | 530 | 95,15 | 1516 | 489 |  |
| MTRR ---- candidate |  | C/T or T/T | 0.88 (0.58-1.34) | 0.54 | 0.97 | 0.90 | 76 | 6,39 | 27 | 4,85 | 1516 | 489 |  |
| MTRR ---- candidate | rs2303080 | T/T | 1.00 (.-.) | . | . | . | 1113 | 93,61 | 531 | 95,33 | 1516 | 489 |  |
| MTRR ---- candidate |  | T/A or A/A | 0.87 (0.56-1.33) | 0.51 | 0.96 | 0.90 | 76 | 6,39 | 26 | 4,67 | 1516 | 489 |  |
| MTRR ---- tag | rs7715062 | G/G | 1.00 (.-.) | . | . | . | 408 | 34,31 | 176 | 31,60 | 1516 | 489 |  |
| MTRR ---- tag |  | G/T or T/T | 1.00 (0.83-1.21) | 0.99 | 1.00 | 0.99 | 781 | 65,69 | 381 | 68,40 | 1516 | 489 |  |
| MTRR ---- tag | rs9282787 | T/T | 1.00 (.-.) | . | . | . | 793 | 66,69 | 340 | 61,04 | 1516 | 489 |  |
| MTRR ---- tag |  | T/C or C/C | 1.08 (0.90-1.30) | 0.42 | 0.93 | 0.90 | 396 | 33,31 | 217 | 38,96 | 1516 | 489 |  |
| MTRR ---- candidate literature | rs9332 | C/C | 1.00 (.-.) | . | . | . | 959 | 80,66 | 444 | 79,71 | 1516 | 489 |  |
| MTRR ---- candidate literature |  | C/T or T/T | 1.03 (0.83-1.29) | 0.78 | 0.99 | 0.90 | 230 | 19,34 | 113 | 20,29 | 1516 | 489 |  |
| NFKB1 ---- NA | rs1609798 | C/C | 1.00 (.-.) | . | . | . | 557 | 46,85 | 262 | 47,04 | 1516 | 489 |  |
| NFKB1 ---- NA |  | C/T or T/T | 0.99 (0.83-1.19) | 0.92 | 0.99 | 0.96 | 632 | 53,15 | 295 | 52,96 | 1516 | 489 |  |
| NFKB1 ---- tag | rs230540 | T/T | 1.00 (.-.) | . | . | . | 510 | 42,89 | 232 | 41,65 | 1516 | 489 |  |
| NFKB1 ---- tag |  | T/C or C/C | 0.94 (0.78-1.13) | 0.51 | 0.96 | 0.96 | 679 | 57,11 | 325 | 58,35 | 1516 | 489 |  |
| NFKB1 ---- tag | rs230541 | A/A | 1.00 (.-.) | . | . | . | 407 | 34,23 | 188 | 33,75 | 1516 | 489 |  |
| NFKB1 ---- tag |  | A/G or G/G | 0.97 (0.80-1.18) | 0.78 | 0.99 | 0.96 | 782 | 65,77 | 369 | 66,25 | 1516 | 489 |  |
| NFKB1 ---- NA | rs230547 | C/C | 1.00 (.-.) | . | . | . | 965 | 81,16 | 453 | 81,33 | 1516 | 489 |  |
| NFKB1 ---- NA |  | C/T or T/T | 1.01 (0.80-1.26) | 0.96 | 0.99 | 0.96 | 224 | 18,84 | 104 | 18,67 | 1516 | 489 |  |
| NFKB1 ---- tag | rs3774934 | G/G | 1.00 (.-.) | . | . | . | 950 | 79,90 | 441 | 79,17 | 1516 | 489 |  |
| NFKB1 ---- tag |  | G/A or A/A | 1.02 (0.82-1.26) | 0.87 | 0.99 | 0.96 | 239 | 20,10 | 116 | 20,83 | 1516 | 489 |  |
| NFKB1 ---- tag | rs3774968 | G/G | 1.00 (.-.) | . | . | . | 389 | 32,72 | 167 | 29,98 | 1516 | 489 |  |
| NFKB1 ---- tag |  | G/A or A/A | 1.16 (0.95-1.41) | 0.15 | 0.83 | 0.96 | 800 | 67,28 | 390 | 70,02 | 1516 | 489 |  |
| NFKB1 ---- NA | rs4648022 | C/C | 1.00 (.-.) | . | . | . | 981 | 82,51 | 479 | 86,00 | 1516 | 489 |  |
| NFKB1 ---- NA |  | C/T or T/T | 1.02 (0.79-1.32) | 0.86 | 0.99 | 0.96 | 208 | 17,49 | 78 | 14,00 | 1516 | 489 |  |
| NFKB1 ---- NA | rs4648090 | G/G | 1.00 (.-.) | . | . | . | 857 | 72,08 | 426 | 76,48 | 1516 | 489 |  |
| NFKB1 ---- NA |  | G/A or A/A | 0.95 (0.77-1.17) | 0.61 | 0.99 | 0.96 | 332 | 27,92 | 131 | 23,52 | 1516 | 489 |  |
| NFKB1 ---- tag | rs4648110 | T/T | 1.00 (.-.) | . | . | . | 752 | 63,25 | 356 | 63,91 | 1516 | 489 |  |
| NFKB1 ---- tag |  | T/A or A/A | 1.05 (0.88-1.27) | 0.58 | 0.99 | 0.96 | 437 | 36,75 | 201 | 36,09 | 1516 | 489 |  |
| NFKB1 ---- tag | rs4648141 | G/G | 1.00 (.-.) | . | . | . | 817 | 68,71 | 388 | 69,66 | 1516 | 489 |  |
| NFKB1 ---- tag |  | G/A or A/A | 1.08 (0.89-1.32) | 0.42 | 0.93 | 0.96 | 372 | 31,29 | 169 | 30,34 | 1516 | 489 |  |
| NFKB1 ---- tag | rs4698863 | C/C | 1.00 (.-.) | . | . | . | 545 | 45,84 | 258 | 46,32 | 1516 | 489 |  |
| NFKB1 ---- tag |  | C/T or T/T | 0.95 (0.80-1.14) | 0.59 | 0.99 | 0.96 | 644 | 54,16 | 299 | 53,68 | 1516 | 489 |  |
| NFKB1 ---- NA | rs7674640 | T/T | 1.00 (.-.) | . | . | . | 273 | 22,96 | 126 | 22,62 | 1516 | 489 |  |
| NFKB1 ---- NA |  | T/C or C/C | 1.01 (0.82-1.25) | 0.94 | 0.99 | 0.96 | 916 | 77,04 | 431 | 77,38 | 1516 | 489 |  |
| NFKB1 ---- tag | rs909332 | A/A | 1.00 (.-.) | . | . | . | 1073 | 90,24 | 512 | 91,92 | 1516 | 489 |  |
| NFKB1 ---- tag |  | A/T or T/T | 1.03 (0.74-1.43) | 0.85 | 0.99 | 0.96 | 116 | 9,76 | 45 | 8,08 | 1516 | 489 |  |
| NFKB1 ---- tag | rs997476 | C/C | 1.00 (.-.) | . | . | . | 1068 | 89,82 | 482 | 86,54 | 1516 | 489 |  |
| NFKB1 ---- tag |  | C/A or A/A | 1.28 (0.99-1.66) | 0.06 | 0.66 | 0.90 | 121 | 10,18 | 75 | 13,46 | 1516 | 489 |  |
| NME1 ---- NA | rs10514981 | T/T | 1.00 (.-.) | . | . | . | 721 | 60,64 | 360 | 64,63 | 1516 | 489 |  |
| NME1 ---- NA |  | T/G or G/G | 0.89 (0.74-1.07) | 0.21 | 0.85 | 0.89 | 468 | 39,36 | 197 | 35,37 | 1516 | 489 |  |
| NME1 ---- NA | rs11651252 | T/T | 1.00 (.-.) | . | . | . | 1056 | 88,81 | 495 | 88,87 | 1516 | 489 |  |
| NME1 ---- NA |  | T/C or C/C | 0.93 (0.70-1.24) | 0.63 | 0.99 | 0.89 | 133 | 11,19 | 62 | 11,13 | 1516 | 489 |  |
| NME1 ---- tag | rs11652793 | T/T | 1.00 (.-.) | . | . | . | 773 | 65,01 | 389 | 69,84 | 1516 | 489 |  |
| NME1 ---- tag |  | T/C or C/C | 0.92 (0.76-1.12) | 0.43 | 0.93 | 0.89 | 416 | 34,99 | 168 | 30,16 | 1516 | 489 |  |
| NME1 ---- NA | rs11868380 | C/C | 1.00 (.-.) | . | . | . | 750 | 63,08 | 343 | 61,58 | 1516 | 489 |  |
| NME1 ---- NA |  | C/G or G/G | 1.00 (0.83-1.20) | 0.99 | 1.00 | 0.99 | 439 | 36,92 | 214 | 38,42 | 1516 | 489 |  |
| NME1 ---- NA | rs1558252 | T/T | 1.00 (.-.) | . | . | . | 576 | 48,44 | 256 | 45,96 | 1516 | 489 |  |
| NME1 ---- NA |  | T/C or C/C | 1.01 (0.85-1.21) | 0.87 | 0.99 | 0.99 | 613 | 51,56 | 301 | 54,04 | 1516 | 489 |  |
| NME1 ---- NA | rs1558253 | T/T | 1.00 (.-.) | . | . | . | 1058 | 88,98 | 483 | 86,71 | 1516 | 489 |  |
| NME1 ---- NA |  | T/G or G/G | 1.14 (0.88-1.48) | 0.32 | 0.90 | 0.89 | 131 | 11,02 | 74 | 13,29 | 1516 | 489 |  |
| NME1 ---- tag | rs16949683 | C/C | 1.00 (.-.) | . | . | . | 1107 | 93,10 | 512 | 91,92 | 1516 | 489 |  |
| NME1 ---- tag |  | C/T or T/T | 1.09 (0.78-1.52) | 0.61 | 0.99 | 0.89 | 82 | 6,90 | 45 | 8,08 | 1516 | 489 |  |
| NME1 ---- tag | rs2318784 | C/C | 1.00 (.-.) | . | . | . | 909 | 76,45 | 425 | 76,30 | 1516 | 489 |  |
| NME1 ---- tag |  | C/T or T/T | 1.05 (0.85-1.30) | 0.65 | 0.99 | 0.89 | 280 | 23,55 | 132 | 23,70 | 1516 | 489 |  |
| NME1 ---- NA | rs2318785 | G/G | 1.00 (.-.) | . | . | . | 372 | 31,29 | 176 | 31,60 | 1516 | 489 |  |
| NME1 ---- NA |  | G/A or A/A | 0.95 (0.78-1.15) | 0.60 | 0.99 | 0.89 | 817 | 68,71 | 381 | 68,40 | 1516 | 489 |  |
| NME1 ---- tag | rs3760469 | G/G | 1.00 (.-.) | . | . | . | 310 | 26,07 | 138 | 24,78 | 1516 | 489 |  |
| NME1 ---- tag |  | G/T or T/T | 1.01 (0.82-1.24) | 0.95 | 0.99 | 0.99 | 879 | 73,93 | 419 | 75,22 | 1516 | 489 |  |
| NME1 ---- NA | rs4605213 | G/G | 1.00 (.-.) | . | . | . | 494 | 41,55 | 250 | 44,88 | 1516 | 489 |  |
| NME1 ---- NA |  | G/C or C/C | 0.93 (0.78-1.12) | 0.45 | 0.93 | 0.89 | 695 | 58,45 | 307 | 55,12 | 1516 | 489 |  |
| NME1 ---- NA | rs7207090 | A/A | 1.00 (.-.) | . | . | . | 306 | 25,74 | 132 | 23,70 | 1516 | 489 |  |
| NME1 ---- NA |  | A/T or T/T | 0.95 (0.77-1.18) | 0.65 | 0.99 | 0.89 | 883 | 74,26 | 425 | 76,30 | 1516 | 489 |  |
| NME1 ---- tag | rs7222463 | A/A | 1.00 (.-.) | . | . | . | 314 | 26,41 | 160 | 28,73 | 1516 | 489 |  |
| NME1 ---- tag |  | A/C or C/C | 0.92 (0.75-1.12) | 0.39 | 0.93 | 0.89 | 875 | 73,59 | 397 | 71,27 | 1516 | 489 |  |
| NME1 ---- tag | rs7226059 | C/C | 1.00 (.-.) | . | . | . | 550 | 46,26 | 235 | 42,19 | 1516 | 489 |  |
| NME1 ---- tag |  | C/T or T/T | 1.14 (0.95-1.37) | 0.15 | 0.83 | 0.89 | 639 | 53,74 | 322 | 57,81 | 1516 | 489 |  |
| NME1 ---- NA | rs880178 | G/G | 1.00 (.-.) | . | . | . | 307 | 25,82 | 135 | 24,24 | 1516 | 489 |  |
| NME1 ---- NA |  | G/T or T/T | 1.02 (0.83-1.26) | 0.84 | 0.99 | 0.99 | 882 | 74,18 | 422 | 75,76 | 1516 | 489 |  |
| NME2 ---- tag | rs7220360 | C/C | 1.00 (.-.) | . | . | . | 314 | 26,41 | 158 | 28,37 | 1516 | 489 |  |
| NME2 ---- tag |  | C/G or G/G | 0.92 (0.76-1.13) | 0.43 | 0.93 | 0.43 | 875 | 73,59 | 399 | 71,63 | 1516 | 489 |  |
| PON1 ---- tag | rs2269829 | A/A | 1.00 (.-.) | . | . | . | 623 | 52,40 | 253 | 45,42 | 1516 | 489 |  |
| PON1 ---- tag |  | A/G or G/G | 1.18 (0.99-1.41) | 0.07 | 0.66 | 0.08 | 566 | 47,60 | 304 | 54,58 | 1516 | 489 |  |
| PON1 ---- tag | rs3917527 | A/A | 1.00 (.-.) | . | . | . | 1058 | 88,98 | 513 | 92,10 | 1516 | 489 |  |
| PON1 ---- tag |  | A/G or G/G | 0.72 (0.53-0.99) | 0.05 | 0.59 | 0.08 | 131 | 11,02 | 44 | 7,90 | 1516 | 489 |  |
| PON1 ---- tag | rs3917538 | C/C | 1.00 (.-.) | . | . | . | 707 | 59,46 | 282 | 50,63 | 1516 | 489 |  |
| PON1 ---- tag |  | C/T or T/T | 1.28 (1.07-1.53) | 0.01 | 0.59 | 0.04 | 482 | 40,54 | 275 | 49,37 | 1516 | 489 |  |
| PON1 ---- tag | rs757158 | C/C | 1.00 (.-.) | . | . | . | 415 | 34,90 | 206 | 36,98 | 1516 | 489 |  |
| PON1 ---- tag |  | C/T or T/T | 0.83 (0.69-1.00) | 0.05 | 0.61 | 0.08 | 774 | 65,10 | 351 | 63,02 | 1516 | 489 |  |
| PON1 ---- candidate | rs854560 | A/A | 1.00 (.-.) | . | . | . | 463 | 38,94 | 236 | 42,37 | 1516 | 489 |  |
| PON1 ---- candidate |  | A/T or T/T | 0.98 (0.82-1.18) | 0.87 | 0.99 | 0.87 | 726 | 61,06 | 321 | 57,63 | 1516 | 489 |  |
| PRDM2 ---- tag | rs1015370 | C/C | 1.00 (.-.) | . | . | . | 599 | 50,38 | 305 | 54,76 | 1516 | 489 |  |
| PRDM2 ---- tag |  | C/T or T/T | 0.91 (0.76-1.08) | 0.28 | 0.88 | 0.60 | 590 | 49,62 | 252 | 45,24 | 1516 | 489 |  |
| PRDM2 ---- tag | rs1203634 | A/A | 1.00 (.-.) | . | . | . | 737 | 61,98 | 328 | 58,89 | 1516 | 489 |  |
| PRDM2 ---- tag |  | A/G or G/G | 1.21 (1.01-1.45) | 0.04 | 0.59 | 0.16 | 452 | 38,02 | 229 | 41,11 | 1516 | 489 |  |
| PRDM2 ---- tag | rs1203645 | A/A | 1.00 (.-.) | . | . | . | 497 | 41,80 | 216 | 38,78 | 1516 | 489 |  |
| PRDM2 ---- tag |  | A/C or C/C | 1.25 (1.04-1.51) | 0.02 | 0.59 | 0.16 | 692 | 58,20 | 341 | 61,22 | 1516 | 489 |  |
| PRDM2 ---- tag | rs1406416 | C/C | 1.00 (.-.) | . | . | . | 642 | 53,99 | 298 | 53,50 | 1516 | 489 |  |
| PRDM2 ---- tag |  | C/T or T/T | 0.96 (0.80-1.15) | 0.68 | 0.99 | 0.87 | 547 | 46,01 | 259 | 46,50 | 1516 | 489 |  |
| PRDM2 ---- candidate | rs17350795 | G/G | 1.00 (.-.) | . | . | . | 1140 | 95,88 | 531 | 95,33 | 1516 | 489 |  |
| PRDM2 ---- candidate |  | G/A or A/A | 0.93 (0.60-1.43) | 0.73 | 0.99 | 0.87 | 49 | 4,12 | 26 | 4,67 | 1516 | 489 |  |
| PRDM2 ---- tag | rs1980472 | C/C | 1.00 (.-.) | . | . | . | 682 | 57,36 | 292 | 52,42 | 1516 | 489 |  |
| PRDM2 ---- tag |  | C/G or G/G | 1.22 (1.02-1.46) | 0.03 | 0.59 | 0.16 | 507 | 42,64 | 265 | 47,58 | 1516 | 489 |  |
| PRDM2 ---- tag | rs2235515 | G/G | 1.00 (.-.) | . | . | . | 704 | 59,21 | 313 | 56,19 | 1516 | 489 |  |
| PRDM2 ---- tag |  | G/A or A/A | 1.15 (0.96-1.37) | 0.14 | 0.83 | 0.43 | 485 | 40,79 | 244 | 43,81 | 1516 | 489 |  |
| PRDM2 ---- tag | rs2244634 | A/A | 1.00 (.-.) | . | . | . | 761 | 64,00 | 360 | 64,63 | 1516 | 489 |  |
| PRDM2 ---- tag |  | A/C or C/C | 1.00 (0.83-1.21) | 0.97 | 0.99 | 0.99 | 428 | 36,00 | 197 | 35,37 | 1516 | 489 |  |
| PRDM2 ---- tag | rs2245213 | G/G | 1.00 (.-.) | . | . | . | 841 | 70,73 | 389 | 69,84 | 1516 | 489 |  |
| PRDM2 ---- tag |  | G/T or T/T | 1.06 (0.87-1.29) | 0.54 | 0.97 | 0.87 | 348 | 29,27 | 168 | 30,16 | 1516 | 489 |  |
| PRDM2 ---- tag | rs2294484 | C/C | 1.00 (.-.) | . | . | . | 988 | 83,10 | 470 | 84,38 | 1516 | 489 |  |
| PRDM2 ---- tag |  | C/G or G/G | 1.07 (0.83-1.37) | 0.61 | 0.99 | 0.87 | 201 | 16,90 | 87 | 15,62 | 1516 | 489 |  |
| PRDM2 ---- tag | rs2744689 | G/G | 1.00 (.-.) | . | . | . | 849 | 71,40 | 398 | 71,45 | 1516 | 489 |  |
| PRDM2 ---- tag |  | G/A or A/A | 1.00 (0.82-1.22) | 0.99 | 1.00 | 0.99 | 340 | 28,60 | 159 | 28,55 | 1516 | 489 |  |
| PRDM2 ---- tag | rs6690270 | A/A | 1.00 (.-.) | . | . | . | 478 | 40,20 | 222 | 39,86 | 1516 | 489 |  |
| PRDM2 ---- tag |  | A/G or G/G | 0.91 (0.76-1.09) | 0.30 | 0.89 | 0.60 | 711 | 59,80 | 335 | 60,14 | 1516 | 489 |  |
| RRM1 ---- tag | rs10835601 | G/G | 1.00 (.-.) | . | . | . | 578 | 48,61 | 269 | 48,29 | 1516 | 489 |  |
| RRM1 ---- tag |  | G/A or A/A | 1.00 (0.84-1.20) | 1.00 | 1.00 | 1.00 | 611 | 51,39 | 288 | 51,71 | 1516 | 489 |  |
| RRM1 ---- tag | rs10835613 | C/C | 1.00 (.-.) | . | . | . | 397 | 33,39 | 193 | 34,65 | 1516 | 489 |  |
| RRM1 ---- tag |  | C/G or G/G | 0.97 (0.80-1.17) | 0.75 | 0.99 | 0.94 | 792 | 66,61 | 364 | 65,35 | 1516 | 489 |  |
| RRM1 ---- NA | rs10835677 | G/G | 1.00 (.-.) | . | . | . | 992 | 83,43 | 457 | 82,05 | 1516 | 489 |  |
| RRM1 ---- NA |  | G/A or A/A | 0.96 (0.76-1.22) | 0.75 | 0.99 | 0.94 | 197 | 16,57 | 100 | 17,95 | 1516 | 489 |  |
| RRM1 ---- tag | rs10835678 | A/A | 1.00 (.-.) | . | . | . | 1055 | 88,73 | 496 | 89,05 | 1516 | 489 |  |
| RRM1 ---- tag |  | A/G or G/G | 0.93 (0.70-1.25) | 0.63 | 0.99 | 0.94 | 134 | 11,27 | 61 | 10,95 | 1516 | 489 |  |
| RRM1 ---- tag | rs12288551 | C/C | 1.00 (.-.) | . | . | . | 1102 | 92,68 | 507 | 91,02 | 1516 | 489 |  |
| RRM1 ---- tag |  | C/G or G/G | 1.16 (0.85-1.59) | 0.36 | 0.93 | 0.94 | 87 | 7,32 | 50 | 8,98 | 1516 | 489 |  |
| RRM1 ---- NA | rs12806698 | C/C | 1.00 (.-.) | . | . | . | 620 | 52,14 | 282 | 50,63 | 1516 | 489 |  |
| RRM1 ---- NA |  | C/A or A/A | 1.05 (0.88-1.26) | 0.61 | 0.99 | 0.94 | 569 | 47,86 | 275 | 49,37 | 1516 | 489 |  |
| RRM1 ---- NA | rs1465952 | T/T | 1.00 (.-.) | . | . | . | 970 | 81,58 | 458 | 82,23 | 1516 | 489 |  |
| RRM1 ---- NA |  | T/C or C/C | 0.99 (0.78-1.25) | 0.90 | 0.99 | 1.00 | 219 | 18,42 | 99 | 17,77 | 1516 | 489 |  |
| RRM1 ---- tag | rs4910904 | A/A | 1.00 (.-.) | . | . | . | 519 | 43,65 | 231 | 41,47 | 1516 | 489 |  |
| RRM1 ---- tag |  | A/G or G/G | 1.08 (0.90-1.29) | 0.43 | 0.93 | 0.94 | 670 | 56,35 | 326 | 58,53 | 1516 | 489 |  |
| RRM1 ---- tag | rs7103860 | T/T | 1.00 (.-.) | . | . | . | 903 | 75,95 | 429 | 77,02 | 1516 | 489 |  |
| RRM1 ---- tag |  | T/C or C/C | 1.09 (0.88-1.35) | 0.42 | 0.93 | 0.94 | 286 | 24,05 | 128 | 22,98 | 1516 | 489 |  |
| RRM1 ---- tag | rs7115496 | C/C | 1.00 (.-.) | . | . | . | 1008 | 84,78 | 485 | 87,07 | 1516 | 489 |  |
| RRM1 ---- tag |  | C/T or T/T | 0.92 (0.71-1.20) | 0.55 | 0.98 | 0.94 | 181 | 15,22 | 72 | 12,93 | 1516 | 489 |  |
| RRM2 ---- NA | rs1138729 | A/A | 1.00 (.-.) | . | . | . | 862 | 72,50 | 409 | 73,43 | 1516 | 489 |  |
| RRM2 ---- NA |  | A/G or G/G | 1.12 (0.92-1.37) | 0.27 | 0.88 | 0.77 | 327 | 27,50 | 148 | 26,57 | 1516 | 489 |  |
| RRM2 ---- tag | rs4668664 | G/G | 1.00 (.-.) | . | . | . | 594 | 49,96 | 284 | 50,99 | 1516 | 489 |  |
| RRM2 ---- tag |  | G/A or A/A | 0.93 (0.78-1.11) | 0.42 | 0.93 | 0.77 | 595 | 50,04 | 273 | 49,01 | 1516 | 489 |  |
| RRM2 ---- NA | rs6741290 | C/C | 1.00 (.-.) | . | . | . | 371 | 31,20 | 187 | 33,57 | 1516 | 489 |  |
| RRM2 ---- NA |  | C/T or T/T | 0.97 (0.80-1.17) | 0.73 | 0.99 | 0.77 | 818 | 68,80 | 370 | 66,43 | 1516 | 489 |  |
| RRM2 ---- tag | rs7574663 | C/C | 1.00 (.-.) | . | . | . | 769 | 64,68 | 372 | 66,79 | 1516 | 489 |  |
| RRM2 ---- tag |  | C/G or G/G | 1.03 (0.85-1.24) | 0.77 | 0.99 | 0.77 | 420 | 35,32 | 185 | 33,21 | 1516 | 489 |  |
| SHMT1 ---- candidate | rs1979277 | G/G | 1.00 (.-.) | . | . | . | 578 | 48,61 | 277 | 49,73 | 1516 | 489 |  |
| SHMT1 ---- candidate |  | G/A or A/A | 0.98 (0.82-1.17) | 0.84 | 0.99 | 0.93 | 611 | 51,39 | 280 | 50,27 | 1516 | 489 |  |
| SHMT1 ---- tag | rs2168781 | G/G | 1.00 (.-.) | . | . | . | 415 | 34,90 | 206 | 36,98 | 1516 | 489 |  |
| SHMT1 ---- tag |  | G/C or C/C | 1.02 (0.85-1.23) | 0.83 | 0.99 | 0.93 | 774 | 65,10 | 351 | 63,02 | 1516 | 489 |  |
| SHMT1 ---- tag | rs4924849 | C/C | 1.00 (.-.) | . | . | . | 603 | 50,71 | 292 | 52,42 | 1516 | 489 |  |
| SHMT1 ---- tag |  | C/T or T/T | 0.97 (0.81-1.16) | 0.74 | 0.99 | 0.93 | 586 | 49,29 | 265 | 47,58 | 1516 | 489 |  |
| SHMT1 ---- candidate literature | rs9909104 | T/T | 1.00 (.-.) | . | . | . | 624 | 52,48 | 287 | 51,53 | 1516 | 489 |  |
| SHMT1 ---- candidate literature |  | T/C or C/C | 0.99 (0.83-1.19) | 0.93 | 0.99 | 0.93 | 565 | 47,52 | 270 | 48,47 | 1516 | 489 |  |
| SHMT2 ---- tag | rs10876968 | G/G | 1.00 (.-.) | . | . | . | 640 | 53,83 | 307 | 55,12 | 1516 | 489 |  |
| SHMT2 ---- tag |  | G/T or T/T | 0.96 (0.80-1.15) | 0.68 | 0.99 | 0.81 | 549 | 46,17 | 250 | 44,88 | 1516 | 489 |  |
| SHMT2 ---- tag | rs1800165 | T/T | 1.00 (.-.) | . | . | . | 585 | 49,20 | 264 | 47,40 | 1516 | 489 |  |
| SHMT2 ---- tag |  | T/C or C/C | 1.14 (0.95-1.36) | 0.16 | 0.83 | 0.38 | 604 | 50,80 | 293 | 52,60 | 1516 | 489 |  |
| SHMT2 ---- tag | rs7133939 | T/T | 1.00 (.-.) | . | . | . | 359 | 30,19 | 173 | 31,06 | 1516 | 489 |  |
| SHMT2 ---- tag |  | T/A or A/A | 1.02 (0.84-1.24) | 0.81 | 0.99 | 0.81 | 830 | 69,81 | 384 | 68,94 | 1516 | 489 |  |
| SHMT2 ---- tag | rs7485577 | G/G | 1.00 (.-.) | . | . | . | 645 | 54,25 | 290 | 52,06 | 1516 | 489 |  |
| SHMT2 ---- tag |  | G/A or A/A | 1.12 (0.93-1.34) | 0.23 | 0.85 | 0.38 | 544 | 45,75 | 267 | 47,94 | 1516 | 489 |  |
| SHMT2 ---- tag | rs7489231 | T/T | 1.00 (.-.) | . | . | . | 526 | 44,24 | 241 | 43,27 | 1516 | 489 |  |
| SHMT2 ---- tag |  | T/C or C/C | 1.16 (0.97-1.40) | 0.10 | 0.79 | 0.38 | 663 | 55,76 | 316 | 56,73 | 1516 | 489 |  |
| SLC19A1 ---- candidate | rs1051266 | G/G | 1.00 (.-.) | . | . | . | 370 | 31,12 | 171 | 30,70 | 1516 | 489 |  |
| SLC19A1 ---- candidate |  | G/A or A/A | 0.89 (0.73-1.08) | 0.23 | 0.85 | 0.33 | 819 | 68,88 | 386 | 69,30 | 1516 | 489 |  |
| SLC19A1 ---- candidate literature | rs1131596 | T/T | 1.00 (.-.) | . | . | . | 371 | 31,20 | 171 | 30,70 | 1516 | 489 |  |
| SLC19A1 ---- candidate literature |  | T/C or C/C | 0.89 (0.74-1.08) | 0.25 | 0.86 | 0.33 | 818 | 68,80 | 386 | 69,30 | 1516 | 489 |  |
| SLC19A1 ---- tag | rs12483553 | G/G | 1.00 (.-.) | . | . | . | 969 | 81,50 | 434 | 77,92 | 1516 | 489 |  |
| SLC19A1 ---- tag |  | G/A or A/A | 1.18 (0.95-1.46) | 0.14 | 0.83 | 0.33 | 220 | 18,50 | 123 | 22,08 | 1516 | 489 |  |
| SLC19A1 ---- candidate literature | rs12659 | C/C | 1.00 (.-.) | . | . | . | 383 | 32,21 | 180 | 32,32 | 1516 | 489 |  |
| SLC19A1 ---- candidate literature |  | C/T or T/T | 0.88 (0.73-1.06) | 0.18 | 0.85 | 0.33 | 806 | 67,79 | 377 | 67,68 | 1516 | 489 |  |
| SLC19A1 ---- tag | rs3788190 | G/G | 1.00 (.-.) | . | . | . | 368 | 30,95 | 164 | 29,44 | 1516 | 489 |  |
| SLC19A1 ---- tag |  | G/A or A/A | 0.90 (0.74-1.09) | 0.28 | 0.88 | 0.33 | 821 | 69,05 | 393 | 70,56 | 1516 | 489 |  |
| SLC19A1 ---- tag | rs3788205 | C/C | 1.00 (.-.) | . | . | . | 570 | 47,94 | 301 | 54,04 | 1516 | 489 |  |
| SLC19A1 ---- tag |  | C/T or T/T | 0.93 (0.78-1.12) | 0.46 | 0.93 | 0.46 | 619 | 52,06 | 256 | 45,96 | 1516 | 489 |  |
| SLC19A1 ---- tag | rs7279664 | G/G | 1.00 (.-.) | . | . | . | 458 | 38,52 | 230 | 41,29 | 1516 | 489 |  |
| SLC19A1 ---- tag |  | G/T or T/T | 0.86 (0.72-1.04) | 0.12 | 0.82 | 0.33 | 731 | 61,48 | 327 | 58,71 | 1516 | 489 |  |
| SLC29A1 ---- NA | rs1057985 | C/C | 1.00 (.-.) | . | . | . | 513 | 43,15 | 236 | 42,37 | 1516 | 489 |  |
| SLC29A1 ---- NA |  | C/T or T/T | 1.15 (0.96-1.38) | 0.13 | 0.83 | 0.31 | 676 | 56,85 | 321 | 57,63 | 1516 | 489 |  |
| SLC29A1 ---- NA | rs6458375 | C/C | 1.00 (.-.) | . | . | . | 663 | 55,76 | 322 | 57,81 | 1516 | 489 |  |
| SLC29A1 ---- NA |  | C/T or T/T | 0.82 (0.69-0.98) | 0.03 | 0.59 | 0.18 | 526 | 44,24 | 235 | 42,19 | 1516 | 489 |  |
| SLC29A1 ---- NA | rs666462 | C/C | 1.00 (.-.) | . | . | . | 322 | 27,08 | 160 | 28,73 | 1516 | 489 |  |
| SLC29A1 ---- NA |  | C/T or T/T | 0.99 (0.81-1.21) | 0.90 | 0.99 | 0.90 | 867 | 72,92 | 397 | 71,27 | 1516 | 489 |  |
| SLC29A1 ---- NA | rs6905285 | A/A | 1.00 (.-.) | . | . | . | 443 | 37,26 | 195 | 35,01 | 1516 | 489 |  |
| SLC29A1 ---- NA |  | A/T or T/T | 0.93 (0.77-1.13) | 0.47 | 0.94 | 0.65 | 746 | 62,74 | 362 | 64,99 | 1516 | 489 |  |
| SLC29A1 ---- NA | rs693955 | G/G | 1.00 (.-.) | . | . | . | 789 | 66,36 | 366 | 65,71 | 1516 | 489 |  |
| SLC29A1 ---- NA |  | G/T or T/T | 1.21 (1.00-1.46) | 0.05 | 0.63 | 0.18 | 400 | 33,64 | 191 | 34,29 | 1516 | 489 |  |
| SLC29A1 ---- NA | rs747199 | C/C | 1.00 (.-.) | . | . | . | 783 | 65,85 | 349 | 62,66 | 1516 | 489 |  |
| SLC29A1 ---- NA |  | C/G or G/G | 1.05 (0.87-1.27) | 0.59 | 0.99 | 0.68 | 406 | 34,15 | 208 | 37,34 | 1516 | 489 |  |
| SLC29A1 ---- NA | rs9357436 | G/G | 1.00 (.-.) | . | . | . | 842 | 70,82 | 384 | 68,94 | 1516 | 489 |  |
| SLC29A1 ---- NA |  | G/A or A/A | 1.12 (0.92-1.36) | 0.24 | 0.86 | 0.43 | 347 | 29,18 | 173 | 31,06 | 1516 | 489 |  |
| TCN2 ---- tag | rs10418 | C/C | 1.00 (.-.) | . | . | . | 686 | 57,70 | 326 | 58,53 | 1516 | 489 |  |
| TCN2 ---- tag |  | C/T or T/T | 1.01 (0.84-1.21) | 0.92 | 0.99 | 0.92 | 503 | 42,30 | 231 | 41,47 | 1516 | 489 |  |
| TCN2 ---- candidate/singleton | rs1131603 | T/T | 1.00 (.-.) | . | . | . | 1058 | 88,98 | 487 | 87,43 | 1516 | 489 |  |
| TCN2 ---- candidate/singleton |  | T/C or C/C | 1.08 (0.82-1.42) | 0.59 | 0.99 | 0.91 | 131 | 11,02 | 70 | 12,57 | 1516 | 489 |  |
| TCN2 ---- tag | rs1544468 | A/A | 1.00 (.-.) | . | . | . | 300 | 25,23 | 137 | 24,60 | 1516 | 489 |  |
| TCN2 ---- tag |  | A/G or G/G | 1.07 (0.87-1.32) | 0.51 | 0.96 | 0.91 | 889 | 74,77 | 420 | 75,40 | 1516 | 489 |  |
| TCN2 ---- candidate/tag | rs1801198 | C/C | 1.00 (.-.) | . | . | . | 352 | 29,60 | 175 | 31,42 | 1516 | 489 |  |
| TCN2 ---- candidate/tag |  | C/G or G/G | 0.93 (0.77-1.13) | 0.49 | 0.95 | 0.91 | 837 | 70,40 | 382 | 68,58 | 1516 | 489 |  |
| TCN2 ---- tag | rs4820872 | G/G | 1.00 (.-.) | . | . | . | 432 | 36,33 | 214 | 38,42 | 1516 | 489 |  |
| TCN2 ---- tag |  | G/A or A/A | 0.98 (0.81-1.18) | 0.82 | 0.99 | 0.91 | 757 | 63,67 | 343 | 61,58 | 1516 | 489 |  |
| TCN2 ---- tag | rs4820874 | A/A | 1.00 (.-.) | . | . | . | 833 | 70,06 | 409 | 73,43 | 1516 | 489 |  |
| TCN2 ---- tag |  | A/G or G/G | 0.92 (0.75-1.13) | 0.43 | 0.93 | 0.91 | 356 | 29,94 | 148 | 26,57 | 1516 | 489 |  |
| TCN2 ---- tag | rs4820886 | T/T | 1.00 (.-.) | . | . | . | 949 | 79,81 | 453 | 81,33 | 1516 | 489 |  |
| TCN2 ---- tag |  | T/G or G/G | 1.02 (0.81-1.29) | 0.84 | 0.99 | 0.91 | 240 | 20,19 | 104 | 18,67 | 1516 | 489 |  |
| TCN2 ---- candidate | rs4820889 | G/G | 1.00 (.-.) | . | . | . | 1109 | 93,27 | 522 | 93,72 | 1516 | 489 |  |
| TCN2 ---- candidate |  | G/A or A/A | 0.86 (0.60-1.24) | 0.43 | 0.93 | 0.91 | 80 | 6,73 | 35 | 6,28 | 1516 | 489 |  |
| TCN2 ---- tag | rs5997711 | C/C | 1.00 (.-.) | . | . | . | 383 | 32,21 | 192 | 34,47 | 1516 | 489 |  |
| TCN2 ---- tag |  | C/T or T/T | 0.95 (0.79-1.15) | 0.62 | 0.99 | 0.91 | 806 | 67,79 | 365 | 65,53 | 1516 | 489 |  |
| TCN2 ---- tag | rs740234 | T/T | 1.00 (.-.) | . | . | . | 784 | 65,94 | 347 | 62,30 | 1516 | 489 |  |
| TCN2 ---- tag |  | T/C or C/C | 1.04 (0.86-1.25) | 0.70 | 0.99 | 0.91 | 405 | 34,06 | 210 | 37,70 | 1516 | 489 |  |
| TCN2 ---- tag | rs740235 | G/G | 1.00 (.-.) | . | . | . | 407 | 34,23 | 177 | 31,78 | 1516 | 489 |  |
| TCN2 ---- tag |  | G/A or A/A | 1.10 (0.91-1.34) | 0.31 | 0.90 | 0.91 | 782 | 65,77 | 380 | 68,22 | 1516 | 489 |  |
| TCN2 ---- candidate/singleton | rs9606756 | A/A | 1.00 (.-.) | . | . | . | 949 | 79,81 | 443 | 79,53 | 1516 | 489 |  |
| TCN2 ---- candidate/singleton |  | A/G or G/G | 1.09 (0.87-1.36) | 0.45 | 0.93 | 0.91 | 240 | 20,19 | 114 | 20,47 | 1516 | 489 |  |
| TCN2 ---- candidate | rs9621049 | C/C | 1.00 (.-.) | . | . | . | 949 | 79,81 | 453 | 81,33 | 1516 | 489 |  |
| TCN2 ---- candidate |  | C/T or T/T | 1.02 (0.81-1.29) | 0.84 | 0.99 | 0.91 | 240 | 20,19 | 104 | 18,67 | 1516 | 489 |  |
| TK1 ---- NA | rs1065769 | G/G | 1.00 (.-.) | . | . | . | 565 | 47,52 | 252 | 45,24 | 1516 | 489 |  |
| TK1 ---- NA |  | G/A or A/A | 1.10 (0.91-1.32) | 0.32 | 0.90 | 0.89 | 624 | 52,48 | 305 | 54,76 | 1516 | 489 |  |
| TK1 ---- NA | rs12232476 | G/G | 1.00 (.-.) | . | . | . | 999 | 84,02 | 467 | 83,84 | 1516 | 489 |  |
| TK1 ---- NA |  | G/A or A/A | 1.04 (0.81-1.32) | 0.76 | 0.99 | 0.89 | 190 | 15,98 | 90 | 16,16 | 1516 | 489 |  |
| TK1 ---- tag | rs16970907 | G/G | 1.00 (.-.) | . | . | . | 1027 | 86,38 | 484 | 86,89 | 1516 | 489 |  |
| TK1 ---- tag |  | G/C or C/C | 0.98 (0.75-1.29) | 0.89 | 0.99 | 0.89 | 162 | 13,62 | 73 | 13,11 | 1516 | 489 |  |
| TK1 ---- tag | rs1811086 | C/C | 1.00 (.-.) | . | . | . | 1106 | 93,02 | 516 | 92,64 | 1516 | 489 |  |
| TK1 ---- tag |  | C/T or T/T | 0.86 (0.60-1.24) | 0.42 | 0.93 | 0.89 | 83 | 6,98 | 41 | 7,36 | 1516 | 489 |  |
| TK1 ---- tag | rs2292235 | C/C | 1.00 (.-.) | . | . | . | 375 | 31,54 | 183 | 32,85 | 1516 | 489 |  |
| TK1 ---- tag |  | C/A or A/A | 0.98 (0.81-1.18) | 0.82 | 0.99 | 0.89 | 814 | 68,46 | 374 | 67,15 | 1516 | 489 |  |
| TK1 ---- tag | rs2854701 | A/A | 1.00 (.-.) | . | . | . | 485 | 40,79 | 208 | 37,34 | 1516 | 489 |  |
| TK1 ---- tag |  | A/G or G/G | 1.16 (0.97-1.40) | 0.11 | 0.82 | 0.89 | 704 | 59,21 | 349 | 62,66 | 1516 | 489 |  |
| TK1 ---- tag | rs2854702 | G/G | 1.00 (.-.) | . | . | . | 918 | 77,21 | 425 | 76,30 | 1516 | 489 |  |
| TK1 ---- tag |  | G/A or A/A | 0.98 (0.79-1.21) | 0.84 | 0.99 | 0.89 | 271 | 22,79 | 132 | 23,70 | 1516 | 489 |  |
| TK1 ---- tag | rs9897765 | G/G | 1.00 (.-.) | . | . | . | 639 | 53,74 | 288 | 51,71 | 1516 | 489 |  |
| TK1 ---- tag |  | G/A or A/A | 1.02 (0.86-1.23) | 0.79 | 0.99 | 0.89 | 550 | 46,26 | 269 | 48,29 | 1516 | 489 |  |
| TYMP ---- NA | rs131815 | G/G | 1.00 (.-.) | . | . | . | 631 | 53,07 | 294 | 52,78 | 1516 | 489 |  |
| TYMP ---- NA |  | G/A or A/A | 1.15 (0.96-1.37) | 0.13 | 0.83 | 0.20 | 558 | 46,93 | 263 | 47,22 | 1516 | 489 |  |
| TYMP ---- tag | rs131816 | A/A | 1.00 (.-.) | . | . | . | 705 | 59,29 | 350 | 62,84 | 1516 | 489 |  |
| TYMP ---- tag |  | A/G or G/G | 0.81 (0.67-0.97) | 0.02 | 0.59 | 0.14 | 484 | 40,71 | 207 | 37,16 | 1516 | 489 |  |
| TYMP ---- NA | rs131817 | C/C | 1.00 (.-.) | . | . | . | 380 | 31,96 | 186 | 33,39 | 1516 | 489 |  |
| TYMP ---- NA |  | C/T or T/T | 1.08 (0.89-1.31) | 0.42 | 0.93 | 0.42 | 809 | 68,04 | 371 | 66,61 | 1516 | 489 |  |
| TYMP ---- NA | rs140521 | T/T | 1.00 (.-.) | . | . | . | 604 | 50,80 | 294 | 52,78 | 1516 | 489 |  |
| TYMP ---- NA |  | T/G or G/G | 0.85 (0.71-1.02) | 0.09 | 0.74 | 0.20 | 585 | 49,20 | 263 | 47,22 | 1516 | 489 |  |
| TYMP ---- NA | rs140522 | G/G | 1.00 (.-.) | . | . | . | 533 | 44,83 | 259 | 46,50 | 1516 | 489 |  |
| TYMP ---- NA |  | G/A or A/A | 0.86 (0.72-1.03) | 0.11 | 0.82 | 0.20 | 656 | 55,17 | 298 | 53,50 | 1516 | 489 |  |
| TYMP ---- NA | rs140524 | G/G | 1.00 (.-.) | . | . | . | 809 | 68,04 | 360 | 64,63 | 1516 | 489 |  |
| TYMP ---- NA |  | G/A or A/A | 1.09 (0.91-1.32) | 0.36 | 0.93 | 0.42 | 380 | 31,96 | 197 | 35,37 | 1516 | 489 |  |
| TYMS ---- candidate literature | rs1001761 | C/C | 1.00 (.-.) | . | . | . | 344 | 28,93 | 182 | 32,68 | 1516 | 489 |  |
| TYMS ---- candidate literature |  | C/T or T/T | 0.82 (0.68-0.99) | 0.04 | 0.59 | 0.09 | 845 | 71,07 | 375 | 67,32 | 1516 | 489 |  |
| TYMS ---- candidate literature/tag | rs10502289 | T/T | 1.00 (.-.) | . | . | . | 739 | 62,15 | 362 | 64,99 | 1516 | 489 |  |
| TYMS ---- candidate literature/tag |  | T/A or A/A | 0.89 (0.74-1.08) | 0.23 | 0.85 | 0.30 | 450 | 37,85 | 195 | 35,01 | 1516 | 489 |  |
| TYMS ---- tag | rs15872 | C/C | 1.00 (.-.) | . | . | . | 545 | 45,84 | 273 | 49,01 | 1516 | 489 |  |
| TYMS ---- tag |  | C/T or T/T | 0.85 (0.71-1.01) | 0.07 | 0.66 | 0.13 | 644 | 54,16 | 284 | 50,99 | 1516 | 489 |  |
| TYMS ---- tag | rs2244500 | T/T | 1.00 (.-.) | . | . | . | 344 | 28,93 | 183 | 32,85 | 1516 | 489 |  |
| TYMS ---- tag |  | T/C or C/C | 0.81 (0.67-0.98) | 0.03 | 0.59 | 0.09 | 845 | 71,07 | 374 | 67,15 | 1516 | 489 |  |
| TYMS ---- tag | rs2741182 | G/G | 1.00 (.-.) | . | . | . | 722 | 60,72 | 340 | 61,04 | 1516 | 489 |  |
| TYMS ---- tag |  | G/C or C/C | 1.00 (0.83-1.20) | 0.99 | 1.00 | 0.99 | 467 | 39,28 | 217 | 38,96 | 1516 | 489 |  |
| TYMS ---- candidate literature | rs2847149 | G/G | 1.00 (.-.) | . | . | . | 344 | 28,93 | 182 | 32,68 | 1516 | 489 |  |
| TYMS ---- candidate literature |  | G/A or A/A | 0.82 (0.68-0.99) | 0.04 | 0.59 | 0.09 | 845 | 71,07 | 375 | 67,32 | 1516 | 489 |  |
| TYMS ---- candidate literature | rs2853533 | G/G | 1.00 (.-.) | . | . | . | 899 | 75,61 | 409 | 73,43 | 1516 | 489 |  |
| TYMS ---- candidate literature |  | G/C or C/C | 0.97 (0.79-1.19) | 0.75 | 0.99 | 0.85 | 290 | 24,39 | 148 | 26,57 | 1516 | 489 |  |
| TYMS ---- tag | rs495139 | C/C | 1.00 (.-.) | . | . | . | 416 | 34,99 | 162 | 29,08 | 1516 | 489 |  |
| TYMS ---- tag |  | C/G or G/G | 1.39 (1.14-1.69) | 0.00 | 0.45 | 0.01 | 773 | 65,01 | 395 | 70,92 | 1516 | 489 |  |
| TYMS ---- candidate literature | rs502396 | T/T | 1.00 (.-.) | . | . | . | 336 | 28,26 | 181 | 32,50 | 1516 | 489 |  |
| TYMS ---- candidate literature |  | T/C or C/C | 0.87 (0.72-1.06) | 0.16 | 0.84 | 0.24 | 853 | 71,74 | 376 | 67,50 | 1516 | 489 |  |
| UMPH2 ---- tag | rs2291028 | A/A | 1.00 (.-.) | . | . | . | 518 | 43,57 | 224 | 40,22 | 1516 | 489 |  |
| UMPH2 ---- tag |  | A/G or G/G | 1.10 (0.92-1.33) | 0.30 | 0.89 | 0.89 | 671 | 56,43 | 333 | 59,78 | 1516 | 489 |  |
| UMPH2 ---- NA | rs4789143 | A/A | 1.00 (.-.) | . | . | . | 915 | 76,96 | 440 | 78,99 | 1516 | 489 |  |
| UMPH2 ---- NA |  | A/G or G/G | 1.00 (0.80-1.24) | 0.97 | 0.99 | 0.97 | 274 | 23,04 | 117 | 21,01 | 1516 | 489 |  |
| UMPH2 ---- NA | rs750844 | G/G | 1.00 (.-.) | . | . | . | 602 | 50,63 | 273 | 49,01 | 1516 | 489 |  |
| UMPH2 ---- NA |  | G/A or A/A | 1.04 (0.87-1.24) | 0.68 | 0.99 | 0.97 | 587 | 49,37 | 284 | 50,99 | 1516 | 489 |  |
| UMPK ---- tag | rs11582877 | C/C | 1.00 (.-.) | . | . | . | 874 | 73,51 | 401 | 71,99 | 1516 | 489 |  |
| UMPK ---- tag |  | C/T or T/T | 1.05 (0.86-1.29) | 0.60 | 0.99 | 0.80 | 315 | 26,49 | 156 | 28,01 | 1516 | 489 |  |
| UMPK ---- tag | rs2622903 | A/A | 1.00 (.-.) | . | . | . | 582 | 48,95 | 276 | 49,55 | 1516 | 489 |  |
| UMPK ---- tag |  | A/G or G/G | 1.06 (0.89-1.27) | 0.50 | 0.96 | 0.80 | 607 | 51,05 | 281 | 50,45 | 1516 | 489 |  |
| UMPK ---- tag | rs2820989 | C/C | 1.00 (.-.) | . | . | . | 359 | 30,19 | 173 | 31,06 | 1516 | 489 |  |
| UMPK ---- tag |  | C/G or G/G | 1.05 (0.86-1.27) | 0.64 | 0.99 | 0.80 | 830 | 69,81 | 384 | 68,94 | 1516 | 489 |  |
| UMPK ---- tag | rs6660321 | A/A | 1.00 (.-.) | . | . | . | 902 | 75,86 | 417 | 74,87 | 1516 | 489 |  |
| UMPK ---- tag |  | A/C or C/C | 1.01 (0.82-1.24) | 0.92 | 0.99 | 0.92 | 287 | 24,14 | 140 | 25,13 | 1516 | 489 |  |
| UMPK ---- tag | rs6690084 | T/T | 1.00 (.-.) | . | . | . | 1028 | 86,46 | 474 | 85,10 | 1516 | 489 |  |
| UMPK ---- tag |  | T/C or C/C | 1.15 (0.89-1.47) | 0.28 | 0.88 | 0.80 | 161 | 13,54 | 83 | 14,90 | 1516 | 489 |  |
| UMPS ---- NA | rs1162 | A/A | 1.00 (.-.) | . | . | . | 555 | 46,68 | 257 | 46,14 | 1516 | 489 |  |
| UMPS ---- NA |  | A/G or G/G | 1.11 (0.93-1.32) | 0.26 | 0.87 | 0.79 | 634 | 53,32 | 300 | 53,86 | 1516 | 489 |  |
| UMPS ---- tag | rs13146 | C/C | 1.00 (.-.) | . | . | . | 822 | 69,13 | 385 | 69,12 | 1516 | 489 |  |
| UMPS ---- tag |  | C/T or T/T | 1.01 (0.83-1.22) | 0.95 | 0.99 | 0.95 | 367 | 30,87 | 172 | 30,88 | 1516 | 489 |  |
| UMPS ---- tag | rs16835902 | C/C | 1.00 (.-.) | . | . | . | 388 | 32,63 | 182 | 32,68 | 1516 | 489 |  |
| UMPS ---- tag |  | C/G or G/G | 0.98 (0.81-1.19) | 0.82 | 0.99 | 0.95 | 801 | 67,37 | 375 | 67,32 | 1516 | 489 |  |
| UMPS ---- tag | rs17282057 | T/T | 1.00 (.-.) | . | . | . | 907 | 76,28 | 421 | 75,58 | 1516 | 489 |  |
| UMPS ---- tag |  | T/C or C/C | 1.01 (0.82-1.25) | 0.91 | 0.99 | 0.95 | 282 | 23,72 | 136 | 24,42 | 1516 | 489 |  |
| UMPS ---- tag | rs606552 | A/A | 1.00 (.-.) | . | . | . | 601 | 50,55 | 306 | 54,94 | 1516 | 489 |  |
| UMPS ---- tag |  | A/G or G/G | 0.89 (0.75-1.07) | 0.22 | 0.85 | 0.79 | 588 | 49,45 | 251 | 45,06 | 1516 | 489 |  |
| UMPS ---- tag | rs694897 | C/C | 1.00 (.-.) | . | . | . | 510 | 42,89 | 218 | 39,14 | 1516 | 489 |  |
| UMPS ---- tag |  | C/G or G/G | 1.01 (0.84-1.21) | 0.92 | 0.99 | 0.95 | 679 | 57,11 | 339 | 60,86 | 1516 | 489 |  |
| UNG ---- NA | rs1059262 | T/T | 1.00 (.-.) | . | . | . | 801 | 67,37 | 392 | 70,38 | 1516 | 489 |  |
| UNG ---- NA |  | T/G or G/G | 0.91 (0.75-1.11) | 0.37 | 0.93 | 0.71 | 388 | 32,63 | 165 | 29,62 | 1516 | 489 |  |
| UNG ---- tag | rs2160603 | T/T | 1.00 (.-.) | . | . | . | 822 | 69,13 | 392 | 70,38 | 1516 | 489 |  |
| UNG ---- tag |  | T/C or C/C | 1.13 (0.92-1.38) | 0.23 | 0.85 | 0.70 | 367 | 30,87 | 165 | 29,62 | 1516 | 489 |  |
| UNG ---- tag | rs246079 | A/A | 1.00 (.-.) | . | . | . | 380 | 31,96 | 175 | 31,42 | 1516 | 489 |  |
| UNG ---- tag |  | A/G or G/G | 1.14 (0.94-1.39) | 0.19 | 0.85 | 0.70 | 809 | 68,04 | 382 | 68,58 | 1516 | 489 |  |
| UNG ---- NA | rs246085 | T/T | 1.00 (.-.) | . | . | . | 1052 | 88,48 | 493 | 88,51 | 1516 | 489 |  |
| UNG ---- NA |  | T/C or C/C | 1.01 (0.76-1.34) | 0.93 | 0.99 | 0.97 | 137 | 11,52 | 64 | 11,49 | 1516 | 489 |  |
| UNG ---- NA | rs2569987 | A/A | 1.00 (.-.) | . | . | . | 814 | 68,46 | 389 | 69,84 | 1516 | 489 |  |
| UNG ---- NA |  | A/G or G/G | 0.93 (0.76-1.13) | 0.47 | 0.94 | 0.71 | 375 | 31,54 | 168 | 30,16 | 1516 | 489 |  |
| UNG ---- tag | rs3219243 | T/T | 1.00 (.-.) | . | . | . | 766 | 64,42 | 349 | 62,66 | 1516 | 489 |  |
| UNG ---- tag |  | T/C or C/C | 1.00 (0.83-1.20) | 0.97 | 0.99 | 0.97 | 423 | 35,58 | 208 | 37,34 | 1516 | 489 |  |

|  | | | | | | | | **Ctrl** | | **Cases** | |  | | |
| --- | --- | --- | --- | --- | --- | --- | --- | --- | --- | --- | --- | --- | --- | --- |
| **Gene** | **SNP** | **Genotype** | **HR (95%-CI)** | **p** | **LR__pTrend_** | **FDR__pTrend_** | **FDR_(byGene)_pTrend_** | **N** | **%** | **N** | **%** | **NObsUsed** | **Events** | **LowCount** |
| AARS ---- tag | rs2070203 | T/T | 1.00 (.-.) | . | 0.65 | 0.96 | 0.65 | 313 | 26,32 | 163 | 29,26 | 1516 | 489 |  |
| AARS ---- tag |  | T/C | 0.95 (0.77-1.18) | 0.64 | . | . | . | 597 | 50,21 | 270 | 48,47 | 1516 | 489 |  |
| AARS ---- tag |  | C/C | 1.07 (0.83-1.38) | 0.59 | . | . | . | 279 | 23,47 | 124 | 22,26 | 1516 | 489 |  |
| AARS ---- tag | rs34087264 | G/G | 1.00 (.-.) | . | 0.40 | 0.96 | 0.65 | 356 | 29,94 | 167 | 29,98 | 1516 | 489 |  |
| AARS ---- tag |  | G/A | 0.78 (0.64-0.97) | 0.02 | . | . | . | 593 | 49,87 | 260 | 46,68 | 1516 | 489 |  |
| AARS ---- tag |  | A/A | 0.93 (0.72-1.19) | 0.54 | . | . | . | 240 | 20,19 | 130 | 23,34 | 1516 | 489 |  |
| ABCC4 ---- tag | rs10508023 | G/G | 1.00 (.-.) | . | 0.26 | 0.95 | 0.86 | 940 | 79,06 | 429 | 77,02 | 1516 | 489 | low_count |
| ABCC4 ---- tag |  | G/C | 1.07 (0.86-1.32) | 0.55 | . | . | . | 234 | 19,68 | 120 | 21,54 | 1516 | 489 |  |
| ABCC4 ---- tag |  | C/C | 1.73 (0.85-3.52) | 0.13 | . | . | . | 15 | 1,26 | 8 | 1,44 | 1516 | 489 |  |
| ABCC4 ---- tag | rs1059751 | T/T | 1.00 (.-.) | . | 0.47 | 0.96 | 0.95 | 340 | 28,60 | 158 | 28,37 | 1516 | 489 |  |
| ABCC4 ---- tag |  | T/C | 1.03 (0.84-1.27) | 0.77 | . | . | . | 582 | 48,95 | 276 | 49,55 | 1516 | 489 |  |
| ABCC4 ---- tag |  | C/C | 1.10 (0.85-1.42) | 0.46 | . | . | . | 267 | 22,46 | 123 | 22,08 | 1516 | 489 |  |
| ABCC4 ---- tag | rs11568643 | A/A | 1.00 (.-.) | . | 0.11 | 0.80 | 0.66 | 1000 | 84,10 | 455 | 81,69 | 1516 | 489 | low_count |
| ABCC4 ---- tag |  | A/G | 1.27 (1.01-1.60) | 0.04 | . | . | . | 183 | 15,39 | 98 | 17,59 | 1516 | 489 |  |
| ABCC4 ---- tag |  | G/G | 0.59 (0.14-2.39) | 0.46 | . | . | . | 6 | 0,50 | 4 | 0,72 | 1516 | 489 |  |
| ABCC4 ---- NA | rs11568658 | G/G | 1.00 (.-.) | . | 0.46 | 0.96 | 0.95 | 1127 | 94,79 | 527 | 94,61 | 1516 | 489 | low_count |
| ABCC4 ---- NA |  | G/T | 1.10 (0.72-1.67) | 0.67 | . | . | . | 61 | 5,13 | 29 | 5,21 | 1516 | 489 |  |
| ABCC4 ---- NA |  | T/T | 4.21 (0.58-30.68) | 0.16 | . | . | . | 1 | 0,08 | 1 | 0,18 | 1516 | 489 |  |
| ABCC4 ---- tag | rs12864049 | T/T | 1.00 (.-.) | . | 0.89 | 0.98 | 0.96 | 890 | 74,85 | 416 | 74,69 | 1516 | 489 | low_count |
| ABCC4 ---- tag |  | T/C | 1.00 (0.81-1.23) | 0.97 | . | . | . | 275 | 23,13 | 131 | 23,52 | 1516 | 489 |  |
| ABCC4 ---- tag |  | C/C | 1.13 (0.58-2.20) | 0.73 | . | . | . | 24 | 2,02 | 10 | 1,80 | 1516 | 489 |  |
| ABCC4 ---- tag | rs1628382 | G/G | 1.00 (.-.) | . | 0.76 | 0.97 | 0.96 | 741 | 62,32 | 347 | 62,30 | 1516 | 489 |  |
| ABCC4 ---- tag |  | G/A | 0.99 (0.82-1.19) | 0.89 | . | . | . | 400 | 33,64 | 190 | 34,11 | 1516 | 489 |  |
| ABCC4 ---- tag |  | A/A | 1.22 (0.75-1.96) | 0.42 | . | . | . | 48 | 4,04 | 20 | 3,59 | 1516 | 489 |  |
| ABCC4 ---- tag | rs1678354 | C/C | 1.00 (.-.) | . | 0.65 | 0.96 | 0.96 | 500 | 42,05 | 237 | 42,55 | 1516 | 489 |  |
| ABCC4 ---- tag |  | C/G | 0.99 (0.81-1.19) | 0.88 | . | . | . | 541 | 45,50 | 255 | 45,78 | 1516 | 489 |  |
| ABCC4 ---- tag |  | G/G | 1.11 (0.83-1.50) | 0.48 | . | . | . | 148 | 12,45 | 65 | 11,67 | 1516 | 489 |  |
| ABCC4 ---- tag | rs1678383 | T/T | 1.00 (.-.) | . | 0.46 | 0.96 | 0.95 | 964 | 81,08 | 450 | 80,79 | 1516 | 489 | low_count |
| ABCC4 ---- tag |  | T/G | 0.86 (0.68-1.09) | 0.23 | . | . | . | 214 | 18,00 | 100 | 17,95 | 1516 | 489 |  |
| ABCC4 ---- tag |  | G/G | 1.45 (0.64-3.28) | 0.37 | . | . | . | 11 | 0,93 | 7 | 1,26 | 1516 | 489 |  |
| ABCC4 ---- tag | rs1678395 | G/G | 1.00 (.-.) | . | 0.26 | 0.95 | 0.86 | 1031 | 86,71 | 476 | 85,46 | 1516 | 489 | low_count |
| ABCC4 ---- tag |  | G/A | 0.87 (0.67-1.13) | 0.31 | . | . | . | 153 | 12,87 | 79 | 14,18 | 1516 | 489 |  |
| ABCC4 ---- tag |  | A/A | 0.71 (0.17-2.90) | 0.64 | . | . | . | 5 | 0,42 | 2 | 0,36 | 1516 | 489 |  |
| ABCC4 ---- tag | rs1678405 | T/T | 1.00 (.-.) | . | 0.05 | 0.77 | 0.65 | 562 | 47,27 | 255 | 45,78 | 1516 | 489 |  |
| ABCC4 ---- tag |  | T/C | 1.05 (0.87-1.27) | 0.62 | . | . | . | 524 | 44,07 | 246 | 44,17 | 1516 | 489 |  |
| ABCC4 ---- tag |  | C/C | 1.46 (1.08-1.98) | 0.01 | . | . | . | 103 | 8,66 | 56 | 10,05 | 1516 | 489 |  |
| ABCC4 ---- tag | rs17189540 | A/A | 1.00 (.-.) | . | 0.16 | 0.91 | 0.85 | 1042 | 87,64 | 477 | 85,64 | 1516 | 489 | low_count |
| ABCC4 ---- tag |  | A/G | 1.16 (0.90-1.50) | 0.25 | . | . | . | 143 | 12,03 | 77 | 13,82 | 1516 | 489 |  |
| ABCC4 ---- tag |  | G/G | 2.30 (0.57-9.34) | 0.25 | . | . | . | 4 | 0,34 | 3 | 0,54 | 1516 | 489 |  |
| ABCC4 ---- tag | rs17235152 | T/T | 1.00 (.-.) | . | 0.97 | 0.99 | 0.99 | 864 | 72,67 | 409 | 73,43 | 1516 | 489 | low_count |
| ABCC4 ---- tag |  | T/C | 1.04 (0.84-1.28) | 0.73 | . | . | . | 294 | 24,73 | 138 | 24,78 | 1516 | 489 |  |
| ABCC4 ---- tag |  | C/C | 0.81 (0.42-1.59) | 0.55 | . | . | . | 31 | 2,61 | 10 | 1,80 | 1516 | 489 |  |
| ABCC4 ---- tag | rs17268122 | G/G | 1.00 (.-.) | . | 0.63 | 0.96 | 0.96 | 721 | 60,64 | 347 | 62,30 | 1516 | 489 |  |
| ABCC4 ---- tag |  | G/T | 1.05 (0.86-1.27) | 0.65 | . | . | . | 413 | 34,74 | 182 | 32,68 | 1516 | 489 |  |
| ABCC4 ---- tag |  | T/T | 0.78 (0.52-1.19) | 0.26 | . | . | . | 55 | 4,63 | 28 | 5,03 | 1516 | 489 |  |
| ABCC4 ---- tag | rs17268170 | C/C | 1.00 (.-.) | . | 0.81 | 0.98 | 0.96 | 980 | 82,42 | 450 | 80,79 | 1516 | 489 | low_count |
| ABCC4 ---- tag |  | C/T | 1.00 (0.79-1.26) | 0.97 | . | . | . | 202 | 16,99 | 99 | 17,77 | 1516 | 489 |  |
| ABCC4 ---- tag |  | T/T | 1.28 (0.59-2.78) | 0.53 | . | . | . | 7 | 0,59 | 8 | 1,44 | 1516 | 489 |  |
| ABCC4 ---- tag | rs1729764 | A/A | 1.00 (.-.) | . | 0.44 | 0.96 | 0.95 | 937 | 78,81 | 453 | 81,33 | 1516 | 489 | low_count |
| ABCC4 ---- tag |  | A/G | 0.82 (0.64-1.04) | 0.11 | . | . | . | 243 | 20,44 | 93 | 16,70 | 1516 | 489 |  |
| ABCC4 ---- tag |  | G/G | 1.66 (0.84-3.28) | 0.15 | . | . | . | 9 | 0,76 | 11 | 1,97 | 1516 | 489 |  |
| ABCC4 ---- tag | rs1729767 | T/T | 1.00 (.-.) | . | 0.51 | 0.96 | 0.96 | 641 | 53,91 | 285 | 51,17 | 1516 | 489 |  |
| ABCC4 ---- tag |  | T/C | 1.06 (0.88-1.28) | 0.52 | . | . | . | 464 | 39,02 | 237 | 42,55 | 1516 | 489 |  |
| ABCC4 ---- tag |  | C/C | 1.07 (0.75-1.54) | 0.70 | . | . | . | 84 | 7,06 | 35 | 6,28 | 1516 | 489 |  |
| ABCC4 ---- tag | rs17300935 | C/C | 1.00 (.-.) | . | 0.85 | 0.98 | 0.96 | 876 | 73,68 | 407 | 73,07 | 1516 | 489 | low_count |
| ABCC4 ---- tag |  | C/G | 1.09 (0.88-1.34) | 0.44 | . | . | . | 283 | 23,80 | 142 | 25,49 | 1516 | 489 |  |
| ABCC4 ---- tag |  | G/G | 0.70 (0.33-1.50) | 0.37 | . | . | . | 30 | 2,52 | 8 | 1,44 | 1516 | 489 |  |
| ABCC4 ---- tag | rs1750190 | G/G | 1.00 (.-.) | . | 0.92 | 0.98 | 0.96 | 321 | 27,00 | 132 | 23,70 | 1516 | 489 |  |
| ABCC4 ---- tag |  | G/A | 1.25 (1.00-1.56) | 0.05 | . | . | . | 573 | 48,19 | 297 | 53,32 | 1516 | 489 |  |
| ABCC4 ---- tag |  | A/A | 1.01 (0.78-1.32) | 0.92 | . | . | . | 295 | 24,81 | 128 | 22,98 | 1516 | 489 |  |
| ABCC4 ---- tag | rs1750996 | A/A | 1.00 (.-.) | . | 0.71 | 0.97 | 0.96 | 798 | 67,12 | 378 | 67,86 | 1516 | 489 |  |
| ABCC4 ---- tag |  | A/G | 0.86 (0.71-1.06) | 0.16 | . | . | . | 360 | 30,28 | 158 | 28,37 | 1516 | 489 |  |
| ABCC4 ---- tag |  | G/G | 1.40 (0.87-2.26) | 0.16 | . | . | . | 31 | 2,61 | 21 | 3,77 | 1516 | 489 |  |
| ABCC4 ---- tag | rs1751025 | C/C | 1.00 (.-.) | . | 0.01 | 0.73 | 0.21 | 571 | 48,02 | 255 | 45,78 | 1516 | 489 |  |
| ABCC4 ---- tag |  | C/G | 1.11 (0.91-1.34) | 0.31 | . | . | . | 510 | 42,89 | 236 | 42,37 | 1516 | 489 |  |
| ABCC4 ---- tag |  | G/G | 1.53 (1.15-2.03) | 0.00 | . | . | . | 108 | 9,08 | 66 | 11,85 | 1516 | 489 |  |
| ABCC4 ---- tag | rs1751051 | T/T | 1.00 (.-.) | . | 0.85 | 0.98 | 0.96 | 509 | 42,81 | 232 | 41,65 | 1516 | 489 |  |
| ABCC4 ---- tag |  | T/A | 0.93 (0.76-1.13) | 0.45 | . | . | . | 534 | 44,91 | 258 | 46,32 | 1516 | 489 |  |
| ABCC4 ---- tag |  | A/A | 1.11 (0.83-1.49) | 0.48 | . | . | . | 146 | 12,28 | 67 | 12,03 | 1516 | 489 |  |
| ABCC4 ---- tag | rs1764416 | G/G | 1.00 (.-.) | . | 0.30 | 0.95 | 0.86 | 1016 | 85,45 | 486 | 87,25 | 1516 | 489 | low_count |
| ABCC4 ---- tag |  | G/A | 0.87 (0.66-1.15) | 0.34 | . | . | . | 166 | 13,96 | 71 | 12,75 | 1516 | 489 |  |
| ABCC4 ---- tag |  | A/A | 0.00 (0.00-3E167) | 0.96 | . | . | . | 7 | 0,59 | 0 | 0,00 | 1516 | 489 |  |
| ABCC4 ---- tag | rs2274401 | T/T | 1.00 (.-.) | . | 0.21 | 0.92 | 0.86 | 730 | 61,40 | 358 | 64,27 | 1516 | 489 |  |
| ABCC4 ---- tag |  | T/C | 0.91 (0.75-1.11) | 0.36 | . | . | . | 400 | 33,64 | 178 | 31,96 | 1516 | 489 |  |
| ABCC4 ---- tag |  | C/C | 0.79 (0.49-1.27) | 0.32 | . | . | . | 59 | 4,96 | 21 | 3,77 | 1516 | 489 |  |
| ABCC4 ---- tag | rs2892716 | C/C | 1.00 (.-.) | . | 0.90 | 0.98 | 0.96 | 443 | 37,26 | 235 | 42,19 | 1516 | 489 |  |
| ABCC4 ---- tag |  | C/T | 1.07 (0.88-1.29) | 0.52 | . | . | . | 580 | 48,78 | 263 | 47,22 | 1516 | 489 |  |
| ABCC4 ---- tag |  | T/T | 0.92 (0.68-1.25) | 0.60 | . | . | . | 166 | 13,96 | 59 | 10,59 | 1516 | 489 |  |
| ABCC4 ---- tag | rs3782964 | C/C | 1.00 (.-.) | . | 0.33 | 0.96 | 0.86 | 782 | 65,77 | 397 | 71,27 | 1516 | 489 |  |
| ABCC4 ---- tag |  | C/T | 0.84 (0.69-1.04) | 0.11 | . | . | . | 381 | 32,04 | 142 | 25,49 | 1516 | 489 |  |
| ABCC4 ---- tag |  | T/T | 1.26 (0.71-2.26) | 0.43 | . | . | . | 26 | 2,19 | 18 | 3,23 | 1516 | 489 |  |
| ABCC4 ---- tag | rs3818494 | C/C | 1.00 (.-.) | . | 0.02 | 0.73 | 0.33 | 535 | 45,00 | 244 | 43,81 | 1516 | 489 |  |
| ABCC4 ---- tag |  | C/G | 1.21 (1.00-1.47) | 0.05 | . | . | . | 534 | 44,91 | 247 | 44,34 | 1516 | 489 |  |
| ABCC4 ---- tag |  | G/G | 1.33 (1.00-1.78) | 0.05 | . | . | . | 120 | 10,09 | 66 | 11,85 | 1516 | 489 |  |
| ABCC4 ---- tag | rs3864997 | G/G | 1.00 (.-.) | . | 0.48 | 0.96 | 0.95 | 303 | 25,48 | 147 | 26,39 | 1516 | 489 |  |
| ABCC4 ---- tag |  | G/T | 1.17 (0.94-1.45) | 0.16 | . | . | . | 617 | 51,89 | 292 | 52,42 | 1516 | 489 |  |
| ABCC4 ---- tag |  | T/T | 1.08 (0.83-1.41) | 0.55 | . | . | . | 269 | 22,62 | 118 | 21,18 | 1516 | 489 |  |
| ABCC4 ---- tag | rs4148421 | G/G | 1.00 (.-.) | . | 0.57 | 0.96 | 0.96 | 352 | 29,60 | 157 | 28,19 | 1516 | 489 |  |
| ABCC4 ---- tag |  | G/A | 1.02 (0.83-1.26) | 0.86 | . | . | . | 598 | 50,29 | 292 | 52,42 | 1516 | 489 |  |
| ABCC4 ---- tag |  | A/A | 0.92 (0.70-1.20) | 0.52 | . | . | . | 239 | 20,10 | 108 | 19,39 | 1516 | 489 |  |
| ABCC4 ---- tag | rs4148446 | G/G | 1.00 (.-.) | . | 0.92 | 0.98 | 0.96 | 388 | 32,63 | 206 | 36,98 | 1516 | 489 |  |
| ABCC4 ---- tag |  | G/A | 1.04 (0.85-1.27) | 0.69 | . | . | . | 590 | 49,62 | 269 | 48,29 | 1516 | 489 |  |
| ABCC4 ---- tag |  | A/A | 0.96 (0.73-1.27) | 0.78 | . | . | . | 211 | 17,75 | 82 | 14,72 | 1516 | 489 |  |
| ABCC4 ---- tag | rs4148455 | G/G | 1.00 (.-.) | . | 0.87 | 0.98 | 0.96 | 904 | 76,03 | 421 | 75,58 | 1516 | 489 | low_count |
| ABCC4 ---- tag |  | G/A | 1.10 (0.89-1.36) | 0.38 | . | . | . | 261 | 21,95 | 128 | 22,98 | 1516 | 489 |  |
| ABCC4 ---- tag |  | A/A | 0.67 (0.32-1.43) | 0.30 | . | . | . | 24 | 2,02 | 8 | 1,44 | 1516 | 489 |  |
| ABCC4 ---- tag | rs4148540 | C/C | 1.00 (.-.) | . | 0.67 | 0.96 | 0.96 | 1066 | 89,66 | 482 | 86,54 | 1516 | 489 | low_count |
| ABCC4 ---- tag |  | C/T | 1.08 (0.82-1.41) | 0.59 | . | . | . | 118 | 9,92 | 72 | 12,93 | 1516 | 489 |  |
| ABCC4 ---- tag |  | T/T | 0.92 (0.29-2.92) | 0.89 | . | . | . | 5 | 0,42 | 3 | 0,54 | 1516 | 489 |  |
| ABCC4 ---- tag | rs4148542 | G/G | 1.00 (.-.) | . | 1.00 | 1.00 | 1.00 | 333 | 28,01 | 146 | 26,21 | 1516 | 489 |  |
| ABCC4 ---- tag |  | G/A | 0.97 (0.78-1.21) | 0.79 | . | . | . | 570 | 47,94 | 279 | 50,09 | 1516 | 489 |  |
| ABCC4 ---- tag |  | A/A | 1.00 (0.78-1.29) | 0.99 | . | . | . | 286 | 24,05 | 132 | 23,70 | 1516 | 489 |  |
| ABCC4 ---- tag | rs4148544 | G/G | 1.00 (.-.) | . | 0.91 | 0.98 | 0.96 | 510 | 42,89 | 239 | 42,91 | 1516 | 489 |  |
| ABCC4 ---- tag |  | G/A | 0.96 (0.80-1.17) | 0.71 | . | . | . | 542 | 45,58 | 243 | 43,63 | 1516 | 489 |  |
| ABCC4 ---- tag |  | A/A | 1.05 (0.79-1.40) | 0.73 | . | . | . | 137 | 11,52 | 75 | 13,46 | 1516 | 489 |  |
| ABCC4 ---- tag | rs4283094 | C/C | 1.00 (.-.) | . | 0.88 | 0.98 | 0.96 | 309 | 25,99 | 126 | 22,62 | 1516 | 489 |  |
| ABCC4 ---- tag |  | C/G | 1.05 (0.84-1.32) | 0.65 | . | . | . | 608 | 51,14 | 287 | 51,53 | 1516 | 489 |  |
| ABCC4 ---- tag |  | G/G | 1.02 (0.79-1.32) | 0.87 | . | . | . | 272 | 22,88 | 144 | 25,85 | 1516 | 489 |  |
| ABCC4 ---- tag | rs4636781 | A/A | 1.00 (.-.) | . | 0.11 | 0.80 | 0.66 | 855 | 71,91 | 389 | 69,84 | 1516 | 489 |  |
| ABCC4 ---- tag |  | A/G | 1.15 (0.94-1.41) | 0.17 | . | . | . | 307 | 25,82 | 153 | 27,47 | 1516 | 489 |  |
| ABCC4 ---- tag |  | G/G | 1.30 (0.76-2.24) | 0.34 | . | . | . | 27 | 2,27 | 15 | 2,69 | 1516 | 489 |  |
| ABCC4 ---- tag | rs4771910 | T/T | 1.00 (.-.) | . | 0.09 | 0.79 | 0.66 | 576 | 48,44 | 281 | 50,45 | 1516 | 489 |  |
| ABCC4 ---- tag |  | T/C | 0.88 (0.73-1.07) | 0.20 | . | . | . | 502 | 42,22 | 231 | 41,47 | 1516 | 489 |  |
| ABCC4 ---- tag |  | C/C | 0.78 (0.55-1.10) | 0.16 | . | . | . | 111 | 9,34 | 45 | 8,08 | 1516 | 489 |  |
| ABCC4 ---- tag | rs4773850 | T/T | 1.00 (.-.) | . | 0.01 | 0.73 | 0.21 | 531 | 44,66 | 289 | 51,89 | 1516 | 489 |  |
| ABCC4 ---- tag |  | T/G | 0.86 (0.71-1.04) | 0.12 | . | . | . | 537 | 45,16 | 219 | 39,32 | 1516 | 489 |  |
| ABCC4 ---- tag |  | G/G | 0.64 (0.46-0.90) | 0.01 | . | . | . | 121 | 10,18 | 49 | 8,80 | 1516 | 489 |  |
| ABCC4 ---- tag | rs7981095 | A/A | 1.00 (.-.) | . | 0.79 | 0.98 | 0.96 | 772 | 64,93 | 353 | 63,38 | 1516 | 489 |  |
| ABCC4 ---- tag |  | A/T | 0.93 (0.76-1.13) | 0.45 | . | . | . | 379 | 31,88 | 178 | 31,96 | 1516 | 489 |  |
| ABCC4 ---- tag |  | T/T | 1.15 (0.72-1.87) | 0.56 | . | . | . | 38 | 3,20 | 26 | 4,67 | 1516 | 489 |  |
| ABCC4 ---- tag | rs8001444 | C/C | 1.00 (.-.) | . | 0.57 | 0.96 | 0.96 | 410 | 34,48 | 187 | 33,57 | 1516 | 489 |  |
| ABCC4 ---- tag |  | C/T | 0.91 (0.75-1.12) | 0.38 | . | . | . | 561 | 47,18 | 275 | 49,37 | 1516 | 489 |  |
| ABCC4 ---- tag |  | T/T | 0.95 (0.73-1.23) | 0.68 | . | . | . | 218 | 18,33 | 95 | 17,06 | 1516 | 489 |  |
| ABCC4 ---- tag | rs931111 | T/T | 1.00 (.-.) | . | 0.72 | 0.97 | 0.96 | 799 | 67,20 | 373 | 66,97 | 1516 | 489 |  |
| ABCC4 ---- tag |  | T/C | 1.05 (0.86-1.29) | 0.61 | . | . | . | 349 | 29,35 | 159 | 28,55 | 1516 | 489 |  |
| ABCC4 ---- tag |  | C/C | 0.76 (0.48-1.23) | 0.26 | . | . | . | 41 | 3,45 | 25 | 4,49 | 1516 | 489 |  |
| ABCC4 ---- tag | rs943288 | T/T | 1.00 (.-.) | . | 0.61 | 0.96 | 0.96 | 910 | 76,53 | 424 | 76,12 | 1516 | 489 |  |
| ABCC4 ---- tag |  | T/A | 1.02 (0.82-1.28) | 0.83 | . | . | . | 259 | 21,78 | 120 | 21,54 | 1516 | 489 |  |
| ABCC4 ---- tag |  | A/A | 1.21 (0.68-2.16) | 0.52 | . | . | . | 20 | 1,68 | 13 | 2,33 | 1516 | 489 |  |
| ABCC4 ---- tag | rs943290 | A/A | 1.00 (.-.) | . | 0.25 | 0.95 | 0.86 | 643 | 54,08 | 305 | 54,76 | 1516 | 489 |  |
| ABCC4 ---- tag |  | A/G | 0.86 (0.71-1.04) | 0.12 | . | . | . | 455 | 38,27 | 214 | 38,42 | 1516 | 489 |  |
| ABCC4 ---- tag |  | G/G | 0.96 (0.66-1.38) | 0.81 | . | . | . | 91 | 7,65 | 38 | 6,82 | 1516 | 489 |  |
| ABCC4 ---- tag | rs9516530 | C/C | 1.00 (.-.) | . | 0.30 | 0.95 | 0.86 | 648 | 54,50 | 310 | 55,66 | 1516 | 489 |  |
| ABCC4 ---- tag |  | C/T | 0.91 (0.75-1.10) | 0.32 | . | . | . | 446 | 37,51 | 207 | 37,16 | 1516 | 489 |  |
| ABCC4 ---- tag |  | T/T | 0.89 (0.62-1.28) | 0.54 | . | . | . | 95 | 7,99 | 40 | 7,18 | 1516 | 489 |  |
| ABCC4 ---- tag | rs9516551 | C/C | 1.00 (.-.) | . | 0.07 | 0.79 | 0.66 | 947 | 79,65 | 440 | 78,99 | 1516 | 489 | low_count |
| ABCC4 ---- tag |  | C/A | 0.81 (0.65-1.01) | 0.06 | . | . | . | 231 | 19,43 | 113 | 20,29 | 1516 | 489 |  |
| ABCC4 ---- tag |  | A/A | 0.85 (0.31-2.32) | 0.76 | . | . | . | 11 | 0,93 | 4 | 0,72 | 1516 | 489 |  |
| ABCC4 ---- tag | rs9524822 | T/T | 1.00 (.-.) | . | 0.35 | 0.96 | 0.86 | 764 | 64,26 | 367 | 65,89 | 1516 | 489 |  |
| ABCC4 ---- tag |  | T/C | 0.87 (0.71-1.06) | 0.16 | . | . | . | 388 | 32,63 | 165 | 29,62 | 1516 | 489 |  |
| ABCC4 ---- tag |  | C/C | 1.07 (0.65-1.75) | 0.79 | . | . | . | 37 | 3,11 | 25 | 4,49 | 1516 | 489 |  |
| ABCC4 ---- tag | rs9524861 | G/G | 1.00 (.-.) | . | 0.34 | 0.96 | 0.86 | 619 | 52,06 | 270 | 48,47 | 1516 | 489 |  |
| ABCC4 ---- tag |  | G/C | 1.03 (0.85-1.24) | 0.79 | . | . | . | 474 | 39,87 | 239 | 42,91 | 1516 | 489 |  |
| ABCC4 ---- tag |  | C/C | 0.78 (0.56-1.08) | 0.14 | . | . | . | 96 | 8,07 | 48 | 8,62 | 1516 | 489 |  |
| ABCC4 ---- tag | rs9524902 | T/T | 1.00 (.-.) | . | 0.30 | 0.95 | 0.86 | 324 | 27,25 | 163 | 29,26 | 1516 | 489 |  |
| ABCC4 ---- tag |  | T/C | 0.80 (0.64-0.98) | 0.04 | . | . | . | 592 | 49,79 | 270 | 48,47 | 1516 | 489 |  |
| ABCC4 ---- tag |  | C/C | 0.89 (0.69-1.14) | 0.37 | . | . | . | 273 | 22,96 | 124 | 22,26 | 1516 | 489 |  |
| ABCC4 ---- tag | rs9556455 | G/G | 1.00 (.-.) | . | 0.30 | 0.95 | 0.86 | 905 | 76,11 | 413 | 74,15 | 1516 | 489 | low_count |
| ABCC4 ---- tag |  | G/A | 1.19 (0.97-1.46) | 0.10 | . | . | . | 269 | 22,62 | 134 | 24,06 | 1516 | 489 |  |
| ABCC4 ---- tag |  | A/A | 0.81 (0.40-1.65) | 0.56 | . | . | . | 15 | 1,26 | 10 | 1,80 | 1516 | 489 |  |
| ABCC4 ---- NA | rs9561778 | G/G | 1.00 (.-.) | . | 0.65 | 0.96 | 0.96 | 772 | 64,93 | 370 | 66,43 | 1516 | 489 |  |
| ABCC4 ---- NA |  | G/T | 1.06 (0.87-1.29) | 0.58 | . | . | . | 365 | 30,70 | 171 | 30,70 | 1516 | 489 |  |
| ABCC4 ---- NA |  | T/T | 0.67 (0.38-1.17) | 0.16 | . | . | . | 52 | 4,37 | 16 | 2,87 | 1516 | 489 |  |
| ABCC4 ---- tag | rs9561811 | C/C | 1.00 (.-.) | . | 0.10 | 0.80 | 0.66 | 787 | 66,19 | 379 | 68,04 | 1516 | 489 |  |
| ABCC4 ---- tag |  | C/T | 1.05 (0.86-1.29) | 0.63 | . | . | . | 370 | 31,12 | 155 | 27,83 | 1516 | 489 |  |
| ABCC4 ---- tag |  | T/T | 1.68 (1.09-2.60) | 0.02 | . | . | . | 32 | 2,69 | 23 | 4,13 | 1516 | 489 |  |
| ABCC4 ---- tag | rs9590183 | T/T | 1.00 (.-.) | . | 0.49 | 0.96 | 0.95 | 1038 | 87,30 | 494 | 88,69 | 1516 | 489 | low_count |
| ABCC4 ---- tag |  | T/A | 0.89 (0.67-1.19) | 0.42 | . | . | . | 144 | 12,11 | 60 | 10,77 | 1516 | 489 |  |
| ABCC4 ---- tag |  | A/A | 1.14 (0.28-4.62) | 0.86 | . | . | . | 7 | 0,59 | 3 | 0,54 | 1516 | 489 |  |
| ABCC4 ---- tag | rs997777 | T/T | 1.00 (.-.) | . | 0.74 | 0.97 | 0.96 | 600 | 50,46 | 271 | 48,65 | 1516 | 489 |  |
| ABCC4 ---- tag |  | T/A | 0.99 (0.82-1.20) | 0.90 | . | . | . | 494 | 41,55 | 237 | 42,55 | 1516 | 489 |  |
| ABCC4 ---- tag |  | A/A | 1.10 (0.80-1.51) | 0.57 | . | . | . | 95 | 7,99 | 49 | 8,80 | 1516 | 489 |  |
| ADH1B ---- tag | rs1159918 | G/G | 1.00 (.-.) | . | 0.62 | 0.96 | 0.73 | 527 | 44,32 | 255 | 45,78 | 1516 | 489 |  |
| ADH1B ---- tag |  | G/T | 0.90 (0.74-1.09) | 0.29 | . | . | . | 523 | 43,99 | 248 | 44,52 | 1516 | 489 |  |
| ADH1B ---- tag |  | T/T | 1.02 (0.74-1.40) | 0.92 | . | . | . | 139 | 11,69 | 54 | 9,69 | 1516 | 489 |  |
| ADH1B ---- candidate literature | rs1229984 | G/G | 1.00 (.-.) | . | 0.39 | 0.96 | 0.67 | 1076 | 90,50 | 505 | 90,66 | 1516 | 489 | low_count |
| ADH1B ---- candidate literature |  | G/A | 0.94 (0.69-1.27) | 0.67 | . | . | . | 108 | 9,08 | 51 | 9,16 | 1516 | 489 |  |
| ADH1B ---- candidate literature |  | A/A | 0.35 (0.05-2.52) | 0.30 | . | . | . | 5 | 0,42 | 1 | 0,18 | 1516 | 489 |  |
| ADH1B ---- tag | rs12507573 | C/C | 1.00 (.-.) | . | 0.73 | 0.97 | 0.73 | 381 | 32,04 | 159 | 28,55 | 1516 | 489 |  |
| ADH1B ---- tag |  | C/A | 1.01 (0.82-1.24) | 0.94 | . | . | . | 565 | 47,52 | 280 | 50,27 | 1516 | 489 |  |
| ADH1B ---- tag |  | A/A | 1.05 (0.81-1.36) | 0.71 | . | . | . | 243 | 20,44 | 118 | 21,18 | 1516 | 489 |  |
| ADH1B ---- tag | rs1693457 | T/T | 1.00 (.-.) | . | 0.06 | 0.77 | 0.29 | 820 | 68,97 | 401 | 71,99 | 1516 | 489 |  |
| ADH1B ---- tag |  | T/C | 0.85 (0.69-1.04) | 0.12 | . | . | . | 323 | 27,17 | 144 | 25,85 | 1516 | 489 |  |
| ADH1B ---- tag |  | C/C | 0.68 (0.37-1.26) | 0.22 | . | . | . | 46 | 3,87 | 12 | 2,15 | 1516 | 489 |  |
| ADH1B ---- tag | rs2066701 | C/C | 1.00 (.-.) | . | 0.40 | 0.96 | 0.67 | 542 | 45,58 | 243 | 43,63 | 1516 | 489 |  |
| ADH1B ---- tag |  | C/T | 1.06 (0.88-1.28) | 0.53 | . | . | . | 523 | 43,99 | 255 | 45,78 | 1516 | 489 |  |
| ADH1B ---- tag |  | T/T | 1.12 (0.83-1.52) | 0.46 | . | . | . | 124 | 10,43 | 59 | 10,59 | 1516 | 489 |  |
| ADH1C ---- tag | rs11936869 | C/C | 1.00 (.-.) | . | 0.29 | 0.95 | 0.88 | 606 | 50,97 | 301 | 54,04 | 1516 | 489 |  |
| ADH1C ---- tag |  | C/G | 0.88 (0.72-1.06) | 0.18 | . | . | . | 479 | 40,29 | 208 | 37,34 | 1516 | 489 |  |
| ADH1C ---- tag |  | G/G | 0.93 (0.67-1.31) | 0.70 | . | . | . | 104 | 8,75 | 48 | 8,62 | 1516 | 489 |  |
| ADH1C ---- tag | rs1229849 | T/T | 1.00 (.-.) | . | 0.45 | 0.96 | 0.90 | 637 | 53,57 | 285 | 51,17 | 1516 | 489 |  |
| ADH1C ---- tag |  | T/A | 0.98 (0.81-1.18) | 0.81 | . | . | . | 479 | 40,29 | 234 | 42,01 | 1516 | 489 |  |
| ADH1C ---- tag |  | A/A | 1.33 (0.92-1.92) | 0.12 | . | . | . | 73 | 6,14 | 38 | 6,82 | 1516 | 489 |  |
| ADH1C ---- tag | rs1229863 | A/A | 1.00 (.-.) | . | 0.73 | 0.97 | 0.90 | 873 | 73,42 | 406 | 72,89 | 1516 | 489 |  |
| ADH1C ---- tag |  | A/T | 0.92 (0.74-1.14) | 0.42 | . | . | . | 286 | 24,05 | 134 | 24,06 | 1516 | 489 |  |
| ADH1C ---- tag |  | T/T | 1.13 (0.68-1.88) | 0.63 | . | . | . | 30 | 2,52 | 17 | 3,05 | 1516 | 489 |  |
| ADH1C ---- tag | rs1229980 | C/C | 1.00 (.-.) | . | 0.29 | 0.95 | 0.88 | 1073 | 90,24 | 490 | 87,97 | 1516 | 489 | low_count |
| ADH1C ---- tag |  | C/G | 1.12 (0.84-1.48) | 0.43 | . | . | . | 113 | 9,50 | 65 | 11,67 | 1516 | 489 |  |
| ADH1C ---- tag |  | G/G | 2.26 (0.56-9.15) | 0.25 | . | . | . | 3 | 0,25 | 2 | 0,36 | 1516 | 489 |  |
| ADH1C ---- candidate | rs1693482 | C/C | 1.00 (.-.) | . | 0.90 | 0.98 | 0.90 | 491 | 41,30 | 229 | 41,11 | 1516 | 489 |  |
| ADH1C ---- candidate |  | C/T | 0.89 (0.73-1.08) | 0.25 | . | . | . | 545 | 45,84 | 246 | 44,17 | 1516 | 489 |  |
| ADH1C ---- candidate |  | T/T | 1.06 (0.80-1.40) | 0.68 | . | . | . | 153 | 12,87 | 82 | 14,72 | 1516 | 489 |  |
| ADH1C ---- tag | rs2173201 | C/C | 1.00 (.-.) | . | 0.58 | 0.96 | 0.90 | 676 | 56,85 | 329 | 59,07 | 1516 | 489 |  |
| ADH1C ---- tag |  | C/A | 0.91 (0.75-1.10) | 0.31 | . | . | . | 436 | 36,67 | 191 | 34,29 | 1516 | 489 |  |
| ADH1C ---- tag |  | A/A | 1.03 (0.71-1.51) | 0.86 | . | . | . | 77 | 6,48 | 37 | 6,64 | 1516 | 489 |  |
| ADH1C ---- tag | rs2298753 | T/T | 1.00 (.-.) | . | 0.22 | 0.92 | 0.88 | 977 | 82,17 | 465 | 83,48 | 1516 | 489 | low_count |
| ADH1C ---- tag |  | T/C | 0.88 (0.68-1.13) | 0.30 | . | . | . | 195 | 16,40 | 84 | 15,08 | 1516 | 489 |  |
| ADH1C ---- tag |  | C/C | 0.74 (0.33-1.66) | 0.46 | . | . | . | 17 | 1,43 | 8 | 1,44 | 1516 | 489 |  |
| ADH1C ---- tag | rs2866152 | G/G | 1.00 (.-.) | . | 0.77 | 0.97 | 0.90 | 727 | 61,14 | 331 | 59,43 | 1516 | 489 |  |
| ADH1C ---- tag |  | G/C | 0.97 (0.80-1.17) | 0.75 | . | . | . | 411 | 34,57 | 204 | 36,62 | 1516 | 489 |  |
| ADH1C ---- tag |  | C/C | 1.28 (0.81-2.02) | 0.30 | . | . | . | 51 | 4,29 | 22 | 3,95 | 1516 | 489 |  |
| ADH1C ---- tag | rs904096 | T/T | 1.00 (.-.) | . | 0.86 | 0.98 | 0.90 | 484 | 40,71 | 227 | 40,75 | 1516 | 489 |  |
| ADH1C ---- tag |  | T/G | 0.88 (0.73-1.07) | 0.21 | . | . | . | 552 | 46,43 | 248 | 44,52 | 1516 | 489 |  |
| ADH1C ---- tag |  | G/G | 1.05 (0.80-1.39) | 0.71 | . | . | . | 153 | 12,87 | 82 | 14,72 | 1516 | 489 |  |
| BHMT ---- tag | rs10944 | A/A | 1.00 (.-.) | . | 0.74 | 0.97 | 0.87 | 283 | 23,80 | 136 | 24,42 | 1516 | 489 |  |
| BHMT ---- tag |  | A/C | 0.90 (0.72-1.12) | 0.34 | . | . | . | 621 | 52,23 | 288 | 51,71 | 1516 | 489 |  |
| BHMT ---- tag |  | C/C | 0.96 (0.74-1.23) | 0.73 | . | . | . | 285 | 23,97 | 133 | 23,88 | 1516 | 489 |  |
| BHMT ---- tag | rs12655567 | C/C | 1.00 (.-.) | . | 0.48 | 0.96 | 0.87 | 434 | 36,50 | 218 | 39,14 | 1516 | 489 |  |
| BHMT ---- tag |  | C/G | 0.79 (0.65-0.96) | 0.02 | . | . | . | 581 | 48,86 | 251 | 45,06 | 1516 | 489 |  |
| BHMT ---- tag |  | G/G | 1.01 (0.78-1.32) | 0.92 | . | . | . | 174 | 14,63 | 88 | 15,80 | 1516 | 489 |  |
| BHMT ---- tag | rs1291041 | G/G | 1.00 (.-.) | . | 0.48 | 0.96 | 0.87 | 498 | 41,88 | 247 | 44,34 | 1516 | 489 |  |
| BHMT ---- tag |  | G/T | 0.76 (0.63-0.92) | 0.01 | . | . | . | 551 | 46,34 | 236 | 42,37 | 1516 | 489 |  |
| BHMT ---- tag |  | T/T | 1.09 (0.83-1.44) | 0.52 | . | . | . | 140 | 11,77 | 74 | 13,29 | 1516 | 489 |  |
| BHMT ---- tag | rs16876500 | C/C | 1.00 (.-.) | . | 0.94 | 0.99 | 0.94 | 944 | 79,39 | 444 | 79,71 | 1516 | 489 | low_count |
| BHMT ---- tag |  | C/T | 1.08 (0.86-1.36) | 0.49 | . | . | . | 233 | 19,60 | 108 | 19,39 | 1516 | 489 |  |
| BHMT ---- tag |  | T/T | 0.51 (0.16-1.61) | 0.25 | . | . | . | 12 | 1,01 | 5 | 0,90 | 1516 | 489 |  |
| BHMT ---- tag | rs492842 | A/A | 1.00 (.-.) | . | 0.64 | 0.96 | 0.87 | 437 | 36,75 | 215 | 38,60 | 1516 | 489 |  |
| BHMT ---- tag |  | A/G | 0.90 (0.74-1.10) | 0.31 | . | . | . | 596 | 50,13 | 256 | 45,96 | 1516 | 489 |  |
| BHMT ---- tag |  | G/G | 1.17 (0.89-1.54) | 0.27 | . | . | . | 156 | 13,12 | 86 | 15,44 | 1516 | 489 |  |
| BHMT ---- tag | rs558133 | T/T | 1.00 (.-.) | . | 0.39 | 0.96 | 0.87 | 566 | 47,60 | 270 | 48,47 | 1516 | 489 |  |
| BHMT ---- tag |  | T/G | 0.92 (0.76-1.11) | 0.39 | . | . | . | 482 | 40,54 | 228 | 40,93 | 1516 | 489 |  |
| BHMT ---- tag |  | G/G | 0.91 (0.67-1.24) | 0.55 | . | . | . | 141 | 11,86 | 59 | 10,59 | 1516 | 489 |  |
| BHMT ---- tag | rs9637824 | A/A | 1.00 (.-.) | . | 0.62 | 0.96 | 0.87 | 438 | 36,84 | 209 | 37,52 | 1516 | 489 |  |
| BHMT ---- tag |  | A/G | 0.92 (0.76-1.12) | 0.39 | . | . | . | 593 | 49,87 | 263 | 47,22 | 1516 | 489 |  |
| BHMT ---- tag |  | G/G | 1.15 (0.88-1.52) | 0.30 | . | . | . | 158 | 13,29 | 85 | 15,26 | 1516 | 489 |  |
| BHMT2 ---- tag | rs16876512 | C/C | 1.00 (.-.) | . | 0.80 | 0.98 | 0.80 | 939 | 78,97 | 441 | 79,17 | 1516 | 489 | low_count |
| BHMT2 ---- tag |  | C/T | 1.09 (0.87-1.37) | 0.44 | . | . | . | 237 | 19,93 | 110 | 19,75 | 1516 | 489 |  |
| BHMT2 ---- tag |  | T/T | 0.64 (0.24-1.74) | 0.38 | . | . | . | 13 | 1,09 | 6 | 1,08 | 1516 | 489 |  |
| BHMT2 ---- tag | rs2461248 | A/A | 1.00 (.-.) | . | 0.78 | 0.97 | 0.80 | 279 | 23,47 | 134 | 24,06 | 1516 | 489 |  |
| BHMT2 ---- tag |  | A/T | 0.91 (0.73-1.13) | 0.39 | . | . | . | 624 | 52,48 | 286 | 51,35 | 1516 | 489 |  |
| BHMT2 ---- tag |  | T/T | 0.96 (0.74-1.24) | 0.76 | . | . | . | 286 | 24,05 | 137 | 24,60 | 1516 | 489 |  |
| BHMT2 ---- tag | rs2909856 | T/T | 1.00 (.-.) | . | 0.78 | 0.97 | 0.80 | 478 | 40,20 | 233 | 41,83 | 1516 | 489 |  |
| BHMT2 ---- tag |  | T/C | 0.85 (0.70-1.04) | 0.11 | . | . | . | 574 | 48,28 | 246 | 44,17 | 1516 | 489 |  |
| BHMT2 ---- tag |  | C/C | 1.21 (0.91-1.60) | 0.18 | . | . | . | 137 | 11,52 | 78 | 14,00 | 1516 | 489 |  |
| BHMT2 ---- tag | rs476620 | A/A | 1.00 (.-.) | . | 0.62 | 0.96 | 0.80 | 438 | 36,84 | 209 | 37,52 | 1516 | 489 |  |
| BHMT2 ---- tag |  | A/G | 0.92 (0.75-1.12) | 0.39 | . | . | . | 593 | 49,87 | 263 | 47,22 | 1516 | 489 |  |
| BHMT2 ---- tag |  | G/G | 1.15 (0.88-1.52) | 0.30 | . | . | . | 158 | 13,29 | 85 | 15,26 | 1516 | 489 |  |
| BHMT2 ---- candidate literature | rs626105 | G/G | 1.00 (.-.) | . | 0.59 | 0.96 | 0.80 | 745 | 62,66 | 349 | 62,66 | 1516 | 489 |  |
| BHMT2 ---- candidate literature |  | G/A | 0.87 (0.72-1.06) | 0.17 | . | . | . | 396 | 33,31 | 186 | 33,39 | 1516 | 489 |  |
| BHMT2 ---- candidate literature |  | A/A | 1.26 (0.80-1.99) | 0.33 | . | . | . | 48 | 4,04 | 22 | 3,95 | 1516 | 489 |  |
| BHMT2 ---- tag | rs631305 | G/G | 1.00 (.-.) | . | 0.61 | 0.96 | 0.80 | 816 | 68,63 | 388 | 69,66 | 1516 | 489 |  |
| BHMT2 ---- tag |  | G/A | 0.89 (0.72-1.09) | 0.25 | . | . | . | 342 | 28,76 | 152 | 27,29 | 1516 | 489 |  |
| BHMT2 ---- tag |  | A/A | 1.25 (0.74-2.11) | 0.40 | . | . | . | 31 | 2,61 | 17 | 3,05 | 1516 | 489 |  |
| CBS ---- tag | rs11701048 | C/C | 1.00 (.-.) | . | 0.72 | 0.97 | 0.78 | 1008 | 84,78 | 482 | 86,54 | 1516 | 489 | low_count |
| CBS ---- tag |  | C/T | 0.97 (0.75-1.27) | 0.84 | . | . | . | 176 | 14,80 | 73 | 13,11 | 1516 | 489 |  |
| CBS ---- tag |  | T/T | 0.57 (0.08-4.10) | 0.58 | . | . | . | 5 | 0,42 | 2 | 0,36 | 1516 | 489 |  |
| CBS ---- tag | rs234706 | G/G | 1.00 (.-.) | . | 0.78 | 0.97 | 0.78 | 524 | 44,07 | 240 | 43,09 | 1516 | 489 |  |
| CBS ---- tag |  | G/A | 1.03 (0.85-1.25) | 0.75 | . | . | . | 519 | 43,65 | 251 | 45,06 | 1516 | 489 |  |
| CBS ---- tag |  | A/A | 1.03 (0.77-1.38) | 0.85 | . | . | . | 146 | 12,28 | 66 | 11,85 | 1516 | 489 |  |
| CBS ---- tag | rs234711 | C/C | 1.00 (.-.) | . | 0.27 | 0.95 | 0.69 | 714 | 60,05 | 320 | 57,45 | 1516 | 489 |  |
| CBS ---- tag |  | C/A | 1.07 (0.89-1.30) | 0.46 | . | . | . | 411 | 34,57 | 206 | 36,98 | 1516 | 489 |  |
| CBS ---- tag |  | A/A | 1.22 (0.81-1.84) | 0.33 | . | . | . | 64 | 5,38 | 31 | 5,57 | 1516 | 489 |  |
| CBS ---- candidate literature | rs234713 | G/G | 1.00 (.-.) | . | 0.56 | 0.96 | 0.78 | 593 | 49,87 | 273 | 49,01 | 1516 | 489 |  |
| CBS ---- candidate literature |  | G/A | 1.02 (0.85-1.24) | 0.81 | . | . | . | 485 | 40,79 | 234 | 42,01 | 1516 | 489 |  |
| CBS ---- candidate literature |  | A/A | 1.12 (0.80-1.56) | 0.51 | . | . | . | 111 | 9,34 | 50 | 8,98 | 1516 | 489 |  |
| CBS ---- tag | rs2839623 | T/T | 1.00 (.-.) | . | 0.24 | 0.95 | 0.69 | 990 | 83,26 | 451 | 80,97 | 1516 | 489 | low_count |
| CBS ---- tag |  | T/A | 1.16 (0.92-1.47) | 0.21 | . | . | . | 189 | 15,90 | 102 | 18,31 | 1516 | 489 |  |
| CBS ---- tag |  | A/A | 1.07 (0.40-2.89) | 0.89 | . | . | . | 10 | 0,84 | 4 | 0,72 | 1516 | 489 |  |
| CBS ---- tag | rs2839626 | C/C | 1.00 (.-.) | . | 0.77 | 0.97 | 0.78 | 545 | 45,84 | 249 | 44,70 | 1516 | 489 |  |
| CBS ---- tag |  | C/T | 1.07 (0.88-1.29) | 0.51 | . | . | . | 511 | 42,98 | 247 | 44,34 | 1516 | 489 |  |
| CBS ---- tag |  | T/T | 0.88 (0.65-1.20) | 0.43 | . | . | . | 133 | 11,19 | 61 | 10,95 | 1516 | 489 |  |
| CBS ---- tag | rs422791 | T/T | 1.00 (.-.) | . | 0.11 | 0.80 | 0.56 | 606 | 50,97 | 274 | 49,19 | 1516 | 489 |  |
| CBS ---- tag |  | T/C | 1.27 (1.05-1.53) | 0.01 | . | . | . | 489 | 41,13 | 231 | 41,47 | 1516 | 489 |  |
| CBS ---- tag |  | C/C | 1.06 (0.76-1.48) | 0.72 | . | . | . | 94 | 7,91 | 52 | 9,34 | 1516 | 489 |  |
| CBS ---- tag | rs706209 | C/C | 1.00 (.-.) | . | 0.54 | 0.96 | 0.78 | 371 | 31,20 | 161 | 28,90 | 1516 | 489 |  |
| CBS ---- tag |  | C/T | 0.97 (0.78-1.20) | 0.77 | . | . | . | 578 | 48,61 | 280 | 50,27 | 1516 | 489 |  |
| CBS ---- tag |  | T/T | 0.92 (0.71-1.20) | 0.54 | . | . | . | 240 | 20,19 | 116 | 20,83 | 1516 | 489 |  |
| CBS ---- tag | rs719037 | A/A | 1.00 (.-.) | . | 0.03 | 0.73 | 0.30 | 416 | 34,99 | 175 | 31,42 | 1516 | 489 |  |
| CBS ---- tag |  | A/G | 1.23 (1.00-1.51) | 0.05 | . | . | . | 552 | 46,43 | 278 | 49,91 | 1516 | 489 |  |
| CBS ---- tag |  | G/G | 1.30 (0.99-1.69) | 0.06 | . | . | . | 221 | 18,59 | 104 | 18,67 | 1516 | 489 |  |
| CBS ---- tag | rs719038 | T/T | 1.00 (.-.) | . | 0.56 | 0.96 | 0.78 | 515 | 43,31 | 222 | 39,86 | 1516 | 489 |  |
| CBS ---- tag |  | T/C | 0.99 (0.82-1.20) | 0.93 | . | . | . | 515 | 43,31 | 257 | 46,14 | 1516 | 489 |  |
| CBS ---- tag |  | C/C | 0.91 (0.69-1.20) | 0.49 | . | . | . | 159 | 13,37 | 78 | 14,00 | 1516 | 489 |  |
| DHFR ---- tag | rs10474632 | G/G | 1.00 (.-.) | . | 0.03 | 0.73 | 0.34 | 996 | 83,77 | 478 | 85,82 | 1516 | 489 | low_count |
| DHFR ---- tag |  | G/A | 0.72 (0.55-0.94) | 0.02 | . | . | . | 187 | 15,73 | 75 | 13,46 | 1516 | 489 |  |
| DHFR ---- tag |  | A/A | 1.16 (0.43-3.13) | 0.78 | . | . | . | 6 | 0,50 | 4 | 0,72 | 1516 | 489 |  |
| DHFR ---- tag | rs11951910 | T/T | 1.00 (.-.) | . | 0.86 | 0.98 | 0.88 | 961 | 80,82 | 445 | 79,89 | 1516 | 489 | low_count |
| DHFR ---- tag |  | T/C | 1.12 (0.88-1.41) | 0.36 | . | . | . | 210 | 17,66 | 104 | 18,67 | 1516 | 489 |  |
| DHFR ---- tag |  | C/C | 0.59 (0.29-1.22) | 0.15 | . | . | . | 18 | 1,51 | 8 | 1,44 | 1516 | 489 |  |
| DHFR ---- tag | rs1643665 | T/T | 1.00 (.-.) | . | 0.81 | 0.98 | 0.88 | 564 | 47,43 | 256 | 45,96 | 1516 | 489 |  |
| DHFR ---- tag |  | T/C | 1.05 (0.88-1.27) | 0.58 | . | . | . | 501 | 42,14 | 258 | 46,32 | 1516 | 489 |  |
| DHFR ---- tag |  | C/C | 0.86 (0.60-1.23) | 0.41 | . | . | . | 124 | 10,43 | 43 | 7,72 | 1516 | 489 |  |
| DHFR ---- tag | rs1650717 | T/T | 1.00 (.-.) | . | 0.52 | 0.96 | 0.88 | 648 | 54,50 | 291 | 52,24 | 1516 | 489 |  |
| DHFR ---- tag |  | T/G | 1.17 (0.96-1.41) | 0.12 | . | . | . | 447 | 37,59 | 218 | 39,14 | 1516 | 489 |  |
| DHFR ---- tag |  | G/G | 0.96 (0.69-1.33) | 0.79 | . | . | . | 94 | 7,91 | 48 | 8,62 | 1516 | 489 |  |
| DHFR ---- tag | rs1805355 | G/G | 1.00 (.-.) | . | 0.88 | 0.98 | 0.88 | 1039 | 87,38 | 485 | 87,07 | 1516 | 489 | low_count |
| DHFR ---- tag |  | G/A | 0.99 (0.75-1.30) | 0.95 | . | . | . | 143 | 12,03 | 71 | 12,75 | 1516 | 489 |  |
| DHFR ---- tag |  | A/A | 0.73 (0.10-5.25) | 0.76 | . | . | . | 7 | 0,59 | 1 | 0,18 | 1516 | 489 |  |
| DHFR ---- tag | rs6151617 | A/A | 1.00 (.-.) | . | 0.15 | 0.90 | 0.74 | 437 | 36,75 | 205 | 36,80 | 1516 | 489 |  |
| DHFR ---- tag |  | A/G | 0.99 (0.82-1.20) | 0.94 | . | . | . | 546 | 45,92 | 274 | 49,19 | 1516 | 489 |  |
| DHFR ---- tag |  | G/G | 0.78 (0.59-1.03) | 0.09 | . | . | . | 206 | 17,33 | 78 | 14,00 | 1516 | 489 |  |
| DHFR ---- tag | rs6864493 | T/T | 1.00 (.-.) | . | 0.83 | 0.98 | 0.88 | 677 | 56,94 | 317 | 56,91 | 1516 | 489 |  |
| DHFR ---- tag |  | T/C | 0.97 (0.80-1.16) | 0.71 | . | . | . | 435 | 36,59 | 210 | 37,70 | 1516 | 489 |  |
| DHFR ---- tag |  | C/C | 1.01 (0.68-1.52) | 0.95 | . | . | . | 77 | 6,48 | 30 | 5,39 | 1516 | 489 |  |
| DHFR ---- tag | rs836788 | G/G | 1.00 (.-.) | . | 0.76 | 0.97 | 0.88 | 507 | 42,64 | 239 | 42,91 | 1516 | 489 |  |
| DHFR ---- tag |  | G/A | 0.97 (0.80-1.18) | 0.77 | . | . | . | 519 | 43,65 | 248 | 44,52 | 1516 | 489 |  |
| DHFR ---- tag |  | A/A | 0.97 (0.73-1.28) | 0.81 | . | . | . | 163 | 13,71 | 70 | 12,57 | 1516 | 489 |  |
| DHFR ---- tag | rs836790 | A/A | 1.00 (.-.) | . | 0.22 | 0.92 | 0.74 | 839 | 70,56 | 377 | 67,68 | 1516 | 489 |  |
| DHFR ---- tag |  | A/G | 1.12 (0.92-1.36) | 0.27 | . | . | . | 321 | 27,00 | 167 | 29,98 | 1516 | 489 |  |
| DHFR ---- tag |  | G/G | 1.22 (0.67-2.25) | 0.52 | . | . | . | 29 | 2,44 | 13 | 2,33 | 1516 | 489 |  |
| DHFR ---- tag | rs836817 | G/G | 1.00 (.-.) | . | 0.45 | 0.96 | 0.88 | 560 | 47,10 | 251 | 45,06 | 1516 | 489 |  |
| DHFR ---- tag |  | G/T | 1.14 (0.94-1.38) | 0.18 | . | . | . | 494 | 41,55 | 252 | 45,24 | 1516 | 489 |  |
| DHFR ---- tag |  | T/T | 1.02 (0.75-1.39) | 0.89 | . | . | . | 135 | 11,35 | 54 | 9,69 | 1516 | 489 |  |
| DNMT1 ---- candidate | rs2228612 | A/A | 1.00 (.-.) | . | 0.46 | 0.96 | 0.46 | 1028 | 86,46 | 482 | 86,54 | 1516 | 489 | low_count |
| DNMT1 ---- candidate |  | A/G | 1.12 (0.86-1.46) | 0.42 | . | . | . | 155 | 13,04 | 73 | 13,11 | 1516 | 489 |  |
| DNMT1 ---- candidate |  | G/G | 0.98 (0.24-4.00) | 0.98 | . | . | . | 6 | 0,50 | 2 | 0,36 | 1516 | 489 |  |
| DNMT3A ---- tag | rs10460566 | A/A | 1.00 (.-.) | . | 0.55 | 0.96 | 0.72 | 671 | 56,43 | 335 | 60,14 | 1516 | 489 |  |
| DNMT3A ---- tag |  | A/G | 0.99 (0.82-1.20) | 0.96 | . | . | . | 453 | 38,10 | 199 | 35,73 | 1516 | 489 |  |
| DNMT3A ---- tag |  | G/G | 0.82 (0.52-1.27) | 0.37 | . | . | . | 65 | 5,47 | 23 | 4,13 | 1516 | 489 |  |
| DNMT3A ---- candidate literature | rs11695471 | T/T | 1.00 (.-.) | . | 0.59 | 0.96 | 0.72 | 536 | 45,08 | 236 | 42,37 | 1516 | 489 |  |
| DNMT3A ---- candidate literature |  | T/A | 1.13 (0.93-1.37) | 0.21 | . | . | . | 525 | 44,15 | 253 | 45,42 | 1516 | 489 |  |
| DNMT3A ---- candidate literature |  | A/A | 1.00 (0.74-1.34) | 0.99 | . | . | . | 128 | 10,77 | 68 | 12,21 | 1516 | 489 |  |
| DNMT3A ---- tag | rs11887120 | C/C | 1.00 (.-.) | . | 0.29 | 0.95 | 0.72 | 416 | 34,99 | 195 | 35,01 | 1516 | 489 |  |
| DNMT3A ---- tag |  | C/T | 1.04 (0.85-1.26) | 0.72 | . | . | . | 557 | 46,85 | 273 | 49,01 | 1516 | 489 |  |
| DNMT3A ---- tag |  | T/T | 0.83 (0.64-1.09) | 0.19 | . | . | . | 216 | 18,17 | 89 | 15,98 | 1516 | 489 |  |
| DNMT3A ---- tag | rs12991495 | T/T | 1.00 (.-.) | . | 0.22 | 0.92 | 0.72 | 585 | 49,20 | 248 | 44,52 | 1516 | 489 |  |
| DNMT3A ---- tag |  | T/C | 1.10 (0.91-1.33) | 0.33 | . | . | . | 497 | 41,80 | 247 | 44,34 | 1516 | 489 |  |
| DNMT3A ---- tag |  | C/C | 1.17 (0.86-1.59) | 0.31 | . | . | . | 107 | 9,00 | 62 | 11,13 | 1516 | 489 |  |
| DNMT3A ---- tag | rs13401241 | A/A | 1.00 (.-.) | . | 0.84 | 0.98 | 0.84 | 342 | 28,76 | 149 | 26,75 | 1516 | 489 |  |
| DNMT3A ---- tag |  | A/C | 1.00 (0.81-1.25) | 0.98 | . | . | . | 579 | 48,70 | 294 | 52,78 | 1516 | 489 |  |
| DNMT3A ---- tag |  | C/C | 1.03 (0.79-1.34) | 0.84 | . | . | . | 268 | 22,54 | 114 | 20,47 | 1516 | 489 |  |
| DNMT3A ---- candidate literature | rs13420827 | C/C | 1.00 (.-.) | . | 0.41 | 0.96 | 0.72 | 784 | 65,94 | 374 | 67,15 | 1516 | 489 |  |
| DNMT3A ---- candidate literature |  | C/G | 0.97 (0.79-1.18) | 0.75 | . | . | . | 364 | 30,61 | 159 | 28,55 | 1516 | 489 |  |
| DNMT3A ---- candidate literature |  | G/G | 0.80 (0.51-1.26) | 0.34 | . | . | . | 41 | 3,45 | 24 | 4,31 | 1516 | 489 |  |
| DNMT3A ---- tag | rs13428812 | A/A | 1.00 (.-.) | . | 0.63 | 0.96 | 0.72 | 550 | 46,26 | 277 | 49,73 | 1516 | 489 |  |
| DNMT3A ---- tag |  | A/G | 1.09 (0.90-1.32) | 0.38 | . | . | . | 516 | 43,40 | 237 | 42,55 | 1516 | 489 |  |
| DNMT3A ---- tag |  | G/G | 1.00 (0.71-1.41) | 0.98 | . | . | . | 123 | 10,34 | 43 | 7,72 | 1516 | 489 |  |
| DNMT3A ---- tag | rs4665287 | C/C | 1.00 (.-.) | . | 0.38 | 0.96 | 0.72 | 796 | 66,95 | 380 | 68,22 | 1516 | 489 |  |
| DNMT3A ---- tag |  | C/T | 0.95 (0.78-1.16) | 0.62 | . | . | . | 350 | 29,44 | 152 | 27,29 | 1516 | 489 |  |
| DNMT3A ---- tag |  | T/T | 0.82 (0.53-1.29) | 0.40 | . | . | . | 43 | 3,62 | 25 | 4,49 | 1516 | 489 |  |
| DNMT3B ---- tag | rs13045669 | A/A | 1.00 (.-.) | . | 0.55 | 0.96 | 0.78 | 1099 | 92,43 | 519 | 93,18 | 1516 | 489 | low_count |
| DNMT3B ---- tag |  | A/G | 0.83 (0.55-1.23) | 0.35 | . | . | . | 89 | 7,49 | 37 | 6,64 | 1516 | 489 |  |
| DNMT3B ---- tag |  | G/G | 32.77 (4.40-243.8) | 0.00 | . | . | . | 1 | 0,08 | 1 | 0,18 | 1516 | 489 |  |
| DNMT3B ---- tag | rs17123673 | A/A | 1.00 (.-.) | . | 0.28 | 0.95 | 0.74 | 1092 | 91,84 | 507 | 91,02 | 1516 | 489 | low_count |
| DNMT3B ---- tag |  | A/G | 0.78 (0.56-1.08) | 0.13 | . | . | . | 96 | 8,07 | 47 | 8,44 | 1516 | 489 |  |
| DNMT3B ---- tag |  | G/G | 1.67 (0.51-5.42) | 0.40 | . | . | . | 1 | 0,08 | 3 | 0,54 | 1516 | 489 |  |
| DNMT3B ---- tag | rs183603 | A/A | 1.00 (.-.) | . | 0.14 | 0.88 | 0.74 | 652 | 54,84 | 313 | 56,19 | 1516 | 489 |  |
| DNMT3B ---- tag |  | A/G | 0.93 (0.77-1.13) | 0.47 | . | . | . | 452 | 38,02 | 218 | 39,14 | 1516 | 489 |  |
| DNMT3B ---- tag |  | G/G | 0.71 (0.47-1.09) | 0.12 | . | . | . | 85 | 7,15 | 26 | 4,67 | 1516 | 489 |  |
| DNMT3B ---- tag | rs2235760 | C/C | 1.00 (.-.) | . | 0.74 | 0.97 | 0.82 | 845 | 71,07 | 391 | 70,20 | 1516 | 489 |  |
| DNMT3B ---- tag |  | C/T | 1.10 (0.90-1.35) | 0.36 | . | . | . | 309 | 25,99 | 153 | 27,47 | 1516 | 489 |  |
| DNMT3B ---- tag |  | T/T | 0.58 (0.31-1.10) | 0.09 | . | . | . | 35 | 2,94 | 13 | 2,33 | 1516 | 489 |  |
| DNMT3B ---- tag | rs2424908 | C/C | 1.00 (.-.) | . | 0.45 | 0.96 | 0.74 | 761 | 64,00 | 371 | 66,61 | 1516 | 489 |  |
| DNMT3B ---- tag |  | C/T | 1.01 (0.83-1.23) | 0.96 | . | . | . | 377 | 31,71 | 171 | 30,70 | 1516 | 489 |  |
| DNMT3B ---- tag |  | T/T | 0.68 (0.39-1.18) | 0.17 | . | . | . | 51 | 4,29 | 15 | 2,69 | 1516 | 489 |  |
| DNMT3B ---- candidate literature | rs2424909 | T/T | 1.00 (.-.) | . | 0.36 | 0.96 | 0.74 | 450 | 37,85 | 225 | 40,39 | 1516 | 489 |  |
| DNMT3B ---- candidate literature |  | T/C | 1.10 (0.91-1.34) | 0.33 | . | . | . | 554 | 46,59 | 262 | 47,04 | 1516 | 489 |  |
| DNMT3B ---- candidate literature |  | C/C | 0.79 (0.59-1.06) | 0.12 | . | . | . | 185 | 15,56 | 70 | 12,57 | 1516 | 489 |  |
| DNMT3B ---- tag | rs4911108 | A/A | 1.00 (.-.) | . | 0.32 | 0.96 | 0.74 | 485 | 40,79 | 237 | 42,55 | 1516 | 489 |  |
| DNMT3B ---- tag |  | A/G | 1.09 (0.90-1.32) | 0.40 | . | . | . | 532 | 44,74 | 257 | 46,14 | 1516 | 489 |  |
| DNMT3B ---- tag |  | G/G | 0.78 (0.57-1.05) | 0.10 | . | . | . | 172 | 14,47 | 63 | 11,31 | 1516 | 489 |  |
| DNMT3B ---- tag | rs6058896 | C/C | 1.00 (.-.) | . | 0.65 | 0.96 | 0.81 | 1059 | 89,07 | 494 | 88,69 | 1516 | 489 | low_count |
| DNMT3B ---- tag |  | C/T | 1.01 (0.76-1.35) | 0.95 | . | . | . | 126 | 10,60 | 60 | 10,77 | 1516 | 489 |  |
| DNMT3B ---- tag |  | T/T | 2.06 (0.66-6.45) | 0.22 | . | . | . | 4 | 0,34 | 3 | 0,54 | 1516 | 489 |  |
| DNMT3B ---- tag | rs6119954 | G/G | 1.00 (.-.) | . | 0.44 | 0.96 | 0.74 | 818 | 68,80 | 390 | 70,02 | 1516 | 489 |  |
| DNMT3B ---- tag |  | G/A | 1.05 (0.86-1.29) | 0.61 | . | . | . | 331 | 27,84 | 153 | 27,47 | 1516 | 489 |  |
| DNMT3B ---- tag |  | A/A | 0.57 (0.31-1.04) | 0.07 | . | . | . | 40 | 3,36 | 14 | 2,51 | 1516 | 489 |  |
| DNMT3B ---- tag | rs6579038 | A/A | 1.00 (.-.) | . | 0.82 | 0.98 | 0.82 | 1051 | 88,39 | 493 | 88,51 | 1516 | 489 | low_count |
| DNMT3B ---- tag |  | A/G | 1.00 (0.75-1.33) | 0.99 | . | . | . | 135 | 11,35 | 62 | 11,13 | 1516 | 489 |  |
| DNMT3B ---- tag |  | G/G | 1.69 (0.42-6.85) | 0.46 | . | . | . | 3 | 0,25 | 2 | 0,36 | 1516 | 489 |  |
| DPYD ---- tag | rs1034215 | C/C | 1.00 (.-.) | . | 0.22 | 0.92 | 0.59 | 717 | 60,30 | 358 | 64,27 | 1516 | 489 |  |
| DPYD ---- tag |  | C/T | 0.91 (0.75-1.11) | 0.35 | . | . | . | 398 | 33,47 | 175 | 31,42 | 1516 | 489 |  |
| DPYD ---- tag |  | T/T | 0.80 (0.51-1.27) | 0.35 | . | . | . | 74 | 6,22 | 24 | 4,31 | 1516 | 489 |  |
| DPYD ---- tag | rs10783058 | T/T | 1.00 (.-.) | . | 0.02 | 0.73 | 0.49 | 519 | 43,65 | 212 | 38,06 | 1516 | 489 |  |
| DPYD ---- tag |  | T/C | 1.27 (1.05-1.55) | 0.02 | . | . | . | 528 | 44,41 | 265 | 47,58 | 1516 | 489 |  |
| DPYD ---- tag |  | C/C | 1.30 (0.99-1.72) | 0.06 | . | . | . | 142 | 11,94 | 80 | 14,36 | 1516 | 489 |  |
| DPYD ---- tag | rs10783070 | C/C | 1.00 (.-.) | . | 0.20 | 0.92 | 0.59 | 828 | 69,64 | 394 | 70,74 | 1516 | 489 |  |
| DPYD ---- tag |  | C/T | 1.18 (0.97-1.44) | 0.10 | . | . | . | 332 | 27,92 | 150 | 26,93 | 1516 | 489 |  |
| DPYD ---- tag |  | T/T | 0.99 (0.54-1.81) | 0.97 | . | . | . | 29 | 2,44 | 13 | 2,33 | 1516 | 489 |  |
| DPYD ---- tag | rs10875048 | G/G | 1.00 (.-.) | . | 0.49 | 0.96 | 0.75 | 790 | 66,44 | 370 | 66,43 | 1516 | 489 |  |
| DPYD ---- tag |  | G/A | 0.91 (0.74-1.11) | 0.33 | . | . | . | 367 | 30,87 | 165 | 29,62 | 1516 | 489 |  |
| DPYD ---- tag |  | A/A | 1.02 (0.63-1.66) | 0.93 | . | . | . | 32 | 2,69 | 22 | 3,95 | 1516 | 489 |  |
| DPYD ---- tag | rs10875055 | C/C | 1.00 (.-.) | . | 0.04 | 0.77 | 0.49 | 355 | 29,86 | 129 | 23,16 | 1516 | 489 |  |
| DPYD ---- tag |  | C/T | 1.33 (1.07-1.66) | 0.01 | . | . | . | 594 | 49,96 | 298 | 53,50 | 1516 | 489 |  |
| DPYD ---- tag |  | T/T | 1.29 (0.99-1.69) | 0.06 | . | . | . | 240 | 20,19 | 130 | 23,34 | 1516 | 489 |  |
| DPYD ---- tag | rs10875079 | A/A | 1.00 (.-.) | . | 0.30 | 0.95 | 0.67 | 313 | 26,32 | 148 | 26,57 | 1516 | 489 |  |
| DPYD ---- tag |  | A/G | 0.98 (0.79-1.22) | 0.89 | . | . | . | 581 | 48,86 | 278 | 49,91 | 1516 | 489 |  |
| DPYD ---- tag |  | G/G | 0.87 (0.68-1.12) | 0.30 | . | . | . | 295 | 24,81 | 131 | 23,52 | 1516 | 489 |  |
| DPYD ---- tag | rs10875085 | A/A | 1.00 (.-.) | . | 0.54 | 0.96 | 0.79 | 805 | 67,70 | 378 | 67,86 | 1516 | 489 |  |
| DPYD ---- tag |  | A/T | 0.92 (0.75-1.12) | 0.40 | . | . | . | 354 | 29,77 | 160 | 28,73 | 1516 | 489 |  |
| DPYD ---- tag |  | T/T | 1.02 (0.62-1.70) | 0.93 | . | . | . | 30 | 2,52 | 19 | 3,41 | 1516 | 489 |  |
| DPYD ---- tag | rs10875097 | G/G | 1.00 (.-.) | . | 0.09 | 0.79 | 0.49 | 833 | 70,06 | 365 | 65,53 | 1516 | 489 |  |
| DPYD ---- tag |  | G/A | 1.24 (1.02-1.51) | 0.03 | . | . | . | 320 | 26,91 | 172 | 30,88 | 1516 | 489 |  |
| DPYD ---- tag |  | A/A | 1.03 (0.61-1.74) | 0.91 | . | . | . | 36 | 3,03 | 20 | 3,59 | 1516 | 489 |  |
| DPYD ---- tag | rs11165781 | T/T | 1.00 (.-.) | . | 0.69 | 0.97 | 0.83 | 802 | 67,45 | 382 | 68,58 | 1516 | 489 |  |
| DPYD ---- tag |  | T/C | 0.94 (0.77-1.15) | 0.55 | . | . | . | 353 | 29,69 | 162 | 29,08 | 1516 | 489 |  |
| DPYD ---- tag |  | C/C | 1.07 (0.58-1.95) | 0.83 | . | . | . | 34 | 2,86 | 13 | 2,33 | 1516 | 489 |  |
| DPYD ---- tag | rs11165783 | T/T | 1.00 (.-.) | . | 0.07 | 0.79 | 0.49 | 658 | 55,34 | 303 | 54,40 | 1516 | 489 |  |
| DPYD ---- tag |  | T/C | 1.10 (0.91-1.33) | 0.32 | . | . | . | 446 | 37,51 | 207 | 37,16 | 1516 | 489 |  |
| DPYD ---- tag |  | C/C | 1.36 (0.98-1.89) | 0.07 | . | . | . | 85 | 7,15 | 47 | 8,44 | 1516 | 489 |  |
| DPYD ---- tag | rs11165873 | A/A | 1.00 (.-.) | . | 0.22 | 0.92 | 0.59 | 316 | 26,58 | 169 | 30,34 | 1516 | 489 |  |
| DPYD ---- tag |  | A/T | 0.87 (0.70-1.07) | 0.18 | . | . | . | 606 | 50,97 | 266 | 47,76 | 1516 | 489 |  |
| DPYD ---- tag |  | T/T | 0.86 (0.67-1.11) | 0.26 | . | . | . | 267 | 22,46 | 122 | 21,90 | 1516 | 489 |  |
| DPYD ---- tag | rs11165875 | T/T | 1.00 (.-.) | . | 0.20 | 0.92 | 0.59 | 489 | 41,13 | 209 | 37,52 | 1516 | 489 |  |
| DPYD ---- tag |  | T/C | 1.12 (0.92-1.37) | 0.25 | . | . | . | 547 | 46,01 | 261 | 46,86 | 1516 | 489 |  |
| DPYD ---- tag |  | C/C | 1.16 (0.89-1.52) | 0.27 | . | . | . | 153 | 12,87 | 87 | 15,62 | 1516 | 489 |  |
| DPYD ---- tag | rs11165881 | T/T | 1.00 (.-.) | . | 0.79 | 0.97 | 0.86 | 417 | 35,07 | 204 | 36,62 | 1516 | 489 |  |
| DPYD ---- tag |  | T/C | 1.10 (0.90-1.34) | 0.34 | . | . | . | 555 | 46,68 | 265 | 47,58 | 1516 | 489 |  |
| DPYD ---- tag |  | C/C | 1.00 (0.76-1.30) | 0.98 | . | . | . | 217 | 18,25 | 88 | 15,80 | 1516 | 489 |  |
| DPYD ---- tag | rs11587873 | C/C | 1.00 (.-.) | . | 0.05 | 0.77 | 0.49 | 659 | 55,42 | 340 | 61,04 | 1516 | 489 |  |
| DPYD ---- tag |  | C/T | 0.93 (0.77-1.12) | 0.43 | . | . | . | 458 | 38,52 | 200 | 35,91 | 1516 | 489 |  |
| DPYD ---- tag |  | T/T | 0.53 (0.31-0.90) | 0.02 | . | . | . | 72 | 6,06 | 17 | 3,05 | 1516 | 489 |  |
| DPYD ---- tag | rs12030174 | C/C | 1.00 (.-.) | . | 0.41 | 0.96 | 0.72 | 861 | 72,41 | 401 | 71,99 | 1516 | 489 |  |
| DPYD ---- tag |  | C/T | 0.90 (0.73-1.10) | 0.31 | . | . | . | 306 | 25,74 | 143 | 25,67 | 1516 | 489 |  |
| DPYD ---- tag |  | T/T | 1.02 (0.54-1.93) | 0.94 | . | . | . | 22 | 1,85 | 13 | 2,33 | 1516 | 489 |  |
| DPYD ---- tag | rs12046744 | A/A | 1.00 (.-.) | . | 0.11 | 0.80 | 0.52 | 638 | 53,66 | 305 | 54,76 | 1516 | 489 |  |
| DPYD ---- tag |  | A/C | 1.09 (0.91-1.32) | 0.35 | . | . | . | 458 | 38,52 | 220 | 39,50 | 1516 | 489 |  |
| DPYD ---- tag |  | C/C | 0.53 (0.35-0.80) | 0.00 | . | . | . | 93 | 7,82 | 32 | 5,75 | 1516 | 489 |  |
| DPYD ---- tag | rs12047910 | G/G | 1.00 (.-.) | . | 0.37 | 0.96 | 0.70 | 853 | 71,74 | 413 | 74,15 | 1516 | 489 |  |
| DPYD ---- tag |  | G/A | 0.85 (0.69-1.05) | 0.13 | . | . | . | 313 | 26,32 | 130 | 23,34 | 1516 | 489 |  |
| DPYD ---- tag |  | A/A | 1.28 (0.71-2.29) | 0.41 | . | . | . | 23 | 1,93 | 14 | 2,51 | 1516 | 489 |  |
| DPYD ---- tag | rs12073044 | T/T | 1.00 (.-.) | . | 0.17 | 0.92 | 0.59 | 936 | 78,72 | 453 | 81,33 | 1516 | 489 | low_count |
| DPYD ---- tag |  | T/A | 0.86 (0.68-1.09) | 0.22 | . | . | . | 241 | 20,27 | 101 | 18,13 | 1516 | 489 |  |
| DPYD ---- tag |  | A/A | 0.65 (0.16-2.63) | 0.55 | . | . | . | 12 | 1,01 | 3 | 0,54 | 1516 | 489 |  |
| DPYD ---- tag | rs12126093 | T/T | 1.00 (.-.) | . | 0.47 | 0.96 | 0.75 | 596 | 50,13 | 286 | 51,35 | 1516 | 489 |  |
| DPYD ---- tag |  | T/C | 0.91 (0.75-1.10) | 0.35 | . | . | . | 497 | 41,80 | 219 | 39,32 | 1516 | 489 |  |
| DPYD ---- tag |  | C/C | 0.96 (0.68-1.34) | 0.80 | . | . | . | 96 | 8,07 | 52 | 9,34 | 1516 | 489 |  |
| DPYD ---- tag | rs12134028 | C/C | 1.00 (.-.) | . | 0.16 | 0.91 | 0.59 | 1070 | 89,99 | 496 | 89,05 | 1516 | 489 | low_count |
| DPYD ---- tag |  | C/T | 0.79 (0.58-1.06) | 0.12 | . | . | . | 117 | 9,84 | 59 | 10,59 | 1516 | 489 |  |
| DPYD ---- tag |  | T/T | 1.94 (0.27-14.18) | 0.51 | . | . | . | 2 | 0,17 | 2 | 0,36 | 1516 | 489 |  |
| DPYD ---- tag | rs12740796 | T/T | 1.00 (.-.) | . | 0.62 | 0.96 | 0.83 | 890 | 74,85 | 417 | 74,87 | 1516 | 489 |  |
| DPYD ---- tag |  | T/C | 1.05 (0.85-1.29) | 0.68 | . | . | . | 277 | 23,30 | 128 | 22,98 | 1516 | 489 |  |
| DPYD ---- tag |  | C/C | 1.11 (0.59-2.10) | 0.74 | . | . | . | 22 | 1,85 | 12 | 2,15 | 1516 | 489 |  |
| DPYD ---- tag | rs1333717 | A/A | 1.00 (.-.) | . | 0.25 | 0.95 | 0.60 | 681 | 57,28 | 341 | 61,22 | 1516 | 489 |  |
| DPYD ---- tag |  | A/G | 0.91 (0.75-1.10) | 0.32 | . | . | . | 428 | 36,00 | 188 | 33,75 | 1516 | 489 |  |
| DPYD ---- tag |  | G/G | 0.85 (0.56-1.29) | 0.45 | . | . | . | 80 | 6,73 | 28 | 5,03 | 1516 | 489 |  |
| DPYD ---- tag | rs1413228 | A/A | 1.00 (.-.) | . | 0.91 | 0.98 | 0.91 | 952 | 80,07 | 443 | 79,53 | 1516 | 489 | low_count |
| DPYD ---- tag |  | A/G | 1.04 (0.83-1.31) | 0.72 | . | . | . | 228 | 19,18 | 107 | 19,21 | 1516 | 489 |  |
| DPYD ---- tag |  | G/G | 0.82 (0.34-2.00) | 0.67 | . | . | . | 9 | 0,76 | 7 | 1,26 | 1516 | 489 |  |
| DPYD ---- tag | rs1415681 | G/G | 1.00 (.-.) | . | 0.87 | 0.98 | 0.90 | 884 | 74,35 | 424 | 76,12 | 1516 | 489 |  |
| DPYD ---- tag |  | G/T | 1.12 (0.90-1.39) | 0.31 | . | . | . | 272 | 22,88 | 120 | 21,54 | 1516 | 489 |  |
| DPYD ---- tag |  | T/T | 0.74 (0.41-1.36) | 0.34 | . | . | . | 33 | 2,78 | 13 | 2,33 | 1516 | 489 |  |
| DPYD ---- tag | rs1514495 | C/C | 1.00 (.-.) | . | 0.72 | 0.97 | 0.83 | 690 | 58,03 | 329 | 59,07 | 1516 | 489 |  |
| DPYD ---- tag |  | C/T | 0.97 (0.80-1.17) | 0.72 | . | . | . | 439 | 36,92 | 196 | 35,19 | 1516 | 489 |  |
| DPYD ---- tag |  | T/T | 0.96 (0.65-1.42) | 0.84 | . | . | . | 60 | 5,05 | 32 | 5,75 | 1516 | 489 |  |
| DPYD ---- tag | rs1520658 | A/A | 1.00 (.-.) | . | 0.39 | 0.96 | 0.71 | 943 | 79,31 | 437 | 78,46 | 1516 | 489 | low_count |
| DPYD ---- tag |  | A/G | 1.17 (0.94-1.46) | 0.17 | . | . | . | 231 | 19,43 | 114 | 20,47 | 1516 | 489 |  |
| DPYD ---- tag |  | G/G | 0.68 (0.25-1.84) | 0.45 | . | . | . | 15 | 1,26 | 6 | 1,08 | 1516 | 489 |  |
| DPYD ---- NA | rs17116806 | C/C | 1.00 (.-.) | . | 0.23 | 0.93 | 0.59 | 788 | 66,27 | 346 | 62,12 | 1516 | 489 |  |
| DPYD ---- NA |  | C/A | 1.18 (0.97-1.43) | 0.09 | . | . | . | 360 | 30,28 | 190 | 34,11 | 1516 | 489 |  |
| DPYD ---- NA |  | A/A | 0.99 (0.61-1.60) | 0.97 | . | . | . | 41 | 3,45 | 21 | 3,77 | 1516 | 489 |  |
| DPYD ---- tag | rs17431828 | G/G | 1.00 (.-.) | . | 0.61 | 0.96 | 0.83 | 490 | 41,21 | 240 | 43,09 | 1516 | 489 |  |
| DPYD ---- tag |  | G/C | 1.05 (0.87-1.28) | 0.59 | . | . | . | 543 | 45,67 | 249 | 44,70 | 1516 | 489 |  |
| DPYD ---- tag |  | C/C | 0.86 (0.64-1.17) | 0.34 | . | . | . | 156 | 13,12 | 68 | 12,21 | 1516 | 489 |  |
| DPYD ---- tag | rs17471640 | T/T | 1.00 (.-.) | . | 0.25 | 0.95 | 0.60 | 543 | 45,67 | 269 | 48,29 | 1516 | 489 |  |
| DPYD ---- tag |  | T/C | 0.89 (0.74-1.07) | 0.22 | . | . | . | 531 | 44,66 | 232 | 41,65 | 1516 | 489 |  |
| DPYD ---- tag |  | C/C | 0.89 (0.65-1.22) | 0.47 | . | . | . | 115 | 9,67 | 56 | 10,05 | 1516 | 489 |  |
| DPYD ---- tag | rs17702702 | G/G | 1.00 (.-.) | . | 0.49 | 0.96 | 0.75 | 818 | 68,80 | 393 | 70,56 | 1516 | 489 |  |
| DPYD ---- tag |  | G/C | 0.96 (0.79-1.18) | 0.70 | . | . | . | 333 | 28,01 | 148 | 26,57 | 1516 | 489 |  |
| DPYD ---- tag |  | C/C | 0.82 (0.48-1.41) | 0.48 | . | . | . | 38 | 3,20 | 16 | 2,87 | 1516 | 489 |  |
| DPYD ---- NA | rs1801265 | T/T | 1.00 (.-.) | . | 0.75 | 0.97 | 0.85 | 683 | 57,44 | 331 | 59,43 | 1516 | 489 |  |
| DPYD ---- NA |  | T/C | 1.04 (0.86-1.26) | 0.67 | . | . | . | 428 | 36,00 | 202 | 36,27 | 1516 | 489 |  |
| DPYD ---- NA |  | C/C | 0.78 (0.49-1.25) | 0.30 | . | . | . | 78 | 6,56 | 24 | 4,31 | 1516 | 489 |  |
| DPYD ---- tag | rs2039447 | T/T | 1.00 (.-.) | . | 0.76 | 0.97 | 0.85 | 553 | 46,51 | 257 | 46,14 | 1516 | 489 |  |
| DPYD ---- tag |  | T/C | 1.15 (0.96-1.39) | 0.14 | . | . | . | 520 | 43,73 | 249 | 44,70 | 1516 | 489 |  |
| DPYD ---- tag |  | C/C | 0.89 (0.64-1.25) | 0.51 | . | . | . | 116 | 9,76 | 51 | 9,16 | 1516 | 489 |  |
| DPYD ---- tag | rs2151567 | G/G | 1.00 (.-.) | . | 0.18 | 0.92 | 0.59 | 1074 | 90,33 | 499 | 89,59 | 1516 | 489 | low_count |
| DPYD ---- tag |  | G/A | 1.20 (0.89-1.61) | 0.23 | . | . | . | 112 | 9,42 | 57 | 10,23 | 1516 | 489 |  |
| DPYD ---- tag |  | A/A | 2.52 (0.35-18.16) | 0.36 | . | . | . | 3 | 0,25 | 1 | 0,18 | 1516 | 489 |  |
| DPYD ---- tag | rs2152878 | A/A | 1.00 (.-.) | . | 0.71 | 0.97 | 0.83 | 687 | 57,78 | 330 | 59,25 | 1516 | 489 |  |
| DPYD ---- tag |  | A/G | 1.01 (0.83-1.22) | 0.96 | . | . | . | 427 | 35,91 | 191 | 34,29 | 1516 | 489 |  |
| DPYD ---- tag |  | G/G | 1.11 (0.76-1.62) | 0.60 | . | . | . | 75 | 6,31 | 36 | 6,46 | 1516 | 489 |  |
| DPYD ---- tag | rs2786505 | G/G | 1.00 (.-.) | . | 0.06 | 0.77 | 0.49 | 885 | 74,43 | 417 | 74,87 | 1516 | 489 | low_count |
| DPYD ---- tag |  | G/T | 1.15 (0.93-1.41) | 0.20 | . | . | . | 293 | 24,64 | 130 | 23,34 | 1516 | 489 |  |
| DPYD ---- tag |  | T/T | 1.99 (0.98-4.03) | 0.06 | . | . | . | 11 | 0,93 | 10 | 1,80 | 1516 | 489 |  |
| DPYD ---- tag | rs2786512 | G/G | 1.00 (.-.) | . | 0.50 | 0.96 | 0.75 | 442 | 37,17 | 206 | 36,98 | 1516 | 489 |  |
| DPYD ---- tag |  | G/A | 1.02 (0.84-1.25) | 0.82 | . | . | . | 524 | 44,07 | 259 | 46,50 | 1516 | 489 |  |
| DPYD ---- tag |  | A/A | 0.89 (0.69-1.16) | 0.40 | . | . | . | 223 | 18,76 | 92 | 16,52 | 1516 | 489 |  |
| DPYD ---- tag | rs2786519 | A/A | 1.00 (.-.) | . | 0.28 | 0.95 | 0.65 | 736 | 61,90 | 317 | 56,91 | 1516 | 489 |  |
| DPYD ---- tag |  | A/G | 1.17 (0.96-1.41) | 0.11 | . | . | . | 389 | 32,72 | 206 | 36,98 | 1516 | 489 |  |
| DPYD ---- tag |  | G/G | 1.02 (0.69-1.51) | 0.93 | . | . | . | 64 | 5,38 | 34 | 6,10 | 1516 | 489 |  |
| DPYD ---- tag | rs2811170 | A/A | 1.00 (.-.) | . | 0.05 | 0.77 | 0.49 | 879 | 73,93 | 441 | 79,17 | 1516 | 489 | low_count |
| DPYD ---- tag |  | A/T | 0.73 (0.58-0.92) | 0.01 | . | . | . | 296 | 24,89 | 108 | 19,39 | 1516 | 489 |  |
| DPYD ---- tag |  | T/T | 1.83 (0.86-3.89) | 0.12 | . | . | . | 14 | 1,18 | 8 | 1,44 | 1516 | 489 |  |
| DPYD ---- tag | rs2811199 | G/G | 1.00 (.-.) | . | 0.14 | 0.89 | 0.59 | 847 | 71,24 | 403 | 72,35 | 1516 | 489 |  |
| DPYD ---- tag |  | G/A | 1.17 (0.95-1.43) | 0.13 | . | . | . | 318 | 26,75 | 142 | 25,49 | 1516 | 489 |  |
| DPYD ---- tag |  | A/A | 1.15 (0.61-2.17) | 0.67 | . | . | . | 24 | 2,02 | 12 | 2,15 | 1516 | 489 |  |
| DPYD ---- tag | rs2811219 | T/T | 1.00 (.-.) | . | 0.62 | 0.96 | 0.83 | 672 | 56,52 | 336 | 60,32 | 1516 | 489 |  |
| DPYD ---- tag |  | T/C | 0.89 (0.73-1.07) | 0.22 | . | . | . | 444 | 37,34 | 185 | 33,21 | 1516 | 489 |  |
| DPYD ---- tag |  | C/C | 1.11 (0.76-1.62) | 0.60 | . | . | . | 73 | 6,14 | 36 | 6,46 | 1516 | 489 |  |
| DPYD ---- tag | rs4300257 | A/A | 1.00 (.-.) | . | 0.88 | 0.98 | 0.90 | 762 | 64,09 | 351 | 63,02 | 1516 | 489 |  |
| DPYD ---- tag |  | A/C | 0.95 (0.78-1.16) | 0.62 | . | . | . | 387 | 32,55 | 180 | 32,32 | 1516 | 489 |  |
| DPYD ---- tag |  | C/C | 1.26 (0.80-1.98) | 0.33 | . | . | . | 40 | 3,36 | 26 | 4,67 | 1516 | 489 |  |
| DPYD ---- tag | rs4379706 | T/T | 1.00 (.-.) | . | 0.81 | 0.98 | 0.87 | 668 | 56,18 | 331 | 59,43 | 1516 | 489 |  |
| DPYD ---- tag |  | T/C | 1.03 (0.85-1.24) | 0.79 | . | . | . | 440 | 37,01 | 199 | 35,73 | 1516 | 489 |  |
| DPYD ---- tag |  | C/C | 0.86 (0.55-1.33) | 0.49 | . | . | . | 81 | 6,81 | 27 | 4,85 | 1516 | 489 |  |
| DPYD ---- tag | rs4950021 | T/T | 1.00 (.-.) | . | 0.37 | 0.96 | 0.70 | 363 | 30,53 | 170 | 30,52 | 1516 | 489 |  |
| DPYD ---- tag |  | T/G | 1.07 (0.87-1.32) | 0.52 | . | . | . | 584 | 49,12 | 267 | 47,94 | 1516 | 489 |  |
| DPYD ---- tag |  | G/G | 1.12 (0.87-1.44) | 0.38 | . | . | . | 242 | 20,35 | 120 | 21,54 | 1516 | 489 |  |
| DPYD ---- tag | rs4950033 | T/T | 1.00 (.-.) | . | 0.46 | 0.96 | 0.75 | 341 | 28,68 | 165 | 29,62 | 1516 | 489 |  |
| DPYD ---- tag |  | T/C | 1.01 (0.82-1.25) | 0.92 | . | . | . | 609 | 51,22 | 264 | 47,40 | 1516 | 489 |  |
| DPYD ---- tag |  | C/C | 1.10 (0.86-1.42) | 0.44 | . | . | . | 239 | 20,10 | 128 | 22,98 | 1516 | 489 |  |
| DPYD ---- tag | rs495257 | T/T | 1.00 (.-.) | . | 0.63 | 0.96 | 0.83 | 423 | 35,58 | 185 | 33,21 | 1516 | 489 |  |
| DPYD ---- tag |  | T/C | 1.19 (0.98-1.45) | 0.08 | . | . | . | 573 | 48,19 | 291 | 52,24 | 1516 | 489 |  |
| DPYD ---- tag |  | C/C | 0.99 (0.74-1.31) | 0.93 | . | . | . | 193 | 16,23 | 81 | 14,54 | 1516 | 489 |  |
| DPYD ---- tag | rs552926 | A/A | 1.00 (.-.) | . | 0.03 | 0.73 | 0.49 | 445 | 37,43 | 180 | 32,32 | 1516 | 489 |  |
| DPYD ---- tag |  | A/G | 1.24 (1.01-1.53) | 0.04 | . | . | . | 567 | 47,69 | 271 | 48,65 | 1516 | 489 |  |
| DPYD ---- tag |  | G/G | 1.29 (1.00-1.67) | 0.05 | . | . | . | 177 | 14,89 | 106 | 19,03 | 1516 | 489 |  |
| DPYD ---- tag | rs628959 | A/A | 1.00 (.-.) | . | 0.47 | 0.96 | 0.75 | 565 | 47,52 | 299 | 53,68 | 1516 | 489 |  |
| DPYD ---- tag |  | A/G | 0.91 (0.75-1.10) | 0.35 | . | . | . | 531 | 44,66 | 212 | 38,06 | 1516 | 489 |  |
| DPYD ---- tag |  | G/G | 0.96 (0.68-1.34) | 0.80 | . | . | . | 93 | 7,82 | 46 | 8,26 | 1516 | 489 |  |
| DPYD ---- tag | rs6656660 | G/G | 1.00 (.-.) | . | 0.66 | 0.96 | 0.83 | 902 | 75,86 | 421 | 75,58 | 1516 | 489 |  |
| DPYD ---- tag |  | G/T | 1.03 (0.83-1.28) | 0.81 | . | . | . | 271 | 22,79 | 122 | 21,90 | 1516 | 489 |  |
| DPYD ---- tag |  | T/T | 1.15 (0.64-2.06) | 0.64 | . | . | . | 16 | 1,35 | 14 | 2,51 | 1516 | 489 |  |
| DPYD ---- tag | rs6663670 | A/A | 1.00 (.-.) | . | 0.38 | 0.96 | 0.70 | 847 | 71,24 | 407 | 73,07 | 1516 | 489 | low_count |
| DPYD ---- tag |  | A/C | 1.15 (0.94-1.41) | 0.18 | . | . | . | 317 | 26,66 | 140 | 25,13 | 1516 | 489 |  |
| DPYD ---- tag |  | C/C | 0.85 (0.42-1.72) | 0.65 | . | . | . | 25 | 2,10 | 10 | 1,80 | 1516 | 489 |  |
| DPYD ---- tag | rs6683883 | T/T | 1.00 (.-.) | . | 0.09 | 0.79 | 0.49 | 474 | 39,87 | 193 | 34,65 | 1516 | 489 |  |
| DPYD ---- tag |  | T/C | 1.08 (0.89-1.32) | 0.42 | . | . | . | 552 | 46,43 | 267 | 47,94 | 1516 | 489 |  |
| DPYD ---- tag |  | C/C | 1.27 (0.98-1.66) | 0.08 | . | . | . | 163 | 13,71 | 97 | 17,41 | 1516 | 489 |  |
| DPYD ---- tag | rs6686861 | C/C | 1.00 (.-.) | . | 0.32 | 0.96 | 0.69 | 1042 | 87,64 | 464 | 83,30 | 1516 | 489 | low_count |
| DPYD ---- tag |  | C/T | 1.15 (0.90-1.48) | 0.25 | . | . | . | 142 | 11,94 | 90 | 16,16 | 1516 | 489 |  |
| DPYD ---- tag |  | T/T | 0.87 (0.21-3.50) | 0.84 | . | . | . | 5 | 0,42 | 3 | 0,54 | 1516 | 489 |  |
| DPYD ---- tag | rs7414210 | A/A | 1.00 (.-.) | . | 0.72 | 0.97 | 0.83 | 839 | 70,56 | 408 | 73,25 | 1516 | 489 | low_count |
| DPYD ---- tag |  | A/C | 0.97 (0.79-1.19) | 0.78 | . | . | . | 315 | 26,49 | 139 | 24,96 | 1516 | 489 |  |
| DPYD ---- tag |  | C/C | 0.91 (0.45-1.85) | 0.80 | . | . | . | 35 | 2,94 | 10 | 1,80 | 1516 | 489 |  |
| DPYD ---- tag | rs7530858 | A/A | 1.00 (.-.) | . | 0.20 | 0.92 | 0.59 | 933 | 78,47 | 429 | 77,02 | 1516 | 489 | low_count |
| DPYD ---- tag |  | A/G | 1.14 (0.92-1.42) | 0.22 | . | . | . | 242 | 20,35 | 122 | 21,90 | 1516 | 489 |  |
| DPYD ---- tag |  | G/G | 1.25 (0.46-3.39) | 0.65 | . | . | . | 14 | 1,18 | 6 | 1,08 | 1516 | 489 |  |
| DPYD ---- tag | rs7544128 | C/C | 1.00 (.-.) | . | 0.65 | 0.96 | 0.83 | 640 | 53,83 | 298 | 53,50 | 1516 | 489 |  |
| DPYD ---- tag |  | C/G | 0.99 (0.82-1.20) | 0.93 | . | . | . | 474 | 39,87 | 213 | 38,24 | 1516 | 489 |  |
| DPYD ---- tag |  | G/G | 1.14 (0.81-1.60) | 0.45 | . | . | . | 75 | 6,31 | 46 | 8,26 | 1516 | 489 |  |
| DPYD ---- tag | rs7545340 | G/G | 1.00 (.-.) | . | 0.34 | 0.96 | 0.69 | 653 | 54,92 | 295 | 52,96 | 1516 | 489 |  |
| DPYD ---- tag |  | G/A | 1.13 (0.94-1.37) | 0.19 | . | . | . | 448 | 37,68 | 221 | 39,68 | 1516 | 489 |  |
| DPYD ---- tag |  | A/A | 1.06 (0.75-1.49) | 0.76 | . | . | . | 88 | 7,40 | 41 | 7,36 | 1516 | 489 |  |
| DPYD ---- tag | rs828054 | A/A | 1.00 (.-.) | . | 0.09 | 0.79 | 0.49 | 277 | 23,30 | 149 | 26,75 | 1516 | 489 |  |
| DPYD ---- tag |  | A/C | 1.01 (0.81-1.25) | 0.93 | . | . | . | 593 | 49,87 | 294 | 52,78 | 1516 | 489 |  |
| DPYD ---- tag |  | C/C | 0.79 (0.61-1.03) | 0.08 | . | . | . | 319 | 26,83 | 114 | 20,47 | 1516 | 489 |  |
| DPYD ---- tag | rs885622 | G/G | 1.00 (.-.) | . | 0.08 | 0.79 | 0.49 | 494 | 41,55 | 201 | 36,09 | 1516 | 489 |  |
| DPYD ---- tag |  | G/A | 1.17 (0.96-1.42) | 0.11 | . | . | . | 548 | 46,09 | 271 | 48,65 | 1516 | 489 |  |
| DPYD ---- tag |  | A/A | 1.23 (0.93-1.62) | 0.14 | . | . | . | 147 | 12,36 | 85 | 15,26 | 1516 | 489 |  |
| DPYD ---- tag | rs9437663 | G/G | 1.00 (.-.) | . | 0.87 | 0.98 | 0.90 | 743 | 62,49 | 358 | 64,27 | 1516 | 489 |  |
| DPYD ---- tag |  | G/A | 1.02 (0.84-1.24) | 0.84 | . | . | . | 394 | 33,14 | 178 | 31,96 | 1516 | 489 |  |
| DPYD ---- tag |  | A/A | 0.88 (0.54-1.42) | 0.60 | . | . | . | 52 | 4,37 | 21 | 3,77 | 1516 | 489 |  |
| DPYS ---- tag | rs13249169 | A/A | 1.00 (.-.) | . | 0.53 | 0.96 | 0.65 | 947 | 79,65 | 433 | 77,74 | 1516 | 489 | low_count |
| DPYS ---- tag |  | A/T | 1.10 (0.88-1.38) | 0.39 | . | . | . | 229 | 19,26 | 119 | 21,36 | 1516 | 489 |  |
| DPYS ---- tag |  | T/T | 0.87 (0.36-2.13) | 0.77 | . | . | . | 13 | 1,09 | 5 | 0,90 | 1516 | 489 |  |
| DPYS ---- NA | rs13263121 | T/T | 1.00 (.-.) | . | 0.08 | 0.79 | 0.55 | 488 | 41,04 | 256 | 45,96 | 1516 | 489 |  |
| DPYS ---- NA |  | T/A | 0.82 (0.68-0.99) | 0.04 | . | . | . | 545 | 45,84 | 242 | 43,45 | 1516 | 489 |  |
| DPYS ---- NA |  | A/A | 0.86 (0.63-1.16) | 0.33 | . | . | . | 156 | 13,12 | 59 | 10,59 | 1516 | 489 |  |
| DPYS ---- tag | rs16871361 | T/T | 1.00 (.-.) | . | 0.88 | 0.98 | 0.88 | 1068 | 89,82 | 496 | 89,05 | 1516 | 489 | low_count |
| DPYS ---- tag |  | T/C | 1.04 (0.79-1.39) | 0.77 | . | . | . | 116 | 9,76 | 61 | 10,95 | 1516 | 489 |  |
| DPYS ---- tag |  | C/C | 0.00 (0.00-3E197) | 0.97 | . | . | . | 5 | 0,42 | 0 | 0,00 | 1516 | 489 |  |
| DPYS ---- NA | rs17245950 | T/T | 1.00 (.-.) | . | 0.46 | 0.96 | 0.62 | 918 | 77,21 | 433 | 77,74 | 1516 | 489 | low_count |
| DPYS ---- NA |  | T/A | 0.91 (0.72-1.13) | 0.39 | . | . | . | 257 | 21,61 | 119 | 21,36 | 1516 | 489 |  |
| DPYS ---- NA |  | A/A | 1.09 (0.40-2.97) | 0.87 | . | . | . | 14 | 1,18 | 5 | 0,90 | 1516 | 489 |  |
| DPYS ---- NA | rs2253336 | A/A | 1.00 (.-.) | . | 0.48 | 0.96 | 0.62 | 955 | 80,32 | 449 | 80,61 | 1516 | 489 | low_count |
| DPYS ---- NA |  | A/G | 0.95 (0.75-1.20) | 0.69 | . | . | . | 220 | 18,50 | 104 | 18,67 | 1516 | 489 |  |
| DPYS ---- NA |  | G/G | 0.62 (0.20-1.96) | 0.42 | . | . | . | 14 | 1,18 | 4 | 0,72 | 1516 | 489 |  |
| DPYS ---- tag | rs2280010 | C/C | 1.00 (.-.) | . | 0.02 | 0.73 | 0.48 | 700 | 58,87 | 288 | 51,71 | 1516 | 489 |  |
| DPYS ---- tag |  | C/T | 1.21 (1.00-1.46) | 0.05 | . | . | . | 432 | 36,33 | 233 | 41,83 | 1516 | 489 |  |
| DPYS ---- tag |  | T/T | 1.36 (0.94-1.95) | 0.10 | . | . | . | 57 | 4,79 | 36 | 6,46 | 1516 | 489 |  |
| DPYS ---- tag | rs2333874 | T/T | 1.00 (.-.) | . | 0.44 | 0.96 | 0.62 | 549 | 46,17 | 248 | 44,52 | 1516 | 489 |  |
| DPYS ---- tag |  | T/G | 1.00 (0.83-1.21) | 0.98 | . | . | . | 502 | 42,22 | 247 | 44,34 | 1516 | 489 |  |
| DPYS ---- tag |  | G/G | 1.17 (0.87-1.59) | 0.30 | . | . | . | 138 | 11,61 | 62 | 11,13 | 1516 | 489 |  |
| DPYS ---- NA | rs2669429 | C/C | 1.00 (.-.) | . | 0.35 | 0.96 | 0.59 | 379 | 31,88 | 156 | 28,01 | 1516 | 489 |  |
| DPYS ---- NA |  | C/T | 1.15 (0.93-1.42) | 0.19 | . | . | . | 604 | 50,80 | 290 | 52,06 | 1516 | 489 |  |
| DPYS ---- NA |  | T/T | 1.11 (0.86-1.45) | 0.42 | . | . | . | 206 | 17,33 | 111 | 19,93 | 1516 | 489 |  |
| DPYS ---- tag | rs2669434 | C/C | 1.00 (.-.) | . | 0.13 | 0.85 | 0.55 | 602 | 50,63 | 312 | 56,01 | 1516 | 489 |  |
| DPYS ---- tag |  | C/A | 0.80 (0.66-0.98) | 0.03 | . | . | . | 495 | 41,63 | 204 | 36,62 | 1516 | 489 |  |
| DPYS ---- tag |  | A/A | 0.96 (0.67-1.36) | 0.80 | . | . | . | 92 | 7,74 | 41 | 7,36 | 1516 | 489 |  |
| DPYS ---- tag | rs2853142 | T/T | 1.00 (.-.) | . | 0.20 | 0.92 | 0.55 | 441 | 37,09 | 226 | 40,57 | 1516 | 489 |  |
| DPYS ---- tag |  | T/C | 0.75 (0.61-0.90) | 0.00 | . | . | . | 591 | 49,71 | 255 | 45,78 | 1516 | 489 |  |
| DPYS ---- tag |  | C/C | 1.00 (0.75-1.32) | 0.98 | . | . | . | 157 | 13,20 | 76 | 13,64 | 1516 | 489 |  |
| DPYS ---- NA | rs2853145 | A/A | 1.00 (.-.) | . | 0.34 | 0.96 | 0.59 | 754 | 63,41 | 372 | 66,79 | 1516 | 489 |  |
| DPYS ---- NA |  | A/C | 0.90 (0.74-1.10) | 0.29 | . | . | . | 377 | 31,71 | 165 | 29,62 | 1516 | 489 |  |
| DPYS ---- NA |  | C/C | 0.94 (0.56-1.59) | 0.82 | . | . | . | 58 | 4,88 | 20 | 3,59 | 1516 | 489 |  |
| DPYS ---- tag | rs2853149 | G/G | 1.00 (.-.) | . | 0.12 | 0.80 | 0.55 | 338 | 28,43 | 154 | 27,65 | 1516 | 489 |  |
| DPYS ---- tag |  | G/A | 1.00 (0.81-1.24) | 0.99 | . | . | . | 603 | 50,71 | 284 | 50,99 | 1516 | 489 |  |
| DPYS ---- tag |  | A/A | 1.25 (0.97-1.62) | 0.09 | . | . | . | 248 | 20,86 | 119 | 21,36 | 1516 | 489 |  |
| DPYS ---- tag | rs2853154 | T/T | 1.00 (.-.) | . | 0.11 | 0.80 | 0.55 | 635 | 53,41 | 328 | 58,89 | 1516 | 489 |  |
| DPYS ---- tag |  | T/C | 0.81 (0.67-0.98) | 0.03 | . | . | . | 485 | 40,79 | 198 | 35,55 | 1516 | 489 |  |
| DPYS ---- tag |  | C/C | 0.95 (0.63-1.43) | 0.81 | . | . | . | 69 | 5,80 | 31 | 5,57 | 1516 | 489 |  |
| DPYS ---- tag | rs2853161 | A/A | 1.00 (.-.) | . | 0.38 | 0.96 | 0.60 | 301 | 25,32 | 139 | 24,96 | 1516 | 489 |  |
| DPYS ---- tag |  | A/G | 0.93 (0.75-1.16) | 0.54 | . | . | . | 616 | 51,81 | 294 | 52,78 | 1516 | 489 |  |
| DPYS ---- tag |  | G/G | 0.89 (0.68-1.16) | 0.38 | . | . | . | 272 | 22,88 | 124 | 22,26 | 1516 | 489 |  |
| DPYS ---- NA | rs2959024 | T/T | 1.00 (.-.) | . | 0.71 | 0.97 | 0.74 | 620 | 52,14 | 270 | 48,47 | 1516 | 489 |  |
| DPYS ---- NA |  | T/G | 1.07 (0.89-1.29) | 0.48 | . | . | . | 475 | 39,95 | 244 | 43,81 | 1516 | 489 |  |
| DPYS ---- NA |  | G/G | 0.99 (0.70-1.40) | 0.97 | . | . | . | 94 | 7,91 | 43 | 7,72 | 1516 | 489 |  |
| DPYS ---- NA | rs2959025 | A/A | 1.00 (.-.) | . | 0.70 | 0.97 | 0.74 | 507 | 42,64 | 208 | 37,34 | 1516 | 489 |  |
| DPYS ---- NA |  | A/G | 1.10 (0.91-1.34) | 0.34 | . | . | . | 524 | 44,07 | 273 | 49,01 | 1516 | 489 |  |
| DPYS ---- NA |  | G/G | 1.01 (0.76-1.33) | 0.97 | . | . | . | 158 | 13,29 | 76 | 13,64 | 1516 | 489 |  |
| DPYS ---- tag | rs2959026 | G/G | 1.00 (.-.) | . | 0.26 | 0.95 | 0.57 | 443 | 37,26 | 198 | 35,55 | 1516 | 489 |  |
| DPYS ---- tag |  | G/A | 1.13 (0.93-1.38) | 0.23 | . | . | . | 550 | 46,26 | 272 | 48,83 | 1516 | 489 |  |
| DPYS ---- tag |  | A/A | 1.14 (0.87-1.49) | 0.36 | . | . | . | 196 | 16,48 | 87 | 15,62 | 1516 | 489 |  |
| DPYS ---- NA | rs3133278 | T/T | 1.00 (.-.) | . | 0.26 | 0.95 | 0.57 | 578 | 48,61 | 286 | 51,35 | 1516 | 489 |  |
| DPYS ---- NA |  | T/C | 0.86 (0.72-1.04) | 0.13 | . | . | . | 515 | 43,31 | 229 | 41,11 | 1516 | 489 |  |
| DPYS ---- NA |  | C/C | 0.95 (0.67-1.35) | 0.76 | . | . | . | 96 | 8,07 | 42 | 7,54 | 1516 | 489 |  |
| DPYS ---- tag | rs3750187 | G/G | 1.00 (.-.) | . | 0.15 | 0.91 | 0.55 | 719 | 60,47 | 358 | 64,27 | 1516 | 489 |  |
| DPYS ---- tag |  | G/A | 0.83 (0.68-1.00) | 0.05 | . | . | . | 415 | 34,90 | 179 | 32,14 | 1516 | 489 |  |
| DPYS ---- tag |  | A/A | 1.03 (0.63-1.66) | 0.92 | . | . | . | 55 | 4,63 | 20 | 3,59 | 1516 | 489 |  |
| DPYS ---- tag | rs3793357 | T/T | 1.00 (.-.) | . | 0.68 | 0.97 | 0.74 | 1055 | 88,73 | 499 | 89,59 | 1516 | 489 | low_count |
| DPYS ---- tag |  | T/G | 1.03 (0.76-1.39) | 0.85 | . | . | . | 130 | 10,93 | 56 | 10,05 | 1516 | 489 |  |
| DPYS ---- tag |  | G/G | 1.68 (0.42-6.80) | 0.47 | . | . | . | 4 | 0,34 | 2 | 0,36 | 1516 | 489 |  |
| DPYS ---- tag | rs3793358 | G/G | 1.00 (.-.) | . | 0.19 | 0.92 | 0.55 | 877 | 73,76 | 434 | 77,92 | 1516 | 489 |  |
| DPYS ---- tag |  | G/A | 0.77 (0.61-0.97) | 0.02 | . | . | . | 292 | 24,56 | 109 | 19,57 | 1516 | 489 |  |
| DPYS ---- tag |  | A/A | 1.55 (0.85-2.83) | 0.16 | . | . | . | 20 | 1,68 | 14 | 2,51 | 1516 | 489 |  |
| DPYS ---- tag | rs6468924 | C/C | 1.00 (.-.) | . | 0.34 | 0.96 | 0.59 | 713 | 59,97 | 358 | 64,27 | 1516 | 489 |  |
| DPYS ---- tag |  | C/T | 0.84 (0.68-1.02) | 0.08 | . | . | . | 427 | 35,91 | 175 | 31,42 | 1516 | 489 |  |
| DPYS ---- tag |  | T/T | 1.15 (0.74-1.79) | 0.54 | . | . | . | 49 | 4,12 | 24 | 4,31 | 1516 | 489 |  |
| DUT ---- tag | rs8025164 | G/G | 1.00 (.-.) | . | 0.43 | 0.96 | 0.43 | 855 | 71,91 | 396 | 71,10 | 1516 | 489 |  |
| DUT ---- tag |  | G/A | 0.95 (0.77-1.18) | 0.67 | . | . | . | 301 | 25,32 | 142 | 25,49 | 1516 | 489 |  |
| DUT ---- tag |  | A/A | 1.71 (1.06-2.78) | 0.03 | . | . | . | 33 | 2,78 | 19 | 3,41 | 1516 | 489 |  |
| EHMT1 ---- tag | rs10780190 | C/C | 1.00 (.-.) | . | 0.48 | 0.96 | 0.92 | 1057 | 88,90 | 499 | 89,59 | 1516 | 489 | low_count |
| EHMT1 ---- tag |  | C/T | 1.04 (0.77-1.41) | 0.79 | . | . | . | 130 | 10,93 | 56 | 10,05 | 1516 | 489 |  |
| EHMT1 ---- tag |  | T/T | 4.13 (1.01-16.81) | 0.05 | . | . | . | 2 | 0,17 | 2 | 0,36 | 1516 | 489 |  |
| EHMT1 ---- tag | rs10867083 | G/G | 1.00 (.-.) | . | 0.63 | 0.96 | 0.92 | 536 | 45,08 | 252 | 45,24 | 1516 | 489 |  |
| EHMT1 ---- tag |  | G/A | 1.01 (0.84-1.22) | 0.92 | . | . | . | 534 | 44,91 | 249 | 44,70 | 1516 | 489 |  |
| EHMT1 ---- tag |  | A/A | 0.89 (0.64-1.22) | 0.46 | . | . | . | 119 | 10,01 | 56 | 10,05 | 1516 | 489 |  |
| EHMT1 ---- tag | rs11137190 | C/C | 1.00 (.-.) | . | 0.52 | 0.96 | 0.92 | 609 | 51,22 | 279 | 50,09 | 1516 | 489 |  |
| EHMT1 ---- tag |  | C/G | 1.16 (0.96-1.39) | 0.13 | . | . | . | 488 | 41,04 | 241 | 43,27 | 1516 | 489 |  |
| EHMT1 ---- tag |  | G/G | 0.92 (0.63-1.34) | 0.67 | . | . | . | 92 | 7,74 | 37 | 6,64 | 1516 | 489 |  |
| EHMT1 ---- tag | rs3123510 | G/G | 1.00 (.-.) | . | 0.67 | 0.96 | 0.92 | 415 | 34,90 | 193 | 34,65 | 1516 | 489 |  |
| EHMT1 ---- tag |  | G/A | 1.13 (0.93-1.37) | 0.23 | . | . | . | 563 | 47,35 | 284 | 50,99 | 1516 | 489 |  |
| EHMT1 ---- tag |  | A/A | 1.00 (0.75-1.33) | 0.98 | . | . | . | 211 | 17,75 | 80 | 14,36 | 1516 | 489 |  |
| EHMT1 ---- candidate literature | rs3125795 | G/G | 1.00 (.-.) | . | 0.43 | 0.96 | 0.92 | 1054 | 88,65 | 497 | 89,23 | 1516 | 489 | low_count |
| EHMT1 ---- candidate literature |  | G/T | 1.10 (0.82-1.48) | 0.52 | . | . | . | 132 | 11,10 | 59 | 10,59 | 1516 | 489 |  |
| EHMT1 ---- candidate literature |  | T/T | 2.15 (0.30-15.52) | 0.45 | . | . | . | 3 | 0,25 | 1 | 0,18 | 1516 | 489 |  |
| EHMT1 ---- tag | rs4573359 | G/G | 1.00 (.-.) | . | 0.93 | 0.98 | 0.93 | 986 | 82,93 | 461 | 82,76 | 1516 | 489 | low_count |
| EHMT1 ---- tag |  | G/T | 1.03 (0.81-1.31) | 0.82 | . | . | . | 195 | 16,40 | 94 | 16,88 | 1516 | 489 |  |
| EHMT1 ---- tag |  | T/T | 0.41 (0.06-2.96) | 0.38 | . | . | . | 8 | 0,67 | 2 | 0,36 | 1516 | 489 |  |
| EHMT1 ---- candidate literature | rs4634736 | G/G | 1.00 (.-.) | . | 0.84 | 0.98 | 0.93 | 985 | 82,84 | 462 | 82,94 | 1516 | 489 | low_count |
| EHMT1 ---- candidate literature |  | G/A | 1.01 (0.80-1.29) | 0.92 | . | . | . | 196 | 16,48 | 93 | 16,70 | 1516 | 489 |  |
| EHMT1 ---- candidate literature |  | A/A | 0.41 (0.06-2.95) | 0.38 | . | . | . | 8 | 0,67 | 2 | 0,36 | 1516 | 489 |  |
| EHMT1 ---- tag | rs4876902 | C/C | 1.00 (.-.) | . | 0.09 | 0.79 | 0.92 | 739 | 62,15 | 331 | 59,43 | 1516 | 489 |  |
| EHMT1 ---- tag |  | C/T | 1.15 (0.95-1.39) | 0.15 | . | . | . | 399 | 33,56 | 201 | 36,09 | 1516 | 489 |  |
| EHMT1 ---- tag |  | T/T | 1.28 (0.83-1.97) | 0.27 | . | . | . | 51 | 4,29 | 25 | 4,49 | 1516 | 489 |  |
| EHMT1 ---- tag | rs4876904 | T/T | 1.00 (.-.) | . | 0.44 | 0.96 | 0.92 | 351 | 29,52 | 173 | 31,06 | 1516 | 489 |  |
| EHMT1 ---- tag |  | T/G | 1.02 (0.83-1.26) | 0.84 | . | . | . | 598 | 50,29 | 266 | 47,76 | 1516 | 489 |  |
| EHMT1 ---- tag |  | G/G | 0.90 (0.70-1.15) | 0.40 | . | . | . | 240 | 20,19 | 118 | 21,18 | 1516 | 489 |  |
| EHMT1 ---- tag | rs7390244 | G/G | 1.00 (.-.) | . | 0.90 | 0.98 | 0.93 | 283 | 23,80 | 152 | 27,29 | 1516 | 489 |  |
| EHMT1 ---- tag |  | G/A | 1.01 (0.82-1.26) | 0.90 | . | . | . | 603 | 50,71 | 262 | 47,04 | 1516 | 489 |  |
| EHMT1 ---- tag |  | A/A | 0.98 (0.77-1.26) | 0.90 | . | . | . | 303 | 25,48 | 143 | 25,67 | 1516 | 489 |  |
| EHMT1 ---- tag | rs9314635 | G/G | 1.00 (.-.) | . | 0.29 | 0.95 | 0.92 | 483 | 40,62 | 207 | 37,16 | 1516 | 489 |  |
| EHMT1 ---- tag |  | G/T | 1.22 (1.00-1.47) | 0.05 | . | . | . | 543 | 45,67 | 284 | 50,99 | 1516 | 489 |  |
| EHMT1 ---- tag |  | T/T | 1.04 (0.77-1.41) | 0.79 | . | . | . | 163 | 13,71 | 66 | 11,85 | 1516 | 489 |  |
| EHMT2 ---- candidate/tag | rs2736428 | G/G | 1.00 (.-.) | . | 0.61 | 0.96 | 0.61 | 478 | 40,20 | 238 | 42,73 | 1516 | 489 |  |
| EHMT2 ---- candidate/tag |  | G/A | 0.88 (0.73-1.07) | 0.21 | . | . | . | 555 | 46,68 | 243 | 43,63 | 1516 | 489 |  |
| EHMT2 ---- candidate/tag |  | A/A | 1.00 (0.76-1.33) | 0.99 | . | . | . | 156 | 13,12 | 76 | 13,64 | 1516 | 489 |  |
| EHMT2 ---- tag | rs9267649 | G/G | 1.00 (.-.) | . | 0.21 | 0.92 | 0.41 | 842 | 70,82 | 402 | 72,17 | 1516 | 489 |  |
| EHMT2 ---- tag |  | G/A | 0.88 (0.72-1.09) | 0.24 | . | . | . | 311 | 26,16 | 140 | 25,13 | 1516 | 489 |  |
| EHMT2 ---- tag |  | A/A | 0.83 (0.47-1.49) | 0.54 | . | . | . | 36 | 3,03 | 15 | 2,69 | 1516 | 489 |  |
| FDXR ---- NA | rs2070918 | T/T | 1.00 (.-.) | . | 0.13 | 0.86 | 0.53 | 548 | 46,09 | 260 | 46,68 | 1516 | 489 |  |
| FDXR ---- NA |  | T/C | 1.09 (0.90-1.32) | 0.39 | . | . | . | 502 | 42,22 | 224 | 40,22 | 1516 | 489 |  |
| FDXR ---- NA |  | C/C | 1.24 (0.93-1.63) | 0.14 | . | . | . | 139 | 11,69 | 73 | 13,11 | 1516 | 489 |  |
| FDXR ---- tag | rs509911 | A/A | 1.00 (.-.) | . | 0.57 | 0.96 | 0.70 | 725 | 60,98 | 350 | 62,84 | 1516 | 489 |  |
| FDXR ---- tag |  | A/G | 1.08 (0.89-1.31) | 0.45 | . | . | . | 401 | 33,73 | 181 | 32,50 | 1516 | 489 |  |
| FDXR ---- tag |  | G/G | 1.02 (0.67-1.55) | 0.93 | . | . | . | 63 | 5,30 | 26 | 4,67 | 1516 | 489 |  |
| FDXR ---- NA | rs689882 | G/G | 1.00 (.-.) | . | 0.63 | 0.96 | 0.70 | 602 | 50,63 | 301 | 54,04 | 1516 | 489 |  |
| FDXR ---- NA |  | G/A | 0.96 (0.79-1.17) | 0.71 | . | . | . | 487 | 40,96 | 210 | 37,70 | 1516 | 489 |  |
| FDXR ---- NA |  | A/A | 0.94 (0.68-1.30) | 0.70 | . | . | . | 100 | 8,41 | 46 | 8,26 | 1516 | 489 |  |
| FDXR ---- NA | rs689895 | G/G | 1.00 (.-.) | . | 0.70 | 0.97 | 0.70 | 583 | 49,03 | 283 | 50,81 | 1516 | 489 |  |
| FDXR ---- NA |  | G/C | 0.96 (0.80-1.17) | 0.70 | . | . | . | 499 | 41,97 | 224 | 40,22 | 1516 | 489 |  |
| FDXR ---- NA |  | C/C | 0.96 (0.69-1.33) | 0.80 | . | . | . | 107 | 9,00 | 50 | 8,98 | 1516 | 489 |  |
| FOLH1 ---- candidate literature | rs10839236 | T/T | 1.00 (.-.) | . | 0.98 | 0.99 | 0.98 | 471 | 39,61 | 213 | 38,24 | 1516 | 489 |  |
| FOLH1 ---- candidate literature |  | T/C | 1.12 (0.93-1.36) | 0.24 | . | . | . | 561 | 47,18 | 273 | 49,01 | 1516 | 489 |  |
| FOLH1 ---- candidate literature |  | C/C | 0.93 (0.70-1.24) | 0.62 | . | . | . | 157 | 13,20 | 71 | 12,75 | 1516 | 489 |  |
| FOLH1 ---- tag | rs16906190 | A/A | 1.00 (.-.) | . | 0.92 | 0.98 | 0.98 | 985 | 82,84 | 471 | 84,56 | 1516 | 489 | low_count |
| FOLH1 ---- tag |  | A/G | 1.09 (0.84-1.41) | 0.54 | . | . | . | 191 | 16,06 | 81 | 14,54 | 1516 | 489 |  |
| FOLH1 ---- tag |  | G/G | 0.67 (0.25-1.80) | 0.43 | . | . | . | 13 | 1,09 | 5 | 0,90 | 1516 | 489 |  |
| FOLH1 ---- candidate | rs202676 | T/T | 1.00 (.-.) | . | 0.77 | 0.97 | 0.98 | 737 | 61,98 | 349 | 62,66 | 1516 | 489 |  |
| FOLH1 ---- candidate |  | T/C | 0.95 (0.78-1.16) | 0.62 | . | . | . | 399 | 33,56 | 182 | 32,68 | 1516 | 489 |  |
| FOLH1 ---- candidate |  | C/C | 1.02 (0.67-1.57) | 0.92 | . | . | . | 53 | 4,46 | 26 | 4,67 | 1516 | 489 |  |
| FOLH1 ---- tag | rs202680 | A/A | 1.00 (.-.) | . | 0.75 | 0.97 | 0.98 | 650 | 54,67 | 314 | 56,37 | 1516 | 489 |  |
| FOLH1 ---- tag |  | A/T | 0.95 (0.79-1.15) | 0.63 | . | . | . | 474 | 39,87 | 206 | 36,98 | 1516 | 489 |  |
| FOLH1 ---- tag |  | T/T | 0.99 (0.69-1.44) | 0.97 | . | . | . | 65 | 5,47 | 37 | 6,64 | 1516 | 489 |  |
| FOLH1 ---- candidate literature | rs202720 | G/G | 1.00 (.-.) | . | 0.72 | 0.97 | 0.98 | 735 | 61,82 | 349 | 62,66 | 1516 | 489 |  |
| FOLH1 ---- candidate literature |  | G/C | 0.94 (0.78-1.15) | 0.56 | . | . | . | 401 | 33,73 | 182 | 32,68 | 1516 | 489 |  |
| FOLH1 ---- candidate literature |  | C/C | 1.02 (0.67-1.56) | 0.93 | . | . | . | 53 | 4,46 | 26 | 4,67 | 1516 | 489 |  |
| FOLH1 ---- tag | rs2299650 | G/G | 1.00 (.-.) | . | 0.91 | 0.98 | 0.98 | 464 | 39,02 | 213 | 38,24 | 1516 | 489 |  |
| FOLH1 ---- tag |  | G/T | 1.11 (0.92-1.35) | 0.28 | . | . | . | 567 | 47,69 | 275 | 49,37 | 1516 | 489 |  |
| FOLH1 ---- tag |  | T/T | 0.91 (0.68-1.22) | 0.53 | . | . | . | 158 | 13,29 | 69 | 12,39 | 1516 | 489 |  |
| FOLH1 ---- tag | rs617528 | G/G | 1.00 (.-.) | . | 0.65 | 0.96 | 0.98 | 933 | 78,47 | 430 | 77,20 | 1516 | 489 | low_count |
| FOLH1 ---- tag |  | G/A | 1.14 (0.92-1.42) | 0.22 | . | . | . | 237 | 19,93 | 118 | 21,18 | 1516 | 489 |  |
| FOLH1 ---- tag |  | A/A | 0.74 (0.38-1.44) | 0.37 | . | . | . | 19 | 1,60 | 9 | 1,62 | 1516 | 489 |  |
| FOLH1 ---- tag | rs663877 | T/T | 1.00 (.-.) | . | 0.66 | 0.96 | 0.98 | 914 | 76,87 | 426 | 76,48 | 1516 | 489 |  |
| FOLH1 ---- tag |  | T/G | 0.87 (0.70-1.08) | 0.21 | . | . | . | 262 | 22,04 | 120 | 21,54 | 1516 | 489 |  |
| FOLH1 ---- tag |  | G/G | 1.66 (0.88-3.16) | 0.12 | . | . | . | 13 | 1,09 | 11 | 1,97 | 1516 | 489 |  |
| FOLH1 ---- tag | rs670776 | A/A | 1.00 (.-.) | . | 0.77 | 0.97 | 0.98 | 737 | 61,98 | 349 | 62,66 | 1516 | 489 |  |
| FOLH1 ---- tag |  | A/T | 0.95 (0.78-1.16) | 0.62 | . | . | . | 399 | 33,56 | 182 | 32,68 | 1516 | 489 |  |
| FOLH1 ---- tag |  | T/T | 1.02 (0.67-1.57) | 0.92 | . | . | . | 53 | 4,46 | 26 | 4,67 | 1516 | 489 |  |
| FOLH1 ---- tag | rs7124497 | G/G | 1.00 (.-.) | . | 0.90 | 0.98 | 0.98 | 1090 | 91,67 | 512 | 91,92 | 1516 | 489 | low_count |
| FOLH1 ---- tag |  | G/A | 0.94 (0.67-1.32) | 0.71 | . | . | . | 98 | 8,24 | 44 | 7,90 | 1516 | 489 |  |
| FOLH1 ---- tag |  | A/A | 4.57 (0.63-33.19) | 0.13 | . | . | . | 1 | 0,08 | 1 | 0,18 | 1516 | 489 |  |
| FOLR1 ---- tag | rs651646 | T/T | 1.00 (.-.) | . | 0.54 | 0.96 | 0.54 | 362 | 30,45 | 180 | 32,32 | 1516 | 489 |  |
| FOLR1 ---- tag |  | T/A | 0.98 (0.80-1.20) | 0.83 | . | . | . | 600 | 50,46 | 265 | 47,58 | 1516 | 489 |  |
| FOLR1 ---- tag |  | A/A | 0.92 (0.71-1.19) | 0.53 | . | . | . | 227 | 19,09 | 112 | 20,11 | 1516 | 489 |  |
| FPGS ---- tag | rs10987746 | T/T | 1.00 (.-.) | . | 0.88 | 0.98 | 0.88 | 344 | 28,93 | 153 | 27,47 | 1516 | 489 |  |
| FPGS ---- tag |  | T/C | 0.91 (0.74-1.13) | 0.39 | . | . | . | 599 | 50,38 | 280 | 50,27 | 1516 | 489 |  |
| FPGS ---- tag |  | C/C | 0.99 (0.77-1.28) | 0.95 | . | . | . | 246 | 20,69 | 124 | 22,26 | 1516 | 489 |  |
| FPGS ---- tag | rs7033913 | T/T | 1.00 (.-.) | . | 0.56 | 0.96 | 0.88 | 371 | 31,20 | 181 | 32,50 | 1516 | 489 |  |
| FPGS ---- tag |  | T/C | 0.97 (0.79-1.18) | 0.73 | . | . | . | 572 | 48,11 | 281 | 50,45 | 1516 | 489 |  |
| FPGS ---- tag |  | C/C | 0.92 (0.71-1.21) | 0.56 | . | . | . | 246 | 20,69 | 95 | 17,06 | 1516 | 489 |  |
| FPGS ---- tag | rs7039798 | G/G | 1.00 (.-.) | . | 0.76 | 0.97 | 0.88 | 390 | 32,80 | 167 | 29,98 | 1516 | 489 |  |
| FPGS ---- tag |  | G/A | 0.93 (0.76-1.14) | 0.48 | . | . | . | 580 | 48,78 | 286 | 51,35 | 1516 | 489 |  |
| FPGS ---- tag |  | A/A | 0.98 (0.75-1.27) | 0.85 | . | . | . | 219 | 18,42 | 104 | 18,67 | 1516 | 489 |  |
| GGH ---- tag | rs10957264 | G/G | 1.00 (.-.) | . | 0.71 | 0.97 | 0.77 | 849 | 71,40 | 384 | 68,94 | 1516 | 489 |  |
| GGH ---- tag |  | G/T | 1.15 (0.95-1.41) | 0.16 | . | . | . | 304 | 25,57 | 162 | 29,08 | 1516 | 489 |  |
| GGH ---- tag |  | T/T | 0.64 (0.33-1.25) | 0.19 | . | . | . | 36 | 3,03 | 11 | 1,97 | 1516 | 489 |  |
| GGH ---- candidate literature | rs11545076 | T/T | 1.00 (.-.) | . | 0.12 | 0.80 | 0.26 | 597 | 50,21 | 270 | 48,47 | 1516 | 489 |  |
| GGH ---- candidate literature |  | T/G | 0.97 (0.80-1.16) | 0.71 | . | . | . | 487 | 40,96 | 248 | 44,52 | 1516 | 489 |  |
| GGH ---- candidate literature |  | G/G | 0.70 (0.48-1.01) | 0.06 | . | . | . | 105 | 8,83 | 39 | 7,00 | 1516 | 489 |  |
| GGH ---- candidate | rs11545077 | G/G | 1.00 (.-.) | . | 0.39 | 0.96 | 0.63 | 646 | 54,33 | 295 | 52,96 | 1516 | 489 |  |
| GGH ---- candidate |  | G/A | 1.05 (0.87-1.26) | 0.62 | . | . | . | 460 | 38,69 | 232 | 41,65 | 1516 | 489 |  |
| GGH ---- candidate |  | A/A | 0.70 (0.47-1.05) | 0.08 | . | . | . | 83 | 6,98 | 30 | 5,39 | 1516 | 489 |  |
| GGH ---- candidate | rs11545078 | C/C | 1.00 (.-.) | . | 0.63 | 0.96 | 0.75 | 991 | 83,35 | 447 | 80,25 | 1516 | 489 | low_count |
| GGH ---- candidate |  | C/T | 1.19 (0.94-1.51) | 0.14 | . | . | . | 182 | 15,31 | 104 | 18,67 | 1516 | 489 |  |
| GGH ---- candidate |  | T/T | 0.62 (0.28-1.39) | 0.25 | . | . | . | 16 | 1,35 | 6 | 1,08 | 1516 | 489 |  |
| GGH ---- tag | rs11995525 | G/G | 1.00 (.-.) | . | 0.03 | 0.73 | 0.14 | 650 | 54,67 | 282 | 50,63 | 1516 | 489 |  |
| GGH ---- tag |  | G/A | 1.17 (0.97-1.41) | 0.10 | . | . | . | 460 | 38,69 | 232 | 41,65 | 1516 | 489 |  |
| GGH ---- tag |  | A/A | 1.38 (0.98-1.94) | 0.07 | . | . | . | 79 | 6,64 | 43 | 7,72 | 1516 | 489 |  |
| GGH ---- tag | rs16930073 | G/G | 1.00 (.-.) | . | 0.03 | 0.73 | 0.14 | 940 | 79,06 | 456 | 81,87 | 1516 | 489 | low_count |
| GGH ---- tag |  | G/A | 0.89 (0.70-1.13) | 0.33 | . | . | . | 229 | 19,26 | 96 | 17,24 | 1516 | 489 |  |
| GGH ---- tag |  | A/A | 0.35 (0.14-0.87) | 0.02 | . | . | . | 20 | 1,68 | 5 | 0,90 | 1516 | 489 |  |
| GGH ---- tag | rs17194931 | G/G | 1.00 (.-.) | . | 0.63 | 0.96 | 0.75 | 991 | 83,35 | 447 | 80,25 | 1516 | 489 | low_count |
| GGH ---- tag |  | G/A | 1.19 (0.94-1.51) | 0.14 | . | . | . | 182 | 15,31 | 104 | 18,67 | 1516 | 489 |  |
| GGH ---- tag |  | A/A | 0.62 (0.28-1.39) | 0.25 | . | . | . | 16 | 1,35 | 6 | 1,08 | 1516 | 489 |  |
| GGH ---- candidate literature | rs1800909 | T/T | 1.00 (.-.) | . | 0.09 | 0.79 | 0.26 | 593 | 49,87 | 269 | 48,29 | 1516 | 489 |  |
| GGH ---- candidate literature |  | T/C | 0.95 (0.79-1.14) | 0.57 | . | . | . | 494 | 41,55 | 249 | 44,70 | 1516 | 489 |  |
| GGH ---- candidate literature |  | C/C | 0.69 (0.48-1.00) | 0.05 | . | . | . | 102 | 8,58 | 39 | 7,00 | 1516 | 489 |  |
| GGH ---- candidate literature | rs3758149 | C/C | 1.00 (.-.) | . | 0.12 | 0.80 | 0.26 | 597 | 50,21 | 270 | 48,47 | 1516 | 489 |  |
| GGH ---- candidate literature |  | C/T | 0.97 (0.80-1.16) | 0.71 | . | . | . | 487 | 40,96 | 248 | 44,52 | 1516 | 489 |  |
| GGH ---- candidate literature |  | T/T | 0.70 (0.48-1.01) | 0.06 | . | . | . | 105 | 8,83 | 39 | 7,00 | 1516 | 489 |  |
| GGH ---- tag | rs3780130 | A/A | 1.00 (.-.) | . | 0.01 | 0.73 | 0.07 | 730 | 61,40 | 369 | 66,25 | 1516 | 489 |  |
| GGH ---- tag |  | A/T | 0.79 (0.64-0.96) | 0.02 | . | . | . | 406 | 34,15 | 170 | 30,52 | 1516 | 489 |  |
| GGH ---- tag |  | T/T | 0.63 (0.38-1.06) | 0.08 | . | . | . | 53 | 4,46 | 18 | 3,23 | 1516 | 489 |  |
| GGH ---- tag | rs4446729 | C/C | 1.00 (.-.) | . | 0.91 | 0.98 | 0.91 | 625 | 52,57 | 295 | 52,96 | 1516 | 489 |  |
| GGH ---- tag |  | C/T | 1.02 (0.85-1.23) | 0.81 | . | . | . | 476 | 40,03 | 223 | 40,04 | 1516 | 489 |  |
| GGH ---- tag |  | T/T | 0.99 (0.69-1.43) | 0.96 | . | . | . | 88 | 7,40 | 39 | 7,00 | 1516 | 489 |  |
| GGH ---- tag | rs6472067 | C/C | 1.00 (.-.) | . | 0.15 | 0.90 | 0.27 | 482 | 40,54 | 213 | 38,24 | 1516 | 489 |  |
| GGH ---- tag |  | C/G | 1.17 (0.96-1.42) | 0.11 | . | . | . | 544 | 45,75 | 266 | 47,76 | 1516 | 489 |  |
| GGH ---- tag |  | G/G | 1.16 (0.88-1.54) | 0.30 | . | . | . | 163 | 13,71 | 78 | 14,00 | 1516 | 489 |  |
| GGH ---- tag | rs7010484 | T/T | 1.00 (.-.) | . | 0.47 | 0.96 | 0.68 | 539 | 45,33 | 248 | 44,52 | 1516 | 489 |  |
| GGH ---- tag |  | T/C | 1.20 (0.99-1.45) | 0.06 | . | . | . | 511 | 42,98 | 248 | 44,52 | 1516 | 489 |  |
| GGH ---- tag |  | C/C | 0.97 (0.71-1.32) | 0.85 | . | . | . | 139 | 11,69 | 61 | 10,95 | 1516 | 489 |  |
| GNMT ---- tag | rs1053538 | C/C | 1.00 (.-.) | . | 0.12 | 0.80 | 0.41 | 326 | 27,42 | 156 | 28,01 | 1516 | 489 |  |
| GNMT ---- tag |  | C/G | 0.95 (0.76-1.17) | 0.62 | . | . | . | 587 | 49,37 | 251 | 45,06 | 1516 | 489 |  |
| GNMT ---- tag |  | G/G | 1.24 (0.97-1.58) | 0.09 | . | . | . | 276 | 23,21 | 150 | 26,93 | 1516 | 489 |  |
| GNMT ---- tag | rs2296805 | G/G | 1.00 (.-.) | . | 0.30 | 0.95 | 0.41 | 356 | 29,94 | 190 | 34,11 | 1516 | 489 |  |
| GNMT ---- tag |  | G/T | 0.81 (0.66-0.99) | 0.04 | . | . | . | 610 | 51,30 | 254 | 45,60 | 1516 | 489 |  |
| GNMT ---- tag |  | T/T | 0.91 (0.70-1.17) | 0.45 | . | . | . | 223 | 18,76 | 113 | 20,29 | 1516 | 489 |  |
| GNMT ---- tag | rs6901782 | T/T | 1.00 (.-.) | . | 0.45 | 0.96 | 0.45 | 902 | 75,86 | 432 | 77,56 | 1516 | 489 |  |
| GNMT ---- tag |  | T/C | 1.07 (0.86-1.34) | 0.53 | . | . | . | 263 | 22,12 | 113 | 20,29 | 1516 | 489 |  |
| GNMT ---- tag |  | C/C | 1.16 (0.63-2.12) | 0.63 | . | . | . | 24 | 2,02 | 12 | 2,15 | 1516 | 489 |  |
| GNMT ---- tag | rs6927188 | A/A | 1.00 (.-.) | . | 0.27 | 0.95 | 0.41 | 695 | 58,45 | 310 | 55,66 | 1516 | 489 |  |
| GNMT ---- tag |  | A/G | 1.02 (0.84-1.24) | 0.84 | . | . | . | 418 | 35,16 | 206 | 36,98 | 1516 | 489 |  |
| GNMT ---- tag |  | G/G | 1.33 (0.93-1.91) | 0.12 | . | . | . | 76 | 6,39 | 41 | 7,36 | 1516 | 489 |  |
| MAT1A ---- tag | rs10887708 | G/G | 1.00 (.-.) | . | 0.17 | 0.91 | 0.36 | 582 | 48,95 | 295 | 52,96 | 1516 | 489 |  |
| MAT1A ---- tag |  | G/A | 0.98 (0.81-1.19) | 0.87 | . | . | . | 484 | 40,71 | 218 | 39,14 | 1516 | 489 |  |
| MAT1A ---- tag |  | A/A | 0.73 (0.52-1.04) | 0.08 | . | . | . | 123 | 10,34 | 44 | 7,90 | 1516 | 489 |  |
| MAT1A ---- tag | rs10887718 | T/T | 1.00 (.-.) | . | 0.10 | 0.80 | 0.29 | 340 | 28,60 | 151 | 27,11 | 1516 | 489 |  |
| MAT1A ---- tag |  | T/C | 1.23 (0.99-1.53) | 0.06 | . | . | . | 579 | 48,70 | 271 | 48,65 | 1516 | 489 |  |
| MAT1A ---- tag |  | C/C | 1.23 (0.96-1.57) | 0.11 | . | . | . | 270 | 22,71 | 135 | 24,24 | 1516 | 489 |  |
| MAT1A ---- tag | rs11202403 | C/C | 1.00 (.-.) | . | 0.83 | 0.98 | 0.83 | 774 | 65,10 | 360 | 64,63 | 1516 | 489 |  |
| MAT1A ---- tag |  | C/T | 1.04 (0.86-1.27) | 0.66 | . | . | . | 361 | 30,36 | 180 | 32,32 | 1516 | 489 |  |
| MAT1A ---- tag |  | T/T | 0.79 (0.48-1.32) | 0.37 | . | . | . | 54 | 4,54 | 17 | 3,05 | 1516 | 489 |  |
| MAT1A ---- tag | rs1832683 | C/C | 1.00 (.-.) | . | 0.48 | 0.96 | 0.62 | 826 | 69,47 | 382 | 68,58 | 1516 | 489 |  |
| MAT1A ---- tag |  | C/T | 0.93 (0.76-1.14) | 0.47 | . | . | . | 321 | 27,00 | 154 | 27,65 | 1516 | 489 |  |
| MAT1A ---- tag |  | T/T | 0.93 (0.58-1.50) | 0.77 | . | . | . | 42 | 3,53 | 21 | 3,77 | 1516 | 489 |  |
| MAT1A ---- tag | rs2236568 | C/C | 1.00 (.-.) | . | 0.02 | 0.73 | 0.12 | 374 | 31,46 | 187 | 33,57 | 1516 | 489 |  |
| MAT1A ---- tag |  | C/A | 0.98 (0.80-1.20) | 0.87 | . | . | . | 564 | 47,43 | 279 | 50,09 | 1516 | 489 |  |
| MAT1A ---- tag |  | A/A | 0.70 (0.53-0.92) | 0.01 | . | . | . | 251 | 21,11 | 91 | 16,34 | 1516 | 489 |  |
| MAT1A ---- tag | rs2236569 | A/A | 1.00 (.-.) | . | 0.03 | 0.73 | 0.12 | 540 | 45,42 | 227 | 40,75 | 1516 | 489 |  |
| MAT1A ---- tag |  | A/G | 1.33 (1.10-1.61) | 0.00 | . | . | . | 523 | 43,99 | 262 | 47,04 | 1516 | 489 |  |
| MAT1A ---- tag |  | G/G | 1.20 (0.90-1.62) | 0.22 | . | . | . | 126 | 10,60 | 68 | 12,21 | 1516 | 489 |  |
| MAT1A ---- tag | rs9421467 | G/G | 1.00 (.-.) | . | 0.60 | 0.96 | 0.68 | 1060 | 89,15 | 504 | 90,48 | 1516 | 489 | low_count |
| MAT1A ---- tag |  | G/C | 1.02 (0.77-1.37) | 0.87 | . | . | . | 122 | 10,26 | 53 | 9,52 | 1516 | 489 |  |
| MAT1A ---- tag |  | C/C | 0.00 (0.00-1E204) | 0.96 | . | . | . | 7 | 0,59 | 0 | 0,00 | 1516 | 489 |  |
| MAT1A ---- tag | rs998765 | A/A | 1.00 (.-.) | . | 0.20 | 0.92 | 0.36 | 301 | 25,32 | 149 | 26,75 | 1516 | 489 |  |
| MAT1A ---- tag |  | A/T | 1.08 (0.87-1.34) | 0.49 | . | . | . | 578 | 48,61 | 290 | 52,06 | 1516 | 489 |  |
| MAT1A ---- tag |  | T/T | 0.84 (0.64-1.09) | 0.18 | . | . | . | 310 | 26,07 | 118 | 21,18 | 1516 | 489 |  |
| MAT1A ---- tag | rs998766 | C/C | 1.00 (.-.) | . | 0.28 | 0.95 | 0.43 | 370 | 31,12 | 179 | 32,14 | 1516 | 489 |  |
| MAT1A ---- tag |  | C/G | 1.07 (0.87-1.31) | 0.53 | . | . | . | 575 | 48,36 | 284 | 50,99 | 1516 | 489 |  |
| MAT1A ---- tag |  | G/G | 0.83 (0.63-1.09) | 0.18 | . | . | . | 244 | 20,52 | 94 | 16,88 | 1516 | 489 |  |
| MAT2B ---- tag | rs12655857 | G/G | 1.00 (.-.) | . | 0.53 | 0.96 | 0.96 | 672 | 56,52 | 305 | 54,76 | 1516 | 489 |  |
| MAT2B ---- tag |  | G/T | 1.03 (0.85-1.25) | 0.74 | . | . | . | 437 | 36,75 | 213 | 38,24 | 1516 | 489 |  |
| MAT2B ---- tag |  | T/T | 1.12 (0.79-1.60) | 0.52 | . | . | . | 80 | 6,73 | 39 | 7,00 | 1516 | 489 |  |
| MAT2B ---- tag | rs6869277 | C/C | 1.00 (.-.) | . | 0.95 | 0.99 | 0.96 | 933 | 78,47 | 436 | 78,28 | 1516 | 489 | low_count |
| MAT2B ---- tag |  | C/T | 0.99 (0.79-1.24) | 0.91 | . | . | . | 238 | 20,02 | 116 | 20,83 | 1516 | 489 |  |
| MAT2B ---- tag |  | T/T | 1.04 (0.42-2.53) | 0.94 | . | . | . | 18 | 1,51 | 5 | 0,90 | 1516 | 489 |  |
| MAT2B ---- tag | rs6874065 | A/A | 1.00 (.-.) | . | 0.63 | 0.96 | 0.96 | 328 | 27,59 | 153 | 27,47 | 1516 | 489 |  |
| MAT2B ---- tag |  | A/G | 1.08 (0.87-1.34) | 0.47 | . | . | . | 607 | 51,05 | 284 | 50,99 | 1516 | 489 |  |
| MAT2B ---- tag |  | G/G | 0.92 (0.71-1.20) | 0.56 | . | . | . | 254 | 21,36 | 120 | 21,54 | 1516 | 489 |  |
| MAT2B ---- tag | rs6882306 | T/T | 1.00 (.-.) | . | 0.06 | 0.77 | 0.29 | 793 | 66,69 | 369 | 66,25 | 1516 | 489 |  |
| MAT2B ---- tag |  | T/C | 1.28 (1.06-1.56) | 0.01 | . | . | . | 362 | 30,45 | 175 | 31,42 | 1516 | 489 |  |
| MAT2B ---- tag |  | C/C | 0.91 (0.48-1.72) | 0.76 | . | . | . | 34 | 2,86 | 13 | 2,33 | 1516 | 489 |  |
| MAT2B ---- tag | rs7721639 | T/T | 1.00 (.-.) | . | 0.96 | 0.99 | 0.96 | 832 | 69,97 | 397 | 71,27 | 1516 | 489 |  |
| MAT2B ---- tag |  | T/G | 1.07 (0.87-1.31) | 0.52 | . | . | . | 322 | 27,08 | 148 | 26,57 | 1516 | 489 |  |
| MAT2B ---- tag |  | G/G | 0.76 (0.40-1.44) | 0.40 | . | . | . | 35 | 2,94 | 12 | 2,15 | 1516 | 489 |  |
| MTHFD1 ---- tag | rs1256148 | G/G | 1.00 (.-.) | . | 0.33 | 0.96 | 0.65 | 708 | 59,55 | 324 | 58,17 | 1516 | 489 |  |
| MTHFD1 ---- tag |  | G/A | 1.05 (0.87-1.27) | 0.63 | . | . | . | 423 | 35,58 | 199 | 35,73 | 1516 | 489 |  |
| MTHFD1 ---- tag |  | A/A | 1.22 (0.84-1.76) | 0.30 | . | . | . | 58 | 4,88 | 34 | 6,10 | 1516 | 489 |  |
| MTHFD1 ---- tag | rs13329053 | T/T | 1.00 (.-.) | . | 0.58 | 0.96 | 0.75 | 366 | 30,78 | 182 | 32,68 | 1516 | 489 |  |
| MTHFD1 ---- tag |  | T/C | 0.97 (0.79-1.19) | 0.79 | . | . | . | 580 | 48,78 | 257 | 46,14 | 1516 | 489 |  |
| MTHFD1 ---- tag |  | C/C | 1.09 (0.85-1.41) | 0.50 | . | . | . | 243 | 20,44 | 118 | 21,18 | 1516 | 489 |  |
| MTHFD1 ---- candidate literature | rs2236224 | C/C | 1.00 (.-.) | . | 0.20 | 0.92 | 0.61 | 458 | 38,52 | 221 | 39,68 | 1516 | 489 |  |
| MTHFD1 ---- candidate literature |  | C/T | 1.01 (0.83-1.24) | 0.89 | . | . | . | 549 | 46,17 | 247 | 44,34 | 1516 | 489 |  |
| MTHFD1 ---- candidate literature |  | T/T | 1.23 (0.94-1.60) | 0.13 | . | . | . | 182 | 15,31 | 89 | 15,98 | 1516 | 489 |  |
| MTHFD1 ---- candidate | rs2236225 | C/C | 1.00 (.-.) | . | 0.72 | 0.97 | 0.75 | 370 | 31,12 | 188 | 33,75 | 1516 | 489 |  |
| MTHFD1 ---- candidate |  | C/T | 0.96 (0.79-1.18) | 0.73 | . | . | . | 582 | 48,95 | 260 | 46,68 | 1516 | 489 |  |
| MTHFD1 ---- candidate |  | T/T | 1.07 (0.83-1.38) | 0.62 | . | . | . | 237 | 19,93 | 109 | 19,57 | 1516 | 489 |  |
| MTHFD1 ---- tag | rs2281603 | A/A | 1.00 (.-.) | . | 0.75 | 0.97 | 0.75 | 699 | 58,79 | 322 | 57,81 | 1516 | 489 |  |
| MTHFD1 ---- tag |  | A/G | 0.94 (0.78-1.13) | 0.51 | . | . | . | 433 | 36,42 | 204 | 36,62 | 1516 | 489 |  |
| MTHFD1 ---- tag |  | G/G | 1.04 (0.71-1.52) | 0.85 | . | . | . | 57 | 4,79 | 31 | 5,57 | 1516 | 489 |  |
| MTHFD1 ---- candidate literature | rs8003379 | A/A | 1.00 (.-.) | . | 0.08 | 0.79 | 0.50 | 667 | 56,10 | 316 | 56,73 | 1516 | 489 |  |
| MTHFD1 ---- candidate literature |  | A/C | 1.16 (0.96-1.41) | 0.11 | . | . | . | 433 | 36,42 | 206 | 36,98 | 1516 | 489 |  |
| MTHFD1 ---- candidate literature |  | C/C | 1.23 (0.85-1.80) | 0.28 | . | . | . | 89 | 7,49 | 35 | 6,28 | 1516 | 489 |  |
| MTHFD2 ---- tag | rs10177833 | A/A | 1.00 (.-.) | . | 0.19 | 0.92 | 0.51 | 379 | 31,88 | 150 | 26,93 | 1516 | 489 |  |
| MTHFD2 ---- tag |  | A/C | 1.21 (0.99-1.50) | 0.07 | . | . | . | 589 | 49,54 | 309 | 55,48 | 1516 | 489 |  |
| MTHFD2 ---- tag |  | C/C | 1.16 (0.88-1.53) | 0.29 | . | . | . | 221 | 18,59 | 98 | 17,59 | 1516 | 489 |  |
| MTHFD2 ---- tag | rs702462 | T/T | 1.00 (.-.) | . | 0.74 | 0.97 | 0.86 | 393 | 33,05 | 179 | 32,14 | 1516 | 489 |  |
| MTHFD2 ---- tag |  | T/A | 0.99 (0.81-1.21) | 0.91 | . | . | . | 593 | 49,87 | 284 | 50,99 | 1516 | 489 |  |
| MTHFD2 ---- tag |  | A/A | 0.95 (0.72-1.25) | 0.72 | . | . | . | 203 | 17,07 | 94 | 16,88 | 1516 | 489 |  |
| MTHFD2 ---- candidate literature | rs702465 | A/A | 1.00 (.-.) | . | 0.66 | 0.96 | 0.86 | 338 | 28,43 | 151 | 27,11 | 1516 | 489 |  |
| MTHFD2 ---- candidate literature |  | A/T | 1.08 (0.88-1.34) | 0.47 | . | . | . | 588 | 49,45 | 296 | 53,14 | 1516 | 489 |  |
| MTHFD2 ---- candidate literature |  | T/T | 1.05 (0.81-1.36) | 0.71 | . | . | . | 263 | 22,12 | 110 | 19,75 | 1516 | 489 |  |
| MTHFD2 ---- candidate literature | rs7571842 | A/A | 1.00 (.-.) | . | 0.30 | 0.95 | 0.52 | 354 | 29,77 | 132 | 23,70 | 1516 | 489 |  |
| MTHFD2 ---- candidate literature |  | A/G | 1.20 (0.97-1.50) | 0.09 | . | . | . | 589 | 49,54 | 313 | 56,19 | 1516 | 489 |  |
| MTHFD2 ---- candidate literature |  | G/G | 1.13 (0.87-1.48) | 0.36 | . | . | . | 246 | 20,69 | 112 | 20,11 | 1516 | 489 |  |
| MTHFD2 ---- tag | rs7587117 | T/T | 1.00 (.-.) | . | 0.22 | 0.92 | 0.51 | 506 | 42,56 | 211 | 37,88 | 1516 | 489 |  |
| MTHFD2 ---- tag |  | T/C | 1.12 (0.93-1.36) | 0.24 | . | . | . | 545 | 45,84 | 279 | 50,09 | 1516 | 489 |  |
| MTHFD2 ---- tag |  | C/C | 1.16 (0.85-1.56) | 0.35 | . | . | . | 138 | 11,61 | 67 | 12,03 | 1516 | 489 |  |
| MTHFD2 ---- tag | rs828861 | C/C | 1.00 (.-.) | . | 0.98 | 0.99 | 0.98 | 340 | 28,60 | 155 | 27,83 | 1516 | 489 |  |
| MTHFD2 ---- tag |  | C/G | 1.07 (0.87-1.32) | 0.53 | . | . | . | 595 | 50,04 | 300 | 53,86 | 1516 | 489 |  |
| MTHFD2 ---- tag |  | G/G | 0.98 (0.75-1.28) | 0.89 | . | . | . | 254 | 21,36 | 102 | 18,31 | 1516 | 489 |  |
| MTHFD2 ---- tag | rs828863 | G/G | 1.00 (.-.) | . | 0.03 | 0.73 | 0.20 | 947 | 79,65 | 475 | 85,28 | 1516 | 489 | low_count |
| MTHFD2 ---- tag |  | G/A | 0.70 (0.54-0.91) | 0.01 | . | . | . | 232 | 19,51 | 78 | 14,00 | 1516 | 489 |  |
| MTHFD2 ---- tag |  | A/A | 1.54 (0.57-4.15) | 0.39 | . | . | . | 10 | 0,84 | 4 | 0,72 | 1516 | 489 |  |
| MTHFR ---- tag | rs1476413 | G/G | 1.00 (.-.) | . | 0.84 | 0.98 | 0.96 | 634 | 53,32 | 292 | 52,42 | 1516 | 489 |  |
| MTHFR ---- tag |  | G/A | 0.99 (0.82-1.19) | 0.89 | . | . | . | 477 | 40,12 | 232 | 41,65 | 1516 | 489 |  |
| MTHFR ---- tag |  | A/A | 0.97 (0.66-1.42) | 0.86 | . | . | . | 78 | 6,56 | 33 | 5,92 | 1516 | 489 |  |
| MTHFR ---- tag | rs17376328 | G/G | 1.00 (.-.) | . | 0.18 | 0.92 | 0.83 | 1062 | 89,32 | 474 | 85,10 | 1516 | 489 | low_count |
| MTHFR ---- tag |  | G/A | 1.19 (0.91-1.54) | 0.20 | . | . | . | 122 | 10,26 | 79 | 14,18 | 1516 | 489 |  |
| MTHFR ---- tag |  | A/A | 1.27 (0.47-3.46) | 0.64 | . | . | . | 5 | 0,42 | 4 | 0,72 | 1516 | 489 |  |
| MTHFR ---- tag | rs17421462 | G/G | 1.00 (.-.) | . | 0.85 | 0.98 | 0.96 | 1006 | 84,61 | 478 | 85,82 | 1516 | 489 | low_count |
| MTHFR ---- tag |  | G/A | 0.86 (0.66-1.13) | 0.29 | . | . | . | 180 | 15,14 | 74 | 13,29 | 1516 | 489 |  |
| MTHFR ---- tag |  | A/A | 3.69 (1.51-9.04) | 0.00 | . | . | . | 3 | 0,25 | 5 | 0,90 | 1516 | 489 |  |
| MTHFR ---- candidate | rs1801131 | A/A | 1.00 (.-.) | . | 0.85 | 0.98 | 0.96 | 550 | 46,26 | 251 | 45,06 | 1516 | 489 |  |
| MTHFR ---- candidate |  | A/C | 0.99 (0.82-1.20) | 0.95 | . | . | . | 520 | 43,73 | 255 | 45,78 | 1516 | 489 |  |
| MTHFR ---- candidate |  | C/C | 0.96 (0.69-1.34) | 0.82 | . | . | . | 119 | 10,01 | 51 | 9,16 | 1516 | 489 |  |
| MTHFR ---- candidate | rs1801133 | C/C | 1.00 (.-.) | . | 0.06 | 0.77 | 0.54 | 479 | 40,29 | 244 | 43,81 | 1516 | 489 |  |
| MTHFR ---- candidate |  | C/T | 0.80 (0.66-0.96) | 0.02 | . | . | . | 570 | 47,94 | 257 | 46,14 | 1516 | 489 |  |
| MTHFR ---- candidate |  | T/T | 0.86 (0.62-1.19) | 0.37 | . | . | . | 140 | 11,77 | 56 | 10,05 | 1516 | 489 |  |
| MTHFR ---- tag | rs2066471 | G/G | 1.00 (.-.) | . | 0.54 | 0.96 | 0.96 | 826 | 69,47 | 396 | 71,10 | 1516 | 489 |  |
| MTHFR ---- tag |  | G/A | 0.95 (0.77-1.16) | 0.60 | . | . | . | 338 | 28,43 | 148 | 26,57 | 1516 | 489 |  |
| MTHFR ---- tag |  | A/A | 0.89 (0.49-1.64) | 0.72 | . | . | . | 25 | 2,10 | 13 | 2,33 | 1516 | 489 |  |
| MTHFR ---- tag | rs4846047 | G/G | 1.00 (.-.) | . | 0.58 | 0.96 | 0.96 | 588 | 49,45 | 292 | 52,42 | 1516 | 489 |  |
| MTHFR ---- tag |  | G/C | 0.86 (0.71-1.04) | 0.12 | . | . | . | 519 | 43,65 | 218 | 39,14 | 1516 | 489 |  |
| MTHFR ---- tag |  | C/C | 1.11 (0.79-1.57) | 0.54 | . | . | . | 82 | 6,90 | 47 | 8,44 | 1516 | 489 |  |
| MTHFR ---- tag | rs4846049 | G/G | 1.00 (.-.) | . | 0.82 | 0.98 | 0.96 | 542 | 45,58 | 246 | 44,17 | 1516 | 489 |  |
| MTHFR ---- tag |  | G/T | 0.98 (0.81-1.19) | 0.85 | . | . | . | 526 | 44,24 | 259 | 46,50 | 1516 | 489 |  |
| MTHFR ---- tag |  | T/T | 0.97 (0.70-1.35) | 0.86 | . | . | . | 121 | 10,18 | 52 | 9,34 | 1516 | 489 |  |
| MTHFR ---- tag | rs7538516 | T/T | 1.00 (.-.) | . | 0.97 | 0.99 | 0.97 | 438 | 36,84 | 203 | 36,45 | 1516 | 489 |  |
| MTHFR ---- tag |  | T/C | 0.89 (0.74-1.09) | 0.26 | . | . | . | 590 | 49,62 | 273 | 49,01 | 1516 | 489 |  |
| MTHFR ---- tag |  | C/C | 1.07 (0.81-1.42) | 0.62 | . | . | . | 161 | 13,54 | 81 | 14,54 | 1516 | 489 |  |
| MTR ---- tag | rs10733117 | A/A | 1.00 (.-.) | . | 0.66 | 0.96 | 0.66 | 426 | 35,83 | 195 | 35,01 | 1516 | 489 |  |
| MTR ---- tag |  | A/G | 0.95 (0.78-1.16) | 0.62 | . | . | . | 574 | 48,28 | 268 | 48,11 | 1516 | 489 |  |
| MTR ---- tag |  | G/G | 0.95 (0.72-1.26) | 0.73 | . | . | . | 189 | 15,90 | 94 | 16,88 | 1516 | 489 |  |
| MTR ---- tag | rs12129440 | G/G | 1.00 (.-.) | . | 0.50 | 0.96 | 0.66 | 640 | 53,83 | 318 | 57,09 | 1516 | 489 |  |
| MTR ---- tag |  | G/A | 0.94 (0.78-1.13) | 0.52 | . | . | . | 461 | 38,77 | 208 | 37,34 | 1516 | 489 |  |
| MTR ---- tag |  | A/A | 0.93 (0.61-1.41) | 0.72 | . | . | . | 88 | 7,40 | 31 | 5,57 | 1516 | 489 |  |
| MTR ---- candidate | rs1805087 | A/A | 1.00 (.-.) | . | 0.56 | 0.96 | 0.66 | 789 | 66,36 | 384 | 68,94 | 1516 | 489 |  |
| MTR ---- candidate |  | A/G | 1.01 (0.83-1.24) | 0.89 | . | . | . | 356 | 29,94 | 156 | 28,01 | 1516 | 489 |  |
| MTR ---- candidate |  | G/G | 0.73 (0.43-1.25) | 0.26 | . | . | . | 44 | 3,70 | 17 | 3,05 | 1516 | 489 |  |
| MTR ---- tag | rs3890786 | C/C | 1.00 (.-.) | . | 0.37 | 0.96 | 0.66 | 405 | 34,06 | 185 | 33,21 | 1516 | 489 |  |
| MTR ---- tag |  | C/T | 1.01 (0.82-1.24) | 0.91 | . | . | . | 583 | 49,03 | 274 | 49,19 | 1516 | 489 |  |
| MTR ---- tag |  | T/T | 1.15 (0.88-1.49) | 0.31 | . | . | . | 201 | 16,90 | 98 | 17,59 | 1516 | 489 |  |
| MTR ---- tag | rs4659727 | A/A | 1.00 (.-.) | . | 0.35 | 0.96 | 0.66 | 784 | 65,94 | 384 | 68,94 | 1516 | 489 |  |
| MTR ---- tag |  | A/G | 1.03 (0.85-1.26) | 0.75 | . | . | . | 357 | 30,03 | 157 | 28,19 | 1516 | 489 |  |
| MTR ---- tag |  | G/G | 0.60 (0.34-1.03) | 0.07 | . | . | . | 48 | 4,04 | 16 | 2,87 | 1516 | 489 |  |
| MTRR ---- candidate literature/tag | rs10380 | C/C | 1.00 (.-.) | . | 0.75 | 0.97 | 0.99 | 1000 | 84,10 | 465 | 83,48 | 1516 | 489 | low_count |
| MTRR ---- candidate literature/tag |  | C/T | 1.02 (0.80-1.31) | 0.87 | . | . | . | 181 | 15,22 | 89 | 15,98 | 1516 | 489 |  |
| MTRR ---- candidate literature/tag |  | T/T | 1.36 (0.43-4.30) | 0.60 | . | . | . | 8 | 0,67 | 3 | 0,54 | 1516 | 489 |  |
| MTRR ---- tag | rs10475399 | G/G | 1.00 (.-.) | . | 0.96 | 0.99 | 0.99 | 515 | 43,31 | 241 | 43,27 | 1516 | 489 |  |
| MTRR ---- tag |  | G/A | 1.05 (0.87-1.27) | 0.61 | . | . | . | 543 | 45,67 | 258 | 46,32 | 1516 | 489 |  |
| MTRR ---- tag |  | A/A | 0.96 (0.70-1.31) | 0.80 | . | . | . | 131 | 11,02 | 58 | 10,41 | 1516 | 489 |  |
| MTRR ---- tag | rs11134265 | C/C | 1.00 (.-.) | . | 0.66 | 0.96 | 0.99 | 519 | 43,65 | 250 | 44,88 | 1516 | 489 |  |
| MTRR ---- tag |  | C/T | 1.04 (0.86-1.26) | 0.65 | . | . | . | 539 | 45,33 | 252 | 45,24 | 1516 | 489 |  |
| MTRR ---- tag |  | T/T | 1.05 (0.76-1.43) | 0.78 | . | . | . | 131 | 11,02 | 55 | 9,87 | 1516 | 489 |  |
| MTRR ---- tag | rs13181011 | T/T | 1.00 (.-.) | . | 0.36 | 0.96 | 0.99 | 786 | 66,11 | 338 | 60,68 | 1516 | 489 |  |
| MTRR ---- tag |  | T/C | 0.99 (0.82-1.20) | 0.93 | . | . | . | 361 | 30,36 | 187 | 33,57 | 1516 | 489 |  |
| MTRR ---- tag |  | C/C | 1.42 (0.95-2.13) | 0.09 | . | . | . | 42 | 3,53 | 32 | 5,75 | 1516 | 489 |  |
| MTRR ---- tag | rs161869 | C/C | 1.00 (.-.) | . | 0.58 | 0.96 | 0.99 | 410 | 34,48 | 198 | 35,55 | 1516 | 489 |  |
| MTRR ---- tag |  | C/T | 1.04 (0.85-1.27) | 0.71 | . | . | . | 576 | 48,44 | 272 | 48,83 | 1516 | 489 |  |
| MTRR ---- tag |  | T/T | 1.08 (0.82-1.41) | 0.60 | . | . | . | 203 | 17,07 | 87 | 15,62 | 1516 | 489 |  |
| MTRR ---- tagged by rs162039 | rs162036 | A/A | 1.00 (.-.) | . | 0.64 | 0.96 | 0.99 | 958 | 80,57 | 444 | 79,71 | 1516 | 489 | low_count |
| MTRR ---- tagged by rs162039 |  | A/G | 1.01 (0.80-1.27) | 0.92 | . | . | . | 221 | 18,59 | 107 | 19,21 | 1516 | 489 |  |
| MTRR ---- tagged by rs162039 |  | G/G | 1.49 (0.66-3.37) | 0.34 | . | . | . | 10 | 0,84 | 6 | 1,08 | 1516 | 489 |  |
| MTRR ---- tag | rs162039 | C/C | 1.00 (.-.) | . | 0.64 | 0.96 | 0.99 | 959 | 80,66 | 444 | 79,71 | 1516 | 489 | low_count |
| MTRR ---- tag |  | C/T | 1.01 (0.80-1.27) | 0.92 | . | . | . | 220 | 18,50 | 107 | 19,21 | 1516 | 489 |  |
| MTRR ---- tag |  | T/T | 1.49 (0.66-3.37) | 0.34 | . | . | . | 10 | 0,84 | 6 | 1,08 | 1516 | 489 |  |
| MTRR ---- tag | rs162270 | G/G | 1.00 (.-.) | . | 0.99 | 0.99 | 0.99 | 842 | 70,82 | 388 | 69,66 | 1516 | 489 |  |
| MTRR ---- tag |  | G/T | 1.16 (0.95-1.42) | 0.14 | . | . | . | 312 | 26,24 | 157 | 28,19 | 1516 | 489 |  |
| MTRR ---- tag |  | T/T | 0.56 (0.30-1.06) | 0.07 | . | . | . | 35 | 2,94 | 12 | 2,15 | 1516 | 489 |  |
| MTRR ---- candidate | rs16879334 | C/C | 1.00 (.-.) | . | 0.54 | 0.96 | 0.99 | 1113 | 93,61 | 530 | 95,15 | 1516 | 489 |  |
| MTRR ---- candidate |  | C/G | 0.88 (0.58-1.34) | 0.54 | . | . | . | 76 | 6,39 | 27 | 4,85 | 1516 | 489 |  |
| MTRR ----candidate | rs1801394 | G/G | 1.00 (.-.) | . | 0.48 | 0.96 | 0.99 | 343 | 28,85 | 172 | 30,88 | 1516 | 489 |  |
| MTRR ---- candidate |  | G/A | 0.98 (0.80-1.21) | 0.88 | . | . | . | 601 | 50,55 | 286 | 51,35 | 1516 | 489 |  |
| MTRR ---- candidate |  | A/A | 0.90 (0.69-1.18) | 0.44 | . | . | . | 245 | 20,61 | 99 | 17,77 | 1516 | 489 |  |
| MTRR ---- tag | rs1802059 | G/G | 1.00 (.-.) | . | 0.93 | 0.98 | 0.99 | 462 | 38,86 | 207 | 37,16 | 1516 | 489 |  |
| MTRR ---- tag |  | G/A | 0.98 (0.80-1.19) | 0.81 | . | . | . | 566 | 47,60 | 260 | 46,68 | 1516 | 489 |  |
| MTRR ---- tag |  | A/A | 1.00 (0.76-1.30) | 0.98 | . | . | . | 161 | 13,54 | 90 | 16,16 | 1516 | 489 |  |
| MTRR ---- tag | rs2077744 | T/T | 1.00 (.-.) | . | 0.97 | 0.99 | 0.99 | 866 | 72,83 | 403 | 72,35 | 1516 | 489 | low_count |
| MTRR ---- tag |  | T/C | 1.11 (0.90-1.36) | 0.33 | . | . | . | 292 | 24,56 | 144 | 25,85 | 1516 | 489 |  |
| MTRR ---- tag |  | C/C | 0.67 (0.36-1.27) | 0.22 | . | . | . | 31 | 2,61 | 10 | 1,80 | 1516 | 489 |  |
| MTRR ---- candidate | rs2287780 | C/C | 1.00 (.-.) | . | 0.54 | 0.96 | 0.99 | 1113 | 93,61 | 530 | 95,15 | 1516 | 489 |  |
| MTRR ---- candidate |  | C/T | 0.88 (0.58-1.34) | 0.54 | . | . | . | 76 | 6,39 | 27 | 4,85 | 1516 | 489 |  |
| MTRR ---- candidate | rs2303080 | T/T | 1.00 (.-.) | . | 0.51 | 0.96 | 0.99 | 1113 | 93,61 | 531 | 95,33 | 1516 | 489 |  |
| MTRR ---- candidate |  | T/A | 0.87 (0.56-1.33) | 0.51 | . | . | . | 76 | 6,39 | 26 | 4,67 | 1516 | 489 |  |
| MTRR ---- tag | rs7715062 | G/G | 1.00 (.-.) | . | 0.78 | 0.97 | 0.99 | 408 | 34,31 | 176 | 31,60 | 1516 | 489 |  |
| MTRR ---- tag |  | G/T | 0.98 (0.80-1.21) | 0.88 | . | . | . | 587 | 49,37 | 271 | 48,65 | 1516 | 489 |  |
| MTRR ---- tag |  | T/T | 1.05 (0.81-1.36) | 0.72 | . | . | . | 194 | 16,32 | 110 | 19,75 | 1516 | 489 |  |
| MTRR ---- tag | rs9282787 | T/T | 1.00 (.-.) | . | 0.22 | 0.92 | 0.99 | 793 | 66,69 | 340 | 61,04 | 1516 | 489 |  |
| MTRR ---- tag |  | T/C | 1.04 (0.86-1.26) | 0.69 | . | . | . | 353 | 29,69 | 186 | 33,39 | 1516 | 489 |  |
| MTRR ---- tag |  | C/C | 1.40 (0.93-2.10) | 0.11 | . | . | . | 43 | 3,62 | 31 | 5,57 | 1516 | 489 |  |
| MTRR ---- candidate literature | rs9332 | C/C | 1.00 (.-.) | . | 0.64 | 0.96 | 0.99 | 959 | 80,66 | 444 | 79,71 | 1516 | 489 | low_count |
| MTRR ---- candidate literature |  | C/T | 1.01 (0.80-1.27) | 0.92 | . | . | . | 220 | 18,50 | 107 | 19,21 | 1516 | 489 |  |
| MTRR ---- candidate literature |  | T/T | 1.49 (0.66-3.37) | 0.34 | . | . | . | 10 | 0,84 | 6 | 1,08 | 1516 | 489 |  |
| NFKB1 ---- NA | rs1609798 | C/C | 1.00 (.-.) | . | 0.51 | 0.96 | 0.83 | 557 | 46,85 | 262 | 47,04 | 1516 | 489 |  |
| NFKB1 ---- NA |  | C/T | 1.03 (0.86-1.25) | 0.73 | . | . | . | 519 | 43,65 | 245 | 43,99 | 1516 | 489 |  |
| NFKB1 ---- NA |  | T/T | 0.82 (0.58-1.15) | 0.24 | . | . | . | 113 | 9,50 | 50 | 8,98 | 1516 | 489 |  |
| NFKB1 ---- tag | rs230540 | T/T | 1.00 (.-.) | . | 0.08 | 0.79 | 0.40 | 510 | 42,89 | 232 | 41,65 | 1516 | 489 |  |
| NFKB1 ---- tag |  | T/C | 1.02 (0.84-1.24) | 0.82 | . | . | . | 541 | 45,50 | 268 | 48,11 | 1516 | 489 |  |
| NFKB1 ---- tag |  | C/C | 0.67 (0.49-0.93) | 0.02 | . | . | . | 138 | 11,61 | 57 | 10,23 | 1516 | 489 |  |
| NFKB1 ---- tag | rs230541 | A/A | 1.00 (.-.) | . | 0.09 | 0.79 | 0.40 | 407 | 34,23 | 188 | 33,75 | 1516 | 489 |  |
| NFKB1 ---- tag |  | A/G | 1.07 (0.88-1.31) | 0.49 | . | . | . | 583 | 49,03 | 288 | 51,71 | 1516 | 489 |  |
| NFKB1 ---- tag |  | G/G | 0.72 (0.54-0.96) | 0.03 | . | . | . | 199 | 16,74 | 81 | 14,54 | 1516 | 489 |  |
| NFKB1 ---- NA | rs230547 | C/C | 1.00 (.-.) | . | 0.75 | 0.97 | 0.87 | 965 | 81,16 | 453 | 81,33 | 1516 | 489 | low_count |
| NFKB1 ---- NA |  | C/T | 0.98 (0.78-1.23) | 0.85 | . | . | . | 217 | 18,25 | 96 | 17,24 | 1516 | 489 |  |
| NFKB1 ---- NA |  | T/T | 1.58 (0.74-3.36) | 0.24 | . | . | . | 7 | 0,59 | 8 | 1,44 | 1516 | 489 |  |
| NFKB1 ---- tag | rs3774934 | G/G | 1.00 (.-.) | . | 0.57 | 0.96 | 0.83 | 950 | 79,90 | 441 | 79,17 | 1516 | 489 | low_count |
| NFKB1 ---- tag |  | G/A | 0.98 (0.78-1.22) | 0.84 | . | . | . | 230 | 19,34 | 106 | 19,03 | 1516 | 489 |  |
| NFKB1 ---- tag |  | A/A | 1.85 (0.95-3.60) | 0.07 | . | . | . | 9 | 0,76 | 10 | 1,80 | 1516 | 489 |  |
| NFKB1 ---- tag | rs3774968 | G/G | 1.00 (.-.) | . | 0.30 | 0.95 | 0.78 | 389 | 32,72 | 167 | 29,98 | 1516 | 489 |  |
| NFKB1 ---- tag |  | G/A | 1.17 (0.95-1.45) | 0.14 | . | . | . | 587 | 49,37 | 286 | 51,35 | 1516 | 489 |  |
| NFKB1 ---- tag |  | A/A | 1.12 (0.86-1.46) | 0.40 | . | . | . | 213 | 17,91 | 104 | 18,67 | 1516 | 489 |  |
| NFKB1 ---- NA | rs4648022 | C/C | 1.00 (.-.) | . | 0.95 | 0.99 | 0.95 | 981 | 82,51 | 479 | 86,00 | 1516 | 489 | low_count |
| NFKB1 ---- NA |  | C/T | 1.06 (0.82-1.38) | 0.66 | . | . | . | 199 | 16,74 | 75 | 13,46 | 1516 | 489 |  |
| NFKB1 ---- NA |  | T/T | 0.59 (0.19-1.87) | 0.37 | . | . | . | 9 | 0,76 | 3 | 0,54 | 1516 | 489 |  |
| NFKB1 ---- NA | rs4648090 | G/G | 1.00 (.-.) | . | 0.39 | 0.96 | 0.78 | 857 | 72,08 | 426 | 76,48 | 1516 | 489 | low_count |
| NFKB1 ---- NA |  | G/A | 0.98 (0.79-1.22) | 0.87 | . | . | . | 304 | 25,57 | 123 | 22,08 | 1516 | 489 |  |
| NFKB1 ---- NA |  | A/A | 0.56 (0.25-1.27) | 0.17 | . | . | . | 28 | 2,35 | 8 | 1,44 | 1516 | 489 |  |
| NFKB1 ---- tag | rs4648110 | T/T | 1.00 (.-.) | . | 0.76 | 0.97 | 0.87 | 752 | 63,25 | 356 | 63,91 | 1516 | 489 |  |
| NFKB1 ---- tag |  | T/A | 1.07 (0.89-1.30) | 0.48 | . | . | . | 385 | 32,38 | 183 | 32,85 | 1516 | 489 |  |
| NFKB1 ---- tag |  | A/A | 0.89 (0.53-1.50) | 0.66 | . | . | . | 52 | 4,37 | 18 | 3,23 | 1516 | 489 |  |
| NFKB1 ---- tag | rs4648141 | G/G | 1.00 (.-.) | . | 0.59 | 0.96 | 0.83 | 817 | 68,71 | 388 | 69,66 | 1516 | 489 | low_count |
| NFKB1 ---- tag |  | G/A | 1.11 (0.91-1.35) | 0.32 | . | . | . | 338 | 28,43 | 159 | 28,55 | 1516 | 489 |  |
| NFKB1 ---- tag |  | A/A | 0.85 (0.45-1.60) | 0.62 | . | . | . | 34 | 2,86 | 10 | 1,80 | 1516 | 489 |  |
| NFKB1 ---- tag | rs4698863 | C/C | 1.00 (.-.) | . | 0.32 | 0.96 | 0.78 | 545 | 45,84 | 258 | 46,32 | 1516 | 489 |  |
| NFKB1 ---- tag |  | C/T | 0.99 (0.82-1.20) | 0.91 | . | . | . | 526 | 44,24 | 246 | 44,17 | 1516 | 489 |  |
| NFKB1 ---- tag |  | T/T | 0.81 (0.58-1.12) | 0.21 | . | . | . | 118 | 9,92 | 53 | 9,52 | 1516 | 489 |  |
| NFKB1 ---- NA | rs7674640 | T/T | 1.00 (.-.) | . | 0.38 | 0.96 | 0.78 | 273 | 22,96 | 126 | 22,62 | 1516 | 489 |  |
| NFKB1 ---- NA |  | T/C | 1.07 (0.86-1.34) | 0.55 | . | . | . | 604 | 50,80 | 298 | 53,50 | 1516 | 489 |  |
| NFKB1 ---- NA |  | C/C | 0.89 (0.68-1.16) | 0.39 | . | . | . | 312 | 26,24 | 133 | 23,88 | 1516 | 489 |  |
| NFKB1 ---- tag | rs909332 | A/A | 1.00 (.-.) | . | 0.81 | 0.98 | 0.87 | 1073 | 90,24 | 512 | 91,92 | 1516 | 489 | low_count |
| NFKB1 ---- tag |  | A/T | 1.02 (0.73-1.42) | 0.90 | . | . | . | 114 | 9,59 | 44 | 7,90 | 1516 | 489 |  |
| NFKB1 ---- tag |  | T/T | 1.68 (0.23-12.08) | 0.61 | . | . | . | 2 | 0,17 | 1 | 0,18 | 1516 | 489 |  |
| NFKB1 ---- tag | rs997476 | C/C | 1.00 (.-.) | . | 0.05 | 0.77 | 0.40 | 1068 | 89,82 | 482 | 86,54 | 1516 | 489 | low_count |
| NFKB1 ---- tag |  | C/A | 1.26 (0.97-1.64) | 0.08 | . | . | . | 120 | 10,09 | 73 | 13,11 | 1516 | 489 |  |
| NFKB1 ---- tag |  | A/A | 2.03 (0.50-8.34) | 0.32 | . | . | . | 1 | 0,08 | 2 | 0,36 | 1516 | 489 |  |
| NME1 ---- NA | rs10514981 | T/T | 1.00 (.-.) | . | 0.26 | 0.95 | 0.98 | 721 | 60,64 | 360 | 64,63 | 1516 | 489 |  |
| NME1 ---- NA |  | T/G | 0.89 (0.73-1.07) | 0.21 | . | . | . | 415 | 34,90 | 180 | 32,32 | 1516 | 489 |  |
| NME1 ---- NA |  | G/G | 0.93 (0.54-1.59) | 0.78 | . | . | . | 53 | 4,46 | 17 | 3,05 | 1516 | 489 |  |
| NME1 ---- NA | rs11651252 | T/T | 1.00 (.-.) | . | 0.55 | 0.96 | 0.98 | 1056 | 88,81 | 495 | 88,87 | 1516 | 489 | low_count |
| NME1 ---- NA |  | T/C | 0.95 (0.71-1.27) | 0.72 | . | . | . | 128 | 10,77 | 61 | 10,95 | 1516 | 489 |  |
| NME1 ---- NA |  | C/C | 0.49 (0.07-3.50) | 0.47 | . | . | . | 5 | 0,42 | 1 | 0,18 | 1516 | 489 |  |
| NME1 ---- tag | rs11652793 | T/T | 1.00 (.-.) | . | 0.36 | 0.96 | 0.98 | 773 | 65,01 | 389 | 69,84 | 1516 | 489 |  |
| NME1 ---- tag |  | T/C | 0.94 (0.77-1.14) | 0.52 | . | . | . | 373 | 31,37 | 156 | 28,01 | 1516 | 489 |  |
| NME1 ---- tag |  | C/C | 0.77 (0.39-1.49) | 0.44 | . | . | . | 43 | 3,62 | 12 | 2,15 | 1516 | 489 |  |
| NME1 ---- NA | rs11868380 | C/C | 1.00 (.-.) | . | 0.96 | 0.99 | 0.99 | 750 | 63,08 | 343 | 61,58 | 1516 | 489 |  |
| NME1 ---- NA |  | C/G | 1.00 (0.82-1.22) | 0.99 | . | . | . | 396 | 33,31 | 191 | 34,29 | 1516 | 489 |  |
| NME1 ---- NA |  | G/G | 0.98 (0.63-1.53) | 0.92 | . | . | . | 43 | 3,62 | 23 | 4,13 | 1516 | 489 |  |
| NME1 ---- NA | rs1558252 | T/T | 1.00 (.-.) | . | 0.78 | 0.97 | 0.98 | 576 | 48,44 | 256 | 45,96 | 1516 | 489 |  |
| NME1 ---- NA |  | T/C | 1.01 (0.83-1.21) | 0.95 | . | . | . | 515 | 43,31 | 239 | 42,91 | 1516 | 489 |  |
| NME1 ---- NA |  | C/C | 1.06 (0.77-1.44) | 0.73 | . | . | . | 98 | 8,24 | 62 | 11,13 | 1516 | 489 |  |
| NME1 ---- NA | rs1558253 | T/T | 1.00 (.-.) | . | 0.29 | 0.95 | 0.98 | 1058 | 88,98 | 483 | 86,71 | 1516 | 489 | low_count |
| NME1 ---- NA |  | T/G | 1.14 (0.87-1.47) | 0.34 | . | . | . | 127 | 10,68 | 73 | 13,11 | 1516 | 489 |  |
| NME1 ---- NA |  | G/G | 2.01 (0.28-14.57) | 0.49 | . | . | . | 4 | 0,34 | 1 | 0,18 | 1516 | 489 |  |
| NME1 ---- tag | rs16949683 | C/C | 1.00 (.-.) | . | 0.64 | 0.96 | 0.98 | 1107 | 93,10 | 512 | 91,92 | 1516 | 489 | low_count |
| NME1 ---- tag |  | C/T | 1.10 (0.78-1.53) | 0.59 | . | . | . | 79 | 6,64 | 44 | 7,90 | 1516 | 489 |  |
| NME1 ---- tag |  | T/T | 0.92 (0.13-6.58) | 0.93 | . | . | . | 3 | 0,25 | 1 | 0,18 | 1516 | 489 |  |
| NME1 ---- tag | rs2318784 | C/C | 1.00 (.-.) | . | 0.71 | 0.97 | 0.98 | 909 | 76,45 | 425 | 76,30 | 1516 | 489 | low_count |
| NME1 ---- tag |  | C/T | 1.06 (0.85-1.31) | 0.62 | . | . | . | 260 | 21,87 | 124 | 22,26 | 1516 | 489 |  |
| NME1 ---- tag |  | T/T | 0.96 (0.45-2.03) | 0.91 | . | . | . | 20 | 1,68 | 8 | 1,44 | 1516 | 489 |  |
| NME1 ---- NA | rs2318785 | G/G | 1.00 (.-.) | . | 0.70 | 0.97 | 0.98 | 372 | 31,29 | 176 | 31,60 | 1516 | 489 |  |
| NME1 ---- NA |  | G/A | 0.95 (0.77-1.16) | 0.59 | . | . | . | 605 | 50,88 | 286 | 51,35 | 1516 | 489 |  |
| NME1 ---- NA |  | A/A | 0.96 (0.74-1.25) | 0.77 | . | . | . | 212 | 17,83 | 95 | 17,06 | 1516 | 489 |  |
| NME1 ---- tag | rs3760469 | G/G | 1.00 (.-.) | . | 0.55 | 0.96 | 0.98 | 310 | 26,07 | 138 | 24,78 | 1516 | 489 |  |
| NME1 ---- tag |  | G/T | 0.97 (0.78-1.21) | 0.81 | . | . | . | 614 | 51,64 | 280 | 50,27 | 1516 | 489 |  |
| NME1 ---- tag |  | T/T | 1.08 (0.84-1.40) | 0.54 | . | . | . | 265 | 22,29 | 139 | 24,96 | 1516 | 489 |  |
| NME1 ---- NA | rs4605213 | G/G | 1.00 (.-.) | . | 0.85 | 0.98 | 0.98 | 494 | 41,55 | 250 | 44,88 | 1516 | 489 |  |
| NME1 ---- NA |  | G/C | 0.90 (0.75-1.09) | 0.29 | . | . | . | 553 | 46,51 | 242 | 43,45 | 1516 | 489 |  |
| NME1 ---- NA |  | C/C | 1.06 (0.79-1.42) | 0.70 | . | . | . | 142 | 11,94 | 65 | 11,67 | 1516 | 489 |  |
| NME1 ---- NA | rs7207090 | A/A | 1.00 (.-.) | . | 0.99 | 0.99 | 0.99 | 306 | 25,74 | 132 | 23,70 | 1516 | 489 |  |
| NME1 ---- NA |  | A/T | 0.93 (0.74-1.16) | 0.52 | . | . | . | 607 | 51,05 | 274 | 49,19 | 1516 | 489 |  |
| NME1 ---- NA |  | T/T | 1.00 (0.77-1.29) | 0.99 | . | . | . | 276 | 23,21 | 151 | 27,11 | 1516 | 489 |  |
| NME1 ---- tag | rs7222463 | A/A | 1.00 (.-.) | . | 0.82 | 0.98 | 0.98 | 314 | 26,41 | 160 | 28,73 | 1516 | 489 |  |
| NME1 ---- tag |  | A/C | 0.86 (0.70-1.06) | 0.17 | . | . | . | 615 | 51,72 | 265 | 47,58 | 1516 | 489 |  |
| NME1 ---- tag |  | C/C | 1.05 (0.82-1.35) | 0.70 | . | . | . | 260 | 21,87 | 132 | 23,70 | 1516 | 489 |  |
| NME1 ---- tag | rs7226059 | C/C | 1.00 (.-.) | . | 0.36 | 0.96 | 0.98 | 550 | 46,26 | 235 | 42,19 | 1516 | 489 |  |
| NME1 ---- tag |  | C/T | 1.17 (0.97-1.42) | 0.11 | . | . | . | 511 | 42,98 | 264 | 47,40 | 1516 | 489 |  |
| NME1 ---- tag |  | T/T | 1.03 (0.75-1.40) | 0.86 | . | . | . | 128 | 10,77 | 58 | 10,41 | 1516 | 489 |  |
| NME1 ---- NA | rs880178 | G/G | 1.00 (.-.) | . | 0.47 | 0.96 | 0.98 | 307 | 25,82 | 135 | 24,24 | 1516 | 489 |  |
| NME1 ---- NA |  | G/T | 0.99 (0.79-1.24) | 0.92 | . | . | . | 614 | 51,64 | 285 | 51,17 | 1516 | 489 |  |
| NME1 ---- NA |  | T/T | 1.10 (0.85-1.42) | 0.48 | . | . | . | 268 | 22,54 | 137 | 24,60 | 1516 | 489 |  |
| NME2 ---- tag | rs7220360 | C/C | 1.00 (.-.) | . | 0.80 | 0.98 | 0.80 | 314 | 26,41 | 158 | 28,37 | 1516 | 489 |  |
| NME2 ---- tag |  | C/G | 0.87 (0.70-1.08) | 0.20 | . | . | . | 614 | 51,64 | 267 | 47,94 | 1516 | 489 |  |
| NME2 ---- tag |  | G/G | 1.05 (0.82-1.35) | 0.68 | . | . | . | 261 | 21,95 | 132 | 23,70 | 1516 | 489 |  |
| PON1 ---- tag | rs2269829 | A/A | 1.00 (.-.) | . | 0.03 | 0.73 | 0.07 | 623 | 52,40 | 253 | 45,42 | 1516 | 489 |  |
| PON1 ---- tag |  | A/G | 1.14 (0.94-1.38) | 0.17 | . | . | . | 467 | 39,28 | 245 | 43,99 | 1516 | 489 |  |
| PON1 ---- tag |  | G/G | 1.38 (1.02-1.88) | 0.04 | . | . | . | 99 | 8,33 | 59 | 10,59 | 1516 | 489 |  |
| PON1 ---- tag | rs3917527 | A/A | 1.00 (.-.) | . | 0.06 | 0.77 | 0.10 | 1058 | 88,98 | 513 | 92,10 | 1516 | 489 | low_count |
| PON1 ---- tag |  | A/G | 0.71 (0.52-0.99) | 0.04 | . | . | . | 126 | 10,60 | 42 | 7,54 | 1516 | 489 |  |
| PON1 ---- tag |  | G/G | 1.03 (0.26-4.18) | 0.96 | . | . | . | 5 | 0,42 | 2 | 0,36 | 1516 | 489 |  |
| PON1 ---- tag | rs3917538 | C/C | 1.00 (.-.) | . | <.001 | <.001 | <.001 | 707 | 59,46 | 282 | 50,63 | 1516 | 489 |  |
| PON1 ---- tag |  | C/T | 1.18 (0.97-1.43) | 0.09 | . | . | . | 424 | 35,66 | 224 | 40,22 | 1516 | 489 |  |
| PON1 ---- tag |  | T/T | 2.02 (1.46-2.80) | <.001 | . | . | . | 58 | 4,88 | 51 | 9,16 | 1516 | 489 |  |
| PON1 ---- tag | rs757158 | C/C | 1.00 (.-.) | . | 0.22 | 0.92 | 0.27 | 415 | 34,90 | 206 | 36,98 | 1516 | 489 |  |
| PON1 ---- tag |  | C/T | 0.80 (0.66-0.98) | 0.03 | . | . | . | 580 | 48,78 | 256 | 45,96 | 1516 | 489 |  |
| PON1 ---- tag |  | T/T | 0.91 (0.69-1.19) | 0.48 | . | . | . | 194 | 16,32 | 95 | 17,06 | 1516 | 489 |  |
| PON1 ---- candidate | rs854560 | A/A | 1.00 (.-.) | . | 0.57 | 0.96 | 0.57 | 463 | 38,94 | 236 | 42,37 | 1516 | 489 |  |
| PON1 ---- candidate |  | A/T | 0.94 (0.77-1.14) | 0.51 | . | . | . | 559 | 47,01 | 229 | 41,11 | 1516 | 489 |  |
| PON1 ---- candidate |  | T/T | 1.14 (0.88-1.49) | 0.32 | . | . | . | 167 | 14,05 | 92 | 16,52 | 1516 | 489 |  |
| PRDM2 ---- tag | rs1015370 | C/C | 1.00 (.-.) | . | 0.27 | 0.95 | 0.72 | 599 | 50,38 | 305 | 54,76 | 1516 | 489 |  |
| PRDM2 ---- tag |  | C/T | 0.92 (0.76-1.11) | 0.36 | . | . | . | 467 | 39,28 | 207 | 37,16 | 1516 | 489 |  |
| PRDM2 ---- tag |  | T/T | 0.87 (0.62-1.21) | 0.41 | . | . | . | 123 | 10,34 | 45 | 8,08 | 1516 | 489 |  |
| PRDM2 ---- tag | rs1203634 | A/A | 1.00 (.-.) | . | 0.04 | 0.73 | 0.20 | 737 | 61,98 | 328 | 58,89 | 1516 | 489 |  |
| PRDM2 ---- tag |  | A/G | 1.19 (0.98-1.45) | 0.07 | . | . | . | 397 | 33,39 | 191 | 34,29 | 1516 | 489 |  |
| PRDM2 ---- tag |  | G/G | 1.30 (0.92-1.85) | 0.14 | . | . | . | 55 | 4,63 | 38 | 6,82 | 1516 | 489 |  |
| PRDM2 ---- tag | rs1203645 | A/A | 1.00 (.-.) | . | 0.05 | 0.77 | 0.20 | 497 | 41,80 | 216 | 38,78 | 1516 | 489 |  |
| PRDM2 ---- tag |  | A/C | 1.27 (1.04-1.55) | 0.02 | . | . | . | 521 | 43,82 | 246 | 44,17 | 1516 | 489 |  |
| PRDM2 ---- tag |  | C/C | 1.21 (0.94-1.57) | 0.14 | . | . | . | 171 | 14,38 | 95 | 17,06 | 1516 | 489 |  |
| PRDM2 ---- tag | rs1406416 | C/C | 1.00 (.-.) | . | 0.92 | 0.98 | 0.92 | 642 | 53,99 | 298 | 53,50 | 1516 | 489 |  |
| PRDM2 ---- tag |  | C/T | 0.94 (0.77-1.14) | 0.53 | . | . | . | 450 | 37,85 | 200 | 35,91 | 1516 | 489 |  |
| PRDM2 ---- tag |  | T/T | 1.05 (0.77-1.42) | 0.77 | . | . | . | 97 | 8,16 | 59 | 10,59 | 1516 | 489 |  |
| PRDM2 ---- candidate | rs17350795 | G/G | 1.00 (.-.) | . | 0.73 | 0.97 | 0.88 | 1140 | 95,88 | 531 | 95,33 | 1516 | 489 |  |
| PRDM2 ---- candidate |  | G/A | 0.93 (0.60-1.43) | 0.73 | . | . | . | 49 | 4,12 | 26 | 4,67 | 1516 | 489 |  |
| PRDM2 ---- tag | rs1980472 | C/C | 1.00 (.-.) | . | 0.04 | 0.77 | 0.20 | 682 | 57,36 | 292 | 52,42 | 1516 | 489 |  |
| PRDM2 ---- tag |  | C/G | 1.22 (1.01-1.48) | 0.04 | . | . | . | 428 | 36,00 | 220 | 39,50 | 1516 | 489 |  |
| PRDM2 ---- tag |  | G/G | 1.23 (0.89-1.71) | 0.21 | . | . | . | 79 | 6,64 | 45 | 8,08 | 1516 | 489 |  |
| PRDM2 ---- tag | rs2235515 | G/G | 1.00 (.-.) | . | 0.42 | 0.96 | 0.72 | 704 | 59,21 | 313 | 56,19 | 1516 | 489 |  |
| PRDM2 ---- tag |  | G/A | 1.19 (0.99-1.43) | 0.07 | . | . | . | 416 | 34,99 | 218 | 39,14 | 1516 | 489 |  |
| PRDM2 ---- tag |  | A/A | 0.87 (0.57-1.33) | 0.53 | . | . | . | 69 | 5,80 | 26 | 4,67 | 1516 | 489 |  |
| PRDM2 ---- tag | rs2244634 | A/A | 1.00 (.-.) | . | 0.48 | 0.96 | 0.72 | 761 | 64,00 | 360 | 64,63 | 1516 | 489 |  |
| PRDM2 ---- tag |  | A/C | 1.07 (0.88-1.30) | 0.51 | . | . | . | 367 | 30,87 | 179 | 32,14 | 1516 | 489 |  |
| PRDM2 ---- tag |  | C/C | 0.64 (0.39-1.05) | 0.08 | . | . | . | 61 | 5,13 | 18 | 3,23 | 1516 | 489 |  |
| PRDM2 ---- tag | rs2245213 | G/G | 1.00 (.-.) | . | 0.73 | 0.97 | 0.88 | 841 | 70,73 | 389 | 69,84 | 1516 | 489 | low_count |
| PRDM2 ---- tag |  | G/T | 1.15 (0.94-1.40) | 0.17 | . | . | . | 308 | 25,90 | 160 | 28,73 | 1516 | 489 |  |
| PRDM2 ---- tag |  | T/T | 0.41 (0.19-0.87) | 0.02 | . | . | . | 40 | 3,36 | 8 | 1,44 | 1516 | 489 |  |
| PRDM2 ---- tag | rs2294484 | C/C | 1.00 (.-.) | . | 0.88 | 0.98 | 0.92 | 988 | 83,10 | 470 | 84,38 | 1516 | 489 | low_count |
| PRDM2 ---- tag |  | C/G | 1.11 (0.87-1.43) | 0.41 | . | . | . | 188 | 15,81 | 86 | 15,44 | 1516 | 489 |  |
| PRDM2 ---- tag |  | G/G | 0.27 (0.04-1.92) | 0.19 | . | . | . | 13 | 1,09 | 1 | 0,18 | 1516 | 489 |  |
| PRDM2 ---- tag | rs2744689 | G/G | 1.00 (.-.) | . | 0.37 | 0.96 | 0.72 | 849 | 71,40 | 398 | 71,45 | 1516 | 489 | low_count |
| PRDM2 ---- tag |  | G/A | 1.09 (0.89-1.33) | 0.43 | . | . | . | 305 | 25,65 | 152 | 27,29 | 1516 | 489 |  |
| PRDM2 ---- tag |  | A/A | 0.36 (0.16-0.80) | 0.01 | . | . | . | 35 | 2,94 | 7 | 1,26 | 1516 | 489 |  |
| PRDM2 ---- tag | rs6690270 | A/A | 1.00 (.-.) | . | 0.34 | 0.96 | 0.72 | 478 | 40,20 | 222 | 39,86 | 1516 | 489 |  |
| PRDM2 ---- tag |  | A/G | 0.91 (0.75-1.11) | 0.35 | . | . | . | 560 | 47,10 | 256 | 45,96 | 1516 | 489 |  |
| PRDM2 ---- tag |  | G/G | 0.90 (0.68-1.19) | 0.45 | . | . | . | 151 | 12,70 | 79 | 14,18 | 1516 | 489 |  |
| RRM1 ---- tag | rs10835601 | G/G | 1.00 (.-.) | . | 0.94 | 0.99 | 0.94 | 578 | 48,61 | 269 | 48,29 | 1516 | 489 |  |
| RRM1 ---- tag |  | G/A | 1.00 (0.82-1.20) | 0.96 | . | . | . | 508 | 42,72 | 236 | 42,37 | 1516 | 489 |  |
| RRM1 ---- tag |  | A/A | 1.02 (0.74-1.42) | 0.88 | . | . | . | 103 | 8,66 | 52 | 9,34 | 1516 | 489 |  |
| RRM1 ---- tag | rs10835613 | C/C | 1.00 (.-.) | . | 0.50 | 0.96 | 0.93 | 397 | 33,39 | 193 | 34,65 | 1516 | 489 |  |
| RRM1 ---- tag |  | C/G | 0.91 (0.74-1.11) | 0.36 | . | . | . | 583 | 49,03 | 257 | 46,14 | 1516 | 489 |  |
| RRM1 ---- tag |  | G/G | 1.15 (0.89-1.48) | 0.29 | . | . | . | 209 | 17,58 | 107 | 19,21 | 1516 | 489 |  |
| RRM1 ---- NA | rs10835677 | G/G | 1.00 (.-.) | . | 0.84 | 0.98 | 0.93 | 992 | 83,43 | 457 | 82,05 | 1516 | 489 | low_count |
| RRM1 ---- NA |  | G/A | 0.95 (0.75-1.21) | 0.69 | . | . | . | 185 | 15,56 | 94 | 16,88 | 1516 | 489 |  |
| RRM1 ---- NA |  | A/A | 1.14 (0.51-2.58) | 0.75 | . | . | . | 12 | 1,01 | 6 | 1,08 | 1516 | 489 |  |
| RRM1 ---- tag | rs10835678 | A/A | 1.00 (.-.) | . | 0.81 | 0.98 | 0.93 | 1055 | 88,73 | 496 | 89,05 | 1516 | 489 | low_count |
| RRM1 ---- tag |  | A/G | 0.90 (0.67-1.21) | 0.48 | . | . | . | 131 | 11,02 | 58 | 10,41 | 1516 | 489 |  |
| RRM1 ---- tag |  | G/G | 2.17 (0.68-6.89) | 0.19 | . | . | . | 3 | 0,25 | 3 | 0,54 | 1516 | 489 |  |
| RRM1 ---- tag | rs12288551 | C/C | 1.00 (.-.) | . | 0.42 | 0.96 | 0.93 | 1102 | 92,68 | 507 | 91,02 | 1516 | 489 | low_count |
| RRM1 ---- tag |  | C/G | 1.18 (0.86-1.61) | 0.31 | . | . | . | 86 | 7,23 | 48 | 8,62 | 1516 | 489 |  |
| RRM1 ---- tag |  | G/G | 0.00 (0.00-2E177) | 0.96 | . | . | . | 1 | 0,08 | 2 | 0,36 | 1516 | 489 |  |
| RRM1 ---- NA | rs12806698 | C/C | 1.00 (.-.) | . | 0.40 | 0.96 | 0.93 | 620 | 52,14 | 282 | 50,63 | 1516 | 489 |  |
| RRM1 ---- NA |  | C/A | 1.02 (0.85-1.24) | 0.82 | . | . | . | 482 | 40,54 | 228 | 40,93 | 1516 | 489 |  |
| RRM1 ---- NA |  | A/A | 1.20 (0.86-1.67) | 0.30 | . | . | . | 87 | 7,32 | 47 | 8,44 | 1516 | 489 |  |
| RRM1 ---- NA | rs1465952 | T/T | 1.00 (.-.) | . | 0.77 | 0.97 | 0.93 | 970 | 81,58 | 458 | 82,23 | 1516 | 489 | low_count |
| RRM1 ---- NA |  | T/C | 1.00 (0.79-1.27) | 0.97 | . | . | . | 204 | 17,16 | 96 | 17,24 | 1516 | 489 |  |
| RRM1 ---- NA |  | C/C | 0.55 (0.14-2.22) | 0.40 | . | . | . | 15 | 1,26 | 3 | 0,54 | 1516 | 489 |  |
| RRM1 ---- tag | rs4910904 | A/A | 1.00 (.-.) | . | 0.23 | 0.95 | 0.93 | 519 | 43,65 | 231 | 41,47 | 1516 | 489 |  |
| RRM1 ---- tag |  | A/G | 1.04 (0.86-1.26) | 0.67 | . | . | . | 539 | 45,33 | 254 | 45,60 | 1516 | 489 |  |
| RRM1 ---- tag |  | G/G | 1.22 (0.91-1.63) | 0.18 | . | . | . | 131 | 11,02 | 72 | 12,93 | 1516 | 489 |  |
| RRM1 ---- tag | rs7103860 | T/T | 1.00 (.-.) | . | 0.66 | 0.96 | 0.93 | 903 | 75,95 | 429 | 77,02 | 1516 | 489 | low_count |
| RRM1 ---- tag |  | T/C | 1.13 (0.91-1.39) | 0.28 | . | . | . | 268 | 22,54 | 125 | 22,44 | 1516 | 489 |  |
| RRM1 ---- tag |  | C/C | 0.40 (0.10-1.63) | 0.20 | . | . | . | 18 | 1,51 | 3 | 0,54 | 1516 | 489 |  |
| RRM1 ---- tag | rs7115496 | C/C | 1.00 (.-.) | . | 0.55 | 0.96 | 0.93 | 1008 | 84,78 | 485 | 87,07 | 1516 | 489 | low_count |
| RRM1 ---- tag |  | C/T | 0.92 (0.70-1.21) | 0.57 | . | . | . | 173 | 14,55 | 69 | 12,39 | 1516 | 489 |  |
| RRM1 ---- tag |  | T/T | 0.90 (0.29-2.80) | 0.85 | . | . | . | 8 | 0,67 | 3 | 0,54 | 1516 | 489 |  |
| RRM2 ---- NA | rs1138729 | A/A | 1.00 (.-.) | . | 0.32 | 0.96 | 0.66 | 862 | 72,50 | 409 | 73,43 | 1516 | 489 |  |
| RRM2 ---- NA |  | A/G | 1.13 (0.91-1.39) | 0.27 | . | . | . | 295 | 24,81 | 132 | 23,70 | 1516 | 489 |  |
| RRM2 ---- NA |  | G/G | 1.07 (0.63-1.82) | 0.79 | . | . | . | 32 | 2,69 | 16 | 2,87 | 1516 | 489 |  |
| RRM2 ---- tag | rs4668664 | G/G | 1.00 (.-.) | . | 0.33 | 0.96 | 0.66 | 594 | 49,96 | 284 | 50,99 | 1516 | 489 |  |
| RRM2 ---- tag |  | G/A | 0.95 (0.78-1.14) | 0.56 | . | . | . | 484 | 40,71 | 222 | 39,86 | 1516 | 489 |  |
| RRM2 ---- tag |  | A/A | 0.86 (0.62-1.19) | 0.36 | . | . | . | 111 | 9,34 | 51 | 9,16 | 1516 | 489 |  |
| RRM2 ---- NA | rs6741290 | C/C | 1.00 (.-.) | . | 0.97 | 0.99 | 0.97 | 371 | 31,20 | 187 | 33,57 | 1516 | 489 |  |
| RRM2 ---- NA |  | C/T | 0.94 (0.77-1.16) | 0.59 | . | . | . | 575 | 48,36 | 250 | 44,88 | 1516 | 489 |  |
| RRM2 ---- NA |  | T/T | 1.02 (0.79-1.30) | 0.90 | . | . | . | 243 | 20,44 | 120 | 21,54 | 1516 | 489 |  |
| RRM2 ---- tag | rs7574663 | C/C | 1.00 (.-.) | . | 0.86 | 0.98 | 0.97 | 769 | 64,68 | 372 | 66,79 | 1516 | 489 |  |
| RRM2 ---- tag |  | C/G | 1.04 (0.85-1.26) | 0.72 | . | . | . | 366 | 30,78 | 163 | 29,26 | 1516 | 489 |  |
| RRM2 ---- tag |  | G/G | 0.97 (0.62-1.52) | 0.90 | . | . | . | 54 | 4,54 | 22 | 3,95 | 1516 | 489 |  |
| SHMT1 ---- candidate | rs1979277 | G/G | 1.00 (.-.) | . | 0.91 | 0.98 | 0.95 | 578 | 48,61 | 277 | 49,73 | 1516 | 489 |  |
| SHMT1 ---- candidate |  | G/A | 0.96 (0.80-1.16) | 0.68 | . | . | . | 510 | 42,89 | 233 | 41,83 | 1516 | 489 |  |
| SHMT1 ---- candidate |  | A/A | 1.10 (0.78-1.54) | 0.58 | . | . | . | 101 | 8,49 | 47 | 8,44 | 1516 | 489 |  |
| SHMT1 ---- tag | rs2168781 | G/G | 1.00 (.-.) | . | 0.61 | 0.96 | 0.95 | 415 | 34,90 | 206 | 36,98 | 1516 | 489 |  |
| SHMT1 ---- tag |  | G/C | 1.00 (0.82-1.22) | 1.00 | . | . | . | 595 | 50,04 | 266 | 47,76 | 1516 | 489 |  |
| SHMT1 ---- tag |  | C/C | 1.09 (0.83-1.44) | 0.52 | . | . | . | 179 | 15,05 | 85 | 15,26 | 1516 | 489 |  |
| SHMT1 ---- tag | rs4924849 | C/C | 1.00 (.-.) | . | 0.95 | 0.99 | 0.95 | 603 | 50,71 | 292 | 52,42 | 1516 | 489 |  |
| SHMT1 ---- tag |  | C/T | 0.95 (0.79-1.15) | 0.62 | . | . | . | 495 | 41,63 | 222 | 39,86 | 1516 | 489 |  |
| SHMT1 ---- tag |  | T/T | 1.07 (0.75-1.52) | 0.70 | . | . | . | 91 | 7,65 | 43 | 7,72 | 1516 | 489 |  |
| SHMT1 ---- candidate literature | rs9909104 | T/T | 1.00 (.-.) | . | 0.95 | 0.99 | 0.95 | 624 | 52,48 | 287 | 51,53 | 1516 | 489 |  |
| SHMT1 ---- candidate literature |  | T/C | 0.99 (0.82-1.20) | 0.92 | . | . | . | 474 | 39,87 | 229 | 41,11 | 1516 | 489 |  |
| SHMT1 ---- candidate literature |  | C/C | 1.00 (0.70-1.42) | 0.99 | . | . | . | 91 | 7,65 | 41 | 7,36 | 1516 | 489 |  |
| SHMT2 ---- tag | rs10876968 | G/G | 1.00 (.-.) | . | 0.56 | 0.96 | 0.70 | 640 | 53,83 | 307 | 55,12 | 1516 | 489 |  |
| SHMT2 ---- tag |  | G/T | 0.98 (0.81-1.18) | 0.81 | . | . | . | 449 | 37,76 | 212 | 38,06 | 1516 | 489 |  |
| SHMT2 ---- tag |  | T/T | 0.88 (0.59-1.29) | 0.50 | . | . | . | 100 | 8,41 | 38 | 6,82 | 1516 | 489 |  |
| SHMT2 ---- tag | rs1800165 | T/T | 1.00 (.-.) | . | 0.18 | 0.92 | 0.31 | 585 | 49,20 | 264 | 47,40 | 1516 | 489 |  |
| SHMT2 ---- tag |  | T/C | 1.13 (0.94-1.37) | 0.19 | . | . | . | 498 | 41,88 | 239 | 42,91 | 1516 | 489 |  |
| SHMT2 ---- tag |  | C/C | 1.16 (0.84-1.59) | 0.36 | . | . | . | 106 | 8,92 | 54 | 9,69 | 1516 | 489 |  |
| SHMT2 ---- tag | rs7133939 | T/T | 1.00 (.-.) | . | 0.96 | 0.99 | 0.96 | 359 | 30,19 | 173 | 31,06 | 1516 | 489 |  |
| SHMT2 ---- tag |  | T/A | 1.04 (0.84-1.27) | 0.73 | . | . | . | 574 | 48,28 | 270 | 48,47 | 1516 | 489 |  |
| SHMT2 ---- tag |  | A/A | 1.00 (0.77-1.29) | 0.98 | . | . | . | 256 | 21,53 | 114 | 20,47 | 1516 | 489 |  |
| SHMT2 ---- tag | rs7485577 | G/G | 1.00 (.-.) | . | 0.19 | 0.92 | 0.31 | 645 | 54,25 | 290 | 52,06 | 1516 | 489 |  |
| SHMT2 ---- tag |  | G/A | 1.10 (0.91-1.33) | 0.34 | . | . | . | 457 | 38,44 | 218 | 39,14 | 1516 | 489 |  |
| SHMT2 ---- tag |  | A/A | 1.20 (0.87-1.66) | 0.26 | . | . | . | 87 | 7,32 | 49 | 8,80 | 1516 | 489 |  |
| SHMT2 ---- tag | rs7489231 | T/T | 1.00 (.-.) | . | 0.11 | 0.80 | 0.31 | 526 | 44,24 | 241 | 43,27 | 1516 | 489 |  |
| SHMT2 ---- tag |  | T/C | 1.16 (0.96-1.40) | 0.14 | . | . | . | 532 | 44,74 | 249 | 44,70 | 1516 | 489 |  |
| SHMT2 ---- tag |  | C/C | 1.20 (0.89-1.61) | 0.23 | . | . | . | 131 | 11,02 | 67 | 12,03 | 1516 | 489 |  |
| SLC19A1 ---- candidate | rs1051266 | G/G | 1.00 (.-.) | . | 0.16 | 0.91 | 0.19 | 370 | 31,12 | 171 | 30,70 | 1516 | 489 |  |
| SLC19A1 ---- candidate |  | G/A | 0.91 (0.74-1.12) | 0.38 | . | . | . | 590 | 49,62 | 280 | 50,27 | 1516 | 489 |  |
| SLC19A1 ---- candidate |  | A/A | 0.83 (0.64-1.08) | 0.17 | . | . | . | 229 | 19,26 | 106 | 19,03 | 1516 | 489 |  |
| SLC19A1 ---- candidate literature | rs1131596 | T/T | 1.00 (.-.) | . | 0.16 | 0.91 | 0.19 | 371 | 31,20 | 171 | 30,70 | 1516 | 489 |  |
| SLC19A1 ---- candidate literature |  | T/C | 0.92 (0.75-1.13) | 0.41 | . | . | . | 587 | 49,37 | 280 | 50,27 | 1516 | 489 |  |
| SLC19A1 ---- candidate literature |  | C/C | 0.83 (0.64-1.08) | 0.17 | . | . | . | 231 | 19,43 | 106 | 19,03 | 1516 | 489 |  |
| SLC19A1 ---- tag | rs12483553 | G/G | 1.00 (.-.) | . | 0.16 | 0.91 | 0.19 | 969 | 81,50 | 434 | 77,92 | 1516 | 489 | low_count |
| SLC19A1 ---- tag |  | G/A | 1.18 (0.95-1.47) | 0.13 | . | . | . | 208 | 17,49 | 118 | 21,18 | 1516 | 489 |  |
| SLC19A1 ---- tag |  | A/A | 1.08 (0.40-2.91) | 0.89 | . | . | . | 12 | 1,01 | 5 | 0,90 | 1516 | 489 |  |
| SLC19A1 ---- candidate literature | rs12659 | C/C | 1.00 (.-.) | . | 0.10 | 0.80 | 0.19 | 383 | 32,21 | 180 | 32,32 | 1516 | 489 |  |
| SLC19A1 ---- candidate literature |  | C/T | 0.91 (0.74-1.11) | 0.35 | . | . | . | 577 | 48,53 | 272 | 48,83 | 1516 | 489 |  |
| SLC19A1 ---- candidate literature |  | T/T | 0.80 (0.62-1.04) | 0.10 | . | . | . | 229 | 19,26 | 105 | 18,85 | 1516 | 489 |  |
| SLC19A1 ---- tag | rs3788190 | G/G | 1.00 (.-.) | . | 0.16 | 0.91 | 0.19 | 368 | 30,95 | 164 | 29,44 | 1516 | 489 |  |
| SLC19A1 ---- tag |  | G/A | 0.93 (0.76-1.14) | 0.50 | . | . | . | 574 | 48,28 | 282 | 50,63 | 1516 | 489 |  |
| SLC19A1 ---- tag |  | A/A | 0.83 (0.64-1.07) | 0.15 | . | . | . | 247 | 20,77 | 111 | 19,93 | 1516 | 489 |  |
| SLC19A1 ---- tag | rs3788205 | C/C | 1.00 (.-.) | . | 0.62 | 0.96 | 0.62 | 570 | 47,94 | 301 | 54,04 | 1516 | 489 |  |
| SLC19A1 ---- tag |  | C/T | 0.92 (0.76-1.11) | 0.40 | . | . | . | 529 | 44,49 | 213 | 38,24 | 1516 | 489 |  |
| SLC19A1 ---- tag |  | T/T | 1.00 (0.70-1.43) | 0.99 | . | . | . | 90 | 7,57 | 43 | 7,72 | 1516 | 489 |  |
| SLC19A1 ---- tag | rs7279664 | G/G | 1.00 (.-.) | . | 0.06 | 0.77 | 0.19 | 458 | 38,52 | 230 | 41,29 | 1516 | 489 |  |
| SLC19A1 ---- tag |  | G/T | 0.90 (0.74-1.09) | 0.26 | . | . | . | 557 | 46,85 | 257 | 46,14 | 1516 | 489 |  |
| SLC19A1 ---- tag |  | T/T | 0.76 (0.57-1.02) | 0.07 | . | . | . | 174 | 14,63 | 70 | 12,57 | 1516 | 489 |  |
| SLC29A1 ---- NA | rs1057985 | C/C | 1.00 (.-.) | . | 0.10 | 0.80 | 0.25 | 513 | 43,15 | 236 | 42,37 | 1516 | 489 |  |
| SLC29A1 ---- NA |  | C/T | 1.13 (0.93-1.37) | 0.22 | . | . | . | 530 | 44,58 | 249 | 44,70 | 1516 | 489 |  |
| SLC29A1 ---- NA |  | T/T | 1.24 (0.93-1.66) | 0.14 | . | . | . | 146 | 12,28 | 72 | 12,93 | 1516 | 489 |  |
| SLC29A1 ---- NA | rs6458375 | C/C | 1.00 (.-.) | . | 0.11 | 0.80 | 0.25 | 663 | 55,76 | 322 | 57,81 | 1516 | 489 |  |
| SLC29A1 ---- NA |  | C/T | 0.80 (0.66-0.97) | 0.02 | . | . | . | 450 | 37,85 | 203 | 36,45 | 1516 | 489 |  |
| SLC29A1 ---- NA |  | T/T | 0.99 (0.67-1.44) | 0.94 | . | . | . | 76 | 6,39 | 32 | 5,75 | 1516 | 489 |  |
| SLC29A1 ---- NA | rs666462 | C/C | 1.00 (.-.) | . | 0.74 | 0.97 | 0.89 | 322 | 27,08 | 160 | 28,73 | 1516 | 489 |  |
| SLC29A1 ---- NA |  | C/T | 0.96 (0.78-1.19) | 0.70 | . | . | . | 601 | 50,55 | 263 | 47,22 | 1516 | 489 |  |
| SLC29A1 ---- NA |  | T/T | 1.05 (0.82-1.35) | 0.71 | . | . | . | 266 | 22,37 | 134 | 24,06 | 1516 | 489 |  |
| SLC29A1 ---- NA | rs6905285 | A/A | 1.00 (.-.) | . | 0.99 | 0.99 | 0.99 | 443 | 37,26 | 195 | 35,01 | 1516 | 489 |  |
| SLC29A1 ---- NA |  | A/T | 0.90 (0.73-1.09) | 0.28 | . | . | . | 566 | 47,60 | 266 | 47,76 | 1516 | 489 |  |
| SLC29A1 ---- NA |  | T/T | 1.05 (0.81-1.36) | 0.73 | . | . | . | 180 | 15,14 | 96 | 17,24 | 1516 | 489 |  |
| SLC29A1 ---- NA | rs693955 | G/G | 1.00 (.-.) | . | 0.04 | 0.73 | 0.25 | 789 | 66,36 | 366 | 65,71 | 1516 | 489 |  |
| SLC29A1 ---- NA |  | G/T | 1.19 (0.97-1.44) | 0.09 | . | . | . | 357 | 30,03 | 167 | 29,98 | 1516 | 489 |  |
| SLC29A1 ---- NA |  | T/T | 1.42 (0.89-2.27) | 0.15 | . | . | . | 43 | 3,62 | 24 | 4,31 | 1516 | 489 |  |
| SLC29A1 ---- NA | rs747199 | C/C | 1.00 (.-.) | . | 0.76 | 0.97 | 0.89 | 783 | 65,85 | 349 | 62,66 | 1516 | 489 |  |
| SLC29A1 ---- NA |  | C/G | 1.07 (0.88-1.30) | 0.49 | . | . | . | 361 | 30,36 | 187 | 33,57 | 1516 | 489 |  |
| SLC29A1 ---- NA |  | G/G | 0.91 (0.56-1.49) | 0.71 | . | . | . | 45 | 3,78 | 21 | 3,77 | 1516 | 489 |  |
| SLC29A1 ---- NA | rs9357436 | G/G | 1.00 (.-.) | . | 0.50 | 0.96 | 0.88 | 842 | 70,82 | 384 | 68,94 | 1516 | 489 |  |
| SLC29A1 ---- NA |  | G/A | 1.16 (0.95-1.42) | 0.14 | . | . | . | 315 | 26,49 | 159 | 28,55 | 1516 | 489 |  |
| SLC29A1 ---- NA |  | A/A | 0.77 (0.42-1.41) | 0.39 | . | . | . | 32 | 2,69 | 14 | 2,51 | 1516 | 489 |  |
| TCN2 ---- tag | rs10418 | C/C | 1.00 (.-.) | . | 0.92 | 0.98 | 0.96 | 686 | 57,70 | 326 | 58,53 | 1516 | 489 |  |
| TCN2 ---- tag |  | C/T | 1.01 (0.83-1.22) | 0.93 | . | . | . | 442 | 37,17 | 205 | 36,80 | 1516 | 489 |  |
| TCN2 ---- tag |  | T/T | 1.02 (0.66-1.56) | 0.94 | . | . | . | 61 | 5,13 | 26 | 4,67 | 1516 | 489 |  |
| TCN2 ---- candidate/singleton | rs1131603 | T/T | 1.00 (.-.) | . | 0.44 | 0.96 | 0.82 | 1058 | 88,98 | 487 | 87,43 | 1516 | 489 | low_count |
| TCN2 ---- candidate/singleton |  | T/C | 1.04 (0.79-1.39) | 0.77 | . | . | . | 127 | 10,68 | 65 | 11,67 | 1516 | 489 |  |
| TCN2 ---- candidate/singleton |  | C/C | 1.97 (0.73-5.37) | 0.18 | . | . | . | 4 | 0,34 | 5 | 0,90 | 1516 | 489 |  |
| TCN2 ---- tag | rs1544468 | A/A | 1.00 (.-.) | . | 0.57 | 0.96 | 0.82 | 300 | 25,23 | 137 | 24,60 | 1516 | 489 |  |
| TCN2 ---- tag |  | A/G | 1.07 (0.86-1.33) | 0.56 | . | . | . | 600 | 50,46 | 275 | 49,37 | 1516 | 489 |  |
| TCN2 ---- tag |  | G/G | 1.08 (0.84-1.38) | 0.57 | . | . | . | 289 | 24,31 | 145 | 26,03 | 1516 | 489 |  |
| TCN2 ---- candidate/tag | rs1801198 | C/C | 1.00 (.-.) | . | 0.33 | 0.96 | 0.82 | 352 | 29,60 | 175 | 31,42 | 1516 | 489 |  |
| TCN2 ---- candidate/tag |  | C/G | 0.96 (0.78-1.18) | 0.71 | . | . | . | 587 | 49,37 | 269 | 48,29 | 1516 | 489 |  |
| TCN2 ---- candidate/tag |  | G/G | 0.88 (0.68-1.13) | 0.32 | . | . | . | 250 | 21,03 | 113 | 20,29 | 1516 | 489 |  |
| TCN2 ---- tag | rs4820872 | G/G | 1.00 (.-.) | . | 0.70 | 0.97 | 0.91 | 432 | 36,33 | 214 | 38,42 | 1516 | 489 |  |
| TCN2 ---- tag |  | G/A | 0.99 (0.81-1.22) | 0.96 | . | . | . | 532 | 44,74 | 237 | 42,55 | 1516 | 489 |  |
| TCN2 ---- tag |  | A/A | 0.95 (0.74-1.21) | 0.67 | . | . | . | 225 | 18,92 | 106 | 19,03 | 1516 | 489 |  |
| TCN2 ---- tag | rs4820874 | A/A | 1.00 (.-.) | . | 0.27 | 0.95 | 0.82 | 833 | 70,06 | 409 | 73,43 | 1516 | 489 |  |
| TCN2 ---- tag |  | A/G | 0.95 (0.77-1.18) | 0.66 | . | . | . | 322 | 27,08 | 134 | 24,06 | 1516 | 489 |  |
| TCN2 ---- tag |  | G/G | 0.67 (0.36-1.23) | 0.20 | . | . | . | 34 | 2,86 | 14 | 2,51 | 1516 | 489 |  |
| TCN2 ---- tag | rs4820886 | T/T | 1.00 (.-.) | . | 0.96 | 0.99 | 0.96 | 949 | 79,81 | 453 | 81,33 | 1516 | 489 | low_count |
| TCN2 ---- tag |  | T/G | 1.04 (0.82-1.32) | 0.74 | . | . | . | 222 | 18,67 | 95 | 17,06 | 1516 | 489 |  |
| TCN2 ---- tag |  | G/G | 0.85 (0.40-1.81) | 0.68 | . | . | . | 18 | 1,51 | 9 | 1,62 | 1516 | 489 |  |
| TCN2 ---- candidate | rs4820889 | G/G | 1.00 (.-.) | . | 0.43 | 0.96 | 0.82 | 1109 | 93,27 | 522 | 93,72 | 1516 | 489 |  |
| TCN2 ---- candidate |  | G/A | 0.86 (0.60-1.24) | 0.43 | . | . | . | 80 | 6,73 | 35 | 6,28 | 1516 | 489 |  |
| TCN2 ---- tag | rs5997711 | C/C | 1.00 (.-.) | . | 0.46 | 0.96 | 0.82 | 383 | 32,21 | 192 | 34,47 | 1516 | 489 |  |
| TCN2 ---- tag |  | C/T | 0.98 (0.80-1.19) | 0.81 | . | . | . | 586 | 49,29 | 260 | 46,68 | 1516 | 489 |  |
| TCN2 ---- tag |  | T/T | 0.90 (0.70-1.17) | 0.44 | . | . | . | 220 | 18,50 | 105 | 18,85 | 1516 | 489 |  |
| TCN2 ---- tag | rs740234 | T/T | 1.00 (.-.) | . | 0.53 | 0.96 | 0.82 | 784 | 65,94 | 347 | 62,30 | 1516 | 489 |  |
| TCN2 ---- tag |  | T/C | 1.02 (0.84-1.23) | 0.87 | . | . | . | 369 | 31,03 | 185 | 33,21 | 1516 | 489 |  |
| TCN2 ---- tag |  | C/C | 1.25 (0.78-2.01) | 0.35 | . | . | . | 36 | 3,03 | 25 | 4,49 | 1516 | 489 |  |
| TCN2 ---- tag | rs740235 | G/G | 1.00 (.-.) | . | 0.52 | 0.96 | 0.82 | 407 | 34,23 | 177 | 31,78 | 1516 | 489 |  |
| TCN2 ---- tag |  | G/A | 1.12 (0.91-1.38) | 0.27 | . | . | . | 574 | 48,28 | 276 | 49,55 | 1516 | 489 |  |
| TCN2 ---- tag |  | A/A | 1.06 (0.82-1.38) | 0.66 | . | . | . | 208 | 17,49 | 104 | 18,67 | 1516 | 489 |  |
| TCN2 ---- candidate/singleton | rs9606756 | A/A | 1.00 (.-.) | . | 0.57 | 0.96 | 0.82 | 949 | 79,81 | 443 | 79,53 | 1516 | 489 | low_count |
| TCN2 ---- candidate/singleton |  | A/G | 1.11 (0.88-1.39) | 0.38 | . | . | . | 218 | 18,33 | 105 | 18,85 | 1516 | 489 |  |
| TCN2 ---- candidate/singleton |  | G/G | 0.87 (0.39-1.96) | 0.74 | . | . | . | 22 | 1,85 | 9 | 1,62 | 1516 | 489 |  |
| TCN2 ---- candidate | rs9621049 | C/C | 1.00 (.-.) | . | 0.96 | 0.99 | 0.96 | 949 | 79,81 | 453 | 81,33 | 1516 | 489 | low_count |
| TCN2 ---- candidate |  | C/T | 1.04 (0.82-1.32) | 0.74 | . | . | . | 222 | 18,67 | 95 | 17,06 | 1516 | 489 |  |
| TCN2 ---- candidate |  | T/T | 0.85 (0.40-1.81) | 0.68 | . | . | . | 18 | 1,51 | 9 | 1,62 | 1516 | 489 |  |
| TK1 ---- NA | rs1065769 | G/G | 1.00 (.-.) | . | 0.92 | 0.98 | 0.93 | 565 | 47,52 | 252 | 45,24 | 1516 | 489 |  |
| TK1 ---- NA |  | G/A | 1.16 (0.96-1.40) | 0.13 | . | . | . | 503 | 42,30 | 253 | 45,42 | 1516 | 489 |  |
| TK1 ---- NA |  | A/A | 0.87 (0.63-1.20) | 0.40 | . | . | . | 121 | 10,18 | 52 | 9,34 | 1516 | 489 |  |
| TK1 ---- NA | rs12232476 | G/G | 1.00 (.-.) | . | 0.78 | 0.97 | 0.93 | 999 | 84,02 | 467 | 83,84 | 1516 | 489 | low_count |
| TK1 ---- NA |  | G/A | 1.04 (0.81-1.33) | 0.76 | . | . | . | 182 | 15,31 | 86 | 15,44 | 1516 | 489 |  |
| TK1 ---- NA |  | A/A | 0.99 (0.32-3.11) | 0.99 | . | . | . | 8 | 0,67 | 4 | 0,72 | 1516 | 489 |  |
| TK1 ---- tag | rs16970907 | G/G | 1.00 (.-.) | . | 0.49 | 0.96 | 0.93 | 1027 | 86,38 | 484 | 86,89 | 1516 | 489 | low_count |
| TK1 ---- tag |  | G/C | 1.06 (0.81-1.39) | 0.68 | . | . | . | 154 | 12,95 | 73 | 13,11 | 1516 | 489 |  |
| TK1 ---- tag |  | C/C | 0.00 (0.00-8E189) | 0.96 | . | . | . | 8 | 0,67 | 0 | 0,00 | 1516 | 489 |  |
| TK1 ---- tag | rs1811086 | C/C | 1.00 (.-.) | . | 0.49 | 0.96 | 0.93 | 1106 | 93,02 | 516 | 92,64 | 1516 | 489 | low_count |
| TK1 ---- tag |  | C/T | 0.85 (0.59-1.23) | 0.38 | . | . | . | 82 | 6,90 | 40 | 7,18 | 1516 | 489 |  |
| TK1 ---- tag |  | T/T | 2.18 (0.30-16.08) | 0.45 | . | . | . | 1 | 0,08 | 1 | 0,18 | 1516 | 489 |  |
| TK1 ---- tag | rs2292235 | C/C | 1.00 (.-.) | . | 0.30 | 0.95 | 0.93 | 375 | 31,54 | 183 | 32,85 | 1516 | 489 |  |
| TK1 ---- tag |  | C/A | 1.04 (0.85-1.27) | 0.70 | . | . | . | 559 | 47,01 | 268 | 48,11 | 1516 | 489 |  |
| TK1 ---- tag |  | A/A | 0.85 (0.65-1.10) | 0.21 | . | . | . | 255 | 21,45 | 106 | 19,03 | 1516 | 489 |  |
| TK1 ---- tag | rs2854701 | A/A | 1.00 (.-.) | . | 0.84 | 0.98 | 0.93 | 485 | 40,79 | 208 | 37,34 | 1516 | 489 |  |
| TK1 ---- tag |  | A/G | 1.25 (1.04-1.52) | 0.02 | . | . | . | 540 | 45,42 | 282 | 50,63 | 1516 | 489 |  |
| TK1 ---- tag |  | G/G | 0.86 (0.64-1.17) | 0.35 | . | . | . | 164 | 13,79 | 67 | 12,03 | 1516 | 489 |  |
| TK1 ---- tag | rs2854702 | G/G | 1.00 (.-.) | . | 0.93 | 0.98 | 0.93 | 918 | 77,21 | 425 | 76,30 | 1516 | 489 | low_count |
| TK1 ---- tag |  | G/A | 0.97 (0.78-1.21) | 0.77 | . | . | . | 252 | 21,19 | 122 | 21,90 | 1516 | 489 |  |
| TK1 ---- tag |  | A/A | 1.13 (0.56-2.29) | 0.73 | . | . | . | 19 | 1,60 | 10 | 1,80 | 1516 | 489 |  |
| TK1 ---- tag | rs9897765 | G/G | 1.00 (.-.) | . | 0.88 | 0.98 | 0.93 | 639 | 53,74 | 288 | 51,71 | 1516 | 489 |  |
| TK1 ---- tag |  | G/A | 1.06 (0.87-1.28) | 0.58 | . | . | . | 458 | 38,52 | 230 | 41,29 | 1516 | 489 |  |
| TK1 ---- tag |  | A/A | 0.88 (0.61-1.26) | 0.49 | . | . | . | 92 | 7,74 | 39 | 7,00 | 1516 | 489 |  |
| TYMP ---- NA | rs131815 | G/G | 1.00 (.-.) | . | 0.18 | 0.92 | 0.28 | 631 | 53,07 | 294 | 52,78 | 1516 | 489 |  |
| TYMP ---- NA |  | G/A | 1.15 (0.95-1.39) | 0.14 | . | . | . | 459 | 38,60 | 220 | 39,50 | 1516 | 489 |  |
| TYMP ---- NA |  | A/A | 1.13 (0.80-1.59) | 0.49 | . | . | . | 99 | 8,33 | 43 | 7,72 | 1516 | 489 |  |
| TYMP ---- tag | rs131816 | A/A | 1.00 (.-.) | . | 0.02 | 0.73 | 0.14 | 705 | 59,29 | 350 | 62,84 | 1516 | 489 |  |
| TYMP ---- tag |  | A/G | 0.82 (0.67-1.00) | 0.05 | . | . | . | 412 | 34,65 | 174 | 31,24 | 1516 | 489 |  |
| TYMP ---- tag |  | G/G | 0.73 (0.50-1.09) | 0.13 | . | . | . | 72 | 6,06 | 33 | 5,92 | 1516 | 489 |  |
| TYMP ---- NA | rs131817 | C/C | 1.00 (.-.) | . | 0.40 | 0.96 | 0.40 | 380 | 31,96 | 186 | 33,39 | 1516 | 489 |  |
| TYMP ---- NA |  | C/T | 1.07 (0.88-1.31) | 0.50 | . | . | . | 580 | 48,78 | 266 | 47,76 | 1516 | 489 |  |
| TYMP ---- NA |  | T/T | 1.11 (0.85-1.44) | 0.44 | . | . | . | 229 | 19,26 | 105 | 18,85 | 1516 | 489 |  |
| TYMP ---- NA | rs140521 | T/T | 1.00 (.-.) | . | 0.08 | 0.79 | 0.24 | 604 | 50,80 | 294 | 52,78 | 1516 | 489 |  |
| TYMP ---- NA |  | T/G | 0.87 (0.72-1.05) | 0.15 | . | . | . | 493 | 41,46 | 220 | 39,50 | 1516 | 489 |  |
| TYMP ---- NA |  | G/G | 0.79 (0.56-1.11) | 0.18 | . | . | . | 92 | 7,74 | 43 | 7,72 | 1516 | 489 |  |
| TYMP ---- NA | rs140522 | G/G | 1.00 (.-.) | . | 0.17 | 0.91 | 0.28 | 533 | 44,83 | 259 | 46,50 | 1516 | 489 |  |
| TYMP ---- NA |  | G/A | 0.86 (0.71-1.04) | 0.12 | . | . | . | 534 | 44,91 | 242 | 43,45 | 1516 | 489 |  |
| TYMP ---- NA |  | A/A | 0.87 (0.64-1.19) | 0.40 | . | . | . | 122 | 10,26 | 56 | 10,05 | 1516 | 489 |  |
| TYMP ---- NA | rs140524 | G/G | 1.00 (.-.) | . | 0.34 | 0.96 | 0.40 | 809 | 68,04 | 360 | 64,63 | 1516 | 489 |  |
| TYMP ---- NA |  | G/A | 1.09 (0.89-1.32) | 0.41 | . | . | . | 343 | 28,85 | 176 | 31,60 | 1516 | 489 |  |
| TYMP ---- NA |  | A/A | 1.15 (0.72-1.84) | 0.55 | . | . | . | 37 | 3,11 | 21 | 3,77 | 1516 | 489 |  |
| TYMS ---- candidate literature | rs1001761 | C/C | 1.00 (.-.) | . | 0.04 | 0.73 | 0.11 | 344 | 28,93 | 182 | 32,68 | 1516 | 489 |  |
| TYMS ---- candidate literature |  | C/T | 0.84 (0.68-1.02) | 0.08 | . | . | . | 604 | 50,80 | 280 | 50,27 | 1516 | 489 |  |
| TYMS ---- candidate literature |  | T/T | 0.77 (0.59-1.00) | 0.05 | . | . | . | 241 | 20,27 | 95 | 17,06 | 1516 | 489 |  |
| TYMS ---- candidate literature/tag | rs10502289 | T/T | 1.00 (.-.) | . | 0.26 | 0.95 | 0.33 | 739 | 62,15 | 362 | 64,99 | 1516 | 489 |  |
| TYMS ---- candidate literature/tag |  | T/A | 0.89 (0.73-1.09) | 0.26 | . | . | . | 394 | 33,14 | 171 | 30,70 | 1516 | 489 |  |
| TYMS ---- candidate literature/tag |  | A/A | 0.88 (0.56-1.38) | 0.58 | . | . | . | 56 | 4,71 | 24 | 4,31 | 1516 | 489 |  |
| TYMS ---- tag | rs15872 | C/C | 1.00 (.-.) | . | 0.06 | 0.77 | 0.12 | 545 | 45,84 | 273 | 49,01 | 1516 | 489 |  |
| TYMS ---- tag |  | C/T | 0.87 (0.72-1.04) | 0.13 | . | . | . | 522 | 43,90 | 239 | 42,91 | 1516 | 489 |  |
| TYMS ---- tag |  | T/T | 0.77 (0.54-1.08) | 0.13 | . | . | . | 122 | 10,26 | 45 | 8,08 | 1516 | 489 |  |
| TYMS ---- tag | rs2244500 | T/T | 1.00 (.-.) | . | 0.03 | 0.73 | 0.11 | 344 | 28,93 | 183 | 32,85 | 1516 | 489 |  |
| TYMS ---- tag |  | T/C | 0.83 (0.68-1.02) | 0.07 | . | . | . | 604 | 50,80 | 279 | 50,09 | 1516 | 489 |  |
| TYMS ---- tag |  | C/C | 0.76 (0.58-0.99) | 0.04 | . | . | . | 241 | 20,27 | 95 | 17,06 | 1516 | 489 |  |
| TYMS ---- tag | rs2741182 | G/G | 1.00 (.-.) | . | 0.98 | 0.99 | 0.98 | 722 | 60,72 | 340 | 61,04 | 1516 | 489 |  |
| TYMS ---- tag |  | G/C | 1.00 (0.82-1.21) | 1.00 | . | . | . | 408 | 34,31 | 184 | 33,03 | 1516 | 489 |  |
| TYMS ---- tag |  | C/C | 0.99 (0.68-1.46) | 0.98 | . | . | . | 59 | 4,96 | 33 | 5,92 | 1516 | 489 |  |
| TYMS ---- candidate literature | rs2847149 | G/G | 1.00 (.-.) | . | 0.04 | 0.73 | 0.11 | 344 | 28,93 | 182 | 32,68 | 1516 | 489 |  |
| TYMS ---- candidate literature |  | G/A | 0.84 (0.68-1.02) | 0.08 | . | . | . | 604 | 50,80 | 280 | 50,27 | 1516 | 489 |  |
| TYMS ---- candidate literature |  | A/A | 0.77 (0.59-1.00) | 0.05 | . | . | . | 241 | 20,27 | 95 | 17,06 | 1516 | 489 |  |
| TYMS ---- candidate literature | rs2853533 | G/G | 1.00 (.-.) | . | 0.50 | 0.96 | 0.57 | 899 | 75,61 | 409 | 73,43 | 1516 | 489 | low_count |
| TYMS ---- candidate literature |  | G/C | 1.00 (0.81-1.23) | 1.00 | . | . | . | 268 | 22,54 | 141 | 25,31 | 1516 | 489 |  |
| TYMS ---- candidate literature |  | C/C | 0.54 (0.22-1.30) | 0.17 | . | . | . | 22 | 1,85 | 7 | 1,26 | 1516 | 489 |  |
| TYMS ---- tag | rs495139 | C/C | 1.00 (.-.) | . | 0.07 | 0.79 | 0.12 | 416 | 34,99 | 162 | 29,08 | 1516 | 489 |  |
| TYMS ---- tag |  | C/G | 1.48 (1.20-1.82) | <.001 | . | . | . | 568 | 47,77 | 300 | 53,86 | 1516 | 489 |  |
| TYMS ---- tag |  | G/G | 1.17 (0.89-1.53) | 0.27 | . | . | . | 205 | 17,24 | 95 | 17,06 | 1516 | 489 |  |
| TYMS ---- candidate literature | rs502396 | T/T | 1.00 (.-.) | . | 0.18 | 0.92 | 0.27 | 336 | 28,26 | 181 | 32,50 | 1516 | 489 |  |
| TYMS ---- candidate literature |  | T/C | 0.88 (0.72-1.08) | 0.22 | . | . | . | 599 | 50,38 | 272 | 48,83 | 1516 | 489 |  |
| TYMS ---- candidate literature |  | C/C | 0.85 (0.65-1.10) | 0.22 | . | . | . | 254 | 21,36 | 104 | 18,67 | 1516 | 489 |  |
| UMPH2 ---- tag | rs2291028 | A/A | 1.00 (.-.) | . | 0.25 | 0.95 | 0.74 | 518 | 43,57 | 224 | 40,22 | 1516 | 489 |  |
| UMPH2 ---- tag |  | A/G | 1.09 (0.89-1.32) | 0.40 | . | . | . | 514 | 43,23 | 264 | 47,40 | 1516 | 489 |  |
| UMPH2 ---- tag |  | G/G | 1.17 (0.87-1.57) | 0.29 | . | . | . | 157 | 13,20 | 69 | 12,39 | 1516 | 489 |  |
| UMPH2 ---- NA | rs4789143 | A/A | 1.00 (.-.) | . | 0.95 | 0.99 | 0.95 | 915 | 76,96 | 440 | 78,99 | 1516 | 489 | low_count |
| UMPH2 ---- NA |  | A/G | 0.99 (0.79-1.23) | 0.91 | . | . | . | 257 | 21,61 | 112 | 20,11 | 1516 | 489 |  |
| UMPH2 ---- NA |  | G/G | 1.29 (0.48-3.47) | 0.62 | . | . | . | 17 | 1,43 | 5 | 0,90 | 1516 | 489 |  |
| UMPH2 ---- NA | rs750844 | G/G | 1.00 (.-.) | . | 0.68 | 0.97 | 0.95 | 602 | 50,63 | 273 | 49,01 | 1516 | 489 |  |
| UMPH2 ---- NA |  | G/A | 1.04 (0.86-1.25) | 0.71 | . | . | . | 487 | 40,96 | 239 | 42,91 | 1516 | 489 |  |
| UMPH2 ---- NA |  | A/A | 1.05 (0.75-1.48) | 0.76 | . | . | . | 100 | 8,41 | 45 | 8,08 | 1516 | 489 |  |
| UMPK ---- tag | rs11582877 | C/C | 1.00 (.-.) | . | 0.88 | 0.98 | 0.93 | 874 | 73,51 | 401 | 71,99 | 1516 | 489 |  |
| UMPK ---- tag |  | C/T | 1.09 (0.89-1.33) | 0.42 | . | . | . | 278 | 23,38 | 144 | 25,85 | 1516 | 489 |  |
| UMPK ---- tag |  | T/T | 0.75 (0.40-1.42) | 0.38 | . | . | . | 37 | 3,11 | 12 | 2,15 | 1516 | 489 |  |
| UMPK ---- tag | rs2622903 | A/A | 1.00 (.-.) | . | 0.81 | 0.98 | 0.93 | 582 | 48,95 | 276 | 49,55 | 1516 | 489 |  |
| UMPK ---- tag |  | A/G | 1.13 (0.94-1.36) | 0.20 | . | . | . | 476 | 40,03 | 234 | 42,01 | 1516 | 489 |  |
| UMPK ---- tag |  | G/G | 0.79 (0.55-1.12) | 0.19 | . | . | . | 131 | 11,02 | 47 | 8,44 | 1516 | 489 |  |
| UMPK ---- tag | rs2820989 | C/C | 1.00 (.-.) | . | 0.93 | 0.98 | 0.93 | 359 | 30,19 | 173 | 31,06 | 1516 | 489 |  |
| UMPK ---- tag |  | C/G | 1.08 (0.88-1.33) | 0.46 | . | . | . | 569 | 47,86 | 271 | 48,65 | 1516 | 489 |  |
| UMPK ---- tag |  | G/G | 0.97 (0.75-1.26) | 0.82 | . | . | . | 261 | 21,95 | 113 | 20,29 | 1516 | 489 |  |
| UMPK ---- tag | rs6660321 | A/A | 1.00 (.-.) | . | 0.86 | 0.98 | 0.93 | 902 | 75,86 | 417 | 74,87 | 1516 | 489 | low_count |
| UMPK ---- tag |  | A/C | 1.04 (0.84-1.28) | 0.74 | . | . | . | 260 | 21,87 | 130 | 23,34 | 1516 | 489 |  |
| UMPK ---- tag |  | C/C | 0.74 (0.36-1.49) | 0.40 | . | . | . | 27 | 2,27 | 10 | 1,80 | 1516 | 489 |  |
| UMPK ---- tag | rs6690084 | T/T | 1.00 (.-.) | . | 0.42 | 0.96 | 0.93 | 1028 | 86,46 | 474 | 85,10 | 1516 | 489 | low_count |
| UMPK ---- tag |  | T/C | 1.18 (0.92-1.52) | 0.20 | . | . | . | 152 | 12,78 | 79 | 14,18 | 1516 | 489 |  |
| UMPK ---- tag |  | C/C | 0.68 (0.22-2.13) | 0.51 | . | . | . | 9 | 0,76 | 4 | 0,72 | 1516 | 489 |  |
| UMPS ---- NA | rs1162 | A/A | 1.00 (.-.) | . | 0.09 | 0.79 | 0.51 | 555 | 46,68 | 257 | 46,14 | 1516 | 489 |  |
| UMPS ---- NA |  | A/G | 1.05 (0.87-1.28) | 0.60 | . | . | . | 515 | 43,31 | 229 | 41,11 | 1516 | 489 |  |
| UMPS ---- NA |  | G/G | 1.34 (1.00-1.78) | 0.05 | . | . | . | 119 | 10,01 | 71 | 12,75 | 1516 | 489 |  |
| UMPS ---- tag | rs13146 | C/C | 1.00 (.-.) | . | 0.54 | 0.96 | 0.79 | 822 | 69,13 | 385 | 69,12 | 1516 | 489 |  |
| UMPS ---- tag |  | C/T | 0.96 (0.78-1.17) | 0.68 | . | . | . | 332 | 27,92 | 148 | 26,57 | 1516 | 489 |  |
| UMPS ---- tag |  | T/T | 1.49 (0.94-2.36) | 0.09 | . | . | . | 35 | 2,94 | 24 | 4,31 | 1516 | 489 |  |
| UMPS ---- tag | rs16835902 | C/C | 1.00 (.-.) | . | 0.90 | 0.98 | 0.90 | 388 | 32,63 | 182 | 32,68 | 1516 | 489 |  |
| UMPS ---- tag |  | C/G | 0.96 (0.78-1.17) | 0.69 | . | . | . | 559 | 47,01 | 279 | 50,09 | 1516 | 489 |  |
| UMPS ---- tag |  | G/G | 1.04 (0.80-1.35) | 0.79 | . | . | . | 242 | 20,35 | 96 | 17,24 | 1516 | 489 |  |
| UMPS ---- tag | rs17282057 | T/T | 1.00 (.-.) | . | 0.64 | 0.96 | 0.79 | 907 | 76,28 | 421 | 75,58 | 1516 | 489 |  |
| UMPS ---- tag |  | T/C | 0.98 (0.79-1.22) | 0.85 | . | . | . | 263 | 22,12 | 125 | 22,44 | 1516 | 489 |  |
| UMPS ---- tag |  | C/C | 1.68 (0.86-3.29) | 0.13 | . | . | . | 19 | 1,60 | 11 | 1,97 | 1516 | 489 |  |
| UMPS ---- tag | rs606552 | A/A | 1.00 (.-.) | . | 0.33 | 0.96 | 0.79 | 601 | 50,55 | 306 | 54,94 | 1516 | 489 |  |
| UMPS ---- tag |  | A/G | 0.88 (0.73-1.07) | 0.21 | . | . | . | 487 | 40,96 | 212 | 38,06 | 1516 | 489 |  |
| UMPS ---- tag |  | G/G | 0.94 (0.67-1.33) | 0.73 | . | . | . | 101 | 8,49 | 39 | 7,00 | 1516 | 489 |  |
| UMPS ---- tag | rs694897 | C/C | 1.00 (.-.) | . | 0.66 | 0.96 | 0.79 | 510 | 42,89 | 218 | 39,14 | 1516 | 489 |  |
| UMPS ---- tag |  | C/G | 0.99 (0.81-1.20) | 0.89 | . | . | . | 531 | 44,66 | 258 | 46,32 | 1516 | 489 |  |
| UMPS ---- tag |  | G/G | 1.09 (0.83-1.44) | 0.53 | . | . | . | 148 | 12,45 | 81 | 14,54 | 1516 | 489 |  |
| UNG ---- NA | rs1059262 | T/T | 1.00 (.-.) | . | 0.54 | 0.96 | 0.65 | 801 | 67,37 | 392 | 70,38 | 1516 | 489 |  |
| UNG ---- NA |  | T/G | 0.89 (0.73-1.10) | 0.28 | . | . | . | 348 | 29,27 | 147 | 26,39 | 1516 | 489 |  |
| UNG ---- NA |  | G/G | 1.10 (0.67-1.81) | 0.69 | . | . | . | 40 | 3,36 | 18 | 3,23 | 1516 | 489 |  |
| UNG ---- tag | rs2160603 | T/T | 1.00 (.-.) | . | 0.43 | 0.96 | 0.65 | 822 | 69,13 | 392 | 70,38 | 1516 | 489 |  |
| UNG ---- tag |  | T/C | 1.17 (0.95-1.43) | 0.15 | . | . | . | 322 | 27,08 | 149 | 26,75 | 1516 | 489 |  |
| UNG ---- tag |  | C/C | 0.87 (0.50-1.52) | 0.62 | . | . | . | 45 | 3,78 | 16 | 2,87 | 1516 | 489 |  |
| UNG ---- tag | rs246079 | A/A | 1.00 (.-.) | . | 0.30 | 0.95 | 0.65 | 380 | 31,96 | 175 | 31,42 | 1516 | 489 |  |
| UNG ---- tag |  | A/G | 1.15 (0.93-1.41) | 0.19 | . | . | . | 585 | 49,20 | 279 | 50,09 | 1516 | 489 |  |
| UNG ---- tag |  | G/G | 1.12 (0.86-1.47) | 0.39 | . | . | . | 224 | 18,84 | 103 | 18,49 | 1516 | 489 |  |
| UNG ---- NA | rs246085 | T/T | 1.00 (.-.) | . | 0.88 | 0.98 | 0.88 | 1052 | 88,48 | 493 | 88,51 | 1516 | 489 | low_count |
| UNG ---- NA |  | T/C | 1.01 (0.76-1.34) | 0.96 | . | . | . | 134 | 11,27 | 63 | 11,31 | 1516 | 489 |  |
| UNG ---- NA |  | C/C | 1.62 (0.22-11.71) | 0.63 | . | . | . | 3 | 0,25 | 1 | 0,18 | 1516 | 489 |  |
| UNG ---- NA | rs2569987 | A/A | 1.00 (.-.) | . | 0.24 | 0.95 | 0.65 | 814 | 68,46 | 389 | 69,84 | 1516 | 489 |  |
| UNG ---- NA |  | A/G | 0.98 (0.80-1.20) | 0.82 | . | . | . | 336 | 28,26 | 154 | 27,65 | 1516 | 489 |  |
| UNG ---- NA |  | G/G | 0.62 (0.35-1.10) | 0.10 | . | . | . | 39 | 3,28 | 14 | 2,51 | 1516 | 489 |  |
| UNG ---- tag | rs3219243 | T/T | 1.00 (.-.) | . | 0.51 | 0.96 | 0.65 | 766 | 64,42 | 349 | 62,66 | 1516 | 489 |  |
| UNG ---- tag |  | T/C | 0.94 (0.78-1.15) | 0.57 | . | . | . | 387 | 32,55 | 179 | 32,14 | 1516 | 489 |  |
| UNG ---- tag |  | C/C | 1.49 (0.99-2.25) | 0.06 | . | . | . | 36 | 3,03 | 29 | 5,21 | 1516 | 489 |  |

| **Gene** | **SNP** | **Genotype** | **HR (95%-CI)** | **p** | **FDR__p_** | **FDR_(byGene)_p_** | **NObsUsed** | **Events** | **LowCount** |
| --- | --- | --- | --- | --- | --- | --- | --- | --- | --- |
| AARS ---- tag | rs2070203 | 1 | 1.03 (0.91-1.17) | 0.65 | 0.96 | 0.65 | 1516 | 489 |  |
| AARS ---- tag | rs34087264 | 1 | 0.95 (0.83-1.08) | 0.40 | 0.96 | 0.65 | 1516 | 489 |  |
| ABCC4 ---- tag | rs10508023 | 1 | 1.12 (0.92-1.36) | 0.26 | 0.95 | 0.86 | 1516 | 489 | low_count |
| ABCC4 ---- tag | rs1059751 | 1 | 1.05 (0.92-1.19) | 0.47 | 0.96 | 0.95 | 1516 | 489 |  |
| ABCC4 ---- tag | rs11568643 | 1 | 1.19 (0.96-1.47) | 0.11 | 0.80 | 0.66 | 1516 | 489 | low_count |
| ABCC4 ---- NA | rs11568658 | 1 | 1.16 (0.78-1.74) | 0.46 | 0.96 | 0.95 | 1516 | 489 | low_count |
| ABCC4 ---- tag | rs12864049 | 1 | 1.01 (0.84-1.22) | 0.89 | 0.98 | 0.96 | 1516 | 489 | low_count |
| ABCC4 ---- tag | rs1628382 | 1 | 1.03 (0.87-1.20) | 0.76 | 0.97 | 0.96 | 1516 | 489 |  |
| ABCC4 ---- tag | rs1678354 | 1 | 1.03 (0.90-1.18) | 0.65 | 0.96 | 0.96 | 1516 | 489 |  |
| ABCC4 ---- tag | rs1678383 | 1 | 0.92 (0.74-1.14) | 0.46 | 0.96 | 0.95 | 1516 | 489 | low_count |
| ABCC4 ---- tag | rs1678395 | 1 | 0.87 (0.68-1.11) | 0.26 | 0.95 | 0.86 | 1516 | 489 | low_count |
| ABCC4 ---- tag | rs1678405 | 1 | 1.15 (1.00-1.32) | 0.05 | 0.77 | 0.65 | 1516 | 489 |  |
| ABCC4 ---- tag | rs17189540 | 1 | 1.19 (0.93-1.52) | 0.16 | 0.91 | 0.85 | 1516 | 489 | low_count |
| ABCC4 ---- tag | rs17235152 | 1 | 1.00 (0.83-1.20) | 0.97 | 0.99 | 0.99 | 1516 | 489 | low_count |
| ABCC4 ---- tag | rs17268122 | 1 | 0.96 (0.83-1.12) | 0.63 | 0.96 | 0.96 | 1516 | 489 |  |
| ABCC4 ---- tag | rs17268170 | 1 | 1.03 (0.83-1.26) | 0.81 | 0.98 | 0.96 | 1516 | 489 | low_count |
| ABCC4 ---- tag | rs1729764 | 1 | 0.92 (0.74-1.14) | 0.44 | 0.96 | 0.95 | 1516 | 489 | low_count |
| ABCC4 ---- tag | rs1729767 | 1 | 1.05 (0.91-1.21) | 0.51 | 0.96 | 0.96 | 1516 | 489 |  |
| ABCC4 ---- tag | rs17300935 | 1 | 1.02 (0.85-1.22) | 0.85 | 0.98 | 0.96 | 1516 | 489 | low_count |
| ABCC4 ---- tag | rs1750190 | 1 | 1.01 (0.89-1.14) | 0.92 | 0.98 | 0.96 | 1516 | 489 |  |
| ABCC4 ---- tag | rs1750996 | 1 | 0.97 (0.82-1.15) | 0.71 | 0.97 | 0.96 | 1516 | 489 |  |
| ABCC4 ---- tag | rs1751025 | 1 | 1.20 (1.05-1.37) | 0.01 | 0.73 | 0.21 | 1516 | 489 |  |
| ABCC4 ---- tag | rs1751051 | 1 | 1.01 (0.88-1.16) | 0.85 | 0.98 | 0.96 | 1516 | 489 |  |
| ABCC4 ---- tag | rs1764416 | 1 | 0.86 (0.65-1.14) | 0.30 | 0.95 | 0.86 | 1516 | 489 | low_count |
| ABCC4 ---- tag | rs2274401 | 1 | 0.90 (0.77-1.06) | 0.21 | 0.92 | 0.86 | 1516 | 489 |  |
| ABCC4 ---- tag | rs2892716 | 1 | 0.99 (0.87-1.13) | 0.90 | 0.98 | 0.96 | 1516 | 489 |  |
| ABCC4 ---- tag | rs3782964 | 1 | 0.91 (0.76-1.10) | 0.33 | 0.96 | 0.86 | 1516 | 489 |  |
| ABCC4 ---- tag | rs3818494 | 1 | 1.17 (1.03-1.33) | 0.02 | 0.73 | 0.33 | 1516 | 489 |  |
| ABCC4 ---- tag | rs3864997 | 1 | 1.05 (0.92-1.19) | 0.48 | 0.96 | 0.95 | 1516 | 489 |  |
| ABCC4 ---- tag | rs4148421 | 1 | 0.96 (0.85-1.10) | 0.57 | 0.96 | 0.96 | 1516 | 489 |  |
| ABCC4 ---- tag | rs4148446 | 1 | 0.99 (0.87-1.13) | 0.92 | 0.98 | 0.96 | 1516 | 489 |  |
| ABCC4 ---- tag | rs4148455 | 1 | 1.02 (0.84-1.22) | 0.87 | 0.98 | 0.96 | 1516 | 489 | low_count |
| ABCC4 ---- tag | rs4148540 | 1 | 1.05 (0.83-1.34) | 0.67 | 0.96 | 0.96 | 1516 | 489 | low_count |
| ABCC4 ---- tag | rs4148542 | 1 | 1.00 (0.88-1.14) | 1.00 | 1.00 | 1.00 | 1516 | 489 |  |
| ABCC4 ---- tag | rs4148544 | 1 | 1.01 (0.88-1.15) | 0.91 | 0.98 | 0.96 | 1516 | 489 |  |
| ABCC4 ---- tag | rs4283094 | 1 | 1.01 (0.89-1.15) | 0.88 | 0.98 | 0.96 | 1516 | 489 |  |
| ABCC4 ---- tag | rs4636781 | 1 | 1.15 (0.97-1.36) | 0.11 | 0.80 | 0.66 | 1516 | 489 |  |
| ABCC4 ---- tag | rs4771910 | 1 | 0.88 (0.77-1.02) | 0.09 | 0.79 | 0.66 | 1516 | 489 |  |
| ABCC4 ---- tag | rs4773850 | 1 | 0.83 (0.72-0.95) | 0.01 | 0.73 | 0.21 | 1516 | 489 |  |
| ABCC4 ---- tag | rs7981095 | 1 | 0.98 (0.83-1.15) | 0.79 | 0.98 | 0.96 | 1516 | 489 |  |
| ABCC4 ---- tag | rs8001444 | 1 | 0.96 (0.84-1.10) | 0.57 | 0.96 | 0.96 | 1516 | 489 |  |
| ABCC4 ---- tag | rs931111 | 1 | 0.97 (0.83-1.14) | 0.72 | 0.97 | 0.96 | 1516 | 489 |  |
| ABCC4 ---- tag | rs943288 | 1 | 1.05 (0.87-1.26) | 0.61 | 0.96 | 0.96 | 1516 | 489 |  |
| ABCC4 ---- tag | rs943290 | 1 | 0.92 (0.79-1.06) | 0.25 | 0.95 | 0.86 | 1516 | 489 |  |
| ABCC4 ---- tag | rs9516530 | 1 | 0.93 (0.80-1.07) | 0.30 | 0.95 | 0.86 | 1516 | 489 |  |
| ABCC4 ---- tag | rs9516551 | 1 | 0.82 (0.67-1.02) | 0.07 | 0.79 | 0.66 | 1516 | 489 | low_count |
| ABCC4 ---- tag | rs9524822 | 1 | 0.92 (0.78-1.09) | 0.35 | 0.96 | 0.86 | 1516 | 489 |  |
| ABCC4 ---- tag | rs9524861 | 1 | 0.94 (0.82-1.07) | 0.34 | 0.96 | 0.86 | 1516 | 489 |  |
| ABCC4 ---- tag | rs9524902 | 1 | 0.93 (0.82-1.06) | 0.30 | 0.95 | 0.86 | 1516 | 489 |  |
| ABCC4 ---- tag | rs9556455 | 1 | 1.10 (0.92-1.31) | 0.30 | 0.95 | 0.86 | 1516 | 489 | low_count |
| ABCC4 ---- NA | rs9561778 | 1 | 0.96 (0.82-1.13) | 0.65 | 0.96 | 0.96 | 1516 | 489 |  |
| ABCC4 ---- tag | rs9561811 | 1 | 1.15 (0.98-1.35) | 0.10 | 0.80 | 0.66 | 1516 | 489 |  |
| ABCC4 ---- tag | rs9590183 | 1 | 0.91 (0.69-1.19) | 0.49 | 0.96 | 0.95 | 1516 | 489 | low_count |
| ABCC4 ---- tag | rs997777 | 1 | 1.02 (0.89-1.18) | 0.74 | 0.97 | 0.96 | 1516 | 489 |  |
| ADH1B ---- tag | rs1159918 | 1 | 0.96 (0.84-1.11) | 0.62 | 0.96 | 0.73 | 1516 | 489 |  |
| ADH1B ---- candidate literature | rs1229984 | 1 | 0.88 (0.66-1.18) | 0.39 | 0.96 | 0.67 | 1516 | 489 | low_count |
| ADH1B ---- tag | rs12507573 | 1 | 1.02 (0.90-1.16) | 0.73 | 0.97 | 0.73 | 1516 | 489 |  |
| ADH1B ---- tag | rs1693457 | 1 | 0.84 (0.71-1.01) | 0.06 | 0.77 | 0.29 | 1516 | 489 |  |
| ADH1B ---- tag | rs2066701 | 1 | 1.06 (0.93-1.21) | 0.40 | 0.96 | 0.67 | 1516 | 489 |  |
| ADH1C ---- tag | rs11936869 | 1 | 0.93 (0.80-1.07) | 0.29 | 0.95 | 0.88 | 1516 | 489 |  |
| ADH1C ---- tag | rs1229849 | 1 | 1.06 (0.91-1.23) | 0.45 | 0.96 | 0.90 | 1516 | 489 |  |
| ADH1C ---- tag | rs1229863 | 1 | 0.97 (0.81-1.16) | 0.73 | 0.97 | 0.90 | 1516 | 489 |  |
| ADH1C ---- tag | rs1229980 | 1 | 1.15 (0.88-1.51) | 0.29 | 0.95 | 0.88 | 1516 | 489 | low_count |
| ADH1C ---- candidate | rs1693482 | 1 | 0.99 (0.87-1.13) | 0.90 | 0.98 | 0.90 | 1516 | 489 |  |
| ADH1C ---- tag | rs2173201 | 1 | 0.96 (0.83-1.11) | 0.58 | 0.96 | 0.90 | 1516 | 489 |  |
| ADH1C ---- tag | rs2298753 | 1 | 0.87 (0.70-1.08) | 0.22 | 0.92 | 0.88 | 1516 | 489 | low_count |
| ADH1C ---- tag | rs2866152 | 1 | 1.02 (0.87-1.20) | 0.77 | 0.97 | 0.90 | 1516 | 489 |  |
| ADH1C ---- tag | rs904096 | 1 | 0.99 (0.86-1.13) | 0.86 | 0.98 | 0.90 | 1516 | 489 |  |
| BHMT ---- tag | rs10944 | 1 | 0.98 (0.86-1.11) | 0.74 | 0.97 | 0.87 | 1516 | 489 |  |
| BHMT ---- tag | rs12655567 | 1 | 0.95 (0.83-1.09) | 0.48 | 0.96 | 0.87 | 1516 | 489 |  |
| BHMT ---- tag | rs1291041 | 1 | 0.95 (0.83-1.09) | 0.48 | 0.96 | 0.87 | 1516 | 489 |  |
| BHMT ---- tag | rs16876500 | 1 | 1.01 (0.82-1.24) | 0.94 | 0.99 | 0.94 | 1516 | 489 | low_count |
| BHMT ---- tag | rs492842 | 1 | 1.03 (0.90-1.18) | 0.64 | 0.96 | 0.87 | 1516 | 489 |  |
| BHMT ---- tag | rs558133 | 1 | 0.94 (0.82-1.08) | 0.39 | 0.96 | 0.87 | 1516 | 489 |  |
| BHMT ---- tag | rs9637824 | 1 | 1.03 (0.90-1.18) | 0.62 | 0.96 | 0.87 | 1516 | 489 |  |
| BHMT2 ---- tag | rs16876512 | 1 | 1.03 (0.84-1.26) | 0.80 | 0.98 | 0.80 | 1516 | 489 | low_count |
| BHMT2 ---- tag | rs2461248 | 1 | 0.98 (0.86-1.12) | 0.78 | 0.97 | 0.80 | 1516 | 489 |  |
| BHMT2 ---- tag | rs2909856 | 1 | 1.02 (0.89-1.17) | 0.78 | 0.97 | 0.80 | 1516 | 489 |  |
| BHMT2 ---- tag | rs476620 | 1 | 1.03 (0.90-1.18) | 0.62 | 0.96 | 0.80 | 1516 | 489 |  |
| BHMT2 ---- candidate literature | rs626105 | 1 | 0.96 (0.81-1.13) | 0.59 | 0.96 | 0.80 | 1516 | 489 |  |
| BHMT2 ---- tag | rs631305 | 1 | 0.96 (0.80-1.14) | 0.61 | 0.96 | 0.80 | 1516 | 489 |  |
| CBS ---- tag | rs11701048 | 1 | 0.95 (0.74-1.23) | 0.72 | 0.97 | 0.78 | 1516 | 489 | low_count |
| CBS ---- tag | rs234706 | 1 | 1.02 (0.89-1.16) | 0.78 | 0.97 | 0.78 | 1516 | 489 |  |
| CBS ---- tag | rs234711 | 1 | 1.09 (0.94-1.27) | 0.27 | 0.95 | 0.69 | 1516 | 489 |  |
| CBS ---- candidate literature | rs234713 | 1 | 1.04 (0.91-1.20) | 0.56 | 0.96 | 0.78 | 1516 | 489 |  |
| CBS ---- tag | rs2839623 | 1 | 1.14 (0.92-1.41) | 0.24 | 0.95 | 0.69 | 1516 | 489 | low_count |
| CBS ---- tag | rs2839626 | 1 | 0.98 (0.86-1.12) | 0.77 | 0.97 | 0.78 | 1516 | 489 |  |
| CBS ---- tag | rs422791 | 1 | 1.12 (0.98-1.28) | 0.11 | 0.80 | 0.56 | 1516 | 489 |  |
| CBS ---- tag | rs706209 | 1 | 0.96 (0.84-1.09) | 0.54 | 0.96 | 0.78 | 1516 | 489 |  |
| CBS ---- tag | rs719037 | 1 | 1.15 (1.01-1.31) | 0.03 | 0.73 | 0.30 | 1516 | 489 |  |
| CBS ---- tag | rs719038 | 1 | 0.96 (0.85-1.09) | 0.56 | 0.96 | 0.78 | 1516 | 489 |  |
| DHFR ---- tag | rs10474632 | 1 | 0.77 (0.60-0.98) | 0.03 | 0.73 | 0.34 | 1516 | 489 | low_count |
| DHFR ---- tag | rs11951910 | 1 | 0.98 (0.81-1.19) | 0.86 | 0.98 | 0.88 | 1516 | 489 | low_count |
| DHFR ---- tag | rs1643665 | 1 | 0.98 (0.85-1.13) | 0.81 | 0.98 | 0.88 | 1516 | 489 |  |
| DHFR ---- tag | rs1650717 | 1 | 1.05 (0.91-1.20) | 0.52 | 0.96 | 0.88 | 1516 | 489 |  |
| DHFR ---- tag | rs1805355 | 1 | 0.98 (0.75-1.27) | 0.88 | 0.98 | 0.88 | 1516 | 489 | low_count |
| DHFR ---- tag | rs6151617 | 1 | 0.91 (0.80-1.03) | 0.15 | 0.90 | 0.74 | 1516 | 489 |  |
| DHFR ---- tag | rs6864493 | 1 | 0.98 (0.85-1.14) | 0.83 | 0.98 | 0.88 | 1516 | 489 |  |
| DHFR ---- tag | rs836788 | 1 | 0.98 (0.86-1.12) | 0.76 | 0.97 | 0.88 | 1516 | 489 |  |
| DHFR ---- tag | rs836790 | 1 | 1.11 (0.94-1.32) | 0.22 | 0.92 | 0.74 | 1516 | 489 |  |
| DHFR ---- tag | rs836817 | 1 | 1.05 (0.92-1.20) | 0.45 | 0.96 | 0.88 | 1516 | 489 |  |
| DNMT1 ---- candidate | rs2228612 | 1 | 1.10 (0.86-1.41) | 0.46 | 0.96 | 0.46 | 1516 | 489 | low_count |
| DNMT3A ---- tag | rs10460566 | 1 | 0.95 (0.82-1.11) | 0.55 | 0.96 | 0.72 | 1516 | 489 |  |
| DNMT3A ---- candidate literature | rs11695471 | 1 | 1.04 (0.91-1.18) | 0.59 | 0.96 | 0.72 | 1516 | 489 |  |
| DNMT3A ---- tag | rs11887120 | 1 | 0.93 (0.82-1.06) | 0.29 | 0.95 | 0.72 | 1516 | 489 |  |
| DNMT3A ---- tag | rs12991495 | 1 | 1.09 (0.95-1.25) | 0.22 | 0.92 | 0.72 | 1516 | 489 |  |
| DNMT3A ---- tag | rs13401241 | 1 | 1.01 (0.89-1.16) | 0.84 | 0.98 | 0.84 | 1516 | 489 |  |
| DNMT3A ---- candidate literature | rs13420827 | 1 | 0.93 (0.80-1.10) | 0.41 | 0.96 | 0.72 | 1516 | 489 |  |
| DNMT3A ---- tag | rs13428812 | 1 | 1.04 (0.90-1.19) | 0.63 | 0.96 | 0.72 | 1516 | 489 |  |
| DNMT3A ---- tag | rs4665287 | 1 | 0.93 (0.79-1.09) | 0.38 | 0.96 | 0.72 | 1516 | 489 |  |
| DNMT3B ---- tag | rs13045669 | 1 | 0.89 (0.60-1.30) | 0.55 | 0.96 | 0.78 | 1516 | 489 | low_count |
| DNMT3B ---- tag | rs17123673 | 1 | 0.85 (0.63-1.14) | 0.28 | 0.95 | 0.74 | 1516 | 489 | low_count |
| DNMT3B ---- tag | rs183603 | 1 | 0.89 (0.77-1.04) | 0.14 | 0.88 | 0.74 | 1516 | 489 |  |
| DNMT3B ---- tag | rs2235760 | 1 | 0.97 (0.82-1.15) | 0.74 | 0.97 | 0.82 | 1516 | 489 |  |
| DNMT3B ---- tag | rs2424908 | 1 | 0.94 (0.79-1.11) | 0.45 | 0.96 | 0.74 | 1516 | 489 |  |
| DNMT3B ---- candidate literature | rs2424909 | 1 | 0.94 (0.83-1.07) | 0.36 | 0.96 | 0.74 | 1516 | 489 |  |
| DNMT3B ---- tag | rs4911108 | 1 | 0.94 (0.82-1.07) | 0.32 | 0.96 | 0.74 | 1516 | 489 |  |
| DNMT3B ---- tag | rs6058896 | 1 | 1.06 (0.81-1.39) | 0.65 | 0.96 | 0.81 | 1516 | 489 | low_count |
| DNMT3B ---- tag | rs6119954 | 1 | 0.94 (0.79-1.11) | 0.44 | 0.96 | 0.74 | 1516 | 489 |  |
| DNMT3B ---- tag | rs6579038 | 1 | 1.03 (0.79-1.35) | 0.82 | 0.98 | 0.82 | 1516 | 489 | low_count |
| DPYD ---- tag | rs1034215 | 1 | 0.91 (0.77-1.06) | 0.22 | 0.92 | 0.59 | 1516 | 489 |  |
| DPYD ---- tag | rs10783058 | 1 | 1.17 (1.03-1.33) | 0.02 | 0.73 | 0.49 | 1516 | 489 |  |
| DPYD ---- tag | rs10783070 | 1 | 1.12 (0.94-1.33) | 0.20 | 0.92 | 0.59 | 1516 | 489 |  |
| DPYD ---- tag | rs10875048 | 1 | 0.94 (0.80-1.11) | 0.49 | 0.96 | 0.75 | 1516 | 489 |  |
| DPYD ---- tag | rs10875055 | 1 | 1.14 (1.00-1.30) | 0.04 | 0.77 | 0.49 | 1516 | 489 |  |
| DPYD ---- tag | rs10875079 | 1 | 0.94 (0.83-1.06) | 0.30 | 0.95 | 0.67 | 1516 | 489 |  |
| DPYD ---- tag | rs10875085 | 1 | 0.95 (0.80-1.12) | 0.54 | 0.96 | 0.79 | 1516 | 489 |  |
| DPYD ---- tag | rs10875097 | 1 | 1.15 (0.98-1.35) | 0.09 | 0.79 | 0.49 | 1516 | 489 |  |
| DPYD ---- tag | rs11165781 | 1 | 0.97 (0.81-1.15) | 0.69 | 0.97 | 0.83 | 1516 | 489 |  |
| DPYD ---- tag | rs11165783 | 1 | 1.14 (0.99-1.31) | 0.07 | 0.79 | 0.49 | 1516 | 489 |  |
| DPYD ---- tag | rs11165873 | 1 | 0.92 (0.81-1.05) | 0.22 | 0.92 | 0.59 | 1516 | 489 |  |
| DPYD ---- tag | rs11165875 | 1 | 1.09 (0.96-1.23) | 0.20 | 0.92 | 0.59 | 1516 | 489 |  |
| DPYD ---- tag | rs11165881 | 1 | 1.02 (0.90-1.15) | 0.79 | 0.97 | 0.86 | 1516 | 489 |  |
| DPYD ---- tag | rs11587873 | 1 | 0.85 (0.73-1.00) | 0.05 | 0.77 | 0.49 | 1516 | 489 |  |
| DPYD ---- tag | rs12030174 | 1 | 0.93 (0.77-1.11) | 0.41 | 0.96 | 0.72 | 1516 | 489 |  |
| DPYD ---- tag | rs12046744 | 1 | 0.89 (0.77-1.03) | 0.11 | 0.80 | 0.52 | 1516 | 489 |  |
| DPYD ---- tag | rs12047910 | 1 | 0.92 (0.77-1.10) | 0.37 | 0.96 | 0.70 | 1516 | 489 |  |
| DPYD ---- tag | rs12073044 | 1 | 0.86 (0.68-1.07) | 0.17 | 0.92 | 0.59 | 1516 | 489 | low_count |
| DPYD ---- tag | rs12126093 | 1 | 0.95 (0.82-1.09) | 0.47 | 0.96 | 0.75 | 1516 | 489 |  |
| DPYD ---- tag | rs12134028 | 1 | 0.81 (0.60-1.09) | 0.16 | 0.91 | 0.59 | 1516 | 489 | low_count |
| DPYD ---- tag | rs12740796 | 1 | 1.05 (0.87-1.26) | 0.62 | 0.96 | 0.83 | 1516 | 489 |  |
| DPYD ---- tag | rs1333717 | 1 | 0.91 (0.78-1.06) | 0.25 | 0.95 | 0.60 | 1516 | 489 |  |
| DPYD ---- tag | rs1413228 | 1 | 1.01 (0.82-1.24) | 0.91 | 0.98 | 0.91 | 1516 | 489 | low_count |
| DPYD ---- tag | rs1415681 | 1 | 1.02 (0.85-1.21) | 0.87 | 0.98 | 0.90 | 1516 | 489 |  |
| DPYD ---- tag | rs1514495 | 1 | 0.97 (0.84-1.13) | 0.72 | 0.97 | 0.83 | 1516 | 489 |  |
| DPYD ---- tag | rs1520658 | 1 | 1.09 (0.89-1.33) | 0.39 | 0.96 | 0.71 | 1516 | 489 | low_count |
| DPYD ---- NA | rs17116806 | 1 | 1.10 (0.94-1.29) | 0.23 | 0.93 | 0.59 | 1516 | 489 |  |
| DPYD ---- tag | rs17431828 | 1 | 0.97 (0.85-1.10) | 0.61 | 0.96 | 0.83 | 1516 | 489 |  |
| DPYD ---- tag | rs17471640 | 1 | 0.92 (0.80-1.06) | 0.25 | 0.95 | 0.60 | 1516 | 489 |  |
| DPYD ---- tag | rs17702702 | 1 | 0.94 (0.80-1.11) | 0.49 | 0.96 | 0.75 | 1516 | 489 |  |
| DPYD ---- NA | rs1801265 | 1 | 0.98 (0.84-1.14) | 0.75 | 0.97 | 0.85 | 1516 | 489 |  |
| DPYD ---- tag | rs2039447 | 1 | 1.02 (0.89-1.17) | 0.76 | 0.97 | 0.85 | 1516 | 489 |  |
| DPYD ---- tag | rs2151567 | 1 | 1.22 (0.92-1.63) | 0.18 | 0.92 | 0.59 | 1516 | 489 | low_count |
| DPYD ---- tag | rs2152878 | 1 | 1.03 (0.89-1.19) | 0.71 | 0.97 | 0.83 | 1516 | 489 |  |
| DPYD ---- tag | rs2786505 | 1 | 1.20 (0.99-1.45) | 0.06 | 0.77 | 0.49 | 1516 | 489 | low_count |
| DPYD ---- tag | rs2786512 | 1 | 0.96 (0.85-1.09) | 0.50 | 0.96 | 0.75 | 1516 | 489 |  |
| DPYD ---- tag | rs2786519 | 1 | 1.08 (0.94-1.25) | 0.28 | 0.95 | 0.65 | 1516 | 489 |  |
| DPYD ---- tag | rs2811170 | 1 | 0.81 (0.66-1.00) | 0.05 | 0.77 | 0.49 | 1516 | 489 | low_count |
| DPYD ---- tag | rs2811199 | 1 | 1.14 (0.96-1.36) | 0.14 | 0.89 | 0.59 | 1516 | 489 |  |
| DPYD ---- tag | rs2811219 | 1 | 0.96 (0.83-1.12) | 0.62 | 0.96 | 0.83 | 1516 | 489 |  |
| DPYD ---- tag | rs4300257 | 1 | 1.01 (0.86-1.19) | 0.88 | 0.98 | 0.90 | 1516 | 489 |  |
| DPYD ---- tag | rs4379706 | 1 | 0.98 (0.84-1.14) | 0.81 | 0.98 | 0.87 | 1516 | 489 |  |
| DPYD ---- tag | rs4950021 | 1 | 1.06 (0.93-1.20) | 0.37 | 0.96 | 0.70 | 1516 | 489 |  |
| DPYD ---- tag | rs4950033 | 1 | 1.05 (0.92-1.19) | 0.46 | 0.96 | 0.75 | 1516 | 489 |  |
| DPYD ---- tag | rs495257 | 1 | 1.03 (0.91-1.18) | 0.63 | 0.96 | 0.83 | 1516 | 489 |  |
| DPYD ---- tag | rs552926 | 1 | 1.15 (1.01-1.30) | 0.03 | 0.73 | 0.49 | 1516 | 489 |  |
| DPYD ---- tag | rs628959 | 1 | 0.95 (0.82-1.09) | 0.47 | 0.96 | 0.75 | 1516 | 489 |  |
| DPYD ---- tag | rs6656660 | 1 | 1.04 (0.87-1.25) | 0.66 | 0.96 | 0.83 | 1516 | 489 |  |
| DPYD ---- tag | rs6663670 | 1 | 1.08 (0.91-1.29) | 0.38 | 0.96 | 0.70 | 1516 | 489 | low_count |
| DPYD ---- tag | rs6683883 | 1 | 1.12 (0.98-1.27) | 0.09 | 0.79 | 0.49 | 1516 | 489 |  |
| DPYD ---- tag | rs6686861 | 1 | 1.12 (0.89-1.42) | 0.32 | 0.96 | 0.69 | 1516 | 489 | low_count |
| DPYD ---- tag | rs7414210 | 1 | 0.97 (0.80-1.16) | 0.72 | 0.97 | 0.83 | 1516 | 489 | low_count |
| DPYD ---- tag | rs7530858 | 1 | 1.14 (0.93-1.39) | 0.20 | 0.92 | 0.59 | 1516 | 489 | low_count |
| DPYD ---- tag | rs7544128 | 1 | 1.03 (0.90-1.19) | 0.65 | 0.96 | 0.83 | 1516 | 489 |  |
| DPYD ---- tag | rs7545340 | 1 | 1.07 (0.93-1.23) | 0.34 | 0.96 | 0.69 | 1516 | 489 |  |
| DPYD ---- tag | rs828054 | 1 | 0.89 (0.79-1.02) | 0.09 | 0.79 | 0.49 | 1516 | 489 |  |
| DPYD ---- tag | rs885622 | 1 | 1.12 (0.99-1.28) | 0.08 | 0.79 | 0.49 | 1516 | 489 |  |
| DPYD ---- tag | rs9437663 | 1 | 0.99 (0.84-1.16) | 0.87 | 0.98 | 0.90 | 1516 | 489 |  |
| DPYS ---- tag | rs13249169 | 1 | 1.07 (0.87-1.30) | 0.53 | 0.96 | 0.65 | 1516 | 489 | low_count |
| DPYS ---- NA | rs13263121 | 1 | 0.88 (0.77-1.01) | 0.08 | 0.79 | 0.55 | 1516 | 489 |  |
| DPYS ---- tag | rs16871361 | 1 | 1.02 (0.77-1.36) | 0.88 | 0.98 | 0.88 | 1516 | 489 | low_count |
| DPYS ---- NA | rs17245950 | 1 | 0.92 (0.75-1.14) | 0.46 | 0.96 | 0.62 | 1516 | 489 | low_count |
| DPYS ---- NA | rs2253336 | 1 | 0.93 (0.75-1.15) | 0.48 | 0.96 | 0.62 | 1516 | 489 | low_count |
| DPYS ---- tag | rs2280010 | 1 | 1.18 (1.03-1.37) | 0.02 | 0.73 | 0.48 | 1516 | 489 |  |
| DPYS ---- tag | rs2333874 | 1 | 1.06 (0.92-1.21) | 0.44 | 0.96 | 0.62 | 1516 | 489 |  |
| DPYS ---- NA | rs2669429 | 1 | 1.06 (0.94-1.21) | 0.35 | 0.96 | 0.59 | 1516 | 489 |  |
| DPYS ---- tag | rs2669434 | 1 | 0.89 (0.77-1.03) | 0.13 | 0.85 | 0.55 | 1516 | 489 |  |
| DPYS ---- tag | rs2853142 | 1 | 0.91 (0.79-1.05) | 0.20 | 0.92 | 0.55 | 1516 | 489 |  |
| DPYS ---- NA | rs2853145 | 1 | 0.92 (0.78-1.09) | 0.34 | 0.96 | 0.59 | 1516 | 489 |  |
| DPYS ---- tag | rs2853149 | 1 | 1.11 (0.97-1.27) | 0.12 | 0.80 | 0.55 | 1516 | 489 |  |
| DPYS ---- tag | rs2853154 | 1 | 0.88 (0.75-1.03) | 0.11 | 0.80 | 0.55 | 1516 | 489 |  |
| DPYS ---- tag | rs2853161 | 1 | 0.94 (0.83-1.08) | 0.38 | 0.96 | 0.60 | 1516 | 489 |  |
| DPYS ---- NA | rs2959024 | 1 | 1.03 (0.89-1.18) | 0.71 | 0.97 | 0.74 | 1516 | 489 |  |
| DPYS ---- NA | rs2959025 | 1 | 1.03 (0.90-1.17) | 0.70 | 0.97 | 0.74 | 1516 | 489 |  |
| DPYS ---- tag | rs2959026 | 1 | 1.08 (0.95-1.23) | 0.26 | 0.95 | 0.57 | 1516 | 489 |  |
| DPYS ---- NA | rs3133278 | 1 | 0.92 (0.79-1.06) | 0.26 | 0.95 | 0.57 | 1516 | 489 |  |
| DPYS ---- tag | rs3750187 | 1 | 0.89 (0.75-1.05) | 0.15 | 0.91 | 0.55 | 1516 | 489 |  |
| DPYS ---- tag | rs3793357 | 1 | 1.06 (0.80-1.40) | 0.68 | 0.97 | 0.74 | 1516 | 489 | low_count |
| DPYS ---- tag | rs3793358 | 1 | 0.88 (0.72-1.07) | 0.19 | 0.92 | 0.55 | 1516 | 489 |  |
| DPYS ---- tag | rs6468924 | 1 | 0.92 (0.78-1.09) | 0.34 | 0.96 | 0.59 | 1516 | 489 |  |
| DUT ---- tag | rs8025164 | 1 | 1.07 (0.90-1.28) | 0.43 | 0.96 | 0.43 | 1516 | 489 |  |
| EHMT1 ---- tag | rs10780190 | 1 | 1.11 (0.83-1.48) | 0.48 | 0.96 | 0.92 | 1516 | 489 | low_count |
| EHMT1 ---- tag | rs10867083 | 1 | 0.97 (0.84-1.11) | 0.63 | 0.96 | 0.92 | 1516 | 489 |  |
| EHMT1 ---- tag | rs11137190 | 1 | 1.05 (0.91-1.21) | 0.52 | 0.96 | 0.92 | 1516 | 489 |  |
| EHMT1 ---- tag | rs3123510 | 1 | 1.03 (0.90-1.17) | 0.67 | 0.96 | 0.92 | 1516 | 489 |  |
| EHMT1 ---- candidate literature | rs3125795 | 1 | 1.12 (0.84-1.49) | 0.43 | 0.96 | 0.92 | 1516 | 489 | low_count |
| EHMT1 ---- tag | rs4573359 | 1 | 0.99 (0.79-1.24) | 0.93 | 0.98 | 0.93 | 1516 | 489 | low_count |
| EHMT1 ---- candidate literature | rs4634736 | 1 | 0.98 (0.78-1.23) | 0.84 | 0.98 | 0.93 | 1516 | 489 | low_count |
| EHMT1 ---- tag | rs4876902 | 1 | 1.14 (0.98-1.33) | 0.09 | 0.79 | 0.92 | 1516 | 489 |  |
| EHMT1 ---- tag | rs4876904 | 1 | 0.95 (0.84-1.08) | 0.44 | 0.96 | 0.92 | 1516 | 489 |  |
| EHMT1 ---- tag | rs7390244 | 1 | 0.99 (0.88-1.12) | 0.90 | 0.98 | 0.93 | 1516 | 489 |  |
| EHMT1 ---- tag | rs9314635 | 1 | 1.07 (0.94-1.23) | 0.29 | 0.95 | 0.92 | 1516 | 489 |  |
| EHMT2 ---- candidate/tag | rs2736428 | 1 | 0.97 (0.85-1.10) | 0.61 | 0.96 | 0.61 | 1516 | 489 |  |
| EHMT2 ---- tag | rs9267649 | 1 | 0.89 (0.75-1.06) | 0.21 | 0.92 | 0.41 | 1516 | 489 |  |
| FDXR ---- NA | rs2070918 | 1 | 1.10 (0.97-1.26) | 0.13 | 0.86 | 0.53 | 1516 | 489 |  |
| FDXR ---- tag | rs509911 | 1 | 1.05 (0.90-1.22) | 0.57 | 0.96 | 0.70 | 1516 | 489 |  |
| FDXR ---- NA | rs689882 | 1 | 0.97 (0.84-1.11) | 0.63 | 0.96 | 0.70 | 1516 | 489 |  |
| FDXR ---- NA | rs689895 | 1 | 0.97 (0.85-1.12) | 0.70 | 0.97 | 0.70 | 1516 | 489 |  |
| FOLH1 ---- candidate literature | rs10839236 | 1 | 1.00 (0.88-1.14) | 0.98 | 0.99 | 0.98 | 1516 | 489 |  |
| FOLH1 ---- tag | rs16906190 | 1 | 1.01 (0.81-1.27) | 0.92 | 0.98 | 0.98 | 1516 | 489 | low_count |
| FOLH1 ---- candidate | rs202676 | 1 | 0.98 (0.84-1.14) | 0.77 | 0.97 | 0.98 | 1516 | 489 |  |
| FOLH1 ---- tag | rs202680 | 1 | 0.98 (0.84-1.13) | 0.75 | 0.97 | 0.98 | 1516 | 489 |  |
| FOLH1 ---- candidate literature | rs202720 | 1 | 0.97 (0.83-1.14) | 0.72 | 0.97 | 0.98 | 1516 | 489 |  |
| FOLH1 ---- tag | rs2299650 | 1 | 0.99 (0.87-1.13) | 0.91 | 0.98 | 0.98 | 1516 | 489 |  |
| FOLH1 ---- tag | rs617528 | 1 | 1.04 (0.87-1.25) | 0.65 | 0.96 | 0.98 | 1516 | 489 | low_count |
| FOLH1 ---- tag | rs663877 | 1 | 0.96 (0.79-1.16) | 0.66 | 0.96 | 0.98 | 1516 | 489 |  |
| FOLH1 ---- tag | rs670776 | 1 | 0.98 (0.84-1.14) | 0.77 | 0.97 | 0.98 | 1516 | 489 |  |
| FOLH1 ---- tag | rs7124497 | 1 | 0.98 (0.70-1.36) | 0.90 | 0.98 | 0.98 | 1516 | 489 | low_count |
| FOLR1 ---- tag | rs651646 | 1 | 0.96 (0.85-1.09) | 0.54 | 0.96 | 0.54 | 1516 | 489 |  |
| FPGS ---- tag | rs10987746 | 1 | 0.99 (0.87-1.13) | 0.88 | 0.98 | 0.88 | 1516 | 489 |  |
| FPGS ---- tag | rs7033913 | 1 | 0.96 (0.84-1.10) | 0.56 | 0.96 | 0.88 | 1516 | 489 |  |
| FPGS ---- tag | rs7039798 | 1 | 0.98 (0.86-1.12) | 0.76 | 0.97 | 0.88 | 1516 | 489 |  |
| GGH ---- tag | rs10957264 | 1 | 1.03 (0.87-1.22) | 0.71 | 0.97 | 0.77 | 1516 | 489 |  |
| GGH ---- candidate literature | rs11545076 | 1 | 0.89 (0.78-1.03) | 0.12 | 0.80 | 0.26 | 1516 | 489 |  |
| GGH ---- candidate | rs11545077 | 1 | 0.94 (0.81-1.08) | 0.39 | 0.96 | 0.63 | 1516 | 489 |  |
| GGH ---- candidate | rs11545078 | 1 | 1.05 (0.86-1.28) | 0.63 | 0.96 | 0.75 | 1516 | 489 | low_count |
| GGH ---- tag | rs11995525 | 1 | 1.17 (1.02-1.35) | 0.03 | 0.73 | 0.14 | 1516 | 489 |  |
| GGH ---- tag | rs16930073 | 1 | 0.80 (0.65-0.98) | 0.03 | 0.73 | 0.14 | 1516 | 489 | low_count |
| GGH ---- tag | rs17194931 | 1 | 1.05 (0.86-1.28) | 0.63 | 0.96 | 0.75 | 1516 | 489 | low_count |
| GGH ---- candidate literature | rs1800909 | 1 | 0.88 (0.77-1.02) | 0.09 | 0.79 | 0.26 | 1516 | 489 |  |
| GGH ---- candidate literature | rs3758149 | 1 | 0.89 (0.78-1.03) | 0.12 | 0.80 | 0.26 | 1516 | 489 |  |
| GGH ---- tag | rs3780130 | 1 | 0.79 (0.67-0.93) | 0.01 | 0.73 | 0.07 | 1516 | 489 |  |
| GGH ---- tag | rs4446729 | 1 | 1.01 (0.87-1.16) | 0.91 | 0.98 | 0.91 | 1516 | 489 |  |
| GGH ---- tag | rs6472067 | 1 | 1.10 (0.97-1.25) | 0.15 | 0.90 | 0.27 | 1516 | 489 |  |
| GGH ---- tag | rs7010484 | 1 | 1.05 (0.92-1.20) | 0.47 | 0.96 | 0.68 | 1516 | 489 |  |
| GNMT ---- tag | rs1053538 | 1 | 1.11 (0.98-1.26) | 0.12 | 0.80 | 0.41 | 1516 | 489 |  |
| GNMT ---- tag | rs2296805 | 1 | 0.93 (0.82-1.06) | 0.30 | 0.95 | 0.41 | 1516 | 489 |  |
| GNMT ---- tag | rs6901782 | 1 | 1.07 (0.89-1.30) | 0.45 | 0.96 | 0.45 | 1516 | 489 |  |
| GNMT ---- tag | rs6927188 | 1 | 1.09 (0.94-1.26) | 0.27 | 0.95 | 0.41 | 1516 | 489 |  |
| MAT1A ---- tag | rs10887708 | 1 | 0.91 (0.79-1.04) | 0.17 | 0.91 | 0.36 | 1516 | 489 |  |
| MAT1A ---- tag | rs10887718 | 1 | 1.11 (0.98-1.25) | 0.10 | 0.80 | 0.29 | 1516 | 489 |  |
| MAT1A ---- tag | rs11202403 | 1 | 0.98 (0.84-1.15) | 0.83 | 0.98 | 0.83 | 1516 | 489 |  |
| MAT1A ---- tag | rs1832683 | 1 | 0.94 (0.80-1.11) | 0.48 | 0.96 | 0.62 | 1516 | 489 |  |
| MAT1A ---- tag | rs2236568 | 1 | 0.86 (0.76-0.98) | 0.02 | 0.73 | 0.12 | 1516 | 489 |  |
| MAT1A ---- tag | rs2236569 | 1 | 1.16 (1.02-1.32) | 0.03 | 0.73 | 0.12 | 1516 | 489 |  |
| MAT1A ---- tag | rs9421467 | 1 | 0.93 (0.70-1.23) | 0.60 | 0.96 | 0.68 | 1516 | 489 | low_count |
| MAT1A ---- tag | rs998765 | 1 | 0.92 (0.81-1.04) | 0.20 | 0.92 | 0.36 | 1516 | 489 |  |
| MAT1A ---- tag | rs998766 | 1 | 0.93 (0.82-1.06) | 0.28 | 0.95 | 0.43 | 1516 | 489 |  |
| MAT2B ---- tag | rs12655857 | 1 | 1.05 (0.91-1.21) | 0.53 | 0.96 | 0.96 | 1516 | 489 |  |
| MAT2B ---- tag | rs6869277 | 1 | 0.99 (0.81-1.22) | 0.95 | 0.99 | 0.96 | 1516 | 489 | low_count |
| MAT2B ---- tag | rs6874065 | 1 | 0.97 (0.85-1.10) | 0.63 | 0.96 | 0.96 | 1516 | 489 |  |
| MAT2B ---- tag | rs6882306 | 1 | 1.18 (0.99-1.39) | 0.06 | 0.77 | 0.29 | 1516 | 489 |  |
| MAT2B ---- tag | rs7721639 | 1 | 1.01 (0.84-1.20) | 0.96 | 0.99 | 0.96 | 1516 | 489 |  |
| MTHFD1 ---- tag | rs1256148 | 1 | 1.08 (0.93-1.25) | 0.33 | 0.96 | 0.65 | 1516 | 489 |  |
| MTHFD1 ---- tag | rs13329053 | 1 | 1.04 (0.91-1.18) | 0.58 | 0.96 | 0.75 | 1516 | 489 |  |
| MTHFD1 ---- candidate literature | rs2236224 | 1 | 1.09 (0.96-1.24) | 0.20 | 0.92 | 0.61 | 1516 | 489 |  |
| MTHFD1 ---- candidate | rs2236225 | 1 | 1.02 (0.90-1.16) | 0.72 | 0.97 | 0.75 | 1516 | 489 |  |
| MTHFD1 ---- tag | rs2281603 | 1 | 0.98 (0.84-1.13) | 0.75 | 0.97 | 0.75 | 1516 | 489 |  |
| MTHFD1 ---- candidate literature | rs8003379 | 1 | 1.14 (0.98-1.31) | 0.08 | 0.79 | 0.50 | 1516 | 489 |  |
| MTHFD2 ---- tag | rs10177833 | 1 | 1.09 (0.96-1.25) | 0.19 | 0.92 | 0.51 | 1516 | 489 |  |
| MTHFD2 ---- tag | rs702462 | 1 | 0.98 (0.86-1.12) | 0.74 | 0.97 | 0.86 | 1516 | 489 |  |
| MTHFD2 ---- candidate literature | rs702465 | 1 | 1.03 (0.91-1.17) | 0.66 | 0.96 | 0.86 | 1516 | 489 |  |
| MTHFD2 ---- candidate literature | rs7571842 | 1 | 1.07 (0.94-1.22) | 0.30 | 0.95 | 0.52 | 1516 | 489 |  |
| MTHFD2 ---- tag | rs7587117 | 1 | 1.09 (0.95-1.25) | 0.22 | 0.92 | 0.51 | 1516 | 489 |  |
| MTHFD2 ---- tag | rs828861 | 1 | 1.00 (0.88-1.14) | 0.98 | 0.99 | 0.98 | 1516 | 489 |  |
| MTHFD2 ---- tag | rs828863 | 1 | 0.76 (0.60-0.97) | 0.03 | 0.73 | 0.20 | 1516 | 489 | low_count |
| MTHFR ---- tag | rs1476413 | 1 | 0.99 (0.85-1.14) | 0.84 | 0.98 | 0.96 | 1516 | 489 |  |
| MTHFR ---- tag | rs17376328 | 1 | 1.17 (0.93-1.49) | 0.18 | 0.92 | 0.83 | 1516 | 489 | low_count |
| MTHFR ---- tag | rs17421462 | 1 | 0.98 (0.76-1.25) | 0.85 | 0.98 | 0.96 | 1516 | 489 | low_count |
| MTHFR ---- candidate | rs1801131 | 1 | 0.99 (0.86-1.13) | 0.85 | 0.98 | 0.96 | 1516 | 489 |  |
| MTHFR ---- candidate | rs1801133 | 1 | 0.87 (0.75-1.01) | 0.06 | 0.77 | 0.54 | 1516 | 489 |  |
| MTHFR ---- tag | rs2066471 | 1 | 0.95 (0.79-1.13) | 0.54 | 0.96 | 0.96 | 1516 | 489 |  |
| MTHFR ---- tag | rs4846047 | 1 | 0.96 (0.83-1.11) | 0.58 | 0.96 | 0.96 | 1516 | 489 |  |
| MTHFR ---- tag | rs4846049 | 1 | 0.98 (0.86-1.13) | 0.82 | 0.98 | 0.96 | 1516 | 489 |  |
| MTHFR ---- tag | rs7538516 | 1 | 1.00 (0.87-1.14) | 0.97 | 0.99 | 0.97 | 1516 | 489 |  |
| MTR ---- tag | rs10733117 | 1 | 0.97 (0.85-1.11) | 0.66 | 0.96 | 0.66 | 1516 | 489 |  |
| MTR ---- tag | rs12129440 | 1 | 0.95 (0.81-1.11) | 0.50 | 0.96 | 0.66 | 1516 | 489 |  |
| MTR ---- candidate | rs1805087 | 1 | 0.95 (0.81-1.12) | 0.56 | 0.96 | 0.66 | 1516 | 489 |  |
| MTR ---- tag | rs3890786 | 1 | 1.06 (0.93-1.21) | 0.37 | 0.96 | 0.66 | 1516 | 489 |  |
| MTR ---- tag | rs4659727 | 1 | 0.93 (0.79-1.09) | 0.35 | 0.96 | 0.66 | 1516 | 489 |  |
| MTRR ---- candidate literature/tag | rs10380 | 1 | 1.04 (0.82-1.31) | 0.75 | 0.97 | 0.99 | 1516 | 489 | low_count |
| MTRR ---- tag | rs10475399 | 1 | 1.00 (0.88-1.15) | 0.96 | 0.99 | 0.99 | 1516 | 489 |  |
| MTRR ---- tag | rs11134265 | 1 | 1.03 (0.90-1.18) | 0.66 | 0.96 | 0.99 | 1516 | 489 |  |
| MTRR ---- tag | rs13181011 | 1 | 1.08 (0.92-1.26) | 0.36 | 0.96 | 0.99 | 1516 | 489 |  |
| MTRR ---- tag | rs161869 | 1 | 1.04 (0.91-1.18) | 0.58 | 0.96 | 0.99 | 1516 | 489 |  |
| MTRR ---- tagged by rs162039 | rs162036 | 1 | 1.05 (0.85-1.29) | 0.64 | 0.96 | 0.99 | 1516 | 489 | low_count |
| MTRR ---- tag | rs162039 | 1 | 1.05 (0.85-1.29) | 0.64 | 0.96 | 0.99 | 1516 | 489 | low_count |
| MTRR ---- tag | rs162270 | 1 | 1.00 (0.85-1.18) | 0.99 | 0.99 | 0.99 | 1516 | 489 |  |
| MTRR ---- candidate | rs16879334 | 1 | 0.88 (0.58-1.34) | 0.54 | 0.96 | 0.99 | 1516 | 489 |  |
| MTRR ---- candidate | rs1801394 | 1 | 0.95 (0.84-1.09) | 0.48 | 0.96 | 0.99 | 1516 | 489 |  |
| MTRR ---- tag | rs1802059 | 1 | 0.99 (0.87-1.13) | 0.93 | 0.98 | 0.99 | 1516 | 489 |  |
| MTRR ---- tag | rs2077744 | 1 | 1.00 (0.84-1.19) | 0.97 | 0.99 | 0.99 | 1516 | 489 | low_count |
| MTRR ---- candidate | rs2287780 | 1 | 0.88 (0.58-1.34) | 0.54 | 0.96 | 0.99 | 1516 | 489 |  |
| MTRR ---- candidate | rs2303080 | 1 | 0.87 (0.56-1.33) | 0.51 | 0.96 | 0.99 | 1516 | 489 |  |
| MTRR ---- tag | rs7715062 | 1 | 1.02 (0.90-1.16) | 0.78 | 0.97 | 0.99 | 1516 | 489 |  |
| MTRR ---- tag | rs9282787 | 1 | 1.10 (0.94-1.29) | 0.22 | 0.92 | 0.99 | 1516 | 489 |  |
| MTRR ---- candidate literature | rs9332 | 1 | 1.05 (0.85-1.29) | 0.64 | 0.96 | 0.99 | 1516 | 489 | low_count |
| NFKB1 ---- NA | rs1609798 | 1 | 0.95 (0.83-1.10) | 0.51 | 0.96 | 0.83 | 1516 | 489 |  |
| NFKB1 ---- tag | rs230540 | 1 | 0.89 (0.77-1.01) | 0.08 | 0.79 | 0.40 | 1516 | 489 |  |
| NFKB1 ---- tag | rs230541 | 1 | 0.89 (0.78-1.02) | 0.09 | 0.79 | 0.40 | 1516 | 489 |  |
| NFKB1 ---- NA | rs230547 | 1 | 1.03 (0.84-1.27) | 0.75 | 0.97 | 0.87 | 1516 | 489 | low_count |
| NFKB1 ---- tag | rs3774934 | 1 | 1.06 (0.87-1.29) | 0.57 | 0.96 | 0.83 | 1516 | 489 | low_count |
| NFKB1 ---- tag | rs3774968 | 1 | 1.07 (0.94-1.22) | 0.30 | 0.95 | 0.78 | 1516 | 489 |  |
| NFKB1 ---- NA | rs4648022 | 1 | 0.99 (0.78-1.25) | 0.95 | 0.99 | 0.95 | 1516 | 489 | low_count |
| NFKB1 ---- NA | rs4648090 | 1 | 0.92 (0.76-1.11) | 0.39 | 0.96 | 0.78 | 1516 | 489 | low_count |
| NFKB1 ---- tag | rs4648110 | 1 | 1.03 (0.87-1.20) | 0.76 | 0.97 | 0.87 | 1516 | 489 |  |
| NFKB1 ---- tag | rs4648141 | 1 | 1.05 (0.88-1.24) | 0.59 | 0.96 | 0.83 | 1516 | 489 | low_count |
| NFKB1 ---- tag | rs4698863 | 1 | 0.93 (0.81-1.07) | 0.32 | 0.96 | 0.78 | 1516 | 489 |  |
| NFKB1 ---- NA | rs7674640 | 1 | 0.94 (0.83-1.07) | 0.38 | 0.96 | 0.78 | 1516 | 489 |  |
| NFKB1 ---- tag | rs909332 | 1 | 1.04 (0.76-1.43) | 0.81 | 0.98 | 0.87 | 1516 | 489 | low_count |
| NFKB1 ---- tag | rs997476 | 1 | 1.28 (1.00-1.64) | 0.05 | 0.77 | 0.40 | 1516 | 489 | low_count |
| NME1 ---- NA | rs10514981 | 1 | 0.91 (0.77-1.07) | 0.26 | 0.95 | 0.98 | 1516 | 489 |  |
| NME1 ---- NA | rs11651252 | 1 | 0.92 (0.70-1.21) | 0.55 | 0.96 | 0.98 | 1516 | 489 | low_count |
| NME1 ---- tag | rs11652793 | 1 | 0.92 (0.77-1.10) | 0.36 | 0.96 | 0.98 | 1516 | 489 |  |
| NME1 ---- NA | rs11868380 | 1 | 1.00 (0.85-1.17) | 0.96 | 0.99 | 0.99 | 1516 | 489 |  |
| NME1 ---- NA | rs1558252 | 1 | 1.02 (0.89-1.17) | 0.78 | 0.97 | 0.98 | 1516 | 489 |  |
| NME1 ---- NA | rs1558253 | 1 | 1.15 (0.89-1.48) | 0.29 | 0.95 | 0.98 | 1516 | 489 | low_count |
| NME1 ---- tag | rs16949683 | 1 | 1.08 (0.79-1.48) | 0.64 | 0.96 | 0.98 | 1516 | 489 | low_count |
| NME1 ---- tag | rs2318784 | 1 | 1.04 (0.86-1.25) | 0.71 | 0.97 | 0.98 | 1516 | 489 | low_count |
| NME1 ---- NA | rs2318785 | 1 | 0.97 (0.86-1.11) | 0.70 | 0.97 | 0.98 | 1516 | 489 |  |
| NME1 ---- tag | rs3760469 | 1 | 1.04 (0.91-1.19) | 0.55 | 0.96 | 0.98 | 1516 | 489 |  |
| NME1 ---- NA | rs4605213 | 1 | 0.99 (0.86-1.13) | 0.85 | 0.98 | 0.98 | 1516 | 489 |  |
| NME1 ---- NA | rs7207090 | 1 | 1.00 (0.88-1.14) | 0.99 | 0.99 | 0.99 | 1516 | 489 |  |
| NME1 ---- tag | rs7222463 | 1 | 1.02 (0.89-1.16) | 0.82 | 0.98 | 0.98 | 1516 | 489 |  |
| NME1 ---- tag | rs7226059 | 1 | 1.06 (0.93-1.22) | 0.36 | 0.96 | 0.98 | 1516 | 489 |  |
| NME1 ---- NA | rs880178 | 1 | 1.05 (0.92-1.20) | 0.47 | 0.96 | 0.98 | 1516 | 489 |  |
| NME2 ---- tag | rs7220360 | 1 | 1.02 (0.89-1.16) | 0.80 | 0.98 | 0.80 | 1516 | 489 |  |
| PON1 ---- tag | rs2269829 | 1 | 1.16 (1.02-1.33) | 0.03 | 0.73 | 0.07 | 1516 | 489 |  |
| PON1 ---- tag | rs3917527 | 1 | 0.75 (0.55-1.01) | 0.06 | 0.77 | 0.10 | 1516 | 489 | low_count |
| PON1 ---- tag | rs3917538 | 1 | 1.31 (1.14-1.51) | <.001 | <.001 | <.001 | 1516 | 489 |  |
| PON1 ---- tag | rs757158 | 1 | 0.92 (0.80-1.05) | 0.22 | 0.92 | 0.27 | 1516 | 489 |  |
| PON1 ---- candidate | rs854560 | 1 | 1.04 (0.91-1.18) | 0.57 | 0.96 | 0.57 | 1516 | 489 |  |
| PRDM2 ---- tag | rs1015370 | 1 | 0.93 (0.80-1.06) | 0.27 | 0.95 | 0.72 | 1516 | 489 |  |
| PRDM2 ---- tag | rs1203634 | 1 | 1.16 (1.01-1.34) | 0.04 | 0.73 | 0.20 | 1516 | 489 |  |
| PRDM2 ---- tag | rs1203645 | 1 | 1.13 (1.00-1.27) | 0.05 | 0.77 | 0.20 | 1516 | 489 |  |
| PRDM2 ---- tag | rs1406416 | 1 | 0.99 (0.87-1.14) | 0.92 | 0.98 | 0.92 | 1516 | 489 |  |
| PRDM2 ---- candidate | rs17350795 | 1 | 0.93 (0.60-1.43) | 0.73 | 0.97 | 0.88 | 1516 | 489 |  |
| PRDM2 ---- tag | rs1980472 | 1 | 1.15 (1.01-1.32) | 0.04 | 0.77 | 0.20 | 1516 | 489 |  |
| PRDM2 ---- tag | rs2235515 | 1 | 1.06 (0.92-1.23) | 0.42 | 0.96 | 0.72 | 1516 | 489 |  |
| PRDM2 ---- tag | rs2244634 | 1 | 0.94 (0.81-1.11) | 0.48 | 0.96 | 0.72 | 1516 | 489 |  |
| PRDM2 ---- tag | rs2245213 | 1 | 0.97 (0.82-1.15) | 0.73 | 0.97 | 0.88 | 1516 | 489 | low_count |
| PRDM2 ---- tag | rs2294484 | 1 | 1.02 (0.81-1.29) | 0.88 | 0.98 | 0.92 | 1516 | 489 | low_count |
| PRDM2 ---- tag | rs2744689 | 1 | 0.92 (0.78-1.10) | 0.37 | 0.96 | 0.72 | 1516 | 489 | low_count |
| PRDM2 ---- tag | rs6690270 | 1 | 0.94 (0.82-1.07) | 0.34 | 0.96 | 0.72 | 1516 | 489 |  |
| RRM1 ---- tag | rs10835601 | 1 | 1.01 (0.87-1.16) | 0.94 | 0.99 | 0.94 | 1516 | 489 |  |
| RRM1 ---- tag | rs10835613 | 1 | 1.05 (0.92-1.19) | 0.50 | 0.96 | 0.93 | 1516 | 489 |  |
| RRM1 ---- NA | rs10835677 | 1 | 0.98 (0.79-1.21) | 0.84 | 0.98 | 0.93 | 1516 | 489 | low_count |
| RRM1 ---- tag | rs10835678 | 1 | 0.97 (0.73-1.27) | 0.81 | 0.98 | 0.93 | 1516 | 489 | low_count |
| RRM1 ---- tag | rs12288551 | 1 | 1.14 (0.83-1.55) | 0.42 | 0.96 | 0.93 | 1516 | 489 | low_count |
| RRM1 ---- NA | rs12806698 | 1 | 1.06 (0.92-1.23) | 0.40 | 0.96 | 0.93 | 1516 | 489 |  |
| RRM1 ---- NA | rs1465952 | 1 | 0.97 (0.77-1.21) | 0.77 | 0.97 | 0.93 | 1516 | 489 | low_count |
| RRM1 ---- tag | rs4910904 | 1 | 1.09 (0.95-1.24) | 0.23 | 0.95 | 0.93 | 1516 | 489 |  |
| RRM1 ---- tag | rs7103860 | 1 | 1.05 (0.86-1.28) | 0.66 | 0.96 | 0.93 | 1516 | 489 | low_count |
| RRM1 ---- tag | rs7115496 | 1 | 0.93 (0.72-1.19) | 0.55 | 0.96 | 0.93 | 1516 | 489 | low_count |
| RRM2 ---- NA | rs1138729 | 1 | 1.09 (0.92-1.29) | 0.32 | 0.96 | 0.66 | 1516 | 489 |  |
| RRM2 ---- tag | rs4668664 | 1 | 0.93 (0.81-1.07) | 0.33 | 0.96 | 0.66 | 1516 | 489 |  |
| RRM2 ---- NA | rs6741290 | 1 | 1.00 (0.89-1.13) | 0.97 | 0.99 | 0.97 | 1516 | 489 |  |
| RRM2 ---- tag | rs7574663 | 1 | 1.01 (0.87-1.19) | 0.86 | 0.98 | 0.97 | 1516 | 489 |  |
| SHMT1 ---- candidate | rs1979277 | 1 | 1.01 (0.87-1.16) | 0.91 | 0.98 | 0.95 | 1516 | 489 |  |
| SHMT1 ---- tag | rs2168781 | 1 | 1.04 (0.91-1.18) | 0.61 | 0.96 | 0.95 | 1516 | 489 |  |
| SHMT1 ---- tag | rs4924849 | 1 | 1.00 (0.86-1.15) | 0.95 | 0.99 | 0.95 | 1516 | 489 |  |
| SHMT1 ---- candidate literature | rs9909104 | 1 | 1.00 (0.86-1.15) | 0.95 | 0.99 | 0.95 | 1516 | 489 |  |
| SHMT2 ---- tag | rs10876968 | 1 | 0.96 (0.83-1.11) | 0.56 | 0.96 | 0.70 | 1516 | 489 |  |
| SHMT2 ---- tag | rs1800165 | 1 | 1.10 (0.96-1.26) | 0.18 | 0.92 | 0.31 | 1516 | 489 |  |
| SHMT2 ---- tag | rs7133939 | 1 | 1.00 (0.88-1.14) | 0.96 | 0.99 | 0.96 | 1516 | 489 |  |
| SHMT2 ---- tag | rs7485577 | 1 | 1.10 (0.96-1.26) | 0.19 | 0.92 | 0.31 | 1516 | 489 |  |
| SHMT2 ---- tag | rs7489231 | 1 | 1.11 (0.98-1.27) | 0.11 | 0.80 | 0.31 | 1516 | 489 |  |
| SLC19A1 ---- candidate | rs1051266 | 1 | 0.91 (0.80-1.04) | 0.16 | 0.91 | 0.19 | 1516 | 489 |  |
| SLC19A1 ---- candidate literature | rs1131596 | 1 | 0.91 (0.80-1.04) | 0.16 | 0.91 | 0.19 | 1516 | 489 |  |
| SLC19A1 ---- tag | rs12483553 | 1 | 1.16 (0.95-1.41) | 0.16 | 0.91 | 0.19 | 1516 | 489 | low_count |
| SLC19A1 ---- candidate literature | rs12659 | 1 | 0.90 (0.79-1.02) | 0.10 | 0.80 | 0.19 | 1516 | 489 |  |
| SLC19A1 ---- tag | rs3788190 | 1 | 0.91 (0.80-1.04) | 0.16 | 0.91 | 0.19 | 1516 | 489 |  |
| SLC19A1 ---- tag | rs3788205 | 1 | 0.96 (0.83-1.12) | 0.62 | 0.96 | 0.62 | 1516 | 489 |  |
| SLC19A1 ---- tag | rs7279664 | 1 | 0.88 (0.77-1.00) | 0.06 | 0.77 | 0.19 | 1516 | 489 |  |
| SLC29A1 ---- NA | rs1057985 | 1 | 1.12 (0.98-1.28) | 0.10 | 0.80 | 0.25 | 1516 | 489 |  |
| SLC29A1 ---- NA | rs6458375 | 1 | 0.88 (0.76-1.03) | 0.11 | 0.80 | 0.25 | 1516 | 489 |  |
| SLC29A1 ---- NA | rs666462 | 1 | 1.02 (0.90-1.16) | 0.74 | 0.97 | 0.89 | 1516 | 489 |  |
| SLC29A1 ---- NA | rs6905285 | 1 | 1.00 (0.88-1.14) | 0.99 | 0.99 | 0.99 | 1516 | 489 |  |
| SLC29A1 ---- NA | rs693955 | 1 | 1.19 (1.01-1.39) | 0.04 | 0.73 | 0.25 | 1516 | 489 |  |
| SLC29A1 ---- NA | rs747199 | 1 | 1.02 (0.87-1.20) | 0.76 | 0.97 | 0.89 | 1516 | 489 |  |
| SLC29A1 ---- NA | rs9357436 | 1 | 1.06 (0.90-1.25) | 0.50 | 0.96 | 0.88 | 1516 | 489 |  |
| TCN2 ---- tag | rs10418 | 1 | 1.01 (0.86-1.18) | 0.92 | 0.98 | 0.96 | 1516 | 489 |  |
| TCN2 ---- candidate/singleton | rs1131603 | 1 | 1.11 (0.86-1.43) | 0.44 | 0.96 | 0.82 | 1516 | 489 | low_count |
| TCN2 ---- tag | rs1544468 | 1 | 1.04 (0.92-1.17) | 0.57 | 0.96 | 0.82 | 1516 | 489 |  |
| TCN2 ---- candidate/tag | rs1801198 | 1 | 0.94 (0.83-1.06) | 0.33 | 0.96 | 0.82 | 1516 | 489 |  |
| TCN2 ---- tag | rs4820872 | 1 | 0.98 (0.87-1.10) | 0.70 | 0.97 | 0.91 | 1516 | 489 |  |
| TCN2 ---- tag | rs4820874 | 1 | 0.91 (0.76-1.08) | 0.27 | 0.95 | 0.82 | 1516 | 489 |  |
| TCN2 ---- tag | rs4820886 | 1 | 1.00 (0.82-1.23) | 0.96 | 0.99 | 0.96 | 1516 | 489 | low_count |
| TCN2 ---- candidate | rs4820889 | 1 | 0.86 (0.60-1.24) | 0.43 | 0.96 | 0.82 | 1516 | 489 |  |
| TCN2 ---- tag | rs5997711 | 1 | 0.95 (0.84-1.08) | 0.46 | 0.96 | 0.82 | 1516 | 489 |  |
| TCN2 ---- tag | rs740234 | 1 | 1.05 (0.90-1.24) | 0.53 | 0.96 | 0.82 | 1516 | 489 |  |
| TCN2 ---- tag | rs740235 | 1 | 1.04 (0.92-1.18) | 0.52 | 0.96 | 0.82 | 1516 | 489 |  |
| TCN2 ---- candidate/singleton | rs9606756 | 1 | 1.06 (0.87-1.29) | 0.57 | 0.96 | 0.82 | 1516 | 489 | low_count |
| TCN2 ---- candidate | rs9621049 | 1 | 1.00 (0.82-1.23) | 0.96 | 0.99 | 0.96 | 1516 | 489 | low_count |
| TK1 ---- NA | rs1065769 | 1 | 1.01 (0.88-1.15) | 0.92 | 0.98 | 0.93 | 1516 | 489 |  |
| TK1 ---- NA | rs12232476 | 1 | 1.03 (0.82-1.30) | 0.78 | 0.97 | 0.93 | 1516 | 489 | low_count |
| TK1 ---- tag | rs16970907 | 1 | 0.91 (0.71-1.18) | 0.49 | 0.96 | 0.93 | 1516 | 489 | low_count |
| TK1 ---- tag | rs1811086 | 1 | 0.88 (0.62-1.26) | 0.49 | 0.96 | 0.93 | 1516 | 489 | low_count |
| TK1 ---- tag | rs2292235 | 1 | 0.94 (0.83-1.06) | 0.30 | 0.95 | 0.93 | 1516 | 489 |  |
| TK1 ---- tag | rs2854701 | 1 | 1.01 (0.89-1.15) | 0.84 | 0.98 | 0.93 | 1516 | 489 |  |
| TK1 ---- tag | rs2854702 | 1 | 0.99 (0.82-1.20) | 0.93 | 0.98 | 0.93 | 1516 | 489 | low_count |
| TK1 ---- tag | rs9897765 | 1 | 0.99 (0.86-1.14) | 0.88 | 0.98 | 0.93 | 1516 | 489 |  |
| TYMP ---- NA | rs131815 | 1 | 1.10 (0.96-1.27) | 0.18 | 0.92 | 0.28 | 1516 | 489 |  |
| TYMP ---- tag | rs131816 | 1 | 0.84 (0.72-0.98) | 0.02 | 0.73 | 0.14 | 1516 | 489 |  |
| TYMP ---- NA | rs131817 | 1 | 1.06 (0.93-1.20) | 0.40 | 0.96 | 0.40 | 1516 | 489 |  |
| TYMP ---- NA | rs140521 | 1 | 0.88 (0.76-1.01) | 0.08 | 0.79 | 0.24 | 1516 | 489 |  |
| TYMP ---- NA | rs140522 | 1 | 0.91 (0.79-1.04) | 0.17 | 0.91 | 0.28 | 1516 | 489 |  |
| TYMP ---- NA | rs140524 | 1 | 1.08 (0.92-1.27) | 0.34 | 0.96 | 0.40 | 1516 | 489 |  |
| TYMS ---- candidate literature | rs1001761 | 1 | 0.87 (0.76-0.99) | 0.04 | 0.73 | 0.11 | 1516 | 489 |  |
| TYMS ---- candidate literature/tag | rs10502289 | 1 | 0.91 (0.78-1.07) | 0.26 | 0.95 | 0.33 | 1516 | 489 |  |
| TYMS ---- tag | rs15872 | 1 | 0.87 (0.76-1.00) | 0.06 | 0.77 | 0.12 | 1516 | 489 |  |
| TYMS ---- tag | rs2244500 | 1 | 0.86 (0.76-0.99) | 0.03 | 0.73 | 0.11 | 1516 | 489 |  |
| TYMS ---- tag | rs2741182 | 1 | 1.00 (0.86-1.16) | 0.98 | 0.99 | 0.98 | 1516 | 489 |  |
| TYMS ---- candidate literature | rs2847149 | 1 | 0.87 (0.76-0.99) | 0.04 | 0.73 | 0.11 | 1516 | 489 |  |
| TYMS ---- candidate literature | rs2853533 | 1 | 0.94 (0.78-1.13) | 0.50 | 0.96 | 0.57 | 1516 | 489 | low_count |
| TYMS ---- tag | rs495139 | 1 | 1.12 (0.99-1.27) | 0.07 | 0.79 | 0.12 | 1516 | 489 |  |
| TYMS ---- candidate literature | rs502396 | 1 | 0.92 (0.80-1.04) | 0.18 | 0.92 | 0.27 | 1516 | 489 |  |
| UMPH2 ---- tag | rs2291028 | 1 | 1.08 (0.95-1.24) | 0.25 | 0.95 | 0.74 | 1516 | 489 |  |
| UMPH2 ---- NA | rs4789143 | 1 | 1.01 (0.82-1.24) | 0.95 | 0.99 | 0.95 | 1516 | 489 | low_count |
| UMPH2 ---- NA | rs750844 | 1 | 1.03 (0.89-1.19) | 0.68 | 0.97 | 0.95 | 1516 | 489 |  |
| UMPK ---- tag | rs11582877 | 1 | 1.01 (0.85-1.20) | 0.88 | 0.98 | 0.93 | 1516 | 489 |  |
| UMPK ---- tag | rs2622903 | 1 | 0.98 (0.86-1.13) | 0.81 | 0.98 | 0.93 | 1516 | 489 |  |
| UMPK ---- tag | rs2820989 | 1 | 0.99 (0.88-1.13) | 0.93 | 0.98 | 0.93 | 1516 | 489 |  |
| UMPK ---- tag | rs6660321 | 1 | 0.98 (0.82-1.18) | 0.86 | 0.98 | 0.93 | 1516 | 489 | low_count |
| UMPK ---- tag | rs6690084 | 1 | 1.10 (0.87-1.38) | 0.42 | 0.96 | 0.93 | 1516 | 489 | low_count |
| UMPS ---- NA | rs1162 | 1 | 1.12 (0.98-1.28) | 0.09 | 0.79 | 0.51 | 1516 | 489 |  |
| UMPS ---- tag | rs13146 | 1 | 1.05 (0.89-1.24) | 0.54 | 0.96 | 0.79 | 1516 | 489 |  |
| UMPS ---- tag | rs16835902 | 1 | 1.01 (0.89-1.15) | 0.90 | 0.98 | 0.90 | 1516 | 489 |  |
| UMPS ---- tag | rs17282057 | 1 | 1.05 (0.86-1.27) | 0.64 | 0.96 | 0.79 | 1516 | 489 |  |
| UMPS ---- tag | rs606552 | 1 | 0.93 (0.81-1.07) | 0.33 | 0.96 | 0.79 | 1516 | 489 |  |
| UMPS ---- tag | rs694897 | 1 | 1.03 (0.90-1.18) | 0.66 | 0.96 | 0.79 | 1516 | 489 |  |
| UNG ---- NA | rs1059262 | 1 | 0.95 (0.80-1.12) | 0.54 | 0.96 | 0.65 | 1516 | 489 |  |
| UNG ---- tag | rs2160603 | 1 | 1.07 (0.90-1.27) | 0.43 | 0.96 | 0.65 | 1516 | 489 |  |
| UNG ---- tag | rs246079 | 1 | 1.07 (0.94-1.22) | 0.30 | 0.95 | 0.65 | 1516 | 489 |  |
| UNG ---- NA | rs246085 | 1 | 1.02 (0.77-1.35) | 0.88 | 0.98 | 0.88 | 1516 | 489 | low_count |
| UNG ---- NA | rs2569987 | 1 | 0.90 (0.76-1.07) | 0.24 | 0.95 | 0.65 | 1516 | 489 |  |
| UNG ---- tag | rs3219243 | 1 | 1.06 (0.90-1.24) | 0.51 | 0.96 | 0.65 | 1516 | 489 |  |

*adjusted for age, sex, stage, grade, BMI, alcohol intake

**candidate, FDR-adjusted cut-off for significance of p-value = 0.02

^ǂ^dominant model (HR_het_)

^1^p_trend_:p-Value for trend

^2^p_trendFDR_:FDR adjusted trend

^3^p_FDRGenwide_:FDR adjusted genewide effect
