# Supplementary material for: Pathway analysis of genetic variants in folate‐mediated one‐carbon metabolism‐related genes and survival in a prospectively followed cohort of colorectal cancer patients
Source: Cancer Med. 2018 May 29;7(7):2797–807. doi: 10.1002/cam4.1407 (PMC6051204; doi:10.1002/cam4.1407)
Supplement: Supplementary file 6 — Table S8. Associations between selected polymorphisms in FOCM‐related genes and disease‐free survival. [file CAM4-7-2797-s006.docx]

| **Supplementary Table 8. Associations between selected polymorphisms in FOCM-related genes and disease-free survival** | | | | | | | | | | | | | |
| --- | --- | --- | --- | --- | --- | --- | --- | --- | --- | --- | --- | --- | --- |
| **Gene** | **SNP** | **Genotype** | **HR (95%-CI)** | **p** | **FDR_p** | **FDR_(byGene)_p_** | **N** | **%** | **N** | **%** | **NObsUsed** | **Events** | **LowCount** |
| AARS ---- tag | rs2070203 | T/T | 1.00 (.-.) | . | . | . | 329 | 27,17 | 147 | 27,53 | 1473 | 414 |  |
| AARS ---- tag |  | T/C or C/C | 1.13 (0.90-1.41) | 0.29 | 0.89 | 0.29 | 882 | 72,83 | 387 | 72,47 | 1473 | 414 |  |
| AARS ---- tag | rs34087264 | G/G | 1.00 (.-.) | . | . | . | 365 | 30,14 | 157 | 29,40 | 1473 | 414 |  |
| AARS ---- tag |  | G/A or A/A | 0.82 (0.67-1.02) | 0.07 | 0.84 | 0.15 | 846 | 69,86 | 377 | 70,60 | 1473 | 414 |  |
| ABCC4 ---- tag | rs10508023 | G/G | 1.00 (.-.) | . | . | . | 949 | 78,36 | 419 | 78,46 | 1473 | 414 |  |
| ABCC4 ---- tag |  | G/C or C/C | 0.96 (0.76-1.22) | 0.75 | 0.94 | 0.88 | 262 | 21,64 | 115 | 21,54 | 1473 | 414 |  |
| ABCC4 ---- tag | rs1059751 | T/T | 1.00 (.-.) | . | . | . | 351 | 28,98 | 146 | 27,34 | 1473 | 414 |  |
| ABCC4 ---- tag |  | T/C or C/C | 1.10 (0.88-1.37) | 0.40 | 0.91 | 0.80 | 860 | 71,02 | 388 | 72,66 | 1473 | 414 |  |
| ABCC4 ---- tag | rs11568643 | A/A | 1.00 (.-.) | . | . | . | 1011 | 83,48 | 443 | 82,96 | 1473 | 414 |  |
| ABCC4 ---- tag |  | A/G or G/G | 0.98 (0.75-1.27) | 0.86 | 0.97 | 0.94 | 200 | 16,52 | 91 | 17,04 | 1473 | 414 |  |
| ABCC4 ---- NA | rs11568658 | G/G | 1.00 (.-.) | . | . | . | 1143 | 94,38 | 510 | 95,51 | 1473 | 414 |  |
| ABCC4 ---- NA |  | G/T or T/T | 1.09 (0.69-1.74) | 0.71 | 0.93 | 0.88 | 68 | 5,62 | 24 | 4,49 | 1473 | 414 |  |
| ABCC4 ---- tag | rs12864049 | T/T | 1.00 (.-.) | . | . | . | 909 | 75,06 | 396 | 74,16 | 1473 | 414 |  |
| ABCC4 ---- tag |  | T/C or C/C | 1.09 (0.87-1.36) | 0.47 | 0.93 | 0.84 | 302 | 24,94 | 138 | 25,84 | 1473 | 414 |  |
| ABCC4 ---- tag | rs1628382 | G/G | 1.00 (.-.) | . | . | . | 758 | 62,59 | 329 | 61,61 | 1473 | 414 |  |
| ABCC4 ---- tag |  | G/A or A/A | 1.07 (0.88-1.31) | 0.50 | 0.93 | 0.84 | 453 | 37,41 | 205 | 38,39 | 1473 | 414 |  |
| ABCC4 ---- tag | rs1678354 | C/C | 1.00 (.-.) | . | . | . | 518 | 42,77 | 218 | 40,82 | 1473 | 414 |  |
| ABCC4 ---- tag |  | C/G or G/G | 1.18 (0.96-1.43) | 0.11 | 0.84 | 0.56 | 693 | 57,23 | 316 | 59,18 | 1473 | 414 |  |
| ABCC4 ---- tag | rs1678383 | T/T | 1.00 (.-.) | . | . | . | 981 | 81,01 | 432 | 80,90 | 1473 | 414 |  |
| ABCC4 ---- tag |  | T/G or G/G | 0.88 (0.68-1.13) | 0.31 | 0.89 | 0.76 | 230 | 18,99 | 102 | 19,10 | 1473 | 414 |  |
| ABCC4 ---- tag | rs1678395 | G/G | 1.00 (.-.) | . | . | . | 1050 | 86,71 | 456 | 85,39 | 1473 | 414 |  |
| ABCC4 ---- tag |  | G/A or A/A | 0.78 (0.59-1.04) | 0.09 | 0.84 | 0.56 | 161 | 13,29 | 78 | 14,61 | 1473 | 414 |  |
| ABCC4 ---- tag | rs1678405 | T/T | 1.00 (.-.) | . | . | . | 572 | 47,23 | 245 | 45,88 | 1473 | 414 |  |
| ABCC4 ---- tag |  | T/C or C/C | 1.13 (0.93-1.37) | 0.23 | 0.86 | 0.62 | 639 | 52,77 | 289 | 54,12 | 1473 | 414 |  |
| ABCC4 ---- tag | rs17189540 | A/A | 1.00 (.-.) | . | . | . | 1066 | 88,03 | 452 | 84,64 | 1473 | 414 |  |
| ABCC4 ---- tag |  | A/G or G/G | 1.16 (0.88-1.52) | 0.29 | 0.89 | 0.76 | 145 | 11,97 | 82 | 15,36 | 1473 | 414 |  |
| ABCC4 ---- tag | rs17235152 | T/T | 1.00 (.-.) | . | . | . | 883 | 72,91 | 389 | 72,85 | 1473 | 414 |  |
| ABCC4 ---- tag |  | T/C or C/C | 1.06 (0.85-1.32) | 0.60 | 0.93 | 0.88 | 328 | 27,09 | 145 | 27,15 | 1473 | 414 |  |
| ABCC4 ---- tag | rs17268122 | G/G | 1.00 (.-.) | . | . | . | 733 | 60,53 | 334 | 62,55 | 1473 | 414 |  |
| ABCC4 ---- tag |  | G/T or T/T | 0.94 (0.77-1.15) | 0.54 | 0.93 | 0.88 | 478 | 39,47 | 200 | 37,45 | 1473 | 414 |  |
| ABCC4 ---- tag | rs17268170 | C/C | 1.00 (.-.) | . | . | . | 1000 | 82,58 | 429 | 80,34 | 1473 | 414 |  |
| ABCC4 ---- tag |  | C/T or T/T | 1.00 (0.78-1.27) | 1.00 | 1.00 | 1.00 | 211 | 17,42 | 105 | 19,66 | 1473 | 414 |  |
| ABCC4 ---- tag | rs1729764 | A/A | 1.00 (.-.) | . | . | . | 954 | 78,78 | 435 | 81,46 | 1473 | 414 |  |
| ABCC4 ---- tag |  | A/G or G/G | 0.91 (0.71-1.18) | 0.49 | 0.93 | 0.84 | 257 | 21,22 | 99 | 18,54 | 1473 | 414 |  |
| ABCC4 ---- tag | rs1729767 | T/T | 1.00 (.-.) | . | . | . | 653 | 53,92 | 272 | 50,94 | 1473 | 414 |  |
| ABCC4 ---- tag |  | T/C or C/C | 1.17 (0.96-1.42) | 0.11 | 0.84 | 0.56 | 558 | 46,08 | 262 | 49,06 | 1473 | 414 |  |
| ABCC4 ---- tag | rs17300935 | C/C | 1.00 (.-.) | . | . | . | 886 | 73,16 | 397 | 74,34 | 1473 | 414 |  |
| ABCC4 ---- tag |  | C/G or G/G | 1.01 (0.80-1.26) | 0.96 | 0.99 | 0.98 | 325 | 26,84 | 137 | 25,66 | 1473 | 414 |  |
| ABCC4 ---- tag | rs1750190 | G/G | 1.00 (.-.) | . | . | . | 325 | 26,84 | 128 | 23,97 | 1473 | 414 |  |
| ABCC4 ---- tag |  | G/A or A/A | 1.19 (0.95-1.50) | 0.13 | 0.84 | 0.56 | 886 | 73,16 | 406 | 76,03 | 1473 | 414 |  |
| ABCC4 ---- tag | rs1750996 | A/A | 1.00 (.-.) | . | . | . | 813 | 67,13 | 362 | 67,79 | 1473 | 414 |  |
| ABCC4 ---- tag |  | A/G or G/G | 1.01 (0.82-1.25) | 0.91 | 0.98 | 0.97 | 398 | 32,87 | 172 | 32,21 | 1473 | 414 |  |
| ABCC4 ---- tag | rs1751025 | C/C | 1.00 (.-.) | . | . | . | 581 | 47,98 | 245 | 45,88 | 1473 | 414 |  |
| ABCC4 ---- tag |  | C/G or G/G | 1.16 (0.96-1.42) | 0.13 | 0.84 | 0.56 | 630 | 52,02 | 289 | 54,12 | 1473 | 414 |  |
| ABCC4 ---- tag | rs1751051 | T/T | 1.00 (.-.) | . | . | . | 521 | 43,02 | 219 | 41,01 | 1473 | 414 |  |
| ABCC4 ---- tag |  | T/A or A/A | 0.92 (0.76-1.13) | 0.44 | 0.93 | 0.84 | 690 | 56,98 | 315 | 58,99 | 1473 | 414 |  |
| ABCC4 ---- tag | rs1764416 | G/G | 1.00 (.-.) | . | . | . | 1039 | 85,80 | 462 | 86,52 | 1473 | 414 |  |
| ABCC4 ---- tag |  | G/A or A/A | 0.93 (0.69-1.26) | 0.65 | 0.93 | 0.88 | 172 | 14,20 | 72 | 13,48 | 1473 | 414 |  |
| ABCC4 ---- tag | rs2274401 | T/T | 1.00 (.-.) | . | . | . | 752 | 62,10 | 335 | 62,73 | 1473 | 414 |  |
| ABCC4 ---- tag |  | T/C or C/C | 0.98 (0.80-1.20) | 0.85 | 0.97 | 0.94 | 459 | 37,90 | 199 | 37,27 | 1473 | 414 |  |
| ABCC4 ---- tag | rs2892716 | C/C | 1.00 (.-.) | . | . | . | 463 | 38,23 | 215 | 40,26 | 1473 | 414 |  |
| ABCC4 ---- tag |  | C/T or T/T | 1.15 (0.94-1.41) | 0.18 | 0.84 | 0.56 | 748 | 61,77 | 319 | 59,74 | 1473 | 414 |  |
| ABCC4 ---- tag | rs3782964 | C/C | 1.00 (.-.) | . | . | . | 801 | 66,14 | 378 | 70,79 | 1473 | 414 |  |
| ABCC4 ---- tag |  | C/T or T/T | 0.87 (0.70-1.08) | 0.21 | 0.84 | 0.60 | 410 | 33,86 | 156 | 29,21 | 1473 | 414 |  |
| ABCC4 ---- tag | rs3818494 | C/C | 1.00 (.-.) | . | . | . | 543 | 44,84 | 236 | 44,19 | 1473 | 414 |  |
| ABCC4 ---- tag |  | C/G or G/G | 1.31 (1.07-1.60) | 0.01 | 0.84 | 0.42 | 668 | 55,16 | 298 | 55,81 | 1473 | 414 |  |
| ABCC4 ---- tag | rs3864997 | G/G | 1.00 (.-.) | . | . | . | 309 | 25,52 | 141 | 26,40 | 1473 | 414 |  |
| ABCC4 ---- tag |  | G/T or T/T | 1.05 (0.84-1.31) | 0.69 | 0.93 | 0.88 | 902 | 74,48 | 393 | 73,60 | 1473 | 414 |  |
| ABCC4 ---- tag | rs4148421 | G/G | 1.00 (.-.) | . | . | . | 361 | 29,81 | 148 | 27,72 | 1473 | 414 |  |
| ABCC4 ---- tag |  | G/A or A/A | 0.98 (0.79-1.22) | 0.85 | 0.97 | 0.94 | 850 | 70,19 | 386 | 72,28 | 1473 | 414 |  |
| ABCC4 ---- tag | rs4148446 | G/G | 1.00 (.-.) | . | . | . | 401 | 33,11 | 193 | 36,14 | 1473 | 414 |  |
| ABCC4 ---- tag |  | G/A or A/A | 1.06 (0.86-1.30) | 0.60 | 0.93 | 0.88 | 810 | 66,89 | 341 | 63,86 | 1473 | 414 |  |
| ABCC4 ---- tag | rs4148455 | G/G | 1.00 (.-.) | . | . | . | 922 | 76,14 | 402 | 75,28 | 1473 | 414 |  |
| ABCC4 ---- tag |  | G/A or A/A | 1.22 (0.98-1.53) | 0.08 | 0.84 | 0.56 | 289 | 23,86 | 132 | 24,72 | 1473 | 414 |  |
| ABCC4 ---- tag | rs4148540 | C/C | 1.00 (.-.) | . | . | . | 1085 | 89,60 | 462 | 86,52 | 1473 | 414 |  |
| ABCC4 ---- tag |  | C/T or T/T | 0.99 (0.75-1.31) | 0.94 | 0.99 | 0.98 | 126 | 10,40 | 72 | 13,48 | 1473 | 414 |  |
| ABCC4 ---- tag | rs4148542 | G/G | 1.00 (.-.) | . | . | . | 335 | 27,66 | 144 | 26,97 | 1473 | 414 |  |
| ABCC4 ---- tag |  | G/A or A/A | 0.98 (0.79-1.23) | 0.87 | 0.97 | 0.94 | 876 | 72,34 | 390 | 73,03 | 1473 | 414 |  |
| ABCC4 ---- tag | rs4148544 | G/G | 1.00 (.-.) | . | . | . | 502 | 41,45 | 247 | 46,25 | 1473 | 414 |  |
| ABCC4 ---- tag |  | G/A or A/A | 0.82 (0.68-1.00) | 0.05 | 0.84 | 0.56 | 709 | 58,55 | 287 | 53,75 | 1473 | 414 |  |
| ABCC4 ---- tag | rs4283094 | C/C | 1.00 (.-.) | . | . | . | 321 | 26,51 | 114 | 21,35 | 1473 | 414 |  |
| ABCC4 ---- tag |  | C/G or G/G | 1.09 (0.86-1.38) | 0.46 | 0.93 | 0.84 | 890 | 73,49 | 420 | 78,65 | 1473 | 414 |  |
| ABCC4 ---- tag | rs4636781 | A/A | 1.00 (.-.) | . | . | . | 866 | 71,51 | 377 | 70,60 | 1473 | 414 |  |
| ABCC4 ---- tag |  | A/G or G/G | 1.27 (1.03-1.58) | 0.03 | 0.84 | 0.56 | 345 | 28,49 | 157 | 29,40 | 1473 | 414 |  |
| ABCC4 ---- tag | rs4771910 | T/T | 1.00 (.-.) | . | . | . | 595 | 49,13 | 261 | 48,88 | 1473 | 414 |  |
| ABCC4 ---- tag |  | T/C or C/C | 0.96 (0.79-1.17) | 0.66 | 0.93 | 0.88 | 616 | 50,87 | 273 | 51,12 | 1473 | 414 |  |
| ABCC4 ---- tag | rs4773850 | T/T | 1.00 (.-.) | . | . | . | 547 | 45,17 | 273 | 51,12 | 1473 | 414 |  |
| ABCC4 ---- tag |  | T/G or G/G | 0.87 (0.71-1.05) | 0.14 | 0.84 | 0.56 | 664 | 54,83 | 261 | 48,88 | 1473 | 414 |  |
| ABCC4 ---- tag | rs7981095 | A/A | 1.00 (.-.) | . | . | . | 781 | 64,49 | 343 | 64,23 | 1473 | 414 |  |
| ABCC4 ---- tag |  | A/T or T/T | 0.87 (0.70-1.07) | 0.18 | 0.84 | 0.56 | 430 | 35,51 | 191 | 35,77 | 1473 | 414 |  |
| ABCC4 ---- tag | rs8001444 | C/C | 1.00 (.-.) | . | . | . | 419 | 34,60 | 177 | 33,15 | 1473 | 414 |  |
| ABCC4 ---- tag |  | C/T or T/T | 0.94 (0.77-1.16) | 0.57 | 0.93 | 0.88 | 792 | 65,40 | 357 | 66,85 | 1473 | 414 |  |
| ABCC4 ---- tag | rs931111 | T/T | 1.00 (.-.) | . | . | . | 822 | 67,88 | 349 | 65,36 | 1473 | 414 |  |
| ABCC4 ---- tag |  | T/C or C/C | 1.10 (0.90-1.35) | 0.37 | 0.90 | 0.79 | 389 | 32,12 | 185 | 34,64 | 1473 | 414 |  |
| ABCC4 ---- tag | rs943288 | T/T | 1.00 (.-.) | . | . | . | 924 | 76,30 | 409 | 76,59 | 1473 | 414 |  |
| ABCC4 ---- tag |  | T/A or A/A | 1.17 (0.93-1.48) | 0.17 | 0.84 | 0.56 | 287 | 23,70 | 125 | 23,41 | 1473 | 414 |  |
| ABCC4 ---- tag | rs943290 | A/A | 1.00 (.-.) | . | . | . | 663 | 54,75 | 284 | 53,18 | 1473 | 414 |  |
| ABCC4 ---- tag |  | A/G or G/G | 0.91 (0.75-1.11) | 0.34 | 0.90 | 0.79 | 548 | 45,25 | 250 | 46,82 | 1473 | 414 |  |
| ABCC4 ---- tag | rs9516530 | C/C | 1.00 (.-.) | . | . | . | 663 | 54,75 | 294 | 55,06 | 1473 | 414 |  |
| ABCC4 ---- tag |  | C/T or T/T | 0.97 (0.79-1.18) | 0.73 | 0.94 | 0.88 | 548 | 45,25 | 240 | 44,94 | 1473 | 414 |  |
| ABCC4 ---- tag | rs9516551 | C/C | 1.00 (.-.) | . | . | . | 968 | 79,93 | 419 | 78,46 | 1473 | 414 |  |
| ABCC4 ---- tag |  | C/A or A/A | 0.90 (0.71-1.13) | 0.36 | 0.90 | 0.79 | 243 | 20,07 | 115 | 21,54 | 1473 | 414 |  |
| ABCC4 ---- tag | rs9524822 | T/T | 1.00 (.-.) | . | . | . | 780 | 64,41 | 351 | 65,73 | 1473 | 414 |  |
| ABCC4 ---- tag |  | T/C or C/C | 0.87 (0.70-1.07) | 0.17 | 0.84 | 0.56 | 431 | 35,59 | 183 | 34,27 | 1473 | 414 |  |
| ABCC4 ---- tag | rs9524861 | G/G | 1.00 (.-.) | . | . | . | 626 | 51,69 | 263 | 49,25 | 1473 | 414 |  |
| ABCC4 ---- tag |  | G/C or C/C | 0.92 (0.75-1.11) | 0.38 | 0.91 | 0.79 | 585 | 48,31 | 271 | 50,75 | 1473 | 414 |  |
| ABCC4 ---- tag | rs9524902 | T/T | 1.00 (.-.) | . | . | . | 330 | 27,25 | 157 | 29,40 | 1473 | 414 |  |
| ABCC4 ---- tag |  | T/C or C/C | 0.86 (0.70-1.07) | 0.18 | 0.84 | 0.56 | 881 | 72,75 | 377 | 70,60 | 1473 | 414 |  |
| ABCC4 ---- tag | rs9556455 | G/G | 1.00 (.-.) | . | . | . | 915 | 75,56 | 402 | 75,28 | 1473 | 414 |  |
| ABCC4 ---- tag |  | G/A or A/A | 1.04 (0.84-1.30) | 0.70 | 0.93 | 0.88 | 296 | 24,44 | 132 | 24,72 | 1473 | 414 |  |
| ABCC4 ---- NA | rs9561778 | G/G | 1.00 (.-.) | . | . | . | 793 | 65,48 | 348 | 65,17 | 1473 | 414 |  |
| ABCC4 ---- NA |  | G/T or T/T | 1.04 (0.85-1.28) | 0.69 | 0.93 | 0.88 | 418 | 34,52 | 186 | 34,83 | 1473 | 414 |  |
| ABCC4 ---- tag | rs9561811 | C/C | 1.00 (.-.) | . | . | . | 805 | 66,47 | 360 | 67,42 | 1473 | 414 |  |
| ABCC4 ---- tag |  | C/T or T/T | 1.15 (0.94-1.41) | 0.18 | 0.84 | 0.56 | 406 | 33,53 | 174 | 32,58 | 1473 | 414 |  |
| ABCC4 ---- tag | rs9590183 | T/T | 1.00 (.-.) | . | . | . | 1059 | 87,45 | 473 | 88,58 | 1473 | 414 |  |
| ABCC4 ---- tag |  | T/A or A/A | 0.71 (0.51-0.98) | 0.04 | 0.84 | 0.56 | 152 | 12,55 | 61 | 11,42 | 1473 | 414 |  |
| ABCC4 ---- tag | rs997777 | T/T | 1.00 (.-.) | . | . | . | 604 | 49,88 | 266 | 49,81 | 1473 | 414 |  |
| ABCC4 ---- tag |  | T/A or A/A | 1.03 (0.85-1.26) | 0.74 | 0.94 | 0.88 | 607 | 50,12 | 268 | 50,19 | 1473 | 414 |  |
| ADH1B ---- tag | rs1159918 | G/G | 1.00 (.-.) | . | . | . | 537 | 44,34 | 244 | 45,69 | 1473 | 414 |  |
| ADH1B ---- tag |  | G/T or T/T | 1.02 (0.84-1.24) | 0.83 | 0.96 | 0.83 | 674 | 55,66 | 290 | 54,31 | 1473 | 414 |  |
| ADH1B ---- candidate literature | rs1229984 | G/G | 1.00 (.-.) | . | . | . | 1090 | 90,01 | 490 | 91,76 | 1473 | 414 |  |
| ADH1B ---- candidate literature |  | G/A or A/A | 0.74 (0.52-1.07) | 0.11 | 0.84 | 0.44 | 121 | 9,99 | 44 | 8,24 | 1473 | 414 |  |
| ADH1B ---- tag | rs12507573 | C/C | 1.00 (.-.) | . | . | . | 379 | 31,30 | 161 | 30,15 | 1473 | 414 |  |
| ADH1B ---- tag |  | C/A or A/A | 0.91 (0.74-1.13) | 0.39 | 0.91 | 0.57 | 832 | 68,70 | 373 | 69,85 | 1473 | 414 |  |
| ADH1B ---- tag | rs1693457 | T/T | 1.00 (.-.) | . | . | . | 834 | 68,87 | 386 | 72,28 | 1473 | 414 |  |
| ADH1B ---- tag |  | T/C or C/C | 0.86 (0.69-1.07) | 0.18 | 0.84 | 0.44 | 377 | 31,13 | 148 | 27,72 | 1473 | 414 |  |
| ADH1B ---- tag | rs2066701 | C/C | 1.00 (.-.) | . | . | . | 554 | 45,75 | 230 | 43,07 | 1473 | 414 |  |
| ADH1B ---- tag |  | C/T or T/T | 1.08 (0.89-1.31) | 0.45 | 0.93 | 0.57 | 657 | 54,25 | 304 | 56,93 | 1473 | 414 |  |
| ADH1C ---- tag | rs11936869 | C/C | 1.00 (.-.) | . | . | . | 619 | 51,11 | 287 | 53,75 | 1473 | 414 |  |
| ADH1C ---- tag |  | C/G or G/G | 0.96 (0.79-1.17) | 0.69 | 0.93 | 0.83 | 592 | 48,89 | 247 | 46,25 | 1473 | 414 |  |
| ADH1C ---- tag | rs1229849 | T/T | 1.00 (.-.) | . | . | . | 653 | 53,92 | 269 | 50,37 | 1473 | 414 |  |
| ADH1C ---- tag |  | T/A or A/A | 1.11 (0.91-1.35) | 0.30 | 0.89 | 0.83 | 558 | 46,08 | 265 | 49,63 | 1473 | 414 |  |
| ADH1C ---- tag | rs1229863 | A/A | 1.00 (.-.) | . | . | . | 892 | 73,66 | 386 | 72,28 | 1473 | 414 |  |
| ADH1C ---- tag |  | A/T or T/T | 0.98 (0.78-1.22) | 0.83 | 0.96 | 0.83 | 319 | 26,34 | 148 | 27,72 | 1473 | 414 |  |
| ADH1C ---- tag | rs1229980 | C/C | 1.00 (.-.) | . | . | . | 1095 | 90,42 | 467 | 87,45 | 1473 | 414 |  |
| ADH1C ---- tag |  | C/G or G/G | 1.26 (0.94-1.70) | 0.13 | 0.84 | 0.83 | 116 | 9,58 | 67 | 12,55 | 1473 | 414 |  |
| ADH1C ---- candidate | rs1693482 | C/C | 1.00 (.-.) | . | . | . | 507 | 41,87 | 213 | 39,89 | 1473 | 414 |  |
| ADH1C ---- candidate |  | C/T or T/T | 1.02 (0.84-1.25) | 0.81 | 0.96 | 0.83 | 704 | 58,13 | 321 | 60,11 | 1473 | 414 |  |
| ADH1C ---- tag | rs2173201 | C/C | 1.00 (.-.) | . | . | . | 685 | 56,56 | 319 | 59,74 | 1473 | 414 |  |
| ADH1C ---- tag |  | C/A or A/A | 0.97 (0.79-1.18) | 0.74 | 0.94 | 0.83 | 526 | 43,44 | 215 | 40,26 | 1473 | 414 |  |
| ADH1C ---- tag | rs2298753 | T/T | 1.00 (.-.) | . | . | . | 995 | 82,16 | 446 | 83,52 | 1473 | 414 |  |
| ADH1C ---- tag |  | T/C or C/C | 0.85 (0.65-1.11) | 0.24 | 0.87 | 0.83 | 216 | 17,84 | 88 | 16,48 | 1473 | 414 |  |
| ADH1C ---- tag | rs2866152 | G/G | 1.00 (.-.) | . | . | . | 744 | 61,44 | 314 | 58,80 | 1473 | 414 |  |
| ADH1C ---- tag |  | G/C or C/C | 1.04 (0.86-1.27) | 0.67 | 0.93 | 0.83 | 467 | 38,56 | 220 | 41,20 | 1473 | 414 |  |
| ADH1C ---- tag | rs904096 | T/T | 1.00 (.-.) | . | . | . | 501 | 41,37 | 210 | 39,33 | 1473 | 414 |  |
| ADH1C ---- tag |  | T/G or G/G | 1.02 (0.84-1.25) | 0.83 | 0.96 | 0.83 | 710 | 58,63 | 324 | 60,67 | 1473 | 414 |  |
| BHMT ---- tag | rs10944 | A/A | 1.00 (.-.) | . | . | . | 288 | 23,78 | 131 | 24,53 | 1473 | 414 |  |
| BHMT ---- tag |  | A/C or C/C | 0.88 (0.70-1.10) | 0.26 | 0.89 | 0.52 | 923 | 76,22 | 403 | 75,47 | 1473 | 414 |  |
| BHMT ---- tag | rs12655567 | C/C | 1.00 (.-.) | . | . | . | 437 | 36,09 | 214 | 40,07 | 1473 | 414 |  |
| BHMT ---- tag |  | C/G or G/G | 0.80 (0.65-0.98) | 0.03 | 0.84 | 0.10 | 774 | 63,91 | 320 | 59,93 | 1473 | 414 |  |
| BHMT ---- tag | rs1291041 | G/G | 1.00 (.-.) | . | . | . | 499 | 41,21 | 245 | 45,88 | 1473 | 414 |  |
| BHMT ---- tag |  | G/T or T/T | 0.78 (0.64-0.95) | 0.02 | 0.84 | 0.10 | 712 | 58,79 | 289 | 54,12 | 1473 | 414 |  |
| BHMT ---- tag | rs16876500 | C/C | 1.00 (.-.) | . | . | . | 965 | 79,69 | 422 | 79,03 | 1473 | 414 |  |
| BHMT ---- tag |  | C/T or T/T | 1.07 (0.84-1.37) | 0.56 | 0.93 | 0.70 | 246 | 20,31 | 112 | 20,97 | 1473 | 414 |  |
| BHMT ---- tag | rs492842 | A/A | 1.00 (.-.) | . | . | . | 443 | 36,58 | 209 | 39,14 | 1473 | 414 |  |
| BHMT ---- tag |  | A/G or G/G | 0.96 (0.79-1.17) | 0.71 | 0.93 | 0.71 | 768 | 63,42 | 325 | 60,86 | 1473 | 414 |  |
| BHMT ---- tag | rs558133 | T/T | 1.00 (.-.) | . | . | . | 579 | 47,81 | 257 | 48,13 | 1473 | 414 |  |
| BHMT ---- tag |  | T/G or G/G | 0.90 (0.74-1.10) | 0.30 | 0.89 | 0.52 | 632 | 52,19 | 277 | 51,87 | 1473 | 414 |  |
| BHMT ---- tag | rs9637824 | A/A | 1.00 (.-.) | . | . | . | 444 | 36,66 | 203 | 38,01 | 1473 | 414 |  |
| BHMT ---- tag |  | A/G or G/G | 0.95 (0.78-1.16) | 0.60 | 0.93 | 0.70 | 767 | 63,34 | 331 | 61,99 | 1473 | 414 |  |
| BHMT2 ---- tag | rs16876512 | C/C | 1.00 (.-.) | . | . | . | 958 | 79,11 | 421 | 78,84 | 1473 | 414 |  |
| BHMT2 ---- tag |  | C/T or T/T | 1.06 (0.84-1.36) | 0.61 | 0.93 | 0.65 | 253 | 20,89 | 113 | 21,16 | 1473 | 414 |  |
| BHMT2 ---- tag | rs2461248 | A/A | 1.00 (.-.) | . | . | . | 283 | 23,37 | 130 | 24,34 | 1473 | 414 |  |
| BHMT2 ---- tag |  | A/T or T/T | 0.88 (0.70-1.10) | 0.26 | 0.89 | 0.53 | 928 | 76,63 | 404 | 75,66 | 1473 | 414 |  |
| BHMT2 ---- tag | rs2909856 | T/T | 1.00 (.-.) | . | . | . | 486 | 40,13 | 225 | 42,13 | 1473 | 414 |  |
| BHMT2 ---- tag |  | T/C or C/C | 0.95 (0.78-1.15) | 0.59 | 0.93 | 0.65 | 725 | 59,87 | 309 | 57,87 | 1473 | 414 |  |
| BHMT2 ---- tag | rs476620 | A/A | 1.00 (.-.) | . | . | . | 445 | 36,75 | 202 | 37,83 | 1473 | 414 |  |
| BHMT2 ---- tag |  | A/G or G/G | 0.95 (0.78-1.17) | 0.65 | 0.93 | 0.65 | 766 | 63,25 | 332 | 62,17 | 1473 | 414 |  |
| BHMT2 ---- candidate literature | rs626105 | G/G | 1.00 (.-.) | . | . | . | 753 | 62,18 | 341 | 63,86 | 1473 | 414 |  |
| BHMT2 ---- candidate literature |  | G/A or A/A | 0.84 (0.68-1.03) | 0.09 | 0.84 | 0.53 | 458 | 37,82 | 193 | 36,14 | 1473 | 414 |  |
| BHMT2 ---- tag | rs631305 | G/G | 1.00 (.-.) | . | . | . | 830 | 68,54 | 374 | 70,04 | 1473 | 414 |  |
| BHMT2 ---- tag |  | G/A or A/A | 0.87 (0.70-1.08) | 0.22 | 0.85 | 0.53 | 381 | 31,46 | 160 | 29,96 | 1473 | 414 |  |
| CBS ---- tag | rs11701048 | C/C | 1.00 (.-.) | . | . | . | 1024 | 84,56 | 465 | 87,08 | 1473 | 414 |  |
| CBS ---- tag |  | C/T or T/T | 0.93 (0.70-1.24) | 0.64 | 0.93 | 0.64 | 187 | 15,44 | 69 | 12,92 | 1473 | 414 |  |
| CBS ---- tag | rs234706 | G/G | 1.00 (.-.) | . | . | . | 535 | 44,18 | 228 | 42,70 | 1473 | 414 |  |
| CBS ---- tag |  | G/A or A/A | 1.05 (0.86-1.28) | 0.64 | 0.93 | 0.64 | 676 | 55,82 | 306 | 57,30 | 1473 | 414 |  |
| CBS ---- tag | rs234711 | C/C | 1.00 (.-.) | . | . | . | 728 | 60,12 | 305 | 57,12 | 1473 | 414 |  |
| CBS ---- tag |  | C/A or A/A | 1.14 (0.93-1.39) | 0.20 | 0.84 | 0.55 | 483 | 39,88 | 229 | 42,88 | 1473 | 414 |  |
| CBS ---- candidate literature | rs234713 | G/G | 1.00 (.-.) | . | . | . | 602 | 49,71 | 263 | 49,25 | 1473 | 414 |  |
| CBS ---- candidate literature |  | G/A or A/A | 1.06 (0.87-1.29) | 0.56 | 0.93 | 0.64 | 609 | 50,29 | 271 | 50,75 | 1473 | 414 |  |
| CBS ---- tag | rs2839623 | T/T | 1.00 (.-.) | . | . | . | 1000 | 82,58 | 440 | 82,40 | 1473 | 414 |  |
| CBS ---- tag |  | T/A or A/A | 1.06 (0.82-1.38) | 0.63 | 0.93 | 0.64 | 211 | 17,42 | 94 | 17,60 | 1473 | 414 |  |
| CBS ---- tag | rs2839626 | C/C | 1.00 (.-.) | . | . | . | 560 | 46,24 | 234 | 43,82 | 1473 | 414 |  |
| CBS ---- tag |  | C/T or T/T | 1.13 (0.93-1.38) | 0.22 | 0.85 | 0.55 | 651 | 53,76 | 300 | 56,18 | 1473 | 414 |  |
| CBS ---- tag | rs422791 | T/T | 1.00 (.-.) | . | . | . | 618 | 51,03 | 262 | 49,06 | 1473 | 414 |  |
| CBS ---- tag |  | T/C or C/C | 1.16 (0.96-1.41) | 0.13 | 0.84 | 0.55 | 593 | 48,97 | 272 | 50,94 | 1473 | 414 |  |
| CBS ---- tag | rs706209 | C/C | 1.00 (.-.) | . | . | . | 371 | 30,64 | 161 | 30,15 | 1473 | 414 |  |
| CBS ---- tag |  | C/T or T/T | 0.94 (0.76-1.17) | 0.59 | 0.93 | 0.64 | 840 | 69,36 | 373 | 69,85 | 1473 | 414 |  |
| CBS ---- tag | rs719037 | A/A | 1.00 (.-.) | . | . | . | 417 | 34,43 | 174 | 32,58 | 1473 | 414 |  |
| CBS ---- tag |  | A/G or G/G | 1.19 (0.96-1.46) | 0.11 | 0.84 | 0.55 | 794 | 65,57 | 360 | 67,42 | 1473 | 414 |  |
| CBS ---- tag | rs719038 | T/T | 1.00 (.-.) | . | . | . | 524 | 43,27 | 213 | 39,89 | 1473 | 414 |  |
| CBS ---- tag |  | T/C or C/C | 1.06 (0.87-1.30) | 0.55 | 0.93 | 0.64 | 687 | 56,73 | 321 | 60,11 | 1473 | 414 |  |
| DHFR ---- tag | rs10474632 | G/G | 1.00 (.-.) | . | . | . | 1020 | 84,23 | 453 | 84,83 | 1473 | 414 |  |
| DHFR ---- tag |  | G/A or A/A | 0.91 (0.69-1.19) | 0.49 | 0.93 | 0.69 | 191 | 15,77 | 81 | 15,17 | 1473 | 414 |  |
| DHFR ---- tag | rs11951910 | T/T | 1.00 (.-.) | . | . | . | 976 | 80,59 | 429 | 80,34 | 1473 | 414 |  |
| DHFR ---- tag |  | T/C or C/C | 1.15 (0.90-1.47) | 0.27 | 0.89 | 0.57 | 235 | 19,41 | 105 | 19,66 | 1473 | 414 |  |
| DHFR ---- tag | rs1643665 | T/T | 1.00 (.-.) | . | . | . | 568 | 46,90 | 252 | 47,19 | 1473 | 414 |  |
| DHFR ---- tag |  | T/C or C/C | 0.95 (0.78-1.16) | 0.63 | 0.93 | 0.70 | 643 | 53,10 | 282 | 52,81 | 1473 | 414 |  |
| DHFR ---- tag | rs1650717 | T/T | 1.00 (.-.) | . | . | . | 661 | 54,58 | 277 | 51,87 | 1473 | 414 |  |
| DHFR ---- tag |  | T/G or G/G | 1.16 (0.95-1.41) | 0.15 | 0.84 | 0.49 | 550 | 45,42 | 257 | 48,13 | 1473 | 414 |  |
| DHFR ---- tag | rs1805355 | G/G | 1.00 (.-.) | . | . | . | 1067 | 88,11 | 457 | 85,58 | 1473 | 414 |  |
| DHFR ---- tag |  | G/A or A/A | 1.29 (0.97-1.70) | 0.08 | 0.84 | 0.38 | 144 | 11,89 | 77 | 14,42 | 1473 | 414 |  |
| DHFR ---- tag | rs6151617 | A/A | 1.00 (.-.) | . | . | . | 449 | 37,08 | 193 | 36,14 | 1473 | 414 |  |
| DHFR ---- tag |  | A/G or G/G | 0.98 (0.80-1.19) | 0.81 | 0.96 | 0.81 | 762 | 62,92 | 341 | 63,86 | 1473 | 414 |  |
| DHFR ---- tag | rs6864493 | T/T | 1.00 (.-.) | . | . | . | 679 | 56,07 | 315 | 58,99 | 1473 | 414 |  |
| DHFR ---- tag |  | T/C or C/C | 0.83 (0.68-1.02) | 0.07 | 0.84 | 0.38 | 532 | 43,93 | 219 | 41,01 | 1473 | 414 |  |
| DHFR ---- tag | rs836788 | G/G | 1.00 (.-.) | . | . | . | 519 | 42,86 | 226 | 42,32 | 1473 | 414 |  |
| DHFR ---- tag |  | G/A or A/A | 1.05 (0.86-1.28) | 0.61 | 0.93 | 0.70 | 692 | 57,14 | 308 | 57,68 | 1473 | 414 |  |
| DHFR ---- tag | rs836790 | A/A | 1.00 (.-.) | . | . | . | 855 | 70,60 | 360 | 67,42 | 1473 | 414 |  |
| DHFR ---- tag |  | A/G or G/G | 1.11 (0.90-1.36) | 0.34 | 0.90 | 0.57 | 356 | 29,40 | 174 | 32,58 | 1473 | 414 |  |
| DHFR ---- tag | rs836817 | G/G | 1.00 (.-.) | . | . | . | 568 | 46,90 | 242 | 45,32 | 1473 | 414 |  |
| DHFR ---- tag |  | G/T or T/T | 1.11 (0.91-1.35) | 0.31 | 0.89 | 0.57 | 643 | 53,10 | 292 | 54,68 | 1473 | 414 |  |
| DNMT1 ---- candidate | rs2228612 | A/A | 1.00 (.-.) | . | . | . | 1046 | 86,37 | 463 | 86,70 | 1473 | 414 |  |
| DNMT1 ---- candidate |  | A/G or G/G | 1.05 (0.80-1.40) | 0.72 | 0.93 | 0.72 | 165 | 13,63 | 71 | 13,30 | 1473 | 414 |  |
| DNMT3A ---- tag | rs10460566 | A/A | 1.00 (.-.) | . | . | . | 676 | 55,82 | 329 | 61,61 | 1473 | 414 |  |
| DNMT3A ---- tag |  | A/G or G/G | 0.97 (0.80-1.19) | 0.79 | 0.95 | 0.79 | 535 | 44,18 | 205 | 38,39 | 1473 | 414 |  |
| DNMT3A ---- candidate literature | rs11695471 | T/T | 1.00 (.-.) | . | . | . | 539 | 44,51 | 233 | 43,63 | 1473 | 414 |  |
| DNMT3A ---- candidate literature |  | T/A or A/A | 1.05 (0.87-1.28) | 0.59 | 0.93 | 0.69 | 672 | 55,49 | 301 | 56,37 | 1473 | 414 |  |
| DNMT3A ---- tag | rs11887120 | C/C | 1.00 (.-.) | . | . | . | 423 | 34,93 | 188 | 35,21 | 1473 | 414 |  |
| DNMT3A ---- tag |  | C/T or T/T | 0.93 (0.76-1.14) | 0.49 | 0.93 | 0.69 | 788 | 65,07 | 346 | 64,79 | 1473 | 414 |  |
| DNMT3A ---- tag | rs12991495 | T/T | 1.00 (.-.) | . | . | . | 584 | 48,22 | 249 | 46,63 | 1473 | 414 |  |
| DNMT3A ---- tag |  | T/C or C/C | 1.06 (0.87-1.29) | 0.56 | 0.93 | 0.69 | 627 | 51,78 | 285 | 53,37 | 1473 | 414 |  |
| DNMT3A ---- tag | rs13401241 | A/A | 1.00 (.-.) | . | . | . | 342 | 28,24 | 149 | 27,90 | 1473 | 414 |  |
| DNMT3A ---- tag |  | A/C or C/C | 0.94 (0.75-1.18) | 0.60 | 0.93 | 0.69 | 869 | 71,76 | 385 | 72,10 | 1473 | 414 |  |
| DNMT3A ---- candidate literature | rs13420827 | C/C | 1.00 (.-.) | . | . | . | 798 | 65,90 | 360 | 67,42 | 1473 | 414 |  |
| DNMT3A ---- candidate literature |  | C/G or G/G | 0.92 (0.75-1.14) | 0.45 | 0.93 | 0.69 | 413 | 34,10 | 174 | 32,58 | 1473 | 414 |  |
| DNMT3A ---- tag | rs13428812 | A/A | 1.00 (.-.) | . | . | . | 569 | 46,99 | 257 | 48,13 | 1473 | 414 |  |
| DNMT3A ---- tag |  | A/G or G/G | 1.08 (0.89-1.32) | 0.42 | 0.93 | 0.69 | 642 | 53,01 | 277 | 51,87 | 1473 | 414 |  |
| DNMT3A ---- tag | rs4665287 | C/C | 1.00 (.-.) | . | . | . | 810 | 66,89 | 366 | 68,54 | 1473 | 414 |  |
| DNMT3A ---- tag |  | C/T or T/T | 0.90 (0.73-1.11) | 0.34 | 0.90 | 0.69 | 401 | 33,11 | 168 | 31,46 | 1473 | 414 |  |
| DNMT3B ---- tag | rs13045669 | A/A | 1.00 (.-.) | . | . | . | 1121 | 92,57 | 496 | 92,88 | 1473 | 414 |  |
| DNMT3B ---- tag |  | A/G or G/G | 0.69 (0.44-1.08) | 0.11 | 0.84 | 0.42 | 90 | 7,43 | 38 | 7,12 | 1473 | 414 |  |
| DNMT3B ---- tag | rs17123673 | A/A | 1.00 (.-.) | . | . | . | 1111 | 91,74 | 487 | 91,20 | 1473 | 414 |  |
| DNMT3B ---- tag |  | A/G or G/G | 0.98 (0.70-1.36) | 0.90 | 0.97 | 0.90 | 100 | 8,26 | 47 | 8,80 | 1473 | 414 |  |
| DNMT3B ---- tag | rs183603 | A/A | 1.00 (.-.) | . | . | . | 662 | 54,67 | 302 | 56,55 | 1473 | 414 |  |
| DNMT3B ---- tag |  | A/G or G/G | 0.85 (0.69-1.03) | 0.10 | 0.84 | 0.42 | 549 | 45,33 | 232 | 43,45 | 1473 | 414 |  |
| DNMT3B ---- tag | rs2235760 | C/C | 1.00 (.-.) | . | . | . | 864 | 71,35 | 371 | 69,48 | 1473 | 414 |  |
| DNMT3B ---- tag |  | C/T or T/T | 1.11 (0.90-1.38) | 0.32 | 0.89 | 0.51 | 347 | 28,65 | 163 | 30,52 | 1473 | 414 |  |
| DNMT3B ---- tag | rs2424908 | C/C | 1.00 (.-.) | . | . | . | 777 | 64,16 | 354 | 66,29 | 1473 | 414 |  |
| DNMT3B ---- tag |  | C/T or T/T | 0.85 (0.69-1.05) | 0.13 | 0.84 | 0.42 | 434 | 35,84 | 180 | 33,71 | 1473 | 414 |  |
| DNMT3B ---- candidate literature | rs2424909 | T/T | 1.00 (.-.) | . | . | . | 456 | 37,65 | 218 | 40,82 | 1473 | 414 |  |
| DNMT3B ---- candidate literature |  | T/C or C/C | 0.90 (0.74-1.10) | 0.30 | 0.89 | 0.51 | 755 | 62,35 | 316 | 59,18 | 1473 | 414 |  |
| DNMT3B ---- tag | rs4911108 | A/A | 1.00 (.-.) | . | . | . | 492 | 40,63 | 229 | 42,88 | 1473 | 414 |  |
| DNMT3B ---- tag |  | A/G or G/G | 0.90 (0.74-1.10) | 0.32 | 0.89 | 0.51 | 719 | 59,37 | 305 | 57,12 | 1473 | 414 |  |
| DNMT3B ---- tag | rs6058896 | C/C | 1.00 (.-.) | . | . | . | 1075 | 88,77 | 477 | 89,33 | 1473 | 414 |  |
| DNMT3B ---- tag |  | C/T or T/T | 1.15 (0.85-1.57) | 0.36 | 0.90 | 0.51 | 136 | 11,23 | 57 | 10,67 | 1473 | 414 |  |
| DNMT3B ---- tag | rs6119954 | G/G | 1.00 (.-.) | . | . | . | 836 | 69,03 | 371 | 69,48 | 1473 | 414 |  |
| DNMT3B ---- tag |  | G/A or A/A | 1.05 (0.85-1.30) | 0.67 | 0.93 | 0.74 | 375 | 30,97 | 163 | 30,52 | 1473 | 414 |  |
| DNMT3B ---- tag | rs6579038 | A/A | 1.00 (.-.) | . | . | . | 1067 | 88,11 | 476 | 89,14 | 1473 | 414 |  |
| DNMT3B ---- tag |  | A/G or G/G | 1.14 (0.84-1.55) | 0.40 | 0.91 | 0.51 | 144 | 11,89 | 58 | 10,86 | 1473 | 414 |  |
| DPYD ---- tag | rs1034215 | C/C | 1.00 (.-.) | . | . | . | 744 | 61,44 | 331 | 61,99 | 1473 | 414 |  |
| DPYD ---- tag |  | C/T or T/T | 1.02 (0.84-1.25) | 0.82 | 0.96 | 0.93 | 467 | 38,56 | 203 | 38,01 | 1473 | 414 |  |
| DPYD ---- tag | rs10783058 | T/T | 1.00 (.-.) | . | . | . | 521 | 43,02 | 210 | 39,33 | 1473 | 414 |  |
| DPYD ---- tag |  | T/C or C/C | 1.18 (0.96-1.44) | 0.11 | 0.84 | 0.47 | 690 | 56,98 | 324 | 60,67 | 1473 | 414 |  |
| DPYD ---- tag | rs10783070 | C/C | 1.00 (.-.) | . | . | . | 851 | 70,27 | 371 | 69,48 | 1473 | 414 |  |
| DPYD ---- tag |  | C/T or T/T | 1.26 (1.02-1.56) | 0.03 | 0.84 | 0.36 | 360 | 29,73 | 163 | 30,52 | 1473 | 414 |  |
| DPYD ---- tag | rs10875048 | G/G | 1.00 (.-.) | . | . | . | 806 | 66,56 | 354 | 66,29 | 1473 | 414 |  |
| DPYD ---- tag |  | G/A or A/A | 0.95 (0.77-1.17) | 0.61 | 0.93 | 0.84 | 405 | 33,44 | 180 | 33,71 | 1473 | 414 |  |
| DPYD ---- tag | rs10875055 | C/C | 1.00 (.-.) | . | . | . | 358 | 29,56 | 126 | 23,60 | 1473 | 414 |  |
| DPYD ---- tag |  | C/T or T/T | 1.30 (1.03-1.63) | 0.03 | 0.84 | 0.36 | 853 | 70,44 | 408 | 76,40 | 1473 | 414 |  |
| DPYD ---- tag | rs10875079 | A/A | 1.00 (.-.) | . | . | . | 311 | 25,68 | 150 | 28,09 | 1473 | 414 |  |
| DPYD ---- tag |  | A/G or G/G | 0.80 (0.64-0.99) | 0.04 | 0.84 | 0.36 | 900 | 74,32 | 384 | 71,91 | 1473 | 414 |  |
| DPYD ---- tag | rs10875085 | A/A | 1.00 (.-.) | . | . | . | 832 | 68,70 | 351 | 65,73 | 1473 | 414 |  |
| DPYD ---- tag |  | A/T or T/T | 1.08 (0.88-1.32) | 0.49 | 0.93 | 0.84 | 379 | 31,30 | 183 | 34,27 | 1473 | 414 |  |
| DPYD ---- tag | rs10875097 | G/G | 1.00 (.-.) | . | . | . | 843 | 69,61 | 354 | 66,29 | 1473 | 414 |  |
| DPYD ---- tag |  | G/A or A/A | 1.18 (0.96-1.45) | 0.12 | 0.84 | 0.47 | 368 | 30,39 | 180 | 33,71 | 1473 | 414 |  |
| DPYD ---- tag | rs11165781 | T/T | 1.00 (.-.) | . | . | . | 819 | 67,63 | 364 | 68,16 | 1473 | 414 |  |
| DPYD ---- tag |  | T/C or C/C | 1.01 (0.82-1.25) | 0.92 | 0.98 | 1.00 | 392 | 32,37 | 170 | 31,84 | 1473 | 414 |  |
| DPYD ---- tag | rs11165783 | T/T | 1.00 (.-.) | . | . | . | 667 | 55,08 | 294 | 55,06 | 1473 | 414 |  |
| DPYD ---- tag |  | T/C or C/C | 1.06 (0.87-1.29) | 0.54 | 0.93 | 0.84 | 544 | 44,92 | 240 | 44,94 | 1473 | 414 |  |
| DPYD ---- tag | rs11165873 | A/A | 1.00 (.-.) | . | . | . | 319 | 26,34 | 165 | 30,90 | 1473 | 414 |  |
| DPYD ---- tag |  | A/T or T/T | 0.82 (0.66-1.00) | 0.06 | 0.84 | 0.36 | 892 | 73,66 | 369 | 69,10 | 1473 | 414 |  |
| DPYD ---- tag | rs11165875 | T/T | 1.00 (.-.) | . | . | . | 498 | 41,12 | 200 | 37,45 | 1473 | 414 |  |
| DPYD ---- tag |  | T/C or C/C | 1.12 (0.92-1.38) | 0.25 | 0.89 | 0.67 | 713 | 58,88 | 334 | 62,55 | 1473 | 414 |  |
| DPYD ---- tag | rs11165881 | T/T | 1.00 (.-.) | . | . | . | 428 | 35,34 | 193 | 36,14 | 1473 | 414 |  |
| DPYD ---- tag |  | T/C or C/C | 1.07 (0.87-1.31) | 0.53 | 0.93 | 0.84 | 783 | 64,66 | 341 | 63,86 | 1473 | 414 |  |
| DPYD ---- tag | rs11587873 | C/C | 1.00 (.-.) | . | . | . | 670 | 55,33 | 328 | 61,42 | 1473 | 414 |  |
| DPYD ---- tag |  | C/T or T/T | 0.86 (0.70-1.05) | 0.13 | 0.84 | 0.47 | 541 | 44,67 | 206 | 38,58 | 1473 | 414 |  |
| DPYD ---- tag | rs12030174 | C/C | 1.00 (.-.) | . | . | . | 886 | 73,16 | 375 | 70,22 | 1473 | 414 |  |
| DPYD ---- tag |  | C/T or T/T | 1.03 (0.83-1.28) | 0.76 | 0.94 | 0.92 | 325 | 26,84 | 159 | 29,78 | 1473 | 414 |  |
| DPYD ---- tag | rs12046744 | A/A | 1.00 (.-.) | . | . | . | 644 | 53,18 | 298 | 55,81 | 1473 | 414 |  |
| DPYD ---- tag |  | A/C or C/C | 0.96 (0.79-1.17) | 0.69 | 0.93 | 0.87 | 567 | 46,82 | 236 | 44,19 | 1473 | 414 |  |
| DPYD ---- tag | rs12047910 | G/G | 1.00 (.-.) | . | . | . | 875 | 72,25 | 391 | 73,22 | 1473 | 414 |  |
| DPYD ---- tag |  | G/A or A/A | 0.93 (0.75-1.16) | 0.51 | 0.93 | 0.84 | 336 | 27,75 | 143 | 26,78 | 1473 | 414 |  |
| DPYD ---- tag | rs12073044 | T/T | 1.00 (.-.) | . | . | . | 969 | 80,02 | 419 | 78,46 | 1473 | 414 |  |
| DPYD ---- tag |  | T/A or A/A | 1.12 (0.88-1.42) | 0.36 | 0.90 | 0.76 | 242 | 19,98 | 115 | 21,54 | 1473 | 414 |  |
| DPYD ---- tag | rs12126093 | T/T | 1.00 (.-.) | . | . | . | 599 | 49,46 | 282 | 52,81 | 1473 | 414 |  |
| DPYD ---- tag |  | T/C or C/C | 0.85 (0.70-1.03) | 0.10 | 0.84 | 0.47 | 612 | 50,54 | 252 | 47,19 | 1473 | 414 |  |
| DPYD ---- tag | rs12134028 | C/C | 1.00 (.-.) | . | . | . | 1088 | 89,84 | 477 | 89,33 | 1473 | 414 |  |
| DPYD ---- tag |  | C/T or T/T | 0.76 (0.55-1.07) | 0.12 | 0.84 | 0.47 | 123 | 10,16 | 57 | 10,67 | 1473 | 414 |  |
| DPYD ---- tag | rs12740796 | T/T | 1.00 (.-.) | . | . | . | 906 | 74,81 | 400 | 74,91 | 1473 | 414 |  |
| DPYD ---- tag |  | T/C or C/C | 1.06 (0.84-1.32) | 0.63 | 0.93 | 0.84 | 305 | 25,19 | 134 | 25,09 | 1473 | 414 |  |
| DPYD ---- tag | rs1333717 | A/A | 1.00 (.-.) | . | . | . | 706 | 58,30 | 316 | 59,18 | 1473 | 414 |  |
| DPYD ---- tag |  | A/G or G/G | 1.02 (0.83-1.24) | 0.87 | 0.97 | 0.97 | 505 | 41,70 | 218 | 40,82 | 1473 | 414 |  |
| DPYD ---- tag | rs1413228 | A/A | 1.00 (.-.) | . | . | . | 965 | 79,69 | 429 | 80,34 | 1473 | 414 |  |
| DPYD ---- tag |  | A/G or G/G | 0.94 (0.73-1.20) | 0.62 | 0.93 | 0.84 | 246 | 20,31 | 105 | 19,66 | 1473 | 414 |  |
| DPYD ---- tag | rs1415681 | G/G | 1.00 (.-.) | . | . | . | 900 | 74,32 | 407 | 76,22 | 1473 | 414 |  |
| DPYD ---- tag |  | G/T or T/T | 1.03 (0.82-1.29) | 0.82 | 0.96 | 0.93 | 311 | 25,68 | 127 | 23,78 | 1473 | 414 |  |
| DPYD ---- tag | rs1514495 | C/C | 1.00 (.-.) | . | . | . | 699 | 57,72 | 319 | 59,74 | 1473 | 414 |  |
| DPYD ---- tag |  | C/T or T/T | 0.96 (0.78-1.17) | 0.66 | 0.93 | 0.84 | 512 | 42,28 | 215 | 40,26 | 1473 | 414 |  |
| DPYD ---- tag | rs1520658 | A/A | 1.00 (.-.) | . | . | . | 960 | 79,27 | 419 | 78,46 | 1473 | 414 |  |
| DPYD ---- tag |  | A/G or G/G | 1.10 (0.87-1.40) | 0.41 | 0.91 | 0.76 | 251 | 20,73 | 115 | 21,54 | 1473 | 414 |  |
| DPYD ---- NA | rs17116806 | C/C | 1.00 (.-.) | . | . | . | 798 | 65,90 | 335 | 62,73 | 1473 | 414 |  |
| DPYD ---- NA |  | C/A or A/A | 1.12 (0.91-1.37) | 0.29 | 0.89 | 0.70 | 413 | 34,10 | 199 | 37,27 | 1473 | 414 |  |
| DPYD ---- tag | rs17431828 | G/G | 1.00 (.-.) | . | . | . | 497 | 41,04 | 232 | 43,45 | 1473 | 414 |  |
| DPYD ---- tag |  | G/C or C/C | 0.99 (0.82-1.21) | 0.96 | 0.99 | 1.00 | 714 | 58,96 | 302 | 56,55 | 1473 | 414 |  |
| DPYD ---- tag | rs17471640 | T/T | 1.00 (.-.) | . | . | . | 559 | 46,16 | 252 | 47,19 | 1473 | 414 |  |
| DPYD ---- tag |  | T/C or C/C | 0.92 (0.76-1.12) | 0.39 | 0.91 | 0.76 | 652 | 53,84 | 282 | 52,81 | 1473 | 414 |  |
| DPYD ---- tag | rs17702702 | G/G | 1.00 (.-.) | . | . | . | 839 | 69,28 | 371 | 69,48 | 1473 | 414 |  |
| DPYD ---- tag |  | G/C or C/C | 1.00 (0.81-1.23) | 0.99 | 1.00 | 1.00 | 372 | 30,72 | 163 | 30,52 | 1473 | 414 |  |
| DPYD ---- NA | rs1801265 | T/T | 1.00 (.-.) | . | . | . | 703 | 58,05 | 311 | 58,24 | 1473 | 414 |  |
| DPYD ---- NA |  | T/C or C/C | 1.16 (0.95-1.42) | 0.14 | 0.84 | 0.47 | 508 | 41,95 | 223 | 41,76 | 1473 | 414 |  |
| DPYD ---- tag | rs2039447 | T/T | 1.00 (.-.) | . | . | . | 558 | 46,08 | 251 | 47,00 | 1473 | 414 |  |
| DPYD ---- tag |  | T/C or C/C | 1.16 (0.95-1.41) | 0.15 | 0.84 | 0.47 | 653 | 53,92 | 283 | 53,00 | 1473 | 414 |  |
| DPYD ---- tag | rs2151567 | G/G | 1.00 (.-.) | . | . | . | 1096 | 90,50 | 476 | 89,14 | 1473 | 414 |  |
| DPYD ---- tag |  | G/A or A/A | 1.36 (1.00-1.86) | 0.05 | 0.84 | 0.36 | 115 | 9,50 | 58 | 10,86 | 1473 | 414 |  |
| DPYD ---- tag | rs2152878 | A/A | 1.00 (.-.) | . | . | . | 705 | 58,22 | 311 | 58,24 | 1473 | 414 |  |
| DPYD ---- tag |  | A/G or G/G | 1.06 (0.87-1.29) | 0.57 | 0.93 | 0.84 | 506 | 41,78 | 223 | 41,76 | 1473 | 414 |  |
| DPYD ---- tag | rs2786505 | G/G | 1.00 (.-.) | . | . | . | 906 | 74,81 | 396 | 74,16 | 1473 | 414 |  |
| DPYD ---- tag |  | G/T or T/T | 1.25 (1.00-1.55) | 0.05 | 0.84 | 0.36 | 305 | 25,19 | 138 | 25,84 | 1473 | 414 |  |
| DPYD ---- tag | rs2786512 | G/G | 1.00 (.-.) | . | . | . | 447 | 36,91 | 201 | 37,64 | 1473 | 414 |  |
| DPYD ---- tag |  | G/A or A/A | 1.00 (0.81-1.22) | 0.97 | 0.99 | 1.00 | 764 | 63,09 | 333 | 62,36 | 1473 | 414 |  |
| DPYD ---- tag | rs2786519 | A/A | 1.00 (.-.) | . | . | . | 742 | 61,27 | 310 | 58,05 | 1473 | 414 |  |
| DPYD ---- tag |  | A/G or G/G | 1.12 (0.91-1.36) | 0.28 | 0.89 | 0.70 | 469 | 38,73 | 224 | 41,95 | 1473 | 414 |  |
| DPYD ---- tag | rs2811170 | A/A | 1.00 (.-.) | . | . | . | 903 | 74,57 | 416 | 77,90 | 1473 | 414 |  |
| DPYD ---- tag |  | A/T or T/T | 0.82 (0.65-1.04) | 0.10 | 0.84 | 0.47 | 308 | 25,43 | 118 | 22,10 | 1473 | 414 |  |
| DPYD ---- tag | rs2811199 | G/G | 1.00 (.-.) | . | . | . | 869 | 71,76 | 381 | 71,35 | 1473 | 414 |  |
| DPYD ---- tag |  | G/A or A/A | 1.25 (1.01-1.54) | 0.04 | 0.84 | 0.36 | 342 | 28,24 | 153 | 28,65 | 1473 | 414 |  |
| DPYD ---- tag | rs2811219 | T/T | 1.00 (.-.) | . | . | . | 695 | 57,39 | 313 | 58,61 | 1473 | 414 |  |
| DPYD ---- tag |  | T/C or C/C | 0.96 (0.78-1.17) | 0.65 | 0.93 | 0.84 | 516 | 42,61 | 221 | 41,39 | 1473 | 414 |  |
| DPYD ---- tag | rs4300257 | A/A | 1.00 (.-.) | . | . | . | 787 | 64,99 | 325 | 60,86 | 1473 | 414 |  |
| DPYD ---- tag |  | A/C or C/C | 1.07 (0.87-1.30) | 0.53 | 0.93 | 0.84 | 424 | 35,01 | 209 | 39,14 | 1473 | 414 |  |
| DPYD ---- tag | rs4379706 | T/T | 1.00 (.-.) | . | . | . | 689 | 56,90 | 310 | 58,05 | 1473 | 414 |  |
| DPYD ---- tag |  | T/C or C/C | 1.15 (0.95-1.40) | 0.16 | 0.84 | 0.49 | 522 | 43,10 | 224 | 41,95 | 1473 | 414 |  |
| DPYD ---- tag | rs4950021 | T/T | 1.00 (.-.) | . | . | . | 363 | 29,98 | 170 | 31,84 | 1473 | 414 |  |
| DPYD ---- tag |  | T/G or G/G | 0.99 (0.80-1.23) | 0.95 | 0.99 | 1.00 | 848 | 70,02 | 364 | 68,16 | 1473 | 414 |  |
| DPYD ---- tag | rs4950033 | T/T | 1.00 (.-.) | . | . | . | 349 | 28,82 | 157 | 29,40 | 1473 | 414 |  |
| DPYD ---- tag |  | T/C or C/C | 1.03 (0.83-1.28) | 0.77 | 0.94 | 0.92 | 862 | 71,18 | 377 | 70,60 | 1473 | 414 |  |
| DPYD ---- tag | rs495257 | T/T | 1.00 (.-.) | . | . | . | 430 | 35,51 | 177 | 33,15 | 1473 | 414 |  |
| DPYD ---- tag |  | T/C or C/C | 1.14 (0.92-1.40) | 0.23 | 0.87 | 0.64 | 781 | 64,49 | 357 | 66,85 | 1473 | 414 |  |
| DPYD ---- tag | rs552926 | A/A | 1.00 (.-.) | . | . | . | 446 | 36,83 | 179 | 33,52 | 1473 | 414 |  |
| DPYD ---- tag |  | A/G or G/G | 1.11 (0.90-1.37) | 0.33 | 0.89 | 0.73 | 765 | 63,17 | 355 | 66,48 | 1473 | 414 |  |
| DPYD ---- tag | rs628959 | A/A | 1.00 (.-.) | . | . | . | 593 | 48,97 | 270 | 50,56 | 1473 | 414 |  |
| DPYD ---- tag |  | A/G or G/G | 1.08 (0.89-1.31) | 0.43 | 0.93 | 0.78 | 618 | 51,03 | 264 | 49,44 | 1473 | 414 |  |
| DPYD ---- tag | rs6656660 | G/G | 1.00 (.-.) | . | . | . | 915 | 75,56 | 407 | 76,22 | 1473 | 414 |  |
| DPYD ---- tag |  | G/T or T/T | 1.00 (0.79-1.26) | 1.00 | 1.00 | 1.00 | 296 | 24,44 | 127 | 23,78 | 1473 | 414 |  |
| DPYD ---- tag | rs6663670 | A/A | 1.00 (.-.) | . | . | . | 872 | 72,01 | 382 | 71,54 | 1473 | 414 |  |
| DPYD ---- tag |  | A/C or C/C | 1.25 (1.01-1.55) | 0.04 | 0.84 | 0.36 | 339 | 27,99 | 152 | 28,46 | 1473 | 414 |  |
| DPYD ---- tag | rs6683883 | T/T | 1.00 (.-.) | . | . | . | 484 | 39,97 | 183 | 34,27 | 1473 | 414 |  |
| DPYD ---- tag |  | T/C or C/C | 1.16 (0.95-1.42) | 0.15 | 0.84 | 0.47 | 727 | 60,03 | 351 | 65,73 | 1473 | 414 |  |
| DPYD ---- tag | rs6686861 | C/C | 1.00 (.-.) | . | . | . | 1051 | 86,79 | 454 | 85,02 | 1473 | 414 |  |
| DPYD ---- tag |  | C/T or T/T | 1.09 (0.83-1.42) | 0.55 | 0.93 | 0.84 | 160 | 13,21 | 80 | 14,98 | 1473 | 414 |  |
| DPYD ---- tag | rs7414210 | A/A | 1.00 (.-.) | . | . | . | 855 | 70,60 | 392 | 73,41 | 1473 | 414 |  |
| DPYD ---- tag |  | A/C or C/C | 0.97 (0.78-1.21) | 0.78 | 0.95 | 0.92 | 356 | 29,40 | 142 | 26,59 | 1473 | 414 |  |
| DPYD ---- tag | rs7530858 | A/A | 1.00 (.-.) | . | . | . | 950 | 78,45 | 412 | 77,15 | 1473 | 414 |  |
| DPYD ---- tag |  | A/G or G/G | 1.15 (0.91-1.45) | 0.23 | 0.87 | 0.64 | 261 | 21,55 | 122 | 22,85 | 1473 | 414 |  |
| DPYD ---- tag | rs7544128 | C/C | 1.00 (.-.) | . | . | . | 663 | 54,75 | 275 | 51,50 | 1473 | 414 |  |
| DPYD ---- tag |  | C/G or G/G | 1.09 (0.90-1.33) | 0.38 | 0.91 | 0.76 | 548 | 45,25 | 259 | 48,50 | 1473 | 414 |  |
| DPYD ---- tag | rs7545340 | G/G | 1.00 (.-.) | . | . | . | 666 | 55,00 | 282 | 52,81 | 1473 | 414 |  |
| DPYD ---- tag |  | G/A or A/A | 1.05 (0.86-1.28) | 0.63 | 0.93 | 0.84 | 545 | 45,00 | 252 | 47,19 | 1473 | 414 |  |
| DPYD ---- tag | rs828054 | A/A | 1.00 (.-.) | . | . | . | 273 | 22,54 | 152 | 28,46 | 1473 | 414 |  |
| DPYD ---- tag |  | A/C or C/C | 0.89 (0.72-1.12) | 0.32 | 0.89 | 0.73 | 938 | 77,46 | 382 | 71,54 | 1473 | 414 |  |
| DPYD ---- tag | rs885622 | G/G | 1.00 (.-.) | . | . | . | 505 | 41,70 | 190 | 35,58 | 1473 | 414 |  |
| DPYD ---- tag |  | G/A or A/A | 1.22 (1.00-1.50) | 0.05 | 0.84 | 0.36 | 706 | 58,30 | 344 | 64,42 | 1473 | 414 |  |
| DPYD ---- tag | rs9437663 | G/G | 1.00 (.-.) | . | . | . | 758 | 62,59 | 342 | 64,04 | 1473 | 414 |  |
| DPYD ---- tag |  | G/A or A/A | 1.09 (0.89-1.34) | 0.40 | 0.91 | 0.76 | 453 | 37,41 | 192 | 35,96 | 1473 | 414 |  |
| DPYS ---- tag | rs13249169 | A/A | 1.00 (.-.) | . | . | . | 960 | 79,27 | 419 | 78,46 | 1473 | 414 |  |
| DPYS ---- tag |  | A/T or T/T | 1.04 (0.82-1.32) | 0.75 | 0.94 | 0.87 | 251 | 20,73 | 115 | 21,54 | 1473 | 414 |  |
| DPYS ---- NA | rs13263121 | T/T | 1.00 (.-.) | . | . | . | 506 | 41,78 | 237 | 44,38 | 1473 | 414 |  |
| DPYS ---- NA |  | T/A or A/A | 0.96 (0.79-1.17) | 0.72 | 0.93 | 0.87 | 705 | 58,22 | 297 | 55,62 | 1473 | 414 |  |
| DPYS ---- tag | rs16871361 | T/T | 1.00 (.-.) | . | . | . | 1083 | 89,43 | 480 | 89,89 | 1473 | 414 |  |
| DPYS ---- tag |  | T/C or C/C | 0.90 (0.65-1.24) | 0.51 | 0.93 | 0.87 | 128 | 10,57 | 54 | 10,11 | 1473 | 414 |  |
| DPYS ---- NA | rs17245950 | T/T | 1.00 (.-.) | . | . | . | 935 | 77,21 | 415 | 77,72 | 1473 | 414 |  |
| DPYS ---- NA |  | T/A or A/A | 0.95 (0.75-1.20) | 0.66 | 0.93 | 0.87 | 276 | 22,79 | 119 | 22,28 | 1473 | 414 |  |
| DPYS ---- NA | rs2253336 | A/A | 1.00 (.-.) | . | . | . | 967 | 79,85 | 436 | 81,65 | 1473 | 414 |  |
| DPYS ---- NA |  | A/G or G/G | 0.85 (0.66-1.10) | 0.21 | 0.84 | 0.87 | 244 | 20,15 | 98 | 18,35 | 1473 | 414 |  |
| DPYS ---- tag | rs2280010 | C/C | 1.00 (.-.) | . | . | . | 698 | 57,64 | 290 | 54,31 | 1473 | 414 |  |
| DPYS ---- tag |  | C/T or T/T | 1.16 (0.95-1.42) | 0.13 | 0.84 | 0.87 | 513 | 42,36 | 244 | 45,69 | 1473 | 414 |  |
| DPYS ---- tag | rs2333874 | T/T | 1.00 (.-.) | . | . | . | 565 | 46,66 | 232 | 43,45 | 1473 | 414 |  |
| DPYS ---- tag |  | T/G or G/G | 1.01 (0.83-1.23) | 0.92 | 0.98 | 0.92 | 646 | 53,34 | 302 | 56,55 | 1473 | 414 |  |
| DPYS ---- NA | rs2669429 | C/C | 1.00 (.-.) | . | . | . | 374 | 30,88 | 161 | 30,15 | 1473 | 414 |  |
| DPYS ---- NA |  | C/T or T/T | 1.13 (0.91-1.41) | 0.27 | 0.89 | 0.87 | 837 | 69,12 | 373 | 69,85 | 1473 | 414 |  |
| DPYS ---- tag | rs2669434 | C/C | 1.00 (.-.) | . | . | . | 625 | 51,61 | 288 | 53,93 | 1473 | 414 |  |
| DPYS ---- tag |  | C/A or A/A | 0.91 (0.74-1.11) | 0.34 | 0.90 | 0.87 | 586 | 48,39 | 246 | 46,07 | 1473 | 414 |  |
| DPYS ---- tag | rs2853142 | T/T | 1.00 (.-.) | . | . | . | 453 | 37,41 | 213 | 39,89 | 1473 | 414 |  |
| DPYS ---- tag |  | T/C or C/C | 0.90 (0.74-1.10) | 0.30 | 0.89 | 0.87 | 758 | 62,59 | 321 | 60,11 | 1473 | 414 |  |
| DPYS ---- NA | rs2853145 | A/A | 1.00 (.-.) | . | . | . | 773 | 63,83 | 353 | 66,10 | 1473 | 414 |  |
| DPYS ---- NA |  | A/C or C/C | 0.95 (0.77-1.17) | 0.63 | 0.93 | 0.87 | 438 | 36,17 | 181 | 33,90 | 1473 | 414 |  |
| DPYS ---- tag | rs2853149 | G/G | 1.00 (.-.) | . | . | . | 343 | 28,32 | 149 | 27,90 | 1473 | 414 |  |
| DPYS ---- tag |  | G/A or A/A | 0.92 (0.74-1.14) | 0.43 | 0.93 | 0.87 | 868 | 71,68 | 385 | 72,10 | 1473 | 414 |  |
| DPYS ---- tag | rs2853154 | T/T | 1.00 (.-.) | . | . | . | 655 | 54,09 | 307 | 57,49 | 1473 | 414 |  |
| DPYS ---- tag |  | T/C or C/C | 0.96 (0.79-1.16) | 0.65 | 0.93 | 0.87 | 556 | 45,91 | 227 | 42,51 | 1473 | 414 |  |
| DPYS ---- tag | rs2853161 | A/A | 1.00 (.-.) | . | . | . | 310 | 25,60 | 130 | 24,34 | 1473 | 414 |  |
| DPYS ---- tag |  | A/G or G/G | 1.01 (0.80-1.28) | 0.92 | 0.98 | 0.92 | 901 | 74,40 | 404 | 75,66 | 1473 | 414 |  |
| DPYS ---- NA | rs2959024 | T/T | 1.00 (.-.) | . | . | . | 636 | 52,52 | 254 | 47,57 | 1473 | 414 |  |
| DPYS ---- NA |  | T/G or G/G | 1.14 (0.93-1.38) | 0.21 | 0.84 | 0.87 | 575 | 47,48 | 280 | 52,43 | 1473 | 414 |  |
| DPYS ---- NA | rs2959025 | A/A | 1.00 (.-.) | . | . | . | 515 | 42,53 | 199 | 37,27 | 1473 | 414 |  |
| DPYS ---- NA |  | A/G or G/G | 1.10 (0.90-1.34) | 0.36 | 0.90 | 0.87 | 696 | 57,47 | 335 | 62,73 | 1473 | 414 |  |
| DPYS ---- tag | rs2959026 | G/G | 1.00 (.-.) | . | . | . | 457 | 37,74 | 184 | 34,46 | 1473 | 414 |  |
| DPYS ---- tag |  | G/A or A/A | 1.11 (0.91-1.36) | 0.31 | 0.89 | 0.87 | 754 | 62,26 | 350 | 65,54 | 1473 | 414 |  |
| DPYS ---- NA | rs3133278 | T/T | 1.00 (.-.) | . | . | . | 592 | 48,89 | 271 | 50,75 | 1473 | 414 |  |
| DPYS ---- NA |  | T/C or C/C | 0.96 (0.79-1.17) | 0.70 | 0.93 | 0.87 | 619 | 51,11 | 263 | 49,25 | 1473 | 414 |  |
| DPYS ---- tag | rs3750187 | G/G | 1.00 (.-.) | . | . | . | 738 | 60,94 | 339 | 63,48 | 1473 | 414 |  |
| DPYS ---- tag |  | G/A or A/A | 0.96 (0.79-1.18) | 0.71 | 0.93 | 0.87 | 473 | 39,06 | 195 | 36,52 | 1473 | 414 |  |
| DPYS ---- tag | rs3793357 | T/T | 1.00 (.-.) | . | . | . | 1077 | 88,93 | 476 | 89,14 | 1473 | 414 |  |
| DPYS ---- tag |  | T/G or G/G | 1.06 (0.77-1.46) | 0.71 | 0.93 | 0.87 | 134 | 11,07 | 58 | 10,86 | 1473 | 414 |  |
| DPYS ---- tag | rs3793358 | G/G | 1.00 (.-.) | . | . | . | 896 | 73,99 | 414 | 77,53 | 1473 | 414 |  |
| DPYS ---- tag |  | G/A or A/A | 0.89 (0.70-1.12) | 0.31 | 0.89 | 0.87 | 315 | 26,01 | 120 | 22,47 | 1473 | 414 |  |
| DPYS ---- tag | rs6468924 | C/C | 1.00 (.-.) | . | . | . | 739 | 61,02 | 331 | 61,99 | 1473 | 414 |  |
| DPYS ---- tag |  | C/T or T/T | 0.97 (0.79-1.20) | 0.80 | 0.95 | 0.88 | 472 | 38,98 | 203 | 38,01 | 1473 | 414 |  |
| DUT ---- tag | rs8025164 | G/G | 1.00 (.-.) | . | . | . | 867 | 71,59 | 384 | 71,91 | 1473 | 414 |  |
| DUT ---- tag |  | G/A or A/A | 0.92 (0.73-1.14) | 0.43 | 0.93 | 0.43 | 344 | 28,41 | 150 | 28,09 | 1473 | 414 |  |
| EHMT1 ---- tag | rs10780190 | C/C | 1.00 (.-.) | . | . | . | 1076 | 88,85 | 480 | 89,89 | 1473 | 414 |  |
| EHMT1 ---- tag |  | C/T or T/T | 0.96 (0.68-1.36) | 0.83 | 0.96 | 0.88 | 135 | 11,15 | 54 | 10,11 | 1473 | 414 |  |
| EHMT1 ---- tag | rs10867083 | G/G | 1.00 (.-.) | . | . | . | 537 | 44,34 | 251 | 47,00 | 1473 | 414 |  |
| EHMT1 ---- tag |  | G/A or A/A | 0.98 (0.80-1.19) | 0.80 | 0.95 | 0.88 | 674 | 55,66 | 283 | 53,00 | 1473 | 414 |  |
| EHMT1 ---- tag | rs11137190 | C/C | 1.00 (.-.) | . | . | . | 619 | 51,11 | 269 | 50,37 | 1473 | 414 |  |
| EHMT1 ---- tag |  | C/G or G/G | 1.10 (0.90-1.33) | 0.35 | 0.90 | 0.77 | 592 | 48,89 | 265 | 49,63 | 1473 | 414 |  |
| EHMT1 ---- tag | rs3123510 | G/G | 1.00 (.-.) | . | . | . | 418 | 34,52 | 190 | 35,58 | 1473 | 414 |  |
| EHMT1 ---- tag |  | G/A or A/A | 1.02 (0.83-1.24) | 0.88 | 0.97 | 0.88 | 793 | 65,48 | 344 | 64,42 | 1473 | 414 |  |
| EHMT1 ---- candidate literature | rs3125795 | G/G | 1.00 (.-.) | . | . | . | 1071 | 88,44 | 480 | 89,89 | 1473 | 414 |  |
| EHMT1 ---- candidate literature |  | G/T or T/T | 0.95 (0.67-1.34) | 0.77 | 0.94 | 0.88 | 140 | 11,56 | 54 | 10,11 | 1473 | 414 |  |
| EHMT1 ---- tag | rs4573359 | G/G | 1.00 (.-.) | . | . | . | 1001 | 82,66 | 445 | 83,33 | 1473 | 414 |  |
| EHMT1 ---- tag |  | G/T or T/T | 0.84 (0.64-1.10) | 0.20 | 0.84 | 0.66 | 210 | 17,34 | 89 | 16,67 | 1473 | 414 |  |
| EHMT1 ---- candidate literature | rs4634736 | G/G | 1.00 (.-.) | . | . | . | 1000 | 82,58 | 446 | 83,52 | 1473 | 414 |  |
| EHMT1 ---- candidate literature |  | G/A or A/A | 0.83 (0.63-1.08) | 0.17 | 0.84 | 0.66 | 211 | 17,42 | 88 | 16,48 | 1473 | 414 |  |
| EHMT1 ---- tag | rs4876902 | C/C | 1.00 (.-.) | . | . | . | 749 | 61,85 | 321 | 60,11 | 1473 | 414 |  |
| EHMT1 ---- tag |  | C/T or T/T | 1.19 (0.98-1.46) | 0.08 | 0.84 | 0.66 | 462 | 38,15 | 213 | 39,89 | 1473 | 414 |  |
| EHMT1 ---- tag | rs4876904 | T/T | 1.00 (.-.) | . | . | . | 359 | 29,64 | 164 | 30,71 | 1473 | 414 |  |
| EHMT1 ---- tag |  | T/G or G/G | 0.95 (0.77-1.17) | 0.62 | 0.93 | 0.88 | 852 | 70,36 | 370 | 69,29 | 1473 | 414 |  |
| EHMT1 ---- tag | rs7390244 | G/G | 1.00 (.-.) | . | . | . | 298 | 24,61 | 137 | 25,66 | 1473 | 414 |  |
| EHMT1 ---- tag |  | G/A or A/A | 1.07 (0.86-1.34) | 0.55 | 0.93 | 0.88 | 913 | 75,39 | 397 | 74,34 | 1473 | 414 |  |
| EHMT1 ---- tag | rs9314635 | G/G | 1.00 (.-.) | . | . | . | 487 | 40,21 | 203 | 38,01 | 1473 | 414 |  |
| EHMT1 ---- tag |  | G/T or T/T | 1.13 (0.92-1.38) | 0.24 | 0.87 | 0.66 | 724 | 59,79 | 331 | 61,99 | 1473 | 414 |  |
| EHMT2 ---- candidate/tag | rs2736428 | G/G | 1.00 (.-.) | . | . | . | 478 | 39,47 | 238 | 44,57 | 1473 | 414 |  |
| EHMT2 ---- candidate/tag |  | G/A or A/A | 0.80 (0.66-0.98) | 0.03 | 0.84 | 0.06 | 733 | 60,53 | 296 | 55,43 | 1473 | 414 |  |
| EHMT2 ---- tag | rs9267649 | G/G | 1.00 (.-.) | . | . | . | 862 | 71,18 | 381 | 71,35 | 1473 | 414 |  |
| EHMT2 ---- tag |  | G/A or A/A | 0.97 (0.78-1.21) | 0.80 | 0.95 | 0.80 | 349 | 28,82 | 153 | 28,65 | 1473 | 414 |  |
| FDXR ---- NA | rs2070918 | T/T | 1.00 (.-.) | . | . | . | 548 | 45,25 | 259 | 48,50 | 1473 | 414 |  |
| FDXR ---- NA |  | T/C or C/C | 1.20 (0.99-1.46) | 0.07 | 0.84 | 0.19 | 663 | 54,75 | 275 | 51,50 | 1473 | 414 |  |
| FDXR ---- tag | rs509911 | A/A | 1.00 (.-.) | . | . | . | 732 | 60,45 | 342 | 64,04 | 1473 | 414 |  |
| FDXR ---- tag |  | A/G or G/G | 1.19 (0.97-1.46) | 0.09 | 0.84 | 0.19 | 479 | 39,55 | 192 | 35,96 | 1473 | 414 |  |
| FDXR ---- NA | rs689882 | G/G | 1.00 (.-.) | . | . | . | 608 | 50,21 | 294 | 55,06 | 1473 | 414 |  |
| FDXR ---- NA |  | G/A or A/A | 0.99 (0.81-1.21) | 0.93 | 0.98 | 0.94 | 603 | 49,79 | 240 | 44,94 | 1473 | 414 |  |
| FDXR ---- NA | rs689895 | G/G | 1.00 (.-.) | . | . | . | 592 | 48,89 | 273 | 51,12 | 1473 | 414 |  |
| FDXR ---- NA |  | G/C or C/C | 1.01 (0.83-1.22) | 0.94 | 0.99 | 0.94 | 619 | 51,11 | 261 | 48,88 | 1473 | 414 |  |
| FOLH1 ---- candidate literature | rs10839236 | T/T | 1.00 (.-.) | . | . | . | 473 | 39,06 | 211 | 39,51 | 1473 | 414 |  |
| FOLH1 ---- candidate literature |  | T/C or C/C | 0.95 (0.77-1.16) | 0.59 | 0.93 | 0.75 | 738 | 60,94 | 323 | 60,49 | 1473 | 414 |  |
| FOLH1 ---- tag | rs16906190 | A/A | 1.00 (.-.) | . | . | . | 1002 | 82,74 | 453 | 84,83 | 1473 | 414 |  |
| FOLH1 ---- tag |  | A/G or G/G | 0.85 (0.65-1.13) | 0.27 | 0.89 | 0.68 | 209 | 17,26 | 81 | 15,17 | 1473 | 414 |  |
| FOLH1 ---- candidate | rs202676 | T/T | 1.00 (.-.) | . | . | . | 751 | 62,01 | 335 | 62,73 | 1473 | 414 |  |
| FOLH1 ---- candidate |  | T/C or C/C | 0.87 (0.71-1.07) | 0.19 | 0.84 | 0.65 | 460 | 37,99 | 199 | 37,27 | 1473 | 414 |  |
| FOLH1 ---- tag | rs202680 | A/A | 1.00 (.-.) | . | . | . | 666 | 55,00 | 298 | 55,81 | 1473 | 414 |  |
| FOLH1 ---- tag |  | A/T or T/T | 0.92 (0.76-1.12) | 0.43 | 0.93 | 0.75 | 545 | 45,00 | 236 | 44,19 | 1473 | 414 |  |
| FOLH1 ---- candidate literature | rs202720 | G/G | 1.00 (.-.) | . | . | . | 749 | 61,85 | 335 | 62,73 | 1473 | 414 |  |
| FOLH1 ---- candidate literature |  | G/C or C/C | 0.87 (0.71-1.07) | 0.18 | 0.84 | 0.65 | 462 | 38,15 | 199 | 37,27 | 1473 | 414 |  |
| FOLH1 ---- tag | rs2299650 | G/G | 1.00 (.-.) | . | . | . | 467 | 38,56 | 210 | 39,33 | 1473 | 414 |  |
| FOLH1 ---- tag |  | G/T or T/T | 0.93 (0.76-1.14) | 0.51 | 0.93 | 0.75 | 744 | 61,44 | 324 | 60,67 | 1473 | 414 |  |
| FOLH1 ---- tag | rs617528 | G/G | 1.00 (.-.) | . | . | . | 938 | 77,46 | 424 | 79,40 | 1473 | 414 |  |
| FOLH1 ---- tag |  | G/A or A/A | 0.96 (0.76-1.21) | 0.71 | 0.93 | 0.75 | 273 | 22,54 | 110 | 20,60 | 1473 | 414 |  |
| FOLH1 ---- tag | rs663877 | T/T | 1.00 (.-.) | . | . | . | 934 | 77,13 | 406 | 76,03 | 1473 | 414 |  |
| FOLH1 ---- tag |  | T/G or G/G | 0.96 (0.77-1.21) | 0.75 | 0.94 | 0.75 | 277 | 22,87 | 128 | 23,97 | 1473 | 414 |  |
| FOLH1 ---- tag | rs670776 | A/A | 1.00 (.-.) | . | . | . | 751 | 62,01 | 335 | 62,73 | 1473 | 414 |  |
| FOLH1 ---- tag |  | A/T or T/T | 0.87 (0.71-1.07) | 0.19 | 0.84 | 0.65 | 460 | 37,99 | 199 | 37,27 | 1473 | 414 |  |
| FOLH1 ---- tag | rs7124497 | G/G | 1.00 (.-.) | . | . | . | 1112 | 91,82 | 489 | 91,57 | 1473 | 414 |  |
| FOLH1 ---- tag |  | G/A or A/A | 1.08 (0.77-1.53) | 0.64 | 0.93 | 0.75 | 99 | 8,18 | 45 | 8,43 | 1473 | 414 |  |
| FOLR1 ---- tag | rs651646 | T/T | 1.00 (.-.) | . | . | . | 362 | 29,89 | 180 | 33,71 | 1473 | 414 |  |
| FOLR1 ---- tag |  | T/A or A/A | 0.85 (0.69-1.04) | 0.11 | 0.84 | 0.11 | 849 | 70,11 | 354 | 66,29 | 1473 | 414 |  |
| FPGS ---- tag | rs10987746 | T/T | 1.00 (.-.) | . | . | . | 352 | 29,07 | 144 | 26,97 | 1473 | 414 |  |
| FPGS ---- tag |  | T/C or C/C | 0.93 (0.75-1.16) | 0.54 | 0.93 | 0.66 | 859 | 70,93 | 390 | 73,03 | 1473 | 414 |  |
| FPGS ---- tag | rs7033913 | T/T | 1.00 (.-.) | . | . | . | 380 | 31,38 | 172 | 32,21 | 1473 | 414 |  |
| FPGS ---- tag |  | T/C or C/C | 0.93 (0.76-1.14) | 0.49 | 0.93 | 0.66 | 831 | 68,62 | 362 | 67,79 | 1473 | 414 |  |
| FPGS ---- tag | rs7039798 | G/G | 1.00 (.-.) | . | . | . | 396 | 32,70 | 160 | 29,96 | 1473 | 414 |  |
| FPGS ---- tag |  | G/A or A/A | 0.95 (0.77-1.18) | 0.66 | 0.93 | 0.66 | 815 | 67,30 | 374 | 70,04 | 1473 | 414 |  |
| GGH ---- tag | rs10957264 | G/G | 1.00 (.-.) | . | . | . | 847 | 69,94 | 386 | 72,28 | 1473 | 414 |  |
| GGH ---- tag |  | G/T or T/T | 0.93 (0.75-1.16) | 0.54 | 0.93 | 0.84 | 364 | 30,06 | 148 | 27,72 | 1473 | 414 |  |
| GGH ---- candidate literature | rs11545076 | T/T | 1.00 (.-.) | . | . | . | 603 | 49,79 | 264 | 49,44 | 1473 | 414 |  |
| GGH ---- candidate literature |  | T/G or G/G | 0.93 (0.77-1.13) | 0.48 | 0.93 | 0.84 | 608 | 50,21 | 270 | 50,56 | 1473 | 414 |  |
| GGH ---- candidate | rs11545077 | G/G | 1.00 (.-.) | . | . | . | 652 | 53,84 | 289 | 54,12 | 1473 | 414 |  |
| GGH ---- candidate |  | G/A or A/A | 0.98 (0.80-1.19) | 0.81 | 0.96 | 0.94 | 559 | 46,16 | 245 | 45,88 | 1473 | 414 |  |
| GGH ---- candidate | rs11545078 | C/C | 1.00 (.-.) | . | . | . | 1002 | 82,74 | 436 | 81,65 | 1473 | 414 |  |
| GGH ---- candidate |  | C/T or T/T | 0.99 (0.77-1.28) | 0.94 | 0.99 | 0.94 | 209 | 17,26 | 98 | 18,35 | 1473 | 414 |  |
| GGH ---- tag | rs11995525 | G/G | 1.00 (.-.) | . | . | . | 653 | 53,92 | 278 | 52,06 | 1473 | 414 |  |
| GGH ---- tag |  | G/A or A/A | 1.09 (0.89-1.32) | 0.40 | 0.91 | 0.84 | 558 | 46,08 | 256 | 47,94 | 1473 | 414 |  |
| GGH ---- tag | rs16930073 | G/G | 1.00 (.-.) | . | . | . | 965 | 79,69 | 430 | 80,52 | 1473 | 414 |  |
| GGH ---- tag |  | G/A or A/A | 0.97 (0.76-1.24) | 0.83 | 0.96 | 0.94 | 246 | 20,31 | 104 | 19,48 | 1473 | 414 |  |
| GGH ---- tag | rs17194931 | G/G | 1.00 (.-.) | . | . | . | 1002 | 82,74 | 436 | 81,65 | 1473 | 414 |  |
| GGH ---- tag |  | G/A or A/A | 0.99 (0.77-1.28) | 0.94 | 0.99 | 0.94 | 209 | 17,26 | 98 | 18,35 | 1473 | 414 |  |
| GGH ---- candidate literature | rs1800909 | T/T | 1.00 (.-.) | . | . | . | 598 | 49,38 | 264 | 49,44 | 1473 | 414 |  |
| GGH ---- candidate literature |  | T/C or C/C | 0.91 (0.75-1.11) | 0.35 | 0.90 | 0.84 | 613 | 50,62 | 270 | 50,56 | 1473 | 414 |  |
| GGH ---- candidate literature | rs3758149 | C/C | 1.00 (.-.) | . | . | . | 603 | 49,79 | 264 | 49,44 | 1473 | 414 |  |
| GGH ---- candidate literature |  | C/T or T/T | 0.93 (0.77-1.13) | 0.48 | 0.93 | 0.84 | 608 | 50,21 | 270 | 50,56 | 1473 | 414 |  |
| GGH ---- tag | rs3780130 | A/A | 1.00 (.-.) | . | . | . | 751 | 62,01 | 348 | 65,17 | 1473 | 414 |  |
| GGH ---- tag |  | A/T or T/T | 0.87 (0.71-1.07) | 0.18 | 0.84 | 0.84 | 460 | 37,99 | 186 | 34,83 | 1473 | 414 |  |
| GGH ---- tag | rs4446729 | C/C | 1.00 (.-.) | . | . | . | 648 | 53,51 | 271 | 50,75 | 1473 | 414 |  |
| GGH ---- tag |  | C/T or T/T | 1.06 (0.87-1.29) | 0.58 | 0.93 | 0.84 | 563 | 46,49 | 263 | 49,25 | 1473 | 414 |  |
| GGH ---- tag | rs6472067 | C/C | 1.00 (.-.) | . | . | . | 482 | 39,80 | 213 | 39,89 | 1473 | 414 |  |
| GGH ---- tag |  | C/G or G/G | 1.09 (0.90-1.34) | 0.38 | 0.91 | 0.84 | 729 | 60,20 | 321 | 60,11 | 1473 | 414 |  |
| GGH ---- tag | rs7010484 | T/T | 1.00 (.-.) | . | . | . | 552 | 45,58 | 234 | 43,82 | 1473 | 414 |  |
| GGH ---- tag |  | T/C or C/C | 1.08 (0.89-1.31) | 0.44 | 0.93 | 0.84 | 659 | 54,42 | 300 | 56,18 | 1473 | 414 |  |
| GNMT ---- tag | rs1053538 | C/C | 1.00 (.-.) | . | . | . | 336 | 27,75 | 146 | 27,34 | 1473 | 414 |  |
| GNMT ---- tag |  | C/G or G/G | 1.02 (0.82-1.26) | 0.87 | 0.97 | 0.87 | 875 | 72,25 | 388 | 72,66 | 1473 | 414 |  |
| GNMT ---- tag | rs2296805 | G/G | 1.00 (.-.) | . | . | . | 366 | 30,22 | 180 | 33,71 | 1473 | 414 |  |
| GNMT ---- tag |  | G/T or T/T | 0.87 (0.71-1.08) | 0.21 | 0.84 | 0.81 | 845 | 69,78 | 354 | 66,29 | 1473 | 414 |  |
| GNMT ---- tag | rs6901782 | T/T | 1.00 (.-.) | . | . | . | 919 | 75,89 | 414 | 77,53 | 1473 | 414 |  |
| GNMT ---- tag |  | T/C or C/C | 1.10 (0.88-1.39) | 0.41 | 0.91 | 0.81 | 292 | 24,11 | 120 | 22,47 | 1473 | 414 |  |
| GNMT ---- tag | rs6927188 | A/A | 1.00 (.-.) | . | . | . | 706 | 58,30 | 299 | 55,99 | 1473 | 414 |  |
| GNMT ---- tag |  | A/G or G/G | 0.98 (0.80-1.19) | 0.82 | 0.96 | 0.87 | 505 | 41,70 | 235 | 44,01 | 1473 | 414 |  |
| MAT1A ---- tag | rs10887708 | G/G | 1.00 (.-.) | . | . | . | 603 | 49,79 | 273 | 51,12 | 1473 | 414 |  |
| MAT1A ---- tag |  | G/A or A/A | 1.07 (0.88-1.31) | 0.48 | 0.93 | 0.86 | 608 | 50,21 | 261 | 48,88 | 1473 | 414 |  |
| MAT1A ---- tag | rs10887718 | T/T | 1.00 (.-.) | . | . | . | 349 | 28,82 | 141 | 26,40 | 1473 | 414 |  |
| MAT1A ---- tag |  | T/C or C/C | 1.25 (1.00-1.56) | 0.05 | 0.84 | 0.21 | 862 | 71,18 | 393 | 73,60 | 1473 | 414 |  |
| MAT1A ---- tag | rs11202403 | C/C | 1.00 (.-.) | . | . | . | 790 | 65,24 | 344 | 64,42 | 1473 | 414 |  |
| MAT1A ---- tag |  | C/T or T/T | 0.97 (0.79-1.19) | 0.76 | 0.94 | 0.89 | 421 | 34,76 | 190 | 35,58 | 1473 | 414 |  |
| MAT1A ---- tag | rs1832683 | C/C | 1.00 (.-.) | . | . | . | 839 | 69,28 | 369 | 69,10 | 1473 | 414 |  |
| MAT1A ---- tag |  | C/T or T/T | 0.86 (0.69-1.06) | 0.16 | 0.84 | 0.48 | 372 | 30,72 | 165 | 30,90 | 1473 | 414 |  |
| MAT1A ---- tag | rs2236568 | C/C | 1.00 (.-.) | . | . | . | 389 | 32,12 | 172 | 32,21 | 1473 | 414 |  |
| MAT1A ---- tag |  | C/A or A/A | 1.03 (0.83-1.27) | 0.80 | 0.95 | 0.89 | 822 | 67,88 | 362 | 67,79 | 1473 | 414 |  |
| MAT1A ---- tag | rs2236569 | A/A | 1.00 (.-.) | . | . | . | 542 | 44,76 | 224 | 41,95 | 1473 | 414 |  |
| MAT1A ---- tag |  | A/G or G/G | 1.28 (1.05-1.56) | 0.02 | 0.84 | 0.14 | 669 | 55,24 | 310 | 58,05 | 1473 | 414 |  |
| MAT1A ---- tag | rs9421467 | G/G | 1.00 (.-.) | . | . | . | 1090 | 90,01 | 473 | 88,58 | 1473 | 414 |  |
| MAT1A ---- tag |  | G/C or C/C | 1.15 (0.85-1.55) | 0.35 | 0.90 | 0.79 | 121 | 9,99 | 61 | 11,42 | 1473 | 414 |  |
| MAT1A ---- tag | rs998765 | A/A | 1.00 (.-.) | . | . | . | 309 | 25,52 | 141 | 26,40 | 1473 | 414 |  |
| MAT1A ---- tag |  | A/T or T/T | 1.06 (0.85-1.33) | 0.61 | 0.93 | 0.89 | 902 | 74,48 | 393 | 73,60 | 1473 | 414 |  |
| MAT1A ---- tag | rs998766 | C/C | 1.00 (.-.) | . | . | . | 376 | 31,05 | 173 | 32,40 | 1473 | 414 |  |
| MAT1A ---- tag |  | C/G or G/G | 1.01 (0.82-1.25) | 0.89 | 0.97 | 0.89 | 835 | 68,95 | 361 | 67,60 | 1473 | 414 |  |
| MAT2B ---- tag | rs12655857 | G/G | 1.00 (.-.) | . | . | . | 676 | 55,82 | 301 | 56,37 | 1473 | 414 |  |
| MAT2B ---- tag |  | G/T or T/T | 1.03 (0.85-1.26) | 0.76 | 0.94 | 0.76 | 535 | 44,18 | 233 | 43,63 | 1473 | 414 |  |
| MAT2B ---- tag | rs6869277 | C/C | 1.00 (.-.) | . | . | . | 948 | 78,28 | 420 | 78,65 | 1473 | 414 |  |
| MAT2B ---- tag |  | C/T or T/T | 0.94 (0.74-1.20) | 0.63 | 0.93 | 0.76 | 263 | 21,72 | 114 | 21,35 | 1473 | 414 |  |
| MAT2B ---- tag | rs6874065 | A/A | 1.00 (.-.) | . | . | . | 331 | 27,33 | 150 | 28,09 | 1473 | 414 |  |
| MAT2B ---- tag |  | A/G or G/G | 0.94 (0.76-1.16) | 0.57 | 0.93 | 0.76 | 880 | 72,67 | 384 | 71,91 | 1473 | 414 |  |
| MAT2B ---- tag | rs6882306 | T/T | 1.00 (.-.) | . | . | . | 809 | 66,80 | 352 | 65,92 | 1473 | 414 |  |
| MAT2B ---- tag |  | T/C or C/C | 1.35 (1.10-1.66) | 0.00 | 0.84 | 0.02 | 402 | 33,20 | 182 | 34,08 | 1473 | 414 |  |
| MAT2B ---- tag | rs7721639 | T/T | 1.00 (.-.) | . | . | . | 862 | 71,18 | 366 | 68,54 | 1473 | 414 |  |
| MAT2B ---- tag |  | T/G or G/G | 1.29 (1.05-1.59) | 0.02 | 0.84 | 0.05 | 349 | 28,82 | 168 | 31,46 | 1473 | 414 |  |
| MTHFD1 ---- tag | rs1256148 | G/G | 1.00 (.-.) | . | . | . | 715 | 59,04 | 316 | 59,18 | 1473 | 414 |  |
| MTHFD1 ---- tag |  | G/A or A/A | 0.95 (0.78-1.16) | 0.61 | 0.93 | 0.73 | 496 | 40,96 | 218 | 40,82 | 1473 | 414 |  |
| MTHFD1 ---- tag | rs13329053 | T/T | 1.00 (.-.) | . | . | . | 378 | 31,21 | 169 | 31,65 | 1473 | 414 |  |
| MTHFD1 ---- tag |  | T/C or C/C | 1.12 (0.90-1.38) | 0.32 | 0.89 | 0.55 | 833 | 68,79 | 365 | 68,35 | 1473 | 414 |  |
| MTHFD1 ---- candidate literature | rs2236224 | C/C | 1.00 (.-.) | . | . | . | 467 | 38,56 | 211 | 39,51 | 1473 | 414 |  |
| MTHFD1 ---- candidate literature |  | C/T or T/T | 1.14 (0.93-1.40) | 0.19 | 0.84 | 0.55 | 744 | 61,44 | 323 | 60,49 | 1473 | 414 |  |
| MTHFD1 ---- candidate | rs2236225 | C/C | 1.00 (.-.) | . | . | . | 383 | 31,63 | 174 | 32,58 | 1473 | 414 |  |
| MTHFD1 ---- candidate |  | C/T or T/T | 1.10 (0.89-1.36) | 0.37 | 0.90 | 0.55 | 828 | 68,37 | 360 | 67,42 | 1473 | 414 |  |
| MTHFD1 ---- tag | rs2281603 | A/A | 1.00 (.-.) | . | . | . | 712 | 58,79 | 309 | 57,87 | 1473 | 414 |  |
| MTHFD1 ---- tag |  | A/G or G/G | 0.98 (0.81-1.20) | 0.86 | 0.97 | 0.86 | 499 | 41,21 | 225 | 42,13 | 1473 | 414 |  |
| MTHFD1 ---- candidate literature | rs8003379 | A/A | 1.00 (.-.) | . | . | . | 669 | 55,24 | 314 | 58,80 | 1473 | 414 |  |
| MTHFD1 ---- candidate literature |  | A/C or C/C | 1.15 (0.94-1.40) | 0.17 | 0.84 | 0.55 | 542 | 44,76 | 220 | 41,20 | 1473 | 414 |  |
| MTHFD2 ---- tag | rs10177833 | A/A | 1.00 (.-.) | . | . | . | 374 | 30,88 | 154 | 28,84 | 1473 | 414 |  |
| MTHFD2 ---- tag |  | A/C or C/C | 0.94 (0.76-1.16) | 0.58 | 0.93 | 0.81 | 837 | 69,12 | 380 | 71,16 | 1473 | 414 |  |
| MTHFD2 ---- tag | rs702462 | T/T | 1.00 (.-.) | . | . | . | 395 | 32,62 | 177 | 33,15 | 1473 | 414 |  |
| MTHFD2 ---- tag |  | T/A or A/A | 0.91 (0.74-1.12) | 0.38 | 0.91 | 0.81 | 816 | 67,38 | 357 | 66,85 | 1473 | 414 |  |
| MTHFD2 ---- candidate literature | rs702465 | A/A | 1.00 (.-.) | . | . | . | 343 | 28,32 | 146 | 27,34 | 1473 | 414 |  |
| MTHFD2 ---- candidate literature |  | A/T or T/T | 1.09 (0.87-1.35) | 0.46 | 0.93 | 0.81 | 868 | 71,68 | 388 | 72,66 | 1473 | 414 |  |
| MTHFD2 ---- candidate literature | rs7571842 | A/A | 1.00 (.-.) | . | . | . | 352 | 29,07 | 133 | 24,91 | 1473 | 414 |  |
| MTHFD2 ---- candidate literature |  | A/G or G/G | 0.98 (0.79-1.22) | 0.88 | 0.97 | 0.92 | 859 | 70,93 | 401 | 75,09 | 1473 | 414 |  |
| MTHFD2 ---- tag | rs7587117 | T/T | 1.00 (.-.) | . | . | . | 504 | 41,62 | 212 | 39,70 | 1473 | 414 |  |
| MTHFD2 ---- tag |  | T/C or C/C | 0.99 (0.81-1.21) | 0.92 | 0.98 | 0.92 | 707 | 58,38 | 322 | 60,30 | 1473 | 414 |  |
| MTHFD2 ---- tag | rs828861 | C/C | 1.00 (.-.) | . | . | . | 349 | 28,82 | 146 | 27,34 | 1473 | 414 |  |
| MTHFD2 ---- tag |  | C/G or G/G | 1.09 (0.88-1.35) | 0.45 | 0.93 | 0.81 | 862 | 71,18 | 388 | 72,66 | 1473 | 414 |  |
| MTHFD2 ---- tag | rs828863 | G/G | 1.00 (.-.) | . | . | . | 975 | 80,51 | 447 | 83,71 | 1473 | 414 |  |
| MTHFD2 ---- tag |  | G/A or A/A | 0.88 (0.68-1.16) | 0.37 | 0.90 | 0.81 | 236 | 19,49 | 87 | 16,29 | 1473 | 414 |  |
| MTHFR ---- tag | rs1476413 | G/G | 1.00 (.-.) | . | . | . | 631 | 52,11 | 294 | 55,06 | 1473 | 414 |  |
| MTHFR ---- tag |  | G/A or A/A | 0.91 (0.74-1.10) | 0.33 | 0.89 | 0.58 | 580 | 47,89 | 240 | 44,94 | 1473 | 414 |  |
| MTHFR ---- tag | rs17376328 | G/G | 1.00 (.-.) | . | . | . | 1078 | 89,02 | 457 | 85,58 | 1473 | 414 |  |
| MTHFR ---- tag |  | G/A or A/A | 1.12 (0.84-1.49) | 0.45 | 0.93 | 0.58 | 133 | 10,98 | 77 | 14,42 | 1473 | 414 |  |
| MTHFR ---- tag | rs17421462 | G/G | 1.00 (.-.) | . | . | . | 1028 | 84,89 | 456 | 85,39 | 1473 | 414 |  |
| MTHFR ---- tag |  | G/A or A/A | 0.98 (0.74-1.30) | 0.91 | 0.98 | 0.91 | 183 | 15,11 | 78 | 14,61 | 1473 | 414 |  |
| MTHFR ---- candidate | rs1801131 | A/A | 1.00 (.-.) | . | . | . | 548 | 45,25 | 252 | 47,19 | 1473 | 414 |  |
| MTHFR ---- candidate |  | A/C or C/C | 0.94 (0.77-1.14) | 0.51 | 0.93 | 0.58 | 663 | 54,75 | 282 | 52,81 | 1473 | 414 |  |
| MTHFR ---- candidate | rs1801133 | C/C | 1.00 (.-.) | . | . | . | 502 | 41,45 | 221 | 41,39 | 1473 | 414 |  |
| MTHFR ---- candidate |  | C/T or T/T | 0.86 (0.71-1.05) | 0.14 | 0.84 | 0.58 | 709 | 58,55 | 313 | 58,61 | 1473 | 414 |  |
| MTHFR ---- tag | rs2066471 | G/G | 1.00 (.-.) | . | . | . | 835 | 68,95 | 386 | 72,28 | 1473 | 414 |  |
| MTHFR ---- tag |  | G/A or A/A | 0.91 (0.73-1.13) | 0.40 | 0.91 | 0.58 | 376 | 31,05 | 148 | 27,72 | 1473 | 414 |  |
| MTHFR ---- tag | rs4846047 | G/G | 1.00 (.-.) | . | . | . | 598 | 49,38 | 281 | 52,62 | 1473 | 414 |  |
| MTHFR ---- tag |  | G/C or C/C | 0.90 (0.74-1.10) | 0.31 | 0.89 | 0.58 | 613 | 50,62 | 253 | 47,38 | 1473 | 414 |  |
| MTHFR ---- tag | rs4846049 | G/G | 1.00 (.-.) | . | . | . | 538 | 44,43 | 249 | 46,63 | 1473 | 414 |  |
| MTHFR ---- tag |  | G/T or T/T | 0.92 (0.75-1.12) | 0.39 | 0.91 | 0.58 | 673 | 55,57 | 285 | 53,37 | 1473 | 414 |  |
| MTHFR ---- tag | rs7538516 | T/T | 1.00 (.-.) | . | . | . | 438 | 36,17 | 202 | 37,83 | 1473 | 414 |  |
| MTHFR ---- tag |  | T/C or C/C | 0.92 (0.75-1.13) | 0.43 | 0.93 | 0.58 | 773 | 63,83 | 332 | 62,17 | 1473 | 414 |  |
| MTR ---- tag | rs10733117 | A/A | 1.00 (.-.) | . | . | . | 429 | 35,43 | 192 | 35,96 | 1473 | 414 |  |
| MTR ---- tag |  | A/G or G/G | 0.95 (0.78-1.17) | 0.65 | 0.93 | 0.65 | 782 | 64,57 | 342 | 64,04 | 1473 | 414 |  |
| MTR ---- tag | rs12129440 | G/G | 1.00 (.-.) | . | . | . | 647 | 53,43 | 311 | 58,24 | 1473 | 414 |  |
| MTR ---- tag |  | G/A or A/A | 0.88 (0.72-1.07) | 0.20 | 0.84 | 0.52 | 564 | 46,57 | 223 | 41,76 | 1473 | 414 |  |
| MTR ---- candidate | rs1805087 | A/A | 1.00 (.-.) | . | . | . | 810 | 66,89 | 362 | 67,79 | 1473 | 414 |  |
| MTR ---- candidate |  | A/G or G/G | 0.94 (0.76-1.16) | 0.55 | 0.93 | 0.65 | 401 | 33,11 | 172 | 32,21 | 1473 | 414 |  |
| MTR ---- tag | rs3890786 | C/C | 1.00 (.-.) | . | . | . | 410 | 33,86 | 180 | 33,71 | 1473 | 414 |  |
| MTR ---- tag |  | C/T or T/T | 1.14 (0.93-1.41) | 0.21 | 0.84 | 0.52 | 801 | 66,14 | 354 | 66,29 | 1473 | 414 |  |
| MTR ---- tag | rs4659727 | A/A | 1.00 (.-.) | . | . | . | 806 | 66,56 | 361 | 67,60 | 1473 | 414 |  |
| MTR ---- tag |  | A/G or G/G | 0.94 (0.76-1.16) | 0.56 | 0.93 | 0.65 | 405 | 33,44 | 173 | 32,40 | 1473 | 414 |  |
| MTRR ---- candidate literature/tag | rs10380 | C/C | 1.00 (.-.) | . | . | . | 1023 | 84,48 | 442 | 82,77 | 1473 | 414 |  |
| MTRR ---- candidate literature/tag |  | C/T or T/T | 1.21 (0.93-1.56) | 0.16 | 0.84 | 0.62 | 188 | 15,52 | 92 | 17,23 | 1473 | 414 |  |
| MTRR ---- tag | rs10475399 | G/G | 1.00 (.-.) | . | . | . | 528 | 43,60 | 228 | 42,70 | 1473 | 414 |  |
| MTRR ---- tag |  | G/A or A/A | 1.07 (0.88-1.30) | 0.52 | 0.93 | 1.00 | 683 | 56,40 | 306 | 57,30 | 1473 | 414 |  |
| MTRR ---- tag | rs11134265 | C/C | 1.00 (.-.) | . | . | . | 533 | 44,01 | 236 | 44,19 | 1473 | 414 |  |
| MTRR ---- tag |  | C/T or T/T | 1.06 (0.87-1.29) | 0.54 | 0.93 | 1.00 | 678 | 55,99 | 298 | 55,81 | 1473 | 414 |  |
| MTRR ---- tag | rs13181011 | T/T | 1.00 (.-.) | . | . | . | 788 | 65,07 | 336 | 62,92 | 1473 | 414 |  |
| MTRR ---- tag |  | T/C or C/C | 0.96 (0.78-1.17) | 0.66 | 0.93 | 1.00 | 423 | 34,93 | 198 | 37,08 | 1473 | 414 |  |
| MTRR ---- tag | rs161869 | C/C | 1.00 (.-.) | . | . | . | 419 | 34,60 | 189 | 35,39 | 1473 | 414 |  |
| MTRR ---- tag |  | C/T or T/T | 1.06 (0.86-1.29) | 0.60 | 0.93 | 1.00 | 792 | 65,40 | 345 | 64,61 | 1473 | 414 |  |
| MTRR ---- tagged by rs162039 | rs162036 | A/A | 1.00 (.-.) | . | . | . | 978 | 80,76 | 424 | 79,40 | 1473 | 414 |  |
| MTRR ---- tagged by rs162039 |  | A/G or G/G | 1.17 (0.92-1.49) | 0.20 | 0.84 | 0.62 | 233 | 19,24 | 110 | 20,60 | 1473 | 414 |  |
| MTRR ---- tag | rs162039 | C/C | 1.00 (.-.) | . | . | . | 979 | 80,84 | 424 | 79,40 | 1473 | 414 |  |
| MTRR ---- tag |  | C/T or T/T | 1.17 (0.92-1.49) | 0.20 | 0.84 | 0.62 | 232 | 19,16 | 110 | 20,60 | 1473 | 414 |  |
| MTRR ---- tag | rs162270 | G/G | 1.00 (.-.) | . | . | . | 857 | 70,77 | 372 | 69,66 | 1473 | 414 |  |
| MTRR ---- tag |  | G/T or T/T | 1.03 (0.83-1.27) | 0.79 | 0.95 | 1.00 | 354 | 29,23 | 162 | 30,34 | 1473 | 414 |  |
| MTRR ---- candidate | rs16879334 | C/C | 1.00 (.-.) | . | . | . | 1139 | 94,05 | 503 | 94,19 | 1473 | 414 |  |
| MTRR ---- candidate |  | C/G or G/G | 0.99 (0.66-1.48) | 0.97 | 0.99 | 1.00 | 72 | 5,95 | 31 | 5,81 | 1473 | 414 |  |
| MTRR ---- singleton | rs1801394 | G/G | 1.00 (.-.) | . | . | . | 360 | 29,73 | 155 | 29,03 | 1473 | 414 |  |
| MTRR ---- singleton |  | G/A or A/A | 1.06 (0.86-1.32) | 0.59 | 0.93 | 1.00 | 851 | 70,27 | 379 | 70,97 | 1473 | 414 |  |
| MTRR ---- tag | rs1802059 | G/G | 1.00 (.-.) | . | . | . | 459 | 37,90 | 210 | 39,33 | 1473 | 414 |  |
| MTRR ---- tag |  | G/A or A/A | 0.85 (0.70-1.04) | 0.11 | 0.84 | 0.62 | 752 | 62,10 | 324 | 60,67 | 1473 | 414 |  |
| MTRR ---- tag | rs2077744 | T/T | 1.00 (.-.) | . | . | . | 876 | 72,34 | 392 | 73,41 | 1473 | 414 |  |
| MTRR ---- tag |  | T/C or C/C | 1.00 (0.80-1.24) | 1.00 | 1.00 | 1.00 | 335 | 27,66 | 142 | 26,59 | 1473 | 414 |  |
| MTRR ---- candidate | rs2287780 | C/C | 1.00 (.-.) | . | . | . | 1139 | 94,05 | 503 | 94,19 | 1473 | 414 |  |
| MTRR ---- candidate |  | C/T or T/T | 0.99 (0.66-1.48) | 0.97 | 0.99 | 1.00 | 72 | 5,95 | 31 | 5,81 | 1473 | 414 |  |
| MTRR ---- candidate | rs2303080 | T/T | 1.00 (.-.) | . | . | . | 1139 | 94,05 | 504 | 94,38 | 1473 | 414 |  |
| MTRR ---- candidate |  | T/A or A/A | 0.97 (0.64-1.46) | 0.88 | 0.97 | 1.00 | 72 | 5,95 | 30 | 5,62 | 1473 | 414 |  |
| MTRR ---- tag | rs7715062 | G/G | 1.00 (.-.) | . | . | . | 406 | 33,53 | 178 | 33,33 | 1473 | 414 |  |
| MTRR ---- tag |  | G/T or T/T | 0.88 (0.72-1.08) | 0.22 | 0.85 | 0.62 | 805 | 66,47 | 356 | 66,67 | 1473 | 414 |  |
| MTRR ---- tag | rs9282787 | T/T | 1.00 (.-.) | . | . | . | 794 | 65,57 | 339 | 63,48 | 1473 | 414 |  |
| MTRR ---- tag |  | T/C or C/C | 0.99 (0.81-1.21) | 0.89 | 0.97 | 1.00 | 417 | 34,43 | 195 | 36,52 | 1473 | 414 |  |
| MTRR ---- candidate literature | rs9332 | C/C | 1.00 (.-.) | . | . | . | 979 | 80,84 | 424 | 79,40 | 1473 | 414 |  |
| MTRR ---- candidate literature |  | C/T or T/T | 1.17 (0.92-1.49) | 0.20 | 0.84 | 0.62 | 232 | 19,16 | 110 | 20,60 | 1473 | 414 |  |
| NFKB1 ---- NA | rs1609798 | C/C | 1.00 (.-.) | . | . | . | 573 | 47,32 | 245 | 45,88 | 1473 | 414 |  |
| NFKB1 ---- NA |  | C/T or T/T | 1.06 (0.87-1.29) | 0.55 | 0.93 | 1.00 | 638 | 52,68 | 289 | 54,12 | 1473 | 414 |  |
| NFKB1 ---- tag | rs230540 | T/T | 1.00 (.-.) | . | . | . | 514 | 42,44 | 227 | 42,51 | 1473 | 414 |  |
| NFKB1 ---- tag |  | T/C or C/C | 0.97 (0.80-1.19) | 0.79 | 0.95 | 1.00 | 697 | 57,56 | 307 | 57,49 | 1473 | 414 |  |
| NFKB1 ---- tag | rs230541 | A/A | 1.00 (.-.) | . | . | . | 410 | 33,86 | 184 | 34,46 | 1473 | 414 |  |
| NFKB1 ---- tag |  | A/G or G/G | 0.99 (0.80-1.21) | 0.91 | 0.98 | 1.00 | 801 | 66,14 | 350 | 65,54 | 1473 | 414 |  |
| NFKB1 ---- NA | rs230547 | C/C | 1.00 (.-.) | . | . | . | 981 | 81,01 | 436 | 81,65 | 1473 | 414 |  |
| NFKB1 ---- NA |  | C/T or T/T | 0.92 (0.72-1.18) | 0.53 | 0.93 | 1.00 | 230 | 18,99 | 98 | 18,35 | 1473 | 414 |  |
| NFKB1 ---- tag | rs3774934 | G/G | 1.00 (.-.) | . | . | . | 964 | 79,60 | 426 | 79,78 | 1473 | 414 |  |
| NFKB1 ---- tag |  | G/A or A/A | 0.88 (0.69-1.11) | 0.28 | 0.89 | 1.00 | 247 | 20,40 | 108 | 20,22 | 1473 | 414 |  |
| NFKB1 ---- tag | rs3774968 | G/G | 1.00 (.-.) | . | . | . | 391 | 32,29 | 165 | 30,90 | 1473 | 414 |  |
| NFKB1 ---- tag |  | G/A or A/A | 1.00 (0.81-1.23) | 1.00 | 1.00 | 1.00 | 820 | 67,71 | 369 | 69,10 | 1473 | 414 |  |
| NFKB1 ---- NA | rs4648022 | C/C | 1.00 (.-.) | . | . | . | 1008 | 83,24 | 452 | 84,64 | 1473 | 414 |  |
| NFKB1 ---- NA |  | C/T or T/T | 1.20 (0.91-1.57) | 0.19 | 0.84 | 1.00 | 203 | 16,76 | 82 | 15,36 | 1473 | 414 |  |
| NFKB1 ---- NA | rs4648090 | G/G | 1.00 (.-.) | . | . | . | 878 | 72,50 | 405 | 75,84 | 1473 | 414 |  |
| NFKB1 ---- NA |  | G/A or A/A | 1.04 (0.83-1.30) | 0.74 | 0.94 | 1.00 | 333 | 27,50 | 129 | 24,16 | 1473 | 414 |  |
| NFKB1 ---- tag | rs4648110 | T/T | 1.00 (.-.) | . | . | . | 765 | 63,17 | 343 | 64,23 | 1473 | 414 |  |
| NFKB1 ---- tag |  | T/A or A/A | 0.99 (0.81-1.22) | 0.94 | 0.99 | 1.00 | 446 | 36,83 | 191 | 35,77 | 1473 | 414 |  |
| NFKB1 ---- tag | rs4648141 | G/G | 1.00 (.-.) | . | . | . | 837 | 69,12 | 368 | 68,91 | 1473 | 414 |  |
| NFKB1 ---- tag |  | G/A or A/A | 1.07 (0.87-1.32) | 0.54 | 0.93 | 1.00 | 374 | 30,88 | 166 | 31,09 | 1473 | 414 |  |
| NFKB1 ---- tag | rs4698863 | C/C | 1.00 (.-.) | . | . | . | 562 | 46,41 | 240 | 44,94 | 1473 | 414 |  |
| NFKB1 ---- tag |  | C/T or T/T | 1.02 (0.84-1.24) | 0.86 | 0.97 | 1.00 | 649 | 53,59 | 294 | 55,06 | 1473 | 414 |  |
| NFKB1 ---- NA | rs7674640 | T/T | 1.00 (.-.) | . | . | . | 276 | 22,79 | 123 | 23,03 | 1473 | 414 |  |
| NFKB1 ---- NA |  | T/C or C/C | 1.00 (0.79-1.26) | 0.98 | 1.00 | 1.00 | 935 | 77,21 | 411 | 76,97 | 1473 | 414 |  |
| NFKB1 ---- tag | rs909332 | A/A | 1.00 (.-.) | . | . | . | 1098 | 90,67 | 486 | 91,01 | 1473 | 414 |  |
| NFKB1 ---- tag |  | A/T or T/T | 1.19 (0.85-1.66) | 0.31 | 0.89 | 1.00 | 113 | 9,33 | 48 | 8,99 | 1473 | 414 |  |
| NFKB1 ---- tag | rs997476 | C/C | 1.00 (.-.) | . | . | . | 1082 | 89,35 | 467 | 87,45 | 1473 | 414 |  |
| NFKB1 ---- tag |  | C/A or A/A | 1.00 (0.75-1.35) | 0.98 | 1.00 | 1.00 | 129 | 10,65 | 67 | 12,55 | 1473 | 414 |  |
| NME1 ---- NA | rs10514981 | T/T | 1.00 (.-.) | . | . | . | 745 | 61,52 | 335 | 62,73 | 1473 | 414 |  |
| NME1 ---- NA |  | T/G or G/G | 0.93 (0.76-1.14) | 0.50 | 0.93 | 0.91 | 466 | 38,48 | 199 | 37,27 | 1473 | 414 |  |
| NME1 ---- NA | rs11651252 | T/T | 1.00 (.-.) | . | . | . | 1079 | 89,10 | 471 | 88,20 | 1473 | 414 |  |
| NME1 ---- NA |  | T/C or C/C | 1.05 (0.77-1.44) | 0.74 | 0.94 | 0.91 | 132 | 10,90 | 63 | 11,80 | 1473 | 414 |  |
| NME1 ---- tag | rs11652793 | T/T | 1.00 (.-.) | . | . | . | 798 | 65,90 | 363 | 67,98 | 1473 | 414 |  |
| NME1 ---- tag |  | T/C or C/C | 0.94 (0.77-1.16) | 0.58 | 0.93 | 0.91 | 413 | 34,10 | 171 | 32,02 | 1473 | 414 |  |
| NME1 ---- NA | rs11868380 | C/C | 1.00 (.-.) | . | . | . | 763 | 63,01 | 330 | 61,80 | 1473 | 414 |  |
| NME1 ---- NA |  | C/G or G/G | 1.01 (0.83-1.24) | 0.89 | 0.97 | 0.91 | 448 | 36,99 | 204 | 38,20 | 1473 | 414 |  |
| NME1 ---- NA | rs1558252 | T/T | 1.00 (.-.) | . | . | . | 593 | 48,97 | 239 | 44,76 | 1473 | 414 |  |
| NME1 ---- NA |  | T/C or C/C | 1.12 (0.92-1.37) | 0.24 | 0.87 | 0.91 | 618 | 51,03 | 295 | 55,24 | 1473 | 414 |  |
| NME1 ---- NA | rs1558253 | T/T | 1.00 (.-.) | . | . | . | 1081 | 89,27 | 460 | 86,14 | 1473 | 414 |  |
| NME1 ---- NA |  | T/G or G/G | 1.22 (0.93-1.62) | 0.16 | 0.84 | 0.91 | 130 | 10,73 | 74 | 13,86 | 1473 | 414 |  |
| NME1 ---- tag | rs16949683 | C/C | 1.00 (.-.) | . | . | . | 1124 | 92,82 | 494 | 92,51 | 1473 | 414 |  |
| NME1 ---- tag |  | C/T or T/T | 1.07 (0.74-1.54) | 0.74 | 0.94 | 0.91 | 87 | 7,18 | 40 | 7,49 | 1473 | 414 |  |
| NME1 ---- tag | rs2318784 | C/C | 1.00 (.-.) | . | . | . | 919 | 75,89 | 414 | 77,53 | 1473 | 414 |  |
| NME1 ---- tag |  | C/T or T/T | 1.03 (0.82-1.30) | 0.78 | 0.95 | 0.91 | 292 | 24,11 | 120 | 22,47 | 1473 | 414 |  |
| NME1 ---- NA | rs2318785 | G/G | 1.00 (.-.) | . | . | . | 387 | 31,96 | 160 | 29,96 | 1473 | 414 |  |
| NME1 ---- NA |  | G/A or A/A | 0.99 (0.80-1.22) | 0.91 | 0.98 | 0.91 | 824 | 68,04 | 374 | 70,04 | 1473 | 414 |  |
| NME1 ---- tag | rs3760469 | G/G | 1.00 (.-.) | . | . | . | 327 | 27,00 | 121 | 22,66 | 1473 | 414 |  |
| NME1 ---- tag |  | G/T or T/T | 1.08 (0.86-1.37) | 0.51 | 0.93 | 0.91 | 884 | 73,00 | 413 | 77,34 | 1473 | 414 |  |
| NME1 ---- NA | rs4605213 | G/G | 1.00 (.-.) | . | . | . | 508 | 41,95 | 235 | 44,01 | 1473 | 414 |  |
| NME1 ---- NA |  | G/C or C/C | 0.95 (0.78-1.16) | 0.61 | 0.93 | 0.91 | 703 | 58,05 | 299 | 55,99 | 1473 | 414 |  |
| NME1 ---- NA | rs7207090 | A/A | 1.00 (.-.) | . | . | . | 305 | 25,19 | 133 | 24,91 | 1473 | 414 |  |
| NME1 ---- NA |  | A/T or T/T | 0.88 (0.71-1.11) | 0.28 | 0.89 | 0.91 | 906 | 74,81 | 401 | 75,09 | 1473 | 414 |  |
| NME1 ---- tag | rs7222463 | A/A | 1.00 (.-.) | . | . | . | 323 | 26,67 | 151 | 28,28 | 1473 | 414 |  |
| NME1 ---- tag |  | A/C or C/C | 0.89 (0.72-1.11) | 0.30 | 0.89 | 0.91 | 888 | 73,33 | 383 | 71,72 | 1473 | 414 |  |
| NME1 ---- tag | rs7226059 | C/C | 1.00 (.-.) | . | . | . | 567 | 46,82 | 218 | 40,82 | 1473 | 414 |  |
| NME1 ---- tag |  | C/T or T/T | 1.21 (0.99-1.48) | 0.06 | 0.84 | 0.87 | 644 | 53,18 | 316 | 59,18 | 1473 | 414 |  |
| NME1 ---- NA | rs880178 | G/G | 1.00 (.-.) | . | . | . | 315 | 26,01 | 127 | 23,78 | 1473 | 414 |  |
| NME1 ---- NA |  | G/T or T/T | 1.02 (0.81-1.28) | 0.90 | 0.97 | 0.91 | 896 | 73,99 | 407 | 76,22 | 1473 | 414 |  |
| NME2 ---- tag | rs7220360 | C/C | 1.00 (.-.) | . | . | . | 323 | 26,67 | 149 | 27,90 | 1473 | 414 |  |
| NME2 ---- tag |  | C/G or G/G | 0.90 (0.73-1.12) | 0.36 | 0.90 | 0.36 | 888 | 73,33 | 385 | 72,10 | 1473 | 414 |  |
| PON1 ---- tag | rs2269829 | A/A | 1.00 (.-.) | . | . | . | 620 | 51,20 | 256 | 47,94 | 1473 | 414 |  |
| PON1 ---- tag |  | A/G or G/G | 1.01 (0.83-1.23) | 0.89 | 0.97 | 0.96 | 591 | 48,80 | 278 | 52,06 | 1473 | 414 |  |
| PON1 ---- tag | rs3917527 | A/A | 1.00 (.-.) | . | . | . | 1081 | 89,27 | 489 | 91,57 | 1473 | 414 |  |
| PON1 ---- tag |  | A/G or G/G | 0.75 (0.54-1.04) | 0.08 | 0.84 | 0.42 | 130 | 10,73 | 45 | 8,43 | 1473 | 414 |  |
| PON1 ---- tag | rs3917538 | C/C | 1.00 (.-.) | . | . | . | 704 | 58,13 | 285 | 53,37 | 1473 | 414 |  |
| PON1 ---- tag |  | C/T or T/T | 1.12 (0.92-1.37) | 0.24 | 0.87 | 0.61 | 507 | 41,87 | 249 | 46,63 | 1473 | 414 |  |
| PON1 ---- tag | rs757158 | C/C | 1.00 (.-.) | . | . | . | 424 | 35,01 | 197 | 36,89 | 1473 | 414 |  |
| PON1 ---- tag |  | C/T or T/T | 0.93 (0.76-1.14) | 0.48 | 0.93 | 0.80 | 787 | 64,99 | 337 | 63,11 | 1473 | 414 |  |
| PON1 ---- candidate | rs854560 | A/A | 1.00 (.-.) | . | . | . | 477 | 39,39 | 221 | 41,39 | 1473 | 414 |  |
| PON1 ---- candidate |  | A/T or T/T | 1.01 (0.82-1.23) | 0.96 | 0.99 | 0.96 | 734 | 60,61 | 313 | 58,61 | 1473 | 414 |  |
| PRDM2 ---- tag | rs1015370 | C/C | 1.00 (.-.) | . | . | . | 608 | 50,21 | 296 | 55,43 | 1473 | 414 |  |
| PRDM2 ---- tag |  | C/T or T/T | 0.91 (0.75-1.11) | 0.35 | 0.90 | 0.94 | 603 | 49,79 | 238 | 44,57 | 1473 | 414 |  |
| PRDM2 ---- tag | rs1203634 | A/A | 1.00 (.-.) | . | . | . | 746 | 61,60 | 318 | 59,55 | 1473 | 414 |  |
| PRDM2 ---- tag |  | A/G or G/G | 1.07 (0.88-1.31) | 0.48 | 0.93 | 0.94 | 465 | 38,40 | 216 | 40,45 | 1473 | 414 |  |
| PRDM2 ---- tag | rs1203645 | A/A | 1.00 (.-.) | . | . | . | 487 | 40,21 | 226 | 42,32 | 1473 | 414 |  |
| PRDM2 ---- tag |  | A/C or C/C | 1.02 (0.84-1.24) | 0.87 | 0.97 | 0.96 | 724 | 59,79 | 308 | 57,68 | 1473 | 414 |  |
| PRDM2 ---- tag | rs1406416 | C/C | 1.00 (.-.) | . | . | . | 666 | 55,00 | 273 | 51,12 | 1473 | 414 |  |
| PRDM2 ---- tag |  | C/T or T/T | 1.07 (0.88-1.30) | 0.48 | 0.93 | 0.94 | 545 | 45,00 | 261 | 48,88 | 1473 | 414 |  |
| PRDM2 ---- candidate | rs17350795 | G/G | 1.00 (.-.) | . | . | . | 1158 | 95,62 | 512 | 95,88 | 1473 | 414 |  |
| PRDM2 ---- candidate |  | G/A or A/A | 0.73 (0.44-1.21) | 0.22 | 0.86 | 0.94 | 53 | 4,38 | 22 | 4,12 | 1473 | 414 |  |
| PRDM2 ---- tag | rs1980472 | C/C | 1.00 (.-.) | . | . | . | 683 | 56,40 | 290 | 54,31 | 1473 | 414 |  |
| PRDM2 ---- tag |  | C/G or G/G | 1.04 (0.86-1.27) | 0.68 | 0.93 | 0.94 | 528 | 43,60 | 244 | 45,69 | 1473 | 414 |  |
| PRDM2 ---- tag | rs2235515 | G/G | 1.00 (.-.) | . | . | . | 711 | 58,71 | 306 | 57,30 | 1473 | 414 |  |
| PRDM2 ---- tag |  | G/A or A/A | 1.05 (0.86-1.28) | 0.64 | 0.93 | 0.94 | 500 | 41,29 | 228 | 42,70 | 1473 | 414 |  |
| PRDM2 ---- tag | rs2244634 | A/A | 1.00 (.-.) | . | . | . | 773 | 63,83 | 348 | 65,17 | 1473 | 414 |  |
| PRDM2 ---- tag |  | A/C or C/C | 0.99 (0.80-1.21) | 0.90 | 0.97 | 0.96 | 438 | 36,17 | 186 | 34,83 | 1473 | 414 |  |
| PRDM2 ---- tag | rs2245213 | G/G | 1.00 (.-.) | . | . | . | 855 | 70,60 | 375 | 70,22 | 1473 | 414 |  |
| PRDM2 ---- tag |  | G/T or T/T | 1.04 (0.84-1.29) | 0.71 | 0.93 | 0.94 | 356 | 29,40 | 159 | 29,78 | 1473 | 414 |  |
| PRDM2 ---- tag | rs2294484 | C/C | 1.00 (.-.) | . | . | . | 1003 | 82,82 | 455 | 85,21 | 1473 | 414 |  |
| PRDM2 ---- tag |  | C/G or G/G | 0.99 (0.75-1.31) | 0.96 | 0.99 | 0.96 | 208 | 17,18 | 79 | 14,79 | 1473 | 414 |  |
| PRDM2 ---- tag | rs2744689 | G/G | 1.00 (.-.) | . | . | . | 868 | 71,68 | 379 | 70,97 | 1473 | 414 |  |
| PRDM2 ---- tag |  | G/A or A/A | 1.04 (0.84-1.29) | 0.70 | 0.93 | 0.94 | 343 | 28,32 | 155 | 29,03 | 1473 | 414 |  |
| PRDM2 ---- tag | rs6690270 | A/A | 1.00 (.-.) | . | . | . | 494 | 40,79 | 205 | 38,39 | 1473 | 414 |  |
| PRDM2 ---- tag |  | A/G or G/G | 1.05 (0.86-1.28) | 0.62 | 0.93 | 0.94 | 717 | 59,21 | 329 | 61,61 | 1473 | 414 |  |
| RRM1 ---- tag | rs10835601 | G/G | 1.00 (.-.) | . | . | . | 591 | 48,80 | 256 | 47,94 | 1473 | 414 |  |
| RRM1 ---- tag |  | G/A or A/A | 1.15 (0.95-1.40) | 0.16 | 0.84 | 0.52 | 620 | 51,20 | 278 | 52,06 | 1473 | 414 |  |
| RRM1 ---- tag | rs10835613 | C/C | 1.00 (.-.) | . | . | . | 407 | 33,61 | 182 | 34,08 | 1473 | 414 |  |
| RRM1 ---- tag |  | C/G or G/G | 1.12 (0.91-1.38) | 0.27 | 0.89 | 0.65 | 804 | 66,39 | 352 | 65,92 | 1473 | 414 |  |
| RRM1 ---- NA | rs10835677 | G/G | 1.00 (.-.) | . | . | . | 1017 | 83,98 | 431 | 80,71 | 1473 | 414 |  |
| RRM1 ---- NA |  | G/A or A/A | 1.05 (0.82-1.35) | 0.70 | 0.93 | 0.78 | 194 | 16,02 | 103 | 19,29 | 1473 | 414 |  |
| RRM1 ---- tag | rs10835678 | A/A | 1.00 (.-.) | . | . | . | 1075 | 88,77 | 475 | 88,95 | 1473 | 414 |  |
| RRM1 ---- tag |  | A/G or G/G | 0.92 (0.67-1.26) | 0.61 | 0.93 | 0.78 | 136 | 11,23 | 59 | 11,05 | 1473 | 414 |  |
| RRM1 ---- tag | rs12288551 | C/C | 1.00 (.-.) | . | . | . | 1128 | 93,15 | 480 | 89,89 | 1473 | 414 |  |
| RRM1 ---- tag |  | C/G or G/G | 1.27 (0.92-1.76) | 0.15 | 0.84 | 0.52 | 83 | 6,85 | 54 | 10,11 | 1473 | 414 |  |
| RRM1 ---- NA | rs12806698 | C/C | 1.00 (.-.) | . | . | . | 624 | 51,53 | 277 | 51,87 | 1473 | 414 |  |
| RRM1 ---- NA |  | C/A or A/A | 1.10 (0.91-1.34) | 0.33 | 0.89 | 0.65 | 587 | 48,47 | 257 | 48,13 | 1473 | 414 |  |
| RRM1 ---- NA | rs1465952 | T/T | 1.00 (.-.) | . | . | . | 987 | 81,50 | 440 | 82,40 | 1473 | 414 |  |
| RRM1 ---- NA |  | T/C or C/C | 0.95 (0.73-1.22) | 0.67 | 0.93 | 0.78 | 224 | 18,50 | 94 | 17,60 | 1473 | 414 |  |
| RRM1 ---- tag | rs4910904 | A/A | 1.00 (.-.) | . | . | . | 530 | 43,77 | 219 | 41,01 | 1473 | 414 |  |
| RRM1 ---- tag |  | A/G or G/G | 1.25 (1.02-1.52) | 0.03 | 0.84 | 0.31 | 681 | 56,23 | 315 | 58,99 | 1473 | 414 |  |
| RRM1 ---- tag | rs7103860 | T/T | 1.00 (.-.) | . | . | . | 915 | 75,56 | 416 | 77,90 | 1473 | 414 |  |
| RRM1 ---- tag |  | T/C or C/C | 1.01 (0.80-1.27) | 0.95 | 0.99 | 0.95 | 296 | 24,44 | 118 | 22,10 | 1473 | 414 |  |
| RRM1 ---- tag | rs7115496 | C/C | 1.00 (.-.) | . | . | . | 1026 | 84,72 | 466 | 87,27 | 1473 | 414 |  |
| RRM1 ---- tag |  | C/T or T/T | 0.92 (0.69-1.23) | 0.59 | 0.93 | 0.78 | 185 | 15,28 | 68 | 12,73 | 1473 | 414 |  |
| RRM2 ---- NA | rs1138729 | A/A | 1.00 (.-.) | . | . | . | 884 | 73,00 | 386 | 72,28 | 1473 | 414 |  |
| RRM2 ---- NA |  | A/G or G/G | 1.11 (0.90-1.37) | 0.34 | 0.90 | 0.77 | 327 | 27,00 | 148 | 27,72 | 1473 | 414 |  |
| RRM2 ---- tag | rs4668664 | G/G | 1.00 (.-.) | . | . | . | 607 | 50,12 | 270 | 50,56 | 1473 | 414 |  |
| RRM2 ---- tag |  | G/A or A/A | 0.97 (0.80-1.18) | 0.77 | 0.94 | 0.77 | 604 | 49,88 | 264 | 49,44 | 1473 | 414 |  |
| RRM2 ---- NA | rs6741290 | C/C | 1.00 (.-.) | . | . | . | 388 | 32,04 | 169 | 31,65 | 1473 | 414 |  |
| RRM2 ---- NA |  | C/T or T/T | 1.07 (0.87-1.32) | 0.51 | 0.93 | 0.77 | 823 | 67,96 | 365 | 68,35 | 1473 | 414 |  |
| RRM2 ---- tag | rs7574663 | C/C | 1.00 (.-.) | . | . | . | 796 | 65,73 | 344 | 64,42 | 1473 | 414 |  |
| RRM2 ---- tag |  | C/G or G/G | 1.05 (0.85-1.28) | 0.66 | 0.93 | 0.77 | 415 | 34,27 | 190 | 35,58 | 1473 | 414 |  |
| SHMT1 ---- candidate | rs1979277 | G/G | 1.00 (.-.) | . | . | . | 582 | 48,06 | 273 | 51,12 | 1473 | 414 |  |
| SHMT1 ---- candidate |  | G/A or A/A | 0.89 (0.73-1.08) | 0.25 | 0.88 | 0.52 | 629 | 51,94 | 261 | 48,88 | 1473 | 414 |  |
| SHMT1 ---- tag | rs2168781 | G/G | 1.00 (.-.) | . | . | . | 413 | 34,10 | 208 | 38,95 | 1473 | 414 |  |
| SHMT1 ---- tag |  | G/C or C/C | 0.92 (0.75-1.12) | 0.39 | 0.91 | 0.52 | 798 | 65,90 | 326 | 61,05 | 1473 | 414 |  |
| SHMT1 ---- tag | rs4924849 | C/C | 1.00 (.-.) | . | . | . | 611 | 50,45 | 284 | 53,18 | 1473 | 414 |  |
| SHMT1 ---- tag |  | C/T or T/T | 0.90 (0.74-1.10) | 0.30 | 0.89 | 0.52 | 600 | 49,55 | 250 | 46,82 | 1473 | 414 |  |
| SHMT1 ---- candidate literature | rs9909104 | T/T | 1.00 (.-.) | . | . | . | 621 | 51,28 | 289 | 54,12 | 1473 | 414 |  |
| SHMT1 ---- candidate literature |  | T/C or C/C | 0.94 (0.77-1.15) | 0.54 | 0.93 | 0.54 | 590 | 48,72 | 245 | 45,88 | 1473 | 414 |  |
| SHMT2 ---- tag | rs10876968 | G/G | 1.00 (.-.) | . | . | . | 655 | 54,09 | 292 | 54,68 | 1473 | 414 |  |
| SHMT2 ---- tag |  | G/T or T/T | 0.97 (0.80-1.18) | 0.74 | 0.94 | 0.85 | 556 | 45,91 | 242 | 45,32 | 1473 | 414 |  |
| SHMT2 ---- tag | rs1800165 | T/T | 1.00 (.-.) | . | . | . | 586 | 48,39 | 263 | 49,25 | 1473 | 414 |  |
| SHMT2 ---- tag |  | T/C or C/C | 1.08 (0.89-1.32) | 0.43 | 0.93 | 0.85 | 625 | 51,61 | 271 | 50,75 | 1473 | 414 |  |
| SHMT2 ---- tag | rs7133939 | T/T | 1.00 (.-.) | . | . | . | 360 | 29,73 | 172 | 32,21 | 1473 | 414 |  |
| SHMT2 ---- tag |  | T/A or A/A | 0.98 (0.79-1.21) | 0.85 | 0.97 | 0.85 | 851 | 70,27 | 362 | 67,79 | 1473 | 414 |  |
| SHMT2 ---- tag | rs7485577 | G/G | 1.00 (.-.) | . | . | . | 644 | 53,18 | 291 | 54,49 | 1473 | 414 |  |
| SHMT2 ---- tag |  | G/A or A/A | 1.05 (0.87-1.28) | 0.60 | 0.93 | 0.85 | 567 | 46,82 | 243 | 45,51 | 1473 | 414 |  |
| SHMT2 ---- tag | rs7489231 | T/T | 1.00 (.-.) | . | . | . | 533 | 44,01 | 234 | 43,82 | 1473 | 414 |  |
| SHMT2 ---- tag |  | T/C or C/C | 1.22 (1.00-1.49) | 0.05 | 0.84 | 0.23 | 678 | 55,99 | 300 | 56,18 | 1473 | 414 |  |
| SLC19A1 ---- candidate | rs1051266 | G/G | 1.00 (.-.) | . | . | . | 388 | 32,04 | 153 | 28,65 | 1473 | 414 |  |
| SLC19A1 ---- candidate |  | G/A or A/A | 1.10 (0.89-1.36) | 0.39 | 0.91 | 0.68 | 823 | 67,96 | 381 | 71,35 | 1473 | 414 |  |
| SLC19A1 ---- candidate literature | rs1131596 | T/T | 1.00 (.-.) | . | . | . | 389 | 32,12 | 153 | 28,65 | 1473 | 414 |  |
| SLC19A1 ---- candidate literature |  | T/C or C/C | 1.10 (0.89-1.36) | 0.38 | 0.91 | 0.68 | 822 | 67,88 | 381 | 71,35 | 1473 | 414 |  |
| SLC19A1 ---- tag | rs12483553 | G/G | 1.00 (.-.) | . | . | . | 986 | 81,42 | 416 | 77,90 | 1473 | 414 |  |
| SLC19A1 ---- tag |  | G/A or A/A | 1.22 (0.97-1.53) | 0.09 | 0.84 | 0.60 | 225 | 18,58 | 118 | 22,10 | 1473 | 414 |  |
| SLC19A1 ---- candidate literature | rs12659 | C/C | 1.00 (.-.) | . | . | . | 401 | 33,11 | 162 | 30,34 | 1473 | 414 |  |
| SLC19A1 ---- candidate literature |  | C/T or T/T | 1.08 (0.87-1.33) | 0.49 | 0.93 | 0.69 | 810 | 66,89 | 372 | 69,66 | 1473 | 414 |  |
| SLC19A1 ---- tag | rs3788190 | G/G | 1.00 (.-.) | . | . | . | 379 | 31,30 | 153 | 28,65 | 1473 | 414 |  |
| SLC19A1 ---- tag |  | G/A or A/A | 1.04 (0.84-1.29) | 0.71 | 0.93 | 0.82 | 832 | 68,70 | 381 | 71,35 | 1473 | 414 |  |
| SLC19A1 ---- tag | rs3788205 | C/C | 1.00 (.-.) | . | . | . | 593 | 48,97 | 278 | 52,06 | 1473 | 414 |  |
| SLC19A1 ---- tag |  | C/T or T/T | 0.98 (0.81-1.20) | 0.88 | 0.97 | 0.88 | 618 | 51,03 | 256 | 47,94 | 1473 | 414 |  |
| SLC19A1 ---- tag | rs7279664 | G/G | 1.00 (.-.) | . | . | . | 467 | 38,56 | 221 | 41,39 | 1473 | 414 |  |
| SLC19A1 ---- tag |  | G/T or T/T | 0.90 (0.74-1.09) | 0.27 | 0.89 | 0.68 | 744 | 61,44 | 313 | 58,61 | 1473 | 414 |  |
| SLC29A1 ---- NA | rs1057985 | C/C | 1.00 (.-.) | . | . | . | 511 | 42,20 | 238 | 44,57 | 1473 | 414 |  |
| SLC29A1 ---- NA |  | C/T or T/T | 1.03 (0.85-1.26) | 0.76 | 0.94 | 0.86 | 700 | 57,80 | 296 | 55,43 | 1473 | 414 |  |
| SLC29A1 ---- NA | rs6458375 | C/C | 1.00 (.-.) | . | . | . | 675 | 55,74 | 310 | 58,05 | 1473 | 414 |  |
| SLC29A1 ---- NA |  | C/T or T/T | 0.84 (0.69-1.03) | 0.09 | 0.84 | 0.63 | 536 | 44,26 | 224 | 41,95 | 1473 | 414 |  |
| SLC29A1 ---- NA | rs666462 | C/C | 1.00 (.-.) | . | . | . | 334 | 27,58 | 148 | 27,72 | 1473 | 414 |  |
| SLC29A1 ---- NA |  | C/T or T/T | 1.05 (0.84-1.31) | 0.68 | 0.93 | 0.86 | 877 | 72,42 | 386 | 72,28 | 1473 | 414 |  |
| SLC29A1 ---- NA | rs6905285 | A/A | 1.00 (.-.) | . | . | . | 451 | 37,24 | 186 | 34,83 | 1473 | 414 |  |
| SLC29A1 ---- NA |  | A/T or T/T | 0.96 (0.78-1.17) | 0.67 | 0.93 | 0.86 | 760 | 62,76 | 348 | 65,17 | 1473 | 414 |  |
| SLC29A1 ---- NA | rs693955 | G/G | 1.00 (.-.) | . | . | . | 801 | 66,14 | 353 | 66,10 | 1473 | 414 |  |
| SLC29A1 ---- NA |  | G/T or T/T | 1.08 (0.88-1.33) | 0.48 | 0.93 | 0.86 | 410 | 33,86 | 181 | 33,90 | 1473 | 414 |  |
| SLC29A1 ---- NA | rs747199 | C/C | 1.00 (.-.) | . | . | . | 782 | 64,57 | 349 | 65,36 | 1473 | 414 |  |
| SLC29A1 ---- NA |  | C/G or G/G | 0.98 (0.80-1.20) | 0.86 | 0.97 | 0.86 | 429 | 35,43 | 185 | 34,64 | 1473 | 414 |  |
| SLC29A1 ---- NA | rs9357436 | G/G | 1.00 (.-.) | . | . | . | 845 | 69,78 | 381 | 71,35 | 1473 | 414 |  |
| SLC29A1 ---- NA |  | G/A or A/A | 1.03 (0.84-1.28) | 0.75 | 0.94 | 0.86 | 366 | 30,22 | 153 | 28,65 | 1473 | 414 |  |
| TCN2 ---- tag | rs10418 | C/C | 1.00 (.-.) | . | . | . | 712 | 58,79 | 299 | 55,99 | 1473 | 414 |  |
| TCN2 ---- tag |  | C/T or T/T | 1.08 (0.89-1.31) | 0.46 | 0.93 | 0.91 | 499 | 41,21 | 235 | 44,01 | 1473 | 414 |  |
| TCN2 ---- candidate/singleton | rs1131603 | T/T | 1.00 (.-.) | . | . | . | 1076 | 88,85 | 468 | 87,64 | 1473 | 414 |  |
| TCN2 ---- candidate/singleton |  | T/C or C/C | 1.06 (0.79-1.42) | 0.70 | 0.93 | 0.91 | 135 | 11,15 | 66 | 12,36 | 1473 | 414 |  |
| TCN2 ---- tag | rs1544468 | A/A | 1.00 (.-.) | . | . | . | 308 | 25,43 | 129 | 24,16 | 1473 | 414 |  |
| TCN2 ---- tag |  | A/G or G/G | 1.03 (0.82-1.28) | 0.83 | 0.96 | 0.94 | 903 | 74,57 | 405 | 75,84 | 1473 | 414 |  |
| TCN2 ---- candidate/tag | rs1801198 | C/C | 1.00 (.-.) | . | . | . | 354 | 29,23 | 172 | 32,21 | 1473 | 414 |  |
| TCN2 ---- candidate/tag |  | C/G or G/G | 0.82 (0.67-1.01) | 0.06 | 0.84 | 0.68 | 857 | 70,77 | 362 | 67,79 | 1473 | 414 |  |
| TCN2 ---- tag | rs4820872 | G/G | 1.00 (.-.) | . | . | . | 441 | 36,42 | 204 | 38,20 | 1473 | 414 |  |
| TCN2 ---- tag |  | G/A or A/A | 0.89 (0.73-1.08) | 0.24 | 0.87 | 0.79 | 770 | 63,58 | 330 | 61,80 | 1473 | 414 |  |
| TCN2 ---- tag | rs4820874 | A/A | 1.00 (.-.) | . | . | . | 853 | 70,44 | 388 | 72,66 | 1473 | 414 |  |
| TCN2 ---- tag |  | A/G or G/G | 1.02 (0.82-1.27) | 0.87 | 0.97 | 0.94 | 358 | 29,56 | 146 | 27,34 | 1473 | 414 |  |
| TCN2 ---- tag | rs4820886 | T/T | 1.00 (.-.) | . | . | . | 963 | 79,52 | 438 | 82,02 | 1473 | 414 |  |
| TCN2 ---- tag |  | T/G or G/G | 1.06 (0.83-1.37) | 0.64 | 0.93 | 0.91 | 248 | 20,48 | 96 | 17,98 | 1473 | 414 |  |
| TCN2 ---- candidate | rs4820889 | G/G | 1.00 (.-.) | . | . | . | 1126 | 92,98 | 504 | 94,38 | 1473 | 414 |  |
| TCN2 ---- candidate |  | G/A or A/A | 0.74 (0.49-1.12) | 0.16 | 0.84 | 0.68 | 85 | 7,02 | 30 | 5,62 | 1473 | 414 |  |
| TCN2 ---- tag | rs5997711 | C/C | 1.00 (.-.) | . | . | . | 386 | 31,87 | 188 | 35,21 | 1473 | 414 |  |
| TCN2 ---- tag |  | C/T or T/T | 0.85 (0.70-1.04) | 0.12 | 0.84 | 0.68 | 825 | 68,13 | 346 | 64,79 | 1473 | 414 |  |
| TCN2 ---- tag | rs740234 | T/T | 1.00 (.-.) | . | . | . | 794 | 65,57 | 337 | 63,11 | 1473 | 414 |  |
| TCN2 ---- tag |  | T/C or C/C | 1.05 (0.86-1.28) | 0.66 | 0.93 | 0.91 | 417 | 34,43 | 197 | 36,89 | 1473 | 414 |  |
| TCN2 ---- tag | rs740235 | G/G | 1.00 (.-.) | . | . | . | 418 | 34,52 | 166 | 31,09 | 1473 | 414 |  |
| TCN2 ---- tag |  | G/A or A/A | 1.06 (0.86-1.31) | 0.59 | 0.93 | 0.91 | 793 | 65,48 | 368 | 68,91 | 1473 | 414 |  |
| TCN2 ---- candidate/singleton | rs9606756 | A/A | 1.00 (.-.) | . | . | . | 956 | 78,94 | 435 | 81,46 | 1473 | 414 |  |
| TCN2 ---- candidate/singleton |  | A/G or G/G | 1.00 (0.78-1.29) | 0.98 | 1.00 | 0.98 | 255 | 21,06 | 99 | 18,54 | 1473 | 414 |  |
| TCN2 ---- candidate | rs9621049 | C/C | 1.00 (.-.) | . | . | . | 963 | 79,52 | 438 | 82,02 | 1473 | 414 |  |
| TCN2 ---- candidate |  | C/T or T/T | 1.06 (0.83-1.37) | 0.64 | 0.93 | 0.91 | 248 | 20,48 | 96 | 17,98 | 1473 | 414 |  |
| TK1 ---- NA | rs1065769 | G/G | 1.00 (.-.) | . | . | . | 573 | 47,32 | 243 | 45,51 | 1473 | 414 |  |
| TK1 ---- NA |  | G/A or A/A | 1.10 (0.90-1.34) | 0.34 | 0.90 | 0.73 | 638 | 52,68 | 291 | 54,49 | 1473 | 414 |  |
| TK1 ---- NA | rs12232476 | G/G | 1.00 (.-.) | . | . | . | 1022 | 84,39 | 443 | 82,96 | 1473 | 414 |  |
| TK1 ---- NA |  | G/A or A/A | 1.04 (0.80-1.36) | 0.75 | 0.94 | 0.86 | 189 | 15,61 | 91 | 17,04 | 1473 | 414 |  |
| TK1 ---- tag | rs16970907 | G/G | 1.00 (.-.) | . | . | . | 1050 | 86,71 | 460 | 86,14 | 1473 | 414 |  |
| TK1 ---- tag |  | G/C or C/C | 1.16 (0.87-1.54) | 0.32 | 0.89 | 0.73 | 161 | 13,29 | 74 | 13,86 | 1473 | 414 |  |
| TK1 ---- tag | rs1811086 | C/C | 1.00 (.-.) | . | . | . | 1128 | 93,15 | 493 | 92,32 | 1473 | 414 |  |
| TK1 ---- tag |  | C/T or T/T | 1.08 (0.74-1.58) | 0.68 | 0.93 | 0.86 | 83 | 6,85 | 41 | 7,68 | 1473 | 414 |  |
| TK1 ---- tag | rs2292235 | C/C | 1.00 (.-.) | . | . | . | 379 | 31,30 | 178 | 33,33 | 1473 | 414 |  |
| TK1 ---- tag |  | C/A or A/A | 0.91 (0.74-1.12) | 0.37 | 0.90 | 0.73 | 832 | 68,70 | 356 | 66,67 | 1473 | 414 |  |
| TK1 ---- tag | rs2854701 | A/A | 1.00 (.-.) | . | . | . | 491 | 40,55 | 201 | 37,64 | 1473 | 414 |  |
| TK1 ---- tag |  | A/G or G/G | 1.17 (0.96-1.43) | 0.12 | 0.84 | 0.73 | 720 | 59,45 | 333 | 62,36 | 1473 | 414 |  |
| TK1 ---- tag | rs2854702 | G/G | 1.00 (.-.) | . | . | . | 941 | 77,70 | 401 | 75,09 | 1473 | 414 |  |
| TK1 ---- tag |  | G/A or A/A | 1.07 (0.85-1.34) | 0.57 | 0.93 | 0.86 | 270 | 22,30 | 133 | 24,91 | 1473 | 414 |  |
| TK1 ---- tag | rs9897765 | G/G | 1.00 (.-.) | . | . | . | 649 | 53,59 | 277 | 51,87 | 1473 | 414 |  |
| TK1 ---- tag |  | G/A or A/A | 1.00 (0.82-1.22) | 0.99 | 1.00 | 0.99 | 562 | 46,41 | 257 | 48,13 | 1473 | 414 |  |
| TYMP ---- NA | rs131815 | G/G | 1.00 (.-.) | . | . | . | 639 | 52,77 | 286 | 53,56 | 1473 | 414 |  |
| TYMP ---- NA |  | G/A or A/A | 1.11 (0.91-1.36) | 0.29 | 0.89 | 0.35 | 572 | 47,23 | 248 | 46,44 | 1473 | 414 |  |
| TYMP ---- tag | rs131816 | A/A | 1.00 (.-.) | . | . | . | 716 | 59,12 | 339 | 63,48 | 1473 | 414 |  |
| TYMP ---- tag |  | A/G or G/G | 0.78 (0.63-0.95) | 0.02 | 0.84 | 0.10 | 495 | 40,88 | 195 | 36,52 | 1473 | 414 |  |
| TYMP ---- NA | rs131817 | C/C | 1.00 (.-.) | . | . | . | 395 | 32,62 | 171 | 32,02 | 1473 | 414 |  |
| TYMP ---- NA |  | C/T or T/T | 1.14 (0.92-1.42) | 0.22 | 0.85 | 0.33 | 816 | 67,38 | 363 | 67,98 | 1473 | 414 |  |
| TYMP ---- NA | rs140521 | T/T | 1.00 (.-.) | . | . | . | 617 | 50,95 | 281 | 52,62 | 1473 | 414 |  |
| TYMP ---- NA |  | T/G or G/G | 0.86 (0.71-1.05) | 0.14 | 0.84 | 0.28 | 594 | 49,05 | 253 | 47,38 | 1473 | 414 |  |
| TYMP ---- NA | rs140522 | G/G | 1.00 (.-.) | . | . | . | 537 | 44,34 | 255 | 47,75 | 1473 | 414 |  |
| TYMP ---- NA |  | G/A or A/A | 0.83 (0.68-1.01) | 0.06 | 0.84 | 0.17 | 674 | 55,66 | 279 | 52,25 | 1473 | 414 |  |
| TYMP ---- NA | rs140524 | G/G | 1.00 (.-.) | . | . | . | 823 | 67,96 | 345 | 64,61 | 1473 | 414 |  |
| TYMP ---- NA |  | G/A or A/A | 1.07 (0.87-1.31) | 0.53 | 0.93 | 0.53 | 388 | 32,04 | 189 | 35,39 | 1473 | 414 |  |
| TYMS ---- candidate literature | rs1001761 | C/C | 1.00 (.-.) | . | . | . | 349 | 28,82 | 177 | 33,15 | 1473 | 414 |  |
| TYMS ---- candidate literature |  | C/T or T/T | 0.84 (0.68-1.04) | 0.11 | 0.84 | 0.19 | 862 | 71,18 | 357 | 66,85 | 1473 | 414 |  |
| TYMS ---- candidate literature/tag | rs10502289 | T/T | 1.00 (.-.) | . | . | . | 743 | 61,35 | 357 | 66,85 | 1473 | 414 |  |
| TYMS ---- candidate literature/tag |  | T/A or A/A | 0.82 (0.67-1.01) | 0.06 | 0.84 | 0.19 | 468 | 38,65 | 177 | 33,15 | 1473 | 414 |  |
| TYMS ---- tag | rs15872 | C/C | 1.00 (.-.) | . | . | . | 551 | 45,50 | 267 | 50,00 | 1473 | 414 |  |
| TYMS ---- tag |  | C/T or T/T | 0.86 (0.71-1.05) | 0.14 | 0.84 | 0.22 | 660 | 54,50 | 267 | 50,00 | 1473 | 414 |  |
| TYMS ---- tag | rs2244500 | T/T | 1.00 (.-.) | . | . | . | 350 | 28,90 | 177 | 33,15 | 1473 | 414 |  |
| TYMS ---- tag |  | T/C or C/C | 0.84 (0.68-1.04) | 0.11 | 0.84 | 0.19 | 861 | 71,10 | 357 | 66,85 | 1473 | 414 |  |
| TYMS ---- tag | rs2741182 | G/G | 1.00 (.-.) | . | . | . | 739 | 61,02 | 323 | 60,49 | 1473 | 414 |  |
| TYMS ---- tag |  | G/C or C/C | 0.92 (0.75-1.12) | 0.42 | 0.93 | 0.47 | 472 | 38,98 | 211 | 39,51 | 1473 | 414 |  |
| TYMS ---- candidate literature | rs2847149 | G/G | 1.00 (.-.) | . | . | . | 349 | 28,82 | 177 | 33,15 | 1473 | 414 |  |
| TYMS ---- candidate literature |  | G/A or A/A | 0.84 (0.68-1.04) | 0.11 | 0.84 | 0.19 | 862 | 71,18 | 357 | 66,85 | 1473 | 414 |  |
| TYMS ---- candidate literature | rs2853533 | G/G | 1.00 (.-.) | . | . | . | 914 | 75,47 | 393 | 73,60 | 1473 | 414 |  |
| TYMS ---- candidate literature |  | G/C or C/C | 1.00 (0.80-1.24) | 0.97 | 0.99 | 0.97 | 297 | 24,53 | 141 | 26,40 | 1473 | 414 |  |
| TYMS ---- tag | rs495139 | C/C | 1.00 (.-.) | . | . | . | 415 | 34,27 | 162 | 30,34 | 1473 | 414 |  |
| TYMS ---- tag |  | C/G or G/G | 1.35 (1.09-1.68) | 0.01 | 0.84 | 0.05 | 796 | 65,73 | 372 | 69,66 | 1473 | 414 |  |
| TYMS ---- candidate literature | rs502396 | T/T | 1.00 (.-.) | . | . | . | 338 | 27,91 | 179 | 33,52 | 1473 | 414 |  |
| TYMS ---- candidate literature |  | T/C or C/C | 0.86 (0.70-1.06) | 0.17 | 0.84 | 0.22 | 873 | 72,09 | 355 | 66,48 | 1473 | 414 |  |
| UMPH2 ---- tag | rs2291028 | A/A | 1.00 (.-.) | . | . | . | 521 | 43,02 | 221 | 41,39 | 1473 | 414 |  |
| UMPH2 ---- tag |  | A/G or G/G | 1.08 (0.88-1.32) | 0.44 | 0.93 | 0.67 | 690 | 56,98 | 313 | 58,61 | 1473 | 414 |  |
| UMPH2 ---- NA | rs4789143 | A/A | 1.00 (.-.) | . | . | . | 926 | 76,47 | 428 | 80,15 | 1473 | 414 |  |
| UMPH2 ---- NA |  | A/G or G/G | 0.94 (0.74-1.19) | 0.61 | 0.93 | 0.67 | 285 | 23,53 | 106 | 19,85 | 1473 | 414 |  |
| UMPH2 ---- NA | rs750844 | G/G | 1.00 (.-.) | . | . | . | 612 | 50,54 | 262 | 49,06 | 1473 | 414 |  |
| UMPH2 ---- NA |  | G/A or A/A | 1.04 (0.86-1.27) | 0.67 | 0.93 | 0.67 | 599 | 49,46 | 272 | 50,94 | 1473 | 414 |  |
| UMPK ---- tag | rs11582877 | C/C | 1.00 (.-.) | . | . | . | 893 | 73,74 | 381 | 71,35 | 1473 | 414 |  |
| UMPK ---- tag |  | C/T or T/T | 1.05 (0.85-1.31) | 0.64 | 0.93 | 0.84 | 318 | 26,26 | 153 | 28,65 | 1473 | 414 |  |
| UMPK ---- tag | rs2622903 | A/A | 1.00 (.-.) | . | . | . | 592 | 48,89 | 266 | 49,81 | 1473 | 414 |  |
| UMPK ---- tag |  | A/G or G/G | 0.96 (0.79-1.17) | 0.72 | 0.93 | 0.84 | 619 | 51,11 | 268 | 50,19 | 1473 | 414 |  |
| UMPK ---- tag | rs2820989 | C/C | 1.00 (.-.) | . | . | . | 365 | 30,14 | 167 | 31,27 | 1473 | 414 |  |
| UMPK ---- tag |  | C/G or G/G | 1.02 (0.83-1.26) | 0.84 | 0.97 | 0.84 | 846 | 69,86 | 367 | 68,73 | 1473 | 414 |  |
| UMPK ---- tag | rs6660321 | A/A | 1.00 (.-.) | . | . | . | 923 | 76,22 | 395 | 73,97 | 1473 | 414 |  |
| UMPK ---- tag |  | A/C or C/C | 1.03 (0.83-1.29) | 0.79 | 0.95 | 0.84 | 288 | 23,78 | 139 | 26,03 | 1473 | 414 |  |
| UMPK ---- tag | rs6690084 | T/T | 1.00 (.-.) | . | . | . | 1050 | 86,71 | 451 | 84,46 | 1473 | 414 |  |
| UMPK ---- tag |  | T/C or C/C | 1.22 (0.93-1.60) | 0.15 | 0.84 | 0.76 | 161 | 13,29 | 83 | 15,54 | 1473 | 414 |  |
| UMPS ---- NA | rs1162 | A/A | 1.00 (.-.) | . | . | . | 578 | 47,73 | 233 | 43,63 | 1473 | 414 |  |
| UMPS ---- NA |  | A/G or G/G | 1.29 (1.06-1.57) | 0.01 | 0.84 | 0.07 | 633 | 52,27 | 301 | 56,37 | 1473 | 414 |  |
| UMPS ---- tag | rs13146 | C/C | 1.00 (.-.) | . | . | . | 843 | 69,61 | 363 | 67,98 | 1473 | 414 |  |
| UMPS ---- tag |  | C/T or T/T | 1.13 (0.92-1.39) | 0.26 | 0.89 | 0.31 | 368 | 30,39 | 171 | 32,02 | 1473 | 414 |  |
| UMPS ---- tag | rs16835902 | C/C | 1.00 (.-.) | . | . | . | 392 | 32,37 | 178 | 33,33 | 1473 | 414 |  |
| UMPS ---- tag |  | C/G or G/G | 0.89 (0.72-1.09) | 0.26 | 0.89 | 0.31 | 819 | 67,63 | 356 | 66,67 | 1473 | 414 |  |
| UMPS ---- tag | rs17282057 | T/T | 1.00 (.-.) | . | . | . | 937 | 77,37 | 390 | 73,03 | 1473 | 414 |  |
| UMPS ---- tag |  | T/C or C/C | 1.14 (0.92-1.43) | 0.23 | 0.87 | 0.31 | 274 | 22,63 | 144 | 26,97 | 1473 | 414 |  |
| UMPS ---- tag | rs606552 | A/A | 1.00 (.-.) | . | . | . | 604 | 49,88 | 303 | 56,74 | 1473 | 414 |  |
| UMPS ---- tag |  | A/G or G/G | 0.80 (0.66-0.98) | 0.03 | 0.84 | 0.08 | 607 | 50,12 | 231 | 43,26 | 1473 | 414 |  |
| UMPS ---- tag | rs694897 | C/C | 1.00 (.-.) | . | . | . | 517 | 42,69 | 210 | 39,33 | 1473 | 414 |  |
| UMPS ---- tag |  | C/G or G/G | 0.97 (0.80-1.19) | 0.79 | 0.95 | 0.79 | 694 | 57,31 | 324 | 60,67 | 1473 | 414 |  |
| UNG ---- NA | rs1059262 | T/T | 1.00 (.-.) | . | . | . | 827 | 68,29 | 366 | 68,54 | 1473 | 414 |  |
| UNG ---- NA |  | T/G or G/G | 1.01 (0.82-1.24) | 0.96 | 0.99 | 0.96 | 384 | 31,71 | 168 | 31,46 | 1473 | 414 |  |
| UNG ---- tag | rs2160603 | T/T | 1.00 (.-.) | . | . | . | 839 | 69,28 | 375 | 70,22 | 1473 | 414 |  |
| UNG ---- tag |  | T/C or C/C | 1.16 (0.93-1.44) | 0.19 | 0.84 | 0.86 | 372 | 30,72 | 159 | 29,78 | 1473 | 414 |  |
| UNG ---- tag | rs246079 | A/A | 1.00 (.-.) | . | . | . | 385 | 31,79 | 170 | 31,84 | 1473 | 414 |  |
| UNG ---- tag |  | A/G or G/G | 1.07 (0.87-1.32) | 0.53 | 0.93 | 0.86 | 826 | 68,21 | 364 | 68,16 | 1473 | 414 |  |
| UNG ---- NA | rs246085 | T/T | 1.00 (.-.) | . | . | . | 1066 | 88,03 | 478 | 89,51 | 1473 | 414 |  |
| UNG ---- NA |  | T/C or C/C | 0.89 (0.66-1.22) | 0.48 | 0.93 | 0.86 | 145 | 11,97 | 56 | 10,49 | 1473 | 414 |  |
| UNG ---- NA | rs2569987 | A/A | 1.00 (.-.) | . | . | . | 832 | 68,70 | 370 | 69,29 | 1473 | 414 |  |
| UNG ---- NA |  | A/G or G/G | 0.96 (0.78-1.20) | 0.74 | 0.94 | 0.89 | 379 | 31,30 | 164 | 30,71 | 1473 | 414 |  |
| UNG ---- tag | rs3219243 | T/T | 1.00 (.-.) | . | . | . | 778 | 64,24 | 336 | 62,92 | 1473 | 414 |  |
| UNG ---- tag |  | T/C or C/C | 0.94 (0.77-1.16) | 0.58 | 0.93 | 0.86 | 433 | 35,76 | 198 | 37,08 | 1473 | 414 |  |

|  | | | | | | | | **Ctrl** | | **Cases** | |  | | |
| --- | --- | --- | --- | --- | --- | --- | --- | --- | --- | --- | --- | --- | --- | --- |
| **Gene** | **SNP** | **Genotype** | **HR (95%-CI)** | **p** | **LR__pTrend_** | **FDR__pTrend_** | **FDR_(byGene)_pTrend_** | **N** | **%** | **N** | **%** | **NObsUsed** | **Events** | **LowCount** |
| AARS ---- tag | rs2070203 | T/T | 1.00 (.-.) | . | 0.24 | 0.90 | 0.24 | 329 | 27,17 | 147 | 27,53 | 1473 | 414 |  |
| AARS ---- tag |  | T/C | 1.11 (0.87-1.40) | 0.41 | . | . | . | 600 | 49,55 | 267 | 50,00 | 1473 | 414 |  |
| AARS ---- tag |  | C/C | 1.18 (0.89-1.57) | 0.25 | . | . | . | 282 | 23,29 | 120 | 22,47 | 1473 | 414 |  |
| AARS ---- tag | rs34087264 | G/G | 1.00 (.-.) | . | 0.09 | 0.86 | 0.19 | 365 | 30,14 | 157 | 29,40 | 1473 | 414 |  |
| AARS ---- tag |  | G/A | 0.84 (0.67-1.05) | 0.12 | . | . | . | 590 | 48,72 | 263 | 49,25 | 1473 | 414 |  |
| AARS ---- tag |  | A/A | 0.80 (0.60-1.05) | 0.11 | . | . | . | 256 | 21,14 | 114 | 21,35 | 1473 | 414 |  |
| ABCC4 ---- tag | rs10508023 | G/G | 1.00 (.-.) | . | 0.71 | 0.98 | 0.93 | 949 | 78,36 | 419 | 78,46 | 1473 | 414 | low_count |
| ABCC4 ---- tag |  | G/C | 0.97 (0.76-1.23) | 0.79 | . | . | . | 246 | 20,31 | 108 | 20,22 | 1473 | 414 |  |
| ABCC4 ---- tag |  | C/C | 0.84 (0.31-2.27) | 0.74 | . | . | . | 16 | 1,32 | 7 | 1,31 | 1473 | 414 |  |
| ABCC4 ---- tag | rs1059751 | T/T | 1.00 (.-.) | . | 0.20 | 0.86 | 0.78 | 351 | 28,98 | 146 | 27,34 | 1473 | 414 |  |
| ABCC4 ---- tag |  | T/C | 1.06 (0.84-1.33) | 0.65 | . | . | . | 594 | 49,05 | 264 | 49,44 | 1473 | 414 |  |
| ABCC4 ---- tag |  | C/C | 1.20 (0.91-1.58) | 0.19 | . | . | . | 266 | 21,97 | 124 | 23,22 | 1473 | 414 |  |
| ABCC4 ---- tag | rs11568643 | A/A | 1.00 (.-.) | . | 0.82 | 0.99 | 0.93 | 1011 | 83,48 | 443 | 82,96 | 1473 | 414 | low_count |
| ABCC4 ---- tag |  | A/G | 0.98 (0.75-1.28) | 0.89 | . | . | . | 194 | 16,02 | 87 | 16,29 | 1473 | 414 |  |
| ABCC4 ---- tag |  | G/G | 0.81 (0.20-3.31) | 0.77 | . | . | . | 6 | 0,50 | 4 | 0,75 | 1473 | 414 |  |
| ABCC4 ---- NA | rs11568658 | G/G | 1.00 (.-.) | . | 0.57 | 0.97 | 0.93 | 1143 | 94,38 | 510 | 95,51 | 1473 | 414 | low_count |
| ABCC4 ---- NA |  | G/T | 1.05 (0.65-1.69) | 0.85 | . | . | . | 67 | 5,53 | 23 | 4,31 | 1473 | 414 |  |
| ABCC4 ---- NA |  | T/T | 5.54 (0.76-40.63) | 0.09 | . | . | . | 1 | 0,08 | 1 | 0,19 | 1473 | 414 |  |
| ABCC4 ---- tag | rs12864049 | T/T | 1.00 (.-.) | . | 0.41 | 0.94 | 0.90 | 909 | 75,06 | 396 | 74,16 | 1473 | 414 |  |
| ABCC4 ---- tag |  | T/C | 1.07 (0.85-1.35) | 0.55 | . | . | . | 279 | 23,04 | 127 | 23,78 | 1473 | 414 |  |
| ABCC4 ---- tag |  | C/C | 1.24 (0.66-2.36) | 0.50 | . | . | . | 23 | 1,90 | 11 | 2,06 | 1473 | 414 |  |
| ABCC4 ---- tag | rs1628382 | G/G | 1.00 (.-.) | . | 0.39 | 0.94 | 0.90 | 758 | 62,59 | 329 | 61,61 | 1473 | 414 |  |
| ABCC4 ---- tag |  | G/A | 1.05 (0.86-1.30) | 0.63 | . | . | . | 406 | 33,53 | 184 | 34,46 | 1473 | 414 |  |
| ABCC4 ---- tag |  | A/A | 1.26 (0.76-2.06) | 0.37 | . | . | . | 47 | 3,88 | 21 | 3,93 | 1473 | 414 |  |
| ABCC4 ---- tag | rs1678354 | C/C | 1.00 (.-.) | . | 0.27 | 0.90 | 0.83 | 518 | 42,77 | 218 | 40,82 | 1473 | 414 |  |
| ABCC4 ---- tag |  | C/G | 1.20 (0.98-1.48) | 0.08 | . | . | . | 540 | 44,59 | 256 | 47,94 | 1473 | 414 |  |
| ABCC4 ---- tag |  | G/G | 1.07 (0.77-1.49) | 0.68 | . | . | . | 153 | 12,63 | 60 | 11,24 | 1473 | 414 |  |
| ABCC4 ---- tag | rs1678383 | T/T | 1.00 (.-.) | . | 0.65 | 0.97 | 0.93 | 981 | 81,01 | 432 | 80,90 | 1473 | 414 | low_count |
| ABCC4 ---- tag |  | T/G | 0.82 (0.63-1.06) | 0.13 | . | . | . | 220 | 18,17 | 94 | 17,60 | 1473 | 414 |  |
| ABCC4 ---- tag |  | G/G | 2.64 (1.24-5.64) | 0.01 | . | . | . | 10 | 0,83 | 8 | 1,50 | 1473 | 414 |  |
| ABCC4 ---- tag | rs1678395 | G/G | 1.00 (.-.) | . | 0.08 | 0.85 | 0.53 | 1050 | 86,71 | 456 | 85,39 | 1473 | 414 | low_count |
| ABCC4 ---- tag |  | G/A | 0.79 (0.60-1.05) | 0.10 | . | . | . | 157 | 12,96 | 75 | 14,04 | 1473 | 414 |  |
| ABCC4 ---- tag |  | A/A | 0.62 (0.15-2.55) | 0.51 | . | . | . | 4 | 0,33 | 3 | 0,56 | 1473 | 414 |  |
| ABCC4 ---- tag | rs1678405 | T/T | 1.00 (.-.) | . | 0.04 | 0.85 | 0.38 | 572 | 47,23 | 245 | 45,88 | 1473 | 414 |  |
| ABCC4 ---- tag |  | T/C | 1.06 (0.86-1.30) | 0.61 | . | . | . | 532 | 43,93 | 237 | 44,38 | 1473 | 414 |  |
| ABCC4 ---- tag |  | C/C | 1.54 (1.11-2.13) | 0.01 | . | . | . | 107 | 8,84 | 52 | 9,74 | 1473 | 414 |  |
| ABCC4 ---- tag | rs17189540 | A/A | 1.00 (.-.) | . | 0.27 | 0.90 | 0.83 | 1066 | 88,03 | 452 | 84,64 | 1473 | 414 | low_count |
| ABCC4 ---- tag |  | A/G | 1.15 (0.87-1.51) | 0.32 | . | . | . | 140 | 11,56 | 80 | 14,98 | 1473 | 414 |  |
| ABCC4 ---- tag |  | G/G | 1.93 (0.27-13.96) | 0.51 | . | . | . | 5 | 0,41 | 2 | 0,37 | 1473 | 414 |  |
| ABCC4 ---- tag | rs17235152 | T/T | 1.00 (.-.) | . | 0.69 | 0.98 | 0.93 | 883 | 72,91 | 389 | 72,85 | 1473 | 414 | low_count |
| ABCC4 ---- tag |  | T/C | 1.07 (0.85-1.35) | 0.56 | . | . | . | 297 | 24,53 | 135 | 25,28 | 1473 | 414 |  |
| ABCC4 ---- tag |  | C/C | 0.95 (0.49-1.87) | 0.89 | . | . | . | 31 | 2,56 | 10 | 1,87 | 1473 | 414 |  |
| ABCC4 ---- tag | rs17268122 | G/G | 1.00 (.-.) | . | 0.40 | 0.94 | 0.90 | 733 | 60,53 | 334 | 62,55 | 1473 | 414 |  |
| ABCC4 ---- tag |  | G/T | 0.96 (0.78-1.19) | 0.73 | . | . | . | 423 | 34,93 | 172 | 32,21 | 1473 | 414 |  |
| ABCC4 ---- tag |  | T/T | 0.80 (0.50-1.28) | 0.35 | . | . | . | 55 | 4,54 | 28 | 5,24 | 1473 | 414 |  |
| ABCC4 ---- tag | rs17268170 | C/C | 1.00 (.-.) | . | 0.83 | 0.99 | 0.93 | 1000 | 82,58 | 429 | 80,34 | 1473 | 414 | low_count |
| ABCC4 ---- tag |  | C/T | 0.98 (0.76-1.25) | 0.86 | . | . | . | 203 | 16,76 | 98 | 18,35 | 1473 | 414 |  |
| ABCC4 ---- tag |  | T/T | 1.62 (0.65-4.02) | 0.30 | . | . | . | 8 | 0,66 | 7 | 1,31 | 1473 | 414 |  |
| ABCC4 ---- tag | rs1729764 | A/A | 1.00 (.-.) | . | 0.68 | 0.98 | 0.93 | 954 | 78,78 | 435 | 81,46 | 1473 | 414 | low_count |
| ABCC4 ---- tag |  | A/G | 0.89 (0.68-1.15) | 0.36 | . | . | . | 245 | 20,23 | 91 | 17,04 | 1473 | 414 |  |
| ABCC4 ---- tag |  | G/G | 1.69 (0.69-4.15) | 0.25 | . | . | . | 12 | 0,99 | 8 | 1,50 | 1473 | 414 |  |
| ABCC4 ---- tag | rs1729767 | T/T | 1.00 (.-.) | . | 0.27 | 0.90 | 0.83 | 653 | 53,92 | 272 | 50,94 | 1473 | 414 |  |
| ABCC4 ---- tag |  | T/C | 1.20 (0.98-1.47) | 0.08 | . | . | . | 471 | 38,89 | 230 | 43,07 | 1473 | 414 |  |
| ABCC4 ---- tag |  | C/C | 1.00 (0.66-1.51) | 0.99 | . | . | . | 87 | 7,18 | 32 | 5,99 | 1473 | 414 |  |
| ABCC4 ---- tag | rs17300935 | C/C | 1.00 (.-.) | . | 0.82 | 0.99 | 0.93 | 886 | 73,16 | 397 | 74,34 | 1473 | 414 | low_count |
| ABCC4 ---- tag |  | C/G | 1.03 (0.82-1.30) | 0.78 | . | . | . | 295 | 24,36 | 129 | 24,16 | 1473 | 414 |  |
| ABCC4 ---- tag |  | G/G | 0.69 (0.30-1.56) | 0.37 | . | . | . | 30 | 2,48 | 8 | 1,50 | 1473 | 414 |  |
| ABCC4 ---- tag | rs1750190 | G/G | 1.00 (.-.) | . | 0.66 | 0.97 | 0.93 | 325 | 26,84 | 128 | 23,97 | 1473 | 414 |  |
| ABCC4 ---- tag |  | G/A | 1.26 (0.99-1.60) | 0.06 | . | . | . | 583 | 48,14 | 286 | 53,56 | 1473 | 414 |  |
| ABCC4 ---- tag |  | A/A | 1.06 (0.80-1.41) | 0.67 | . | . | . | 303 | 25,02 | 120 | 22,47 | 1473 | 414 |  |
| ABCC4 ---- tag | rs1750996 | A/A | 1.00 (.-.) | . | 0.50 | 0.96 | 0.93 | 813 | 67,13 | 362 | 67,79 | 1473 | 414 |  |
| ABCC4 ---- tag |  | A/G | 0.96 (0.77-1.20) | 0.72 | . | . | . | 365 | 30,14 | 153 | 28,65 | 1473 | 414 |  |
| ABCC4 ---- tag |  | G/G | 1.64 (0.98-2.74) | 0.06 | . | . | . | 33 | 2,73 | 19 | 3,56 | 1473 | 414 |  |
| ABCC4 ---- tag | rs1751025 | C/C | 1.00 (.-.) | . | 0.04 | 0.85 | 0.38 | 581 | 47,98 | 245 | 45,88 | 1473 | 414 |  |
| ABCC4 ---- tag |  | C/G | 1.11 (0.90-1.37) | 0.33 | . | . | . | 515 | 42,53 | 230 | 43,07 | 1473 | 414 |  |
| ABCC4 ---- tag |  | G/G | 1.41 (1.03-1.93) | 0.03 | . | . | . | 115 | 9,50 | 59 | 11,05 | 1473 | 414 |  |
| ABCC4 ---- tag | rs1751051 | T/T | 1.00 (.-.) | . | 0.87 | 0.99 | 0.93 | 521 | 43,02 | 219 | 41,01 | 1473 | 414 |  |
| ABCC4 ---- tag |  | T/A | 0.89 (0.72-1.10) | 0.28 | . | . | . | 542 | 44,76 | 250 | 46,82 | 1473 | 414 |  |
| ABCC4 ---- tag |  | A/A | 1.07 (0.78-1.46) | 0.68 | . | . | . | 148 | 12,22 | 65 | 12,17 | 1473 | 414 |  |
| ABCC4 ---- tag | rs1764416 | G/G | 1.00 (.-.) | . | 0.62 | 0.97 | 0.93 | 1039 | 85,80 | 462 | 86,52 | 1473 | 414 | low_count |
| ABCC4 ---- tag |  | G/A | 0.94 (0.69-1.27) | 0.68 | . | . | . | 166 | 13,71 | 71 | 13,30 | 1473 | 414 |  |
| ABCC4 ---- tag |  | A/A | 0.00 (0.00-2E213) | 0.97 | . | . | . | 6 | 0,50 | 1 | 0,19 | 1473 | 414 |  |
| ABCC4 ---- tag | rs2274401 | T/T | 1.00 (.-.) | . | 0.59 | 0.97 | 0.93 | 752 | 62,10 | 335 | 62,73 | 1473 | 414 |  |
| ABCC4 ---- tag |  | T/C | 1.01 (0.82-1.25) | 0.90 | . | . | . | 398 | 32,87 | 180 | 33,71 | 1473 | 414 |  |
| ABCC4 ---- tag |  | C/C | 0.75 (0.43-1.28) | 0.29 | . | . | . | 61 | 5,04 | 19 | 3,56 | 1473 | 414 |  |
| ABCC4 ---- tag | rs2892716 | C/C | 1.00 (.-.) | . | 0.43 | 0.94 | 0.90 | 463 | 38,23 | 215 | 40,26 | 1473 | 414 |  |
| ABCC4 ---- tag |  | C/T | 1.18 (0.96-1.46) | 0.12 | . | . | . | 584 | 48,22 | 258 | 48,31 | 1473 | 414 |  |
| ABCC4 ---- tag |  | T/T | 1.03 (0.75-1.43) | 0.84 | . | . | . | 164 | 13,54 | 61 | 11,42 | 1473 | 414 |  |
| ABCC4 ---- tag | rs3782964 | C/C | 1.00 (.-.) | . | 0.32 | 0.94 | 0.88 | 801 | 66,14 | 378 | 70,79 | 1473 | 414 |  |
| ABCC4 ---- tag |  | C/T | 0.85 (0.68-1.06) | 0.16 | . | . | . | 382 | 31,54 | 140 | 26,22 | 1473 | 414 |  |
| ABCC4 ---- tag |  | T/T | 1.18 (0.60-2.30) | 0.64 | . | . | . | 28 | 2,31 | 16 | 3,00 | 1473 | 414 |  |
| ABCC4 ---- tag | rs3818494 | C/C | 1.00 (.-.) | . | 0.00 | 0.60 | 0.21 | 543 | 44,84 | 236 | 44,19 | 1473 | 414 |  |
| ABCC4 ---- tag |  | C/G | 1.27 (1.02-1.56) | 0.03 | . | . | . | 545 | 45,00 | 235 | 44,01 | 1473 | 414 |  |
| ABCC4 ---- tag |  | G/G | 1.49 (1.09-2.03) | 0.01 | . | . | . | 123 | 10,16 | 63 | 11,80 | 1473 | 414 |  |
| ABCC4 ---- tag | rs3864997 | G/G | 1.00 (.-.) | . | 0.87 | 0.99 | 0.93 | 309 | 25,52 | 141 | 26,40 | 1473 | 414 |  |
| ABCC4 ---- tag |  | G/T | 1.08 (0.86-1.36) | 0.52 | . | . | . | 624 | 51,53 | 285 | 53,37 | 1473 | 414 |  |
| ABCC4 ---- tag |  | T/T | 0.97 (0.72-1.29) | 0.81 | . | . | . | 278 | 22,96 | 108 | 20,22 | 1473 | 414 |  |
| ABCC4 ---- tag | rs4148421 | G/G | 1.00 (.-.) | . | 0.54 | 0.97 | 0.93 | 361 | 29,81 | 148 | 27,72 | 1473 | 414 |  |
| ABCC4 ---- tag |  | G/A | 1.01 (0.80-1.27) | 0.94 | . | . | . | 601 | 49,63 | 288 | 53,93 | 1473 | 414 |  |
| ABCC4 ---- tag |  | A/A | 0.91 (0.68-1.21) | 0.50 | . | . | . | 249 | 20,56 | 98 | 18,35 | 1473 | 414 |  |
| ABCC4 ---- tag | rs4148446 | G/G | 1.00 (.-.) | . | 0.86 | 0.99 | 0.93 | 401 | 33,11 | 193 | 36,14 | 1473 | 414 |  |
| ABCC4 ---- tag |  | G/A | 1.08 (0.87-1.34) | 0.50 | . | . | . | 597 | 49,30 | 261 | 48,88 | 1473 | 414 |  |
| ABCC4 ---- tag |  | A/A | 0.99 (0.74-1.34) | 0.97 | . | . | . | 213 | 17,59 | 80 | 14,98 | 1473 | 414 |  |
| ABCC4 ---- tag | rs4148455 | G/G | 1.00 (.-.) | . | 0.19 | 0.86 | 0.78 | 922 | 76,14 | 402 | 75,28 | 1473 | 414 | low_count |
| ABCC4 ---- tag |  | G/A | 1.27 (1.00-1.60) | 0.05 | . | . | . | 265 | 21,88 | 124 | 23,22 | 1473 | 414 |  |
| ABCC4 ---- tag |  | A/A | 0.82 (0.38-1.75) | 0.61 | . | . | . | 24 | 1,98 | 8 | 1,50 | 1473 | 414 |  |
| ABCC4 ---- tag | rs4148540 | C/C | 1.00 (.-.) | . | 0.82 | 0.99 | 0.93 | 1085 | 89,60 | 462 | 86,52 | 1473 | 414 | low_count |
| ABCC4 ---- tag |  | C/T | 1.01 (0.76-1.35) | 0.94 | . | . | . | 121 | 9,99 | 69 | 12,92 | 1473 | 414 |  |
| ABCC4 ---- tag |  | T/T | 0.70 (0.22-2.22) | 0.54 | . | . | . | 5 | 0,41 | 3 | 0,56 | 1473 | 414 |  |
| ABCC4 ---- tag | rs4148542 | G/G | 1.00 (.-.) | . | 0.75 | 0.98 | 0.93 | 335 | 27,66 | 144 | 26,97 | 1473 | 414 |  |
| ABCC4 ---- tag |  | G/A | 0.99 (0.79-1.26) | 0.96 | . | . | . | 580 | 47,89 | 268 | 50,19 | 1473 | 414 |  |
| ABCC4 ---- tag |  | A/A | 0.95 (0.72-1.26) | 0.74 | . | . | . | 296 | 24,44 | 122 | 22,85 | 1473 | 414 |  |
| ABCC4 ---- tag | rs4148544 | G/G | 1.00 (.-.) | . | 0.19 | 0.86 | 0.78 | 502 | 41,45 | 247 | 46,25 | 1473 | 414 |  |
| ABCC4 ---- tag |  | G/A | 0.79 (0.64-0.98) | 0.03 | . | . | . | 569 | 46,99 | 215 | 40,26 | 1473 | 414 |  |
| ABCC4 ---- tag |  | A/A | 0.94 (0.68-1.28) | 0.68 | . | . | . | 140 | 11,56 | 72 | 13,48 | 1473 | 414 |  |
| ABCC4 ---- tag | rs4283094 | C/C | 1.00 (.-.) | . | 0.89 | 0.99 | 0.93 | 321 | 26,51 | 114 | 21,35 | 1473 | 414 |  |
| ABCC4 ---- tag |  | C/G | 1.15 (0.90-1.48) | 0.27 | . | . | . | 605 | 49,96 | 289 | 54,12 | 1473 | 414 |  |
| ABCC4 ---- tag |  | G/G | 0.99 (0.74-1.32) | 0.94 | . | . | . | 285 | 23,53 | 131 | 24,53 | 1473 | 414 |  |
| ABCC4 ---- tag | rs4636781 | A/A | 1.00 (.-.) | . | 0.02 | 0.85 | 0.38 | 866 | 71,51 | 377 | 70,60 | 1473 | 414 |  |
| ABCC4 ---- tag |  | A/G | 1.24 (0.99-1.55) | 0.06 | . | . | . | 318 | 26,26 | 142 | 26,59 | 1473 | 414 |  |
| ABCC4 ---- tag |  | G/G | 1.66 (0.95-2.92) | 0.08 | . | . | . | 27 | 2,23 | 15 | 2,81 | 1473 | 414 |  |
| ABCC4 ---- tag | rs4771910 | T/T | 1.00 (.-.) | . | 0.56 | 0.97 | 0.93 | 595 | 49,13 | 261 | 48,88 | 1473 | 414 |  |
| ABCC4 ---- tag |  | T/C | 0.97 (0.79-1.19) | 0.77 | . | . | . | 507 | 41,87 | 226 | 42,32 | 1473 | 414 |  |
| ABCC4 ---- tag |  | C/C | 0.90 (0.63-1.28) | 0.55 | . | . | . | 109 | 9,00 | 47 | 8,80 | 1473 | 414 |  |
| ABCC4 ---- tag | rs4773850 | T/T | 1.00 (.-.) | . | 0.03 | 0.85 | 0.38 | 547 | 45,17 | 273 | 51,12 | 1473 | 414 |  |
| ABCC4 ---- tag |  | T/G | 0.93 (0.76-1.14) | 0.48 | . | . | . | 542 | 44,76 | 213 | 39,89 | 1473 | 414 |  |
| ABCC4 ---- tag |  | G/G | 0.65 (0.45-0.94) | 0.02 | . | . | . | 122 | 10,07 | 48 | 8,99 | 1473 | 414 |  |
| ABCC4 ---- tag | rs7981095 | A/A | 1.00 (.-.) | . | 0.26 | 0.90 | 0.83 | 781 | 64,49 | 343 | 64,23 | 1473 | 414 |  |
| ABCC4 ---- tag |  | A/T | 0.86 (0.69-1.06) | 0.16 | . | . | . | 389 | 32,12 | 168 | 31,46 | 1473 | 414 |  |
| ABCC4 ---- tag |  | T/T | 0.98 (0.57-1.69) | 0.94 | . | . | . | 41 | 3,39 | 23 | 4,31 | 1473 | 414 |  |
| ABCC4 ---- tag | rs8001444 | C/C | 1.00 (.-.) | . | 0.46 | 0.95 | 0.92 | 419 | 34,60 | 177 | 33,15 | 1473 | 414 |  |
| ABCC4 ---- tag |  | C/T | 0.96 (0.77-1.19) | 0.71 | . | . | . | 569 | 46,99 | 267 | 50,00 | 1473 | 414 |  |
| ABCC4 ---- tag |  | T/T | 0.90 (0.67-1.20) | 0.46 | . | . | . | 223 | 18,41 | 90 | 16,85 | 1473 | 414 |  |
| ABCC4 ---- tag | rs931111 | T/T | 1.00 (.-.) | . | 0.71 | 0.98 | 0.93 | 822 | 67,88 | 349 | 65,36 | 1473 | 414 |  |
| ABCC4 ---- tag |  | T/C | 1.15 (0.93-1.42) | 0.20 | . | . | . | 348 | 28,74 | 160 | 29,96 | 1473 | 414 |  |
| ABCC4 ---- tag |  | C/C | 0.83 (0.52-1.34) | 0.46 | . | . | . | 41 | 3,39 | 25 | 4,68 | 1473 | 414 |  |
| ABCC4 ---- tag | rs943288 | T/T | 1.00 (.-.) | . | 0.08 | 0.85 | 0.53 | 924 | 76,30 | 409 | 76,59 | 1473 | 414 |  |
| ABCC4 ---- tag |  | T/A | 1.12 (0.88-1.42) | 0.37 | . | . | . | 267 | 22,05 | 112 | 20,97 | 1473 | 414 |  |
| ABCC4 ---- tag |  | A/A | 1.83 (1.02-3.29) | 0.04 | . | . | . | 20 | 1,65 | 13 | 2,43 | 1473 | 414 |  |
| ABCC4 ---- tag | rs943290 | A/A | 1.00 (.-.) | . | 0.43 | 0.94 | 0.90 | 663 | 54,75 | 284 | 53,18 | 1473 | 414 |  |
| ABCC4 ---- tag |  | A/G | 0.90 (0.74-1.11) | 0.33 | . | . | . | 456 | 37,65 | 213 | 39,89 | 1473 | 414 |  |
| ABCC4 ---- tag |  | G/G | 0.95 (0.64-1.41) | 0.80 | . | . | . | 92 | 7,60 | 37 | 6,93 | 1473 | 414 |  |
| ABCC4 ---- tag | rs9516530 | C/C | 1.00 (.-.) | . | 0.89 | 0.99 | 0.93 | 663 | 54,75 | 294 | 55,06 | 1473 | 414 |  |
| ABCC4 ---- tag |  | C/T | 0.93 (0.75-1.15) | 0.49 | . | . | . | 457 | 37,74 | 196 | 36,70 | 1473 | 414 |  |
| ABCC4 ---- tag |  | T/T | 1.16 (0.81-1.67) | 0.41 | . | . | . | 91 | 7,51 | 44 | 8,24 | 1473 | 414 |  |
| ABCC4 ---- tag | rs9516551 | C/C | 1.00 (.-.) | . | 0.34 | 0.94 | 0.88 | 968 | 79,93 | 419 | 78,46 | 1473 | 414 | low_count |
| ABCC4 ---- tag |  | C/A | 0.90 (0.71-1.14) | 0.40 | . | . | . | 232 | 19,16 | 111 | 20,79 | 1473 | 414 |  |
| ABCC4 ---- tag |  | A/A | 0.77 (0.28-2.11) | 0.61 | . | . | . | 11 | 0,91 | 4 | 0,75 | 1473 | 414 |  |
| ABCC4 ---- tag | rs9524822 | T/T | 1.00 (.-.) | . | 0.30 | 0.92 | 0.87 | 780 | 64,41 | 351 | 65,73 | 1473 | 414 |  |
| ABCC4 ---- tag |  | T/C | 0.85 (0.68-1.05) | 0.13 | . | . | . | 392 | 32,37 | 160 | 29,96 | 1473 | 414 |  |
| ABCC4 ---- tag |  | C/C | 1.07 (0.63-1.81) | 0.80 | . | . | . | 39 | 3,22 | 23 | 4,31 | 1473 | 414 |  |
| ABCC4 ---- tag | rs9524861 | G/G | 1.00 (.-.) | . | 0.14 | 0.86 | 0.72 | 626 | 51,69 | 263 | 49,25 | 1473 | 414 |  |
| ABCC4 ---- tag |  | G/C | 0.97 (0.79-1.19) | 0.77 | . | . | . | 485 | 40,05 | 227 | 42,51 | 1473 | 414 |  |
| ABCC4 ---- tag |  | C/C | 0.71 (0.50-1.03) | 0.07 | . | . | . | 100 | 8,26 | 44 | 8,24 | 1473 | 414 |  |
| ABCC4 ---- tag | rs9524902 | T/T | 1.00 (.-.) | . | 0.55 | 0.97 | 0.93 | 330 | 27,25 | 157 | 29,40 | 1473 | 414 |  |
| ABCC4 ---- tag |  | T/C | 0.83 (0.66-1.04) | 0.11 | . | . | . | 606 | 50,04 | 255 | 47,75 | 1473 | 414 |  |
| ABCC4 ---- tag |  | C/C | 0.93 (0.71-1.22) | 0.61 | . | . | . | 275 | 22,71 | 122 | 22,85 | 1473 | 414 |  |
| ABCC4 ---- tag | rs9556455 | G/G | 1.00 (.-.) | . | 0.96 | 0.99 | 0.96 | 915 | 75,56 | 402 | 75,28 | 1473 | 414 | low_count |
| ABCC4 ---- tag |  | G/A | 1.09 (0.87-1.36) | 0.46 | . | . | . | 279 | 23,04 | 124 | 23,22 | 1473 | 414 |  |
| ABCC4 ---- tag |  | A/A | 0.56 (0.23-1.37) | 0.20 | . | . | . | 17 | 1,40 | 8 | 1,50 | 1473 | 414 |  |
| ABCC4 ---- NA | rs9561778 | G/G | 1.00 (.-.) | . | 0.89 | 0.99 | 0.93 | 793 | 65,48 | 348 | 65,17 | 1473 | 414 |  |
| ABCC4 ---- NA |  | G/T | 1.10 (0.89-1.35) | 0.40 | . | . | . | 365 | 30,14 | 171 | 32,02 | 1473 | 414 |  |
| ABCC4 ---- NA |  | T/T | 0.67 (0.37-1.24) | 0.20 | . | . | . | 53 | 4,38 | 15 | 2,81 | 1473 | 414 |  |
| ABCC4 ---- tag | rs9561811 | C/C | 1.00 (.-.) | . | 0.13 | 0.86 | 0.72 | 805 | 66,47 | 360 | 67,42 | 1473 | 414 |  |
| ABCC4 ---- tag |  | C/T | 1.13 (0.91-1.40) | 0.28 | . | . | . | 370 | 30,55 | 155 | 29,03 | 1473 | 414 |  |
| ABCC4 ---- tag |  | T/T | 1.36 (0.82-2.27) | 0.23 | . | . | . | 36 | 2,97 | 19 | 3,56 | 1473 | 414 |  |
| ABCC4 ---- tag | rs9590183 | T/T | 1.00 (.-.) | . | 0.04 | 0.85 | 0.38 | 1059 | 87,45 | 473 | 88,58 | 1473 | 414 | low_count |
| ABCC4 ---- tag |  | T/A | 0.70 (0.51-0.98) | 0.04 | . | . | . | 145 | 11,97 | 59 | 11,05 | 1473 | 414 |  |
| ABCC4 ---- tag |  | A/A | 1.10 (0.15-7.89) | 0.93 | . | . | . | 7 | 0,58 | 2 | 0,37 | 1473 | 414 |  |
| ABCC4 ---- tag | rs997777 | T/T | 1.00 (.-.) | . | 0.92 | 0.99 | 0.94 | 604 | 49,88 | 266 | 49,81 | 1473 | 414 |  |
| ABCC4 ---- tag |  | T/A | 1.05 (0.86-1.29) | 0.63 | . | . | . | 507 | 41,87 | 224 | 41,95 | 1473 | 414 |  |
| ABCC4 ---- tag |  | A/A | 0.96 (0.67-1.38) | 0.82 | . | . | . | 100 | 8,26 | 44 | 8,24 | 1473 | 414 |  |
| ADH1B ---- tag | rs1159918 | G/G | 1.00 (.-.) | . | 0.89 | 0.99 | 0.89 | 537 | 44,34 | 244 | 45,69 | 1473 | 414 |  |
| ADH1B ---- tag |  | G/T | 1.03 (0.83-1.26) | 0.81 | . | . | . | 532 | 43,93 | 239 | 44,76 | 1473 | 414 |  |
| ADH1B ---- tag |  | T/T | 1.00 (0.71-1.43) | 0.98 | . | . | . | 142 | 11,73 | 51 | 9,55 | 1473 | 414 |  |
| ADH1B ---- candidate literature | rs1229984 | G/G | 1.00 (.-.) | . | 0.07 | 0.85 | 0.37 | 1090 | 90,01 | 490 | 91,76 | 1473 | 414 | low_count |
| ADH1B ---- candidate literature |  | G/A | 0.79 (0.55-1.13) | 0.20 | . | . | . | 115 | 9,50 | 44 | 8,24 | 1473 | 414 |  |
| ADH1B ---- candidate literature |  | A/A | 0.00 (0.00-5E234) | 0.97 | . | . | . | 6 | 0,50 | 0 | 0,00 | 1473 | 414 |  |
| ADH1B ---- tag | rs12507573 | C/C | 1.00 (.-.) | . | 0.56 | 0.97 | 0.71 | 379 | 31,30 | 161 | 30,15 | 1473 | 414 |  |
| ADH1B ---- tag |  | C/A | 0.90 (0.72-1.13) | 0.37 | . | . | . | 583 | 48,14 | 262 | 49,06 | 1473 | 414 |  |
| ADH1B ---- tag |  | A/A | 0.94 (0.71-1.24) | 0.64 | . | . | . | 249 | 20,56 | 111 | 20,79 | 1473 | 414 |  |
| ADH1B ---- tag | rs1693457 | T/T | 1.00 (.-.) | . | 0.15 | 0.86 | 0.37 | 834 | 68,87 | 386 | 72,28 | 1473 | 414 |  |
| ADH1B ---- tag |  | T/C | 0.87 (0.70-1.09) | 0.24 | . | . | . | 331 | 27,33 | 136 | 25,47 | 1473 | 414 |  |
| ADH1B ---- tag |  | C/C | 0.72 (0.37-1.41) | 0.34 | . | . | . | 46 | 3,80 | 12 | 2,25 | 1473 | 414 |  |
| ADH1B ---- tag | rs2066701 | C/C | 1.00 (.-.) | . | 0.35 | 0.94 | 0.58 | 554 | 45,75 | 230 | 43,07 | 1473 | 414 |  |
| ADH1B ---- tag |  | C/T | 1.06 (0.86-1.30) | 0.59 | . | . | . | 533 | 44,01 | 245 | 45,88 | 1473 | 414 |  |
| ADH1B ---- tag |  | T/T | 1.17 (0.84-1.64) | 0.36 | . | . | . | 124 | 10,24 | 59 | 11,05 | 1473 | 414 |  |
| ADH1C ---- tag | rs11936869 | C/C | 1.00 (.-.) | . | 0.99 | 1.00 | 0.99 | 619 | 51,11 | 287 | 53,75 | 1473 | 414 |  |
| ADH1C ---- tag |  | C/G | 0.93 (0.76-1.15) | 0.50 | . | . | . | 491 | 40,55 | 196 | 36,70 | 1473 | 414 |  |
| ADH1C ---- tag |  | G/G | 1.10 (0.78-1.55) | 0.58 | . | . | . | 101 | 8,34 | 51 | 9,55 | 1473 | 414 |  |
| ADH1C ---- tag | rs1229849 | T/T | 1.00 (.-.) | . | 0.12 | 0.86 | 0.53 | 653 | 53,92 | 269 | 50,37 | 1473 | 414 |  |
| ADH1C ---- tag |  | T/A | 1.06 (0.87-1.30) | 0.57 | . | . | . | 490 | 40,46 | 223 | 41,76 | 1473 | 414 |  |
| ADH1C ---- tag |  | A/A | 1.44 (0.99-2.09) | 0.06 | . | . | . | 68 | 5,62 | 42 | 7,87 | 1473 | 414 |  |
| ADH1C ---- tag | rs1229863 | A/A | 1.00 (.-.) | . | 0.83 | 0.99 | 0.98 | 892 | 73,66 | 386 | 72,28 | 1473 | 414 |  |
| ADH1C ---- tag |  | A/T | 0.93 (0.73-1.18) | 0.56 | . | . | . | 291 | 24,03 | 129 | 24,16 | 1473 | 414 |  |
| ADH1C ---- tag |  | T/T | 1.34 (0.80-2.24) | 0.26 | . | . | . | 28 | 2,31 | 19 | 3,56 | 1473 | 414 |  |
| ADH1C ---- tag | rs1229980 | C/C | 1.00 (.-.) | . | 0.08 | 0.85 | 0.53 | 1095 | 90,42 | 467 | 87,45 | 1473 | 414 | low_count |
| ADH1C ---- tag |  | C/G | 1.22 (0.89-1.66) | 0.21 | . | . | . | 114 | 9,41 | 64 | 11,99 | 1473 | 414 |  |
| ADH1C ---- tag |  | G/G | 2.83 (0.89-9.03) | 0.08 | . | . | . | 2 | 0,17 | 3 | 0,56 | 1473 | 414 |  |
| ADH1C ---- candidate | rs1693482 | C/C | 1.00 (.-.) | . | 0.48 | 0.95 | 0.84 | 507 | 41,87 | 213 | 39,89 | 1473 | 414 |  |
| ADH1C ---- candidate |  | C/T | 0.99 (0.80-1.22) | 0.92 | . | . | . | 554 | 45,75 | 237 | 44,38 | 1473 | 414 |  |
| ADH1C ---- candidate |  | T/T | 1.15 (0.86-1.55) | 0.35 | . | . | . | 150 | 12,39 | 84 | 15,73 | 1473 | 414 |  |
| ADH1C ---- tag | rs2173201 | C/C | 1.00 (.-.) | . | 0.87 | 0.99 | 0.98 | 685 | 56,56 | 319 | 59,74 | 1473 | 414 |  |
| ADH1C ---- tag |  | C/A | 0.93 (0.75-1.15) | 0.49 | . | . | . | 452 | 37,32 | 175 | 32,77 | 1473 | 414 |  |
| ADH1C ---- tag |  | A/A | 1.20 (0.82-1.76) | 0.35 | . | . | . | 74 | 6,11 | 40 | 7,49 | 1473 | 414 |  |
| ADH1C ---- tag | rs2298753 | T/T | 1.00 (.-.) | . | 0.28 | 0.90 | 0.83 | 995 | 82,16 | 446 | 83,52 | 1473 | 414 | low_count |
| ADH1C ---- tag |  | T/C | 0.85 (0.64-1.12) | 0.25 | . | . | . | 201 | 16,60 | 78 | 14,61 | 1473 | 414 |  |
| ADH1C ---- tag |  | C/C | 0.90 (0.39-2.06) | 0.80 | . | . | . | 15 | 1,24 | 10 | 1,87 | 1473 | 414 |  |
| ADH1C ---- tag | rs2866152 | G/G | 1.00 (.-.) | . | 0.56 | 0.97 | 0.84 | 744 | 61,44 | 314 | 58,80 | 1473 | 414 |  |
| ADH1C ---- tag |  | G/C | 1.03 (0.84-1.26) | 0.78 | . | . | . | 419 | 34,60 | 196 | 36,70 | 1473 | 414 |  |
| ADH1C ---- tag |  | C/C | 1.17 (0.74-1.85) | 0.51 | . | . | . | 48 | 3,96 | 24 | 4,49 | 1473 | 414 |  |
| ADH1C ---- tag | rs904096 | T/T | 1.00 (.-.) | . | 0.49 | 0.96 | 0.84 | 501 | 41,37 | 210 | 39,33 | 1473 | 414 |  |
| ADH1C ---- tag |  | T/G | 0.99 (0.80-1.22) | 0.90 | . | . | . | 560 | 46,24 | 240 | 44,94 | 1473 | 414 |  |
| ADH1C ---- tag |  | G/G | 1.15 (0.86-1.55) | 0.35 | . | . | . | 150 | 12,39 | 84 | 15,73 | 1473 | 414 |  |
| BHMT ---- tag | rs10944 | A/A | 1.00 (.-.) | . | 0.86 | 0.99 | 0.98 | 288 | 23,78 | 131 | 24,53 | 1473 | 414 |  |
| BHMT ---- tag |  | A/C | 0.82 (0.64-1.04) | 0.10 | . | . | . | 638 | 52,68 | 270 | 50,56 | 1473 | 414 |  |
| BHMT ---- tag |  | C/C | 1.01 (0.77-1.33) | 0.92 | . | . | . | 285 | 23,53 | 133 | 24,91 | 1473 | 414 |  |
| BHMT ---- tag | rs12655567 | C/C | 1.00 (.-.) | . | 0.26 | 0.90 | 0.87 | 437 | 36,09 | 214 | 40,07 | 1473 | 414 |  |
| BHMT ---- tag |  | C/G | 0.75 (0.61-0.93) | 0.01 | . | . | . | 591 | 48,80 | 241 | 45,13 | 1473 | 414 |  |
| BHMT ---- tag |  | G/G | 0.96 (0.72-1.28) | 0.78 | . | . | . | 183 | 15,11 | 79 | 14,79 | 1473 | 414 |  |
| BHMT ---- tag | rs1291041 | G/G | 1.00 (.-.) | . | 0.37 | 0.94 | 0.87 | 499 | 41,21 | 245 | 45,88 | 1473 | 414 |  |
| BHMT ---- tag |  | G/T | 0.71 (0.58-0.88) | 0.00 | . | . | . | 566 | 46,74 | 221 | 41,39 | 1473 | 414 |  |
| BHMT ---- tag |  | T/T | 1.11 (0.82-1.49) | 0.51 | . | . | . | 146 | 12,06 | 68 | 12,73 | 1473 | 414 |  |
| BHMT ---- tag | rs16876500 | C/C | 1.00 (.-.) | . | 0.76 | 0.98 | 0.98 | 965 | 79,69 | 422 | 79,03 | 1473 | 414 | low_count |
| BHMT ---- tag |  | C/T | 1.11 (0.86-1.42) | 0.42 | . | . | . | 236 | 19,49 | 105 | 19,66 | 1473 | 414 |  |
| BHMT ---- tag |  | T/T | 0.68 (0.25-1.85) | 0.45 | . | . | . | 10 | 0,83 | 7 | 1,31 | 1473 | 414 |  |
| BHMT ---- tag | rs492842 | A/A | 1.00 (.-.) | . | 0.98 | 0.99 | 0.98 | 443 | 36,58 | 209 | 39,14 | 1473 | 414 |  |
| BHMT ---- tag |  | A/G | 0.94 (0.76-1.16) | 0.55 | . | . | . | 604 | 49,88 | 247 | 46,25 | 1473 | 414 |  |
| BHMT ---- tag |  | G/G | 1.05 (0.78-1.40) | 0.77 | . | . | . | 164 | 13,54 | 78 | 14,61 | 1473 | 414 |  |
| BHMT ---- tag | rs558133 | T/T | 1.00 (.-.) | . | 0.35 | 0.94 | 0.87 | 579 | 47,81 | 257 | 48,13 | 1473 | 414 |  |
| BHMT ---- tag |  | T/G | 0.90 (0.73-1.11) | 0.32 | . | . | . | 487 | 40,21 | 223 | 41,76 | 1473 | 414 |  |
| BHMT ---- tag |  | G/G | 0.90 (0.65-1.26) | 0.55 | . | . | . | 145 | 11,97 | 54 | 10,11 | 1473 | 414 |  |
| BHMT ---- tag | rs9637824 | A/A | 1.00 (.-.) | . | 0.93 | 0.99 | 0.98 | 444 | 36,66 | 203 | 38,01 | 1473 | 414 |  |
| BHMT ---- tag |  | A/G | 0.91 (0.74-1.13) | 0.40 | . | . | . | 600 | 49,55 | 255 | 47,75 | 1473 | 414 |  |
| BHMT ---- tag |  | G/G | 1.08 (0.80-1.45) | 0.62 | . | . | . | 167 | 13,79 | 76 | 14,23 | 1473 | 414 |  |
| BHMT2 ---- tag | rs16876512 | C/C | 1.00 (.-.) | . | 0.82 | 0.99 | 0.96 | 958 | 79,11 | 421 | 78,84 | 1473 | 414 | low_count |
| BHMT2 ---- tag |  | C/T | 1.10 (0.86-1.41) | 0.46 | . | . | . | 242 | 19,98 | 105 | 19,66 | 1473 | 414 |  |
| BHMT2 ---- tag |  | T/T | 0.66 (0.24-1.80) | 0.42 | . | . | . | 11 | 0,91 | 8 | 1,50 | 1473 | 414 |  |
| BHMT2 ---- tag | rs2461248 | A/A | 1.00 (.-.) | . | 0.96 | 0.99 | 0.96 | 283 | 23,37 | 130 | 24,34 | 1473 | 414 |  |
| BHMT2 ---- tag |  | A/T | 0.82 (0.65-1.05) | 0.11 | . | . | . | 641 | 52,93 | 268 | 50,19 | 1473 | 414 |  |
| BHMT2 ---- tag |  | T/T | 0.99 (0.76-1.30) | 0.96 | . | . | . | 287 | 23,70 | 136 | 25,47 | 1473 | 414 |  |
| BHMT2 ---- tag | rs2909856 | T/T | 1.00 (.-.) | . | 0.94 | 0.99 | 0.96 | 486 | 40,13 | 225 | 42,13 | 1473 | 414 |  |
| BHMT2 ---- tag |  | T/C | 0.91 (0.74-1.12) | 0.38 | . | . | . | 580 | 47,89 | 239 | 44,76 | 1473 | 414 |  |
| BHMT2 ---- tag |  | C/C | 1.09 (0.81-1.48) | 0.57 | . | . | . | 145 | 11,97 | 70 | 13,11 | 1473 | 414 |  |
| BHMT2 ---- tag | rs476620 | A/A | 1.00 (.-.) | . | 0.89 | 0.99 | 0.96 | 445 | 36,75 | 202 | 37,83 | 1473 | 414 |  |
| BHMT2 ---- tag |  | A/G | 0.92 (0.74-1.14) | 0.44 | . | . | . | 599 | 49,46 | 256 | 47,94 | 1473 | 414 |  |
| BHMT2 ---- tag |  | G/G | 1.08 (0.80-1.45) | 0.61 | . | . | . | 167 | 13,79 | 76 | 14,23 | 1473 | 414 |  |
| BHMT2 ---- candidate literature | rs626105 | G/G | 1.00 (.-.) | . | 0.23 | 0.90 | 0.96 | 753 | 62,18 | 341 | 63,86 | 1473 | 414 |  |
| BHMT2 ---- candidate literature |  | G/A | 0.81 (0.65-1.00) | 0.05 | . | . | . | 407 | 33,61 | 174 | 32,58 | 1473 | 414 |  |
| BHMT2 ---- candidate literature |  | A/A | 1.19 (0.71-1.98) | 0.51 | . | . | . | 51 | 4,21 | 19 | 3,56 | 1473 | 414 |  |
| BHMT2 ---- tag | rs631305 | G/G | 1.00 (.-.) | . | 0.41 | 0.94 | 0.96 | 830 | 68,54 | 374 | 70,04 | 1473 | 414 |  |
| BHMT2 ---- tag |  | G/A | 0.84 (0.67-1.05) | 0.13 | . | . | . | 349 | 28,82 | 144 | 26,97 | 1473 | 414 |  |
| BHMT2 ---- tag |  | A/A | 1.24 (0.70-2.17) | 0.46 | . | . | . | 32 | 2,64 | 16 | 3,00 | 1473 | 414 |  |
| CBS ---- tag | rs11701048 | C/C | 1.00 (.-.) | . | 0.57 | 0.97 | 0.92 | 1024 | 84,56 | 465 | 87,08 | 1473 | 414 | low_count |
| CBS ---- tag |  | C/T | 0.95 (0.71-1.27) | 0.72 | . | . | . | 182 | 15,03 | 67 | 12,55 | 1473 | 414 |  |
| CBS ---- tag |  | T/T | 0.52 (0.07-3.71) | 0.51 | . | . | . | 5 | 0,41 | 2 | 0,37 | 1473 | 414 |  |
| CBS ---- tag | rs234706 | G/G | 1.00 (.-.) | . | 0.91 | 0.99 | 0.97 | 535 | 44,18 | 228 | 42,70 | 1473 | 414 |  |
| CBS ---- tag |  | G/A | 1.09 (0.89-1.34) | 0.41 | . | . | . | 524 | 43,27 | 246 | 46,07 | 1473 | 414 |  |
| CBS ---- tag |  | A/A | 0.89 (0.64-1.25) | 0.52 | . | . | . | 152 | 12,55 | 60 | 11,24 | 1473 | 414 |  |
| CBS ---- tag | rs234711 | C/C | 1.00 (.-.) | . | 0.26 | 0.90 | 0.75 | 728 | 60,12 | 305 | 57,12 | 1473 | 414 |  |
| CBS ---- tag |  | C/A | 1.15 (0.93-1.41) | 0.19 | . | . | . | 415 | 34,27 | 202 | 37,83 | 1473 | 414 |  |
| CBS ---- tag |  | A/A | 1.07 (0.66-1.75) | 0.77 | . | . | . | 68 | 5,62 | 27 | 5,06 | 1473 | 414 |  |
| CBS ---- candidate literature | rs234713 | G/G | 1.00 (.-.) | . | 0.64 | 0.97 | 0.92 | 602 | 49,71 | 263 | 49,25 | 1473 | 414 |  |
| CBS ---- candidate literature |  | G/A | 1.06 (0.87-1.31) | 0.55 | . | . | . | 496 | 40,96 | 223 | 41,76 | 1473 | 414 |  |
| CBS ---- candidate literature |  | A/A | 1.03 (0.71-1.50) | 0.87 | . | . | . | 113 | 9,33 | 48 | 8,99 | 1473 | 414 |  |
| CBS ---- tag | rs2839623 | T/T | 1.00 (.-.) | . | 0.74 | 0.98 | 0.92 | 1000 | 82,58 | 440 | 82,40 | 1473 | 414 | low_count |
| CBS ---- tag |  | T/A | 1.08 (0.83-1.40) | 0.56 | . | . | . | 199 | 16,43 | 92 | 17,23 | 1473 | 414 |  |
| CBS ---- tag |  | A/A | 0.71 (0.17-2.84) | 0.62 | . | . | . | 12 | 0,99 | 2 | 0,37 | 1473 | 414 |  |
| CBS ---- tag | rs2839626 | C/C | 1.00 (.-.) | . | 0.30 | 0.92 | 0.75 | 560 | 46,24 | 234 | 43,82 | 1473 | 414 |  |
| CBS ---- tag |  | C/T | 1.14 (0.92-1.40) | 0.23 | . | . | . | 522 | 43,10 | 235 | 44,01 | 1473 | 414 |  |
| CBS ---- tag |  | T/T | 1.11 (0.80-1.53) | 0.53 | . | . | . | 129 | 10,65 | 65 | 12,17 | 1473 | 414 |  |
| CBS ---- tag | rs422791 | T/T | 1.00 (.-.) | . | 0.30 | 0.92 | 0.75 | 618 | 51,03 | 262 | 49,06 | 1473 | 414 |  |
| CBS ---- tag |  | T/C | 1.20 (0.98-1.47) | 0.09 | . | . | . | 496 | 40,96 | 223 | 41,76 | 1473 | 414 |  |
| CBS ---- tag |  | C/C | 1.02 (0.71-1.47) | 0.90 | . | . | . | 97 | 8,01 | 49 | 9,18 | 1473 | 414 |  |
| CBS ---- tag | rs706209 | C/C | 1.00 (.-.) | . | 0.97 | 0.99 | 0.97 | 371 | 30,64 | 161 | 30,15 | 1473 | 414 |  |
| CBS ---- tag |  | C/T | 0.91 (0.73-1.14) | 0.43 | . | . | . | 602 | 49,71 | 255 | 47,75 | 1473 | 414 |  |
| CBS ---- tag |  | T/T | 1.02 (0.78-1.35) | 0.88 | . | . | . | 238 | 19,65 | 118 | 22,10 | 1473 | 414 |  |
| CBS ---- tag | rs719037 | A/A | 1.00 (.-.) | . | 0.09 | 0.86 | 0.75 | 417 | 34,43 | 174 | 32,58 | 1473 | 414 |  |
| CBS ---- tag |  | A/G | 1.16 (0.93-1.45) | 0.19 | . | . | . | 571 | 47,15 | 258 | 48,31 | 1473 | 414 |  |
| CBS ---- tag |  | G/G | 1.26 (0.94-1.68) | 0.12 | . | . | . | 223 | 18,41 | 102 | 19,10 | 1473 | 414 |  |
| CBS ---- tag | rs719038 | T/T | 1.00 (.-.) | . | 0.37 | 0.94 | 0.75 | 524 | 43,27 | 213 | 39,89 | 1473 | 414 |  |
| CBS ---- tag |  | T/C | 1.03 (0.84-1.28) | 0.76 | . | . | . | 529 | 43,68 | 242 | 45,32 | 1473 | 414 |  |
| CBS ---- tag |  | C/C | 1.16 (0.86-1.56) | 0.33 | . | . | . | 158 | 13,05 | 79 | 14,79 | 1473 | 414 |  |
| DHFR ---- tag | rs10474632 | G/G | 1.00 (.-.) | . | 0.42 | 0.94 | 0.60 | 1020 | 84,23 | 453 | 84,83 | 1473 | 414 | low_count |
| DHFR ---- tag |  | G/A | 0.92 (0.70-1.22) | 0.58 | . | . | . | 184 | 15,19 | 78 | 14,61 | 1473 | 414 |  |
| DHFR ---- tag |  | A/A | 0.67 (0.21-2.12) | 0.50 | . | . | . | 7 | 0,58 | 3 | 0,56 | 1473 | 414 |  |
| DHFR ---- tag | rs11951910 | T/T | 1.00 (.-.) | . | 0.66 | 0.98 | 0.66 | 976 | 80,59 | 429 | 80,34 | 1473 | 414 | low_count |
| DHFR ---- tag |  | T/C | 1.24 (0.96-1.60) | 0.09 | . | . | . | 214 | 17,67 | 100 | 18,73 | 1473 | 414 |  |
| DHFR ---- tag |  | C/C | 0.46 (0.17-1.26) | 0.13 | . | . | . | 21 | 1,73 | 5 | 0,94 | 1473 | 414 |  |
| DHFR ---- tag | rs1643665 | T/T | 1.00 (.-.) | . | 0.28 | 0.90 | 0.60 | 568 | 46,90 | 252 | 47,19 | 1473 | 414 |  |
| DHFR ---- tag |  | T/C | 1.00 (0.82-1.23) | 0.97 | . | . | . | 520 | 42,94 | 239 | 44,76 | 1473 | 414 |  |
| DHFR ---- tag |  | C/C | 0.74 (0.50-1.08) | 0.12 | . | . | . | 123 | 10,16 | 43 | 8,05 | 1473 | 414 |  |
| DHFR ---- tag | rs1650717 | T/T | 1.00 (.-.) | . | 0.40 | 0.94 | 0.60 | 661 | 54,58 | 277 | 51,87 | 1473 | 414 |  |
| DHFR ---- tag |  | T/G | 1.20 (0.98-1.48) | 0.08 | . | . | . | 448 | 36,99 | 217 | 40,64 | 1473 | 414 |  |
| DHFR ---- tag |  | G/G | 0.97 (0.67-1.40) | 0.85 | . | . | . | 102 | 8,42 | 40 | 7,49 | 1473 | 414 |  |
| DHFR ---- tag | rs1805355 | G/G | 1.00 (.-.) | . | 0.12 | 0.86 | 0.60 | 1067 | 88,11 | 457 | 85,58 | 1473 | 414 | low_count |
| DHFR ---- tag |  | G/A | 1.32 (1.00-1.74) | 0.05 | . | . | . | 138 | 11,40 | 75 | 14,04 | 1473 | 414 |  |
| DHFR ---- tag |  | A/A | 0.00 (0.00-2E223) | 0.97 | . | . | . | 6 | 0,50 | 2 | 0,37 | 1473 | 414 |  |
| DHFR ---- tag | rs6151617 | A/A | 1.00 (.-.) | . | 0.26 | 0.90 | 0.60 | 449 | 37,08 | 193 | 36,14 | 1473 | 414 |  |
| DHFR ---- tag |  | A/G | 1.05 (0.85-1.30) | 0.67 | . | . | . | 554 | 45,75 | 266 | 49,81 | 1473 | 414 |  |
| DHFR ---- tag |  | G/G | 0.79 (0.58-1.07) | 0.12 | . | . | . | 208 | 17,18 | 75 | 14,04 | 1473 | 414 |  |
| DHFR ---- tag | rs6864493 | T/T | 1.00 (.-.) | . | 0.07 | 0.85 | 0.60 | 679 | 56,07 | 315 | 58,99 | 1473 | 414 |  |
| DHFR ---- tag |  | T/C | 0.84 (0.69-1.04) | 0.11 | . | . | . | 454 | 37,49 | 190 | 35,58 | 1473 | 414 |  |
| DHFR ---- tag |  | C/C | 0.77 (0.49-1.21) | 0.26 | . | . | . | 78 | 6,44 | 29 | 5,43 | 1473 | 414 |  |
| DHFR ---- tag | rs836788 | G/G | 1.00 (.-.) | . | 0.59 | 0.97 | 0.66 | 519 | 42,86 | 226 | 42,32 | 1473 | 414 |  |
| DHFR ---- tag |  | G/A | 1.05 (0.85-1.29) | 0.68 | . | . | . | 530 | 43,77 | 237 | 44,38 | 1473 | 414 |  |
| DHFR ---- tag |  | A/A | 1.08 (0.80-1.45) | 0.64 | . | . | . | 162 | 13,38 | 71 | 13,30 | 1473 | 414 |  |
| DHFR ---- tag | rs836790 | A/A | 1.00 (.-.) | . | 0.34 | 0.94 | 0.60 | 855 | 70,60 | 360 | 67,42 | 1473 | 414 |  |
| DHFR ---- tag |  | A/G | 1.10 (0.89-1.37) | 0.37 | . | . | . | 326 | 26,92 | 162 | 30,34 | 1473 | 414 |  |
| DHFR ---- tag |  | G/G | 1.14 (0.59-2.24) | 0.69 | . | . | . | 30 | 2,48 | 12 | 2,25 | 1473 | 414 |  |
| DHFR ---- tag | rs836817 | G/G | 1.00 (.-.) | . | 0.59 | 0.97 | 0.66 | 568 | 46,90 | 242 | 45,32 | 1473 | 414 |  |
| DHFR ---- tag |  | G/T | 1.14 (0.93-1.40) | 0.22 | . | . | . | 507 | 41,87 | 239 | 44,76 | 1473 | 414 |  |
| DHFR ---- tag |  | T/T | 0.99 (0.71-1.38) | 0.96 | . | . | . | 136 | 11,23 | 53 | 9,93 | 1473 | 414 |  |
| DNMT1 ---- candidate | rs2228612 | A/A | 1.00 (.-.) | . | 0.73 | 0.98 | 0.73 | 1046 | 86,37 | 463 | 86,70 | 1473 | 414 | low_count |
| DNMT1 ---- candidate |  | A/G | 1.06 (0.79-1.41) | 0.71 | . | . | . | 159 | 13,13 | 69 | 12,92 | 1473 | 414 |  |
| DNMT1 ---- candidate |  | G/G | 1.00 (0.24-4.08) | 1.00 | . | . | . | 6 | 0,50 | 2 | 0,37 | 1473 | 414 |  |
| DNMT3A ---- tag | rs10460566 | A/A | 1.00 (.-.) | . | 0.56 | 0.97 | 0.72 | 676 | 55,82 | 329 | 61,61 | 1473 | 414 |  |
| DNMT3A ---- tag |  | A/G | 1.00 (0.81-1.24) | 0.98 | . | . | . | 469 | 38,73 | 183 | 34,27 | 1473 | 414 |  |
| DNMT3A ---- tag |  | G/G | 0.79 (0.48-1.29) | 0.34 | . | . | . | 66 | 5,45 | 22 | 4,12 | 1473 | 414 |  |
| DNMT3A ---- candidate literature | rs11695471 | T/T | 1.00 (.-.) | . | 0.56 | 0.97 | 0.72 | 539 | 44,51 | 233 | 43,63 | 1473 | 414 |  |
| DNMT3A ---- candidate literature |  | T/A | 1.05 (0.85-1.29) | 0.67 | . | . | . | 547 | 45,17 | 230 | 43,07 | 1473 | 414 |  |
| DNMT3A ---- candidate literature |  | A/A | 1.08 (0.80-1.48) | 0.61 | . | . | . | 125 | 10,32 | 71 | 13,30 | 1473 | 414 |  |
| DNMT3A ---- tag | rs11887120 | C/C | 1.00 (.-.) | . | 0.44 | 0.94 | 0.72 | 423 | 34,93 | 188 | 35,21 | 1473 | 414 |  |
| DNMT3A ---- tag |  | C/T | 0.94 (0.76-1.17) | 0.60 | . | . | . | 581 | 47,98 | 248 | 46,44 | 1473 | 414 |  |
| DNMT3A ---- tag |  | T/T | 0.90 (0.68-1.19) | 0.46 | . | . | . | 207 | 17,09 | 98 | 18,35 | 1473 | 414 |  |
| DNMT3A ---- tag | rs12991495 | T/T | 1.00 (.-.) | . | 0.24 | 0.90 | 0.72 | 584 | 48,22 | 249 | 46,63 | 1473 | 414 |  |
| DNMT3A ---- tag |  | T/C | 1.01 (0.82-1.24) | 0.95 | . | . | . | 525 | 43,35 | 218 | 40,82 | 1473 | 414 |  |
| DNMT3A ---- tag |  | C/C | 1.28 (0.94-1.75) | 0.12 | . | . | . | 102 | 8,42 | 67 | 12,55 | 1473 | 414 |  |
| DNMT3A ---- tag | rs13401241 | A/A | 1.00 (.-.) | . | 0.96 | 0.99 | 0.96 | 342 | 28,24 | 149 | 27,90 | 1473 | 414 |  |
| DNMT3A ---- tag |  | A/C | 0.92 (0.73-1.16) | 0.49 | . | . | . | 593 | 48,97 | 279 | 52,25 | 1473 | 414 |  |
| DNMT3A ---- tag |  | C/C | 1.00 (0.75-1.33) | 0.99 | . | . | . | 276 | 22,79 | 106 | 19,85 | 1473 | 414 |  |
| DNMT3A ---- candidate literature | rs13420827 | C/C | 1.00 (.-.) | . | 0.36 | 0.94 | 0.72 | 798 | 65,90 | 360 | 67,42 | 1473 | 414 |  |
| DNMT3A ---- candidate literature |  | C/G | 0.94 (0.76-1.17) | 0.57 | . | . | . | 370 | 30,55 | 152 | 28,46 | 1473 | 414 |  |
| DNMT3A ---- candidate literature |  | G/G | 0.81 (0.49-1.33) | 0.41 | . | . | . | 43 | 3,55 | 22 | 4,12 | 1473 | 414 |  |
| DNMT3A ---- tag | rs13428812 | A/A | 1.00 (.-.) | . | 0.63 | 0.97 | 0.72 | 569 | 46,99 | 257 | 48,13 | 1473 | 414 |  |
| DNMT3A ---- tag |  | A/G | 1.11 (0.90-1.36) | 0.34 | . | . | . | 517 | 42,69 | 236 | 44,19 | 1473 | 414 |  |
| DNMT3A ---- tag |  | G/G | 0.97 (0.66-1.44) | 0.90 | . | . | . | 125 | 10,32 | 41 | 7,68 | 1473 | 414 |  |
| DNMT3A ---- tag | rs4665287 | C/C | 1.00 (.-.) | . | 0.27 | 0.90 | 0.72 | 810 | 66,89 | 366 | 68,54 | 1473 | 414 |  |
| DNMT3A ---- tag |  | C/T | 0.92 (0.74-1.15) | 0.47 | . | . | . | 355 | 29,31 | 146 | 27,34 | 1473 | 414 |  |
| DNMT3A ---- tag |  | T/T | 0.79 (0.48-1.29) | 0.34 | . | . | . | 46 | 3,80 | 22 | 4,12 | 1473 | 414 |  |
| DNMT3B ---- tag | rs13045669 | A/A | 1.00 (.-.) | . | 0.11 | 0.86 | 0.32 | 1121 | 92,57 | 496 | 92,88 | 1473 | 414 | low_count |
| DNMT3B ---- tag |  | A/G | 0.69 (0.44-1.08) | 0.11 | . | . | . | 89 | 7,35 | 37 | 6,93 | 1473 | 414 |  |
| DNMT3B ---- tag |  | G/G | . (.-.) | . | . | . | . | 1 | 0,08 | 1 | 0,19 | 1473 | 414 | low_count |
| DNMT3B ---- tag | rs17123673 | A/A | 1.00 (.-.) | . | 0.95 | 0.99 | 0.95 | 1111 | 91,74 | 487 | 91,20 | 1473 | 414 | low_count |
| DNMT3B ---- tag |  | A/G | 0.95 (0.67-1.33) | 0.75 | . | . | . | 98 | 8,09 | 45 | 8,43 | 1473 | 414 |  |
| DNMT3B ---- tag |  | G/G | 2.54 (0.61-10.56) | 0.20 | . | . | . | 2 | 0,17 | 2 | 0,37 | 1473 | 414 |  |
| DNMT3B ---- tag | rs183603 | A/A | 1.00 (.-.) | . | 0.05 | 0.85 | 0.26 | 662 | 54,67 | 302 | 56,55 | 1473 | 414 |  |
| DNMT3B ---- tag |  | A/G | 0.88 (0.71-1.08) | 0.21 | . | . | . | 464 | 38,32 | 206 | 38,58 | 1473 | 414 |  |
| DNMT3B ---- tag |  | G/G | 0.66 (0.42-1.05) | 0.08 | . | . | . | 85 | 7,02 | 26 | 4,87 | 1473 | 414 |  |
| DNMT3B ---- tag | rs2235760 | C/C | 1.00 (.-.) | . | 0.52 | 0.97 | 0.65 | 864 | 71,35 | 371 | 69,48 | 1473 | 414 |  |
| DNMT3B ---- tag |  | C/T | 1.15 (0.92-1.43) | 0.22 | . | . | . | 315 | 26,01 | 147 | 27,53 | 1473 | 414 |  |
| DNMT3B ---- tag |  | T/T | 0.86 (0.47-1.58) | 0.63 | . | . | . | 32 | 2,64 | 16 | 3,00 | 1473 | 414 |  |
| DNMT3B ---- tag | rs2424908 | C/C | 1.00 (.-.) | . | 0.04 | 0.85 | 0.26 | 777 | 64,16 | 354 | 66,29 | 1473 | 414 |  |
| DNMT3B ---- tag |  | C/T | 0.90 (0.73-1.12) | 0.35 | . | . | . | 381 | 31,46 | 167 | 31,27 | 1473 | 414 |  |
| DNMT3B ---- tag |  | T/T | 0.46 (0.23-0.89) | 0.02 | . | . | . | 53 | 4,38 | 13 | 2,43 | 1473 | 414 |  |
| DNMT3B ---- candidate literature | rs2424909 | T/T | 1.00 (.-.) | . | 0.15 | 0.86 | 0.32 | 456 | 37,65 | 218 | 40,82 | 1473 | 414 |  |
| DNMT3B ---- candidate literature |  | T/C | 0.94 (0.76-1.16) | 0.58 | . | . | . | 576 | 47,56 | 240 | 44,94 | 1473 | 414 |  |
| DNMT3B ---- candidate literature |  | C/C | 0.79 (0.59-1.07) | 0.13 | . | . | . | 179 | 14,78 | 76 | 14,23 | 1473 | 414 |  |
| DNMT3B ---- tag | rs4911108 | A/A | 1.00 (.-.) | . | 0.16 | 0.86 | 0.32 | 492 | 40,63 | 229 | 42,88 | 1473 | 414 |  |
| DNMT3B ---- tag |  | A/G | 0.94 (0.77-1.17) | 0.60 | . | . | . | 553 | 45,66 | 236 | 44,19 | 1473 | 414 |  |
| DNMT3B ---- tag |  | G/G | 0.79 (0.58-1.08) | 0.14 | . | . | . | 166 | 13,71 | 69 | 12,92 | 1473 | 414 |  |
| DNMT3B ---- tag | rs6058896 | C/C | 1.00 (.-.) | . | 0.32 | 0.94 | 0.48 | 1075 | 88,77 | 477 | 89,33 | 1473 | 414 | low_count |
| DNMT3B ---- tag |  | C/T | 1.14 (0.83-1.56) | 0.42 | . | . | . | 132 | 10,90 | 54 | 10,11 | 1473 | 414 |  |
| DNMT3B ---- tag |  | T/T | 1.70 (0.42-6.89) | 0.46 | . | . | . | 4 | 0,33 | 3 | 0,56 | 1473 | 414 |  |
| DNMT3B ---- tag | rs6119954 | G/G | 1.00 (.-.) | . | 0.75 | 0.98 | 0.84 | 836 | 69,03 | 371 | 69,48 | 1473 | 414 |  |
| DNMT3B ---- tag |  | G/A | 1.06 (0.85-1.32) | 0.62 | . | . | . | 340 | 28,08 | 144 | 26,97 | 1473 | 414 |  |
| DNMT3B ---- tag |  | A/A | 0.98 (0.57-1.68) | 0.93 | . | . | . | 35 | 2,89 | 19 | 3,56 | 1473 | 414 |  |
| DNMT3B ---- tag | rs6579038 | A/A | 1.00 (.-.) | . | 0.33 | 0.94 | 0.48 | 1067 | 88,11 | 476 | 89,14 | 1473 | 414 | low_count |
| DNMT3B ---- tag |  | A/G | 1.12 (0.82-1.53) | 0.49 | . | . | . | 141 | 11,64 | 56 | 10,49 | 1473 | 414 |  |
| DNMT3B ---- tag |  | G/G | 2.08 (0.51-8.45) | 0.31 | . | . | . | 3 | 0,25 | 2 | 0,37 | 1473 | 414 |  |
| DPYD ---- tag | rs1034215 | C/C | 1.00 (.-.) | . | 0.50 | 0.96 | 0.72 | 744 | 61,44 | 331 | 61,99 | 1473 | 414 |  |
| DPYD ---- tag |  | C/T | 0.99 (0.80-1.22) | 0.89 | . | . | . | 402 | 33,20 | 170 | 31,84 | 1473 | 414 |  |
| DPYD ---- tag |  | T/T | 1.32 (0.86-2.01) | 0.20 | . | . | . | 65 | 5,37 | 33 | 6,18 | 1473 | 414 |  |
| DPYD ---- tag | rs10783058 | T/T | 1.00 (.-.) | . | 0.32 | 0.94 | 0.68 | 521 | 43,02 | 210 | 39,33 | 1473 | 414 |  |
| DPYD ---- tag |  | T/C | 1.22 (0.98-1.50) | 0.07 | . | . | . | 538 | 44,43 | 255 | 47,75 | 1473 | 414 |  |
| DPYD ---- tag |  | C/C | 1.06 (0.78-1.45) | 0.69 | . | . | . | 152 | 12,55 | 69 | 12,92 | 1473 | 414 |  |
| DPYD ---- tag | rs10783070 | C/C | 1.00 (.-.) | . | 0.03 | 0.85 | 0.37 | 851 | 70,27 | 371 | 69,48 | 1473 | 414 |  |
| DPYD ---- tag |  | C/T | 1.26 (1.01-1.56) | 0.04 | . | . | . | 333 | 27,50 | 148 | 27,72 | 1473 | 414 |  |
| DPYD ---- tag |  | T/T | 1.35 (0.72-2.56) | 0.35 | . | . | . | 27 | 2,23 | 15 | 2,81 | 1473 | 414 |  |
| DPYD ---- tag | rs10875048 | G/G | 1.00 (.-.) | . | 0.63 | 0.97 | 0.74 | 806 | 66,56 | 354 | 66,29 | 1473 | 414 |  |
| DPYD ---- tag |  | G/A | 0.95 (0.77-1.17) | 0.62 | . | . | . | 368 | 30,39 | 163 | 30,52 | 1473 | 414 |  |
| DPYD ---- tag |  | A/A | 0.95 (0.53-1.70) | 0.85 | . | . | . | 37 | 3,06 | 17 | 3,18 | 1473 | 414 |  |
| DPYD ---- tag | rs10875055 | C/C | 1.00 (.-.) | . | 0.08 | 0.85 | 0.42 | 358 | 29,56 | 126 | 23,60 | 1473 | 414 |  |
| DPYD ---- tag |  | C/T | 1.30 (1.02-1.66) | 0.03 | . | . | . | 615 | 50,78 | 277 | 51,87 | 1473 | 414 |  |
| DPYD ---- tag |  | T/T | 1.28 (0.96-1.71) | 0.09 | . | . | . | 238 | 19,65 | 131 | 24,53 | 1473 | 414 |  |
| DPYD ---- tag | rs10875079 | A/A | 1.00 (.-.) | . | 0.01 | 0.85 | 0.37 | 311 | 25,68 | 150 | 28,09 | 1473 | 414 |  |
| DPYD ---- tag |  | A/G | 0.85 (0.67-1.06) | 0.15 | . | . | . | 590 | 48,72 | 268 | 50,19 | 1473 | 414 |  |
| DPYD ---- tag |  | G/G | 0.70 (0.53-0.92) | 0.01 | . | . | . | 310 | 25,60 | 116 | 21,72 | 1473 | 414 |  |
| DPYD ---- tag | rs10875085 | A/A | 1.00 (.-.) | . | 0.25 | 0.90 | 0.64 | 832 | 68,70 | 351 | 65,73 | 1473 | 414 |  |
| DPYD ---- tag |  | A/T | 1.03 (0.83-1.28) | 0.80 | . | . | . | 351 | 28,98 | 162 | 30,34 | 1473 | 414 |  |
| DPYD ---- tag |  | T/T | 1.57 (0.96-2.58) | 0.07 | . | . | . | 28 | 2,31 | 21 | 3,93 | 1473 | 414 |  |
| DPYD ---- tag | rs10875097 | G/G | 1.00 (.-.) | . | 0.26 | 0.90 | 0.64 | 843 | 69,61 | 354 | 66,29 | 1473 | 414 |  |
| DPYD ---- tag |  | G/A | 1.22 (0.98-1.51) | 0.08 | . | . | . | 328 | 27,09 | 164 | 30,71 | 1473 | 414 |  |
| DPYD ---- tag |  | A/A | 0.88 (0.48-1.61) | 0.67 | . | . | . | 40 | 3,30 | 16 | 3,00 | 1473 | 414 |  |
| DPYD ---- tag | rs11165781 | T/T | 1.00 (.-.) | . | 0.96 | 0.99 | 0.96 | 819 | 67,63 | 364 | 68,16 | 1473 | 414 |  |
| DPYD ---- tag |  | T/C | 1.01 (0.82-1.26) | 0.90 | . | . | . | 356 | 29,40 | 159 | 29,78 | 1473 | 414 |  |
| DPYD ---- tag |  | C/C | 0.95 (0.45-2.03) | 0.90 | . | . | . | 36 | 2,97 | 11 | 2,06 | 1473 | 414 |  |
| DPYD ---- tag | rs11165783 | T/T | 1.00 (.-.) | . | 0.41 | 0.94 | 0.72 | 667 | 55,08 | 294 | 55,06 | 1473 | 414 |  |
| DPYD ---- tag |  | T/C | 1.04 (0.85-1.28) | 0.70 | . | . | . | 458 | 37,82 | 194 | 36,33 | 1473 | 414 |  |
| DPYD ---- tag |  | C/C | 1.17 (0.82-1.67) | 0.38 | . | . | . | 86 | 7,10 | 46 | 8,61 | 1473 | 414 |  |
| DPYD ---- tag | rs11165873 | A/A | 1.00 (.-.) | . | 0.12 | 0.86 | 0.56 | 319 | 26,34 | 165 | 30,90 | 1473 | 414 |  |
| DPYD ---- tag |  | A/T | 0.81 (0.65-1.02) | 0.07 | . | . | . | 618 | 51,03 | 254 | 47,57 | 1473 | 414 |  |
| DPYD ---- tag |  | T/T | 0.82 (0.62-1.08) | 0.16 | . | . | . | 274 | 22,63 | 115 | 21,54 | 1473 | 414 |  |
| DPYD ---- tag | rs11165875 | T/T | 1.00 (.-.) | . | 0.16 | 0.86 | 0.57 | 498 | 41,12 | 200 | 37,45 | 1473 | 414 |  |
| DPYD ---- tag |  | T/C | 1.09 (0.88-1.35) | 0.42 | . | . | . | 560 | 46,24 | 248 | 46,44 | 1473 | 414 |  |
| DPYD ---- tag |  | C/C | 1.23 (0.92-1.63) | 0.17 | . | . | . | 153 | 12,63 | 86 | 16,10 | 1473 | 414 |  |
| DPYD ---- tag | rs11165881 | T/T | 1.00 (.-.) | . | 0.70 | 0.98 | 0.80 | 428 | 35,34 | 193 | 36,14 | 1473 | 414 |  |
| DPYD ---- tag |  | T/C | 1.08 (0.87-1.34) | 0.49 | . | . | . | 564 | 46,57 | 255 | 47,75 | 1473 | 414 |  |
| DPYD ---- tag |  | C/C | 1.03 (0.77-1.38) | 0.83 | . | . | . | 219 | 18,08 | 86 | 16,10 | 1473 | 414 |  |
| DPYD ---- tag | rs11587873 | C/C | 1.00 (.-.) | . | 0.03 | 0.85 | 0.37 | 670 | 55,33 | 328 | 61,42 | 1473 | 414 |  |
| DPYD ---- tag |  | C/T | 0.91 (0.74-1.12) | 0.37 | . | . | . | 467 | 38,56 | 191 | 35,77 | 1473 | 414 |  |
| DPYD ---- tag |  | T/T | 0.45 (0.24-0.84) | 0.01 | . | . | . | 74 | 6,11 | 15 | 2,81 | 1473 | 414 |  |
| DPYD ---- tag | rs12030174 | C/C | 1.00 (.-.) | . | 0.45 | 0.95 | 0.72 | 886 | 73,16 | 375 | 70,22 | 1473 | 414 |  |
| DPYD ---- tag |  | C/T | 0.99 (0.79-1.24) | 0.94 | . | . | . | 304 | 25,10 | 145 | 27,15 | 1473 | 414 |  |
| DPYD ---- tag |  | T/T | 1.75 (0.95-3.22) | 0.07 | . | . | . | 21 | 1,73 | 14 | 2,62 | 1473 | 414 |  |
| DPYD ---- tag | rs12046744 | A/A | 1.00 (.-.) | . | 0.25 | 0.90 | 0.64 | 644 | 53,18 | 298 | 55,81 | 1473 | 414 |  |
| DPYD ---- tag |  | A/C | 1.03 (0.84-1.27) | 0.76 | . | . | . | 477 | 39,39 | 201 | 37,64 | 1473 | 414 |  |
| DPYD ---- tag |  | C/C | 0.67 (0.44-1.02) | 0.06 | . | . | . | 90 | 7,43 | 35 | 6,55 | 1473 | 414 |  |
| DPYD ---- tag | rs12047910 | G/G | 1.00 (.-.) | . | 0.56 | 0.97 | 0.72 | 875 | 72,25 | 391 | 73,22 | 1473 | 414 | low_count |
| DPYD ---- tag |  | G/A | 0.93 (0.74-1.16) | 0.49 | . | . | . | 309 | 25,52 | 133 | 24,91 | 1473 | 414 |  |
| DPYD ---- tag |  | A/A | 1.01 (0.47-2.15) | 0.99 | . | . | . | 27 | 2,23 | 10 | 1,87 | 1473 | 414 |  |
| DPYD ---- tag | rs12073044 | T/T | 1.00 (.-.) | . | 0.44 | 0.94 | 0.72 | 969 | 80,02 | 419 | 78,46 | 1473 | 414 | low_count |
| DPYD ---- tag |  | T/A | 1.13 (0.89-1.44) | 0.30 | . | . | . | 229 | 18,91 | 113 | 21,16 | 1473 | 414 |  |
| DPYD ---- tag |  | A/A | 0.52 (0.07-3.71) | 0.51 | . | . | . | 13 | 1,07 | 2 | 0,37 | 1473 | 414 |  |
| DPYD ---- tag | rs12126093 | T/T | 1.00 (.-.) | . | 0.15 | 0.86 | 0.57 | 599 | 49,46 | 282 | 52,81 | 1473 | 414 |  |
| DPYD ---- tag |  | T/C | 0.84 (0.68-1.04) | 0.10 | . | . | . | 513 | 42,36 | 203 | 38,01 | 1473 | 414 |  |
| DPYD ---- tag |  | C/C | 0.87 (0.60-1.26) | 0.46 | . | . | . | 99 | 8,18 | 49 | 9,18 | 1473 | 414 |  |
| DPYD ---- tag | rs12134028 | C/C | 1.00 (.-.) | . | 0.13 | 0.86 | 0.57 | 1088 | 89,84 | 477 | 89,33 | 1473 | 414 | low_count |
| DPYD ---- tag |  | C/T | 0.76 (0.54-1.06) | 0.11 | . | . | . | 121 | 9,99 | 55 | 10,30 | 1473 | 414 |  |
| DPYD ---- tag |  | T/T | 1.12 (0.15-8.23) | 0.91 | . | . | . | 2 | 0,17 | 2 | 0,37 | 1473 | 414 |  |
| DPYD ---- tag | rs12740796 | T/T | 1.00 (.-.) | . | 0.55 | 0.97 | 0.72 | 906 | 74,81 | 400 | 74,91 | 1473 | 414 |  |
| DPYD ---- tag |  | T/C | 1.04 (0.83-1.31) | 0.72 | . | . | . | 283 | 23,37 | 122 | 22,85 | 1473 | 414 |  |
| DPYD ---- tag |  | C/C | 1.24 (0.63-2.43) | 0.53 | . | . | . | 22 | 1,82 | 12 | 2,25 | 1473 | 414 |  |
| DPYD ---- tag | rs1333717 | A/A | 1.00 (.-.) | . | 0.47 | 0.95 | 0.72 | 706 | 58,30 | 316 | 59,18 | 1473 | 414 |  |
| DPYD ---- tag |  | A/G | 0.97 (0.79-1.20) | 0.80 | . | . | . | 433 | 35,76 | 182 | 34,08 | 1473 | 414 |  |
| DPYD ---- tag |  | G/G | 1.35 (0.91-2.03) | 0.14 | . | . | . | 72 | 5,95 | 36 | 6,74 | 1473 | 414 |  |
| DPYD ---- tag | rs1413228 | A/A | 1.00 (.-.) | . | 0.55 | 0.97 | 0.72 | 965 | 79,69 | 429 | 80,34 | 1473 | 414 | low_count |
| DPYD ---- tag |  | A/G | 0.95 (0.74-1.23) | 0.71 | . | . | . | 236 | 19,49 | 99 | 18,54 | 1473 | 414 |  |
| DPYD ---- tag |  | G/G | 0.74 (0.27-2.00) | 0.55 | . | . | . | 10 | 0,83 | 6 | 1,12 | 1473 | 414 |  |
| DPYD ---- tag | rs1415681 | G/G | 1.00 (.-.) | . | 0.70 | 0.98 | 0.80 | 900 | 74,32 | 407 | 76,22 | 1473 | 414 |  |
| DPYD ---- tag |  | G/T | 1.01 (0.80-1.27) | 0.95 | . | . | . | 279 | 23,04 | 113 | 21,16 | 1473 | 414 |  |
| DPYD ---- tag |  | T/T | 1.20 (0.67-2.15) | 0.54 | . | . | . | 32 | 2,64 | 14 | 2,62 | 1473 | 414 |  |
| DPYD ---- tag | rs1514495 | C/C | 1.00 (.-.) | . | 0.47 | 0.95 | 0.72 | 699 | 57,72 | 319 | 59,74 | 1473 | 414 |  |
| DPYD ---- tag |  | C/T | 0.98 (0.80-1.21) | 0.85 | . | . | . | 447 | 36,91 | 188 | 35,21 | 1473 | 414 |  |
| DPYD ---- tag |  | T/T | 0.81 (0.51-1.27) | 0.36 | . | . | . | 65 | 5,37 | 27 | 5,06 | 1473 | 414 |  |
| DPYD ---- tag | rs1520658 | A/A | 1.00 (.-.) | . | 0.49 | 0.96 | 0.72 | 960 | 79,27 | 419 | 78,46 | 1473 | 414 | low_count |
| DPYD ---- tag |  | A/G | 1.12 (0.88-1.42) | 0.36 | . | . | . | 237 | 19,57 | 108 | 20,22 | 1473 | 414 |  |
| DPYD ---- tag |  | G/G | 0.86 (0.32-2.33) | 0.77 | . | . | . | 14 | 1,16 | 7 | 1,31 | 1473 | 414 |  |
| DPYD ---- NA | rs17116806 | C/C | 1.00 (.-.) | . | 0.62 | 0.97 | 0.74 | 798 | 65,90 | 335 | 62,73 | 1473 | 414 |  |
| DPYD ---- NA |  | C/A | 1.16 (0.94-1.43) | 0.16 | . | . | . | 367 | 30,31 | 183 | 34,27 | 1473 | 414 |  |
| DPYD ---- NA |  | A/A | 0.75 (0.42-1.35) | 0.34 | . | . | . | 46 | 3,80 | 16 | 3,00 | 1473 | 414 |  |
| DPYD ---- tag | rs17431828 | G/G | 1.00 (.-.) | . | 0.89 | 0.99 | 0.94 | 497 | 41,04 | 232 | 43,45 | 1473 | 414 |  |
| DPYD ---- tag |  | G/C | 1.00 (0.81-1.23) | 0.99 | . | . | . | 560 | 46,24 | 232 | 43,45 | 1473 | 414 |  |
| DPYD ---- tag |  | C/C | 0.97 (0.71-1.33) | 0.86 | . | . | . | 154 | 12,72 | 70 | 13,11 | 1473 | 414 |  |
| DPYD ---- tag | rs17471640 | T/T | 1.00 (.-.) | . | 0.52 | 0.97 | 0.72 | 559 | 46,16 | 252 | 47,19 | 1473 | 414 |  |
| DPYD ---- tag |  | T/C | 0.91 (0.74-1.12) | 0.36 | . | . | . | 539 | 44,51 | 224 | 41,95 | 1473 | 414 |  |
| DPYD ---- tag |  | C/C | 0.96 (0.68-1.37) | 0.84 | . | . | . | 113 | 9,33 | 58 | 10,86 | 1473 | 414 |  |
| DPYD ---- tag | rs17702702 | G/G | 1.00 (.-.) | . | 0.96 | 0.99 | 0.96 | 839 | 69,28 | 371 | 69,48 | 1473 | 414 |  |
| DPYD ---- tag |  | G/C | 1.00 (0.80-1.24) | 0.98 | . | . | . | 335 | 27,66 | 146 | 27,34 | 1473 | 414 |  |
| DPYD ---- tag |  | C/C | 1.04 (0.60-1.79) | 0.89 | . | . | . | 37 | 3,06 | 17 | 3,18 | 1473 | 414 |  |
| DPYD ---- NA | rs1801265 | T/T | 1.00 (.-.) | . | 0.25 | 0.90 | 0.64 | 703 | 58,05 | 311 | 58,24 | 1473 | 414 |  |
| DPYD ---- NA |  | T/C | 1.19 (0.97-1.46) | 0.10 | . | . | . | 434 | 35,84 | 195 | 36,52 | 1473 | 414 |  |
| DPYD ---- NA |  | C/C | 1.00 (0.62-1.60) | 0.99 | . | . | . | 74 | 6,11 | 28 | 5,24 | 1473 | 414 |  |
| DPYD ---- tag | rs2039447 | T/T | 1.00 (.-.) | . | 0.33 | 0.94 | 0.68 | 558 | 46,08 | 251 | 47,00 | 1473 | 414 |  |
| DPYD ---- tag |  | T/C | 1.19 (0.97-1.45) | 0.10 | . | . | . | 537 | 44,34 | 232 | 43,45 | 1473 | 414 |  |
| DPYD ---- tag |  | C/C | 1.02 (0.71-1.47) | 0.90 | . | . | . | 116 | 9,58 | 51 | 9,55 | 1473 | 414 |  |
| DPYD ---- tag | rs2151567 | G/G | 1.00 (.-.) | . | 0.04 | 0.85 | 0.37 | 1096 | 90,50 | 476 | 89,14 | 1473 | 414 | low_count |
| DPYD ---- tag |  | G/A | 1.35 (0.99-1.85) | 0.06 | . | . | . | 112 | 9,25 | 57 | 10,67 | 1473 | 414 |  |
| DPYD ---- tag |  | A/A | 2.52 (0.35-18.23) | 0.36 | . | . | . | 3 | 0,25 | 1 | 0,19 | 1473 | 414 |  |
| DPYD ---- tag | rs2152878 | A/A | 1.00 (.-.) | . | 0.38 | 0.94 | 0.72 | 705 | 58,22 | 311 | 58,24 | 1473 | 414 |  |
| DPYD ---- tag |  | A/G | 1.03 (0.83-1.27) | 0.79 | . | . | . | 430 | 35,51 | 188 | 35,21 | 1473 | 414 |  |
| DPYD ---- tag |  | G/G | 1.25 (0.84-1.86) | 0.27 | . | . | . | 76 | 6,28 | 35 | 6,55 | 1473 | 414 |  |
| DPYD ---- tag | rs2786505 | G/G | 1.00 (.-.) | . | 0.04 | 0.85 | 0.37 | 906 | 74,81 | 396 | 74,16 | 1473 | 414 | low_count |
| DPYD ---- tag |  | G/T | 1.23 (0.98-1.54) | 0.07 | . | . | . | 293 | 24,19 | 129 | 24,16 | 1473 | 414 |  |
| DPYD ---- tag |  | T/T | 1.72 (0.76-3.88) | 0.19 | . | . | . | 12 | 0,99 | 9 | 1,69 | 1473 | 414 |  |
| DPYD ---- tag | rs2786512 | G/G | 1.00 (.-.) | . | 0.54 | 0.97 | 0.72 | 447 | 36,91 | 201 | 37,64 | 1473 | 414 |  |
| DPYD ---- tag |  | G/A | 1.05 (0.84-1.30) | 0.69 | . | . | . | 534 | 44,10 | 248 | 46,44 | 1473 | 414 |  |
| DPYD ---- tag |  | A/A | 0.89 (0.67-1.18) | 0.41 | . | . | . | 230 | 18,99 | 85 | 15,92 | 1473 | 414 |  |
| DPYD ---- tag | rs2786519 | A/A | 1.00 (.-.) | . | 0.58 | 0.97 | 0.73 | 742 | 61,27 | 310 | 58,05 | 1473 | 414 |  |
| DPYD ---- tag |  | A/G | 1.16 (0.94-1.42) | 0.17 | . | . | . | 401 | 33,11 | 194 | 36,33 | 1473 | 414 |  |
| DPYD ---- tag |  | G/G | 0.88 (0.56-1.39) | 0.59 | . | . | . | 68 | 5,62 | 30 | 5,62 | 1473 | 414 |  |
| DPYD ---- tag | rs2811170 | A/A | 1.00 (.-.) | . | 0.18 | 0.86 | 0.57 | 903 | 74,57 | 416 | 77,90 | 1473 | 414 | low_count |
| DPYD ---- tag |  | A/T | 0.80 (0.62-1.02) | 0.07 | . | . | . | 292 | 24,11 | 112 | 20,97 | 1473 | 414 |  |
| DPYD ---- tag |  | T/T | 1.53 (0.63-3.75) | 0.35 | . | . | . | 16 | 1,32 | 6 | 1,12 | 1473 | 414 |  |
| DPYD ---- tag | rs2811199 | G/G | 1.00 (.-.) | . | 0.04 | 0.85 | 0.37 | 869 | 71,76 | 381 | 71,35 | 1473 | 414 |  |
| DPYD ---- tag |  | G/A | 1.24 (1.00-1.55) | 0.05 | . | . | . | 319 | 26,34 | 140 | 26,22 | 1473 | 414 |  |
| DPYD ---- tag |  | A/A | 1.33 (0.65-2.70) | 0.43 | . | . | . | 23 | 1,90 | 13 | 2,43 | 1473 | 414 |  |
| DPYD ---- tag | rs2811219 | T/T | 1.00 (.-.) | . | 0.88 | 0.99 | 0.94 | 695 | 57,39 | 313 | 58,61 | 1473 | 414 |  |
| DPYD ---- tag |  | T/C | 0.93 (0.76-1.15) | 0.53 | . | . | . | 436 | 36,00 | 192 | 35,96 | 1473 | 414 |  |
| DPYD ---- tag |  | C/C | 1.12 (0.72-1.73) | 0.62 | . | . | . | 80 | 6,61 | 29 | 5,43 | 1473 | 414 |  |
| DPYD ---- tag | rs4300257 | A/A | 1.00 (.-.) | . | 0.15 | 0.86 | 0.57 | 787 | 64,99 | 325 | 60,86 | 1473 | 414 |  |
| DPYD ---- tag |  | A/C | 1.00 (0.81-1.23) | 0.97 | . | . | . | 385 | 31,79 | 182 | 34,08 | 1473 | 414 |  |
| DPYD ---- tag |  | C/C | 1.95 (1.24-3.07) | 0.00 | . | . | . | 39 | 3,22 | 27 | 5,06 | 1473 | 414 |  |
| DPYD ---- tag | rs4379706 | T/T | 1.00 (.-.) | . | 0.32 | 0.94 | 0.68 | 689 | 56,90 | 310 | 58,05 | 1473 | 414 |  |
| DPYD ---- tag |  | T/C | 1.18 (0.96-1.45) | 0.11 | . | . | . | 443 | 36,58 | 195 | 36,52 | 1473 | 414 |  |
| DPYD ---- tag |  | C/C | 0.97 (0.61-1.54) | 0.90 | . | . | . | 79 | 6,52 | 29 | 5,43 | 1473 | 414 |  |
| DPYD ---- tag | rs4950021 | T/T | 1.00 (.-.) | . | 0.56 | 0.97 | 0.72 | 363 | 29,98 | 170 | 31,84 | 1473 | 414 |  |
| DPYD ---- tag |  | T/G | 0.95 (0.76-1.19) | 0.64 | . | . | . | 610 | 50,37 | 240 | 44,94 | 1473 | 414 |  |
| DPYD ---- tag |  | G/G | 1.10 (0.84-1.45) | 0.47 | . | . | . | 238 | 19,65 | 124 | 23,22 | 1473 | 414 |  |
| DPYD ---- tag | rs4950033 | T/T | 1.00 (.-.) | . | 0.33 | 0.94 | 0.68 | 349 | 28,82 | 157 | 29,40 | 1473 | 414 |  |
| DPYD ---- tag |  | T/C | 0.98 (0.78-1.23) | 0.86 | . | . | . | 617 | 50,95 | 255 | 47,75 | 1473 | 414 |  |
| DPYD ---- tag |  | C/C | 1.15 (0.88-1.51) | 0.30 | . | . | . | 245 | 20,23 | 122 | 22,85 | 1473 | 414 |  |
| DPYD ---- tag | rs495257 | T/T | 1.00 (.-.) | . | 0.44 | 0.94 | 0.72 | 430 | 35,51 | 177 | 33,15 | 1473 | 414 |  |
| DPYD ---- tag |  | T/C | 1.16 (0.93-1.44) | 0.19 | . | . | . | 582 | 48,06 | 282 | 52,81 | 1473 | 414 |  |
| DPYD ---- tag |  | C/C | 1.07 (0.79-1.45) | 0.67 | . | . | . | 199 | 16,43 | 75 | 14,04 | 1473 | 414 |  |
| DPYD ---- tag | rs552926 | A/A | 1.00 (.-.) | . | 0.73 | 0.98 | 0.81 | 446 | 36,83 | 179 | 33,52 | 1473 | 414 |  |
| DPYD ---- tag |  | A/G | 1.16 (0.92-1.44) | 0.21 | . | . | . | 575 | 47,48 | 263 | 49,25 | 1473 | 414 |  |
| DPYD ---- tag |  | G/G | 1.01 (0.76-1.35) | 0.94 | . | . | . | 190 | 15,69 | 92 | 17,23 | 1473 | 414 |  |
| DPYD ---- tag | rs628959 | A/A | 1.00 (.-.) | . | 0.39 | 0.94 | 0.72 | 593 | 48,97 | 270 | 50,56 | 1473 | 414 |  |
| DPYD ---- tag |  | A/G | 1.07 (0.87-1.31) | 0.52 | . | . | . | 529 | 43,68 | 214 | 40,07 | 1473 | 414 |  |
| DPYD ---- tag |  | G/G | 1.14 (0.79-1.62) | 0.48 | . | . | . | 89 | 7,35 | 50 | 9,36 | 1473 | 414 |  |
| DPYD ---- tag | rs6656660 | G/G | 1.00 (.-.) | . | 0.92 | 0.99 | 0.96 | 915 | 75,56 | 407 | 76,22 | 1473 | 414 |  |
| DPYD ---- tag |  | G/T | 0.99 (0.78-1.26) | 0.93 | . | . | . | 279 | 23,04 | 114 | 21,35 | 1473 | 414 |  |
| DPYD ---- tag |  | T/T | 1.10 (0.60-2.03) | 0.76 | . | . | . | 17 | 1,40 | 13 | 2,43 | 1473 | 414 |  |
| DPYD ---- tag | rs6663670 | A/A | 1.00 (.-.) | . | 0.04 | 0.85 | 0.37 | 872 | 72,01 | 382 | 71,54 | 1473 | 414 |  |
| DPYD ---- tag |  | A/C | 1.25 (1.00-1.55) | 0.05 | . | . | . | 317 | 26,18 | 139 | 26,03 | 1473 | 414 |  |
| DPYD ---- tag |  | C/C | 1.33 (0.65-2.70) | 0.44 | . | . | . | 22 | 1,82 | 13 | 2,43 | 1473 | 414 |  |
| DPYD ---- tag | rs6683883 | T/T | 1.00 (.-.) | . | 0.05 | 0.85 | 0.37 | 484 | 39,97 | 183 | 34,27 | 1473 | 414 |  |
| DPYD ---- tag |  | T/C | 1.11 (0.89-1.38) | 0.36 | . | . | . | 569 | 46,99 | 250 | 46,82 | 1473 | 414 |  |
| DPYD ---- tag |  | C/C | 1.33 (1.01-1.77) | 0.05 | . | . | . | 158 | 13,05 | 101 | 18,91 | 1473 | 414 |  |
| DPYD ---- tag | rs6686861 | C/C | 1.00 (.-.) | . | 0.59 | 0.97 | 0.73 | 1051 | 86,79 | 454 | 85,02 | 1473 | 414 | low_count |
| DPYD ---- tag |  | C/T | 1.09 (0.83-1.44) | 0.53 | . | . | . | 156 | 12,88 | 76 | 14,23 | 1473 | 414 |  |
| DPYD ---- tag |  | T/T | 0.91 (0.22-3.71) | 0.90 | . | . | . | 4 | 0,33 | 4 | 0,75 | 1473 | 414 |  |
| DPYD ---- tag | rs7414210 | A/A | 1.00 (.-.) | . | 0.43 | 0.94 | 0.72 | 855 | 70,60 | 392 | 73,41 | 1473 | 414 | low_count |
| DPYD ---- tag |  | A/C | 1.02 (0.82-1.27) | 0.87 | . | . | . | 317 | 26,18 | 136 | 25,47 | 1473 | 414 |  |
| DPYD ---- tag |  | C/C | 0.35 (0.11-1.10) | 0.07 | . | . | . | 39 | 3,22 | 6 | 1,12 | 1473 | 414 |  |
| DPYD ---- tag | rs7530858 | A/A | 1.00 (.-.) | . | 0.26 | 0.90 | 0.64 | 950 | 78,45 | 412 | 77,15 | 1473 | 414 | low_count |
| DPYD ---- tag |  | A/G | 1.16 (0.92-1.46) | 0.22 | . | . | . | 246 | 20,31 | 117 | 21,91 | 1473 | 414 |  |
| DPYD ---- tag |  | G/G | 1.00 (0.32-3.14) | 1.00 | . | . | . | 15 | 1,24 | 5 | 0,94 | 1473 | 414 |  |
| DPYD ---- tag | rs7544128 | C/C | 1.00 (.-.) | . | 0.17 | 0.86 | 0.57 | 663 | 54,75 | 275 | 51,50 | 1473 | 414 |  |
| DPYD ---- tag |  | C/G | 1.05 (0.85-1.29) | 0.67 | . | . | . | 470 | 38,81 | 216 | 40,45 | 1473 | 414 |  |
| DPYD ---- tag |  | G/G | 1.38 (0.95-2.01) | 0.09 | . | . | . | 78 | 6,44 | 43 | 8,05 | 1473 | 414 |  |
| DPYD ---- tag | rs7545340 | G/G | 1.00 (.-.) | . | 0.79 | 0.99 | 0.87 | 666 | 55,00 | 282 | 52,81 | 1473 | 414 |  |
| DPYD ---- tag |  | G/A | 1.07 (0.87-1.31) | 0.55 | . | . | . | 455 | 37,57 | 213 | 39,89 | 1473 | 414 |  |
| DPYD ---- tag |  | A/A | 0.98 (0.67-1.42) | 0.90 | . | . | . | 90 | 7,43 | 39 | 7,30 | 1473 | 414 |  |
| DPYD ---- tag | rs828054 | A/A | 1.00 (.-.) | . | 0.07 | 0.85 | 0.41 | 273 | 22,54 | 152 | 28,46 | 1473 | 414 |  |
| DPYD ---- tag |  | A/C | 0.96 (0.76-1.21) | 0.72 | . | . | . | 617 | 50,95 | 270 | 50,56 | 1473 | 414 |  |
| DPYD ---- tag |  | C/C | 0.77 (0.58-1.02) | 0.07 | . | . | . | 321 | 26,51 | 112 | 20,97 | 1473 | 414 |  |
| DPYD ---- tag | rs885622 | G/G | 1.00 (.-.) | . | 0.06 | 0.85 | 0.37 | 505 | 41,70 | 190 | 35,58 | 1473 | 414 |  |
| DPYD ---- tag |  | G/A | 1.21 (0.98-1.50) | 0.08 | . | . | . | 563 | 46,49 | 256 | 47,94 | 1473 | 414 |  |
| DPYD ---- tag |  | A/A | 1.27 (0.95-1.72) | 0.11 | . | . | . | 143 | 11,81 | 88 | 16,48 | 1473 | 414 |  |
| DPYD ---- tag | rs9437663 | G/G | 1.00 (.-.) | . | 0.23 | 0.90 | 0.64 | 758 | 62,59 | 342 | 64,04 | 1473 | 414 |  |
| DPYD ---- tag |  | G/A | 1.05 (0.85-1.31) | 0.65 | . | . | . | 406 | 33,53 | 166 | 31,09 | 1473 | 414 |  |
| DPYD ---- tag |  | A/A | 1.42 (0.89-2.25) | 0.14 | . | . | . | 47 | 3,88 | 26 | 4,87 | 1473 | 414 |  |
| DPYS ---- tag | rs13249169 | A/A | 1.00 (.-.) | . | 0.95 | 0.99 | 0.99 | 960 | 79,27 | 419 | 78,46 | 1473 | 414 | low_count |
| DPYS ---- tag |  | A/T | 1.07 (0.84-1.36) | 0.60 | . | . | . | 237 | 19,57 | 111 | 20,79 | 1473 | 414 |  |
| DPYS ---- tag |  | T/T | 0.67 (0.25-1.82) | 0.43 | . | . | . | 14 | 1,16 | 4 | 0,75 | 1473 | 414 |  |
| DPYS ---- NA | rs13263121 | T/T | 1.00 (.-.) | . | 0.79 | 0.99 | 0.99 | 506 | 41,78 | 237 | 44,38 | 1473 | 414 |  |
| DPYS ---- NA |  | T/A | 0.96 (0.78-1.18) | 0.71 | . | . | . | 547 | 45,17 | 240 | 44,94 | 1473 | 414 |  |
| DPYS ---- NA |  | A/A | 0.98 (0.71-1.36) | 0.90 | . | . | . | 158 | 13,05 | 57 | 10,67 | 1473 | 414 |  |
| DPYS ---- tag | rs16871361 | T/T | 1.00 (.-.) | . | 0.47 | 0.95 | 0.99 | 1083 | 89,43 | 480 | 89,89 | 1473 | 414 | low_count |
| DPYS ---- tag |  | T/C | 0.91 (0.66-1.25) | 0.56 | . | . | . | 123 | 10,16 | 54 | 10,11 | 1473 | 414 |  |
| DPYS ---- tag |  | C/C | 0.00 (0.00-8E165) | 0.96 | . | . | . | 5 | 0,41 | 0 | 0,00 | 1473 | 414 |  |
| DPYS ---- NA | rs17245950 | T/T | 1.00 (.-.) | . | 0.61 | 0.97 | 0.99 | 935 | 77,21 | 415 | 77,72 | 1473 | 414 | low_count |
| DPYS ---- NA |  | T/A | 0.96 (0.75-1.22) | 0.72 | . | . | . | 261 | 21,55 | 115 | 21,54 | 1473 | 414 |  |
| DPYS ---- NA |  | A/A | 0.76 (0.24-2.40) | 0.64 | . | . | . | 15 | 1,24 | 4 | 0,75 | 1473 | 414 |  |
| DPYS ---- NA | rs2253336 | A/A | 1.00 (.-.) | . | 0.15 | 0.86 | 0.99 | 967 | 79,85 | 436 | 81,65 | 1473 | 414 | low_count |
| DPYS ---- NA |  | A/G | 0.87 (0.67-1.13) | 0.31 | . | . | . | 230 | 18,99 | 94 | 17,60 | 1473 | 414 |  |
| DPYS ---- NA |  | G/G | 0.43 (0.11-1.73) | 0.23 | . | . | . | 14 | 1,16 | 4 | 0,75 | 1473 | 414 |  |
| DPYS ---- tag | rs2280010 | C/C | 1.00 (.-.) | . | 0.19 | 0.86 | 0.99 | 698 | 57,64 | 290 | 54,31 | 1473 | 414 |  |
| DPYS ---- tag |  | C/T | 1.17 (0.95-1.44) | 0.13 | . | . | . | 452 | 37,32 | 212 | 39,70 | 1473 | 414 |  |
| DPYS ---- tag |  | T/T | 1.11 (0.73-1.69) | 0.61 | . | . | . | 61 | 5,04 | 32 | 5,99 | 1473 | 414 |  |
| DPYS ---- tag | rs2333874 | T/T | 1.00 (.-.) | . | 0.89 | 0.99 | 0.99 | 565 | 46,66 | 232 | 43,45 | 1473 | 414 |  |
| DPYS ---- tag |  | T/G | 1.03 (0.84-1.26) | 0.80 | . | . | . | 502 | 41,45 | 246 | 46,07 | 1473 | 414 |  |
| DPYS ---- tag |  | G/G | 0.94 (0.67-1.32) | 0.72 | . | . | . | 144 | 11,89 | 56 | 10,49 | 1473 | 414 |  |
| DPYS ---- NA | rs2669429 | C/C | 1.00 (.-.) | . | 0.39 | 0.94 | 0.99 | 374 | 30,88 | 161 | 30,15 | 1473 | 414 |  |
| DPYS ---- NA |  | C/T | 1.14 (0.90-1.43) | 0.27 | . | . | . | 624 | 51,53 | 269 | 50,37 | 1473 | 414 |  |
| DPYS ---- NA |  | T/T | 1.12 (0.84-1.48) | 0.45 | . | . | . | 213 | 17,59 | 104 | 19,48 | 1473 | 414 |  |
| DPYS ---- tag | rs2669434 | C/C | 1.00 (.-.) | . | 0.66 | 0.98 | 0.99 | 625 | 51,61 | 288 | 53,93 | 1473 | 414 |  |
| DPYS ---- tag |  | C/A | 0.88 (0.71-1.08) | 0.22 | . | . | . | 492 | 40,63 | 207 | 38,76 | 1473 | 414 |  |
| DPYS ---- tag |  | A/A | 1.10 (0.75-1.61) | 0.61 | . | . | . | 94 | 7,76 | 39 | 7,30 | 1473 | 414 |  |
| DPYS ---- tag | rs2853142 | T/T | 1.00 (.-.) | . | 0.73 | 0.98 | 0.99 | 453 | 37,41 | 213 | 39,89 | 1473 | 414 |  |
| DPYS ---- tag |  | T/C | 0.87 (0.70-1.07) | 0.19 | . | . | . | 594 | 49,05 | 252 | 47,19 | 1473 | 414 |  |
| DPYS ---- tag |  | C/C | 1.04 (0.76-1.42) | 0.79 | . | . | . | 164 | 13,54 | 69 | 12,92 | 1473 | 414 |  |
| DPYS ---- NA | rs2853145 | A/A | 1.00 (.-.) | . | 0.68 | 0.98 | 0.99 | 773 | 63,83 | 353 | 66,10 | 1473 | 414 |  |
| DPYS ---- NA |  | A/C | 0.95 (0.77-1.17) | 0.62 | . | . | . | 379 | 31,30 | 162 | 30,34 | 1473 | 414 |  |
| DPYS ---- NA |  | C/C | 0.99 (0.57-1.69) | 0.96 | . | . | . | 59 | 4,87 | 19 | 3,56 | 1473 | 414 |  |
| DPYS ---- tag | rs2853149 | G/G | 1.00 (.-.) | . | 0.78 | 0.99 | 0.99 | 343 | 28,32 | 149 | 27,90 | 1473 | 414 |  |
| DPYS ---- tag |  | G/A | 0.89 (0.71-1.12) | 0.33 | . | . | . | 615 | 50,78 | 271 | 50,75 | 1473 | 414 |  |
| DPYS ---- tag |  | A/A | 0.98 (0.74-1.30) | 0.88 | . | . | . | 253 | 20,89 | 114 | 21,35 | 1473 | 414 |  |
| DPYS ---- tag | rs2853154 | T/T | 1.00 (.-.) | . | 0.96 | 0.99 | 0.99 | 655 | 54,09 | 307 | 57,49 | 1473 | 414 |  |
| DPYS ---- tag |  | T/C | 0.93 (0.76-1.14) | 0.48 | . | . | . | 485 | 40,05 | 198 | 37,08 | 1473 | 414 |  |
| DPYS ---- tag |  | C/C | 1.17 (0.77-1.78) | 0.46 | . | . | . | 71 | 5,86 | 29 | 5,43 | 1473 | 414 |  |
| DPYS ---- tag | rs2853161 | A/A | 1.00 (.-.) | . | 0.83 | 0.99 | 0.99 | 310 | 25,60 | 130 | 24,34 | 1473 | 414 |  |
| DPYS ---- tag |  | A/G | 1.03 (0.81-1.32) | 0.80 | . | . | . | 613 | 50,62 | 296 | 55,43 | 1473 | 414 |  |
| DPYS ---- tag |  | G/G | 0.97 (0.72-1.30) | 0.82 | . | . | . | 288 | 23,78 | 108 | 20,22 | 1473 | 414 |  |
| DPYS ---- NA | rs2959024 | T/T | 1.00 (.-.) | . | 0.42 | 0.94 | 0.99 | 636 | 52,52 | 254 | 47,57 | 1473 | 414 |  |
| DPYS ---- NA |  | T/G | 1.16 (0.95-1.43) | 0.15 | . | . | . | 479 | 39,55 | 239 | 44,76 | 1473 | 414 |  |
| DPYS ---- NA |  | G/G | 0.99 (0.68-1.45) | 0.97 | . | . | . | 96 | 7,93 | 41 | 7,68 | 1473 | 414 |  |
| DPYS ---- NA | rs2959025 | A/A | 1.00 (.-.) | . | 0.51 | 0.96 | 0.99 | 515 | 42,53 | 199 | 37,27 | 1473 | 414 |  |
| DPYS ---- NA |  | A/G | 1.11 (0.90-1.38) | 0.33 | . | . | . | 535 | 44,18 | 262 | 49,06 | 1473 | 414 |  |
| DPYS ---- NA |  | G/G | 1.06 (0.78-1.43) | 0.71 | . | . | . | 161 | 13,29 | 73 | 13,67 | 1473 | 414 |  |
| DPYS ---- tag | rs2959026 | G/G | 1.00 (.-.) | . | 0.99 | 1.00 | 0.99 | 457 | 37,74 | 184 | 34,46 | 1473 | 414 |  |
| DPYS ---- tag |  | G/A | 1.18 (0.95-1.46) | 0.13 | . | . | . | 548 | 45,25 | 273 | 51,12 | 1473 | 414 |  |
| DPYS ---- tag |  | A/A | 0.91 (0.67-1.24) | 0.56 | . | . | . | 206 | 17,01 | 77 | 14,42 | 1473 | 414 |  |
| DPYS ---- NA | rs3133278 | T/T | 1.00 (.-.) | . | 0.92 | 0.99 | 0.99 | 592 | 48,89 | 271 | 50,75 | 1473 | 414 |  |
| DPYS ---- NA |  | T/C | 0.94 (0.77-1.16) | 0.58 | . | . | . | 521 | 43,02 | 223 | 41,76 | 1473 | 414 |  |
| DPYS ---- NA |  | C/C | 1.06 (0.74-1.53) | 0.75 | . | . | . | 98 | 8,09 | 40 | 7,49 | 1473 | 414 |  |
| DPYS ---- tag | rs3750187 | G/G | 1.00 (.-.) | . | 0.85 | 0.99 | 0.99 | 738 | 60,94 | 339 | 63,48 | 1473 | 414 |  |
| DPYS ---- tag |  | G/A | 0.95 (0.77-1.17) | 0.63 | . | . | . | 417 | 34,43 | 176 | 32,96 | 1473 | 414 |  |
| DPYS ---- tag |  | A/A | 1.09 (0.66-1.79) | 0.75 | . | . | . | 56 | 4,62 | 19 | 3,56 | 1473 | 414 |  |
| DPYS ---- tag | rs3793357 | T/T | 1.00 (.-.) | . | 0.74 | 0.98 | 0.99 | 1077 | 88,93 | 476 | 89,14 | 1473 | 414 | low_count |
| DPYS ---- tag |  | T/G | 1.07 (0.77-1.47) | 0.70 | . | . | . | 130 | 10,73 | 56 | 10,49 | 1473 | 414 |  |
| DPYS ---- tag |  | G/G | 0.90 (0.13-6.50) | 0.92 | . | . | . | 4 | 0,33 | 2 | 0,37 | 1473 | 414 |  |
| DPYS ---- tag | rs3793358 | G/G | 1.00 (.-.) | . | 0.52 | 0.97 | 0.99 | 896 | 73,99 | 414 | 77,53 | 1473 | 414 |  |
| DPYS ---- tag |  | G/A | 0.85 (0.67-1.09) | 0.20 | . | . | . | 293 | 24,19 | 108 | 20,22 | 1473 | 414 |  |
| DPYS ---- tag |  | A/A | 1.41 (0.72-2.75) | 0.31 | . | . | . | 22 | 1,82 | 12 | 2,25 | 1473 | 414 |  |
| DPYS ---- tag | rs6468924 | C/C | 1.00 (.-.) | . | 0.95 | 0.99 | 0.99 | 739 | 61,02 | 331 | 61,99 | 1473 | 414 |  |
| DPYS ---- tag |  | C/T | 0.95 (0.77-1.18) | 0.63 | . | . | . | 421 | 34,76 | 181 | 33,90 | 1473 | 414 |  |
| DPYS ---- tag |  | T/T | 1.20 (0.74-1.94) | 0.46 | . | . | . | 51 | 4,21 | 22 | 4,12 | 1473 | 414 |  |
| DUT ---- tag | rs8025164 | G/G | 1.00 (.-.) | . | 0.67 | 0.98 | 0.67 | 867 | 71,59 | 384 | 71,91 | 1473 | 414 |  |
| DUT ---- tag |  | G/A | 0.88 (0.70-1.11) | 0.30 | . | . | . | 307 | 25,35 | 135 | 25,28 | 1473 | 414 |  |
| DUT ---- tag |  | A/A | 1.34 (0.72-2.47) | 0.35 | . | . | . | 37 | 3,06 | 15 | 2,81 | 1473 | 414 |  |
| EHMT1 ---- tag | rs10780190 | C/C | 1.00 (.-.) | . | 0.94 | 0.99 | 0.97 | 1076 | 88,85 | 480 | 89,89 | 1473 | 414 | low_count |
| EHMT1 ---- tag |  | C/T | 0.94 (0.66-1.34) | 0.73 | . | . | . | 133 | 10,98 | 52 | 9,74 | 1473 | 414 |  |
| EHMT1 ---- tag |  | T/T | 4.50 (0.62-32.79) | 0.14 | . | . | . | 2 | 0,17 | 2 | 0,37 | 1473 | 414 |  |
| EHMT1 ---- tag | rs10867083 | G/G | 1.00 (.-.) | . | 0.45 | 0.94 | 0.94 | 537 | 44,34 | 251 | 47,00 | 1473 | 414 |  |
| EHMT1 ---- tag |  | G/A | 1.01 (0.83-1.24) | 0.89 | . | . | . | 549 | 45,33 | 233 | 43,63 | 1473 | 414 |  |
| EHMT1 ---- tag |  | A/A | 0.81 (0.56-1.16) | 0.25 | . | . | . | 125 | 10,32 | 50 | 9,36 | 1473 | 414 |  |
| EHMT1 ---- tag | rs11137190 | C/C | 1.00 (.-.) | . | 0.77 | 0.98 | 0.94 | 619 | 51,11 | 269 | 50,37 | 1473 | 414 |  |
| EHMT1 ---- tag |  | C/G | 1.15 (0.94-1.40) | 0.19 | . | . | . | 501 | 41,37 | 227 | 42,51 | 1473 | 414 |  |
| EHMT1 ---- tag |  | G/G | 0.85 (0.56-1.29) | 0.44 | . | . | . | 91 | 7,51 | 38 | 7,12 | 1473 | 414 |  |
| EHMT1 ---- tag | rs3123510 | G/G | 1.00 (.-.) | . | 0.97 | 0.99 | 0.97 | 418 | 34,52 | 190 | 35,58 | 1473 | 414 |  |
| EHMT1 ---- tag |  | G/A | 1.03 (0.83-1.27) | 0.80 | . | . | . | 586 | 48,39 | 261 | 48,88 | 1473 | 414 |  |
| EHMT1 ---- tag |  | A/A | 0.98 (0.72-1.32) | 0.87 | . | . | . | 207 | 17,09 | 83 | 15,54 | 1473 | 414 |  |
| EHMT1 ---- candidate literature | rs3125795 | G/G | 1.00 (.-.) | . | 0.74 | 0.98 | 0.94 | 1071 | 88,44 | 480 | 89,89 | 1473 | 414 | low_count |
| EHMT1 ---- candidate literature |  | G/T | 0.96 (0.68-1.35) | 0.81 | . | . | . | 137 | 11,31 | 53 | 9,93 | 1473 | 414 |  |
| EHMT1 ---- candidate literature |  | T/T | 0.00 (0.00-.) | 0.98 | . | . | . | 3 | 0,25 | 1 | 0,19 | 1473 | 414 |  |
| EHMT1 ---- tag | rs4573359 | G/G | 1.00 (.-.) | . | 0.20 | 0.86 | 0.55 | 1001 | 82,66 | 445 | 83,33 | 1473 | 414 | low_count |
| EHMT1 ---- tag |  | G/T | 0.84 (0.64-1.10) | 0.21 | . | . | . | 203 | 16,76 | 86 | 16,10 | 1473 | 414 |  |
| EHMT1 ---- tag |  | T/T | 0.77 (0.19-3.10) | 0.71 | . | . | . | 7 | 0,58 | 3 | 0,56 | 1473 | 414 |  |
| EHMT1 ---- candidate literature | rs4634736 | G/G | 1.00 (.-.) | . | 0.17 | 0.86 | 0.55 | 1000 | 82,58 | 446 | 83,52 | 1473 | 414 | low_count |
| EHMT1 ---- candidate literature |  | G/A | 0.83 (0.63-1.09) | 0.18 | . | . | . | 204 | 16,85 | 85 | 15,92 | 1473 | 414 |  |
| EHMT1 ---- candidate literature |  | A/A | 0.77 (0.19-3.10) | 0.71 | . | . | . | 7 | 0,58 | 3 | 0,56 | 1473 | 414 |  |
| EHMT1 ---- tag | rs4876902 | C/C | 1.00 (.-.) | . | 0.09 | 0.86 | 0.55 | 749 | 61,85 | 321 | 60,11 | 1473 | 414 |  |
| EHMT1 ---- tag |  | C/T | 1.19 (0.97-1.46) | 0.10 | . | . | . | 412 | 34,02 | 187 | 35,02 | 1473 | 414 |  |
| EHMT1 ---- tag |  | T/T | 1.24 (0.77-2.01) | 0.38 | . | . | . | 50 | 4,13 | 26 | 4,87 | 1473 | 414 |  |
| EHMT1 ---- tag | rs4876904 | T/T | 1.00 (.-.) | . | 0.20 | 0.86 | 0.55 | 359 | 29,64 | 164 | 30,71 | 1473 | 414 |  |
| EHMT1 ---- tag |  | T/G | 1.02 (0.81-1.27) | 0.89 | . | . | . | 607 | 50,12 | 257 | 48,13 | 1473 | 414 |  |
| EHMT1 ---- tag |  | G/G | 0.83 (0.63-1.09) | 0.17 | . | . | . | 245 | 20,23 | 113 | 21,16 | 1473 | 414 |  |
| EHMT1 ---- tag | rs7390244 | G/G | 1.00 (.-.) | . | 0.69 | 0.98 | 0.94 | 298 | 24,61 | 137 | 25,66 | 1473 | 414 |  |
| EHMT1 ---- tag |  | G/A | 1.08 (0.85-1.37) | 0.53 | . | . | . | 613 | 50,62 | 251 | 47,00 | 1473 | 414 |  |
| EHMT1 ---- tag |  | A/A | 1.06 (0.81-1.38) | 0.69 | . | . | . | 300 | 24,77 | 146 | 27,34 | 1473 | 414 |  |
| EHMT1 ---- tag | rs9314635 | G/G | 1.00 (.-.) | . | 0.74 | 0.98 | 0.94 | 487 | 40,21 | 203 | 38,01 | 1473 | 414 |  |
| EHMT1 ---- tag |  | G/T | 1.18 (0.96-1.46) | 0.11 | . | . | . | 557 | 46,00 | 270 | 50,56 | 1473 | 414 |  |
| EHMT1 ---- tag |  | T/T | 0.91 (0.64-1.29) | 0.60 | . | . | . | 167 | 13,79 | 61 | 11,42 | 1473 | 414 |  |
| EHMT2 ---- candidate/tag | rs2736428 | G/G | 1.00 (.-.) | . | 0.02 | 0.85 | 0.05 | 478 | 39,47 | 238 | 44,57 | 1473 | 414 |  |
| EHMT2 ---- candidate/tag |  | G/A | 0.83 (0.67-1.02) | 0.07 | . | . | . | 568 | 46,90 | 229 | 42,88 | 1473 | 414 |  |
| EHMT2 ---- candidate/tag |  | A/A | 0.73 (0.53-1.01) | 0.06 | . | . | . | 165 | 13,63 | 67 | 12,55 | 1473 | 414 |  |
| EHMT2 ---- tag | rs9267649 | G/G | 1.00 (.-.) | . | 0.77 | 0.98 | 0.77 | 862 | 71,18 | 381 | 71,35 | 1473 | 414 |  |
| EHMT2 ---- tag |  | G/A | 0.98 (0.78-1.22) | 0.85 | . | . | . | 315 | 26,01 | 136 | 25,47 | 1473 | 414 |  |
| EHMT2 ---- tag |  | A/A | 0.93 (0.52-1.67) | 0.81 | . | . | . | 34 | 2,81 | 17 | 3,18 | 1473 | 414 |  |
| FDXR ---- NA | rs2070918 | T/T | 1.00 (.-.) | . | 0.12 | 0.86 | 0.49 | 548 | 45,25 | 259 | 48,50 | 1473 | 414 |  |
| FDXR ---- NA |  | T/C | 1.21 (0.98-1.49) | 0.08 | . | . | . | 510 | 42,11 | 216 | 40,45 | 1473 | 414 |  |
| FDXR ---- NA |  | C/C | 1.17 (0.85-1.61) | 0.32 | . | . | . | 153 | 12,63 | 59 | 11,05 | 1473 | 414 |  |
| FDXR ---- tag | rs509911 | A/A | 1.00 (.-.) | . | 0.30 | 0.92 | 0.59 | 732 | 60,45 | 342 | 64,04 | 1473 | 414 |  |
| FDXR ---- tag |  | A/G | 1.25 (1.01-1.54) | 0.04 | . | . | . | 411 | 33,94 | 171 | 32,02 | 1473 | 414 |  |
| FDXR ---- tag |  | G/G | 0.90 (0.56-1.44) | 0.66 | . | . | . | 68 | 5,62 | 21 | 3,93 | 1473 | 414 |  |
| FDXR ---- NA | rs689882 | G/G | 1.00 (.-.) | . | 0.92 | 0.99 | 0.92 | 608 | 50,21 | 294 | 55,06 | 1473 | 414 |  |
| FDXR ---- NA |  | G/A | 0.99 (0.80-1.22) | 0.94 | . | . | . | 500 | 41,29 | 197 | 36,89 | 1473 | 414 |  |
| FDXR ---- NA |  | A/A | 0.99 (0.69-1.41) | 0.94 | . | . | . | 103 | 8,51 | 43 | 8,05 | 1473 | 414 |  |
| FDXR ---- NA | rs689895 | G/G | 1.00 (.-.) | . | 0.75 | 0.98 | 0.92 | 592 | 48,89 | 273 | 51,12 | 1473 | 414 |  |
| FDXR ---- NA |  | G/C | 1.04 (0.85-1.28) | 0.69 | . | . | . | 507 | 41,87 | 216 | 40,45 | 1473 | 414 |  |
| FDXR ---- NA |  | C/C | 0.88 (0.61-1.25) | 0.47 | . | . | . | 112 | 9,25 | 45 | 8,43 | 1473 | 414 |  |
| FOLH1 ---- candidate literature | rs10839236 | T/T | 1.00 (.-.) | . | 0.33 | 0.94 | 0.60 | 473 | 39,06 | 211 | 39,51 | 1473 | 414 |  |
| FOLH1 ---- candidate literature |  | T/C | 0.98 (0.79-1.21) | 0.85 | . | . | . | 569 | 46,99 | 264 | 49,44 | 1473 | 414 |  |
| FOLH1 ---- candidate literature |  | C/C | 0.83 (0.60-1.14) | 0.25 | . | . | . | 169 | 13,96 | 59 | 11,05 | 1473 | 414 |  |
| FOLH1 ---- tag | rs16906190 | A/A | 1.00 (.-.) | . | 0.23 | 0.90 | 0.60 | 1002 | 82,74 | 453 | 84,83 | 1473 | 414 | low_count |
| FOLH1 ---- tag |  | A/G | 0.87 (0.65-1.17) | 0.37 | . | . | . | 195 | 16,10 | 77 | 14,42 | 1473 | 414 |  |
| FOLH1 ---- tag |  | G/G | 0.66 (0.24-1.77) | 0.41 | . | . | . | 14 | 1,16 | 4 | 0,75 | 1473 | 414 |  |
| FOLH1 ---- candidate | rs202676 | T/T | 1.00 (.-.) | . | 0.42 | 0.94 | 0.60 | 751 | 62,01 | 335 | 62,73 | 1473 | 414 |  |
| FOLH1 ---- candidate |  | T/C | 0.84 (0.68-1.04) | 0.11 | . | . | . | 405 | 33,44 | 175 | 32,77 | 1473 | 414 |  |
| FOLH1 ---- candidate |  | C/C | 1.14 (0.73-1.78) | 0.57 | . | . | . | 55 | 4,54 | 24 | 4,49 | 1473 | 414 |  |
| FOLH1 ---- tag | rs202680 | A/A | 1.00 (.-.) | . | 0.64 | 0.97 | 0.71 | 666 | 55,00 | 298 | 55,81 | 1473 | 414 |  |
| FOLH1 ---- tag |  | A/T | 0.91 (0.74-1.11) | 0.34 | . | . | . | 476 | 39,31 | 203 | 38,01 | 1473 | 414 |  |
| FOLH1 ---- tag |  | T/T | 1.04 (0.70-1.55) | 0.84 | . | . | . | 69 | 5,70 | 33 | 6,18 | 1473 | 414 |  |
| FOLH1 ---- candidate literature | rs202720 | G/G | 1.00 (.-.) | . | 0.40 | 0.94 | 0.60 | 749 | 61,85 | 335 | 62,73 | 1473 | 414 |  |
| FOLH1 ---- candidate literature |  | G/C | 0.84 (0.68-1.04) | 0.11 | . | . | . | 407 | 33,61 | 175 | 32,77 | 1473 | 414 |  |
| FOLH1 ---- candidate literature |  | C/C | 1.14 (0.73-1.78) | 0.58 | . | . | . | 55 | 4,54 | 24 | 4,49 | 1473 | 414 |  |
| FOLH1 ---- tag | rs2299650 | G/G | 1.00 (.-.) | . | 0.29 | 0.92 | 0.60 | 467 | 38,56 | 210 | 39,33 | 1473 | 414 |  |
| FOLH1 ---- tag |  | G/T | 0.97 (0.78-1.19) | 0.75 | . | . | . | 575 | 47,48 | 266 | 49,81 | 1473 | 414 |  |
| FOLH1 ---- tag |  | T/T | 0.83 (0.60-1.14) | 0.24 | . | . | . | 169 | 13,96 | 58 | 10,86 | 1473 | 414 |  |
| FOLH1 ---- tag | rs617528 | G/G | 1.00 (.-.) | . | 0.37 | 0.94 | 0.60 | 938 | 77,46 | 424 | 79,40 | 1473 | 414 | low_count |
| FOLH1 ---- tag |  | G/A | 1.02 (0.80-1.29) | 0.88 | . | . | . | 249 | 20,56 | 106 | 19,85 | 1473 | 414 |  |
| FOLH1 ---- tag |  | A/A | 0.34 (0.11-1.07) | 0.07 | . | . | . | 24 | 1,98 | 4 | 0,75 | 1473 | 414 |  |
| FOLH1 ---- tag | rs663877 | T/T | 1.00 (.-.) | . | 0.98 | 0.99 | 0.98 | 934 | 77,13 | 406 | 76,03 | 1473 | 414 | low_count |
| FOLH1 ---- tag |  | T/G | 0.93 (0.74-1.17) | 0.55 | . | . | . | 262 | 21,64 | 119 | 22,28 | 1473 | 414 |  |
| FOLH1 ---- tag |  | G/G | 1.75 (0.82-3.73) | 0.15 | . | . | . | 15 | 1,24 | 9 | 1,69 | 1473 | 414 |  |
| FOLH1 ---- tag | rs670776 | A/A | 1.00 (.-.) | . | 0.42 | 0.94 | 0.60 | 751 | 62,01 | 335 | 62,73 | 1473 | 414 |  |
| FOLH1 ---- tag |  | A/T | 0.84 (0.68-1.04) | 0.11 | . | . | . | 405 | 33,44 | 175 | 32,77 | 1473 | 414 |  |
| FOLH1 ---- tag |  | T/T | 1.14 (0.73-1.78) | 0.57 | . | . | . | 55 | 4,54 | 24 | 4,49 | 1473 | 414 |  |
| FOLH1 ---- tag | rs7124497 | G/G | 1.00 (.-.) | . | 0.54 | 0.97 | 0.68 | 1112 | 91,82 | 489 | 91,57 | 1473 | 414 | low_count |
| FOLH1 ---- tag |  | G/A | 1.06 (0.75-1.50) | 0.74 | . | . | . | 98 | 8,09 | 44 | 8,24 | 1473 | 414 |  |
| FOLH1 ---- tag |  | A/A | 5.55 (0.76-40.53) | 0.09 | . | . | . | 1 | 0,08 | 1 | 0,19 | 1473 | 414 |  |
| FOLR1 ---- tag | rs651646 | T/T | 1.00 (.-.) | . | 0.07 | 0.85 | 0.07 | 362 | 29,89 | 180 | 33,71 | 1473 | 414 |  |
| FOLR1 ---- tag |  | T/A | 0.87 (0.70-1.09) | 0.23 | . | . | . | 612 | 50,54 | 253 | 47,38 | 1473 | 414 |  |
| FOLR1 ---- tag |  | A/A | 0.78 (0.59-1.03) | 0.08 | . | . | . | 237 | 19,57 | 101 | 18,91 | 1473 | 414 |  |
| FPGS ---- tag | rs10987746 | T/T | 1.00 (.-.) | . | 0.86 | 0.99 | 0.92 | 352 | 29,07 | 144 | 26,97 | 1473 | 414 |  |
| FPGS ---- tag |  | T/C | 0.89 (0.71-1.12) | 0.33 | . | . | . | 610 | 50,37 | 269 | 50,37 | 1473 | 414 |  |
| FPGS ---- tag |  | C/C | 1.04 (0.79-1.37) | 0.78 | . | . | . | 249 | 20,56 | 121 | 22,66 | 1473 | 414 |  |
| FPGS ---- tag | rs7033913 | T/T | 1.00 (.-.) | . | 0.62 | 0.97 | 0.92 | 380 | 31,38 | 172 | 32,21 | 1473 | 414 |  |
| FPGS ---- tag |  | T/C | 0.92 (0.74-1.15) | 0.48 | . | . | . | 582 | 48,06 | 270 | 50,56 | 1473 | 414 |  |
| FPGS ---- tag |  | C/C | 0.95 (0.71-1.26) | 0.71 | . | . | . | 249 | 20,56 | 92 | 17,23 | 1473 | 414 |  |
| FPGS ---- tag | rs7039798 | G/G | 1.00 (.-.) | . | 0.92 | 0.99 | 0.92 | 396 | 32,70 | 160 | 29,96 | 1473 | 414 |  |
| FPGS ---- tag |  | G/A | 0.92 (0.74-1.16) | 0.49 | . | . | . | 594 | 49,05 | 272 | 50,94 | 1473 | 414 |  |
| FPGS ---- tag |  | A/A | 1.04 (0.78-1.38) | 0.80 | . | . | . | 221 | 18,25 | 102 | 19,10 | 1473 | 414 |  |
| GGH ---- tag | rs10957264 | G/G | 1.00 (.-.) | . | 0.28 | 0.90 | 0.50 | 847 | 69,94 | 386 | 72,28 | 1473 | 414 |  |
| GGH ---- tag |  | G/T | 0.98 (0.79-1.23) | 0.89 | . | . | . | 329 | 27,17 | 136 | 25,47 | 1473 | 414 |  |
| GGH ---- tag |  | T/T | 0.55 (0.27-1.12) | 0.10 | . | . | . | 35 | 2,89 | 12 | 2,25 | 1473 | 414 |  |
| GGH ---- candidate literature | rs11545076 | T/T | 1.00 (.-.) | . | 0.20 | 0.86 | 0.47 | 603 | 49,79 | 264 | 49,44 | 1473 | 414 |  |
| GGH ---- candidate literature |  | T/G | 0.98 (0.80-1.20) | 0.84 | . | . | . | 504 | 41,62 | 230 | 43,07 | 1473 | 414 |  |
| GGH ---- candidate literature |  | G/G | 0.72 (0.49-1.06) | 0.10 | . | . | . | 104 | 8,59 | 40 | 7,49 | 1473 | 414 |  |
| GGH ---- candidate | rs11545077 | G/G | 1.00 (.-.) | . | 0.31 | 0.92 | 0.50 | 652 | 53,84 | 289 | 54,12 | 1473 | 414 |  |
| GGH ---- candidate |  | G/A | 1.04 (0.85-1.28) | 0.69 | . | . | . | 475 | 39,22 | 216 | 40,45 | 1473 | 414 |  |
| GGH ---- candidate |  | A/A | 0.66 (0.43-1.02) | 0.06 | . | . | . | 84 | 6,94 | 29 | 5,43 | 1473 | 414 |  |
| GGH ---- candidate | rs11545078 | C/C | 1.00 (.-.) | . | 0.58 | 0.97 | 0.58 | 1002 | 82,74 | 436 | 81,65 | 1473 | 414 | low_count |
| GGH ---- candidate |  | C/T | 1.06 (0.82-1.38) | 0.65 | . | . | . | 194 | 16,02 | 91 | 17,04 | 1473 | 414 |  |
| GGH ---- candidate |  | T/T | 0.51 (0.21-1.25) | 0.14 | . | . | . | 15 | 1,24 | 7 | 1,31 | 1473 | 414 |  |
| GGH ---- tag | rs11995525 | G/G | 1.00 (.-.) | . | 0.11 | 0.86 | 0.47 | 653 | 53,92 | 278 | 52,06 | 1473 | 414 |  |
| GGH ---- tag |  | G/A | 1.02 (0.83-1.26) | 0.83 | . | . | . | 479 | 39,55 | 213 | 39,89 | 1473 | 414 |  |
| GGH ---- tag |  | A/A | 1.54 (1.07-2.21) | 0.02 | . | . | . | 79 | 6,52 | 43 | 8,05 | 1473 | 414 |  |
| GGH ---- tag | rs16930073 | G/G | 1.00 (.-.) | . | 0.51 | 0.96 | 0.58 | 965 | 79,69 | 430 | 80,52 | 1473 | 414 | low_count |
| GGH ---- tag |  | G/A | 1.03 (0.81-1.32) | 0.80 | . | . | . | 225 | 18,58 | 100 | 18,73 | 1473 | 414 |  |
| GGH ---- tag |  | A/A | 0.45 (0.17-1.23) | 0.12 | . | . | . | 21 | 1,73 | 4 | 0,75 | 1473 | 414 |  |
| GGH ---- tag | rs17194931 | G/G | 1.00 (.-.) | . | 0.58 | 0.97 | 0.58 | 1002 | 82,74 | 436 | 81,65 | 1473 | 414 | low_count |
| GGH ---- tag |  | G/A | 1.06 (0.82-1.38) | 0.65 | . | . | . | 194 | 16,02 | 91 | 17,04 | 1473 | 414 |  |
| GGH ---- tag |  | A/A | 0.51 (0.21-1.25) | 0.14 | . | . | . | 15 | 1,24 | 7 | 1,31 | 1473 | 414 |  |
| GGH ---- candidate literature | rs1800909 | T/T | 1.00 (.-.) | . | 0.14 | 0.86 | 0.47 | 598 | 49,38 | 264 | 49,44 | 1473 | 414 |  |
| GGH ---- candidate literature |  | T/C | 0.96 (0.78-1.17) | 0.66 | . | . | . | 512 | 42,28 | 230 | 43,07 | 1473 | 414 |  |
| GGH ---- candidate literature |  | C/C | 0.71 (0.49-1.05) | 0.09 | . | . | . | 101 | 8,34 | 40 | 7,49 | 1473 | 414 |  |
| GGH ---- candidate literature | rs3758149 | C/C | 1.00 (.-.) | . | 0.20 | 0.86 | 0.47 | 603 | 49,79 | 264 | 49,44 | 1473 | 414 |  |
| GGH ---- candidate literature |  | C/T | 0.98 (0.80-1.20) | 0.84 | . | . | . | 504 | 41,62 | 230 | 43,07 | 1473 | 414 |  |
| GGH ---- candidate literature |  | T/T | 0.72 (0.49-1.06) | 0.10 | . | . | . | 104 | 8,59 | 40 | 7,49 | 1473 | 414 |  |
| GGH ---- tag | rs3780130 | A/A | 1.00 (.-.) | . | 0.20 | 0.86 | 0.47 | 751 | 62,01 | 348 | 65,17 | 1473 | 414 |  |
| GGH ---- tag |  | A/T | 0.87 (0.70-1.08) | 0.21 | . | . | . | 405 | 33,44 | 170 | 31,84 | 1473 | 414 |  |
| GGH ---- tag |  | T/T | 0.85 (0.49-1.47) | 0.57 | . | . | . | 55 | 4,54 | 16 | 3,00 | 1473 | 414 |  |
| GGH ---- tag | rs4446729 | C/C | 1.00 (.-.) | . | 0.53 | 0.97 | 0.58 | 648 | 53,51 | 271 | 50,75 | 1473 | 414 |  |
| GGH ---- tag |  | C/T | 1.05 (0.85-1.29) | 0.66 | . | . | . | 479 | 39,55 | 220 | 41,20 | 1473 | 414 |  |
| GGH ---- tag |  | T/T | 1.11 (0.76-1.63) | 0.58 | . | . | . | 84 | 6,94 | 43 | 8,05 | 1473 | 414 |  |
| GGH ---- tag | rs6472067 | C/C | 1.00 (.-.) | . | 0.22 | 0.89 | 0.47 | 482 | 39,80 | 213 | 39,89 | 1473 | 414 |  |
| GGH ---- tag |  | C/G | 1.06 (0.86-1.31) | 0.60 | . | . | . | 567 | 46,82 | 242 | 45,32 | 1473 | 414 |  |
| GGH ---- tag |  | G/G | 1.22 (0.91-1.65) | 0.19 | . | . | . | 162 | 13,38 | 79 | 14,79 | 1473 | 414 |  |
| GGH ---- tag | rs7010484 | T/T | 1.00 (.-.) | . | 0.49 | 0.96 | 0.58 | 552 | 45,58 | 234 | 43,82 | 1473 | 414 |  |
| GGH ---- tag |  | T/C | 1.08 (0.88-1.33) | 0.48 | . | . | . | 526 | 43,44 | 233 | 43,63 | 1473 | 414 |  |
| GGH ---- tag |  | C/C | 1.08 (0.79-1.48) | 0.62 | . | . | . | 133 | 10,98 | 67 | 12,55 | 1473 | 414 |  |
| GNMT ---- tag | rs1053538 | C/C | 1.00 (.-.) | . | 0.41 | 0.94 | 0.55 | 336 | 27,75 | 146 | 27,34 | 1473 | 414 |  |
| GNMT ---- tag |  | C/G | 0.97 (0.77-1.22) | 0.79 | . | . | . | 584 | 48,22 | 253 | 47,38 | 1473 | 414 |  |
| GNMT ---- tag |  | G/G | 1.13 (0.86-1.49) | 0.36 | . | . | . | 291 | 24,03 | 135 | 25,28 | 1473 | 414 |  |
| GNMT ---- tag | rs2296805 | G/G | 1.00 (.-.) | . | 0.36 | 0.94 | 0.55 | 366 | 30,22 | 180 | 33,71 | 1473 | 414 |  |
| GNMT ---- tag |  | G/T | 0.86 (0.69-1.08) | 0.20 | . | . | . | 614 | 50,70 | 249 | 46,63 | 1473 | 414 |  |
| GNMT ---- tag |  | T/T | 0.90 (0.68-1.19) | 0.45 | . | . | . | 231 | 19,08 | 105 | 19,66 | 1473 | 414 |  |
| GNMT ---- tag | rs6901782 | T/T | 1.00 (.-.) | . | 0.41 | 0.94 | 0.55 | 919 | 75,89 | 414 | 77,53 | 1473 | 414 |  |
| GNMT ---- tag |  | T/C | 1.10 (0.86-1.40) | 0.44 | . | . | . | 269 | 22,21 | 107 | 20,04 | 1473 | 414 |  |
| GNMT ---- tag |  | C/C | 1.13 (0.61-2.07) | 0.70 | . | . | . | 23 | 1,90 | 13 | 2,43 | 1473 | 414 |  |
| GNMT ---- tag | rs6927188 | A/A | 1.00 (.-.) | . | 0.96 | 0.99 | 0.96 | 706 | 58,30 | 299 | 55,99 | 1473 | 414 |  |
| GNMT ---- tag |  | A/G | 0.96 (0.78-1.18) | 0.68 | . | . | . | 422 | 34,85 | 201 | 37,64 | 1473 | 414 |  |
| GNMT ---- tag |  | G/G | 1.12 (0.74-1.71) | 0.59 | . | . | . | 83 | 6,85 | 34 | 6,37 | 1473 | 414 |  |
| MAT1A ---- tag | rs10887708 | G/G | 1.00 (.-.) | . | 0.90 | 0.99 | 0.90 | 603 | 49,79 | 273 | 51,12 | 1473 | 414 |  |
| MAT1A ---- tag |  | G/A | 1.12 (0.91-1.38) | 0.29 | . | . | . | 484 | 39,97 | 218 | 40,82 | 1473 | 414 |  |
| MAT1A ---- tag |  | A/A | 0.89 (0.62-1.29) | 0.55 | . | . | . | 124 | 10,24 | 43 | 8,05 | 1473 | 414 |  |
| MAT1A ---- tag | rs10887718 | T/T | 1.00 (.-.) | . | 0.06 | 0.85 | 0.51 | 349 | 28,82 | 141 | 26,40 | 1473 | 414 |  |
| MAT1A ---- tag |  | T/C | 1.23 (0.97-1.56) | 0.09 | . | . | . | 584 | 48,22 | 266 | 49,81 | 1473 | 414 |  |
| MAT1A ---- tag |  | C/C | 1.30 (0.98-1.71) | 0.07 | . | . | . | 278 | 22,96 | 127 | 23,78 | 1473 | 414 |  |
| MAT1A ---- tag | rs11202403 | C/C | 1.00 (.-.) | . | 0.47 | 0.95 | 0.61 | 790 | 65,24 | 344 | 64,42 | 1473 | 414 |  |
| MAT1A ---- tag |  | C/T | 1.01 (0.82-1.24) | 0.95 | . | . | . | 365 | 30,14 | 175 | 32,77 | 1473 | 414 |  |
| MAT1A ---- tag |  | T/T | 0.71 (0.41-1.22) | 0.22 | . | . | . | 56 | 4,62 | 15 | 2,81 | 1473 | 414 |  |
| MAT1A ---- tag | rs1832683 | C/C | 1.00 (.-.) | . | 0.29 | 0.91 | 0.59 | 839 | 69,28 | 369 | 69,10 | 1473 | 414 |  |
| MAT1A ---- tag |  | C/T | 0.83 (0.67-1.04) | 0.11 | . | . | . | 333 | 27,50 | 141 | 26,40 | 1473 | 414 |  |
| MAT1A ---- tag |  | T/T | 1.04 (0.64-1.69) | 0.88 | . | . | . | 39 | 3,22 | 24 | 4,49 | 1473 | 414 |  |
| MAT1A ---- tag | rs2236568 | C/C | 1.00 (.-.) | . | 0.25 | 0.90 | 0.59 | 389 | 32,12 | 172 | 32,21 | 1473 | 414 |  |
| MAT1A ---- tag |  | C/A | 1.14 (0.91-1.42) | 0.26 | . | . | . | 574 | 47,40 | 268 | 50,19 | 1473 | 414 |  |
| MAT1A ---- tag |  | A/A | 0.79 (0.59-1.07) | 0.13 | . | . | . | 248 | 20,48 | 94 | 17,60 | 1473 | 414 |  |
| MAT1A ---- tag | rs2236569 | A/A | 1.00 (.-.) | . | 0.15 | 0.86 | 0.59 | 542 | 44,76 | 224 | 41,95 | 1473 | 414 |  |
| MAT1A ---- tag |  | A/G | 1.35 (1.10-1.66) | 0.01 | . | . | . | 535 | 44,18 | 250 | 46,82 | 1473 | 414 |  |
| MAT1A ---- tag |  | G/G | 1.03 (0.74-1.45) | 0.85 | . | . | . | 134 | 11,07 | 60 | 11,24 | 1473 | 414 |  |
| MAT1A ---- tag | rs9421467 | G/G | 1.00 (.-.) | . | 0.39 | 0.94 | 0.59 | 1090 | 90,01 | 473 | 88,58 | 1473 | 414 | low_count |
| MAT1A ---- tag |  | G/C | 1.16 (0.86-1.57) | 0.33 | . | . | . | 115 | 9,50 | 60 | 11,24 | 1473 | 414 |  |
| MAT1A ---- tag |  | C/C | 0.85 (0.12-6.11) | 0.87 | . | . | . | 6 | 0,50 | 1 | 0,19 | 1473 | 414 |  |
| MAT1A ---- tag | rs998765 | A/A | 1.00 (.-.) | . | 0.69 | 0.98 | 0.78 | 309 | 25,52 | 141 | 26,40 | 1473 | 414 |  |
| MAT1A ---- tag |  | A/T | 1.12 (0.89-1.42) | 0.34 | . | . | . | 595 | 49,13 | 272 | 50,94 | 1473 | 414 |  |
| MAT1A ---- tag |  | T/T | 0.94 (0.71-1.25) | 0.68 | . | . | . | 307 | 25,35 | 121 | 22,66 | 1473 | 414 |  |
| MAT1A ---- tag | rs998766 | C/C | 1.00 (.-.) | . | 0.39 | 0.94 | 0.59 | 376 | 31,05 | 173 | 32,40 | 1473 | 414 |  |
| MAT1A ---- tag |  | C/G | 1.08 (0.87-1.35) | 0.47 | . | . | . | 587 | 48,47 | 271 | 50,75 | 1473 | 414 |  |
| MAT1A ---- tag |  | G/G | 0.84 (0.63-1.13) | 0.26 | . | . | . | 248 | 20,48 | 90 | 16,85 | 1473 | 414 |  |
| MAT2B ---- tag | rs12655857 | G/G | 1.00 (.-.) | . | 0.64 | 0.97 | 0.80 | 676 | 55,82 | 301 | 56,37 | 1473 | 414 |  |
| MAT2B ---- tag |  | G/T | 1.02 (0.82-1.25) | 0.88 | . | . | . | 452 | 37,32 | 198 | 37,08 | 1473 | 414 |  |
| MAT2B ---- tag |  | T/T | 1.12 (0.76-1.64) | 0.57 | . | . | . | 83 | 6,85 | 35 | 6,55 | 1473 | 414 |  |
| MAT2B ---- tag | rs6869277 | C/C | 1.00 (.-.) | . | 0.87 | 0.99 | 0.87 | 948 | 78,28 | 420 | 78,65 | 1473 | 414 | low_count |
| MAT2B ---- tag |  | C/T | 0.91 (0.71-1.17) | 0.45 | . | . | . | 246 | 20,31 | 108 | 20,22 | 1473 | 414 |  |
| MAT2B ---- tag |  | T/T | 1.74 (0.77-3.95) | 0.18 | . | . | . | 17 | 1,40 | 6 | 1,12 | 1473 | 414 |  |
| MAT2B ---- tag | rs6874065 | A/A | 1.00 (.-.) | . | 0.12 | 0.86 | 0.20 | 331 | 27,33 | 150 | 28,09 | 1473 | 414 |  |
| MAT2B ---- tag |  | A/G | 1.02 (0.81-1.28) | 0.86 | . | . | . | 621 | 51,28 | 269 | 50,37 | 1473 | 414 |  |
| MAT2B ---- tag |  | G/G | 0.78 (0.59-1.04) | 0.09 | . | . | . | 259 | 21,39 | 115 | 21,54 | 1473 | 414 |  |
| MAT2B ---- tag | rs6882306 | T/T | 1.00 (.-.) | . | 0.00 | 0.45 | 0.01 | 809 | 66,80 | 352 | 65,92 | 1473 | 414 |  |
| MAT2B ---- tag |  | T/C | 1.31 (1.05-1.62) | 0.01 | . | . | . | 375 | 30,97 | 162 | 30,34 | 1473 | 414 |  |
| MAT2B ---- tag |  | C/C | 1.91 (1.15-3.16) | 0.01 | . | . | . | 27 | 2,23 | 20 | 3,75 | 1473 | 414 |  |
| MAT2B ---- tag | rs7721639 | T/T | 1.00 (.-.) | . | 0.03 | 0.85 | 0.09 | 862 | 71,18 | 366 | 68,54 | 1473 | 414 |  |
| MAT2B ---- tag |  | T/G | 1.31 (1.05-1.62) | 0.02 | . | . | . | 315 | 26,01 | 155 | 29,03 | 1473 | 414 |  |
| MAT2B ---- tag |  | G/G | 1.13 (0.61-2.10) | 0.69 | . | . | . | 34 | 2,81 | 13 | 2,43 | 1473 | 414 |  |
| MTHFD1 ---- tag | rs1256148 | G/G | 1.00 (.-.) | . | 0.75 | 0.98 | 0.90 | 715 | 59,04 | 316 | 59,18 | 1473 | 414 |  |
| MTHFD1 ---- tag |  | G/A | 0.88 (0.71-1.09) | 0.25 | . | . | . | 439 | 36,25 | 183 | 34,27 | 1473 | 414 |  |
| MTHFD1 ---- tag |  | A/A | 1.39 (0.95-2.03) | 0.09 | . | . | . | 57 | 4,71 | 35 | 6,55 | 1473 | 414 |  |
| MTHFD1 ---- tag | rs13329053 | T/T | 1.00 (.-.) | . | 0.20 | 0.86 | 0.32 | 378 | 31,21 | 169 | 31,65 | 1473 | 414 |  |
| MTHFD1 ---- tag |  | T/C | 1.08 (0.86-1.36) | 0.51 | . | . | . | 585 | 48,31 | 252 | 47,19 | 1473 | 414 |  |
| MTHFD1 ---- tag |  | C/C | 1.20 (0.91-1.57) | 0.20 | . | . | . | 248 | 20,48 | 113 | 21,16 | 1473 | 414 |  |
| MTHFD1 ---- candidate literature | rs2236224 | C/C | 1.00 (.-.) | . | 0.15 | 0.86 | 0.32 | 467 | 38,56 | 211 | 39,51 | 1473 | 414 |  |
| MTHFD1 ---- candidate literature |  | C/T | 1.12 (0.90-1.39) | 0.30 | . | . | . | 556 | 45,91 | 240 | 44,94 | 1473 | 414 |  |
| MTHFD1 ---- candidate literature |  | T/T | 1.22 (0.91-1.62) | 0.18 | . | . | . | 188 | 15,52 | 83 | 15,54 | 1473 | 414 |  |
| MTHFD1 ---- candidate | rs2236225 | C/C | 1.00 (.-.) | . | 0.21 | 0.89 | 0.32 | 383 | 31,63 | 174 | 32,58 | 1473 | 414 |  |
| MTHFD1 ---- candidate |  | C/T | 1.07 (0.85-1.34) | 0.58 | . | . | . | 589 | 48,64 | 253 | 47,38 | 1473 | 414 |  |
| MTHFD1 ---- candidate |  | T/T | 1.20 (0.91-1.58) | 0.20 | . | . | . | 239 | 19,74 | 107 | 20,04 | 1473 | 414 |  |
| MTHFD1 ---- tag | rs2281603 | A/A | 1.00 (.-.) | . | 0.93 | 0.99 | 0.93 | 712 | 58,79 | 309 | 57,87 | 1473 | 414 |  |
| MTHFD1 ---- tag |  | A/G | 0.98 (0.80-1.20) | 0.82 | . | . | . | 440 | 36,33 | 196 | 36,70 | 1473 | 414 |  |
| MTHFD1 ---- tag |  | G/G | 1.03 (0.66-1.59) | 0.91 | . | . | . | 59 | 4,87 | 29 | 5,43 | 1473 | 414 |  |
| MTHFD1 ---- candidate literature | rs8003379 | A/A | 1.00 (.-.) | . | 0.07 | 0.85 | 0.32 | 669 | 55,24 | 314 | 58,80 | 1473 | 414 |  |
| MTHFD1 ---- candidate literature |  | A/C | 1.10 (0.89-1.35) | 0.40 | . | . | . | 457 | 37,74 | 181 | 33,90 | 1473 | 414 |  |
| MTHFD1 ---- candidate literature |  | C/C | 1.44 (0.99-2.08) | 0.06 | . | . | . | 85 | 7,02 | 39 | 7,30 | 1473 | 414 |  |
| MTHFD2 ---- tag | rs10177833 | A/A | 1.00 (.-.) | . | 0.72 | 0.98 | 0.85 | 374 | 30,88 | 154 | 28,84 | 1473 | 414 |  |
| MTHFD2 ---- tag |  | A/C | 0.94 (0.75-1.17) | 0.56 | . | . | . | 615 | 50,78 | 283 | 53,00 | 1473 | 414 |  |
| MTHFD2 ---- tag |  | C/C | 0.96 (0.72-1.28) | 0.79 | . | . | . | 222 | 18,33 | 97 | 18,16 | 1473 | 414 |  |
| MTHFD2 ---- tag | rs702462 | T/T | 1.00 (.-.) | . | 0.30 | 0.92 | 0.85 | 395 | 32,62 | 177 | 33,15 | 1473 | 414 |  |
| MTHFD2 ---- tag |  | T/A | 0.93 (0.74-1.16) | 0.51 | . | . | . | 610 | 50,37 | 266 | 49,81 | 1473 | 414 |  |
| MTHFD2 ---- tag |  | A/A | 0.86 (0.64-1.15) | 0.32 | . | . | . | 206 | 17,01 | 91 | 17,04 | 1473 | 414 |  |
| MTHFD2 ---- candidate literature | rs702465 | A/A | 1.00 (.-.) | . | 0.29 | 0.91 | 0.85 | 343 | 28,32 | 146 | 27,34 | 1473 | 414 |  |
| MTHFD2 ---- candidate literature |  | A/T | 1.05 (0.84-1.33) | 0.66 | . | . | . | 603 | 49,79 | 280 | 52,43 | 1473 | 414 |  |
| MTHFD2 ---- candidate literature |  | T/T | 1.17 (0.88-1.54) | 0.28 | . | . | . | 265 | 21,88 | 108 | 20,22 | 1473 | 414 |  |
| MTHFD2 ---- candidate literature | rs7571842 | A/A | 1.00 (.-.) | . | 0.73 | 0.98 | 0.85 | 352 | 29,07 | 133 | 24,91 | 1473 | 414 |  |
| MTHFD2 ---- candidate literature |  | A/G | 1.00 (0.79-1.26) | 0.99 | . | . | . | 607 | 50,12 | 295 | 55,24 | 1473 | 414 |  |
| MTHFD2 ---- candidate literature |  | G/G | 0.95 (0.71-1.26) | 0.71 | . | . | . | 252 | 20,81 | 106 | 19,85 | 1473 | 414 |  |
| MTHFD2 ---- tag | rs7587117 | T/T | 1.00 (.-.) | . | 0.89 | 0.99 | 0.89 | 504 | 41,62 | 212 | 39,70 | 1473 | 414 |  |
| MTHFD2 ---- tag |  | T/C | 0.99 (0.80-1.22) | 0.95 | . | . | . | 568 | 46,90 | 256 | 47,94 | 1473 | 414 |  |
| MTHFD2 ---- tag |  | C/C | 0.98 (0.71-1.34) | 0.89 | . | . | . | 139 | 11,48 | 66 | 12,36 | 1473 | 414 |  |
| MTHFD2 ---- tag | rs828861 | C/C | 1.00 (.-.) | . | 0.44 | 0.94 | 0.85 | 349 | 28,82 | 146 | 27,34 | 1473 | 414 |  |
| MTHFD2 ---- tag |  | C/G | 1.08 (0.86-1.36) | 0.52 | . | . | . | 606 | 50,04 | 288 | 53,93 | 1473 | 414 |  |
| MTHFD2 ---- tag |  | G/G | 1.11 (0.84-1.48) | 0.46 | . | . | . | 256 | 21,14 | 100 | 18,73 | 1473 | 414 |  |
| MTHFD2 ---- tag | rs828863 | G/G | 1.00 (.-.) | . | 0.62 | 0.97 | 0.85 | 975 | 80,51 | 447 | 83,71 | 1473 | 414 | low_count |
| MTHFD2 ---- tag |  | G/A | 0.84 (0.64-1.11) | 0.22 | . | . | . | 227 | 18,74 | 82 | 15,36 | 1473 | 414 |  |
| MTHFD2 ---- tag |  | A/A | 2.40 (0.98-5.89) | 0.06 | . | . | . | 9 | 0,74 | 5 | 0,94 | 1473 | 414 |  |
| MTHFR ---- tag | rs1476413 | G/G | 1.00 (.-.) | . | 0.39 | 0.94 | 0.72 | 631 | 52,11 | 294 | 55,06 | 1473 | 414 |  |
| MTHFR ---- tag |  | G/A | 0.90 (0.74-1.11) | 0.33 | . | . | . | 502 | 41,45 | 207 | 38,76 | 1473 | 414 |  |
| MTHFR ---- tag |  | A/A | 0.92 (0.61-1.40) | 0.71 | . | . | . | 78 | 6,44 | 33 | 6,18 | 1473 | 414 |  |
| MTHFR ---- tag | rs17376328 | G/G | 1.00 (.-.) | . | 0.53 | 0.97 | 0.72 | 1078 | 89,02 | 457 | 85,58 | 1473 | 414 | low_count |
| MTHFR ---- tag |  | G/A | 1.13 (0.85-1.52) | 0.40 | . | . | . | 127 | 10,49 | 74 | 13,86 | 1473 | 414 |  |
| MTHFR ---- tag |  | A/A | 0.88 (0.28-2.80) | 0.83 | . | . | . | 6 | 0,50 | 3 | 0,56 | 1473 | 414 |  |
| MTHFR ---- tag | rs17421462 | G/G | 1.00 (.-.) | . | 0.72 | 0.98 | 0.72 | 1028 | 84,89 | 456 | 85,39 | 1473 | 414 | low_count |
| MTHFR ---- tag |  | G/A | 0.92 (0.69-1.24) | 0.59 | . | . | . | 180 | 14,86 | 73 | 13,67 | 1473 | 414 |  |
| MTHFR ---- tag |  | A/A | 3.23 (1.31-7.94) | 0.01 | . | . | . | 3 | 0,25 | 5 | 0,94 | 1473 | 414 |  |
| MTHFR ---- candidate | rs1801131 | A/A | 1.00 (.-.) | . | 0.65 | 0.97 | 0.72 | 548 | 45,25 | 252 | 47,19 | 1473 | 414 |  |
| MTHFR ---- candidate |  | A/C | 0.93 (0.76-1.14) | 0.47 | . | . | . | 545 | 45,00 | 230 | 43,07 | 1473 | 414 |  |
| MTHFR ---- candidate |  | C/C | 0.98 (0.69-1.40) | 0.93 | . | . | . | 118 | 9,74 | 52 | 9,74 | 1473 | 414 |  |
| MTHFR ---- candidate | rs1801133 | C/C | 1.00 (.-.) | . | 0.18 | 0.86 | 0.72 | 502 | 41,45 | 221 | 41,39 | 1473 | 414 |  |
| MTHFR ----candidate |  | C/T | 0.86 (0.70-1.06) | 0.16 | . | . | . | 569 | 46,99 | 257 | 48,13 | 1473 | 414 |  |
| MTHFR ---- candidate |  | T/T | 0.85 (0.60-1.21) | 0.37 | . | . | . | 140 | 11,56 | 56 | 10,49 | 1473 | 414 |  |
| MTHFR ---- tag | rs2066471 | G/G | 1.00 (.-.) | . | 0.44 | 0.94 | 0.72 | 835 | 68,95 | 386 | 72,28 | 1473 | 414 |  |
| MTHFR ---- tag |  | G/A | 0.91 (0.72-1.14) | 0.40 | . | . | . | 350 | 28,90 | 136 | 25,47 | 1473 | 414 |  |
| MTHFR ---- tag |  | A/A | 0.93 (0.49-1.76) | 0.83 | . | . | . | 26 | 2,15 | 12 | 2,25 | 1473 | 414 |  |
| MTHFR ---- tag | rs4846047 | G/G | 1.00 (.-.) | . | 0.51 | 0.96 | 0.72 | 598 | 49,38 | 281 | 52,62 | 1473 | 414 |  |
| MTHFR ---- tag |  | G/C | 0.88 (0.72-1.08) | 0.24 | . | . | . | 526 | 43,44 | 211 | 39,51 | 1473 | 414 |  |
| MTHFR ---- tag |  | C/C | 1.02 (0.70-1.49) | 0.92 | . | . | . | 87 | 7,18 | 42 | 7,87 | 1473 | 414 |  |
| MTHFR ---- tag | rs4846049 | G/G | 1.00 (.-.) | . | 0.57 | 0.97 | 0.72 | 538 | 44,43 | 249 | 46,63 | 1473 | 414 |  |
| MTHFR ---- tag |  | G/T | 0.90 (0.74-1.11) | 0.33 | . | . | . | 554 | 45,75 | 231 | 43,26 | 1473 | 414 |  |
| MTHFR ---- tag |  | T/T | 0.99 (0.70-1.40) | 0.95 | . | . | . | 119 | 9,83 | 54 | 10,11 | 1473 | 414 |  |
| MTHFR ---- tag | rs7538516 | T/T | 1.00 (.-.) | . | 0.67 | 0.98 | 0.72 | 438 | 36,17 | 202 | 37,83 | 1473 | 414 |  |
| MTHFR ---- tag |  | T/C | 0.91 (0.73-1.12) | 0.37 | . | . | . | 606 | 50,04 | 257 | 48,13 | 1473 | 414 |  |
| MTHFR ---- tag |  | C/C | 0.98 (0.72-1.33) | 0.91 | . | . | . | 167 | 13,79 | 75 | 14,04 | 1473 | 414 |  |
| MTR ---- tag | rs10733117 | A/A | 1.00 (.-.) | . | 0.44 | 0.94 | 0.45 | 429 | 35,43 | 192 | 35,96 | 1473 | 414 |  |
| MTR ---- tag |  | A/G | 0.98 (0.79-1.21) | 0.84 | . | . | . | 585 | 48,31 | 256 | 47,94 | 1473 | 414 |  |
| MTR ---- tag |  | G/G | 0.87 (0.64-1.19) | 0.40 | . | . | . | 197 | 16,27 | 86 | 16,10 | 1473 | 414 |  |
| MTR ---- tag | rs12129440 | G/G | 1.00 (.-.) | . | 0.24 | 0.90 | 0.45 | 647 | 53,43 | 311 | 58,24 | 1473 | 414 |  |
| MTR ---- tag |  | G/A | 0.88 (0.71-1.08) | 0.21 | . | . | . | 476 | 39,31 | 192 | 35,96 | 1473 | 414 |  |
| MTR ---- tag |  | A/A | 0.89 (0.56-1.43) | 0.64 | . | . | . | 88 | 7,27 | 31 | 5,81 | 1473 | 414 |  |
| MTR ---- candidate | rs1805087 | A/A | 1.00 (.-.) | . | 0.45 | 0.95 | 0.45 | 810 | 66,89 | 362 | 67,79 | 1473 | 414 |  |
| MTR ---- candidate |  | A/G | 0.96 (0.77-1.19) | 0.68 | . | . | . | 361 | 29,81 | 151 | 28,28 | 1473 | 414 |  |
| MTR ---- candidate |  | G/G | 0.81 (0.48-1.39) | 0.45 | . | . | . | 40 | 3,30 | 21 | 3,93 | 1473 | 414 |  |
| MTR ---- tag | rs3890786 | C/C | 1.00 (.-.) | . | 0.17 | 0.86 | 0.45 | 410 | 33,86 | 180 | 33,71 | 1473 | 414 |  |
| MTR ---- tag |  | C/T | 1.12 (0.90-1.40) | 0.30 | . | . | . | 596 | 49,22 | 260 | 48,69 | 1473 | 414 |  |
| MTR ---- tag |  | T/T | 1.21 (0.90-1.61) | 0.20 | . | . | . | 205 | 16,93 | 94 | 17,60 | 1473 | 414 |  |
| MTR ---- tag | rs4659727 | A/A | 1.00 (.-.) | . | 0.35 | 0.94 | 0.45 | 806 | 66,56 | 361 | 67,60 | 1473 | 414 |  |
| MTR ---- tag |  | A/G | 0.98 (0.79-1.21) | 0.83 | . | . | . | 361 | 29,81 | 153 | 28,65 | 1473 | 414 |  |
| MTR ---- tag |  | G/G | 0.70 (0.40-1.22) | 0.21 | . | . | . | 44 | 3,63 | 20 | 3,75 | 1473 | 414 |  |
| MTRR ---- candidate literature/tag | rs10380 | C/C | 1.00 (.-.) | . | 0.14 | 0.86 | 0.48 | 1023 | 84,48 | 442 | 82,77 | 1473 | 414 | low_count |
| MTRR ---- candidate literature/tag |  | C/T | 1.20 (0.92-1.55) | 0.18 | . | . | . | 179 | 14,78 | 90 | 16,85 | 1473 | 414 |  |
| MTRR ---- candidate literature/tag |  | T/T | 1.77 (0.43-7.21) | 0.43 | . | . | . | 9 | 0,74 | 2 | 0,37 | 1473 | 414 |  |
| MTRR ---- tag | rs10475399 | G/G | 1.00 (.-.) | . | 0.43 | 0.94 | 0.97 | 528 | 43,60 | 228 | 42,70 | 1473 | 414 |  |
| MTRR ---- tag |  | G/A | 1.05 (0.85-1.29) | 0.65 | . | . | . | 555 | 45,83 | 245 | 45,88 | 1473 | 414 |  |
| MTRR ---- tag |  | A/A | 1.13 (0.83-1.55) | 0.43 | . | . | . | 128 | 10,57 | 61 | 11,42 | 1473 | 414 |  |
| MTRR ---- tag | rs11134265 | C/C | 1.00 (.-.) | . | 0.60 | 0.97 | 0.97 | 533 | 44,01 | 236 | 44,19 | 1473 | 414 |  |
| MTRR ---- tag |  | C/T | 1.06 (0.86-1.31) | 0.56 | . | . | . | 546 | 45,09 | 244 | 45,69 | 1473 | 414 |  |
| MTRR ---- tag |  | T/T | 1.06 (0.76-1.47) | 0.74 | . | . | . | 132 | 10,90 | 54 | 10,11 | 1473 | 414 |  |
| MTRR ---- tag | rs13181011 | T/T | 1.00 (.-.) | . | 0.57 | 0.97 | 0.97 | 788 | 65,07 | 336 | 62,92 | 1473 | 414 |  |
| MTRR ---- tag |  | T/C | 0.97 (0.78-1.19) | 0.77 | . | . | . | 374 | 30,88 | 173 | 32,40 | 1473 | 414 |  |
| MTRR ---- tag |  | C/C | 0.86 (0.52-1.42) | 0.57 | . | . | . | 49 | 4,05 | 25 | 4,68 | 1473 | 414 |  |
| MTRR ---- tag | rs161869 | C/C | 1.00 (.-.) | . | 0.14 | 0.86 | 0.48 | 419 | 34,60 | 189 | 35,39 | 1473 | 414 |  |
| MTRR ---- tag |  | C/T | 0.98 (0.79-1.22) | 0.87 | . | . | . | 593 | 48,97 | 254 | 47,57 | 1473 | 414 |  |
| MTRR ---- tag |  | T/T | 1.31 (0.99-1.74) | 0.06 | . | . | . | 199 | 16,43 | 91 | 17,04 | 1473 | 414 |  |
| MTRR ---- tagged by rs162039 | rs162036 | A/A | 1.00 (.-.) | . | 0.14 | 0.86 | 0.48 | 978 | 80,76 | 424 | 79,40 | 1473 | 414 | low_count |
| MTRR ---- tagged by rs162039 |  | A/G | 1.15 (0.89-1.47) | 0.28 | . | . | . | 222 | 18,33 | 105 | 19,66 | 1473 | 414 |  |
| MTRR ---- tagged by rs162039 |  | G/G | 1.85 (0.75-4.55) | 0.18 | . | . | . | 11 | 0,91 | 5 | 0,94 | 1473 | 414 |  |
| MTRR ---- tag | rs162039 | C/C | 1.00 (.-.) | . | 0.14 | 0.86 | 0.48 | 979 | 80,84 | 424 | 79,40 | 1473 | 414 | low_count |
| MTRR ---- tag |  | C/T | 1.15 (0.89-1.47) | 0.28 | . | . | . | 221 | 18,25 | 105 | 19,66 | 1473 | 414 |  |
| MTRR ---- tag |  | T/T | 1.85 (0.75-4.55) | 0.18 | . | . | . | 11 | 0,91 | 5 | 0,94 | 1473 | 414 |  |
| MTRR ---- tag | rs162270 | G/G | 1.00 (.-.) | . | 0.95 | 0.99 | 0.97 | 857 | 70,77 | 372 | 69,66 | 1473 | 414 |  |
| MTRR ---- tag |  | G/T | 1.05 (0.84-1.31) | 0.66 | . | . | . | 322 | 26,59 | 147 | 27,53 | 1473 | 414 |  |
| MTRR ---- tag |  | T/T | 0.88 (0.50-1.54) | 0.66 | . | . | . | 32 | 2,64 | 15 | 2,81 | 1473 | 414 |  |
| MTRR ---- candidate | rs16879334 | C/C | 1.00 (.-.) | . | 0.97 | 0.99 | 0.97 | 1139 | 94,05 | 503 | 94,19 | 1473 | 414 |  |
| MTRR ---- candidate |  | C/G | 0.99 (0.66-1.48) | 0.97 | . | . | . | 72 | 5,95 | 31 | 5,81 | 1473 | 414 |  |
| MTRR ---- singleton | rs1801394 | G/G | 1.00 (.-.) | . | 0.96 | 0.99 | 0.97 | 360 | 29,73 | 155 | 29,03 | 1473 | 414 |  |
| MTRR ---- singleton |  | G/A | 1.09 (0.87-1.37) | 0.45 | . | . | . | 602 | 49,71 | 284 | 53,18 | 1473 | 414 |  |
| MTRR ---- singleton |  | A/A | 0.99 (0.74-1.32) | 0.92 | . | . | . | 249 | 20,56 | 95 | 17,79 | 1473 | 414 |  |
| MTRR ---- tag | rs1802059 | G/G | 1.00 (.-.) | . | 0.20 | 0.86 | 0.56 | 459 | 37,90 | 210 | 39,33 | 1473 | 414 |  |
| MTRR ---- tag |  | G/A | 0.84 (0.68-1.04) | 0.12 | . | . | . | 582 | 48,06 | 243 | 45,51 | 1473 | 414 |  |
| MTRR ---- tag |  | A/A | 0.87 (0.65-1.18) | 0.37 | . | . | . | 170 | 14,04 | 81 | 15,17 | 1473 | 414 |  |
| MTRR ---- tag | rs2077744 | T/T | 1.00 (.-.) | . | 0.88 | 0.99 | 0.97 | 876 | 72,34 | 392 | 73,41 | 1473 | 414 |  |
| MTRR ---- tag |  | T/C | 0.98 (0.78-1.24) | 0.89 | . | . | . | 309 | 25,52 | 127 | 23,78 | 1473 | 414 |  |
| MTRR ---- tag |  | C/C | 1.13 (0.66-1.95) | 0.66 | . | . | . | 26 | 2,15 | 15 | 2,81 | 1473 | 414 |  |
| MTRR ---- candidate | rs2287780 | C/C | 1.00 (.-.) | . | 0.97 | 0.99 | 0.97 | 1139 | 94,05 | 503 | 94,19 | 1473 | 414 |  |
| MTRR ---- candidate |  | C/T | 0.99 (0.66-1.48) | 0.97 | . | . | . | 72 | 5,95 | 31 | 5,81 | 1473 | 414 |  |
| MTRR ---- candidate | rs2303080 | T/T | 1.00 (.-.) | . | 0.88 | 0.99 | 0.97 | 1139 | 94,05 | 504 | 94,38 | 1473 | 414 |  |
| MTRR ---- candidate |  | T/A | 0.97 (0.64-1.46) | 0.88 | . | . | . | 72 | 5,95 | 30 | 5,62 | 1473 | 414 |  |
| MTRR ---- tag | rs7715062 | G/G | 1.00 (.-.) | . | 0.47 | 0.95 | 0.97 | 406 | 33,53 | 178 | 33,33 | 1473 | 414 |  |
| MTRR ---- tag |  | G/T | 0.86 (0.69-1.07) | 0.17 | . | . | . | 598 | 49,38 | 259 | 48,50 | 1473 | 414 |  |
| MTRR ---- tag |  | T/T | 0.94 (0.71-1.25) | 0.66 | . | . | . | 207 | 17,09 | 97 | 18,16 | 1473 | 414 |  |
| MTRR ---- tag | rs9282787 | T/T | 1.00 (.-.) | . | 0.74 | 0.98 | 0.97 | 794 | 65,57 | 339 | 63,48 | 1473 | 414 |  |
| MTRR ---- tag |  | T/C | 1.00 (0.81-1.24) | 0.97 | . | . | . | 367 | 30,31 | 171 | 32,02 | 1473 | 414 |  |
| MTRR ---- tag |  | C/C | 0.86 (0.52-1.42) | 0.56 | . | . | . | 50 | 4,13 | 24 | 4,49 | 1473 | 414 |  |
| MTRR ---- candidate literature | rs9332 | C/C | 1.00 (.-.) | . | 0.14 | 0.86 | 0.48 | 979 | 80,84 | 424 | 79,40 | 1473 | 414 | low_count |
| MTRR ---- candidate literature |  | C/T | 1.15 (0.89-1.47) | 0.28 | . | . | . | 221 | 18,25 | 105 | 19,66 | 1473 | 414 |  |
| MTRR ---- candidate literature |  | T/T | 1.85 (0.75-4.55) | 0.18 | . | . | . | 11 | 0,91 | 5 | 0,94 | 1473 | 414 |  |
| NFKB1 ---- NA | rs1609798 | C/C | 1.00 (.-.) | . | 0.72 | 0.98 | 0.98 | 573 | 47,32 | 245 | 45,88 | 1473 | 414 |  |
| NFKB1 ---- NA |  | C/T | 1.08 (0.88-1.32) | 0.49 | . | . | . | 526 | 43,44 | 238 | 44,57 | 1473 | 414 |  |
| NFKB1 ---- NA |  | T/T | 1.00 (0.70-1.42) | 0.99 | . | . | . | 112 | 9,25 | 51 | 9,55 | 1473 | 414 |  |
| NFKB1 ---- tag | rs230540 | T/T | 1.00 (.-.) | . | 0.42 | 0.94 | 0.98 | 514 | 42,44 | 227 | 42,51 | 1473 | 414 |  |
| NFKB1 ---- tag |  | T/C | 1.02 (0.83-1.25) | 0.87 | . | . | . | 559 | 46,16 | 250 | 46,82 | 1473 | 414 |  |
| NFKB1 ---- tag |  | C/C | 0.83 (0.59-1.15) | 0.26 | . | . | . | 138 | 11,40 | 57 | 10,67 | 1473 | 414 |  |
| NFKB1 ---- tag | rs230541 | A/A | 1.00 (.-.) | . | 0.51 | 0.96 | 0.98 | 410 | 33,86 | 184 | 34,46 | 1473 | 414 |  |
| NFKB1 ---- tag |  | A/G | 1.03 (0.83-1.28) | 0.79 | . | . | . | 602 | 49,71 | 269 | 50,37 | 1473 | 414 |  |
| NFKB1 ---- tag |  | G/G | 0.88 (0.65-1.18) | 0.39 | . | . | . | 199 | 16,43 | 81 | 15,17 | 1473 | 414 |  |
| NFKB1 ---- NA | rs230547 | C/C | 1.00 (.-.) | . | 0.61 | 0.97 | 0.98 | 981 | 81,01 | 436 | 81,65 | 1473 | 414 | low_count |
| NFKB1 ---- NA |  | C/T | 0.91 (0.71-1.17) | 0.47 | . | . | . | 220 | 18,17 | 93 | 17,42 | 1473 | 414 |  |
| NFKB1 ---- NA |  | T/T | 1.14 (0.47-2.80) | 0.77 | . | . | . | 10 | 0,83 | 5 | 0,94 | 1473 | 414 |  |
| NFKB1 ---- tag | rs3774934 | G/G | 1.00 (.-.) | . | 0.48 | 0.95 | 0.98 | 964 | 79,60 | 426 | 79,78 | 1473 | 414 | low_count |
| NFKB1 ---- tag |  | G/A | 0.84 (0.65-1.08) | 0.17 | . | . | . | 236 | 19,49 | 100 | 18,73 | 1473 | 414 |  |
| NFKB1 ---- tag |  | A/A | 1.50 (0.74-3.04) | 0.26 | . | . | . | 11 | 0,91 | 8 | 1,50 | 1473 | 414 |  |
| NFKB1 ---- tag | rs3774968 | G/G | 1.00 (.-.) | . | 1.00 | 1.00 | 1.00 | 391 | 32,29 | 165 | 30,90 | 1473 | 414 |  |
| NFKB1 ---- tag |  | G/A | 1.00 (0.80-1.25) | 1.00 | . | . | . | 601 | 49,63 | 271 | 50,75 | 1473 | 414 |  |
| NFKB1 ---- tag |  | A/A | 1.00 (0.75-1.33) | 1.00 | . | . | . | 219 | 18,08 | 98 | 18,35 | 1473 | 414 |  |
| NFKB1 ---- NA | rs4648022 | C/C | 1.00 (.-.) | . | 0.21 | 0.89 | 0.98 | 1008 | 83,24 | 452 | 84,64 | 1473 | 414 | low_count |
| NFKB1 ---- NA |  | C/T | 1.20 (0.91-1.59) | 0.19 | . | . | . | 195 | 16,10 | 78 | 14,61 | 1473 | 414 |  |
| NFKB1 ---- NA |  | T/T | 1.14 (0.41-3.11) | 0.81 | . | . | . | 8 | 0,66 | 4 | 0,75 | 1473 | 414 |  |
| NFKB1 ---- NA | rs4648090 | G/G | 1.00 (.-.) | . | 0.77 | 0.98 | 0.98 | 878 | 72,50 | 405 | 75,84 | 1473 | 414 | low_count |
| NFKB1 ---- NA |  | G/A | 1.04 (0.83-1.31) | 0.74 | . | . | . | 307 | 25,35 | 119 | 22,28 | 1473 | 414 |  |
| NFKB1 ---- NA |  | A/A | 1.02 (0.50-2.07) | 0.96 | . | . | . | 26 | 2,15 | 10 | 1,87 | 1473 | 414 |  |
| NFKB1 ---- tag | rs4648110 | T/T | 1.00 (.-.) | . | 0.84 | 0.99 | 0.98 | 765 | 63,17 | 343 | 64,23 | 1473 | 414 |  |
| NFKB1 ---- tag |  | T/A | 0.97 (0.79-1.20) | 0.78 | . | . | . | 396 | 32,70 | 171 | 32,02 | 1473 | 414 |  |
| NFKB1 ---- tag |  | A/A | 1.21 (0.74-1.99) | 0.45 | . | . | . | 50 | 4,13 | 20 | 3,75 | 1473 | 414 |  |
| NFKB1 ---- tag | rs4648141 | G/G | 1.00 (.-.) | . | 0.44 | 0.94 | 0.98 | 837 | 69,12 | 368 | 68,91 | 1473 | 414 |  |
| NFKB1 ---- tag |  | G/A | 1.05 (0.85-1.31) | 0.65 | . | . | . | 342 | 28,24 | 154 | 28,84 | 1473 | 414 |  |
| NFKB1 ---- tag |  | A/A | 1.26 (0.71-2.26) | 0.43 | . | . | . | 32 | 2,64 | 12 | 2,25 | 1473 | 414 |  |
| NFKB1 ---- tag | rs4698863 | C/C | 1.00 (.-.) | . | 0.97 | 0.99 | 1.00 | 562 | 46,41 | 240 | 44,94 | 1473 | 414 |  |
| NFKB1 ---- tag |  | C/T | 1.03 (0.84-1.26) | 0.79 | . | . | . | 531 | 43,85 | 241 | 45,13 | 1473 | 414 |  |
| NFKB1 ---- tag |  | T/T | 0.98 (0.69-1.38) | 0.89 | . | . | . | 118 | 9,74 | 53 | 9,93 | 1473 | 414 |  |
| NFKB1 ---- NA | rs7674640 | T/T | 1.00 (.-.) | . | 0.84 | 0.99 | 0.98 | 276 | 22,79 | 123 | 23,03 | 1473 | 414 |  |
| NFKB1 ---- NA |  | T/C | 1.01 (0.79-1.29) | 0.93 | . | . | . | 621 | 51,28 | 280 | 52,43 | 1473 | 414 |  |
| NFKB1 ---- NA |  | C/C | 0.97 (0.73-1.29) | 0.84 | . | . | . | 314 | 25,93 | 131 | 24,53 | 1473 | 414 |  |
| NFKB1 ---- tag | rs909332 | A/A | 1.00 (.-.) | . | 0.27 | 0.90 | 0.98 | 1098 | 90,67 | 486 | 91,01 | 1473 | 414 | low_count |
| NFKB1 ---- tag |  | A/T | 1.17 (0.84-1.64) | 0.36 | . | . | . | 111 | 9,17 | 47 | 8,80 | 1473 | 414 |  |
| NFKB1 ---- tag |  | T/T | 2.64 (0.36-19.17) | 0.34 | . | . | . | 2 | 0,17 | 1 | 0,19 | 1473 | 414 |  |
| NFKB1 ---- tag | rs997476 | C/C | 1.00 (.-.) | . | 0.81 | 0.99 | 0.98 | 1082 | 89,35 | 467 | 87,45 | 1473 | 414 | low_count |
| NFKB1 ---- tag |  | C/A | 0.98 (0.72-1.31) | 0.87 | . | . | . | 128 | 10,57 | 65 | 12,17 | 1473 | 414 |  |
| NFKB1 ---- tag |  | A/A | 3.89 (0.94-16.00) | 0.06 | . | . | . | 1 | 0,08 | 2 | 0,37 | 1473 | 414 |  |
| NME1 ---- NA | rs10514981 | T/T | 1.00 (.-.) | . | 0.54 | 0.97 | 0.96 | 745 | 61,52 | 335 | 62,73 | 1473 | 414 |  |
| NME1 ---- NA |  | T/G | 0.93 (0.76-1.14) | 0.49 | . | . | . | 415 | 34,27 | 180 | 33,71 | 1473 | 414 |  |
| NME1 ---- NA |  | G/G | 0.97 (0.55-1.69) | 0.90 | . | . | . | 51 | 4,21 | 19 | 3,56 | 1473 | 414 |  |
| NME1 ---- NA | rs11651252 | T/T | 1.00 (.-.) | . | 0.96 | 0.99 | 0.96 | 1079 | 89,10 | 471 | 88,20 | 1473 | 414 | low_count |
| NME1 ---- NA |  | T/C | 1.10 (0.80-1.51) | 0.54 | . | . | . | 127 | 10,49 | 62 | 11,61 | 1473 | 414 |  |
| NME1 ---- NA |  | C/C | 0.36 (0.05-2.59) | 0.31 | . | . | . | 5 | 0,41 | 1 | 0,19 | 1473 | 414 |  |
| NME1 ---- tag | rs11652793 | T/T | 1.00 (.-.) | . | 0.71 | 0.98 | 0.96 | 798 | 65,90 | 363 | 67,98 | 1473 | 414 |  |
| NME1 ---- tag |  | T/C | 0.93 (0.75-1.15) | 0.52 | . | . | . | 374 | 30,88 | 155 | 29,03 | 1473 | 414 |  |
| NME1 ---- tag |  | C/C | 1.13 (0.60-2.13) | 0.72 | . | . | . | 39 | 3,22 | 16 | 3,00 | 1473 | 414 |  |
| NME1 ---- NA | rs11868380 | C/C | 1.00 (.-.) | . | 0.91 | 0.99 | 0.96 | 763 | 63,01 | 330 | 61,80 | 1473 | 414 |  |
| NME1 ---- NA |  | C/G | 1.04 (0.84-1.28) | 0.74 | . | . | . | 401 | 33,11 | 185 | 34,64 | 1473 | 414 |  |
| NME1 ---- NA |  | G/G | 0.86 (0.52-1.43) | 0.56 | . | . | . | 47 | 3,88 | 19 | 3,56 | 1473 | 414 |  |
| NME1 ---- NA | rs1558252 | T/T | 1.00 (.-.) | . | 0.57 | 0.97 | 0.96 | 593 | 48,97 | 239 | 44,76 | 1473 | 414 |  |
| NME1 ---- NA |  | T/C | 1.16 (0.95-1.43) | 0.15 | . | . | . | 509 | 42,03 | 244 | 45,69 | 1473 | 414 |  |
| NME1 ---- NA |  | C/C | 0.96 (0.67-1.37) | 0.80 | . | . | . | 109 | 9,00 | 51 | 9,55 | 1473 | 414 |  |
| NME1 ---- NA | rs1558253 | T/T | 1.00 (.-.) | . | 0.14 | 0.86 | 0.96 | 1081 | 89,27 | 460 | 86,14 | 1473 | 414 | low_count |
| NME1 ---- NA |  | T/G | 1.22 (0.92-1.61) | 0.17 | . | . | . | 126 | 10,40 | 73 | 13,67 | 1473 | 414 |  |
| NME1 ---- NA |  | G/G | 2.08 (0.29-15.17) | 0.47 | . | . | . | 4 | 0,33 | 1 | 0,19 | 1473 | 414 |  |
| NME1 ---- tag | rs16949683 | C/C | 1.00 (.-.) | . | 0.74 | 0.98 | 0.96 | 1124 | 92,82 | 494 | 92,51 | 1473 | 414 | low_count |
| NME1 ---- tag |  | C/T | 1.06 (0.73-1.55) | 0.75 | . | . | . | 84 | 6,94 | 39 | 7,30 | 1473 | 414 |  |
| NME1 ---- tag |  | T/T | 1.11 (0.15-8.09) | 0.91 | . | . | . | 3 | 0,25 | 1 | 0,19 | 1473 | 414 |  |
| NME1 ---- tag | rs2318784 | C/C | 1.00 (.-.) | . | 0.77 | 0.98 | 0.96 | 919 | 75,89 | 414 | 77,53 | 1473 | 414 | low_count |
| NME1 ---- tag |  | C/T | 1.03 (0.81-1.31) | 0.80 | . | . | . | 271 | 22,38 | 113 | 21,16 | 1473 | 414 |  |
| NME1 ---- tag |  | T/T | 1.08 (0.48-2.44) | 0.86 | . | . | . | 21 | 1,73 | 7 | 1,31 | 1473 | 414 |  |
| NME1 ---- NA | rs2318785 | G/G | 1.00 (.-.) | . | 0.52 | 0.97 | 0.96 | 387 | 31,96 | 160 | 29,96 | 1473 | 414 |  |
| NME1 ---- NA |  | G/A | 1.02 (0.82-1.28) | 0.83 | . | . | . | 600 | 49,55 | 291 | 54,49 | 1473 | 414 |  |
| NME1 ---- NA |  | A/A | 0.88 (0.65-1.19) | 0.42 | . | . | . | 224 | 18,50 | 83 | 15,54 | 1473 | 414 |  |
| NME1 ---- tag | rs3760469 | G/G | 1.00 (.-.) | . | 0.85 | 0.99 | 0.96 | 327 | 27,00 | 121 | 22,66 | 1473 | 414 |  |
| NME1 ---- tag |  | G/T | 1.11 (0.87-1.41) | 0.42 | . | . | . | 598 | 49,38 | 295 | 55,24 | 1473 | 414 |  |
| NME1 ---- tag |  | T/T | 1.02 (0.76-1.38) | 0.88 | . | . | . | 286 | 23,62 | 118 | 22,10 | 1473 | 414 |  |
| NME1 ---- NA | rs4605213 | G/G | 1.00 (.-.) | . | 0.94 | 0.99 | 0.96 | 508 | 41,95 | 235 | 44,01 | 1473 | 414 |  |
| NME1 ---- NA |  | G/C | 0.92 (0.75-1.14) | 0.45 | . | . | . | 560 | 46,24 | 235 | 44,01 | 1473 | 414 |  |
| NME1 ---- NA |  | C/C | 1.06 (0.77-1.45) | 0.72 | . | . | . | 143 | 11,81 | 64 | 11,99 | 1473 | 414 |  |
| NME1 ---- NA | rs7207090 | A/A | 1.00 (.-.) | . | 0.86 | 0.99 | 0.96 | 305 | 25,19 | 133 | 24,91 | 1473 | 414 |  |
| NME1 ---- NA |  | A/T | 0.84 (0.66-1.07) | 0.16 | . | . | . | 623 | 51,45 | 257 | 48,13 | 1473 | 414 |  |
| NME1 ---- NA |  | T/T | 0.97 (0.74-1.28) | 0.84 | . | . | . | 283 | 23,37 | 144 | 26,97 | 1473 | 414 |  |
| NME1 ---- tag | rs7222463 | A/A | 1.00 (.-.) | . | 0.87 | 0.99 | 0.96 | 323 | 26,67 | 151 | 28,28 | 1473 | 414 |  |
| NME1 ---- tag |  | A/C | 0.83 (0.66-1.05) | 0.12 | . | . | . | 620 | 51,20 | 259 | 48,50 | 1473 | 414 |  |
| NME1 ---- tag |  | C/C | 1.05 (0.80-1.37) | 0.74 | . | . | . | 268 | 22,13 | 124 | 23,22 | 1473 | 414 |  |
| NME1 ---- tag | rs7226059 | C/C | 1.00 (.-.) | . | 0.35 | 0.94 | 0.96 | 567 | 46,82 | 218 | 40,82 | 1473 | 414 |  |
| NME1 ---- tag |  | C/T | 1.28 (1.04-1.57) | 0.02 | . | . | . | 516 | 42,61 | 258 | 48,31 | 1473 | 414 |  |
| NME1 ---- tag |  | T/T | 0.98 (0.70-1.37) | 0.90 | . | . | . | 128 | 10,57 | 58 | 10,86 | 1473 | 414 |  |
| NME1 ---- NA | rs880178 | G/G | 1.00 (.-.) | . | 0.73 | 0.98 | 0.96 | 315 | 26,01 | 127 | 23,78 | 1473 | 414 |  |
| NME1 ---- NA |  | G/T | 1.00 (0.78-1.27) | 1.00 | . | . | . | 612 | 50,54 | 286 | 53,56 | 1473 | 414 |  |
| NME1 ---- NA |  | T/T | 1.05 (0.79-1.40) | 0.73 | . | . | . | 284 | 23,45 | 121 | 22,66 | 1473 | 414 |  |
| NME2 ---- tag | rs7220360 | C/C | 1.00 (.-.) | . | 0.83 | 0.99 | 0.83 | 323 | 26,67 | 149 | 27,90 | 1473 | 414 |  |
| NME2 ---- tag |  | C/G | 0.84 (0.67-1.06) | 0.15 | . | . | . | 619 | 51,11 | 261 | 48,88 | 1473 | 414 |  |
| NME2 ---- tag |  | G/G | 1.05 (0.80-1.38) | 0.71 | . | . | . | 269 | 22,21 | 124 | 23,22 | 1473 | 414 |  |
| PON1 ---- tag | rs2269829 | A/A | 1.00 (.-.) | . | 0.83 | 0.99 | 0.94 | 620 | 51,20 | 256 | 47,94 | 1473 | 414 |  |
| PON1 ---- tag |  | A/G | 1.01 (0.82-1.23) | 0.95 | . | . | . | 481 | 39,72 | 230 | 43,07 | 1473 | 414 |  |
| PON1 ---- tag |  | G/G | 1.05 (0.74-1.49) | 0.80 | . | . | . | 110 | 9,08 | 48 | 8,99 | 1473 | 414 |  |
| PON1 ---- tag | rs3917527 | A/A | 1.00 (.-.) | . | 0.08 | 0.85 | 0.20 | 1081 | 89,27 | 489 | 91,57 | 1473 | 414 | low_count |
| PON1 ---- tag |  | A/G | 0.75 (0.54-1.05) | 0.10 | . | . | . | 124 | 10,24 | 44 | 8,24 | 1473 | 414 |  |
| PON1 ---- tag |  | G/G | 0.57 (0.08-4.10) | 0.58 | . | . | . | 6 | 0,50 | 1 | 0,19 | 1473 | 414 |  |
| PON1 ---- tag | rs3917538 | C/C | 1.00 (.-.) | . | 0.08 | 0.85 | 0.20 | 704 | 58,13 | 285 | 53,37 | 1473 | 414 |  |
| PON1 ---- tag |  | C/T | 1.07 (0.87-1.31) | 0.55 | . | . | . | 439 | 36,25 | 208 | 38,95 | 1473 | 414 |  |
| PON1 ---- tag |  | T/T | 1.53 (1.04-2.24) | 0.03 | . | . | . | 68 | 5,62 | 41 | 7,68 | 1473 | 414 |  |
| PON1 ---- tag | rs757158 | C/C | 1.00 (.-.) | . | 0.94 | 0.99 | 0.94 | 424 | 35,01 | 197 | 36,89 | 1473 | 414 |  |
| PON1 ---- tag |  | C/T | 0.89 (0.71-1.10) | 0.27 | . | . | . | 593 | 48,97 | 243 | 45,51 | 1473 | 414 |  |
| PON1 ---- tag |  | T/T | 1.08 (0.81-1.44) | 0.61 | . | . | . | 194 | 16,02 | 94 | 17,60 | 1473 | 414 |  |
| PON1 ---- candidate | rs854560 | A/A | 1.00 (.-.) | . | 0.82 | 0.99 | 0.94 | 477 | 39,39 | 221 | 41,39 | 1473 | 414 |  |
| PON1 ---- candidate |  | A/T | 0.99 (0.80-1.23) | 0.94 | . | . | . | 558 | 46,08 | 230 | 43,07 | 1473 | 414 |  |
| PON1 ---- candidate |  | T/T | 1.05 (0.78-1.41) | 0.75 | . | . | . | 176 | 14,53 | 83 | 15,54 | 1473 | 414 |  |
| PRDM2 ---- tag | rs1015370 | C/C | 1.00 (.-.) | . | 0.43 | 0.94 | 0.86 | 608 | 50,21 | 296 | 55,43 | 1473 | 414 |  |
| PRDM2 ---- tag |  | C/T | 0.91 (0.74-1.12) | 0.35 | . | . | . | 484 | 39,97 | 189 | 35,39 | 1473 | 414 |  |
| PRDM2 ---- tag |  | T/T | 0.93 (0.66-1.31) | 0.68 | . | . | . | 119 | 9,83 | 49 | 9,18 | 1473 | 414 |  |
| PRDM2 ---- tag | rs1203634 | A/A | 1.00 (.-.) | . | 0.46 | 0.95 | 0.86 | 746 | 61,60 | 318 | 59,55 | 1473 | 414 |  |
| PRDM2 ---- tag |  | A/G | 1.06 (0.86-1.32) | 0.56 | . | . | . | 412 | 34,02 | 176 | 32,96 | 1473 | 414 |  |
| PRDM2 ---- tag |  | G/G | 1.12 (0.76-1.64) | 0.57 | . | . | . | 53 | 4,38 | 40 | 7,49 | 1473 | 414 |  |
| PRDM2 ---- tag | rs1203645 | A/A | 1.00 (.-.) | . | 0.76 | 0.98 | 0.86 | 487 | 40,21 | 226 | 42,32 | 1473 | 414 |  |
| PRDM2 ---- tag |  | A/C | 1.00 (0.81-1.24) | 1.00 | . | . | . | 553 | 45,66 | 213 | 39,89 | 1473 | 414 |  |
| PRDM2 ---- tag |  | C/C | 1.05 (0.80-1.38) | 0.72 | . | . | . | 171 | 14,12 | 95 | 17,79 | 1473 | 414 |  |
| PRDM2 ---- tag | rs1406416 | C/C | 1.00 (.-.) | . | 0.19 | 0.86 | 0.86 | 666 | 55,00 | 273 | 51,12 | 1473 | 414 |  |
| PRDM2 ---- tag |  | C/T | 1.01 (0.82-1.25) | 0.93 | . | . | . | 448 | 36,99 | 202 | 37,83 | 1473 | 414 |  |
| PRDM2 ---- tag |  | T/T | 1.32 (0.96-1.81) | 0.08 | . | . | . | 97 | 8,01 | 59 | 11,05 | 1473 | 414 |  |
| PRDM2 ---- candidate | rs17350795 | G/G | 1.00 (.-.) | . | 0.22 | 0.90 | 0.86 | 1158 | 95,62 | 512 | 95,88 | 1473 | 414 |  |
| PRDM2 ---- candidate |  | G/A | 0.73 (0.44-1.21) | 0.22 | . | . | . | 53 | 4,38 | 22 | 4,12 | 1473 | 414 |  |
| PRDM2 ---- tag | rs1980472 | C/C | 1.00 (.-.) | . | 0.77 | 0.98 | 0.86 | 683 | 56,40 | 290 | 54,31 | 1473 | 414 |  |
| PRDM2 ---- tag |  | C/G | 1.05 (0.85-1.29) | 0.64 | . | . | . | 449 | 37,08 | 199 | 37,27 | 1473 | 414 |  |
| PRDM2 ---- tag |  | G/G | 1.01 (0.70-1.45) | 0.97 | . | . | . | 79 | 6,52 | 45 | 8,43 | 1473 | 414 |  |
| PRDM2 ---- tag | rs2235515 | G/G | 1.00 (.-.) | . | 0.97 | 0.99 | 0.97 | 711 | 58,71 | 306 | 57,30 | 1473 | 414 |  |
| PRDM2 ---- tag |  | G/A | 1.09 (0.89-1.34) | 0.41 | . | . | . | 431 | 35,59 | 202 | 37,83 | 1473 | 414 |  |
| PRDM2 ---- tag |  | A/A | 0.81 (0.51-1.28) | 0.36 | . | . | . | 69 | 5,70 | 26 | 4,87 | 1473 | 414 |  |
| PRDM2 ---- tag | rs2244634 | A/A | 1.00 (.-.) | . | 0.37 | 0.94 | 0.86 | 773 | 63,83 | 348 | 65,17 | 1473 | 414 |  |
| PRDM2 ---- tag |  | A/C | 1.06 (0.85-1.31) | 0.60 | . | . | . | 378 | 31,21 | 167 | 31,27 | 1473 | 414 |  |
| PRDM2 ---- tag |  | C/C | 0.62 (0.37-1.03) | 0.07 | . | . | . | 60 | 4,95 | 19 | 3,56 | 1473 | 414 |  |
| PRDM2 ---- tag | rs2245213 | G/G | 1.00 (.-.) | . | 0.51 | 0.96 | 0.86 | 855 | 70,60 | 375 | 70,22 | 1473 | 414 | low_count |
| PRDM2 ---- tag |  | G/T | 1.15 (0.92-1.42) | 0.22 | . | . | . | 315 | 26,01 | 152 | 28,46 | 1473 | 414 |  |
| PRDM2 ---- tag |  | T/T | 0.31 (0.13-0.77) | 0.01 | . | . | . | 41 | 3,39 | 7 | 1,31 | 1473 | 414 |  |
| PRDM2 ---- tag | rs2294484 | C/C | 1.00 (.-.) | . | 0.79 | 0.99 | 0.86 | 1003 | 82,82 | 455 | 85,21 | 1473 | 414 | low_count |
| PRDM2 ---- tag |  | C/G | 1.02 (0.77-1.35) | 0.88 | . | . | . | 195 | 16,10 | 78 | 14,61 | 1473 | 414 |  |
| PRDM2 ---- tag |  | G/G | 0.37 (0.05-2.65) | 0.32 | . | . | . | 13 | 1,07 | 1 | 0,19 | 1473 | 414 |  |
| PRDM2 ---- tag | rs2744689 | G/G | 1.00 (.-.) | . | 0.59 | 0.97 | 0.86 | 868 | 71,68 | 379 | 70,97 | 1473 | 414 | low_count |
| PRDM2 ---- tag |  | G/A | 1.14 (0.91-1.41) | 0.25 | . | . | . | 309 | 25,52 | 147 | 27,53 | 1473 | 414 |  |
| PRDM2 ---- tag |  | A/A | 0.38 (0.17-0.87) | 0.02 | . | . | . | 34 | 2,81 | 8 | 1,50 | 1473 | 414 |  |
| PRDM2 ---- tag | rs6690270 | A/A | 1.00 (.-.) | . | 0.64 | 0.97 | 0.86 | 494 | 40,79 | 205 | 38,39 | 1473 | 414 |  |
| PRDM2 ---- tag |  | A/G | 1.05 (0.85-1.29) | 0.66 | . | . | . | 565 | 46,66 | 251 | 47,00 | 1473 | 414 |  |
| PRDM2 ---- tag |  | G/G | 1.06 (0.79-1.43) | 0.70 | . | . | . | 152 | 12,55 | 78 | 14,61 | 1473 | 414 |  |
| RRM1 ---- tag | rs10835601 | G/G | 1.00 (.-.) | . | 0.08 | 0.85 | 0.29 | 591 | 48,80 | 256 | 47,94 | 1473 | 414 |  |
| RRM1 ---- tag |  | G/A | 1.11 (0.90-1.37) | 0.31 | . | . | . | 519 | 42,86 | 224 | 41,95 | 1473 | 414 |  |
| RRM1 ---- tag |  | A/A | 1.35 (0.97-1.88) | 0.08 | . | . | . | 101 | 8,34 | 54 | 10,11 | 1473 | 414 |  |
| RRM1 ---- tag | rs10835613 | C/C | 1.00 (.-.) | . | 0.10 | 0.86 | 0.29 | 407 | 33,61 | 182 | 34,08 | 1473 | 414 |  |
| RRM1 ---- tag |  | C/G | 1.07 (0.86-1.34) | 0.54 | . | . | . | 587 | 48,47 | 253 | 47,38 | 1473 | 414 |  |
| RRM1 ---- tag |  | G/G | 1.28 (0.97-1.69) | 0.09 | . | . | . | 217 | 17,92 | 99 | 18,54 | 1473 | 414 |  |
| RRM1 ---- NA | rs10835677 | G/G | 1.00 (.-.) | . | 0.63 | 0.97 | 0.73 | 1017 | 83,98 | 431 | 80,71 | 1473 | 414 | low_count |
| RRM1 ---- NA |  | G/A | 1.04 (0.80-1.34) | 0.78 | . | . | . | 183 | 15,11 | 96 | 17,98 | 1473 | 414 |  |
| RRM1 ---- NA |  | A/A | 1.24 (0.55-2.80) | 0.61 | . | . | . | 11 | 0,91 | 7 | 1,31 | 1473 | 414 |  |
| RRM1 ---- tag | rs10835678 | A/A | 1.00 (.-.) | . | 0.66 | 0.97 | 0.73 | 1075 | 88,77 | 475 | 88,95 | 1473 | 414 | low_count |
| RRM1 ---- tag |  | A/G | 0.91 (0.66-1.26) | 0.57 | . | . | . | 132 | 10,90 | 57 | 10,67 | 1473 | 414 |  |
| RRM1 ---- tag |  | G/G | 1.18 (0.29-4.83) | 0.82 | . | . | . | 4 | 0,33 | 2 | 0,37 | 1473 | 414 |  |
| RRM1 ---- tag | rs12288551 | C/C | 1.00 (.-.) | . | 0.12 | 0.86 | 0.29 | 1128 | 93,15 | 480 | 89,89 | 1473 | 414 | low_count |
| RRM1 ---- tag |  | C/G | 1.25 (0.90-1.74) | 0.18 | . | . | . | 82 | 6,77 | 52 | 9,74 | 1473 | 414 |  |
| RRM1 ---- tag |  | G/G | 3.92 (0.50-30.44) | 0.19 | . | . | . | 1 | 0,08 | 2 | 0,37 | 1473 | 414 |  |
| RRM1 ---- NA | rs12806698 | C/C | 1.00 (.-.) | . | 0.15 | 0.86 | 0.31 | 624 | 51,53 | 277 | 51,87 | 1473 | 414 |  |
| RRM1 ---- NA |  | C/A | 1.06 (0.86-1.30) | 0.58 | . | . | . | 498 | 41,12 | 212 | 39,70 | 1473 | 414 |  |
| RRM1 ---- NA |  | A/A | 1.34 (0.95-1.91) | 0.10 | . | . | . | 89 | 7,35 | 45 | 8,43 | 1473 | 414 |  |
| RRM1 ---- NA | rs1465952 | T/T | 1.00 (.-.) | . | 0.62 | 0.97 | 0.73 | 987 | 81,50 | 440 | 82,40 | 1473 | 414 | low_count |
| RRM1 ---- NA |  | T/C | 0.96 (0.74-1.24) | 0.73 | . | . | . | 209 | 17,26 | 91 | 17,04 | 1473 | 414 |  |
| RRM1 ---- NA |  | C/C | 0.71 (0.17-2.86) | 0.63 | . | . | . | 15 | 1,24 | 3 | 0,56 | 1473 | 414 |  |
| RRM1 ---- tag | rs4910904 | A/A | 1.00 (.-.) | . | 0.02 | 0.85 | 0.20 | 530 | 43,77 | 219 | 41,01 | 1473 | 414 |  |
| RRM1 ---- tag |  | A/G | 1.21 (0.98-1.50) | 0.07 | . | . | . | 547 | 45,17 | 246 | 46,07 | 1473 | 414 |  |
| RRM1 ---- tag |  | G/G | 1.39 (1.01-1.90) | 0.04 | . | . | . | 134 | 11,07 | 69 | 12,92 | 1473 | 414 |  |
| RRM1 ---- tag | rs7103860 | T/T | 1.00 (.-.) | . | 0.79 | 0.99 | 0.79 | 915 | 75,56 | 416 | 77,90 | 1473 | 414 | low_count |
| RRM1 ---- tag |  | T/C | 1.04 (0.82-1.32) | 0.73 | . | . | . | 278 | 22,96 | 115 | 21,54 | 1473 | 414 |  |
| RRM1 ---- tag |  | C/C | 0.41 (0.10-1.64) | 0.21 | . | . | . | 18 | 1,49 | 3 | 0,56 | 1473 | 414 |  |
| RRM1 ---- tag | rs7115496 | C/C | 1.00 (.-.) | . | 0.47 | 0.95 | 0.73 | 1026 | 84,72 | 466 | 87,27 | 1473 | 414 | low_count |
| RRM1 ---- tag |  | C/T | 0.95 (0.71-1.28) | 0.74 | . | . | . | 176 | 14,53 | 66 | 12,36 | 1473 | 414 |  |
| RRM1 ---- tag |  | T/T | 0.54 (0.13-2.16) | 0.38 | . | . | . | 9 | 0,74 | 2 | 0,37 | 1473 | 414 |  |
| RRM2 ---- NA | rs1138729 | A/A | 1.00 (.-.) | . | 0.23 | 0.90 | 0.67 | 884 | 73,00 | 386 | 72,28 | 1473 | 414 |  |
| RRM2 ---- NA |  | A/G | 1.08 (0.86-1.35) | 0.53 | . | . | . | 297 | 24,53 | 130 | 24,34 | 1473 | 414 |  |
| RRM2 ---- NA |  | G/G | 1.41 (0.83-2.40) | 0.20 | . | . | . | 30 | 2,48 | 18 | 3,37 | 1473 | 414 |  |
| RRM2 ---- tag | rs4668664 | G/G | 1.00 (.-.) | . | 0.43 | 0.94 | 0.67 | 607 | 50,12 | 270 | 50,56 | 1473 | 414 |  |
| RRM2 ---- tag |  | G/A | 1.01 (0.83-1.24) | 0.89 | . | . | . | 489 | 40,38 | 217 | 40,64 | 1473 | 414 |  |
| RRM2 ---- tag |  | A/A | 0.80 (0.56-1.15) | 0.23 | . | . | . | 115 | 9,50 | 47 | 8,80 | 1473 | 414 |  |
| RRM2 ---- NA | rs6741290 | C/C | 1.00 (.-.) | . | 0.80 | 0.99 | 0.80 | 388 | 32,04 | 169 | 31,65 | 1473 | 414 |  |
| RRM2 ---- NA |  | C/T | 1.10 (0.88-1.37) | 0.42 | . | . | . | 573 | 47,32 | 252 | 47,19 | 1473 | 414 |  |
| RRM2 ---- NA |  | T/T | 1.02 (0.78-1.34) | 0.88 | . | . | . | 250 | 20,64 | 113 | 21,16 | 1473 | 414 |  |
| RRM2 ---- tag | rs7574663 | C/C | 1.00 (.-.) | . | 0.51 | 0.96 | 0.67 | 796 | 65,73 | 344 | 64,42 | 1473 | 414 |  |
| RRM2 ---- tag |  | C/G | 1.02 (0.83-1.27) | 0.84 | . | . | . | 364 | 30,06 | 165 | 30,90 | 1473 | 414 |  |
| RRM2 ---- tag |  | G/G | 1.22 (0.78-1.91) | 0.39 | . | . | . | 51 | 4,21 | 25 | 4,68 | 1473 | 414 |  |
| SHMT1 ---- candidate | rs1979277 | G/G | 1.00 (.-.) | . | 0.74 | 0.98 | 0.95 | 582 | 48,06 | 273 | 51,12 | 1473 | 414 |  |
| SHMT1 ---- candidate |  | G/A | 0.84 (0.69-1.04) | 0.11 | . | . | . | 529 | 43,68 | 213 | 39,89 | 1473 | 414 |  |
| SHMT1 ---- candidate |  | A/A | 1.19 (0.83-1.70) | 0.34 | . | . | . | 100 | 8,26 | 48 | 8,99 | 1473 | 414 |  |
| SHMT1 ---- tag | rs2168781 | G/G | 1.00 (.-.) | . | 0.95 | 0.99 | 0.95 | 413 | 34,10 | 208 | 38,95 | 1473 | 414 |  |
| SHMT1 ---- tag |  | G/C | 0.87 (0.70-1.08) | 0.21 | . | . | . | 621 | 51,28 | 239 | 44,76 | 1473 | 414 |  |
| SHMT1 ---- tag |  | C/C | 1.08 (0.80-1.45) | 0.62 | . | . | . | 177 | 14,62 | 87 | 16,29 | 1473 | 414 |  |
| SHMT1 ---- tag | rs4924849 | C/C | 1.00 (.-.) | . | 0.76 | 0.98 | 0.95 | 611 | 50,45 | 284 | 53,18 | 1473 | 414 |  |
| SHMT1 ---- tag |  | C/T | 0.86 (0.70-1.05) | 0.14 | . | . | . | 510 | 42,11 | 206 | 38,58 | 1473 | 414 |  |
| SHMT1 ---- tag |  | T/T | 1.18 (0.82-1.70) | 0.36 | . | . | . | 90 | 7,43 | 44 | 8,24 | 1473 | 414 |  |
| SHMT1 ---- candidate literature | rs9909104 | T/T | 1.00 (.-.) | . | 0.74 | 0.98 | 0.95 | 621 | 51,28 | 289 | 54,12 | 1473 | 414 |  |
| SHMT1 ---- candidate literature |  | T/C | 0.92 (0.75-1.14) | 0.46 | . | . | . | 501 | 41,37 | 202 | 37,83 | 1473 | 414 |  |
| SHMT1 ---- candidate literature |  | C/C | 1.02 (0.71-1.47) | 0.90 | . | . | . | 89 | 7,35 | 43 | 8,05 | 1473 | 414 |  |
| SHMT2 ---- tag | rs10876968 | G/G | 1.00 (.-.) | . | 1.00 | 1.00 | 1.00 | 655 | 54,09 | 292 | 54,68 | 1473 | 414 |  |
| SHMT2 ---- tag |  | G/T | 0.94 (0.77-1.16) | 0.59 | . | . | . | 465 | 38,40 | 195 | 36,52 | 1473 | 414 |  |
| SHMT2 ---- tag |  | T/T | 1.09 (0.76-1.57) | 0.64 | . | . | . | 91 | 7,51 | 47 | 8,80 | 1473 | 414 |  |
| SHMT2 ---- tag | rs1800165 | T/T | 1.00 (.-.) | . | 0.35 | 0.94 | 0.67 | 586 | 48,39 | 263 | 49,25 | 1473 | 414 |  |
| SHMT2 ---- tag |  | T/C | 1.07 (0.87-1.31) | 0.55 | . | . | . | 515 | 42,53 | 221 | 41,39 | 1473 | 414 |  |
| SHMT2 ---- tag |  | C/C | 1.17 (0.83-1.64) | 0.38 | . | . | . | 110 | 9,08 | 50 | 9,36 | 1473 | 414 |  |
| SHMT2 ---- tag | rs7133939 | T/T | 1.00 (.-.) | . | 0.64 | 0.97 | 0.80 | 360 | 29,73 | 172 | 32,21 | 1473 | 414 |  |
| SHMT2 ---- tag |  | T/A | 1.00 (0.80-1.25) | 0.98 | . | . | . | 595 | 49,13 | 249 | 46,63 | 1473 | 414 |  |
| SHMT2 ---- tag |  | A/A | 0.93 (0.70-1.23) | 0.60 | . | . | . | 256 | 21,14 | 113 | 21,16 | 1473 | 414 |  |
| SHMT2 ---- tag | rs7485577 | G/G | 1.00 (.-.) | . | 0.40 | 0.94 | 0.67 | 644 | 53,18 | 291 | 54,49 | 1473 | 414 |  |
| SHMT2 ---- tag |  | G/A | 1.02 (0.83-1.26) | 0.83 | . | . | . | 475 | 39,22 | 199 | 37,27 | 1473 | 414 |  |
| SHMT2 ---- tag |  | A/A | 1.20 (0.85-1.71) | 0.30 | . | . | . | 92 | 7,60 | 44 | 8,24 | 1473 | 414 |  |
| SHMT2 ---- tag | rs7489231 | T/T | 1.00 (.-.) | . | 0.06 | 0.85 | 0.32 | 533 | 44,01 | 234 | 43,82 | 1473 | 414 |  |
| SHMT2 ---- tag |  | T/C | 1.22 (0.99-1.50) | 0.06 | . | . | . | 542 | 44,76 | 239 | 44,76 | 1473 | 414 |  |
| SHMT2 ---- tag |  | C/C | 1.24 (0.90-1.72) | 0.19 | . | . | . | 136 | 11,23 | 61 | 11,42 | 1473 | 414 |  |
| SLC19A1 ---- candidate | rs1051266 | G/G | 1.00 (.-.) | . | 0.80 | 0.99 | 0.80 | 388 | 32,04 | 153 | 28,65 | 1473 | 414 |  |
| SLC19A1 ---- candidate |  | G/A | 1.17 (0.94-1.47) | 0.16 | . | . | . | 579 | 47,81 | 290 | 54,31 | 1473 | 414 |  |
| SLC19A1 ---- candidate |  | A/A | 0.91 (0.67-1.22) | 0.53 | . | . | . | 244 | 20,15 | 91 | 17,04 | 1473 | 414 |  |
| SLC19A1 ---- candidate literature | rs1131596 | T/T | 1.00 (.-.) | . | 0.80 | 0.99 | 0.80 | 389 | 32,12 | 153 | 28,65 | 1473 | 414 |  |
| SLC19A1 ---- candidate literature |  | T/C | 1.18 (0.94-1.47) | 0.15 | . | . | . | 576 | 47,56 | 290 | 54,31 | 1473 | 414 |  |
| SLC19A1 ---- candidate literature |  | C/C | 0.91 (0.67-1.22) | 0.52 | . | . | . | 246 | 20,31 | 91 | 17,04 | 1473 | 414 |  |
| SLC19A1 ---- tag | rs12483553 | G/G | 1.00 (.-.) | . | 0.07 | 0.85 | 0.46 | 986 | 81,42 | 416 | 77,90 | 1473 | 414 | low_count |
| SLC19A1 ---- tag |  | G/A | 1.20 (0.95-1.51) | 0.12 | . | . | . | 214 | 17,67 | 112 | 20,97 | 1473 | 414 |  |
| SLC19A1 ---- tag |  | A/A | 1.68 (0.69-4.12) | 0.26 | . | . | . | 11 | 0,91 | 6 | 1,12 | 1473 | 414 |  |
| SLC19A1 ---- candidate literature | rs12659 | C/C | 1.00 (.-.) | . | 0.63 | 0.97 | 0.80 | 401 | 33,11 | 162 | 30,34 | 1473 | 414 |  |
| SLC19A1 ---- candidate literature |  | C/T | 1.16 (0.93-1.44) | 0.18 | . | . | . | 567 | 46,82 | 282 | 52,81 | 1473 | 414 |  |
| SLC19A1 ---- candidate literature |  | T/T | 0.87 (0.65-1.17) | 0.36 | . | . | . | 243 | 20,07 | 90 | 16,85 | 1473 | 414 |  |
| SLC19A1 ---- tag | rs3788190 | G/G | 1.00 (.-.) | . | 0.47 | 0.95 | 0.80 | 379 | 31,30 | 153 | 28,65 | 1473 | 414 |  |
| SLC19A1 ---- tag |  | G/A | 1.12 (0.90-1.40) | 0.31 | . | . | . | 570 | 47,07 | 286 | 53,56 | 1473 | 414 |  |
| SLC19A1 ---- tag |  | A/A | 0.86 (0.64-1.15) | 0.31 | . | . | . | 262 | 21,64 | 95 | 17,79 | 1473 | 414 |  |
| SLC19A1 ---- tag | rs3788205 | C/C | 1.00 (.-.) | . | 0.60 | 0.97 | 0.80 | 593 | 48,97 | 278 | 52,06 | 1473 | 414 |  |
| SLC19A1 ---- tag |  | C/T | 1.02 (0.83-1.25) | 0.89 | . | . | . | 525 | 43,35 | 216 | 40,45 | 1473 | 414 |  |
| SLC19A1 ---- tag |  | T/T | 0.85 (0.58-1.24) | 0.39 | . | . | . | 93 | 7,68 | 40 | 7,49 | 1473 | 414 |  |
| SLC19A1 ---- tag | rs7279664 | G/G | 1.00 (.-.) | . | 0.47 | 0.95 | 0.80 | 467 | 38,56 | 221 | 41,39 | 1473 | 414 |  |
| SLC19A1 ---- tag |  | G/T | 0.88 (0.71-1.08) | 0.23 | . | . | . | 581 | 47,98 | 232 | 43,45 | 1473 | 414 |  |
| SLC19A1 ---- tag |  | T/T | 0.95 (0.71-1.28) | 0.74 | . | . | . | 163 | 13,46 | 81 | 15,17 | 1473 | 414 |  |
| SLC29A1 ---- NA | rs1057985 | C/C | 1.00 (.-.) | . | 1.00 | 1.00 | 1.00 | 511 | 42,20 | 238 | 44,57 | 1473 | 414 |  |
| SLC29A1 ---- NA |  | C/T | 1.05 (0.85-1.30) | 0.63 | . | . | . | 544 | 44,92 | 234 | 43,82 | 1473 | 414 |  |
| SLC29A1 ---- NA |  | T/T | 0.96 (0.69-1.32) | 0.78 | . | . | . | 156 | 12,88 | 62 | 11,61 | 1473 | 414 |  |
| SLC29A1 ---- NA | rs6458375 | C/C | 1.00 (.-.) | . | 0.10 | 0.86 | 0.69 | 675 | 55,74 | 310 | 58,05 | 1473 | 414 |  |
| SLC29A1 ---- NA |  | C/T | 0.85 (0.69-1.04) | 0.11 | . | . | . | 458 | 37,82 | 194 | 36,33 | 1473 | 414 |  |
| SLC29A1 ---- NA |  | T/T | 0.81 (0.51-1.28) | 0.37 | . | . | . | 78 | 6,44 | 30 | 5,62 | 1473 | 414 |  |
| SLC29A1 ---- NA | rs666462 | C/C | 1.00 (.-.) | . | 0.65 | 0.97 | 1.00 | 334 | 27,58 | 148 | 27,72 | 1473 | 414 |  |
| SLC29A1 ---- NA |  | C/T | 1.04 (0.82-1.31) | 0.74 | . | . | . | 602 | 49,71 | 261 | 48,88 | 1473 | 414 |  |
| SLC29A1 ---- NA |  | T/T | 1.07 (0.81-1.40) | 0.65 | . | . | . | 275 | 22,71 | 125 | 23,41 | 1473 | 414 |  |
| SLC29A1 ---- NA | rs6905285 | A/A | 1.00 (.-.) | . | 0.92 | 0.99 | 1.00 | 451 | 37,24 | 186 | 34,83 | 1473 | 414 |  |
| SLC29A1 ---- NA |  | A/T | 0.93 (0.75-1.15) | 0.49 | . | . | . | 571 | 47,15 | 261 | 48,88 | 1473 | 414 |  |
| SLC29A1 ---- NA |  | T/T | 1.05 (0.79-1.40) | 0.73 | . | . | . | 189 | 15,61 | 87 | 16,29 | 1473 | 414 |  |
| SLC29A1 ---- NA | rs693955 | G/G | 1.00 (.-.) | . | 0.55 | 0.97 | 1.00 | 801 | 66,14 | 353 | 66,10 | 1473 | 414 |  |
| SLC29A1 ---- NA |  | G/T | 1.09 (0.88-1.34) | 0.45 | . | . | . | 363 | 29,98 | 161 | 30,15 | 1473 | 414 |  |
| SLC29A1 ---- NA |  | T/T | 1.01 (0.57-1.77) | 0.98 | . | . | . | 47 | 3,88 | 20 | 3,75 | 1473 | 414 |  |
| SLC29A1 ---- NA | rs747199 | C/C | 1.00 (.-.) | . | 0.81 | 0.99 | 1.00 | 782 | 64,57 | 349 | 65,36 | 1473 | 414 |  |
| SLC29A1 ---- NA |  | C/G | 0.99 (0.80-1.22) | 0.91 | . | . | . | 383 | 31,63 | 165 | 30,90 | 1473 | 414 |  |
| SLC29A1 ---- NA |  | G/G | 0.93 (0.56-1.55) | 0.79 | . | . | . | 46 | 3,80 | 20 | 3,75 | 1473 | 414 |  |
| SLC29A1 ---- NA | rs9357436 | G/G | 1.00 (.-.) | . | 0.95 | 0.99 | 1.00 | 845 | 69,78 | 381 | 71,35 | 1473 | 414 |  |
| SLC29A1 ---- NA |  | G/A | 1.07 (0.86-1.34) | 0.53 | . | . | . | 334 | 27,58 | 139 | 26,03 | 1473 | 414 |  |
| SLC29A1 ---- NA |  | A/A | 0.75 (0.41-1.39) | 0.36 | . | . | . | 32 | 2,64 | 14 | 2,62 | 1473 | 414 |  |
| TCN2 ---- tag | rs10418 | C/C | 1.00 (.-.) | . | 0.38 | 0.94 | 0.70 | 712 | 58,79 | 299 | 55,99 | 1473 | 414 |  |
| TCN2 ---- tag |  | C/T | 1.06 (0.87-1.30) | 0.56 | . | . | . | 442 | 36,50 | 205 | 38,39 | 1473 | 414 |  |
| TCN2 ---- tag |  | T/T | 1.19 (0.78-1.83) | 0.42 | . | . | . | 57 | 4,71 | 30 | 5,62 | 1473 | 414 |  |
| TCN2 ---- candidate/singleton | rs1131603 | T/T | 1.00 (.-.) | . | 0.92 | 0.99 | 0.95 | 1076 | 88,85 | 468 | 87,64 | 1473 | 414 | low_count |
| TCN2 ---- candidate/singleton |  | T/C | 1.10 (0.82-1.48) | 0.52 | . | . | . | 128 | 10,57 | 64 | 11,99 | 1473 | 414 |  |
| TCN2 ---- candidate/singleton |  | C/C | 0.35 (0.05-2.49) | 0.29 | . | . | . | 7 | 0,58 | 2 | 0,37 | 1473 | 414 |  |
| TCN2 ---- tag | rs1544468 | A/A | 1.00 (.-.) | . | 0.28 | 0.91 | 0.61 | 308 | 25,43 | 129 | 24,16 | 1473 | 414 |  |
| TCN2 ---- tag |  | A/G | 0.96 (0.75-1.22) | 0.72 | . | . | . | 613 | 50,62 | 262 | 49,06 | 1473 | 414 |  |
| TCN2 ---- tag |  | G/G | 1.15 (0.88-1.51) | 0.29 | . | . | . | 290 | 23,95 | 143 | 26,78 | 1473 | 414 |  |
| TCN2 ---- candidate/tag | rs1801198 | C/C | 1.00 (.-.) | . | 0.12 | 0.86 | 0.60 | 354 | 29,23 | 172 | 32,21 | 1473 | 414 |  |
| TCN2 ---- candidate/tag |  | C/G | 0.82 (0.65-1.02) | 0.07 | . | . | . | 600 | 49,55 | 256 | 47,94 | 1473 | 414 |  |
| TCN2 ---- candidate/tag |  | G/G | 0.82 (0.63-1.08) | 0.16 | . | . | . | 257 | 21,22 | 106 | 19,85 | 1473 | 414 |  |
| TCN2 ---- tag | rs4820872 | G/G | 1.00 (.-.) | . | 0.14 | 0.86 | 0.60 | 441 | 36,42 | 204 | 38,20 | 1473 | 414 |  |
| TCN2 ---- tag |  | G/A | 0.93 (0.74-1.15) | 0.49 | . | . | . | 537 | 44,34 | 232 | 43,45 | 1473 | 414 |  |
| TCN2 ---- tag |  | A/A | 0.81 (0.62-1.07) | 0.14 | . | . | . | 233 | 19,24 | 98 | 18,35 | 1473 | 414 |  |
| TCN2 ---- tag | rs4820874 | A/A | 1.00 (.-.) | . | 0.95 | 0.99 | 0.95 | 853 | 70,44 | 388 | 72,66 | 1473 | 414 |  |
| TCN2 ---- tag |  | A/G | 1.03 (0.82-1.29) | 0.81 | . | . | . | 324 | 26,75 | 132 | 24,72 | 1473 | 414 |  |
| TCN2 ---- tag |  | G/G | 0.93 (0.49-1.77) | 0.82 | . | . | . | 34 | 2,81 | 14 | 2,62 | 1473 | 414 |  |
| TCN2 ---- tag | rs4820886 | T/T | 1.00 (.-.) | . | 0.70 | 0.98 | 0.93 | 963 | 79,52 | 438 | 82,02 | 1473 | 414 | low_count |
| TCN2 ---- tag |  | T/G | 1.07 (0.83-1.38) | 0.61 | . | . | . | 229 | 18,91 | 88 | 16,48 | 1473 | 414 |  |
| TCN2 ---- tag |  | G/G | 0.93 (0.34-2.50) | 0.88 | . | . | . | 19 | 1,57 | 8 | 1,50 | 1473 | 414 |  |
| TCN2 ---- candidate | rs4820889 | G/G | 1.00 (.-.) | . | 0.16 | 0.86 | 0.60 | 1126 | 92,98 | 504 | 94,38 | 1473 | 414 |  |
| TCN2 ---- candidate |  | G/A | 0.74 (0.49-1.12) | 0.16 | . | . | . | 85 | 7,02 | 30 | 5,62 | 1473 | 414 |  |
| TCN2 ---- tag | rs5997711 | C/C | 1.00 (.-.) | . | 0.20 | 0.87 | 0.60 | 386 | 31,87 | 188 | 35,21 | 1473 | 414 |  |
| TCN2 ---- tag |  | C/T | 0.85 (0.68-1.06) | 0.14 | . | . | . | 599 | 49,46 | 247 | 46,25 | 1473 | 414 |  |
| TCN2 ---- tag |  | T/T | 0.86 (0.65-1.13) | 0.29 | . | . | . | 226 | 18,66 | 99 | 18,54 | 1473 | 414 |  |
| TCN2 ---- tag | rs740234 | T/T | 1.00 (.-.) | . | 0.72 | 0.98 | 0.93 | 794 | 65,57 | 337 | 63,11 | 1473 | 414 |  |
| TCN2 ---- tag |  | T/C | 1.05 (0.85-1.29) | 0.64 | . | . | . | 378 | 31,21 | 176 | 32,96 | 1473 | 414 |  |
| TCN2 ---- tag |  | C/C | 1.00 (0.58-1.73) | 1.00 | . | . | . | 39 | 3,22 | 21 | 3,93 | 1473 | 414 |  |
| TCN2 ---- tag | rs740235 | G/G | 1.00 (.-.) | . | 0.23 | 0.90 | 0.60 | 418 | 34,52 | 166 | 31,09 | 1473 | 414 |  |
| TCN2 ---- tag |  | G/A | 1.00 (0.80-1.25) | 1.00 | . | . | . | 591 | 48,80 | 259 | 48,50 | 1473 | 414 |  |
| TCN2 ---- tag |  | A/A | 1.20 (0.92-1.58) | 0.18 | . | . | . | 202 | 16,68 | 109 | 20,41 | 1473 | 414 |  |
| TCN2 ---- candidate/singleton | rs9606756 | A/A | 1.00 (.-.) | . | 0.89 | 0.99 | 0.95 | 956 | 78,94 | 435 | 81,46 | 1473 | 414 | low_count |
| TCN2 ---- candidate/singleton |  | A/G | 1.02 (0.79-1.32) | 0.87 | . | . | . | 232 | 19,16 | 91 | 17,04 | 1473 | 414 |  |
| TCN2 ---- candidate/singleton |  | G/G | 0.69 (0.22-2.15) | 0.52 | . | . | . | 23 | 1,90 | 8 | 1,50 | 1473 | 414 |  |
| TCN2 ---- candidate | rs9621049 | C/C | 1.00 (.-.) | . | 0.70 | 0.98 | 0.93 | 963 | 79,52 | 438 | 82,02 | 1473 | 414 | low_count |
| TCN2 ---- candidate |  | C/T | 1.07 (0.83-1.38) | 0.61 | . | . | . | 229 | 18,91 | 88 | 16,48 | 1473 | 414 |  |
| TCN2 ---- candidate |  | T/T | 0.93 (0.34-2.50) | 0.88 | . | . | . | 19 | 1,57 | 8 | 1,50 | 1473 | 414 |  |
| TK1 ---- NA | rs1065769 | G/G | 1.00 (.-.) | . | 0.62 | 0.97 | 0.86 | 573 | 47,32 | 243 | 45,51 | 1473 | 414 |  |
| TK1 ---- NA |  | G/A | 1.23 (1.00-1.51) | 0.05 | . | . | . | 507 | 41,87 | 249 | 46,63 | 1473 | 414 |  |
| TK1 ---- NA |  | A/A | 0.70 (0.48-1.01) | 0.06 | . | . | . | 131 | 10,82 | 42 | 7,87 | 1473 | 414 |  |
| TK1 ---- NA | rs12232476 | G/G | 1.00 (.-.) | . | 0.65 | 0.97 | 0.86 | 1022 | 84,39 | 443 | 82,96 | 1473 | 414 | low_count |
| TK1 ---- NA |  | G/A | 1.02 (0.78-1.35) | 0.86 | . | . | . | 182 | 15,03 | 86 | 16,10 | 1473 | 414 |  |
| TK1 ---- NA |  | A/A | 1.49 (0.55-4.04) | 0.44 | . | . | . | 7 | 0,58 | 5 | 0,94 | 1473 | 414 |  |
| TK1 ---- tag | rs16970907 | G/G | 1.00 (.-.) | . | 0.76 | 0.98 | 0.87 | 1050 | 86,71 | 460 | 86,14 | 1473 | 414 | low_count |
| TK1 ---- tag |  | G/C | 1.27 (0.95-1.69) | 0.10 | . | . | . | 153 | 12,63 | 74 | 13,86 | 1473 | 414 |  |
| TK1 ---- tag |  | C/C | 0.00 (0.00-8E180) | 0.96 | . | . | . | 8 | 0,66 | 0 | 0,00 | 1473 | 414 |  |
| TK1 ---- tag | rs1811086 | C/C | 1.00 (.-.) | . | 0.60 | 0.97 | 0.86 | 1128 | 93,15 | 493 | 92,32 | 1473 | 414 | low_count |
| TK1 ---- tag |  | C/T | 1.07 (0.73-1.56) | 0.74 | . | . | . | 82 | 6,77 | 40 | 7,49 | 1473 | 414 |  |
| TK1 ---- tag |  | T/T | 2.45 (0.33-18.23) | 0.38 | . | . | . | 1 | 0,08 | 1 | 0,19 | 1473 | 414 |  |
| TK1 ---- tag | rs2292235 | C/C | 1.00 (.-.) | . | 0.26 | 0.90 | 0.86 | 379 | 31,30 | 178 | 33,33 | 1473 | 414 |  |
| TK1 ---- tag |  | C/A | 0.94 (0.75-1.17) | 0.56 | . | . | . | 571 | 47,15 | 256 | 47,94 | 1473 | 414 |  |
| TK1 ---- tag |  | A/A | 0.85 (0.64-1.13) | 0.26 | . | . | . | 261 | 21,55 | 100 | 18,73 | 1473 | 414 |  |
| TK1 ---- tag | rs2854701 | A/A | 1.00 (.-.) | . | 0.96 | 0.99 | 0.96 | 491 | 40,55 | 201 | 37,64 | 1473 | 414 |  |
| TK1 ---- tag |  | A/G | 1.30 (1.05-1.60) | 0.02 | . | . | . | 550 | 45,42 | 272 | 50,94 | 1473 | 414 |  |
| TK1 ---- tag |  | G/G | 0.79 (0.57-1.12) | 0.18 | . | . | . | 170 | 14,04 | 61 | 11,42 | 1473 | 414 |  |
| TK1 ---- tag | rs2854702 | G/G | 1.00 (.-.) | . | 0.42 | 0.94 | 0.86 | 941 | 77,70 | 401 | 75,09 | 1473 | 414 | low_count |
| TK1 ---- tag |  | G/A | 1.04 (0.82-1.32) | 0.75 | . | . | . | 251 | 20,73 | 123 | 23,03 | 1473 | 414 |  |
| TK1 ---- tag |  | A/A | 1.53 (0.75-3.10) | 0.24 | . | . | . | 19 | 1,57 | 10 | 1,87 | 1473 | 414 |  |
| TK1 ---- tag | rs9897765 | G/G | 1.00 (.-.) | . | 0.42 | 0.94 | 0.86 | 649 | 53,59 | 277 | 51,87 | 1473 | 414 |  |
| TK1 ---- tag |  | G/A | 1.07 (0.87-1.32) | 0.50 | . | . | . | 461 | 38,07 | 227 | 42,51 | 1473 | 414 |  |
| TK1 ---- tag |  | A/A | 0.69 (0.45-1.05) | 0.09 | . | . | . | 101 | 8,34 | 30 | 5,62 | 1473 | 414 |  |
| TYMP ---- NA | rs131815 | G/G | 1.00 (.-.) | . | 0.17 | 0.86 | 0.29 | 639 | 52,77 | 286 | 53,56 | 1473 | 414 |  |
| TYMP ---- NA |  | G/A | 1.08 (0.88-1.33) | 0.46 | . | . | . | 472 | 38,98 | 206 | 38,58 | 1473 | 414 |  |
| TYMP ---- NA |  | A/A | 1.30 (0.91-1.85) | 0.16 | . | . | . | 100 | 8,26 | 42 | 7,87 | 1473 | 414 |  |
| TYMP ---- tag | rs131816 | A/A | 1.00 (.-.) | . | 0.02 | 0.85 | 0.10 | 716 | 59,12 | 339 | 63,48 | 1473 | 414 |  |
| TYMP ---- tag |  | A/G | 0.79 (0.64-0.98) | 0.04 | . | . | . | 420 | 34,68 | 165 | 30,90 | 1473 | 414 |  |
| TYMP ---- tag |  | G/G | 0.70 (0.46-1.09) | 0.11 | . | . | . | 75 | 6,19 | 30 | 5,62 | 1473 | 414 |  |
| TYMP ---- NA | rs131817 | C/C | 1.00 (.-.) | . | 0.29 | 0.91 | 0.35 | 395 | 32,62 | 171 | 32,02 | 1473 | 414 |  |
| TYMP ---- NA |  | C/T | 1.14 (0.91-1.43) | 0.24 | . | . | . | 578 | 47,73 | 267 | 50,00 | 1473 | 414 |  |
| TYMP ---- NA |  | T/T | 1.15 (0.86-1.53) | 0.36 | . | . | . | 238 | 19,65 | 96 | 17,98 | 1473 | 414 |  |
| TYMP ---- NA | rs140521 | T/T | 1.00 (.-.) | . | 0.20 | 0.86 | 0.29 | 617 | 50,95 | 281 | 52,62 | 1473 | 414 |  |
| TYMP ---- NA |  | T/G | 0.86 (0.70-1.06) | 0.15 | . | . | . | 502 | 41,45 | 210 | 39,33 | 1473 | 414 |  |
| TYMP ---- NA |  | G/G | 0.88 (0.61-1.27) | 0.48 | . | . | . | 92 | 7,60 | 43 | 8,05 | 1473 | 414 |  |
| TYMP ---- NA | rs140522 | G/G | 1.00 (.-.) | . | 0.05 | 0.85 | 0.14 | 537 | 44,34 | 255 | 47,75 | 1473 | 414 |  |
| TYMP ---- NA |  | G/A | 0.84 (0.69-1.04) | 0.11 | . | . | . | 546 | 45,09 | 229 | 42,88 | 1473 | 414 |  |
| TYMP ---- NA |  | A/A | 0.75 (0.52-1.07) | 0.12 | . | . | . | 128 | 10,57 | 50 | 9,36 | 1473 | 414 |  |
| TYMP ---- NA | rs140524 | G/G | 1.00 (.-.) | . | 0.65 | 0.97 | 0.65 | 823 | 67,96 | 345 | 64,61 | 1473 | 414 |  |
| TYMP ---- NA |  | G/A | 1.08 (0.88-1.34) | 0.46 | . | . | . | 345 | 28,49 | 174 | 32,58 | 1473 | 414 |  |
| TYMP ---- NA |  | A/A | 0.94 (0.54-1.66) | 0.84 | . | . | . | 43 | 3,55 | 15 | 2,81 | 1473 | 414 |  |
| TYMS ---- candidate literature | rs1001761 | C/C | 1.00 (.-.) | . | 0.06 | 0.85 | 0.19 | 349 | 28,82 | 177 | 33,15 | 1473 | 414 |  |
| TYMS ---- candidate literature |  | C/T | 0.87 (0.70-1.09) | 0.22 | . | . | . | 620 | 51,20 | 264 | 49,44 | 1473 | 414 |  |
| TYMS ---- candidate literature |  | T/T | 0.76 (0.57-1.02) | 0.07 | . | . | . | 242 | 19,98 | 93 | 17,42 | 1473 | 414 |  |
| TYMS ---- candidate literature/tag | rs10502289 | T/T | 1.00 (.-.) | . | 0.18 | 0.86 | 0.27 | 743 | 61,35 | 357 | 66,85 | 1473 | 414 |  |
| TYMS ---- candidate literature/tag |  | T/A | 0.79 (0.63-0.98) | 0.03 | . | . | . | 414 | 34,19 | 151 | 28,28 | 1473 | 414 |  |
| TYMS ---- candidate literature/tag |  | A/A | 1.07 (0.69-1.68) | 0.76 | . | . | . | 54 | 4,46 | 26 | 4,87 | 1473 | 414 |  |
| TYMS ---- tag | rs15872 | C/C | 1.00 (.-.) | . | 0.40 | 0.94 | 0.49 | 551 | 45,50 | 267 | 50,00 | 1473 | 414 |  |
| TYMS ---- tag |  | C/T | 0.83 (0.68-1.02) | 0.08 | . | . | . | 544 | 44,92 | 216 | 40,45 | 1473 | 414 |  |
| TYMS ---- tag |  | T/T | 1.03 (0.73-1.47) | 0.85 | . | . | . | 116 | 9,58 | 51 | 9,55 | 1473 | 414 |  |
| TYMS ---- tag | rs2244500 | T/T | 1.00 (.-.) | . | 0.06 | 0.85 | 0.19 | 350 | 28,90 | 177 | 33,15 | 1473 | 414 |  |
| TYMS ---- tag |  | T/C | 0.88 (0.70-1.09) | 0.23 | . | . | . | 619 | 51,11 | 264 | 49,44 | 1473 | 414 |  |
| TYMS ---- tag |  | C/C | 0.76 (0.56-1.02) | 0.06 | . | . | . | 242 | 19,98 | 93 | 17,42 | 1473 | 414 |  |
| TYMS ---- tag | rs2741182 | G/G | 1.00 (.-.) | . | 0.44 | 0.94 | 0.49 | 739 | 61,02 | 323 | 60,49 | 1473 | 414 |  |
| TYMS ---- tag |  | G/C | 0.92 (0.75-1.14) | 0.45 | . | . | . | 409 | 33,77 | 182 | 34,08 | 1473 | 414 |  |
| TYMS ---- tag |  | C/C | 0.91 (0.59-1.39) | 0.66 | . | . | . | 63 | 5,20 | 29 | 5,43 | 1473 | 414 |  |
| TYMS ---- candidate literature | rs2847149 | G/G | 1.00 (.-.) | . | 0.06 | 0.85 | 0.19 | 349 | 28,82 | 177 | 33,15 | 1473 | 414 |  |
| TYMS ---- candidate literature |  | G/A | 0.87 (0.70-1.09) | 0.22 | . | . | . | 620 | 51,20 | 264 | 49,44 | 1473 | 414 |  |
| TYMS ---- candidate literature |  | A/A | 0.76 (0.57-1.02) | 0.07 | . | . | . | 242 | 19,98 | 93 | 17,42 | 1473 | 414 |  |
| TYMS ---- candidate literature | rs2853533 | G/G | 1.00 (.-.) | . | 0.69 | 0.98 | 0.69 | 914 | 75,47 | 393 | 73,60 | 1473 | 414 | low_count |
| TYMS ---- candidate literature |  | G/C | 1.03 (0.83-1.29) | 0.77 | . | . | . | 274 | 22,63 | 135 | 25,28 | 1473 | 414 |  |
| TYMS ---- candidate literature |  | C/C | 0.51 (0.19-1.38) | 0.19 | . | . | . | 23 | 1,90 | 6 | 1,12 | 1473 | 414 |  |
| TYMS ---- tag | rs495139 | C/C | 1.00 (.-.) | . | 0.11 | 0.86 | 0.26 | 415 | 34,27 | 162 | 30,34 | 1473 | 414 |  |
| TYMS ---- tag |  | C/G | 1.44 (1.15-1.80) | 0.00 | . | . | . | 590 | 48,72 | 278 | 52,06 | 1473 | 414 |  |
| TYMS ---- tag |  | G/G | 1.15 (0.85-1.55) | 0.36 | . | . | . | 206 | 17,01 | 94 | 17,60 | 1473 | 414 |  |
| TYMS ---- candidate literature | rs502396 | T/T | 1.00 (.-.) | . | 0.17 | 0.86 | 0.27 | 338 | 27,91 | 179 | 33,52 | 1473 | 414 |  |
| TYMS ---- candidate literature |  | T/C | 0.88 (0.71-1.09) | 0.24 | . | . | . | 617 | 50,95 | 254 | 47,57 | 1473 | 414 |  |
| TYMS ---- candidate literature |  | C/C | 0.83 (0.62-1.11) | 0.20 | . | . | . | 256 | 21,14 | 101 | 18,91 | 1473 | 414 |  |
| UMPH2 ---- tag | rs2291028 | A/A | 1.00 (.-.) | . | 0.25 | 0.90 | 0.61 | 521 | 43,02 | 221 | 41,39 | 1473 | 414 |  |
| UMPH2 ---- tag |  | A/G | 1.05 (0.85-1.29) | 0.67 | . | . | . | 533 | 44,01 | 244 | 45,69 | 1473 | 414 |  |
| UMPH2 ---- tag |  | G/G | 1.23 (0.90-1.68) | 0.20 | . | . | . | 157 | 12,96 | 69 | 12,92 | 1473 | 414 |  |
| UMPH2 ---- NA | rs4789143 | A/A | 1.00 (.-.) | . | 0.73 | 0.98 | 0.73 | 926 | 76,47 | 428 | 80,15 | 1473 | 414 | low_count |
| UMPH2 ---- NA |  | A/G | 0.92 (0.72-1.18) | 0.52 | . | . | . | 270 | 22,30 | 99 | 18,54 | 1473 | 414 |  |
| UMPH2 ---- NA |  | G/G | 1.45 (0.53-3.91) | 0.47 | . | . | . | 15 | 1,24 | 7 | 1,31 | 1473 | 414 |  |
| UMPH2 ---- NA | rs750844 | G/G | 1.00 (.-.) | . | 0.40 | 0.94 | 0.61 | 612 | 50,54 | 262 | 49,06 | 1473 | 414 |  |
| UMPH2 ---- NA |  | G/A | 1.01 (0.82-1.24) | 0.92 | . | . | . | 502 | 41,45 | 224 | 41,95 | 1473 | 414 |  |
| UMPH2 ---- NA |  | A/A | 1.22 (0.86-1.73) | 0.26 | . | . | . | 97 | 8,01 | 48 | 8,99 | 1473 | 414 |  |
| UMPK ---- tag | rs11582877 | C/C | 1.00 (.-.) | . | 0.89 | 0.99 | 0.98 | 893 | 73,74 | 381 | 71,35 | 1473 | 414 |  |
| UMPK ---- tag |  | C/T | 1.09 (0.87-1.36) | 0.46 | . | . | . | 281 | 23,20 | 141 | 26,40 | 1473 | 414 |  |
| UMPK ---- tag |  | T/T | 0.77 (0.40-1.50) | 0.44 | . | . | . | 37 | 3,06 | 12 | 2,25 | 1473 | 414 |  |
| UMPK ---- tag | rs2622903 | A/A | 1.00 (.-.) | . | 0.58 | 0.97 | 0.98 | 592 | 48,89 | 266 | 49,81 | 1473 | 414 |  |
| UMPK ---- tag |  | A/G | 0.98 (0.80-1.20) | 0.85 | . | . | . | 490 | 40,46 | 219 | 41,01 | 1473 | 414 |  |
| UMPK ---- tag |  | G/G | 0.88 (0.61-1.28) | 0.52 | . | . | . | 129 | 10,65 | 49 | 9,18 | 1473 | 414 |  |
| UMPK ---- tag | rs2820989 | C/C | 1.00 (.-.) | . | 0.77 | 0.98 | 0.98 | 365 | 30,14 | 167 | 31,27 | 1473 | 414 |  |
| UMPK ---- tag |  | C/G | 1.06 (0.84-1.32) | 0.63 | . | . | . | 589 | 48,64 | 250 | 46,82 | 1473 | 414 |  |
| UMPK ---- tag |  | G/G | 0.95 (0.72-1.25) | 0.69 | . | . | . | 257 | 21,22 | 117 | 21,91 | 1473 | 414 |  |
| UMPK ---- tag | rs6660321 | A/A | 1.00 (.-.) | . | 0.98 | 0.99 | 0.98 | 923 | 76,22 | 395 | 73,97 | 1473 | 414 | low_count |
| UMPK ---- tag |  | A/C | 1.06 (0.84-1.33) | 0.64 | . | . | . | 261 | 21,55 | 129 | 24,16 | 1473 | 414 |  |
| UMPK ---- tag |  | C/C | 0.79 (0.39-1.61) | 0.52 | . | . | . | 27 | 2,23 | 10 | 1,87 | 1473 | 414 |  |
| UMPK ---- tag | rs6690084 | T/T | 1.00 (.-.) | . | 0.26 | 0.90 | 0.98 | 1050 | 86,71 | 451 | 84,46 | 1473 | 414 | low_count |
| UMPK ---- tag |  | T/C | 1.26 (0.96-1.67) | 0.10 | . | . | . | 152 | 12,55 | 79 | 14,79 | 1473 | 414 |  |
| UMPK ---- tag |  | C/C | 0.72 (0.23-2.27) | 0.58 | . | . | . | 9 | 0,74 | 4 | 0,75 | 1473 | 414 |  |
| UMPS ---- NA | rs1162 | A/A | 1.00 (.-.) | . | 0.00 | 0.60 | 0.02 | 578 | 47,73 | 233 | 43,63 | 1473 | 414 |  |
| UMPS ---- NA |  | A/G | 1.22 (0.99-1.50) | 0.06 | . | . | . | 513 | 42,36 | 231 | 43,26 | 1473 | 414 |  |
| UMPS ---- NA |  | G/G | 1.57 (1.15-2.13) | 0.00 | . | . | . | 120 | 9,91 | 70 | 13,11 | 1473 | 414 |  |
| UMPS ---- tag | rs13146 | C/C | 1.00 (.-.) | . | 0.09 | 0.86 | 0.18 | 843 | 69,61 | 363 | 67,98 | 1473 | 414 |  |
| UMPS ---- tag |  | C/T | 1.06 (0.85-1.32) | 0.59 | . | . | . | 333 | 27,50 | 147 | 27,53 | 1473 | 414 |  |
| UMPS ---- tag |  | T/T | 1.71 (1.08-2.72) | 0.02 | . | . | . | 35 | 2,89 | 24 | 4,49 | 1473 | 414 |  |
| UMPS ---- tag | rs16835902 | C/C | 1.00 (.-.) | . | 0.59 | 0.97 | 0.71 | 392 | 32,37 | 178 | 33,33 | 1473 | 414 |  |
| UMPS ---- tag |  | C/G | 0.86 (0.69-1.07) | 0.18 | . | . | . | 572 | 47,23 | 266 | 49,81 | 1473 | 414 |  |
| UMPS ---- tag |  | G/G | 0.97 (0.73-1.29) | 0.82 | . | . | . | 247 | 20,40 | 90 | 16,85 | 1473 | 414 |  |
| UMPS ---- tag | rs17282057 | T/T | 1.00 (.-.) | . | 0.18 | 0.86 | 0.28 | 937 | 77,37 | 390 | 73,03 | 1473 | 414 |  |
| UMPS ---- tag |  | T/C | 1.13 (0.90-1.42) | 0.30 | . | . | . | 255 | 21,06 | 133 | 24,91 | 1473 | 414 |  |
| UMPS ---- tag |  | C/C | 1.53 (0.67-3.46) | 0.31 | . | . | . | 19 | 1,57 | 11 | 2,06 | 1473 | 414 |  |
| UMPS ---- tag | rs606552 | A/A | 1.00 (.-.) | . | 0.04 | 0.85 | 0.11 | 604 | 49,88 | 303 | 56,74 | 1473 | 414 |  |
| UMPS ---- tag |  | A/G | 0.81 (0.66-0.99) | 0.04 | . | . | . | 501 | 41,37 | 198 | 37,08 | 1473 | 414 |  |
| UMPS ---- tag |  | G/G | 0.78 (0.52-1.16) | 0.21 | . | . | . | 106 | 8,75 | 33 | 6,18 | 1473 | 414 |  |
| UMPS ---- tag | rs694897 | C/C | 1.00 (.-.) | . | 0.98 | 0.99 | 0.98 | 517 | 42,69 | 210 | 39,33 | 1473 | 414 |  |
| UMPS ---- tag |  | C/G | 0.95 (0.77-1.18) | 0.67 | . | . | . | 537 | 44,34 | 252 | 47,19 | 1473 | 414 |  |
| UMPS ---- tag |  | G/G | 1.04 (0.76-1.42) | 0.79 | . | . | . | 157 | 12,96 | 72 | 13,48 | 1473 | 414 |  |
| UNG ---- NA | rs1059262 | T/T | 1.00 (.-.) | . | 1.00 | 1.00 | 1.00 | 827 | 68,29 | 366 | 68,54 | 1473 | 414 |  |
| UNG ---- NA |  | T/G | 1.01 (0.81-1.26) | 0.92 | . | . | . | 340 | 28,08 | 154 | 28,84 | 1473 | 414 |  |
| UNG ---- NA |  | G/G | 0.96 (0.53-1.71) | 0.88 | . | . | . | 44 | 3,63 | 14 | 2,62 | 1473 | 414 |  |
| UNG ---- tag | rs2160603 | T/T | 1.00 (.-.) | . | 0.17 | 0.86 | 0.97 | 839 | 69,28 | 375 | 70,22 | 1473 | 414 |  |
| UNG ---- tag |  | T/C | 1.14 (0.91-1.43) | 0.26 | . | . | . | 331 | 27,33 | 139 | 26,03 | 1473 | 414 |  |
| UNG ---- tag |  | C/C | 1.27 (0.76-2.11) | 0.36 | . | . | . | 41 | 3,39 | 20 | 3,75 | 1473 | 414 |  |
| UNG ---- tag | rs246079 | A/A | 1.00 (.-.) | . | 0.65 | 0.97 | 0.97 | 385 | 31,79 | 170 | 31,84 | 1473 | 414 |  |
| UNG ---- tag |  | A/G | 1.08 (0.86-1.34) | 0.52 | . | . | . | 601 | 49,63 | 262 | 49,06 | 1473 | 414 |  |
| UNG ---- tag |  | G/G | 1.05 (0.79-1.41) | 0.72 | . | . | . | 225 | 18,58 | 102 | 19,10 | 1473 | 414 |  |
| UNG ---- NA | rs246085 | T/T | 1.00 (.-.) | . | 0.54 | 0.97 | 0.97 | 1066 | 88,03 | 478 | 89,51 | 1473 | 414 | low_count |
| UNG ---- NA |  | T/C | 0.88 (0.65-1.21) | 0.43 | . | . | . | 142 | 11,73 | 55 | 10,30 | 1473 | 414 |  |
| UNG ---- NA |  | C/C | 2.25 (0.31-16.33) | 0.42 | . | . | . | 3 | 0,25 | 1 | 0,19 | 1473 | 414 |  |
| UNG ---- NA | rs2569987 | A/A | 1.00 (.-.) | . | 0.45 | 0.94 | 0.97 | 832 | 68,70 | 370 | 69,29 | 1473 | 414 |  |
| UNG ---- NA |  | A/G | 1.01 (0.81-1.27) | 0.91 | . | . | . | 338 | 27,91 | 152 | 28,46 | 1473 | 414 |  |
| UNG ---- NA |  | G/G | 0.66 (0.36-1.21) | 0.18 | . | . | . | 41 | 3,39 | 12 | 2,25 | 1473 | 414 |  |
| UNG ---- tag | rs3219243 | T/T | 1.00 (.-.) | . | 0.90 | 0.99 | 1.00 | 778 | 64,24 | 336 | 62,92 | 1473 | 414 |  |
| UNG ---- tag |  | T/C | 0.89 (0.72-1.11) | 0.30 | . | . | . | 396 | 32,70 | 170 | 31,84 | 1473 | 414 |  |
| UNG ---- tag |  | C/C | 1.46 (0.92-2.30) | 0.11 | . | . | . | 37 | 3,06 | 28 | 5,24 | 1473 | 414 |  |

| **Gene** | **SNP** | **Genotype** | **HR (95%-CI)** | **p** | **FDR__p_** | **FDR_(byGene)_p_** | **NObsUsed** | **Events** | **LowCount** |
| --- | --- | --- | --- | --- | --- | --- | --- | --- | --- |
| AARS ---- tag | rs2070203 | 1 | 1.09 (0.95-1.25) | 0.24 | 0.90 | 0.24 | 1473 | 414 |  |
| AARS ---- tag | rs34087264 | 1 | 0.89 (0.77-1.02) | 0.09 | 0.86 | 0.19 | 1473 | 414 |  |
| ABCC4 ---- tag | rs10508023 | 1 | 0.96 (0.77-1.19) | 0.71 | 0.98 | 0.93 | 1473 | 414 | low_count |
| ABCC4 ---- tag | rs1059751 | 1 | 1.10 (0.95-1.26) | 0.20 | 0.86 | 0.78 | 1473 | 414 |  |
| ABCC4 ---- tag | rs11568643 | 1 | 0.97 (0.76-1.25) | 0.82 | 0.99 | 0.93 | 1473 | 414 | low_count |
| ABCC4 ---- NA | rs11568658 | 1 | 1.14 (0.73-1.78) | 0.57 | 0.97 | 0.93 | 1473 | 414 | low_count |
| ABCC4 ---- tag | rs12864049 | 1 | 1.09 (0.89-1.32) | 0.41 | 0.94 | 0.90 | 1473 | 414 |  |
| ABCC4 ---- tag | rs1628382 | 1 | 1.08 (0.91-1.28) | 0.39 | 0.94 | 0.90 | 1473 | 414 |  |
| ABCC4 ---- tag | rs1678354 | 1 | 1.08 (0.94-1.25) | 0.27 | 0.90 | 0.83 | 1473 | 414 |  |
| ABCC4 ---- tag | rs1678383 | 1 | 0.95 (0.75-1.20) | 0.65 | 0.97 | 0.93 | 1473 | 414 | low_count |
| ABCC4 ---- tag | rs1678395 | 1 | 0.79 (0.61-1.03) | 0.08 | 0.85 | 0.53 | 1473 | 414 | low_count |
| ABCC4 ---- tag | rs1678405 | 1 | 1.17 (1.01-1.36) | 0.04 | 0.85 | 0.38 | 1473 | 414 |  |
| ABCC4 ---- tag | rs17189540 | 1 | 1.16 (0.89-1.52) | 0.27 | 0.90 | 0.83 | 1473 | 414 | low_count |
| ABCC4 ---- tag | rs17235152 | 1 | 1.04 (0.86-1.26) | 0.69 | 0.98 | 0.93 | 1473 | 414 | low_count |
| ABCC4 ---- tag | rs17268122 | 1 | 0.93 (0.79-1.10) | 0.40 | 0.94 | 0.90 | 1473 | 414 |  |
| ABCC4 ---- tag | rs17268170 | 1 | 1.03 (0.82-1.29) | 0.83 | 0.99 | 0.93 | 1473 | 414 | low_count |
| ABCC4 ---- tag | rs1729764 | 1 | 0.95 (0.75-1.21) | 0.68 | 0.98 | 0.93 | 1473 | 414 | low_count |
| ABCC4 ---- tag | rs1729767 | 1 | 1.09 (0.94-1.27) | 0.27 | 0.90 | 0.83 | 1473 | 414 |  |
| ABCC4 ---- tag | rs17300935 | 1 | 0.98 (0.80-1.19) | 0.82 | 0.99 | 0.93 | 1473 | 414 | low_count |
| ABCC4 ---- tag | rs1750190 | 1 | 1.03 (0.90-1.18) | 0.66 | 0.97 | 0.93 | 1473 | 414 |  |
| ABCC4 ---- tag | rs1750996 | 1 | 1.07 (0.89-1.28) | 0.50 | 0.96 | 0.93 | 1473 | 414 |  |
| ABCC4 ---- tag | rs1751025 | 1 | 1.16 (1.00-1.34) | 0.04 | 0.85 | 0.38 | 1473 | 414 |  |
| ABCC4 ---- tag | rs1751051 | 1 | 0.99 (0.85-1.15) | 0.87 | 0.99 | 0.93 | 1473 | 414 |  |
| ABCC4 ---- tag | rs1764416 | 1 | 0.93 (0.69-1.25) | 0.62 | 0.97 | 0.93 | 1473 | 414 | low_count |
| ABCC4 ---- tag | rs2274401 | 1 | 0.95 (0.80-1.13) | 0.59 | 0.97 | 0.93 | 1473 | 414 |  |
| ABCC4 ---- tag | rs2892716 | 1 | 1.06 (0.92-1.22) | 0.43 | 0.94 | 0.90 | 1473 | 414 |  |
| ABCC4 ---- tag | rs3782964 | 1 | 0.90 (0.74-1.10) | 0.32 | 0.94 | 0.88 | 1473 | 414 |  |
| ABCC4 ---- tag | rs3818494 | 1 | 1.23 (1.07-1.42) | 0.00 | 0.60 | 0.21 | 1473 | 414 |  |
| ABCC4 ---- tag | rs3864997 | 1 | 0.99 (0.86-1.14) | 0.87 | 0.99 | 0.93 | 1473 | 414 |  |
| ABCC4 ---- tag | rs4148421 | 1 | 0.96 (0.83-1.10) | 0.54 | 0.97 | 0.93 | 1473 | 414 |  |
| ABCC4 ---- tag | rs4148446 | 1 | 1.01 (0.88-1.17) | 0.86 | 0.99 | 0.93 | 1473 | 414 |  |
| ABCC4 ---- tag | rs4148455 | 1 | 1.14 (0.94-1.39) | 0.19 | 0.86 | 0.78 | 1473 | 414 | low_count |
| ABCC4 ---- tag | rs4148540 | 1 | 0.97 (0.75-1.25) | 0.82 | 0.99 | 0.93 | 1473 | 414 | low_count |
| ABCC4 ---- tag | rs4148542 | 1 | 0.98 (0.85-1.12) | 0.75 | 0.98 | 0.93 | 1473 | 414 |  |
| ABCC4 ---- tag | rs4148544 | 1 | 0.91 (0.78-1.05) | 0.19 | 0.86 | 0.78 | 1473 | 414 |  |
| ABCC4 ---- tag | rs4283094 | 1 | 0.99 (0.86-1.14) | 0.89 | 0.99 | 0.93 | 1473 | 414 |  |
| ABCC4 ---- tag | rs4636781 | 1 | 1.26 (1.05-1.51) | 0.02 | 0.85 | 0.38 | 1473 | 414 |  |
| ABCC4 ---- tag | rs4771910 | 1 | 0.96 (0.82-1.11) | 0.56 | 0.97 | 0.93 | 1473 | 414 |  |
| ABCC4 ---- tag | rs4773850 | 1 | 0.85 (0.74-0.99) | 0.03 | 0.85 | 0.38 | 1473 | 414 |  |
| ABCC4 ---- tag | rs7981095 | 1 | 0.90 (0.75-1.08) | 0.26 | 0.90 | 0.83 | 1473 | 414 |  |
| ABCC4 ---- tag | rs8001444 | 1 | 0.95 (0.82-1.09) | 0.46 | 0.95 | 0.92 | 1473 | 414 |  |
| ABCC4 ---- tag | rs931111 | 1 | 1.03 (0.88-1.21) | 0.71 | 0.98 | 0.93 | 1473 | 414 |  |
| ABCC4 ---- tag | rs943288 | 1 | 1.20 (0.98-1.46) | 0.08 | 0.85 | 0.53 | 1473 | 414 |  |
| ABCC4 ---- tag | rs943290 | 1 | 0.94 (0.80-1.10) | 0.43 | 0.94 | 0.90 | 1473 | 414 |  |
| ABCC4 ---- tag | rs9516530 | 1 | 1.01 (0.87-1.18) | 0.89 | 0.99 | 0.93 | 1473 | 414 |  |
| ABCC4 ---- tag | rs9516551 | 1 | 0.90 (0.72-1.12) | 0.34 | 0.94 | 0.88 | 1473 | 414 | low_count |
| ABCC4 ---- tag | rs9524822 | 1 | 0.91 (0.76-1.09) | 0.30 | 0.92 | 0.87 | 1473 | 414 |  |
| ABCC4 ---- tag | rs9524861 | 1 | 0.89 (0.77-1.04) | 0.14 | 0.86 | 0.72 | 1473 | 414 |  |
| ABCC4 ---- tag | rs9524902 | 1 | 0.96 (0.83-1.10) | 0.55 | 0.97 | 0.93 | 1473 | 414 |  |
| ABCC4 ---- tag | rs9556455 | 1 | 0.99 (0.82-1.21) | 0.96 | 0.99 | 0.96 | 1473 | 414 | low_count |
| ABCC4 ---- NA | rs9561778 | 1 | 0.99 (0.83-1.18) | 0.89 | 0.99 | 0.93 | 1473 | 414 |  |
| ABCC4 ---- tag | rs9561811 | 1 | 1.14 (0.96-1.36) | 0.13 | 0.86 | 0.72 | 1473 | 414 |  |
| ABCC4 ---- tag | rs9590183 | 1 | 0.72 (0.53-0.99) | 0.04 | 0.85 | 0.38 | 1473 | 414 | low_count |
| ABCC4 ---- tag | rs997777 | 1 | 1.01 (0.87-1.17) | 0.92 | 0.99 | 0.94 | 1473 | 414 |  |
| ADH1B ---- tag | rs1159918 | 1 | 1.01 (0.87-1.17) | 0.89 | 0.99 | 0.89 | 1473 | 414 |  |
| ADH1B ---- candidate literature | rs1229984 | 1 | 0.73 (0.51-1.03) | 0.07 | 0.85 | 0.37 | 1473 | 414 | low_count |
| ADH1B ---- tag | rs12507573 | 1 | 0.96 (0.83-1.10) | 0.56 | 0.97 | 0.71 | 1473 | 414 |  |
| ADH1B ---- tag | rs1693457 | 1 | 0.87 (0.72-1.05) | 0.15 | 0.86 | 0.37 | 1473 | 414 |  |
| ADH1B ---- tag | rs2066701 | 1 | 1.07 (0.92-1.25) | 0.35 | 0.94 | 0.58 | 1473 | 414 |  |
| ADH1C ---- tag | rs11936869 | 1 | 1.00 (0.86-1.16) | 0.99 | 1.00 | 0.99 | 1473 | 414 |  |
| ADH1C ---- tag | rs1229849 | 1 | 1.13 (0.97-1.33) | 0.12 | 0.86 | 0.53 | 1473 | 414 |  |
| ADH1C ---- tag | rs1229863 | 1 | 1.02 (0.84-1.23) | 0.83 | 0.99 | 0.98 | 1473 | 414 |  |
| ADH1C ---- tag | rs1229980 | 1 | 1.29 (0.97-1.71) | 0.08 | 0.85 | 0.53 | 1473 | 414 | low_count |
| ADH1C ---- candidate | rs1693482 | 1 | 1.05 (0.91-1.21) | 0.48 | 0.95 | 0.84 | 1473 | 414 |  |
| ADH1C ---- tag | rs2173201 | 1 | 1.01 (0.86-1.19) | 0.87 | 0.99 | 0.98 | 1473 | 414 |  |
| ADH1C ---- tag | rs2298753 | 1 | 0.88 (0.69-1.11) | 0.28 | 0.90 | 0.83 | 1473 | 414 | low_count |
| ADH1C ---- tag | rs2866152 | 1 | 1.05 (0.89-1.24) | 0.56 | 0.97 | 0.84 | 1473 | 414 |  |
| ADH1C ---- tag | rs904096 | 1 | 1.05 (0.91-1.21) | 0.49 | 0.96 | 0.84 | 1473 | 414 |  |
| BHMT ---- tag | rs10944 | 1 | 1.01 (0.88-1.17) | 0.86 | 0.99 | 0.98 | 1473 | 414 |  |
| BHMT ---- tag | rs12655567 | 1 | 0.92 (0.79-1.06) | 0.26 | 0.90 | 0.87 | 1473 | 414 |  |
| BHMT ---- tag | rs1291041 | 1 | 0.93 (0.80-1.09) | 0.37 | 0.94 | 0.87 | 1473 | 414 |  |
| BHMT ---- tag | rs16876500 | 1 | 1.04 (0.83-1.29) | 0.76 | 0.98 | 0.98 | 1473 | 414 | low_count |
| BHMT ---- tag | rs492842 | 1 | 1.00 (0.87-1.15) | 0.98 | 0.99 | 0.98 | 1473 | 414 |  |
| BHMT ---- tag | rs558133 | 1 | 0.93 (0.80-1.08) | 0.35 | 0.94 | 0.87 | 1473 | 414 |  |
| BHMT ---- tag | rs9637824 | 1 | 1.01 (0.87-1.16) | 0.93 | 0.99 | 0.98 | 1473 | 414 |  |
| BHMT2 ---- tag | rs16876512 | 1 | 1.03 (0.82-1.28) | 0.82 | 0.99 | 0.96 | 1473 | 414 | low_count |
| BHMT2 ---- tag | rs2461248 | 1 | 1.00 (0.87-1.16) | 0.96 | 0.99 | 0.96 | 1473 | 414 |  |
| BHMT2 ---- tag | rs2909856 | 1 | 1.01 (0.87-1.16) | 0.94 | 0.99 | 0.96 | 1473 | 414 |  |
| BHMT2 ---- tag | rs476620 | 1 | 1.01 (0.87-1.17) | 0.89 | 0.99 | 0.96 | 1473 | 414 |  |
| BHMT2 ---- candidate literature | rs626105 | 1 | 0.90 (0.75-1.07) | 0.23 | 0.90 | 0.96 | 1473 | 414 |  |
| BHMT2 ---- tag | rs631305 | 1 | 0.92 (0.76-1.12) | 0.41 | 0.94 | 0.96 | 1473 | 414 |  |
| CBS ---- tag | rs11701048 | 1 | 0.92 (0.70-1.22) | 0.57 | 0.97 | 0.92 | 1473 | 414 | low_count |
| CBS ---- tag | rs234706 | 1 | 0.99 (0.86-1.15) | 0.91 | 0.99 | 0.97 | 1473 | 414 |  |
| CBS ---- tag | rs234711 | 1 | 1.10 (0.93-1.30) | 0.26 | 0.90 | 0.75 | 1473 | 414 |  |
| CBS ---- candidate literature | rs234713 | 1 | 1.04 (0.89-1.21) | 0.64 | 0.97 | 0.92 | 1473 | 414 |  |
| CBS ---- tag | rs2839623 | 1 | 1.04 (0.82-1.33) | 0.74 | 0.98 | 0.92 | 1473 | 414 | low_count |
| CBS ---- tag | rs2839626 | 1 | 1.08 (0.93-1.24) | 0.30 | 0.92 | 0.75 | 1473 | 414 |  |
| CBS ---- tag | rs422791 | 1 | 1.08 (0.93-1.25) | 0.30 | 0.92 | 0.75 | 1473 | 414 |  |
| CBS ---- tag | rs706209 | 1 | 1.00 (0.87-1.15) | 0.97 | 0.99 | 0.97 | 1473 | 414 |  |
| CBS ---- tag | rs719037 | 1 | 1.13 (0.98-1.30) | 0.09 | 0.86 | 0.75 | 1473 | 414 |  |
| CBS ---- tag | rs719038 | 1 | 1.07 (0.93-1.23) | 0.37 | 0.94 | 0.75 | 1473 | 414 |  |
| DHFR ---- tag | rs10474632 | 1 | 0.90 (0.70-1.16) | 0.42 | 0.94 | 0.60 | 1473 | 414 | low_count |
| DHFR ---- tag | rs11951910 | 1 | 1.05 (0.85-1.30) | 0.66 | 0.98 | 0.66 | 1473 | 414 | low_count |
| DHFR ---- tag | rs1643665 | 1 | 0.92 (0.79-1.07) | 0.28 | 0.90 | 0.60 | 1473 | 414 |  |
| DHFR ---- tag | rs1650717 | 1 | 1.07 (0.92-1.23) | 0.40 | 0.94 | 0.60 | 1473 | 414 |  |
| DHFR ---- tag | rs1805355 | 1 | 1.24 (0.94-1.62) | 0.12 | 0.86 | 0.60 | 1473 | 414 | low_count |
| DHFR ---- tag | rs6151617 | 1 | 0.92 (0.80-1.06) | 0.26 | 0.90 | 0.60 | 1473 | 414 |  |
| DHFR ---- tag | rs6864493 | 1 | 0.86 (0.73-1.01) | 0.07 | 0.85 | 0.60 | 1473 | 414 |  |
| DHFR ---- tag | rs836788 | 1 | 1.04 (0.90-1.20) | 0.59 | 0.97 | 0.66 | 1473 | 414 |  |
| DHFR ---- tag | rs836790 | 1 | 1.09 (0.91-1.32) | 0.34 | 0.94 | 0.60 | 1473 | 414 |  |
| DHFR ---- tag | rs836817 | 1 | 1.04 (0.90-1.20) | 0.59 | 0.97 | 0.66 | 1473 | 414 |  |
| DNMT1 ---- candidate | rs2228612 | 1 | 1.05 (0.80-1.37) | 0.73 | 0.98 | 0.73 | 1473 | 414 | low_count |
| DNMT3A ---- tag | rs10460566 | 1 | 0.95 (0.80-1.12) | 0.56 | 0.97 | 0.72 | 1473 | 414 |  |
| DNMT3A ---- candidate literature | rs11695471 | 1 | 1.04 (0.90-1.20) | 0.56 | 0.97 | 0.72 | 1473 | 414 |  |
| DNMT3A ---- tag | rs11887120 | 1 | 0.95 (0.83-1.09) | 0.44 | 0.94 | 0.72 | 1473 | 414 |  |
| DNMT3A ---- tag | rs12991495 | 1 | 1.09 (0.94-1.26) | 0.24 | 0.90 | 0.72 | 1473 | 414 |  |
| DNMT3A ---- tag | rs13401241 | 1 | 1.00 (0.86-1.15) | 0.96 | 0.99 | 0.96 | 1473 | 414 |  |
| DNMT3A ---- candidate literature | rs13420827 | 1 | 0.92 (0.78-1.10) | 0.36 | 0.94 | 0.72 | 1473 | 414 |  |
| DNMT3A ---- tag | rs13428812 | 1 | 1.04 (0.89-1.21) | 0.63 | 0.97 | 0.72 | 1473 | 414 |  |
| DNMT3A ---- tag | rs4665287 | 1 | 0.91 (0.76-1.08) | 0.27 | 0.90 | 0.72 | 1473 | 414 |  |
| DNMT3B ---- tag | rs13045669 | 1 | 0.69 (0.44-1.08) | 0.11 | 0.86 | 0.32 | 1473 | 414 | low_count |
| DNMT3B ---- tag | rs17123673 | 1 | 1.01 (0.74-1.39) | 0.95 | 0.99 | 0.95 | 1473 | 414 | low_count |
| DNMT3B ---- tag | rs183603 | 1 | 0.85 (0.72-1.00) | 0.05 | 0.85 | 0.26 | 1473 | 414 |  |
| DNMT3B ---- tag | rs2235760 | 1 | 1.06 (0.89-1.27) | 0.52 | 0.97 | 0.65 | 1473 | 414 |  |
| DNMT3B ---- tag | rs2424908 | 1 | 0.82 (0.69-0.99) | 0.04 | 0.85 | 0.26 | 1473 | 414 |  |
| DNMT3B ---- candidate literature | rs2424909 | 1 | 0.90 (0.79-1.04) | 0.15 | 0.86 | 0.32 | 1473 | 414 |  |
| DNMT3B ---- tag | rs4911108 | 1 | 0.90 (0.79-1.04) | 0.16 | 0.86 | 0.32 | 1473 | 414 |  |
| DNMT3B ---- tag | rs6058896 | 1 | 1.16 (0.87-1.56) | 0.32 | 0.94 | 0.48 | 1473 | 414 | low_count |
| DNMT3B ---- tag | rs6119954 | 1 | 1.03 (0.86-1.23) | 0.75 | 0.98 | 0.84 | 1473 | 414 |  |
| DNMT3B ---- tag | rs6579038 | 1 | 1.15 (0.86-1.55) | 0.33 | 0.94 | 0.48 | 1473 | 414 | low_count |
| DPYD ---- tag | rs1034215 | 1 | 1.06 (0.90-1.25) | 0.50 | 0.96 | 0.72 | 1473 | 414 |  |
| DPYD ---- tag | rs10783058 | 1 | 1.07 (0.93-1.23) | 0.32 | 0.94 | 0.68 | 1473 | 414 |  |
| DPYD ---- tag | rs10783070 | 1 | 1.23 (1.02-1.48) | 0.03 | 0.85 | 0.37 | 1473 | 414 |  |
| DPYD ---- tag | rs10875048 | 1 | 0.96 (0.80-1.15) | 0.63 | 0.97 | 0.74 | 1473 | 414 |  |
| DPYD ---- tag | rs10875055 | 1 | 1.13 (0.99-1.30) | 0.08 | 0.85 | 0.42 | 1473 | 414 |  |
| DPYD ---- tag | rs10875079 | 1 | 0.84 (0.73-0.96) | 0.01 | 0.85 | 0.37 | 1473 | 414 |  |
| DPYD ---- tag | rs10875085 | 1 | 1.11 (0.93-1.32) | 0.25 | 0.90 | 0.64 | 1473 | 414 |  |
| DPYD ---- tag | rs10875097 | 1 | 1.11 (0.93-1.32) | 0.26 | 0.90 | 0.64 | 1473 | 414 |  |
| DPYD ---- tag | rs11165781 | 1 | 1.01 (0.83-1.22) | 0.96 | 0.99 | 0.96 | 1473 | 414 |  |
| DPYD ---- tag | rs11165783 | 1 | 1.07 (0.92-1.24) | 0.41 | 0.94 | 0.72 | 1473 | 414 |  |
| DPYD ---- tag | rs11165873 | 1 | 0.89 (0.78-1.03) | 0.12 | 0.86 | 0.56 | 1473 | 414 |  |
| DPYD ---- tag | rs11165875 | 1 | 1.10 (0.96-1.27) | 0.16 | 0.86 | 0.57 | 1473 | 414 |  |
| DPYD ---- tag | rs11165881 | 1 | 1.03 (0.90-1.18) | 0.70 | 0.98 | 0.80 | 1473 | 414 |  |
| DPYD ---- tag | rs11587873 | 1 | 0.82 (0.69-0.98) | 0.03 | 0.85 | 0.37 | 1473 | 414 |  |
| DPYD ---- tag | rs12030174 | 1 | 1.08 (0.89-1.31) | 0.45 | 0.95 | 0.72 | 1473 | 414 |  |
| DPYD ---- tag | rs12046744 | 1 | 0.91 (0.78-1.07) | 0.25 | 0.90 | 0.64 | 1473 | 414 |  |
| DPYD ---- tag | rs12047910 | 1 | 0.94 (0.77-1.15) | 0.56 | 0.97 | 0.72 | 1473 | 414 | low_count |
| DPYD ---- tag | rs12073044 | 1 | 1.09 (0.87-1.38) | 0.44 | 0.94 | 0.72 | 1473 | 414 | low_count |
| DPYD ---- tag | rs12126093 | 1 | 0.89 (0.76-1.04) | 0.15 | 0.86 | 0.57 | 1473 | 414 |  |
| DPYD ---- tag | rs12134028 | 1 | 0.78 (0.56-1.08) | 0.13 | 0.86 | 0.57 | 1473 | 414 | low_count |
| DPYD ---- tag | rs12740796 | 1 | 1.06 (0.87-1.30) | 0.55 | 0.97 | 0.72 | 1473 | 414 |  |
| DPYD ---- tag | rs1333717 | 1 | 1.06 (0.90-1.25) | 0.47 | 0.95 | 0.72 | 1473 | 414 |  |
| DPYD ---- tag | rs1413228 | 1 | 0.93 (0.74-1.17) | 0.55 | 0.97 | 0.72 | 1473 | 414 | low_count |
| DPYD ---- tag | rs1415681 | 1 | 1.04 (0.86-1.26) | 0.70 | 0.98 | 0.80 | 1473 | 414 |  |
| DPYD ---- tag | rs1514495 | 1 | 0.94 (0.80-1.11) | 0.47 | 0.95 | 0.72 | 1473 | 414 |  |
| DPYD ---- tag | rs1520658 | 1 | 1.08 (0.87-1.34) | 0.49 | 0.96 | 0.72 | 1473 | 414 | low_count |
| DPYD ---- NA | rs17116806 | 1 | 1.04 (0.88-1.24) | 0.62 | 0.97 | 0.74 | 1473 | 414 |  |
| DPYD ---- tag | rs17431828 | 1 | 0.99 (0.86-1.14) | 0.89 | 0.99 | 0.94 | 1473 | 414 |  |
| DPYD ---- tag | rs17471640 | 1 | 0.95 (0.82-1.11) | 0.52 | 0.97 | 0.72 | 1473 | 414 |  |
| DPYD ---- tag | rs17702702 | 1 | 1.01 (0.84-1.20) | 0.96 | 0.99 | 0.96 | 1473 | 414 |  |
| DPYD ---- NA | rs1801265 | 1 | 1.10 (0.93-1.29) | 0.25 | 0.90 | 0.64 | 1473 | 414 |  |
| DPYD ---- tag | rs2039447 | 1 | 1.08 (0.93-1.25) | 0.33 | 0.94 | 0.68 | 1473 | 414 |  |
| DPYD ---- tag | rs2151567 | 1 | 1.37 (1.01-1.85) | 0.04 | 0.85 | 0.37 | 1473 | 414 | low_count |
| DPYD ---- tag | rs2152878 | 1 | 1.07 (0.92-1.26) | 0.38 | 0.94 | 0.72 | 1473 | 414 |  |
| DPYD ---- tag | rs2786505 | 1 | 1.24 (1.01-1.52) | 0.04 | 0.85 | 0.37 | 1473 | 414 | low_count |
| DPYD ---- tag | rs2786512 | 1 | 0.96 (0.84-1.10) | 0.54 | 0.97 | 0.72 | 1473 | 414 |  |
| DPYD ---- tag | rs2786519 | 1 | 1.05 (0.89-1.23) | 0.58 | 0.97 | 0.73 | 1473 | 414 |  |
| DPYD ---- tag | rs2811170 | 1 | 0.86 (0.68-1.07) | 0.18 | 0.86 | 0.57 | 1473 | 414 | low_count |
| DPYD ---- tag | rs2811199 | 1 | 1.22 (1.01-1.48) | 0.04 | 0.85 | 0.37 | 1473 | 414 |  |
| DPYD ---- tag | rs2811219 | 1 | 0.99 (0.83-1.17) | 0.88 | 0.99 | 0.94 | 1473 | 414 |  |
| DPYD ---- tag | rs4300257 | 1 | 1.14 (0.96-1.36) | 0.15 | 0.86 | 0.57 | 1473 | 414 |  |
| DPYD ---- tag | rs4379706 | 1 | 1.09 (0.92-1.28) | 0.32 | 0.94 | 0.68 | 1473 | 414 |  |
| DPYD ---- tag | rs4950021 | 1 | 1.04 (0.91-1.20) | 0.56 | 0.97 | 0.72 | 1473 | 414 |  |
| DPYD ---- tag | rs4950033 | 1 | 1.07 (0.93-1.23) | 0.33 | 0.94 | 0.68 | 1473 | 414 |  |
| DPYD ---- tag | rs495257 | 1 | 1.06 (0.92-1.22) | 0.44 | 0.94 | 0.72 | 1473 | 414 |  |
| DPYD ---- tag | rs552926 | 1 | 1.02 (0.89-1.17) | 0.73 | 0.98 | 0.81 | 1473 | 414 |  |
| DPYD ---- tag | rs628959 | 1 | 1.07 (0.92-1.24) | 0.39 | 0.94 | 0.72 | 1473 | 414 |  |
| DPYD ---- tag | rs6656660 | 1 | 1.01 (0.83-1.23) | 0.92 | 0.99 | 0.96 | 1473 | 414 |  |
| DPYD ---- tag | rs6663670 | 1 | 1.22 (1.01-1.48) | 0.04 | 0.85 | 0.37 | 1473 | 414 |  |
| DPYD ---- tag | rs6683883 | 1 | 1.15 (1.00-1.32) | 0.05 | 0.85 | 0.37 | 1473 | 414 |  |
| DPYD ---- tag | rs6686861 | 1 | 1.07 (0.83-1.38) | 0.59 | 0.97 | 0.73 | 1473 | 414 | low_count |
| DPYD ---- tag | rs7414210 | 1 | 0.92 (0.75-1.13) | 0.43 | 0.94 | 0.72 | 1473 | 414 | low_count |
| DPYD ---- tag | rs7530858 | 1 | 1.13 (0.91-1.41) | 0.26 | 0.90 | 0.64 | 1473 | 414 | low_count |
| DPYD ---- tag | rs7544128 | 1 | 1.12 (0.95-1.30) | 0.17 | 0.86 | 0.57 | 1473 | 414 |  |
| DPYD ---- tag | rs7545340 | 1 | 1.02 (0.88-1.19) | 0.79 | 0.99 | 0.87 | 1473 | 414 |  |
| DPYD ---- tag | rs828054 | 1 | 0.88 (0.77-1.01) | 0.07 | 0.85 | 0.41 | 1473 | 414 |  |
| DPYD ---- tag | rs885622 | 1 | 1.14 (1.00-1.32) | 0.06 | 0.85 | 0.37 | 1473 | 414 |  |
| DPYD ---- tag | rs9437663 | 1 | 1.11 (0.94-1.32) | 0.23 | 0.90 | 0.64 | 1473 | 414 |  |
| DPYS ---- tag | rs13249169 | 1 | 1.01 (0.81-1.25) | 0.95 | 0.99 | 0.99 | 1473 | 414 | low_count |
| DPYS ---- NA | rs13263121 | 1 | 0.98 (0.85-1.13) | 0.79 | 0.99 | 0.99 | 1473 | 414 |  |
| DPYS ---- tag | rs16871361 | 1 | 0.89 (0.65-1.22) | 0.47 | 0.95 | 0.99 | 1473 | 414 | low_count |
| DPYS ---- NA | rs17245950 | 1 | 0.94 (0.75-1.18) | 0.61 | 0.97 | 0.99 | 1473 | 414 | low_count |
| DPYS ---- NA | rs2253336 | 1 | 0.84 (0.66-1.07) | 0.15 | 0.86 | 0.99 | 1473 | 414 | low_count |
| DPYS ---- tag | rs2280010 | 1 | 1.11 (0.95-1.30) | 0.19 | 0.86 | 0.99 | 1473 | 414 |  |
| DPYS ---- tag | rs2333874 | 1 | 0.99 (0.85-1.15) | 0.89 | 0.99 | 0.99 | 1473 | 414 |  |
| DPYS ---- NA | rs2669429 | 1 | 1.06 (0.93-1.22) | 0.39 | 0.94 | 0.99 | 1473 | 414 |  |
| DPYS ---- tag | rs2669434 | 1 | 0.96 (0.82-1.13) | 0.66 | 0.98 | 0.99 | 1473 | 414 |  |
| DPYS ---- tag | rs2853142 | 1 | 0.97 (0.84-1.13) | 0.73 | 0.98 | 0.99 | 1473 | 414 |  |
| DPYS ---- NA | rs2853145 | 1 | 0.96 (0.81-1.15) | 0.68 | 0.98 | 0.99 | 1473 | 414 |  |
| DPYS ---- tag | rs2853149 | 1 | 0.98 (0.85-1.13) | 0.78 | 0.99 | 0.99 | 1473 | 414 |  |
| DPYS ---- tag | rs2853154 | 1 | 1.00 (0.85-1.17) | 0.96 | 0.99 | 0.99 | 1473 | 414 |  |
| DPYS ---- tag | rs2853161 | 1 | 0.98 (0.85-1.14) | 0.83 | 0.99 | 0.99 | 1473 | 414 |  |
| DPYS ---- NA | rs2959024 | 1 | 1.06 (0.91-1.24) | 0.42 | 0.94 | 0.99 | 1473 | 414 |  |
| DPYS ---- NA | rs2959025 | 1 | 1.05 (0.91-1.21) | 0.51 | 0.96 | 0.99 | 1473 | 414 |  |
| DPYS ---- tag | rs2959026 | 1 | 1.00 (0.87-1.15) | 0.99 | 1.00 | 0.99 | 1473 | 414 |  |
| DPYS ---- NA | rs3133278 | 1 | 0.99 (0.85-1.16) | 0.92 | 0.99 | 0.99 | 1473 | 414 |  |
| DPYS ---- tag | rs3750187 | 1 | 0.98 (0.83-1.17) | 0.85 | 0.99 | 0.99 | 1473 | 414 |  |
| DPYS ---- tag | rs3793357 | 1 | 1.05 (0.78-1.43) | 0.74 | 0.98 | 0.99 | 1473 | 414 | low_count |
| DPYS ---- tag | rs3793358 | 1 | 0.93 (0.76-1.15) | 0.52 | 0.97 | 0.99 | 1473 | 414 |  |
| DPYS ---- tag | rs6468924 | 1 | 1.01 (0.84-1.20) | 0.95 | 0.99 | 0.99 | 1473 | 414 |  |
| DUT ---- tag | rs8025164 | 1 | 0.96 (0.79-1.17) | 0.67 | 0.98 | 0.67 | 1473 | 414 |  |
| EHMT1 ---- tag | rs10780190 | 1 | 0.99 (0.70-1.39) | 0.94 | 0.99 | 0.97 | 1473 | 414 | low_count |
| EHMT1 ---- tag | rs10867083 | 1 | 0.94 (0.81-1.10) | 0.45 | 0.94 | 0.94 | 1473 | 414 |  |
| EHMT1 ---- tag | rs11137190 | 1 | 1.02 (0.88-1.19) | 0.77 | 0.98 | 0.94 | 1473 | 414 |  |
| EHMT1 ---- tag | rs3123510 | 1 | 1.00 (0.86-1.15) | 0.97 | 0.99 | 0.97 | 1473 | 414 |  |
| EHMT1 ---- candidate literature | rs3125795 | 1 | 0.94 (0.67-1.33) | 0.74 | 0.98 | 0.94 | 1473 | 414 | low_count |
| EHMT1 ---- tag | rs4573359 | 1 | 0.85 (0.66-1.09) | 0.20 | 0.86 | 0.55 | 1473 | 414 | low_count |
| EHMT1 ---- candidate literature | rs4634736 | 1 | 0.84 (0.65-1.08) | 0.17 | 0.86 | 0.55 | 1473 | 414 | low_count |
| EHMT1 ---- tag | rs4876902 | 1 | 1.16 (0.98-1.37) | 0.09 | 0.86 | 0.55 | 1473 | 414 |  |
| EHMT1 ---- tag | rs4876904 | 1 | 0.92 (0.80-1.05) | 0.20 | 0.86 | 0.55 | 1473 | 414 |  |
| EHMT1 ---- tag | rs7390244 | 1 | 1.03 (0.90-1.17) | 0.69 | 0.98 | 0.94 | 1473 | 414 |  |
| EHMT1 ---- tag | rs9314635 | 1 | 1.03 (0.89-1.19) | 0.74 | 0.98 | 0.94 | 1473 | 414 |  |
| EHMT2 ---- candidate/tag | rs2736428 | 1 | 0.85 (0.73-0.98) | 0.02 | 0.85 | 0.05 | 1473 | 414 |  |
| EHMT2 ---- tag | rs9267649 | 1 | 0.97 (0.81-1.17) | 0.77 | 0.98 | 0.77 | 1473 | 414 |  |
| FDXR ---- NA | rs2070918 | 1 | 1.12 (0.97-1.29) | 0.12 | 0.86 | 0.49 | 1473 | 414 |  |
| FDXR ---- tag | rs509911 | 1 | 1.09 (0.93-1.28) | 0.30 | 0.92 | 0.59 | 1473 | 414 |  |
| FDXR ---- NA | rs689882 | 1 | 0.99 (0.85-1.15) | 0.92 | 0.99 | 0.92 | 1473 | 414 |  |
| FDXR ---- NA | rs689895 | 1 | 0.98 (0.84-1.13) | 0.75 | 0.98 | 0.92 | 1473 | 414 |  |
| FOLH1 ---- candidate literature | rs10839236 | 1 | 0.93 (0.80-1.07) | 0.33 | 0.94 | 0.60 | 1473 | 414 |  |
| FOLH1 ---- tag | rs16906190 | 1 | 0.86 (0.67-1.10) | 0.23 | 0.90 | 0.60 | 1473 | 414 | low_count |
| FOLH1 ---- candidate | rs202676 | 1 | 0.93 (0.78-1.11) | 0.42 | 0.94 | 0.60 | 1473 | 414 |  |
| FOLH1 ---- tag | rs202680 | 1 | 0.96 (0.82-1.13) | 0.64 | 0.97 | 0.71 | 1473 | 414 |  |
| FOLH1 ---- candidate literature | rs202720 | 1 | 0.93 (0.78-1.10) | 0.40 | 0.94 | 0.60 | 1473 | 414 |  |
| FOLH1 ---- tag | rs2299650 | 1 | 0.93 (0.80-1.07) | 0.29 | 0.92 | 0.60 | 1473 | 414 |  |
| FOLH1 ---- tag | rs617528 | 1 | 0.91 (0.73-1.12) | 0.37 | 0.94 | 0.60 | 1473 | 414 | low_count |
| FOLH1 ---- tag | rs663877 | 1 | 1.00 (0.81-1.24) | 0.98 | 0.99 | 0.98 | 1473 | 414 | low_count |
| FOLH1 ---- tag | rs670776 | 1 | 0.93 (0.78-1.11) | 0.42 | 0.94 | 0.60 | 1473 | 414 |  |
| FOLH1 ---- tag | rs7124497 | 1 | 1.11 (0.79-1.55) | 0.54 | 0.97 | 0.68 | 1473 | 414 | low_count |
| FOLR1 ---- tag | rs651646 | 1 | 0.88 (0.77-1.01) | 0.07 | 0.85 | 0.07 | 1473 | 414 |  |
| FPGS ---- tag | rs10987746 | 1 | 1.01 (0.88-1.17) | 0.86 | 0.99 | 0.92 | 1473 | 414 |  |
| FPGS ---- tag | rs7033913 | 1 | 0.96 (0.84-1.11) | 0.62 | 0.97 | 0.92 | 1473 | 414 |  |
| FPGS ---- tag | rs7039798 | 1 | 1.01 (0.87-1.16) | 0.92 | 0.99 | 0.92 | 1473 | 414 |  |
| GGH ---- tag | rs10957264 | 1 | 0.90 (0.75-1.09) | 0.28 | 0.90 | 0.50 | 1473 | 414 |  |
| GGH ---- candidate literature | rs11545076 | 1 | 0.90 (0.78-1.05) | 0.20 | 0.86 | 0.47 | 1473 | 414 |  |
| GGH ---- candidate | rs11545077 | 1 | 0.92 (0.79-1.08) | 0.31 | 0.92 | 0.50 | 1473 | 414 |  |
| GGH ---- candidate | rs11545078 | 1 | 0.94 (0.75-1.17) | 0.58 | 0.97 | 0.58 | 1473 | 414 | low_count |
| GGH ---- tag | rs11995525 | 1 | 1.14 (0.97-1.33) | 0.11 | 0.86 | 0.47 | 1473 | 414 |  |
| GGH ---- tag | rs16930073 | 1 | 0.93 (0.75-1.15) | 0.51 | 0.96 | 0.58 | 1473 | 414 | low_count |
| GGH ---- tag | rs17194931 | 1 | 0.94 (0.75-1.17) | 0.58 | 0.97 | 0.58 | 1473 | 414 | low_count |
| GGH ---- candidate literature | rs1800909 | 1 | 0.89 (0.77-1.04) | 0.14 | 0.86 | 0.47 | 1473 | 414 |  |
| GGH ---- candidate literature | rs3758149 | 1 | 0.90 (0.78-1.05) | 0.20 | 0.86 | 0.47 | 1473 | 414 |  |
| GGH ---- tag | rs3780130 | 1 | 0.89 (0.74-1.06) | 0.20 | 0.86 | 0.47 | 1473 | 414 |  |
| GGH ---- tag | rs4446729 | 1 | 1.05 (0.90-1.23) | 0.53 | 0.97 | 0.58 | 1473 | 414 |  |
| GGH ---- tag | rs6472067 | 1 | 1.09 (0.95-1.26) | 0.22 | 0.89 | 0.47 | 1473 | 414 |  |
| GGH ---- tag | rs7010484 | 1 | 1.05 (0.91-1.21) | 0.49 | 0.96 | 0.58 | 1473 | 414 |  |
| GNMT ---- tag | rs1053538 | 1 | 1.06 (0.92-1.22) | 0.41 | 0.94 | 0.55 | 1473 | 414 |  |
| GNMT ---- tag | rs2296805 | 1 | 0.94 (0.81-1.08) | 0.36 | 0.94 | 0.55 | 1473 | 414 |  |
| GNMT ---- tag | rs6901782 | 1 | 1.09 (0.89-1.32) | 0.41 | 0.94 | 0.55 | 1473 | 414 |  |
| GNMT ---- tag | rs6927188 | 1 | 1.00 (0.85-1.18) | 0.96 | 0.99 | 0.96 | 1473 | 414 |  |
| MAT1A ---- tag | rs10887708 | 1 | 1.01 (0.87-1.17) | 0.90 | 0.99 | 0.90 | 1473 | 414 |  |
| MAT1A ---- tag | rs10887718 | 1 | 1.14 (1.00-1.31) | 0.06 | 0.85 | 0.51 | 1473 | 414 |  |
| MAT1A ---- tag | rs11202403 | 1 | 0.94 (0.79-1.12) | 0.47 | 0.95 | 0.61 | 1473 | 414 |  |
| MAT1A ---- tag | rs1832683 | 1 | 0.91 (0.76-1.09) | 0.29 | 0.91 | 0.59 | 1473 | 414 |  |
| MAT1A ---- tag | rs2236568 | 1 | 0.92 (0.81-1.06) | 0.25 | 0.90 | 0.59 | 1473 | 414 |  |
| MAT1A ---- tag | rs2236569 | 1 | 1.11 (0.96-1.28) | 0.15 | 0.86 | 0.59 | 1473 | 414 |  |
| MAT1A ---- tag | rs9421467 | 1 | 1.13 (0.85-1.51) | 0.39 | 0.94 | 0.59 | 1473 | 414 | low_count |
| MAT1A ---- tag | rs998765 | 1 | 0.97 (0.85-1.11) | 0.69 | 0.98 | 0.78 | 1473 | 414 |  |
| MAT1A ---- tag | rs998766 | 1 | 0.94 (0.82-1.08) | 0.39 | 0.94 | 0.59 | 1473 | 414 |  |
| MAT2B ---- tag | rs12655857 | 1 | 1.04 (0.89-1.22) | 0.64 | 0.97 | 0.80 | 1473 | 414 |  |
| MAT2B ---- tag | rs6869277 | 1 | 0.98 (0.78-1.23) | 0.87 | 0.99 | 0.87 | 1473 | 414 | low_count |
| MAT2B ---- tag | rs6874065 | 1 | 0.90 (0.78-1.03) | 0.12 | 0.86 | 0.20 | 1473 | 414 |  |
| MAT2B ---- tag | rs6882306 | 1 | 1.34 (1.12-1.59) | 0.00 | 0.45 | 0.01 | 1473 | 414 |  |
| MAT2B ---- tag | rs7721639 | 1 | 1.22 (1.02-1.46) | 0.03 | 0.85 | 0.09 | 1473 | 414 |  |
| MTHFD1 ---- tag | rs1256148 | 1 | 1.03 (0.87-1.21) | 0.75 | 0.98 | 0.90 | 1473 | 414 |  |
| MTHFD1 ---- tag | rs13329053 | 1 | 1.09 (0.95-1.25) | 0.20 | 0.86 | 0.32 | 1473 | 414 |  |
| MTHFD1 ---- candidate literature | rs2236224 | 1 | 1.11 (0.96-1.27) | 0.15 | 0.86 | 0.32 | 1473 | 414 |  |
| MTHFD1 ---- candidate | rs2236225 | 1 | 1.09 (0.95-1.25) | 0.21 | 0.89 | 0.32 | 1473 | 414 |  |
| MTHFD1 ---- tag | rs2281603 | 1 | 0.99 (0.84-1.17) | 0.93 | 0.99 | 0.93 | 1473 | 414 |  |
| MTHFD1 ---- candidate literature | rs8003379 | 1 | 1.15 (0.99-1.35) | 0.07 | 0.85 | 0.32 | 1473 | 414 |  |
| MTHFD2 ---- tag | rs10177833 | 1 | 0.97 (0.85-1.12) | 0.72 | 0.98 | 0.85 | 1473 | 414 |  |
| MTHFD2 ---- tag | rs702462 | 1 | 0.93 (0.80-1.07) | 0.30 | 0.92 | 0.85 | 1473 | 414 |  |
| MTHFD2 ---- candidate literature | rs702465 | 1 | 1.08 (0.94-1.24) | 0.29 | 0.91 | 0.85 | 1473 | 414 |  |
| MTHFD2 ---- candidate literature | rs7571842 | 1 | 0.98 (0.85-1.12) | 0.73 | 0.98 | 0.85 | 1473 | 414 |  |
| MTHFD2 ---- tag | rs7587117 | 1 | 0.99 (0.86-1.15) | 0.89 | 0.99 | 0.89 | 1473 | 414 |  |
| MTHFD2 ---- tag | rs828861 | 1 | 1.06 (0.92-1.22) | 0.44 | 0.94 | 0.85 | 1473 | 414 |  |
| MTHFD2 ---- tag | rs828863 | 1 | 0.94 (0.73-1.21) | 0.62 | 0.97 | 0.85 | 1473 | 414 | low_count |
| MTHFR ---- tag | rs1476413 | 1 | 0.93 (0.79-1.09) | 0.39 | 0.94 | 0.72 | 1473 | 414 |  |
| MTHFR ---- tag | rs17376328 | 1 | 1.09 (0.84-1.41) | 0.53 | 0.97 | 0.72 | 1473 | 414 | low_count |
| MTHFR ---- tag | rs17421462 | 1 | 1.05 (0.81-1.37) | 0.72 | 0.98 | 0.72 | 1473 | 414 | low_count |
| MTHFR ---- candidate | rs1801131 | 1 | 0.97 (0.83-1.12) | 0.65 | 0.97 | 0.72 | 1473 | 414 |  |
| MTHFR ---- candidate | rs1801133 | 1 | 0.90 (0.77-1.05) | 0.18 | 0.86 | 0.72 | 1473 | 414 |  |
| MTHFR ---- tag | rs2066471 | 1 | 0.93 (0.76-1.12) | 0.44 | 0.94 | 0.72 | 1473 | 414 |  |
| MTHFR ---- tag | rs4846047 | 1 | 0.95 (0.81-1.11) | 0.51 | 0.96 | 0.72 | 1473 | 414 |  |
| MTHFR ---- tag | rs4846049 | 1 | 0.96 (0.82-1.12) | 0.57 | 0.97 | 0.72 | 1473 | 414 |  |
| MTHFR ---- tag | rs7538516 | 1 | 0.97 (0.84-1.12) | 0.67 | 0.98 | 0.72 | 1473 | 414 |  |
| MTR ---- tag | rs10733117 | 1 | 0.94 (0.82-1.09) | 0.44 | 0.94 | 0.45 | 1473 | 414 |  |
| MTR ---- tag | rs12129440 | 1 | 0.90 (0.76-1.07) | 0.24 | 0.90 | 0.45 | 1473 | 414 |  |
| MTR ---- candidate | rs1805087 | 1 | 0.93 (0.78-1.12) | 0.45 | 0.95 | 0.45 | 1473 | 414 |  |
| MTR ---- tag | rs3890786 | 1 | 1.10 (0.96-1.27) | 0.17 | 0.86 | 0.45 | 1473 | 414 |  |
| MTR ---- tag | rs4659727 | 1 | 0.92 (0.77-1.10) | 0.35 | 0.94 | 0.45 | 1473 | 414 |  |
| MTRR ---- candidate literature/tag | rs10380 | 1 | 1.21 (0.94-1.55) | 0.14 | 0.86 | 0.48 | 1473 | 414 | low_count |
| MTRR ---- tag | rs10475399 | 1 | 1.06 (0.92-1.23) | 0.43 | 0.94 | 0.97 | 1473 | 414 |  |
| MTRR ---- tag | rs11134265 | 1 | 1.04 (0.90-1.20) | 0.60 | 0.97 | 0.97 | 1473 | 414 |  |
| MTRR ---- tag | rs13181011 | 1 | 0.95 (0.80-1.13) | 0.57 | 0.97 | 0.97 | 1473 | 414 |  |
| MTRR ---- tag | rs161869 | 1 | 1.11 (0.97-1.28) | 0.14 | 0.86 | 0.48 | 1473 | 414 |  |
| MTRR ---- tagged by rs162039 | rs162036 | 1 | 1.19 (0.95-1.48) | 0.14 | 0.86 | 0.48 | 1473 | 414 | low_count |
| MTRR ---- tag | rs162039 | 1 | 1.19 (0.95-1.48) | 0.14 | 0.86 | 0.48 | 1473 | 414 | low_count |
| MTRR ---- tag | rs162270 | 1 | 1.01 (0.84-1.20) | 0.95 | 0.99 | 0.97 | 1473 | 414 |  |
| MTRR ---- candidate | rs16879334 | 1 | 0.99 (0.66-1.48) | 0.97 | 0.99 | 0.97 | 1473 | 414 |  |
| MTRR ---- singleton | rs1801394 | 1 | 1.00 (0.87-1.15) | 0.96 | 0.99 | 0.97 | 1473 | 414 |  |
| MTRR ---- tag | rs1802059 | 1 | 0.91 (0.79-1.05) | 0.20 | 0.86 | 0.56 | 1473 | 414 |  |
| MTRR ---- tag | rs2077744 | 1 | 1.01 (0.84-1.22) | 0.88 | 0.99 | 0.97 | 1473 | 414 |  |
| MTRR ---- candidate | rs2287780 | 1 | 0.99 (0.66-1.48) | 0.97 | 0.99 | 0.97 | 1473 | 414 |  |
| MTRR ---- candidate | rs2303080 | 1 | 0.97 (0.64-1.46) | 0.88 | 0.99 | 0.97 | 1473 | 414 |  |
| MTRR ---- tag | rs7715062 | 1 | 0.95 (0.82-1.09) | 0.47 | 0.95 | 0.97 | 1473 | 414 |  |
| MTRR ---- tag | rs9282787 | 1 | 0.97 (0.82-1.15) | 0.74 | 0.98 | 0.97 | 1473 | 414 |  |
| MTRR ---- candidate literature | rs9332 | 1 | 1.19 (0.95-1.48) | 0.14 | 0.86 | 0.48 | 1473 | 414 | low_count |
| NFKB1 ---- NA | rs1609798 | 1 | 1.03 (0.89-1.19) | 0.72 | 0.98 | 0.98 | 1473 | 414 |  |
| NFKB1 ---- tag | rs230540 | 1 | 0.94 (0.82-1.09) | 0.42 | 0.94 | 0.98 | 1473 | 414 |  |
| NFKB1 ---- tag | rs230541 | 1 | 0.95 (0.83-1.10) | 0.51 | 0.96 | 0.98 | 1473 | 414 |  |
| NFKB1 ---- NA | rs230547 | 1 | 0.94 (0.75-1.18) | 0.61 | 0.97 | 0.98 | 1473 | 414 | low_count |
| NFKB1 ---- tag | rs3774934 | 1 | 0.92 (0.74-1.15) | 0.48 | 0.95 | 0.98 | 1473 | 414 | low_count |
| NFKB1 ---- tag | rs3774968 | 1 | 1.00 (0.87-1.15) | 1.00 | 1.00 | 1.00 | 1473 | 414 |  |
| NFKB1 ---- NA | rs4648022 | 1 | 1.17 (0.92-1.49) | 0.21 | 0.89 | 0.98 | 1473 | 414 | low_count |
| NFKB1 ---- NA | rs4648090 | 1 | 1.03 (0.84-1.26) | 0.77 | 0.98 | 0.98 | 1473 | 414 | low_count |
| NFKB1 ---- tag | rs4648110 | 1 | 1.02 (0.85-1.21) | 0.84 | 0.99 | 0.98 | 1473 | 414 |  |
| NFKB1 ---- tag | rs4648141 | 1 | 1.08 (0.89-1.29) | 0.44 | 0.94 | 0.98 | 1473 | 414 |  |
| NFKB1 ---- tag | rs4698863 | 1 | 1.00 (0.86-1.16) | 0.97 | 0.99 | 1.00 | 1473 | 414 |  |
| NFKB1 ---- NA | rs7674640 | 1 | 0.99 (0.86-1.13) | 0.84 | 0.99 | 0.98 | 1473 | 414 |  |
| NFKB1 ---- tag | rs909332 | 1 | 1.20 (0.87-1.66) | 0.27 | 0.90 | 0.98 | 1473 | 414 | low_count |
| NFKB1 ---- tag | rs997476 | 1 | 1.04 (0.78-1.38) | 0.81 | 0.99 | 0.98 | 1473 | 414 | low_count |
| NME1 ---- NA | rs10514981 | 1 | 0.95 (0.79-1.13) | 0.54 | 0.97 | 0.96 | 1473 | 414 |  |
| NME1 ---- NA | rs11651252 | 1 | 1.01 (0.75-1.35) | 0.96 | 0.99 | 0.96 | 1473 | 414 | low_count |
| NME1 ---- tag | rs11652793 | 1 | 0.96 (0.80-1.16) | 0.71 | 0.98 | 0.96 | 1473 | 414 |  |
| NME1 ---- NA | rs11868380 | 1 | 0.99 (0.84-1.17) | 0.91 | 0.99 | 0.96 | 1473 | 414 |  |
| NME1 ---- NA | rs1558252 | 1 | 1.04 (0.90-1.21) | 0.57 | 0.97 | 0.96 | 1473 | 414 |  |
| NME1 ---- NA | rs1558253 | 1 | 1.23 (0.93-1.61) | 0.14 | 0.86 | 0.96 | 1473 | 414 | low_count |
| NME1 ---- tag | rs16949683 | 1 | 1.06 (0.75-1.51) | 0.74 | 0.98 | 0.96 | 1473 | 414 | low_count |
| NME1 ---- tag | rs2318784 | 1 | 1.03 (0.84-1.27) | 0.77 | 0.98 | 0.96 | 1473 | 414 | low_count |
| NME1 ---- NA | rs2318785 | 1 | 0.95 (0.83-1.10) | 0.52 | 0.97 | 0.96 | 1473 | 414 |  |
| NME1 ---- tag | rs3760469 | 1 | 1.01 (0.88-1.17) | 0.85 | 0.99 | 0.96 | 1473 | 414 |  |
| NME1 ---- NA | rs4605213 | 1 | 0.99 (0.86-1.15) | 0.94 | 0.99 | 0.96 | 1473 | 414 |  |
| NME1 ---- NA | rs7207090 | 1 | 0.99 (0.86-1.14) | 0.86 | 0.99 | 0.96 | 1473 | 414 |  |
| NME1 ---- tag | rs7222463 | 1 | 1.01 (0.88-1.17) | 0.87 | 0.99 | 0.96 | 1473 | 414 |  |
| NME1 ---- tag | rs7226059 | 1 | 1.07 (0.93-1.23) | 0.35 | 0.94 | 0.96 | 1473 | 414 |  |
| NME1 ---- NA | rs880178 | 1 | 1.03 (0.89-1.18) | 0.73 | 0.98 | 0.96 | 1473 | 414 |  |
| NME2 ---- tag | rs7220360 | 1 | 1.02 (0.88-1.17) | 0.83 | 0.99 | 0.83 | 1473 | 414 |  |
| PON1 ---- tag | rs2269829 | 1 | 1.02 (0.88-1.18) | 0.83 | 0.99 | 0.94 | 1473 | 414 |  |
| PON1 ---- tag | rs3917527 | 1 | 0.75 (0.55-1.03) | 0.08 | 0.85 | 0.20 | 1473 | 414 | low_count |
| PON1 ---- tag | rs3917538 | 1 | 1.15 (0.98-1.35) | 0.08 | 0.85 | 0.20 | 1473 | 414 |  |
| PON1 ---- tag | rs757158 | 1 | 1.01 (0.87-1.16) | 0.94 | 0.99 | 0.94 | 1473 | 414 |  |
| PON1 ---- candidate | rs854560 | 1 | 1.02 (0.88-1.17) | 0.82 | 0.99 | 0.94 | 1473 | 414 |  |
| PRDM2 ---- tag | rs1015370 | 1 | 0.94 (0.81-1.09) | 0.43 | 0.94 | 0.86 | 1473 | 414 |  |
| PRDM2 ---- tag | rs1203634 | 1 | 1.06 (0.91-1.24) | 0.46 | 0.95 | 0.86 | 1473 | 414 |  |
| PRDM2 ---- tag | rs1203645 | 1 | 1.02 (0.89-1.17) | 0.76 | 0.98 | 0.86 | 1473 | 414 |  |
| PRDM2 ---- tag | rs1406416 | 1 | 1.10 (0.95-1.27) | 0.19 | 0.86 | 0.86 | 1473 | 414 |  |
| PRDM2 ---- candidate | rs17350795 | 1 | 0.73 (0.44-1.21) | 0.22 | 0.90 | 0.86 | 1473 | 414 |  |
| PRDM2 ---- tag | rs1980472 | 1 | 1.02 (0.88-1.19) | 0.77 | 0.98 | 0.86 | 1473 | 414 |  |
| PRDM2 ---- tag | rs2235515 | 1 | 1.00 (0.85-1.17) | 0.97 | 0.99 | 0.97 | 1473 | 414 |  |
| PRDM2 ---- tag | rs2244634 | 1 | 0.93 (0.78-1.10) | 0.37 | 0.94 | 0.86 | 1473 | 414 |  |
| PRDM2 ---- tag | rs2245213 | 1 | 0.94 (0.78-1.13) | 0.51 | 0.96 | 0.86 | 1473 | 414 | low_count |
| PRDM2 ---- tag | rs2294484 | 1 | 0.96 (0.74-1.25) | 0.79 | 0.99 | 0.86 | 1473 | 414 | low_count |
| PRDM2 ---- tag | rs2744689 | 1 | 0.95 (0.79-1.14) | 0.59 | 0.97 | 0.86 | 1473 | 414 | low_count |
| PRDM2 ---- tag | rs6690270 | 1 | 1.03 (0.90-1.19) | 0.64 | 0.97 | 0.86 | 1473 | 414 |  |
| RRM1 ---- tag | rs10835601 | 1 | 1.14 (0.99-1.33) | 0.08 | 0.85 | 0.29 | 1473 | 414 |  |
| RRM1 ---- tag | rs10835613 | 1 | 1.12 (0.98-1.29) | 0.10 | 0.86 | 0.29 | 1473 | 414 |  |
| RRM1 ---- NA | rs10835677 | 1 | 1.06 (0.84-1.32) | 0.63 | 0.97 | 0.73 | 1473 | 414 | low_count |
| RRM1 ---- tag | rs10835678 | 1 | 0.93 (0.69-1.26) | 0.66 | 0.97 | 0.73 | 1473 | 414 | low_count |
| RRM1 ---- tag | rs12288551 | 1 | 1.29 (0.94-1.77) | 0.12 | 0.86 | 0.29 | 1473 | 414 | low_count |
| RRM1 ---- NA | rs12806698 | 1 | 1.12 (0.96-1.30) | 0.15 | 0.86 | 0.31 | 1473 | 414 |  |
| RRM1 ---- NA | rs1465952 | 1 | 0.94 (0.74-1.20) | 0.62 | 0.97 | 0.73 | 1473 | 414 | low_count |
| RRM1 ---- tag | rs4910904 | 1 | 1.19 (1.03-1.37) | 0.02 | 0.85 | 0.20 | 1473 | 414 |  |
| RRM1 ---- tag | rs7103860 | 1 | 0.97 (0.78-1.21) | 0.79 | 0.99 | 0.79 | 1473 | 414 | low_count |
| RRM1 ---- tag | rs7115496 | 1 | 0.91 (0.69-1.19) | 0.47 | 0.95 | 0.73 | 1473 | 414 | low_count |
| RRM2 ---- NA | rs1138729 | 1 | 1.12 (0.93-1.34) | 0.23 | 0.90 | 0.67 | 1473 | 414 |  |
| RRM2 ---- tag | rs4668664 | 1 | 0.94 (0.81-1.09) | 0.43 | 0.94 | 0.67 | 1473 | 414 |  |
| RRM2 ---- NA | rs6741290 | 1 | 1.02 (0.89-1.16) | 0.80 | 0.99 | 0.80 | 1473 | 414 |  |
| RRM2 ---- tag | rs7574663 | 1 | 1.06 (0.90-1.25) | 0.51 | 0.96 | 0.67 | 1473 | 414 |  |
| SHMT1 ---- candidate | rs1979277 | 1 | 0.97 (0.83-1.14) | 0.74 | 0.98 | 0.95 | 1473 | 414 |  |
| SHMT1 ---- tag | rs2168781 | 1 | 1.00 (0.86-1.15) | 0.95 | 0.99 | 0.95 | 1473 | 414 |  |
| SHMT1 ---- tag | rs4924849 | 1 | 0.98 (0.83-1.14) | 0.76 | 0.98 | 0.95 | 1473 | 414 |  |
| SHMT1 ---- candidate literature | rs9909104 | 1 | 0.97 (0.83-1.14) | 0.74 | 0.98 | 0.95 | 1473 | 414 |  |
| SHMT2 ---- tag | rs10876968 | 1 | 1.00 (0.86-1.17) | 1.00 | 1.00 | 1.00 | 1473 | 414 |  |
| SHMT2 ---- tag | rs1800165 | 1 | 1.07 (0.93-1.25) | 0.35 | 0.94 | 0.67 | 1473 | 414 |  |
| SHMT2 ---- tag | rs7133939 | 1 | 0.97 (0.84-1.11) | 0.64 | 0.97 | 0.80 | 1473 | 414 |  |
| SHMT2 ---- tag | rs7485577 | 1 | 1.07 (0.92-1.24) | 0.40 | 0.94 | 0.67 | 1473 | 414 |  |
| SHMT2 ---- tag | rs7489231 | 1 | 1.15 (0.99-1.32) | 0.06 | 0.85 | 0.32 | 1473 | 414 |  |
| SLC19A1 ---- candidate | rs1051266 | 1 | 0.98 (0.85-1.13) | 0.80 | 0.99 | 0.80 | 1473 | 414 |  |
| SLC19A1 ---- candidate literature | rs1131596 | 1 | 0.98 (0.86-1.13) | 0.80 | 0.99 | 0.80 | 1473 | 414 |  |
| SLC19A1 ---- tag | rs12483553 | 1 | 1.22 (0.99-1.51) | 0.07 | 0.85 | 0.46 | 1473 | 414 | low_count |
| SLC19A1 ---- candidate literature | rs12659 | 1 | 0.97 (0.84-1.11) | 0.63 | 0.97 | 0.80 | 1473 | 414 |  |
| SLC19A1 ---- tag | rs3788190 | 1 | 0.95 (0.83-1.09) | 0.47 | 0.95 | 0.80 | 1473 | 414 |  |
| SLC19A1 ---- tag | rs3788205 | 1 | 0.96 (0.82-1.12) | 0.60 | 0.97 | 0.80 | 1473 | 414 |  |
| SLC19A1 ---- tag | rs7279664 | 1 | 0.95 (0.82-1.09) | 0.47 | 0.95 | 0.80 | 1473 | 414 |  |
| SLC29A1 ---- NA | rs1057985 | 1 | 1.00 (0.87-1.16) | 1.00 | 1.00 | 1.00 | 1473 | 414 |  |
| SLC29A1 ---- NA | rs6458375 | 1 | 0.87 (0.74-1.03) | 0.10 | 0.86 | 0.69 | 1473 | 414 |  |
| SLC29A1 ---- NA | rs666462 | 1 | 1.03 (0.90-1.18) | 0.65 | 0.97 | 1.00 | 1473 | 414 |  |
| SLC29A1 ---- NA | rs6905285 | 1 | 1.01 (0.87-1.16) | 0.92 | 0.99 | 1.00 | 1473 | 414 |  |
| SLC29A1 ---- NA | rs693955 | 1 | 1.06 (0.88-1.26) | 0.55 | 0.97 | 1.00 | 1473 | 414 |  |
| SLC29A1 ---- NA | rs747199 | 1 | 0.98 (0.82-1.16) | 0.81 | 0.99 | 1.00 | 1473 | 414 |  |
| SLC29A1 ---- NA | rs9357436 | 1 | 0.99 (0.83-1.19) | 0.95 | 0.99 | 1.00 | 1473 | 414 |  |
| TCN2 ---- tag | rs10418 | 1 | 1.08 (0.92-1.26) | 0.38 | 0.94 | 0.70 | 1473 | 414 |  |
| TCN2 ---- candidate/singleton | rs1131603 | 1 | 1.01 (0.77-1.33) | 0.92 | 0.99 | 0.95 | 1473 | 414 | low_count |
| TCN2 ---- tag | rs1544468 | 1 | 1.08 (0.94-1.23) | 0.28 | 0.91 | 0.61 | 1473 | 414 |  |
| TCN2 ---- candidate/tag | rs1801198 | 1 | 0.90 (0.78-1.03) | 0.12 | 0.86 | 0.60 | 1473 | 414 |  |
| TCN2 ---- tag | rs4820872 | 1 | 0.91 (0.79-1.03) | 0.14 | 0.86 | 0.60 | 1473 | 414 |  |
| TCN2 ---- tag | rs4820874 | 1 | 1.01 (0.83-1.22) | 0.95 | 0.99 | 0.95 | 1473 | 414 |  |
| TCN2 ---- tag | rs4820886 | 1 | 1.05 (0.83-1.32) | 0.70 | 0.98 | 0.93 | 1473 | 414 | low_count |
| TCN2 ---- candidate | rs4820889 | 1 | 0.74 (0.49-1.12) | 0.16 | 0.86 | 0.60 | 1473 | 414 |  |
| TCN2 ---- tag | rs5997711 | 1 | 0.91 (0.80-1.05) | 0.20 | 0.87 | 0.60 | 1473 | 414 |  |
| TCN2 ---- tag | rs740234 | 1 | 1.03 (0.87-1.23) | 0.72 | 0.98 | 0.93 | 1473 | 414 |  |
| TCN2 ---- tag | rs740235 | 1 | 1.09 (0.95-1.25) | 0.23 | 0.90 | 0.60 | 1473 | 414 |  |
| TCN2 ---- candidate/singleton | rs9606756 | 1 | 0.98 (0.78-1.24) | 0.89 | 0.99 | 0.95 | 1473 | 414 | low_count |
| TCN2 ---- candidate | rs9621049 | 1 | 1.05 (0.83-1.32) | 0.70 | 0.98 | 0.93 | 1473 | 414 | low_count |
| TK1 ---- NA | rs1065769 | 1 | 0.96 (0.83-1.11) | 0.62 | 0.97 | 0.86 | 1473 | 414 |  |
| TK1 ---- NA | rs12232476 | 1 | 1.06 (0.83-1.36) | 0.65 | 0.97 | 0.86 | 1473 | 414 | low_count |
| TK1 ---- tag | rs16970907 | 1 | 1.04 (0.80-1.36) | 0.76 | 0.98 | 0.87 | 1473 | 414 | low_count |
| TK1 ---- tag | rs1811086 | 1 | 1.10 (0.76-1.59) | 0.60 | 0.97 | 0.86 | 1473 | 414 | low_count |
| TK1 ---- tag | rs2292235 | 1 | 0.92 (0.81-1.06) | 0.26 | 0.90 | 0.86 | 1473 | 414 |  |
| TK1 ---- tag | rs2854701 | 1 | 1.00 (0.87-1.15) | 0.96 | 0.99 | 0.96 | 1473 | 414 |  |
| TK1 ---- tag | rs2854702 | 1 | 1.09 (0.88-1.34) | 0.42 | 0.94 | 0.86 | 1473 | 414 | low_count |
| TK1 ---- tag | rs9897765 | 1 | 0.94 (0.80-1.09) | 0.42 | 0.94 | 0.86 | 1473 | 414 |  |
| TYMP ---- NA | rs131815 | 1 | 1.11 (0.96-1.30) | 0.17 | 0.86 | 0.29 | 1473 | 414 |  |
| TYMP ---- tag | rs131816 | 1 | 0.81 (0.69-0.96) | 0.02 | 0.85 | 0.10 | 1473 | 414 |  |
| TYMP ---- NA | rs131817 | 1 | 1.08 (0.94-1.24) | 0.29 | 0.91 | 0.35 | 1473 | 414 |  |
| TYMP ---- NA | rs140521 | 1 | 0.90 (0.77-1.05) | 0.20 | 0.86 | 0.29 | 1473 | 414 |  |
| TYMP ---- NA | rs140522 | 1 | 0.86 (0.74-1.00) | 0.05 | 0.85 | 0.14 | 1473 | 414 |  |
| TYMP ---- NA | rs140524 | 1 | 1.04 (0.87-1.24) | 0.65 | 0.97 | 0.65 | 1473 | 414 |  |
| TYMS ---- candidate literature | rs1001761 | 1 | 0.87 (0.76-1.01) | 0.06 | 0.85 | 0.19 | 1473 | 414 |  |
| TYMS ---- candidate literature/tag | rs10502289 | 1 | 0.89 (0.75-1.06) | 0.18 | 0.86 | 0.27 | 1473 | 414 |  |
| TYMS ---- tag | rs15872 | 1 | 0.93 (0.80-1.09) | 0.40 | 0.94 | 0.49 | 1473 | 414 |  |
| TYMS ---- tag | rs2244500 | 1 | 0.87 (0.76-1.00) | 0.06 | 0.85 | 0.19 | 1473 | 414 |  |
| TYMS ---- tag | rs2741182 | 1 | 0.94 (0.80-1.10) | 0.44 | 0.94 | 0.49 | 1473 | 414 |  |
| TYMS ---- candidate literature | rs2847149 | 1 | 0.87 (0.76-1.01) | 0.06 | 0.85 | 0.19 | 1473 | 414 |  |
| TYMS ---- candidate literature | rs2853533 | 1 | 0.96 (0.78-1.17) | 0.69 | 0.98 | 0.69 | 1473 | 414 | low_count |
| TYMS ---- tag | rs495139 | 1 | 1.12 (0.97-1.28) | 0.11 | 0.86 | 0.26 | 1473 | 414 |  |
| TYMS ---- candidate literature | rs502396 | 1 | 0.91 (0.79-1.04) | 0.17 | 0.86 | 0.27 | 1473 | 414 |  |
| UMPH2 ---- tag | rs2291028 | 1 | 1.09 (0.94-1.26) | 0.25 | 0.90 | 0.61 | 1473 | 414 |  |
| UMPH2 ---- NA | rs4789143 | 1 | 0.96 (0.76-1.21) | 0.73 | 0.98 | 0.73 | 1473 | 414 | low_count |
| UMPH2 ---- NA | rs750844 | 1 | 1.07 (0.92-1.24) | 0.40 | 0.94 | 0.61 | 1473 | 414 |  |
| UMPK ---- tag | rs11582877 | 1 | 1.01 (0.84-1.22) | 0.89 | 0.99 | 0.98 | 1473 | 414 |  |
| UMPK ---- tag | rs2622903 | 1 | 0.96 (0.82-1.12) | 0.58 | 0.97 | 0.98 | 1473 | 414 |  |
| UMPK ---- tag | rs2820989 | 1 | 0.98 (0.86-1.12) | 0.77 | 0.98 | 0.98 | 1473 | 414 |  |
| UMPK ---- tag | rs6660321 | 1 | 1.00 (0.83-1.22) | 0.98 | 0.99 | 0.98 | 1473 | 414 | low_count |
| UMPK ---- tag | rs6690084 | 1 | 1.15 (0.90-1.47) | 0.26 | 0.90 | 0.98 | 1473 | 414 | low_count |
| UMPS ---- NA | rs1162 | 1 | 1.24 (1.08-1.43) | 0.00 | 0.60 | 0.02 | 1473 | 414 |  |
| UMPS ---- tag | rs13146 | 1 | 1.16 (0.98-1.38) | 0.09 | 0.86 | 0.18 | 1473 | 414 |  |
| UMPS ---- tag | rs16835902 | 1 | 0.96 (0.83-1.11) | 0.59 | 0.97 | 0.71 | 1473 | 414 |  |
| UMPS ---- tag | rs17282057 | 1 | 1.15 (0.94-1.41) | 0.18 | 0.86 | 0.28 | 1473 | 414 |  |
| UMPS ---- tag | rs606552 | 1 | 0.84 (0.72-0.99) | 0.04 | 0.85 | 0.11 | 1473 | 414 |  |
| UMPS ---- tag | rs694897 | 1 | 1.00 (0.87-1.16) | 0.98 | 0.99 | 0.98 | 1473 | 414 |  |
| UNG ---- NA | rs1059262 | 1 | 1.00 (0.83-1.20) | 1.00 | 1.00 | 1.00 | 1473 | 414 |  |
| UNG ---- tag | rs2160603 | 1 | 1.13 (0.95-1.36) | 0.17 | 0.86 | 0.97 | 1473 | 414 |  |
| UNG ---- tag | rs246079 | 1 | 1.03 (0.90-1.19) | 0.65 | 0.97 | 0.97 | 1473 | 414 |  |
| UNG ---- NA | rs246085 | 1 | 0.91 (0.67-1.23) | 0.54 | 0.97 | 0.97 | 1473 | 414 | low_count |
| UNG ---- NA | rs2569987 | 1 | 0.93 (0.78-1.12) | 0.45 | 0.94 | 0.97 | 1473 | 414 |  |
| UNG ---- tag | rs3219243 | 1 | 1.01 (0.85-1.21) | 0.90 | 0.99 | 1.00 | 1473 | 414 |  |

*adjusted for age, sex, stage, grade, BMI, alcohol intake

**candidate, FDR-adjusted cut-off for significance of p-value = 0.02

^ǂ^dominant model (HR_het_)

^1^p_trend_:p-Value for trend

^2^p_trendFDR_:FDR adjusted trend

^3^p_FDRGenwide_:FDR adjusted genewide effect
