# Supplementary material for: Pathway analysis of genetic variants in folate‐mediated one‐carbon metabolism‐related genes and survival in a prospectively followed cohort of colorectal cancer patients
Source: Cancer Med. 2018 May 29;7(7):2797–807. doi: 10.1002/cam4.1407 (PMC6051204; doi:10.1002/cam4.1407)
Supplement: Supplementary file 7 — Table S9. Associations between polymorphisms in FOCM‐related genes and overall survival stratified by 5‐FU‐based chemotherapy [file CAM4-7-2797-s007.docx]

| **Supplementary Table 9. Associations between polymorphisms in FOCM-related genes and overall survival stratified by 5-FU-based chemotherapy*** | | | | | | | | | | | | | | | | | |
| --- | --- | --- | --- | --- | --- | --- | --- | --- | --- | --- | --- | --- | --- | --- | --- | --- | --- |
|  | | | **adchem_5fu = 0** | | | | **adchem_5fu = 1** | | | | **one reference** | | | | | | |
|  | | | **Ctrl** | | **Cases** | | **Ctrl** | | **Cases** | | **adchem_5fu = 0** | | **adchem_5fu = 1** | |  | | |
| **Gene** | **SNP** | **Genotype** | **N** | **%** | **N** | **%** | **N** | **%** | **N** | **%** | **HR (95%-CI)** | **p** | **HR (95%-CI)** | **p** | **LR_pInt** | **FDR_pInt** | **FDR(byGene)_pInt** |
| AARS ---- tag | rs2070203 | T/T | 16 | 27,59 | 12 | 35,29 | 115 | 30,50 | 73 | 27,04 | 1.00 (.-.) | . | 0.86 (0.43-1.72) | 0.66 | 0.23 | 0.99 | 0.89 |
| AARS ---- tag |  | T/C or C/C | 42 | 72,41 | 22 | 64,71 | 262 | 69,50 | 197 | 72,96 | 0.71 (0.32-1.56) | 0.39 | 1.03 (0.53-2.00) | 0.93 | . | . | . |
| AARS ---- tag | rs34087264 | G/G | 22 | 37,93 | 6 | 17,65 | 97 | 25,73 | 84 | 31,11 | 1.00 (.-.) | . | 2.44 (0.97-6.11) | 0.06 | 0.06 | 0.64 | 0.11 |
| AARS ---- tag |  | G/A or A/A | 36 | 62,07 | 28 | 82,35 | 280 | 74,27 | 186 | 68,89 | 1.93 (0.72-5.19) | 0.19 | 1.90 (0.77-4.70) | 0.17 | . | . | . |
| ABCC4 ---- tag | rs10508023 | G/G | 48 | 82,76 | 26 | 76,47 | 298 | 79,05 | 219 | 81,11 | 1.00 (.-.) | . | 1.45 (0.91-2.30) | 0.12 | 0.11 | 0.93 | 0.81 |
| ABCC4 ---- tag |  | G/C or C/C | 10 | 17,24 | 8 | 23,53 | 79 | 20,95 | 51 | 18,89 | 1.86 (0.76-4.52) | 0.17 | 1.18 (0.69-2.01) | 0.54 | . | . | . |
| ABCC4 ---- tag | rs1059751 | T/T | 21 | 36,21 | 7 | 20,59 | 88 | 23,34 | 71 | 26,30 | 1.00 (.-.) | . | 1.48 (0.67-3.29) | 0.33 | 0.59 | 0.99 | 0.95 |
| ABCC4 ---- tag |  | T/C or C/C | 37 | 63,79 | 27 | 79,41 | 289 | 76,66 | 199 | 73,70 | 1.33 (0.56-3.16) | 0.53 | 1.53 (0.71-3.30) | 0.28 | . | . | . |
| ABCC4 ---- tag | rs11568643 | A/A | 50 | 86,21 | 26 | 76,47 | 320 | 84,88 | 222 | 82,22 | 1.00 (.-.) | . | 1.41 (0.88-2.24) | 0.15 | 0.20 | 0.66 | 0.60 |
| ABCC4 ---- tag |  | A/G or G/G | 8 | 13,79 | 8 | 23,53 | 57 | 15,12 | 48 | 17,78 | 2.19 (0.92-5.24) | 0.08 | 1.64 (0.95-2.83) | 0.08 | . | . | . |
| ABCC4 ---- NA | rs11568658 | G/G | 55 | 94,83 | 32 | 94,12 | 356 | 94,43 | 258 | 95,56 | 1.00 (.-.) | . | 1.30 (0.85-1.99) | 0.23 | 0.27 | 0.99 | 0.95 |
| ABCC4 ---- NA |  | G/T or T/T | 3 | 5,17 | 2 | 5,88 | 21 | 5,57 | 12 | 4,44 | 2.64 (0.61-11.37) | 0.19 | 1.27 (0.58-2.75) | 0.55 | . | . | . |
| ABCC4 ---- tag | rs12864049 | T/T | 38 | 65,52 | 26 | 76,47 | 292 | 77,45 | 200 | 74,07 | 1.00 (.-.) | . | 1.16 (0.72-1.86) | 0.54 | 0.59 | 0.99 | 0.98 |
| ABCC4 ---- tag |  | T/C or C/C | 20 | 34,48 | 8 | 23,53 | 85 | 22,55 | 70 | 25,93 | 0.82 (0.35-1.96) | 0.66 | 1.22 (0.73-2.03) | 0.44 | . | . | . |
| ABCC4 ---- tag | rs1628382 | G/G | 39 | 67,24 | 22 | 64,71 | 235 | 62,33 | 163 | 60,37 | 1.00 (.-.) | . | 1.28 (0.77-2.13) | 0.35 | 0.82 | 0.99 | 0.98 |
| ABCC4 ---- tag |  | G/A or A/A | 19 | 32,76 | 12 | 35,29 | 142 | 37,67 | 107 | 39,63 | 1.22 (0.55-2.69) | 0.63 | 1.41 (0.83-2.38) | 0.20 | . | . | . |
| ABCC4 ---- tag | rs1678354 | C/C | 18 | 31,03 | 14 | 41,18 | 169 | 44,83 | 110 | 40,74 | 1.00 (.-.) | . | 1.10 (0.58-2.09) | 0.77 | 0.63 | 0.99 | 0.98 |
| ABCC4 ---- tag |  | C/G or G/G | 40 | 68,97 | 20 | 58,82 | 208 | 55,17 | 160 | 59,26 | 0.93 (0.43-2.00) | 0.85 | 1.25 (0.66-2.34) | 0.49 | . | . | . |
| ABCC4 ---- tag | rs1678383 | T/T | 47 | 81,03 | 28 | 82,35 | 308 | 81,70 | 223 | 82,59 | 1.00 (.-.) | . | 1.16 (0.73-1.84) | 0.53 | 0.54 | 0.90 | 0.72 |
| ABCC4 ---- tag |  | T/G or G/G | 11 | 18,97 | 6 | 17,65 | 69 | 18,30 | 47 | 17,41 | 0.73 (0.27-1.94) | 0.52 | 1.16 (0.68-1.97) | 0.59 | . | . | . |
| ABCC4 ---- tag | rs1678395 | G/G | 48 | 82,76 | 29 | 85,29 | 317 | 84,08 | 230 | 85,19 | 1.00 (.-.) | . | 1.29 (0.83-2.00) | 0.25 | 0.95 | 0.88 | 0.71 |
| ABCC4 ---- tag |  | G/A or A/A | 10 | 17,24 | 5 | 14,71 | 60 | 15,92 | 40 | 14,81 | 0.75 (0.22-2.49) | 0.64 | 0.92 (0.55-1.56) | 0.77 | . | . | . |
| ABCC4 ---- tag | rs1678405 | T/T | 26 | 44,83 | 19 | 55,88 | 167 | 44,30 | 121 | 44,81 | 1.00 (.-.) | . | 1.32 (0.78-2.25) | 0.30 | 0.68 | 0.86 | 0.71 |
| ABCC4 ---- tag |  | T/C or C/C | 32 | 55,17 | 15 | 44,12 | 210 | 55,70 | 149 | 55,19 | 1.22 (0.56-2.68) | 0.62 | 1.35 (0.80-2.29) | 0.27 | . | . | . |
| ABCC4 ---- tag | rs17189540 | A/A | 52 | 89,66 | 27 | 79,41 | 335 | 88,86 | 228 | 84,44 | 1.00 (.-.) | . | 1.23 (0.78-1.94) | 0.36 | 0.80 | 0.85 | 0.70 |
| ABCC4 ---- tag |  | A/G or G/G | 6 | 10,34 | 7 | 20,59 | 42 | 11,14 | 42 | 15,56 | 1.27 (0.50-3.24) | 0.62 | 1.78 (1.03-3.06) | 0.04 | . | . | . |
| ABCC4 ---- tag | rs17235152 | T/T | 39 | 67,24 | 26 | 76,47 | 269 | 71,35 | 204 | 75,56 | 1.00 (.-.) | . | 1.20 (0.74-1.95) | 0.47 | 0.86 | 0.99 | 0.98 |
| ABCC4 ---- tag |  | T/C or C/C | 19 | 32,76 | 8 | 23,53 | 108 | 28,65 | 66 | 24,44 | 0.83 (0.36-1.91) | 0.65 | 1.07 (0.62-1.85) | 0.80 | . | . | . |
| ABCC4 ---- tag | rs17268122 | G/G | 24 | 41,38 | 22 | 64,71 | 236 | 62,60 | 184 | 68,15 | 1.00 (.-.) | . | 0.85 (0.50-1.44) | 0.55 | 0.07 | 1.00 | 0.99 |
| ABCC4 ---- tag |  | G/T or T/T | 34 | 58,62 | 12 | 35,29 | 141 | 37,40 | 86 | 31,85 | 0.43 (0.20-0.97) | 0.04 | 0.80 (0.45-1.39) | 0.43 | . | . | . |
| ABCC4 ---- tag | rs17268170 | C/C | 47 | 81,03 | 24 | 70,59 | 314 | 83,29 | 216 | 80,00 | 1.00 (.-.) | . | 1.35 (0.83-2.19) | 0.23 | 0.53 | 0.99 | 0.98 |
| ABCC4 ---- tag |  | C/T or T/T | 11 | 18,97 | 10 | 29,41 | 63 | 16,71 | 54 | 20,00 | 1.54 (0.67-3.51) | 0.31 | 1.55 (0.90-2.68) | 0.11 | . | . | . |
| ABCC4 ---- tag | rs1729764 | A/A | 43 | 74,14 | 28 | 82,35 | 287 | 76,13 | 218 | 80,74 | 1.00 (.-.) | . | 1.21 (0.79-1.87) | 0.38 | 0.54 | 0.85 | 0.70 |
| ABCC4 ---- tag |  | A/G or G/G | 15 | 25,86 | 6 | 17,65 | 90 | 23,87 | 52 | 19,26 | 0.52 (0.12-2.25) | 0.38 | 0.99 (0.59-1.64) | 0.96 | . | . | . |
| ABCC4 ---- tag | rs1729767 | T/T | 28 | 48,28 | 15 | 44,12 | 202 | 53,58 | 137 | 50,74 | 1.00 (.-.) | . | 1.35 (0.75-2.41) | 0.31 | 0.69 | 0.95 | 0.91 |
| ABCC4 ---- tag |  | T/C or C/C | 30 | 51,72 | 19 | 55,88 | 175 | 46,42 | 133 | 49,26 | 1.24 (0.58-2.65) | 0.57 | 1.42 (0.79-2.56) | 0.24 | . | . | . |
| ABCC4 ---- tag | rs17300935 | C/C | 40 | 68,97 | 21 | 61,76 | 278 | 73,74 | 207 | 76,67 | 1.00 (.-.) | . | 1.26 (0.76-2.09) | 0.38 | 0.91 | 0.99 | 0.95 |
| ABCC4 ---- tag |  | C/G or G/G | 18 | 31,03 | 13 | 38,24 | 99 | 26,26 | 63 | 23,33 | 1.01 (0.46-2.22) | 0.98 | 1.21 (0.69-2.11) | 0.51 | . | . | . |
| ABCC4 ---- tag | rs1750190 | G/G | 19 | 32,76 | 7 | 20,59 | 101 | 26,79 | 62 | 22,96 | 1.00 (.-.) | . | 1.76 (0.69-4.46) | 0.24 | 0.34 | 0.83 | 0.70 |
| ABCC4 ---- tag |  | G/A or A/A | 39 | 67,24 | 27 | 79,41 | 276 | 73,21 | 208 | 77,04 | 2.15 (0.79-5.85) | 0.13 | 2.31 (0.94-5.71) | 0.07 | . | . | . |
| ABCC4 ---- tag | rs1750996 | A/A | 33 | 56,90 | 21 | 61,76 | 247 | 65,52 | 178 | 65,93 | 1.00 (.-.) | . | 1.25 (0.76-2.05) | 0.38 | 0.97 | 0.92 | 0.72 |
| ABCC4 ---- tag |  | A/G or G/G | 25 | 43,10 | 13 | 38,24 | 130 | 34,48 | 92 | 34,07 | 0.97 (0.43-2.18) | 0.94 | 1.19 (0.71-2.01) | 0.52 | . | . | . |
| ABCC4 ---- tag | rs1751025 | C/C | 32 | 55,17 | 13 | 38,24 | 181 | 48,01 | 120 | 44,44 | 1.00 (.-.) | . | 1.71 (0.91-3.23) | 0.10 | 0.10 | 0.66 | 0.60 |
| ABCC4 ---- tag |  | C/G or G/G | 26 | 44,83 | 21 | 61,76 | 196 | 51,99 | 150 | 55,56 | 2.39 (1.09-5.27) | 0.03 | 2.06 (1.10-3.86) | 0.02 | . | . | . |
| ABCC4 ---- tag | rs1751051 | T/T | 25 | 43,10 | 15 | 44,12 | 151 | 40,05 | 121 | 44,81 | 1.00 (.-.) | . | 1.12 (0.63-2.00) | 0.70 | 0.62 | 0.99 | 0.98 |
| ABCC4 ---- tag |  | T/A or A/A | 33 | 56,90 | 19 | 55,88 | 226 | 59,95 | 149 | 55,19 | 0.72 (0.34-1.56) | 0.41 | 0.99 (0.56-1.76) | 0.98 | . | . | . |
| ABCC4 ---- tag | rs1764416 | G/G | 50 | 86,21 | 34 | 100,00 | 320 | 84,88 | 237 | 87,78 | 1.00 (.-.) | . | 1.23 (0.81-1.86) | 0.34 | 0.17 | 0.90 | 0.72 |
| ABCC4 ---- tag |  | G/A or A/A | 8 | 13,79 | 0 | 0,00 | 57 | 15,12 | 33 | 12,22 | 0.00 (0.00-1E286) | 0.97 | 0.94 (0.54-1.65) | 0.83 | . | . | . |
| ABCC4 ---- tag | rs2274401 | T/T | 35 | 60,34 | 23 | 67,65 | 217 | 57,56 | 184 | 68,15 | 1.00 (.-.) | . | 1.18 (0.74-1.86) | 0.49 | 0.57 | 0.87 | 0.71 |
| ABCC4 ---- tag |  | T/C or C/C | 23 | 39,66 | 11 | 32,35 | 160 | 42,44 | 86 | 31,85 | 0.63 (0.25-1.58) | 0.33 | 0.98 (0.60-1.60) | 0.93 | . | . | . |
| ABCC4 ---- tag | rs2892716 | C/C | 26 | 44,83 | 17 | 50,00 | 124 | 32,89 | 107 | 39,63 | 1.00 (.-.) | . | 1.71 (0.97-3.01) | 0.06 | 0.09 | 0.99 | 0.98 |
| ABCC4 ---- tag |  | C/T or T/T | 32 | 55,17 | 17 | 50,00 | 253 | 67,11 | 163 | 60,37 | 1.70 (0.78-3.67) | 0.18 | 1.44 (0.82-2.51) | 0.21 | . | . | . |
| ABCC4 ---- tag | rs3782964 | C/C | 43 | 74,14 | 24 | 70,59 | 242 | 64,19 | 191 | 70,74 | 1.00 (.-.) | . | 1.36 (0.84-2.20) | 0.21 | 0.47 | 0.85 | 0.70 |
| ABCC4 ---- tag |  | C/T or T/T | 15 | 25,86 | 10 | 29,41 | 135 | 35,81 | 79 | 29,26 | 1.16 (0.51-2.66) | 0.72 | 1.14 (0.68-1.91) | 0.63 | . | . | . |
| ABCC4 ---- tag | rs3818494 | C/C | 33 | 56,90 | 18 | 52,94 | 170 | 45,09 | 111 | 41,11 | 1.00 (.-.) | . | 1.32 (0.76-2.29) | 0.33 | 0.58 | 0.59 | 0.60 |
| ABCC4 ---- tag |  | C/G or G/G | 25 | 43,10 | 16 | 47,06 | 207 | 54,91 | 159 | 58,89 | 1.50 (0.70-3.24) | 0.30 | 1.58 (0.92-2.71) | 0.10 | . | . | . |
| ABCC4 ---- tag | rs3864997 | G/G | 21 | 36,21 | 11 | 32,35 | 92 | 24,40 | 63 | 23,33 | 1.00 (.-.) | . | 1.74 (0.85-3.58) | 0.13 | 0.19 | 0.85 | 0.70 |
| ABCC4 ---- tag |  | G/T or T/T | 37 | 63,79 | 23 | 67,65 | 285 | 75,60 | 207 | 76,67 | 1.80 (0.81-4.02) | 0.15 | 1.79 (0.90-3.55) | 0.10 | . | . | . |
| ABCC4 ---- tag | rs4148421 | G/G | 13 | 22,41 | 6 | 17,65 | 118 | 31,30 | 78 | 28,89 | 1.00 (.-.) | . | 1.37 (0.49-3.85) | 0.55 | 0.84 | 0.99 | 0.98 |
| ABCC4 ---- tag |  | G/A or A/A | 45 | 77,59 | 28 | 82,35 | 259 | 68,70 | 192 | 71,11 | 1.17 (0.40-3.46) | 0.77 | 1.44 (0.52-3.99) | 0.48 | . | . | . |
| ABCC4 ---- tag | rs4148446 | G/G | 24 | 41,38 | 14 | 41,18 | 114 | 30,24 | 94 | 34,81 | 1.00 (.-.) | . | 1.57 (0.84-2.94) | 0.16 | 0.32 | 0.99 | 0.98 |
| ABCC4 ---- tag |  | G/A or A/A | 34 | 58,62 | 20 | 58,82 | 263 | 69,76 | 176 | 65,19 | 1.32 (0.62-2.85) | 0.47 | 1.38 (0.75-2.54) | 0.30 | . | . | . |
| ABCC4 ---- tag | rs4148455 | G/G | 43 | 74,14 | 27 | 79,41 | 285 | 75,60 | 202 | 74,81 | 1.00 (.-.) | . | 1.24 (0.77-2.00) | 0.37 | 1.00 | 0.99 | 0.95 |
| ABCC4 ---- tag |  | G/A or A/A | 15 | 25,86 | 7 | 20,59 | 92 | 24,40 | 68 | 25,19 | 1.03 (0.43-2.45) | 0.95 | 1.28 (0.76-2.17) | 0.35 | . | . | . |
| ABCC4 ---- tag | rs4148540 | C/C | 50 | 86,21 | 30 | 88,24 | 331 | 87,80 | 230 | 85,19 | 1.00 (.-.) | . | 1.24 (0.80-1.91) | 0.34 | 0.91 | 0.99 | 0.95 |
| ABCC4 ---- tag |  | C/T or T/T | 8 | 13,79 | 4 | 11,76 | 46 | 12,20 | 40 | 14,81 | 0.88 (0.26-2.97) | 0.84 | 1.18 (0.70-1.99) | 0.54 | . | . | . |
| ABCC4 ---- tag | rs4148542 | G/G | 12 | 20,69 | 3 | 8,82 | 105 | 27,85 | 68 | 25,19 | 1.00 (.-.) | . | 2.73 (0.37-20.05) | 0.32 | 0.37 | 0.99 | 0.98 |
| ABCC4 ---- tag |  | G/A or A/A | 46 | 79,31 | 31 | 91,18 | 272 | 72,15 | 202 | 74,81 | 2.37 (0.32-17.73) | 0.40 | 2.83 (0.39-20.52) | 0.30 | . | . | . |
| ABCC4 ---- tag | rs4148544 | G/G | 23 | 39,66 | 9 | 26,47 | 157 | 41,64 | 119 | 44,07 | 1.00 (.-.) | . | 1.74 (0.75-4.06) | 0.20 | 0.34 | 0.99 | 0.98 |
| ABCC4 ---- tag |  | G/A or A/A | 35 | 60,34 | 25 | 73,53 | 220 | 58,36 | 151 | 55,93 | 1.56 (0.62-3.91) | 0.34 | 1.74 (0.74-4.05) | 0.20 | . | . | . |
| ABCC4 ---- tag | rs4283094 | C/C | 9 | 15,52 | 6 | 17,65 | 102 | 27,06 | 61 | 22,59 | 1.00 (.-.) | . | 0.71 (0.27-1.86) | 0.49 | 0.23 | 0.99 | 0.98 |
| ABCC4 ---- tag |  | C/G or G/G | 49 | 84,48 | 28 | 82,35 | 275 | 72,94 | 209 | 77,41 | 0.62 (0.23-1.69) | 0.35 | 0.88 (0.34-2.23) | 0.78 | . | . | . |
| ABCC4 ---- tag | rs4636781 | A/A | 45 | 77,59 | 27 | 79,41 | 269 | 71,35 | 187 | 69,26 | 1.00 (.-.) | . | 1.25 (0.77-2.01) | 0.37 | 0.94 | 0.83 | 0.70 |
| ABCC4 ---- tag |  | A/G or G/G | 13 | 22,41 | 7 | 20,59 | 108 | 28,65 | 83 | 30,74 | 1.17 (0.49-2.80) | 0.72 | 1.41 (0.85-2.36) | 0.18 | . | . | . |
| ABCC4 ---- tag | rs4771910 | T/T | 30 | 51,72 | 16 | 47,06 | 178 | 47,21 | 129 | 47,78 | 1.00 (.-.) | . | 1.54 (0.82-2.90) | 0.18 | 0.35 | 0.83 | 0.70 |
| ABCC4 ---- tag |  | T/C or C/C | 28 | 48,28 | 18 | 52,94 | 199 | 52,79 | 141 | 52,22 | 1.25 (0.58-2.70) | 0.57 | 1.31 (0.70-2.47) | 0.40 | . | . | . |
| ABCC4 ---- tag | rs4773850 | T/T | 26 | 44,83 | 18 | 52,94 | 165 | 43,77 | 142 | 52,59 | 1.00 (.-.) | . | 1.55 (0.90-2.69) | 0.12 | 0.28 | 0.59 | 0.60 |
| ABCC4 ---- tag |  | T/G or G/G | 32 | 55,17 | 16 | 47,06 | 212 | 56,23 | 128 | 47,41 | 1.11 (0.52-2.37) | 0.78 | 1.11 (0.64-1.93) | 0.71 | . | . | . |
| ABCC4 ---- tag | rs7981095 | A/A | 39 | 67,24 | 20 | 58,82 | 248 | 65,78 | 171 | 63,33 | 1.00 (.-.) | . | 1.26 (0.75-2.11) | 0.38 | 0.92 | 0.99 | 0.95 |
| ABCC4 ---- tag |  | A/T or T/T | 19 | 32,76 | 14 | 41,18 | 129 | 34,22 | 99 | 36,67 | 1.04 (0.47-2.28) | 0.92 | 1.26 (0.73-2.16) | 0.41 | . | . | . |
| ABCC4 ---- tag | rs8001444 | C/C | 18 | 31,03 | 12 | 35,29 | 130 | 34,48 | 106 | 39,26 | 1.00 (.-.) | . | 1.32 (0.65-2.67) | 0.45 | 0.79 | 0.93 | 0.81 |
| ABCC4 ---- tag |  | C/T or T/T | 40 | 68,97 | 22 | 64,71 | 247 | 65,52 | 164 | 60,74 | 0.77 (0.34-1.74) | 0.54 | 0.91 (0.45-1.82) | 0.79 | . | . | . |
| ABCC4 ---- tag | rs931111 | T/T | 32 | 55,17 | 23 | 67,65 | 257 | 68,17 | 179 | 66,30 | 1.00 (.-.) | . | 1.28 (0.77-2.10) | 0.34 | 0.90 | 0.99 | 0.98 |
| ABCC4 ---- tag |  | T/C or C/C | 26 | 44,83 | 11 | 32,35 | 120 | 31,83 | 91 | 33,70 | 0.95 (0.42-2.12) | 0.89 | 1.14 (0.68-1.91) | 0.61 | . | . | . |
| ABCC4 ---- tag | rs943288 | T/T | 47 | 81,03 | 28 | 82,35 | 285 | 75,60 | 202 | 74,81 | 1.00 (.-.) | . | 1.27 (0.80-2.02) | 0.32 | 0.83 | 0.99 | 0.98 |
| ABCC4 ---- tag |  | T/A or A/A | 11 | 18,97 | 6 | 17,65 | 92 | 24,40 | 68 | 25,19 | 1.17 (0.47-2.90) | 0.74 | 1.33 (0.79-2.23) | 0.28 | . | . | . |
| ABCC4 ---- tag | rs943290 | A/A | 31 | 53,45 | 21 | 61,76 | 182 | 48,28 | 153 | 56,67 | 1.00 (.-.) | . | 1.21 (0.74-1.97) | 0.44 | 0.63 | 0.83 | 0.70 |
| ABCC4 ---- tag |  | A/G or G/G | 27 | 46,55 | 13 | 38,24 | 195 | 51,72 | 117 | 43,33 | 0.62 (0.27-1.44) | 0.27 | 0.93 (0.57-1.52) | 0.78 | . | . | . |
| ABCC4 ---- tag | rs9516530 | C/C | 31 | 53,45 | 23 | 67,65 | 208 | 55,17 | 153 | 56,67 | 1.00 (.-.) | . | 1.11 (0.67-1.85) | 0.69 | 0.47 | 0.88 | 0.71 |
| ABCC4 ---- tag |  | C/T or T/T | 27 | 46,55 | 11 | 32,35 | 169 | 44,83 | 117 | 43,33 | 0.68 (0.30-1.52) | 0.35 | 1.03 (0.61-1.72) | 0.92 | . | . | . |
| ABCC4 ---- tag | rs9516551 | C/C | 43 | 74,14 | 23 | 67,65 | 291 | 77,19 | 210 | 77,78 | 1.00 (.-.) | . | 1.22 (0.75-2.00) | 0.43 | 0.97 | 0.66 | 0.60 |
| ABCC4 ---- tag |  | C/A or A/A | 15 | 25,86 | 11 | 32,35 | 86 | 22,81 | 60 | 22,22 | 0.82 (0.37-1.83) | 0.63 | 1.02 (0.59-1.74) | 0.95 | . | . | . |
| ABCC4 ---- tag | rs9524822 | T/T | 45 | 77,59 | 21 | 61,76 | 240 | 63,66 | 173 | 64,07 | 1.00 (.-.) | . | 1.51 (0.89-2.57) | 0.13 | 0.22 | 0.85 | 0.70 |
| ABCC4 ---- tag |  | T/C or C/C | 13 | 22,41 | 13 | 38,24 | 137 | 36,34 | 97 | 35,93 | 1.48 (0.69-3.18) | 0.32 | 1.34 (0.77-2.32) | 0.30 | . | . | . |
| ABCC4 ---- tag | rs9524861 | G/G | 23 | 39,66 | 15 | 44,12 | 200 | 53,05 | 139 | 51,48 | 1.00 (.-.) | . | 0.90 (0.50-1.63) | 0.72 | 0.18 | 0.99 | 0.98 |
| ABCC4 ---- tag |  | G/C or C/C | 35 | 60,34 | 19 | 55,88 | 177 | 46,95 | 131 | 48,52 | 0.61 (0.29-1.30) | 0.20 | 0.95 (0.52-1.73) | 0.88 | . | . | . |
| ABCC4 ---- tag | rs9524902 | T/T | 15 | 25,86 | 8 | 23,53 | 98 | 25,99 | 93 | 34,44 | 1.00 (.-.) | . | 1.35 (0.61-2.96) | 0.46 | 0.74 | 0.66 | 0.60 |
| ABCC4 ---- tag |  | T/C or C/C | 43 | 74,14 | 26 | 76,47 | 279 | 74,01 | 177 | 65,56 | 0.72 (0.30-1.72) | 0.46 | 0.83 (0.38-1.82) | 0.65 | . | . | . |
| ABCC4 ---- tag | rs9556455 | G/G | 45 | 77,59 | 23 | 67,65 | 291 | 77,19 | 203 | 75,19 | 1.00 (.-.) | . | 1.38 (0.83-2.30) | 0.22 | 0.50 | 0.84 | 0.70 |
| ABCC4 ---- tag |  | G/A or A/A | 13 | 22,41 | 11 | 32,35 | 86 | 22,81 | 67 | 24,81 | 1.41 (0.63-3.15) | 0.40 | 1.45 (0.83-2.52) | 0.19 | . | . | . |
| ABCC4 ---- NA | rs9561778 | G/G | 36 | 62,07 | 24 | 70,59 | 235 | 62,33 | 190 | 70,37 | 1.00 (.-.) | . | 1.22 (0.77-1.93) | 0.39 | 0.78 | 0.99 | 0.98 |
| ABCC4 ---- NA |  | G/T or T/T | 22 | 37,93 | 10 | 29,41 | 142 | 37,67 | 80 | 29,63 | 0.74 (0.28-1.99) | 0.56 | 1.05 (0.64-1.72) | 0.85 | . | . | . |
| ABCC4 ---- tag | rs9561811 | C/C | 40 | 68,97 | 25 | 73,53 | 252 | 66,84 | 178 | 65,93 | 1.00 (.-.) | . | 1.40 (0.86-2.28) | 0.18 | 0.24 | 0.88 | 0.71 |
| ABCC4 ---- tag |  | C/T or T/T | 18 | 31,03 | 9 | 26,47 | 125 | 33,16 | 92 | 34,07 | 1.90 (0.81-4.47) | 0.14 | 1.52 (0.91-2.54) | 0.11 | . | . | . |
| ABCC4 ---- tag | rs9590183 | T/T | 52 | 89,66 | 32 | 94,12 | 321 | 85,15 | 236 | 87,41 | 1.00 (.-.) | . | 1.20 (0.78-1.84) | 0.41 | 0.58 | 0.93 | 0.87 |
| ABCC4 ---- tag |  | T/A or A/A | 6 | 10,34 | 2 | 5,88 | 56 | 14,85 | 34 | 12,59 | 0.56 (0.13-2.42) | 0.44 | 1.01 (0.58-1.77) | 0.97 | . | . | . |
| ABCC4 ---- tag | rs997777 | T/T | 26 | 44,83 | 16 | 47,06 | 185 | 49,07 | 134 | 49,63 | 1.00 (.-.) | . | 1.35 (0.77-2.38) | 0.29 | 0.66 | 0.99 | 0.98 |
| ABCC4 ---- tag |  | T/A or A/A | 32 | 55,17 | 18 | 52,94 | 192 | 50,93 | 136 | 50,37 | 1.11 (0.52-2.37) | 0.79 | 1.25 (0.71-2.20) | 0.44 | . | . | . |
| ADH1B ---- tag | rs1159918 | G/G | 21 | 36,21 | 12 | 35,29 | 172 | 45,62 | 130 | 48,15 | 1.00 (.-.) | . | 0.87 (0.45-1.67) | 0.68 | 0.24 | 0.93 | 0.64 |
| ADH1B ---- tag |  | G/T or T/T | 37 | 63,79 | 22 | 64,71 | 205 | 54,38 | 140 | 51,85 | 0.56 (0.25-1.22) | 0.15 | 0.81 (0.42-1.55) | 0.52 | . | . | . |
| ADH1B ---- candidate literature | rs1229984 | G/G | 51 | 87,93 | 32 | 94,12 | 340 | 90,19 | 245 | 90,74 | 1.00 (.-.) | . | 1.14 (0.74-1.74) | 0.55 | 0.25 | 0.96 | 0.64 |
| ADH1B ---- candidate literature |  | G/A or A/A | 7 | 12,07 | 2 | 5,88 | 37 | 9,81 | 25 | 9,26 | 0.40 (0.09-1.72) | 0.22 | 1.03 (0.57-1.84) | 0.93 | . | . | . |
| ADH1B ---- tag | rs12507573 | C/C | 17 | 29,31 | 14 | 41,18 | 115 | 30,50 | 73 | 27,04 | 1.00 (.-.) | . | 1.17 (0.59-2.35) | 0.65 | 0.84 | 0.99 | 0.85 |
| ADH1B ---- tag |  | C/A or A/A | 41 | 70,69 | 20 | 58,82 | 262 | 69,50 | 197 | 72,96 | 0.85 (0.38-1.87) | 0.68 | 1.09 (0.56-2.11) | 0.81 | . | . | . |
| ADH1B ---- tag | rs1693457 | T/T | 38 | 65,52 | 21 | 61,76 | 261 | 69,23 | 195 | 72,22 | 1.00 (.-.) | . | 1.01 (0.61-1.67) | 0.97 | 0.30 | 0.66 | 0.37 |
| ADH1B ---- tag |  | T/C or C/C | 20 | 34,48 | 13 | 38,24 | 116 | 30,77 | 75 | 27,78 | 0.55 (0.25-1.21) | 0.14 | 0.87 (0.51-1.49) | 0.61 | . | . | . |
| ADH1B ---- tag | rs2066701 | C/C | 30 | 51,72 | 9 | 26,47 | 178 | 47,21 | 123 | 45,56 | 1.00 (.-.) | . | 2.23 (1.02-4.87) | 0.05 | 0.05 | 0.93 | 0.64 |
| ADH1B ---- tag |  | C/T or T/T | 28 | 48,28 | 25 | 73,53 | 199 | 52,79 | 147 | 54,44 | 2.56 (1.08-6.11) | 0.03 | 2.35 (1.08-5.12) | 0.03 | . | . | . |
| ADH1C ---- tag | rs11936869 | C/C | 29 | 50,00 | 13 | 38,24 | 194 | 51,46 | 152 | 56,30 | 1.00 (.-.) | . | 1.43 (0.78-2.64) | 0.25 | 0.51 | 0.85 | 0.63 |
| ADH1C ---- tag |  | C/G or G/G | 29 | 50,00 | 21 | 61,76 | 183 | 48,54 | 118 | 43,70 | 1.16 (0.54-2.47) | 0.71 | 1.26 (0.68-2.34) | 0.46 | . | . | . |
| ADH1C ---- tag | rs1229849 | T/T | 33 | 56,90 | 20 | 58,82 | 204 | 54,11 | 130 | 48,15 | 1.00 (.-.) | . | 1.33 (0.77-2.30) | 0.30 | 0.68 | 0.99 | 0.95 |
| ADH1C ---- tag |  | T/A or A/A | 25 | 43,10 | 14 | 41,18 | 173 | 45,89 | 140 | 51,85 | 1.20 (0.56-2.57) | 0.65 | 1.34 (0.78-2.32) | 0.29 | . | . | . |
| ADH1C ---- tag | rs1229863 | A/A | 42 | 72,41 | 24 | 70,59 | 275 | 72,94 | 198 | 73,33 | 1.00 (.-.) | . | 1.37 (0.85-2.20) | 0.20 | 0.41 | 0.97 | 0.69 |
| ADH1C ---- tag |  | A/T or T/T | 16 | 27,59 | 10 | 29,41 | 102 | 27,06 | 72 | 26,67 | 1.37 (0.58-3.26) | 0.48 | 1.26 (0.75-2.11) | 0.39 | . | . | . |
| ADH1C ---- tag | rs1229980 | C/C | 52 | 89,66 | 30 | 88,24 | 343 | 90,98 | 240 | 88,89 | 1.00 (.-.) | . | 1.33 (0.86-2.06) | 0.20 | 0.16 | 0.93 | 0.63 |
| ADH1C ---- tag |  | C/G or G/G | 6 | 10,34 | 4 | 11,76 | 34 | 9,02 | 30 | 11,11 | 3.21 (0.94-10.98) | 0.06 | 1.50 (0.86-2.63) | 0.15 | . | . | . |
| ADH1C ---- candidate | rs1693482 | C/C | 27 | 46,55 | 15 | 44,12 | 157 | 41,64 | 106 | 39,26 | 1.00 (.-.) | . | 1.39 (0.76-2.54) | 0.28 | 0.61 | 0.93 | 0.63 |
| ADH1C ---- candidate |  | C/T or T/T | 31 | 53,45 | 19 | 55,88 | 220 | 58,36 | 164 | 60,74 | 1.10 (0.52-2.34) | 0.81 | 1.24 (0.69-2.25) | 0.47 | . | . | . |
| ADH1C ---- tag | rs2173201 | C/C | 33 | 56,90 | 14 | 41,18 | 220 | 58,36 | 160 | 59,26 | 1.00 (.-.) | . | 1.65 (0.92-2.96) | 0.09 | 0.12 | 0.93 | 0.63 |
| ADH1C ---- tag |  | C/A or A/A | 25 | 43,10 | 20 | 58,82 | 157 | 41,64 | 110 | 40,74 | 1.99 (0.93-4.23) | 0.08 | 1.74 (0.96-3.14) | 0.07 | . | . | . |
| ADH1C ---- tag | rs2298753 | T/T | 48 | 82,76 | 28 | 82,35 | 304 | 80,64 | 225 | 83,33 | 1.00 (.-.) | . | 1.26 (0.81-1.97) | 0.31 | 0.89 | 0.86 | 0.63 |
| ADH1C ---- tag |  | T/C or C/C | 10 | 17,24 | 6 | 17,65 | 73 | 19,36 | 45 | 16,67 | 0.87 (0.30-2.54) | 0.80 | 1.01 (0.59-1.74) | 0.96 | . | . | . |
| ADH1C ---- tag | rs2866152 | G/G | 37 | 63,79 | 24 | 70,59 | 232 | 61,54 | 153 | 56,67 | 1.00 (.-.) | . | 1.17 (0.71-1.94) | 0.53 | 0.71 | 0.99 | 0.95 |
| ADH1C ---- tag |  | G/C or C/C | 21 | 36,21 | 10 | 29,41 | 145 | 38,46 | 117 | 43,33 | 0.87 (0.39-1.94) | 0.73 | 1.19 (0.71-2.00) | 0.50 | . | . | . |
| ADH1C ---- tag | rs904096 | T/T | 27 | 46,55 | 15 | 44,12 | 154 | 40,85 | 105 | 38,89 | 1.00 (.-.) | . | 1.41 (0.77-2.57) | 0.26 | 0.58 | 0.93 | 0.63 |
| ADH1C ---- tag |  | T/G or G/G | 31 | 53,45 | 19 | 55,88 | 223 | 59,15 | 165 | 61,11 | 1.10 (0.52-2.34) | 0.81 | 1.23 (0.68-2.23) | 0.49 | . | . | . |
| BHMT ---- tag | rs10944 | A/A | 17 | 29,31 | 6 | 17,65 | 85 | 22,55 | 65 | 24,07 | 1.00 (.-.) | . | 1.75 (0.69-4.46) | 0.24 | 0.39 | 0.93 | 0.72 |
| BHMT ---- tag |  | A/C or C/C | 41 | 70,69 | 28 | 82,35 | 292 | 77,45 | 205 | 75,93 | 1.38 (0.52-3.67) | 0.52 | 1.57 (0.63-3.90) | 0.34 | . | . | . |
| BHMT ---- tag | rs12655567 | C/C | 22 | 37,93 | 14 | 41,18 | 133 | 35,28 | 104 | 38,52 | 1.00 (.-.) | . | 1.34 (0.73-2.46) | 0.35 | 0.83 | 0.66 | 0.21 |
| BHMT ---- tag |  | C/G or G/G | 36 | 62,07 | 20 | 58,82 | 244 | 64,72 | 166 | 61,48 | 0.84 (0.39-1.80) | 0.65 | 1.03 (0.57-1.86) | 0.93 | . | . | . |
| BHMT ---- tag | rs1291041 | G/G | 27 | 46,55 | 15 | 44,12 | 153 | 40,58 | 121 | 44,81 | 1.00 (.-.) | . | 1.42 (0.78-2.58) | 0.25 | 0.59 | 0.59 | 0.21 |
| BHMT ---- tag |  | G/T or T/T | 31 | 53,45 | 19 | 55,88 | 224 | 59,42 | 149 | 55,19 | 0.95 (0.44-2.04) | 0.89 | 1.08 (0.60-1.95) | 0.80 | . | . | . |
| BHMT ---- tag | rs16876500 | C/C | 47 | 81,03 | 28 | 82,35 | 304 | 80,64 | 210 | 77,78 | 1.00 (.-.) | . | 1.14 (0.73-1.80) | 0.56 | 0.38 | 0.99 | 0.72 |
| BHMT ---- tag |  | C/T or T/T | 11 | 18,97 | 6 | 17,65 | 73 | 19,36 | 60 | 22,22 | 0.80 (0.30-2.14) | 0.65 | 1.44 (0.86-2.41) | 0.17 | . | . | . |
| BHMT ---- tag | rs492842 | A/A | 24 | 41,38 | 13 | 38,24 | 142 | 37,67 | 109 | 40,37 | 1.00 (.-.) | . | 1.56 (0.79-3.07) | 0.20 | 0.38 | 0.99 | 0.72 |
| BHMT ---- tag |  | A/G or G/G | 34 | 58,62 | 21 | 61,76 | 235 | 62,33 | 161 | 59,63 | 1.27 (0.57-2.82) | 0.55 | 1.37 (0.70-2.68) | 0.35 | . | . | . |
| BHMT ---- tag | rs558133 | T/T | 25 | 43,10 | 18 | 52,94 | 184 | 48,81 | 125 | 46,30 | 1.00 (.-.) | . | 1.29 (0.71-2.34) | 0.41 | 0.88 | 0.93 | 0.72 |
| BHMT ---- tag |  | T/G or G/G | 33 | 56,90 | 16 | 47,06 | 193 | 51,19 | 145 | 53,70 | 1.17 (0.54-2.50) | 0.69 | 1.41 (0.78-2.56) | 0.26 | . | . | . |
| BHMT ---- tag | rs9637824 | A/A | 22 | 37,93 | 12 | 35,29 | 142 | 37,67 | 105 | 38,89 | 1.00 (.-.) | . | 1.58 (0.80-3.12) | 0.19 | 0.36 | 0.99 | 0.72 |
| BHMT ---- tag |  | A/G or G/G | 36 | 62,07 | 22 | 64,71 | 235 | 62,33 | 165 | 61,11 | 1.32 (0.59-2.95) | 0.49 | 1.42 (0.72-2.78) | 0.31 | . | . | . |
| BHMT2 ---- tag | rs16876512 | C/C | 47 | 81,03 | 28 | 82,35 | 301 | 79,84 | 210 | 77,78 | 1.00 (.-.) | . | 1.15 (0.73-1.80) | 0.55 | 0.40 | 0.99 | 0.71 |
| BHMT2 ---- tag |  | C/T or T/T | 11 | 18,97 | 6 | 17,65 | 76 | 20,16 | 60 | 22,22 | 0.80 (0.30-2.14) | 0.66 | 1.41 (0.84-2.37) | 0.19 | . | . | . |
| BHMT2 ---- tag | rs2461248 | T/T | 17 | 29,31 | 5 | 14,71 | 84 | 22,28 | 65 | 24,07 | 1.00 (.-.) | . | 2.13 (0.76-5.98) | 0.15 | 0.22 | 0.94 | 0.70 |
| BHMT2 ---- tag |  | T/A or A/A | 41 | 70,69 | 29 | 85,29 | 293 | 77,72 | 205 | 75,93 | 1.73 (0.59-5.06) | 0.31 | 1.90 (0.69-5.23) | 0.21 | . | . | . |
| BHMT2 ---- tag | rs2909856 | T/T | 25 | 43,10 | 15 | 44,12 | 155 | 41,11 | 115 | 42,59 | 1.00 (.-.) | . | 1.51 (0.81-2.80) | 0.19 | 0.40 | 0.93 | 0.70 |
| BHMT2 ---- tag |  | T/C or C/C | 33 | 56,90 | 19 | 55,88 | 222 | 58,89 | 155 | 57,41 | 1.21 (0.56-2.61) | 0.63 | 1.30 (0.70-2.39) | 0.41 | . | . | . |
| BHMT2 ---- tag | rs476620 | A/A | 22 | 37,93 | 12 | 35,29 | 142 | 37,67 | 104 | 38,52 | 1.00 (.-.) | . | 1.56 (0.79-3.10) | 0.20 | 0.38 | 0.99 | 0.71 |
| BHMT2 ---- tag |  | A/G or G/G | 36 | 62,07 | 22 | 64,71 | 235 | 62,33 | 166 | 61,48 | 1.32 (0.59-2.95) | 0.49 | 1.42 (0.73-2.80) | 0.30 | . | . | . |
| BHMT2 ---- candidate literature | rs626105 | G/G | 33 | 56,90 | 23 | 67,65 | 236 | 62,60 | 174 | 64,44 | 1.00 (.-.) | . | 1.29 (0.78-2.12) | 0.33 | 0.85 | 0.89 | 0.70 |
| BHMT2 ---- candidate literature |  | G/A or A/A | 25 | 43,10 | 11 | 32,35 | 141 | 37,40 | 96 | 35,56 | 0.91 (0.41-2.04) | 0.82 | 1.08 (0.64-1.81) | 0.78 | . | . | . |
| BHMT2 ---- tag | rs631305 | G/G | 37 | 63,79 | 26 | 76,47 | 263 | 69,76 | 191 | 70,74 | 1.00 (.-.) | . | 1.26 (0.78-2.02) | 0.34 | 0.94 | 0.93 | 0.70 |
| BHMT2 ---- tag |  | G/A or A/A | 21 | 36,21 | 8 | 23,53 | 114 | 30,24 | 79 | 29,26 | 0.92 (0.38-2.19) | 0.85 | 1.11 (0.67-1.86) | 0.68 | . | . | . |
| CBS ---- tag | rs11701048 | C/C | 50 | 86,21 | 28 | 82,35 | 318 | 84,35 | 237 | 87,78 | 1.00 (.-.) | . | 1.38 (0.87-2.18) | 0.17 | 0.24 | 0.99 | 0.79 |
| CBS ---- tag |  | C/T or T/T | 8 | 13,79 | 6 | 17,65 | 59 | 15,65 | 33 | 12,22 | 1.59 (0.63-3.99) | 0.33 | 1.17 (0.66-2.08) | 0.59 | . | . | . |
| CBS ---- tag | rs234706 | G/G | 25 | 43,10 | 11 | 32,35 | 166 | 44,03 | 108 | 40,00 | 1.00 (.-.) | . | 1.57 (0.77-3.23) | 0.22 | 0.40 | 0.99 | 0.79 |
| CBS ---- tag |  | G/A or A/A | 33 | 56,90 | 23 | 67,65 | 211 | 55,97 | 162 | 60,00 | 1.48 (0.66-3.33) | 0.34 | 1.62 (0.81-3.26) | 0.18 | . | . | . |
| CBS ---- tag | rs234711 | C/C | 35 | 60,34 | 19 | 55,88 | 227 | 60,21 | 149 | 55,19 | 1.00 (.-.) | . | 1.33 (0.77-2.31) | 0.31 | 0.68 | 0.93 | 0.79 |
| CBS ---- tag |  | C/A or A/A | 23 | 39,66 | 15 | 44,12 | 150 | 39,79 | 121 | 44,81 | 1.22 (0.56-2.64) | 0.61 | 1.37 (0.79-2.38) | 0.26 | . | . | . |
| CBS ---- candidate literature | rs234713 | G/G | 30 | 51,72 | 16 | 47,06 | 187 | 49,60 | 127 | 47,04 | 1.00 (.-.) | . | 1.54 (0.84-2.83) | 0.16 | 0.31 | 0.99 | 0.79 |
| CBS ---- candidate literature |  | G/A or A/A | 28 | 48,28 | 18 | 52,94 | 190 | 50,40 | 143 | 52,96 | 1.45 (0.68-3.10) | 0.33 | 1.47 (0.81-2.66) | 0.20 | . | . | . |
| CBS ---- tag | rs2839623 | T/T | 47 | 81,03 | 30 | 88,24 | 312 | 82,76 | 221 | 81,85 | 1.00 (.-.) | . | 1.23 (0.79-1.92) | 0.36 | 0.92 | 0.85 | 0.71 |
| CBS ---- tag |  | T/A or A/A | 11 | 18,97 | 4 | 11,76 | 65 | 17,24 | 49 | 18,15 | 0.92 (0.31-2.72) | 0.89 | 1.20 (0.71-2.05) | 0.50 | . | . | . |
| CBS ---- tag | rs2839626 | C/C | 26 | 44,83 | 14 | 41,18 | 187 | 49,60 | 123 | 45,56 | 1.00 (.-.) | . | 1.19 (0.64-2.20) | 0.58 | 0.84 | 0.99 | 0.79 |
| CBS ---- tag |  | C/T or T/T | 32 | 55,17 | 20 | 58,82 | 190 | 50,40 | 147 | 54,44 | 1.05 (0.49-2.25) | 0.90 | 1.36 (0.74-2.50) | 0.33 | . | . | . |
| CBS ---- tag | rs422791 | T/T | 28 | 48,28 | 20 | 58,82 | 196 | 51,99 | 118 | 43,70 | 1.00 (.-.) | . | 1.07 (0.62-1.83) | 0.81 | 0.48 | 0.59 | 0.14 |
| CBS ---- tag |  | T/C or C/C | 30 | 51,72 | 14 | 41,18 | 181 | 48,01 | 152 | 56,30 | 0.96 (0.44-2.09) | 0.92 | 1.38 (0.81-2.35) | 0.24 | . | . | . |
| CBS ---- tag | rs706209 | C/C | 18 | 31,03 | 8 | 23,53 | 124 | 32,89 | 86 | 31,85 | 1.00 (.-.) | . | 1.13 (0.48-2.68) | 0.78 | 0.81 | 0.99 | 0.79 |
| CBS ---- tag |  | C/T or T/T | 40 | 68,97 | 26 | 76,47 | 253 | 67,11 | 184 | 68,15 | 0.94 (0.37-2.36) | 0.89 | 1.20 (0.52-2.76) | 0.67 | . | . | . |
| CBS ---- tag | rs719037 | A/A | 19 | 32,76 | 14 | 41,18 | 129 | 34,22 | 75 | 27,78 | 1.00 (.-.) | . | 1.21 (0.63-2.32) | 0.56 | 0.97 | 0.59 | 0.14 |
| CBS ---- tag |  | A/G or G/G | 39 | 67,24 | 20 | 58,82 | 248 | 65,78 | 195 | 72,22 | 1.10 (0.51-2.38) | 0.81 | 1.36 (0.73-2.51) | 0.34 | . | . | . |
| CBS ---- tag | rs719038 | T/T | 27 | 46,55 | 12 | 35,29 | 170 | 45,09 | 120 | 44,44 | 1.00 (.-.) | . | 1.25 (0.64-2.42) | 0.51 | 0.98 | 0.99 | 0.79 |
| CBS ---- tag |  | T/C or C/C | 31 | 53,45 | 22 | 64,71 | 207 | 54,91 | 150 | 55,56 | 1.00 (0.45-2.22) | 1.00 | 1.24 (0.64-2.40) | 0.52 | . | . | . |
| DHFR ---- tag | rs10474632 | G/G | 52 | 89,66 | 29 | 85,29 | 313 | 83,02 | 225 | 83,33 | 1.00 (.-.) | . | 1.26 (0.81-1.97) | 0.30 | 0.88 | 0.59 | 0.20 |
| DHFR ---- tag |  | G/A or A/A | 6 | 10,34 | 5 | 14,71 | 64 | 16,98 | 45 | 16,67 | 0.86 (0.29-2.53) | 0.79 | 1.00 (0.58-1.70) | 0.99 | . | . | . |
| DHFR ---- tag | rs11951910 | T/T | 47 | 81,03 | 26 | 76,47 | 306 | 81,17 | 220 | 81,48 | 1.00 (.-.) | . | 1.11 (0.70-1.77) | 0.64 | 0.30 | 0.99 | 0.89 |
| DHFR ---- tag |  | T/C or C/C | 11 | 18,97 | 8 | 23,53 | 71 | 18,83 | 50 | 18,52 | 0.74 (0.29-1.85) | 0.52 | 1.36 (0.80-2.32) | 0.26 | . | . | . |
| DHFR ---- tag | rs1643665 | T/T | 30 | 51,72 | 14 | 41,18 | 176 | 46,68 | 119 | 44,07 | 1.00 (.-.) | . | 1.14 (0.59-2.19) | 0.69 | 0.74 | 0.99 | 0.89 |
| DHFR ---- tag |  | T/C or C/C | 28 | 48,28 | 20 | 58,82 | 201 | 53,32 | 151 | 55,93 | 0.94 (0.43-2.04) | 0.87 | 1.23 (0.65-2.33) | 0.53 | . | . | . |
| DHFR ---- tag | rs1650717 | T/T | 26 | 44,83 | 17 | 50,00 | 208 | 55,17 | 147 | 54,44 | 1.00 (.-.) | . | 1.10 (0.61-1.98) | 0.74 | 0.54 | 0.85 | 0.59 |
| DHFR ---- tag |  | T/G or G/G | 32 | 55,17 | 17 | 50,00 | 169 | 44,83 | 123 | 45,56 | 0.91 (0.43-1.93) | 0.80 | 1.29 (0.71-2.33) | 0.41 | . | . | . |
| DHFR ---- tag | rs1805355 | G/G | 48 | 82,76 | 26 | 76,47 | 328 | 87,00 | 239 | 88,52 | 1.00 (.-.) | . | 1.49 (0.93-2.39) | 0.09 | 0.05 | 0.99 | 0.91 |
| DHFR ---- tag |  | G/A or A/A | 10 | 17,24 | 8 | 23,53 | 49 | 13,00 | 31 | 11,48 | 2.26 (0.94-5.40) | 0.07 | 1.17 (0.64-2.13) | 0.61 | . | . | . |
| DHFR ---- tag | rs6151617 | A/A | 24 | 41,38 | 12 | 35,29 | 132 | 35,01 | 94 | 34,81 | 1.00 (.-.) | . | 1.20 (0.59-2.47) | 0.62 | 0.92 | 0.95 | 0.89 |
| DHFR ---- tag |  | A/G or G/G | 34 | 58,62 | 22 | 64,71 | 245 | 64,99 | 176 | 65,19 | 0.93 (0.41-2.10) | 0.87 | 1.18 (0.58-2.37) | 0.65 | . | . | . |
| DHFR ---- tag | rs6864493 | T/T | 36 | 62,07 | 19 | 55,88 | 210 | 55,70 | 147 | 54,44 | 1.00 (.-.) | . | 0.98 (0.56-1.70) | 0.94 | 0.24 | 0.99 | 0.89 |
| DHFR ---- tag |  | T/C or C/C | 22 | 37,93 | 15 | 44,12 | 167 | 44,30 | 123 | 45,56 | 0.63 (0.29-1.38) | 0.25 | 1.01 (0.58-1.76) | 0.97 | . | . | . |
| DHFR ---- tag | rs836788 | G/G | 25 | 43,10 | 19 | 55,88 | 163 | 43,24 | 112 | 41,48 | 1.00 (.-.) | . | 1.41 (0.81-2.45) | 0.22 | 0.51 | 0.99 | 0.89 |
| DHFR ---- tag |  | G/A or A/A | 33 | 56,90 | 15 | 44,12 | 214 | 56,76 | 158 | 58,52 | 1.21 (0.57-2.59) | 0.62 | 1.30 (0.76-2.23) | 0.34 | . | . | . |
| DHFR ---- tag | rs836790 | A/A | 37 | 63,79 | 22 | 64,71 | 270 | 71,62 | 183 | 67,78 | 1.00 (.-.) | . | 1.18 (0.71-1.97) | 0.53 | 0.69 | 0.85 | 0.59 |
| DHFR ---- tag |  | A/G or G/G | 21 | 36,21 | 12 | 35,29 | 107 | 28,38 | 87 | 32,22 | 0.99 (0.46-2.14) | 0.98 | 1.38 (0.80-2.37) | 0.25 | . | . | . |
| DHFR ---- tag | rs836817 | G/G | 25 | 43,10 | 21 | 61,76 | 186 | 49,34 | 120 | 44,44 | 1.00 (.-.) | . | 1.31 (0.78-2.20) | 0.32 | 0.63 | 0.85 | 0.59 |
| DHFR ---- tag |  | G/T or T/T | 33 | 56,90 | 13 | 38,24 | 191 | 50,66 | 150 | 55,56 | 1.33 (0.60-2.93) | 0.48 | 1.41 (0.85-2.37) | 0.19 | . | . | . |
| DNMT1 ---- candidate | rs2228612 | A/A | 48 | 82,76 | 27 | 79,41 | 338 | 89,66 | 232 | 85,93 | 1.00 (.-.) | . | 1.29 (0.82-2.02) | 0.28 | 0.75 | 0.93 | 0.43 |
| DNMT1 ---- candidate |  | A/G or G/G | 10 | 17,24 | 7 | 20,59 | 39 | 10,34 | 38 | 14,07 | 1.36 (0.51-3.65) | 0.54 | 1.48 (0.85-2.58) | 0.17 | . | . | . |
| DNMT3A ---- tag | rs10460566 | A/A | 34 | 58,62 | 22 | 64,71 | 222 | 58,89 | 160 | 59,26 | 1.00 (.-.) | . | 1.47 (0.88-2.45) | 0.14 | 0.23 | 0.99 | 0.92 |
| DNMT3A ---- tag |  | A/G or G/G | 24 | 41,38 | 12 | 35,29 | 155 | 41,11 | 110 | 40,74 | 1.55 (0.70-3.42) | 0.28 | 1.35 (0.80-2.28) | 0.26 | . | . | . |
| DNMT3A ---- candidate literature | rs11695471 | T/T | 28 | 48,28 | 12 | 35,29 | 164 | 43,50 | 117 | 43,33 | 1.00 (.-.) | . | 1.18 (0.64-2.18) | 0.59 | 0.85 | 0.89 | 0.89 |
| DNMT3A ---- candidate literature |  | T/A or A/A | 30 | 51,72 | 22 | 64,71 | 213 | 56,50 | 153 | 56,67 | 1.12 (0.52-2.41) | 0.77 | 1.44 (0.79-2.62) | 0.24 | . | . | . |
| DNMT3A ---- tag | rs11887120 | C/C | 20 | 34,48 | 13 | 38,24 | 133 | 35,28 | 96 | 35,56 | 1.00 (.-.) | . | 1.05 (0.52-2.11) | 0.90 | 0.57 | 0.99 | 0.92 |
| DNMT3A ---- tag |  | C/T or T/T | 38 | 65,52 | 21 | 61,76 | 244 | 64,72 | 174 | 64,44 | 0.82 (0.37-1.84) | 0.64 | 1.11 (0.56-2.21) | 0.77 | . | . | . |
| DNMT3A ---- tag | rs12991495 | T/T | 30 | 51,72 | 14 | 41,18 | 173 | 45,89 | 127 | 47,04 | 1.00 (.-.) | . | 1.15 (0.64-2.06) | 0.65 | 0.71 | 0.86 | 0.89 |
| DNMT3A ---- tag |  | T/C or C/C | 28 | 48,28 | 20 | 58,82 | 204 | 54,11 | 143 | 52,96 | 0.98 (0.45-2.10) | 0.95 | 1.31 (0.73-2.34) | 0.37 | . | . | . |
| DNMT3A ---- tag | rs13401241 | A/A | 21 | 36,21 | 8 | 23,53 | 108 | 28,65 | 72 | 26,67 | 1.00 (.-.) | . | 2.11 (0.89-5.01) | 0.09 | 0.14 | 0.99 | 0.92 |
| DNMT3A ---- tag |  | A/C or C/C | 37 | 63,79 | 26 | 76,47 | 269 | 71,35 | 198 | 73,33 | 1.69 (0.68-4.24) | 0.26 | 1.77 (0.76-4.11) | 0.19 | . | . | . |
| DNMT3A ---- candidate literature | rs13420827 | C/C | 39 | 67,24 | 24 | 70,59 | 230 | 61,01 | 188 | 69,63 | 1.00 (.-.) | . | 1.18 (0.71-1.97) | 0.51 | 0.78 | 0.99 | 0.89 |
| DNMT3A ---- candidate literature |  | C/G or G/G | 19 | 32,76 | 10 | 29,41 | 147 | 38,99 | 82 | 30,37 | 0.78 (0.35-1.72) | 0.54 | 1.04 (0.61-1.77) | 0.89 | . | . | . |
| DNMT3A ---- tag | rs13428812 | A/A | 24 | 41,38 | 21 | 61,76 | 188 | 49,87 | 119 | 44,07 | 1.00 (.-.) | . | 1.01 (0.59-1.72) | 0.98 | 0.26 | 0.93 | 0.89 |
| DNMT3A ---- tag |  | A/G or G/G | 34 | 58,62 | 13 | 38,24 | 189 | 50,13 | 151 | 55,93 | 0.78 (0.36-1.68) | 0.53 | 1.24 (0.72-2.13) | 0.43 | . | . | . |
| DNMT3A ---- tag | rs4665287 | C/C | 40 | 68,97 | 24 | 70,59 | 236 | 62,60 | 192 | 71,11 | 1.00 (.-.) | . | 1.19 (0.72-1.98) | 0.50 | 0.82 | 0.95 | 0.89 |
| DNMT3A ---- tag |  | C/T or T/T | 18 | 31,03 | 10 | 29,41 | 141 | 37,40 | 78 | 28,89 | 0.78 (0.35-1.72) | 0.53 | 1.02 (0.60-1.74) | 0.94 | . | . | . |
| DNMT3B ---- tag | rs13045669 | A/A | 54 | 93,10 | 31 | 91,18 | 348 | 92,31 | 251 | 92,96 | 1.00 (.-.) | . | 1.26 (0.82-1.93) | 0.30 | 0.82 | 0.93 | 0.97 |
| DNMT3B ---- tag |  | A/G or G/G | 4 | 6,90 | 3 | 8,82 | 29 | 7,69 | 19 | 7,04 | 0.90 (0.21-3.92) | 0.89 | 0.94 (0.48-1.85) | 0.87 | . | . | . |
| DNMT3B ---- tag | rs17123673 | A/A | 54 | 93,10 | 29 | 85,29 | 347 | 92,04 | 248 | 91,85 | 1.00 (.-.) | . | 1.31 (0.84-2.03) | 0.24 | 0.50 | 0.85 | 0.97 |
| DNMT3B ---- tag |  | A/G or G/G | 4 | 6,90 | 5 | 14,71 | 30 | 7,96 | 22 | 8,15 | 1.35 (0.46-3.96) | 0.59 | 1.15 (0.62-2.15) | 0.66 | . | . | . |
| DNMT3B ---- tag | rs183603 | A/A | 30 | 51,72 | 20 | 58,82 | 200 | 53,05 | 147 | 54,44 | 1.00 (.-.) | . | 0.97 (0.58-1.64) | 0.92 | 0.19 | 0.88 | 0.97 |
| DNMT3B ---- tag |  | A/G or G/G | 28 | 48,28 | 14 | 41,18 | 177 | 46,95 | 123 | 45,56 | 0.56 (0.26-1.21) | 0.14 | 0.93 (0.55-1.58) | 0.79 | . | . | . |
| DNMT3B ---- tag | rs2235760 | C/C | 39 | 67,24 | 23 | 67,65 | 267 | 70,82 | 200 | 74,07 | 1.00 (.-.) | . | 1.39 (0.86-2.25) | 0.18 | 0.35 | 0.99 | 0.97 |
| DNMT3B ---- tag |  | C/T or T/T | 19 | 32,76 | 11 | 32,35 | 110 | 29,18 | 70 | 25,93 | 1.24 (0.54-2.87) | 0.61 | 1.12 (0.66-1.90) | 0.67 | . | . | . |
| DNMT3B ---- tag | rs2424908 | C/C | 35 | 60,34 | 25 | 73,53 | 232 | 61,54 | 176 | 65,19 | 1.00 (.-.) | . | 0.98 (0.61-1.57) | 0.94 | 0.10 | 0.99 | 0.97 |
| DNMT3B ---- tag |  | C/T or T/T | 23 | 39,66 | 9 | 26,47 | 145 | 38,46 | 94 | 34,81 | 0.46 (0.19-1.09) | 0.08 | 0.94 (0.57-1.54) | 0.79 | . | . | . |
| DNMT3B ---- candidate literature | rs2424909 | T/T | 17 | 29,31 | 14 | 41,18 | 139 | 36,87 | 118 | 43,70 | 1.00 (.-.) | . | 1.01 (0.56-1.82) | 0.99 | 0.40 | 0.99 | 0.97 |
| DNMT3B ---- candidate literature |  | T/C or C/C | 41 | 70,69 | 20 | 58,82 | 238 | 63,13 | 152 | 56,30 | 0.60 (0.28-1.29) | 0.19 | 0.86 (0.47-1.55) | 0.61 | . | . | . |
| DNMT3B ---- tag | rs4911108 | A/A | 19 | 32,76 | 14 | 41,18 | 150 | 39,79 | 123 | 45,56 | 1.00 (.-.) | . | 1.03 (0.57-1.86) | 0.92 | 0.44 | 0.99 | 0.97 |
| DNMT3B ---- tag |  | A/G or G/G | 39 | 67,24 | 20 | 58,82 | 227 | 60,21 | 147 | 54,44 | 0.63 (0.30-1.36) | 0.24 | 0.89 (0.50-1.61) | 0.71 | . | . | . |
| DNMT3B ---- tag | rs6058896 | C/C | 50 | 86,21 | 31 | 91,18 | 336 | 89,12 | 241 | 89,26 | 1.00 (.-.) | . | 1.26 (0.82-1.95) | 0.29 | 0.80 | 0.99 | 0.97 |
| DNMT3B ---- tag |  | C/T or T/T | 8 | 13,79 | 3 | 8,82 | 41 | 10,88 | 29 | 10,74 | 1.15 (0.34-3.86) | 0.82 | 1.22 (0.68-2.19) | 0.50 | . | . | . |
| DNMT3B ---- tag | rs6119954 | G/G | 36 | 62,07 | 24 | 70,59 | 263 | 69,76 | 199 | 73,70 | 1.00 (.-.) | . | 1.25 (0.77-2.02) | 0.37 | 0.95 | 0.99 | 0.97 |
| DNMT3B ---- tag |  | G/A or A/A | 22 | 37,93 | 10 | 29,41 | 114 | 30,24 | 71 | 26,30 | 0.88 (0.38-2.03) | 0.77 | 1.07 (0.63-1.81) | 0.81 | . | . | . |
| DNMT3B ---- tag | rs6579038 | A/A | 50 | 86,21 | 33 | 97,06 | 334 | 88,59 | 240 | 88,89 | 1.00 (.-.) | . | 1.22 (0.80-1.86) | 0.36 | 0.64 | 0.99 | 0.97 |
| DNMT3B ---- tag |  | A/G or G/G | 8 | 13,79 | 1 | 2,94 | 43 | 11,41 | 30 | 11,11 | 0.61 (0.08-4.54) | 0.63 | 1.17 (0.67-2.06) | 0.58 | . | . | . |
| DPYD ---- tag | rs1034215 | C/C | 38 | 65,52 | 19 | 55,88 | 209 | 55,44 | 173 | 64,07 | 1.00 (.-.) | . | 1.90 (1.07-3.37) | 0.03 | 0.01 | 0.87 | 0.53 |
| DPYD ---- tag |  | C/T or T/T | 20 | 34,48 | 15 | 44,12 | 168 | 44,56 | 97 | 35,93 | 2.17 (1.00-4.67) | 0.05 | 1.46 (0.80-2.64) | 0.22 | . | . | . |
| DPYD ---- tag | rs10783058 | T/T | 30 | 51,72 | 18 | 52,94 | 153 | 40,58 | 100 | 37,04 | 1.00 (.-.) | . | 0.91 (0.51-1.62) | 0.75 | 0.17 | 0.59 | 0.29 |
| DPYD ---- tag |  | T/C or C/C | 28 | 48,28 | 16 | 47,06 | 224 | 59,42 | 170 | 62,96 | 0.72 (0.34-1.54) | 0.40 | 1.16 (0.66-2.03) | 0.61 | . | . | . |
| DPYD ---- tag | rs10783070 | C/C | 43 | 74,14 | 27 | 79,41 | 273 | 72,41 | 184 | 68,15 | 1.00 (.-.) | . | 1.17 (0.73-1.88) | 0.52 | 0.68 | 0.83 | 0.51 |
| DPYD ---- tag |  | C/T or T/T | 15 | 25,86 | 7 | 20,59 | 104 | 27,59 | 86 | 31,85 | 1.06 (0.45-2.53) | 0.89 | 1.50 (0.91-2.48) | 0.11 | . | . | . |
| DPYD ---- tag | rs10875048 | G/G | 40 | 68,97 | 23 | 67,65 | 262 | 69,50 | 180 | 66,67 | 1.00 (.-.) | . | 1.15 (0.69-1.92) | 0.59 | 0.66 | 0.93 | 0.63 |
| DPYD ---- tag |  | G/A or A/A | 18 | 31,03 | 11 | 32,35 | 115 | 30,50 | 90 | 33,33 | 0.78 (0.36-1.68) | 0.53 | 1.07 (0.63-1.84) | 0.79 | . | . | . |
| DPYD ---- tag | rs10875055 | C/C | 31 | 53,45 | 6 | 17,65 | 109 | 28,91 | 51 | 18,89 | 1.00 (.-.) | . | 1.66 (0.70-3.94) | 0.25 | 0.33 | 0.59 | 0.29 |
| DPYD ---- tag |  | C/T or T/T | 27 | 46,55 | 28 | 82,35 | 268 | 71,09 | 219 | 81,11 | 2.54 (1.03-6.30) | 0.04 | 2.65 (1.16-6.05) | 0.02 | . | . | . |
| DPYD ---- tag | rs10875079 | A/A | 10 | 17,24 | 9 | 26,47 | 100 | 26,53 | 78 | 28,89 | 1.00 (.-.) | . | 1.54 (0.60-3.96) | 0.37 | 0.60 | 0.99 | 0.81 |
| DPYD ---- tag |  | A/G or G/G | 48 | 82,76 | 25 | 73,53 | 277 | 73,47 | 192 | 71,11 | 1.23 (0.45-3.31) | 0.69 | 1.44 (0.57-3.64) | 0.44 | . | . | . |
| DPYD ---- tag | rs10875085 | A/A | 39 | 67,24 | 24 | 70,59 | 264 | 70,03 | 181 | 67,04 | 1.00 (.-.) | . | 1.19 (0.73-1.94) | 0.48 | 0.75 | 0.93 | 0.69 |
| DPYD ---- tag |  | A/T or T/T | 19 | 32,76 | 10 | 29,41 | 113 | 29,97 | 89 | 32,96 | 0.79 (0.34-1.85) | 0.59 | 1.09 (0.66-1.82) | 0.73 | . | . | . |
| DPYD ---- tag | rs10875097 | G/G | 42 | 72,41 | 20 | 58,82 | 260 | 68,97 | 179 | 66,30 | 1.00 (.-.) | . | 1.73 (0.99-3.04) | 0.06 | 0.04 | 0.59 | 0.51 |
| DPYD ---- tag |  | G/A or A/A | 16 | 27,59 | 14 | 41,18 | 117 | 31,03 | 91 | 33,70 | 2.40 (1.13-5.09) | 0.02 | 1.81 (1.00-3.27) | 0.05 | . | . | . |
| DPYD ---- tag | rs11165781 | T/T | 40 | 68,97 | 25 | 73,53 | 264 | 70,03 | 177 | 65,56 | 1.00 (.-.) | . | 1.12 (0.70-1.78) | 0.64 | 0.41 | 0.99 | 0.81 |
| DPYD ---- tag |  | T/C or C/C | 18 | 31,03 | 9 | 26,47 | 113 | 29,97 | 93 | 34,44 | 0.75 (0.28-2.02) | 0.58 | 1.28 (0.78-2.08) | 0.32 | . | . | . |
| DPYD ---- tag | rs11165783 | T/T | 32 | 55,17 | 19 | 55,88 | 207 | 54,91 | 148 | 54,81 | 1.00 (.-.) | . | 1.43 (0.81-2.53) | 0.22 | 0.43 | 0.83 | 0.51 |
| DPYD ---- tag |  | T/C or C/C | 26 | 44,83 | 15 | 44,12 | 170 | 45,09 | 122 | 45,19 | 1.48 (0.69-3.15) | 0.31 | 1.54 (0.87-2.74) | 0.14 | . | . | . |
| DPYD ---- tag | rs11165873 | A/A | 14 | 24,14 | 7 | 20,59 | 105 | 27,85 | 82 | 30,37 | 1.00 (.-.) | . | 1.44 (0.57-3.65) | 0.44 | 0.69 | 0.83 | 0.51 |
| DPYD ---- tag |  | A/T or T/T | 44 | 75,86 | 27 | 79,41 | 272 | 72,15 | 188 | 69,63 | 1.10 (0.41-2.98) | 0.84 | 1.30 (0.52-3.24) | 0.58 | . | . | . |
| DPYD ---- tag | rs11165875 | T/T | 24 | 41,38 | 16 | 47,06 | 149 | 39,52 | 109 | 40,37 | 1.00 (.-.) | . | 1.36 (0.75-2.44) | 0.31 | 0.74 | 0.85 | 0.51 |
| DPYD ---- tag |  | T/C or C/C | 34 | 58,62 | 18 | 52,94 | 228 | 60,48 | 161 | 59,63 | 1.00 (0.47-2.13) | 1.00 | 1.18 (0.66-2.09) | 0.57 | . | . | . |
| DPYD ---- tag | rs11165881 | T/T | 22 | 37,93 | 17 | 50,00 | 129 | 34,22 | 90 | 33,33 | 1.00 (.-.) | . | 0.87 (0.46-1.64) | 0.66 | 0.17 | 0.94 | 0.71 |
| DPYD ---- tag |  | T/C or C/C | 36 | 62,07 | 17 | 50,00 | 248 | 65,78 | 180 | 66,67 | 0.77 (0.35-1.66) | 0.50 | 1.21 (0.66-2.21) | 0.54 | . | . | . |
| DPYD ---- tag | rs11587873 | C/C | 34 | 58,62 | 21 | 61,76 | 202 | 53,58 | 172 | 63,70 | 1.00 (.-.) | . | 1.19 (0.70-2.03) | 0.51 | 0.92 | 0.85 | 0.51 |
| DPYD ---- tag |  | C/T or T/T | 24 | 41,38 | 13 | 38,24 | 175 | 46,42 | 98 | 36,30 | 0.73 (0.34-1.56) | 0.42 | 0.91 (0.53-1.58) | 0.74 | . | . | . |
| DPYD ---- tag | rs12030174 | C/C | 42 | 72,41 | 24 | 70,59 | 278 | 73,74 | 193 | 71,48 | 1.00 (.-.) | . | 1.24 (0.77-2.01) | 0.38 | 0.99 | 0.92 | 0.61 |
| DPYD ---- tag |  | C/T or T/T | 16 | 27,59 | 10 | 29,41 | 99 | 26,26 | 77 | 28,52 | 0.85 (0.36-1.98) | 0.70 | 1.05 (0.62-1.76) | 0.86 | . | . | . |
| DPYD ---- tag | rs12046744 | A/A | 30 | 51,72 | 19 | 55,88 | 205 | 54,38 | 152 | 56,30 | 1.00 (.-.) | . | 1.34 (0.79-2.29) | 0.28 | 0.61 | 0.99 | 0.92 |
| DPYD ---- tag |  | A/C or C/C | 28 | 48,28 | 15 | 44,12 | 172 | 45,62 | 118 | 43,70 | 1.39 (0.65-2.98) | 0.40 | 1.50 (0.87-2.59) | 0.14 | . | . | . |
| DPYD ---- tag | rs12047910 | G/G | 43 | 74,14 | 24 | 70,59 | 279 | 74,01 | 202 | 74,81 | 1.00 (.-.) | . | 1.37 (0.83-2.27) | 0.22 | 0.46 | 0.85 | 0.51 |
| DPYD ---- tag |  | G/A or A/A | 15 | 25,86 | 10 | 29,41 | 98 | 25,99 | 68 | 25,19 | 1.17 (0.53-2.55) | 0.70 | 1.16 (0.68-2.00) | 0.58 | . | . | . |
| DPYD ---- tag | rs12073044 | T/T | 50 | 86,21 | 29 | 85,29 | 285 | 75,60 | 216 | 80,00 | 1.00 (.-.) | . | 1.33 (0.85-2.08) | 0.21 | 0.59 | 0.85 | 0.51 |
| DPYD ---- tag |  | T/A or A/A | 8 | 13,79 | 5 | 14,71 | 92 | 24,40 | 54 | 20,00 | 1.02 (0.35-3.02) | 0.97 | 0.99 (0.59-1.65) | 0.96 | . | . | . |
| DPYD ---- tag | rs12126093 | T/T | 25 | 43,10 | 12 | 35,29 | 194 | 51,46 | 144 | 53,33 | 1.00 (.-.) | . | 1.24 (0.64-2.40) | 0.53 | 0.96 | 0.93 | 0.62 |
| DPYD ---- tag |  | T/C or C/C | 33 | 56,90 | 22 | 64,71 | 183 | 48,54 | 126 | 46,67 | 0.93 (0.42-2.05) | 0.86 | 1.13 (0.58-2.19) | 0.72 | . | . | . |
| DPYD ---- tag | rs12134028 | C/C | 52 | 89,66 | 29 | 85,29 | 344 | 91,25 | 238 | 88,15 | 1.00 (.-.) | . | 1.12 (0.72-1.75) | 0.60 | 0.30 | 0.83 | 0.51 |
| DPYD ---- tag |  | C/T or T/T | 6 | 10,34 | 5 | 14,71 | 33 | 8,75 | 32 | 11,85 | 0.51 (0.15-1.72) | 0.28 | 1.09 (0.62-1.91) | 0.77 | . | . | . |
| DPYD ---- tag | rs12740796 | T/T | 37 | 63,79 | 28 | 82,35 | 278 | 73,74 | 210 | 77,78 | 1.00 (.-.) | . | 1.20 (0.77-1.87) | 0.42 | 0.62 | 0.99 | 0.85 |
| DPYD ---- tag |  | T/C or C/C | 21 | 36,21 | 6 | 17,65 | 99 | 26,26 | 60 | 22,22 | 0.69 (0.23-2.02) | 0.49 | 1.09 (0.66-1.78) | 0.75 | . | . | . |
| DPYD ---- tag | rs1333717 | A/A | 37 | 63,79 | 18 | 52,94 | 202 | 53,58 | 163 | 60,37 | 1.00 (.-.) | . | 1.87 (1.05-3.32) | 0.03 | 0.02 | 0.87 | 0.53 |
| DPYD ---- tag |  | A/G or G/G | 21 | 36,21 | 16 | 47,06 | 175 | 46,42 | 107 | 39,63 | 2.11 (0.98-4.56) | 0.06 | 1.50 (0.83-2.71) | 0.18 | . | . | . |
| DPYD ---- tag | rs1413228 | A/A | 46 | 79,31 | 31 | 91,18 | 306 | 81,17 | 206 | 76,30 | 1.00 (.-.) | . | 1.06 (0.69-1.63) | 0.80 | 0.06 | 0.99 | 0.93 |
| DPYD ---- tag |  | A/G or G/G | 12 | 20,69 | 3 | 8,82 | 71 | 18,83 | 64 | 23,70 | 0.37 (0.09-1.58) | 0.18 | 1.32 (0.80-2.16) | 0.27 | . | . | . |
| DPYD ---- tag | rs1415681 | G/G | 43 | 74,14 | 29 | 85,29 | 270 | 71,62 | 202 | 74,81 | 1.00 (.-.) | . | 1.10 (0.70-1.73) | 0.69 | 0.26 | 0.97 | 0.78 |
| DPYD ---- tag |  | G/T or T/T | 15 | 25,86 | 5 | 14,71 | 107 | 28,38 | 68 | 25,19 | 0.64 (0.22-1.87) | 0.42 | 1.29 (0.79-2.12) | 0.31 | . | . | . |
| DPYD ---- tag | rs1514495 | C/C | 31 | 53,45 | 19 | 55,88 | 238 | 63,13 | 158 | 58,52 | 1.00 (.-.) | . | 1.19 (0.69-2.03) | 0.53 | 0.80 | 0.99 | 0.89 |
| DPYD ---- tag |  | C/T or T/T | 27 | 46,55 | 15 | 44,12 | 139 | 36,87 | 112 | 41,48 | 0.87 (0.40-1.90) | 0.73 | 1.15 (0.66-1.99) | 0.62 | . | . | . |
| DPYD ---- tag | rs1520658 | A/A | 41 | 70,69 | 30 | 88,24 | 307 | 81,43 | 224 | 82,96 | 1.00 (.-.) | . | 1.10 (0.72-1.70) | 0.66 | 0.12 | 0.86 | 0.53 |
| DPYD ---- tag |  | A/G or G/G | 17 | 29,31 | 4 | 11,76 | 70 | 18,57 | 46 | 17,04 | 0.36 (0.09-1.56) | 0.17 | 1.13 (0.68-1.86) | 0.64 | . | . | . |
| DPYD ---- NA | rs17116806 | C/C | 36 | 62,07 | 19 | 55,88 | 249 | 66,05 | 172 | 63,70 | 1.00 (.-.) | . | 1.55 (0.86-2.78) | 0.15 | 0.29 | 0.83 | 0.51 |
| DPYD ---- NA |  | C/A or A/A | 22 | 37,93 | 15 | 44,12 | 128 | 33,95 | 98 | 36,30 | 1.63 (0.77-3.47) | 0.21 | 1.63 (0.89-3.00) | 0.12 | . | . | . |
| DPYD ---- tag | rs17431828 | G/G | 26 | 44,83 | 12 | 35,29 | 147 | 38,99 | 122 | 45,19 | 1.00 (.-.) | . | 1.52 (0.78-2.97) | 0.22 | 0.42 | 0.99 | 0.95 |
| DPYD ---- tag |  | G/C or C/C | 32 | 55,17 | 22 | 64,71 | 230 | 61,01 | 148 | 54,81 | 1.35 (0.61-2.97) | 0.46 | 1.47 (0.75-2.87) | 0.26 | . | . | . |
| DPYD ---- tag | rs17471640 | T/T | 23 | 39,66 | 12 | 35,29 | 160 | 42,44 | 133 | 49,26 | 1.00 (.-.) | . | 1.69 (0.86-3.30) | 0.13 | 0.20 | 0.85 | 0.51 |
| DPYD ---- tag |  | T/C or C/C | 35 | 60,34 | 22 | 64,71 | 217 | 57,56 | 137 | 50,74 | 1.40 (0.63-3.08) | 0.41 | 1.39 (0.71-2.71) | 0.34 | . | . | . |
| DPYD ---- tag | rs17702702 | G/G | 34 | 58,62 | 25 | 73,53 | 259 | 68,70 | 193 | 71,48 | 1.00 (.-.) | . | 1.34 (0.84-2.16) | 0.22 | 0.61 | 0.99 | 0.81 |
| DPYD ---- tag |  | G/C or C/C | 24 | 41,38 | 9 | 26,47 | 118 | 31,30 | 77 | 28,52 | 1.06 (0.44-2.54) | 0.90 | 1.12 (0.68-1.85) | 0.66 | . | . | . |
| DPYD ---- NA | rs1801265 | T/T | 34 | 58,62 | 22 | 64,71 | 211 | 55,97 | 163 | 60,37 | 1.00 (.-.) | . | 1.42 (0.85-2.39) | 0.18 | 0.40 | 0.99 | 0.95 |
| DPYD ---- NA |  | T/C or C/C | 24 | 41,38 | 12 | 35,29 | 166 | 44,03 | 107 | 39,63 | 1.23 (0.57-2.67) | 0.60 | 1.23 (0.72-2.08) | 0.45 | . | . | . |
| DPYD ---- tag | rs2039447 | T/T | 23 | 39,66 | 22 | 64,71 | 176 | 46,68 | 134 | 49,63 | 1.00 (.-.) | . | 1.17 (0.71-1.92) | 0.54 | 0.56 | 0.88 | 0.56 |
| DPYD ---- tag |  | T/C or C/C | 35 | 60,34 | 12 | 35,29 | 201 | 53,32 | 136 | 50,37 | 0.66 (0.29-1.49) | 0.32 | 0.99 (0.60-1.63) | 0.97 | . | . | . |
| DPYD ---- tag | rs2151567 | G/G | 50 | 86,21 | 33 | 97,06 | 343 | 90,98 | 243 | 90,00 | 1.00 (.-.) | . | 1.22 (0.80-1.86) | 0.35 | 0.77 | 0.85 | 0.51 |
| DPYD ---- tag |  | G/A or A/A | 8 | 13,79 | 1 | 2,94 | 34 | 9,02 | 27 | 10,00 | 0.83 (0.11-6.16) | 0.85 | 1.36 (0.76-2.43) | 0.31 | . | . | . |
| DPYD ---- tag | rs2152878 | A/A | 30 | 51,72 | 21 | 61,76 | 214 | 56,76 | 156 | 57,78 | 1.00 (.-.) | . | 1.05 (0.62-1.77) | 0.86 | 0.31 | 0.99 | 0.94 |
| DPYD ---- tag |  | A/G or G/G | 28 | 48,28 | 13 | 38,24 | 163 | 43,24 | 114 | 42,22 | 0.78 (0.36-1.69) | 0.52 | 1.25 (0.73-2.14) | 0.42 | . | . | . |
| DPYD ---- tag | rs2786505 | G/G | 45 | 77,59 | 28 | 82,35 | 285 | 75,60 | 195 | 72,22 | 1.00 (.-.) | . | 1.14 (0.71-1.81) | 0.59 | 0.47 | 0.82 | 0.51 |
| DPYD ---- tag |  | G/T or T/T | 13 | 22,41 | 6 | 17,65 | 92 | 24,40 | 75 | 27,78 | 0.88 (0.35-2.21) | 0.79 | 1.42 (0.86-2.34) | 0.17 | . | . | . |
| DPYD ---- tag | rs2786512 | G/G | 16 | 27,59 | 17 | 50,00 | 151 | 40,05 | 89 | 32,96 | 1.00 (.-.) | . | 0.66 (0.38-1.15) | 0.14 | 0.01 | 0.99 | 0.94 |
| DPYD ---- tag |  | G/A or A/A | 42 | 72,41 | 17 | 50,00 | 226 | 59,95 | 181 | 67,04 | 0.39 (0.18-0.84) | 0.02 | 0.81 (0.48-1.38) | 0.44 | . | . | . |
| DPYD ---- tag | rs2786519 | A/A | 38 | 65,52 | 18 | 52,94 | 235 | 62,33 | 154 | 57,04 | 1.00 (.-.) | . | 1.71 (0.95-3.08) | 0.08 | 0.08 | 0.83 | 0.51 |
| DPYD ---- tag |  | A/G or G/G | 20 | 34,48 | 16 | 47,06 | 142 | 37,67 | 116 | 42,96 | 2.22 (1.04-4.77) | 0.04 | 1.87 (1.02-3.41) | 0.04 | . | . | . |
| DPYD ---- tag | rs2811170 | A/A | 44 | 75,86 | 24 | 70,59 | 272 | 72,15 | 212 | 78,52 | 1.00 (.-.) | . | 1.22 (0.76-1.97) | 0.41 | 0.96 | 0.59 | 0.29 |
| DPYD ---- tag |  | A/T or T/T | 14 | 24,14 | 10 | 29,41 | 105 | 27,85 | 58 | 21,48 | 0.75 (0.32-1.74) | 0.50 | 0.89 (0.52-1.53) | 0.68 | . | . | . |
| DPYD ---- tag | rs2811199 | G/G | 44 | 75,86 | 27 | 79,41 | 277 | 73,47 | 189 | 70,00 | 1.00 (.-.) | . | 1.18 (0.74-1.90) | 0.49 | 0.74 | 0.83 | 0.51 |
| DPYD ---- tag |  | G/A or A/A | 14 | 24,14 | 7 | 20,59 | 100 | 26,53 | 81 | 30,00 | 1.06 (0.45-2.53) | 0.89 | 1.47 (0.89-2.43) | 0.13 | . | . | . |
| DPYD ---- tag | rs2811219 | T/T | 33 | 56,90 | 21 | 61,76 | 203 | 53,85 | 158 | 58,52 | 1.00 (.-.) | . | 1.23 (0.73-2.07) | 0.44 | 0.94 | 0.92 | 0.61 |
| DPYD ---- tag |  | T/C or C/C | 25 | 43,10 | 13 | 38,24 | 174 | 46,15 | 112 | 41,48 | 0.83 (0.38-1.81) | 0.64 | 1.06 (0.62-1.80) | 0.84 | . | . | . |
| DPYD ---- tag | rs4300257 | A/A | 39 | 67,24 | 21 | 61,76 | 244 | 64,72 | 172 | 63,70 | 1.00 (.-.) | . | 1.37 (0.83-2.28) | 0.22 | 0.54 | 0.99 | 0.93 |
| DPYD ---- tag |  | A/C or C/C | 19 | 32,76 | 13 | 38,24 | 133 | 35,28 | 98 | 36,30 | 1.08 (0.49-2.40) | 0.85 | 1.14 (0.67-1.93) | 0.62 | . | . | . |
| DPYD ---- tag | rs4379706 | T/T | 34 | 58,62 | 22 | 64,71 | 204 | 54,11 | 164 | 60,74 | 1.00 (.-.) | . | 1.44 (0.86-2.41) | 0.17 | 0.36 | 0.99 | 0.97 |
| DPYD ---- tag |  | T/C or C/C | 24 | 41,38 | 12 | 35,29 | 173 | 45,89 | 106 | 39,26 | 1.24 (0.57-2.69) | 0.59 | 1.20 (0.71-2.05) | 0.49 | . | . | . |
| DPYD ---- tag | rs4950021 | T/T | 21 | 36,21 | 12 | 35,29 | 116 | 30,77 | 80 | 29,63 | 1.00 (.-.) | . | 1.27 (0.66-2.46) | 0.47 | 0.87 | 0.93 | 0.66 |
| DPYD ---- tag |  | T/G or G/G | 37 | 63,79 | 22 | 64,71 | 261 | 69,23 | 190 | 70,37 | 1.20 (0.56-2.60) | 0.64 | 1.43 (0.76-2.68) | 0.27 | . | . | . |
| DPYD ---- tag | rs4950033 | T/T | 19 | 32,76 | 13 | 38,24 | 99 | 26,26 | 83 | 30,74 | 1.00 (.-.) | . | 1.08 (0.57-2.04) | 0.82 | 0.53 | 0.99 | 0.89 |
| DPYD ---- tag |  | T/C or C/C | 39 | 67,24 | 21 | 61,76 | 278 | 73,74 | 187 | 69,26 | 0.68 (0.32-1.47) | 0.33 | 0.95 (0.52-1.74) | 0.88 | . | . | . |
| DPYD ---- tag | rs495257 | T/T | 19 | 32,76 | 21 | 61,76 | 129 | 34,22 | 84 | 31,11 | 1.00 (.-.) | . | 0.76 (0.45-1.29) | 0.31 | 0.02 | 0.85 | 0.51 |
| DPYD ---- tag |  | T/C or C/C | 39 | 67,24 | 13 | 38,24 | 248 | 65,78 | 186 | 68,89 | 0.44 (0.20-0.98) | 0.04 | 0.93 (0.56-1.54) | 0.78 | . | . | . |
| DPYD ---- tag | rs552926 | A/A | 26 | 44,83 | 15 | 44,12 | 133 | 35,28 | 85 | 31,48 | 1.00 (.-.) | . | 0.92 (0.50-1.70) | 0.79 | 0.24 | 0.59 | 0.29 |
| DPYD ---- tag |  | A/G or G/G | 32 | 55,17 | 19 | 55,88 | 244 | 64,72 | 185 | 68,52 | 0.80 (0.37-1.70) | 0.56 | 1.19 (0.66-2.16) | 0.56 | . | . | . |
| DPYD ---- tag | rs628959 | A/A | 24 | 41,38 | 16 | 47,06 | 174 | 46,15 | 145 | 53,70 | 1.00 (.-.) | . | 1.07 (0.58-1.98) | 0.83 | 0.63 | 0.93 | 0.62 |
| DPYD ---- tag |  | A/G or G/G | 34 | 58,62 | 18 | 52,94 | 203 | 53,85 | 125 | 46,30 | 0.71 (0.33-1.53) | 0.38 | 0.93 (0.50-1.73) | 0.82 | . | . | . |
| DPYD ---- tag | rs6656660 | G/G | 43 | 74,14 | 30 | 88,24 | 297 | 78,78 | 199 | 73,70 | 1.00 (.-.) | . | 1.08 (0.69-1.68) | 0.74 | 0.15 | 0.99 | 0.89 |
| DPYD ---- tag |  | G/T or T/T | 15 | 25,86 | 4 | 11,76 | 80 | 21,22 | 71 | 26,30 | 0.53 (0.16-1.78) | 0.30 | 1.31 (0.80-2.14) | 0.28 | . | . | . |
| DPYD ---- tag | rs6663670 | A/A | 43 | 74,14 | 26 | 76,47 | 274 | 72,68 | 191 | 70,74 | 1.00 (.-.) | . | 1.20 (0.74-1.92) | 0.46 | 0.79 | 0.86 | 0.53 |
| DPYD ---- tag |  | A/C or C/C | 15 | 25,86 | 8 | 23,53 | 103 | 27,32 | 79 | 29,26 | 1.05 (0.44-2.49) | 0.92 | 1.42 (0.85-2.35) | 0.18 | . | . | . |
| DPYD ---- tag | rs6683883 | T/T | 32 | 55,17 | 8 | 23,53 | 147 | 38,99 | 87 | 32,22 | 1.00 (.-.) | . | 2.02 (0.87-4.68) | 0.10 | 0.14 | 0.85 | 0.51 |
| DPYD ---- tag |  | T/C or C/C | 26 | 44,83 | 26 | 76,47 | 230 | 61,01 | 183 | 67,78 | 2.49 (1.00-6.24) | 0.05 | 2.51 (1.10-5.74) | 0.03 | . | . | . |
| DPYD ---- tag | rs6686861 | C/C | 51 | 87,93 | 31 | 91,18 | 333 | 88,33 | 225 | 83,33 | 1.00 (.-.) | . | 1.01 (0.66-1.55) | 0.95 | 0.02 | 0.88 | 0.55 |
| DPYD ---- tag |  | C/T or T/T | 7 | 12,07 | 3 | 8,82 | 44 | 11,67 | 45 | 16,67 | 0.25 (0.06-1.06) | 0.06 | 1.17 (0.70-1.97) | 0.55 | . | . | . |
| DPYD ---- tag | rs7414210 | A/A | 41 | 70,69 | 28 | 82,35 | 265 | 70,29 | 201 | 74,44 | 1.00 (.-.) | . | 1.42 (0.90-2.22) | 0.13 | 0.09 | 0.99 | 0.90 |
| DPYD ---- tag |  | A/C or C/C | 17 | 29,31 | 6 | 17,65 | 112 | 29,71 | 69 | 25,56 | 2.65 (0.87-8.11) | 0.09 | 1.25 (0.77-2.04) | 0.37 | . | . | . |
| DPYD ---- tag | rs7530858 | A/A | 46 | 79,31 | 30 | 88,24 | 299 | 79,31 | 202 | 74,81 | 1.00 (.-.) | . | 1.18 (0.75-1.84) | 0.47 | 0.65 | 0.85 | 0.51 |
| DPYD ---- tag |  | A/G or G/G | 12 | 20,69 | 4 | 11,76 | 78 | 20,69 | 68 | 25,19 | 0.91 (0.31-2.66) | 0.86 | 1.37 (0.84-2.24) | 0.20 | . | . | . |
| DPYD ---- tag | rs7544128 | C/C | 30 | 51,72 | 18 | 52,94 | 207 | 54,91 | 152 | 56,30 | 1.00 (.-.) | . | 1.45 (0.84-2.48) | 0.18 | 0.40 | 0.99 | 0.94 |
| DPYD ---- tag |  | C/G or G/G | 28 | 48,28 | 16 | 47,06 | 170 | 45,09 | 118 | 43,70 | 1.22 (0.56-2.64) | 0.61 | 1.24 (0.72-2.14) | 0.44 | . | . | . |
| DPYD ---- tag | rs7545340 | G/G | 33 | 56,90 | 16 | 47,06 | 215 | 57,03 | 151 | 55,93 | 1.00 (.-.) | . | 1.56 (0.87-2.82) | 0.14 | 0.23 | 0.85 | 0.52 |
| DPYD ---- tag |  | G/A or A/A | 25 | 43,10 | 18 | 52,94 | 162 | 42,97 | 119 | 44,07 | 1.79 (0.84-3.79) | 0.13 | 1.72 (0.95-3.13) | 0.07 | . | . | . |
| DPYD ---- tag | rs828054 | C/C | 8 | 13,79 | 5 | 14,71 | 84 | 22,28 | 84 | 31,11 | 1.00 (.-.) | . | 1.45 (0.51-4.14) | 0.49 | 0.68 | 0.97 | 0.78 |
| DPYD ---- tag |  | C/A or A/A | 50 | 86,21 | 29 | 85,29 | 293 | 77,72 | 186 | 68,89 | 0.86 (0.29-2.57) | 0.79 | 0.99 (0.35-2.79) | 0.99 | . | . | . |
| DPYD ---- tag | rs885622 | G/G | 36 | 62,07 | 9 | 26,47 | 153 | 40,58 | 87 | 32,22 | 1.00 (.-.) | . | 1.96 (0.93-4.12) | 0.08 | 0.06 | 0.66 | 0.51 |
| DPYD ---- tag |  | G/A or A/A | 22 | 37,93 | 25 | 73,53 | 224 | 59,42 | 183 | 67,78 | 3.09 (1.34-7.11) | 0.01 | 2.67 (1.29-5.50) | 0.01 | . | . | . |
| DPYD ---- tag | rs9437663 | G/G | 39 | 67,24 | 24 | 70,59 | 244 | 64,72 | 168 | 62,22 | 1.00 (.-.) | . | 1.11 (0.69-1.78) | 0.68 | 0.41 | 0.99 | 0.97 |
| DPYD ---- tag |  | G/A or A/A | 19 | 32,76 | 10 | 29,41 | 133 | 35,28 | 102 | 37,78 | 0.81 (0.32-2.04) | 0.66 | 1.34 (0.81-2.19) | 0.25 | . | . | . |
| DPYS ---- tag | rs13249169 | A/A | 45 | 77,59 | 30 | 88,24 | 296 | 78,51 | 210 | 77,78 | 1.00 (.-.) | . | 1.08 (0.70-1.68) | 0.72 | 0.17 | 0.93 | 0.61 |
| DPYS ---- tag |  | A/T or T/T | 13 | 22,41 | 4 | 11,76 | 81 | 21,49 | 60 | 22,22 | 0.50 (0.17-1.46) | 0.21 | 1.14 (0.69-1.88) | 0.62 | . | . | . |
| DPYS ---- NA | rs13263121 | T/T | 26 | 44,83 | 15 | 44,12 | 169 | 44,83 | 123 | 45,56 | 1.00 (.-.) | . | 1.25 (0.67-2.33) | 0.48 | 0.96 | 0.59 | 0.19 |
| DPYS ---- NA |  | T/A or A/A | 32 | 55,17 | 19 | 55,88 | 208 | 55,17 | 147 | 54,44 | 0.87 (0.41-1.85) | 0.71 | 1.06 (0.58-1.96) | 0.85 | . | . | . |
| DPYS ---- tag | rs16871361 | T/T | 53 | 91,38 | 31 | 91,18 | 339 | 89,92 | 231 | 85,56 | 1.00 (.-.) | . | 1.21 (0.78-1.87) | 0.39 | 0.75 | 0.99 | 0.82 |
| DPYS ---- tag |  | T/C or C/C | 5 | 8,62 | 3 | 8,82 | 38 | 10,08 | 39 | 14,44 | 0.89 (0.26-3.01) | 0.85 | 1.31 (0.77-2.24) | 0.32 | . | . | . |
| DPYS ---- NA | rs17245950 | T/T | 47 | 81,03 | 27 | 79,41 | 283 | 75,07 | 213 | 78,89 | 1.00 (.-.) | . | 1.29 (0.82-2.03) | 0.26 | 0.70 | 0.93 | 0.61 |
| DPYS ---- NA |  | T/A or A/A | 11 | 18,97 | 7 | 20,59 | 94 | 24,93 | 57 | 21,11 | 1.05 (0.39-2.81) | 0.93 | 1.10 (0.65-1.84) | 0.73 | . | . | . |
| DPYS ---- NA | rs2253336 | A/A | 49 | 84,48 | 29 | 85,29 | 296 | 78,51 | 218 | 80,74 | 1.00 (.-.) | . | 1.22 (0.79-1.89) | 0.36 | 0.69 | 0.99 | 0.67 |
| DPYS ---- NA |  | A/G or G/G | 9 | 15,52 | 5 | 14,71 | 81 | 21,49 | 52 | 19,26 | 0.68 (0.16-2.94) | 0.60 | 1.11 (0.67-1.83) | 0.69 | . | . | . |
| DPYS ---- tag | rs2280010 | C/C | 33 | 56,90 | 14 | 41,18 | 233 | 61,80 | 142 | 52,59 | 1.00 (.-.) | . | 1.15 (0.62-2.14) | 0.66 | 0.62 | 0.59 | 0.19 |
| DPYS ---- tag |  | C/T or T/T | 25 | 43,10 | 20 | 58,82 | 144 | 38,20 | 128 | 47,41 | 1.28 (0.59-2.74) | 0.53 | 1.80 (0.97-3.34) | 0.06 | . | . | . |
| DPYS ---- tag | rs2333874 | T/T | 21 | 36,21 | 16 | 47,06 | 159 | 42,18 | 116 | 42,96 | 1.00 (.-.) | . | 1.31 (0.71-2.44) | 0.39 | 0.81 | 0.99 | 0.80 |
| DPYS ---- tag |  | T/G or G/G | 37 | 63,79 | 18 | 52,94 | 218 | 57,82 | 154 | 57,04 | 0.99 (0.46-2.12) | 0.98 | 1.18 (0.64-2.18) | 0.60 | . | . | . |
| DPYS ---- NA | rs2669429 | C/C | 15 | 25,86 | 8 | 23,53 | 130 | 34,48 | 70 | 25,93 | 1.00 (.-.) | . | 0.66 (0.30-1.48) | 0.32 | 0.09 | 0.85 | 0.40 |
| DPYS ---- NA |  | C/T or T/T | 43 | 74,14 | 26 | 76,47 | 247 | 65,52 | 200 | 74,07 | 0.70 (0.29-1.66) | 0.41 | 1.07 (0.49-2.33) | 0.86 | . | . | . |
| DPYS ---- tag | rs2669434 | C/C | 32 | 55,17 | 21 | 61,76 | 179 | 47,48 | 149 | 55,19 | 1.00 (.-.) | . | 1.17 (0.70-1.93) | 0.55 | 0.65 | 0.59 | 0.19 |
| DPYS ---- tag |  | C/A or A/A | 26 | 44,83 | 13 | 38,24 | 198 | 52,52 | 121 | 44,81 | 0.68 (0.31-1.49) | 0.33 | 0.95 (0.57-1.60) | 0.86 | . | . | . |
| DPYS ---- tag | rs2853142 | T/T | 30 | 51,72 | 12 | 35,29 | 137 | 36,34 | 115 | 42,59 | 1.00 (.-.) | . | 1.93 (1.02-3.65) | 0.04 | 0.06 | 0.59 | 0.19 |
| DPYS ---- tag |  | T/C or C/C | 28 | 48,28 | 22 | 64,71 | 240 | 63,66 | 155 | 57,41 | 1.41 (0.65-3.06) | 0.39 | 1.26 (0.67-2.36) | 0.48 | . | . | . |
| DPYS ---- NA | rs2853145 | A/A | 47 | 81,03 | 21 | 61,76 | 244 | 64,72 | 187 | 69,26 | 1.00 (.-.) | . | 1.60 (0.95-2.68) | 0.08 | 0.09 | 0.89 | 0.54 |
| DPYS ---- NA |  | A/C or C/C | 11 | 18,97 | 13 | 38,24 | 133 | 35,28 | 83 | 30,74 | 1.65 (0.74-3.68) | 0.22 | 1.24 (0.72-2.13) | 0.45 | . | . | . |
| DPYS ---- tag | rs2853149 | G/G | 12 | 20,69 | 10 | 29,41 | 102 | 27,06 | 72 | 26,67 | 1.00 (.-.) | . | 1.03 (0.46-2.32) | 0.94 | 0.61 | 0.97 | 0.67 |
| DPYS ---- tag |  | G/A or A/A | 46 | 79,31 | 24 | 70,59 | 275 | 72,94 | 198 | 73,33 | 0.86 (0.36-2.04) | 0.73 | 1.13 (0.52-2.47) | 0.76 | . | . | . |
| DPYS ---- tag | rs2853154 | T/T | 38 | 65,52 | 22 | 64,71 | 212 | 56,23 | 153 | 56,67 | 1.00 (.-.) | . | 1.44 (0.87-2.39) | 0.15 | 0.41 | 0.59 | 0.19 |
| DPYS ---- tag |  | T/C or C/C | 20 | 34,48 | 12 | 35,29 | 165 | 43,77 | 117 | 43,33 | 1.14 (0.51-2.56) | 0.75 | 1.14 (0.68-1.89) | 0.62 | . | . | . |
| DPYS ---- tag | rs2853161 | A/A | 17 | 29,31 | 6 | 17,65 | 106 | 28,12 | 67 | 24,81 | 1.00 (.-.) | . | 1.43 (0.59-3.45) | 0.43 | 0.71 | 0.93 | 0.61 |
| DPYS ---- tag |  | A/G or G/G | 41 | 70,69 | 28 | 82,35 | 271 | 71,88 | 203 | 75,19 | 1.23 (0.49-3.10) | 0.67 | 1.46 (0.63-3.41) | 0.38 | . | . | . |
| DPYS ---- NA | rs2959024 | T/T | 33 | 56,90 | 14 | 41,18 | 199 | 52,79 | 125 | 46,30 | 1.00 (.-.) | . | 1.00 (0.54-1.84) | 0.99 | 0.32 | 0.98 | 0.67 |
| DPYS ---- NA |  | T/G or G/G | 25 | 43,10 | 20 | 58,82 | 178 | 47,21 | 145 | 53,70 | 0.93 (0.43-1.99) | 0.85 | 1.40 (0.77-2.56) | 0.28 | . | . | . |
| DPYS ---- NA | rs2959025 | A/A | 25 | 43,10 | 13 | 38,24 | 162 | 42,97 | 105 | 38,89 | 1.00 (.-.) | . | 1.18 (0.62-2.23) | 0.61 | 0.85 | 0.93 | 0.61 |
| DPYS ---- NA |  | A/G or G/G | 33 | 56,90 | 21 | 61,76 | 215 | 57,03 | 165 | 61,11 | 1.10 (0.51-2.38) | 0.81 | 1.40 (0.75-2.61) | 0.29 | . | . | . |
| DPYS ---- tag | rs2959026 | G/G | 17 | 29,31 | 13 | 38,24 | 132 | 35,01 | 96 | 35,56 | 1.00 (.-.) | . | 0.99 (0.50-1.96) | 0.99 | 0.44 | 0.85 | 0.40 |
| DPYS ---- tag |  | G/A or A/A | 41 | 70,69 | 21 | 61,76 | 245 | 64,99 | 174 | 64,44 | 0.76 (0.35-1.66) | 0.49 | 1.05 (0.54-2.04) | 0.88 | . | . | . |
| DPYS ---- NA | rs3133278 | T/T | 32 | 55,17 | 18 | 52,94 | 191 | 50,66 | 138 | 51,11 | 1.00 (.-.) | . | 1.40 (0.79-2.49) | 0.25 | 0.57 | 0.83 | 0.37 |
| DPYS ---- NA |  | T/C or C/C | 26 | 44,83 | 16 | 47,06 | 186 | 49,34 | 132 | 48,89 | 1.09 (0.52-2.31) | 0.82 | 1.21 (0.68-2.14) | 0.51 | . | . | . |
| DPYS ---- tag | rs3750187 | G/G | 42 | 72,41 | 20 | 58,82 | 236 | 62,60 | 169 | 62,59 | 1.00 (.-.) | . | 1.63 (0.95-2.80) | 0.08 | 0.10 | 0.66 | 0.24 |
| DPYS ---- tag |  | G/A or A/A | 16 | 27,59 | 14 | 41,18 | 141 | 37,40 | 101 | 37,41 | 1.73 (0.80-3.73) | 0.16 | 1.40 (0.80-2.44) | 0.23 | . | . | . |
| DPYS ---- tag | rs3793357 | T/T | 52 | 89,66 | 30 | 88,24 | 332 | 88,06 | 244 | 90,37 | 1.00 (.-.) | . | 1.12 (0.73-1.74) | 0.60 | 0.24 | 0.99 | 0.80 |
| DPYS ---- tag |  | T/G or G/G | 6 | 10,34 | 4 | 11,76 | 45 | 11,94 | 26 | 9,63 | 0.58 (0.19-1.75) | 0.34 | 1.30 (0.71-2.37) | 0.40 | . | . | . |
| DPYS ---- tag | rs3793358 | G/G | 50 | 86,21 | 28 | 82,35 | 287 | 76,13 | 216 | 80,00 | 1.00 (.-.) | . | 1.39 (0.89-2.19) | 0.15 | 0.40 | 0.66 | 0.22 |
| DPYS ---- tag |  | G/A or A/A | 8 | 13,79 | 6 | 17,65 | 90 | 23,87 | 54 | 20,00 | 1.16 (0.39-3.41) | 0.79 | 0.96 (0.58-1.61) | 0.89 | . | . | . |
| DPYS ---- tag | rs6468924 | C/C | 37 | 63,79 | 24 | 70,59 | 221 | 58,62 | 175 | 64,81 | 1.00 (.-.) | . | 1.16 (0.72-1.88) | 0.54 | 0.61 | 0.83 | 0.37 |
| DPYS ---- tag |  | C/T or T/T | 21 | 36,21 | 10 | 29,41 | 156 | 41,38 | 95 | 35,19 | 0.70 (0.30-1.63) | 0.41 | 1.02 (0.62-1.70) | 0.92 | . | . | . |
| DUT ---- tag | rs8025164 | G/G | 45 | 77,59 | 21 | 61,76 | 251 | 66,58 | 193 | 71,48 | 1.00 (.-.) | . | 1.52 (0.93-2.49) | 0.10 | 0.10 | 0.99 | 0.89 |
| DUT ---- tag |  | G/A or A/A | 13 | 22,41 | 13 | 38,24 | 126 | 33,42 | 77 | 28,52 | 1.72 (0.76-3.86) | 0.19 | 1.23 (0.72-2.10) | 0.44 | . | . | . |
| EHMT1 ---- tag | rs10780190 | C/C | 54 | 93,10 | 30 | 88,24 | 335 | 88,86 | 243 | 90,00 | 1.00 (.-.) | . | 1.35 (0.87-2.07) | 0.18 | 0.12 | 0.99 | 0.97 |
| EHMT1 ---- tag |  | C/T or T/T | 4 | 6,90 | 4 | 11,76 | 42 | 11,14 | 27 | 10,00 | 2.75 (0.77-9.83) | 0.12 | 1.14 (0.63-2.08) | 0.66 | . | . | . |
| EHMT1 ---- tag | rs10867083 | G/G | 23 | 39,66 | 17 | 50,00 | 181 | 48,01 | 123 | 45,56 | 1.00 (.-.) | . | 1.02 (0.55-1.86) | 0.96 | 0.40 | 0.99 | 0.97 |
| EHMT1 ---- tag |  | G/A or A/A | 35 | 60,34 | 17 | 50,00 | 196 | 51,99 | 147 | 54,44 | 0.68 (0.31-1.48) | 0.33 | 0.99 (0.55-1.78) | 0.96 | . | . | . |
| EHMT1 ---- tag | rs11137190 | C/C | 32 | 55,17 | 17 | 50,00 | 197 | 52,25 | 137 | 50,74 | 1.00 (.-.) | . | 1.20 (0.70-2.06) | 0.51 | 0.97 | 0.85 | 0.81 |
| EHMT1 ---- tag |  | C/G or G/G | 26 | 44,83 | 17 | 50,00 | 180 | 47,75 | 133 | 49,26 | 1.21 (0.56-2.62) | 0.63 | 1.48 (0.86-2.52) | 0.15 | . | . | . |
| EHMT1 ---- tag | rs3123510 | G/G | 23 | 39,66 | 12 | 35,29 | 148 | 39,26 | 100 | 37,04 | 1.00 (.-.) | . | 1.34 (0.72-2.49) | 0.36 | 0.68 | 0.91 | 0.90 |
| EHMT1 ---- tag |  | G/A or A/A | 35 | 60,34 | 22 | 64,71 | 229 | 60,74 | 170 | 62,96 | 1.35 (0.62-2.92) | 0.45 | 1.52 (0.83-2.77) | 0.18 | . | . | . |
| EHMT1 ---- candidate literature | rs3125795 | G/G | 53 | 91,38 | 30 | 88,24 | 336 | 89,12 | 242 | 89,63 | 1.00 (.-.) | . | 1.34 (0.87-2.06) | 0.19 | 0.14 | 0.94 | 0.97 |
| EHMT1 ---- candidate literature |  | G/T or T/T | 5 | 8,62 | 4 | 11,76 | 41 | 10,88 | 28 | 10,37 | 2.76 (0.77-9.85) | 0.12 | 1.20 (0.67-2.17) | 0.54 | . | . | . |
| EHMT1 ---- tag | rs4573359 | G/G | 49 | 84,48 | 29 | 85,29 | 317 | 84,08 | 228 | 84,44 | 1.00 (.-.) | . | 1.23 (0.79-1.91) | 0.35 | 0.98 | 0.99 | 0.97 |
| EHMT1 ---- tag |  | G/T or T/T | 9 | 15,52 | 5 | 14,71 | 60 | 15,92 | 42 | 15,56 | 1.05 (0.31-3.55) | 0.93 | 1.32 (0.78-2.21) | 0.30 | . | . | . |
| EHMT1 ---- candidate literature | rs4634736 | G/G | 49 | 84,48 | 29 | 85,29 | 316 | 83,82 | 229 | 84,81 | 1.00 (.-.) | . | 1.24 (0.80-1.92) | 0.34 | 0.98 | 0.99 | 0.97 |
| EHMT1 ---- candidate literature |  | G/A or A/A | 9 | 15,52 | 5 | 14,71 | 61 | 16,18 | 41 | 15,19 | 1.05 (0.31-3.55) | 0.94 | 1.28 (0.76-2.16) | 0.35 | . | . | . |
| EHMT1 ---- tag | rs4876902 | C/C | 43 | 74,14 | 18 | 52,94 | 239 | 63,40 | 166 | 61,48 | 1.00 (.-.) | . | 1.35 (0.80-2.31) | 0.26 | 0.58 | 0.81 | 0.58 |
| EHMT1 ---- tag |  | C/T or T/T | 15 | 25,86 | 16 | 47,06 | 138 | 36,60 | 104 | 38,52 | 1.52 (0.71-3.26) | 0.28 | 1.63 (0.95-2.82) | 0.08 | . | . | . |
| EHMT1 ---- tag | rs4876904 | T/T | 19 | 32,76 | 12 | 35,29 | 108 | 28,65 | 81 | 30,00 | 1.00 (.-.) | . | 1.12 (0.55-2.29) | 0.76 | 0.73 | 0.99 | 0.97 |
| EHMT1 ---- tag |  | T/G or G/G | 39 | 67,24 | 22 | 64,71 | 269 | 71,35 | 189 | 70,00 | 0.89 (0.40-2.00) | 0.78 | 1.16 (0.58-2.31) | 0.68 | . | . | . |
| EHMT1 ---- tag | rs7390244 | A/A | 15 | 25,86 | 8 | 23,53 | 88 | 23,34 | 71 | 26,30 | 1.00 (.-.) | . | 1.33 (0.56-3.15) | 0.52 | 0.87 | 0.99 | 0.97 |
| EHMT1 ---- tag |  | A/G or G/G | 43 | 74,14 | 26 | 76,47 | 289 | 76,66 | 199 | 73,70 | 1.18 (0.47-2.99) | 0.72 | 1.45 (0.63-3.34) | 0.39 | . | . | . |
| EHMT1 ---- tag | rs9314635 | G/G | 29 | 50,00 | 13 | 38,24 | 159 | 42,18 | 105 | 38,89 | 1.00 (.-.) | . | 1.34 (0.74-2.43) | 0.34 | 0.59 | 0.68 | 0.58 |
| EHMT1 ---- tag |  | G/T or T/T | 29 | 50,00 | 21 | 61,76 | 218 | 57,82 | 165 | 61,11 | 1.48 (0.69-3.15) | 0.32 | 1.58 (0.89-2.82) | 0.12 | . | . | . |
| EHMT2 ---- candidate/tag | rs2736428 | G/G | 20 | 34,48 | 13 | 38,24 | 146 | 38,73 | 120 | 44,44 | 1.00 (.-.) | . | 1.14 (0.58-2.22) | 0.70 | 0.80 | 0.89 | 0.31 |
| EHMT2 ---- candidate/tag |  | G/A or A/A | 38 | 65,52 | 21 | 61,76 | 231 | 61,27 | 150 | 55,56 | 0.76 (0.35-1.68) | 0.50 | 0.96 (0.50-1.87) | 0.92 | . | . | . |
| EHMT2 ---- tag | rs9267649 | G/G | 47 | 81,03 | 24 | 70,59 | 270 | 71,62 | 195 | 72,22 | 1.00 (.-.) | . | 1.38 (0.85-2.24) | 0.19 | 0.35 | 0.85 | 0.31 |
| EHMT2 ---- tag |  | G/A or A/A | 11 | 18,97 | 10 | 29,41 | 107 | 28,38 | 75 | 27,78 | 1.52 (0.66-3.52) | 0.32 | 1.37 (0.81-2.30) | 0.24 | . | . | . |
| FDXR ---- NA | rs2070918 | T/T | 26 | 44,83 | 11 | 32,35 | 195 | 51,72 | 127 | 47,04 | 1.00 (.-.) | . | 2.02 (0.97-4.19) | 0.06 | 0.07 | 0.85 | 0.68 |
| FDXR ---- NA |  | T/C or C/C | 32 | 55,17 | 23 | 67,65 | 182 | 48,28 | 143 | 52,96 | 2.52 (1.09-5.81) | 0.03 | 2.30 (1.11-4.75) | 0.03 | . | . | . |
| FDXR ---- tag | rs509911 | A/A | 34 | 58,62 | 20 | 58,82 | 242 | 64,19 | 172 | 63,70 | 1.00 (.-.) | . | 1.55 (0.91-2.65) | 0.11 | 0.12 | 0.95 | 0.68 |
| FDXR ---- tag |  | A/G or G/G | 24 | 41,38 | 14 | 41,18 | 135 | 35,81 | 98 | 36,30 | 2.01 (0.94-4.32) | 0.07 | 1.63 (0.94-2.83) | 0.08 | . | . | . |
| FDXR ---- NA | rs689882 | G/G | 27 | 46,55 | 16 | 47,06 | 205 | 54,38 | 142 | 52,59 | 1.00 (.-.) | . | 1.45 (0.80-2.62) | 0.22 | 0.44 | 0.99 | 0.68 |
| FDXR ---- NA |  | G/A or A/A | 31 | 53,45 | 18 | 52,94 | 172 | 45,62 | 128 | 47,41 | 1.35 (0.63-2.87) | 0.44 | 1.43 (0.79-2.60) | 0.24 | . | . | . |
| FDXR ---- NA | rs689895 | G/G | 31 | 53,45 | 17 | 50,00 | 192 | 50,93 | 131 | 48,52 | 1.00 (.-.) | . | 1.62 (0.91-2.88) | 0.10 | 0.14 | 0.99 | 0.68 |
| FDXR ---- NA |  | G/C or C/C | 27 | 46,55 | 17 | 50,00 | 185 | 49,07 | 139 | 51,48 | 1.78 (0.83-3.78) | 0.14 | 1.56 (0.89-2.76) | 0.12 | . | . | . |
| FOLH1 ---- candidate literature | rs10839236 | T/T | 22 | 37,93 | 14 | 41,18 | 165 | 43,77 | 101 | 37,41 | 1.00 (.-.) | . | 0.88 (0.44-1.75) | 0.71 | 0.24 | 0.93 | 0.79 |
| FOLH1 ---- candidate literature |  | T/C or C/C | 36 | 62,07 | 20 | 58,82 | 212 | 56,23 | 169 | 62,59 | 0.71 (0.32-1.57) | 0.40 | 1.04 (0.53-2.04) | 0.91 | . | . | . |
| FOLH1 ---- tag | rs16906190 | A/A | 44 | 75,86 | 30 | 88,24 | 308 | 81,70 | 225 | 83,33 | 1.00 (.-.) | . | 1.15 (0.73-1.81) | 0.54 | 0.45 | 0.99 | 0.79 |
| FOLH1 ---- tag |  | A/G or G/G | 14 | 24,14 | 4 | 11,76 | 69 | 18,30 | 45 | 16,67 | 0.70 (0.23-2.09) | 0.52 | 1.25 (0.73-2.12) | 0.42 | . | . | . |
| FOLH1 ---- candidate | rs202676 | T/T | 33 | 56,90 | 23 | 67,65 | 239 | 63,40 | 166 | 61,48 | 1.00 (.-.) | . | 1.10 (0.64-1.88) | 0.73 | 0.50 | 0.99 | 0.79 |
| FOLH1 ---- candidate |  | T/C or C/C | 25 | 43,10 | 11 | 32,35 | 138 | 36,60 | 104 | 38,52 | 0.83 (0.38-1.81) | 0.64 | 1.21 (0.70-2.10) | 0.49 | . | . | . |
| FOLH1 ---- tag | rs202680 | A/A | 29 | 50,00 | 22 | 64,71 | 215 | 57,03 | 149 | 55,19 | 1.00 (.-.) | . | 1.14 (0.66-1.97) | 0.65 | 0.64 | 0.99 | 0.79 |
| FOLH1 ---- tag |  | A/T or T/T | 29 | 50,00 | 12 | 35,29 | 162 | 42,97 | 121 | 44,81 | 0.87 (0.40-1.86) | 0.72 | 1.20 (0.69-2.09) | 0.53 | . | . | . |
| FOLH1 ---- candidate literature | rs202720 | G/G | 32 | 55,17 | 23 | 67,65 | 240 | 63,66 | 166 | 61,48 | 1.00 (.-.) | . | 1.06 (0.62-1.81) | 0.83 | 0.38 | 0.99 | 0.79 |
| FOLH1 ---- candidate literature |  | G/C or C/C | 26 | 44,83 | 11 | 32,35 | 137 | 36,34 | 104 | 38,52 | 0.77 (0.36-1.68) | 0.52 | 1.18 (0.68-2.04) | 0.55 | . | . | . |
| FOLH1 ---- tag | rs2299650 | G/G | 20 | 34,48 | 15 | 44,12 | 163 | 43,24 | 101 | 37,41 | 1.00 (.-.) | . | 0.78 (0.40-1.52) | 0.47 | 0.11 | 0.96 | 0.79 |
| FOLH1 ---- tag |  | G/T or T/T | 38 | 65,52 | 19 | 55,88 | 214 | 56,76 | 169 | 62,59 | 0.59 (0.27-1.28) | 0.18 | 0.92 (0.48-1.75) | 0.79 | . | . | . |
| FOLH1 ---- tag | rs617528 | G/G | 45 | 77,59 | 25 | 73,53 | 303 | 80,37 | 209 | 77,41 | 1.00 (.-.) | . | 1.14 (0.71-1.81) | 0.59 | 0.46 | 0.93 | 0.79 |
| FOLH1 ---- tag |  | G/A or A/A | 13 | 22,41 | 9 | 26,47 | 74 | 19,63 | 61 | 22,59 | 0.77 (0.32-1.85) | 0.56 | 1.24 (0.74-2.07) | 0.41 | . | . | . |
| FOLH1 ---- tag | rs663877 | T/T | 46 | 79,31 | 26 | 76,47 | 300 | 79,58 | 206 | 76,30 | 1.00 (.-.) | . | 1.16 (0.71-1.90) | 0.57 | 0.59 | 0.93 | 0.79 |
| FOLH1 ---- tag |  | T/G or G/G | 12 | 20,69 | 8 | 23,53 | 77 | 20,42 | 64 | 23,70 | 0.88 (0.38-2.06) | 0.77 | 1.30 (0.76-2.22) | 0.33 | . | . | . |
| FOLH1 ---- tag | rs670776 | A/A | 33 | 56,90 | 23 | 67,65 | 239 | 63,40 | 166 | 61,48 | 1.00 (.-.) | . | 1.10 (0.64-1.88) | 0.73 | 0.50 | 0.99 | 0.79 |
| FOLH1 ---- tag |  | A/T or T/T | 25 | 43,10 | 11 | 32,35 | 138 | 36,60 | 104 | 38,52 | 0.83 (0.38-1.81) | 0.64 | 1.21 (0.70-2.10) | 0.49 | . | . | . |
| FOLH1 ---- tag | rs7124497 | G/G | 54 | 93,10 | 33 | 97,06 | 349 | 92,57 | 250 | 92,59 | 1.00 (.-.) | . | 1.26 (0.83-1.92) | 0.27 | 0.68 | 0.99 | 0.80 |
| FOLH1 ---- tag |  | G/A or A/A | 4 | 6,90 | 1 | 2,94 | 28 | 7,43 | 20 | 7,41 | 1.35 (0.18-10.19) | 0.77 | 1.07 (0.56-2.06) | 0.84 | . | . | . |
| FOLR1 ---- tag | rs651646 | T/T | 18 | 31,03 | 9 | 26,47 | 129 | 34,22 | 86 | 31,85 | 1.00 (.-.) | . | 1.59 (0.71-3.52) | 0.26 | 0.46 | 0.99 | 0.68 |
| FOLR1 ---- tag |  | T/A or A/A | 40 | 68,97 | 25 | 73,53 | 248 | 65,78 | 184 | 68,15 | 1.45 (0.61-3.49) | 0.40 | 1.64 (0.75-3.58) | 0.21 | . | . | . |
| FPGS ---- tag | rs10987746 | T/T | 16 | 27,59 | 8 | 23,53 | 113 | 29,97 | 76 | 28,15 | 1.00 (.-.) | . | 1.42 (0.61-3.31) | 0.42 | 0.71 | 0.96 | 0.63 |
| FPGS ---- tag |  | T/C or C/C | 42 | 72,41 | 26 | 76,47 | 264 | 70,03 | 194 | 71,85 | 1.03 (0.41-2.55) | 0.96 | 1.21 (0.53-2.77) | 0.65 | . | . | . |
| FPGS ---- tag | rs7033913 | T/T | 21 | 36,21 | 12 | 35,29 | 121 | 32,10 | 86 | 31,85 | 1.00 (.-.) | . | 1.33 (0.66-2.68) | 0.42 | 0.82 | 0.99 | 0.63 |
| FPGS ---- tag |  | T/C or C/C | 37 | 63,79 | 22 | 64,71 | 256 | 67,90 | 184 | 68,15 | 1.06 (0.48-2.35) | 0.88 | 1.28 (0.66-2.49) | 0.47 | . | . | . |
| FPGS ---- tag | rs7039798 | G/G | 18 | 31,03 | 8 | 23,53 | 129 | 34,22 | 80 | 29,63 | 1.00 (.-.) | . | 1.32 (0.57-3.09) | 0.52 | 0.86 | 0.97 | 0.63 |
| FPGS ---- tag |  | G/A or A/A | 40 | 68,97 | 26 | 76,47 | 248 | 65,78 | 190 | 70,37 | 1.06 (0.43-2.64) | 0.90 | 1.29 (0.56-2.94) | 0.55 | . | . | . |
| GGH ---- tag | rs10957264 | G/G | 39 | 67,24 | 27 | 79,41 | 260 | 68,97 | 192 | 71,11 | 1.00 (.-.) | . | 1.16 (0.73-1.83) | 0.54 | 0.50 | 0.92 | 0.42 |
| GGH ---- tag |  | G/T or T/T | 19 | 32,76 | 7 | 20,59 | 117 | 31,03 | 78 | 28,89 | 0.66 (0.24-1.76) | 0.40 | 1.07 (0.65-1.76) | 0.79 | . | . | . |
| GGH ---- candidate literature | rs11545076 | T/T | 30 | 51,72 | 17 | 50,00 | 182 | 48,28 | 134 | 49,63 | 1.00 (.-.) | . | 1.33 (0.72-2.46) | 0.36 | 0.76 | 0.93 | 0.42 |
| GGH ---- candidate literature |  | T/G or G/G | 28 | 48,28 | 17 | 50,00 | 195 | 51,72 | 136 | 50,37 | 0.99 (0.46-2.14) | 0.98 | 1.16 (0.63-2.13) | 0.64 | . | . | . |
| GGH ---- candidate | rs11545077 | G/G | 32 | 55,17 | 19 | 55,88 | 199 | 52,79 | 144 | 53,33 | 1.00 (.-.) | . | 1.20 (0.68-2.13) | 0.53 | 0.88 | 0.99 | 0.93 |
| GGH ---- candidate |  | G/A or A/A | 26 | 44,83 | 15 | 44,12 | 178 | 47,21 | 126 | 46,67 | 0.89 (0.41-1.93) | 0.77 | 1.14 (0.64-2.03) | 0.66 | . | . | . |
| GGH ---- candidate | rs11545078 | C/C | 48 | 82,76 | 30 | 88,24 | 310 | 82,23 | 221 | 81,85 | 1.00 (.-.) | . | 1.25 (0.80-1.94) | 0.32 | 0.97 | 0.89 | 0.42 |
| GGH ---- candidate |  | C/T or T/T | 10 | 17,24 | 4 | 11,76 | 67 | 17,77 | 49 | 18,15 | 0.98 (0.29-3.29) | 0.97 | 1.19 (0.71-2.00) | 0.51 | . | . | . |
| GGH ---- tag | rs11995525 | G/G | 30 | 51,72 | 15 | 44,12 | 218 | 57,82 | 133 | 49,26 | 1.00 (.-.) | . | 1.32 (0.70-2.50) | 0.39 | 0.88 | 0.59 | 0.29 |
| GGH ---- tag |  | G/A or A/A | 28 | 48,28 | 19 | 55,88 | 159 | 42,18 | 137 | 50,74 | 1.52 (0.69-3.35) | 0.30 | 1.89 (1.00-3.56) | 0.05 | . | . | . |
| GGH ---- tag | rs16930073 | G/G | 44 | 75,86 | 26 | 76,47 | 296 | 78,51 | 218 | 80,74 | 1.00 (.-.) | . | 1.25 (0.78-2.01) | 0.35 | 0.88 | 0.80 | 0.33 |
| GGH ---- tag |  | G/A or A/A | 14 | 24,14 | 8 | 23,53 | 81 | 21,49 | 52 | 19,26 | 0.96 (0.41-2.29) | 0.93 | 1.13 (0.66-1.94) | 0.67 | . | . | . |
| GGH ---- tag | rs17194931 | G/G | 48 | 82,76 | 30 | 88,24 | 310 | 82,23 | 221 | 81,85 | 1.00 (.-.) | . | 1.25 (0.80-1.94) | 0.32 | 0.97 | 0.89 | 0.42 |
| GGH ---- tag |  | G/A or A/A | 10 | 17,24 | 4 | 11,76 | 67 | 17,77 | 49 | 18,15 | 0.98 (0.29-3.29) | 0.97 | 1.19 (0.71-2.00) | 0.51 | . | . | . |
| GGH ---- candidate literature | rs1800909 | T/T | 30 | 51,72 | 17 | 50,00 | 181 | 48,01 | 134 | 49,63 | 1.00 (.-.) | . | 1.36 (0.74-2.51) | 0.33 | 0.69 | 0.88 | 0.42 |
| GGH ---- candidate literature |  | T/C or C/C | 28 | 48,28 | 17 | 50,00 | 196 | 51,99 | 136 | 50,37 | 0.99 (0.46-2.13) | 0.97 | 1.14 (0.62-2.09) | 0.68 | . | . | . |
| GGH ---- candidate literature | rs3758149 | C/C | 30 | 51,72 | 17 | 50,00 | 182 | 48,28 | 134 | 49,63 | 1.00 (.-.) | . | 1.33 (0.72-2.46) | 0.36 | 0.76 | 0.93 | 0.42 |
| GGH ---- candidate literature |  | C/T or T/T | 28 | 48,28 | 17 | 50,00 | 195 | 51,72 | 136 | 50,37 | 0.99 (0.46-2.14) | 0.98 | 1.16 (0.63-2.13) | 0.64 | . | . | . |
| GGH ---- tag | rs3780130 | A/A | 34 | 58,62 | 20 | 58,82 | 224 | 59,42 | 171 | 63,33 | 1.00 (.-.) | . | 1.48 (0.89-2.48) | 0.13 | 0.28 | 0.59 | 0.09 |
| GGH ---- tag |  | A/T or T/T | 24 | 41,38 | 14 | 41,18 | 153 | 40,58 | 99 | 36,67 | 1.26 (0.58-2.76) | 0.56 | 1.17 (0.69-1.99) | 0.55 | . | . | . |
| GGH ---- tag | rs4446729 | C/C | 36 | 62,07 | 20 | 58,82 | 198 | 52,52 | 148 | 54,81 | 1.00 (.-.) | . | 1.12 (0.67-1.88) | 0.66 | 0.54 | 0.99 | 0.92 |
| GGH ---- tag |  | C/T or T/T | 22 | 37,93 | 14 | 41,18 | 179 | 47,48 | 122 | 45,19 | 0.73 (0.33-1.63) | 0.44 | 1.06 (0.63-1.80) | 0.82 | . | . | . |
| GGH ---- tag | rs6472067 | C/C | 21 | 36,21 | 10 | 29,41 | 162 | 42,97 | 97 | 35,93 | 1.00 (.-.) | . | 1.76 (0.83-3.70) | 0.14 | 0.19 | 0.79 | 0.33 |
| GGH ---- tag |  | C/G or G/G | 37 | 63,79 | 24 | 70,59 | 215 | 57,03 | 173 | 64,07 | 2.28 (0.97-5.32) | 0.06 | 2.24 (1.08-4.63) | 0.03 | . | . | . |
| GGH ---- tag | rs7010484 | T/T | 33 | 56,90 | 18 | 52,94 | 177 | 46,95 | 126 | 46,67 | 1.00 (.-.) | . | 1.09 (0.62-1.92) | 0.77 | 0.52 | 0.83 | 0.36 |
| GGH ---- tag |  | T/C or C/C | 25 | 43,10 | 16 | 47,06 | 200 | 53,05 | 144 | 53,33 | 0.83 (0.38-1.78) | 0.63 | 1.17 (0.67-2.06) | 0.57 | . | . | . |
| GNMT ---- tag | rs1053538 | C/C | 22 | 37,93 | 9 | 26,47 | 93 | 24,67 | 71 | 26,30 | 1.00 (.-.) | . | 1.87 (0.80-4.38) | 0.15 | 0.23 | 0.99 | 0.75 |
| GNMT ---- tag |  | C/G or G/G | 36 | 62,07 | 25 | 73,53 | 284 | 75,33 | 199 | 73,70 | 1.82 (0.73-4.53) | 0.20 | 1.93 (0.84-4.39) | 0.12 | . | . | . |
| GNMT ---- tag | rs2296805 | G/G | 17 | 29,31 | 14 | 41,18 | 114 | 30,24 | 89 | 32,96 | 1.00 (.-.) | . | 1.50 (0.80-2.80) | 0.21 | 0.49 | 0.66 | 0.28 |
| GNMT ---- tag |  | G/T or T/T | 41 | 70,69 | 20 | 58,82 | 263 | 69,76 | 181 | 67,04 | 1.08 (0.50-2.31) | 0.85 | 1.21 (0.66-2.23) | 0.53 | . | . | . |
| GNMT ---- tag | rs6901782 | T/T | 38 | 65,52 | 23 | 67,65 | 290 | 76,92 | 215 | 79,63 | 1.00 (.-.) | . | 1.23 (0.75-2.02) | 0.42 | 0.77 | 0.94 | 0.70 |
| GNMT ---- tag |  | T/C or C/C | 20 | 34,48 | 11 | 32,35 | 87 | 23,08 | 55 | 20,37 | 1.07 (0.49-2.36) | 0.86 | 1.49 (0.85-2.62) | 0.16 | . | . | . |
| GNMT ---- tag | rs6927188 | A/A | 42 | 72,41 | 20 | 58,82 | 212 | 56,23 | 144 | 53,33 | 1.00 (.-.) | . | 1.42 (0.83-2.44) | 0.20 | 0.40 | 0.97 | 0.70 |
| GNMT ---- tag |  | A/G or G/G | 16 | 27,59 | 14 | 41,18 | 165 | 43,77 | 126 | 46,67 | 1.40 (0.66-2.99) | 0.39 | 1.41 (0.82-2.42) | 0.22 | . | . | . |
| MAT1A ---- tag | rs10887708 | G/G | 30 | 51,72 | 20 | 58,82 | 190 | 50,40 | 148 | 54,81 | 1.00 (.-.) | . | 1.21 (0.71-2.08) | 0.48 | 0.90 | 0.93 | 0.82 |
| MAT1A ---- tag |  | G/A or A/A | 28 | 48,28 | 14 | 41,18 | 187 | 49,60 | 122 | 45,19 | 0.80 (0.37-1.72) | 0.56 | 1.02 (0.59-1.76) | 0.94 | . | . | . |
| MAT1A ---- tag | rs10887718 | T/T | 18 | 31,03 | 9 | 26,47 | 107 | 28,38 | 72 | 26,67 | 1.00 (.-.) | . | 1.67 (0.70-3.97) | 0.25 | 0.42 | 0.59 | 0.20 |
| MAT1A ---- tag |  | T/C or C/C | 40 | 68,97 | 25 | 73,53 | 270 | 71,62 | 198 | 73,33 | 1.64 (0.65-4.12) | 0.29 | 1.85 (0.80-4.29) | 0.15 | . | . | . |
| MAT1A ---- tag | rs11202403 | C/C | 41 | 70,69 | 24 | 70,59 | 242 | 64,19 | 169 | 62,59 | 1.00 (.-.) | . | 1.43 (0.88-2.32) | 0.15 | 0.11 | 0.99 | 0.98 |
| MAT1A ---- tag |  | C/T or T/T | 17 | 29,31 | 10 | 29,41 | 135 | 35,81 | 101 | 37,41 | 2.29 (0.98-5.33) | 0.06 | 1.53 (0.93-2.52) | 0.09 | . | . | . |
| MAT1A ---- tag | rs1832683 | C/C | 37 | 63,79 | 25 | 73,53 | 255 | 67,64 | 186 | 68,89 | 1.00 (.-.) | . | 1.06 (0.67-1.69) | 0.81 | 0.21 | 0.93 | 0.82 |
| MAT1A ---- tag |  | C/T or T/T | 21 | 36,21 | 9 | 26,47 | 122 | 32,36 | 84 | 31,11 | 0.52 (0.21-1.32) | 0.17 | 1.00 (0.61-1.65) | 0.99 | . | . | . |
| MAT1A ---- tag | rs2236568 | C/C | 14 | 24,14 | 15 | 44,12 | 123 | 32,63 | 92 | 34,07 | 1.00 (.-.) | . | 0.83 (0.46-1.47) | 0.52 | 0.08 | 0.88 | 0.80 |
| MAT1A ---- tag |  | C/A or A/A | 44 | 75,86 | 19 | 55,88 | 254 | 67,37 | 178 | 65,93 | 0.43 (0.20-0.90) | 0.03 | 0.71 (0.41-1.25) | 0.24 | . | . | . |
| MAT1A ---- tag | rs2236569 | A/A | 24 | 41,38 | 15 | 44,12 | 183 | 48,54 | 106 | 39,26 | 1.00 (.-.) | . | 1.07 (0.56-2.05) | 0.84 | 0.52 | 0.59 | 0.05 |
| MAT1A ---- tag |  | A/G or G/G | 34 | 58,62 | 19 | 55,88 | 194 | 51,46 | 164 | 60,74 | 1.09 (0.50-2.37) | 0.83 | 1.53 (0.81-2.90) | 0.19 | . | . | . |
| MAT1A ---- tag | rs9421467 | G/G | 51 | 87,93 | 32 | 94,12 | 336 | 89,12 | 239 | 88,52 | 1.00 (.-.) | . | 1.23 (0.80-1.89) | 0.35 | 0.83 | 0.99 | 0.98 |
| MAT1A ---- tag |  | G/C or C/C | 7 | 12,07 | 2 | 5,88 | 41 | 10,88 | 31 | 11,48 | 0.83 (0.19-3.59) | 0.80 | 1.19 (0.69-2.07) | 0.53 | . | . | . |
| MAT1A ---- tag | rs998765 | A/A | 17 | 29,31 | 13 | 38,24 | 96 | 25,46 | 75 | 27,78 | 1.00 (.-.) | . | 1.39 (0.71-2.69) | 0.33 | 0.71 | 0.99 | 0.98 |
| MAT1A ---- tag |  | A/T or T/T | 41 | 70,69 | 21 | 61,76 | 281 | 74,54 | 195 | 72,22 | 1.06 (0.48-2.31) | 0.89 | 1.25 (0.67-2.35) | 0.49 | . | . | . |
| MAT1A ---- tag | rs998766 | C/C | 23 | 39,66 | 15 | 44,12 | 113 | 29,97 | 90 | 33,33 | 1.00 (.-.) | . | 1.33 (0.73-2.44) | 0.35 | 0.78 | 1.00 | 0.98 |
| MAT1A ---- tag |  | C/G or G/G | 35 | 60,34 | 19 | 55,88 | 264 | 70,03 | 180 | 66,67 | 1.04 (0.49-2.25) | 0.91 | 1.24 (0.69-2.22) | 0.47 | . | . | . |
| MAT2B ---- tag | rs12655857 | G/G | 27 | 46,55 | 18 | 52,94 | 212 | 56,23 | 147 | 54,44 | 1.00 (.-.) | . | 1.02 (0.58-1.78) | 0.96 | 0.34 | 0.99 | 0.93 |
| MAT2B ---- tag |  | G/T or T/T | 31 | 53,45 | 16 | 47,06 | 165 | 43,77 | 123 | 45,56 | 0.69 (0.32-1.47) | 0.34 | 1.03 (0.59-1.83) | 0.91 | . | . | . |
| MAT2B ---- tag | rs6869277 | C/C | 45 | 77,59 | 28 | 82,35 | 306 | 81,17 | 209 | 77,41 | 1.00 (.-.) | . | 1.20 (0.76-1.92) | 0.44 | 0.79 | 0.99 | 0.93 |
| MAT2B ---- tag |  | C/T or T/T | 13 | 22,41 | 6 | 17,65 | 71 | 18,83 | 61 | 22,59 | 0.88 (0.35-2.19) | 0.78 | 1.21 (0.71-2.05) | 0.49 | . | . | . |
| MAT2B ---- tag | rs6874065 | A/A | 19 | 32,76 | 10 | 29,41 | 105 | 27,85 | 77 | 28,52 | 1.00 (.-.) | . | 1.31 (0.65-2.67) | 0.45 | 0.81 | 0.99 | 0.93 |
| MAT2B ---- tag |  | A/G or G/G | 39 | 67,24 | 24 | 70,59 | 272 | 72,15 | 193 | 71,48 | 1.26 (0.56-2.83) | 0.58 | 1.48 (0.75-2.94) | 0.26 | . | . | . |
| MAT2B ---- tag | rs6882306 | T/T | 44 | 75,86 | 26 | 76,47 | 263 | 69,76 | 184 | 68,15 | 1.00 (.-.) | . | 1.21 (0.75-1.95) | 0.43 | 0.78 | 0.59 | 0.10 |
| MAT2B ---- tag |  | T/C or C/C | 14 | 24,14 | 8 | 23,53 | 114 | 30,24 | 86 | 31,85 | 1.18 (0.50-2.75) | 0.71 | 1.62 (0.97-2.72) | 0.07 | . | . | . |
| MAT2B ---- tag | rs7721639 | T/T | 42 | 72,41 | 25 | 73,53 | 270 | 71,62 | 196 | 72,59 | 1.00 (.-.) | . | 1.24 (0.78-1.97) | 0.37 | 0.99 | 0.99 | 0.93 |
| MAT2B ---- tag |  | T/G or G/G | 16 | 27,59 | 9 | 26,47 | 107 | 28,38 | 74 | 27,41 | 1.05 (0.43-2.55) | 0.92 | 1.31 (0.78-2.18) | 0.31 | . | . | . |
| MTHFD1 ---- tag | rs1256148 | G/G | 35 | 60,34 | 17 | 50,00 | 229 | 60,74 | 156 | 57,78 | 1.00 (.-.) | . | 1.56 (0.88-2.74) | 0.13 | 0.22 | 0.93 | 0.88 |
| MTHFD1 ---- tag |  | G/A or A/A | 23 | 39,66 | 17 | 50,00 | 148 | 39,26 | 114 | 42,22 | 1.50 (0.70-3.23) | 0.30 | 1.39 (0.78-2.47) | 0.26 | . | . | . |
| MTHFD1 ---- tag | rs13329053 | T/T | 13 | 22,41 | 8 | 23,53 | 121 | 32,10 | 93 | 34,44 | 1.00 (.-.) | . | 1.05 (0.50-2.21) | 0.89 | 0.58 | 0.99 | 0.95 |
| MTHFD1 ---- tag |  | T/C or C/C | 45 | 77,59 | 26 | 76,47 | 256 | 67,90 | 177 | 65,56 | 0.96 (0.41-2.22) | 0.92 | 1.30 (0.63-2.68) | 0.48 | . | . | . |
| MTHFD1 ---- candidate literature | rs2236224 | C/C | 20 | 34,48 | 8 | 23,53 | 146 | 38,73 | 114 | 42,22 | 1.00 (.-.) | . | 1.40 (0.68-2.92) | 0.36 | 0.75 | 0.96 | 0.88 |
| MTHFD1 ---- candidate literature |  | C/T or T/T | 38 | 65,52 | 26 | 76,47 | 231 | 61,27 | 156 | 57,78 | 1.48 (0.63-3.45) | 0.37 | 1.80 (0.87-3.72) | 0.11 | . | . | . |
| MTHFD1 ---- candidate | rs2236225 | C/C | 15 | 25,86 | 7 | 20,59 | 119 | 31,56 | 98 | 36,30 | 1.00 (.-.) | . | 1.18 (0.54-2.61) | 0.68 | 0.85 | 0.99 | 0.95 |
| MTHFD1 ---- candidate |  | C/T or T/T | 43 | 74,14 | 27 | 79,41 | 258 | 68,44 | 172 | 63,70 | 1.07 (0.45-2.58) | 0.88 | 1.39 (0.64-3.02) | 0.41 | . | . | . |
| MTHFD1 ---- tag | rs2281603 | A/A | 36 | 62,07 | 24 | 70,59 | 225 | 59,68 | 150 | 55,56 | 1.00 (.-.) | . | 1.31 (0.80-2.16) | 0.28 | 0.78 | 0.99 | 0.88 |
| MTHFD1 ---- tag |  | A/G or G/G | 22 | 37,93 | 10 | 29,41 | 152 | 40,32 | 120 | 44,44 | 1.01 (0.45-2.26) | 0.99 | 1.17 (0.71-1.94) | 0.54 | . | . | . |
| MTHFD1 ---- candidate literature | rs8003379 | A/A | 35 | 60,34 | 17 | 50,00 | 221 | 58,62 | 150 | 55,56 | 1.00 (.-.) | . | 1.25 (0.70-2.24) | 0.46 | 0.99 | 0.69 | 0.49 |
| MTHFD1 ---- candidate literature |  | A/C or C/C | 23 | 39,66 | 17 | 50,00 | 156 | 41,38 | 120 | 44,44 | 1.24 (0.58-2.65) | 0.57 | 1.56 (0.87-2.81) | 0.14 | . | . | . |
| MTHFD2 ---- tag | rs10177833 | A/A | 18 | 31,03 | 8 | 23,53 | 129 | 34,22 | 77 | 28,52 | 1.00 (.-.) | . | 1.62 (0.69-3.82) | 0.27 | 0.46 | 0.66 | 0.26 |
| MTHFD2 ---- tag |  | A/C or C/C | 40 | 68,97 | 26 | 76,47 | 248 | 65,78 | 193 | 71,48 | 1.65 (0.65-4.17) | 0.29 | 1.87 (0.82-4.31) | 0.14 | . | . | . |
| MTHFD2 ---- tag | rs702462 | T/T | 23 | 39,66 | 13 | 38,24 | 128 | 33,95 | 88 | 32,59 | 1.00 (.-.) | . | 1.60 (0.78-3.28) | 0.20 | 0.37 | 0.99 | 0.83 |
| MTHFD2 ---- tag |  | T/A or A/A | 35 | 60,34 | 21 | 61,76 | 249 | 66,05 | 182 | 67,41 | 1.31 (0.57-2.99) | 0.53 | 1.42 (0.71-2.84) | 0.33 | . | . | . |
| MTHFD2 ---- candidate literature | rs702465 | A/A | 20 | 34,48 | 8 | 23,53 | 110 | 29,18 | 68 | 25,19 | 1.00 (.-.) | . | 1.36 (0.61-3.04) | 0.46 | 0.79 | 0.95 | 0.69 |
| MTHFD2 ---- candidate literature |  | A/T or T/T | 38 | 65,52 | 26 | 76,47 | 267 | 70,82 | 202 | 74,81 | 1.55 (0.64-3.74) | 0.33 | 1.85 (0.85-4.05) | 0.12 | . | . | . |
| MTHFD2 ---- candidate literature | rs7571842 | A/A | 17 | 29,31 | 7 | 20,59 | 119 | 31,56 | 69 | 25,56 | 1.00 (.-.) | . | 1.67 (0.66-4.23) | 0.28 | 0.46 | 0.82 | 0.26 |
| MTHFD2 ---- candidate literature |  | A/G or G/G | 41 | 70,69 | 27 | 79,41 | 258 | 68,44 | 201 | 74,44 | 1.57 (0.59-4.18) | 0.37 | 1.80 (0.73-4.44) | 0.20 | . | . | . |
| MTHFD2 ---- tag | rs7587117 | T/T | 27 | 46,55 | 13 | 38,24 | 171 | 45,36 | 103 | 38,15 | 1.00 (.-.) | . | 1.54 (0.76-3.12) | 0.23 | 0.43 | 0.85 | 0.35 |
| MTHFD2 ---- tag |  | T/C or C/C | 31 | 53,45 | 21 | 61,76 | 206 | 54,64 | 167 | 61,85 | 1.53 (0.67-3.48) | 0.31 | 1.67 (0.84-3.35) | 0.15 | . | . | . |
| MTHFD2 ---- tag | rs828861 | C/C | 21 | 36,21 | 8 | 23,53 | 108 | 28,65 | 69 | 25,56 | 1.00 (.-.) | . | 1.51 (0.67-3.37) | 0.32 | 0.55 | 0.99 | 0.78 |
| MTHFD2 ---- tag |  | C/G or G/G | 37 | 63,79 | 26 | 76,47 | 269 | 71,35 | 201 | 74,44 | 1.62 (0.67-3.92) | 0.28 | 1.85 (0.85-4.05) | 0.12 | . | . | . |
| MTHFD2 ---- tag | rs828863 | G/G | 48 | 82,76 | 31 | 91,18 | 299 | 79,31 | 232 | 85,93 | 1.00 (.-.) | . | 1.26 (0.82-1.93) | 0.30 | 0.97 | 0.59 | 0.10 |
| MTHFD2 ---- tag |  | G/A or A/A | 10 | 17,24 | 3 | 8,82 | 78 | 20,69 | 38 | 14,07 | 0.65 (0.19-2.19) | 0.49 | 0.84 (0.49-1.43) | 0.52 | . | . | . |
| MTHFR ---- tag | rs1476413 | G/G | 31 | 53,45 | 18 | 52,94 | 199 | 52,79 | 143 | 52,96 | 1.00 (.-.) | . | 1.21 (0.69-2.15) | 0.50 | 0.92 | 0.99 | 0.90 |
| MTHFR ---- tag |  | G/A or A/A | 27 | 46,55 | 16 | 47,06 | 178 | 47,21 | 127 | 47,04 | 1.09 (0.51-2.32) | 0.83 | 1.38 (0.78-2.44) | 0.27 | . | . | . |
| MTHFR ---- tag | rs17376328 | G/G | 53 | 91,38 | 29 | 85,29 | 331 | 87,80 | 227 | 84,07 | 1.00 (.-.) | . | 1.22 (0.78-1.93) | 0.39 | 0.87 | 0.85 | 0.69 |
| MTHFR ---- tag |  | G/A or A/A | 5 | 8,62 | 5 | 14,71 | 46 | 12,20 | 43 | 15,93 | 1.01 (0.37-2.73) | 0.99 | 1.34 (0.78-2.31) | 0.28 | . | . | . |
| MTHFR ---- tag | rs17421462 | G/G | 49 | 84,48 | 28 | 82,35 | 325 | 86,21 | 229 | 84,81 | 1.00 (.-.) | . | 1.26 (0.80-1.99) | 0.33 | 0.95 | 0.96 | 0.84 |
| MTHFR ---- tag |  | G/A or A/A | 9 | 15,52 | 6 | 17,65 | 52 | 13,79 | 41 | 15,19 | 1.24 (0.46-3.37) | 0.67 | 1.51 (0.87-2.62) | 0.14 | . | . | . |
| MTHFR ---- candidate | rs1801131 | A/A | 25 | 43,10 | 16 | 47,06 | 175 | 46,42 | 127 | 47,04 | 1.00 (.-.) | . | 1.37 (0.74-2.53) | 0.31 | 0.66 | 0.99 | 0.90 |
| MTHFR ---- candidate |  | A/C or C/C | 33 | 56,90 | 18 | 52,94 | 202 | 53,58 | 143 | 52,96 | 1.19 (0.56-2.56) | 0.65 | 1.36 (0.74-2.50) | 0.32 | . | . | . |
| MTHFR ---- candidate | rs1801133 | C/C | 21 | 36,21 | 13 | 38,24 | 153 | 40,58 | 127 | 47,04 | 1.00 (.-.) | . | 1.12 (0.59-2.11) | 0.73 | 0.71 | 0.59 | 0.18 |
| MTHFR ----candidate |  | C/T or T/T | 37 | 63,79 | 21 | 61,76 | 224 | 59,42 | 143 | 52,96 | 0.60 (0.28-1.30) | 0.20 | 0.79 (0.42-1.46) | 0.45 | . | . | . |
| MTHFR ---- tag | rs2066471 | G/G | 38 | 65,52 | 26 | 76,47 | 267 | 70,82 | 187 | 69,26 | 1.00 (.-.) | . | 1.10 (0.68-1.78) | 0.69 | 0.37 | 0.99 | 0.84 |
| MTHFR ---- tag |  | G/A or A/A | 20 | 34,48 | 8 | 23,53 | 110 | 29,18 | 83 | 30,74 | 0.91 (0.39-2.09) | 0.82 | 1.49 (0.90-2.48) | 0.12 | . | . | . |
| MTHFR ---- tag | rs4846047 | G/G | 26 | 44,83 | 19 | 55,88 | 194 | 51,46 | 132 | 48,89 | 1.00 (.-.) | . | 1.01 (0.56-1.81) | 0.98 | 0.32 | 0.85 | 0.69 |
| MTHFR ---- tag |  | G/C or C/C | 32 | 55,17 | 15 | 44,12 | 183 | 48,54 | 138 | 51,11 | 0.86 (0.40-1.83) | 0.70 | 1.30 (0.73-2.32) | 0.37 | . | . | . |
| MTHFR ---- tag | rs4846049 | G/G | 25 | 43,10 | 15 | 44,12 | 172 | 45,62 | 124 | 45,93 | 1.00 (.-.) | . | 1.32 (0.69-2.50) | 0.40 | 0.81 | 0.99 | 0.90 |
| MTHFR ---- tag |  | G/T or T/T | 33 | 56,90 | 19 | 55,88 | 205 | 54,38 | 146 | 54,07 | 1.11 (0.51-2.41) | 0.79 | 1.32 (0.70-2.49) | 0.39 | . | . | . |
| MTHFR ---- tag | rs7538516 | T/T | 22 | 37,93 | 13 | 38,24 | 138 | 36,60 | 95 | 35,19 | 1.00 (.-.) | . | 1.22 (0.62-2.41) | 0.56 | 0.96 | 0.93 | 0.84 |
| MTHFR ---- tag |  | T/C or C/C | 36 | 62,07 | 21 | 61,76 | 239 | 63,40 | 175 | 64,81 | 1.09 (0.50-2.39) | 0.83 | 1.36 (0.70-2.63) | 0.36 | . | . | . |
| MTR ---- tag | rs10733117 | A/A | 23 | 39,66 | 12 | 35,29 | 132 | 35,01 | 102 | 37,78 | 1.00 (.-.) | . | 0.74 (0.37-1.47) | 0.39 | 0.11 | 0.99 | 0.83 |
| MTR ---- tag |  | A/G or G/G | 35 | 60,34 | 22 | 64,71 | 245 | 64,99 | 168 | 62,22 | 0.48 (0.22-1.07) | 0.07 | 0.73 (0.37-1.43) | 0.36 | . | . | . |
| MTR ---- tag | rs12129440 | G/G | 34 | 58,62 | 22 | 64,71 | 208 | 55,17 | 154 | 57,04 | 1.00 (.-.) | . | 1.18 (0.71-1.97) | 0.52 | 0.78 | 0.95 | 0.83 |
| MTR ---- tag |  | G/A or A/A | 24 | 41,38 | 12 | 35,29 | 169 | 44,83 | 116 | 42,96 | 0.97 (0.44-2.13) | 0.94 | 1.29 (0.77-2.17) | 0.34 | . | . | . |
| MTR ---- candidate | rs1805087 | A/A | 42 | 72,41 | 24 | 70,59 | 250 | 66,31 | 187 | 69,26 | 1.00 (.-.) | . | 1.35 (0.83-2.20) | 0.22 | 0.50 | 0.99 | 0.83 |
| MTR ---- candidate |  | A/G or G/G | 16 | 27,59 | 10 | 29,41 | 127 | 33,69 | 83 | 30,74 | 1.23 (0.53-2.82) | 0.63 | 1.21 (0.72-2.04) | 0.46 | . | . | . |
| MTR ---- tag | rs3890786 | C/C | 21 | 36,21 | 16 | 47,06 | 133 | 35,28 | 89 | 32,96 | 1.00 (.-.) | . | 1.36 (0.73-2.54) | 0.33 | 0.65 | 0.99 | 0.83 |
| MTR ---- tag |  | C/T or T/T | 37 | 63,79 | 18 | 52,94 | 244 | 64,72 | 181 | 67,04 | 1.26 (0.59-2.69) | 0.55 | 1.42 (0.78-2.60) | 0.25 | . | . | . |
| MTR ---- tag | rs4659727 | A/A | 42 | 72,41 | 24 | 70,59 | 249 | 66,05 | 187 | 69,26 | 1.00 (.-.) | . | 1.35 (0.83-2.20) | 0.22 | 0.49 | 0.99 | 0.83 |
| MTR ---- tag |  | A/G or G/G | 16 | 27,59 | 10 | 29,41 | 128 | 33,95 | 83 | 30,74 | 1.23 (0.53-2.82) | 0.63 | 1.21 (0.72-2.03) | 0.47 | . | . | . |
| MTRR ---- candidate literature/tag | rs10380 | C/C | 53 | 91,38 | 23 | 67,65 | 319 | 84,62 | 226 | 83,70 | 1.00 (.-.) | . | 1.46 (0.90-2.37) | 0.12 | 0.11 | 0.99 | 0.90 |
| MTRR ---- candidate literature/tag |  | C/T or T/T | 5 | 8,62 | 11 | 32,35 | 58 | 15,38 | 44 | 16,30 | 2.39 (0.99-5.75) | 0.05 | 1.56 (0.89-2.74) | 0.12 | . | . | . |
| MTRR ---- tag | rs10475399 | G/G | 26 | 44,83 | 11 | 32,35 | 165 | 43,77 | 115 | 42,59 | 1.00 (.-.) | . | 1.23 (0.65-2.31) | 0.53 | 0.96 | 0.99 | 0.90 |
| MTRR ---- tag |  | G/A or A/A | 32 | 55,17 | 23 | 67,65 | 212 | 56,23 | 155 | 57,41 | 1.04 (0.48-2.26) | 0.92 | 1.30 (0.70-2.43) | 0.41 | . | . | . |
| MTRR ---- tag | rs11134265 | C/C | 23 | 39,66 | 12 | 35,29 | 169 | 44,83 | 121 | 44,81 | 1.00 (.-.) | . | 1.17 (0.62-2.19) | 0.63 | 0.79 | 0.99 | 0.90 |
| MTRR ---- tag |  | C/T or T/T | 35 | 60,34 | 22 | 64,71 | 208 | 55,17 | 149 | 55,19 | 0.98 (0.45-2.13) | 0.95 | 1.28 (0.68-2.39) | 0.44 | . | . | . |
| MTRR ---- tag | rs13181011 | T/T | 35 | 60,34 | 20 | 58,82 | 239 | 63,40 | 169 | 62,59 | 1.00 (.-.) | . | 1.28 (0.78-2.09) | 0.34 | 0.86 | 0.99 | 0.90 |
| MTRR ---- tag |  | T/C or C/C | 23 | 39,66 | 14 | 41,18 | 138 | 36,60 | 101 | 37,41 | 1.03 (0.46-2.33) | 0.94 | 1.22 (0.73-2.04) | 0.45 | . | . | . |
| MTRR ---- tag | rs161869 | C/C | 25 | 43,10 | 7 | 20,59 | 125 | 33,16 | 93 | 34,44 | 1.00 (.-.) | . | 1.69 (0.67-4.27) | 0.27 | 0.46 | 0.99 | 0.90 |
| MTRR ---- tag |  | C/T or T/T | 33 | 56,90 | 27 | 79,41 | 252 | 66,84 | 177 | 65,56 | 1.72 (0.64-4.60) | 0.28 | 2.00 (0.81-4.95) | 0.14 | . | . | . |
| MTRR ---- tagged by rs162039 | rs162036 | A/A | 52 | 89,66 | 21 | 61,76 | 304 | 80,64 | 215 | 79,63 | 1.00 (.-.) | . | 1.48 (0.90-2.44) | 0.12 | 0.11 | 0.99 | 0.90 |
| MTRR ---- tagged by rs162039 |  | A/G or G/G | 6 | 10,34 | 13 | 38,24 | 73 | 19,36 | 55 | 20,37 | 2.47 (1.06-5.75) | 0.04 | 1.70 (0.98-2.94) | 0.06 | . | . | . |
| MTRR ---- tag | rs162039 | C/C | 52 | 89,66 | 21 | 61,76 | 305 | 80,90 | 215 | 79,63 | 1.00 (.-.) | . | 1.48 (0.90-2.44) | 0.12 | 0.11 | 0.99 | 0.90 |
| MTRR ---- tag |  | C/T or T/T | 6 | 10,34 | 13 | 38,24 | 72 | 19,10 | 55 | 20,37 | 2.47 (1.06-5.75) | 0.04 | 1.70 (0.98-2.94) | 0.06 | . | . | . |
| MTRR ---- tag | rs162270 | G/G | 41 | 70,69 | 24 | 70,59 | 271 | 71,88 | 191 | 70,74 | 1.00 (.-.) | . | 1.15 (0.70-1.87) | 0.58 | 0.56 | 0.93 | 0.90 |
| MTRR ---- tag |  | G/T or T/T | 17 | 29,31 | 10 | 29,41 | 106 | 28,12 | 79 | 29,26 | 0.80 (0.34-1.85) | 0.60 | 1.18 (0.70-2.01) | 0.53 | . | . | . |
| MTRR ---- candidate | rs16879334 | C/C | 54 | 93,10 | 33 | 97,06 | 354 | 93,90 | 253 | 93,70 | 1.00 (.-.) | . | 1.23 (0.81-1.88) | 0.33 | 0.83 | 0.97 | 0.90 |
| MTRR ---- candidate |  | C/G or G/G | 4 | 6,90 | 1 | 2,94 | 23 | 6,10 | 17 | 6,30 | 0.75 (0.10-5.61) | 0.78 | 1.15 (0.60-2.21) | 0.67 | . | . | . |
| MTRR ---- candidate | rs1801394 | G/G | 19 | 32,76 | 10 | 29,41 | 119 | 31,56 | 80 | 29,63 | 1.00 (.-.) | . | 1.02 (0.50-2.08) | 0.95 | 0.52 | 0.99 | 0.90 |
| MTRR ----candidate |  | G/A or A/A | 39 | 67,24 | 24 | 70,59 | 258 | 68,44 | 190 | 70,37 | 0.90 (0.40-2.02) | 0.79 | 1.22 (0.61-2.41) | 0.58 | . | . | . |
| MTRR ---- tag | rs1802059 | G/G | 26 | 44,83 | 17 | 50,00 | 145 | 38,46 | 104 | 38,52 | 1.00 (.-.) | . | 1.23 (0.70-2.16) | 0.46 | 0.95 | 0.99 | 0.90 |
| MTRR ---- tag |  | G/A or A/A | 32 | 55,17 | 17 | 50,00 | 232 | 61,54 | 166 | 61,48 | 0.94 (0.44-2.00) | 0.87 | 1.19 (0.69-2.05) | 0.54 | . | . | . |
| MTRR ---- tag | rs2077744 | T/T | 39 | 67,24 | 25 | 73,53 | 277 | 73,47 | 199 | 73,70 | 1.00 (.-.) | . | 1.17 (0.71-1.93) | 0.53 | 0.71 | 0.99 | 0.90 |
| MTRR ---- tag |  | T/C or C/C | 19 | 32,76 | 9 | 26,47 | 100 | 26,53 | 71 | 26,30 | 0.85 (0.38-1.92) | 0.70 | 1.17 (0.68-2.04) | 0.57 | . | . | . |
| MTRR ---- candidate | rs2287780 | C/C | 54 | 93,10 | 33 | 97,06 | 354 | 93,90 | 253 | 93,70 | 1.00 (.-.) | . | 1.23 (0.81-1.88) | 0.33 | 0.83 | 0.97 | 0.90 |
| MTRR ---- candidate |  | C/T or T/T | 4 | 6,90 | 1 | 2,94 | 23 | 6,10 | 17 | 6,30 | 0.75 (0.10-5.61) | 0.78 | 1.15 (0.60-2.21) | 0.67 | . | . | . |
| MTRR ---- candidate | rs2303080 | T/T | 54 | 93,10 | 33 | 97,06 | 354 | 93,90 | 254 | 94,07 | 1.00 (.-.) | . | 1.23 (0.81-1.88) | 0.33 | 0.84 | 0.96 | 0.90 |
| MTRR ---- candidate |  | T/A or A/A | 4 | 6,90 | 1 | 2,94 | 23 | 6,10 | 16 | 5,93 | 0.75 (0.10-5.60) | 0.78 | 1.14 (0.58-2.21) | 0.70 | . | . | . |
| MTRR ---- tag | rs7715062 | G/G | 21 | 36,21 | 13 | 38,24 | 127 | 33,69 | 91 | 33,70 | 1.00 (.-.) | . | 1.55 (0.81-2.95) | 0.19 | 0.37 | 1.00 | 0.99 |
| MTRR ---- tag |  | G/T or T/T | 37 | 63,79 | 21 | 61,76 | 250 | 66,31 | 179 | 66,30 | 1.35 (0.62-2.92) | 0.45 | 1.43 (0.77-2.68) | 0.26 | . | . | . |
| MTRR ---- tag | rs9282787 | T/T | 37 | 63,79 | 21 | 61,76 | 241 | 63,93 | 171 | 63,33 | 1.00 (.-.) | . | 1.32 (0.80-2.16) | 0.27 | 0.66 | 0.93 | 0.90 |
| MTRR ---- tag |  | T/C or C/C | 21 | 36,21 | 13 | 38,24 | 136 | 36,07 | 99 | 36,67 | 1.16 (0.52-2.61) | 0.72 | 1.26 (0.75-2.11) | 0.38 | . | . | . |
| MTRR ---- candidate literature | rs9332 | C/C | 52 | 89,66 | 21 | 61,76 | 305 | 80,90 | 215 | 79,63 | 1.00 (.-.) | . | 1.48 (0.90-2.44) | 0.12 | 0.11 | 0.99 | 0.90 |
| MTRR ---- candidate literature |  | C/T or T/T | 6 | 10,34 | 13 | 38,24 | 72 | 19,10 | 55 | 20,37 | 2.47 (1.06-5.75) | 0.04 | 1.70 (0.98-2.94) | 0.06 | . | . | . |
| NFKB1 ---- NA | rs1609798 | C/C | 19 | 32,76 | 17 | 50,00 | 174 | 46,15 | 130 | 48,15 | 1.00 (.-.) | . | 1.25 (0.72-2.19) | 0.43 | 0.98 | 0.99 | 0.96 |
| NFKB1 ---- NA |  | C/T or T/T | 39 | 67,24 | 17 | 50,00 | 203 | 53,85 | 140 | 51,85 | 0.94 (0.43-2.02) | 0.87 | 1.17 (0.67-2.04) | 0.59 | . | . | . |
| NFKB1 ---- tag | rs230540 | T/T | 18 | 31,03 | 14 | 41,18 | 156 | 41,38 | 113 | 41,85 | 1.00 (.-.) | . | 1.23 (0.67-2.24) | 0.50 | 0.94 | 0.96 | 0.96 |
| NFKB1 ---- tag |  | T/C or C/C | 40 | 68,97 | 20 | 58,82 | 221 | 58,62 | 157 | 58,15 | 0.89 (0.42-1.92) | 0.78 | 1.13 (0.63-2.05) | 0.68 | . | . | . |
| NFKB1 ---- tag | rs230541 | A/A | 14 | 24,14 | 11 | 32,35 | 123 | 32,63 | 94 | 34,81 | 1.00 (.-.) | . | 1.07 (0.55-2.09) | 0.85 | 0.59 | 0.99 | 0.96 |
| NFKB1 ---- tag |  | A/G or G/G | 44 | 75,86 | 23 | 67,65 | 254 | 67,37 | 176 | 65,19 | 0.81 (0.37-1.77) | 0.60 | 1.08 (0.56-2.09) | 0.81 | . | . | . |
| NFKB1 ---- NA | rs230547 | C/C | 52 | 89,66 | 26 | 76,47 | 290 | 76,92 | 228 | 84,44 | 1.00 (.-.) | . | 1.43 (0.90-2.27) | 0.14 | 0.14 | 0.99 | 0.96 |
| NFKB1 ---- NA |  | C/T or T/T | 6 | 10,34 | 8 | 23,53 | 87 | 23,08 | 42 | 15,56 | 1.60 (0.67-3.82) | 0.29 | 1.09 (0.62-1.89) | 0.77 | . | . | . |
| NFKB1 ---- tag | rs3774934 | G/G | 50 | 86,21 | 26 | 76,47 | 285 | 75,60 | 221 | 81,85 | 1.00 (.-.) | . | 1.39 (0.87-2.22) | 0.16 | 0.23 | 0.99 | 0.96 |
| NFKB1 ---- tag |  | G/A or A/A | 8 | 13,79 | 8 | 23,53 | 92 | 24,40 | 49 | 18,15 | 1.47 (0.61-3.49) | 0.39 | 1.13 (0.65-1.95) | 0.66 | . | . | . |
| NFKB1 ---- tag | rs3774968 | G/G | 29 | 50,00 | 10 | 29,41 | 119 | 31,56 | 80 | 29,63 | 1.00 (.-.) | . | 1.99 (0.94-4.21) | 0.07 | 0.08 | 0.83 | 0.96 |
| NFKB1 ---- tag |  | G/A or A/A | 29 | 50,00 | 24 | 70,59 | 258 | 68,44 | 190 | 70,37 | 2.23 (0.97-5.12) | 0.06 | 2.10 (1.01-4.36) | 0.05 | . | . | . |
| NFKB1 ---- NA | rs4648022 | C/C | 45 | 77,59 | 32 | 94,12 | 318 | 84,35 | 233 | 86,30 | 1.00 (.-.) | . | 1.13 (0.73-1.74) | 0.58 | 0.21 | 0.99 | 0.96 |
| NFKB1 ---- NA |  | C/T or T/T | 13 | 22,41 | 2 | 5,88 | 59 | 15,65 | 37 | 13,70 | 0.52 (0.12-2.20) | 0.37 | 1.38 (0.80-2.36) | 0.24 | . | . | . |
| NFKB1 ---- NA | rs4648090 | G/G | 40 | 68,97 | 30 | 88,24 | 281 | 74,54 | 201 | 74,44 | 1.00 (.-.) | . | 1.08 (0.69-1.68) | 0.74 | 0.15 | 0.99 | 0.96 |
| NFKB1 ---- NA |  | G/A or A/A | 18 | 31,03 | 4 | 11,76 | 96 | 25,46 | 69 | 25,56 | 0.49 (0.15-1.64) | 0.25 | 1.23 (0.76-1.99) | 0.41 | . | . | . |
| NFKB1 ---- tag | rs4648110 | T/T | 37 | 63,79 | 25 | 73,53 | 246 | 65,25 | 172 | 63,70 | 1.00 (.-.) | . | 0.92 (0.57-1.49) | 0.74 | 0.04 | 0.99 | 0.96 |
| NFKB1 ---- tag |  | T/A or A/A | 21 | 36,21 | 9 | 26,47 | 131 | 34,75 | 98 | 36,30 | 0.43 (0.17-1.08) | 0.07 | 1.03 (0.63-1.68) | 0.92 | . | . | . |
| NFKB1 ---- tag | rs4648141 | G/G | 40 | 68,97 | 26 | 76,47 | 269 | 71,35 | 193 | 71,48 | 1.00 (.-.) | . | 0.90 (0.56-1.45) | 0.67 | 0.02 | 0.93 | 0.96 |
| NFKB1 ---- tag |  | G/A or A/A | 18 | 31,03 | 8 | 23,53 | 108 | 28,65 | 77 | 28,52 | 0.41 (0.16-1.04) | 0.06 | 1.07 (0.65-1.78) | 0.78 | . | . | . |
| NFKB1 ---- tag | rs4698863 | C/C | 17 | 29,31 | 16 | 47,06 | 169 | 44,83 | 129 | 47,78 | 1.00 (.-.) | . | 1.15 (0.65-2.06) | 0.63 | 0.73 | 0.99 | 0.96 |
| NFKB1 ---- tag |  | C/T or T/T | 41 | 70,69 | 18 | 52,94 | 208 | 55,17 | 141 | 52,22 | 0.79 (0.37-1.70) | 0.55 | 1.06 (0.59-1.88) | 0.85 | . | . | . |
| NFKB1 ---- NA | rs7674640 | C/C | 8 | 13,79 | 8 | 23,53 | 87 | 23,08 | 64 | 23,70 | 1.00 (.-.) | . | 0.77 (0.35-1.66) | 0.50 | 0.19 | 0.99 | 0.96 |
| NFKB1 ---- NA |  | C/T or T/T | 50 | 86,21 | 26 | 76,47 | 290 | 76,92 | 206 | 76,30 | 0.53 (0.23-1.23) | 0.14 | 0.75 (0.36-1.59) | 0.46 | . | . | . |
| NFKB1 ---- tag | rs909332 | A/A | 54 | 93,10 | 29 | 85,29 | 334 | 88,59 | 252 | 93,33 | 1.00 (.-.) | . | 1.37 (0.87-2.15) | 0.17 | 0.20 | 0.99 | 0.96 |
| NFKB1 ---- tag |  | A/T or T/T | 4 | 6,90 | 5 | 14,71 | 43 | 11,41 | 18 | 6,67 | 1.96 (0.73-5.26) | 0.18 | 1.25 (0.64-2.43) | 0.52 | . | . | . |
| NFKB1 ---- tag | rs997476 | C/C | 54 | 93,10 | 29 | 85,29 | 339 | 89,92 | 240 | 88,89 | 1.00 (.-.) | . | 1.10 (0.71-1.71) | 0.66 | 0.19 | 0.66 | 0.90 |
| NFKB1 ---- tag |  | C/A or A/A | 4 | 6,90 | 5 | 14,71 | 38 | 10,08 | 30 | 11,11 | 0.51 (0.15-1.72) | 0.28 | 1.24 (0.71-2.17) | 0.46 | . | . | . |
| NME1 ---- NA | rs10514981 | T/T | 31 | 53,45 | 22 | 64,71 | 230 | 61,01 | 175 | 64,81 | 1.00 (.-.) | . | 1.25 (0.75-2.08) | 0.39 | 0.94 | 0.85 | 0.89 |
| NME1 ---- NA |  | T/G or G/G | 27 | 46,55 | 12 | 35,29 | 147 | 38,99 | 95 | 35,19 | 0.93 (0.43-2.04) | 0.86 | 1.13 (0.66-1.93) | 0.65 | . | . | . |
| NME1 ---- NA | rs11651252 | T/T | 48 | 82,76 | 27 | 79,41 | 340 | 90,19 | 240 | 88,89 | 1.00 (.-.) | . | 1.45 (0.92-2.30) | 0.11 | 0.05 | 0.99 | 0.89 |
| NME1 ---- NA |  | T/C or C/C | 10 | 17,24 | 7 | 20,59 | 37 | 9,81 | 30 | 11,11 | 2.76 (1.09-6.98) | 0.03 | 1.36 (0.75-2.46) | 0.31 | . | . | . |
| NME1 ---- tag | rs11652793 | T/T | 34 | 58,62 | 24 | 70,59 | 240 | 63,66 | 189 | 70,00 | 1.00 (.-.) | . | 1.16 (0.71-1.88) | 0.55 | 0.65 | 0.93 | 0.89 |
| NME1 ---- tag |  | T/C or C/C | 24 | 41,38 | 10 | 29,41 | 137 | 36,34 | 81 | 30,00 | 0.76 (0.33-1.73) | 0.51 | 1.08 (0.64-1.81) | 0.79 | . | . | . |
| NME1 ---- NA | rs11868380 | C/C | 37 | 63,79 | 17 | 50,00 | 234 | 62,07 | 168 | 62,22 | 1.00 (.-.) | . | 1.34 (0.73-2.47) | 0.34 | 0.76 | 1.00 | 0.99 |
| NME1 ---- NA |  | C/G or G/G | 21 | 36,21 | 17 | 50,00 | 143 | 37,93 | 102 | 37,78 | 1.18 (0.55-2.55) | 0.67 | 1.40 (0.75-2.62) | 0.30 | . | . | . |
| NME1 ---- NA | rs1558252 | T/T | 32 | 55,17 | 19 | 55,88 | 194 | 51,46 | 117 | 43,33 | 1.00 (.-.) | . | 1.20 (0.68-2.11) | 0.54 | 0.92 | 0.99 | 0.99 |
| NME1 ---- NA |  | T/C or C/C | 26 | 44,83 | 15 | 44,12 | 183 | 48,54 | 153 | 56,67 | 1.16 (0.54-2.47) | 0.71 | 1.44 (0.83-2.52) | 0.19 | . | . | . |
| NME1 ---- NA | rs1558253 | T/T | 56 | 96,55 | 31 | 91,18 | 336 | 89,12 | 234 | 86,67 | 1.00 (.-.) | . | 1.28 (0.83-1.98) | 0.26 | 0.59 | 0.90 | 0.89 |
| NME1 ---- NA |  | T/G or G/G | 2 | 3,45 | 3 | 8,82 | 41 | 10,88 | 36 | 13,33 | 1.48 (0.43-5.04) | 0.53 | 1.32 (0.77-2.26) | 0.32 | . | . | . |
| NME1 ---- tag | rs16949683 | C/C | 53 | 91,38 | 29 | 85,29 | 355 | 94,16 | 250 | 92,59 | 1.00 (.-.) | . | 1.43 (0.91-2.25) | 0.12 | 0.02 | 0.99 | 0.89 |
| NME1 ---- tag |  | C/T or T/T | 5 | 8,62 | 5 | 14,71 | 22 | 5,84 | 20 | 7,41 | 4.82 (1.79-12.99) | 0.00 | 1.66 (0.87-3.16) | 0.13 | . | . | . |
| NME1 ---- tag | rs2318784 | C/C | 39 | 67,24 | 23 | 67,65 | 295 | 78,25 | 202 | 74,81 | 1.00 (.-.) | . | 1.35 (0.83-2.20) | 0.23 | 0.48 | 0.99 | 0.89 |
| NME1 ---- tag |  | C/T or T/T | 19 | 32,76 | 11 | 32,35 | 82 | 21,75 | 68 | 25,19 | 1.49 (0.64-3.46) | 0.36 | 1.44 (0.85-2.44) | 0.17 | . | . | . |
| NME1 ---- NA | rs2318785 | G/G | 19 | 32,76 | 13 | 38,24 | 115 | 30,50 | 75 | 27,78 | 1.00 (.-.) | . | 1.28 (0.65-2.54) | 0.47 | 0.88 | 0.99 | 0.89 |
| NME1 ---- NA |  | G/A or A/A | 39 | 67,24 | 21 | 61,76 | 262 | 69,50 | 195 | 72,22 | 1.14 (0.52-2.52) | 0.74 | 1.37 (0.72-2.64) | 0.34 | . | . | . |
| NME1 ---- tag | rs3760469 | G/G | 20 | 34,48 | 12 | 35,29 | 96 | 25,46 | 62 | 22,96 | 1.00 (.-.) | . | 1.44 (0.70-2.98) | 0.32 | 0.57 | 0.99 | 0.99 |
| NME1 ---- tag |  | G/T or T/T | 38 | 65,52 | 22 | 64,71 | 281 | 74,54 | 208 | 77,04 | 1.37 (0.60-3.11) | 0.46 | 1.53 (0.77-3.03) | 0.22 | . | . | . |
| NME1 ---- NA | rs4605213 | G/G | 23 | 39,66 | 15 | 44,12 | 152 | 40,32 | 118 | 43,70 | 1.00 (.-.) | . | 1.54 (0.83-2.85) | 0.17 | 0.32 | 0.93 | 0.89 |
| NME1 ---- NA |  | G/C or C/C | 35 | 60,34 | 19 | 55,88 | 225 | 59,68 | 152 | 56,30 | 1.42 (0.66-3.02) | 0.37 | 1.46 (0.79-2.70) | 0.23 | . | . | . |
| NME1 ---- NA | rs7207090 | A/A | 15 | 25,86 | 10 | 29,41 | 96 | 25,46 | 64 | 23,70 | 1.00 (.-.) | . | 1.72 (0.79-3.74) | 0.17 | 0.31 | 0.99 | 0.89 |
| NME1 ---- NA |  | A/T or T/T | 43 | 74,14 | 24 | 70,59 | 281 | 74,54 | 206 | 76,30 | 1.42 (0.62-3.29) | 0.41 | 1.55 (0.75-3.22) | 0.24 | . | . | . |
| NME1 ---- tag | rs7222463 | A/A | 10 | 17,24 | 8 | 23,53 | 99 | 26,26 | 76 | 28,15 | 1.00 (.-.) | . | 1.23 (0.48-3.16) | 0.67 | 0.98 | 0.93 | 0.89 |
| NME1 ---- tag |  | A/C or C/C | 48 | 82,76 | 26 | 76,47 | 278 | 73,74 | 194 | 71,85 | 1.00 (0.37-2.69) | 1.00 | 1.25 (0.49-3.15) | 0.64 | . | . | . |
| NME1 ---- tag | rs7226059 | C/C | 27 | 46,55 | 13 | 38,24 | 159 | 42,18 | 119 | 44,07 | 1.00 (.-.) | . | 1.84 (0.99-3.41) | 0.05 | 0.06 | 0.83 | 0.89 |
| NME1 ---- tag |  | C/T or T/T | 31 | 53,45 | 21 | 61,76 | 218 | 57,82 | 151 | 55,93 | 1.93 (0.90-4.17) | 0.09 | 1.63 (0.88-3.01) | 0.12 | . | . | . |
| NME1 ---- NA | rs880178 | G/G | 13 | 22,41 | 10 | 29,41 | 98 | 25,99 | 62 | 22,96 | 1.00 (.-.) | . | 1.09 (0.51-2.37) | 0.82 | 0.72 | 0.99 | 0.99 |
| NME1 ---- NA |  | G/T or T/T | 45 | 77,59 | 24 | 70,59 | 279 | 74,01 | 208 | 77,04 | 0.94 (0.41-2.15) | 0.88 | 1.21 (0.58-2.49) | 0.61 | . | . | . |
| NME2 ---- tag | rs7220360 | C/C | 10 | 17,24 | 8 | 23,53 | 99 | 26,26 | 76 | 28,15 | 1.00 (.-.) | . | 1.23 (0.48-3.16) | 0.67 | 0.98 | 0.93 | 0.43 |
| NME2 ---- tag |  | C/G or G/G | 48 | 82,76 | 26 | 76,47 | 278 | 73,74 | 194 | 71,85 | 1.00 (0.37-2.69) | 1.00 | 1.25 (0.49-3.15) | 0.64 | . | . | . |
| PON1 ---- tag | rs2269829 | A/A | 33 | 56,90 | 19 | 55,88 | 203 | 53,85 | 125 | 46,30 | 1.00 (.-.) | . | 1.27 (0.73-2.19) | 0.40 | 0.87 | 0.66 | 0.08 |
| PON1 ---- tag |  | A/G or G/G | 25 | 43,10 | 15 | 44,12 | 174 | 46,15 | 145 | 53,70 | 1.22 (0.56-2.62) | 0.62 | 1.44 (0.83-2.49) | 0.19 | . | . | . |
| PON1 ---- tag | rs3917527 | A/A | 52 | 89,66 | 32 | 94,12 | 334 | 88,59 | 253 | 93,70 | 1.00 (.-.) | . | 1.34 (0.88-2.05) | 0.17 | 0.28 | 0.59 | 0.08 |
| PON1 ---- tag |  | A/G or G/G | 6 | 10,34 | 2 | 5,88 | 43 | 11,41 | 17 | 6,30 | 1.28 (0.30-5.49) | 0.74 | 0.66 (0.35-1.25) | 0.21 | . | . | . |
| PON1 ---- tag | rs3917538 | C/C | 37 | 63,79 | 20 | 58,82 | 231 | 61,27 | 132 | 48,89 | 1.00 (.-.) | . | 1.18 (0.69-2.01) | 0.55 | 0.85 | 0.59 | 0.04 |
| PON1 ---- tag |  | C/T or T/T | 21 | 36,21 | 14 | 41,18 | 146 | 38,73 | 138 | 51,11 | 1.30 (0.59-2.83) | 0.52 | 1.65 (0.97-2.83) | 0.07 | . | . | . |
| PON1 ---- tag | rs757158 | C/C | 20 | 34,48 | 11 | 32,35 | 144 | 38,20 | 102 | 37,78 | 1.00 (.-.) | . | 1.43 (0.73-2.80) | 0.29 | 0.60 | 0.61 | 0.08 |
| PON1 ---- tag |  | C/T or T/T | 38 | 65,52 | 23 | 67,65 | 233 | 61,80 | 168 | 62,22 | 0.95 (0.43-2.07) | 0.89 | 1.09 (0.56-2.11) | 0.80 | . | . | . |
| PON1 ---- candidate | rs854560 | A/A | 20 | 34,48 | 13 | 38,24 | 129 | 34,22 | 121 | 44,81 | 1.00 (.-.) | . | 1.29 (0.66-2.53) | 0.45 | 0.86 | 0.99 | 0.87 |
| PON1 ---- candidate |  | A/T or T/T | 38 | 65,52 | 21 | 61,76 | 248 | 65,78 | 149 | 55,19 | 1.02 (0.46-2.24) | 0.97 | 1.22 (0.63-2.39) | 0.56 | . | . | . |
| PRDM2 ---- tag | rs1015370 | C/C | 25 | 43,10 | 22 | 64,71 | 196 | 51,99 | 157 | 58,15 | 1.00 (.-.) | . | 1.31 (0.78-2.22) | 0.31 | 0.76 | 0.88 | 0.60 |
| PRDM2 ---- tag |  | C/T or T/T | 33 | 56,90 | 12 | 35,29 | 181 | 48,01 | 113 | 41,85 | 0.94 (0.43-2.04) | 0.88 | 1.09 (0.64-1.86) | 0.76 | . | . | . |
| PRDM2 ---- tag | rs1203634 | A/A | 37 | 63,79 | 24 | 70,59 | 240 | 63,66 | 162 | 60,00 | 1.00 (.-.) | . | 1.13 (0.69-1.83) | 0.63 | 0.50 | 0.59 | 0.16 |
| PRDM2 ---- tag |  | A/G or G/G | 21 | 36,21 | 10 | 29,41 | 137 | 36,34 | 108 | 40,00 | 0.82 (0.36-1.87) | 0.63 | 1.24 (0.75-2.05) | 0.41 | . | . | . |
| PRDM2 ---- tag | rs1203645 | A/A | 27 | 46,55 | 14 | 41,18 | 162 | 42,97 | 106 | 39,26 | 1.00 (.-.) | . | 1.28 (0.69-2.38) | 0.44 | 0.89 | 0.59 | 0.16 |
| PRDM2 ---- tag |  | A/C or C/C | 31 | 53,45 | 20 | 58,82 | 215 | 57,03 | 164 | 60,74 | 1.22 (0.57-2.62) | 0.60 | 1.48 (0.80-2.72) | 0.21 | . | . | . |
| PRDM2 ---- tag | rs1406416 | C/C | 33 | 56,90 | 15 | 44,12 | 194 | 51,46 | 140 | 51,85 | 1.00 (.-.) | . | 1.22 (0.64-2.32) | 0.56 | 0.94 | 0.99 | 0.87 |
| PRDM2 ---- tag |  | C/T or T/T | 25 | 43,10 | 19 | 55,88 | 183 | 48,54 | 130 | 48,15 | 0.97 (0.44-2.11) | 0.93 | 1.22 (0.64-2.32) | 0.56 | . | . | . |
| PRDM2 ---- candidate | rs17350795 | G/G | 57 | 98,28 | 33 | 97,06 | 359 | 95,23 | 256 | 94,81 | 1.00 (.-.) | . | 1.23 (0.80-1.87) | 0.34 | 0.75 | 0.99 | 0.87 |
| PRDM2 ---- candidate |  | G/A or A/A | 1 | 1,72 | 1 | 2,94 | 18 | 4,77 | 14 | 5,19 | 0.53 (0.07-4.03) | 0.54 | 0.90 (0.44-1.85) | 0.78 | . | . | . |
| PRDM2 ---- tag | rs1980472 | C/C | 36 | 62,07 | 21 | 61,76 | 218 | 57,82 | 144 | 53,33 | 1.00 (.-.) | . | 1.17 (0.70-1.95) | 0.55 | 0.71 | 0.59 | 0.16 |
| PRDM2 ---- tag |  | C/G or G/G | 22 | 37,93 | 13 | 38,24 | 159 | 42,18 | 126 | 46,67 | 0.88 (0.40-1.94) | 0.76 | 1.21 (0.72-2.04) | 0.48 | . | . | . |
| PRDM2 ---- tag | rs2235515 | G/G | 33 | 56,90 | 19 | 55,88 | 229 | 60,74 | 159 | 58,89 | 1.00 (.-.) | . | 1.22 (0.69-2.17) | 0.49 | 0.91 | 0.83 | 0.43 |
| PRDM2 ---- tag |  | G/A or A/A | 25 | 43,10 | 15 | 44,12 | 148 | 39,26 | 111 | 41,11 | 1.03 (0.49-2.19) | 0.93 | 1.32 (0.74-2.37) | 0.35 | . | . | . |
| PRDM2 ---- tag | rs2244634 | A/A | 33 | 56,90 | 24 | 70,59 | 247 | 65,52 | 181 | 67,04 | 1.00 (.-.) | . | 1.21 (0.75-1.95) | 0.44 | 0.84 | 0.99 | 0.99 |
| PRDM2 ---- tag |  | A/C or C/C | 25 | 43,10 | 10 | 29,41 | 130 | 34,48 | 89 | 32,96 | 0.93 (0.40-2.19) | 0.88 | 1.24 (0.75-2.06) | 0.41 | . | . | . |
| PRDM2 ---- tag | rs2245213 | G/G | 39 | 67,24 | 25 | 73,53 | 278 | 73,74 | 198 | 73,33 | 1.00 (.-.) | . | 1.29 (0.80-2.08) | 0.31 | 0.78 | 0.97 | 0.87 |
| PRDM2 ---- tag |  | G/T or T/T | 19 | 32,76 | 9 | 26,47 | 99 | 26,26 | 72 | 26,67 | 1.14 (0.49-2.64) | 0.77 | 1.28 (0.76-2.16) | 0.36 | . | . | . |
| PRDM2 ---- tag | rs2294484 | C/C | 48 | 82,76 | 26 | 76,47 | 316 | 83,82 | 222 | 82,22 | 1.00 (.-.) | . | 1.18 (0.74-1.86) | 0.49 | 0.50 | 0.99 | 0.87 |
| PRDM2 ---- tag |  | C/G or G/G | 10 | 17,24 | 8 | 23,53 | 61 | 16,18 | 48 | 17,78 | 1.04 (0.42-2.59) | 0.94 | 1.70 (1.00-2.90) | 0.05 | . | . | . |
| PRDM2 ---- tag | rs2744689 | G/G | 40 | 68,97 | 26 | 76,47 | 279 | 74,01 | 199 | 73,70 | 1.00 (.-.) | . | 1.27 (0.80-2.03) | 0.31 | 0.83 | 1.00 | 0.99 |
| PRDM2 ---- tag |  | G/A or A/A | 18 | 31,03 | 8 | 23,53 | 98 | 25,99 | 71 | 26,30 | 1.08 (0.44-2.63) | 0.87 | 1.24 (0.74-2.07) | 0.41 | . | . | . |
| PRDM2 ---- tag | rs6690270 | A/A | 26 | 44,83 | 10 | 29,41 | 142 | 37,67 | 106 | 39,26 | 1.00 (.-.) | . | 1.49 (0.71-3.14) | 0.30 | 0.54 | 0.89 | 0.60 |
| PRDM2 ---- tag |  | A/G or G/G | 32 | 55,17 | 24 | 70,59 | 235 | 62,33 | 164 | 60,74 | 1.22 (0.53-2.81) | 0.64 | 1.39 (0.66-2.90) | 0.38 | . | . | . |
| RRM1 ---- tag | rs10835601 | G/G | 26 | 44,83 | 21 | 61,76 | 187 | 49,60 | 130 | 48,15 | 1.00 (.-.) | . | 1.09 (0.64-1.84) | 0.76 | 0.46 | 1.00 | 1.00 |
| RRM1 ---- tag |  | G/A or A/A | 32 | 55,17 | 13 | 38,24 | 190 | 50,40 | 140 | 51,85 | 0.76 (0.35-1.65) | 0.50 | 1.13 (0.67-1.91) | 0.64 | . | . | . |
| RRM1 ---- tag | rs10835613 | C/C | 20 | 34,48 | 12 | 35,29 | 139 | 36,87 | 96 | 35,56 | 1.00 (.-.) | . | 1.23 (0.64-2.33) | 0.54 | 0.97 | 0.99 | 0.94 |
| RRM1 ---- tag |  | C/G or G/G | 38 | 65,52 | 22 | 64,71 | 238 | 63,13 | 174 | 64,44 | 1.04 (0.48-2.25) | 0.91 | 1.30 (0.70-2.41) | 0.41 | . | . | . |
| RRM1 ---- NA | rs10835677 | G/G | 49 | 84,48 | 31 | 91,18 | 303 | 80,37 | 223 | 82,59 | 1.00 (.-.) | . | 1.19 (0.77-1.83) | 0.43 | 0.49 | 0.99 | 0.94 |
| RRM1 ---- NA |  | G/A or A/A | 9 | 15,52 | 3 | 8,82 | 74 | 19,63 | 47 | 17,41 | 0.59 (0.14-2.52) | 0.47 | 1.15 (0.69-1.90) | 0.59 | . | . | . |
| RRM1 ---- tag | rs10835678 | A/A | 53 | 91,38 | 30 | 88,24 | 332 | 88,06 | 244 | 90,37 | 1.00 (.-.) | . | 1.29 (0.84-1.98) | 0.24 | 0.49 | 0.99 | 0.94 |
| RRM1 ---- tag |  | A/G or G/G | 5 | 8,62 | 4 | 11,76 | 45 | 11,94 | 26 | 9,63 | 1.60 (0.36-7.05) | 0.54 | 1.15 (0.65-2.06) | 0.63 | . | . | . |
| RRM1 ---- tag | rs12288551 | C/C | 53 | 91,38 | 33 | 97,06 | 348 | 92,31 | 237 | 87,78 | 1.00 (.-.) | . | 1.20 (0.79-1.83) | 0.38 | 0.69 | 0.93 | 0.94 |
| RRM1 ---- tag |  | C/G or G/G | 5 | 8,62 | 1 | 2,94 | 29 | 7,69 | 33 | 12,22 | 0.92 (0.12-6.87) | 0.93 | 1.65 (0.94-2.87) | 0.08 | . | . | . |
| RRM1 ---- NA | rs12806698 | C/C | 29 | 50,00 | 19 | 55,88 | 203 | 53,85 | 139 | 51,48 | 1.00 (.-.) | . | 1.07 (0.64-1.81) | 0.79 | 0.41 | 0.99 | 0.94 |
| RRM1 ---- NA |  | C/A or A/A | 29 | 50,00 | 15 | 44,12 | 174 | 46,15 | 131 | 48,52 | 0.76 (0.35-1.67) | 0.50 | 1.15 (0.68-1.95) | 0.59 | . | . | . |
| RRM1 ---- NA | rs1465952 | T/T | 47 | 81,03 | 25 | 73,53 | 308 | 81,70 | 227 | 84,07 | 1.00 (.-.) | . | 1.26 (0.81-1.99) | 0.31 | 0.85 | 0.99 | 1.00 |
| RRM1 ---- NA |  | T/C or C/C | 11 | 18,97 | 9 | 26,47 | 69 | 18,30 | 43 | 15,93 | 0.98 (0.36-2.63) | 0.96 | 1.11 (0.65-1.90) | 0.70 | . | . | . |
| RRM1 ---- tag | rs4910904 | A/A | 25 | 43,10 | 14 | 41,18 | 180 | 47,75 | 112 | 41,48 | 1.00 (.-.) | . | 1.09 (0.59-2.01) | 0.78 | 0.60 | 0.93 | 0.94 |
| RRM1 ---- tag |  | A/G or G/G | 33 | 56,90 | 20 | 58,82 | 197 | 52,25 | 158 | 58,52 | 0.95 (0.44-2.02) | 0.89 | 1.28 (0.70-2.34) | 0.42 | . | . | . |
| RRM1 ---- tag | rs7103860 | T/T | 44 | 75,86 | 22 | 64,71 | 293 | 77,72 | 216 | 80,00 | 1.00 (.-.) | . | 1.28 (0.79-2.08) | 0.32 | 0.81 | 0.93 | 0.94 |
| RRM1 ---- tag |  | T/C or C/C | 14 | 24,14 | 12 | 35,29 | 84 | 22,28 | 54 | 20,00 | 1.13 (0.49-2.62) | 0.77 | 1.30 (0.76-2.23) | 0.35 | . | . | . |
| RRM1 ---- tag | rs7115496 | C/C | 53 | 91,38 | 30 | 88,24 | 321 | 85,15 | 240 | 88,89 | 1.00 (.-.) | . | 1.29 (0.84-1.99) | 0.24 | 0.53 | 0.98 | 0.94 |
| RRM1 ---- tag |  | C/T or T/T | 5 | 8,62 | 4 | 11,76 | 56 | 14,85 | 30 | 11,11 | 1.46 (0.33-6.40) | 0.62 | 1.12 (0.64-1.95) | 0.70 | . | . | . |
| RRM2 ---- NA | rs1138729 | A/A | 40 | 68,97 | 23 | 67,65 | 266 | 70,56 | 206 | 76,30 | 1.00 (.-.) | . | 1.35 (0.81-2.25) | 0.24 | 0.55 | 0.88 | 0.77 |
| RRM2 ---- NA |  | A/G or G/G | 18 | 31,03 | 11 | 32,35 | 111 | 29,44 | 64 | 23,70 | 1.27 (0.57-2.81) | 0.56 | 1.32 (0.76-2.29) | 0.32 | . | . | . |
| RRM2 ---- tag | rs4668664 | G/G | 25 | 43,10 | 16 | 47,06 | 185 | 49,07 | 138 | 51,11 | 1.00 (.-.) | . | 1.24 (0.68-2.28) | 0.48 | 0.98 | 0.93 | 0.77 |
| RRM2 ---- tag |  | G/A or A/A | 33 | 56,90 | 18 | 52,94 | 192 | 50,93 | 132 | 48,89 | 0.98 (0.46-2.10) | 0.96 | 1.21 (0.66-2.23) | 0.54 | . | . | . |
| RRM2 ---- NA | rs6741290 | C/C | 14 | 24,14 | 11 | 32,35 | 110 | 29,18 | 97 | 35,93 | 1.00 (.-.) | . | 1.21 (0.58-2.54) | 0.61 | 0.95 | 0.99 | 0.77 |
| RRM2 ---- NA |  | C/T or T/T | 44 | 75,86 | 23 | 67,65 | 267 | 70,82 | 173 | 64,07 | 0.89 (0.39-2.05) | 0.79 | 1.11 (0.54-2.29) | 0.78 | . | . | . |
| RRM2 ---- tag | rs7574663 | C/C | 35 | 60,34 | 22 | 64,71 | 228 | 60,48 | 182 | 67,41 | 1.00 (.-.) | . | 1.18 (0.71-1.98) | 0.52 | 0.78 | 0.99 | 0.77 |
| RRM2 ---- tag |  | C/G or G/G | 23 | 39,66 | 12 | 35,29 | 149 | 39,52 | 88 | 32,59 | 0.84 (0.38-1.85) | 0.67 | 1.12 (0.66-1.92) | 0.67 | . | . | . |
| SHMT1 ---- candidate | rs1979277 | G/G | 32 | 55,17 | 18 | 52,94 | 166 | 44,03 | 135 | 50,00 | 1.00 (.-.) | . | 1.23 (0.72-2.10) | 0.45 | 0.94 | 0.99 | 0.93 |
| SHMT1 ---- candidate |  | G/A or A/A | 26 | 44,83 | 16 | 47,06 | 211 | 55,97 | 135 | 50,00 | 0.79 (0.37-1.68) | 0.54 | 1.00 (0.58-1.72) | 1.00 | . | . | . |
| SHMT1 ---- tag | rs2168781 | G/G | 25 | 43,10 | 14 | 41,18 | 116 | 30,77 | 99 | 36,67 | 1.00 (.-.) | . | 1.22 (0.67-2.23) | 0.51 | 0.93 | 0.99 | 0.93 |
| SHMT1 ---- tag |  | G/C or C/C | 33 | 56,90 | 20 | 58,82 | 261 | 69,23 | 171 | 63,33 | 0.87 (0.41-1.85) | 0.71 | 1.10 (0.61-1.98) | 0.76 | . | . | . |
| SHMT1 ---- tag | rs4924849 | C/C | 32 | 55,17 | 18 | 52,94 | 176 | 46,68 | 142 | 52,59 | 1.00 (.-.) | . | 1.20 (0.70-2.06) | 0.50 | 0.87 | 0.99 | 0.93 |
| SHMT1 ---- tag |  | C/T or T/T | 26 | 44,83 | 16 | 47,06 | 201 | 53,32 | 128 | 47,41 | 0.79 (0.37-1.69) | 0.55 | 1.02 (0.59-1.76) | 0.95 | . | . | . |
| SHMT1 ---- candidate literature | rs9909104 | T/T | 23 | 39,66 | 19 | 55,88 | 200 | 53,05 | 149 | 55,19 | 1.00 (.-.) | . | 0.93 (0.54-1.62) | 0.81 | 0.19 | 0.99 | 0.93 |
| SHMT1 ---- candidate literature |  | T/C or C/C | 35 | 60,34 | 15 | 44,12 | 177 | 46,95 | 121 | 44,81 | 0.57 (0.27-1.22) | 0.15 | 0.91 (0.52-1.60) | 0.75 | . | . | . |
| SHMT2 ---- tag | rs10876968 | G/G | 28 | 48,28 | 18 | 52,94 | 199 | 52,79 | 148 | 54,81 | 1.00 (.-.) | . | 1.13 (0.66-1.96) | 0.65 | 0.62 | 0.99 | 0.81 |
| SHMT2 ---- tag |  | G/T or T/T | 30 | 51,72 | 16 | 47,06 | 178 | 47,21 | 122 | 45,19 | 0.86 (0.40-1.85) | 0.70 | 1.20 (0.69-2.09) | 0.53 | . | . | . |
| SHMT2 ---- tag | rs1800165 | T/T | 26 | 44,83 | 18 | 52,94 | 185 | 49,07 | 128 | 47,41 | 1.00 (.-.) | . | 1.17 (0.67-2.04) | 0.59 | 0.77 | 0.83 | 0.38 |
| SHMT2 ---- tag |  | T/C or C/C | 32 | 55,17 | 16 | 47,06 | 192 | 50,93 | 142 | 52,59 | 0.97 (0.45-2.06) | 0.93 | 1.27 (0.73-2.21) | 0.39 | . | . | . |
| SHMT2 ---- tag | rs7133939 | T/T | 12 | 20,69 | 11 | 32,35 | 114 | 30,24 | 86 | 31,85 | 1.00 (.-.) | . | 1.08 (0.56-2.08) | 0.82 | 0.60 | 0.99 | 0.81 |
| SHMT2 ---- tag |  | T/A or A/A | 46 | 79,31 | 23 | 67,65 | 263 | 69,76 | 184 | 68,15 | 0.84 (0.38-1.84) | 0.67 | 1.13 (0.60-2.14) | 0.70 | . | . | . |
| SHMT2 ---- tag | rs7485577 | G/G | 31 | 53,45 | 19 | 55,88 | 208 | 55,17 | 139 | 51,48 | 1.00 (.-.) | . | 1.27 (0.74-2.17) | 0.39 | 0.86 | 0.85 | 0.38 |
| SHMT2 ---- tag |  | G/A or A/A | 27 | 46,55 | 15 | 44,12 | 169 | 44,83 | 131 | 48,52 | 1.19 (0.55-2.55) | 0.66 | 1.40 (0.82-2.40) | 0.22 | . | . | . |
| SHMT2 ---- tag | rs7489231 | T/T | 23 | 39,66 | 16 | 47,06 | 168 | 44,56 | 120 | 44,44 | 1.00 (.-.) | . | 1.21 (0.69-2.12) | 0.51 | 0.94 | 0.79 | 0.38 |
| SHMT2 ---- tag |  | T/C or C/C | 35 | 60,34 | 18 | 52,94 | 209 | 55,44 | 150 | 55,56 | 1.07 (0.50-2.28) | 0.87 | 1.33 (0.77-2.32) | 0.31 | . | . | . |
| SLC19A1 ---- candidate | rs1051266 | G/G | 15 | 25,86 | 12 | 35,29 | 120 | 31,83 | 76 | 28,15 | 1.00 (.-.) | . | 0.80 (0.42-1.54) | 0.51 | 0.12 | 0.85 | 0.33 |
| SLC19A1 ---- candidate |  | G/A or A/A | 43 | 74,14 | 22 | 64,71 | 257 | 68,17 | 194 | 71,85 | 0.52 (0.24-1.11) | 0.09 | 0.81 (0.43-1.50) | 0.49 | . | . | . |
| SLC19A1 ---- candidate literature | rs1131596 | T/T | 16 | 27,59 | 12 | 35,29 | 120 | 31,83 | 76 | 28,15 | 1.00 (.-.) | . | 0.86 (0.45-1.63) | 0.64 | 0.18 | 0.86 | 0.33 |
| SLC19A1 ---- candidate literature |  | T/C or C/C | 42 | 72,41 | 22 | 64,71 | 257 | 68,17 | 194 | 71,85 | 0.57 (0.26-1.21) | 0.14 | 0.86 (0.46-1.59) | 0.63 | . | . | . |
| SLC19A1 ---- tag | rs12483553 | G/G | 46 | 79,31 | 25 | 73,53 | 304 | 80,64 | 217 | 80,37 | 1.00 (.-.) | . | 1.42 (0.87-2.32) | 0.16 | 0.26 | 0.83 | 0.33 |
| SLC19A1 ---- tag |  | G/A or A/A | 12 | 20,69 | 9 | 26,47 | 73 | 19,36 | 53 | 19,63 | 1.87 (0.83-4.22) | 0.13 | 1.58 (0.91-2.74) | 0.10 | . | . | . |
| SLC19A1 ---- candidate literature | rs12659 | C/C | 15 | 25,86 | 12 | 35,29 | 125 | 33,16 | 81 | 30,00 | 1.00 (.-.) | . | 0.82 (0.43-1.56) | 0.54 | 0.13 | 0.85 | 0.33 |
| SLC19A1 ---- candidate literature |  | C/T or T/T | 43 | 74,14 | 22 | 64,71 | 252 | 66,84 | 189 | 70,00 | 0.52 (0.24-1.11) | 0.09 | 0.80 (0.43-1.49) | 0.48 | . | . | . |
| SLC19A1 ---- tag | rs3788190 | G/G | 14 | 24,14 | 12 | 35,29 | 120 | 31,83 | 77 | 28,52 | 1.00 (.-.) | . | 0.78 (0.41-1.48) | 0.44 | 0.10 | 0.88 | 0.33 |
| SLC19A1 ---- tag |  | G/A or A/A | 44 | 75,86 | 22 | 64,71 | 257 | 68,17 | 193 | 71,48 | 0.47 (0.22-1.02) | 0.06 | 0.75 (0.40-1.39) | 0.36 | . | . | . |
| SLC19A1 ---- tag | rs3788205 | C/C | 24 | 41,38 | 19 | 55,88 | 188 | 49,87 | 150 | 55,56 | 1.00 (.-.) | . | 1.11 (0.64-1.92) | 0.71 | 0.58 | 0.93 | 0.46 |
| SLC19A1 ---- tag |  | C/T or T/T | 34 | 58,62 | 15 | 44,12 | 189 | 50,13 | 120 | 44,44 | 0.79 (0.37-1.68) | 0.54 | 1.10 (0.62-1.93) | 0.75 | . | . | . |
| SLC19A1 ---- tag | rs7279664 | G/G | 20 | 34,48 | 15 | 44,12 | 148 | 39,26 | 114 | 42,22 | 1.00 (.-.) | . | 0.91 (0.50-1.66) | 0.75 | 0.23 | 0.82 | 0.33 |
| SLC19A1 ---- tag |  | G/T or T/T | 38 | 65,52 | 19 | 55,88 | 229 | 60,74 | 156 | 57,78 | 0.50 (0.23-1.09) | 0.08 | 0.75 (0.42-1.36) | 0.35 | . | . | . |
| SLC29A1 ---- NA | rs1057985 | C/C | 20 | 34,48 | 11 | 32,35 | 166 | 44,03 | 127 | 47,04 | 1.00 (.-.) | . | 1.60 (0.77-3.34) | 0.21 | 0.38 | 0.83 | 0.31 |
| SLC29A1 ---- NA |  | C/T or T/T | 38 | 65,52 | 23 | 67,65 | 211 | 55,97 | 143 | 52,96 | 1.46 (0.64-3.35) | 0.37 | 1.60 (0.77-3.33) | 0.21 | . | . | . |
| SLC29A1 ---- NA | rs6458375 | C/C | 37 | 63,79 | 16 | 47,06 | 198 | 52,52 | 154 | 57,04 | 1.00 (.-.) | . | 1.45 (0.80-2.62) | 0.22 | 0.43 | 0.59 | 0.18 |
| SLC29A1 ---- NA |  | C/T or T/T | 21 | 36,21 | 18 | 52,94 | 179 | 47,48 | 116 | 42,96 | 1.16 (0.54-2.50) | 0.70 | 1.21 (0.66-2.22) | 0.53 | . | . | . |
| SLC29A1 ---- NA | rs666462 | C/C | 16 | 27,59 | 9 | 26,47 | 95 | 25,20 | 80 | 29,63 | 1.00 (.-.) | . | 1.40 (0.66-2.96) | 0.38 | 0.71 | 0.99 | 0.90 |
| SLC29A1 ---- NA |  | C/T or T/T | 42 | 72,41 | 25 | 73,53 | 282 | 74,80 | 190 | 70,37 | 1.14 (0.49-2.66) | 0.77 | 1.34 (0.65-2.76) | 0.43 | . | . | . |
| SLC29A1 ---- NA | rs6905285 | A/A | 17 | 29,31 | 12 | 35,29 | 155 | 41,11 | 94 | 34,81 | 1.00 (.-.) | . | 1.08 (0.52-2.21) | 0.84 | 0.65 | 0.94 | 0.65 |
| SLC29A1 ---- NA |  | A/T or T/T | 41 | 70,69 | 22 | 64,71 | 222 | 58,89 | 176 | 65,19 | 0.82 (0.36-1.85) | 0.63 | 1.08 (0.53-2.18) | 0.83 | . | . | . |
| SLC29A1 ---- NA | rs693955 | G/G | 35 | 60,34 | 20 | 58,82 | 248 | 65,78 | 183 | 67,78 | 1.00 (.-.) | . | 1.34 (0.79-2.29) | 0.28 | 0.69 | 0.63 | 0.18 |
| SLC29A1 ---- NA |  | G/T or T/T | 23 | 39,66 | 14 | 41,18 | 129 | 34,22 | 87 | 32,22 | 1.30 (0.61-2.80) | 0.50 | 1.48 (0.84-2.59) | 0.17 | . | . | . |
| SLC29A1 ---- NA | rs747199 | C/C | 34 | 58,62 | 26 | 76,47 | 247 | 65,52 | 177 | 65,56 | 1.00 (.-.) | . | 1.02 (0.63-1.63) | 0.95 | 0.13 | 0.99 | 0.68 |
| SLC29A1 ---- NA |  | C/G or G/G | 24 | 41,38 | 8 | 23,53 | 130 | 34,48 | 93 | 34,44 | 0.53 (0.21-1.32) | 0.17 | 1.08 (0.66-1.76) | 0.75 | . | . | . |
| SLC29A1 ---- NA | rs9357436 | G/G | 38 | 65,52 | 25 | 73,53 | 272 | 72,15 | 197 | 72,96 | 1.00 (.-.) | . | 1.16 (0.72-1.89) | 0.54 | 0.63 | 0.86 | 0.43 |
| SLC29A1 ---- NA |  | G/A or A/A | 20 | 34,48 | 9 | 26,47 | 105 | 27,85 | 73 | 27,04 | 0.81 (0.35-1.86) | 0.62 | 1.16 (0.69-1.96) | 0.57 | . | . | . |
| TCN2 ---- tag | rs10418 | C/C | 34 | 58,62 | 15 | 44,12 | 218 | 57,82 | 156 | 57,78 | 1.00 (.-.) | . | 1.57 (0.85-2.89) | 0.15 | 0.28 | 0.99 | 0.92 |
| TCN2 ---- tag |  | C/T or T/T | 24 | 41,38 | 19 | 55,88 | 159 | 42,18 | 114 | 42,22 | 1.63 (0.75-3.51) | 0.22 | 1.63 (0.88-3.04) | 0.12 | . | . | . |
| TCN2 ---- candidate/singleton | rs1131603 | T/T | 55 | 94,83 | 29 | 85,29 | 329 | 87,27 | 240 | 88,89 | 1.00 (.-.) | . | 1.39 (0.89-2.17) | 0.14 | 0.14 | 0.99 | 0.91 |
| TCN2 ---- candidate/singleton |  | T/C or C/C | 3 | 5,17 | 5 | 14,71 | 48 | 12,73 | 30 | 11,11 | 1.77 (0.60-5.20) | 0.30 | 0.96 (0.54-1.71) | 0.89 | . | . | . |
| TCN2 ---- tag | rs1544468 | A/A | 20 | 34,48 | 10 | 29,41 | 102 | 27,06 | 65 | 24,07 | 1.00 (.-.) | . | 1.40 (0.65-3.00) | 0.39 | 0.71 | 0.96 | 0.91 |
| TCN2 ---- tag |  | A/G or G/G | 38 | 65,52 | 24 | 70,59 | 275 | 72,94 | 205 | 75,93 | 1.27 (0.54-2.97) | 0.58 | 1.50 (0.71-3.16) | 0.29 | . | . | . |
| TCN2 ---- candidate/tag | rs1801198 | C/C | 12 | 20,69 | 13 | 38,24 | 115 | 30,50 | 87 | 32,22 | 1.00 (.-.) | . | 0.72 (0.37-1.39) | 0.33 | 0.07 | 0.95 | 0.91 |
| TCN2 ---- candidate/tag |  | C/G or G/G | 46 | 79,31 | 21 | 61,76 | 262 | 69,50 | 183 | 67,78 | 0.44 (0.20-0.96) | 0.04 | 0.69 (0.36-1.31) | 0.26 | . | . | . |
| TCN2 ---- tag | rs4820872 | G/G | 15 | 25,86 | 12 | 35,29 | 141 | 37,40 | 105 | 38,89 | 1.00 (.-.) | . | 1.09 (0.56-2.15) | 0.80 | 0.65 | 0.99 | 0.91 |
| TCN2 ---- tag |  | G/A or A/A | 43 | 74,14 | 22 | 64,71 | 236 | 62,60 | 165 | 61,11 | 0.83 (0.38-1.84) | 0.66 | 1.11 (0.57-2.16) | 0.77 | . | . | . |
| TCN2 ---- tag | rs4820874 | A/A | 44 | 75,86 | 24 | 70,59 | 257 | 68,17 | 201 | 74,44 | 1.00 (.-.) | . | 1.38 (0.85-2.23) | 0.19 | 0.40 | 0.93 | 0.91 |
| TCN2 ---- tag |  | A/G or G/G | 14 | 24,14 | 10 | 29,41 | 120 | 31,83 | 69 | 25,56 | 1.32 (0.57-3.07) | 0.52 | 1.24 (0.73-2.09) | 0.43 | . | . | . |
| TCN2 ---- tag | rs4820886 | T/T | 50 | 86,21 | 29 | 85,29 | 300 | 79,58 | 220 | 81,48 | 1.00 (.-.) | . | 1.28 (0.83-1.99) | 0.26 | 0.53 | 0.99 | 0.91 |
| TCN2 ---- tag |  | T/G or G/G | 8 | 13,79 | 5 | 14,71 | 77 | 20,42 | 50 | 18,52 | 1.56 (0.46-5.27) | 0.48 | 1.31 (0.79-2.18) | 0.30 | . | . | . |
| TCN2 ---- candidate | rs4820889 | G/G | 53 | 91,38 | 33 | 97,06 | 347 | 92,04 | 252 | 93,33 | 1.00 (.-.) | . | 1.17 (0.77-1.78) | 0.47 | 0.31 | 0.93 | 0.91 |
| TCN2 ---- candidate |  | G/A or A/A | 5 | 8,62 | 1 | 2,94 | 30 | 7,96 | 18 | 6,67 | 0.29 (0.04-2.15) | 0.23 | 0.87 (0.46-1.65) | 0.67 | . | . | . |
| TCN2 ---- tag | rs5997711 | C/C | 14 | 24,14 | 14 | 41,18 | 127 | 33,69 | 96 | 35,56 | 1.00 (.-.) | . | 0.72 (0.38-1.35) | 0.31 | 0.05 | 0.99 | 0.91 |
| TCN2 ---- tag |  | C/T or T/T | 44 | 75,86 | 20 | 58,82 | 250 | 66,31 | 174 | 64,44 | 0.45 (0.21-0.96) | 0.04 | 0.73 (0.40-1.35) | 0.32 | . | . | . |
| TCN2 ---- tag | rs740234 | T/T | 41 | 70,69 | 24 | 70,59 | 253 | 67,11 | 166 | 61,48 | 1.00 (.-.) | . | 1.28 (0.77-2.14) | 0.34 | 0.85 | 0.99 | 0.91 |
| TCN2 ---- tag |  | T/C or C/C | 17 | 29,31 | 10 | 29,41 | 124 | 32,89 | 104 | 38,52 | 1.13 (0.51-2.51) | 0.76 | 1.34 (0.78-2.29) | 0.29 | . | . | . |
| TCN2 ---- tag | rs740235 | G/G | 25 | 43,10 | 12 | 35,29 | 133 | 35,28 | 83 | 30,74 | 1.00 (.-.) | . | 1.39 (0.68-2.84) | 0.37 | 0.72 | 0.90 | 0.91 |
| TCN2 ---- tag |  | G/A or A/A | 33 | 56,90 | 22 | 64,71 | 244 | 64,72 | 187 | 69,26 | 1.30 (0.57-2.94) | 0.54 | 1.53 (0.75-3.12) | 0.24 | . | . | . |
| TCN2 ---- candidate/singleton | rs9606756 | A/A | 49 | 84,48 | 27 | 79,41 | 297 | 78,78 | 219 | 81,11 | 1.00 (.-.) | . | 1.29 (0.83-2.01) | 0.26 | 0.62 | 0.93 | 0.91 |
| TCN2 ---- candidate/singleton |  | A/G or G/G | 9 | 15,52 | 7 | 20,59 | 80 | 21,22 | 51 | 18,89 | 1.31 (0.44-3.85) | 0.63 | 1.26 (0.76-2.10) | 0.38 | . | . | . |
| TCN2 ---- candidate | rs9621049 | C/C | 50 | 86,21 | 29 | 85,29 | 300 | 79,58 | 220 | 81,48 | 1.00 (.-.) | . | 1.28 (0.83-1.99) | 0.26 | 0.53 | 0.99 | 0.91 |
| TCN2 ---- candidate |  | C/T or T/T | 8 | 13,79 | 5 | 14,71 | 77 | 20,42 | 50 | 18,52 | 1.56 (0.46-5.27) | 0.48 | 1.31 (0.79-2.18) | 0.30 | . | . | . |
| TK1 ---- NA | rs1065769 | G/G | 29 | 50,00 | 14 | 41,18 | 178 | 47,21 | 129 | 47,78 | 1.00 (.-.) | . | 1.05 (0.58-1.89) | 0.87 | 0.43 | 0.90 | 0.89 |
| TK1 ---- NA |  | G/A or A/A | 29 | 50,00 | 20 | 58,82 | 199 | 52,79 | 141 | 52,22 | 0.84 (0.39-1.79) | 0.65 | 1.22 (0.68-2.19) | 0.51 | . | . | . |
| TK1 ---- NA | rs12232476 | G/G | 48 | 82,76 | 26 | 76,47 | 319 | 84,62 | 225 | 83,33 | 1.00 (.-.) | . | 1.22 (0.78-1.93) | 0.39 | 0.87 | 0.99 | 0.89 |
| TK1 ---- NA |  | G/A or A/A | 10 | 17,24 | 8 | 23,53 | 58 | 15,38 | 45 | 16,67 | 0.99 (0.37-2.67) | 0.99 | 1.33 (0.78-2.27) | 0.30 | . | . | . |
| TK1 ---- tag | rs16970907 | G/G | 46 | 79,31 | 31 | 91,18 | 332 | 88,06 | 241 | 89,26 | 1.00 (.-.) | . | 1.22 (0.80-1.86) | 0.36 | 0.56 | 0.99 | 0.89 |
| TK1 ---- tag |  | G/C or C/C | 12 | 20,69 | 3 | 8,82 | 45 | 11,94 | 29 | 10,74 | 0.49 (0.07-3.65) | 0.49 | 1.06 (0.60-1.88) | 0.84 | . | . | . |
| TK1 ---- tag | rs1811086 | C/C | 54 | 93,10 | 32 | 94,12 | 359 | 95,23 | 243 | 90,00 | 1.00 (.-.) | . | 1.19 (0.77-1.83) | 0.44 | 0.46 | 0.93 | 0.89 |
| TK1 ---- tag |  | C/T or T/T | 4 | 6,90 | 2 | 5,88 | 18 | 4,77 | 27 | 10,00 | 0.95 (0.22-4.12) | 0.94 | 1.94 (1.07-3.50) | 0.03 | . | . | . |
| TK1 ---- tag | rs2292235 | C/C | 20 | 34,48 | 11 | 32,35 | 127 | 33,69 | 88 | 32,59 | 1.00 (.-.) | . | 0.78 (0.41-1.50) | 0.46 | 0.10 | 0.99 | 0.89 |
| TK1 ---- tag |  | C/A or A/A | 38 | 65,52 | 23 | 67,65 | 250 | 66,31 | 182 | 67,41 | 0.60 (0.28-1.30) | 0.19 | 0.96 (0.51-1.80) | 0.89 | . | . | . |
| TK1 ---- tag | rs2854701 | A/A | 24 | 41,38 | 12 | 35,29 | 163 | 43,24 | 100 | 37,04 | 1.00 (.-.) | . | 1.07 (0.56-2.03) | 0.84 | 0.52 | 0.82 | 0.89 |
| TK1 ---- tag |  | A/G or G/G | 34 | 58,62 | 22 | 64,71 | 214 | 56,76 | 170 | 62,96 | 1.02 (0.47-2.20) | 0.95 | 1.43 (0.76-2.69) | 0.27 | . | . | . |
| TK1 ---- tag | rs2854702 | G/G | 45 | 77,59 | 23 | 67,65 | 303 | 80,37 | 196 | 72,59 | 1.00 (.-.) | . | 1.20 (0.74-1.95) | 0.47 | 0.69 | 0.99 | 0.89 |
| TK1 ---- tag |  | G/A or A/A | 13 | 22,41 | 11 | 32,35 | 74 | 19,63 | 74 | 27,41 | 1.09 (0.47-2.52) | 0.84 | 1.56 (0.92-2.65) | 0.10 | . | . | . |
| TK1 ---- tag | rs9897765 | G/G | 36 | 62,07 | 16 | 47,06 | 199 | 52,79 | 141 | 52,22 | 1.00 (.-.) | . | 1.15 (0.64-2.06) | 0.65 | 0.66 | 0.99 | 0.89 |
| TK1 ---- tag |  | G/A or A/A | 22 | 37,93 | 18 | 52,94 | 178 | 47,21 | 129 | 47,78 | 0.95 (0.44-2.02) | 0.89 | 1.30 (0.72-2.36) | 0.38 | . | . | . |
| TYMP ---- NA | rs131815 | G/G | 24 | 41,38 | 21 | 61,76 | 206 | 54,64 | 146 | 54,07 | 1.00 (.-.) | . | 0.96 (0.56-1.65) | 0.88 | 0.18 | 0.83 | 0.20 |
| TYMP ---- NA |  | G/A or A/A | 34 | 58,62 | 13 | 38,24 | 171 | 45,36 | 124 | 45,93 | 0.78 (0.36-1.65) | 0.51 | 1.29 (0.75-2.21) | 0.35 | . | . | . |
| TYMP ---- tag | rs131816 | A/A | 38 | 65,52 | 21 | 61,76 | 230 | 61,01 | 172 | 63,70 | 1.00 (.-.) | . | 1.27 (0.75-2.14) | 0.37 | 0.88 | 0.59 | 0.14 |
| TYMP ---- tag |  | A/G or G/G | 20 | 34,48 | 13 | 38,24 | 147 | 38,99 | 98 | 36,30 | 0.84 (0.39-1.82) | 0.66 | 1.00 (0.59-1.72) | 0.99 | . | . | . |
| TYMP ---- NA | rs131817 | C/C | 15 | 25,86 | 13 | 38,24 | 120 | 31,83 | 92 | 34,07 | 1.00 (.-.) | . | 1.32 (0.65-2.70) | 0.44 | 0.81 | 0.93 | 0.42 |
| TYMP ---- NA |  | C/T or T/T | 43 | 74,14 | 21 | 61,76 | 257 | 68,17 | 178 | 65,93 | 1.25 (0.56-2.80) | 0.59 | 1.49 (0.75-2.98) | 0.26 | . | . | . |
| TYMP ---- NA | rs140521 | T/T | 36 | 62,07 | 16 | 47,06 | 203 | 53,85 | 137 | 50,74 | 1.00 (.-.) | . | 1.64 (0.89-3.02) | 0.12 | 0.19 | 0.74 | 0.20 |
| TYMP ---- NA |  | T/G or G/G | 22 | 37,93 | 18 | 52,94 | 174 | 46,15 | 133 | 49,26 | 1.56 (0.73-3.34) | 0.25 | 1.50 (0.81-2.76) | 0.20 | . | . | . |
| TYMP ---- NA | rs140522 | G/G | 29 | 50,00 | 14 | 41,18 | 175 | 46,42 | 134 | 49,63 | 1.00 (.-.) | . | 1.53 (0.85-2.76) | 0.16 | 0.32 | 0.82 | 0.20 |
| TYMP ---- NA |  | G/A or A/A | 29 | 50,00 | 20 | 58,82 | 202 | 53,58 | 136 | 50,37 | 1.20 (0.57-2.54) | 0.63 | 1.22 (0.68-2.21) | 0.50 | . | . | . |
| TYMP ---- NA | rs140524 | G/G | 38 | 65,52 | 21 | 61,76 | 251 | 66,58 | 180 | 66,67 | 1.00 (.-.) | . | 1.53 (0.92-2.53) | 0.10 | 0.12 | 0.93 | 0.42 |
| TYMP ---- NA |  | G/A or A/A | 20 | 34,48 | 13 | 38,24 | 126 | 33,42 | 90 | 33,33 | 1.70 (0.76-3.79) | 0.19 | 1.30 (0.75-2.22) | 0.35 | . | . | . |
| TYMS ---- candidate literature | rs1001761 | C/C | 17 | 29,31 | 12 | 35,29 | 110 | 29,18 | 88 | 32,59 | 1.00 (.-.) | . | 1.49 (0.74-3.00) | 0.27 | 0.53 | 0.59 | 0.09 |
| TYMS ---- candidate literature |  | C/T or T/T | 41 | 70,69 | 22 | 64,71 | 267 | 70,82 | 182 | 67,41 | 1.02 (0.46-2.28) | 0.96 | 1.16 (0.58-2.29) | 0.68 | . | . | . |
| TYMS ---- candidate literature/tag | rs10502289 | T/T | 33 | 56,90 | 26 | 76,47 | 238 | 63,13 | 174 | 64,44 | 1.00 (.-.) | . | 1.00 (0.63-1.60) | 1.00 | 0.10 | 0.85 | 0.30 |
| TYMS ---- candidate literature/tag |  | T/A or A/A | 25 | 43,10 | 8 | 23,53 | 139 | 36,87 | 96 | 35,56 | 0.42 (0.17-1.05) | 0.07 | 0.90 (0.55-1.47) | 0.67 | . | . | . |
| TYMS ---- tag | rs15872 | C/C | 28 | 48,28 | 17 | 50,00 | 182 | 48,28 | 137 | 50,74 | 1.00 (.-.) | . | 1.20 (0.67-2.16) | 0.54 | 0.93 | 0.66 | 0.13 |
| TYMS ---- tag |  | C/T or T/T | 30 | 51,72 | 17 | 50,00 | 195 | 51,72 | 133 | 49,26 | 0.77 (0.36-1.62) | 0.49 | 0.95 (0.53-1.71) | 0.87 | . | . | . |
| TYMS ---- tag | rs2244500 | T/T | 17 | 29,31 | 12 | 35,29 | 110 | 29,18 | 88 | 32,59 | 1.00 (.-.) | . | 1.49 (0.74-3.00) | 0.27 | 0.53 | 0.59 | 0.09 |
| TYMS ---- tag |  | T/C or C/C | 41 | 70,69 | 22 | 64,71 | 267 | 70,82 | 182 | 67,41 | 1.02 (0.46-2.28) | 0.96 | 1.16 (0.58-2.29) | 0.68 | . | . | . |
| TYMS ---- tag | rs2741182 | G/G | 36 | 62,07 | 21 | 61,76 | 214 | 56,76 | 171 | 63,33 | 1.00 (.-.) | . | 1.47 (0.87-2.47) | 0.15 | 0.27 | 1.00 | 0.99 |
| TYMS ---- tag |  | G/C or C/C | 22 | 37,93 | 13 | 38,24 | 163 | 43,24 | 99 | 36,67 | 1.39 (0.65-3.00) | 0.40 | 1.29 (0.75-2.21) | 0.36 | . | . | . |
| TYMS ---- candidate literature | rs2847149 | G/G | 17 | 29,31 | 12 | 35,29 | 110 | 29,18 | 88 | 32,59 | 1.00 (.-.) | . | 1.49 (0.74-3.00) | 0.27 | 0.53 | 0.59 | 0.09 |
| TYMS ---- candidate literature |  | G/A or A/A | 41 | 70,69 | 22 | 64,71 | 267 | 70,82 | 182 | 67,41 | 1.02 (0.46-2.28) | 0.96 | 1.16 (0.58-2.29) | 0.68 | . | . | . |
| TYMS ---- candidate literature | rs2853533 | G/G | 45 | 77,59 | 24 | 70,59 | 284 | 75,33 | 200 | 74,07 | 1.00 (.-.) | . | 1.24 (0.76-2.03) | 0.39 | 1.00 | 0.99 | 0.85 |
| TYMS ---- candidate literature |  | G/C or C/C | 13 | 22,41 | 10 | 29,41 | 93 | 24,67 | 70 | 25,93 | 0.94 (0.42-2.13) | 0.89 | 1.17 (0.69-1.98) | 0.57 | . | . | . |
| TYMS ---- tag | rs495139 | C/C | 20 | 34,48 | 7 | 20,59 | 149 | 39,52 | 83 | 30,74 | 1.00 (.-.) | . | 1.56 (0.61-3.96) | 0.35 | 0.67 | 0.45 | 0.01 |
| TYMS ---- tag |  | C/G or G/G | 38 | 65,52 | 27 | 79,41 | 228 | 60,48 | 187 | 69,26 | 1.79 (0.67-4.80) | 0.24 | 2.24 (0.90-5.58) | 0.08 | . | . | . |
| TYMS ---- candidate literature | rs502396 | T/T | 19 | 32,76 | 12 | 35,29 | 109 | 28,91 | 90 | 33,33 | 1.00 (.-.) | . | 1.49 (0.73-3.01) | 0.27 | 0.53 | 0.84 | 0.24 |
| TYMS ---- candidate literature |  | T/C or C/C | 39 | 67,24 | 22 | 64,71 | 268 | 71,09 | 180 | 66,67 | 1.12 (0.50-2.50) | 0.78 | 1.27 (0.64-2.51) | 0.50 | . | . | . |
| UMPH2 ---- tag | rs2291028 | A/A | 19 | 32,76 | 12 | 35,29 | 163 | 43,24 | 117 | 43,33 | 1.00 (.-.) | . | 1.38 (0.68-2.80) | 0.37 | 0.69 | 0.89 | 0.89 |
| UMPH2 ---- tag |  | A/G or G/G | 39 | 67,24 | 22 | 64,71 | 214 | 56,76 | 153 | 56,67 | 1.13 (0.50-2.57) | 0.76 | 1.32 (0.66-2.67) | 0.44 | . | . | . |
| UMPH2 ---- NA | rs4789143 | A/A | 41 | 70,69 | 27 | 79,41 | 284 | 75,33 | 220 | 81,48 | 1.00 (.-.) | . | 1.55 (0.96-2.49) | 0.07 | 0.04 | 0.99 | 0.97 |
| UMPH2 ---- NA |  | A/G or G/G | 17 | 29,31 | 7 | 20,59 | 93 | 24,67 | 50 | 18,52 | 2.06 (0.84-5.02) | 0.11 | 1.10 (0.64-1.88) | 0.74 | . | . | . |
| UMPH2 ---- NA | rs750844 | G/G | 26 | 44,83 | 13 | 38,24 | 190 | 50,40 | 143 | 52,96 | 1.00 (.-.) | . | 1.72 (0.88-3.37) | 0.11 | 0.17 | 0.99 | 0.97 |
| UMPH2 ---- NA |  | G/A or A/A | 32 | 55,17 | 21 | 61,76 | 187 | 49,60 | 127 | 47,04 | 1.50 (0.67-3.34) | 0.32 | 1.45 (0.73-2.86) | 0.28 | . | . | . |
| UMPK ---- tag | rs11582877 | C/C | 43 | 74,14 | 26 | 76,47 | 281 | 74,54 | 193 | 71,48 | 1.00 (.-.) | . | 1.11 (0.70-1.77) | 0.65 | 0.36 | 0.99 | 0.80 |
| UMPK ---- tag |  | C/T or T/T | 15 | 25,86 | 8 | 23,53 | 96 | 25,46 | 77 | 28,52 | 0.63 (0.25-1.59) | 0.33 | 1.09 (0.66-1.80) | 0.74 | . | . | . |
| UMPK ---- tag | rs2622903 | A/A | 30 | 51,72 | 16 | 47,06 | 188 | 49,87 | 128 | 47,41 | 1.00 (.-.) | . | 1.13 (0.62-2.05) | 0.69 | 0.66 | 0.96 | 0.80 |
| UMPK ---- tag |  | A/G or G/G | 28 | 48,28 | 18 | 52,94 | 189 | 50,13 | 142 | 52,59 | 0.92 (0.43-1.97) | 0.83 | 1.24 (0.69-2.25) | 0.47 | . | . | . |
| UMPK ---- tag | rs2820989 | C/C | 18 | 31,03 | 12 | 35,29 | 118 | 31,30 | 82 | 30,37 | 1.00 (.-.) | . | 0.95 (0.48-1.89) | 0.89 | 0.37 | 0.99 | 0.80 |
| UMPK ---- tag |  | C/G or G/G | 40 | 68,97 | 22 | 64,71 | 259 | 68,70 | 188 | 69,63 | 0.68 (0.31-1.52) | 0.35 | 0.96 (0.49-1.86) | 0.90 | . | . | . |
| UMPK ---- tag | rs6660321 | A/A | 44 | 75,86 | 26 | 76,47 | 289 | 76,66 | 200 | 74,07 | 1.00 (.-.) | . | 1.13 (0.71-1.79) | 0.62 | 0.41 | 0.99 | 0.92 |
| UMPK ---- tag |  | A/C or C/C | 14 | 24,14 | 8 | 23,53 | 88 | 23,34 | 70 | 25,93 | 0.64 (0.26-1.61) | 0.34 | 1.07 (0.64-1.77) | 0.80 | . | . | . |
| UMPK ---- tag | rs6690084 | T/T | 53 | 91,38 | 32 | 94,12 | 329 | 87,27 | 233 | 86,30 | 1.00 (.-.) | . | 1.12 (0.74-1.69) | 0.61 | 0.02 | 0.88 | 0.80 |
| UMPK ---- tag |  | T/C or C/C | 5 | 8,62 | 2 | 5,88 | 48 | 12,73 | 37 | 13,70 | 0.00 (0.00-2E241) | 0.97 | 1.20 (0.71-2.02) | 0.49 | . | . | . |
| UMPS ---- NA | rs1162 | A/A | 31 | 53,45 | 16 | 47,06 | 179 | 47,48 | 128 | 47,41 | 1.00 (.-.) | . | 1.04 (0.58-1.86) | 0.88 | 0.42 | 0.87 | 0.79 |
| UMPS ---- NA |  | A/G or G/G | 27 | 46,55 | 18 | 52,94 | 198 | 52,52 | 142 | 52,59 | 0.77 (0.36-1.64) | 0.50 | 1.12 (0.63-1.99) | 0.71 | . | . | . |
| UMPS ---- tag | rs13146 | C/C | 42 | 72,41 | 23 | 67,65 | 265 | 70,29 | 187 | 69,26 | 1.00 (.-.) | . | 1.08 (0.66-1.78) | 0.75 | 0.39 | 0.99 | 0.95 |
| UMPS ---- tag |  | C/T or T/T | 16 | 27,59 | 11 | 32,35 | 112 | 29,71 | 83 | 30,74 | 0.69 (0.31-1.55) | 0.37 | 1.09 (0.64-1.84) | 0.75 | . | . | . |
| UMPS ---- tag | rs16835902 | C/C | 18 | 31,03 | 17 | 50,00 | 121 | 32,10 | 88 | 32,59 | 1.00 (.-.) | . | 1.09 (0.60-1.98) | 0.77 | 0.57 | 0.99 | 0.95 |
| UMPS ---- tag |  | C/G or G/G | 40 | 68,97 | 17 | 50,00 | 256 | 67,90 | 182 | 67,41 | 0.81 (0.38-1.71) | 0.58 | 1.11 (0.63-1.97) | 0.71 | . | . | . |
| UMPS ---- tag | rs17282057 | T/T | 47 | 81,03 | 27 | 79,41 | 286 | 75,86 | 204 | 75,56 | 1.00 (.-.) | . | 1.29 (0.82-2.02) | 0.28 | 0.79 | 0.99 | 0.95 |
| UMPS ---- tag |  | T/C or C/C | 11 | 18,97 | 7 | 20,59 | 91 | 24,14 | 66 | 24,44 | 1.03 (0.39-2.75) | 0.95 | 1.15 (0.70-1.89) | 0.59 | . | . | . |
| UMPS ---- tag | rs606552 | A/A | 25 | 43,10 | 22 | 64,71 | 192 | 50,93 | 151 | 55,93 | 1.00 (.-.) | . | 1.14 (0.67-1.94) | 0.64 | 0.62 | 0.85 | 0.79 |
| UMPS ---- tag |  | A/G or G/G | 33 | 56,90 | 12 | 35,29 | 185 | 49,07 | 119 | 44,07 | 0.78 (0.36-1.69) | 0.53 | 1.09 (0.64-1.87) | 0.75 | . | . | . |
| UMPS ---- tag | rs694897 | C/C | 27 | 46,55 | 14 | 41,18 | 152 | 40,32 | 98 | 36,30 | 1.00 (.-.) | . | 1.55 (0.83-2.87) | 0.17 | 0.30 | 0.99 | 0.95 |
| UMPS ---- tag |  | C/G or G/G | 31 | 53,45 | 20 | 58,82 | 225 | 59,68 | 172 | 63,70 | 1.53 (0.71-3.30) | 0.28 | 1.53 (0.84-2.79) | 0.16 | . | . | . |
| UNG ---- NA | rs1059262 | T/T | 37 | 63,79 | 25 | 73,53 | 248 | 65,78 | 194 | 71,85 | 1.00 (.-.) | . | 1.02 (0.64-1.62) | 0.93 | 0.16 | 0.93 | 0.71 |
| UNG ---- NA |  | T/G or G/G | 21 | 36,21 | 9 | 26,47 | 129 | 34,22 | 76 | 28,15 | 0.42 (0.17-1.05) | 0.06 | 0.84 (0.51-1.39) | 0.50 | . | . | . |
| UNG ---- tag | rs2160603 | T/T | 37 | 63,79 | 27 | 79,41 | 256 | 67,90 | 182 | 67,41 | 1.00 (.-.) | . | 0.97 (0.61-1.55) | 0.91 | 0.06 | 0.85 | 0.70 |
| UNG ---- tag |  | T/C or C/C | 21 | 36,21 | 7 | 20,59 | 121 | 32,10 | 88 | 32,59 | 0.46 (0.17-1.24) | 0.13 | 1.14 (0.69-1.89) | 0.61 | . | . | . |
| UNG ---- tag | rs246079 | A/A | 14 | 24,14 | 10 | 29,41 | 112 | 29,71 | 90 | 33,33 | 1.00 (.-.) | . | 1.12 (0.55-2.25) | 0.76 | 0.72 | 0.85 | 0.70 |
| UNG ---- tag |  | A/G or G/G | 44 | 75,86 | 24 | 70,59 | 265 | 70,29 | 180 | 66,67 | 0.82 (0.37-1.82) | 0.63 | 1.07 (0.54-2.11) | 0.84 | . | . | . |
| UNG ---- NA | rs246085 | T/T | 51 | 87,93 | 24 | 70,59 | 331 | 87,80 | 242 | 89,63 | 1.00 (.-.) | . | 1.37 (0.85-2.21) | 0.20 | 0.50 | 0.99 | 0.97 |
| UNG ---- NA |  | T/C or C/C | 7 | 12,07 | 10 | 29,41 | 46 | 12,20 | 28 | 10,37 | 1.55 (0.67-3.60) | 0.31 | 1.52 (0.82-2.79) | 0.18 | . | . | . |
| UNG ---- NA | rs2569987 | A/A | 46 | 79,31 | 25 | 73,53 | 247 | 65,52 | 201 | 74,44 | 1.00 (.-.) | . | 1.70 (1.04-2.78) | 0.03 | 0.00 | 0.94 | 0.71 |
| UNG ---- NA |  | A/G or G/G | 12 | 20,69 | 9 | 26,47 | 130 | 34,48 | 69 | 25,56 | 3.11 (1.39-6.96) | 0.01 | 1.32 (0.77-2.26) | 0.32 | . | . | . |
| UNG ---- tag | rs3219243 | T/T | 33 | 56,90 | 23 | 67,65 | 247 | 65,52 | 176 | 65,19 | 1.00 (.-.) | . | 1.24 (0.77-2.01) | 0.38 | 0.90 | 0.99 | 0.97 |
| UNG ---- tag |  | T/C or C/C | 25 | 43,10 | 11 | 32,35 | 130 | 34,48 | 94 | 34,81 | 0.84 (0.35-2.03) | 0.70 | 1.11 (0.67-1.82) | 0.69 | . | . | . |

|  | | |  | | | |  | | | |  | | | | | | |
| --- | --- | --- | --- | --- | --- | --- | --- | --- | --- | --- | --- | --- | --- | --- | --- | --- | --- |
|  | | | **adchem_5fu = 0** | | | | **adchem_5fu = 1** | | | | **one reference** | | | | | | |
|  | | | **Ctrl** | | **Cases** | | **Ctrl** | | **Cases** | | **adchem_5fu = 0** | | **adchem_5fu = 1** | |  | | |
| **Gene** | **SNP** | **Genotype** | **N** | **%** | **N** | **%** | **N** | **%** | **N** | **%** | **HR (95%-CI)** | **p** | **HR (95%-CI)** | **p** | **LR_pTrend** | **FDR_pTrend** | **FDR(byGene)_pTrend** |
| AARS ---- tag | rs2070203 | T/T | 16 | 27,59 | 12 | 35,29 | 115 | 30,50 | 73 | 27,04 | 1.00 (.-.) | . | 0.85 (0.42-1.71) | 0.65 | 0.09 | 0.99 | 0.89 |
| AARS ---- tag |  | T/C | 24 | 41,38 | 17 | 50,00 | 191 | 50,66 | 137 | 50,74 | 0.86 (0.38-1.98) | 0.73 | 1.00 (0.51-1.95) | 0.99 | . | . | . |
| AARS ---- tag |  | C/C | 18 | 31,03 | 5 | 14,71 | 71 | 18,83 | 60 | 22,22 | 0.43 (0.13-1.41) | 0.16 | 1.07 (0.53-2.17) | 0.84 | . | . | . |
| AARS ---- tag | rs34087264 | G/G | 22 | 37,93 | 6 | 17,65 | 97 | 25,73 | 84 | 31,11 | 1.00 (.-.) | . | 2.43 (0.97-6.11) | 0.06 | 0.08 | 0.64 | 0.11 |
| AARS ---- tag |  | G/A | 22 | 37,93 | 18 | 52,94 | 189 | 50,13 | 130 | 48,15 | 1.85 (0.66-5.22) | 0.24 | 1.96 (0.79-4.87) | 0.15 | . | . | . |
| AARS ---- tag |  | A/A | 14 | 24,14 | 10 | 29,41 | 91 | 24,14 | 56 | 20,74 | 2.10 (0.67-6.56) | 0.20 | 1.77 (0.69-4.52) | 0.24 | . | . | . |
| ABCC4 ---- tag | rs10508023 | G/G | 48 | 82,76 | 26 | 76,47 | 298 | 79,05 | 219 | 81,11 | 1.00 (.-.) | . | 1.44 (0.91-2.29) | 0.12 | 0.24 | 0.93 | 0.81 |
| ABCC4 ---- tag |  | G/C | 9 | 15,52 | 8 | 23,53 | 75 | 19,89 | 45 | 16,67 | 2.01 (0.82-4.89) | 0.13 | 1.07 (0.62-1.84) | 0.80 | . | . | . |
| ABCC4 ---- tag |  | C/C | 1 | 1,72 | 0 | 0,00 | 4 | 1,06 | 6 | 2,22 | 0.00 (0.00-I) | 0.98 | 3.10 (1.22-7.89) | 0.02 | . | . | . |
| ABCC4 ---- tag | rs1059751 | T/T | 21 | 36,21 | 7 | 20,59 | 88 | 23,34 | 71 | 26,30 | 1.00 (.-.) | . | 1.48 (0.67-3.29) | 0.33 | 0.83 | 0.99 | 0.95 |
| ABCC4 ---- tag |  | T/C | 29 | 50,00 | 24 | 70,59 | 211 | 55,97 | 141 | 52,22 | 1.38 (0.57-3.31) | 0.47 | 1.49 (0.69-3.24) | 0.31 | . | . | . |
| ABCC4 ---- tag |  | C/C | 8 | 13,79 | 3 | 8,82 | 78 | 20,69 | 58 | 21,48 | 0.78 (0.09-6.49) | 0.82 | 1.62 (0.73-3.63) | 0.24 | . | . | . |
| ABCC4 ---- tag | rs11568643 | A/A | 50 | 86,21 | 26 | 76,47 | 320 | 84,88 | 222 | 82,22 | 1.00 (.-.) | . | 1.39 (0.87-2.21) | 0.17 | 0.64 | 0.66 | 0.60 |
| ABCC4 ---- tag |  | A/G | 8 | 13,79 | 7 | 20,59 | 57 | 15,12 | 46 | 17,04 | 3.12 (1.24-7.86) | 0.02 | 1.59 (0.92-2.75) | 0.10 | . | . | . |
| ABCC4 ---- tag |  | G/G | 0 | 0,00 | 1 | 2,94 | 0 | 0,00 | 2 | 0,74 | 0.74 (0.10-5.81) | 0.78 | 3.20 (0.40-25.29) | 0.27 | . | . | . |
| ABCC4 ---- NA | rs11568658 | G/G | 55 | 94,83 | 32 | 94,12 | 356 | 94,43 | 258 | 95,56 | 1.00 (.-.) | . | 1.30 (0.85-1.99) | 0.23 | 0.27 | 0.99 | 0.95 |
| ABCC4 ---- NA |  | G/T | 3 | 5,17 | 2 | 5,88 | 21 | 5,57 | 12 | 4,44 | 1.00 (.-.) | . | 1.30 (0.85-1.99) | 0.23 | . | . | . |
| ABCC4 ---- NA |  | T/T | 0 | 0,00 | 0 | 0,00 | 0 | 0,00 | 0 | 0,00 | 2.64 (0.61-11.37) | 0.19 | 1.27 (0.58-2.75) | 0.55 | . | . | . |
| ABCC4 ---- tag | rs12864049 | T/T | 38 | 65,52 | 26 | 76,47 | 292 | 77,45 | 200 | 74,07 | 1.00 (.-.) | . | 1.16 (0.72-1.86) | 0.54 | 0.82 | 0.99 | 0.98 |
| ABCC4 ---- tag |  | T/C | 18 | 31,03 | 7 | 20,59 | 78 | 20,69 | 66 | 24,44 | 0.72 (0.29-1.81) | 0.49 | 1.22 (0.73-2.03) | 0.46 | . | . | . |
| ABCC4 ---- tag |  | C/C | 2 | 3,45 | 1 | 2,94 | 7 | 1,86 | 4 | 1,48 | 4.76 (0.62-36.57) | 0.13 | 1.36 (0.39-4.72) | 0.63 | . | . | . |
| ABCC4 ---- tag | rs1628382 | G/G | 39 | 67,24 | 22 | 64,71 | 235 | 62,33 | 163 | 60,37 | 1.00 (.-.) | . | 1.28 (0.77-2.13) | 0.34 | 0.74 | 0.99 | 0.98 |
| ABCC4 ---- tag |  | G/A | 17 | 29,31 | 11 | 32,35 | 124 | 32,89 | 96 | 35,56 | 1.16 (0.51-2.64) | 0.72 | 1.39 (0.82-2.35) | 0.23 | . | . | . |
| ABCC4 ---- tag |  | A/A | 2 | 3,45 | 1 | 2,94 | 18 | 4,77 | 11 | 4,07 | 1.97 (0.25-15.28) | 0.52 | 1.59 (0.73-3.44) | 0.24 | . | . | . |
| ABCC4 ---- tag | rs1678354 | C/C | 18 | 31,03 | 14 | 41,18 | 169 | 44,83 | 110 | 40,74 | 1.00 (.-.) | . | 1.11 (0.58-2.10) | 0.76 | 0.72 | 0.99 | 0.98 |
| ABCC4 ---- tag |  | C/G | 34 | 58,62 | 17 | 50,00 | 163 | 43,24 | 129 | 47,78 | 0.80 (0.36-1.78) | 0.58 | 1.25 (0.66-2.35) | 0.50 | . | . | . |
| ABCC4 ---- tag |  | G/G | 6 | 10,34 | 3 | 8,82 | 45 | 11,94 | 31 | 11,48 | 3.61 (0.97-13.38) | 0.06 | 1.26 (0.62-2.57) | 0.52 | . | . | . |
| ABCC4 ---- tag | rs1678383 | T/T | 47 | 81,03 | 28 | 82,35 | 308 | 81,70 | 223 | 82,59 | 1.00 (.-.) | . | 1.16 (0.73-1.84) | 0.53 | 0.55 | 0.90 | 0.72 |
| ABCC4 ---- tag |  | T/G | 10 | 17,24 | 6 | 17,65 | 63 | 16,71 | 45 | 16,67 | 0.73 (0.27-1.94) | 0.52 | 1.17 (0.68-1.99) | 0.58 | . | . | . |
| ABCC4 ---- tag |  | G/G | 1 | 1,72 | 0 | 0,00 | 6 | 1,59 | 2 | 0,74 | 0.89 (0.22-3.63) | 0.87 | 1.03 (0.24-4.43) | 0.97 | . | . | . |
| ABCC4 ---- tag | rs1678395 | G/G | 48 | 82,76 | 29 | 85,29 | 317 | 84,08 | 230 | 85,19 | 1.00 (.-.) | . | 1.29 (0.83-2.00) | 0.25 | 0.96 | 0.88 | 0.71 |
| ABCC4 ---- tag |  | G/A | 10 | 17,24 | 5 | 14,71 | 56 | 14,85 | 39 | 14,44 | 0.75 (0.22-2.50) | 0.64 | 0.93 (0.55-1.58) | 0.80 | . | . | . |
| ABCC4 ---- tag |  | A/A | 0 | 0,00 | 0 | 0,00 | 4 | 1,06 | 1 | 0,37 | 0.54 (0.07-4.05) | 0.55 | 0.69 (0.09-5.42) | 0.73 | . | . | . |
| ABCC4 ---- tag | rs1678405 | T/T | 26 | 44,83 | 19 | 55,88 | 167 | 44,30 | 121 | 44,81 | 1.00 (.-.) | . | 1.33 (0.78-2.26) | 0.30 | 0.55 | 0.86 | 0.71 |
| ABCC4 ---- tag |  | T/C | 29 | 50,00 | 13 | 38,24 | 179 | 47,48 | 118 | 43,70 | 1.09 (0.47-2.50) | 0.84 | 1.27 (0.74-2.18) | 0.38 | . | . | . |
| ABCC4 ---- tag |  | C/C | 3 | 5,17 | 2 | 5,88 | 31 | 8,22 | 31 | 11,48 | 2.76 (0.60-12.66) | 0.19 | 1.71 (0.92-3.18) | 0.09 | . | . | . |
| ABCC4 ---- tag | rs17189540 | A/A | 52 | 89,66 | 27 | 79,41 | 335 | 88,86 | 228 | 84,44 | 1.00 (.-.) | . | 1.23 (0.78-1.94) | 0.36 | 0.80 | 0.85 | 0.70 |
| ABCC4 ---- tag |  | A/G | 6 | 10,34 | 6 | 17,65 | 42 | 11,14 | 42 | 15,56 | 1.00 (.-.) | . | 1.23 (0.78-1.94) | 0.36 | . | . | . |
| ABCC4 ---- tag |  | G/G | 0 | 0,00 | 1 | 2,94 | 0 | 0,00 | 0 | 0,00 | 1.27 (0.50-3.24) | 0.62 | 1.78 (1.03-3.06) | 0.04 | . | . | . |
| ABCC4 ---- tag | rs17235152 | T/T | 39 | 67,24 | 26 | 76,47 | 269 | 71,35 | 204 | 75,56 | 1.00 (.-.) | . | 1.19 (0.73-1.94) | 0.48 | 0.85 | 0.99 | 0.98 |
| ABCC4 ---- tag |  | T/C | 17 | 29,31 | 8 | 23,53 | 98 | 25,99 | 63 | 23,33 | 0.84 (0.36-1.93) | 0.68 | 1.10 (0.64-1.90) | 0.73 | . | . | . |
| ABCC4 ---- tag |  | C/C | 2 | 3,45 | 0 | 0,00 | 10 | 2,65 | 3 | 1,11 | 0.00 (0.00-7E252) | 0.98 | 0.71 (0.20-2.50) | 0.60 | . | . | . |
| ABCC4 ---- tag | rs17268122 | G/G | 24 | 41,38 | 22 | 64,71 | 236 | 62,60 | 184 | 68,15 | 1.00 (.-.) | . | 0.86 (0.51-1.45) | 0.56 | 0.06 | 1.00 | 0.99 |
| ABCC4 ---- tag |  | G/T | 29 | 50,00 | 11 | 32,35 | 123 | 32,63 | 72 | 26,67 | 0.49 (0.21-1.11) | 0.09 | 0.82 (0.46-1.46) | 0.51 | . | . | . |
| ABCC4 ---- tag |  | T/T | 5 | 8,62 | 1 | 2,94 | 18 | 4,77 | 14 | 5,19 | 0.23 (0.03-1.74) | 0.15 | 0.70 (0.33-1.50) | 0.36 | . | . | . |
| ABCC4 ---- tag | rs17268170 | C/C | 47 | 81,03 | 24 | 70,59 | 314 | 83,29 | 216 | 80,00 | 1.00 (.-.) | . | 1.34 (0.83-2.18) | 0.23 | 0.56 | 0.99 | 0.98 |
| ABCC4 ---- tag |  | C/T | 11 | 18,97 | 6 | 17,65 | 62 | 16,45 | 52 | 19,26 | 1.38 (0.54-3.55) | 0.50 | 1.52 (0.88-2.64) | 0.13 | . | . | . |
| ABCC4 ---- tag |  | T/T | 0 | 0,00 | 4 | 11,76 | 1 | 0,27 | 2 | 0,74 | 2.03 (0.55-7.54) | 0.29 | 2.30 (0.52-10.06) | 0.27 | . | . | . |
| ABCC4 ---- tag | rs1729764 | A/A | 43 | 74,14 | 28 | 82,35 | 287 | 76,13 | 218 | 80,74 | 1.00 (.-.) | . | 1.21 (0.79-1.87) | 0.38 | 0.45 | 0.85 | 0.70 |
| ABCC4 ---- tag |  | A/G | 14 | 24,14 | 4 | 11,76 | 86 | 22,81 | 47 | 17,41 | 0.56 (0.13-2.42) | 0.44 | 0.95 (0.57-1.60) | 0.85 | . | . | . |
| ABCC4 ---- tag |  | G/G | 1 | 1,72 | 2 | 5,88 | 4 | 1,06 | 5 | 1,85 | 0.00 (0.00-1E233) | 0.97 | 1.42 (0.52-3.91) | 0.49 | . | . | . |
| ABCC4 ---- tag | rs1729767 | T/T | 28 | 48,28 | 15 | 44,12 | 202 | 53,58 | 137 | 50,74 | 1.00 (.-.) | . | 1.36 (0.76-2.44) | 0.30 | 0.37 | 0.95 | 0.91 |
| ABCC4 ---- tag |  | T/C | 25 | 43,10 | 17 | 50,00 | 153 | 40,58 | 119 | 44,07 | 1.14 (0.52-2.49) | 0.75 | 1.49 (0.82-2.69) | 0.19 | . | . | . |
| ABCC4 ---- tag |  | C/C | 5 | 8,62 | 2 | 5,88 | 22 | 5,84 | 14 | 5,19 | 3.22 (0.70-14.74) | 0.13 | 1.12 (0.51-2.46) | 0.78 | . | . | . |
| ABCC4 ---- tag | rs17300935 | C/C | 40 | 68,97 | 21 | 61,76 | 278 | 73,74 | 207 | 76,67 | 1.00 (.-.) | . | 1.25 (0.75-2.08) | 0.39 | 0.87 | 0.99 | 0.95 |
| ABCC4 ---- tag |  | C/G | 16 | 27,59 | 13 | 38,24 | 91 | 24,14 | 61 | 22,59 | 1.02 (0.47-2.26) | 0.95 | 1.26 (0.72-2.21) | 0.42 | . | . | . |
| ABCC4 ---- tag |  | G/G | 2 | 3,45 | 0 | 0,00 | 8 | 2,12 | 2 | 0,74 | 0.00 (0.00-1E253) | 0.98 | 0.55 (0.12-2.44) | 0.43 | . | . | . |
| ABCC4 ---- tag | rs1750190 | G/G | 19 | 32,76 | 7 | 20,59 | 101 | 26,79 | 62 | 22,96 | 1.00 (.-.) | . | 1.77 (0.69-4.49) | 0.23 | 0.74 | 0.83 | 0.70 |
| ABCC4 ---- tag |  | G/A | 27 | 46,55 | 19 | 55,88 | 186 | 49,34 | 138 | 51,11 | 2.45 (0.86-6.97) | 0.09 | 2.36 (0.95-5.87) | 0.07 | . | . | . |
| ABCC4 ---- tag |  | A/A | 12 | 20,69 | 8 | 23,53 | 90 | 23,87 | 70 | 25,93 | 1.72 (0.53-5.57) | 0.36 | 2.26 (0.89-5.72) | 0.08 | . | . | . |
| ABCC4 ---- tag | rs1750996 | A/A | 33 | 56,90 | 21 | 61,76 | 247 | 65,52 | 178 | 65,93 | 1.00 (.-.) | . | 1.25 (0.76-2.05) | 0.38 | 0.89 | 0.92 | 0.72 |
| ABCC4 ---- tag |  | A/G | 22 | 37,93 | 11 | 32,35 | 122 | 32,36 | 83 | 30,74 | 1.01 (0.45-2.27) | 0.98 | 1.17 (0.69-1.99) | 0.56 | . | . | . |
| ABCC4 ---- tag |  | G/G | 3 | 5,17 | 2 | 5,88 | 8 | 2,12 | 9 | 3,33 | 0.00 (0.00-7E304) | 0.98 | 1.35 (0.58-3.12) | 0.49 | . | . | . |
| ABCC4 ---- tag | rs1751025 | C/C | 32 | 55,17 | 13 | 38,24 | 181 | 48,01 | 120 | 44,44 | 1.00 (.-.) | . | 1.71 (0.91-3.21) | 0.10 | 0.21 | 0.66 | 0.60 |
| ABCC4 ---- tag |  | C/G | 21 | 36,21 | 15 | 44,12 | 166 | 44,03 | 114 | 42,22 | 2.29 (0.98-5.34) | 0.05 | 1.89 (1.00-3.57) | 0.05 | . | . | . |
| ABCC4 ---- tag |  | G/G | 5 | 8,62 | 6 | 17,65 | 30 | 7,96 | 36 | 13,33 | 2.67 (0.88-8.12) | 0.08 | 2.71 (1.36-5.42) | 0.01 | . | . | . |
| ABCC4 ---- tag | rs1751051 | T/T | 25 | 43,10 | 15 | 44,12 | 151 | 40,05 | 121 | 44,81 | 1.00 (.-.) | . | 1.12 (0.63-2.00) | 0.70 | 0.49 | 0.99 | 0.98 |
| ABCC4 ---- tag |  | T/A | 23 | 39,66 | 15 | 44,12 | 177 | 46,95 | 118 | 43,70 | 0.76 (0.33-1.72) | 0.51 | 0.96 (0.54-1.71) | 0.88 | . | . | . |
| ABCC4 ---- tag |  | A/A | 10 | 17,24 | 4 | 11,76 | 49 | 13,00 | 31 | 11,48 | 0.62 (0.17-2.24) | 0.46 | 1.16 (0.59-2.26) | 0.67 | . | . | . |
| ABCC4 ---- tag | rs1764416 | G/G | 50 | 86,21 | 34 | 100,00 | 320 | 84,88 | 237 | 87,78 | 1.00 (.-.) | . | 1.23 (0.81-1.86) | 0.34 | 0.17 | 0.90 | 0.72 |
| ABCC4 ---- tag |  | G/A | 8 | 13,79 | 0 | 0,00 | 56 | 14,85 | 33 | 12,22 | 1.00 (.-.) | . | 1.23 (0.81-1.86) | 0.34 | . | . | . |
| ABCC4 ---- tag |  | A/A | 0 | 0,00 | 0 | 0,00 | 1 | 0,27 | 0 | 0,00 | 0.00 (0.00-1E286) | 0.97 | 0.94 (0.54-1.65) | 0.83 | . | . | . |
| ABCC4 ---- tag | rs2274401 | T/T | 35 | 60,34 | 23 | 67,65 | 217 | 57,56 | 184 | 68,15 | 1.00 (.-.) | . | 1.17 (0.73-1.85) | 0.52 | 0.37 | 0.87 | 0.71 |
| ABCC4 ---- tag |  | T/C | 21 | 36,21 | 11 | 32,35 | 139 | 36,87 | 76 | 28,15 | 0.79 (0.32-1.96) | 0.60 | 0.97 (0.59-1.60) | 0.92 | . | . | . |
| ABCC4 ---- tag |  | C/C | 2 | 3,45 | 0 | 0,00 | 21 | 5,57 | 10 | 3,70 | 0.00 (0.00-4E231) | 0.97 | 0.95 (0.42-2.13) | 0.90 | . | . | . |
| ABCC4 ---- tag | rs2892716 | C/C | 26 | 44,83 | 17 | 50,00 | 124 | 32,89 | 107 | 39,63 | 1.00 (.-.) | . | 1.72 (0.98-3.03) | 0.06 | 0.24 | 0.99 | 0.98 |
| ABCC4 ---- tag |  | C/T | 28 | 48,28 | 17 | 50,00 | 185 | 49,07 | 131 | 48,52 | 1.88 (0.87-4.08) | 0.11 | 1.47 (0.83-2.61) | 0.18 | . | . | . |
| ABCC4 ---- tag |  | T/T | 4 | 6,90 | 0 | 0,00 | 68 | 18,04 | 32 | 11,85 | 0.00 (0.00-1E234) | 0.97 | 1.34 (0.71-2.54) | 0.37 | . | . | . |
| ABCC4 ---- tag | rs3782964 | C/C | 43 | 74,14 | 24 | 70,59 | 242 | 64,19 | 191 | 70,74 | 1.00 (.-.) | . | 1.35 (0.84-2.18) | 0.22 | 0.44 | 0.85 | 0.70 |
| ABCC4 ---- tag |  | C/T | 14 | 24,14 | 8 | 23,53 | 126 | 33,42 | 68 | 25,19 | 1.08 (0.45-2.58) | 0.86 | 1.07 (0.63-1.81) | 0.81 | . | . | . |
| ABCC4 ---- tag |  | T/T | 1 | 1,72 | 2 | 5,88 | 9 | 2,39 | 11 | 4,07 | 2.36 (0.31-18.14) | 0.41 | 2.24 (0.89-5.66) | 0.09 | . | . | . |
| ABCC4 ---- tag | rs3818494 | C/C | 33 | 56,90 | 18 | 52,94 | 170 | 45,09 | 111 | 41,11 | 1.00 (.-.) | . | 1.31 (0.76-2.28) | 0.34 | 0.82 | 0.59 | 0.60 |
| ABCC4 ---- tag |  | C/G | 20 | 34,48 | 14 | 41,18 | 166 | 44,03 | 120 | 44,44 | 1.60 (0.73-3.50) | 0.24 | 1.50 (0.87-2.61) | 0.15 | . | . | . |
| ABCC4 ---- tag |  | G/G | 5 | 8,62 | 2 | 5,88 | 41 | 10,88 | 39 | 14,44 | 0.87 (0.11-6.76) | 0.90 | 1.81 (0.98-3.35) | 0.06 | . | . | . |
| ABCC4 ---- tag | rs3864997 | G/G | 21 | 36,21 | 11 | 32,35 | 92 | 24,40 | 63 | 23,33 | 1.00 (.-.) | . | 1.76 (0.86-3.60) | 0.13 | 0.08 | 0.85 | 0.70 |
| ABCC4 ---- tag |  | G/T | 30 | 51,72 | 17 | 50,00 | 201 | 53,32 | 142 | 52,59 | 1.60 (0.69-3.71) | 0.28 | 1.86 (0.93-3.71) | 0.08 | . | . | . |
| ABCC4 ---- tag |  | T/T | 7 | 12,07 | 6 | 17,65 | 84 | 22,28 | 65 | 24,07 | 2.87 (0.94-8.75) | 0.06 | 1.68 (0.82-3.45) | 0.16 | . | . | . |
| ABCC4 ---- tag | rs4148421 | G/G | 13 | 22,41 | 6 | 17,65 | 118 | 31,30 | 78 | 28,89 | 1.00 (.-.) | . | 1.38 (0.49-3.85) | 0.54 | 0.85 | 0.99 | 0.98 |
| ABCC4 ---- tag |  | G/A | 36 | 62,07 | 18 | 52,94 | 189 | 50,13 | 142 | 52,59 | 1.15 (0.38-3.52) | 0.80 | 1.44 (0.52-4.01) | 0.49 | . | . | . |
| ABCC4 ---- tag |  | A/A | 9 | 15,52 | 10 | 29,41 | 70 | 18,57 | 50 | 18,52 | 1.22 (0.35-4.22) | 0.76 | 1.44 (0.51-4.11) | 0.49 | . | . | . |
| ABCC4 ---- tag | rs4148446 | G/G | 24 | 41,38 | 14 | 41,18 | 114 | 30,24 | 94 | 34,81 | 1.00 (.-.) | . | 1.58 (0.84-2.95) | 0.16 | 0.40 | 0.99 | 0.98 |
| ABCC4 ---- tag |  | G/A | 28 | 48,28 | 18 | 52,94 | 186 | 49,34 | 130 | 48,15 | 1.34 (0.62-2.93) | 0.46 | 1.39 (0.74-2.60) | 0.30 | . | . | . |
| ABCC4 ---- tag |  | A/A | 6 | 10,34 | 2 | 5,88 | 77 | 20,42 | 46 | 17,04 | 1.11 (0.14-8.62) | 0.92 | 1.35 (0.70-2.63) | 0.37 | . | . | . |
| ABCC4 ---- tag | rs4148455 | G/G | 43 | 74,14 | 27 | 79,41 | 285 | 75,60 | 202 | 74,81 | 1.00 (.-.) | . | 1.22 (0.76-1.96) | 0.41 | 0.66 | 0.99 | 0.95 |
| ABCC4 ---- tag |  | G/A | 14 | 24,14 | 6 | 17,65 | 83 | 22,02 | 66 | 24,44 | 0.90 (0.36-2.25) | 0.82 | 1.34 (0.79-2.26) | 0.28 | . | . | . |
| ABCC4 ---- tag |  | A/A | 1 | 1,72 | 1 | 2,94 | 9 | 2,39 | 2 | 0,74 | 5.03 (0.63-40.15) | 0.13 | 0.49 (0.11-2.14) | 0.34 | . | . | . |
| ABCC4 ---- tag | rs4148540 | C/C | 50 | 86,21 | 30 | 88,24 | 331 | 87,80 | 230 | 85,19 | 1.00 (.-.) | . | 1.24 (0.80-1.92) | 0.33 | 0.88 | 0.99 | 0.95 |
| ABCC4 ---- tag |  | C/T | 8 | 13,79 | 4 | 11,76 | 44 | 11,67 | 37 | 13,70 | 0.89 (0.26-2.97) | 0.84 | 1.15 (0.67-1.96) | 0.61 | . | . | . |
| ABCC4 ---- tag |  | T/T | 0 | 0,00 | 0 | 0,00 | 2 | 0,53 | 3 | 1,11 | 1.27 (0.39-4.21) | 0.69 | 1.58 (0.45-5.58) | 0.48 | . | . | . |
| ABCC4 ---- tag | rs4148542 | G/G | 12 | 20,69 | 3 | 8,82 | 105 | 27,85 | 68 | 25,19 | 1.00 (.-.) | . | 2.73 (0.37-20.02) | 0.32 | 0.55 | 0.99 | 0.98 |
| ABCC4 ---- tag |  | G/A | 27 | 46,55 | 24 | 70,59 | 198 | 52,52 | 141 | 52,22 | 2.35 (0.31-17.79) | 0.41 | 2.87 (0.39-20.84) | 0.30 | . | . | . |
| ABCC4 ---- tag |  | A/A | 19 | 32,76 | 7 | 20,59 | 74 | 19,63 | 61 | 22,59 | 2.45 (0.30-20.15) | 0.41 | 2.74 (0.37-20.12) | 0.32 | . | . | . |
| ABCC4 ---- tag | rs4148544 | G/G | 23 | 39,66 | 9 | 26,47 | 157 | 41,64 | 119 | 44,07 | 1.00 (.-.) | . | 1.73 (0.74-4.05) | 0.20 | 0.44 | 0.99 | 0.98 |
| ABCC4 ---- tag |  | G/A | 25 | 43,10 | 21 | 61,76 | 185 | 49,07 | 114 | 42,22 | 1.58 (0.62-4.04) | 0.34 | 1.77 (0.76-4.14) | 0.19 | . | . | . |
| ABCC4 ---- tag |  | A/A | 10 | 17,24 | 4 | 11,76 | 35 | 9,28 | 37 | 13,70 | 1.47 (0.41-5.34) | 0.56 | 1.62 (0.65-4.01) | 0.30 | . | . | . |
| ABCC4 ---- tag | rs4283094 | C/C | 9 | 15,52 | 6 | 17,65 | 102 | 27,06 | 61 | 22,59 | 1.00 (.-.) | . | 0.72 (0.27-1.87) | 0.50 | 0.10 | 0.99 | 0.98 |
| ABCC4 ---- tag |  | C/G | 32 | 55,17 | 17 | 50,00 | 196 | 51,99 | 145 | 53,70 | 0.78 (0.27-2.24) | 0.65 | 0.88 (0.34-2.26) | 0.80 | . | . | . |
| ABCC4 ---- tag |  | G/G | 17 | 29,31 | 11 | 32,35 | 79 | 20,95 | 64 | 23,70 | 0.48 (0.16-1.47) | 0.20 | 0.89 (0.34-2.32) | 0.81 | . | . | . |
| ABCC4 ---- tag | rs4636781 | A/A | 45 | 77,59 | 27 | 79,41 | 269 | 71,35 | 187 | 69,26 | 1.00 (.-.) | . | 1.24 (0.77-2.01) | 0.37 | 0.75 | 0.83 | 0.70 |
| ABCC4 ---- tag |  | A/G | 13 | 22,41 | 6 | 17,65 | 97 | 25,73 | 75 | 27,78 | 1.04 (0.41-2.61) | 0.94 | 1.37 (0.81-2.29) | 0.24 | . | . | . |
| ABCC4 ---- tag |  | G/G | 0 | 0,00 | 1 | 2,94 | 11 | 2,92 | 8 | 2,96 | 5.04 (0.63-40.22) | 0.13 | 1.94 (0.81-4.63) | 0.13 | . | . | . |
| ABCC4 ---- tag | rs4771910 | T/T | 30 | 51,72 | 16 | 47,06 | 178 | 47,21 | 129 | 47,78 | 1.00 (.-.) | . | 1.54 (0.82-2.91) | 0.18 | 0.44 | 0.83 | 0.70 |
| ABCC4 ---- tag |  | T/C | 25 | 43,10 | 15 | 44,12 | 166 | 44,03 | 115 | 42,59 | 1.28 (0.58-2.83) | 0.54 | 1.34 (0.71-2.53) | 0.37 | . | . | . |
| ABCC4 ---- tag |  | C/C | 3 | 5,17 | 3 | 8,82 | 33 | 8,75 | 26 | 9,63 | 1.05 (0.22-4.94) | 0.95 | 1.23 (0.58-2.58) | 0.59 | . | . | . |
| ABCC4 ---- tag | rs4773850 | T/T | 26 | 44,83 | 18 | 52,94 | 165 | 43,77 | 142 | 52,59 | 1.00 (.-.) | . | 1.55 (0.89-2.69) | 0.12 | 0.26 | 0.59 | 0.60 |
| ABCC4 ---- tag |  | T/G | 29 | 50,00 | 12 | 35,29 | 167 | 44,30 | 99 | 36,67 | 1.07 (0.48-2.42) | 0.87 | 1.13 (0.64-1.98) | 0.67 | . | . | . |
| ABCC4 ---- tag |  | G/G | 3 | 5,17 | 4 | 11,76 | 45 | 11,94 | 29 | 10,74 | 1.27 (0.36-4.53) | 0.72 | 1.04 (0.53-2.03) | 0.91 | . | . | . |
| ABCC4 ---- tag | rs7981095 | A/A | 39 | 67,24 | 20 | 58,82 | 248 | 65,78 | 171 | 63,33 | 1.00 (.-.) | . | 1.26 (0.75-2.11) | 0.39 | 0.85 | 0.99 | 0.95 |
| ABCC4 ---- tag |  | A/T | 18 | 31,03 | 10 | 29,41 | 119 | 31,56 | 88 | 32,59 | 0.99 (0.44-2.22) | 0.98 | 1.23 (0.71-2.12) | 0.46 | . | . | . |
| ABCC4 ---- tag |  | T/T | 1 | 1,72 | 4 | 11,76 | 10 | 2,65 | 11 | 4,07 | 1.94 (0.25-15.23) | 0.53 | 1.58 (0.67-3.74) | 0.30 | . | . | . |
| ABCC4 ---- tag | rs8001444 | C/C | 18 | 31,03 | 12 | 35,29 | 130 | 34,48 | 106 | 39,26 | 1.00 (.-.) | . | 1.32 (0.65-2.67) | 0.45 | 0.83 | 0.93 | 0.81 |
| ABCC4 ---- tag |  | C/T | 34 | 58,62 | 19 | 55,88 | 182 | 48,28 | 115 | 42,59 | 0.81 (0.35-1.84) | 0.61 | 0.86 (0.43-1.75) | 0.68 | . | . | . |
| ABCC4 ---- tag |  | T/T | 6 | 10,34 | 3 | 8,82 | 65 | 17,24 | 49 | 18,15 | 0.58 (0.12-2.74) | 0.49 | 1.04 (0.49-2.18) | 0.92 | . | . | . |
| ABCC4 ---- tag | rs931111 | T/T | 32 | 55,17 | 23 | 67,65 | 257 | 68,17 | 179 | 66,30 | 1.00 (.-.) | . | 1.27 (0.77-2.10) | 0.34 | 0.97 | 0.99 | 0.98 |
| ABCC4 ---- tag |  | T/C | 22 | 37,93 | 9 | 26,47 | 108 | 28,65 | 76 | 28,15 | 0.98 (0.40-2.39) | 0.96 | 1.12 (0.66-1.89) | 0.67 | . | . | . |
| ABCC4 ---- tag |  | C/C | 4 | 6,90 | 2 | 5,88 | 12 | 3,18 | 15 | 5,56 | 0.87 (0.20-3.83) | 0.85 | 1.32 (0.60-2.92) | 0.49 | . | . | . |
| ABCC4 ---- tag | rs943288 | T/T | 47 | 81,03 | 28 | 82,35 | 285 | 75,60 | 202 | 74,81 | 1.00 (.-.) | . | 1.26 (0.79-2.01) | 0.33 | 0.62 | 0.99 | 0.98 |
| ABCC4 ---- tag |  | T/A | 11 | 18,97 | 5 | 14,71 | 84 | 22,28 | 62 | 22,96 | 1.00 (0.38-2.68) | 0.99 | 1.29 (0.76-2.18) | 0.34 | . | . | . |
| ABCC4 ---- tag |  | A/A | 0 | 0,00 | 1 | 2,94 | 8 | 2,12 | 6 | 2,22 | 5.13 (0.64-40.88) | 0.12 | 1.58 (0.62-4.04) | 0.34 | . | . | . |
| ABCC4 ---- tag | rs943290 | A/A | 31 | 53,45 | 21 | 61,76 | 182 | 48,28 | 153 | 56,67 | 1.00 (.-.) | . | 1.21 (0.75-1.97) | 0.44 | 0.51 | 0.83 | 0.70 |
| ABCC4 ---- tag |  | A/G | 21 | 36,21 | 11 | 32,35 | 164 | 43,50 | 100 | 37,04 | 0.68 (0.28-1.65) | 0.40 | 0.91 (0.56-1.50) | 0.72 | . | . | . |
| ABCC4 ---- tag |  | G/G | 6 | 10,34 | 2 | 5,88 | 31 | 8,22 | 17 | 6,30 | 0.39 (0.05-3.01) | 0.37 | 1.05 (0.53-2.08) | 0.89 | . | . | . |
| ABCC4 ---- tag | rs9516530 | C/C | 31 | 53,45 | 23 | 67,65 | 208 | 55,17 | 153 | 56,67 | 1.00 (.-.) | . | 1.11 (0.67-1.85) | 0.69 | 0.52 | 0.88 | 0.71 |
| ABCC4 ---- tag |  | C/T | 25 | 43,10 | 9 | 26,47 | 131 | 34,75 | 97 | 35,93 | 0.67 (0.29-1.55) | 0.35 | 1.04 (0.62-1.76) | 0.87 | . | . | . |
| ABCC4 ---- tag |  | T/T | 2 | 3,45 | 2 | 5,88 | 38 | 10,08 | 20 | 7,41 | 0.78 (0.10-6.08) | 0.81 | 0.95 (0.49-1.85) | 0.88 | . | . | . |
| ABCC4 ---- tag | rs9516551 | C/C | 43 | 74,14 | 23 | 67,65 | 291 | 77,19 | 210 | 77,78 | 1.00 (.-.) | . | 1.22 (0.74-1.99) | 0.44 | 0.92 | 0.66 | 0.60 |
| ABCC4 ---- tag |  | C/A | 14 | 24,14 | 11 | 32,35 | 82 | 21,75 | 57 | 21,11 | 0.83 (0.37-1.87) | 0.66 | 1.03 (0.60-1.77) | 0.92 | . | . | . |
| ABCC4 ---- tag |  | A/A | 1 | 1,72 | 0 | 0,00 | 4 | 1,06 | 3 | 1,11 | 0.00 (0.00-2E242) | 0.97 | 0.82 (0.23-2.88) | 0.75 | . | . | . |
| ABCC4 ---- tag | rs9524822 | T/T | 45 | 77,59 | 21 | 61,76 | 240 | 63,66 | 173 | 64,07 | 1.00 (.-.) | . | 1.50 (0.88-2.55) | 0.14 | 0.05 | 0.85 | 0.70 |
| ABCC4 ---- tag |  | T/C | 13 | 22,41 | 9 | 26,47 | 123 | 32,63 | 83 | 30,74 | 1.15 (0.50-2.64) | 0.74 | 1.31 (0.75-2.28) | 0.34 | . | . | . |
| ABCC4 ---- tag |  | C/C | 0 | 0,00 | 4 | 11,76 | 14 | 3,71 | 14 | 5,19 | 9.20 (2.50-33.82) | 0.00 | 1.50 (0.63-3.57) | 0.36 | . | . | . |
| ABCC4 ---- tag | rs9524861 | G/G | 23 | 39,66 | 15 | 44,12 | 200 | 53,05 | 139 | 51,48 | 1.00 (.-.) | . | 0.89 (0.49-1.61) | 0.70 | 0.11 | 0.99 | 0.98 |
| ABCC4 ---- tag |  | G/C | 26 | 44,83 | 15 | 44,12 | 151 | 40,05 | 110 | 40,74 | 0.79 (0.35-1.76) | 0.56 | 1.01 (0.55-1.84) | 0.99 | . | . | . |
| ABCC4 ---- tag |  | C/C | 9 | 15,52 | 4 | 11,76 | 26 | 6,90 | 21 | 7,78 | 0.32 (0.09-1.15) | 0.08 | 0.75 (0.36-1.55) | 0.44 | . | . | . |
| ABCC4 ---- tag | rs9524902 | T/T | 15 | 25,86 | 8 | 23,53 | 98 | 25,99 | 93 | 34,44 | 1.00 (.-.) | . | 1.35 (0.61-2.96) | 0.46 | 0.85 | 0.66 | 0.60 |
| ABCC4 ---- tag |  | T/C | 31 | 53,45 | 16 | 47,06 | 195 | 51,72 | 123 | 45,56 | 0.72 (0.28-1.82) | 0.49 | 0.79 (0.36-1.74) | 0.57 | . | . | . |
| ABCC4 ---- tag |  | C/C | 12 | 20,69 | 10 | 29,41 | 84 | 22,28 | 54 | 20,00 | 0.72 (0.24-2.12) | 0.55 | 0.93 (0.41-2.10) | 0.86 | . | . | . |
| ABCC4 ---- tag | rs9556455 | G/G | 45 | 77,59 | 23 | 67,65 | 291 | 77,19 | 203 | 75,19 | 1.00 (.-.) | . | 1.38 (0.83-2.30) | 0.22 | 0.53 | 0.84 | 0.70 |
| ABCC4 ---- tag |  | G/A | 13 | 22,41 | 9 | 26,47 | 84 | 22,28 | 60 | 22,22 | 1.41 (0.60-3.31) | 0.44 | 1.47 (0.84-2.57) | 0.18 | . | . | . |
| ABCC4 ---- tag |  | A/A | 0 | 0,00 | 2 | 5,88 | 2 | 0,53 | 7 | 2,59 | 1.45 (0.32-6.66) | 0.63 | 1.28 (0.48-3.40) | 0.63 | . | . | . |
| ABCC4 ---- NA | rs9561778 | G/G | 36 | 62,07 | 24 | 70,59 | 235 | 62,33 | 190 | 70,37 | 1.00 (.-.) | . | 1.21 (0.76-1.91) | 0.42 | 0.55 | 0.99 | 0.98 |
| ABCC4 ---- NA |  | G/T | 20 | 34,48 | 10 | 29,41 | 125 | 33,16 | 73 | 27,04 | 1.00 (0.38-2.65) | 1.00 | 1.07 (0.65-1.77) | 0.78 | . | . | . |
| ABCC4 ---- NA |  | T/T | 2 | 3,45 | 0 | 0,00 | 17 | 4,51 | 7 | 2,59 | 0.00 (0.00-2E231) | 0.97 | 0.76 (0.30-1.94) | 0.57 | . | . | . |
| ABCC4 ---- tag | rs9561811 | C/C | 40 | 68,97 | 25 | 73,53 | 252 | 66,84 | 178 | 65,93 | 1.00 (.-.) | . | 1.39 (0.86-2.27) | 0.18 | 0.28 | 0.88 | 0.71 |
| ABCC4 ---- tag |  | C/T | 17 | 29,31 | 9 | 26,47 | 112 | 29,71 | 77 | 28,52 | 1.89 (0.80-4.44) | 0.15 | 1.42 (0.84-2.38) | 0.19 | . | . | . |
| ABCC4 ---- tag |  | T/T | 1 | 1,72 | 0 | 0,00 | 13 | 3,45 | 15 | 5,56 | 0.00 (0.00-I) | 0.98 | 2.26 (1.12-4.55) | 0.02 | . | . | . |
| ABCC4 ---- tag | rs9590183 | T/T | 52 | 89,66 | 32 | 94,12 | 321 | 85,15 | 236 | 87,41 | 1.00 (.-.) | . | 1.20 (0.78-1.84) | 0.41 | 0.59 | 0.93 | 0.87 |
| ABCC4 ---- tag |  | T/A | 6 | 10,34 | 2 | 5,88 | 54 | 14,32 | 34 | 12,59 | 0.56 (0.13-2.43) | 0.44 | 1.02 (0.58-1.79) | 0.94 | . | . | . |
| ABCC4 ---- tag |  | A/A | 0 | 0,00 | 0 | 0,00 | 2 | 0,53 | 0 | 0,00 | 0.00 (0.00-3E278) | 0.98 | 0.00 (0.00-4E278) | 0.98 | . | . | . |
| ABCC4 ---- tag | rs997777 | T/T | 26 | 44,83 | 16 | 47,06 | 185 | 49,07 | 134 | 49,63 | 1.00 (.-.) | . | 1.36 (0.77-2.39) | 0.29 | 0.50 | 0.99 | 0.98 |
| ABCC4 ---- tag |  | T/A | 24 | 41,38 | 14 | 41,18 | 162 | 42,97 | 115 | 42,59 | 1.06 (0.48-2.34) | 0.89 | 1.30 (0.73-2.30) | 0.38 | . | . | . |
| ABCC4 ---- tag |  | A/A | 8 | 13,79 | 4 | 11,76 | 30 | 7,96 | 21 | 7,78 | 1.63 (0.36-7.38) | 0.52 | 1.09 (0.54-2.19) | 0.82 | . | . | . |
| ADH1B ---- tag | rs1159918 | G/G | 21 | 36,21 | 12 | 35,29 | 172 | 45,62 | 130 | 48,15 | 1.00 (.-.) | . | 0.86 (0.45-1.67) | 0.66 | 0.26 | 0.93 | 0.64 |
| ADH1B ---- tag |  | G/T | 31 | 53,45 | 16 | 47,06 | 162 | 42,97 | 122 | 45,19 | 0.58 (0.26-1.32) | 0.20 | 0.80 (0.42-1.55) | 0.52 | . | . | . |
| ADH1B ---- tag |  | T/T | 6 | 10,34 | 6 | 17,65 | 43 | 11,41 | 18 | 6,67 | 0.48 (0.14-1.61) | 0.24 | 0.80 (0.36-1.79) | 0.59 | . | . | . |
| ADH1B ---- candidate literature | rs1229984 | G/G | 51 | 87,93 | 32 | 94,12 | 340 | 90,19 | 245 | 90,74 | 1.00 (.-.) | . | 1.15 (0.75-1.76) | 0.53 | 0.26 | 0.96 | 0.64 |
| ADH1B ---- candidate literature |  | G/A | 6 | 10,34 | 2 | 5,88 | 35 | 9,28 | 25 | 9,26 | 0.45 (0.10-1.92) | 0.28 | 1.09 (0.61-1.97) | 0.77 | . | . | . |
| ADH1B ---- candidate literature |  | A/A | 1 | 1,72 | 0 | 0,00 | 2 | 0,53 | 0 | 0,00 | 0.00 (0.00-I) | 0.99 | 0.00 (0.00-I) | 0.98 | . | . | . |
| ADH1B ---- tag | rs12507573 | C/C | 17 | 29,31 | 14 | 41,18 | 115 | 30,50 | 73 | 27,04 | 1.00 (.-.) | . | 1.17 (0.58-2.34) | 0.66 | 0.17 | 0.99 | 0.85 |
| ADH1B ---- tag |  | C/A | 25 | 43,10 | 20 | 58,82 | 179 | 47,48 | 131 | 48,52 | 1.04 (0.47-2.31) | 0.92 | 1.02 (0.52-2.01) | 0.95 | . | . | . |
| ADH1B ---- tag |  | A/A | 16 | 27,59 | 0 | 0,00 | 83 | 22,02 | 66 | 24,44 | 0.00 (0.00-6E305) | 0.97 | 1.25 (0.63-2.51) | 0.52 | . | . | . |
| ADH1B ---- tag | rs1693457 | T/T | 38 | 65,52 | 21 | 61,76 | 261 | 69,23 | 195 | 72,22 | 1.00 (.-.) | . | 1.01 (0.61-1.67) | 0.97 | 0.49 | 0.66 | 0.37 |
| ADH1B ---- tag |  | T/C | 19 | 32,76 | 11 | 32,35 | 100 | 26,53 | 71 | 26,30 | 0.51 (0.23-1.15) | 0.10 | 0.91 (0.53-1.57) | 0.74 | . | . | . |
| ADH1B ---- tag |  | C/C | 1 | 1,72 | 2 | 5,88 | 16 | 4,24 | 4 | 1,48 | 2.21 (0.28-17.38) | 0.45 | 0.52 (0.17-1.59) | 0.25 | . | . | . |
| ADH1B ---- tag | rs2066701 | C/C | 30 | 51,72 | 9 | 26,47 | 178 | 47,21 | 123 | 45,56 | 1.00 (.-.) | . | 2.23 (1.02-4.88) | 0.04 | 0.04 | 0.93 | 0.64 |
| ADH1B ---- tag |  | C/T | 26 | 44,83 | 23 | 67,65 | 160 | 42,44 | 118 | 43,70 | 2.44 (1.01-5.87) | 0.05 | 2.24 (1.02-4.89) | 0.04 | . | . | . |
| ADH1B ---- tag |  | T/T | 2 | 3,45 | 2 | 5,88 | 39 | 10,34 | 29 | 10,74 | 5.53 (1.11-27.49) | 0.04 | 2.97 (1.27-6.94) | 0.01 | . | . | . |
| ADH1C ---- tag | rs11936869 | C/C | 29 | 50,00 | 13 | 38,24 | 194 | 51,46 | 152 | 56,30 | 1.00 (.-.) | . | 1.43 (0.78-2.65) | 0.25 | 0.60 | 0.85 | 0.63 |
| ADH1C ---- tag |  | C/G | 24 | 41,38 | 16 | 47,06 | 148 | 39,26 | 97 | 35,93 | 1.17 (0.52-2.60) | 0.71 | 1.23 (0.66-2.30) | 0.52 | . | . | . |
| ADH1C ---- tag |  | G/G | 5 | 8,62 | 5 | 14,71 | 35 | 9,28 | 21 | 7,78 | 1.11 (0.30-4.10) | 0.88 | 1.43 (0.68-3.02) | 0.35 | . | . | . |
| ADH1C ---- tag | rs1229849 | T/T | 33 | 56,90 | 20 | 58,82 | 204 | 54,11 | 130 | 48,15 | 1.00 (.-.) | . | 1.35 (0.78-2.32) | 0.28 | 0.84 | 0.99 | 0.95 |
| ADH1C ---- tag |  | T/A | 22 | 37,93 | 13 | 38,24 | 155 | 41,11 | 119 | 44,07 | 1.29 (0.59-2.85) | 0.52 | 1.24 (0.72-2.16) | 0.44 | . | . | . |
| ADH1C ---- tag |  | A/A | 3 | 5,17 | 1 | 2,94 | 18 | 4,77 | 21 | 7,78 | 0.65 (0.08-5.06) | 0.68 | 2.52 (1.25-5.07) | 0.01 | . | . | . |
| ADH1C ---- tag | rs1229863 | A/A | 42 | 72,41 | 24 | 70,59 | 275 | 72,94 | 198 | 73,33 | 1.00 (.-.) | . | 1.39 (0.86-2.24) | 0.18 | 0.73 | 0.97 | 0.69 |
| ADH1C ---- tag |  | A/T | 15 | 25,86 | 8 | 23,53 | 93 | 24,67 | 65 | 24,07 | 1.72 (0.68-4.40) | 0.25 | 1.21 (0.71-2.05) | 0.48 | . | . | . |
| ADH1C ---- tag |  | T/T | 1 | 1,72 | 2 | 5,88 | 9 | 2,39 | 7 | 2,59 | 0.62 (0.08-4.77) | 0.65 | 2.00 (0.83-4.83) | 0.12 | . | . | . |
| ADH1C ---- tag | rs1229980 | C/C | 52 | 89,66 | 30 | 88,24 | 343 | 90,98 | 240 | 88,89 | 1.00 (.-.) | . | 1.34 (0.86-2.07) | 0.19 | 0.17 | 0.93 | 0.63 |
| ADH1C ---- tag |  | C/G | 6 | 10,34 | 4 | 11,76 | 34 | 9,02 | 28 | 10,37 | 3.19 (0.93-10.94) | 0.06 | 1.42 (0.80-2.50) | 0.23 | . | . | . |
| ADH1C ---- tag |  | G/G | 0 | 0,00 | 0 | 0,00 | 0 | 0,00 | 2 | 0,74 | 4.99 (1.17-21.31) | 0.03 | 6.66 (1.47-30.22) | 0.01 | . | . | . |
| ADH1C ---- candidate | rs1693482 | C/C | 27 | 46,55 | 15 | 44,12 | 157 | 41,64 | 106 | 39,26 | 1.00 (.-.) | . | 1.40 (0.77-2.55) | 0.27 | 0.58 | 0.93 | 0.63 |
| ADH1C ---- candidate |  | C/T | 23 | 39,66 | 16 | 47,06 | 173 | 45,89 | 120 | 44,44 | 1.35 (0.62-2.97) | 0.45 | 1.12 (0.61-2.04) | 0.72 | . | . | . |
| ADH1C ---- candidate |  | T/T | 8 | 13,79 | 3 | 8,82 | 47 | 12,47 | 44 | 16,30 | 0.51 (0.11-2.28) | 0.38 | 1.86 (0.96-3.61) | 0.07 | . | . | . |
| ADH1C ---- tag | rs2173201 | C/C | 33 | 56,90 | 14 | 41,18 | 220 | 58,36 | 160 | 59,26 | 1.00 (.-.) | . | 1.64 (0.91-2.95) | 0.10 | 0.11 | 0.93 | 0.63 |
| ADH1C ---- tag |  | C/A | 21 | 36,21 | 18 | 52,94 | 132 | 35,01 | 94 | 34,81 | 1.89 (0.87-4.13) | 0.11 | 1.71 (0.94-3.11) | 0.08 | . | . | . |
| ADH1C ---- tag |  | A/A | 4 | 6,90 | 2 | 5,88 | 25 | 6,63 | 16 | 5,93 | 2.90 (0.63-13.38) | 0.17 | 1.90 (0.88-4.12) | 0.10 | . | . | . |
| ADH1C ---- tag | rs2298753 | T/T | 48 | 82,76 | 28 | 82,35 | 304 | 80,64 | 225 | 83,33 | 1.00 (.-.) | . | 1.27 (0.81-1.98) | 0.30 | 0.97 | 0.86 | 0.63 |
| ADH1C ---- tag |  | T/C | 9 | 15,52 | 4 | 11,76 | 69 | 18,30 | 43 | 15,93 | 1.06 (0.31-3.59) | 0.93 | 0.98 (0.57-1.69) | 0.94 | . | . | . |
| ADH1C ---- tag |  | C/C | 1 | 1,72 | 2 | 5,88 | 4 | 1,06 | 2 | 0,74 | 0.58 (0.08-4.42) | 0.60 | 2.64 (0.58-12.10) | 0.21 | . | . | . |
| ADH1C ---- tag | rs2866152 | G/G | 37 | 63,79 | 24 | 70,59 | 232 | 61,54 | 153 | 56,67 | 1.00 (.-.) | . | 1.19 (0.72-1.96) | 0.51 | 0.49 | 0.99 | 0.95 |
| ADH1C ---- tag |  | G/C | 20 | 34,48 | 9 | 26,47 | 130 | 34,48 | 103 | 38,15 | 0.91 (0.39-2.12) | 0.83 | 1.13 (0.67-1.91) | 0.64 | . | . | . |
| ADH1C ---- tag |  | C/C | 1 | 1,72 | 1 | 2,94 | 15 | 3,98 | 14 | 5,19 | 0.60 (0.08-4.63) | 0.63 | 2.12 (0.99-4.51) | 0.05 | . | . | . |
| ADH1C ---- tag | rs904096 | T/T | 27 | 46,55 | 15 | 44,12 | 154 | 40,85 | 105 | 38,89 | 1.00 (.-.) | . | 1.42 (0.78-2.59) | 0.25 | 0.61 | 0.93 | 0.63 |
| ADH1C ---- tag |  | T/G | 23 | 39,66 | 16 | 47,06 | 176 | 46,68 | 121 | 44,81 | 1.36 (0.62-2.97) | 0.45 | 1.11 (0.61-2.02) | 0.74 | . | . | . |
| ADH1C ---- tag |  | G/G | 8 | 13,79 | 3 | 8,82 | 47 | 12,47 | 44 | 16,30 | 0.51 (0.11-2.28) | 0.38 | 1.86 (0.96-3.61) | 0.07 | . | . | . |
| BHMT ---- tag | rs10944 | A/A | 17 | 29,31 | 6 | 17,65 | 85 | 22,55 | 65 | 24,07 | 1.00 (.-.) | . | 1.74 (0.68-4.43) | 0.25 | 0.84 | 0.93 | 0.72 |
| BHMT ---- tag |  | A/C | 25 | 43,10 | 20 | 58,82 | 201 | 53,32 | 139 | 51,48 | 1.50 (0.55-4.11) | 0.43 | 1.51 (0.60-3.78) | 0.38 | . | . | . |
| BHMT ---- tag |  | C/C | 16 | 27,59 | 8 | 23,53 | 91 | 24,14 | 66 | 24,44 | 1.11 (0.33-3.72) | 0.87 | 1.66 (0.66-4.23) | 0.28 | . | . | . |
| BHMT ---- tag | rs12655567 | C/C | 22 | 37,93 | 14 | 41,18 | 133 | 35,28 | 104 | 38,52 | 1.00 (.-.) | . | 1.34 (0.73-2.46) | 0.34 | 0.90 | 0.66 | 0.21 |
| BHMT ---- tag |  | C/G | 30 | 51,72 | 15 | 44,12 | 188 | 49,87 | 132 | 48,89 | 0.85 (0.38-1.90) | 0.70 | 1.03 (0.57-1.87) | 0.93 | . | . | . |
| BHMT ---- tag |  | G/G | 6 | 10,34 | 5 | 14,71 | 56 | 14,85 | 34 | 12,59 | 0.78 (0.21-2.86) | 0.71 | 1.02 (0.52-2.00) | 0.96 | . | . | . |
| BHMT ---- tag | rs1291041 | G/G | 27 | 46,55 | 15 | 44,12 | 153 | 40,58 | 121 | 44,81 | 1.00 (.-.) | . | 1.42 (0.78-2.58) | 0.25 | 0.98 | 0.59 | 0.21 |
| BHMT ---- tag |  | G/T | 26 | 44,83 | 15 | 44,12 | 176 | 46,68 | 120 | 44,44 | 1.05 (0.48-2.31) | 0.91 | 1.03 (0.57-1.88) | 0.92 | . | . | . |
| BHMT ---- tag |  | T/T | 5 | 8,62 | 4 | 11,76 | 48 | 12,73 | 29 | 10,74 | 0.58 (0.13-2.64) | 0.48 | 1.31 (0.66-2.60) | 0.44 | . | . | . |
| BHMT ---- tag | rs16876500 | C/C | 47 | 81,03 | 28 | 82,35 | 304 | 80,64 | 210 | 77,78 | 1.00 (.-.) | . | 1.15 (0.73-1.80) | 0.56 | 0.26 | 0.99 | 0.72 |
| BHMT ---- tag |  | C/T | 10 | 17,24 | 5 | 14,71 | 68 | 18,04 | 57 | 21,11 | 0.91 (0.31-2.73) | 0.87 | 1.43 (0.85-2.41) | 0.17 | . | . | . |
| BHMT ---- tag |  | T/T | 1 | 1,72 | 1 | 2,94 | 5 | 1,33 | 3 | 1,11 | 0.54 (0.07-4.07) | 0.55 | 1.67 (0.37-7.44) | 0.50 | . | . | . |
| BHMT ---- tag | rs492842 | A/A | 24 | 41,38 | 13 | 38,24 | 142 | 37,67 | 109 | 40,37 | 1.00 (.-.) | . | 1.57 (0.80-3.09) | 0.19 | 0.85 | 0.99 | 0.72 |
| BHMT ---- tag |  | A/G | 22 | 37,93 | 19 | 55,88 | 187 | 49,60 | 122 | 45,19 | 1.44 (0.64-3.25) | 0.38 | 1.33 (0.68-2.62) | 0.40 | . | . | . |
| BHMT ---- tag |  | G/G | 12 | 20,69 | 2 | 5,88 | 48 | 12,73 | 39 | 14,44 | 0.67 (0.15-3.12) | 0.61 | 1.57 (0.75-3.28) | 0.23 | . | . | . |
| BHMT ---- tag | rs558133 | T/T | 25 | 43,10 | 18 | 52,94 | 184 | 48,81 | 125 | 46,30 | 1.00 (.-.) | . | 1.28 (0.70-2.33) | 0.42 | 0.66 | 0.93 | 0.72 |
| BHMT ---- tag |  | T/G | 23 | 39,66 | 9 | 26,47 | 151 | 40,05 | 120 | 44,44 | 1.04 (0.43-2.49) | 0.93 | 1.43 (0.79-2.62) | 0.24 | . | . | . |
| BHMT ---- tag |  | G/G | 10 | 17,24 | 7 | 20,59 | 42 | 11,14 | 25 | 9,26 | 1.42 (0.53-3.79) | 0.48 | 1.29 (0.64-2.60) | 0.48 | . | . | . |
| BHMT ---- tag | rs9637824 | A/A | 22 | 37,93 | 12 | 35,29 | 142 | 37,67 | 105 | 38,89 | 1.00 (.-.) | . | 1.58 (0.80-3.12) | 0.19 | 0.48 | 0.99 | 0.72 |
| BHMT ---- tag |  | A/G | 26 | 44,83 | 19 | 55,88 | 186 | 49,34 | 127 | 47,04 | 1.32 (0.58-3.02) | 0.51 | 1.37 (0.69-2.71) | 0.36 | . | . | . |
| BHMT ---- tag |  | G/G | 10 | 17,24 | 3 | 8,82 | 49 | 13,00 | 38 | 14,07 | 1.35 (0.36-5.00) | 0.66 | 1.59 (0.76-3.34) | 0.22 | . | . | . |
| BHMT2 ---- tag | rs16876512 | C/C | 47 | 81,03 | 28 | 82,35 | 301 | 79,84 | 210 | 77,78 | 1.00 (.-.) | . | 1.15 (0.73-1.81) | 0.55 | 0.27 | 0.99 | 0.71 |
| BHMT2 ---- tag |  | C/T | 10 | 17,24 | 5 | 14,71 | 71 | 18,83 | 56 | 20,74 | 0.92 (0.31-2.74) | 0.88 | 1.38 (0.82-2.33) | 0.22 | . | . | . |
| BHMT2 ---- tag |  | T/T | 1 | 1,72 | 1 | 2,94 | 5 | 1,33 | 4 | 1,48 | 0.53 (0.07-4.05) | 0.54 | 2.19 (0.63-7.61) | 0.22 | . | . | . |
| BHMT2 ---- tag | rs2461248 | T/T | 17 | 29,31 | 5 | 14,71 | 84 | 22,28 | 65 | 24,07 | 1.00 (.-.) | . | 2.11 (0.75-5.94) | 0.16 | 0.68 | 0.94 | 0.70 |
| BHMT2 ---- tag |  | T/A | 25 | 43,10 | 21 | 61,76 | 201 | 53,32 | 138 | 51,11 | 1.90 (0.64-5.69) | 0.25 | 1.83 (0.66-5.06) | 0.25 | . | . | . |
| BHMT2 ---- tag |  | A/A | 16 | 27,59 | 8 | 23,53 | 92 | 24,40 | 67 | 24,81 | 1.35 (0.37-4.89) | 0.65 | 2.02 (0.72-5.67) | 0.18 | . | . | . |
| BHMT2 ---- tag | rs2909856 | T/T | 25 | 43,10 | 15 | 44,12 | 155 | 41,11 | 115 | 42,59 | 1.00 (.-.) | . | 1.51 (0.81-2.81) | 0.19 | 0.78 | 0.93 | 0.70 |
| BHMT2 ---- tag |  | T/C | 23 | 39,66 | 17 | 50,00 | 178 | 47,21 | 119 | 44,07 | 1.30 (0.59-2.88) | 0.51 | 1.22 (0.66-2.27) | 0.52 | . | . | . |
| BHMT2 ---- tag |  | C/C | 10 | 17,24 | 2 | 5,88 | 44 | 11,67 | 36 | 13,33 | 0.81 (0.18-3.68) | 0.79 | 1.64 (0.82-3.29) | 0.16 | . | . | . |
| BHMT2 ---- tag | rs476620 | A/A | 22 | 37,93 | 12 | 35,29 | 142 | 37,67 | 104 | 38,52 | 1.00 (.-.) | . | 1.56 (0.79-3.10) | 0.20 | 0.50 | 0.99 | 0.71 |
| BHMT2 ---- tag |  | A/G | 26 | 44,83 | 19 | 55,88 | 186 | 49,34 | 128 | 47,41 | 1.32 (0.58-3.02) | 0.51 | 1.38 (0.70-2.73) | 0.35 | . | . | . |
| BHMT2 ---- tag |  | G/G | 10 | 17,24 | 3 | 8,82 | 49 | 13,00 | 38 | 14,07 | 1.35 (0.36-5.00) | 0.65 | 1.59 (0.76-3.34) | 0.22 | . | . | . |
| BHMT2 ---- candidate literature | rs626105 | G/G | 33 | 56,90 | 23 | 67,65 | 236 | 62,60 | 174 | 64,44 | 1.00 (.-.) | . | 1.30 (0.79-2.15) | 0.30 | 0.90 | 0.89 | 0.70 |
| BHMT2 ---- candidate literature |  | G/A | 22 | 37,93 | 11 | 32,35 | 131 | 34,75 | 88 | 32,59 | 0.99 (0.44-2.22) | 0.97 | 1.06 (0.63-1.78) | 0.84 | . | . | . |
| BHMT2 ---- candidate literature |  | A/A | 3 | 5,17 | 0 | 0,00 | 10 | 2,65 | 8 | 2,96 | 0.00 (0.00-7E299) | 0.98 | 1.63 (0.66-4.01) | 0.29 | . | . | . |
| BHMT2 ---- tag | rs631305 | G/G | 37 | 63,79 | 26 | 76,47 | 263 | 69,76 | 191 | 70,74 | 1.00 (.-.) | . | 1.27 (0.79-2.05) | 0.32 | 0.80 | 0.93 | 0.70 |
| BHMT2 ---- tag |  | G/A | 18 | 31,03 | 8 | 23,53 | 105 | 27,85 | 71 | 26,30 | 1.01 (0.42-2.42) | 0.98 | 1.08 (0.65-1.81) | 0.77 | . | . | . |
| BHMT2 ---- tag |  | A/A | 3 | 5,17 | 0 | 0,00 | 9 | 2,39 | 8 | 2,96 | 0.00 (0.00-2E300) | 0.98 | 1.75 (0.72-4.26) | 0.22 | . | . | . |
| CBS ---- tag | rs11701048 | C/C | 50 | 86,21 | 28 | 82,35 | 318 | 84,35 | 237 | 87,78 | 1.00 (.-.) | . | 1.38 (0.87-2.18) | 0.17 | 0.24 | 0.99 | 0.79 |
| CBS ---- tag |  | C/T | 8 | 13,79 | 6 | 17,65 | 58 | 15,38 | 32 | 11,85 | 1.59 (0.63-4.00) | 0.32 | 1.18 (0.66-2.09) | 0.58 | . | . | . |
| CBS ---- tag |  | T/T | 0 | 0,00 | 0 | 0,00 | 1 | 0,27 | 1 | 0,37 | 0.00 (0.00-8E253) | 0.97 | 0.00 (0.00-1E254) | 0.98 | . | . | . |
| CBS ---- tag | rs234706 | G/G | 25 | 43,10 | 11 | 32,35 | 166 | 44,03 | 108 | 40,00 | 1.00 (.-.) | . | 1.57 (0.77-3.22) | 0.22 | 0.46 | 0.99 | 0.79 |
| CBS ---- tag |  | G/A | 26 | 44,83 | 18 | 52,94 | 166 | 44,03 | 125 | 46,30 | 1.46 (0.63-3.40) | 0.37 | 1.60 (0.79-3.24) | 0.19 | . | . | . |
| CBS ---- tag |  | A/A | 7 | 12,07 | 5 | 14,71 | 45 | 11,94 | 37 | 13,70 | 1.55 (0.46-5.19) | 0.47 | 1.70 (0.79-3.63) | 0.17 | . | . | . |
| CBS ---- tag | rs234711 | C/C | 35 | 60,34 | 19 | 55,88 | 227 | 60,21 | 149 | 55,19 | 1.00 (.-.) | . | 1.32 (0.76-2.30) | 0.32 | 0.49 | 0.93 | 0.79 |
| CBS ---- tag |  | C/A | 20 | 34,48 | 13 | 38,24 | 128 | 33,95 | 103 | 38,15 | 1.12 (0.51-2.48) | 0.77 | 1.34 (0.77-2.35) | 0.30 | . | . | . |
| CBS ---- tag |  | A/A | 3 | 5,17 | 2 | 5,88 | 22 | 5,84 | 18 | 6,67 | 8.94 (1.12-71.21) | 0.04 | 1.53 (0.74-3.18) | 0.25 | . | . | . |
| CBS ---- candidate literature | rs234713 | G/G | 30 | 51,72 | 16 | 47,06 | 187 | 49,60 | 127 | 47,04 | 1.00 (.-.) | . | 1.54 (0.84-2.82) | 0.16 | 0.27 | 0.99 | 0.79 |
| CBS ---- candidate literature |  | G/A | 22 | 37,93 | 14 | 41,18 | 153 | 40,58 | 118 | 43,70 | 1.37 (0.62-3.06) | 0.44 | 1.47 (0.80-2.68) | 0.21 | . | . | . |
| CBS ---- candidate literature |  | A/A | 6 | 10,34 | 4 | 11,76 | 37 | 9,81 | 25 | 9,26 | 1.89 (0.52-6.87) | 0.33 | 1.48 (0.73-3.01) | 0.28 | . | . | . |
| CBS ---- tag | rs2839623 | T/T | 47 | 81,03 | 30 | 88,24 | 312 | 82,76 | 221 | 81,85 | 1.00 (.-.) | . | 1.24 (0.79-1.93) | 0.35 | 0.83 | 0.85 | 0.71 |
| CBS ---- tag |  | T/A | 11 | 18,97 | 4 | 11,76 | 63 | 16,71 | 46 | 17,04 | 0.93 (0.32-2.74) | 0.90 | 1.14 (0.67-1.96) | 0.63 | . | . | . |
| CBS ---- tag |  | A/A | 0 | 0,00 | 0 | 0,00 | 2 | 0,53 | 3 | 1,11 | 3.44 (1.07-11.04) | 0.04 | 4.26 (1.23-14.75) | 0.02 | . | . | . |
| CBS ---- tag | rs2839626 | C/C | 26 | 44,83 | 14 | 41,18 | 187 | 49,60 | 123 | 45,56 | 1.00 (.-.) | . | 1.19 (0.65-2.21) | 0.57 | 0.47 | 0.99 | 0.79 |
| CBS ---- tag |  | C/T | 25 | 43,10 | 17 | 50,00 | 150 | 39,79 | 126 | 46,67 | 1.30 (0.59-2.87) | 0.52 | 1.40 (0.76-2.59) | 0.28 | . | . | . |
| CBS ---- tag |  | T/T | 7 | 12,07 | 3 | 8,82 | 40 | 10,61 | 21 | 7,78 | 0.47 (0.10-2.11) | 0.32 | 1.17 (0.56-2.45) | 0.67 | . | . | . |
| CBS ---- tag | rs422791 | T/T | 28 | 48,28 | 20 | 58,82 | 196 | 51,99 | 118 | 43,70 | 1.00 (.-.) | . | 1.08 (0.63-1.86) | 0.77 | 0.54 | 0.59 | 0.14 |
| CBS ---- tag |  | T/C | 23 | 39,66 | 12 | 35,29 | 143 | 37,93 | 126 | 46,67 | 1.08 (0.48-2.42) | 0.85 | 1.52 (0.89-2.61) | 0.13 | . | . | . |
| CBS ---- tag |  | C/C | 7 | 12,07 | 2 | 5,88 | 38 | 10,08 | 26 | 9,63 | 0.50 (0.06-3.89) | 0.51 | 0.93 (0.47-1.84) | 0.85 | . | . | . |
| CBS ---- tag | rs706209 | C/C | 18 | 31,03 | 8 | 23,53 | 124 | 32,89 | 86 | 31,85 | 1.00 (.-.) | . | 1.13 (0.48-2.68) | 0.78 | 0.39 | 0.99 | 0.79 |
| CBS ---- tag |  | C/T | 28 | 48,28 | 16 | 47,06 | 184 | 48,81 | 136 | 50,37 | 1.08 (0.40-2.86) | 0.89 | 1.15 (0.49-2.66) | 0.75 | . | . | . |
| CBS ---- tag |  | T/T | 12 | 20,69 | 10 | 29,41 | 69 | 18,30 | 48 | 17,78 | 0.74 (0.25-2.20) | 0.59 | 1.38 (0.57-3.31) | 0.47 | . | . | . |
| CBS ---- tag | rs719037 | A/A | 19 | 32,76 | 14 | 41,18 | 129 | 34,22 | 75 | 27,78 | 1.00 (.-.) | . | 1.21 (0.63-2.31) | 0.57 | 0.65 | 0.59 | 0.14 |
| CBS ---- tag |  | A/G | 26 | 44,83 | 17 | 50,00 | 175 | 46,42 | 140 | 51,85 | 1.24 (0.56-2.74) | 0.60 | 1.34 (0.71-2.49) | 0.37 | . | . | . |
| CBS ---- tag |  | G/G | 13 | 22,41 | 3 | 8,82 | 73 | 19,36 | 55 | 20,37 | 0.62 (0.14-2.85) | 0.54 | 1.41 (0.72-2.76) | 0.31 | . | . | . |
| CBS ---- tag | rs719038 | T/T | 27 | 46,55 | 12 | 35,29 | 170 | 45,09 | 120 | 44,44 | 1.00 (.-.) | . | 1.26 (0.65-2.44) | 0.50 | 0.42 | 0.99 | 0.79 |
| CBS ---- tag |  | T/C | 23 | 39,66 | 18 | 52,94 | 163 | 43,24 | 120 | 44,44 | 1.27 (0.56-2.90) | 0.57 | 1.22 (0.63-2.38) | 0.56 | . | . | . |
| CBS ---- tag |  | C/C | 8 | 13,79 | 4 | 11,76 | 44 | 11,67 | 30 | 11,11 | 0.49 (0.13-1.81) | 0.28 | 1.36 (0.65-2.84) | 0.41 | . | . | . |
| DHFR ---- tag | rs10474632 | G/G | 52 | 89,66 | 29 | 85,29 | 313 | 83,02 | 225 | 83,33 | 1.00 (.-.) | . | 1.26 (0.81-1.96) | 0.31 | 0.84 | 0.59 | 0.20 |
| DHFR ---- tag |  | G/A | 6 | 10,34 | 5 | 14,71 | 60 | 15,92 | 45 | 16,67 | 0.86 (0.29-2.52) | 0.78 | 1.04 (0.61-1.77) | 0.89 | . | . | . |
| DHFR ---- tag |  | A/A | 0 | 0,00 | 0 | 0,00 | 4 | 1,06 | 0 | 0,00 | 0.00 (0.00-9E243) | 0.97 | 0.00 (0.00-1E244) | 0.97 | . | . | . |
| DHFR ---- tag | rs11951910 | T/T | 47 | 81,03 | 26 | 76,47 | 306 | 81,17 | 220 | 81,48 | 1.00 (.-.) | . | 1.11 (0.70-1.77) | 0.65 | 0.26 | 0.99 | 0.89 |
| DHFR ---- tag |  | T/C | 11 | 18,97 | 7 | 20,59 | 67 | 17,77 | 46 | 17,04 | 0.71 (0.26-1.91) | 0.50 | 1.28 (0.74-2.20) | 0.38 | . | . | . |
| DHFR ---- tag |  | C/C | 0 | 0,00 | 1 | 2,94 | 4 | 1,06 | 4 | 1,48 | 0.86 (0.11-6.72) | 0.89 | 3.27 (1.09-9.79) | 0.03 | . | . | . |
| DHFR ---- tag | rs1643665 | T/T | 30 | 51,72 | 14 | 41,18 | 176 | 46,68 | 119 | 44,07 | 1.00 (.-.) | . | 1.14 (0.59-2.18) | 0.69 | 0.89 | 0.99 | 0.89 |
| DHFR ---- tag |  | T/C | 21 | 36,21 | 15 | 44,12 | 157 | 41,64 | 131 | 48,52 | 0.94 (0.41-2.13) | 0.87 | 1.29 (0.68-2.46) | 0.44 | . | . | . |
| DHFR ---- tag |  | C/C | 7 | 12,07 | 5 | 14,71 | 44 | 11,67 | 20 | 7,41 | 0.93 (0.29-3.04) | 0.91 | 0.90 (0.40-2.00) | 0.79 | . | . | . |
| DHFR ---- tag | rs1650717 | T/T | 26 | 44,83 | 17 | 50,00 | 208 | 55,17 | 147 | 54,44 | 1.00 (.-.) | . | 1.10 (0.61-1.98) | 0.75 | 0.50 | 0.85 | 0.59 |
| DHFR ---- tag |  | T/G | 30 | 51,72 | 11 | 32,35 | 140 | 37,14 | 99 | 36,67 | 0.87 (0.38-2.02) | 0.75 | 1.22 (0.67-2.23) | 0.52 | . | . | . |
| DHFR ---- tag |  | G/G | 2 | 3,45 | 6 | 17,65 | 29 | 7,69 | 24 | 8,89 | 0.98 (0.34-2.82) | 0.97 | 1.62 (0.79-3.30) | 0.19 | . | . | . |
| DHFR ---- tag | rs1805355 | G/G | 48 | 82,76 | 26 | 76,47 | 328 | 87,00 | 239 | 88,52 | 1.00 (.-.) | . | 1.49 (0.93-2.39) | 0.09 | 0.05 | 0.99 | 0.91 |
| DHFR ---- tag |  | G/A | 10 | 17,24 | 8 | 23,53 | 48 | 12,73 | 30 | 11,11 | 2.26 (0.94-5.41) | 0.07 | 1.13 (0.62-2.07) | 0.69 | . | . | . |
| DHFR ---- tag |  | A/A | 0 | 0,00 | 0 | 0,00 | 1 | 0,27 | 1 | 0,37 | 3.43 (0.46-25.54) | 0.23 | 5.12 (0.66-39.83) | 0.12 | . | . | . |
| DHFR ---- tag | rs6151617 | A/A | 24 | 41,38 | 12 | 35,29 | 132 | 35,01 | 94 | 34,81 | 1.00 (.-.) | . | 1.20 (0.58-2.45) | 0.63 | 0.90 | 0.95 | 0.89 |
| DHFR ---- tag |  | A/G | 26 | 44,83 | 14 | 41,18 | 173 | 45,89 | 137 | 50,74 | 0.98 (0.41-2.33) | 0.96 | 1.30 (0.64-2.62) | 0.47 | . | . | . |
| DHFR ---- tag |  | G/G | 8 | 13,79 | 8 | 23,53 | 72 | 19,10 | 39 | 14,44 | 0.84 (0.29-2.42) | 0.75 | 0.84 (0.39-1.81) | 0.65 | . | . | . |
| DHFR ---- tag | rs6864493 | T/T | 36 | 62,07 | 19 | 55,88 | 210 | 55,70 | 147 | 54,44 | 1.00 (.-.) | . | 0.98 (0.56-1.69) | 0.93 | 0.24 | 0.99 | 0.89 |
| DHFR ---- tag |  | T/C | 18 | 31,03 | 13 | 38,24 | 140 | 37,14 | 110 | 40,74 | 0.66 (0.29-1.50) | 0.33 | 1.02 (0.58-1.77) | 0.96 | . | . | . |
| DHFR ---- tag |  | C/C | 4 | 6,90 | 2 | 5,88 | 27 | 7,16 | 13 | 4,81 | 0.51 (0.11-2.30) | 0.38 | 0.95 (0.42-2.15) | 0.90 | . | . | . |
| DHFR ---- tag | rs836788 | G/G | 25 | 43,10 | 19 | 55,88 | 163 | 43,24 | 112 | 41,48 | 1.00 (.-.) | . | 1.41 (0.81-2.45) | 0.22 | 0.27 | 0.99 | 0.89 |
| DHFR ---- tag |  | G/A | 27 | 46,55 | 11 | 32,35 | 161 | 42,71 | 124 | 45,93 | 1.06 (0.47-2.43) | 0.88 | 1.36 (0.79-2.35) | 0.26 | . | . | . |
| DHFR ---- tag |  | A/A | 6 | 10,34 | 4 | 11,76 | 53 | 14,06 | 34 | 12,59 | 2.07 (0.59-7.24) | 0.26 | 1.10 (0.58-2.07) | 0.77 | . | . | . |
| DHFR ---- tag | rs836790 | A/A | 37 | 63,79 | 22 | 64,71 | 270 | 71,62 | 183 | 67,78 | 1.00 (.-.) | . | 1.18 (0.71-1.97) | 0.53 | 0.94 | 0.85 | 0.59 |
| DHFR ---- tag |  | A/G | 20 | 34,48 | 11 | 32,35 | 96 | 25,46 | 81 | 30,00 | 0.92 (0.42-2.05) | 0.84 | 1.40 (0.81-2.42) | 0.23 | . | . | . |
| DHFR ---- tag |  | G/G | 1 | 1,72 | 1 | 2,94 | 11 | 2,92 | 6 | 2,22 | 2.96 (0.38-22.89) | 0.30 | 1.10 (0.40-3.06) | 0.85 | . | . | . |
| DHFR ---- tag | rs836817 | G/G | 25 | 43,10 | 21 | 61,76 | 186 | 49,34 | 120 | 44,44 | 1.00 (.-.) | . | 1.29 (0.77-2.17) | 0.34 | 0.41 | 0.85 | 0.59 |
| DHFR ---- tag |  | G/T | 27 | 46,55 | 11 | 32,35 | 147 | 38,99 | 128 | 47,41 | 1.28 (0.57-2.90) | 0.55 | 1.52 (0.91-2.55) | 0.11 | . | . | . |
| DHFR ---- tag |  | T/T | 6 | 10,34 | 2 | 5,88 | 44 | 11,67 | 22 | 8,15 | 1.73 (0.22-13.26) | 0.60 | 0.97 (0.50-1.87) | 0.93 | . | . | . |
| DNMT1 ---- candidate | rs2228612 | A/A | 48 | 82,76 | 27 | 79,41 | 338 | 89,66 | 232 | 85,93 | 1.00 (.-.) | . | 1.29 (0.82-2.02) | 0.28 | 0.72 | 0.93 | 0.43 |
| DNMT1 ---- candidate |  | A/G | 10 | 17,24 | 7 | 20,59 | 38 | 10,08 | 37 | 13,70 | 1.36 (0.51-3.65) | 0.54 | 1.50 (0.85-2.62) | 0.16 | . | . | . |
| DNMT1 ---- candidate |  | G/G | 0 | 0,00 | 0 | 0,00 | 1 | 0,27 | 1 | 0,37 | 0.78 (0.11-5.77) | 0.81 | 1.01 (0.13-7.76) | 0.99 | . | . | . |
| DNMT3A ---- tag | rs10460566 | A/A | 34 | 58,62 | 22 | 64,71 | 222 | 58,89 | 160 | 59,26 | 1.00 (.-.) | . | 1.46 (0.88-2.43) | 0.15 | 0.59 | 0.99 | 0.92 |
| DNMT3A ---- tag |  | A/G | 18 | 31,03 | 12 | 35,29 | 137 | 36,34 | 95 | 35,19 | 1.92 (0.87-4.24) | 0.11 | 1.35 (0.80-2.30) | 0.26 | . | . | . |
| DNMT3A ---- tag |  | G/G | 6 | 10,34 | 0 | 0,00 | 18 | 4,77 | 15 | 5,56 | 0.00 (0.00-I) | 0.98 | 1.25 (0.60-2.59) | 0.55 | . | . | . |
| DNMT3A ---- candidate literature | rs11695471 | T/T | 28 | 48,28 | 12 | 35,29 | 164 | 43,50 | 117 | 43,33 | 1.00 (.-.) | . | 1.18 (0.64-2.17) | 0.60 | 0.70 | 0.89 | 0.89 |
| DNMT3A ---- candidate literature |  | T/A | 24 | 41,38 | 15 | 44,12 | 162 | 42,97 | 121 | 44,81 | 1.01 (0.44-2.30) | 0.99 | 1.57 (0.85-2.88) | 0.15 | . | . | . |
| DNMT3A ---- candidate literature |  | A/A | 6 | 10,34 | 7 | 20,59 | 51 | 13,53 | 32 | 11,85 | 1.51 (0.48-4.72) | 0.48 | 1.07 (0.53-2.14) | 0.85 | . | . | . |
| DNMT3A ---- tag | rs11887120 | C/C | 20 | 34,48 | 13 | 38,24 | 133 | 35,28 | 96 | 35,56 | 1.00 (.-.) | . | 1.05 (0.52-2.11) | 0.90 | 0.57 | 0.99 | 0.92 |
| DNMT3A ---- tag |  | C/T | 29 | 50,00 | 17 | 50,00 | 181 | 48,01 | 126 | 46,67 | 0.89 (0.39-2.04) | 0.78 | 1.23 (0.61-2.47) | 0.56 | . | . | . |
| DNMT3A ---- tag |  | T/T | 9 | 15,52 | 4 | 11,76 | 63 | 16,71 | 48 | 17,78 | 0.59 (0.16-2.21) | 0.43 | 0.90 (0.43-1.88) | 0.78 | . | . | . |
| DNMT3A ---- tag | rs12991495 | T/T | 30 | 51,72 | 14 | 41,18 | 173 | 45,89 | 127 | 47,04 | 1.00 (.-.) | . | 1.14 (0.64-2.06) | 0.65 | 0.90 | 0.86 | 0.89 |
| DNMT3A ---- tag |  | T/C | 22 | 37,93 | 14 | 41,18 | 162 | 42,97 | 114 | 42,22 | 0.85 (0.37-1.95) | 0.70 | 1.33 (0.74-2.39) | 0.35 | . | . | . |
| DNMT3A ---- tag |  | C/C | 6 | 10,34 | 6 | 17,65 | 42 | 11,14 | 29 | 10,74 | 1.55 (0.50-4.84) | 0.45 | 1.22 (0.61-2.44) | 0.57 | . | . | . |
| DNMT3A ---- tag | rs13401241 | A/A | 21 | 36,21 | 8 | 23,53 | 108 | 28,65 | 72 | 26,67 | 1.00 (.-.) | . | 2.06 (0.87-4.91) | 0.10 | 0.59 | 0.99 | 0.92 |
| DNMT3A ---- tag |  | A/C | 24 | 41,38 | 19 | 55,88 | 185 | 49,07 | 151 | 55,93 | 2.06 (0.80-5.28) | 0.13 | 1.75 (0.75-4.08) | 0.20 | . | . | . |
| DNMT3A ---- tag |  | C/C | 13 | 22,41 | 7 | 20,59 | 84 | 22,28 | 47 | 17,41 | 1.02 (0.30-3.44) | 0.97 | 1.70 (0.70-4.11) | 0.24 | . | . | . |
| DNMT3A ---- candidate literature | rs13420827 | C/C | 39 | 67,24 | 24 | 70,59 | 230 | 61,01 | 188 | 69,63 | 1.00 (.-.) | . | 1.19 (0.71-1.97) | 0.51 | 0.92 | 0.99 | 0.89 |
| DNMT3A ---- candidate literature |  | C/G | 15 | 25,86 | 6 | 17,65 | 133 | 35,28 | 73 | 27,04 | 0.79 (0.31-2.03) | 0.63 | 1.08 (0.63-1.85) | 0.78 | . | . | . |
| DNMT3A ---- candidate literature |  | G/G | 4 | 6,90 | 4 | 11,76 | 14 | 3,71 | 9 | 3,33 | 0.72 (0.24-2.22) | 0.57 | 0.78 (0.33-1.86) | 0.58 | . | . | . |
| DNMT3A ---- tag | rs13428812 | A/A | 24 | 41,38 | 21 | 61,76 | 188 | 49,87 | 119 | 44,07 | 1.00 (.-.) | . | 1.01 (0.59-1.73) | 0.97 | 0.21 | 0.93 | 0.89 |
| DNMT3A ---- tag |  | A/G | 26 | 44,83 | 11 | 32,35 | 156 | 41,38 | 131 | 48,52 | 0.87 (0.40-1.92) | 0.73 | 1.28 (0.74-2.21) | 0.37 | . | . | . |
| DNMT3A ---- tag |  | G/G | 8 | 13,79 | 2 | 5,88 | 33 | 8,75 | 20 | 7,41 | 0.37 (0.05-2.78) | 0.33 | 1.08 (0.53-2.18) | 0.83 | . | . | . |
| DNMT3A ---- tag | rs4665287 | C/C | 40 | 68,97 | 24 | 70,59 | 236 | 62,60 | 192 | 71,11 | 1.00 (.-.) | . | 1.19 (0.72-1.98) | 0.49 | 0.86 | 0.95 | 0.89 |
| DNMT3A ---- tag |  | C/T | 15 | 25,86 | 5 | 14,71 | 126 | 33,42 | 69 | 25,56 | 0.66 (0.24-1.82) | 0.43 | 1.06 (0.62-1.82) | 0.83 | . | . | . |
| DNMT3A ---- tag |  | T/T | 3 | 5,17 | 5 | 14,71 | 15 | 3,98 | 9 | 3,33 | 0.90 (0.32-2.54) | 0.85 | 0.79 (0.33-1.87) | 0.59 | . | . | . |
| DNMT3B ---- tag | rs13045669 | A/A | 54 | 93,10 | 31 | 91,18 | 348 | 92,31 | 251 | 92,96 | 1.00 (.-.) | . | 1.28 (0.83-1.97) | 0.26 | 0.89 | 0.93 | 0.97 |
| DNMT3B ---- tag |  | A/G | 4 | 6,90 | 3 | 8,82 | 29 | 7,69 | 18 | 6,67 | 0.90 (0.21-3.95) | 0.89 | 0.88 (0.44-1.75) | 0.72 | . | . | . |
| DNMT3B ---- tag |  | G/G | 0 | 0,00 | 0 | 0,00 | 0 | 0,00 | 1 | 0,37 | I (0.00-I) | 0.31 | I (0.00-I) | 0.31 | . | . | . |
| DNMT3B ---- tag | rs17123673 | A/A | 54 | 93,10 | 29 | 85,29 | 347 | 92,04 | 248 | 91,85 | 1.00 (.-.) | . | 1.31 (0.84-2.04) | 0.23 | 0.54 | 0.85 | 0.97 |
| DNMT3B ---- tag |  | A/G | 4 | 6,90 | 5 | 14,71 | 30 | 7,96 | 20 | 7,41 | 1.36 (0.46-4.01) | 0.58 | 1.11 (0.58-2.12) | 0.75 | . | . | . |
| DNMT3B ---- tag |  | G/G | 0 | 0,00 | 0 | 0,00 | 0 | 0,00 | 2 | 0,74 | 1.28 (0.29-5.55) | 0.74 | 1.68 (0.36-7.80) | 0.51 | . | . | . |
| DNMT3B ---- tag | rs183603 | A/A | 30 | 51,72 | 20 | 58,82 | 200 | 53,05 | 147 | 54,44 | 1.00 (.-.) | . | 0.96 (0.57-1.62) | 0.88 | 0.23 | 0.88 | 0.97 |
| DNMT3B ---- tag |  | A/G | 20 | 34,48 | 13 | 38,24 | 146 | 38,73 | 111 | 41,11 | 0.59 (0.27-1.31) | 0.20 | 1.01 (0.59-1.72) | 0.98 | . | . | . |
| DNMT3B ---- tag |  | G/G | 8 | 13,79 | 1 | 2,94 | 31 | 8,22 | 12 | 4,44 | 0.32 (0.04-2.49) | 0.28 | 0.53 (0.24-1.16) | 0.11 | . | . | . |
| DNMT3B ---- tag | rs2235760 | C/C | 39 | 67,24 | 23 | 67,65 | 267 | 70,82 | 200 | 74,07 | 1.00 (.-.) | . | 1.39 (0.86-2.25) | 0.18 | 0.49 | 0.99 | 0.97 |
| DNMT3B ---- tag |  | C/T | 17 | 29,31 | 11 | 32,35 | 99 | 26,26 | 62 | 22,96 | 1.35 (0.58-3.12) | 0.48 | 1.15 (0.68-1.97) | 0.60 | . | . | . |
| DNMT3B ---- tag |  | T/T | 2 | 3,45 | 0 | 0,00 | 11 | 2,92 | 8 | 2,96 | 0.00 (0.00-I) | 0.98 | 0.93 (0.38-2.24) | 0.87 | . | . | . |
| DNMT3B ---- tag | rs2424908 | C/C | 35 | 60,34 | 25 | 73,53 | 232 | 61,54 | 176 | 65,19 | 1.00 (.-.) | . | 0.98 (0.61-1.57) | 0.93 | 0.12 | 0.99 | 0.97 |
| DNMT3B ---- tag |  | C/T | 19 | 32,76 | 9 | 26,47 | 125 | 33,16 | 85 | 31,48 | 0.46 (0.19-1.10) | 0.08 | 1.01 (0.61-1.67) | 0.98 | . | . | . |
| DNMT3B ---- tag |  | T/T | 4 | 6,90 | 0 | 0,00 | 20 | 5,31 | 9 | 3,33 | 0.00 (0.00-1E281) | 0.98 | 0.56 (0.24-1.30) | 0.18 | . | . | . |
| DNMT3B ---- candidate literature | rs2424909 | T/T | 17 | 29,31 | 14 | 41,18 | 139 | 36,87 | 118 | 43,70 | 1.00 (.-.) | . | 1.01 (0.56-1.83) | 0.97 | 0.24 | 0.99 | 0.97 |
| DNMT3B ---- candidate literature |  | T/C | 28 | 48,28 | 19 | 55,88 | 173 | 45,89 | 114 | 42,22 | 0.70 (0.32-1.53) | 0.37 | 0.92 (0.50-1.68) | 0.79 | . | . | . |
| DNMT3B ---- candidate literature |  | C/C | 13 | 22,41 | 1 | 2,94 | 65 | 17,24 | 38 | 14,07 | 0.22 (0.03-1.70) | 0.15 | 0.75 (0.39-1.44) | 0.38 | . | . | . |
| DNMT3B ---- tag | rs4911108 | A/A | 19 | 32,76 | 14 | 41,18 | 150 | 39,79 | 123 | 45,56 | 1.00 (.-.) | . | 1.04 (0.58-1.87) | 0.90 | 0.18 | 0.99 | 0.97 |
| DNMT3B ---- tag |  | A/G | 26 | 44,83 | 20 | 58,82 | 166 | 44,03 | 112 | 41,48 | 0.80 (0.37-1.72) | 0.56 | 0.98 (0.54-1.79) | 0.95 | . | . | . |
| DNMT3B ---- tag |  | G/G | 13 | 22,41 | 0 | 0,00 | 61 | 16,18 | 35 | 12,96 | 0.00 (0.00-I) | 0.97 | 0.74 (0.38-1.43) | 0.36 | . | . | . |
| DNMT3B ---- tag | rs6058896 | C/C | 50 | 86,21 | 31 | 91,18 | 336 | 89,12 | 241 | 89,26 | 1.00 (.-.) | . | 1.26 (0.82-1.95) | 0.30 | 0.97 | 0.99 | 0.97 |
| DNMT3B ---- tag |  | C/T | 6 | 10,34 | 3 | 8,82 | 40 | 10,61 | 28 | 10,37 | 1.30 (0.39-4.35) | 0.67 | 1.19 (0.66-2.15) | 0.56 | . | . | . |
| DNMT3B ---- tag |  | T/T | 2 | 3,45 | 0 | 0,00 | 1 | 0,27 | 1 | 0,37 | 0.00 (0.00-3E289) | 0.98 | 2.56 (0.33-19.69) | 0.37 | . | . | . |
| DNMT3B ---- tag | rs6119954 | G/G | 36 | 62,07 | 24 | 70,59 | 263 | 69,76 | 199 | 73,70 | 1.00 (.-.) | . | 1.24 (0.77-2.01) | 0.37 | 0.91 | 0.99 | 0.97 |
| DNMT3B ---- tag |  | G/A | 19 | 32,76 | 10 | 29,41 | 99 | 26,26 | 63 | 23,33 | 0.96 (0.42-2.22) | 0.93 | 1.13 (0.66-1.92) | 0.66 | . | . | . |
| DNMT3B ---- tag |  | A/A | 3 | 5,17 | 0 | 0,00 | 15 | 3,98 | 8 | 2,96 | 0.00 (0.00-4E248) | 0.97 | 0.76 (0.31-1.83) | 0.53 | . | . | . |
| DNMT3B ---- tag | rs6579038 | A/A | 50 | 86,21 | 33 | 97,06 | 334 | 88,59 | 240 | 88,89 | 1.00 (.-.) | . | 1.22 (0.80-1.86) | 0.36 | 0.62 | 0.99 | 0.97 |
| DNMT3B ---- tag |  | A/G | 8 | 13,79 | 1 | 2,94 | 42 | 11,14 | 29 | 10,74 | 0.61 (0.08-4.55) | 0.63 | 1.15 (0.65-2.02) | 0.63 | . | . | . |
| DNMT3B ---- tag |  | G/G | 0 | 0,00 | 0 | 0,00 | 1 | 0,27 | 1 | 0,37 | 2.02 (0.27-15.07) | 0.49 | 2.46 (0.32-18.85) | 0.39 | . | . | . |
| DPYD ---- tag | rs1034215 | C/C | 38 | 65,52 | 19 | 55,88 | 209 | 55,44 | 173 | 64,07 | 1.00 (.-.) | . | 1.91 (1.07-3.38) | 0.03 | 0.03 | 0.87 | 0.53 |
| DPYD ---- tag |  | C/T | 18 | 31,03 | 15 | 44,12 | 145 | 38,46 | 81 | 30,00 | 2.18 (1.01-4.71) | 0.05 | 1.39 (0.76-2.55) | 0.28 | . | . | . |
| DPYD ---- tag |  | T/T | 2 | 3,45 | 0 | 0,00 | 23 | 6,10 | 16 | 5,93 | 0.00 (0.00-3E253) | 0.98 | 1.95 (0.90-4.26) | 0.09 | . | . | . |
| DPYD ---- tag | rs10783058 | T/T | 30 | 51,72 | 18 | 52,94 | 153 | 40,58 | 100 | 37,04 | 1.00 (.-.) | . | 0.91 (0.51-1.62) | 0.74 | 0.35 | 0.59 | 0.29 |
| DPYD ---- tag |  | T/C | 22 | 37,93 | 11 | 32,35 | 185 | 49,07 | 127 | 47,04 | 0.62 (0.26-1.49) | 0.29 | 1.15 (0.65-2.04) | 0.64 | . | . | . |
| DPYD ---- tag |  | C/C | 6 | 10,34 | 5 | 14,71 | 39 | 10,34 | 43 | 15,93 | 0.97 (0.34-2.78) | 0.95 | 1.18 (0.63-2.20) | 0.60 | . | . | . |
| DPYD ---- tag | rs10783070 | C/C | 43 | 74,14 | 27 | 79,41 | 273 | 72,41 | 184 | 68,15 | 1.00 (.-.) | . | 1.19 (0.74-1.91) | 0.48 | 0.37 | 0.83 | 0.51 |
| DPYD ---- tag |  | C/T | 13 | 22,41 | 6 | 17,65 | 97 | 25,73 | 78 | 28,89 | 1.26 (0.50-3.18) | 0.62 | 1.47 (0.89-2.44) | 0.14 | . | . | . |
| DPYD ---- tag |  | T/T | 2 | 3,45 | 1 | 2,94 | 7 | 1,86 | 8 | 2,96 | 0.53 (0.07-4.10) | 0.54 | 2.31 (0.95-5.64) | 0.07 | . | . | . |
| DPYD ---- tag | rs10875048 | G/G | 40 | 68,97 | 23 | 67,65 | 262 | 69,50 | 180 | 66,67 | 1.00 (.-.) | . | 1.15 (0.68-1.92) | 0.61 | 0.33 | 0.93 | 0.63 |
| DPYD ---- tag |  | G/A | 13 | 22,41 | 10 | 29,41 | 110 | 29,18 | 80 | 29,63 | 0.92 (0.42-2.04) | 0.84 | 1.01 (0.58-1.73) | 0.98 | . | . | . |
| DPYD ---- tag |  | A/A | 5 | 8,62 | 1 | 2,94 | 5 | 1,33 | 10 | 3,70 | 0.30 (0.04-2.31) | 0.25 | 1.96 (0.86-4.48) | 0.11 | . | . | . |
| DPYD ---- tag | rs10875055 | C/C | 31 | 53,45 | 6 | 17,65 | 109 | 28,91 | 51 | 18,89 | 1.00 (.-.) | . | 1.66 (0.70-3.93) | 0.25 | 0.55 | 0.59 | 0.29 |
| DPYD ---- tag |  | C/T | 20 | 34,48 | 22 | 64,71 | 195 | 51,72 | 144 | 53,33 | 2.58 (1.02-6.54) | 0.05 | 2.43 (1.06-5.59) | 0.04 | . | . | . |
| DPYD ---- tag |  | T/T | 7 | 12,07 | 6 | 17,65 | 73 | 19,36 | 75 | 27,78 | 2.40 (0.66-8.70) | 0.18 | 3.18 (1.36-7.45) | 0.01 | . | . | . |
| DPYD ---- tag | rs10875079 | A/A | 10 | 17,24 | 9 | 26,47 | 100 | 26,53 | 78 | 28,89 | 1.00 (.-.) | . | 1.55 (0.60-3.99) | 0.36 | 0.67 | 0.99 | 0.81 |
| DPYD ---- tag |  | A/G | 37 | 63,79 | 17 | 50,00 | 184 | 48,81 | 130 | 48,15 | 1.30 (0.46-3.64) | 0.62 | 1.55 (0.61-3.95) | 0.36 | . | . | . |
| DPYD ---- tag |  | G/G | 11 | 18,97 | 8 | 23,53 | 93 | 24,67 | 62 | 22,96 | 1.13 (0.35-3.66) | 0.84 | 1.30 (0.50-3.34) | 0.59 | . | . | . |
| DPYD ---- tag | rs10875085 | A/A | 39 | 67,24 | 24 | 70,59 | 264 | 70,03 | 181 | 67,04 | 1.00 (.-.) | . | 1.19 (0.73-1.93) | 0.49 | 0.70 | 0.93 | 0.69 |
| DPYD ---- tag |  | A/T | 17 | 29,31 | 9 | 26,47 | 103 | 27,32 | 79 | 29,26 | 0.83 (0.35-1.93) | 0.66 | 1.13 (0.67-1.89) | 0.65 | . | . | . |
| DPYD ---- tag |  | T/T | 2 | 3,45 | 1 | 2,94 | 10 | 2,65 | 10 | 3,70 | 0.00 (0.00-1E241) | 0.97 | 0.83 (0.36-1.94) | 0.67 | . | . | . |
| DPYD ---- tag | rs10875097 | G/G | 42 | 72,41 | 20 | 58,82 | 260 | 68,97 | 179 | 66,30 | 1.00 (.-.) | . | 1.72 (0.98-3.03) | 0.06 | 0.03 | 0.59 | 0.51 |
| DPYD ---- tag |  | G/A | 16 | 27,59 | 13 | 38,24 | 103 | 27,32 | 82 | 30,37 | 2.27 (1.05-4.90) | 0.04 | 1.80 (0.99-3.27) | 0.05 | . | . | . |
| DPYD ---- tag |  | A/A | 0 | 0,00 | 1 | 2,94 | 14 | 3,71 | 9 | 3,33 | 6.78 (0.83-55.35) | 0.07 | 1.72 (0.67-4.42) | 0.26 | . | . | . |
| DPYD ---- tag | rs11165781 | T/T | 40 | 68,97 | 25 | 73,53 | 264 | 70,03 | 177 | 65,56 | 1.00 (.-.) | . | 1.12 (0.71-1.78) | 0.62 | 0.34 | 0.99 | 0.81 |
| DPYD ---- tag |  | T/C | 17 | 29,31 | 8 | 23,53 | 106 | 28,12 | 86 | 31,85 | 0.78 (0.26-2.31) | 0.65 | 1.25 (0.76-2.03) | 0.38 | . | . | . |
| DPYD ---- tag |  | C/C | 1 | 1,72 | 1 | 2,94 | 7 | 1,86 | 7 | 2,59 | 0.68 (0.09-5.20) | 0.71 | 2.26 (0.83-6.15) | 0.11 | . | . | . |
| DPYD ---- tag | rs11165783 | T/T | 32 | 55,17 | 19 | 55,88 | 207 | 54,91 | 148 | 54,81 | 1.00 (.-.) | . | 1.44 (0.81-2.53) | 0.21 | 0.24 | 0.83 | 0.51 |
| DPYD ---- tag |  | T/C | 23 | 39,66 | 13 | 38,24 | 138 | 36,60 | 99 | 36,67 | 1.33 (0.61-2.92) | 0.48 | 1.53 (0.85-2.74) | 0.16 | . | . | . |
| DPYD ---- tag |  | C/C | 3 | 5,17 | 2 | 5,88 | 32 | 8,49 | 23 | 8,52 | 4.25 (0.93-19.32) | 0.06 | 1.63 (0.82-3.24) | 0.16 | . | . | . |
| DPYD ---- tag | rs11165873 | A/A | 14 | 24,14 | 7 | 20,59 | 105 | 27,85 | 82 | 30,37 | 1.00 (.-.) | . | 1.45 (0.57-3.65) | 0.43 | 0.60 | 0.83 | 0.51 |
| DPYD ---- tag |  | A/T | 28 | 48,28 | 16 | 47,06 | 197 | 52,25 | 130 | 48,15 | 1.03 (0.36-2.95) | 0.96 | 1.27 (0.51-3.19) | 0.61 | . | . | . |
| DPYD ---- tag |  | T/T | 16 | 27,59 | 11 | 32,35 | 75 | 19,89 | 58 | 21,48 | 1.22 (0.40-3.68) | 0.72 | 1.37 (0.53-3.54) | 0.51 | . | . | . |
| DPYD ---- tag | rs11165875 | T/T | 24 | 41,38 | 16 | 47,06 | 149 | 39,52 | 109 | 40,37 | 1.00 (.-.) | . | 1.35 (0.75-2.42) | 0.32 | 0.68 | 0.85 | 0.51 |
| DPYD ---- tag |  | T/C | 29 | 50,00 | 12 | 35,29 | 183 | 48,54 | 118 | 43,70 | 0.89 (0.39-2.03) | 0.77 | 1.10 (0.61-1.97) | 0.76 | . | . | . |
| DPYD ---- tag |  | C/C | 5 | 8,62 | 6 | 17,65 | 45 | 11,94 | 43 | 15,93 | 1.44 (0.46-4.52) | 0.53 | 1.40 (0.75-2.64) | 0.30 | . | . | . |
| DPYD ---- tag | rs11165881 | T/T | 22 | 37,93 | 17 | 50,00 | 129 | 34,22 | 90 | 33,33 | 1.00 (.-.) | . | 0.87 (0.46-1.64) | 0.67 | 0.38 | 0.94 | 0.71 |
| DPYD ---- tag |  | T/C | 25 | 43,10 | 14 | 41,18 | 174 | 46,15 | 134 | 49,63 | 0.73 (0.33-1.65) | 0.46 | 1.24 (0.67-2.29) | 0.50 | . | . | . |
| DPYD ---- tag |  | C/C | 11 | 18,97 | 3 | 8,82 | 74 | 19,63 | 46 | 17,04 | 0.96 (0.26-3.53) | 0.96 | 1.14 (0.59-2.21) | 0.69 | . | . | . |
| DPYD ---- tag | rs11587873 | C/C | 34 | 58,62 | 21 | 61,76 | 202 | 53,58 | 172 | 63,70 | 1.00 (.-.) | . | 1.19 (0.70-2.03) | 0.51 | 0.93 | 0.85 | 0.51 |
| DPYD ---- tag |  | C/T | 20 | 34,48 | 12 | 35,29 | 144 | 38,20 | 90 | 33,33 | 0.71 (0.33-1.54) | 0.39 | 0.97 (0.55-1.68) | 0.90 | . | . | . |
| DPYD ---- tag |  | T/T | 4 | 6,90 | 1 | 2,94 | 31 | 8,22 | 8 | 2,96 | 1.06 (0.14-8.20) | 0.95 | 0.56 (0.23-1.39) | 0.21 | . | . | . |
| DPYD ---- tag | rs12030174 | C/C | 42 | 72,41 | 24 | 70,59 | 278 | 73,74 | 193 | 71,48 | 1.00 (.-.) | . | 1.24 (0.77-2.01) | 0.38 | 0.94 | 0.92 | 0.61 |
| DPYD ---- tag |  | C/T | 15 | 25,86 | 9 | 26,47 | 90 | 23,87 | 70 | 25,93 | 0.89 (0.38-2.07) | 0.78 | 1.08 (0.64-1.83) | 0.77 | . | . | . |
| DPYD ---- tag |  | T/T | 1 | 1,72 | 1 | 2,94 | 9 | 2,39 | 7 | 2,59 | 0.00 (0.00-2E241) | 0.97 | 0.74 (0.27-2.03) | 0.56 | . | . | . |
| DPYD ---- tag | rs12046744 | A/A | 30 | 51,72 | 19 | 55,88 | 205 | 54,38 | 152 | 56,30 | 1.00 (.-.) | . | 1.35 (0.79-2.30) | 0.28 | 0.48 | 0.99 | 0.92 |
| DPYD ---- tag |  | A/C | 24 | 41,38 | 12 | 35,29 | 138 | 36,60 | 101 | 37,41 | 1.43 (0.64-3.20) | 0.39 | 1.72 (0.99-2.98) | 0.06 | . | . | . |
| DPYD ---- tag |  | C/C | 4 | 6,90 | 3 | 8,82 | 34 | 9,02 | 17 | 6,30 | 1.20 (0.27-5.38) | 0.81 | 0.88 (0.43-1.84) | 0.74 | . | . | . |
| DPYD ---- tag | rs12047910 | G/G | 43 | 74,14 | 24 | 70,59 | 279 | 74,01 | 202 | 74,81 | 1.00 (.-.) | . | 1.37 (0.83-2.27) | 0.22 | 0.44 | 0.85 | 0.51 |
| DPYD ---- tag |  | G/A | 14 | 24,14 | 9 | 26,47 | 95 | 25,20 | 61 | 22,59 | 1.08 (0.48-2.42) | 0.86 | 1.07 (0.62-1.85) | 0.81 | . | . | . |
| DPYD ---- tag |  | A/A | 1 | 1,72 | 1 | 2,94 | 3 | 0,80 | 7 | 2,59 | 5.02 (0.64-39.42) | 0.13 | 3.37 (1.37-8.29) | 0.01 | . | . | . |
| DPYD ---- tag | rs12073044 | T/T | 50 | 86,21 | 29 | 85,29 | 285 | 75,60 | 216 | 80,00 | 1.00 (.-.) | . | 1.33 (0.85-2.08) | 0.22 | 0.57 | 0.85 | 0.51 |
| DPYD ---- tag |  | T/A | 8 | 13,79 | 5 | 14,71 | 88 | 23,34 | 53 | 19,63 | 1.03 (0.35-3.03) | 0.96 | 1.00 (0.60-1.68) | 0.99 | . | . | . |
| DPYD ---- tag |  | A/A | 0 | 0,00 | 0 | 0,00 | 4 | 1,06 | 1 | 0,37 | 0.00 (0.00-4E222) | 0.97 | 0.00 (0.00-5E222) | 0.97 | . | . | . |
| DPYD ---- tag | rs12126093 | T/T | 25 | 43,10 | 12 | 35,29 | 194 | 51,46 | 144 | 53,33 | 1.00 (.-.) | . | 1.24 (0.64-2.40) | 0.52 | 0.87 | 0.93 | 0.62 |
| DPYD ---- tag |  | T/C | 28 | 48,28 | 17 | 50,00 | 158 | 41,91 | 101 | 37,41 | 0.91 (0.40-2.08) | 0.82 | 1.14 (0.58-2.22) | 0.71 | . | . | . |
| DPYD ---- tag |  | C/C | 5 | 8,62 | 5 | 14,71 | 25 | 6,63 | 25 | 9,26 | 1.02 (0.31-3.34) | 0.98 | 1.10 (0.50-2.42) | 0.81 | . | . | . |
| DPYD ---- tag | rs12134028 | C/C | 52 | 89,66 | 29 | 85,29 | 344 | 91,25 | 238 | 88,15 | 1.00 (.-.) | . | 1.13 (0.72-1.75) | 0.60 | 0.28 | 0.83 | 0.51 |
| DPYD ---- tag |  | C/T | 6 | 10,34 | 4 | 11,76 | 33 | 8,75 | 31 | 11,48 | 0.51 (0.15-1.72) | 0.28 | 1.07 (0.61-1.89) | 0.81 | . | . | . |
| DPYD ---- tag |  | T/T | 0 | 0,00 | 1 | 2,94 | 0 | 0,00 | 1 | 0,37 | 1.65 (0.22-12.18) | 0.63 | 1.85 (0.24-14.32) | 0.55 | . | . | . |
| DPYD ---- tag | rs12740796 | T/T | 37 | 63,79 | 28 | 82,35 | 278 | 73,74 | 210 | 77,78 | 1.00 (.-.) | . | 1.20 (0.77-1.87) | 0.42 | 0.74 | 0.99 | 0.85 |
| DPYD ---- tag |  | T/C | 19 | 32,76 | 5 | 14,71 | 92 | 24,40 | 55 | 20,37 | 0.61 (0.18-2.05) | 0.42 | 1.07 (0.65-1.76) | 0.80 | . | . | . |
| DPYD ---- tag |  | C/C | 2 | 3,45 | 1 | 2,94 | 7 | 1,86 | 5 | 1,85 | 1.14 (0.15-8.63) | 0.90 | 1.37 (0.51-3.67) | 0.53 | . | . | . |
| DPYD ---- tag | rs1333717 | A/A | 37 | 63,79 | 18 | 52,94 | 202 | 53,58 | 163 | 60,37 | 1.00 (.-.) | . | 1.87 (1.05-3.33) | 0.03 | 0.04 | 0.87 | 0.53 |
| DPYD ---- tag |  | A/G | 18 | 31,03 | 16 | 47,06 | 151 | 40,05 | 90 | 33,33 | 2.14 (0.99-4.60) | 0.05 | 1.46 (0.80-2.66) | 0.21 | . | . | . |
| DPYD ---- tag |  | G/G | 3 | 5,17 | 0 | 0,00 | 24 | 6,37 | 17 | 6,30 | 0.00 (0.00-1E253) | 0.98 | 1.74 (0.81-3.74) | 0.15 | . | . | . |
| DPYD ---- tag | rs1413228 | A/A | 46 | 79,31 | 31 | 91,18 | 306 | 81,17 | 206 | 76,30 | 1.00 (.-.) | . | 1.06 (0.69-1.63) | 0.80 | 0.13 | 0.99 | 0.93 |
| DPYD ---- tag |  | A/G | 12 | 20,69 | 2 | 5,88 | 66 | 17,51 | 60 | 22,22 | 0.24 (0.03-1.78) | 0.16 | 1.35 (0.82-2.22) | 0.24 | . | . | . |
| DPYD ---- tag |  | G/G | 0 | 0,00 | 1 | 2,94 | 5 | 1,33 | 4 | 1,48 | 0.84 (0.11-6.50) | 0.87 | 0.88 (0.20-3.80) | 0.87 | . | . | . |
| DPYD ---- tag | rs1415681 | G/G | 43 | 74,14 | 29 | 85,29 | 270 | 71,62 | 202 | 74,81 | 1.00 (.-.) | . | 1.09 (0.69-1.72) | 0.71 | 0.26 | 0.97 | 0.78 |
| DPYD ---- tag |  | G/T | 14 | 24,14 | 5 | 14,71 | 99 | 26,26 | 59 | 21,85 | 0.64 (0.22-1.87) | 0.41 | 1.26 (0.75-2.10) | 0.38 | . | . | . |
| DPYD ---- tag |  | T/T | 1 | 1,72 | 0 | 0,00 | 8 | 2,12 | 9 | 3,33 | 1.37 (0.65-2.87) | 0.40 | 1.49 (0.66-3.36) | 0.33 | . | . | . |
| DPYD ---- tag | rs1514495 | C/C | 31 | 53,45 | 19 | 55,88 | 238 | 63,13 | 158 | 58,52 | 1.00 (.-.) | . | 1.19 (0.70-2.03) | 0.53 | 0.44 | 0.99 | 0.89 |
| DPYD ---- tag |  | C/T | 20 | 34,48 | 13 | 38,24 | 120 | 31,83 | 93 | 34,44 | 1.09 (0.48-2.49) | 0.83 | 1.11 (0.63-1.93) | 0.72 | . | . | . |
| DPYD ---- tag |  | T/T | 7 | 12,07 | 2 | 5,88 | 19 | 5,04 | 19 | 7,04 | 0.45 (0.10-2.01) | 0.30 | 1.43 (0.69-2.94) | 0.34 | . | . | . |
| DPYD ---- tag | rs1520658 | A/A | 41 | 70,69 | 30 | 88,24 | 307 | 81,43 | 224 | 82,96 | 1.00 (.-.) | . | 1.10 (0.72-1.69) | 0.66 | 0.13 | 0.86 | 0.53 |
| DPYD ---- tag |  | A/G | 16 | 27,59 | 4 | 11,76 | 63 | 16,71 | 44 | 16,30 | 0.38 (0.09-1.64) | 0.20 | 1.17 (0.71-1.94) | 0.54 | . | . | . |
| DPYD ---- tag |  | G/G | 1 | 1,72 | 0 | 0,00 | 7 | 1,86 | 2 | 0,74 | 0.00 (0.00-4E240) | 0.97 | 0.62 (0.14-2.64) | 0.52 | . | . | . |
| DPYD ---- NA | rs17116806 | C/C | 36 | 62,07 | 19 | 55,88 | 249 | 66,05 | 172 | 63,70 | 1.00 (.-.) | . | 1.53 (0.85-2.75) | 0.16 | 0.11 | 0.83 | 0.51 |
| DPYD ---- NA |  | C/A | 22 | 37,93 | 13 | 38,24 | 109 | 28,91 | 86 | 31,85 | 1.43 (0.65-3.13) | 0.37 | 1.62 (0.87-2.99) | 0.13 | . | . | . |
| DPYD ---- NA |  | A/A | 0 | 0,00 | 2 | 5,88 | 19 | 5,04 | 12 | 4,44 | 8.10 (1.75-37.42) | 0.01 | 1.53 (0.65-3.59) | 0.33 | . | . | . |
| DPYD ---- tag | rs17431828 | G/G | 26 | 44,83 | 12 | 35,29 | 147 | 38,99 | 122 | 45,19 | 1.00 (.-.) | . | 1.52 (0.78-2.96) | 0.22 | 0.47 | 0.99 | 0.95 |
| DPYD ---- tag |  | G/C | 24 | 41,38 | 17 | 50,00 | 181 | 48,01 | 114 | 42,22 | 1.40 (0.61-3.20) | 0.43 | 1.54 (0.79-3.02) | 0.21 | . | . | . |
| DPYD ---- tag |  | C/C | 8 | 13,79 | 5 | 14,71 | 49 | 13,00 | 34 | 12,59 | 1.19 (0.37-3.86) | 0.77 | 1.22 (0.57-2.59) | 0.61 | . | . | . |
| DPYD ---- tag | rs17471640 | T/T | 23 | 39,66 | 12 | 35,29 | 160 | 42,44 | 133 | 49,26 | 1.00 (.-.) | . | 1.69 (0.87-3.31) | 0.12 | 0.33 | 0.85 | 0.51 |
| DPYD ---- tag |  | T/C | 27 | 46,55 | 17 | 50,00 | 186 | 49,34 | 111 | 41,11 | 1.46 (0.63-3.34) | 0.38 | 1.37 (0.70-2.69) | 0.36 | . | . | . |
| DPYD ---- tag |  | C/C | 8 | 13,79 | 5 | 14,71 | 31 | 8,22 | 26 | 9,63 | 1.24 (0.38-4.02) | 0.72 | 1.49 (0.67-3.29) | 0.33 | . | . | . |
| DPYD ---- tag | rs17702702 | G/G | 34 | 58,62 | 25 | 73,53 | 259 | 68,70 | 193 | 71,48 | 1.00 (.-.) | . | 1.34 (0.83-2.16) | 0.22 | 0.67 | 0.99 | 0.81 |
| DPYD ---- tag |  | G/C | 20 | 34,48 | 8 | 23,53 | 107 | 28,38 | 68 | 25,19 | 1.10 (0.43-2.77) | 0.85 | 1.12 (0.67-1.86) | 0.67 | . | . | . |
| DPYD ---- tag |  | C/C | 4 | 6,90 | 1 | 2,94 | 11 | 2,92 | 9 | 3,33 | 0.89 (0.12-6.79) | 0.91 | 1.13 (0.51-2.52) | 0.77 | . | . | . |
| DPYD ---- NA | rs1801265 | T/T | 34 | 58,62 | 22 | 64,71 | 211 | 55,97 | 163 | 60,37 | 1.00 (.-.) | . | 1.43 (0.85-2.39) | 0.18 | 0.45 | 0.99 | 0.95 |
| DPYD ---- NA |  | T/C | 21 | 36,21 | 12 | 35,29 | 139 | 36,87 | 94 | 34,81 | 1.27 (0.58-2.76) | 0.55 | 1.26 (0.73-2.15) | 0.41 | . | . | . |
| DPYD ---- NA |  | C/C | 3 | 5,17 | 0 | 0,00 | 27 | 7,16 | 13 | 4,81 | 0.00 (0.00-8E300) | 0.98 | 1.04 (0.47-2.29) | 0.93 | . | . | . |
| DPYD ---- tag | rs2039447 | T/T | 23 | 39,66 | 22 | 64,71 | 176 | 46,68 | 134 | 49,63 | 1.00 (.-.) | . | 1.17 (0.71-1.92) | 0.54 | 0.60 | 0.88 | 0.56 |
| DPYD ---- tag |  | T/C | 24 | 41,38 | 8 | 23,53 | 164 | 43,50 | 115 | 42,59 | 0.78 (0.28-2.12) | 0.62 | 1.02 (0.61-1.69) | 0.95 | . | . | . |
| DPYD ---- tag |  | C/C | 11 | 18,97 | 4 | 11,76 | 37 | 9,81 | 21 | 7,78 | 0.56 (0.19-1.67) | 0.30 | 0.89 (0.46-1.71) | 0.73 | . | . | . |
| DPYD ---- tag | rs2151567 | G/G | 50 | 86,21 | 33 | 97,06 | 343 | 90,98 | 243 | 90,00 | 1.00 (.-.) | . | 1.23 (0.80-1.87) | 0.34 | 0.74 | 0.85 | 0.51 |
| DPYD ---- tag |  | G/A | 8 | 13,79 | 1 | 2,94 | 33 | 8,75 | 26 | 9,63 | 0.83 (0.11-6.15) | 0.85 | 1.31 (0.73-2.37) | 0.36 | . | . | . |
| DPYD ---- tag |  | A/A | 0 | 0,00 | 0 | 0,00 | 1 | 0,27 | 1 | 0,37 | 5.35 (0.71-40.41) | 0.10 | 6.56 (0.83-51.75) | 0.07 | . | . | . |
| DPYD ---- tag | rs2152878 | A/A | 30 | 51,72 | 21 | 61,76 | 214 | 56,76 | 156 | 57,78 | 1.00 (.-.) | . | 1.05 (0.62-1.77) | 0.85 | 0.49 | 0.99 | 0.94 |
| DPYD ---- tag |  | A/G | 25 | 43,10 | 8 | 23,53 | 141 | 37,40 | 96 | 35,56 | 0.67 (0.28-1.59) | 0.36 | 1.22 (0.71-2.11) | 0.47 | . | . | . |
| DPYD ---- tag |  | G/G | 3 | 5,17 | 5 | 14,71 | 22 | 5,84 | 18 | 6,67 | 1.33 (0.37-4.74) | 0.66 | 1.45 (0.70-3.00) | 0.32 | . | . | . |
| DPYD ---- tag | rs2786505 | G/G | 45 | 77,59 | 28 | 82,35 | 285 | 75,60 | 195 | 72,22 | 1.00 (.-.) | . | 1.14 (0.72-1.81) | 0.58 | 0.40 | 0.82 | 0.51 |
| DPYD ---- tag |  | G/T | 13 | 22,41 | 6 | 17,65 | 90 | 23,87 | 69 | 25,56 | 0.88 (0.35-2.20) | 0.79 | 1.35 (0.82-2.23) | 0.24 | . | . | . |
| DPYD ---- tag |  | T/T | 0 | 0,00 | 0 | 0,00 | 2 | 0,53 | 6 | 2,22 | 3.82 (1.54-9.49) | 0.00 | 4.35 (1.60-11.81) | 0.00 | . | . | . |
| DPYD ---- tag | rs2786512 | G/G | 16 | 27,59 | 17 | 50,00 | 151 | 40,05 | 89 | 32,96 | 1.00 (.-.) | . | 0.66 (0.38-1.15) | 0.14 | 0.01 | 0.99 | 0.94 |
| DPYD ---- tag |  | G/A | 29 | 50,00 | 12 | 35,29 | 158 | 41,91 | 136 | 50,37 | 0.43 (0.18-1.02) | 0.06 | 0.82 (0.48-1.41) | 0.48 | . | . | . |
| DPYD ---- tag |  | A/A | 13 | 22,41 | 5 | 14,71 | 68 | 18,04 | 45 | 16,67 | 0.33 (0.11-1.01) | 0.05 | 0.79 (0.43-1.42) | 0.43 | . | . | . |
| DPYD ---- tag | rs2786519 | A/A | 38 | 65,52 | 18 | 52,94 | 235 | 62,33 | 154 | 57,04 | 1.00 (.-.) | . | 1.70 (0.94-3.07) | 0.08 | 0.07 | 0.83 | 0.51 |
| DPYD ---- tag |  | A/G | 19 | 32,76 | 12 | 35,29 | 118 | 31,30 | 103 | 38,15 | 2.07 (0.91-4.71) | 0.08 | 1.98 (1.08-3.63) | 0.03 | . | . | . |
| DPYD ---- tag |  | G/G | 1 | 1,72 | 4 | 11,76 | 24 | 6,37 | 13 | 4,81 | 2.69 (0.84-8.60) | 0.10 | 1.23 (0.53-2.88) | 0.63 | . | . | . |
| DPYD ---- tag | rs2811170 | A/A | 44 | 75,86 | 24 | 70,59 | 272 | 72,15 | 212 | 78,52 | 1.00 (.-.) | . | 1.22 (0.76-1.97) | 0.41 | 0.98 | 0.59 | 0.29 |
| DPYD ---- tag |  | A/T | 14 | 24,14 | 10 | 29,41 | 100 | 26,53 | 56 | 20,74 | 0.75 (0.32-1.74) | 0.50 | 0.89 (0.52-1.53) | 0.67 | . | . | . |
| DPYD ---- tag |  | T/T | 0 | 0,00 | 0 | 0,00 | 5 | 1,33 | 2 | 0,74 | 0.85 (0.12-6.19) | 0.87 | 1.03 (0.14-7.90) | 0.97 | . | . | . |
| DPYD ---- tag | rs2811199 | G/G | 44 | 75,86 | 27 | 79,41 | 277 | 73,47 | 189 | 70,00 | 1.00 (.-.) | . | 1.20 (0.75-1.93) | 0.45 | 0.38 | 0.83 | 0.51 |
| DPYD ---- tag |  | G/A | 13 | 22,41 | 6 | 17,65 | 94 | 24,93 | 74 | 27,41 | 1.25 (0.50-3.16) | 0.63 | 1.41 (0.85-2.35) | 0.18 | . | . | . |
| DPYD ---- tag |  | A/A | 1 | 1,72 | 1 | 2,94 | 6 | 1,59 | 7 | 2,59 | 0.55 (0.07-4.25) | 0.56 | 3.74 (1.47-9.56) | 0.01 | . | . | . |
| DPYD ---- tag | rs2811219 | T/T | 33 | 56,90 | 21 | 61,76 | 203 | 53,85 | 158 | 58,52 | 1.00 (.-.) | . | 1.24 (0.73-2.08) | 0.42 | 0.70 | 0.92 | 0.61 |
| DPYD ---- tag |  | T/C | 24 | 41,38 | 12 | 35,29 | 155 | 41,11 | 94 | 34,81 | 0.89 (0.40-1.97) | 0.78 | 1.01 (0.59-1.74) | 0.96 | . | . | . |
| DPYD ---- tag |  | C/C | 1 | 1,72 | 1 | 2,94 | 19 | 5,04 | 18 | 6,67 | 0.50 (0.06-3.94) | 0.51 | 1.42 (0.70-2.90) | 0.33 | . | . | . |
| DPYD ---- tag | rs4300257 | A/A | 39 | 67,24 | 21 | 61,76 | 244 | 64,72 | 172 | 63,70 | 1.00 (.-.) | . | 1.37 (0.83-2.28) | 0.22 | 0.70 | 0.99 | 0.93 |
| DPYD ---- tag |  | A/C | 17 | 29,31 | 10 | 29,41 | 119 | 31,56 | 84 | 31,11 | 1.13 (0.49-2.57) | 0.78 | 1.10 (0.65-1.88) | 0.72 | . | . | . |
| DPYD ---- tag |  | C/C | 2 | 3,45 | 3 | 8,82 | 14 | 3,71 | 14 | 5,19 | 0.79 (0.10-6.06) | 0.83 | 1.45 (0.67-3.14) | 0.34 | . | . | . |
| DPYD ---- tag | rs4379706 | T/T | 34 | 58,62 | 22 | 64,71 | 204 | 54,11 | 164 | 60,74 | 1.00 (.-.) | . | 1.44 (0.86-2.41) | 0.17 | 0.16 | 0.99 | 0.97 |
| DPYD ---- tag |  | T/C | 21 | 36,21 | 10 | 29,41 | 144 | 38,20 | 92 | 34,07 | 1.06 (0.46-2.41) | 0.90 | 1.23 (0.72-2.10) | 0.45 | . | . | . |
| DPYD ---- tag |  | C/C | 3 | 5,17 | 2 | 5,88 | 29 | 7,69 | 14 | 5,19 | 5.29 (1.11-25.29) | 0.04 | 1.06 (0.49-2.30) | 0.88 | . | . | . |
| DPYD ---- tag | rs4950021 | T/T | 21 | 36,21 | 12 | 35,29 | 116 | 30,77 | 80 | 29,63 | 1.00 (.-.) | . | 1.27 (0.66-2.46) | 0.47 | 0.88 | 0.93 | 0.66 |
| DPYD ---- tag |  | T/G | 22 | 37,93 | 17 | 50,00 | 177 | 46,95 | 127 | 47,04 | 1.22 (0.54-2.77) | 0.63 | 1.44 (0.76-2.73) | 0.26 | . | . | . |
| DPYD ---- tag |  | G/G | 15 | 25,86 | 5 | 14,71 | 84 | 22,28 | 63 | 23,33 | 1.13 (0.35-3.62) | 0.84 | 1.40 (0.72-2.74) | 0.32 | . | . | . |
| DPYD ---- tag | rs4950033 | T/T | 19 | 32,76 | 13 | 38,24 | 99 | 26,26 | 83 | 30,74 | 1.00 (.-.) | . | 1.07 (0.57-2.03) | 0.83 | 0.39 | 0.99 | 0.89 |
| DPYD ---- tag |  | T/C | 27 | 46,55 | 16 | 47,06 | 196 | 51,99 | 123 | 45,56 | 0.73 (0.33-1.62) | 0.44 | 0.94 (0.51-1.74) | 0.85 | . | . | . |
| DPYD ---- tag |  | C/C | 12 | 20,69 | 5 | 14,71 | 82 | 21,75 | 64 | 23,70 | 0.53 (0.14-1.92) | 0.33 | 0.96 (0.51-1.82) | 0.91 | . | . | . |
| DPYD ---- tag | rs495257 | T/T | 19 | 32,76 | 21 | 61,76 | 129 | 34,22 | 84 | 31,11 | 1.00 (.-.) | . | 0.76 (0.45-1.30) | 0.32 | 0.04 | 0.85 | 0.51 |
| DPYD ---- tag |  | T/C | 27 | 46,55 | 8 | 23,53 | 198 | 52,52 | 154 | 57,04 | 0.50 (0.19-1.27) | 0.14 | 0.99 (0.60-1.66) | 0.98 | . | . | . |
| DPYD ---- tag |  | C/C | 12 | 20,69 | 5 | 14,71 | 50 | 13,26 | 32 | 11,85 | 0.38 (0.13-1.15) | 0.09 | 0.72 (0.39-1.33) | 0.30 | . | . | . |
| DPYD ---- tag | rs552926 | A/A | 26 | 44,83 | 15 | 44,12 | 133 | 35,28 | 85 | 31,48 | 1.00 (.-.) | . | 0.93 (0.51-1.73) | 0.83 | 0.59 | 0.59 | 0.29 |
| DPYD ---- tag |  | A/G | 25 | 43,10 | 13 | 38,24 | 192 | 50,93 | 134 | 49,63 | 0.73 (0.31-1.70) | 0.47 | 1.31 (0.71-2.41) | 0.38 | . | . | . |
| DPYD ---- tag |  | G/G | 7 | 12,07 | 6 | 17,65 | 52 | 13,79 | 51 | 18,89 | 1.01 (0.35-2.94) | 0.99 | 1.03 (0.54-1.94) | 0.94 | . | . | . |
| DPYD ---- tag | rs628959 | A/A | 24 | 41,38 | 16 | 47,06 | 174 | 46,15 | 145 | 53,70 | 1.00 (.-.) | . | 1.07 (0.58-1.99) | 0.82 | 0.73 | 0.93 | 0.62 |
| DPYD ---- tag |  | A/G | 31 | 53,45 | 15 | 44,12 | 175 | 46,42 | 102 | 37,78 | 0.70 (0.32-1.53) | 0.37 | 0.95 (0.51-1.79) | 0.88 | . | . | . |
| DPYD ---- tag |  | G/G | 3 | 5,17 | 3 | 8,82 | 28 | 7,43 | 23 | 8,52 | 0.82 (0.18-3.81) | 0.80 | 0.86 (0.41-1.79) | 0.68 | . | . | . |
| DPYD ---- tag | rs6656660 | G/G | 43 | 74,14 | 30 | 88,24 | 297 | 78,78 | 199 | 73,70 | 1.00 (.-.) | . | 1.08 (0.69-1.68) | 0.73 | 0.21 | 0.99 | 0.89 |
| DPYD ---- tag |  | G/T | 14 | 24,14 | 3 | 8,82 | 72 | 19,10 | 61 | 22,59 | 0.45 (0.10-1.92) | 0.28 | 1.29 (0.79-2.13) | 0.31 | . | . | . |
| DPYD ---- tag |  | T/T | 1 | 1,72 | 1 | 2,94 | 8 | 2,12 | 10 | 3,70 | 0.84 (0.11-6.49) | 0.87 | 1.50 (0.65-3.43) | 0.34 | . | . | . |
| DPYD ---- tag | rs6663670 | A/A | 43 | 74,14 | 26 | 76,47 | 274 | 72,68 | 191 | 70,74 | 1.00 (.-.) | . | 1.21 (0.75-1.95) | 0.43 | 0.42 | 0.86 | 0.53 |
| DPYD ---- tag |  | A/C | 14 | 24,14 | 7 | 20,59 | 98 | 25,99 | 71 | 26,30 | 1.23 (0.49-3.10) | 0.66 | 1.36 (0.82-2.26) | 0.24 | . | . | . |
| DPYD ---- tag |  | C/C | 1 | 1,72 | 1 | 2,94 | 5 | 1,33 | 8 | 2,96 | 0.54 (0.07-4.24) | 0.56 | 3.95 (1.55-10.10) | 0.00 | . | . | . |
| DPYD ---- tag | rs6683883 | T/T | 32 | 55,17 | 8 | 23,53 | 147 | 38,99 | 87 | 32,22 | 1.00 (.-.) | . | 2.03 (0.87-4.70) | 0.10 | 0.17 | 0.85 | 0.51 |
| DPYD ---- tag |  | T/C | 23 | 39,66 | 22 | 64,71 | 179 | 47,48 | 129 | 47,78 | 2.41 (0.95-6.11) | 0.06 | 2.32 (1.01-5.32) | 0.05 | . | . | . |
| DPYD ---- tag |  | C/C | 3 | 5,17 | 4 | 11,76 | 51 | 13,53 | 54 | 20,00 | 3.45 (0.81-14.75) | 0.10 | 3.15 (1.33-7.46) | 0.01 | . | . | . |
| DPYD ---- tag | rs6686861 | C/C | 51 | 87,93 | 31 | 91,18 | 333 | 88,33 | 225 | 83,33 | 1.00 (.-.) | . | 1.01 (0.66-1.55) | 0.95 | 0.02 | 0.88 | 0.55 |
| DPYD ---- tag |  | C/T | 7 | 12,07 | 3 | 8,82 | 41 | 10,88 | 43 | 15,93 | 0.25 (0.06-1.06) | 0.06 | 1.19 (0.71-2.00) | 0.51 | . | . | . |
| DPYD ---- tag |  | T/T | 0 | 0,00 | 0 | 0,00 | 3 | 0,80 | 2 | 0,74 | 0.68 (0.09-4.95) | 0.71 | 0.69 (0.09-5.21) | 0.72 | . | . | . |
| DPYD ---- tag | rs7414210 | A/A | 41 | 70,69 | 28 | 82,35 | 265 | 70,29 | 201 | 74,44 | 1.00 (.-.) | . | 1.42 (0.91-2.23) | 0.12 | 0.06 | 0.99 | 0.90 |
| DPYD ---- tag |  | A/C | 16 | 27,59 | 5 | 14,71 | 99 | 26,26 | 64 | 23,70 | 2.19 (0.62-7.70) | 0.22 | 1.24 (0.76-2.03) | 0.39 | . | . | . |
| DPYD ---- tag |  | C/C | 1 | 1,72 | 1 | 2,94 | 13 | 3,45 | 5 | 1,85 | 7.43 (0.96-57.39) | 0.06 | 1.50 (0.50-4.46) | 0.47 | . | . | . |
| DPYD ---- tag | rs7530858 | A/A | 46 | 79,31 | 30 | 88,24 | 299 | 79,31 | 202 | 74,81 | 1.00 (.-.) | . | 1.18 (0.76-1.84) | 0.47 | 0.63 | 0.85 | 0.51 |
| DPYD ---- tag |  | A/G | 12 | 20,69 | 4 | 11,76 | 75 | 19,89 | 65 | 24,07 | 0.91 (0.31-2.66) | 0.86 | 1.36 (0.83-2.22) | 0.22 | . | . | . |
| DPYD ---- tag |  | G/G | 0 | 0,00 | 0 | 0,00 | 3 | 0,80 | 3 | 1,11 | 1.94 (0.47-8.05) | 0.36 | 2.29 (0.52-10.09) | 0.27 | . | . | . |
| DPYD ---- tag | rs7544128 | C/C | 30 | 51,72 | 18 | 52,94 | 207 | 54,91 | 152 | 56,30 | 1.00 (.-.) | . | 1.45 (0.84-2.49) | 0.18 | 0.43 | 0.99 | 0.94 |
| DPYD ---- tag |  | C/G | 24 | 41,38 | 12 | 35,29 | 147 | 38,99 | 96 | 35,56 | 1.18 (0.52-2.66) | 0.69 | 1.21 (0.70-2.10) | 0.50 | . | . | . |
| DPYD ---- tag |  | G/G | 4 | 6,90 | 4 | 11,76 | 23 | 6,10 | 22 | 8,15 | 1.50 (0.34-6.70) | 0.59 | 1.43 (0.72-2.83) | 0.31 | . | . | . |
| DPYD ---- tag | rs7545340 | G/G | 33 | 56,90 | 16 | 47,06 | 215 | 57,03 | 151 | 55,93 | 1.00 (.-.) | . | 1.55 (0.86-2.80) | 0.15 | 0.18 | 0.85 | 0.52 |
| DPYD ---- tag |  | G/A | 23 | 39,66 | 15 | 44,12 | 133 | 35,28 | 97 | 35,93 | 1.68 (0.77-3.67) | 0.19 | 1.69 (0.92-3.10) | 0.09 | . | . | . |
| DPYD ---- tag |  | A/A | 2 | 3,45 | 3 | 8,82 | 29 | 7,69 | 22 | 8,15 | 2.83 (0.60-13.45) | 0.19 | 1.79 (0.88-3.63) | 0.11 | . | . | . |
| DPYD ---- tag | rs828054 | C/C | 8 | 13,79 | 5 | 14,71 | 84 | 22,28 | 84 | 31,11 | 1.00 (.-.) | . | 1.42 (0.50-4.06) | 0.51 | 0.97 | 0.97 | 0.78 |
| DPYD ---- tag |  | C/A | 29 | 50,00 | 24 | 70,59 | 191 | 50,66 | 138 | 51,11 | 0.95 (0.31-2.88) | 0.93 | 1.07 (0.38-3.01) | 0.91 | . | . | . |
| DPYD ---- tag |  | A/A | 21 | 36,21 | 5 | 14,71 | 102 | 27,06 | 48 | 17,78 | 0.55 (0.13-2.27) | 0.41 | 0.79 (0.27-2.31) | 0.67 | . | . | . |
| DPYD ---- tag | rs885622 | G/G | 36 | 62,07 | 9 | 26,47 | 153 | 40,58 | 87 | 32,22 | 1.00 (.-.) | . | 1.96 (0.93-4.12) | 0.08 | 0.11 | 0.66 | 0.51 |
| DPYD ---- tag |  | G/A | 18 | 31,03 | 20 | 58,82 | 178 | 47,21 | 136 | 50,37 | 3.10 (1.32-7.28) | 0.01 | 2.55 (1.23-5.29) | 0.01 | . | . | . |
| DPYD ---- tag |  | A/A | 4 | 6,90 | 5 | 14,71 | 46 | 12,20 | 47 | 17,41 | 3.05 (0.77-12.07) | 0.11 | 3.06 (1.41-6.61) | 0.00 | . | . | . |
| DPYD ---- tag | rs9437663 | G/G | 39 | 67,24 | 24 | 70,59 | 244 | 64,72 | 168 | 62,22 | 1.00 (.-.) | . | 1.11 (0.69-1.78) | 0.68 | 0.26 | 0.99 | 0.97 |
| DPYD ---- tag |  | G/A | 15 | 25,86 | 9 | 26,47 | 117 | 31,03 | 87 | 32,22 | 1.01 (0.38-2.71) | 0.98 | 1.33 (0.81-2.20) | 0.26 | . | . | . |
| DPYD ---- tag |  | A/A | 4 | 6,90 | 1 | 2,94 | 16 | 4,24 | 15 | 5,56 | 0.41 (0.05-3.09) | 0.39 | 1.34 (0.65-2.78) | 0.43 | . | . | . |
| DPYS ---- tag | rs13249169 | A/A | 45 | 77,59 | 30 | 88,24 | 296 | 78,51 | 210 | 77,78 | 1.00 (.-.) | . | 1.08 (0.70-1.68) | 0.72 | 0.17 | 0.93 | 0.61 |
| DPYS ---- tag |  | A/T | 13 | 22,41 | 4 | 11,76 | 77 | 20,42 | 56 | 20,74 | 0.50 (0.17-1.46) | 0.21 | 1.14 (0.68-1.90) | 0.61 | . | . | . |
| DPYS ---- tag |  | T/T | 0 | 0,00 | 0 | 0,00 | 4 | 1,06 | 4 | 1,48 | 0.99 (0.36-2.73) | 0.99 | 1.07 (0.36-3.18) | 0.90 | . | . | . |
| DPYS ---- NA | rs13263121 | T/T | 26 | 44,83 | 15 | 44,12 | 169 | 44,83 | 123 | 45,56 | 1.00 (.-.) | . | 1.24 (0.67-2.31) | 0.50 | 0.64 | 0.59 | 0.19 |
| DPYS ---- NA |  | T/A | 29 | 50,00 | 17 | 50,00 | 160 | 42,44 | 118 | 43,70 | 0.79 (0.36-1.72) | 0.55 | 1.08 (0.58-2.00) | 0.81 | . | . | . |
| DPYS ---- NA |  | A/A | 3 | 5,17 | 2 | 5,88 | 48 | 12,73 | 29 | 10,74 | 2.73 (0.60-12.46) | 0.20 | 0.96 (0.47-1.97) | 0.92 | . | . | . |
| DPYS ---- tag | rs16871361 | T/T | 53 | 91,38 | 31 | 91,18 | 339 | 89,92 | 231 | 85,56 | 1.00 (.-.) | . | 1.21 (0.78-1.87) | 0.39 | 0.76 | 0.99 | 0.82 |
| DPYS ---- tag |  | T/C | 5 | 8,62 | 3 | 8,82 | 37 | 9,81 | 39 | 14,44 | 0.89 (0.26-3.01) | 0.85 | 1.32 (0.77-2.25) | 0.31 | . | . | . |
| DPYS ---- tag |  | C/C | 0 | 0,00 | 0 | 0,00 | 1 | 0,27 | 0 | 0,00 | 0.00 (0.00-5E294) | 0.98 | 0.00 (0.00-6E294) | 0.98 | . | . | . |
| DPYS ---- NA | rs17245950 | T/T | 47 | 81,03 | 27 | 79,41 | 283 | 75,07 | 213 | 78,89 | 1.00 (.-.) | . | 1.30 (0.83-2.04) | 0.26 | 0.43 | 0.93 | 0.61 |
| DPYS ---- NA |  | T/A | 10 | 17,24 | 6 | 17,65 | 88 | 23,34 | 54 | 20,00 | 0.85 (0.29-2.52) | 0.77 | 1.12 (0.66-1.88) | 0.68 | . | . | . |
| DPYS ---- NA |  | A/A | 1 | 1,72 | 1 | 2,94 | 6 | 1,59 | 3 | 1,11 | 8.56 (1.08-68.03) | 0.04 | 0.90 (0.20-3.96) | 0.89 | . | . | . |
| DPYS ---- NA | rs2253336 | A/A | 49 | 84,48 | 29 | 85,29 | 296 | 78,51 | 218 | 80,74 | 1.00 (.-.) | . | 1.22 (0.79-1.89) | 0.36 | 0.68 | 0.99 | 0.67 |
| DPYS ---- NA |  | A/G | 8 | 13,79 | 5 | 14,71 | 77 | 20,42 | 49 | 18,15 | 0.68 (0.16-2.94) | 0.60 | 1.10 (0.66-1.83) | 0.70 | . | . | . |
| DPYS ---- NA |  | G/G | 1 | 1,72 | 0 | 0,00 | 4 | 1,06 | 3 | 1,11 | 1.00 (0.24-4.12) | 1.00 | 1.22 (0.28-5.34) | 0.79 | . | . | . |
| DPYS ---- tag | rs2280010 | C/C | 33 | 56,90 | 14 | 41,18 | 233 | 61,80 | 142 | 52,59 | 1.00 (.-.) | . | 1.15 (0.62-2.14) | 0.66 | 0.37 | 0.59 | 0.19 |
| DPYS ---- tag |  | C/T | 21 | 36,21 | 17 | 50,00 | 132 | 35,01 | 111 | 41,11 | 1.35 (0.61-3.01) | 0.46 | 1.75 (0.94-3.26) | 0.08 | . | . | . |
| DPYS ---- tag |  | T/T | 4 | 6,90 | 3 | 8,82 | 12 | 3,18 | 17 | 6,30 | 1.02 (0.28-3.75) | 0.98 | 2.18 (1.01-4.74) | 0.05 | . | . | . |
| DPYS ---- tag | rs2333874 | T/T | 21 | 36,21 | 16 | 47,06 | 159 | 42,18 | 116 | 42,96 | 1.00 (.-.) | . | 1.32 (0.71-2.46) | 0.37 | 1.00 | 0.99 | 0.80 |
| DPYS ---- tag |  | T/G | 28 | 48,28 | 16 | 47,06 | 170 | 45,09 | 123 | 45,56 | 1.02 (0.46-2.23) | 0.97 | 1.14 (0.62-2.12) | 0.67 | . | . | . |
| DPYS ---- tag |  | G/G | 9 | 15,52 | 2 | 5,88 | 48 | 12,73 | 31 | 11,48 | 0.90 (0.20-4.09) | 0.89 | 1.42 (0.70-2.90) | 0.33 | . | . | . |
| DPYS ---- NA | rs2669429 | C/C | 15 | 25,86 | 8 | 23,53 | 130 | 34,48 | 70 | 25,93 | 1.00 (.-.) | . | 0.66 (0.30-1.48) | 0.32 | 0.19 | 0.85 | 0.40 |
| DPYS ---- NA |  | C/T | 31 | 53,45 | 15 | 44,12 | 177 | 46,95 | 153 | 56,67 | 0.68 (0.26-1.75) | 0.42 | 1.09 (0.50-2.38) | 0.82 | . | . | . |
| DPYS ---- NA |  | T/T | 12 | 20,69 | 11 | 32,35 | 70 | 18,57 | 47 | 17,41 | 0.72 (0.26-1.98) | 0.53 | 1.00 (0.44-2.28) | 1.00 | . | . | . |
| DPYS ---- tag | rs2669434 | C/C | 32 | 55,17 | 21 | 61,76 | 179 | 47,48 | 149 | 55,19 | 1.00 (.-.) | . | 1.16 (0.70-1.92) | 0.57 | 0.82 | 0.59 | 0.19 |
| DPYS ---- tag |  | C/A | 21 | 36,21 | 8 | 23,53 | 166 | 44,03 | 102 | 37,78 | 0.51 (0.20-1.29) | 0.15 | 0.96 (0.57-1.62) | 0.89 | . | . | . |
| DPYS ---- tag |  | A/A | 5 | 8,62 | 5 | 14,71 | 32 | 8,49 | 19 | 7,04 | 1.43 (0.47-4.36) | 0.53 | 0.87 (0.43-1.76) | 0.69 | . | . | . |
| DPYS ---- tag | rs2853142 | T/T | 30 | 51,72 | 12 | 35,29 | 137 | 36,34 | 115 | 42,59 | 1.00 (.-.) | . | 1.94 (1.03-3.66) | 0.04 | 0.13 | 0.59 | 0.19 |
| DPYS ---- tag |  | T/C | 23 | 39,66 | 17 | 50,00 | 196 | 51,99 | 119 | 44,07 | 1.38 (0.62-3.10) | 0.43 | 1.19 (0.63-2.24) | 0.60 | . | . | . |
| DPYS ---- tag |  | C/C | 5 | 8,62 | 5 | 14,71 | 44 | 11,67 | 36 | 13,33 | 1.60 (0.43-6.00) | 0.49 | 1.60 (0.78-3.26) | 0.20 | . | . | . |
| DPYS ---- NA | rs2853145 | A/A | 47 | 81,03 | 21 | 61,76 | 244 | 64,72 | 187 | 69,26 | 1.00 (.-.) | . | 1.61 (0.96-2.70) | 0.07 | 0.06 | 0.89 | 0.54 |
| DPYS ---- NA |  | A/C | 11 | 18,97 | 11 | 32,35 | 124 | 32,89 | 73 | 27,04 | 1.49 (0.65-3.41) | 0.34 | 1.18 (0.68-2.05) | 0.55 | . | . | . |
| DPYS ---- NA |  | C/C | 0 | 0,00 | 2 | 5,88 | 9 | 2,39 | 10 | 3,70 | 21.64 (2.72-172.0) | 0.00 | 2.22 (0.90-5.48) | 0.08 | . | . | . |
| DPYS ---- tag | rs2853149 | G/G | 12 | 20,69 | 10 | 29,41 | 102 | 27,06 | 72 | 26,67 | 1.00 (.-.) | . | 1.04 (0.46-2.32) | 0.93 | 0.41 | 0.97 | 0.67 |
| DPYS ---- tag |  | G/A | 28 | 48,28 | 19 | 55,88 | 188 | 49,87 | 137 | 50,74 | 0.90 (0.36-2.21) | 0.81 | 1.06 (0.48-2.34) | 0.88 | . | . | . |
| DPYS ---- tag |  | A/A | 18 | 31,03 | 5 | 14,71 | 87 | 23,08 | 61 | 22,59 | 0.75 (0.23-2.42) | 0.63 | 1.32 (0.58-2.98) | 0.51 | . | . | . |
| DPYS ---- tag | rs2853154 | T/T | 38 | 65,52 | 22 | 64,71 | 212 | 56,23 | 153 | 56,67 | 1.00 (.-.) | . | 1.44 (0.87-2.38) | 0.15 | 0.53 | 0.59 | 0.19 |
| DPYS ---- tag |  | T/C | 18 | 31,03 | 10 | 29,41 | 149 | 39,52 | 103 | 38,15 | 1.20 (0.53-2.69) | 0.66 | 1.15 (0.69-1.93) | 0.59 | . | . | . |
| DPYS ---- tag |  | C/C | 2 | 3,45 | 2 | 5,88 | 16 | 4,24 | 14 | 5,19 | 0.00 (0.00-7E240) | 0.97 | 1.03 (0.49-2.15) | 0.95 | . | . | . |
| DPYS ---- tag | rs2853161 | A/A | 17 | 29,31 | 6 | 17,65 | 106 | 28,12 | 67 | 24,81 | 1.00 (.-.) | . | 1.42 (0.59-3.43) | 0.43 | 0.99 | 0.93 | 0.61 |
| DPYS ---- tag |  | A/G | 33 | 56,90 | 22 | 64,71 | 192 | 50,93 | 144 | 53,33 | 1.29 (0.50-3.30) | 0.60 | 1.45 (0.62-3.40) | 0.39 | . | . | . |
| DPYS ---- tag |  | G/G | 8 | 13,79 | 6 | 17,65 | 79 | 20,95 | 59 | 21,85 | 0.91 (0.22-3.81) | 0.90 | 1.46 (0.60-3.51) | 0.40 | . | . | . |
| DPYS ---- NA | rs2959024 | T/T | 33 | 56,90 | 14 | 41,18 | 199 | 52,79 | 125 | 46,30 | 1.00 (.-.) | . | 0.99 (0.54-1.83) | 0.98 | 0.34 | 0.98 | 0.67 |
| DPYS ---- NA |  | T/G | 21 | 36,21 | 17 | 50,00 | 149 | 39,52 | 124 | 45,93 | 0.98 (0.45-2.14) | 0.95 | 1.44 (0.78-2.65) | 0.24 | . | . | . |
| DPYS ---- NA |  | G/G | 4 | 6,90 | 3 | 8,82 | 29 | 7,69 | 21 | 7,78 | 0.69 (0.15-3.22) | 0.64 | 1.18 (0.56-2.47) | 0.66 | . | . | . |
| DPYS ---- NA | rs2959025 | A/A | 25 | 43,10 | 13 | 38,24 | 162 | 42,97 | 105 | 38,89 | 1.00 (.-.) | . | 1.17 (0.62-2.23) | 0.62 | 0.55 | 0.93 | 0.61 |
| DPYS ---- NA |  | A/G | 26 | 44,83 | 17 | 50,00 | 164 | 43,50 | 127 | 47,04 | 1.33 (0.59-2.97) | 0.49 | 1.46 (0.78-2.75) | 0.24 | . | . | . |
| DPYS ---- NA |  | G/G | 7 | 12,07 | 4 | 11,76 | 51 | 13,53 | 38 | 14,07 | 0.60 (0.17-2.19) | 0.44 | 1.23 (0.61-2.46) | 0.57 | . | . | . |
| DPYS ---- tag | rs2959026 | G/G | 17 | 29,31 | 13 | 38,24 | 132 | 35,01 | 96 | 35,56 | 1.00 (.-.) | . | 0.99 (0.50-1.96) | 0.99 | 0.55 | 0.85 | 0.40 |
| DPYS ---- tag |  | G/A | 28 | 48,28 | 16 | 47,06 | 178 | 47,21 | 134 | 49,63 | 0.76 (0.34-1.74) | 0.52 | 1.08 (0.55-2.10) | 0.83 | . | . | . |
| DPYS ---- tag |  | A/A | 13 | 22,41 | 5 | 14,71 | 67 | 17,77 | 40 | 14,81 | 0.75 (0.23-2.43) | 0.63 | 0.96 (0.46-2.00) | 0.92 | . | . | . |
| DPYS ---- NA | rs3133278 | T/T | 32 | 55,17 | 18 | 52,94 | 191 | 50,66 | 138 | 51,11 | 1.00 (.-.) | . | 1.39 (0.78-2.47) | 0.26 | 0.64 | 0.83 | 0.37 |
| DPYS ---- NA |  | T/C | 23 | 39,66 | 16 | 47,06 | 158 | 41,91 | 116 | 42,96 | 1.12 (0.53-2.37) | 0.76 | 1.26 (0.71-2.24) | 0.43 | . | . | . |
| DPYS ---- NA |  | C/C | 3 | 5,17 | 0 | 0,00 | 28 | 7,43 | 16 | 5,93 | 0.00 (0.00-4E298) | 0.98 | 0.93 (0.43-1.98) | 0.84 | . | . | . |
| DPYS ---- tag | rs3750187 | G/G | 42 | 72,41 | 20 | 58,82 | 236 | 62,60 | 169 | 62,59 | 1.00 (.-.) | . | 1.63 (0.95-2.80) | 0.08 | 0.08 | 0.66 | 0.24 |
| DPYS ---- tag |  | G/A | 14 | 24,14 | 13 | 38,24 | 128 | 33,95 | 92 | 34,07 | 1.64 (0.75-3.61) | 0.22 | 1.39 (0.80-2.43) | 0.25 | . | . | . |
| DPYS ---- tag |  | A/A | 2 | 3,45 | 1 | 2,94 | 13 | 3,45 | 9 | 3,33 | 3.90 (0.50-30.51) | 0.20 | 1.54 (0.63-3.73) | 0.34 | . | . | . |
| DPYS ---- tag | rs3793357 | T/T | 52 | 89,66 | 30 | 88,24 | 332 | 88,06 | 244 | 90,37 | 1.00 (.-.) | . | 1.13 (0.73-1.75) | 0.59 | 0.43 | 0.99 | 0.80 |
| DPYS ---- tag |  | T/G | 6 | 10,34 | 3 | 8,82 | 43 | 11,41 | 25 | 9,26 | 0.44 (0.13-1.52) | 0.19 | 1.29 (0.70-2.38) | 0.41 | . | . | . |
| DPYS ---- tag |  | G/G | 0 | 0,00 | 1 | 2,94 | 2 | 0,53 | 1 | 0,37 | 4.12 (0.53-31.74) | 0.18 | 1.74 (0.23-13.21) | 0.59 | . | . | . |
| DPYS ---- tag | rs3793358 | G/G | 50 | 86,21 | 28 | 82,35 | 287 | 76,13 | 216 | 80,00 | 1.00 (.-.) | . | 1.40 (0.90-2.20) | 0.14 | 0.48 | 0.66 | 0.22 |
| DPYS ---- tag |  | G/A | 8 | 13,79 | 5 | 14,71 | 89 | 23,61 | 49 | 18,15 | 1.16 (0.39-3.42) | 0.79 | 0.90 (0.53-1.52) | 0.69 | . | . | . |
| DPYS ---- tag |  | A/A | 0 | 0,00 | 1 | 2,94 | 1 | 0,27 | 5 | 1,85 | 2.27 (0.81-6.35) | 0.12 | 3.18 (1.05-9.67) | 0.04 | . | . | . |
| DPYS ---- tag | rs6468924 | C/C | 37 | 63,79 | 24 | 70,59 | 221 | 58,62 | 175 | 64,81 | 1.00 (.-.) | . | 1.16 (0.72-1.87) | 0.55 | 0.81 | 0.83 | 0.37 |
| DPYS ---- tag |  | C/T | 19 | 32,76 | 6 | 17,65 | 136 | 36,07 | 85 | 31,48 | 0.46 (0.15-1.35) | 0.16 | 1.02 (0.61-1.70) | 0.94 | . | . | . |
| DPYS ---- tag |  | T/T | 2 | 3,45 | 4 | 11,76 | 20 | 5,31 | 10 | 3,70 | 1.60 (0.52-4.92) | 0.41 | 0.98 (0.40-2.38) | 0.96 | . | . | . |
| DUT ---- tag | rs8025164 | G/G | 45 | 77,59 | 21 | 61,76 | 251 | 66,58 | 193 | 71,48 | 1.00 (.-.) | . | 1.54 (0.94-2.52) | 0.09 | 0.26 | 0.99 | 0.89 |
| DUT ---- tag |  | G/A | 10 | 17,24 | 13 | 38,24 | 116 | 30,77 | 64 | 23,70 | 1.80 (0.80-4.05) | 0.16 | 1.08 (0.63-1.87) | 0.78 | . | . | . |
| DUT ---- tag |  | A/A | 3 | 5,17 | 0 | 0,00 | 10 | 2,65 | 13 | 4,81 | 0.00 (0.00-1E243) | 0.97 | 3.40 (1.58-7.32) | 0.00 | . | . | . |
| EHMT1 ---- tag | rs10780190 | C/C | 54 | 93,10 | 30 | 88,24 | 335 | 88,86 | 243 | 90,00 | 1.00 (.-.) | . | 1.35 (0.87-2.08) | 0.18 | 0.12 | 0.99 | 0.97 |
| EHMT1 ---- tag |  | C/T | 3 | 5,17 | 4 | 11,76 | 41 | 10,88 | 27 | 10,00 | 2.75 (0.77-9.83) | 0.12 | 1.16 (0.64-2.10) | 0.64 | . | . | . |
| EHMT1 ---- tag |  | T/T | 1 | 1,72 | 0 | 0,00 | 1 | 0,27 | 0 | 0,00 | 0.00 (0.00-6E237) | 0.97 | 0.00 (0.00-8E237) | 0.97 | . | . | . |
| EHMT1 ---- tag | rs10867083 | G/G | 23 | 39,66 | 17 | 50,00 | 181 | 48,01 | 123 | 45,56 | 1.00 (.-.) | . | 1.00 (0.55-1.84) | 1.00 | 0.76 | 0.99 | 0.97 |
| EHMT1 ---- tag |  | G/A | 31 | 53,45 | 14 | 41,18 | 162 | 42,97 | 124 | 45,93 | 0.62 (0.27-1.43) | 0.26 | 1.04 (0.58-1.89) | 0.89 | . | . | . |
| EHMT1 ---- tag |  | A/A | 4 | 6,90 | 3 | 8,82 | 34 | 9,02 | 23 | 8,52 | 0.92 (0.25-3.37) | 0.90 | 0.68 (0.32-1.42) | 0.30 | . | . | . |
| EHMT1 ---- tag | rs11137190 | C/C | 32 | 55,17 | 17 | 50,00 | 197 | 52,25 | 137 | 50,74 | 1.00 (.-.) | . | 1.19 (0.70-2.05) | 0.52 | 0.89 | 0.85 | 0.81 |
| EHMT1 ---- tag |  | C/G | 22 | 37,93 | 16 | 47,06 | 150 | 39,79 | 114 | 42,22 | 1.31 (0.59-2.90) | 0.51 | 1.54 (0.90-2.63) | 0.12 | . | . | . |
| EHMT1 ---- tag |  | G/G | 4 | 6,90 | 1 | 2,94 | 30 | 7,96 | 19 | 7,04 | 0.64 (0.08-4.86) | 0.66 | 1.16 (0.57-2.36) | 0.69 | . | . | . |
| EHMT1 ---- tag | rs3123510 | G/G | 23 | 39,66 | 12 | 35,29 | 148 | 39,26 | 100 | 37,04 | 1.00 (.-.) | . | 1.33 (0.71-2.48) | 0.37 | 0.68 | 0.91 | 0.90 |
| EHMT1 ---- tag |  | G/A | 28 | 48,28 | 17 | 50,00 | 172 | 45,62 | 136 | 50,37 | 1.37 (0.60-3.16) | 0.46 | 1.56 (0.85-2.87) | 0.15 | . | . | . |
| EHMT1 ---- tag |  | A/A | 7 | 12,07 | 5 | 14,71 | 57 | 15,12 | 34 | 12,59 | 1.26 (0.40-3.95) | 0.70 | 1.33 (0.66-2.67) | 0.42 | . | . | . |
| EHMT1 ---- candidate literature | rs3125795 | G/G | 53 | 91,38 | 30 | 88,24 | 336 | 89,12 | 242 | 89,63 | 1.00 (.-.) | . | 1.34 (0.87-2.07) | 0.19 | 0.14 | 0.94 | 0.97 |
| EHMT1 ---- candidate literature |  | G/T | 4 | 6,90 | 4 | 11,76 | 40 | 10,61 | 28 | 10,37 | 2.76 (0.77-9.84) | 0.12 | 1.21 (0.67-2.19) | 0.52 | . | . | . |
| EHMT1 ---- candidate literature |  | T/T | 1 | 1,72 | 0 | 0,00 | 1 | 0,27 | 0 | 0,00 | 0.00 (0.00-9E237) | 0.97 | 0.00 (0.00-1E238) | 0.97 | . | . | . |
| EHMT1 ---- tag | rs4573359 | G/G | 49 | 84,48 | 29 | 85,29 | 317 | 84,08 | 228 | 84,44 | 1.00 (.-.) | . | 1.23 (0.79-1.91) | 0.36 | 0.98 | 0.99 | 0.97 |
| EHMT1 ---- tag |  | G/T | 9 | 15,52 | 5 | 14,71 | 57 | 15,12 | 42 | 15,56 | 1.05 (0.31-3.54) | 0.94 | 1.35 (0.80-2.26) | 0.26 | . | . | . |
| EHMT1 ---- tag |  | T/T | 0 | 0,00 | 0 | 0,00 | 3 | 0,80 | 0 | 0,00 | 0.00 (0.00-1E225) | 0.97 | 0.00 (0.00-2E225) | 0.97 | . | . | . |
| EHMT1 ---- candidate literature | rs4634736 | G/G | 49 | 84,48 | 29 | 85,29 | 316 | 83,82 | 229 | 84,81 | 1.00 (.-.) | . | 1.24 (0.80-1.92) | 0.34 | 0.95 | 0.99 | 0.97 |
| EHMT1 ---- candidate literature |  | G/A | 9 | 15,52 | 5 | 14,71 | 58 | 15,38 | 41 | 15,19 | 1.05 (0.31-3.54) | 0.94 | 1.31 (0.78-2.21) | 0.30 | . | . | . |
| EHMT1 ---- candidate literature |  | A/A | 0 | 0,00 | 0 | 0,00 | 3 | 0,80 | 0 | 0,00 | 0.00 (0.00-2E225) | 0.97 | 0.00 (0.00-2E225) | 0.97 | . | . | . |
| EHMT1 ---- tag | rs4876902 | C/C | 43 | 74,14 | 18 | 52,94 | 239 | 63,40 | 166 | 61,48 | 1.00 (.-.) | . | 1.36 (0.80-2.31) | 0.26 | 0.73 | 0.81 | 0.58 |
| EHMT1 ---- tag |  | C/T | 13 | 22,41 | 16 | 47,06 | 123 | 32,63 | 92 | 34,07 | 1.64 (0.76-3.50) | 0.21 | 1.66 (0.96-2.88) | 0.07 | . | . | . |
| EHMT1 ---- tag |  | T/T | 2 | 3,45 | 0 | 0,00 | 15 | 3,98 | 12 | 4,44 | 0.00 (0.00-6E289) | 0.97 | 1.49 (0.66-3.36) | 0.33 | . | . | . |
| EHMT1 ---- tag | rs4876904 | T/T | 19 | 32,76 | 12 | 35,29 | 108 | 28,65 | 81 | 30,00 | 1.00 (.-.) | . | 1.11 (0.54-2.27) | 0.78 | 0.35 | 0.99 | 0.97 |
| EHMT1 ---- tag |  | T/G | 24 | 41,38 | 13 | 38,24 | 188 | 49,87 | 127 | 47,04 | 1.19 (0.49-2.90) | 0.70 | 1.14 (0.56-2.29) | 0.72 | . | . | . |
| EHMT1 ---- tag |  | G/G | 15 | 25,86 | 9 | 26,47 | 81 | 21,49 | 62 | 22,96 | 0.65 (0.24-1.72) | 0.38 | 1.17 (0.56-2.42) | 0.68 | . | . | . |
| EHMT1 ---- tag | rs7390244 | A/A | 15 | 25,86 | 8 | 23,53 | 88 | 23,34 | 71 | 26,30 | 1.00 (.-.) | . | 1.31 (0.55-3.11) | 0.54 | 0.71 | 0.99 | 0.97 |
| EHMT1 ---- tag |  | A/G | 30 | 51,72 | 18 | 52,94 | 186 | 49,34 | 127 | 47,04 | 1.18 (0.45-3.11) | 0.73 | 1.57 (0.68-3.64) | 0.29 | . | . | . |
| EHMT1 ---- tag |  | G/G | 13 | 22,41 | 8 | 23,53 | 103 | 27,32 | 72 | 26,67 | 1.19 (0.39-3.68) | 0.76 | 1.23 (0.52-2.92) | 0.64 | . | . | . |
| EHMT1 ---- tag | rs9314635 | G/G | 29 | 50,00 | 13 | 38,24 | 159 | 42,18 | 105 | 38,89 | 1.00 (.-.) | . | 1.33 (0.73-2.42) | 0.35 | 0.72 | 0.68 | 0.58 |
| EHMT1 ---- tag |  | G/T | 22 | 37,93 | 18 | 52,94 | 171 | 45,36 | 134 | 49,63 | 1.58 (0.72-3.45) | 0.25 | 1.63 (0.91-2.92) | 0.10 | . | . | . |
| EHMT1 ---- tag |  | T/T | 7 | 12,07 | 3 | 8,82 | 47 | 12,47 | 31 | 11,48 | 1.00 (0.22-4.53) | 1.00 | 1.38 (0.69-2.74) | 0.36 | . | . | . |
| EHMT2 ---- candidate/tag | rs2736428 | G/G | 20 | 34,48 | 13 | 38,24 | 146 | 38,73 | 120 | 44,44 | 1.00 (.-.) | . | 1.13 (0.58-2.20) | 0.73 | 0.30 | 0.89 | 0.31 |
| EHMT2 ---- candidate/tag |  | G/A | 26 | 44,83 | 18 | 52,94 | 185 | 49,07 | 116 | 42,96 | 0.97 (0.44-2.15) | 0.94 | 0.99 (0.51-1.93) | 0.98 | . | . | . |
| EHMT2 ---- candidate/tag |  | A/A | 12 | 20,69 | 3 | 8,82 | 46 | 12,20 | 34 | 12,59 | 0.16 (0.02-1.28) | 0.08 | 0.82 (0.39-1.72) | 0.60 | . | . | . |
| EHMT2 ---- tag | rs9267649 | G/G | 47 | 81,03 | 24 | 70,59 | 270 | 71,62 | 195 | 72,22 | 1.00 (.-.) | . | 1.38 (0.85-2.24) | 0.19 | 0.45 | 0.85 | 0.31 |
| EHMT2 ---- tag |  | G/A | 8 | 13,79 | 10 | 29,41 | 94 | 24,93 | 67 | 24,81 | 1.59 (0.69-3.67) | 0.28 | 1.34 (0.79-2.27) | 0.27 | . | . | . |
| EHMT2 ---- tag |  | A/A | 3 | 5,17 | 0 | 0,00 | 13 | 3,45 | 8 | 2,96 | 0.00 (0.00-I) | 0.98 | 1.65 (0.65-4.20) | 0.29 | . | . | . |
| FDXR ---- NA | rs2070918 | T/T | 26 | 44,83 | 11 | 32,35 | 195 | 51,72 | 127 | 47,04 | 1.00 (.-.) | . | 2.02 (0.97-4.20) | 0.06 | 0.06 | 0.85 | 0.68 |
| FDXR ---- NA |  | T/C | 26 | 44,83 | 18 | 52,94 | 148 | 39,26 | 107 | 39,63 | 2.28 (0.95-5.47) | 0.07 | 2.26 (1.08-4.71) | 0.03 | . | . | . |
| FDXR ---- NA |  | C/C | 6 | 10,34 | 5 | 14,71 | 34 | 9,02 | 36 | 13,33 | 3.68 (1.16-11.66) | 0.03 | 2.51 (1.14-5.55) | 0.02 | . | . | . |
| FDXR ---- tag | rs509911 | A/A | 34 | 58,62 | 20 | 58,82 | 242 | 64,19 | 172 | 63,70 | 1.00 (.-.) | . | 1.56 (0.91-2.66) | 0.11 | 0.12 | 0.95 | 0.68 |
| FDXR ---- tag |  | A/G | 19 | 32,76 | 12 | 35,29 | 117 | 31,03 | 87 | 32,22 | 1.97 (0.88-4.39) | 0.10 | 1.68 (0.96-2.93) | 0.07 | . | . | . |
| FDXR ---- tag |  | G/G | 5 | 8,62 | 2 | 5,88 | 18 | 4,77 | 11 | 4,07 | 2.32 (0.51-10.61) | 0.28 | 1.38 (0.61-3.09) | 0.44 | . | . | . |
| FDXR ---- NA | rs689882 | G/G | 27 | 46,55 | 16 | 47,06 | 205 | 54,38 | 142 | 52,59 | 1.00 (.-.) | . | 1.45 (0.81-2.62) | 0.21 | 0.46 | 0.99 | 0.68 |
| FDXR ---- NA |  | G/A | 23 | 39,66 | 15 | 44,12 | 147 | 38,99 | 112 | 41,48 | 1.39 (0.63-3.09) | 0.42 | 1.50 (0.82-2.74) | 0.19 | . | . | . |
| FDXR ---- NA |  | A/A | 8 | 13,79 | 3 | 8,82 | 25 | 6,63 | 16 | 5,93 | 1.20 (0.33-4.32) | 0.78 | 1.14 (0.54-2.43) | 0.73 | . | . | . |
| FDXR ---- NA | rs689895 | G/G | 31 | 53,45 | 17 | 50,00 | 192 | 50,93 | 131 | 48,52 | 1.00 (.-.) | . | 1.61 (0.91-2.87) | 0.10 | 0.12 | 0.99 | 0.68 |
| FDXR ---- NA |  | G/C | 22 | 37,93 | 14 | 41,18 | 154 | 40,85 | 123 | 45,56 | 1.81 (0.80-4.07) | 0.16 | 1.67 (0.94-2.96) | 0.08 | . | . | . |
| FDXR ---- NA |  | C/C | 5 | 8,62 | 3 | 8,82 | 31 | 8,22 | 16 | 5,93 | 1.70 (0.47-6.09) | 0.42 | 1.03 (0.48-2.20) | 0.94 | . | . | . |
| FOLH1 ---- candidate literature | rs10839236 | T/T | 22 | 37,93 | 14 | 41,18 | 165 | 43,77 | 101 | 37,41 | 1.00 (.-.) | . | 0.88 (0.44-1.76) | 0.72 | 0.16 | 0.93 | 0.79 |
| FOLH1 ---- candidate literature |  | T/C | 29 | 50,00 | 16 | 47,06 | 164 | 43,50 | 136 | 50,37 | 0.82 (0.35-1.88) | 0.64 | 1.08 (0.55-2.13) | 0.82 | . | . | . |
| FOLH1 ---- candidate literature |  | C/C | 7 | 12,07 | 4 | 11,76 | 48 | 12,73 | 33 | 12,22 | 0.48 (0.14-1.56) | 0.22 | 0.94 (0.45-1.98) | 0.88 | . | . | . |
| FOLH1 ---- tag | rs16906190 | A/A | 44 | 75,86 | 30 | 88,24 | 308 | 81,70 | 225 | 83,33 | 1.00 (.-.) | . | 1.15 (0.73-1.80) | 0.54 | 0.48 | 0.99 | 0.79 |
| FOLH1 ---- tag |  | A/G | 14 | 24,14 | 4 | 11,76 | 63 | 16,71 | 41 | 15,19 | 0.70 (0.23-2.09) | 0.52 | 1.27 (0.74-2.18) | 0.39 | . | . | . |
| FOLH1 ---- tag |  | G/G | 0 | 0,00 | 0 | 0,00 | 6 | 1,59 | 4 | 1,48 | 0.87 (0.28-2.77) | 0.82 | 1.00 (0.29-3.43) | 1.00 | . | . | . |
| FOLH1 ---- candidate | rs202676 | T/T | 33 | 56,90 | 23 | 67,65 | 239 | 63,40 | 166 | 61,48 | 1.00 (.-.) | . | 1.09 (0.64-1.87) | 0.75 | 0.38 | 0.99 | 0.79 |
| FOLH1 ---- candidate |  | T/C | 24 | 41,38 | 10 | 29,41 | 119 | 31,56 | 90 | 33,33 | 0.88 (0.40-1.95) | 0.75 | 1.19 (0.68-2.07) | 0.55 | . | . | . |
| FOLH1 ---- candidate |  | C/C | 1 | 1,72 | 1 | 2,94 | 19 | 5,04 | 14 | 5,19 | 0.51 (0.07-3.99) | 0.52 | 1.36 (0.65-2.85) | 0.42 | . | . | . |
| FOLH1 ---- tag | rs202680 | A/A | 29 | 50,00 | 22 | 64,71 | 215 | 57,03 | 149 | 55,19 | 1.00 (.-.) | . | 1.13 (0.65-1.95) | 0.67 | 0.49 | 0.99 | 0.79 |
| FOLH1 ---- tag |  | A/T | 28 | 48,28 | 11 | 32,35 | 140 | 37,14 | 104 | 38,52 | 0.92 (0.42-2.00) | 0.82 | 1.16 (0.66-2.05) | 0.60 | . | . | . |
| FOLH1 ---- tag |  | T/T | 1 | 1,72 | 1 | 2,94 | 22 | 5,84 | 17 | 6,30 | 0.52 (0.07-4.09) | 0.54 | 1.33 (0.65-2.70) | 0.44 | . | . | . |
| FOLH1 ---- candidate literature | rs202720 | G/G | 32 | 55,17 | 23 | 67,65 | 240 | 63,66 | 166 | 61,48 | 1.00 (.-.) | . | 1.06 (0.62-1.80) | 0.84 | 0.30 | 0.99 | 0.79 |
| FOLH1 ---- candidate literature |  | G/C | 25 | 43,10 | 10 | 29,41 | 118 | 31,30 | 90 | 33,33 | 0.82 (0.37-1.80) | 0.61 | 1.16 (0.66-2.02) | 0.61 | . | . | . |
| FOLH1 ---- candidate literature |  | C/C | 1 | 1,72 | 1 | 2,94 | 19 | 5,04 | 14 | 5,19 | 0.49 (0.06-3.85) | 0.50 | 1.32 (0.63-2.76) | 0.47 | . | . | . |
| FOLH1 ---- tag | rs2299650 | G/G | 20 | 34,48 | 15 | 44,12 | 163 | 43,24 | 101 | 37,41 | 1.00 (.-.) | . | 0.79 (0.41-1.53) | 0.48 | 0.09 | 0.96 | 0.79 |
| FOLH1 ---- tag |  | G/T | 31 | 53,45 | 15 | 44,12 | 166 | 44,03 | 136 | 50,37 | 0.66 (0.29-1.52) | 0.33 | 0.95 (0.49-1.82) | 0.88 | . | . | . |
| FOLH1 ---- tag |  | T/T | 7 | 12,07 | 4 | 11,76 | 48 | 12,73 | 33 | 12,22 | 0.42 (0.13-1.36) | 0.15 | 0.84 (0.41-1.71) | 0.62 | . | . | . |
| FOLH1 ---- tag | rs617528 | G/G | 45 | 77,59 | 25 | 73,53 | 303 | 80,37 | 209 | 77,41 | 1.00 (.-.) | . | 1.14 (0.71-1.81) | 0.59 | 0.42 | 0.93 | 0.79 |
| FOLH1 ---- tag |  | G/A | 12 | 20,69 | 7 | 20,59 | 70 | 18,57 | 58 | 21,48 | 0.90 (0.33-2.42) | 0.83 | 1.29 (0.77-2.15) | 0.34 | . | . | . |
| FOLH1 ---- tag |  | A/A | 1 | 1,72 | 2 | 5,88 | 4 | 1,06 | 3 | 1,11 | 0.57 (0.13-2.48) | 0.45 | 0.75 (0.22-2.57) | 0.65 | . | . | . |
| FOLH1 ---- tag | rs663877 | T/T | 46 | 79,31 | 26 | 76,47 | 300 | 79,58 | 206 | 76,30 | 1.00 (.-.) | . | 1.16 (0.70-1.90) | 0.57 | 0.60 | 0.93 | 0.79 |
| FOLH1 ---- tag |  | T/G | 12 | 20,69 | 8 | 23,53 | 72 | 19,10 | 60 | 22,22 | 0.88 (0.38-2.05) | 0.77 | 1.30 (0.75-2.23) | 0.35 | . | . | . |
| FOLH1 ---- tag |  | G/G | 0 | 0,00 | 0 | 0,00 | 5 | 1,33 | 4 | 1,48 | 1.16 (0.42-3.25) | 0.77 | 1.34 (0.45-3.99) | 0.60 | . | . | . |
| FOLH1 ---- tag | rs670776 | A/A | 33 | 56,90 | 23 | 67,65 | 239 | 63,40 | 166 | 61,48 | 1.00 (.-.) | . | 1.09 (0.64-1.87) | 0.75 | 0.38 | 0.99 | 0.79 |
| FOLH1 ---- tag |  | A/T | 24 | 41,38 | 10 | 29,41 | 119 | 31,56 | 90 | 33,33 | 0.88 (0.40-1.95) | 0.75 | 1.19 (0.68-2.07) | 0.55 | . | . | . |
| FOLH1 ---- tag |  | T/T | 1 | 1,72 | 1 | 2,94 | 19 | 5,04 | 14 | 5,19 | 0.51 (0.07-3.99) | 0.52 | 1.36 (0.65-2.85) | 0.42 | . | . | . |
| FOLH1 ---- tag | rs7124497 | G/G | 54 | 93,10 | 33 | 97,06 | 349 | 92,57 | 250 | 92,59 | 1.00 (.-.) | . | 1.26 (0.83-1.92) | 0.27 | 0.68 | 0.99 | 0.80 |
| FOLH1 ---- tag |  | G/A | 4 | 6,90 | 1 | 2,94 | 28 | 7,43 | 20 | 7,41 | 1.00 (.-.) | . | 1.26 (0.83-1.92) | 0.27 | . | . | . |
| FOLH1 ---- tag |  | A/A | 0 | 0,00 | 0 | 0,00 | 0 | 0,00 | 0 | 0,00 | 1.35 (0.18-10.19) | 0.77 | 1.07 (0.56-2.06) | 0.84 | . | . | . |
| FOLR1 ---- tag | rs651646 | T/T | 18 | 31,03 | 9 | 26,47 | 129 | 34,22 | 86 | 31,85 | 1.00 (.-.) | . | 1.57 (0.71-3.49) | 0.27 | 0.32 | 0.99 | 0.68 |
| FOLR1 ---- tag |  | T/A | 29 | 50,00 | 17 | 50,00 | 180 | 47,75 | 121 | 44,81 | 1.27 (0.49-3.27) | 0.62 | 1.60 (0.72-3.52) | 0.25 | . | . | . |
| FOLR1 ---- tag |  | A/A | 11 | 18,97 | 8 | 23,53 | 68 | 18,04 | 63 | 23,33 | 1.86 (0.66-5.28) | 0.24 | 1.67 (0.74-3.75) | 0.21 | . | . | . |
| FPGS ---- tag | rs10987746 | T/T | 16 | 27,59 | 8 | 23,53 | 113 | 29,97 | 76 | 28,15 | 1.00 (.-.) | . | 1.43 (0.61-3.35) | 0.41 | 0.35 | 0.96 | 0.63 |
| FPGS ---- tag |  | T/C | 25 | 43,10 | 19 | 55,88 | 188 | 49,87 | 129 | 47,78 | 1.34 (0.53-3.39) | 0.54 | 1.15 (0.50-2.65) | 0.74 | . | . | . |
| FPGS ---- tag |  | C/C | 17 | 29,31 | 7 | 20,59 | 76 | 20,16 | 65 | 24,07 | 0.48 (0.13-1.74) | 0.27 | 1.42 (0.60-3.36) | 0.42 | . | . | . |
| FPGS ---- tag | rs7033913 | T/T | 21 | 36,21 | 12 | 35,29 | 121 | 32,10 | 86 | 31,85 | 1.00 (.-.) | . | 1.34 (0.66-2.69) | 0.42 | 0.89 | 0.99 | 0.63 |
| FPGS ---- tag |  | T/C | 24 | 41,38 | 17 | 50,00 | 177 | 46,95 | 142 | 52,59 | 1.12 (0.49-2.54) | 0.79 | 1.33 (0.68-2.62) | 0.41 | . | . | . |
| FPGS ---- tag |  | C/C | 13 | 22,41 | 5 | 14,71 | 79 | 20,95 | 42 | 15,56 | 0.86 (0.23-3.21) | 0.82 | 1.14 (0.55-2.36) | 0.72 | . | . | . |
| FPGS ---- tag | rs7039798 | G/G | 18 | 31,03 | 8 | 23,53 | 129 | 34,22 | 80 | 29,63 | 1.00 (.-.) | . | 1.33 (0.57-3.11) | 0.50 | 0.29 | 0.97 | 0.63 |
| FPGS ---- tag |  | G/A | 24 | 41,38 | 20 | 58,82 | 183 | 48,54 | 138 | 51,11 | 1.38 (0.55-3.52) | 0.49 | 1.25 (0.54-2.87) | 0.60 | . | . | . |
| FPGS ---- tag |  | A/A | 16 | 27,59 | 6 | 17,65 | 65 | 17,24 | 52 | 19,26 | 0.50 (0.14-1.80) | 0.29 | 1.46 (0.61-3.47) | 0.40 | . | . | . |
| GGH ---- tag | rs10957264 | G/G | 39 | 67,24 | 27 | 79,41 | 260 | 68,97 | 192 | 71,11 | 1.00 (.-.) | . | 1.16 (0.73-1.84) | 0.52 | 0.55 | 0.92 | 0.42 |
| GGH ---- tag |  | G/T | 18 | 31,03 | 7 | 20,59 | 98 | 25,99 | 73 | 27,04 | 0.66 (0.25-1.78) | 0.41 | 1.16 (0.70-1.92) | 0.57 | . | . | . |
| GGH ---- tag |  | T/T | 1 | 1,72 | 0 | 0,00 | 19 | 5,04 | 5 | 1,85 | 0.00 (0.00-2E285) | 0.98 | 0.57 (0.21-1.51) | 0.26 | . | . | . |
| GGH ---- candidate literature | rs11545076 | T/T | 30 | 51,72 | 17 | 50,00 | 182 | 48,28 | 134 | 49,63 | 1.00 (.-.) | . | 1.34 (0.72-2.47) | 0.35 | 0.84 | 0.93 | 0.42 |
| GGH ---- candidate literature |  | T/G | 23 | 39,66 | 16 | 47,06 | 161 | 42,71 | 118 | 43,70 | 1.05 (0.48-2.30) | 0.90 | 1.22 (0.66-2.25) | 0.53 | . | . | . |
| GGH ---- candidate literature |  | G/G | 5 | 8,62 | 1 | 2,94 | 34 | 9,02 | 18 | 6,67 | 0.60 (0.08-4.62) | 0.62 | 0.92 (0.43-1.96) | 0.83 | . | . | . |
| GGH ---- candidate | rs11545077 | G/G | 32 | 55,17 | 19 | 55,88 | 199 | 52,79 | 144 | 53,33 | 1.00 (.-.) | . | 1.21 (0.68-2.14) | 0.52 | 0.88 | 0.99 | 0.93 |
| GGH ---- candidate |  | G/A | 21 | 36,21 | 14 | 41,18 | 148 | 39,26 | 114 | 42,22 | 0.95 (0.43-2.08) | 0.89 | 1.21 (0.68-2.16) | 0.52 | . | . | . |
| GGH ---- candidate |  | A/A | 5 | 8,62 | 1 | 2,94 | 30 | 7,96 | 12 | 4,44 | 0.56 (0.07-4.33) | 0.58 | 0.79 (0.36-1.74) | 0.56 | . | . | . |
| GGH ---- candidate | rs11545078 | C/C | 48 | 82,76 | 30 | 88,24 | 310 | 82,23 | 221 | 81,85 | 1.00 (.-.) | . | 1.26 (0.81-1.96) | 0.30 | 0.89 | 0.89 | 0.42 |
| GGH ---- candidate |  | C/T | 9 | 15,52 | 4 | 11,76 | 56 | 14,85 | 46 | 17,04 | 0.99 (0.30-3.35) | 0.99 | 1.36 (0.80-2.31) | 0.25 | . | . | . |
| GGH ---- candidate |  | T/T | 1 | 1,72 | 0 | 0,00 | 11 | 2,92 | 3 | 1,11 | 0.00 (0.00-1E285) | 0.98 | 0.47 (0.14-1.58) | 0.23 | . | . | . |
| GGH ---- tag | rs11995525 | G/G | 30 | 51,72 | 15 | 44,12 | 218 | 57,82 | 133 | 49,26 | 1.00 (.-.) | . | 1.34 (0.71-2.53) | 0.37 | 0.63 | 0.59 | 0.29 |
| GGH ---- tag |  | G/A | 22 | 37,93 | 16 | 47,06 | 143 | 37,93 | 115 | 42,59 | 1.68 (0.74-3.81) | 0.21 | 1.84 (0.97-3.48) | 0.06 | . | . | . |
| GGH ---- tag |  | A/A | 6 | 10,34 | 3 | 8,82 | 16 | 4,24 | 22 | 8,15 | 1.04 (0.28-3.92) | 0.95 | 2.42 (1.12-5.21) | 0.02 | . | . | . |
| GGH ---- tag | rs16930073 | G/G | 44 | 75,86 | 26 | 76,47 | 296 | 78,51 | 218 | 80,74 | 1.00 (.-.) | . | 1.26 (0.78-2.01) | 0.35 | 0.95 | 0.80 | 0.33 |
| GGH ---- tag |  | G/A | 13 | 22,41 | 8 | 23,53 | 73 | 19,36 | 51 | 18,89 | 1.02 (0.43-2.41) | 0.97 | 1.15 (0.67-1.98) | 0.62 | . | . | . |
| GGH ---- tag |  | A/A | 1 | 1,72 | 0 | 0,00 | 8 | 2,12 | 1 | 0,37 | 0.00 (0.00-8E267) | 0.97 | 0.64 (0.09-4.84) | 0.67 | . | . | . |
| GGH ---- tag | rs17194931 | G/G | 48 | 82,76 | 30 | 88,24 | 310 | 82,23 | 221 | 81,85 | 1.00 (.-.) | . | 1.26 (0.81-1.96) | 0.30 | 0.89 | 0.89 | 0.42 |
| GGH ---- tag |  | G/A | 9 | 15,52 | 4 | 11,76 | 56 | 14,85 | 46 | 17,04 | 0.99 (0.30-3.35) | 0.99 | 1.36 (0.80-2.31) | 0.25 | . | . | . |
| GGH ---- tag |  | A/A | 1 | 1,72 | 0 | 0,00 | 11 | 2,92 | 3 | 1,11 | 0.00 (0.00-1E285) | 0.98 | 0.47 (0.14-1.58) | 0.23 | . | . | . |
| GGH ---- candidate literature | rs1800909 | T/T | 30 | 51,72 | 17 | 50,00 | 181 | 48,01 | 134 | 49,63 | 1.00 (.-.) | . | 1.37 (0.74-2.52) | 0.32 | 0.78 | 0.88 | 0.42 |
| GGH ---- candidate literature |  | T/C | 23 | 39,66 | 16 | 47,06 | 163 | 43,24 | 118 | 43,70 | 1.05 (0.48-2.30) | 0.91 | 1.19 (0.64-2.20) | 0.58 | . | . | . |
| GGH ---- candidate literature |  | C/C | 5 | 8,62 | 1 | 2,94 | 33 | 8,75 | 18 | 6,67 | 0.60 (0.08-4.61) | 0.62 | 0.92 (0.43-1.95) | 0.83 | . | . | . |
| GGH ---- candidate literature | rs3758149 | C/C | 30 | 51,72 | 17 | 50,00 | 182 | 48,28 | 134 | 49,63 | 1.00 (.-.) | . | 1.34 (0.72-2.47) | 0.35 | 0.84 | 0.93 | 0.42 |
| GGH ---- candidate literature |  | C/T | 23 | 39,66 | 16 | 47,06 | 161 | 42,71 | 118 | 43,70 | 1.05 (0.48-2.30) | 0.90 | 1.22 (0.66-2.25) | 0.53 | . | . | . |
| GGH ---- candidate literature |  | T/T | 5 | 8,62 | 1 | 2,94 | 34 | 9,02 | 18 | 6,67 | 0.60 (0.08-4.62) | 0.62 | 0.92 (0.43-1.96) | 0.83 | . | . | . |
| GGH ---- tag | rs3780130 | A/A | 34 | 58,62 | 20 | 58,82 | 224 | 59,42 | 171 | 63,33 | 1.00 (.-.) | . | 1.49 (0.89-2.49) | 0.13 | 0.25 | 0.59 | 0.09 |
| GGH ---- tag |  | A/T | 21 | 36,21 | 12 | 35,29 | 134 | 35,54 | 91 | 33,70 | 1.21 (0.54-2.71) | 0.65 | 1.17 (0.69-1.99) | 0.56 | . | . | . |
| GGH ---- tag |  | T/T | 3 | 5,17 | 2 | 5,88 | 19 | 5,04 | 8 | 2,96 | 2.13 (0.28-16.43) | 0.47 | 1.21 (0.50-2.96) | 0.67 | . | . | . |
| GGH ---- tag | rs4446729 | C/C | 36 | 62,07 | 20 | 58,82 | 198 | 52,52 | 148 | 54,81 | 1.00 (.-.) | . | 1.12 (0.67-1.88) | 0.67 | 0.31 | 0.99 | 0.92 |
| GGH ---- tag |  | C/T | 18 | 31,03 | 12 | 35,29 | 155 | 41,11 | 103 | 38,15 | 0.88 (0.38-2.09) | 0.78 | 1.02 (0.60-1.73) | 0.95 | . | . | . |
| GGH ---- tag |  | T/T | 4 | 6,90 | 2 | 5,88 | 24 | 6,37 | 19 | 7,04 | 0.42 (0.09-1.90) | 0.26 | 1.37 (0.67-2.80) | 0.39 | . | . | . |
| GGH ---- tag | rs6472067 | C/C | 21 | 36,21 | 10 | 29,41 | 162 | 42,97 | 97 | 35,93 | 1.00 (.-.) | . | 1.76 (0.84-3.71) | 0.14 | 0.32 | 0.79 | 0.33 |
| GGH ---- tag |  | C/G | 25 | 43,10 | 17 | 50,00 | 165 | 43,77 | 131 | 48,52 | 2.29 (0.93-5.61) | 0.07 | 2.21 (1.06-4.58) | 0.03 | . | . | . |
| GGH ---- tag |  | G/G | 12 | 20,69 | 7 | 20,59 | 50 | 13,26 | 42 | 15,56 | 2.27 (0.75-6.86) | 0.15 | 2.36 (1.08-5.19) | 0.03 | . | . | . |
| GGH ---- tag | rs7010484 | T/T | 33 | 56,90 | 18 | 52,94 | 177 | 46,95 | 126 | 46,67 | 1.00 (.-.) | . | 1.07 (0.61-1.90) | 0.80 | 0.34 | 0.83 | 0.36 |
| GGH ---- tag |  | T/C | 19 | 32,76 | 12 | 35,29 | 154 | 40,85 | 112 | 41,48 | 1.05 (0.45-2.45) | 0.90 | 1.19 (0.67-2.10) | 0.55 | . | . | . |
| GGH ---- tag |  | C/C | 6 | 10,34 | 4 | 11,76 | 46 | 12,20 | 32 | 11,85 | 0.54 (0.17-1.70) | 0.30 | 1.07 (0.55-2.08) | 0.83 | . | . | . |
| GNMT ---- tag | rs1053538 | C/C | 22 | 37,93 | 9 | 26,47 | 93 | 24,67 | 71 | 26,30 | 1.00 (.-.) | . | 1.88 (0.80-4.40) | 0.15 | 0.26 | 0.99 | 0.75 |
| GNMT ---- tag |  | C/G | 28 | 48,28 | 16 | 47,06 | 195 | 51,72 | 131 | 48,52 | 1.68 (0.64-4.41) | 0.29 | 1.83 (0.80-4.21) | 0.15 | . | . | . |
| GNMT ---- tag |  | G/G | 8 | 13,79 | 9 | 26,47 | 89 | 23,61 | 68 | 25,19 | 2.22 (0.74-6.70) | 0.16 | 2.16 (0.92-5.10) | 0.08 | . | . | . |
| GNMT ---- tag | rs2296805 | G/G | 17 | 29,31 | 14 | 41,18 | 114 | 30,24 | 89 | 32,96 | 1.00 (.-.) | . | 1.49 (0.80-2.79) | 0.21 | 0.76 | 0.66 | 0.28 |
| GNMT ---- tag |  | G/T | 28 | 48,28 | 16 | 47,06 | 195 | 51,72 | 129 | 47,78 | 1.37 (0.63-2.98) | 0.43 | 1.17 (0.64-2.17) | 0.61 | . | . | . |
| GNMT ---- tag |  | T/T | 13 | 22,41 | 4 | 11,76 | 68 | 18,04 | 52 | 19,26 | 0.26 (0.03-2.00) | 0.20 | 1.29 (0.67-2.49) | 0.45 | . | . | . |
| GNMT ---- tag | rs6901782 | T/T | 38 | 65,52 | 23 | 67,65 | 290 | 76,92 | 215 | 79,63 | 1.00 (.-.) | . | 1.22 (0.74-2.01) | 0.43 | 0.67 | 0.94 | 0.70 |
| GNMT ---- tag |  | T/C | 18 | 31,03 | 11 | 32,35 | 83 | 22,02 | 49 | 18,15 | 1.14 (0.52-2.51) | 0.74 | 1.50 (0.85-2.66) | 0.16 | . | . | . |
| GNMT ---- tag |  | C/C | 2 | 3,45 | 0 | 0,00 | 4 | 1,06 | 6 | 2,22 | 0.00 (0.00-1E297) | 0.98 | 1.41 (0.51-3.87) | 0.51 | . | . | . |
| GNMT ---- tag | rs6927188 | A/A | 42 | 72,41 | 20 | 58,82 | 212 | 56,23 | 144 | 53,33 | 1.00 (.-.) | . | 1.42 (0.83-2.44) | 0.20 | 0.34 | 0.97 | 0.70 |
| GNMT ---- tag |  | A/G | 15 | 25,86 | 13 | 38,24 | 140 | 37,14 | 108 | 40,00 | 1.34 (0.62-2.92) | 0.46 | 1.40 (0.81-2.43) | 0.23 | . | . | . |
| GNMT ---- tag |  | G/G | 1 | 1,72 | 1 | 2,94 | 25 | 6,63 | 18 | 6,67 | 2.64 (0.34-20.63) | 0.35 | 1.46 (0.70-3.04) | 0.31 | . | . | . |
| MAT1A ---- tag | rs10887708 | G/G | 30 | 51,72 | 20 | 58,82 | 190 | 50,40 | 148 | 54,81 | 1.00 (.-.) | . | 1.20 (0.70-2.06) | 0.50 | 0.91 | 0.93 | 0.82 |
| MAT1A ---- tag |  | G/A | 24 | 41,38 | 10 | 29,41 | 142 | 37,67 | 104 | 38,52 | 0.91 (0.41-2.04) | 0.82 | 1.11 (0.64-1.93) | 0.71 | . | . | . |
| MAT1A ---- tag |  | A/A | 4 | 6,90 | 4 | 11,76 | 45 | 11,94 | 18 | 6,67 | 0.49 (0.11-2.23) | 0.36 | 0.62 (0.30-1.29) | 0.20 | . | . | . |
| MAT1A ---- tag | rs10887718 | T/T | 18 | 31,03 | 9 | 26,47 | 107 | 28,38 | 72 | 26,67 | 1.00 (.-.) | . | 1.68 (0.71-4.00) | 0.24 | 0.34 | 0.59 | 0.20 |
| MAT1A ---- tag |  | T/C | 27 | 46,55 | 14 | 41,18 | 197 | 52,25 | 136 | 50,37 | 1.47 (0.54-3.98) | 0.45 | 1.86 (0.80-4.34) | 0.15 | . | . | . |
| MAT1A ---- tag |  | C/C | 13 | 22,41 | 11 | 32,35 | 73 | 19,36 | 62 | 22,96 | 1.93 (0.68-5.46) | 0.22 | 1.88 (0.79-4.46) | 0.16 | . | . | . |
| MAT1A ---- tag | rs11202403 | C/C | 41 | 70,69 | 24 | 70,59 | 242 | 64,19 | 169 | 62,59 | 1.00 (.-.) | . | 1.42 (0.88-2.31) | 0.15 | 0.30 | 0.99 | 0.98 |
| MAT1A ---- tag |  | C/T | 13 | 22,41 | 10 | 29,41 | 114 | 30,24 | 94 | 34,81 | 3.06 (1.30-7.20) | 0.01 | 1.63 (0.99-2.69) | 0.06 | . | . | . |
| MAT1A ---- tag |  | T/T | 4 | 6,90 | 0 | 0,00 | 21 | 5,57 | 7 | 2,59 | 0.00 (0.00-6E236) | 0.97 | 0.90 (0.37-2.16) | 0.81 | . | . | . |
| MAT1A ---- tag | rs1832683 | C/C | 37 | 63,79 | 25 | 73,53 | 255 | 67,64 | 186 | 68,89 | 1.00 (.-.) | . | 1.05 (0.66-1.67) | 0.84 | 0.49 | 0.93 | 0.82 |
| MAT1A ---- tag |  | C/T | 18 | 31,03 | 6 | 17,65 | 111 | 29,44 | 75 | 27,78 | 0.35 (0.10-1.19) | 0.09 | 0.99 (0.60-1.64) | 0.97 | . | . | . |
| MAT1A ---- tag |  | T/T | 3 | 5,17 | 3 | 8,82 | 11 | 2,92 | 9 | 3,33 | 1.04 (0.30-3.62) | 0.95 | 1.06 (0.44-2.54) | 0.90 | . | . | . |
| MAT1A ---- tag | rs2236568 | C/C | 14 | 24,14 | 15 | 44,12 | 123 | 32,63 | 92 | 34,07 | 1.00 (.-.) | . | 0.81 (0.46-1.45) | 0.48 | 0.52 | 0.88 | 0.80 |
| MAT1A ---- tag |  | C/A | 35 | 60,34 | 11 | 32,35 | 164 | 43,50 | 140 | 51,85 | 0.35 (0.14-0.83) | 0.02 | 0.80 (0.45-1.41) | 0.44 | . | . | . |
| MAT1A ---- tag |  | A/A | 9 | 15,52 | 8 | 23,53 | 90 | 23,87 | 38 | 14,07 | 0.61 (0.23-1.61) | 0.32 | 0.47 (0.24-0.90) | 0.02 | . | . | . |
| MAT1A ---- tag | rs2236569 | A/A | 24 | 41,38 | 15 | 44,12 | 183 | 48,54 | 106 | 39,26 | 1.00 (.-.) | . | 1.07 (0.56-2.06) | 0.83 | 0.90 | 0.59 | 0.05 |
| MAT1A ---- tag |  | A/G | 29 | 50,00 | 10 | 29,41 | 157 | 41,64 | 130 | 48,15 | 0.85 (0.35-2.09) | 0.73 | 1.53 (0.80-2.91) | 0.20 | . | . | . |
| MAT1A ---- tag |  | G/G | 5 | 8,62 | 9 | 26,47 | 37 | 9,81 | 34 | 12,59 | 1.59 (0.63-4.06) | 0.33 | 1.58 (0.77-3.25) | 0.21 | . | . | . |
| MAT1A ---- tag | rs9421467 | G/G | 51 | 87,93 | 32 | 94,12 | 336 | 89,12 | 239 | 88,52 | 1.00 (.-.) | . | 1.23 (0.80-1.89) | 0.35 | 0.92 | 0.99 | 0.98 |
| MAT1A ---- tag |  | G/C | 6 | 10,34 | 2 | 5,88 | 39 | 10,34 | 31 | 11,48 | 0.84 (0.19-3.66) | 0.82 | 1.29 (0.75-2.24) | 0.36 | . | . | . |
| MAT1A ---- tag |  | C/C | 1 | 1,72 | 0 | 0,00 | 2 | 0,53 | 0 | 0,00 | 0.00 (0.00-3E256) | 0.97 | 0.00 (0.00-3E256) | 0.97 | . | . | . |
| MAT1A ---- tag | rs998765 | A/A | 17 | 29,31 | 13 | 38,24 | 96 | 25,46 | 75 | 27,78 | 1.00 (.-.) | . | 1.39 (0.72-2.69) | 0.33 | 0.42 | 0.99 | 0.98 |
| MAT1A ---- tag |  | A/T | 29 | 50,00 | 13 | 38,24 | 175 | 46,42 | 140 | 51,85 | 1.05 (0.45-2.48) | 0.91 | 1.41 (0.75-2.66) | 0.29 | . | . | . |
| MAT1A ---- tag |  | T/T | 12 | 20,69 | 8 | 23,53 | 106 | 28,12 | 55 | 20,37 | 1.10 (0.39-3.06) | 0.86 | 0.95 (0.48-1.87) | 0.89 | . | . | . |
| MAT1A ---- tag | rs998766 | C/C | 23 | 39,66 | 15 | 44,12 | 113 | 29,97 | 90 | 33,33 | 1.00 (.-.) | . | 1.34 (0.73-2.45) | 0.35 | 0.40 | 1.00 | 0.98 |
| MAT1A ---- tag |  | C/G | 25 | 43,10 | 11 | 32,35 | 183 | 48,54 | 141 | 52,22 | 0.97 (0.41-2.33) | 0.95 | 1.38 (0.77-2.49) | 0.28 | . | . | . |
| MAT1A ---- tag |  | G/G | 10 | 17,24 | 8 | 23,53 | 81 | 21,49 | 39 | 14,44 | 1.17 (0.43-3.15) | 0.76 | 0.88 (0.46-1.70) | 0.71 | . | . | . |
| MAT2B ---- tag | rs12655857 | G/G | 27 | 46,55 | 18 | 52,94 | 212 | 56,23 | 147 | 54,44 | 1.00 (.-.) | . | 1.01 (0.57-1.76) | 0.99 | 0.40 | 0.99 | 0.93 |
| MAT2B ---- tag |  | G/T | 27 | 46,55 | 13 | 38,24 | 139 | 36,87 | 108 | 40,00 | 0.72 (0.33-1.58) | 0.41 | 1.08 (0.61-1.92) | 0.78 | . | . | . |
| MAT2B ---- tag |  | T/T | 4 | 6,90 | 3 | 8,82 | 26 | 6,90 | 15 | 5,56 | 0.55 (0.12-2.50) | 0.44 | 0.76 (0.35-1.61) | 0.47 | . | . | . |
| MAT2B ---- tag | rs6869277 | C/C | 45 | 77,59 | 28 | 82,35 | 306 | 81,17 | 209 | 77,41 | 1.00 (.-.) | . | 1.20 (0.76-1.92) | 0.43 | 0.81 | 0.99 | 0.93 |
| MAT2B ---- tag |  | C/T | 12 | 20,69 | 6 | 17,65 | 64 | 16,98 | 58 | 21,48 | 0.89 (0.36-2.23) | 0.81 | 1.25 (0.73-2.13) | 0.42 | . | . | . |
| MAT2B ---- tag |  | T/T | 1 | 1,72 | 0 | 0,00 | 7 | 1,86 | 3 | 1,11 | 0.00 (0.00-1E222) | 0.97 | 0.81 (0.23-2.82) | 0.75 | . | . | . |
| MAT2B ---- tag | rs6874065 | A/A | 19 | 32,76 | 10 | 29,41 | 105 | 27,85 | 77 | 28,52 | 1.00 (.-.) | . | 1.32 (0.65-2.68) | 0.45 | 0.53 | 0.99 | 0.93 |
| MAT2B ---- tag |  | A/G | 28 | 48,28 | 16 | 47,06 | 175 | 46,42 | 129 | 47,78 | 1.15 (0.48-2.76) | 0.75 | 1.55 (0.78-3.11) | 0.21 | . | . | . |
| MAT2B ---- tag |  | G/G | 11 | 18,97 | 8 | 23,53 | 97 | 25,73 | 64 | 23,70 | 1.51 (0.53-4.32) | 0.44 | 1.37 (0.67-2.80) | 0.39 | . | . | . |
| MAT2B ---- tag | rs6882306 | T/T | 44 | 75,86 | 26 | 76,47 | 263 | 69,76 | 184 | 68,15 | 1.00 (.-.) | . | 1.21 (0.75-1.94) | 0.44 | 0.80 | 0.59 | 0.10 |
| MAT2B ---- tag |  | T/C | 13 | 22,41 | 8 | 23,53 | 105 | 27,85 | 78 | 28,89 | 1.19 (0.51-2.79) | 0.69 | 1.60 (0.95-2.69) | 0.08 | . | . | . |
| MAT2B ---- tag |  | C/C | 1 | 1,72 | 0 | 0,00 | 9 | 2,39 | 8 | 2,96 | 0.00 (0.00-I) | 0.98 | 1.97 (0.73-5.33) | 0.18 | . | . | . |
| MAT2B ---- tag | rs7721639 | T/T | 42 | 72,41 | 25 | 73,53 | 270 | 71,62 | 196 | 72,59 | 1.00 (.-.) | . | 1.24 (0.78-1.97) | 0.37 | 0.90 | 0.99 | 0.93 |
| MAT2B ---- tag |  | T/G | 14 | 24,14 | 9 | 26,47 | 94 | 24,93 | 68 | 25,19 | 1.11 (0.46-2.72) | 0.81 | 1.32 (0.79-2.21) | 0.29 | . | . | . |
| MAT2B ---- tag |  | G/G | 2 | 3,45 | 0 | 0,00 | 13 | 3,45 | 6 | 2,22 | 0.00 (0.00-I) | 0.98 | 1.09 (0.37-3.21) | 0.88 | . | . | . |
| MTHFD1 ---- tag | rs1256148 | G/G | 35 | 60,34 | 17 | 50,00 | 229 | 60,74 | 156 | 57,78 | 1.00 (.-.) | . | 1.56 (0.88-2.74) | 0.13 | 0.43 | 0.93 | 0.88 |
| MTHFD1 ---- tag |  | G/A | 22 | 37,93 | 14 | 41,18 | 131 | 34,75 | 98 | 36,30 | 1.61 (0.71-3.68) | 0.26 | 1.32 (0.74-2.36) | 0.34 | . | . | . |
| MTHFD1 ---- tag |  | A/A | 1 | 1,72 | 3 | 8,82 | 17 | 4,51 | 16 | 5,93 | 1.21 (0.33-4.38) | 0.78 | 1.96 (0.91-4.19) | 0.08 | . | . | . |
| MTHFD1 ---- tag | rs13329053 | T/T | 13 | 22,41 | 8 | 23,53 | 121 | 32,10 | 93 | 34,44 | 1.00 (.-.) | . | 1.06 (0.50-2.22) | 0.88 | 0.90 | 0.99 | 0.95 |
| MTHFD1 ---- tag |  | T/C | 32 | 55,17 | 16 | 47,06 | 185 | 49,07 | 121 | 44,81 | 0.81 (0.33-2.01) | 0.65 | 1.22 (0.59-2.55) | 0.59 | . | . | . |
| MTHFD1 ---- tag |  | C/C | 13 | 22,41 | 10 | 29,41 | 71 | 18,83 | 56 | 20,74 | 1.34 (0.48-3.74) | 0.57 | 1.53 (0.71-3.28) | 0.27 | . | . | . |
| MTHFD1 ---- candidate literature | rs2236224 | C/C | 20 | 34,48 | 8 | 23,53 | 146 | 38,73 | 114 | 42,22 | 1.00 (.-.) | . | 1.41 (0.68-2.92) | 0.36 | 0.66 | 0.96 | 0.88 |
| MTHFD1 ---- candidate literature |  | C/T | 30 | 51,72 | 18 | 52,94 | 174 | 46,15 | 113 | 41,85 | 1.30 (0.52-3.23) | 0.57 | 1.70 (0.82-3.54) | 0.16 | . | . | . |
| MTHFD1 ---- candidate literature |  | T/T | 8 | 13,79 | 8 | 23,53 | 57 | 15,12 | 43 | 15,93 | 1.97 (0.69-5.64) | 0.20 | 2.17 (1.00-4.73) | 0.05 | . | . | . |
| MTHFD1 ---- candidate | rs2236225 | C/C | 15 | 25,86 | 7 | 20,59 | 119 | 31,56 | 98 | 36,30 | 1.00 (.-.) | . | 1.18 (0.53-2.60) | 0.69 | 0.85 | 0.99 | 0.95 |
| MTHFD1 ---- candidate |  | C/T | 29 | 50,00 | 19 | 55,88 | 188 | 49,87 | 119 | 44,07 | 1.01 (0.40-2.55) | 0.99 | 1.30 (0.59-2.85) | 0.52 | . | . | . |
| MTHFD1 ---- candidate |  | T/T | 14 | 24,14 | 8 | 23,53 | 70 | 18,57 | 53 | 19,63 | 1.22 (0.41-3.61) | 0.72 | 1.63 (0.72-3.67) | 0.24 | . | . | . |
| MTHFD1 ---- tag | rs2281603 | A/A | 36 | 62,07 | 24 | 70,59 | 225 | 59,68 | 150 | 55,56 | 1.00 (.-.) | . | 1.32 (0.80-2.16) | 0.28 | 0.99 | 0.99 | 0.88 |
| MTHFD1 ---- tag |  | A/G | 19 | 32,76 | 9 | 26,47 | 134 | 35,54 | 104 | 38,52 | 1.14 (0.49-2.63) | 0.77 | 1.18 (0.71-1.96) | 0.53 | . | . | . |
| MTHFD1 ---- tag |  | G/G | 3 | 5,17 | 1 | 2,94 | 18 | 4,77 | 16 | 5,93 | 0.51 (0.07-3.92) | 0.52 | 1.13 (0.56-2.28) | 0.74 | . | . | . |
| MTHFD1 ---- candidate literature | rs8003379 | A/A | 35 | 60,34 | 17 | 50,00 | 221 | 58,62 | 150 | 55,56 | 1.00 (.-.) | . | 1.25 (0.70-2.24) | 0.46 | 0.97 | 0.69 | 0.49 |
| MTHFD1 ---- candidate literature |  | A/C | 19 | 32,76 | 16 | 47,06 | 128 | 33,95 | 101 | 37,41 | 1.26 (0.58-2.71) | 0.56 | 1.55 (0.86-2.81) | 0.14 | . | . | . |
| MTHFD1 ---- candidate literature |  | C/C | 4 | 6,90 | 1 | 2,94 | 28 | 7,43 | 19 | 7,04 | 1.07 (0.13-8.67) | 0.95 | 1.62 (0.77-3.43) | 0.21 | . | . | . |
| MTHFD2 ---- tag | rs10177833 | A/A | 18 | 31,03 | 8 | 23,53 | 129 | 34,22 | 77 | 28,52 | 1.00 (.-.) | . | 1.62 (0.69-3.82) | 0.27 | 0.57 | 0.66 | 0.26 |
| MTHFD2 ---- tag |  | A/C | 27 | 46,55 | 20 | 58,82 | 177 | 46,95 | 147 | 54,44 | 1.69 (0.65-4.39) | 0.28 | 1.92 (0.83-4.44) | 0.13 | . | . | . |
| MTHFD2 ---- tag |  | C/C | 13 | 22,41 | 6 | 17,65 | 71 | 18,83 | 46 | 17,04 | 1.51 (0.44-5.13) | 0.51 | 1.73 (0.72-4.15) | 0.22 | . | . | . |
| MTHFD2 ---- tag | rs702462 | T/T | 23 | 39,66 | 13 | 38,24 | 128 | 33,95 | 88 | 32,59 | 1.00 (.-.) | . | 1.60 (0.78-3.29) | 0.20 | 0.65 | 0.99 | 0.83 |
| MTHFD2 ---- tag |  | T/A | 24 | 41,38 | 15 | 44,12 | 181 | 48,01 | 138 | 51,11 | 1.46 (0.61-3.49) | 0.40 | 1.43 (0.71-2.89) | 0.31 | . | . | . |
| MTHFD2 ---- tag |  | A/A | 11 | 18,97 | 6 | 17,65 | 68 | 18,04 | 44 | 16,30 | 1.01 (0.33-3.11) | 0.99 | 1.38 (0.65-2.95) | 0.40 | . | . | . |
| MTHFD2 ---- candidate literature | rs702465 | A/A | 20 | 34,48 | 8 | 23,53 | 110 | 29,18 | 68 | 25,19 | 1.00 (.-.) | . | 1.36 (0.61-3.04) | 0.46 | 0.87 | 0.95 | 0.69 |
| MTHFD2 ---- candidate literature |  | A/T | 22 | 37,93 | 18 | 52,94 | 183 | 48,54 | 146 | 54,07 | 1.71 (0.68-4.30) | 0.26 | 1.84 (0.84-4.03) | 0.13 | . | . | . |
| MTHFD2 ---- candidate literature |  | T/T | 16 | 27,59 | 8 | 23,53 | 84 | 22,28 | 56 | 20,74 | 1.27 (0.42-3.87) | 0.68 | 1.90 (0.84-4.31) | 0.12 | . | . | . |
| MTHFD2 ---- candidate literature | rs7571842 | A/A | 17 | 29,31 | 7 | 20,59 | 119 | 31,56 | 69 | 25,56 | 1.00 (.-.) | . | 1.66 (0.65-4.20) | 0.29 | 0.84 | 0.82 | 0.26 |
| MTHFD2 ---- candidate literature |  | A/G | 25 | 43,10 | 20 | 58,82 | 183 | 48,54 | 150 | 55,56 | 1.77 (0.64-4.86) | 0.27 | 1.85 (0.74-4.58) | 0.19 | . | . | . |
| MTHFD2 ---- candidate literature |  | G/G | 16 | 27,59 | 7 | 20,59 | 75 | 19,89 | 51 | 18,89 | 1.16 (0.35-3.91) | 0.81 | 1.64 (0.64-4.19) | 0.30 | . | . | . |
| MTHFD2 ---- tag | rs7587117 | T/T | 27 | 46,55 | 13 | 38,24 | 171 | 45,36 | 103 | 38,15 | 1.00 (.-.) | . | 1.54 (0.76-3.11) | 0.23 | 0.47 | 0.85 | 0.35 |
| MTHFD2 ---- tag |  | T/C | 24 | 41,38 | 16 | 47,06 | 159 | 42,18 | 137 | 50,74 | 1.53 (0.65-3.57) | 0.33 | 1.69 (0.84-3.39) | 0.14 | . | . | . |
| MTHFD2 ---- tag |  | C/C | 7 | 12,07 | 5 | 14,71 | 47 | 12,47 | 30 | 11,11 | 1.53 (0.45-5.17) | 0.50 | 1.59 (0.72-3.50) | 0.25 | . | . | . |
| MTHFD2 ---- tag | rs828861 | C/C | 21 | 36,21 | 8 | 23,53 | 108 | 28,65 | 69 | 25,56 | 1.00 (.-.) | . | 1.50 (0.67-3.37) | 0.32 | 0.99 | 0.99 | 0.78 |
| MTHFD2 ---- tag |  | C/G | 22 | 37,93 | 19 | 55,88 | 182 | 48,28 | 149 | 55,19 | 1.88 (0.75-4.69) | 0.18 | 1.88 (0.86-4.13) | 0.12 | . | . | . |
| MTHFD2 ---- tag |  | G/G | 15 | 25,86 | 7 | 20,59 | 87 | 23,08 | 52 | 19,26 | 1.13 (0.35-3.64) | 0.84 | 1.78 (0.78-4.04) | 0.17 | . | . | . |
| MTHFD2 ---- tag | rs828863 | G/G | 48 | 82,76 | 31 | 91,18 | 299 | 79,31 | 232 | 85,93 | 1.00 (.-.) | . | 1.26 (0.82-1.94) | 0.29 | 0.89 | 0.59 | 0.10 |
| MTHFD2 ---- tag |  | G/A | 10 | 17,24 | 3 | 8,82 | 73 | 19,36 | 36 | 13,33 | 0.65 (0.19-2.19) | 0.49 | 0.80 (0.47-1.39) | 0.43 | . | . | . |
| MTHFD2 ---- tag |  | A/A | 0 | 0,00 | 0 | 0,00 | 5 | 1,33 | 2 | 0,74 | 1.96 (0.47-8.13) | 0.35 | 2.47 (0.56-10.84) | 0.23 | . | . | . |
| MTHFR ---- tag | rs1476413 | G/G | 31 | 53,45 | 18 | 52,94 | 199 | 52,79 | 143 | 52,96 | 1.00 (.-.) | . | 1.21 (0.69-2.14) | 0.51 | 0.93 | 0.99 | 0.90 |
| MTHFR ---- tag |  | G/A | 23 | 39,66 | 13 | 38,24 | 153 | 40,58 | 109 | 40,37 | 1.06 (0.47-2.38) | 0.89 | 1.36 (0.77-2.42) | 0.29 | . | . | . |
| MTHFR ---- tag |  | A/A | 4 | 6,90 | 3 | 8,82 | 25 | 6,63 | 18 | 6,67 | 1.22 (0.34-4.35) | 0.76 | 1.51 (0.72-3.14) | 0.27 | . | . | . |
| MTHFR ---- tag | rs17376328 | G/G | 53 | 91,38 | 29 | 85,29 | 331 | 87,80 | 227 | 84,07 | 1.00 (.-.) | . | 1.23 (0.78-1.93) | 0.38 | 0.92 | 0.85 | 0.69 |
| MTHFR ---- tag |  | G/A | 5 | 8,62 | 5 | 14,71 | 43 | 11,41 | 41 | 15,19 | 1.00 (0.37-2.72) | 0.99 | 1.38 (0.80-2.38) | 0.25 | . | . | . |
| MTHFR ---- tag |  | A/A | 0 | 0,00 | 0 | 0,00 | 3 | 0,80 | 2 | 0,74 | 0.78 (0.19-3.26) | 0.74 | 0.96 (0.22-4.20) | 0.95 | . | . | . |
| MTHFR ---- tag | rs17421462 | G/G | 49 | 84,48 | 28 | 82,35 | 325 | 86,21 | 229 | 84,81 | 1.00 (.-.) | . | 1.25 (0.79-1.98) | 0.34 | 0.92 | 0.96 | 0.84 |
| MTHFR ---- tag |  | G/A | 9 | 15,52 | 6 | 17,65 | 51 | 13,53 | 36 | 13,33 | 1.22 (0.45-3.31) | 0.69 | 1.35 (0.77-2.38) | 0.30 | . | . | . |
| MTHFR ---- tag |  | A/A | 0 | 0,00 | 0 | 0,00 | 1 | 0,27 | 5 | 1,85 | 4.47 (1.75-11.39) | 0.00 | 5.59 (2.02-15.43) | 0.00 | . | . | . |
| MTHFR ---- candidate | rs1801131 | A/A | 25 | 43,10 | 16 | 47,06 | 175 | 46,42 | 127 | 47,04 | 1.00 (.-.) | . | 1.37 (0.74-2.54) | 0.31 | 0.60 | 0.99 | 0.90 |
| MTHFR ---- candidate |  | A/C | 29 | 50,00 | 15 | 44,12 | 166 | 44,03 | 113 | 41,85 | 1.11 (0.50-2.48) | 0.79 | 1.30 (0.70-2.39) | 0.41 | . | . | . |
| MTHFR ---- candidate |  | C/C | 4 | 6,90 | 3 | 8,82 | 36 | 9,55 | 30 | 11,11 | 1.81 (0.50-6.50) | 0.37 | 1.75 (0.86-3.56) | 0.12 | . | . | . |
| MTHFR ----candidate | rs1801133 | C/C | 21 | 36,21 | 13 | 38,24 | 153 | 40,58 | 127 | 47,04 | 1.00 (.-.) | . | 1.13 (0.60-2.13) | 0.70 | 0.45 | 0.59 | 0.18 |
| MTHFR ---- candidate |  | C/T | 33 | 56,90 | 19 | 55,88 | 184 | 48,81 | 111 | 41,11 | 0.60 (0.28-1.32) | 0.21 | 0.72 (0.39-1.36) | 0.31 | . | . | . |
| MTHFR ---- candidate |  | T/T | 4 | 6,90 | 2 | 5,88 | 40 | 10,61 | 32 | 11,85 | 0.65 (0.08-5.01) | 0.68 | 1.22 (0.59-2.50) | 0.59 | . | . | . |
| MTHFR ---- tag | rs2066471 | G/G | 38 | 65,52 | 26 | 76,47 | 267 | 70,82 | 187 | 69,26 | 1.00 (.-.) | . | 1.10 (0.68-1.78) | 0.69 | 0.47 | 0.99 | 0.84 |
| MTHFR ---- tag |  | G/A | 19 | 32,76 | 7 | 20,59 | 103 | 27,32 | 76 | 28,15 | 0.85 (0.35-2.04) | 0.72 | 1.47 (0.88-2.45) | 0.14 | . | . | . |
| MTHFR ---- tag |  | A/A | 1 | 1,72 | 1 | 2,94 | 7 | 1,86 | 7 | 2,59 | 1.84 (0.24-14.13) | 0.56 | 1.84 (0.72-4.72) | 0.21 | . | . | . |
| MTHFR ---- tag | rs4846047 | G/G | 26 | 44,83 | 19 | 55,88 | 194 | 51,46 | 132 | 48,89 | 1.00 (.-.) | . | 1.01 (0.57-1.81) | 0.97 | 0.34 | 0.85 | 0.69 |
| MTHFR ---- tag |  | G/C | 27 | 46,55 | 14 | 41,18 | 163 | 43,24 | 115 | 42,59 | 0.84 (0.39-1.82) | 0.66 | 1.22 (0.68-2.19) | 0.50 | . | . | . |
| MTHFR ---- tag |  | C/C | 5 | 8,62 | 1 | 2,94 | 20 | 5,31 | 23 | 8,52 | 1.42 (0.18-10.95) | 0.74 | 1.97 (0.97-4.02) | 0.06 | . | . | . |
| MTHFR ---- tag | rs4846049 | G/G | 25 | 43,10 | 15 | 44,12 | 172 | 45,62 | 124 | 45,93 | 1.00 (.-.) | . | 1.31 (0.69-2.49) | 0.40 | 0.65 | 0.99 | 0.90 |
| MTHFR ---- tag |  | G/T | 29 | 50,00 | 15 | 44,12 | 169 | 44,83 | 116 | 42,96 | 1.00 (0.44-2.27) | 1.00 | 1.25 (0.66-2.37) | 0.49 | . | . | . |
| MTHFR ---- tag |  | T/T | 4 | 6,90 | 4 | 11,76 | 36 | 9,55 | 30 | 11,11 | 1.82 (0.57-5.80) | 0.31 | 1.72 (0.83-3.59) | 0.15 | . | . | . |
| MTHFR ---- tag | rs7538516 | T/T | 22 | 37,93 | 13 | 38,24 | 138 | 36,60 | 95 | 35,19 | 1.00 (.-.) | . | 1.20 (0.61-2.36) | 0.59 | 0.94 | 0.93 | 0.84 |
| MTHFR ---- tag |  | T/C | 27 | 46,55 | 16 | 47,06 | 195 | 51,72 | 132 | 48,89 | 0.95 (0.41-2.19) | 0.90 | 1.21 (0.62-2.36) | 0.57 | . | . | . |
| MTHFR ---- tag |  | C/C | 9 | 15,52 | 5 | 14,71 | 44 | 11,67 | 43 | 15,93 | 1.75 (0.59-5.16) | 0.31 | 1.96 (0.96-3.99) | 0.07 | . | . | . |
| MTR ---- tag | rs10733117 | A/A | 23 | 39,66 | 12 | 35,29 | 132 | 35,01 | 102 | 37,78 | 1.00 (.-.) | . | 0.74 (0.37-1.47) | 0.39 | 0.34 | 0.99 | 0.83 |
| MTR ---- tag |  | A/G | 26 | 44,83 | 11 | 32,35 | 189 | 50,13 | 125 | 46,30 | 0.41 (0.16-1.01) | 0.05 | 0.72 (0.36-1.42) | 0.34 | . | . | . |
| MTR ---- tag |  | G/G | 9 | 15,52 | 11 | 32,35 | 56 | 14,85 | 43 | 15,93 | 0.62 (0.24-1.60) | 0.32 | 0.76 (0.36-1.58) | 0.46 | . | . | . |
| MTR ---- tag | rs12129440 | G/G | 34 | 58,62 | 22 | 64,71 | 208 | 55,17 | 154 | 57,04 | 1.00 (.-.) | . | 1.19 (0.72-1.98) | 0.50 | 0.86 | 0.95 | 0.83 |
| MTR ---- tag |  | G/A | 21 | 36,21 | 10 | 29,41 | 146 | 38,73 | 100 | 37,04 | 0.83 (0.36-1.94) | 0.67 | 1.30 (0.77-2.19) | 0.33 | . | . | . |
| MTR ---- tag |  | A/A | 3 | 5,17 | 2 | 5,88 | 23 | 6,10 | 16 | 5,93 | 2.89 (0.65-12.83) | 0.16 | 1.33 (0.63-2.80) | 0.45 | . | . | . |
| MTR ---- candidate | rs1805087 | A/A | 42 | 72,41 | 24 | 70,59 | 250 | 66,31 | 187 | 69,26 | 1.00 (.-.) | . | 1.35 (0.83-2.19) | 0.22 | 0.72 | 0.99 | 0.83 |
| MTR ---- candidate |  | A/G | 12 | 20,69 | 9 | 26,47 | 109 | 28,91 | 76 | 28,15 | 1.43 (0.62-3.29) | 0.40 | 1.27 (0.76-2.14) | 0.37 | . | . | . |
| MTR ---- candidate |  | G/G | 4 | 6,90 | 1 | 2,94 | 18 | 4,77 | 7 | 2,59 | 0.00 (0.00-4E238) | 0.97 | 0.83 (0.34-2.04) | 0.69 | . | . | . |
| MTR ---- tag | rs3890786 | C/C | 21 | 36,21 | 16 | 47,06 | 133 | 35,28 | 89 | 32,96 | 1.00 (.-.) | . | 1.37 (0.74-2.55) | 0.32 | 0.35 | 0.99 | 0.83 |
| MTR ---- tag |  | C/T | 23 | 39,66 | 11 | 32,35 | 178 | 47,21 | 124 | 45,93 | 0.96 (0.40-2.31) | 0.93 | 1.38 (0.75-2.54) | 0.30 | . | . | . |
| MTR ---- tag |  | T/T | 14 | 24,14 | 7 | 20,59 | 66 | 17,51 | 57 | 21,11 | 2.05 (0.79-5.29) | 0.14 | 1.54 (0.81-2.95) | 0.19 | . | . | . |
| MTR ---- tag | rs4659727 | A/A | 42 | 72,41 | 24 | 70,59 | 249 | 66,05 | 187 | 69,26 | 1.00 (.-.) | . | 1.34 (0.83-2.18) | 0.23 | 0.65 | 0.99 | 0.83 |
| MTR ---- tag |  | A/G | 12 | 20,69 | 9 | 26,47 | 109 | 28,91 | 77 | 28,52 | 1.43 (0.62-3.28) | 0.40 | 1.32 (0.79-2.22) | 0.29 | . | . | . |
| MTR ---- tag |  | G/G | 4 | 6,90 | 1 | 2,94 | 19 | 5,04 | 6 | 2,22 | 0.00 (0.00-1E239) | 0.97 | 0.59 (0.23-1.53) | 0.28 | . | . | . |
| MTRR ---- candidate literature/tag | rs10380 | C/C | 53 | 91,38 | 23 | 67,65 | 319 | 84,62 | 226 | 83,70 | 1.00 (.-.) | . | 1.49 (0.92-2.43) | 0.11 | 0.12 | 0.99 | 0.90 |
| MTRR ---- candidate literature/tag |  | C/T | 5 | 8,62 | 11 | 32,35 | 58 | 15,38 | 43 | 15,93 | 3.13 (1.38-7.12) | 0.01 | 1.52 (0.86-2.69) | 0.15 | . | . | . |
| MTRR ---- candidate literature/tag |  | T/T | 0 | 0,00 | 0 | 0,00 | 0 | 0,00 | 1 | 0,37 | 2E281 (0.00-I) | 0.54 | 3E281 (0.00-I) | 0.54 | . | . | . |
| MTRR ---- tag | rs10475399 | G/G | 26 | 44,83 | 11 | 32,35 | 165 | 43,77 | 115 | 42,59 | 1.00 (.-.) | . | 1.23 (0.65-2.31) | 0.53 | 0.74 | 0.99 | 0.90 |
| MTRR ---- tag |  | G/A | 24 | 41,38 | 18 | 52,94 | 163 | 43,24 | 127 | 47,04 | 1.00 (0.43-2.32) | 0.99 | 1.40 (0.75-2.62) | 0.30 | . | . | . |
| MTRR ---- tag |  | A/A | 8 | 13,79 | 5 | 14,71 | 49 | 13,00 | 28 | 10,37 | 1.19 (0.41-3.51) | 0.75 | 0.95 (0.45-1.99) | 0.89 | . | . | . |
| MTRR ---- tag | rs11134265 | C/C | 23 | 39,66 | 12 | 35,29 | 169 | 44,83 | 121 | 44,81 | 1.00 (.-.) | . | 1.17 (0.62-2.19) | 0.63 | 0.82 | 0.99 | 0.90 |
| MTRR ---- tag |  | C/T | 28 | 48,28 | 19 | 55,88 | 159 | 42,18 | 122 | 45,19 | 1.01 (0.45-2.29) | 0.98 | 1.32 (0.70-2.48) | 0.39 | . | . | . |
| MTRR ---- tag |  | T/T | 7 | 12,07 | 3 | 8,82 | 49 | 13,00 | 27 | 10,00 | 0.87 (0.24-3.18) | 0.83 | 1.08 (0.51-2.27) | 0.84 | . | . | . |
| MTRR ---- tag | rs13181011 | T/T | 35 | 60,34 | 20 | 58,82 | 239 | 63,40 | 169 | 62,59 | 1.00 (.-.) | . | 1.27 (0.78-2.09) | 0.34 | 0.51 | 0.99 | 0.90 |
| MTRR ---- tag |  | T/C | 21 | 36,21 | 12 | 35,29 | 128 | 33,95 | 89 | 32,96 | 0.87 (0.36-2.11) | 0.75 | 1.23 (0.73-2.07) | 0.43 | . | . | . |
| MTRR ---- tag |  | C/C | 2 | 3,45 | 2 | 5,88 | 10 | 2,65 | 12 | 4,44 | 3.22 (0.71-14.54) | 0.13 | 1.11 (0.51-2.45) | 0.79 | . | . | . |
| MTRR ---- tag | rs161869 | C/C | 25 | 43,10 | 7 | 20,59 | 125 | 33,16 | 93 | 34,44 | 1.00 (.-.) | . | 1.70 (0.67-4.29) | 0.27 | 0.53 | 0.99 | 0.90 |
| MTRR ---- tag |  | C/T | 23 | 39,66 | 19 | 55,88 | 195 | 51,72 | 131 | 48,52 | 1.65 (0.60-4.57) | 0.33 | 1.94 (0.78-4.83) | 0.15 | . | . | . |
| MTRR ---- tag |  | T/T | 10 | 17,24 | 8 | 23,53 | 57 | 15,12 | 46 | 17,04 | 1.96 (0.59-6.53) | 0.27 | 2.21 (0.86-5.72) | 0.10 | . | . | . |
| MTRR ---- tagged by rs162039 | rs162036 | A/A | 52 | 89,66 | 21 | 61,76 | 304 | 80,64 | 215 | 79,63 | 1.00 (.-.) | . | 1.49 (0.90-2.44) | 0.12 | 0.12 | 0.99 | 0.90 |
| MTRR ---- tagged by rs162039 |  | A/G | 6 | 10,34 | 13 | 38,24 | 71 | 18,83 | 52 | 19,26 | 2.46 (1.06-5.74) | 0.04 | 1.64 (0.94-2.85) | 0.08 | . | . | . |
| MTRR ---- tagged by rs162039 |  | G/G | 0 | 0,00 | 0 | 0,00 | 2 | 0,53 | 3 | 1,11 | 2.41 (0.75-7.74) | 0.14 | 3.58 (1.02-12.52) | 0.05 | . | . | . |
| MTRR ---- tag | rs162039 | C/C | 52 | 89,66 | 21 | 61,76 | 305 | 80,90 | 215 | 79,63 | 1.00 (.-.) | . | 1.49 (0.90-2.44) | 0.12 | 0.12 | 0.99 | 0.90 |
| MTRR ---- tag |  | C/T | 6 | 10,34 | 13 | 38,24 | 70 | 18,57 | 52 | 19,26 | 2.46 (1.06-5.74) | 0.04 | 1.64 (0.94-2.85) | 0.08 | . | . | . |
| MTRR ---- tag |  | T/T | 0 | 0,00 | 0 | 0,00 | 2 | 0,53 | 3 | 1,11 | 2.41 (0.75-7.74) | 0.14 | 3.58 (1.02-12.52) | 0.05 | . | . | . |
| MTRR ---- tag | rs162270 | G/G | 41 | 70,69 | 24 | 70,59 | 271 | 71,88 | 191 | 70,74 | 1.00 (.-.) | . | 1.15 (0.71-1.87) | 0.57 | 0.58 | 0.93 | 0.90 |
| MTRR ---- tag |  | G/T | 12 | 20,69 | 8 | 23,53 | 94 | 24,93 | 72 | 26,67 | 0.93 (0.36-2.37) | 0.88 | 1.29 (0.75-2.20) | 0.36 | . | . | . |
| MTRR ---- tag |  | T/T | 5 | 8,62 | 2 | 5,88 | 12 | 3,18 | 7 | 2,59 | 0.55 (0.13-2.42) | 0.43 | 0.62 (0.23-1.70) | 0.35 | . | . | . |
| MTRR ---- candidate | rs16879334 | C/C | 54 | 93,10 | 33 | 97,06 | 354 | 93,90 | 253 | 93,70 | 1.00 (.-.) | . | 1.23 (0.81-1.88) | 0.33 | 0.83 | 0.97 | 0.90 |
| MTRR ---- candidate |  | C/G | 4 | 6,90 | 1 | 2,94 | 23 | 6,10 | 17 | 6,30 | 1.00 (.-.) | . | 1.23 (0.81-1.88) | 0.33 | . | . | . |
| MTRR ---- candidate |  | G/G | . | . | . | . | . | . | . | . | 0.75 (0.10-5.61) | 0.78 | 1.15 (0.60-2.21) | 0.67 | . | . | . |
| MTRR ----candidate | rs1801394 | G/G | 19 | 32,76 | 10 | 29,41 | 119 | 31,56 | 80 | 29,63 | 1.00 (.-.) | . | 1.03 (0.50-2.09) | 0.94 | 0.68 | 0.99 | 0.90 |
| MTRR ---- candidate |  | G/A | 26 | 44,83 | 18 | 52,94 | 183 | 48,54 | 143 | 52,96 | 0.93 (0.39-2.23) | 0.87 | 1.30 (0.65-2.59) | 0.46 | . | . | . |
| MTRR ---- candidate |  | A/A | 13 | 22,41 | 6 | 17,65 | 75 | 19,89 | 47 | 17,41 | 0.85 (0.30-2.43) | 0.76 | 1.03 (0.49-2.15) | 0.94 | . | . | . |
| MTRR ---- tag | rs1802059 | G/G | 26 | 44,83 | 17 | 50,00 | 145 | 38,46 | 104 | 38,52 | 1.00 (.-.) | . | 1.23 (0.70-2.16) | 0.47 | 0.98 | 0.99 | 0.90 |
| MTRR ---- tag |  | G/A | 23 | 39,66 | 15 | 44,12 | 178 | 47,21 | 126 | 46,67 | 0.96 (0.43-2.12) | 0.92 | 1.23 (0.71-2.14) | 0.46 | . | . | . |
| MTRR ---- tag |  | A/A | 9 | 15,52 | 2 | 5,88 | 54 | 14,32 | 40 | 14,81 | 0.83 (0.19-3.73) | 0.81 | 1.05 (0.56-1.97) | 0.87 | . | . | . |
| MTRR ---- tag | rs2077744 | T/T | 39 | 67,24 | 25 | 73,53 | 277 | 73,47 | 199 | 73,70 | 1.00 (.-.) | . | 1.18 (0.72-1.94) | 0.52 | 0.61 | 0.99 | 0.90 |
| MTRR ---- tag |  | T/C | 15 | 25,86 | 9 | 26,47 | 89 | 23,61 | 66 | 24,44 | 0.95 (0.42-2.15) | 0.91 | 1.26 (0.72-2.19) | 0.42 | . | . | . |
| MTRR ---- tag |  | C/C | 4 | 6,90 | 0 | 0,00 | 11 | 2,92 | 5 | 1,85 | 0.00 (0.00-1E240) | 0.97 | 0.70 (0.26-1.94) | 0.50 | . | . | . |
| MTRR ---- candidate | rs2287780 | C/C | 54 | 93,10 | 33 | 97,06 | 354 | 93,90 | 253 | 93,70 | 1.00 (.-.) | . | 1.23 (0.81-1.88) | 0.33 | 0.83 | 0.97 | 0.90 |
| MTRR ---- candidate |  | C/T | 4 | 6,90 | 1 | 2,94 | 23 | 6,10 | 17 | 6,30 | 1.00 (.-.) | . | 1.23 (0.81-1.88) | 0.33 | . | . | . |
| MTRR ---- candidate |  | T/T | . | . | . | . | . | . | . | . | 0.75 (0.10-5.61) | 0.78 | 1.15 (0.60-2.21) | 0.67 | . | . | . |
| MTRR ---- candidate | rs2303080 | T/T | 54 | 93,10 | 33 | 97,06 | 354 | 93,90 | 254 | 94,07 | 1.00 (.-.) | . | 1.23 (0.81-1.88) | 0.33 | 0.84 | 0.96 | 0.90 |
| MTRR ---- candidate |  | T/A | 4 | 6,90 | 1 | 2,94 | 23 | 6,10 | 16 | 5,93 | 1.00 (.-.) | . | 1.23 (0.81-1.88) | 0.33 | . | . | . |
| MTRR ---- candidate |  | A/A | . | . | . | . | . | . | . | . | 0.75 (0.10-5.60) | 0.78 | 1.14 (0.58-2.21) | 0.70 | . | . | . |
| MTRR ---- tag | rs7715062 | G/G | 21 | 36,21 | 13 | 38,24 | 127 | 33,69 | 91 | 33,70 | 1.00 (.-.) | . | 1.54 (0.81-2.94) | 0.19 | 0.35 | 1.00 | 0.99 |
| MTRR ---- tag |  | G/T | 26 | 44,83 | 18 | 52,94 | 188 | 49,87 | 131 | 48,52 | 1.32 (0.59-2.97) | 0.50 | 1.47 (0.78-2.77) | 0.23 | . | . | . |
| MTRR ---- tag |  | T/T | 11 | 18,97 | 3 | 8,82 | 62 | 16,45 | 48 | 17,78 | 1.45 (0.39-5.33) | 0.58 | 1.31 (0.66-2.60) | 0.44 | . | . | . |
| MTRR ---- tag | rs9282787 | T/T | 37 | 63,79 | 21 | 61,76 | 241 | 63,93 | 171 | 63,33 | 1.00 (.-.) | . | 1.31 (0.80-2.15) | 0.28 | 0.47 | 0.93 | 0.90 |
| MTRR ---- tag |  | T/C | 18 | 31,03 | 11 | 32,35 | 126 | 33,42 | 87 | 32,22 | 1.04 (0.43-2.50) | 0.94 | 1.27 (0.76-2.15) | 0.36 | . | . | . |
| MTRR ---- tag |  | C/C | 3 | 5,17 | 2 | 5,88 | 10 | 2,65 | 12 | 4,44 | 2.08 (0.46-9.36) | 0.34 | 1.15 (0.52-2.53) | 0.73 | . | . | . |
| MTRR ---- candidate literature | rs9332 | C/C | 52 | 89,66 | 21 | 61,76 | 305 | 80,90 | 215 | 79,63 | 1.00 (.-.) | . | 1.49 (0.90-2.44) | 0.12 | 0.12 | 0.99 | 0.90 |
| MTRR ---- candidate literature |  | C/T | 6 | 10,34 | 13 | 38,24 | 70 | 18,57 | 52 | 19,26 | 2.46 (1.06-5.74) | 0.04 | 1.64 (0.94-2.85) | 0.08 | . | . | . |
| MTRR ---- candidate literature |  | T/T | 0 | 0,00 | 0 | 0,00 | 2 | 0,53 | 3 | 1,11 | 2.41 (0.75-7.74) | 0.14 | 3.58 (1.02-12.52) | 0.05 | . | . | . |
| NFKB1 ---- NA | rs1609798 | C/C | 19 | 32,76 | 17 | 50,00 | 174 | 46,15 | 130 | 48,15 | 1.00 (.-.) | . | 1.25 (0.72-2.17) | 0.44 | 0.72 | 0.99 | 0.96 |
| NFKB1 ---- NA |  | C/T | 28 | 48,28 | 15 | 44,12 | 170 | 45,09 | 113 | 41,85 | 1.10 (0.49-2.45) | 0.82 | 1.18 (0.67-2.07) | 0.58 | . | . | . |
| NFKB1 ---- NA |  | T/T | 11 | 18,97 | 2 | 5,88 | 33 | 8,75 | 27 | 10,00 | 0.51 (0.11-2.28) | 0.38 | 1.10 (0.56-2.17) | 0.79 | . | . | . |
| NFKB1 ---- tag | rs230540 | T/T | 18 | 31,03 | 14 | 41,18 | 156 | 41,38 | 113 | 41,85 | 1.00 (.-.) | . | 1.23 (0.67-2.24) | 0.50 | 0.63 | 0.96 | 0.96 |
| NFKB1 ---- tag |  | T/C | 25 | 43,10 | 18 | 52,94 | 177 | 46,95 | 126 | 46,67 | 1.15 (0.52-2.54) | 0.73 | 1.25 (0.69-2.28) | 0.46 | . | . | . |
| NFKB1 ---- tag |  | C/C | 15 | 25,86 | 2 | 5,88 | 44 | 11,67 | 31 | 11,48 | 0.35 (0.08-1.59) | 0.17 | 0.79 (0.40-1.59) | 0.51 | . | . | . |
| NFKB1 ---- tag | rs230541 | A/A | 14 | 24,14 | 11 | 32,35 | 123 | 32,63 | 94 | 34,81 | 1.00 (.-.) | . | 1.06 (0.54-2.08) | 0.86 | 0.68 | 0.99 | 0.96 |
| NFKB1 ---- tag |  | A/G | 26 | 44,83 | 16 | 47,06 | 185 | 49,07 | 135 | 50,00 | 0.91 (0.39-2.15) | 0.84 | 1.22 (0.63-2.36) | 0.56 | . | . | . |
| NFKB1 ---- tag |  | G/G | 18 | 31,03 | 7 | 20,59 | 69 | 18,30 | 41 | 15,19 | 0.65 (0.23-1.81) | 0.41 | 0.77 (0.37-1.60) | 0.49 | . | . | . |
| NFKB1 ---- NA | rs230547 | C/C | 52 | 89,66 | 26 | 76,47 | 290 | 76,92 | 228 | 84,44 | 1.00 (.-.) | . | 1.43 (0.90-2.27) | 0.14 | 0.18 | 0.99 | 0.96 |
| NFKB1 ---- NA |  | C/T | 6 | 10,34 | 8 | 23,53 | 86 | 22,81 | 38 | 14,07 | 1.60 (0.67-3.81) | 0.29 | 1.02 (0.58-1.80) | 0.94 | . | . | . |
| NFKB1 ---- NA |  | T/T | 0 | 0,00 | 0 | 0,00 | 1 | 0,27 | 4 | 1,48 | 1.52 (0.54-4.29) | 0.43 | 2.17 (0.71-6.65) | 0.17 | . | . | . |
| NFKB1 ---- tag | rs3774934 | G/G | 50 | 86,21 | 26 | 76,47 | 285 | 75,60 | 221 | 81,85 | 1.00 (.-.) | . | 1.40 (0.88-2.23) | 0.16 | 0.27 | 0.99 | 0.96 |
| NFKB1 ---- tag |  | G/A | 8 | 13,79 | 8 | 23,53 | 88 | 23,34 | 45 | 16,67 | 1.47 (0.62-3.50) | 0.39 | 1.09 (0.63-1.90) | 0.75 | . | . | . |
| NFKB1 ---- tag |  | A/A | 0 | 0,00 | 0 | 0,00 | 4 | 1,06 | 4 | 1,48 | 1.22 (0.44-3.33) | 0.70 | 1.70 (0.57-5.08) | 0.35 | . | . | . |
| NFKB1 ---- tag | rs3774968 | G/G | 29 | 50,00 | 10 | 29,41 | 119 | 31,56 | 80 | 29,63 | 1.00 (.-.) | . | 2.00 (0.95-4.23) | 0.07 | 0.19 | 0.83 | 0.96 |
| NFKB1 ---- tag |  | G/A | 20 | 34,48 | 17 | 50,00 | 197 | 52,25 | 137 | 50,74 | 2.31 (0.94-5.66) | 0.07 | 2.06 (0.98-4.31) | 0.06 | . | . | . |
| NFKB1 ---- tag |  | A/A | 9 | 15,52 | 7 | 20,59 | 61 | 16,18 | 53 | 19,63 | 2.11 (0.75-5.90) | 0.16 | 2.23 (1.03-4.86) | 0.04 | . | . | . |
| NFKB1 ---- NA | rs4648022 | C/C | 45 | 77,59 | 32 | 94,12 | 318 | 84,35 | 233 | 86,30 | 1.00 (.-.) | . | 1.13 (0.73-1.74) | 0.58 | 0.27 | 0.99 | 0.96 |
| NFKB1 ---- NA |  | C/T | 13 | 22,41 | 2 | 5,88 | 56 | 14,85 | 36 | 13,33 | 0.52 (0.12-2.23) | 0.38 | 1.48 (0.86-2.55) | 0.15 | . | . | . |
| NFKB1 ---- NA |  | T/T | 0 | 0,00 | 0 | 0,00 | 3 | 0,80 | 1 | 0,37 | 0.33 (0.04-2.56) | 0.29 | 0.37 (0.05-2.99) | 0.35 | . | . | . |
| NFKB1 ---- NA | rs4648090 | G/G | 40 | 68,97 | 30 | 88,24 | 281 | 74,54 | 201 | 74,44 | 1.00 (.-.) | . | 1.08 (0.69-1.67) | 0.74 | 0.18 | 0.99 | 0.96 |
| NFKB1 ---- NA |  | G/A | 18 | 31,03 | 4 | 11,76 | 88 | 23,34 | 64 | 23,70 | 0.49 (0.15-1.65) | 0.25 | 1.28 (0.78-2.07) | 0.33 | . | . | . |
| NFKB1 ---- NA |  | A/A | 0 | 0,00 | 0 | 0,00 | 8 | 2,12 | 5 | 1,85 | 0.62 (0.19-2.05) | 0.43 | 0.67 (0.19-2.34) | 0.53 | . | . | . |
| NFKB1 ---- tag | rs4648110 | T/T | 37 | 63,79 | 25 | 73,53 | 246 | 65,25 | 172 | 63,70 | 1.00 (.-.) | . | 0.92 (0.57-1.49) | 0.74 | 0.04 | 0.99 | 0.96 |
| NFKB1 ---- tag |  | T/A | 20 | 34,48 | 9 | 26,47 | 116 | 30,77 | 89 | 32,96 | 0.44 (0.17-1.09) | 0.08 | 1.03 (0.63-1.69) | 0.90 | . | . | . |
| NFKB1 ---- tag |  | A/A | 1 | 1,72 | 0 | 0,00 | 15 | 3,98 | 9 | 3,33 | 0.00 (0.00-1E223) | 0.97 | 0.94 (0.38-2.30) | 0.89 | . | . | . |
| NFKB1 ---- tag | rs4648141 | G/G | 40 | 68,97 | 26 | 76,47 | 269 | 71,35 | 193 | 71,48 | 1.00 (.-.) | . | 0.90 (0.56-1.45) | 0.66 | 0.02 | 0.93 | 0.96 |
| NFKB1 ---- tag |  | G/A | 17 | 29,31 | 8 | 23,53 | 100 | 26,53 | 71 | 26,30 | 0.42 (0.16-1.05) | 0.06 | 1.07 (0.64-1.79) | 0.79 | . | . | . |
| NFKB1 ---- tag |  | A/A | 1 | 1,72 | 0 | 0,00 | 8 | 2,12 | 6 | 2,22 | 0.00 (0.00-6E222) | 0.97 | 1.10 (0.42-2.88) | 0.84 | . | . | . |
| NFKB1 ---- tag | rs4698863 | C/C | 17 | 29,31 | 16 | 47,06 | 169 | 44,83 | 129 | 47,78 | 1.00 (.-.) | . | 1.15 (0.65-2.05) | 0.63 | 0.62 | 0.99 | 0.96 |
| NFKB1 ---- tag |  | C/T | 29 | 50,00 | 15 | 44,12 | 175 | 46,42 | 113 | 41,85 | 0.86 (0.38-1.93) | 0.71 | 1.07 (0.59-1.91) | 0.83 | . | . | . |
| NFKB1 ---- tag |  | T/T | 12 | 20,69 | 3 | 8,82 | 33 | 8,75 | 28 | 10,37 | 0.61 (0.17-2.20) | 0.45 | 1.01 (0.50-2.01) | 0.99 | . | . | . |
| NFKB1 ---- NA | rs7674640 | C/C | 8 | 13,79 | 8 | 23,53 | 87 | 23,08 | 64 | 23,70 | 1.00 (.-.) | . | 0.78 (0.36-1.68) | 0.52 | 0.03 | 0.99 | 0.96 |
| NFKB1 ---- NA |  | C/T | 27 | 46,55 | 22 | 64,71 | 200 | 53,05 | 141 | 52,22 | 0.74 (0.31-1.75) | 0.49 | 0.79 (0.37-1.67) | 0.53 | . | . | . |
| NFKB1 ---- NA |  | T/T | 23 | 39,66 | 4 | 11,76 | 90 | 23,87 | 65 | 24,07 | 0.20 (0.05-0.78) | 0.02 | 0.71 (0.33-1.54) | 0.39 | . | . | . |
| NFKB1 ---- tag | rs909332 | A/A | 54 | 93,10 | 29 | 85,29 | 334 | 88,59 | 252 | 93,33 | 1.00 (.-.) | . | 1.37 (0.87-2.14) | 0.17 | 0.23 | 0.99 | 0.96 |
| NFKB1 ---- tag |  | A/T | 4 | 6,90 | 5 | 14,71 | 42 | 11,14 | 17 | 6,30 | 1.95 (0.72-5.24) | 0.19 | 1.18 (0.60-2.33) | 0.63 | . | . | . |
| NFKB1 ---- tag |  | T/T | 0 | 0,00 | 0 | 0,00 | 1 | 0,27 | 1 | 0,37 | 3.73 (0.50-27.78) | 0.20 | 5.09 (0.66-39.17) | 0.12 | . | . | . |
| NFKB1 ---- tag | rs997476 | C/C | 54 | 93,10 | 29 | 85,29 | 339 | 89,92 | 240 | 88,89 | 1.00 (.-.) | . | 1.10 (0.71-1.71) | 0.66 | 0.19 | 0.66 | 0.90 |
| NFKB1 ---- tag |  | C/A | 4 | 6,90 | 5 | 14,71 | 38 | 10,08 | 29 | 10,74 | 0.51 (0.15-1.72) | 0.28 | 1.23 (0.70-2.16) | 0.48 | . | . | . |
| NFKB1 ---- tag |  | A/A | 0 | 0,00 | 0 | 0,00 | 0 | 0,00 | 1 | 0,37 | 1.41 (0.19-10.58) | 0.74 | 1.56 (0.20-12.08) | 0.67 | . | . | . |
| NME1 ---- NA | rs10514981 | T/T | 31 | 53,45 | 22 | 64,71 | 230 | 61,01 | 175 | 64,81 | 1.00 (.-.) | . | 1.25 (0.75-2.08) | 0.39 | 0.71 | 0.85 | 0.89 |
| NME1 ---- NA |  | T/G | 25 | 43,10 | 10 | 29,41 | 130 | 34,48 | 88 | 32,59 | 0.86 (0.38-1.92) | 0.71 | 1.15 (0.67-1.97) | 0.61 | . | . | . |
| NME1 ---- NA |  | G/G | 2 | 3,45 | 2 | 5,88 | 17 | 4,51 | 7 | 2,59 | 4.36 (0.55-34.40) | 0.16 | 0.96 (0.37-2.49) | 0.93 | . | . | . |
| NME1 ---- NA | rs11651252 | T/T | 48 | 82,76 | 27 | 79,41 | 340 | 90,19 | 240 | 88,89 | 1.00 (.-.) | . | 1.45 (0.92-2.30) | 0.11 | 0.05 | 0.99 | 0.89 |
| NME1 ---- NA |  | T/C | 10 | 17,24 | 7 | 20,59 | 36 | 9,55 | 30 | 11,11 | 2.76 (1.09-6.98) | 0.03 | 1.36 (0.75-2.47) | 0.31 | . | . | . |
| NME1 ---- NA |  | C/C | 0 | 0,00 | 0 | 0,00 | 1 | 0,27 | 0 | 0,00 | 0.00 (0.00-I) | 0.98 | 0.00 (0.00-I) | 0.98 | . | . | . |
| NME1 ---- tag | rs11652793 | T/T | 34 | 58,62 | 24 | 70,59 | 240 | 63,66 | 189 | 70,00 | 1.00 (.-.) | . | 1.16 (0.71-1.88) | 0.55 | 0.65 | 0.93 | 0.89 |
| NME1 ---- tag |  | T/C | 24 | 41,38 | 9 | 26,47 | 126 | 33,42 | 75 | 27,78 | 0.76 (0.33-1.73) | 0.51 | 1.09 (0.64-1.84) | 0.75 | . | . | . |
| NME1 ---- tag |  | C/C | 0 | 0,00 | 1 | 2,94 | 11 | 2,92 | 6 | 2,22 | 0.78 (0.31-1.96) | 0.60 | 0.91 (0.33-2.52) | 0.86 | . | . | . |
| NME1 ---- NA | rs11868380 | C/C | 37 | 63,79 | 17 | 50,00 | 234 | 62,07 | 168 | 62,22 | 1.00 (.-.) | . | 1.34 (0.73-2.47) | 0.35 | 0.69 | 1.00 | 0.99 |
| NME1 ---- NA |  | C/G | 19 | 32,76 | 17 | 50,00 | 131 | 34,75 | 89 | 32,96 | 1.37 (0.64-2.94) | 0.42 | 1.36 (0.72-2.56) | 0.35 | . | . | . |
| NME1 ---- NA |  | G/G | 2 | 3,45 | 0 | 0,00 | 12 | 3,18 | 13 | 4,81 | 0.00 (0.00-I) | 0.98 | 1.64 (0.72-3.75) | 0.24 | . | . | . |
| NME1 ---- NA | rs1558252 | T/T | 32 | 55,17 | 19 | 55,88 | 194 | 51,46 | 117 | 43,33 | 1.00 (.-.) | . | 1.21 (0.68-2.13) | 0.52 | 0.75 | 0.99 | 0.99 |
| NME1 ---- NA |  | T/C | 25 | 43,10 | 9 | 26,47 | 149 | 39,52 | 123 | 45,56 | 0.94 (0.39-2.26) | 0.90 | 1.44 (0.82-2.53) | 0.20 | . | . | . |
| NME1 ---- NA |  | C/C | 1 | 1,72 | 6 | 17,65 | 34 | 9,02 | 30 | 11,11 | 1.84 (0.64-5.24) | 0.26 | 1.52 (0.78-2.97) | 0.22 | . | . | . |
| NME1 ---- NA | rs1558253 | T/T | 56 | 96,55 | 31 | 91,18 | 336 | 89,12 | 234 | 86,67 | 1.00 (.-.) | . | 1.28 (0.83-1.98) | 0.26 | 0.59 | 0.90 | 0.89 |
| NME1 ---- NA |  | T/G | 2 | 3,45 | 3 | 8,82 | 41 | 10,88 | 36 | 13,33 | 1.00 (.-.) | . | 1.28 (0.83-1.98) | 0.26 | . | . | . |
| NME1 ---- NA |  | G/G | 0 | 0,00 | 0 | 0,00 | 0 | 0,00 | 0 | 0,00 | 1.48 (0.43-5.04) | 0.53 | 1.32 (0.77-2.26) | 0.32 | . | . | . |
| NME1 ---- tag | rs16949683 | C/C | 53 | 91,38 | 29 | 85,29 | 355 | 94,16 | 250 | 92,59 | 1.00 (.-.) | . | 1.43 (0.91-2.25) | 0.12 | 0.02 | 0.99 | 0.89 |
| NME1 ---- tag |  | C/T | 5 | 8,62 | 5 | 14,71 | 21 | 5,57 | 19 | 7,04 | 4.82 (1.79-12.99) | 0.00 | 1.64 (0.85-3.17) | 0.14 | . | . | . |
| NME1 ---- tag |  | T/T | 0 | 0,00 | 0 | 0,00 | 1 | 0,27 | 1 | 0,37 | 1.32 (0.18-9.70) | 0.79 | 1.89 (0.25-14.56) | 0.54 | . | . | . |
| NME1 ---- tag | rs2318784 | C/C | 39 | 67,24 | 23 | 67,65 | 295 | 78,25 | 202 | 74,81 | 1.00 (.-.) | . | 1.35 (0.83-2.20) | 0.23 | 0.46 | 0.99 | 0.89 |
| NME1 ---- tag |  | C/T | 17 | 29,31 | 10 | 29,41 | 77 | 20,42 | 64 | 23,70 | 1.46 (0.61-3.53) | 0.40 | 1.46 (0.86-2.48) | 0.16 | . | . | . |
| NME1 ---- tag |  | T/T | 2 | 3,45 | 1 | 2,94 | 5 | 1,33 | 4 | 1,48 | 1.68 (0.22-13.08) | 0.62 | 1.10 (0.32-3.83) | 0.88 | . | . | . |
| NME1 ---- NA | rs2318785 | G/G | 19 | 32,76 | 13 | 38,24 | 115 | 30,50 | 75 | 27,78 | 1.00 (.-.) | . | 1.29 (0.65-2.56) | 0.46 | 0.76 | 0.99 | 0.89 |
| NME1 ---- NA |  | G/A | 30 | 51,72 | 18 | 52,94 | 193 | 51,19 | 148 | 54,81 | 1.33 (0.59-2.96) | 0.49 | 1.44 (0.74-2.78) | 0.28 | . | . | . |
| NME1 ---- NA |  | A/A | 9 | 15,52 | 3 | 8,82 | 69 | 18,30 | 47 | 17,41 | 0.53 (0.11-2.50) | 0.42 | 1.24 (0.62-2.51) | 0.55 | . | . | . |
| NME1 ---- tag | rs3760469 | G/G | 20 | 34,48 | 12 | 35,29 | 96 | 25,46 | 62 | 22,96 | 1.00 (.-.) | . | 1.45 (0.70-2.99) | 0.32 | 0.85 | 0.99 | 0.99 |
| NME1 ---- tag |  | G/T | 28 | 48,28 | 16 | 47,06 | 196 | 51,99 | 149 | 55,19 | 1.55 (0.66-3.65) | 0.32 | 1.58 (0.79-3.16) | 0.20 | . | . | . |
| NME1 ---- tag |  | T/T | 10 | 17,24 | 6 | 17,65 | 85 | 22,55 | 59 | 21,85 | 0.96 (0.29-3.18) | 0.94 | 1.44 (0.70-2.96) | 0.33 | . | . | . |
| NME1 ---- NA | rs4605213 | G/G | 23 | 39,66 | 15 | 44,12 | 152 | 40,32 | 118 | 43,70 | 1.00 (.-.) | . | 1.54 (0.83-2.85) | 0.17 | 0.43 | 0.93 | 0.89 |
| NME1 ---- NA |  | G/C | 28 | 48,28 | 13 | 38,24 | 182 | 48,28 | 122 | 45,19 | 1.38 (0.60-3.16) | 0.45 | 1.40 (0.76-2.61) | 0.28 | . | . | . |
| NME1 ---- NA |  | C/C | 7 | 12,07 | 6 | 17,65 | 43 | 11,41 | 30 | 11,11 | 1.52 (0.53-4.36) | 0.44 | 1.77 (0.87-3.59) | 0.12 | . | . | . |
| NME1 ---- NA | rs7207090 | A/A | 15 | 25,86 | 10 | 29,41 | 96 | 25,46 | 64 | 23,70 | 1.00 (.-.) | . | 1.74 (0.80-3.78) | 0.16 | 0.27 | 0.99 | 0.89 |
| NME1 ---- NA |  | A/T | 35 | 60,34 | 16 | 47,06 | 199 | 52,79 | 129 | 47,78 | 1.24 (0.50-3.03) | 0.64 | 1.48 (0.71-3.10) | 0.30 | . | . | . |
| NME1 ---- NA |  | T/T | 8 | 13,79 | 8 | 23,53 | 82 | 21,75 | 77 | 28,52 | 1.97 (0.70-5.58) | 0.20 | 1.71 (0.80-3.65) | 0.17 | . | . | . |
| NME1 ---- tag | rs7222463 | A/A | 10 | 17,24 | 8 | 23,53 | 99 | 26,26 | 76 | 28,15 | 1.00 (.-.) | . | 1.23 (0.48-3.16) | 0.67 | 0.99 | 0.93 | 0.89 |
| NME1 ---- tag |  | A/C | 38 | 65,52 | 17 | 50,00 | 193 | 51,19 | 137 | 50,74 | 0.98 (0.35-2.76) | 0.97 | 1.23 (0.49-3.12) | 0.66 | . | . | . |
| NME1 ---- tag |  | C/C | 10 | 17,24 | 9 | 26,47 | 85 | 22,55 | 57 | 21,11 | 1.04 (0.33-3.25) | 0.94 | 1.29 (0.49-3.37) | 0.60 | . | . | . |
| NME1 ---- tag | rs7226059 | C/C | 27 | 46,55 | 13 | 38,24 | 159 | 42,18 | 119 | 44,07 | 1.00 (.-.) | . | 1.84 (0.99-3.41) | 0.05 | 0.20 | 0.83 | 0.89 |
| NME1 ---- tag |  | C/T | 21 | 36,21 | 15 | 44,12 | 177 | 46,95 | 122 | 45,19 | 2.45 (1.05-5.70) | 0.04 | 1.65 (0.89-3.06) | 0.11 | . | . | . |
| NME1 ---- tag |  | T/T | 10 | 17,24 | 6 | 17,65 | 41 | 10,88 | 29 | 10,74 | 1.34 (0.46-3.87) | 0.59 | 1.58 (0.77-3.21) | 0.21 | . | . | . |
| NME1 ---- NA | rs880178 | G/G | 13 | 22,41 | 10 | 29,41 | 98 | 25,99 | 62 | 22,96 | 1.00 (.-.) | . | 1.09 (0.51-2.36) | 0.82 | 0.79 | 0.99 | 0.99 |
| NME1 ---- NA |  | G/T | 35 | 60,34 | 18 | 52,94 | 199 | 52,79 | 146 | 54,07 | 0.93 (0.39-2.22) | 0.87 | 1.20 (0.58-2.49) | 0.63 | . | . | . |
| NME1 ---- NA |  | T/T | 10 | 17,24 | 6 | 17,65 | 80 | 21,22 | 62 | 22,96 | 0.96 (0.31-2.99) | 0.94 | 1.22 (0.57-2.61) | 0.60 | . | . | . |
| NME2 ---- tag | rs7220360 | C/C | 10 | 17,24 | 8 | 23,53 | 99 | 26,26 | 76 | 28,15 | 1.00 (.-.) | . | 1.23 (0.48-3.16) | 0.67 | 0.99 | 0.93 | 0.43 |
| NME2 ---- tag |  | C/G | 38 | 65,52 | 17 | 50,00 | 193 | 51,19 | 137 | 50,74 | 0.98 (0.35-2.76) | 0.97 | 1.23 (0.49-3.12) | 0.66 | . | . | . |
| NME2 ---- tag |  | G/G | 10 | 17,24 | 9 | 26,47 | 85 | 22,55 | 57 | 21,11 | 1.04 (0.33-3.25) | 0.94 | 1.29 (0.49-3.37) | 0.60 | . | . | . |
| PON1 ---- tag | rs2269829 | A/A | 33 | 56,90 | 19 | 55,88 | 203 | 53,85 | 125 | 46,30 | 1.00 (.-.) | . | 1.28 (0.74-2.21) | 0.38 | 0.88 | 0.66 | 0.08 |
| PON1 ---- tag |  | A/G | 20 | 34,48 | 12 | 35,29 | 150 | 39,79 | 114 | 42,22 | 1.31 (0.58-2.94) | 0.52 | 1.38 (0.79-2.39) | 0.26 | . | . | . |
| PON1 ---- tag |  | G/G | 5 | 8,62 | 3 | 8,82 | 24 | 6,37 | 31 | 11,48 | 0.95 (0.21-4.29) | 0.95 | 1.82 (0.94-3.53) | 0.08 | . | . | . |
| PON1 ---- tag | rs3917527 | A/A | 52 | 89,66 | 32 | 94,12 | 334 | 88,59 | 253 | 93,70 | 1.00 (.-.) | . | 1.35 (0.88-2.06) | 0.17 | 0.31 | 0.59 | 0.08 |
| PON1 ---- tag |  | A/G | 6 | 10,34 | 2 | 5,88 | 41 | 10,88 | 16 | 5,93 | 1.28 (0.30-5.50) | 0.74 | 0.64 (0.34-1.23) | 0.18 | . | . | . |
| PON1 ---- tag |  | G/G | 0 | 0,00 | 0 | 0,00 | 2 | 0,53 | 1 | 0,37 | 0.99 (0.13-7.30) | 0.99 | 1.34 (0.17-10.21) | 0.78 | . | . | . |
| PON1 ---- tag | rs3917538 | C/C | 37 | 63,79 | 20 | 58,82 | 231 | 61,27 | 132 | 48,89 | 1.00 (.-.) | . | 1.21 (0.71-2.06) | 0.49 | 0.36 | 0.59 | 0.04 |
| PON1 ---- tag |  | C/T | 18 | 31,03 | 12 | 35,29 | 137 | 36,34 | 112 | 41,48 | 1.55 (0.69-3.47) | 0.29 | 1.54 (0.89-2.64) | 0.12 | . | . | . |
| PON1 ---- tag |  | T/T | 3 | 5,17 | 2 | 5,88 | 9 | 2,39 | 26 | 9,63 | 0.55 (0.07-4.30) | 0.57 | 2.97 (1.51-5.85) | 0.00 | . | . | . |
| PON1 ---- tag | rs757158 | C/C | 20 | 34,48 | 11 | 32,35 | 144 | 38,20 | 102 | 37,78 | 1.00 (.-.) | . | 1.45 (0.74-2.83) | 0.28 | 0.94 | 0.61 | 0.08 |
| PON1 ---- tag |  | C/T | 25 | 43,10 | 17 | 50,00 | 176 | 46,68 | 124 | 45,93 | 1.09 (0.48-2.49) | 0.84 | 1.05 (0.54-2.04) | 0.89 | . | . | . |
| PON1 ---- tag |  | T/T | 13 | 22,41 | 6 | 17,65 | 57 | 15,12 | 44 | 16,30 | 0.63 (0.19-2.06) | 0.45 | 1.29 (0.62-2.66) | 0.50 | . | . | . |
| PON1 ---- candidate | rs854560 | A/A | 20 | 34,48 | 13 | 38,24 | 129 | 34,22 | 121 | 44,81 | 1.00 (.-.) | . | 1.30 (0.67-2.55) | 0.44 | 0.51 | 0.99 | 0.87 |
| PON1 ---- candidate |  | A/T | 25 | 43,10 | 18 | 52,94 | 188 | 49,87 | 101 | 37,41 | 1.21 (0.53-2.74) | 0.65 | 1.11 (0.57-2.20) | 0.75 | . | . | . |
| PON1 ---- candidate |  | T/T | 13 | 22,41 | 3 | 8,82 | 60 | 15,92 | 48 | 17,78 | 0.61 (0.16-2.26) | 0.46 | 1.64 (0.79-3.39) | 0.18 | . | . | . |
| PRDM2 ---- tag | rs1015370 | C/C | 25 | 43,10 | 22 | 64,71 | 196 | 51,99 | 157 | 58,15 | 1.00 (.-.) | . | 1.31 (0.78-2.22) | 0.31 | 0.93 | 0.88 | 0.60 |
| PRDM2 ---- tag |  | C/T | 23 | 39,66 | 10 | 29,41 | 144 | 38,20 | 92 | 34,07 | 0.99 (0.44-2.26) | 0.99 | 1.06 (0.62-1.83) | 0.82 | . | . | . |
| PRDM2 ---- tag |  | T/T | 10 | 17,24 | 2 | 5,88 | 37 | 9,81 | 21 | 7,78 | 0.76 (0.17-3.41) | 0.72 | 1.18 (0.60-2.31) | 0.63 | . | . | . |
| PRDM2 ---- tag | rs1203634 | A/A | 37 | 63,79 | 24 | 70,59 | 240 | 63,66 | 162 | 60,00 | 1.00 (.-.) | . | 1.13 (0.70-1.84) | 0.61 | 0.84 | 0.59 | 0.16 |
| PRDM2 ---- tag |  | A/G | 18 | 31,03 | 6 | 17,65 | 118 | 31,30 | 86 | 31,85 | 0.63 (0.23-1.69) | 0.35 | 1.23 (0.73-2.07) | 0.43 | . | . | . |
| PRDM2 ---- tag |  | G/G | 3 | 5,17 | 4 | 11,76 | 19 | 5,04 | 22 | 8,15 | 1.63 (0.47-5.66) | 0.44 | 1.28 (0.68-2.43) | 0.44 | . | . | . |
| PRDM2 ---- tag | rs1203645 | A/A | 27 | 46,55 | 14 | 41,18 | 162 | 42,97 | 106 | 39,26 | 1.00 (.-.) | . | 1.28 (0.69-2.38) | 0.44 | 0.91 | 0.59 | 0.16 |
| PRDM2 ---- tag |  | A/C | 21 | 36,21 | 11 | 32,35 | 163 | 43,24 | 113 | 41,85 | 1.22 (0.50-2.95) | 0.66 | 1.47 (0.79-2.74) | 0.23 | . | . | . |
| PRDM2 ---- tag |  | C/C | 10 | 17,24 | 9 | 26,47 | 52 | 13,79 | 51 | 18,89 | 1.23 (0.47-3.20) | 0.67 | 1.49 (0.77-2.89) | 0.24 | . | . | . |
| PRDM2 ---- tag | rs1406416 | C/C | 33 | 56,90 | 15 | 44,12 | 194 | 51,46 | 140 | 51,85 | 1.00 (.-.) | . | 1.22 (0.64-2.33) | 0.55 | 0.95 | 0.99 | 0.87 |
| PRDM2 ---- tag |  | C/T | 24 | 41,38 | 12 | 35,29 | 147 | 38,99 | 105 | 38,89 | 0.98 (0.42-2.30) | 0.96 | 1.23 (0.64-2.37) | 0.54 | . | . | . |
| PRDM2 ---- tag |  | T/T | 1 | 1,72 | 7 | 20,59 | 36 | 9,55 | 25 | 9,26 | 0.95 (0.34-2.64) | 0.92 | 1.18 (0.56-2.50) | 0.67 | . | . | . |
| PRDM2 ---- candidate | rs17350795 | G/G | 57 | 98,28 | 33 | 97,06 | 359 | 95,23 | 256 | 94,81 | 1.00 (.-.) | . | 1.23 (0.80-1.87) | 0.34 | 0.75 | 0.99 | 0.87 |
| PRDM2 ---- candidate |  | G/A | 1 | 1,72 | 1 | 2,94 | 18 | 4,77 | 14 | 5,19 | 1.00 (.-.) | . | 1.23 (0.80-1.87) | 0.34 | . | . | . |
| PRDM2 ---- candidate |  | A/A | . | . | . | . | . | . | . | . | 0.53 (0.07-4.03) | 0.54 | 0.90 (0.44-1.85) | 0.78 | . | . | . |
| PRDM2 ---- tag | rs1980472 | C/C | 36 | 62,07 | 21 | 61,76 | 218 | 57,82 | 144 | 53,33 | 1.00 (.-.) | . | 1.17 (0.70-1.96) | 0.55 | 0.88 | 0.59 | 0.16 |
| PRDM2 ---- tag |  | C/G | 17 | 29,31 | 9 | 26,47 | 135 | 35,81 | 98 | 36,30 | 0.79 (0.32-1.91) | 0.59 | 1.18 (0.69-2.02) | 0.54 | . | . | . |
| PRDM2 ---- tag |  | G/G | 5 | 8,62 | 4 | 11,76 | 24 | 6,37 | 28 | 10,37 | 1.25 (0.36-4.34) | 0.73 | 1.29 (0.69-2.42) | 0.42 | . | . | . |
| PRDM2 ---- tag | rs2235515 | G/G | 33 | 56,90 | 19 | 55,88 | 229 | 60,74 | 159 | 58,89 | 1.00 (.-.) | . | 1.22 (0.69-2.16) | 0.49 | 0.86 | 0.83 | 0.43 |
| PRDM2 ---- tag |  | G/A | 24 | 41,38 | 14 | 41,18 | 128 | 33,95 | 98 | 36,30 | 1.00 (0.47-2.14) | 1.00 | 1.38 (0.77-2.47) | 0.29 | . | . | . |
| PRDM2 ---- tag |  | A/A | 1 | 1,72 | 1 | 2,94 | 20 | 5,31 | 13 | 4,81 | 1.88 (0.23-15.30) | 0.55 | 1.01 (0.45-2.26) | 0.99 | . | . | . |
| PRDM2 ---- tag | rs2244634 | A/A | 33 | 56,90 | 24 | 70,59 | 247 | 65,52 | 181 | 67,04 | 1.00 (.-.) | . | 1.21 (0.75-1.94) | 0.44 | 0.83 | 0.99 | 0.99 |
| PRDM2 ---- tag |  | A/C | 22 | 37,93 | 10 | 29,41 | 108 | 28,65 | 81 | 30,00 | 1.01 (0.43-2.39) | 0.98 | 1.33 (0.80-2.22) | 0.28 | . | . | . |
| PRDM2 ---- tag |  | C/C | 3 | 5,17 | 0 | 0,00 | 22 | 5,84 | 8 | 2,96 | 0.00 (0.00-4E236) | 0.97 | 0.74 (0.32-1.71) | 0.47 | . | . | . |
| PRDM2 ---- tag | rs2245213 | G/G | 39 | 67,24 | 25 | 73,53 | 278 | 73,74 | 198 | 73,33 | 1.00 (.-.) | . | 1.28 (0.79-2.06) | 0.32 | 0.62 | 0.97 | 0.87 |
| PRDM2 ---- tag |  | G/T | 19 | 32,76 | 9 | 26,47 | 87 | 23,08 | 69 | 25,56 | 1.13 (0.49-2.63) | 0.78 | 1.41 (0.83-2.38) | 0.20 | . | . | . |
| PRDM2 ---- tag |  | T/T | 0 | 0,00 | 0 | 0,00 | 12 | 3,18 | 3 | 1,11 | 0.32 (0.10-1.02) | 0.05 | 0.41 (0.12-1.41) | 0.16 | . | . | . |
| PRDM2 ---- tag | rs2294484 | C/C | 48 | 82,76 | 26 | 76,47 | 316 | 83,82 | 222 | 82,22 | 1.00 (.-.) | . | 1.18 (0.75-1.87) | 0.47 | 0.62 | 0.99 | 0.87 |
| PRDM2 ---- tag |  | C/G | 10 | 17,24 | 8 | 23,53 | 56 | 14,85 | 47 | 17,41 | 1.03 (0.41-2.58) | 0.94 | 1.78 (1.04-3.05) | 0.04 | . | . | . |
| PRDM2 ---- tag |  | G/G | 0 | 0,00 | 0 | 0,00 | 5 | 1,33 | 1 | 0,37 | 0.53 (0.07-3.83) | 0.53 | 0.62 (0.08-4.69) | 0.65 | . | . | . |
| PRDM2 ---- tag | rs2744689 | G/G | 40 | 68,97 | 26 | 76,47 | 279 | 74,01 | 199 | 73,70 | 1.00 (.-.) | . | 1.27 (0.80-2.02) | 0.32 | 0.68 | 1.00 | 0.99 |
| PRDM2 ---- tag |  | G/A | 18 | 31,03 | 8 | 23,53 | 85 | 22,55 | 67 | 24,81 | 1.09 (0.45-2.66) | 0.85 | 1.38 (0.82-2.31) | 0.22 | . | . | . |
| PRDM2 ---- tag |  | A/A | 0 | 0,00 | 0 | 0,00 | 13 | 3,45 | 4 | 1,48 | 0.37 (0.14-1.02) | 0.06 | 0.47 (0.16-1.40) | 0.18 | . | . | . |
| PRDM2 ---- tag | rs6690270 | A/A | 26 | 44,83 | 10 | 29,41 | 142 | 37,67 | 106 | 39,26 | 1.00 (.-.) | . | 1.50 (0.71-3.16) | 0.29 | 0.74 | 0.89 | 0.60 |
| PRDM2 ---- tag |  | A/G | 27 | 46,55 | 20 | 58,82 | 181 | 48,01 | 123 | 45,56 | 1.52 (0.65-3.58) | 0.34 | 1.38 (0.65-2.90) | 0.40 | . | . | . |
| PRDM2 ---- tag |  | G/G | 5 | 8,62 | 4 | 11,76 | 54 | 14,32 | 41 | 15,19 | 0.55 (0.14-2.13) | 0.39 | 1.43 (0.65-3.16) | 0.38 | . | . | . |
| RRM1 ---- tag | rs10835601 | G/G | 26 | 44,83 | 21 | 61,76 | 187 | 49,60 | 130 | 48,15 | 1.00 (.-.) | . | 1.09 (0.64-1.85) | 0.75 | 0.70 | 1.00 | 1.00 |
| RRM1 ---- tag |  | G/A | 26 | 44,83 | 10 | 29,41 | 156 | 41,38 | 114 | 42,22 | 0.68 (0.29-1.60) | 0.38 | 1.15 (0.68-1.95) | 0.61 | . | . | . |
| RRM1 ---- tag |  | A/A | 6 | 10,34 | 3 | 8,82 | 34 | 9,02 | 26 | 9,63 | 1.12 (0.32-3.91) | 0.86 | 1.08 (0.56-2.06) | 0.83 | . | . | . |
| RRM1 ---- tag | rs10835613 | C/C | 20 | 34,48 | 12 | 35,29 | 139 | 36,87 | 96 | 35,56 | 1.00 (.-.) | . | 1.23 (0.65-2.34) | 0.53 | 0.96 | 0.99 | 0.94 |
| RRM1 ---- tag |  | C/G | 28 | 48,28 | 16 | 47,06 | 178 | 47,21 | 127 | 47,04 | 1.02 (0.44-2.34) | 0.97 | 1.26 (0.67-2.36) | 0.48 | . | . | . |
| RRM1 ---- tag |  | G/G | 10 | 17,24 | 6 | 17,65 | 60 | 15,92 | 47 | 17,41 | 1.13 (0.38-3.30) | 0.83 | 1.43 (0.72-2.81) | 0.31 | . | . | . |
| RRM1 ---- NA | rs10835677 | G/G | 49 | 84,48 | 31 | 91,18 | 303 | 80,37 | 223 | 82,59 | 1.00 (.-.) | . | 1.19 (0.77-1.83) | 0.43 | 0.46 | 0.99 | 0.94 |
| RRM1 ---- NA |  | G/A | 9 | 15,52 | 3 | 8,82 | 70 | 18,57 | 43 | 15,93 | 0.59 (0.14-2.52) | 0.47 | 1.12 (0.67-1.87) | 0.67 | . | . | . |
| RRM1 ---- NA |  | A/A | 0 | 0,00 | 0 | 0,00 | 4 | 1,06 | 4 | 1,48 | 1.36 (0.49-3.76) | 0.55 | 1.62 (0.55-4.80) | 0.39 | . | . | . |
| RRM1 ---- tag | rs10835678 | A/A | 53 | 91,38 | 30 | 88,24 | 332 | 88,06 | 244 | 90,37 | 1.00 (.-.) | . | 1.29 (0.84-1.98) | 0.24 | 0.47 | 0.99 | 0.94 |
| RRM1 ---- tag |  | A/G | 5 | 8,62 | 4 | 11,76 | 43 | 11,41 | 26 | 9,63 | 1.60 (0.36-7.06) | 0.54 | 1.20 (0.67-2.14) | 0.54 | . | . | . |
| RRM1 ---- tag |  | G/G | 0 | 0,00 | 0 | 0,00 | 2 | 0,53 | 0 | 0,00 | 0.00 (0.00-2E268) | 0.97 | 0.00 (0.00-2E268) | 0.97 | . | . | . |
| RRM1 ---- tag | rs12288551 | C/C | 53 | 91,38 | 33 | 97,06 | 348 | 92,31 | 237 | 87,78 | 1.00 (.-.) | . | 1.20 (0.79-1.83) | 0.38 | 0.69 | 0.93 | 0.94 |
| RRM1 ---- tag |  | C/G | 5 | 8,62 | 1 | 2,94 | 29 | 7,69 | 32 | 11,85 | 1.00 (.-.) | . | 1.20 (0.79-1.83) | 0.38 | . | . | . |
| RRM1 ---- tag |  | G/G | 0 | 0,00 | 0 | 0,00 | 0 | 0,00 | 1 | 0,37 | 0.92 (0.12-6.87) | 0.93 | 1.65 (0.94-2.87) | 0.08 | . | . | . |
| RRM1 ---- NA | rs12806698 | C/C | 29 | 50,00 | 19 | 55,88 | 203 | 53,85 | 139 | 51,48 | 1.00 (.-.) | . | 1.08 (0.64-1.82) | 0.77 | 0.79 | 0.99 | 0.94 |
| RRM1 ---- NA |  | C/A | 23 | 39,66 | 11 | 32,35 | 143 | 37,93 | 111 | 41,11 | 0.60 (0.24-1.49) | 0.27 | 1.15 (0.68-1.94) | 0.61 | . | . | . |
| RRM1 ---- NA |  | A/A | 6 | 10,34 | 4 | 11,76 | 31 | 8,22 | 20 | 7,41 | 1.43 (0.47-4.36) | 0.53 | 1.23 (0.61-2.46) | 0.56 | . | . | . |
| RRM1 ---- NA | rs1465952 | T/T | 47 | 81,03 | 25 | 73,53 | 308 | 81,70 | 227 | 84,07 | 1.00 (.-.) | . | 1.27 (0.81-1.99) | 0.30 | 0.81 | 0.99 | 1.00 |
| RRM1 ---- NA |  | T/C | 11 | 18,97 | 9 | 26,47 | 66 | 17,51 | 42 | 15,56 | 0.98 (0.36-2.64) | 0.97 | 1.15 (0.67-1.97) | 0.61 | . | . | . |
| RRM1 ---- NA |  | C/C | 0 | 0,00 | 0 | 0,00 | 3 | 0,80 | 1 | 0,37 | 0.00 (0.00-I) | 0.98 | 0.00 (0.00-I) | 0.98 | . | . | . |
| RRM1 ---- tag | rs4910904 | A/A | 25 | 43,10 | 14 | 41,18 | 180 | 47,75 | 112 | 41,48 | 1.00 (.-.) | . | 1.09 (0.59-2.02) | 0.78 | 0.66 | 0.93 | 0.94 |
| RRM1 ---- tag |  | A/G | 23 | 39,66 | 16 | 47,06 | 158 | 41,91 | 125 | 46,30 | 0.90 (0.40-2.04) | 0.81 | 1.25 (0.68-2.28) | 0.48 | . | . | . |
| RRM1 ---- tag |  | G/G | 10 | 17,24 | 4 | 11,76 | 39 | 10,34 | 33 | 12,22 | 1.11 (0.35-3.54) | 0.85 | 1.47 (0.73-2.94) | 0.28 | . | . | . |
| RRM1 ---- tag | rs7103860 | T/T | 44 | 75,86 | 22 | 64,71 | 293 | 77,72 | 216 | 80,00 | 1.00 (.-.) | . | 1.28 (0.79-2.08) | 0.31 | 0.71 | 0.93 | 0.94 |
| RRM1 ---- tag |  | T/C | 14 | 24,14 | 12 | 35,29 | 78 | 20,69 | 53 | 19,63 | 1.14 (0.49-2.64) | 0.77 | 1.37 (0.80-2.36) | 0.25 | . | . | . |
| RRM1 ---- tag |  | C/C | 0 | 0,00 | 0 | 0,00 | 6 | 1,59 | 1 | 0,37 | 0.00 (0.00-5E219) | 0.96 | 0.00 (0.00-7E219) | 0.96 | . | . | . |
| RRM1 ---- tag | rs7115496 | C/C | 53 | 91,38 | 30 | 88,24 | 321 | 85,15 | 240 | 88,89 | 1.00 (.-.) | . | 1.29 (0.84-1.98) | 0.24 | 0.49 | 0.98 | 0.94 |
| RRM1 ---- tag |  | C/T | 5 | 8,62 | 4 | 11,76 | 52 | 13,79 | 30 | 11,11 | 1.45 (0.33-6.40) | 0.62 | 1.19 (0.68-2.09) | 0.53 | . | . | . |
| RRM1 ---- tag |  | T/T | 0 | 0,00 | 0 | 0,00 | 4 | 1,06 | 0 | 0,00 | 0.00 (0.00-4E286) | 0.97 | 0.00 (0.00-5E286) | 0.97 | . | . | . |
| RRM2 ---- NA | rs1138729 | A/A | 40 | 68,97 | 23 | 67,65 | 266 | 70,56 | 206 | 76,30 | 1.00 (.-.) | . | 1.35 (0.81-2.25) | 0.25 | 0.47 | 0.88 | 0.77 |
| RRM2 ---- NA |  | A/G | 17 | 29,31 | 10 | 29,41 | 103 | 27,32 | 55 | 20,37 | 1.21 (0.53-2.75) | 0.65 | 1.33 (0.76-2.33) | 0.33 | . | . | . |
| RRM2 ---- NA |  | G/G | 1 | 1,72 | 1 | 2,94 | 8 | 2,12 | 9 | 3,33 | 2.25 (0.29-17.48) | 0.44 | 1.27 (0.53-3.05) | 0.59 | . | . | . |
| RRM2 ---- tag | rs4668664 | G/G | 25 | 43,10 | 16 | 47,06 | 185 | 49,07 | 138 | 51,11 | 1.00 (.-.) | . | 1.24 (0.68-2.28) | 0.48 | 0.81 | 0.93 | 0.77 |
| RRM2 ---- tag |  | G/A | 26 | 44,83 | 16 | 47,06 | 151 | 40,05 | 108 | 40,00 | 1.04 (0.48-2.25) | 0.93 | 1.20 (0.65-2.22) | 0.56 | . | . | . |
| RRM2 ---- tag |  | A/A | 7 | 12,07 | 2 | 5,88 | 41 | 10,88 | 24 | 8,89 | 0.55 (0.07-4.37) | 0.57 | 1.26 (0.61-2.62) | 0.53 | . | . | . |
| RRM2 ---- NA | rs6741290 | C/C | 14 | 24,14 | 11 | 32,35 | 110 | 29,18 | 97 | 35,93 | 1.00 (.-.) | . | 1.22 (0.58-2.56) | 0.60 | 0.82 | 0.99 | 0.77 |
| RRM2 ---- NA |  | C/T | 28 | 48,28 | 14 | 41,18 | 184 | 48,81 | 116 | 42,96 | 0.78 (0.31-1.93) | 0.59 | 1.04 (0.50-2.17) | 0.91 | . | . | . |
| RRM2 ---- NA |  | T/T | 16 | 27,59 | 9 | 26,47 | 83 | 22,02 | 57 | 21,11 | 1.17 (0.43-3.19) | 0.76 | 1.29 (0.60-2.76) | 0.52 | . | . | . |
| RRM2 ---- tag | rs7574663 | C/C | 35 | 60,34 | 22 | 64,71 | 228 | 60,48 | 182 | 67,41 | 1.00 (.-.) | . | 1.18 (0.71-1.98) | 0.52 | 0.71 | 0.99 | 0.77 |
| RRM2 ---- tag |  | C/G | 17 | 29,31 | 11 | 32,35 | 135 | 35,81 | 75 | 27,78 | 0.87 (0.38-1.96) | 0.74 | 1.11 (0.65-1.92) | 0.70 | . | . | . |
| RRM2 ---- tag |  | G/G | 6 | 10,34 | 1 | 2,94 | 14 | 3,71 | 13 | 4,81 | 0.67 (0.09-5.07) | 0.70 | 1.19 (0.55-2.54) | 0.66 | . | . | . |
| SHMT1 ---- candidate | rs1979277 | G/G | 32 | 55,17 | 18 | 52,94 | 166 | 44,03 | 135 | 50,00 | 1.00 (.-.) | . | 1.23 (0.72-2.10) | 0.46 | 0.86 | 0.99 | 0.93 |
| SHMT1 ---- candidate |  | G/A | 21 | 36,21 | 11 | 32,35 | 174 | 46,15 | 113 | 41,85 | 0.70 (0.29-1.64) | 0.41 | 0.97 (0.56-1.68) | 0.92 | . | . | . |
| SHMT1 ---- candidate |  | A/A | 5 | 8,62 | 5 | 14,71 | 37 | 9,81 | 22 | 8,15 | 1.09 (0.35-3.34) | 0.88 | 1.17 (0.58-2.36) | 0.67 | . | . | . |
| SHMT1 ---- tag | rs2168781 | G/G | 25 | 43,10 | 14 | 41,18 | 116 | 30,77 | 99 | 36,67 | 1.00 (.-.) | . | 1.22 (0.67-2.23) | 0.51 | 0.90 | 0.99 | 0.93 |
| SHMT1 ---- tag |  | G/C | 24 | 41,38 | 14 | 41,18 | 199 | 52,79 | 126 | 46,67 | 0.87 (0.37-2.03) | 0.75 | 1.08 (0.60-1.97) | 0.79 | . | . | . |
| SHMT1 ---- tag |  | C/C | 9 | 15,52 | 6 | 17,65 | 62 | 16,45 | 45 | 16,67 | 0.86 (0.30-2.45) | 0.78 | 1.15 (0.60-2.21) | 0.68 | . | . | . |
| SHMT1 ---- tag | rs4924849 | C/C | 32 | 55,17 | 18 | 52,94 | 176 | 46,68 | 142 | 52,59 | 1.00 (.-.) | . | 1.20 (0.70-2.05) | 0.51 | 0.94 | 0.99 | 0.93 |
| SHMT1 ---- tag |  | C/T | 21 | 36,21 | 11 | 32,35 | 167 | 44,30 | 106 | 39,26 | 0.70 (0.30-1.65) | 0.41 | 0.98 (0.57-1.70) | 0.95 | . | . | . |
| SHMT1 ---- tag |  | T/T | 5 | 8,62 | 5 | 14,71 | 34 | 9,02 | 22 | 8,15 | 1.09 (0.36-3.35) | 0.88 | 1.21 (0.60-2.46) | 0.59 | . | . | . |
| SHMT1 ---- candidate literature | rs9909104 | T/T | 23 | 39,66 | 19 | 55,88 | 200 | 53,05 | 149 | 55,19 | 1.00 (.-.) | . | 0.93 (0.54-1.63) | 0.81 | 0.43 | 0.99 | 0.93 |
| SHMT1 ---- candidate literature |  | T/C | 30 | 51,72 | 11 | 32,35 | 152 | 40,32 | 104 | 38,52 | 0.49 (0.21-1.13) | 0.09 | 0.90 (0.51-1.59) | 0.73 | . | . | . |
| SHMT1 ---- candidate literature |  | C/C | 5 | 8,62 | 4 | 11,76 | 25 | 6,63 | 17 | 6,30 | 0.95 (0.31-2.95) | 0.93 | 0.97 (0.46-2.05) | 0.93 | . | . | . |
| SHMT2 ---- tag | rs10876968 | G/G | 28 | 48,28 | 18 | 52,94 | 199 | 52,79 | 148 | 54,81 | 1.00 (.-.) | . | 1.14 (0.66-1.97) | 0.63 | 0.63 | 0.99 | 0.81 |
| SHMT2 ---- tag |  | G/T | 25 | 43,10 | 14 | 41,18 | 140 | 37,14 | 105 | 38,89 | 0.91 (0.41-2.01) | 0.82 | 1.26 (0.72-2.21) | 0.42 | . | . | . |
| SHMT2 ---- tag |  | T/T | 5 | 8,62 | 2 | 5,88 | 38 | 10,08 | 17 | 6,30 | 0.55 (0.07-4.18) | 0.56 | 0.91 (0.43-1.92) | 0.80 | . | . | . |
| SHMT2 ---- tag | rs1800165 | T/T | 26 | 44,83 | 18 | 52,94 | 185 | 49,07 | 128 | 47,41 | 1.00 (.-.) | . | 1.16 (0.66-2.03) | 0.60 | 0.96 | 0.83 | 0.38 |
| SHMT2 ---- tag |  | T/C | 26 | 44,83 | 14 | 41,18 | 156 | 41,38 | 113 | 41,85 | 0.88 (0.40-1.94) | 0.75 | 1.25 (0.71-2.19) | 0.44 | . | . | . |
| SHMT2 ---- tag |  | C/C | 6 | 10,34 | 2 | 5,88 | 36 | 9,55 | 29 | 10,74 | 2.09 (0.47-9.44) | 0.34 | 1.35 (0.70-2.58) | 0.37 | . | . | . |
| SHMT2 ---- tag | rs7133939 | T/T | 12 | 20,69 | 11 | 32,35 | 114 | 30,24 | 86 | 31,85 | 1.00 (.-.) | . | 1.07 (0.56-2.07) | 0.83 | 0.34 | 0.99 | 0.81 |
| SHMT2 ---- tag |  | T/A | 32 | 55,17 | 18 | 52,94 | 179 | 47,48 | 128 | 47,41 | 1.01 (0.44-2.28) | 0.99 | 1.14 (0.60-2.17) | 0.68 | . | . | . |
| SHMT2 ---- tag |  | A/A | 14 | 24,14 | 5 | 14,71 | 84 | 22,28 | 56 | 20,74 | 0.48 (0.13-1.77) | 0.27 | 1.10 (0.56-2.16) | 0.79 | . | . | . |
| SHMT2 ---- tag | rs7485577 | G/G | 31 | 53,45 | 19 | 55,88 | 208 | 55,17 | 139 | 51,48 | 1.00 (.-.) | . | 1.26 (0.74-2.17) | 0.40 | 0.54 | 0.85 | 0.38 |
| SHMT2 ---- tag |  | G/A | 23 | 39,66 | 13 | 38,24 | 142 | 37,67 | 106 | 39,26 | 1.05 (0.47-2.36) | 0.90 | 1.39 (0.80-2.42) | 0.24 | . | . | . |
| SHMT2 ---- tag |  | A/A | 4 | 6,90 | 2 | 5,88 | 27 | 7,16 | 25 | 9,26 | 3.21 (0.72-14.27) | 0.13 | 1.42 (0.74-2.72) | 0.29 | . | . | . |
| SHMT2 ---- tag | rs7489231 | T/T | 23 | 39,66 | 16 | 47,06 | 168 | 44,56 | 120 | 44,44 | 1.00 (.-.) | . | 1.20 (0.69-2.10) | 0.52 | 0.75 | 0.79 | 0.38 |
| SHMT2 ---- tag |  | T/C | 30 | 51,72 | 15 | 44,12 | 168 | 44,56 | 113 | 41,85 | 0.95 (0.43-2.11) | 0.91 | 1.27 (0.73-2.24) | 0.40 | . | . | . |
| SHMT2 ---- tag |  | C/C | 5 | 8,62 | 3 | 8,82 | 41 | 10,88 | 37 | 13,70 | 3.09 (0.69-13.88) | 0.14 | 1.51 (0.81-2.82) | 0.19 | . | . | . |
| SLC19A1 ---- candidate | rs1051266 | G/G | 15 | 25,86 | 12 | 35,29 | 120 | 31,83 | 76 | 28,15 | 1.00 (.-.) | . | 0.81 (0.42-1.54) | 0.52 | 0.21 | 0.85 | 0.33 |
| SLC19A1 ---- candidate |  | G/A | 31 | 53,45 | 15 | 44,12 | 185 | 49,07 | 141 | 52,22 | 0.51 (0.22-1.21) | 0.13 | 0.82 (0.44-1.54) | 0.54 | . | . | . |
| SLC19A1 ---- candidate |  | A/A | 12 | 20,69 | 7 | 20,59 | 72 | 19,10 | 53 | 19,63 | 0.53 (0.19-1.45) | 0.22 | 0.78 (0.40-1.50) | 0.45 | . | . | . |
| SLC19A1 ---- candidate literature | rs1131596 | T/T | 16 | 27,59 | 12 | 35,29 | 120 | 31,83 | 76 | 28,15 | 1.00 (.-.) | . | 0.86 (0.45-1.64) | 0.65 | 0.26 | 0.86 | 0.33 |
| SLC19A1 ---- candidate literature |  | T/C | 30 | 51,72 | 15 | 44,12 | 185 | 49,07 | 141 | 52,22 | 0.57 (0.24-1.33) | 0.19 | 0.88 (0.47-1.64) | 0.68 | . | . | . |
| SLC19A1 ---- candidate literature |  | C/C | 12 | 20,69 | 7 | 20,59 | 72 | 19,10 | 53 | 19,63 | 0.57 (0.21-1.54) | 0.27 | 0.83 (0.43-1.59) | 0.57 | . | . | . |
| SLC19A1 ---- tag | rs12483553 | G/G | 46 | 79,31 | 25 | 73,53 | 304 | 80,64 | 217 | 80,37 | 1.00 (.-.) | . | 1.42 (0.87-2.31) | 0.16 | 0.22 | 0.83 | 0.33 |
| SLC19A1 ---- tag |  | G/A | 12 | 20,69 | 9 | 26,47 | 68 | 18,04 | 52 | 19,26 | 1.88 (0.83-4.24) | 0.13 | 1.62 (0.94-2.82) | 0.09 | . | . | . |
| SLC19A1 ---- tag |  | A/A | 0 | 0,00 | 0 | 0,00 | 5 | 1,33 | 1 | 0,37 | 0.46 (0.06-3.35) | 0.45 | 0.66 (0.09-5.01) | 0.69 | . | . | . |
| SLC19A1 ---- candidate literature | rs12659 | C/C | 15 | 25,86 | 12 | 35,29 | 125 | 33,16 | 81 | 30,00 | 1.00 (.-.) | . | 0.82 (0.43-1.57) | 0.56 | 0.26 | 0.85 | 0.33 |
| SLC19A1 ---- candidate literature |  | C/T | 33 | 56,90 | 15 | 44,12 | 179 | 47,48 | 137 | 50,74 | 0.50 (0.21-1.17) | 0.11 | 0.83 (0.44-1.55) | 0.55 | . | . | . |
| SLC19A1 ---- candidate literature |  | T/T | 10 | 17,24 | 7 | 20,59 | 73 | 19,36 | 52 | 19,26 | 0.56 (0.21-1.55) | 0.27 | 0.76 (0.39-1.47) | 0.42 | . | . | . |
| SLC19A1 ---- tag | rs3788190 | G/G | 14 | 24,14 | 12 | 35,29 | 120 | 31,83 | 77 | 28,52 | 1.00 (.-.) | . | 0.78 (0.41-1.48) | 0.45 | 0.17 | 0.88 | 0.33 |
| SLC19A1 ---- tag |  | G/A | 33 | 56,90 | 16 | 47,06 | 178 | 47,21 | 134 | 49,63 | 0.46 (0.20-1.05) | 0.07 | 0.75 (0.40-1.41) | 0.37 | . | . | . |
| SLC19A1 ---- tag |  | A/A | 11 | 18,97 | 6 | 17,65 | 79 | 20,95 | 59 | 21,85 | 0.52 (0.18-1.51) | 0.23 | 0.75 (0.39-1.44) | 0.39 | . | . | . |
| SLC19A1 ---- tag | rs3788205 | C/C | 24 | 41,38 | 19 | 55,88 | 188 | 49,87 | 150 | 55,56 | 1.00 (.-.) | . | 1.11 (0.64-1.92) | 0.72 | 0.69 | 0.93 | 0.46 |
| SLC19A1 ---- tag |  | C/T | 32 | 55,17 | 9 | 26,47 | 154 | 40,85 | 100 | 37,04 | 0.53 (0.21-1.32) | 0.17 | 1.11 (0.63-1.96) | 0.72 | . | . | . |
| SLC19A1 ---- tag |  | T/T | 2 | 3,45 | 6 | 17,65 | 35 | 9,28 | 20 | 7,41 | 1.79 (0.67-4.81) | 0.25 | 1.01 (0.49-2.09) | 0.98 | . | . | . |
| SLC19A1 ---- tag | rs7279664 | G/G | 20 | 34,48 | 15 | 44,12 | 148 | 39,26 | 114 | 42,22 | 1.00 (.-.) | . | 0.90 (0.49-1.65) | 0.74 | 0.10 | 0.82 | 0.33 |
| SLC19A1 ---- tag |  | G/T | 27 | 46,55 | 18 | 52,94 | 168 | 44,56 | 116 | 42,96 | 0.60 (0.28-1.32) | 0.21 | 0.78 (0.42-1.42) | 0.41 | . | . | . |
| SLC19A1 ---- tag |  | T/T | 11 | 18,97 | 1 | 2,94 | 61 | 16,18 | 40 | 14,81 | 0.15 (0.02-1.19) | 0.07 | 0.68 (0.35-1.31) | 0.25 | . | . | . |
| SLC29A1 ---- NA | rs1057985 | C/C | 20 | 34,48 | 11 | 32,35 | 166 | 44,03 | 127 | 47,04 | 1.00 (.-.) | . | 1.61 (0.77-3.34) | 0.21 | 0.52 | 0.83 | 0.31 |
| SLC29A1 ---- NA |  | C/T | 30 | 51,72 | 21 | 61,76 | 163 | 43,24 | 109 | 40,37 | 1.48 (0.64-3.43) | 0.37 | 1.54 (0.74-3.23) | 0.25 | . | . | . |
| SLC29A1 ---- NA |  | T/T | 8 | 13,79 | 2 | 5,88 | 48 | 12,73 | 34 | 12,59 | 1.36 (0.28-6.67) | 0.70 | 1.85 (0.82-4.14) | 0.14 | . | . | . |
| SLC29A1 ---- NA | rs6458375 | C/C | 37 | 63,79 | 16 | 47,06 | 198 | 52,52 | 154 | 57,04 | 1.00 (.-.) | . | 1.45 (0.80-2.63) | 0.22 | 0.37 | 0.59 | 0.18 |
| SLC29A1 ---- NA |  | C/T | 17 | 29,31 | 16 | 47,06 | 158 | 41,91 | 101 | 37,41 | 1.09 (0.49-2.40) | 0.84 | 1.18 (0.64-2.17) | 0.59 | . | . | . |
| SLC29A1 ---- NA |  | T/T | 4 | 6,90 | 2 | 5,88 | 21 | 5,57 | 15 | 5,56 | 2.08 (0.45-9.62) | 0.35 | 1.53 (0.70-3.34) | 0.29 | . | . | . |
| SLC29A1 ---- NA | rs666462 | C/C | 16 | 27,59 | 9 | 26,47 | 95 | 25,20 | 80 | 29,63 | 1.00 (.-.) | . | 1.39 (0.66-2.95) | 0.39 | 0.97 | 0.99 | 0.90 |
| SLC29A1 ---- NA |  | C/T | 26 | 44,83 | 16 | 47,06 | 199 | 52,79 | 129 | 47,78 | 1.20 (0.49-2.93) | 0.69 | 1.30 (0.63-2.72) | 0.48 | . | . | . |
| SLC29A1 ---- NA |  | T/T | 16 | 27,59 | 9 | 26,47 | 83 | 22,02 | 61 | 22,59 | 1.00 (0.34-2.99) | 0.99 | 1.40 (0.66-3.00) | 0.38 | . | . | . |
| SLC29A1 ---- NA | rs6905285 | A/A | 17 | 29,31 | 12 | 35,29 | 155 | 41,11 | 94 | 34,81 | 1.00 (.-.) | . | 1.06 (0.52-2.19) | 0.87 | 0.86 | 0.94 | 0.65 |
| SLC29A1 ---- NA |  | A/T | 33 | 56,90 | 18 | 52,94 | 172 | 45,62 | 126 | 46,67 | 0.76 (0.33-1.75) | 0.52 | 1.01 (0.49-2.06) | 0.98 | . | . | . |
| SLC29A1 ---- NA |  | T/T | 8 | 13,79 | 4 | 11,76 | 50 | 13,26 | 50 | 18,52 | 1.37 (0.36-5.17) | 0.64 | 1.23 (0.59-2.57) | 0.59 | . | . | . |
| SLC29A1 ---- NA | rs693955 | G/G | 35 | 60,34 | 20 | 58,82 | 248 | 65,78 | 183 | 67,78 | 1.00 (.-.) | . | 1.33 (0.78-2.27) | 0.29 | 0.87 | 0.63 | 0.18 |
| SLC29A1 ---- NA |  | G/T | 21 | 36,21 | 14 | 41,18 | 115 | 30,50 | 75 | 27,78 | 1.34 (0.62-2.88) | 0.45 | 1.40 (0.79-2.47) | 0.25 | . | . | . |
| SLC29A1 ---- NA |  | T/T | 2 | 3,45 | 0 | 0,00 | 14 | 3,71 | 12 | 4,44 | 0.00 (0.00-7E300) | 0.98 | 2.33 (1.01-5.39) | 0.05 | . | . | . |
| SLC29A1 ---- NA | rs747199 | C/C | 34 | 58,62 | 26 | 76,47 | 247 | 65,52 | 177 | 65,56 | 1.00 (.-.) | . | 1.00 (0.63-1.61) | 0.99 | 0.36 | 0.99 | 0.68 |
| SLC29A1 ---- NA |  | C/G | 23 | 39,66 | 6 | 17,65 | 114 | 30,24 | 83 | 30,74 | 0.40 (0.13-1.17) | 0.09 | 1.10 (0.67-1.80) | 0.72 | . | . | . |
| SLC29A1 ---- NA |  | G/G | 1 | 1,72 | 2 | 5,88 | 16 | 4,24 | 10 | 3,70 | 1.51 (0.34-6.80) | 0.59 | 0.86 (0.36-2.03) | 0.73 | . | . | . |
| SLC29A1 ---- NA | rs9357436 | G/G | 38 | 65,52 | 25 | 73,53 | 272 | 72,15 | 197 | 72,96 | 1.00 (.-.) | . | 1.15 (0.71-1.87) | 0.57 | 0.90 | 0.86 | 0.43 |
| SLC29A1 ---- NA |  | G/A | 19 | 32,76 | 7 | 20,59 | 95 | 25,20 | 63 | 23,33 | 0.69 (0.27-1.74) | 0.43 | 1.15 (0.68-1.94) | 0.61 | . | . | . |
| SLC29A1 ---- NA |  | A/A | 1 | 1,72 | 2 | 5,88 | 10 | 2,65 | 10 | 3,70 | 1.71 (0.38-7.71) | 0.49 | 1.21 (0.50-2.92) | 0.68 | . | . | . |
| TCN2 ---- tag | rs10418 | C/C | 34 | 58,62 | 15 | 44,12 | 218 | 57,82 | 156 | 57,78 | 1.00 (.-.) | . | 1.59 (0.86-2.93) | 0.14 | 0.41 | 0.99 | 0.92 |
| TCN2 ---- tag |  | C/T | 21 | 36,21 | 19 | 55,88 | 136 | 36,07 | 104 | 38,52 | 1.75 (0.81-3.79) | 0.15 | 1.73 (0.93-3.23) | 0.09 | . | . | . |
| TCN2 ---- tag |  | T/T | 3 | 5,17 | 0 | 0,00 | 23 | 6,10 | 10 | 3,70 | 0.00 (0.00-1E244) | 0.97 | 1.17 (0.48-2.82) | 0.73 | . | . | . |
| TCN2 ---- candidate/singleton | rs1131603 | T/T | 55 | 94,83 | 29 | 85,29 | 329 | 87,27 | 240 | 88,89 | 1.00 (.-.) | . | 1.39 (0.89-2.17) | 0.14 | 0.17 | 0.99 | 0.91 |
| TCN2 ---- candidate/singleton |  | T/C | 3 | 5,17 | 5 | 14,71 | 47 | 12,47 | 27 | 10,00 | 1.77 (0.60-5.21) | 0.30 | 0.91 (0.51-1.65) | 0.76 | . | . | . |
| TCN2 ---- candidate/singleton |  | C/C | 0 | 0,00 | 0 | 0,00 | 1 | 0,27 | 3 | 1,11 | 1.47 (0.35-6.13) | 0.59 | 2.05 (0.47-8.98) | 0.34 | . | . | . |
| TCN2 ---- tag | rs1544468 | A/A | 20 | 34,48 | 10 | 29,41 | 102 | 27,06 | 65 | 24,07 | 1.00 (.-.) | . | 1.39 (0.65-2.99) | 0.39 | 0.21 | 0.96 | 0.91 |
| TCN2 ---- tag |  | A/G | 26 | 44,83 | 13 | 38,24 | 178 | 47,21 | 136 | 50,37 | 0.98 (0.38-2.50) | 0.97 | 1.55 (0.73-3.29) | 0.26 | . | . | . |
| TCN2 ---- tag |  | G/G | 12 | 20,69 | 11 | 32,35 | 97 | 25,73 | 69 | 25,56 | 2.03 (0.75-5.46) | 0.16 | 1.41 (0.65-3.05) | 0.39 | . | . | . |
| TCN2 ---- candidate/tag | rs1801198 | C/C | 12 | 20,69 | 13 | 38,24 | 115 | 30,50 | 87 | 32,22 | 1.00 (.-.) | . | 0.72 (0.37-1.40) | 0.33 | 0.13 | 0.95 | 0.91 |
| TCN2 ---- candidate/tag |  | C/G | 33 | 56,90 | 13 | 38,24 | 177 | 46,95 | 128 | 47,41 | 0.44 (0.19-1.04) | 0.06 | 0.70 (0.37-1.35) | 0.29 | . | . | . |
| TCN2 ---- candidate/tag |  | G/G | 13 | 22,41 | 8 | 23,53 | 85 | 22,55 | 55 | 20,37 | 0.43 (0.15-1.22) | 0.11 | 0.66 (0.33-1.31) | 0.24 | . | . | . |
| TCN2 ---- tag | rs4820872 | G/G | 15 | 25,86 | 12 | 35,29 | 141 | 37,40 | 105 | 38,89 | 1.00 (.-.) | . | 1.09 (0.55-2.14) | 0.80 | 0.48 | 0.99 | 0.91 |
| TCN2 ---- tag |  | G/A | 31 | 53,45 | 12 | 35,29 | 167 | 44,30 | 112 | 41,48 | 0.90 (0.37-2.22) | 0.83 | 1.05 (0.53-2.07) | 0.89 | . | . | . |
| TCN2 ---- tag |  | A/A | 12 | 20,69 | 10 | 29,41 | 69 | 18,30 | 53 | 19,63 | 0.77 (0.30-1.99) | 0.58 | 1.24 (0.61-2.50) | 0.56 | . | . | . |
| TCN2 ---- tag | rs4820874 | A/A | 44 | 75,86 | 24 | 70,59 | 257 | 68,17 | 201 | 74,44 | 1.00 (.-.) | . | 1.40 (0.86-2.27) | 0.18 | 0.54 | 0.93 | 0.91 |
| TCN2 ---- tag |  | A/G | 13 | 22,41 | 9 | 26,47 | 106 | 28,12 | 63 | 23,33 | 1.45 (0.62-3.38) | 0.39 | 1.29 (0.75-2.21) | 0.36 | . | . | . |
| TCN2 ---- tag |  | G/G | 1 | 1,72 | 1 | 2,94 | 14 | 3,71 | 6 | 2,22 | 0.00 (0.00-2E236) | 0.97 | 0.94 (0.35-2.56) | 0.91 | . | . | . |
| TCN2 ---- tag | rs4820886 | T/T | 50 | 86,21 | 29 | 85,29 | 300 | 79,58 | 220 | 81,48 | 1.00 (.-.) | . | 1.29 (0.83-1.99) | 0.26 | 0.51 | 0.99 | 0.91 |
| TCN2 ---- tag |  | T/G | 8 | 13,79 | 4 | 11,76 | 73 | 19,36 | 47 | 17,41 | 1.56 (0.46-5.27) | 0.48 | 1.33 (0.79-2.23) | 0.28 | . | . | . |
| TCN2 ---- tag |  | G/G | 0 | 0,00 | 1 | 2,94 | 4 | 1,06 | 3 | 1,11 | 0.84 (0.26-2.65) | 0.76 | 1.08 (0.32-3.60) | 0.90 | . | . | . |
| TCN2 ---- candidate | rs4820889 | G/G | 53 | 91,38 | 33 | 97,06 | 347 | 92,04 | 252 | 93,33 | 1.00 (.-.) | . | 1.17 (0.77-1.78) | 0.47 | 0.31 | 0.93 | 0.91 |
| TCN2 ---- candidate |  | G/A | 5 | 8,62 | 1 | 2,94 | 30 | 7,96 | 18 | 6,67 | 1.00 (.-.) | . | 1.17 (0.77-1.78) | 0.47 | . | . | . |
| TCN2 ---- candidate |  | A/A | . | . | . | . | . | . | . | . | 0.29 (0.04-2.15) | 0.23 | 0.87 (0.46-1.65) | 0.67 | . | . | . |
| TCN2 ---- tag | rs5997711 | C/C | 14 | 24,14 | 14 | 41,18 | 127 | 33,69 | 96 | 35,56 | 1.00 (.-.) | . | 0.72 (0.38-1.36) | 0.31 | 0.14 | 0.99 | 0.91 |
| TCN2 ---- tag |  | C/T | 33 | 56,90 | 12 | 35,29 | 177 | 46,95 | 124 | 45,93 | 0.42 (0.18-1.00) | 0.05 | 0.75 (0.40-1.40) | 0.37 | . | . | . |
| TCN2 ---- tag |  | T/T | 11 | 18,97 | 8 | 23,53 | 73 | 19,36 | 50 | 18,52 | 0.50 (0.18-1.37) | 0.18 | 0.70 (0.36-1.35) | 0.28 | . | . | . |
| TCN2 ---- tag | rs740234 | T/T | 41 | 70,69 | 24 | 70,59 | 253 | 67,11 | 166 | 61,48 | 1.00 (.-.) | . | 1.28 (0.77-2.13) | 0.35 | 0.88 | 0.99 | 0.91 |
| TCN2 ---- tag |  | T/C | 15 | 25,86 | 9 | 26,47 | 114 | 30,24 | 90 | 33,33 | 1.27 (0.56-2.88) | 0.56 | 1.32 (0.77-2.28) | 0.32 | . | . | . |
| TCN2 ---- tag |  | C/C | 2 | 3,45 | 1 | 2,94 | 10 | 2,65 | 14 | 5,19 | 0.54 (0.07-4.27) | 0.56 | 1.41 (0.61-3.26) | 0.42 | . | . | . |
| TCN2 ---- tag | rs740235 | G/G | 25 | 43,10 | 12 | 35,29 | 133 | 35,28 | 83 | 30,74 | 1.00 (.-.) | . | 1.38 (0.67-2.83) | 0.38 | 0.34 | 0.90 | 0.91 |
| TCN2 ---- tag |  | G/A | 22 | 37,93 | 14 | 41,18 | 176 | 46,68 | 135 | 50,00 | 1.07 (0.43-2.65) | 0.88 | 1.57 (0.77-3.22) | 0.22 | . | . | . |
| TCN2 ---- tag |  | A/A | 11 | 18,97 | 8 | 23,53 | 68 | 18,04 | 52 | 19,26 | 1.83 (0.68-4.92) | 0.23 | 1.42 (0.66-3.02) | 0.37 | . | . | . |
| TCN2 ---- candidate/singleton | rs9606756 | A/A | 49 | 84,48 | 27 | 79,41 | 297 | 78,78 | 219 | 81,11 | 1.00 (.-.) | . | 1.29 (0.83-2.01) | 0.26 | 0.61 | 0.93 | 0.91 |
| TCN2 ---- candidate/singleton |  | A/G | 9 | 15,52 | 6 | 17,65 | 73 | 19,36 | 48 | 17,78 | 1.31 (0.44-3.84) | 0.63 | 1.28 (0.76-2.15) | 0.35 | . | . | . |
| TCN2 ---- candidate/singleton |  | G/G | 0 | 0,00 | 1 | 2,94 | 7 | 1,86 | 3 | 1,11 | 0.73 (0.18-2.98) | 0.66 | 0.94 (0.22-4.02) | 0.93 | . | . | . |
| TCN2 ---- candidate | rs9621049 | C/C | 50 | 86,21 | 29 | 85,29 | 300 | 79,58 | 220 | 81,48 | 1.00 (.-.) | . | 1.29 (0.83-1.99) | 0.26 | 0.51 | 0.99 | 0.91 |
| TCN2 ---- candidate |  | C/T | 8 | 13,79 | 4 | 11,76 | 73 | 19,36 | 47 | 17,41 | 1.56 (0.46-5.27) | 0.48 | 1.33 (0.79-2.23) | 0.28 | . | . | . |
| TCN2 ---- candidate |  | T/T | 0 | 0,00 | 1 | 2,94 | 4 | 1,06 | 3 | 1,11 | 0.84 (0.26-2.65) | 0.76 | 1.08 (0.32-3.60) | 0.90 | . | . | . |
| TK1 ---- NA | rs1065769 | G/G | 29 | 50,00 | 14 | 41,18 | 178 | 47,21 | 129 | 47,78 | 1.00 (.-.) | . | 1.06 (0.59-1.90) | 0.86 | 0.32 | 0.90 | 0.89 |
| TK1 ---- NA |  | G/A | 24 | 41,38 | 18 | 52,94 | 159 | 42,18 | 120 | 44,44 | 1.04 (0.47-2.30) | 0.91 | 1.34 (0.74-2.43) | 0.34 | . | . | . |
| TK1 ---- NA |  | A/A | 5 | 8,62 | 2 | 5,88 | 40 | 10,61 | 21 | 7,78 | 0.39 (0.09-1.76) | 0.22 | 0.82 (0.40-1.70) | 0.60 | . | . | . |
| TK1 ---- NA | rs12232476 | G/G | 48 | 82,76 | 26 | 76,47 | 319 | 84,62 | 225 | 83,33 | 1.00 (.-.) | . | 1.23 (0.78-1.94) | 0.37 | 0.83 | 0.99 | 0.89 |
| TK1 ---- NA |  | G/A | 10 | 17,24 | 8 | 23,53 | 56 | 14,85 | 42 | 15,56 | 1.00 (0.37-2.68) | 0.99 | 1.30 (0.76-2.23) | 0.34 | . | . | . |
| TK1 ---- NA |  | A/A | 0 | 0,00 | 0 | 0,00 | 2 | 0,53 | 3 | 1,11 | 2.10 (0.50-8.82) | 0.31 | 2.58 (0.57-11.61) | 0.22 | . | . | . |
| TK1 ---- tag | rs16970907 | G/G | 46 | 79,31 | 31 | 91,18 | 332 | 88,06 | 241 | 89,26 | 1.00 (.-.) | . | 1.23 (0.81-1.88) | 0.33 | 0.63 | 0.99 | 0.89 |
| TK1 ---- tag |  | G/C | 11 | 18,97 | 3 | 8,82 | 41 | 10,88 | 29 | 10,74 | 0.49 (0.07-3.68) | 0.49 | 1.25 (0.71-2.22) | 0.44 | . | . | . |
| TK1 ---- tag |  | C/C | 1 | 1,72 | 0 | 0,00 | 4 | 1,06 | 0 | 0,00 | 0.00 (0.00-I) | 0.97 | 0.00 (0.00-I) | 0.97 | . | . | . |
| TK1 ---- tag | rs1811086 | C/C | 54 | 93,10 | 32 | 94,12 | 359 | 95,23 | 243 | 90,00 | 1.00 (.-.) | . | 1.19 (0.77-1.82) | 0.44 | 0.50 | 0.93 | 0.89 |
| TK1 ---- tag |  | C/T | 4 | 6,90 | 2 | 5,88 | 18 | 4,77 | 26 | 9,63 | 0.95 (0.22-4.12) | 0.94 | 1.93 (1.06-3.51) | 0.03 | . | . | . |
| TK1 ---- tag |  | T/T | 0 | 0,00 | 0 | 0,00 | 0 | 0,00 | 1 | 0,37 | 1.78 (0.23-13.48) | 0.58 | 2.11 (0.27-16.51) | 0.48 | . | . | . |
| TK1 ---- tag | rs2292235 | C/C | 20 | 34,48 | 11 | 32,35 | 127 | 33,69 | 88 | 32,59 | 1.00 (.-.) | . | 0.78 (0.41-1.49) | 0.45 | 0.19 | 0.99 | 0.89 |
| TK1 ---- tag |  | C/A | 24 | 41,38 | 14 | 41,18 | 179 | 47,48 | 131 | 48,52 | 0.59 (0.24-1.42) | 0.24 | 0.99 (0.52-1.87) | 0.97 | . | . | . |
| TK1 ---- tag |  | A/A | 14 | 24,14 | 9 | 26,47 | 71 | 18,83 | 51 | 18,89 | 0.61 (0.23-1.59) | 0.31 | 0.88 (0.44-1.75) | 0.71 | . | . | . |
| TK1 ---- tag | rs2854701 | A/A | 24 | 41,38 | 12 | 35,29 | 163 | 43,24 | 100 | 37,04 | 1.00 (.-.) | . | 1.05 (0.55-1.99) | 0.89 | 0.32 | 0.82 | 0.89 |
| TK1 ---- tag |  | A/G | 25 | 43,10 | 21 | 61,76 | 158 | 41,91 | 141 | 52,22 | 1.25 (0.58-2.72) | 0.57 | 1.57 (0.83-2.96) | 0.16 | . | . | . |
| TK1 ---- tag |  | G/G | 9 | 15,52 | 1 | 2,94 | 56 | 14,85 | 29 | 10,74 | 0.23 (0.03-1.84) | 0.17 | 0.90 (0.43-1.88) | 0.78 | . | . | . |
| TK1 ---- tag | rs2854702 | G/G | 45 | 77,59 | 23 | 67,65 | 303 | 80,37 | 196 | 72,59 | 1.00 (.-.) | . | 1.20 (0.74-1.95) | 0.47 | 0.69 | 0.99 | 0.89 |
| TK1 ---- tag |  | G/A | 13 | 22,41 | 11 | 32,35 | 71 | 18,83 | 69 | 25,56 | 1.09 (0.47-2.52) | 0.84 | 1.53 (0.90-2.60) | 0.12 | . | . | . |
| TK1 ---- tag |  | A/A | 0 | 0,00 | 0 | 0,00 | 3 | 0,80 | 5 | 1,85 | 1.95 (0.71-5.34) | 0.20 | 2.33 (0.78-7.00) | 0.13 | . | . | . |
| TK1 ---- tag | rs9897765 | G/G | 36 | 62,07 | 16 | 47,06 | 199 | 52,79 | 141 | 52,22 | 1.00 (.-.) | . | 1.14 (0.64-2.05) | 0.65 | 0.36 | 0.99 | 0.89 |
| TK1 ---- tag |  | G/A | 18 | 31,03 | 16 | 47,06 | 145 | 38,46 | 110 | 40,74 | 1.17 (0.53-2.59) | 0.69 | 1.33 (0.73-2.43) | 0.35 | . | . | . |
| TK1 ---- tag |  | A/A | 4 | 6,90 | 2 | 5,88 | 33 | 8,75 | 19 | 7,04 | 0.43 (0.10-1.95) | 0.28 | 1.14 (0.54-2.40) | 0.74 | . | . | . |
| TYMP ---- NA | rs131815 | G/G | 24 | 41,38 | 21 | 61,76 | 206 | 54,64 | 146 | 54,07 | 1.00 (.-.) | . | 0.96 (0.56-1.64) | 0.87 | 0.47 | 0.83 | 0.20 |
| TYMP ---- NA |  | G/A | 30 | 51,72 | 11 | 32,35 | 137 | 36,34 | 108 | 40,00 | 0.70 (0.31-1.55) | 0.38 | 1.34 (0.78-2.31) | 0.29 | . | . | . |
| TYMP ---- NA |  | A/A | 4 | 6,90 | 2 | 5,88 | 34 | 9,02 | 16 | 5,93 | 1.69 (0.38-7.63) | 0.49 | 1.03 (0.51-2.08) | 0.94 | . | . | . |
| TYMP ---- tag | rs131816 | A/A | 38 | 65,52 | 21 | 61,76 | 230 | 61,01 | 172 | 63,70 | 1.00 (.-.) | . | 1.27 (0.76-2.15) | 0.36 | 0.77 | 0.59 | 0.14 |
| TYMP ---- tag |  | A/G | 18 | 31,03 | 11 | 32,35 | 123 | 32,63 | 83 | 30,74 | 0.80 (0.36-1.78) | 0.59 | 1.04 (0.60-1.80) | 0.88 | . | . | . |
| TYMP ---- tag |  | G/G | 2 | 3,45 | 2 | 5,88 | 24 | 6,37 | 15 | 5,56 | 1.57 (0.20-12.09) | 0.67 | 0.83 (0.39-1.75) | 0.62 | . | . | . |
| TYMP ---- NA | rs131817 | C/C | 15 | 25,86 | 13 | 38,24 | 120 | 31,83 | 92 | 34,07 | 1.00 (.-.) | . | 1.32 (0.65-2.68) | 0.45 | 0.81 | 0.93 | 0.42 |
| TYMP ---- NA |  | C/T | 28 | 48,28 | 14 | 41,18 | 187 | 49,60 | 132 | 48,89 | 1.37 (0.57-3.31) | 0.48 | 1.43 (0.71-2.88) | 0.31 | . | . | . |
| TYMP ---- NA |  | T/T | 15 | 25,86 | 7 | 20,59 | 70 | 18,57 | 46 | 17,04 | 1.07 (0.39-2.93) | 0.90 | 1.67 (0.80-3.49) | 0.18 | . | . | . |
| TYMP ---- NA | rs140521 | T/T | 36 | 62,07 | 16 | 47,06 | 203 | 53,85 | 137 | 50,74 | 1.00 (.-.) | . | 1.64 (0.89-3.04) | 0.11 | 0.31 | 0.74 | 0.20 |
| TYMP ---- NA |  | T/G | 21 | 36,21 | 14 | 41,18 | 144 | 38,20 | 110 | 40,74 | 1.64 (0.72-3.74) | 0.24 | 1.48 (0.80-2.76) | 0.21 | . | . | . |
| TYMP ---- NA |  | G/G | 1 | 1,72 | 4 | 11,76 | 30 | 7,96 | 23 | 8,52 | 1.37 (0.43-4.36) | 0.60 | 1.58 (0.76-3.28) | 0.22 | . | . | . |
| TYMP ---- NA | rs140522 | G/G | 29 | 50,00 | 14 | 41,18 | 175 | 46,42 | 134 | 49,63 | 1.00 (.-.) | . | 1.52 (0.84-2.75) | 0.16 | 0.12 | 0.82 | 0.20 |
| TYMP ---- NA |  | G/A | 25 | 43,10 | 17 | 50,00 | 164 | 43,50 | 111 | 41,11 | 1.04 (0.47-2.30) | 0.92 | 1.29 (0.71-2.34) | 0.41 | . | . | . |
| TYMP ---- NA |  | A/A | 4 | 6,90 | 3 | 8,82 | 38 | 10,08 | 25 | 9,26 | 3.02 (0.85-10.79) | 0.09 | 0.99 (0.48-2.01) | 0.97 | . | . | . |
| TYMP ---- NA | rs140524 | G/G | 38 | 65,52 | 21 | 61,76 | 251 | 66,58 | 180 | 66,67 | 1.00 (.-.) | . | 1.53 (0.92-2.53) | 0.10 | 0.10 | 0.93 | 0.42 |
| TYMP ---- NA |  | G/A | 19 | 32,76 | 13 | 38,24 | 116 | 30,77 | 84 | 31,11 | 1.73 (0.77-3.85) | 0.18 | 1.36 (0.79-2.34) | 0.27 | . | . | . |
| TYMP ---- NA |  | A/A | 1 | 1,72 | 0 | 0,00 | 10 | 2,65 | 6 | 2,22 | 0.52 (0.21-1.30) | 0.16 | 0.79 (0.29-2.21) | 0.66 | . | . | . |
| TYMS ---- candidate literature | rs1001761 | C/C | 17 | 29,31 | 12 | 35,29 | 110 | 29,18 | 88 | 32,59 | 1.00 (.-.) | . | 1.49 (0.74-3.02) | 0.26 | 0.82 | 0.59 | 0.09 |
| TYMS ---- candidate literature |  | C/T | 30 | 51,72 | 17 | 50,00 | 181 | 48,01 | 135 | 50,00 | 1.14 (0.50-2.61) | 0.76 | 1.21 (0.60-2.41) | 0.60 | . | . | . |
| TYMS ---- candidate literature |  | T/T | 11 | 18,97 | 5 | 14,71 | 86 | 22,81 | 47 | 17,41 | 0.67 (0.18-2.50) | 0.55 | 1.05 (0.51-2.18) | 0.89 | . | . | . |
| TYMS ---- candidate literature/tag | rs10502289 | T/T | 33 | 56,90 | 26 | 76,47 | 238 | 63,13 | 174 | 64,44 | 1.00 (.-.) | . | 1.00 (0.63-1.60) | 1.00 | 0.09 | 0.85 | 0.30 |
| TYMS ---- candidate literature/tag |  | T/A | 24 | 41,38 | 8 | 23,53 | 119 | 31,56 | 79 | 29,26 | 0.43 (0.17-1.06) | 0.07 | 0.92 (0.55-1.51) | 0.73 | . | . | . |
| TYMS ---- candidate literature/tag |  | A/A | 1 | 1,72 | 0 | 0,00 | 20 | 5,31 | 17 | 6,30 | 0.00 (0.00-6E280) | 0.98 | 0.83 (0.42-1.65) | 0.60 | . | . | . |
| TYMS ---- tag | rs15872 | C/C | 28 | 48,28 | 17 | 50,00 | 182 | 48,28 | 137 | 50,74 | 1.00 (.-.) | . | 1.21 (0.67-2.17) | 0.53 | 0.63 | 0.66 | 0.13 |
| TYMS ---- tag |  | C/T | 26 | 44,83 | 16 | 47,06 | 157 | 41,64 | 107 | 39,63 | 0.82 (0.39-1.74) | 0.61 | 0.96 (0.53-1.74) | 0.89 | . | . | . |
| TYMS ---- tag |  | T/T | 4 | 6,90 | 1 | 2,94 | 38 | 10,08 | 26 | 9,63 | 0.00 (0.00-I) | 0.98 | 0.94 (0.46-1.88) | 0.85 | . | . | . |
| TYMS ---- tag | rs2244500 | T/T | 17 | 29,31 | 12 | 35,29 | 110 | 29,18 | 88 | 32,59 | 1.00 (.-.) | . | 1.49 (0.74-3.02) | 0.26 | 0.81 | 0.59 | 0.09 |
| TYMS ---- tag |  | T/C | 30 | 51,72 | 17 | 50,00 | 180 | 47,75 | 135 | 50,00 | 1.14 (0.50-2.61) | 0.76 | 1.21 (0.60-2.42) | 0.59 | . | . | . |
| TYMS ---- tag |  | C/C | 11 | 18,97 | 5 | 14,71 | 87 | 23,08 | 47 | 17,41 | 0.67 (0.18-2.50) | 0.55 | 1.04 (0.50-2.17) | 0.91 | . | . | . |
| TYMS ---- tag | rs2741182 | G/G | 36 | 62,07 | 21 | 61,76 | 214 | 56,76 | 171 | 63,33 | 1.00 (.-.) | . | 1.46 (0.87-2.46) | 0.16 | 0.29 | 1.00 | 0.99 |
| TYMS ---- tag |  | G/C | 21 | 36,21 | 11 | 32,35 | 137 | 36,34 | 83 | 30,74 | 1.34 (0.61-2.96) | 0.46 | 1.24 (0.71-2.15) | 0.45 | . | . | . |
| TYMS ---- tag |  | C/C | 1 | 1,72 | 2 | 5,88 | 26 | 6,90 | 16 | 5,93 | 2.15 (0.27-17.16) | 0.47 | 1.56 (0.75-3.23) | 0.23 | . | . | . |
| TYMS ---- candidate literature | rs2847149 | G/G | 17 | 29,31 | 12 | 35,29 | 110 | 29,18 | 88 | 32,59 | 1.00 (.-.) | . | 1.49 (0.74-3.02) | 0.26 | 0.82 | 0.59 | 0.09 |
| TYMS ---- candidate literature |  | G/A | 30 | 51,72 | 17 | 50,00 | 181 | 48,01 | 135 | 50,00 | 1.14 (0.50-2.61) | 0.76 | 1.21 (0.60-2.41) | 0.60 | . | . | . |
| TYMS ---- candidate literature |  | A/A | 11 | 18,97 | 5 | 14,71 | 86 | 22,81 | 47 | 17,41 | 0.67 (0.18-2.50) | 0.55 | 1.05 (0.51-2.18) | 0.89 | . | . | . |
| TYMS ---- candidate literature | rs2853533 | G/G | 45 | 77,59 | 24 | 70,59 | 284 | 75,33 | 200 | 74,07 | 1.00 (.-.) | . | 1.24 (0.76-2.03) | 0.39 | 0.99 | 0.99 | 0.85 |
| TYMS ---- candidate literature |  | G/C | 13 | 22,41 | 10 | 29,41 | 83 | 22,02 | 64 | 23,70 | 0.94 (0.42-2.13) | 0.89 | 1.18 (0.69-2.03) | 0.54 | . | . | . |
| TYMS ---- candidate literature |  | C/C | 0 | 0,00 | 0 | 0,00 | 10 | 2,65 | 6 | 2,22 | 0.81 (0.29-2.21) | 0.68 | 1.00 (0.34-2.98) | 0.99 | . | . | . |
| TYMS ---- tag | rs495139 | C/C | 20 | 34,48 | 7 | 20,59 | 149 | 39,52 | 83 | 30,74 | 1.00 (.-.) | . | 1.53 (0.60-3.89) | 0.37 | 0.61 | 0.45 | 0.01 |
| TYMS ---- tag |  | C/G | 28 | 48,28 | 21 | 61,76 | 162 | 42,97 | 136 | 50,37 | 2.16 (0.80-5.88) | 0.13 | 2.25 (0.90-5.65) | 0.08 | . | . | . |
| TYMS ---- tag |  | G/G | 10 | 17,24 | 6 | 17,65 | 66 | 17,51 | 51 | 18,89 | 0.96 (0.25-3.67) | 0.95 | 2.10 (0.82-5.39) | 0.12 | . | . | . |
| TYMS ---- candidate literature | rs502396 | T/T | 19 | 32,76 | 12 | 35,29 | 109 | 28,91 | 90 | 33,33 | 1.00 (.-.) | . | 1.49 (0.74-3.01) | 0.27 | 0.72 | 0.84 | 0.24 |
| TYMS ---- candidate literature |  | T/C | 26 | 44,83 | 16 | 47,06 | 178 | 47,21 | 129 | 47,78 | 1.19 (0.51-2.74) | 0.69 | 1.28 (0.64-2.57) | 0.48 | . | . | . |
| TYMS ---- candidate literature |  | C/C | 13 | 22,41 | 6 | 17,65 | 90 | 23,87 | 51 | 18,89 | 0.93 (0.28-3.05) | 0.90 | 1.23 (0.60-2.54) | 0.58 | . | . | . |
| UMPH2 ---- tag | rs2291028 | A/A | 19 | 32,76 | 12 | 35,29 | 163 | 43,24 | 117 | 43,33 | 1.00 (.-.) | . | 1.38 (0.68-2.80) | 0.37 | 0.12 | 0.89 | 0.89 |
| UMPH2 ---- tag |  | A/G | 29 | 50,00 | 14 | 41,18 | 166 | 44,03 | 125 | 46,30 | 0.82 (0.34-2.01) | 0.67 | 1.32 (0.65-2.68) | 0.44 | . | . | . |
| UMPH2 ---- tag |  | G/G | 10 | 17,24 | 8 | 23,53 | 48 | 12,73 | 28 | 10,37 | 2.85 (1.04-7.82) | 0.04 | 1.30 (0.59-2.88) | 0.51 | . | . | . |
| UMPH2 ---- NA | rs4789143 | A/A | 41 | 70,69 | 27 | 79,41 | 284 | 75,33 | 220 | 81,48 | 1.00 (.-.) | . | 1.55 (0.96-2.48) | 0.07 | 0.03 | 0.99 | 0.97 |
| UMPH2 ---- NA |  | A/G | 15 | 25,86 | 6 | 17,65 | 88 | 23,34 | 48 | 17,78 | 1.89 (0.74-4.85) | 0.19 | 1.08 (0.62-1.86) | 0.79 | . | . | . |
| UMPH2 ---- NA |  | G/G | 2 | 3,45 | 1 | 2,94 | 5 | 1,33 | 2 | 0,74 | 4.54 (0.58-35.86) | 0.15 | 1.70 (0.39-7.41) | 0.48 | . | . | . |
| UMPH2 ---- NA | rs750844 | G/G | 26 | 44,83 | 13 | 38,24 | 190 | 50,40 | 143 | 52,96 | 1.00 (.-.) | . | 1.73 (0.88-3.37) | 0.11 | 0.08 | 0.99 | 0.97 |
| UMPH2 ---- NA |  | G/A | 25 | 43,10 | 17 | 50,00 | 159 | 42,18 | 105 | 38,89 | 1.34 (0.58-3.06) | 0.49 | 1.42 (0.71-2.81) | 0.32 | . | . | . |
| UMPH2 ---- NA |  | A/A | 7 | 12,07 | 4 | 11,76 | 28 | 7,43 | 22 | 8,15 | 3.85 (1.02-14.58) | 0.05 | 1.64 (0.74-3.66) | 0.22 | . | . | . |
| UMPK ---- tag | rs11582877 | C/C | 43 | 74,14 | 26 | 76,47 | 281 | 74,54 | 193 | 71,48 | 1.00 (.-.) | . | 1.11 (0.70-1.77) | 0.65 | 0.49 | 0.99 | 0.80 |
| UMPK ---- tag |  | C/T | 13 | 22,41 | 7 | 20,59 | 83 | 22,02 | 71 | 26,30 | 0.60 (0.22-1.60) | 0.31 | 1.12 (0.67-1.85) | 0.67 | . | . | . |
| UMPK ---- tag |  | T/T | 2 | 3,45 | 1 | 2,94 | 13 | 3,45 | 6 | 2,22 | 0.94 (0.12-7.07) | 0.95 | 0.86 (0.32-2.30) | 0.76 | . | . | . |
| UMPK ---- tag | rs2622903 | A/A | 30 | 51,72 | 16 | 47,06 | 188 | 49,87 | 128 | 47,41 | 1.00 (.-.) | . | 1.14 (0.63-2.06) | 0.67 | 0.53 | 0.96 | 0.80 |
| UMPK ---- tag |  | A/G | 22 | 37,93 | 15 | 44,12 | 150 | 39,79 | 119 | 44,07 | 1.01 (0.46-2.19) | 0.99 | 1.29 (0.71-2.35) | 0.40 | . | . | . |
| UMPK ---- tag |  | G/G | 6 | 10,34 | 3 | 8,82 | 39 | 10,34 | 23 | 8,52 | 0.43 (0.06-3.36) | 0.42 | 1.06 (0.51-2.22) | 0.88 | . | . | . |
| UMPK ---- tag | rs2820989 | C/C | 18 | 31,03 | 12 | 35,29 | 118 | 31,30 | 82 | 30,37 | 1.00 (.-.) | . | 0.95 (0.48-1.89) | 0.89 | 0.30 | 0.99 | 0.80 |
| UMPK ---- tag |  | C/G | 29 | 50,00 | 14 | 41,18 | 180 | 47,75 | 128 | 47,41 | 0.75 (0.32-1.76) | 0.51 | 0.96 (0.49-1.88) | 0.91 | . | . | . |
| UMPK ---- tag |  | G/G | 11 | 18,97 | 8 | 23,53 | 79 | 20,95 | 60 | 22,22 | 0.55 (0.18-1.67) | 0.29 | 0.96 (0.47-1.95) | 0.90 | . | . | . |
| UMPK ---- tag | rs6660321 | A/A | 44 | 75,86 | 26 | 76,47 | 289 | 76,66 | 200 | 74,07 | 1.00 (.-.) | . | 1.12 (0.71-1.79) | 0.62 | 0.50 | 0.99 | 0.92 |
| UMPK ---- tag |  | A/C | 12 | 20,69 | 7 | 20,59 | 81 | 21,49 | 64 | 23,70 | 0.60 (0.22-1.62) | 0.31 | 1.06 (0.64-1.77) | 0.82 | . | . | . |
| UMPK ---- tag |  | C/C | 2 | 3,45 | 1 | 2,94 | 7 | 1,86 | 6 | 2,22 | 0.94 (0.12-7.09) | 0.95 | 1.13 (0.42-3.05) | 0.81 | . | . | . |
| UMPK ---- tag | rs6690084 | T/T | 53 | 91,38 | 32 | 94,12 | 329 | 87,27 | 233 | 86,30 | 1.00 (.-.) | . | 1.11 (0.74-1.69) | 0.61 | 0.02 | 0.88 | 0.80 |
| UMPK ---- tag |  | T/C | 5 | 8,62 | 2 | 5,88 | 48 | 12,73 | 34 | 12,59 | 0.00 (0.00-4E242) | 0.97 | 1.19 (0.70-2.02) | 0.52 | . | . | . |
| UMPK ---- tag |  | C/C | 0 | 0,00 | 0 | 0,00 | 0 | 0,00 | 3 | 1,11 | 1.27 (0.30-5.31) | 0.74 | 1.41 (0.32-6.16) | 0.64 | . | . | . |
| UMPS ---- NA | rs1162 | A/A | 31 | 53,45 | 16 | 47,06 | 179 | 47,48 | 128 | 47,41 | 1.00 (.-.) | . | 1.04 (0.58-1.86) | 0.89 | 0.40 | 0.87 | 0.79 |
| UMPS ---- NA |  | A/G | 25 | 43,10 | 16 | 47,06 | 160 | 42,44 | 106 | 39,26 | 0.76 (0.34-1.68) | 0.50 | 1.06 (0.59-1.91) | 0.84 | . | . | . |
| UMPS ---- NA |  | G/G | 2 | 3,45 | 2 | 5,88 | 38 | 10,08 | 36 | 13,33 | 0.81 (0.18-3.71) | 0.79 | 1.33 (0.68-2.57) | 0.41 | . | . | . |
| UMPS ---- tag | rs13146 | C/C | 42 | 72,41 | 23 | 67,65 | 265 | 70,29 | 187 | 69,26 | 1.00 (.-.) | . | 1.09 (0.66-1.78) | 0.75 | 0.25 | 0.99 | 0.95 |
| UMPS ---- tag |  | C/T | 15 | 25,86 | 10 | 29,41 | 98 | 25,99 | 70 | 25,93 | 0.70 (0.31-1.58) | 0.39 | 0.99 (0.58-1.70) | 0.98 | . | . | . |
| UMPS ---- tag |  | T/T | 1 | 1,72 | 1 | 2,94 | 14 | 3,71 | 13 | 4,81 | 0.00 (0.00-2E233) | 0.97 | 2.00 (0.95-4.25) | 0.07 | . | . | . |
| UMPS ---- tag | rs16835902 | C/C | 18 | 31,03 | 17 | 50,00 | 121 | 32,10 | 88 | 32,59 | 1.00 (.-.) | . | 1.09 (0.60-1.98) | 0.78 | 0.80 | 0.99 | 0.95 |
| UMPS ---- tag |  | C/G | 27 | 46,55 | 13 | 38,24 | 179 | 47,48 | 141 | 52,22 | 0.77 (0.34-1.75) | 0.53 | 1.16 (0.65-2.06) | 0.62 | . | . | . |
| UMPS ---- tag |  | G/G | 13 | 22,41 | 4 | 11,76 | 77 | 20,42 | 41 | 15,19 | 0.93 (0.30-2.88) | 0.90 | 0.98 (0.52-1.87) | 0.95 | . | . | . |
| UMPS ---- tag | rs17282057 | T/T | 47 | 81,03 | 27 | 79,41 | 286 | 75,86 | 204 | 75,56 | 1.00 (.-.) | . | 1.29 (0.82-2.03) | 0.27 | 0.58 | 0.99 | 0.95 |
| UMPS ---- tag |  | T/C | 10 | 17,24 | 6 | 17,65 | 85 | 22,55 | 61 | 22,59 | 0.87 (0.30-2.54) | 0.80 | 1.13 (0.69-1.88) | 0.62 | . | . | . |
| UMPS ---- tag |  | C/C | 1 | 1,72 | 1 | 2,94 | 6 | 1,59 | 5 | 1,85 | 3.84 (0.50-29.28) | 0.20 | 1.60 (0.47-5.47) | 0.45 | . | . | . |
| UMPS ---- tag | rs606552 | A/A | 25 | 43,10 | 22 | 64,71 | 192 | 50,93 | 151 | 55,93 | 1.00 (.-.) | . | 1.14 (0.66-1.94) | 0.64 | 0.57 | 0.85 | 0.79 |
| UMPS ---- tag |  | A/G | 25 | 43,10 | 11 | 32,35 | 151 | 40,05 | 100 | 37,04 | 0.81 (0.37-1.79) | 0.61 | 1.10 (0.64-1.90) | 0.73 | . | . | . |
| UMPS ---- tag |  | G/G | 8 | 13,79 | 1 | 2,94 | 34 | 9,02 | 19 | 7,04 | 0.56 (0.07-4.39) | 0.58 | 1.04 (0.52-2.07) | 0.91 | . | . | . |
| UMPS ---- tag | rs694897 | C/C | 27 | 46,55 | 14 | 41,18 | 152 | 40,32 | 98 | 36,30 | 1.00 (.-.) | . | 1.55 (0.83-2.87) | 0.17 | 0.31 | 0.99 | 0.95 |
| UMPS ---- tag |  | C/G | 24 | 41,38 | 12 | 35,29 | 182 | 48,28 | 132 | 48,89 | 1.44 (0.60-3.41) | 0.41 | 1.51 (0.83-2.78) | 0.18 | . | . | . |
| UMPS ---- tag |  | G/G | 7 | 12,07 | 8 | 23,53 | 43 | 11,41 | 40 | 14,81 | 1.72 (0.63-4.66) | 0.29 | 1.59 (0.81-3.10) | 0.17 | . | . | . |
| UNG ---- NA | rs1059262 | T/T | 37 | 63,79 | 25 | 73,53 | 248 | 65,78 | 194 | 71,85 | 1.00 (.-.) | . | 1.02 (0.64-1.62) | 0.93 | 0.20 | 0.93 | 0.71 |
| UNG ---- NA |  | T/G | 19 | 32,76 | 8 | 23,53 | 113 | 29,97 | 71 | 26,30 | 0.42 (0.16-1.13) | 0.09 | 0.84 (0.51-1.40) | 0.51 | . | . | . |
| UNG ---- NA |  | G/G | 2 | 3,45 | 1 | 2,94 | 16 | 4,24 | 5 | 1,85 | 0.41 (0.05-3.19) | 0.40 | 0.84 (0.28-2.48) | 0.75 | . | . | . |
| UNG ---- tag | rs2160603 | T/T | 37 | 63,79 | 27 | 79,41 | 256 | 67,90 | 182 | 67,41 | 1.00 (.-.) | . | 0.97 (0.61-1.55) | 0.91 | 0.06 | 0.85 | 0.70 |
| UNG ---- tag |  | T/C | 18 | 31,03 | 6 | 17,65 | 104 | 27,59 | 78 | 28,89 | 0.50 (0.19-1.34) | 0.17 | 1.18 (0.70-1.96) | 0.54 | . | . | . |
| UNG ---- tag |  | C/C | 3 | 5,17 | 1 | 2,94 | 17 | 4,51 | 10 | 3,70 | 0.00 (0.00-2E238) | 0.97 | 0.91 (0.41-2.05) | 0.83 | . | . | . |
| UNG ---- tag | rs246079 | A/A | 14 | 24,14 | 10 | 29,41 | 112 | 29,71 | 90 | 33,33 | 1.00 (.-.) | . | 1.13 (0.56-2.28) | 0.73 | 0.36 | 0.85 | 0.70 |
| UNG ---- tag |  | A/G | 31 | 53,45 | 17 | 50,00 | 194 | 51,46 | 128 | 47,41 | 0.94 (0.41-2.16) | 0.89 | 1.02 (0.51-2.02) | 0.96 | . | . | . |
| UNG ---- tag |  | G/G | 13 | 22,41 | 7 | 20,59 | 71 | 18,83 | 52 | 19,26 | 0.59 (0.18-1.93) | 0.39 | 1.28 (0.62-2.67) | 0.50 | . | . | . |
| UNG ---- NA | rs246085 | T/T | 51 | 87,93 | 24 | 70,59 | 331 | 87,80 | 242 | 89,63 | 1.00 (.-.) | . | 1.37 (0.85-2.22) | 0.20 | 0.47 | 0.99 | 0.97 |
| UNG ---- NA |  | T/C | 7 | 12,07 | 10 | 29,41 | 45 | 11,94 | 28 | 10,37 | 1.55 (0.67-3.61) | 0.31 | 1.54 (0.83-2.84) | 0.17 | . | . | . |
| UNG ---- NA |  | C/C | 0 | 0,00 | 0 | 0,00 | 1 | 0,27 | 0 | 0,00 | 0.00 (0.00-1E252) | 0.97 | 0.00 (0.00-1E252) | 0.97 | . | . | . |
| UNG ---- NA | rs2569987 | A/A | 46 | 79,31 | 25 | 73,53 | 247 | 65,52 | 201 | 74,44 | 1.00 (.-.) | . | 1.71 (1.05-2.80) | 0.03 | 0.04 | 0.94 | 0.71 |
| UNG ---- NA |  | A/G | 9 | 15,52 | 9 | 26,47 | 113 | 29,97 | 62 | 22,96 | 3.99 (1.78-8.96) | 0.00 | 1.31 (0.76-2.26) | 0.34 | . | . | . |
| UNG ---- NA |  | G/G | 3 | 5,17 | 0 | 0,00 | 17 | 4,51 | 7 | 2,59 | 0.00 (0.00-5E228) | 0.97 | 1.46 (0.57-3.75) | 0.43 | . | . | . |
| UNG ---- tag | rs3219243 | T/T | 33 | 56,90 | 23 | 67,65 | 247 | 65,52 | 176 | 65,19 | 1.00 (.-.) | . | 1.23 (0.76-1.98) | 0.40 | 0.82 | 0.99 | 0.97 |
| UNG ---- tag |  | T/C | 23 | 39,66 | 10 | 29,41 | 118 | 31,30 | 79 | 29,26 | 0.80 (0.31-2.02) | 0.63 | 1.01 (0.61-1.68) | 0.97 | . | . | . |
| UNG ---- tag |  | C/C | 2 | 3,45 | 1 | 2,94 | 12 | 3,18 | 15 | 5,56 | 1.11 (0.14-9.03) | 0.92 | 1.97 (0.96-4.04) | 0.06 | . | . | . |

|  |  |  | **adchem_5fu = 0** | | | | **adchem_5fu = 1** | | | | **adchem_5fu = 0** |  | **adchem_5fu = 1** |  |  |  |
| --- | --- | --- | --- | --- | --- | --- | --- | --- | --- | --- | --- | --- | --- | --- | --- | --- |
|  |  |  | **Ctrl** | | **Cases** | | **Ctrl** | | **Cases** | |  |  |  |  |  |  |
| **Gene** | **SNP** | **Genotype** | **N** | **%** | **N** | **%** | **N** | **%** | **N** | **%** | **HR (95%-CI)** | **p** | **HR (95%-CI)** | **p** | **FDR_pInt** | **FDR(byGene)_pInt** |
| AARS - tag | rs2070203 | 0 | 16 | 27,59 | 12 | 35,29 | 115 | 30,50 | 73 | 27,04 | 1.00 (.-.) | . | 0.80 (0.43-1.49) | 0.48 | 0.99 | 0.89 |
| AARS - tag |  | 1 | 24 | 41,38 | 17 | 50,00 | 191 | 50,66 | 137 | 50,74 | 0.70 (0.41-1.18) | 0.18 | 0.90 (0.50-1.64) | 0.74 | . | . |
| AARS - tag |  | 2 | 18 | 31,03 | 5 | 14,71 | 71 | 18,83 | 60 | 22,22 | 0.48 (0.17-1.39) | 0.18 | 1.02 (0.55-1.90) | 0.96 | . | . |
| AARS - tag | rs34087264 | 0 | 22 | 37,93 | 6 | 17,65 | 97 | 25,73 | 84 | 31,11 | 1.00 (.-.) | . | 2.04 (0.99-4.24) | 0.06 | 0.64 | 0.11 |
| AARS - tag |  | 1 | 22 | 37,93 | 18 | 52,94 | 189 | 50,13 | 130 | 48,15 | 1.41 (0.83-2.40) | 0.21 | 1.73 (0.85-3.52) | 0.13 | . | . |
| AARS - tag |  | 2 | 14 | 24,14 | 10 | 29,41 | 91 | 24,14 | 56 | 20,74 | 1.99 (0.69-5.76) | 0.21 | 1.46 (0.70-3.06) | 0.32 | . | . |
| ABCC4 - tag | rs10508023 | 0 | 48 | 82,76 | 26 | 76,47 | 298 | 79,05 | 219 | 81,11 | 1.00 (.-.) | . | 1.38 (0.87-2.18) | 0.17 | 0.93 | 0.81 |
| ABCC4 - tag |  | 1 | 9 | 15,52 | 8 | 23,53 | 75 | 19,89 | 45 | 16,67 | 1.56 (0.71-3.45) | 0.27 | 1.25 (0.75-2.08) | 0.39 | . | . |
| ABCC4 - tag |  | 2 | 1 | 1,72 | 0 | 0,00 | 4 | 1,06 | 6 | 2,22 | 2.44 (0.50-11.92) | 0.27 | 1.13 (0.57-2.27) | 0.72 | . | . |
| ABCC4 - tag | rs1059751 | 0 | 21 | 36,21 | 7 | 20,59 | 88 | 23,34 | 71 | 26,30 | 1.00 (.-.) | . | 1.31 (0.65-2.64) | 0.46 | 0.99 | 0.95 |
| ABCC4 - tag |  | 1 | 29 | 50,00 | 24 | 70,59 | 211 | 55,97 | 141 | 52,22 | 1.13 (0.57-2.25) | 0.73 | 1.36 (0.69-2.69) | 0.37 | . | . |
| ABCC4 - tag |  | 2 | 8 | 13,79 | 3 | 8,82 | 78 | 20,69 | 58 | 21,48 | 1.28 (0.32-5.08) | 0.73 | 1.42 (0.70-2.88) | 0.33 | . | . |
| ABCC4 - tag | rs11568643 | 0 | 50 | 86,21 | 26 | 76,47 | 320 | 84,88 | 222 | 82,22 | 1.00 (.-.) | . | 1.31 (0.83-2.06) | 0.24 | 0.66 | 0.60 |
| ABCC4 - tag |  | 1 | 8 | 13,79 | 7 | 20,59 | 57 | 15,12 | 46 | 17,04 | 1.39 (0.75-2.59) | 0.30 | 1.54 (0.91-2.62) | 0.11 | . | . |
| ABCC4 - tag |  | 2 | 0 | 0,00 | 1 | 2,94 | 0 | 0,00 | 2 | 0,74 | 1.94 (0.56-6.71) | 0.30 | 1.81 (0.85-3.85) | 0.12 | . | . |
| ABCC4 - NA | rs11568658 | 0 | 55 | 94,83 | 32 | 94,12 | 356 | 94,43 | 258 | 95,56 | 1.00 (.-.) | . | 1.30 (0.85-1.99) | 0.23 | 0.99 | 0.95 |
| ABCC4 - NA |  | 1 | 3 | 5,17 | 2 | 5,88 | 21 | 5,57 | 12 | 4,44 | 2.64 (0.61-11.37) | 0.19 | 1.27 (0.58-2.75) | 0.55 | . | . |
| ABCC4 - NA |  | 2 | 0 | 0,00 | 0 | 0,00 | 0 | 0,00 | 0 | 0,00 | 6.97 (0.38-129.3) | 0.19 | 1.24 (0.31-4.97) | 0.76 | . | . |
| ABCC4 - tag | rs12864049 | 0 | 38 | 65,52 | 26 | 76,47 | 292 | 77,45 | 200 | 74,07 | 1.00 (.-.) | . | 1.21 (0.75-1.95) | 0.44 | 0.99 | 0.98 |
| ABCC4 - tag |  | 1 | 18 | 31,03 | 7 | 20,59 | 78 | 20,69 | 66 | 24,44 | 0.96 (0.43-2.14) | 0.92 | 1.27 (0.77-2.12) | 0.35 | . | . |
| ABCC4 - tag |  | 2 | 2 | 3,45 | 1 | 2,94 | 7 | 1,86 | 4 | 1,48 | 0.92 (0.18-4.60) | 0.92 | 1.35 (0.69-2.62) | 0.38 | . | . |
| ABCC4 - tag | rs1628382 | 0 | 39 | 67,24 | 22 | 64,71 | 235 | 62,33 | 163 | 60,37 | 1.00 (.-.) | . | 1.29 (0.78-2.14) | 0.32 | 0.99 | 0.98 |
| ABCC4 - tag |  | 1 | 17 | 29,31 | 11 | 32,35 | 124 | 32,89 | 96 | 35,56 | 1.24 (0.62-2.49) | 0.55 | 1.42 (0.85-2.36) | 0.18 | . | . |
| ABCC4 - tag |  | 2 | 2 | 3,45 | 1 | 2,94 | 18 | 4,77 | 11 | 4,07 | 1.54 (0.38-6.20) | 0.55 | 1.55 (0.85-2.85) | 0.16 | . | . |
| ABCC4 - tag | rs1678354 | 0 | 18 | 31,03 | 14 | 41,18 | 169 | 44,83 | 110 | 40,74 | 1.00 (.-.) | . | 1.36 (0.70-2.64) | 0.37 | 0.99 | 0.98 |
| ABCC4 - tag |  | 1 | 34 | 58,62 | 17 | 50,00 | 163 | 43,24 | 129 | 47,78 | 1.24 (0.61-2.52) | 0.55 | 1.47 (0.77-2.83) | 0.24 | . | . |
| ABCC4 - tag |  | 2 | 6 | 10,34 | 3 | 8,82 | 45 | 11,94 | 31 | 11,48 | 1.55 (0.38-6.33) | 0.55 | 1.60 (0.80-3.21) | 0.18 | . | . |
| ABCC4 - tag | rs1678383 | 0 | 47 | 81,03 | 28 | 82,35 | 308 | 81,70 | 223 | 82,59 | 1.00 (.-.) | . | 1.16 (0.73-1.84) | 0.52 | 0.90 | 0.72 |
| ABCC4 - tag |  | 1 | 10 | 17,24 | 6 | 17,65 | 63 | 16,71 | 45 | 16,67 | 0.73 (0.27-1.94) | 0.52 | 1.15 (0.69-1.93) | 0.59 | . | . |
| ABCC4 - tag |  | 2 | 1 | 1,72 | 0 | 0,00 | 6 | 1,59 | 2 | 0,74 | 0.53 (0.07-3.76) | 0.52 | 1.14 (0.55-2.36) | 0.72 | . | . |
| ABCC4 - tag | rs1678395 | 0 | 48 | 82,76 | 29 | 85,29 | 317 | 84,08 | 230 | 85,19 | 1.00 (.-.) | . | 1.29 (0.83-2.00) | 0.25 | 0.88 | 0.71 |
| ABCC4 - tag |  | 1 | 10 | 17,24 | 5 | 14,71 | 56 | 14,85 | 39 | 14,44 | 0.75 (0.22-2.50) | 0.64 | 0.93 (0.56-1.57) | 0.80 | . | . |
| ABCC4 - tag |  | 2 | 0 | 0,00 | 0 | 0,00 | 4 | 1,06 | 1 | 0,37 | 0.56 (0.05-6.24) | 0.64 | 0.68 (0.31-1.46) | 0.32 | . | . |
| ABCC4 - tag | rs1678405 | 0 | 26 | 44,83 | 19 | 55,88 | 167 | 44,30 | 121 | 44,81 | 1.00 (.-.) | . | 1.34 (0.79-2.28) | 0.28 | 0.86 | 0.71 |
| ABCC4 - tag |  | 1 | 29 | 50,00 | 13 | 38,24 | 179 | 47,48 | 118 | 43,70 | 1.34 (0.69-2.59) | 0.39 | 1.44 (0.86-2.42) | 0.17 | . | . |
| ABCC4 - tag |  | 2 | 3 | 5,17 | 2 | 5,88 | 31 | 8,22 | 31 | 11,48 | 1.79 (0.47-6.73) | 0.39 | 1.55 (0.87-2.77) | 0.14 | . | . |
| ABCC4 - tag | rs17189540 | 0 | 52 | 89,66 | 27 | 79,41 | 335 | 88,86 | 228 | 84,44 | 1.00 (.-.) | . | 1.23 (0.78-1.94) | 0.36 | 0.85 | 0.70 |
| ABCC4 - tag |  | 1 | 6 | 10,34 | 6 | 17,65 | 42 | 11,14 | 42 | 15,56 | 1.27 (0.50-3.24) | 0.62 | 1.78 (1.03-3.06) | 0.04 | . | . |
| ABCC4 - tag |  | 2 | 0 | 0,00 | 1 | 2,94 | 0 | 0,00 | 0 | 0,00 | 1.60 (0.25-10.50) | 0.62 | 2.55 (1.15-5.68) | 0.02 | . | . |
| ABCC4 - tag | rs17235152 | 0 | 39 | 67,24 | 26 | 76,47 | 269 | 71,35 | 204 | 75,56 | 1.00 (.-.) | . | 1.20 (0.74-1.94) | 0.47 | 0.99 | 0.98 |
| ABCC4 - tag |  | 1 | 17 | 29,31 | 8 | 23,53 | 98 | 25,99 | 63 | 23,33 | 0.81 (0.36-1.85) | 0.62 | 1.06 (0.62-1.79) | 0.84 | . | . |
| ABCC4 - tag |  | 2 | 2 | 3,45 | 0 | 0,00 | 10 | 2,65 | 3 | 1,11 | 0.66 (0.13-3.41) | 0.62 | 0.94 (0.46-1.88) | 0.85 | . | . |
| ABCC4 - tag | rs17268122 | 0 | 24 | 41,38 | 22 | 64,71 | 236 | 62,60 | 184 | 68,15 | 1.00 (.-.) | . | 0.86 (0.51-1.43) | 0.56 | 1.00 | 0.99 |
| ABCC4 - tag |  | 1 | 29 | 50,00 | 11 | 32,35 | 123 | 32,63 | 72 | 26,67 | 0.48 (0.24-0.96) | 0.04 | 0.80 (0.47-1.36) | 0.41 | . | . |
| ABCC4 - tag |  | 2 | 5 | 8,62 | 1 | 2,94 | 18 | 4,77 | 14 | 5,19 | 0.23 (0.06-0.91) | 0.04 | 0.75 (0.40-1.40) | 0.36 | . | . |
| ABCC4 - tag | rs17268170 | 0 | 47 | 81,03 | 24 | 70,59 | 314 | 83,29 | 216 | 80,00 | 1.00 (.-.) | . | 1.34 (0.84-2.16) | 0.22 | 0.99 | 0.98 |
| ABCC4 - tag |  | 1 | 11 | 18,97 | 6 | 17,65 | 62 | 16,45 | 52 | 19,26 | 1.41 (0.79-2.53) | 0.25 | 1.56 (0.92-2.64) | 0.10 | . | . |
| ABCC4 - tag |  | 2 | 0 | 0,00 | 4 | 11,76 | 1 | 0,27 | 2 | 0,74 | 2.00 (0.62-6.42) | 0.25 | 1.81 (0.88-3.71) | 0.11 | . | . |
| ABCC4 - tag | rs1729764 | 0 | 43 | 74,14 | 28 | 82,35 | 287 | 76,13 | 218 | 80,74 | 1.00 (.-.) | . | 1.20 (0.78-1.84) | 0.41 | 0.85 | 0.70 |
| ABCC4 - tag |  | 1 | 14 | 24,14 | 4 | 11,76 | 86 | 22,81 | 47 | 17,41 | 0.52 (0.13-2.11) | 0.36 | 1.03 (0.64-1.69) | 0.89 | . | . |
| ABCC4 - tag |  | 2 | 1 | 1,72 | 2 | 5,88 | 4 | 1,06 | 5 | 1,85 | 0.27 (0.02-4.44) | 0.36 | 0.89 (0.45-1.77) | 0.75 | . | . |
| ABCC4 - tag | rs1729767 | 0 | 28 | 48,28 | 15 | 44,12 | 202 | 53,58 | 137 | 50,74 | 1.00 (.-.) | . | 1.48 (0.83-2.65) | 0.19 | 0.95 | 0.91 |
| ABCC4 - tag |  | 1 | 25 | 43,10 | 17 | 50,00 | 153 | 40,58 | 119 | 44,07 | 1.39 (0.71-2.70) | 0.34 | 1.48 (0.83-2.65) | 0.18 | . | . |
| ABCC4 - tag |  | 2 | 5 | 8,62 | 2 | 5,88 | 22 | 5,84 | 14 | 5,19 | 1.92 (0.51-7.27) | 0.34 | 1.48 (0.77-2.84) | 0.23 | . | . |
| ABCC4 - tag | rs17300935 | 0 | 40 | 68,97 | 21 | 61,76 | 278 | 73,74 | 207 | 76,67 | 1.00 (.-.) | . | 1.26 (0.76-2.09) | 0.37 | 0.99 | 0.95 |
| ABCC4 - tag |  | 1 | 16 | 27,59 | 13 | 38,24 | 91 | 24,14 | 61 | 22,59 | 0.99 (0.46-2.14) | 0.97 | 1.16 (0.67-2.00) | 0.59 | . | . |
| ABCC4 - tag |  | 2 | 2 | 3,45 | 0 | 0,00 | 8 | 2,12 | 2 | 0,74 | 0.97 (0.21-4.57) | 0.97 | 1.07 (0.52-2.18) | 0.85 | . | . |
| ABCC4 - tag | rs1750190 | 0 | 19 | 32,76 | 7 | 20,59 | 101 | 26,79 | 62 | 22,96 | 1.00 (.-.) | . | 1.35 (0.67-2.69) | 0.40 | 0.83 | 0.70 |
| ABCC4 - tag |  | 1 | 27 | 46,55 | 19 | 55,88 | 186 | 49,34 | 138 | 51,11 | 1.23 (0.74-2.04) | 0.42 | 1.52 (0.78-2.96) | 0.22 | . | . |
| ABCC4 - tag |  | 2 | 12 | 20,69 | 8 | 23,53 | 90 | 23,87 | 70 | 25,93 | 1.52 (0.55-4.18) | 0.42 | 1.71 (0.86-3.41) | 0.13 | . | . |
| ABCC4 - tag | rs1750996 | 0 | 33 | 56,90 | 21 | 61,76 | 247 | 65,52 | 178 | 65,93 | 1.00 (.-.) | . | 1.22 (0.75-1.99) | 0.42 | 0.92 | 0.72 |
| ABCC4 - tag |  | 1 | 22 | 37,93 | 11 | 32,35 | 122 | 32,36 | 83 | 30,74 | 0.92 (0.43-1.99) | 0.84 | 1.19 (0.72-1.97) | 0.50 | . | . |
| ABCC4 - tag |  | 2 | 3 | 5,17 | 2 | 5,88 | 8 | 2,12 | 9 | 3,33 | 0.85 (0.18-3.96) | 0.84 | 1.16 (0.62-2.17) | 0.64 | . | . |
| ABCC4 - tag | rs1751025 | 0 | 32 | 55,17 | 13 | 38,24 | 181 | 48,01 | 120 | 44,44 | 1.00 (.-.) | . | 1.51 (0.86-2.68) | 0.16 | 0.66 | 0.60 |
| ABCC4 - tag |  | 1 | 21 | 36,21 | 15 | 44,12 | 166 | 44,03 | 114 | 42,22 | 1.73 (1.04-2.87) | 0.03 | 1.84 (1.05-3.22) | 0.03 | . | . |
| ABCC4 - tag |  | 2 | 5 | 8,62 | 6 | 17,65 | 30 | 7,96 | 36 | 13,33 | 2.99 (1.08-8.23) | 0.03 | 2.24 (1.22-4.12) | 0.01 | . | . |
| ABCC4 - tag | rs1751051 | 0 | 25 | 43,10 | 15 | 44,12 | 151 | 40,05 | 121 | 44,81 | 1.00 (.-.) | . | 1.08 (0.62-1.88) | 0.79 | 0.99 | 0.98 |
| ABCC4 - tag |  | 1 | 23 | 39,66 | 15 | 44,12 | 177 | 46,95 | 118 | 43,70 | 0.78 (0.44-1.37) | 0.39 | 1.04 (0.60-1.78) | 0.90 | . | . |
| ABCC4 - tag |  | 2 | 10 | 17,24 | 4 | 11,76 | 49 | 13,00 | 31 | 11,48 | 0.60 (0.19-1.89) | 0.39 | 0.99 (0.54-1.82) | 0.99 | . | . |
| ABCC4 - tag | rs1764416 | 0 | 50 | 86,21 | 34 | 100,00 | 320 | 84,88 | 237 | 87,78 | 1.00 (.-.) | . | 1.23 (0.81-1.86) | 0.34 | 0.90 | 0.72 |
| ABCC4 - tag |  | 1 | 8 | 13,79 | 0 | 0,00 | 56 | 14,85 | 33 | 12,22 | 0.00 (0.00-1E286) | 0.97 | 0.94 (0.54-1.65) | 0.83 | . | . |
| ABCC4 - tag |  | 2 | 0 | 0,00 | 0 | 0,00 | 1 | 0,27 | 0 | 0,00 | 0.00 (0.00-I) | 0.97 | 0.72 (0.29-1.78) | 0.48 | . | . |
| ABCC4 - tag | rs2274401 | 0 | 35 | 60,34 | 23 | 67,65 | 217 | 57,56 | 184 | 68,15 | 1.00 (.-.) | . | 1.13 (0.72-1.78) | 0.59 | 0.87 | 0.71 |
| ABCC4 - tag |  | 1 | 21 | 36,21 | 11 | 32,35 | 139 | 36,87 | 76 | 28,15 | 0.59 (0.26-1.33) | 0.20 | 0.97 (0.61-1.56) | 0.91 | . | . |
| ABCC4 - tag |  | 2 | 2 | 3,45 | 0 | 0,00 | 21 | 5,57 | 10 | 3,70 | 0.35 (0.07-1.76) | 0.20 | 0.84 (0.46-1.52) | 0.56 | . | . |
| ABCC4 - tag | rs2892716 | 0 | 26 | 44,83 | 17 | 50,00 | 124 | 32,89 | 107 | 39,63 | 1.00 (.-.) | . | 1.55 (0.91-2.64) | 0.11 | 0.99 | 0.98 |
| ABCC4 - tag |  | 1 | 28 | 48,28 | 17 | 50,00 | 185 | 49,07 | 131 | 48,52 | 1.33 (0.70-2.55) | 0.39 | 1.36 (0.81-2.28) | 0.24 | . | . |
| ABCC4 - tag |  | 2 | 4 | 6,90 | 0 | 0,00 | 68 | 18,04 | 32 | 11,85 | 1.78 (0.49-6.51) | 0.39 | 1.19 (0.67-2.11) | 0.55 | . | . |
| ABCC4 - tag | rs3782964 | 0 | 43 | 74,14 | 24 | 70,59 | 242 | 64,19 | 191 | 70,74 | 1.00 (.-.) | . | 1.36 (0.84-2.20) | 0.21 | 0.85 | 0.70 |
| ABCC4 - tag |  | 1 | 14 | 24,14 | 8 | 23,53 | 126 | 33,42 | 68 | 25,19 | 1.22 (0.59-2.53) | 0.59 | 1.22 (0.74-2.03) | 0.44 | . | . |
| ABCC4 - tag |  | 2 | 1 | 1,72 | 2 | 5,88 | 9 | 2,39 | 11 | 4,07 | 1.50 (0.35-6.40) | 0.59 | 1.10 (0.57-2.12) | 0.79 | . | . |
| ABCC4 - tag | rs3818494 | 0 | 33 | 56,90 | 18 | 52,94 | 170 | 45,09 | 111 | 41,11 | 1.00 (.-.) | . | 1.24 (0.73-2.09) | 0.43 | 0.59 | 0.60 |
| ABCC4 - tag |  | 1 | 20 | 34,48 | 14 | 41,18 | 166 | 44,03 | 120 | 44,44 | 1.26 (0.68-2.33) | 0.46 | 1.44 (0.87-2.40) | 0.16 | . | . |
| ABCC4 - tag |  | 2 | 5 | 8,62 | 2 | 5,88 | 41 | 10,88 | 39 | 14,44 | 1.59 (0.46-5.44) | 0.46 | 1.68 (0.96-2.94) | 0.07 | . | . |
| ABCC4 - tag | rs3864997 | 0 | 21 | 36,21 | 11 | 32,35 | 92 | 24,40 | 63 | 23,33 | 1.00 (.-.) | . | 1.86 (0.96-3.58) | 0.06 | 0.85 | 0.70 |
| ABCC4 - tag |  | 1 | 30 | 51,72 | 17 | 50,00 | 201 | 53,32 | 142 | 52,59 | 1.68 (0.97-2.91) | 0.07 | 1.82 (0.97-3.41) | 0.06 | . | . |
| ABCC4 - tag |  | 2 | 7 | 12,07 | 6 | 17,65 | 84 | 22,28 | 65 | 24,07 | 2.82 (0.93-8.49) | 0.07 | 1.79 (0.93-3.43) | 0.08 | . | . |
| ABCC4 - tag | rs4148421 | 0 | 13 | 22,41 | 6 | 17,65 | 118 | 31,30 | 78 | 28,89 | 1.00 (.-.) | . | 1.34 (0.60-2.99) | 0.48 | 0.99 | 0.98 |
| ABCC4 - tag |  | 1 | 36 | 62,07 | 18 | 52,94 | 189 | 50,13 | 142 | 52,59 | 1.09 (0.60-1.98) | 0.77 | 1.37 (0.63-3.01) | 0.43 | . | . |
| ABCC4 - tag |  | 2 | 9 | 15,52 | 10 | 29,41 | 70 | 18,57 | 50 | 18,52 | 1.19 (0.36-3.93) | 0.77 | 1.41 (0.63-3.18) | 0.41 | . | . |
| ABCC4 - tag | rs4148446 | 0 | 24 | 41,38 | 14 | 41,18 | 114 | 30,24 | 94 | 34,81 | 1.00 (.-.) | . | 1.50 (0.83-2.72) | 0.18 | 0.99 | 0.98 |
| ABCC4 - tag |  | 1 | 28 | 48,28 | 18 | 52,94 | 186 | 49,34 | 130 | 48,15 | 1.23 (0.64-2.35) | 0.54 | 1.38 (0.78-2.45) | 0.27 | . | . |
| ABCC4 - tag |  | 2 | 6 | 10,34 | 2 | 5,88 | 77 | 20,42 | 46 | 17,04 | 1.51 (0.41-5.51) | 0.54 | 1.27 (0.69-2.34) | 0.45 | . | . |
| ABCC4 - tag | rs4148455 | 0 | 43 | 74,14 | 27 | 79,41 | 285 | 75,60 | 202 | 74,81 | 1.00 (.-.) | . | 1.31 (0.81-2.11) | 0.27 | 0.99 | 0.95 |
| ABCC4 - tag |  | 1 | 14 | 24,14 | 6 | 17,65 | 83 | 22,02 | 66 | 24,44 | 1.18 (0.53-2.59) | 0.69 | 1.27 (0.75-2.12) | 0.37 | . | . |
| ABCC4 - tag |  | 2 | 1 | 1,72 | 1 | 2,94 | 9 | 2,39 | 2 | 0,74 | 1.38 (0.28-6.72) | 0.69 | 1.23 (0.62-2.42) | 0.56 | . | . |
| ABCC4 - tag | rs4148540 | 0 | 50 | 86,21 | 30 | 88,24 | 331 | 87,80 | 230 | 85,19 | 1.00 (.-.) | . | 1.23 (0.80-1.90) | 0.35 | 0.99 | 0.95 |
| ABCC4 - tag |  | 1 | 8 | 13,79 | 4 | 11,76 | 44 | 11,67 | 37 | 13,70 | 0.89 (0.26-2.97) | 0.84 | 1.20 (0.72-1.99) | 0.48 | . | . |
| ABCC4 - tag |  | 2 | 0 | 0,00 | 0 | 0,00 | 2 | 0,53 | 3 | 1,11 | 0.78 (0.07-8.84) | 0.84 | 1.17 (0.56-2.45) | 0.67 | . | . |
| ABCC4 - tag | rs4148542 | 0 | 12 | 20,69 | 3 | 8,82 | 105 | 27,85 | 68 | 25,19 | 1.00 (.-.) | . | 1.61 (0.62-4.19) | 0.33 | 0.99 | 0.98 |
| ABCC4 - tag |  | 1 | 27 | 46,55 | 24 | 70,59 | 198 | 52,52 | 141 | 52,22 | 1.25 (0.62-2.50) | 0.53 | 1.61 (0.63-4.14) | 0.32 | . | . |
| ABCC4 - tag |  | 2 | 19 | 32,76 | 7 | 20,59 | 74 | 19,63 | 61 | 22,59 | 1.56 (0.39-6.25) | 0.53 | 1.62 (0.62-4.23) | 0.33 | . | . |
| ABCC4 - tag | rs4148544 | 0 | 23 | 39,66 | 9 | 26,47 | 157 | 41,64 | 119 | 44,07 | 1.00 (.-.) | . | 1.54 (0.76-3.09) | 0.23 | 0.99 | 0.98 |
| ABCC4 - tag |  | 1 | 25 | 43,10 | 21 | 61,76 | 185 | 49,07 | 114 | 42,22 | 1.25 (0.69-2.25) | 0.46 | 1.50 (0.75-3.01) | 0.25 | . | . |
| ABCC4 - tag |  | 2 | 10 | 17,24 | 4 | 11,76 | 35 | 9,28 | 37 | 13,70 | 1.56 (0.48-5.08) | 0.46 | 1.47 (0.70-3.08) | 0.30 | . | . |
| ABCC4 - tag | rs4283094 | 0 | 9 | 15,52 | 6 | 17,65 | 102 | 27,06 | 61 | 22,59 | 1.00 (.-.) | . | 0.70 (0.32-1.50) | 0.36 | 0.99 | 0.98 |
| ABCC4 - tag |  | 1 | 32 | 55,17 | 17 | 50,00 | 196 | 51,99 | 145 | 53,70 | 0.68 (0.40-1.16) | 0.16 | 0.77 (0.37-1.63) | 0.50 | . | . |
| ABCC4 - tag |  | 2 | 17 | 29,31 | 11 | 32,35 | 79 | 20,95 | 64 | 23,70 | 0.46 (0.16-1.35) | 0.16 | 0.86 (0.40-1.85) | 0.70 | . | . |
| ABCC4 - tag | rs4636781 | 0 | 45 | 77,59 | 27 | 79,41 | 269 | 71,35 | 187 | 69,26 | 1.00 (.-.) | . | 1.28 (0.79-2.07) | 0.31 | 0.83 | 0.70 |
| ABCC4 - tag |  | 1 | 13 | 22,41 | 6 | 17,65 | 97 | 25,73 | 75 | 27,78 | 1.32 (0.60-2.88) | 0.49 | 1.48 (0.89-2.44) | 0.13 | . | . |
| ABCC4 - tag |  | 2 | 0 | 0,00 | 1 | 2,94 | 11 | 2,92 | 8 | 2,96 | 1.73 (0.36-8.30) | 0.49 | 1.70 (0.90-3.20) | 0.10 | . | . |
| ABCC4 - tag | rs4771910 | 0 | 30 | 51,72 | 16 | 47,06 | 178 | 47,21 | 129 | 47,78 | 1.00 (.-.) | . | 1.46 (0.81-2.61) | 0.21 | 0.83 | 0.70 |
| ABCC4 - tag |  | 1 | 25 | 43,10 | 15 | 44,12 | 166 | 44,03 | 115 | 42,59 | 1.13 (0.62-2.06) | 0.68 | 1.29 (0.72-2.29) | 0.39 | . | . |
| ABCC4 - tag |  | 2 | 3 | 5,17 | 3 | 8,82 | 33 | 8,75 | 26 | 9,63 | 1.28 (0.39-4.25) | 0.68 | 1.13 (0.60-2.14) | 0.70 | . | . |
| ABCC4 - tag | rs4773850 | 0 | 26 | 44,83 | 18 | 52,94 | 165 | 43,77 | 142 | 52,59 | 1.00 (.-.) | . | 1.53 (0.90-2.60) | 0.11 | 0.59 | 0.60 |
| ABCC4 - tag |  | 1 | 29 | 50,00 | 12 | 35,29 | 167 | 44,30 | 99 | 36,67 | 1.11 (0.63-1.94) | 0.72 | 1.20 (0.71-2.01) | 0.50 | . | . |
| ABCC4 - tag |  | 2 | 3 | 5,17 | 4 | 11,76 | 45 | 11,94 | 29 | 10,74 | 1.22 (0.40-3.76) | 0.72 | 0.93 (0.52-1.68) | 0.82 | . | . |
| ABCC4 - tag | rs7981095 | 0 | 39 | 67,24 | 20 | 58,82 | 248 | 65,78 | 171 | 63,33 | 1.00 (.-.) | . | 1.28 (0.76-2.14) | 0.35 | 0.99 | 0.95 |
| ABCC4 - tag |  | 1 | 18 | 31,03 | 10 | 29,41 | 119 | 31,56 | 88 | 32,59 | 1.10 (0.54-2.24) | 0.79 | 1.31 (0.77-2.22) | 0.32 | . | . |
| ABCC4 - tag |  | 2 | 1 | 1,72 | 4 | 11,76 | 10 | 2,65 | 11 | 4,07 | 1.21 (0.29-5.03) | 0.79 | 1.34 (0.71-2.55) | 0.37 | . | . |
| ABCC4 - tag | rs8001444 | 0 | 18 | 31,03 | 12 | 35,29 | 130 | 34,48 | 106 | 39,26 | 1.00 (.-.) | . | 1.18 (0.61-2.26) | 0.62 | 0.93 | 0.81 |
| ABCC4 - tag |  | 1 | 34 | 58,62 | 19 | 55,88 | 182 | 48,28 | 115 | 42,59 | 0.78 (0.41-1.48) | 0.45 | 0.99 (0.52-1.87) | 0.97 | . | . |
| ABCC4 - tag |  | 2 | 6 | 10,34 | 3 | 8,82 | 65 | 17,24 | 49 | 18,15 | 0.61 (0.17-2.20) | 0.45 | 0.83 (0.42-1.63) | 0.59 | . | . |
| ABCC4 - tag | rs931111 | 0 | 32 | 55,17 | 23 | 67,65 | 257 | 68,17 | 179 | 66,30 | 1.00 (.-.) | . | 1.26 (0.77-2.04) | 0.36 | 0.99 | 0.98 |
| ABCC4 - tag |  | 1 | 22 | 37,93 | 9 | 26,47 | 108 | 28,65 | 76 | 28,15 | 0.94 (0.52-1.72) | 0.85 | 1.17 (0.72-1.91) | 0.53 | . | . |
| ABCC4 - tag |  | 2 | 4 | 6,90 | 2 | 5,88 | 12 | 3,18 | 15 | 5,56 | 0.89 (0.27-2.96) | 0.85 | 1.09 (0.60-1.99) | 0.78 | . | . |
| ABCC4 - tag | rs943288 | 0 | 47 | 81,03 | 28 | 82,35 | 285 | 75,60 | 202 | 74,81 | 1.00 (.-.) | . | 1.30 (0.82-2.08) | 0.26 | 0.99 | 0.98 |
| ABCC4 - tag |  | 1 | 11 | 18,97 | 5 | 14,71 | 84 | 22,28 | 62 | 22,96 | 1.32 (0.59-2.96) | 0.51 | 1.38 (0.83-2.28) | 0.21 | . | . |
| ABCC4 - tag |  | 2 | 0 | 0,00 | 1 | 2,94 | 8 | 2,12 | 6 | 2,22 | 1.73 (0.34-8.77) | 0.51 | 1.46 (0.76-2.82) | 0.26 | . | . |
| ABCC4 - tag | rs943290 | 0 | 31 | 53,45 | 21 | 61,76 | 182 | 48,28 | 153 | 56,67 | 1.00 (.-.) | . | 1.16 (0.72-1.88) | 0.53 | 0.83 | 0.70 |
| ABCC4 - tag |  | 1 | 21 | 36,21 | 11 | 32,35 | 164 | 43,50 | 100 | 37,04 | 0.66 (0.33-1.31) | 0.24 | 0.97 (0.61-1.57) | 0.92 | . | . |
| ABCC4 - tag |  | 2 | 6 | 10,34 | 2 | 5,88 | 31 | 8,22 | 17 | 6,30 | 0.43 (0.11-1.72) | 0.24 | 0.82 (0.46-1.44) | 0.48 | . | . |
| ABCC4 - tag | rs9516530 | 0 | 31 | 53,45 | 23 | 67,65 | 208 | 55,17 | 153 | 56,67 | 1.00 (.-.) | . | 1.13 (0.68-1.87) | 0.63 | 0.88 | 0.71 |
| ABCC4 - tag |  | 1 | 25 | 43,10 | 9 | 26,47 | 131 | 34,75 | 97 | 35,93 | 0.73 (0.36-1.50) | 0.40 | 1.05 (0.64-1.74) | 0.84 | . | . |
| ABCC4 - tag |  | 2 | 2 | 3,45 | 2 | 5,88 | 38 | 10,08 | 20 | 7,41 | 0.54 (0.13-2.25) | 0.40 | 0.98 (0.55-1.75) | 0.95 | . | . |
| ABCC4 - tag | rs9516551 | 0 | 43 | 74,14 | 23 | 67,65 | 291 | 77,19 | 210 | 77,78 | 1.00 (.-.) | . | 1.21 (0.74-1.98) | 0.44 | 0.66 | 0.60 |
| ABCC4 - tag |  | 1 | 14 | 24,14 | 11 | 32,35 | 82 | 21,75 | 57 | 21,11 | 0.80 (0.37-1.76) | 0.58 | 1.02 (0.60-1.72) | 0.95 | . | . |
| ABCC4 - tag |  | 2 | 1 | 1,72 | 0 | 0,00 | 4 | 1,06 | 3 | 1,11 | 0.64 (0.13-3.10) | 0.58 | 0.85 (0.43-1.71) | 0.66 | . | . |
| ABCC4 - tag | rs9524822 | 0 | 45 | 77,59 | 21 | 61,76 | 240 | 63,66 | 173 | 64,07 | 1.00 (.-.) | . | 1.69 (0.98-2.93) | 0.06 | 0.85 | 0.70 |
| ABCC4 - tag |  | 1 | 13 | 22,41 | 9 | 26,47 | 123 | 32,63 | 83 | 30,74 | 1.89 (0.98-3.65) | 0.06 | 1.55 (0.89-2.71) | 0.12 | . | . |
| ABCC4 - tag |  | 2 | 0 | 0,00 | 4 | 11,76 | 14 | 3,71 | 14 | 5,19 | 3.56 (0.95-13.32) | 0.06 | 1.42 (0.73-2.76) | 0.30 | . | . |
| ABCC4 - tag | rs9524861 | 0 | 23 | 39,66 | 15 | 44,12 | 200 | 53,05 | 139 | 51,48 | 1.00 (.-.) | . | 0.87 (0.50-1.51) | 0.61 | 0.99 | 0.98 |
| ABCC4 - tag |  | 1 | 26 | 44,83 | 15 | 44,12 | 151 | 40,05 | 110 | 40,74 | 0.63 (0.37-1.07) | 0.09 | 0.86 (0.50-1.49) | 0.59 | . | . |
| ABCC4 - tag |  | 2 | 9 | 15,52 | 4 | 11,76 | 26 | 6,90 | 21 | 7,78 | 0.39 (0.14-1.14) | 0.09 | 0.85 (0.46-1.57) | 0.61 | . | . |
| ABCC4 - tag | rs9524902 | 0 | 15 | 25,86 | 8 | 23,53 | 98 | 25,99 | 93 | 34,44 | 1.00 (.-.) | . | 1.28 (0.64-2.59) | 0.49 | 0.66 | 0.60 |
| ABCC4 - tag |  | 1 | 31 | 53,45 | 16 | 47,06 | 195 | 51,72 | 123 | 45,56 | 0.84 (0.48-1.47) | 0.55 | 1.02 (0.52-2.02) | 0.95 | . | . |
| ABCC4 - tag |  | 2 | 12 | 20,69 | 10 | 29,41 | 84 | 22,28 | 54 | 20,00 | 0.71 (0.23-2.16) | 0.55 | 0.81 (0.40-1.67) | 0.57 | . | . |
| ABCC4 - tag | rs9556455 | 0 | 45 | 77,59 | 23 | 67,65 | 291 | 77,19 | 203 | 75,19 | 1.00 (.-.) | . | 1.36 (0.83-2.23) | 0.22 | 0.84 | 0.70 |
| ABCC4 - tag |  | 1 | 13 | 22,41 | 9 | 26,47 | 84 | 22,28 | 60 | 22,22 | 1.28 (0.70-2.35) | 0.43 | 1.40 (0.83-2.38) | 0.21 | . | . |
| ABCC4 - tag |  | 2 | 0 | 0,00 | 2 | 5,88 | 2 | 0,53 | 7 | 2,59 | 1.63 (0.48-5.52) | 0.43 | 1.45 (0.74-2.82) | 0.28 | . | . |
| ABCC4 - NA | rs9561778 | 0 | 36 | 62,07 | 24 | 70,59 | 235 | 62,33 | 190 | 70,37 | 1.00 (.-.) | . | 1.18 (0.75-1.85) | 0.47 | 0.99 | 0.98 |
| ABCC4 - NA |  | 1 | 20 | 34,48 | 10 | 29,41 | 125 | 33,16 | 73 | 27,04 | 0.66 (0.29-1.53) | 0.33 | 1.01 (0.63-1.63) | 0.97 | . | . |
| ABCC4 - NA |  | 2 | 2 | 3,45 | 0 | 0,00 | 17 | 4,51 | 7 | 2,59 | 0.44 (0.08-2.33) | 0.33 | 0.86 (0.47-1.59) | 0.64 | . | . |
| ABCC4 - tag | rs9561811 | 0 | 40 | 68,97 | 25 | 73,53 | 252 | 66,84 | 178 | 65,93 | 1.00 (.-.) | . | 1.37 (0.84-2.22) | 0.21 | 0.88 | 0.71 |
| ABCC4 - tag |  | 1 | 17 | 29,31 | 9 | 26,47 | 112 | 29,71 | 77 | 28,52 | 1.87 (0.80-4.36) | 0.15 | 1.55 (0.95-2.54) | 0.08 | . | . |
| ABCC4 - tag |  | 2 | 1 | 1,72 | 0 | 0,00 | 13 | 3,45 | 15 | 5,56 | 3.50 (0.64-19.02) | 0.15 | 1.76 (0.97-3.18) | 0.06 | . | . |
| ABCC4 - tag | rs9590183 | 0 | 52 | 89,66 | 32 | 94,12 | 321 | 85,15 | 236 | 87,41 | 1.00 (.-.) | . | 1.20 (0.78-1.84) | 0.40 | 0.93 | 0.87 |
| ABCC4 - tag |  | 1 | 6 | 10,34 | 2 | 5,88 | 54 | 14,32 | 34 | 12,59 | 0.56 (0.13-2.42) | 0.44 | 1.00 (0.57-1.76) | 0.99 | . | . |
| ABCC4 - tag |  | 2 | 0 | 0,00 | 0 | 0,00 | 2 | 0,53 | 0 | 0,00 | 0.31 (0.02-5.87) | 0.44 | 0.84 (0.35-2.03) | 0.70 | . | . |
| ABCC4 - tag | rs997777 | 0 | 26 | 44,83 | 16 | 47,06 | 185 | 49,07 | 134 | 49,63 | 1.00 (.-.) | . | 1.41 (0.81-2.45) | 0.22 | 0.99 | 0.98 |
| ABCC4 - tag |  | 1 | 24 | 41,38 | 14 | 41,18 | 162 | 42,97 | 115 | 42,59 | 1.16 (0.62-2.18) | 0.65 | 1.29 (0.75-2.24) | 0.36 | . | . |
| ABCC4 - tag |  | 2 | 8 | 13,79 | 4 | 11,76 | 30 | 7,96 | 21 | 7,78 | 1.34 (0.38-4.74) | 0.65 | 1.19 (0.65-2.19) | 0.58 | . | . |
| ADH1B - tag | rs1159918 | 0 | 21 | 36,21 | 12 | 35,29 | 172 | 45,62 | 130 | 48,15 | 1.00 (.-.) | . | 0.90 (0.48-1.69) | 0.74 | 0.93 | 0.64 |
| ADH1B - tag |  | 1 | 31 | 53,45 | 16 | 47,06 | 162 | 42,97 | 122 | 45,19 | 0.66 (0.37-1.19) | 0.17 | 0.85 (0.46-1.59) | 0.62 | . | . |
| ADH1B - tag |  | 2 | 6 | 10,34 | 6 | 17,65 | 43 | 11,41 | 18 | 6,67 | 0.44 (0.13-1.41) | 0.17 | 0.81 (0.41-1.60) | 0.54 | . | . |
| ADH1B - candidate literature | rs1229984 | 0 | 51 | 87,93 | 32 | 94,12 | 340 | 90,19 | 245 | 90,74 | 1.00 (.-.) | . | 1.14 (0.75-1.75) | 0.54 | 0.96 | 0.64 |
| ADH1B - candidate literature |  | 1 | 6 | 10,34 | 2 | 5,88 | 35 | 9,28 | 25 | 9,26 | 0.41 (0.10-1.64) | 0.21 | 0.99 (0.56-1.75) | 0.97 | . | . |
| ADH1B - candidate literature |  | 2 | 1 | 1,72 | 0 | 0,00 | 2 | 0,53 | 0 | 0,00 | 0.17 (0.01-2.68) | 0.21 | 0.86 (0.34-2.13) | 0.74 | . | . |
| ADH1B - tag | rs12507573 | 0 | 17 | 29,31 | 14 | 41,18 | 115 | 30,50 | 73 | 27,04 | 1.00 (.-.) | . | 0.89 (0.48-1.64) | 0.71 | 0.99 | 0.85 |
| ADH1B - tag |  | 1 | 25 | 43,10 | 20 | 58,82 | 179 | 47,48 | 131 | 48,52 | 0.66 (0.35-1.22) | 0.18 | 0.91 (0.51-1.63) | 0.76 | . | . |
| ADH1B - tag |  | 2 | 16 | 27,59 | 0 | 0,00 | 83 | 22,02 | 66 | 24,44 | 0.43 (0.12-1.48) | 0.18 | 0.94 (0.51-1.72) | 0.84 | . | . |
| ADH1B - tag | rs1693457 | 0 | 38 | 65,52 | 21 | 61,76 | 261 | 69,23 | 195 | 72,22 | 1.00 (.-.) | . | 1.07 (0.64-1.79) | 0.79 | 0.66 | 0.37 |
| ADH1B - tag |  | 1 | 19 | 32,76 | 11 | 32,35 | 100 | 26,53 | 71 | 26,30 | 0.64 (0.30-1.35) | 0.24 | 0.90 (0.53-1.54) | 0.71 | . | . |
| ADH1B - tag |  | 2 | 1 | 1,72 | 2 | 5,88 | 16 | 4,24 | 4 | 1,48 | 0.40 (0.09-1.82) | 0.24 | 0.76 (0.39-1.50) | 0.43 | . | . |
| ADH1B - tag | rs2066701 | 0 | 30 | 51,72 | 9 | 26,47 | 178 | 47,21 | 123 | 45,56 | 1.00 (.-.) | . | 2.14 (1.05-4.36) | 0.04 | 0.93 | 0.64 |
| ADH1B - tag |  | 1 | 26 | 44,83 | 23 | 67,65 | 160 | 42,44 | 118 | 43,70 | 2.38 (1.19-4.77) | 0.01 | 2.35 (1.16-4.75) | 0.02 | . | . |
| ADH1B - tag |  | 2 | 2 | 3,45 | 2 | 5,88 | 39 | 10,34 | 29 | 10,74 | 5.67 (1.41-22.76) | 0.01 | 2.58 (1.22-5.47) | 0.01 | . | . |
| ADH1C - tag | rs11936869 | 0 | 29 | 50,00 | 13 | 38,24 | 194 | 51,46 | 152 | 56,30 | 1.00 (.-.) | . | 1.37 (0.78-2.41) | 0.28 | 0.85 | 0.63 |
| ADH1C - tag |  | 1 | 24 | 41,38 | 16 | 47,06 | 148 | 39,26 | 97 | 35,93 | 1.10 (0.62-1.93) | 0.75 | 1.28 (0.73-2.25) | 0.39 | . | . |
| ADH1C - tag |  | 2 | 5 | 8,62 | 5 | 14,71 | 35 | 9,28 | 21 | 7,78 | 1.20 (0.39-3.72) | 0.75 | 1.20 (0.63-2.26) | 0.58 | . | . |
| ADH1C - tag | rs1229849 | 0 | 33 | 56,90 | 20 | 58,82 | 204 | 54,11 | 130 | 48,15 | 1.00 (.-.) | . | 1.19 (0.71-2.01) | 0.50 | 0.99 | 0.95 |
| ADH1C - tag |  | 1 | 22 | 37,93 | 13 | 38,24 | 155 | 41,11 | 119 | 44,07 | 1.05 (0.57-1.95) | 0.87 | 1.35 (0.80-2.26) | 0.26 | . | . |
| ADH1C - tag |  | 2 | 3 | 5,17 | 1 | 2,94 | 18 | 4,77 | 21 | 7,78 | 1.11 (0.32-3.79) | 0.87 | 1.52 (0.83-2.77) | 0.18 | . | . |
| ADH1C - tag | rs1229863 | 0 | 42 | 72,41 | 24 | 70,59 | 275 | 72,94 | 198 | 73,33 | 1.00 (.-.) | . | 1.28 (0.81-2.03) | 0.28 | 0.97 | 0.69 |
| ADH1C - tag |  | 1 | 15 | 25,86 | 8 | 23,53 | 93 | 24,67 | 65 | 24,07 | 1.10 (0.58-2.10) | 0.77 | 1.25 (0.77-2.04) | 0.37 | . | . |
| ADH1C - tag |  | 2 | 1 | 1,72 | 2 | 5,88 | 9 | 2,39 | 7 | 2,59 | 1.21 (0.34-4.39) | 0.77 | 1.22 (0.64-2.31) | 0.54 | . | . |
| ADH1C - tag | rs1229980 | 0 | 52 | 89,66 | 30 | 88,24 | 343 | 90,98 | 240 | 88,89 | 1.00 (.-.) | . | 1.32 (0.85-2.04) | 0.21 | 0.93 | 0.63 |
| ADH1C - tag |  | 1 | 6 | 10,34 | 4 | 11,76 | 34 | 9,02 | 28 | 10,37 | 3.20 (0.94-10.97) | 0.06 | 1.58 (0.91-2.72) | 0.10 | . | . |
| ADH1C - tag |  | 2 | 0 | 0,00 | 0 | 0,00 | 0 | 0,00 | 2 | 0,74 | 10.27 (0.88-120.4) | 0.06 | 1.88 (0.82-4.34) | 0.14 | . | . |
| ADH1C - candidate | rs1693482 | 0 | 27 | 46,55 | 15 | 44,12 | 157 | 41,64 | 106 | 39,26 | 1.00 (.-.) | . | 1.12 (0.65-1.93) | 0.69 | 0.93 | 0.63 |
| ADH1C - candidate |  | 1 | 23 | 39,66 | 16 | 47,06 | 173 | 45,89 | 120 | 44,44 | 0.90 (0.52-1.53) | 0.69 | 1.17 (0.69-2.00) | 0.56 | . | . |
| ADH1C - candidate |  | 2 | 8 | 13,79 | 3 | 8,82 | 47 | 12,47 | 44 | 16,30 | 0.80 (0.27-2.35) | 0.69 | 1.23 (0.68-2.23) | 0.49 | . | . |
| ADH1C - tag | rs2173201 | 0 | 33 | 56,90 | 14 | 41,18 | 220 | 58,36 | 160 | 59,26 | 1.00 (.-.) | . | 1.60 (0.92-2.79) | 0.09 | 0.93 | 0.63 |
| ADH1C - tag |  | 1 | 21 | 36,21 | 18 | 52,94 | 132 | 35,01 | 94 | 34,81 | 1.79 (0.98-3.26) | 0.06 | 1.70 (0.98-2.95) | 0.06 | . | . |
| ADH1C - tag |  | 2 | 4 | 6,90 | 2 | 5,88 | 25 | 6,63 | 16 | 5,93 | 3.21 (0.97-10.66) | 0.06 | 1.80 (0.95-3.38) | 0.07 | . | . |
| ADH1C - tag | rs2298753 | 0 | 48 | 82,76 | 28 | 82,35 | 304 | 80,64 | 225 | 83,33 | 1.00 (.-.) | . | 1.24 (0.80-1.93) | 0.33 | 0.86 | 0.63 |
| ADH1C - tag |  | 1 | 9 | 15,52 | 4 | 11,76 | 69 | 18,30 | 43 | 15,93 | 0.85 (0.39-1.85) | 0.69 | 1.04 (0.62-1.76) | 0.87 | . | . |
| ADH1C - tag |  | 2 | 1 | 1,72 | 2 | 5,88 | 4 | 1,06 | 2 | 0,74 | 0.73 (0.16-3.41) | 0.69 | 0.88 (0.41-1.90) | 0.74 | . | . |
| ADH1C - tag | rs2866152 | 0 | 37 | 63,79 | 24 | 70,59 | 232 | 61,54 | 153 | 56,67 | 1.00 (.-.) | . | 1.13 (0.69-1.84) | 0.64 | 0.99 | 0.95 |
| ADH1C - tag |  | 1 | 20 | 34,48 | 9 | 26,47 | 130 | 34,48 | 103 | 38,15 | 0.85 (0.44-1.66) | 0.64 | 1.23 (0.75-2.03) | 0.42 | . | . |
| ADH1C - tag |  | 2 | 1 | 1,72 | 1 | 2,94 | 15 | 3,98 | 14 | 5,19 | 0.73 (0.19-2.76) | 0.64 | 1.34 (0.73-2.46) | 0.35 | . | . |
| ADH1C - tag | rs904096 | 0 | 27 | 46,55 | 15 | 44,12 | 154 | 40,85 | 105 | 38,89 | 1.00 (.-.) | . | 1.13 (0.65-1.95) | 0.67 | 0.93 | 0.63 |
| ADH1C - tag |  | 1 | 23 | 39,66 | 16 | 47,06 | 176 | 46,68 | 121 | 44,81 | 0.90 (0.52-1.53) | 0.69 | 1.17 (0.69-1.99) | 0.57 | . | . |
| ADH1C - tag |  | 2 | 8 | 13,79 | 3 | 8,82 | 47 | 12,47 | 44 | 16,30 | 0.80 (0.27-2.35) | 0.69 | 1.21 (0.67-2.20) | 0.52 | . | . |
| BHMT - tag | rs10944 | 0 | 17 | 29,31 | 6 | 17,65 | 85 | 22,55 | 65 | 24,07 | 1.00 (.-.) | . | 1.32 (0.63-2.76) | 0.46 | 0.93 | 0.72 |
| BHMT - tag |  | 1 | 25 | 43,10 | 20 | 58,82 | 201 | 53,32 | 139 | 51,48 | 1.04 (0.60-1.81) | 0.88 | 1.30 (0.64-2.63) | 0.47 | . | . |
| BHMT - tag |  | 2 | 16 | 27,59 | 8 | 23,53 | 91 | 24,14 | 66 | 24,44 | 1.09 (0.36-3.26) | 0.88 | 1.27 (0.61-2.64) | 0.52 | . | . |
| BHMT - tag | rs12655567 | 0 | 22 | 37,93 | 14 | 41,18 | 133 | 35,28 | 104 | 38,52 | 1.00 (.-.) | . | 1.30 (0.73-2.32) | 0.37 | 0.66 | 0.21 |
| BHMT - tag |  | 1 | 30 | 51,72 | 15 | 44,12 | 188 | 49,87 | 132 | 48,89 | 0.87 (0.49-1.55) | 0.64 | 1.09 (0.63-1.91) | 0.75 | . | . |
| BHMT - tag |  | 2 | 6 | 10,34 | 5 | 14,71 | 56 | 14,85 | 34 | 12,59 | 0.76 (0.24-2.40) | 0.64 | 0.92 (0.50-1.69) | 0.78 | . | . |
| BHMT - tag | rs1291041 | 0 | 27 | 46,55 | 15 | 44,12 | 153 | 40,58 | 121 | 44,81 | 1.00 (.-.) | . | 1.26 (0.72-2.21) | 0.41 | 0.59 | 0.21 |
| BHMT - tag |  | 1 | 26 | 44,83 | 15 | 44,12 | 176 | 46,68 | 120 | 44,44 | 0.87 (0.49-1.56) | 0.65 | 1.09 (0.63-1.89) | 0.75 | . | . |
| BHMT - tag |  | 2 | 5 | 8,62 | 4 | 11,76 | 48 | 12,73 | 29 | 10,74 | 0.76 (0.24-2.43) | 0.65 | 0.95 (0.52-1.74) | 0.86 | . | . |
| BHMT - tag | rs16876500 | 0 | 47 | 81,03 | 28 | 82,35 | 304 | 80,64 | 210 | 77,78 | 1.00 (.-.) | . | 1.13 (0.73-1.77) | 0.58 | 0.99 | 0.72 |
| BHMT - tag |  | 1 | 10 | 17,24 | 5 | 14,71 | 68 | 18,04 | 57 | 21,11 | 0.80 (0.38-1.69) | 0.57 | 1.41 (0.86-2.33) | 0.18 | . | . |
| BHMT - tag |  | 2 | 1 | 1,72 | 1 | 2,94 | 5 | 1,33 | 3 | 1,11 | 0.65 (0.15-2.87) | 0.57 | 1.76 (0.87-3.54) | 0.11 | . | . |
| BHMT - tag | rs492842 | 0 | 24 | 41,38 | 13 | 38,24 | 142 | 37,67 | 109 | 40,37 | 1.00 (.-.) | . | 1.29 (0.71-2.35) | 0.39 | 0.99 | 0.72 |
| BHMT - tag |  | 1 | 22 | 37,93 | 19 | 55,88 | 187 | 49,60 | 122 | 45,19 | 1.01 (0.57-1.78) | 0.97 | 1.24 (0.69-2.21) | 0.48 | . | . |
| BHMT - tag |  | 2 | 12 | 20,69 | 2 | 5,88 | 48 | 12,73 | 39 | 14,44 | 1.02 (0.33-3.15) | 0.97 | 1.18 (0.63-2.21) | 0.61 | . | . |
| BHMT - tag | rs558133 | 0 | 25 | 43,10 | 18 | 52,94 | 184 | 48,81 | 125 | 46,30 | 1.00 (.-.) | . | 1.36 (0.77-2.39) | 0.29 | 0.93 | 0.72 |
| BHMT - tag |  | 1 | 23 | 39,66 | 9 | 26,47 | 151 | 40,05 | 120 | 44,44 | 1.17 (0.72-1.92) | 0.53 | 1.41 (0.81-2.46) | 0.23 | . | . |
| BHMT - tag |  | 2 | 10 | 17,24 | 7 | 20,59 | 42 | 11,14 | 25 | 9,26 | 1.37 (0.51-3.67) | 0.53 | 1.47 (0.79-2.72) | 0.22 | . | . |
| BHMT - tag | rs9637824 | 0 | 22 | 37,93 | 12 | 35,29 | 142 | 37,67 | 105 | 38,89 | 1.00 (.-.) | . | 1.46 (0.78-2.72) | 0.23 | 0.99 | 0.72 |
| BHMT - tag |  | 1 | 26 | 44,83 | 19 | 55,88 | 186 | 49,34 | 127 | 47,04 | 1.21 (0.67-2.16) | 0.53 | 1.41 (0.77-2.59) | 0.27 | . | . |
| BHMT - tag |  | 2 | 10 | 17,24 | 3 | 8,82 | 49 | 13,00 | 38 | 14,07 | 1.45 (0.45-4.65) | 0.53 | 1.36 (0.70-2.62) | 0.36 | . | . |
| BHMT2 - tag | rs16876512 | 0 | 47 | 81,03 | 28 | 82,35 | 301 | 79,84 | 210 | 77,78 | 1.00 (.-.) | . | 1.13 (0.73-1.77) | 0.58 | 0.99 | 0.71 |
| BHMT2 - tag |  | 1 | 10 | 17,24 | 5 | 14,71 | 71 | 18,83 | 56 | 20,74 | 0.80 (0.38-1.69) | 0.57 | 1.40 (0.85-2.31) | 0.18 | . | . |
| BHMT2 - tag |  | 2 | 1 | 1,72 | 1 | 2,94 | 5 | 1,33 | 4 | 1,48 | 0.65 (0.15-2.87) | 0.57 | 1.74 (0.87-3.47) | 0.12 | . | . |
| BHMT2 - tag | rs2461248 | 0 | 17 | 29,31 | 5 | 14,71 | 84 | 22,28 | 65 | 24,07 | 1.00 (.-.) | . | 1.41 (0.66-3.01) | 0.37 | 0.94 | 0.70 |
| BHMT2 - tag |  | 1 | 25 | 43,10 | 21 | 61,76 | 201 | 53,32 | 138 | 51,11 | 1.11 (0.64-1.94) | 0.71 | 1.39 (0.67-2.87) | 0.38 | . | . |
| BHMT2 - tag |  | 2 | 16 | 27,59 | 8 | 23,53 | 92 | 24,40 | 67 | 24,81 | 1.23 (0.41-3.76) | 0.71 | 1.36 (0.64-2.89) | 0.42 | . | . |
| BHMT2 - tag | rs2909856 | 0 | 25 | 43,10 | 15 | 44,12 | 155 | 41,11 | 115 | 42,59 | 1.00 (.-.) | . | 1.31 (0.74-2.32) | 0.35 | 0.93 | 0.70 |
| BHMT2 - tag |  | 1 | 23 | 39,66 | 17 | 50,00 | 178 | 47,21 | 119 | 44,07 | 1.05 (0.59-1.85) | 0.88 | 1.26 (0.72-2.20) | 0.41 | . | . |
| BHMT2 - tag |  | 2 | 10 | 17,24 | 2 | 5,88 | 44 | 11,67 | 36 | 13,33 | 1.09 (0.35-3.43) | 0.88 | 1.21 (0.66-2.23) | 0.54 | . | . |
| BHMT2 - tag | rs476620 | 0 | 22 | 37,93 | 12 | 35,29 | 142 | 37,67 | 104 | 38,52 | 1.00 (.-.) | . | 1.45 (0.78-2.70) | 0.24 | 0.99 | 0.71 |
| BHMT2 - tag |  | 1 | 26 | 44,83 | 19 | 55,88 | 186 | 49,34 | 128 | 47,41 | 1.21 (0.67-2.16) | 0.53 | 1.41 (0.77-2.59) | 0.27 | . | . |
| BHMT2 - tag |  | 2 | 10 | 17,24 | 3 | 8,82 | 49 | 13,00 | 38 | 14,07 | 1.45 (0.45-4.66) | 0.53 | 1.37 (0.71-2.65) | 0.35 | . | . |
| BHMT2 - candidate literature | rs626105 | 0 | 33 | 56,90 | 23 | 67,65 | 236 | 62,60 | 174 | 64,44 | 1.00 (.-.) | . | 1.23 (0.75-2.00) | 0.41 | 0.89 | 0.70 |
| BHMT2 - candidate literature |  | 1 | 22 | 37,93 | 11 | 32,35 | 131 | 34,75 | 88 | 32,59 | 0.84 (0.40-1.78) | 0.65 | 1.09 (0.66-1.80) | 0.74 | . | . |
| BHMT2 - candidate literature |  | 2 | 3 | 5,17 | 0 | 0,00 | 10 | 2,65 | 8 | 2,96 | 0.71 (0.16-3.18) | 0.65 | 0.97 (0.52-1.80) | 0.91 | . | . |
| BHMT2 - tag | rs631305 | 0 | 37 | 63,79 | 26 | 76,47 | 263 | 69,76 | 191 | 70,74 | 1.00 (.-.) | . | 1.21 (0.76-1.92) | 0.42 | 0.93 | 0.70 |
| BHMT2 - tag |  | 1 | 18 | 31,03 | 8 | 23,53 | 105 | 27,85 | 71 | 26,30 | 0.84 (0.38-1.88) | 0.67 | 1.14 (0.69-1.86) | 0.61 | . | . |
| BHMT2 - tag |  | 2 | 3 | 5,17 | 0 | 0,00 | 9 | 2,39 | 8 | 2,96 | 0.71 (0.14-3.52) | 0.67 | 1.07 (0.57-2.01) | 0.84 | . | . |
| CBS - tag | rs11701048 | 0 | 50 | 86,21 | 28 | 82,35 | 318 | 84,35 | 237 | 87,78 | 1.00 (.-.) | . | 1.38 (0.87-2.18) | 0.17 | 0.99 | 0.79 |
| CBS - tag |  | 1 | 8 | 13,79 | 6 | 17,65 | 58 | 15,38 | 32 | 11,85 | 1.59 (0.63-3.99) | 0.33 | 1.17 (0.66-2.07) | 0.60 | . | . |
| CBS - tag |  | 2 | 0 | 0,00 | 0 | 0,00 | 1 | 0,27 | 1 | 0,37 | 2.52 (0.40-15.95) | 0.33 | 0.98 (0.41-2.36) | 0.97 | . | . |
| CBS - tag | rs234706 | 0 | 25 | 43,10 | 11 | 32,35 | 166 | 44,03 | 108 | 40,00 | 1.00 (.-.) | . | 1.47 (0.78-2.80) | 0.24 | 0.99 | 0.79 |
| CBS - tag |  | 1 | 26 | 44,83 | 18 | 52,94 | 166 | 44,03 | 125 | 46,30 | 1.29 (0.74-2.25) | 0.37 | 1.52 (0.82-2.84) | 0.19 | . | . |
| CBS - tag |  | 2 | 7 | 12,07 | 5 | 14,71 | 45 | 11,94 | 37 | 13,70 | 1.66 (0.55-5.04) | 0.37 | 1.58 (0.81-3.05) | 0.18 | . | . |
| CBS - tag | rs234711 | 0 | 35 | 60,34 | 19 | 55,88 | 227 | 60,21 | 149 | 55,19 | 1.00 (.-.) | . | 1.39 (0.80-2.43) | 0.25 | 0.93 | 0.79 |
| CBS - tag |  | 1 | 20 | 34,48 | 13 | 38,24 | 128 | 33,95 | 103 | 38,15 | 1.37 (0.65-2.88) | 0.40 | 1.45 (0.84-2.52) | 0.18 | . | . |
| CBS - tag |  | 2 | 3 | 5,17 | 2 | 5,88 | 22 | 5,84 | 18 | 6,67 | 1.88 (0.43-8.29) | 0.40 | 1.51 (0.81-2.83) | 0.19 | . | . |
| CBS - candidate literature | rs234713 | 0 | 30 | 51,72 | 16 | 47,06 | 187 | 49,60 | 127 | 47,04 | 1.00 (.-.) | . | 1.53 (0.86-2.72) | 0.15 | 0.99 | 0.79 |
| CBS - candidate literature |  | 1 | 22 | 37,93 | 14 | 41,18 | 153 | 40,58 | 118 | 43,70 | 1.37 (0.78-2.43) | 0.27 | 1.49 (0.85-2.59) | 0.17 | . | . |
| CBS - candidate literature |  | 2 | 6 | 10,34 | 4 | 11,76 | 37 | 9,81 | 25 | 9,26 | 1.89 (0.60-5.90) | 0.27 | 1.44 (0.78-2.66) | 0.25 | . | . |
| CBS - tag | rs2839623 | 0 | 47 | 81,03 | 30 | 88,24 | 312 | 82,76 | 221 | 81,85 | 1.00 (.-.) | . | 1.22 (0.78-1.90) | 0.39 | 0.85 | 0.71 |
| CBS - tag |  | 1 | 11 | 18,97 | 4 | 11,76 | 63 | 16,71 | 46 | 17,04 | 0.92 (0.31-2.71) | 0.88 | 1.27 (0.75-2.14) | 0.37 | . | . |
| CBS - tag |  | 2 | 0 | 0,00 | 0 | 0,00 | 2 | 0,53 | 3 | 1,11 | 0.85 (0.10-7.32) | 0.88 | 1.32 (0.62-2.82) | 0.47 | . | . |
| CBS - tag | rs2839626 | 0 | 26 | 44,83 | 14 | 41,18 | 187 | 49,60 | 123 | 45,56 | 1.00 (.-.) | . | 1.08 (0.62-1.88) | 0.78 | 0.99 | 0.79 |
| CBS - tag |  | 1 | 25 | 43,10 | 17 | 50,00 | 150 | 39,79 | 126 | 46,67 | 0.86 (0.50-1.48) | 0.59 | 1.15 (0.67-1.98) | 0.62 | . | . |
| CBS - tag |  | 2 | 7 | 12,07 | 3 | 8,82 | 40 | 10,61 | 21 | 7,78 | 0.74 (0.25-2.18) | 0.59 | 1.22 (0.67-2.23) | 0.52 | . | . |
| CBS - tag | rs422791 | 0 | 28 | 48,28 | 20 | 58,82 | 196 | 51,99 | 118 | 43,70 | 1.00 (.-.) | . | 1.11 (0.66-1.86) | 0.69 | 0.59 | 0.14 |
| CBS - tag |  | 1 | 23 | 39,66 | 12 | 35,29 | 143 | 37,93 | 126 | 46,67 | 0.89 (0.47-1.67) | 0.72 | 1.21 (0.73-2.01) | 0.45 | . | . |
| CBS - tag |  | 2 | 7 | 12,07 | 2 | 5,88 | 38 | 10,08 | 26 | 9,63 | 0.79 (0.23-2.79) | 0.72 | 1.32 (0.76-2.32) | 0.33 | . | . |
| CBS - tag | rs706209 | 0 | 18 | 31,03 | 8 | 23,53 | 124 | 32,89 | 86 | 31,85 | 1.00 (.-.) | . | 0.97 (0.48-1.96) | 0.93 | 0.99 | 0.79 |
| CBS - tag |  | 1 | 28 | 48,28 | 16 | 47,06 | 184 | 48,81 | 136 | 50,37 | 0.85 (0.51-1.43) | 0.54 | 1.06 (0.53-2.09) | 0.88 | . | . |
| CBS - tag |  | 2 | 12 | 20,69 | 10 | 29,41 | 69 | 18,30 | 48 | 17,78 | 0.73 (0.26-2.03) | 0.54 | 1.15 (0.56-2.35) | 0.70 | . | . |
| CBS - tag | rs719037 | 0 | 19 | 32,76 | 14 | 41,18 | 129 | 34,22 | 75 | 27,78 | 1.00 (.-.) | . | 1.11 (0.62-2.00) | 0.73 | 0.59 | 0.14 |
| CBS - tag |  | 1 | 26 | 44,83 | 17 | 50,00 | 175 | 46,42 | 140 | 51,85 | 0.94 (0.53-1.66) | 0.84 | 1.20 (0.69-2.10) | 0.52 | . | . |
| CBS - tag |  | 2 | 13 | 22,41 | 3 | 8,82 | 73 | 19,36 | 55 | 20,37 | 0.89 (0.28-2.77) | 0.84 | 1.30 (0.72-2.36) | 0.39 | . | . |
| CBS - tag | rs719038 | 0 | 27 | 46,55 | 12 | 35,29 | 170 | 45,09 | 120 | 44,44 | 1.00 (.-.) | . | 1.04 (0.59-1.85) | 0.89 | 0.99 | 0.79 |
| CBS - tag |  | 1 | 23 | 39,66 | 18 | 52,94 | 163 | 43,24 | 120 | 44,44 | 0.81 (0.48-1.37) | 0.43 | 1.06 (0.60-1.87) | 0.84 | . | . |
| CBS - tag |  | 2 | 8 | 13,79 | 4 | 11,76 | 44 | 11,67 | 30 | 11,11 | 0.66 (0.23-1.87) | 0.43 | 1.08 (0.58-2.01) | 0.80 | . | . |
| DHFR - tag | rs10474632 | 0 | 52 | 89,66 | 29 | 85,29 | 313 | 83,02 | 225 | 83,33 | 1.00 (.-.) | . | 1.27 (0.81-1.98) | 0.29 | 0.59 | 0.20 |
| DHFR - tag |  | 1 | 6 | 10,34 | 5 | 14,71 | 60 | 15,92 | 45 | 16,67 | 0.86 (0.29-2.53) | 0.78 | 0.97 (0.57-1.65) | 0.92 | . | . |
| DHFR - tag |  | 2 | 0 | 0,00 | 0 | 0,00 | 4 | 1,06 | 0 | 0,00 | 0.74 (0.09-6.38) | 0.78 | 0.74 (0.35-1.60) | 0.45 | . | . |
| DHFR - tag | rs11951910 | 0 | 47 | 81,03 | 26 | 76,47 | 306 | 81,17 | 220 | 81,48 | 1.00 (.-.) | . | 1.12 (0.70-1.77) | 0.64 | 0.99 | 0.89 |
| DHFR - tag |  | 1 | 11 | 18,97 | 7 | 20,59 | 67 | 17,77 | 46 | 17,04 | 0.80 (0.37-1.73) | 0.57 | 1.42 (0.85-2.38) | 0.19 | . | . |
| DHFR - tag |  | 2 | 0 | 0,00 | 1 | 2,94 | 4 | 1,06 | 4 | 1,48 | 0.64 (0.14-2.98) | 0.57 | 1.81 (0.88-3.71) | 0.11 | . | . |
| DHFR - tag | rs1643665 | 0 | 30 | 51,72 | 14 | 41,18 | 176 | 46,68 | 119 | 44,07 | 1.00 (.-.) | . | 1.20 (0.66-2.20) | 0.55 | 0.99 | 0.89 |
| DHFR - tag |  | 1 | 21 | 36,21 | 15 | 44,12 | 157 | 41,64 | 131 | 48,52 | 0.95 (0.55-1.67) | 0.87 | 1.20 (0.66-2.17) | 0.55 | . | . |
| DHFR - tag |  | 2 | 7 | 12,07 | 5 | 14,71 | 44 | 11,67 | 20 | 7,41 | 0.91 (0.30-2.78) | 0.87 | 1.19 (0.62-2.28) | 0.60 | . | . |
| DHFR - tag | rs1650717 | 0 | 26 | 44,83 | 17 | 50,00 | 208 | 55,17 | 147 | 54,44 | 1.00 (.-.) | . | 1.12 (0.64-1.96) | 0.70 | 0.85 | 0.59 |
| DHFR - tag |  | 1 | 30 | 51,72 | 11 | 32,35 | 140 | 37,14 | 99 | 36,67 | 0.97 (0.58-1.62) | 0.90 | 1.31 (0.75-2.29) | 0.34 | . | . |
| DHFR - tag |  | 2 | 2 | 3,45 | 6 | 17,65 | 29 | 7,69 | 24 | 8,89 | 0.93 (0.33-2.62) | 0.90 | 1.54 (0.82-2.87) | 0.18 | . | . |
| DHFR - tag | rs1805355 | 0 | 48 | 82,76 | 26 | 76,47 | 328 | 87,00 | 239 | 88,52 | 1.00 (.-.) | . | 1.49 (0.93-2.37) | 0.10 | 0.99 | 0.91 |
| DHFR - tag |  | 1 | 10 | 17,24 | 8 | 23,53 | 48 | 12,73 | 30 | 11,11 | 2.25 (0.94-5.40) | 0.07 | 1.21 (0.67-2.18) | 0.52 | . | . |
| DHFR - tag |  | 2 | 0 | 0,00 | 0 | 0,00 | 1 | 0,27 | 1 | 0,37 | 5.08 (0.89-29.15) | 0.07 | 0.99 (0.40-2.44) | 0.98 | . | . |
| DHFR - tag | rs6151617 | 0 | 24 | 41,38 | 12 | 35,29 | 132 | 35,01 | 94 | 34,81 | 1.00 (.-.) | . | 1.27 (0.67-2.41) | 0.47 | 0.95 | 0.89 |
| DHFR - tag |  | 1 | 26 | 44,83 | 14 | 41,18 | 173 | 45,89 | 137 | 50,74 | 0.92 (0.55-1.55) | 0.76 | 1.13 (0.61-2.11) | 0.70 | . | . |
| DHFR - tag |  | 2 | 8 | 13,79 | 8 | 23,53 | 72 | 19,10 | 39 | 14,44 | 0.85 (0.30-2.39) | 0.76 | 1.01 (0.52-1.95) | 0.98 | . | . |
| DHFR - tag | rs6864493 | 0 | 36 | 62,07 | 19 | 55,88 | 210 | 55,70 | 147 | 54,44 | 1.00 (.-.) | . | 0.99 (0.58-1.70) | 0.98 | 0.99 | 0.89 |
| DHFR - tag |  | 1 | 18 | 31,03 | 13 | 38,24 | 140 | 37,14 | 110 | 40,74 | 0.69 (0.37-1.29) | 0.24 | 1.01 (0.59-1.72) | 0.97 | . | . |
| DHFR - tag |  | 2 | 4 | 6,90 | 2 | 5,88 | 27 | 7,16 | 13 | 4,81 | 0.48 (0.14-1.65) | 0.24 | 1.03 (0.56-1.90) | 0.93 | . | . |
| DHFR - tag | rs836788 | 0 | 25 | 43,10 | 19 | 55,88 | 163 | 43,24 | 112 | 41,48 | 1.00 (.-.) | . | 1.51 (0.88-2.59) | 0.13 | 0.99 | 0.89 |
| DHFR - tag |  | 1 | 27 | 46,55 | 11 | 32,35 | 161 | 42,71 | 124 | 45,93 | 1.29 (0.72-2.31) | 0.39 | 1.37 (0.81-2.31) | 0.24 | . | . |
| DHFR - tag |  | 2 | 6 | 10,34 | 4 | 11,76 | 53 | 14,06 | 34 | 12,59 | 1.66 (0.52-5.35) | 0.39 | 1.24 (0.70-2.20) | 0.47 | . | . |
| DHFR - tag | rs836790 | 0 | 37 | 63,79 | 22 | 64,71 | 270 | 71,62 | 183 | 67,78 | 1.00 (.-.) | . | 1.24 (0.74-2.08) | 0.42 | 0.85 | 0.59 |
| DHFR - tag |  | 1 | 20 | 34,48 | 11 | 32,35 | 96 | 25,46 | 81 | 30,00 | 1.08 (0.53-2.23) | 0.83 | 1.38 (0.81-2.37) | 0.24 | . | . |
| DHFR - tag |  | 2 | 1 | 1,72 | 1 | 2,94 | 11 | 2,92 | 6 | 2,22 | 1.17 (0.28-4.98) | 0.83 | 1.54 (0.80-2.99) | 0.20 | . | . |
| DHFR - tag | rs836817 | 0 | 25 | 43,10 | 21 | 61,76 | 186 | 49,34 | 120 | 44,44 | 1.00 (.-.) | . | 1.40 (0.84-2.32) | 0.20 | 0.85 | 0.59 |
| DHFR - tag |  | 1 | 27 | 46,55 | 11 | 32,35 | 147 | 38,99 | 128 | 47,41 | 1.31 (0.66-2.59) | 0.44 | 1.35 (0.82-2.22) | 0.24 | . | . |
| DHFR - tag |  | 2 | 6 | 10,34 | 2 | 5,88 | 44 | 11,67 | 22 | 8,15 | 1.71 (0.44-6.69) | 0.44 | 1.30 (0.74-2.29) | 0.36 | . | . |
| DNMT1 - candidate | rs2228612 | 0 | 48 | 82,76 | 27 | 79,41 | 338 | 89,66 | 232 | 85,93 | 1.00 (.-.) | . | 1.29 (0.82-2.03) | 0.27 | 0.93 | 0.43 |
| DNMT1 - candidate |  | 1 | 10 | 17,24 | 7 | 20,59 | 38 | 10,08 | 37 | 13,70 | 1.36 (0.51-3.65) | 0.54 | 1.44 (0.84-2.49) | 0.19 | . | . |
| DNMT1 - candidate |  | 2 | 0 | 0,00 | 0 | 0,00 | 1 | 0,27 | 1 | 0,37 | 1.86 (0.26-13.34) | 0.54 | 1.61 (0.73-3.59) | 0.24 | . | . |
| DNMT3A - tag | rs10460566 | 0 | 34 | 58,62 | 22 | 64,71 | 222 | 58,89 | 160 | 59,26 | 1.00 (.-.) | . | 1.34 (0.82-2.17) | 0.24 | 0.99 | 0.92 |
| DNMT3A - tag |  | 1 | 18 | 31,03 | 12 | 35,29 | 137 | 36,34 | 95 | 35,19 | 1.13 (0.60-2.13) | 0.71 | 1.24 (0.76-2.03) | 0.38 | . | . |
| DNMT3A - tag |  | 2 | 6 | 10,34 | 0 | 0,00 | 18 | 4,77 | 15 | 5,56 | 1.27 (0.36-4.55) | 0.71 | 1.16 (0.65-2.07) | 0.63 | . | . |
| DNMT3A - candidate literature | rs11695471 | 0 | 28 | 48,28 | 12 | 35,29 | 164 | 43,50 | 117 | 43,33 | 1.00 (.-.) | . | 1.34 (0.75-2.40) | 0.33 | 0.89 | 0.89 |
| DNMT3A - candidate literature |  | 1 | 24 | 41,38 | 15 | 44,12 | 162 | 42,97 | 121 | 44,81 | 1.17 (0.67-2.05) | 0.57 | 1.40 (0.79-2.48) | 0.25 | . | . |
| DNMT3A - candidate literature |  | 2 | 6 | 10,34 | 7 | 20,59 | 51 | 13,53 | 32 | 11,85 | 1.38 (0.45-4.20) | 0.57 | 1.46 (0.79-2.71) | 0.23 | . | . |
| DNMT3A - tag | rs11887120 | 0 | 20 | 34,48 | 13 | 38,24 | 133 | 35,28 | 96 | 35,56 | 1.00 (.-.) | . | 1.07 (0.57-2.03) | 0.83 | 0.99 | 0.92 |
| DNMT3A - tag |  | 1 | 29 | 50,00 | 17 | 50,00 | 181 | 48,01 | 126 | 46,67 | 0.80 (0.45-1.44) | 0.46 | 1.03 (0.55-1.91) | 0.93 | . | . |
| DNMT3A - tag |  | 2 | 9 | 15,52 | 4 | 11,76 | 63 | 16,71 | 48 | 17,78 | 0.64 (0.20-2.07) | 0.46 | 0.98 (0.51-1.88) | 0.96 | . | . |
| DNMT3A - tag | rs12991495 | 0 | 30 | 51,72 | 14 | 41,18 | 173 | 45,89 | 127 | 47,04 | 1.00 (.-.) | . | 1.27 (0.72-2.26) | 0.41 | 0.86 | 0.89 |
| DNMT3A - tag |  | 1 | 22 | 37,93 | 14 | 41,18 | 162 | 42,97 | 114 | 42,22 | 1.12 (0.63-1.98) | 0.70 | 1.37 (0.78-2.41) | 0.27 | . | . |
| DNMT3A - tag |  | 2 | 6 | 10,34 | 6 | 17,65 | 42 | 11,14 | 29 | 10,74 | 1.25 (0.40-3.93) | 0.70 | 1.47 (0.79-2.74) | 0.22 | . | . |
| DNMT3A - tag | rs13401241 | 0 | 21 | 36,21 | 8 | 23,53 | 108 | 28,65 | 72 | 26,67 | 1.00 (.-.) | . | 1.43 (0.72-2.83) | 0.30 | 0.99 | 0.92 |
| DNMT3A - tag |  | 1 | 24 | 41,38 | 19 | 55,88 | 185 | 49,07 | 151 | 55,93 | 1.05 (0.63-1.74) | 0.86 | 1.29 (0.67-2.49) | 0.45 | . | . |
| DNMT3A - tag |  | 2 | 13 | 22,41 | 7 | 20,59 | 84 | 22,28 | 47 | 17,41 | 1.10 (0.40-3.04) | 0.86 | 1.16 (0.58-2.33) | 0.68 | . | . |
| DNMT3A - candidate literature | rs13420827 | 0 | 39 | 67,24 | 24 | 70,59 | 230 | 61,01 | 188 | 69,63 | 1.00 (.-.) | . | 1.21 (0.73-1.98) | 0.46 | 0.99 | 0.89 |
| DNMT3A - candidate literature |  | 1 | 15 | 25,86 | 6 | 17,65 | 133 | 35,28 | 73 | 27,04 | 0.84 (0.50-1.43) | 0.53 | 1.05 (0.63-1.75) | 0.85 | . | . |
| DNMT3A - candidate literature |  | 2 | 4 | 6,90 | 4 | 11,76 | 14 | 3,71 | 9 | 3,33 | 0.71 (0.25-2.05) | 0.53 | 0.91 (0.49-1.70) | 0.78 | . | . |
| DNMT3A - tag | rs13428812 | 0 | 24 | 41,38 | 21 | 61,76 | 188 | 49,87 | 119 | 44,07 | 1.00 (.-.) | . | 1.01 (0.60-1.68) | 0.98 | 0.93 | 0.89 |
| DNMT3A - tag |  | 1 | 26 | 44,83 | 11 | 32,35 | 156 | 41,38 | 131 | 48,52 | 0.75 (0.40-1.40) | 0.37 | 1.13 (0.68-1.89) | 0.63 | . | . |
| DNMT3A - tag |  | 2 | 8 | 13,79 | 2 | 5,88 | 33 | 8,75 | 20 | 7,41 | 0.56 (0.16-1.97) | 0.37 | 1.28 (0.71-2.29) | 0.41 | . | . |
| DNMT3A - tag | rs4665287 | 0 | 40 | 68,97 | 24 | 70,59 | 236 | 62,60 | 192 | 71,11 | 1.00 (.-.) | . | 1.25 (0.76-2.07) | 0.38 | 0.95 | 0.89 |
| DNMT3A - tag |  | 1 | 15 | 25,86 | 5 | 14,71 | 126 | 33,42 | 69 | 25,56 | 0.90 (0.54-1.51) | 0.69 | 1.07 (0.64-1.80) | 0.79 | . | . |
| DNMT3A - tag |  | 2 | 3 | 5,17 | 5 | 14,71 | 15 | 3,98 | 9 | 3,33 | 0.81 (0.29-2.27) | 0.69 | 0.92 (0.49-1.73) | 0.80 | . | . |
| DNMT3B - tag | rs13045669 | 0 | 54 | 93,10 | 31 | 91,18 | 348 | 92,31 | 251 | 92,96 | 1.00 (.-.) | . | 1.25 (0.81-1.92) | 0.31 | 0.93 | 0.97 |
| DNMT3B - tag |  | 1 | 4 | 6,90 | 3 | 8,82 | 29 | 7,69 | 18 | 6,67 | 0.90 (0.21-3.93) | 0.89 | 1.01 (0.53-1.95) | 0.97 | . | . |
| DNMT3B - tag |  | 2 | 0 | 0,00 | 0 | 0,00 | 0 | 0,00 | 1 | 0,37 | 0.82 (0.04-15.43) | 0.89 | 0.82 (0.27-2.50) | 0.73 | . | . |
| DNMT3B - tag | rs17123673 | 0 | 54 | 93,10 | 29 | 85,29 | 347 | 92,04 | 248 | 91,85 | 1.00 (.-.) | . | 1.30 (0.84-2.02) | 0.25 | 0.85 | 0.97 |
| DNMT3B - tag |  | 1 | 4 | 6,90 | 5 | 14,71 | 30 | 7,96 | 20 | 7,41 | 1.35 (0.46-3.96) | 0.59 | 1.20 (0.67-2.16) | 0.55 | . | . |
| DNMT3B - tag |  | 2 | 0 | 0,00 | 0 | 0,00 | 0 | 0,00 | 2 | 0,74 | 1.81 (0.21-15.65) | 0.59 | 1.11 (0.43-2.82) | 0.83 | . | . |
| DNMT3B - tag | rs183603 | 0 | 30 | 51,72 | 20 | 58,82 | 200 | 53,05 | 147 | 54,44 | 1.00 (.-.) | . | 1.01 (0.60-1.67) | 0.98 | 0.88 | 0.97 |
| DNMT3B - tag |  | 1 | 20 | 34,48 | 13 | 38,24 | 146 | 38,73 | 111 | 41,11 | 0.59 (0.30-1.14) | 0.12 | 0.89 (0.54-1.49) | 0.67 | . | . |
| DNMT3B - tag |  | 2 | 8 | 13,79 | 1 | 2,94 | 31 | 8,22 | 12 | 4,44 | 0.34 (0.09-1.31) | 0.12 | 0.79 (0.44-1.44) | 0.45 | . | . |
| DNMT3B - tag | rs2235760 | 0 | 39 | 67,24 | 23 | 67,65 | 267 | 70,82 | 200 | 74,07 | 1.00 (.-.) | . | 1.35 (0.84-2.15) | 0.21 | 0.99 | 0.97 |
| DNMT3B - tag |  | 1 | 17 | 29,31 | 11 | 32,35 | 99 | 26,26 | 62 | 22,96 | 1.10 (0.51-2.35) | 0.81 | 1.11 (0.67-1.83) | 0.68 | . | . |
| DNMT3B - tag |  | 2 | 2 | 3,45 | 0 | 0,00 | 11 | 2,92 | 8 | 2,96 | 1.20 (0.26-5.51) | 0.81 | 0.91 (0.48-1.73) | 0.78 | . | . |
| DNMT3B - tag | rs2424908 | 0 | 35 | 60,34 | 25 | 73,53 | 232 | 61,54 | 176 | 65,19 | 1.00 (.-.) | . | 1.00 (0.63-1.60) | 0.99 | 0.99 | 0.97 |
| DNMT3B - tag |  | 1 | 19 | 32,76 | 9 | 26,47 | 125 | 33,16 | 85 | 31,48 | 0.46 (0.19-1.09) | 0.08 | 0.91 (0.56-1.47) | 0.69 | . | . |
| DNMT3B - tag |  | 2 | 4 | 6,90 | 0 | 0,00 | 20 | 5,31 | 9 | 3,33 | 0.21 (0.04-1.18) | 0.08 | 0.82 (0.45-1.48) | 0.51 | . | . |
| DNMT3B - candidate literature | rs2424909 | 0 | 17 | 29,31 | 14 | 41,18 | 139 | 36,87 | 118 | 43,70 | 1.00 (.-.) | . | 0.97 (0.56-1.69) | 0.91 | 0.99 | 0.97 |
| DNMT3B - candidate literature |  | 1 | 28 | 48,28 | 19 | 55,88 | 173 | 45,89 | 114 | 42,22 | 0.59 (0.31-1.11) | 0.10 | 0.84 (0.49-1.45) | 0.54 | . | . |
| DNMT3B - candidate literature |  | 2 | 13 | 22,41 | 1 | 2,94 | 65 | 17,24 | 38 | 14,07 | 0.35 (0.10-1.23) | 0.10 | 0.73 (0.40-1.33) | 0.30 | . | . |
| DNMT3B - tag | rs4911108 | 0 | 19 | 32,76 | 14 | 41,18 | 150 | 39,79 | 123 | 45,56 | 1.00 (.-.) | . | 0.94 (0.55-1.63) | 0.84 | 0.99 | 0.97 |
| DNMT3B - tag |  | 1 | 26 | 44,83 | 20 | 58,82 | 166 | 44,03 | 112 | 41,48 | 0.55 (0.29-1.05) | 0.07 | 0.82 (0.48-1.39) | 0.46 | . | . |
| DNMT3B - tag |  | 2 | 13 | 22,41 | 0 | 0,00 | 61 | 16,18 | 35 | 12,96 | 0.31 (0.09-1.10) | 0.07 | 0.71 (0.39-1.27) | 0.25 | . | . |
| DNMT3B - tag | rs6058896 | 0 | 50 | 86,21 | 31 | 91,18 | 336 | 89,12 | 241 | 89,26 | 1.00 (.-.) | . | 1.24 (0.81-1.92) | 0.32 | 0.99 | 0.97 |
| DNMT3B - tag |  | 1 | 6 | 10,34 | 3 | 8,82 | 40 | 10,61 | 28 | 10,37 | 1.02 (0.34-3.03) | 0.98 | 1.24 (0.70-2.18) | 0.46 | . | . |
| DNMT3B - tag |  | 2 | 2 | 3,45 | 0 | 0,00 | 1 | 0,27 | 1 | 0,37 | 1.03 (0.12-9.19) | 0.98 | 1.23 (0.50-3.04) | 0.65 | . | . |
| DNMT3B - tag | rs6119954 | 0 | 36 | 62,07 | 24 | 70,59 | 263 | 69,76 | 199 | 73,70 | 1.00 (.-.) | . | 1.22 (0.76-1.95) | 0.40 | 0.99 | 0.97 |
| DNMT3B - tag |  | 1 | 19 | 32,76 | 10 | 29,41 | 99 | 26,26 | 63 | 23,33 | 0.81 (0.38-1.74) | 0.59 | 1.04 (0.63-1.71) | 0.88 | . | . |
| DNMT3B - tag |  | 2 | 3 | 5,17 | 0 | 0,00 | 15 | 3,98 | 8 | 2,96 | 0.66 (0.14-3.03) | 0.59 | 0.88 (0.47-1.66) | 0.70 | . | . |
| DNMT3B - tag | rs6579038 | 0 | 50 | 86,21 | 33 | 97,06 | 334 | 88,59 | 240 | 88,89 | 1.00 (.-.) | . | 1.22 (0.80-1.85) | 0.36 | 0.99 | 0.97 |
| DNMT3B - tag |  | 1 | 8 | 13,79 | 1 | 2,94 | 42 | 11,14 | 29 | 10,74 | 0.61 (0.08-4.54) | 0.63 | 1.20 (0.69-2.08) | 0.52 | . | . |
| DNMT3B - tag |  | 2 | 0 | 0,00 | 0 | 0,00 | 1 | 0,27 | 1 | 0,37 | 0.37 (0.01-20.60) | 0.63 | 1.18 (0.49-2.84) | 0.71 | . | . |
| DPYD - tag | rs1034215 | 0 | 38 | 65,52 | 19 | 55,88 | 209 | 55,44 | 173 | 64,07 | 1.00 (.-.) | . | 1.80 (1.03-3.16) | 0.04 | 0.87 | 0.53 |
| DPYD - tag |  | 1 | 18 | 31,03 | 15 | 44,12 | 145 | 38,46 | 81 | 30,00 | 2.05 (0.98-4.29) | 0.06 | 1.53 (0.86-2.72) | 0.15 | . | . |
| DPYD - tag |  | 2 | 2 | 3,45 | 0 | 0,00 | 23 | 6,10 | 16 | 5,93 | 4.21 (0.96-18.40) | 0.06 | 1.30 (0.67-2.55) | 0.44 | . | . |
| DPYD - tag | rs10783058 | 0 | 30 | 51,72 | 18 | 52,94 | 153 | 40,58 | 100 | 37,04 | 1.00 (.-.) | . | 1.02 (0.58-1.79) | 0.94 | 0.59 | 0.29 |
| DPYD - tag |  | 1 | 22 | 37,93 | 11 | 32,35 | 185 | 49,07 | 127 | 47,04 | 0.88 (0.52-1.52) | 0.66 | 1.18 (0.69-2.05) | 0.54 | . | . |
| DPYD - tag |  | 2 | 6 | 10,34 | 5 | 14,71 | 39 | 10,34 | 43 | 15,93 | 0.78 (0.27-2.30) | 0.66 | 1.37 (0.76-2.48) | 0.29 | . | . |
| DPYD - tag | rs10783070 | 0 | 43 | 74,14 | 27 | 79,41 | 273 | 72,41 | 184 | 68,15 | 1.00 (.-.) | . | 1.13 (0.71-1.79) | 0.61 | 0.83 | 0.51 |
| DPYD - tag |  | 1 | 13 | 22,41 | 6 | 17,65 | 97 | 25,73 | 78 | 28,89 | 0.94 (0.48-1.83) | 0.86 | 1.45 (0.90-2.34) | 0.13 | . | . |
| DPYD - tag |  | 2 | 2 | 3,45 | 1 | 2,94 | 7 | 1,86 | 8 | 2,96 | 0.88 (0.23-3.35) | 0.86 | 1.87 (1.02-3.42) | 0.04 | . | . |
| DPYD - tag | rs10875048 | 0 | 40 | 68,97 | 23 | 67,65 | 262 | 69,50 | 180 | 66,67 | 1.00 (.-.) | . | 1.07 (0.65-1.75) | 0.80 | 0.93 | 0.63 |
| DPYD - tag |  | 1 | 13 | 22,41 | 10 | 29,41 | 110 | 29,18 | 80 | 29,63 | 0.73 (0.40-1.35) | 0.32 | 1.07 (0.65-1.78) | 0.78 | . | . |
| DPYD - tag |  | 2 | 5 | 8,62 | 1 | 2,94 | 5 | 1,33 | 10 | 3,70 | 0.54 (0.16-1.82) | 0.32 | 1.08 (0.58-2.02) | 0.81 | . | . |
| DPYD - tag | rs10875055 | 0 | 31 | 53,45 | 6 | 17,65 | 109 | 28,91 | 51 | 18,89 | 1.00 (.-.) | . | 1.35 (0.68-2.67) | 0.39 | 0.59 | 0.29 |
| DPYD - tag |  | 1 | 20 | 34,48 | 22 | 64,71 | 195 | 51,72 | 144 | 53,33 | 1.65 (0.95-2.87) | 0.08 | 1.86 (0.97-3.58) | 0.06 | . | . |
| DPYD - tag |  | 2 | 7 | 12,07 | 6 | 17,65 | 73 | 19,36 | 75 | 27,78 | 2.72 (0.90-8.22) | 0.08 | 2.56 (1.30-5.05) | 0.01 | . | . |
| DPYD - tag | rs10875079 | 0 | 10 | 17,24 | 9 | 26,47 | 100 | 26,53 | 78 | 28,89 | 1.00 (.-.) | . | 1.41 (0.67-2.98) | 0.37 | 0.99 | 0.81 |
| DPYD - tag |  | 1 | 37 | 63,79 | 17 | 50,00 | 184 | 48,81 | 130 | 48,15 | 1.04 (0.60-1.81) | 0.89 | 1.29 (0.62-2.68) | 0.49 | . | . |
| DPYD - tag |  | 2 | 11 | 18,97 | 8 | 23,53 | 93 | 24,67 | 62 | 22,96 | 1.08 (0.36-3.28) | 0.89 | 1.19 (0.56-2.51) | 0.66 | . | . |
| DPYD - tag | rs10875085 | 0 | 39 | 67,24 | 24 | 70,59 | 264 | 70,03 | 181 | 67,04 | 1.00 (.-.) | . | 1.19 (0.73-1.92) | 0.48 | 0.93 | 0.69 |
| DPYD - tag |  | 1 | 17 | 29,31 | 9 | 26,47 | 103 | 27,32 | 79 | 29,26 | 0.76 (0.34-1.72) | 0.52 | 1.07 (0.65-1.76) | 0.78 | . | . |
| DPYD - tag |  | 2 | 2 | 3,45 | 1 | 2,94 | 10 | 2,65 | 10 | 3,70 | 0.58 (0.12-2.97) | 0.52 | 0.97 (0.53-1.78) | 0.92 | . | . |
| DPYD - tag | rs10875097 | 0 | 42 | 72,41 | 20 | 58,82 | 260 | 68,97 | 179 | 66,30 | 1.00 (.-.) | . | 1.75 (1.01-3.04) | 0.05 | 0.59 | 0.51 |
| DPYD - tag |  | 1 | 16 | 27,59 | 13 | 38,24 | 103 | 27,32 | 82 | 30,37 | 2.37 (1.20-4.66) | 0.01 | 1.81 (1.02-3.19) | 0.04 | . | . |
| DPYD - tag |  | 2 | 0 | 0,00 | 1 | 2,94 | 14 | 3,71 | 9 | 3,33 | 5.61 (1.45-21.75) | 0.01 | 1.86 (0.94-3.67) | 0.07 | . | . |
| DPYD - tag | rs11165781 | 0 | 40 | 68,97 | 25 | 73,53 | 264 | 70,03 | 177 | 65,56 | 1.00 (.-.) | . | 1.11 (0.70-1.76) | 0.66 | 0.99 | 0.81 |
| DPYD - tag |  | 1 | 17 | 29,31 | 8 | 23,53 | 106 | 28,12 | 86 | 31,85 | 0.80 (0.37-1.75) | 0.58 | 1.30 (0.81-2.10) | 0.28 | . | . |
| DPYD - tag |  | 2 | 1 | 1,72 | 1 | 2,94 | 7 | 1,86 | 7 | 2,59 | 0.64 (0.14-3.05) | 0.58 | 1.52 (0.82-2.81) | 0.18 | . | . |
| DPYD - tag | rs11165783 | 0 | 32 | 55,17 | 19 | 55,88 | 207 | 54,91 | 148 | 54,81 | 1.00 (.-.) | . | 1.52 (0.87-2.68) | 0.14 | 0.83 | 0.51 |
| DPYD - tag |  | 1 | 23 | 39,66 | 13 | 38,24 | 138 | 36,60 | 99 | 36,67 | 1.62 (0.84-3.13) | 0.15 | 1.62 (0.92-2.85) | 0.09 | . | . |
| DPYD - tag |  | 2 | 3 | 5,17 | 2 | 5,88 | 32 | 8,49 | 23 | 8,52 | 2.62 (0.70-9.77) | 0.15 | 1.73 (0.92-3.24) | 0.09 | . | . |
| DPYD - tag | rs11165873 | 0 | 14 | 24,14 | 7 | 20,59 | 105 | 27,85 | 82 | 30,37 | 1.00 (.-.) | . | 1.46 (0.67-3.19) | 0.34 | 0.83 | 0.51 |
| DPYD - tag |  | 1 | 28 | 48,28 | 16 | 47,06 | 197 | 52,25 | 130 | 48,15 | 1.12 (0.65-1.93) | 0.68 | 1.41 (0.65-3.02) | 0.38 | . | . |
| DPYD - tag |  | 2 | 16 | 27,59 | 11 | 32,35 | 75 | 19,89 | 58 | 21,48 | 1.25 (0.42-3.73) | 0.68 | 1.35 (0.61-2.99) | 0.46 | . | . |
| DPYD - tag | rs11165875 | 0 | 24 | 41,38 | 16 | 47,06 | 149 | 39,52 | 109 | 40,37 | 1.00 (.-.) | . | 1.35 (0.77-2.38) | 0.30 | 0.85 | 0.51 |
| DPYD - tag |  | 1 | 29 | 50,00 | 12 | 35,29 | 183 | 48,54 | 118 | 43,70 | 1.10 (0.63-1.93) | 0.74 | 1.31 (0.76-2.26) | 0.34 | . | . |
| DPYD - tag |  | 2 | 5 | 8,62 | 6 | 17,65 | 45 | 11,94 | 43 | 15,93 | 1.21 (0.39-3.71) | 0.74 | 1.27 (0.70-2.30) | 0.43 | . | . |
| DPYD - tag | rs11165881 | 0 | 22 | 37,93 | 17 | 50,00 | 129 | 34,22 | 90 | 33,33 | 1.00 (.-.) | . | 1.00 (0.55-1.84) | 1.00 | 0.94 | 0.71 |
| DPYD - tag |  | 1 | 25 | 43,10 | 14 | 41,18 | 174 | 46,15 | 134 | 49,63 | 0.88 (0.48-1.64) | 0.69 | 1.18 (0.66-2.12) | 0.58 | . | . |
| DPYD - tag |  | 2 | 11 | 18,97 | 3 | 8,82 | 74 | 19,63 | 46 | 17,04 | 0.78 (0.23-2.67) | 0.69 | 1.39 (0.75-2.58) | 0.30 | . | . |
| DPYD - tag | rs11587873 | 0 | 34 | 58,62 | 21 | 61,76 | 202 | 53,58 | 172 | 63,70 | 1.00 (.-.) | . | 1.24 (0.73-2.11) | 0.42 | 0.85 | 0.51 |
| DPYD - tag |  | 1 | 20 | 34,48 | 12 | 35,29 | 144 | 38,20 | 90 | 33,33 | 0.79 (0.39-1.58) | 0.50 | 0.95 (0.55-1.63) | 0.85 | . | . |
| DPYD - tag |  | 2 | 4 | 6,90 | 1 | 2,94 | 31 | 8,22 | 8 | 2,96 | 0.62 (0.16-2.50) | 0.50 | 0.72 (0.38-1.37) | 0.32 | . | . |
| DPYD - tag | rs12030174 | 0 | 42 | 72,41 | 24 | 70,59 | 278 | 73,74 | 193 | 71,48 | 1.00 (.-.) | . | 1.23 (0.77-1.99) | 0.39 | 0.92 | 0.61 |
| DPYD - tag |  | 1 | 15 | 25,86 | 9 | 26,47 | 90 | 23,87 | 70 | 25,93 | 0.81 (0.36-1.83) | 0.62 | 1.04 (0.63-1.72) | 0.89 | . | . |
| DPYD - tag |  | 2 | 1 | 1,72 | 1 | 2,94 | 9 | 2,39 | 7 | 2,59 | 0.66 (0.13-3.33) | 0.62 | 0.87 (0.46-1.66) | 0.68 | . | . |
| DPYD - tag | rs12046744 | 0 | 30 | 51,72 | 19 | 55,88 | 205 | 54,38 | 152 | 56,30 | 1.00 (.-.) | . | 1.38 (0.83-2.30) | 0.22 | 0.99 | 0.92 |
| DPYD - tag |  | 1 | 24 | 41,38 | 12 | 35,29 | 138 | 36,60 | 101 | 37,41 | 1.23 (0.69-2.20) | 0.48 | 1.36 (0.81-2.26) | 0.24 | . | . |
| DPYD - tag |  | 2 | 4 | 6,90 | 3 | 8,82 | 34 | 9,02 | 17 | 6,30 | 1.52 (0.48-4.84) | 0.48 | 1.33 (0.75-2.39) | 0.33 | . | . |
| DPYD - tag | rs12047910 | 0 | 43 | 74,14 | 24 | 70,59 | 279 | 74,01 | 202 | 74,81 | 1.00 (.-.) | . | 1.38 (0.83-2.30) | 0.21 | 0.85 | 0.51 |
| DPYD - tag |  | 1 | 14 | 24,14 | 9 | 26,47 | 95 | 25,20 | 61 | 22,59 | 1.29 (0.62-2.67) | 0.50 | 1.30 (0.76-2.22) | 0.33 | . | . |
| DPYD - tag |  | 2 | 1 | 1,72 | 1 | 2,94 | 3 | 0,80 | 7 | 2,59 | 1.65 (0.38-7.13) | 0.50 | 1.23 (0.62-2.42) | 0.56 | . | . |
| DPYD - tag | rs12073044 | 0 | 50 | 86,21 | 29 | 85,29 | 285 | 75,60 | 216 | 80,00 | 1.00 (.-.) | . | 1.33 (0.85-2.09) | 0.21 | 0.85 | 0.51 |
| DPYD - tag |  | 1 | 8 | 13,79 | 5 | 14,71 | 88 | 23,34 | 53 | 19,63 | 1.03 (0.35-3.02) | 0.96 | 0.98 (0.58-1.64) | 0.93 | . | . |
| DPYD - tag |  | 2 | 0 | 0,00 | 0 | 0,00 | 4 | 1,06 | 1 | 0,37 | 1.05 (0.12-9.14) | 0.96 | 0.72 (0.34-1.49) | 0.38 | . | . |
| DPYD - tag | rs12126093 | 0 | 25 | 43,10 | 12 | 35,29 | 194 | 51,46 | 144 | 53,33 | 1.00 (.-.) | . | 1.27 (0.68-2.38) | 0.45 | 0.93 | 0.62 |
| DPYD - tag |  | 1 | 28 | 48,28 | 17 | 50,00 | 158 | 41,91 | 101 | 37,41 | 0.98 (0.55-1.75) | 0.95 | 1.19 (0.64-2.20) | 0.59 | . | . |
| DPYD - tag |  | 2 | 5 | 8,62 | 5 | 14,71 | 25 | 6,63 | 25 | 9,26 | 0.96 (0.30-3.07) | 0.95 | 1.10 (0.56-2.18) | 0.78 | . | . |
| DPYD - tag | rs12134028 | 0 | 52 | 89,66 | 29 | 85,29 | 344 | 91,25 | 238 | 88,15 | 1.00 (.-.) | . | 1.12 (0.72-1.74) | 0.61 | 0.83 | 0.51 |
| DPYD - tag |  | 1 | 6 | 10,34 | 4 | 11,76 | 33 | 8,75 | 31 | 11,48 | 0.51 (0.15-1.72) | 0.28 | 1.11 (0.64-1.92) | 0.72 | . | . |
| DPYD - tag |  | 2 | 0 | 0,00 | 1 | 2,94 | 0 | 0,00 | 1 | 0,37 | 0.26 (0.02-2.96) | 0.28 | 1.09 (0.46-2.56) | 0.84 | . | . |
| DPYD - tag | rs12740796 | 0 | 37 | 63,79 | 28 | 82,35 | 278 | 73,74 | 210 | 77,78 | 1.00 (.-.) | . | 1.21 (0.78-1.89) | 0.40 | 0.99 | 0.85 |
| DPYD - tag |  | 1 | 19 | 32,76 | 5 | 14,71 | 92 | 24,40 | 55 | 20,37 | 0.80 (0.34-1.92) | 0.62 | 1.13 (0.70-1.84) | 0.61 | . | . |
| DPYD - tag |  | 2 | 2 | 3,45 | 1 | 2,94 | 7 | 1,86 | 5 | 1,85 | 0.65 (0.11-3.70) | 0.62 | 1.06 (0.56-2.03) | 0.86 | . | . |
| DPYD - tag | rs1333717 | 0 | 37 | 63,79 | 18 | 52,94 | 202 | 53,58 | 163 | 60,37 | 1.00 (.-.) | . | 1.79 (1.02-3.14) | 0.04 | 0.87 | 0.53 |
| DPYD - tag |  | 1 | 18 | 31,03 | 16 | 47,06 | 151 | 40,05 | 90 | 33,33 | 2.00 (0.96-4.19) | 0.07 | 1.55 (0.87-2.74) | 0.14 | . | . |
| DPYD - tag |  | 2 | 3 | 5,17 | 0 | 0,00 | 24 | 6,37 | 17 | 6,30 | 4.02 (0.92-17.60) | 0.07 | 1.34 (0.69-2.59) | 0.38 | . | . |
| DPYD - tag | rs1413228 | 0 | 46 | 79,31 | 31 | 91,18 | 306 | 81,17 | 206 | 76,30 | 1.00 (.-.) | . | 1.09 (0.71-1.69) | 0.69 | 0.99 | 0.93 |
| DPYD - tag |  | 1 | 12 | 20,69 | 2 | 5,88 | 66 | 17,51 | 60 | 22,22 | 0.56 (0.19-1.63) | 0.29 | 1.30 (0.80-2.12) | 0.29 | . | . |
| DPYD - tag |  | 2 | 0 | 0,00 | 1 | 2,94 | 5 | 1,33 | 4 | 1,48 | 0.31 (0.04-2.65) | 0.29 | 1.55 (0.80-3.02) | 0.20 | . | . |
| DPYD - tag | rs1415681 | 0 | 43 | 74,14 | 29 | 85,29 | 270 | 71,62 | 202 | 74,81 | 1.00 (.-.) | . | 1.09 (0.69-1.72) | 0.71 | 0.97 | 0.78 |
| DPYD - tag |  | 1 | 14 | 24,14 | 5 | 14,71 | 99 | 26,26 | 59 | 21,85 | 0.64 (0.22-1.87) | 0.41 | 1.26 (0.79-2.03) | 0.33 | . | . |
| DPYD - tag |  | 2 | 1 | 1,72 | 0 | 0,00 | 8 | 2,12 | 9 | 3,33 | 0.41 (0.05-3.48) | 0.41 | 1.47 (0.80-2.70) | 0.22 | . | . |
| DPYD - tag | rs1514495 | 0 | 31 | 53,45 | 19 | 55,88 | 238 | 63,13 | 158 | 58,52 | 1.00 (.-.) | . | 1.09 (0.66-1.82) | 0.73 | 0.99 | 0.89 |
| DPYD - tag |  | 1 | 20 | 34,48 | 13 | 38,24 | 120 | 31,83 | 93 | 34,44 | 0.80 (0.46-1.40) | 0.44 | 1.10 (0.66-1.85) | 0.70 | . | . |
| DPYD - tag |  | 2 | 7 | 12,07 | 2 | 5,88 | 19 | 5,04 | 19 | 7,04 | 0.64 (0.21-1.96) | 0.44 | 1.12 (0.61-2.04) | 0.72 | . | . |
| DPYD - tag | rs1520658 | 0 | 41 | 70,69 | 30 | 88,24 | 307 | 81,43 | 224 | 82,96 | 1.00 (.-.) | . | 1.11 (0.72-1.71) | 0.64 | 0.86 | 0.53 |
| DPYD - tag |  | 1 | 16 | 27,59 | 4 | 11,76 | 63 | 16,71 | 44 | 16,30 | 0.37 (0.09-1.52) | 0.17 | 1.09 (0.67-1.77) | 0.73 | . | . |
| DPYD - tag |  | 2 | 1 | 1,72 | 0 | 0,00 | 7 | 1,86 | 2 | 0,74 | 0.14 (0.01-2.32) | 0.17 | 1.07 (0.54-2.13) | 0.85 | . | . |
| DPYD - NA | rs17116806 | 0 | 36 | 62,07 | 19 | 55,88 | 249 | 66,05 | 172 | 63,70 | 1.00 (.-.) | . | 1.71 (0.94-3.09) | 0.08 | 0.83 | 0.51 |
| DPYD - NA |  | 1 | 22 | 37,93 | 13 | 38,24 | 109 | 28,91 | 86 | 31,85 | 1.89 (0.95-3.74) | 0.07 | 1.77 (0.97-3.24) | 0.06 | . | . |
| DPYD - NA |  | 2 | 0 | 0,00 | 2 | 5,88 | 19 | 5,04 | 12 | 4,44 | 3.56 (0.90-14.02) | 0.07 | 1.84 (0.92-3.68) | 0.09 | . | . |
| DPYD - tag | rs17431828 | 0 | 26 | 44,83 | 12 | 35,29 | 147 | 38,99 | 122 | 45,19 | 1.00 (.-.) | . | 1.44 (0.79-2.62) | 0.23 | 0.99 | 0.95 |
| DPYD - tag |  | 1 | 24 | 41,38 | 17 | 50,00 | 181 | 48,01 | 114 | 42,22 | 1.15 (0.68-1.95) | 0.61 | 1.34 (0.74-2.43) | 0.33 | . | . |
| DPYD - tag |  | 2 | 8 | 13,79 | 5 | 14,71 | 49 | 13,00 | 34 | 12,59 | 1.32 (0.46-3.81) | 0.61 | 1.25 (0.66-2.40) | 0.49 | . | . |
| DPYD - tag | rs17471640 | 0 | 23 | 39,66 | 12 | 35,29 | 160 | 42,44 | 133 | 49,26 | 1.00 (.-.) | . | 1.51 (0.83-2.75) | 0.18 | 0.85 | 0.51 |
| DPYD - tag |  | 1 | 27 | 46,55 | 17 | 50,00 | 186 | 49,34 | 111 | 41,11 | 1.17 (0.69-1.99) | 0.56 | 1.33 (0.73-2.40) | 0.35 | . | . |
| DPYD - tag |  | 2 | 8 | 13,79 | 5 | 14,71 | 31 | 8,22 | 26 | 9,63 | 1.37 (0.48-3.94) | 0.56 | 1.17 (0.60-2.25) | 0.65 | . | . |
| DPYD - tag | rs17702702 | 0 | 34 | 58,62 | 25 | 73,53 | 259 | 68,70 | 193 | 71,48 | 1.00 (.-.) | . | 1.32 (0.83-2.11) | 0.24 | 0.99 | 0.81 |
| DPYD - tag |  | 1 | 20 | 34,48 | 8 | 23,53 | 107 | 28,38 | 68 | 25,19 | 1.02 (0.51-2.06) | 0.96 | 1.14 (0.71-1.85) | 0.59 | . | . |
| DPYD - tag |  | 2 | 4 | 6,90 | 1 | 2,94 | 11 | 2,92 | 9 | 3,33 | 1.04 (0.26-4.24) | 0.96 | 0.99 (0.54-1.81) | 0.97 | . | . |
| DPYD - NA | rs1801265 | 0 | 34 | 58,62 | 22 | 64,71 | 211 | 55,97 | 163 | 60,37 | 1.00 (.-.) | . | 1.41 (0.84-2.34) | 0.19 | 0.99 | 0.95 |
| DPYD - NA |  | 1 | 21 | 36,21 | 12 | 35,29 | 139 | 36,87 | 94 | 34,81 | 1.18 (0.56-2.48) | 0.67 | 1.22 (0.73-2.04) | 0.44 | . | . |
| DPYD - NA |  | 2 | 3 | 5,17 | 0 | 0,00 | 27 | 7,16 | 13 | 4,81 | 1.39 (0.31-6.15) | 0.67 | 1.06 (0.58-1.95) | 0.84 | . | . |
| DPYD - tag | rs2039447 | 0 | 23 | 39,66 | 22 | 64,71 | 176 | 46,68 | 134 | 49,63 | 1.00 (.-.) | . | 1.17 (0.72-1.89) | 0.54 | 0.88 | 0.56 |
| DPYD - tag |  | 1 | 24 | 41,38 | 8 | 23,53 | 164 | 43,50 | 115 | 42,59 | 0.75 (0.45-1.26) | 0.28 | 1.01 (0.63-1.64) | 0.95 | . | . |
| DPYD - tag |  | 2 | 11 | 18,97 | 4 | 11,76 | 37 | 9,81 | 21 | 7,78 | 0.56 (0.20-1.58) | 0.28 | 0.88 (0.51-1.54) | 0.66 | . | . |
| DPYD - tag | rs2151567 | 0 | 50 | 86,21 | 33 | 97,06 | 343 | 90,98 | 243 | 90,00 | 1.00 (.-.) | . | 1.22 (0.80-1.86) | 0.36 | 0.85 | 0.51 |
| DPYD - tag |  | 1 | 8 | 13,79 | 1 | 2,94 | 33 | 8,75 | 26 | 9,63 | 0.83 (0.11-6.15) | 0.85 | 1.40 (0.79-2.48) | 0.25 | . | . |
| DPYD - tag |  | 2 | 0 | 0,00 | 0 | 0,00 | 1 | 0,27 | 1 | 0,37 | 0.68 (0.01-37.87) | 0.85 | 1.61 (0.64-4.03) | 0.31 | . | . |
| DPYD - tag | rs2152878 | 0 | 30 | 51,72 | 21 | 61,76 | 214 | 56,76 | 156 | 57,78 | 1.00 (.-.) | . | 1.12 (0.67-1.89) | 0.66 | 0.99 | 0.94 |
| DPYD - tag |  | 1 | 25 | 43,10 | 8 | 23,53 | 141 | 37,40 | 96 | 35,56 | 0.93 (0.51-1.72) | 0.82 | 1.31 (0.78-2.23) | 0.31 | . | . |
| DPYD - tag |  | 2 | 3 | 5,17 | 5 | 14,71 | 22 | 5,84 | 18 | 6,67 | 0.87 (0.26-2.96) | 0.82 | 1.53 (0.83-2.84) | 0.17 | . | . |
| DPYD - tag | rs2786505 | 0 | 45 | 77,59 | 28 | 82,35 | 285 | 75,60 | 195 | 72,22 | 1.00 (.-.) | . | 1.12 (0.70-1.78) | 0.64 | 0.82 | 0.51 |
| DPYD - tag |  | 1 | 13 | 22,41 | 6 | 17,65 | 90 | 23,87 | 69 | 25,56 | 0.88 (0.35-2.20) | 0.79 | 1.47 (0.90-2.40) | 0.13 | . | . |
| DPYD - tag |  | 2 | 0 | 0,00 | 0 | 0,00 | 2 | 0,53 | 6 | 2,22 | 0.78 (0.12-4.85) | 0.79 | 1.93 (1.01-3.66) | 0.05 | . | . |
[truncated: 120,417 more chars]
