# Supplementary material for: Pathway analysis of genetic variants in folate‐mediated one‐carbon metabolism‐related genes and survival in a prospectively followed cohort of colorectal cancer patients
Source: Cancer Med. 2018 May 29;7(7):2797–807. doi: 10.1002/cam4.1407 (PMC6051204; doi:10.1002/cam4.1407)
Supplement: Supplementary file 8 — Table S10. Associations between polymorphisms in FOCM‐related genes and disease‐free survival stratified by 5‐FU‐based chemotherapy*. [file CAM4-7-2797-s008.docx]

| **Supplementary Table 10. Associations between polymorphisms in FOCM-related genes and disease-free survival stratified by 5-FU-based chemotherapy*** | | | | | | | | | | | | | | | | | |
| --- | --- | --- | --- | --- | --- | --- | --- | --- | --- | --- | --- | --- | --- | --- | --- | --- | --- |
|  | | | **adchem_5fu = 0** | | | | **adchem_5fu = 1** | | | | **one reference** | | | | | | |
|  | | | **Ctrl** | | **Cases** | | **Ctrl** | | **Cases** | | **adchem_5fu = 0** | | **adchem_5fu = 1** | |  | | |
| **Gene** | **SNP** | **Genotype** | **N** | **%** | **N** | **%** | **N** | **%** | **N** | **%** | **HR (95%-CI)** | **p** | **HR (95%-CI)** | **p** | **LR_pInt** | **FDR_pInt** | **FDR(byGene)_pInt** |
| AARS ---tag | rs2070203 | T/T | 17 | 28,33 | 11 | 34,38 | 104 | 30,50 | 84 | 27,45 | 1.00 (.-.) | . | 0.93 (0.44-1.94) | 0.84 | 0.23 | 0.97 | 0.23 |
| AARS ---tag |  | T/C or C/C | 43 | 71,67 | 21 | 65,63 | 237 | 69,50 | 222 | 72,55 | 0.69 (0.30-1.59) | 0.38 | 1.11 (0.55-2.25) | 0.77 | . | . | . |
| AARS ---tag | rs34087264 | G/G | 21 | 35,00 | 7 | 21,88 | 86 | 25,22 | 95 | 31,05 | 1.00 (.-.) | . | 2.79 (1.11-6.99) | 0.03 | 0.05 | 0.94 | 0.10 |
| AARS ---tag |  | G/A or A/A | 39 | 65,00 | 25 | 78,13 | 255 | 74,78 | 211 | 68,95 | 2.00 (0.73-5.47) | 0.18 | 2.09 (0.85-5.16) | 0.11 | . | . | . |
| ABCC4 ---tag | rs10508023 | G/G | 49 | 81,67 | 25 | 78,13 | 267 | 78,30 | 250 | 81,70 | 1.00 (.-.) | . | 1.57 (0.98-2.53) | 0.06 | 0.18 | 0.97 | 0.89 |
| ABCC4 ---tag |  | G/C or C/C | 11 | 18,33 | 7 | 21,88 | 74 | 21,70 | 56 | 18,30 | 1.65 (0.60-4.52) | 0.33 | 1.19 (0.69-2.04) | 0.53 | . | . | . |
| ABCC4 ---tag | rs1059751 | T/T | 20 | 33,33 | 8 | 25,00 | 81 | 23,75 | 78 | 25,49 | 1.00 (.-.) | . | 1.53 (0.69-3.39) | 0.29 | 0.74 | 0.97 | 0.95 |
| ABCC4 ---tag |  | T/C or C/C | 40 | 66,67 | 24 | 75,00 | 260 | 76,25 | 228 | 74,51 | 1.19 (0.49-2.90) | 0.70 | 1.56 (0.72-3.36) | 0.26 | . | . | . |
| ABCC4 ---tag | rs11568643 | A/A | 50 | 83,33 | 26 | 81,25 | 286 | 83,87 | 256 | 83,66 | 1.00 (.-.) | . | 1.47 (0.92-2.33) | 0.11 | 0.44 | 0.97 | 0.95 |
| ABCC4 ---tag |  | A/G or G/G | 10 | 16,67 | 6 | 18,75 | 55 | 16,13 | 50 | 16,34 | 1.34 (0.45-3.97) | 0.59 | 1.23 (0.70-2.15) | 0.48 | . | . | . |
| ABCC4 ---NA | rs11568658 | G/G | 56 | 93,33 | 31 | 96,88 | 321 | 94,13 | 293 | 95,75 | 1.00 (.-.) | . | 1.37 (0.89-2.13) | 0.16 | 0.96 | 1.00 | 0.99 |
| ABCC4 ---NA |  | G/T or T/T | 4 | 6,67 | 1 | 3,13 | 20 | 5,87 | 13 | 4,25 | 1.15 (0.15-8.61) | 0.89 | 1.51 (0.73-3.13) | 0.27 | . | . | . |
| ABCC4 ---tag | rs12864049 | T/T | 42 | 70,00 | 22 | 68,75 | 266 | 78,01 | 226 | 73,86 | 1.00 (.-.) | . | 1.29 (0.76-2.18) | 0.35 | 0.61 | 0.97 | 0.95 |
| ABCC4 ---tag |  | T/C or C/C | 18 | 30,00 | 10 | 31,25 | 75 | 21,99 | 80 | 26,14 | 1.05 (0.46-2.40) | 0.91 | 1.69 (0.97-2.96) | 0.06 | . | . | . |
| ABCC4 ---tag | rs1628382 | G/G | 38 | 63,33 | 23 | 71,88 | 209 | 61,29 | 189 | 61,76 | 1.00 (.-.) | . | 1.39 (0.84-2.32) | 0.20 | 0.90 | 1.00 | 0.99 |
| ABCC4 ---tag |  | G/A or A/A | 22 | 36,67 | 9 | 28,13 | 132 | 38,71 | 117 | 38,24 | 1.08 (0.44-2.64) | 0.86 | 1.42 (0.84-2.42) | 0.19 | . | . | . |
| ABCC4 ---tag | rs1678354 | C/C | 20 | 33,33 | 12 | 37,50 | 153 | 44,87 | 126 | 41,18 | 1.00 (.-.) | . | 1.21 (0.60-2.46) | 0.59 | 0.63 | 0.97 | 0.95 |
| ABCC4 ---tag |  | C/G or G/G | 40 | 66,67 | 20 | 62,50 | 188 | 55,13 | 180 | 58,82 | 1.05 (0.46-2.42) | 0.90 | 1.59 (0.79-3.18) | 0.19 | . | . | . |
| ABCC4 ---tag | rs1678383 | T/T | 48 | 80,00 | 27 | 84,38 | 282 | 82,70 | 249 | 81,37 | 1.00 (.-.) | . | 1.10 (0.69-1.75) | 0.68 | 0.05 | 0.94 | 0.70 |
| ABCC4 ---tag |  | T/G or G/G | 12 | 20,00 | 5 | 15,63 | 59 | 17,30 | 57 | 18,63 | 0.35 (0.10-1.20) | 0.10 | 1.21 (0.71-2.06) | 0.48 | . | . | . |
| ABCC4 ---tag | rs1678395 | G/G | 51 | 85,00 | 26 | 81,25 | 285 | 83,58 | 262 | 85,62 | 1.00 (.-.) | . | 1.51 (0.95-2.38) | 0.08 | 0.60 | 0.97 | 0.95 |
| ABCC4 ---tag |  | G/A or A/A | 9 | 15,00 | 6 | 18,75 | 56 | 16,42 | 44 | 14,38 | 0.87 (0.26-2.97) | 0.83 | 0.92 (0.53-1.58) | 0.75 | . | . | . |
| ABCC4 ---tag | rs1678405 | T/T | 24 | 40,00 | 21 | 65,63 | 156 | 45,75 | 132 | 43,14 | 1.00 (.-.) | . | 1.10 (0.64-1.88) | 0.73 | 0.24 | 0.97 | 0.89 |
| ABCC4 ---tag |  | T/C or C/C | 36 | 60,00 | 11 | 34,38 | 185 | 54,25 | 174 | 56,86 | 0.73 (0.31-1.74) | 0.48 | 1.38 (0.81-2.33) | 0.24 | . | . | . |
| ABCC4 ---tag | rs17189540 | A/A | 53 | 88,33 | 26 | 81,25 | 303 | 88,86 | 260 | 84,97 | 1.00 (.-.) | . | 1.42 (0.88-2.27) | 0.15 | 0.76 | 0.97 | 0.95 |
| ABCC4 ---tag |  | A/G or G/G | 7 | 11,67 | 6 | 18,75 | 38 | 11,14 | 46 | 15,03 | 1.28 (0.46-3.52) | 0.63 | 1.52 (0.86-2.70) | 0.15 | . | . | . |
| ABCC4 ---tag | rs17235152 | T/T | 41 | 68,33 | 24 | 75,00 | 247 | 72,43 | 226 | 73,86 | 1.00 (.-.) | . | 1.35 (0.80-2.26) | 0.26 | 0.87 | 0.99 | 0.99 |
| ABCC4 ---tag |  | T/C or C/C | 19 | 31,67 | 8 | 25,00 | 94 | 27,57 | 80 | 26,14 | 0.97 (0.41-2.30) | 0.95 | 1.42 (0.80-2.50) | 0.23 | . | . | . |
| ABCC4 ---tag | rs17268122 | G/G | 28 | 46,67 | 18 | 56,25 | 214 | 62,76 | 206 | 67,32 | 1.00 (.-.) | . | 1.13 (0.63-2.02) | 0.68 | 0.48 | 0.97 | 0.95 |
| ABCC4 ---tag |  | G/T or T/T | 32 | 53,33 | 14 | 43,75 | 127 | 37,24 | 100 | 32,68 | 0.62 (0.28-1.41) | 0.26 | 0.96 (0.52-1.77) | 0.90 | . | . | . |
| ABCC4 ---tag | rs17268170 | C/C | 47 | 78,33 | 24 | 75,00 | 284 | 83,28 | 246 | 80,39 | 1.00 (.-.) | . | 1.46 (0.89-2.40) | 0.13 | 0.59 | 0.97 | 0.95 |
| ABCC4 ---tag |  | C/T or T/T | 13 | 21,67 | 8 | 25,00 | 57 | 16,72 | 60 | 19,61 | 1.25 (0.51-3.07) | 0.62 | 1.40 (0.80-2.44) | 0.24 | . | . | . |
| ABCC4 ---tag | rs1729764 | A/A | 44 | 73,33 | 27 | 84,38 | 259 | 75,95 | 246 | 80,39 | 1.00 (.-.) | . | 1.34 (0.85-2.10) | 0.21 | 0.63 | 0.97 | 0.95 |
| ABCC4 ---tag |  | A/G or G/G | 16 | 26,67 | 5 | 15,63 | 82 | 24,05 | 60 | 19,61 | 0.66 (0.15-2.84) | 0.58 | 1.25 (0.74-2.10) | 0.40 | . | . | . |
| ABCC4 ---tag | rs1729767 | T/T | 29 | 48,33 | 14 | 43,75 | 183 | 53,67 | 156 | 50,98 | 1.00 (.-.) | . | 1.55 (0.83-2.90) | 0.17 | 0.60 | 0.97 | 0.95 |
| ABCC4 ---tag |  | T/C or C/C | 31 | 51,67 | 18 | 56,25 | 158 | 46,33 | 150 | 49,02 | 1.45 (0.65-3.24) | 0.37 | 1.79 (0.95-3.35) | 0.07 | . | . | . |
| ABCC4 ---tag | rs17300935 | C/C | 40 | 66,67 | 21 | 65,63 | 252 | 73,90 | 233 | 76,14 | 1.00 (.-.) | . | 1.35 (0.79-2.30) | 0.27 | 0.87 | 0.99 | 0.99 |
| ABCC4 ---tag |  | C/G or G/G | 20 | 33,33 | 11 | 34,38 | 89 | 26,10 | 73 | 23,86 | 0.99 (0.43-2.28) | 0.98 | 1.44 (0.81-2.56) | 0.22 | . | . | . |
| ABCC4 ---tag | rs1750190 | G/G | 18 | 30,00 | 8 | 25,00 | 91 | 26,69 | 72 | 23,53 | 1.00 (.-.) | . | 1.50 (0.63-3.55) | 0.36 | 0.75 | 0.97 | 0.95 |
| ABCC4 ---tag |  | G/A or A/A | 42 | 70,00 | 24 | 75,00 | 250 | 73,31 | 234 | 76,47 | 1.54 (0.60-3.97) | 0.37 | 1.97 (0.86-4.51) | 0.11 | . | . | . |
| ABCC4 ---tag | rs1750996 | A/A | 33 | 55,00 | 21 | 65,63 | 224 | 65,69 | 201 | 65,69 | 1.00 (.-.) | . | 1.31 (0.78-2.19) | 0.31 | 0.74 | 0.97 | 0.95 |
| ABCC4 ---tag |  | A/G or G/G | 27 | 45,00 | 11 | 34,38 | 117 | 34,31 | 105 | 34,31 | 0.98 (0.42-2.31) | 0.96 | 1.49 (0.87-2.56) | 0.15 | . | . | . |
| ABCC4 ---tag | rs1751025 | C/C | 27 | 45,00 | 18 | 56,25 | 167 | 48,97 | 134 | 43,79 | 1.00 (.-.) | . | 1.35 (0.77-2.40) | 0.30 | 1.00 | 1.00 | 1.00 |
| ABCC4 ---tag |  | C/G or G/G | 33 | 55,00 | 14 | 43,75 | 174 | 51,03 | 172 | 56,21 | 1.13 (0.50-2.54) | 0.77 | 1.52 (0.87-2.68) | 0.14 | . | . | . |
| ABCC4 ---tag | rs1751051 | T/T | 26 | 43,33 | 14 | 43,75 | 136 | 39,88 | 136 | 44,44 | 1.00 (.-.) | . | 1.40 (0.77-2.54) | 0.27 | 0.97 | 1.00 | 0.99 |
| ABCC4 ---tag |  | T/A or A/A | 34 | 56,67 | 18 | 56,25 | 205 | 60,12 | 170 | 55,56 | 0.77 (0.34-1.72) | 0.52 | 1.06 (0.58-1.91) | 0.86 | . | . | . |
| ABCC4 ---tag | rs1764416 | G/G | 53 | 88,33 | 31 | 96,88 | 292 | 85,63 | 265 | 86,60 | 1.00 (.-.) | . | 1.38 (0.89-2.15) | 0.15 | 0.93 | 1.00 | 0.99 |
| ABCC4 ---tag |  | G/A or A/A | 7 | 11,67 | 1 | 3,13 | 49 | 14,37 | 41 | 13,40 | 0.79 (0.10-5.97) | 0.82 | 1.20 (0.67-2.13) | 0.55 | . | . | . |
| ABCC4 ---tag | rs2274401 | T/T | 36 | 60,00 | 22 | 68,75 | 198 | 58,06 | 203 | 66,34 | 1.00 (.-.) | . | 1.34 (0.83-2.17) | 0.23 | 0.77 | 0.97 | 0.95 |
| ABCC4 ---tag |  | T/C or C/C | 24 | 40,00 | 10 | 31,25 | 143 | 41,94 | 103 | 33,66 | 0.72 (0.27-1.93) | 0.51 | 1.12 (0.67-1.88) | 0.65 | . | . | . |
| ABCC4 ---tag | rs2892716 | C/C | 28 | 46,67 | 15 | 46,88 | 117 | 34,31 | 114 | 37,25 | 1.00 (.-.) | . | 1.99 (1.07-3.71) | 0.03 | 0.05 | 0.94 | 0.70 |
| ABCC4 ---tag |  | C/T or T/T | 32 | 53,33 | 17 | 53,13 | 224 | 65,69 | 192 | 62,75 | 2.32 (1.03-5.26) | 0.04 | 1.97 (1.07-3.64) | 0.03 | . | . | . |
| ABCC4 ---tag | rs3782964 | C/C | 48 | 80,00 | 19 | 59,38 | 215 | 63,05 | 218 | 71,24 | 1.00 (.-.) | . | 1.77 (1.03-3.03) | 0.04 | 0.08 | 0.97 | 0.70 |
| ABCC4 ---tag |  | C/T or T/T | 12 | 20,00 | 13 | 40,63 | 126 | 36,95 | 88 | 28,76 | 1.72 (0.77-3.88) | 0.19 | 1.38 (0.78-2.46) | 0.27 | . | . | . |
| ABCC4 ---tag | rs3818494 | C/C | 30 | 50,00 | 21 | 65,63 | 158 | 46,33 | 123 | 40,20 | 1.00 (.-.) | . | 1.02 (0.59-1.75) | 0.94 | 0.18 | 0.97 | 0.89 |
| ABCC4 ---tag |  | C/G or G/G | 30 | 50,00 | 11 | 34,38 | 183 | 53,67 | 183 | 59,80 | 0.86 (0.37-2.04) | 0.74 | 1.62 (0.95-2.75) | 0.08 | . | . | . |
| ABCC4 ---tag | rs3864997 | G/G | 22 | 36,67 | 10 | 31,25 | 79 | 23,17 | 76 | 24,84 | 1.00 (.-.) | . | 2.03 (0.96-4.31) | 0.06 | 0.18 | 0.97 | 0.89 |
| ABCC4 ---tag |  | G/T or T/T | 38 | 63,33 | 22 | 68,75 | 262 | 76,83 | 230 | 75,16 | 1.59 (0.68-3.73) | 0.28 | 1.77 (0.86-3.64) | 0.12 | . | . | . |
| ABCC4 ---tag | rs4148421 | G/G | 15 | 25,00 | 4 | 12,50 | 105 | 30,79 | 91 | 29,74 | 1.00 (.-.) | . | 6.25 (0.86-45.41) | 0.07 | 0.04 | 0.94 | 0.70 |
| ABCC4 ---tag |  | G/A or A/A | 45 | 75,00 | 28 | 87,50 | 236 | 69,21 | 215 | 70,26 | 4.92 (0.66-36.80) | 0.12 | 5.67 (0.78-40.95) | 0.09 | . | . | . |
| ABCC4 ---tag | rs4148446 | G/G | 26 | 43,33 | 12 | 37,50 | 106 | 31,09 | 102 | 33,33 | 1.00 (.-.) | . | 1.91 (0.94-3.88) | 0.07 | 0.20 | 0.97 | 0.89 |
| ABCC4 ---tag |  | G/A or A/A | 34 | 56,67 | 20 | 62,50 | 235 | 68,91 | 204 | 66,67 | 1.69 (0.73-3.91) | 0.22 | 1.84 (0.92-3.68) | 0.08 | . | . | . |
| ABCC4 ---tag | rs4148455 | G/G | 43 | 71,67 | 27 | 84,38 | 261 | 76,54 | 226 | 73,86 | 1.00 (.-.) | . | 1.21 (0.75-1.96) | 0.44 | 0.24 | 0.97 | 0.89 |
| ABCC4 ---tag |  | G/A or A/A | 17 | 28,33 | 5 | 15,63 | 80 | 23,46 | 80 | 26,14 | 0.85 (0.31-2.30) | 0.75 | 1.87 (1.10-3.17) | 0.02 | . | . | . |
| ABCC4 ---tag | rs4148540 | C/C | 52 | 86,67 | 28 | 87,50 | 299 | 87,68 | 262 | 85,62 | 1.00 (.-.) | . | 1.43 (0.91-2.26) | 0.13 | 0.74 | 0.97 | 0.95 |
| ABCC4 ---tag |  | C/T or T/T | 8 | 13,33 | 4 | 12,50 | 42 | 12,32 | 44 | 14,38 | 1.04 (0.30-3.56) | 0.95 | 1.19 (0.69-2.04) | 0.53 | . | . | . |
| ABCC4 ---tag | rs4148542 | G/G | 11 | 18,33 | 4 | 12,50 | 94 | 27,57 | 79 | 25,82 | 1.00 (.-.) | . | 1.45 (0.35-6.02) | 0.61 | 0.97 | 1.00 | 0.99 |
| ABCC4 ---tag |  | G/A or A/A | 49 | 81,67 | 28 | 87,50 | 247 | 72,43 | 227 | 74,18 | 1.19 (0.28-5.13) | 0.82 | 1.67 (0.41-6.83) | 0.48 | . | . | . |
| ABCC4 ---tag | rs4148544 | G/G | 21 | 35,00 | 11 | 34,38 | 134 | 39,30 | 142 | 46,41 | 1.00 (.-.) | . | 1.62 (0.77-3.42) | 0.21 | 0.53 | 0.97 | 0.95 |
| ABCC4 ---tag |  | G/A or A/A | 39 | 65,00 | 21 | 65,63 | 207 | 60,70 | 164 | 53,59 | 1.09 (0.46-2.58) | 0.84 | 1.33 (0.63-2.81) | 0.45 | . | . | . |
| ABCC4 ---tag | rs4283094 | C/C | 10 | 16,67 | 5 | 15,63 | 93 | 27,27 | 70 | 22,88 | 1.00 (.-.) | . | 1.31 (0.39-4.39) | 0.66 | 0.91 | 1.00 | 0.99 |
| ABCC4 ---tag |  | C/G or G/G | 50 | 83,33 | 27 | 84,38 | 248 | 72,73 | 236 | 77,12 | 1.04 (0.30-3.59) | 0.96 | 1.46 (0.45-4.79) | 0.53 | . | . | . |
| ABCC4 ---tag | rs4636781 | A/A | 45 | 75,00 | 27 | 84,38 | 244 | 71,55 | 212 | 69,28 | 1.00 (.-.) | . | 1.23 (0.76-2.00) | 0.40 | 0.41 | 0.97 | 0.95 |
| ABCC4 ---tag |  | A/G or G/G | 15 | 25,00 | 5 | 15,63 | 97 | 28,45 | 94 | 30,72 | 0.97 (0.36-2.63) | 0.96 | 1.81 (1.08-3.05) | 0.03 | . | . | . |
| ABCC4 ---tag | rs4771910 | T/T | 32 | 53,33 | 14 | 43,75 | 161 | 47,21 | 146 | 47,71 | 1.00 (.-.) | . | 1.74 (0.90-3.37) | 0.10 | 0.33 | 0.97 | 0.95 |
| ABCC4 ---tag |  | T/C or C/C | 28 | 46,67 | 18 | 56,25 | 180 | 52,79 | 160 | 52,29 | 1.31 (0.58-2.95) | 0.52 | 1.49 (0.77-2.89) | 0.23 | . | . | . |
| ABCC4 ---tag | rs4773850 | T/T | 30 | 50,00 | 14 | 43,75 | 150 | 43,99 | 157 | 51,31 | 1.00 (.-.) | . | 1.83 (0.99-3.36) | 0.05 | 0.18 | 0.97 | 0.89 |
| ABCC4 ---tag |  | T/G or G/G | 30 | 50,00 | 18 | 56,25 | 191 | 56,01 | 149 | 48,69 | 1.39 (0.63-3.08) | 0.41 | 1.44 (0.78-2.64) | 0.24 | . | . | . |
| ABCC4 ---tag | rs7981095 | A/A | 39 | 65,00 | 20 | 62,50 | 223 | 65,40 | 196 | 64,05 | 1.00 (.-.) | . | 1.27 (0.76-2.13) | 0.36 | 0.63 | 0.97 | 0.95 |
| ABCC4 ---tag |  | A/T or T/T | 21 | 35,00 | 12 | 37,50 | 118 | 34,60 | 110 | 35,95 | 0.70 (0.29-1.65) | 0.41 | 1.11 (0.65-1.91) | 0.71 | . | . | . |
| ABCC4 ---tag | rs8001444 | C/C | 21 | 35,00 | 9 | 28,13 | 118 | 34,60 | 118 | 38,56 | 1.00 (.-.) | . | 1.53 (0.65-3.57) | 0.33 | 0.71 | 0.97 | 0.95 |
| ABCC4 ---tag |  | C/T or T/T | 39 | 65,00 | 23 | 71,88 | 223 | 65,40 | 188 | 61,44 | 0.91 (0.36-2.31) | 0.84 | 1.16 (0.50-2.68) | 0.73 | . | . | . |
| ABCC4 ---tag | rs931111 | T/T | 36 | 60,00 | 19 | 59,38 | 238 | 69,79 | 198 | 64,71 | 1.00 (.-.) | . | 1.48 (0.84-2.60) | 0.17 | 0.67 | 0.97 | 0.95 |
| ABCC4 ---tag |  | T/C or C/C | 24 | 40,00 | 13 | 40,63 | 103 | 30,21 | 108 | 35,29 | 1.27 (0.57-2.81) | 0.56 | 1.56 (0.88-2.77) | 0.13 | . | . | . |
| ABCC4 ---tag | rs943288 | T/T | 47 | 78,33 | 28 | 87,50 | 259 | 75,95 | 228 | 74,51 | 1.00 (.-.) | . | 1.20 (0.75-1.93) | 0.45 | 0.24 | 0.97 | 0.89 |
| ABCC4 ---tag |  | T/A or A/A | 13 | 21,67 | 4 | 12,50 | 82 | 24,05 | 78 | 25,49 | 0.79 (0.27-2.34) | 0.67 | 1.80 (1.07-3.02) | 0.03 | . | . | . |
| ABCC4 ---tag | rs943290 | A/A | 33 | 55,00 | 19 | 59,38 | 165 | 48,39 | 170 | 55,56 | 1.00 (.-.) | . | 1.52 (0.90-2.56) | 0.11 | 0.71 | 0.97 | 0.95 |
| ABCC4 ---tag |  | A/G or G/G | 27 | 45,00 | 13 | 40,63 | 176 | 51,61 | 136 | 44,44 | 0.86 (0.36-2.02) | 0.73 | 1.10 (0.65-1.86) | 0.72 | . | . | . |
| ABCC4 ---tag | rs9516530 | C/C | 32 | 53,33 | 22 | 68,75 | 193 | 56,60 | 168 | 54,90 | 1.00 (.-.) | . | 1.01 (0.60-1.70) | 0.97 | 0.07 | 0.97 | 0.70 |
| ABCC4 ---tag |  | C/T or T/T | 28 | 46,67 | 10 | 31,25 | 148 | 43,40 | 138 | 45,10 | 0.51 (0.21-1.24) | 0.14 | 1.16 (0.69-1.96) | 0.57 | . | . | . |
| ABCC4 ---tag | rs9516551 | C/C | 45 | 75,00 | 21 | 65,63 | 260 | 76,25 | 241 | 78,76 | 1.00 (.-.) | . | 1.60 (0.93-2.77) | 0.09 | 0.29 | 0.97 | 0.95 |
| ABCC4 ---tag |  | C/A or A/A | 15 | 25,00 | 11 | 34,38 | 81 | 23,75 | 65 | 21,24 | 1.25 (0.56-2.82) | 0.58 | 1.25 (0.69-2.25) | 0.46 | . | . | . |
| ABCC4 ---tag | rs9524822 | T/T | 48 | 80,00 | 18 | 56,25 | 213 | 62,46 | 200 | 65,36 | 1.00 (.-.) | . | 1.87 (1.05-3.32) | 0.03 | 0.08 | 0.97 | 0.70 |
| ABCC4 ---tag |  | T/C or C/C | 12 | 20,00 | 14 | 43,75 | 128 | 37,54 | 106 | 34,64 | 1.75 (0.78-3.91) | 0.18 | 1.51 (0.83-2.73) | 0.18 | . | . | . |
| ABCC4 ---tag | rs9524861 | G/G | 25 | 41,67 | 13 | 40,63 | 177 | 51,91 | 162 | 52,94 | 1.00 (.-.) | . | 1.23 (0.63-2.39) | 0.55 | 0.74 | 0.97 | 0.95 |
| ABCC4 ---tag |  | G/C or C/C | 35 | 58,33 | 19 | 59,38 | 164 | 48,09 | 144 | 47,06 | 0.76 (0.34-1.71) | 0.51 | 1.08 (0.55-2.12) | 0.82 | . | . | . |
| ABCC4 ---tag | rs9524902 | T/T | 17 | 28,33 | 6 | 18,75 | 88 | 25,81 | 103 | 33,66 | 1.00 (.-.) | . | 1.71 (0.62-4.75) | 0.30 | 0.57 | 0.97 | 0.95 |
| ABCC4 ---tag |  | T/C or C/C | 43 | 71,67 | 26 | 81,25 | 253 | 74,19 | 203 | 66,34 | 1.01 (0.34-2.98) | 0.99 | 1.26 (0.46-3.46) | 0.65 | . | . | . |
| ABCC4 ---tag | rs9556455 | G/G | 47 | 78,33 | 21 | 65,63 | 262 | 76,83 | 232 | 75,82 | 1.00 (.-.) | . | 1.58 (0.93-2.70) | 0.09 | 0.34 | 0.97 | 0.95 |
| ABCC4 ---tag |  | G/A or A/A | 13 | 21,67 | 11 | 34,38 | 79 | 23,17 | 74 | 24,18 | 1.53 (0.66-3.55) | 0.32 | 1.56 (0.88-2.79) | 0.13 | . | . | . |
| ABCC4 ---NA | rs9561778 | G/G | 37 | 61,67 | 23 | 71,88 | 214 | 62,76 | 211 | 68,95 | 1.00 (.-.) | . | 1.32 (0.82-2.13) | 0.25 | 0.65 | 0.97 | 0.95 |
| ABCC4 ---NA |  | G/T or T/T | 23 | 38,33 | 9 | 28,13 | 127 | 37,24 | 95 | 31,05 | 0.66 (0.22-1.94) | 0.45 | 1.12 (0.67-1.87) | 0.66 | . | . | . |
| ABCC4 ---tag | rs9561811 | C/C | 39 | 65,00 | 26 | 81,25 | 232 | 68,04 | 198 | 64,71 | 1.00 (.-.) | . | 1.37 (0.83-2.26) | 0.21 | 0.87 | 0.99 | 0.99 |
| ABCC4 ---tag |  | C/T or T/T | 21 | 35,00 | 6 | 18,75 | 109 | 31,96 | 108 | 35,29 | 1.29 (0.50-3.33) | 0.60 | 1.62 (0.97-2.73) | 0.07 | . | . | . |
| ABCC4 ---tag | rs9590183 | T/T | 54 | 90,00 | 30 | 93,75 | 293 | 85,92 | 264 | 86,27 | 1.00 (.-.) | . | 1.35 (0.86-2.10) | 0.19 | 0.67 | 0.97 | 0.95 |
| ABCC4 ---tag |  | T/A or A/A | 6 | 10,00 | 2 | 6,25 | 48 | 14,08 | 42 | 13,73 | 0.54 (0.12-2.34) | 0.41 | 1.00 (0.57-1.77) | 1.00 | . | . | . |
| ABCC4 ---tag | rs997777 | T/T | 27 | 45,00 | 15 | 46,88 | 166 | 48,68 | 153 | 50,00 | 1.00 (.-.) | . | 1.65 (0.90-3.01) | 0.10 | 0.35 | 0.97 | 0.95 |
| ABCC4 ---tag |  | T/A or A/A | 33 | 55,00 | 17 | 53,13 | 175 | 51,32 | 153 | 50,00 | 1.47 (0.66-3.29) | 0.35 | 1.62 (0.89-2.97) | 0.12 | . | . | . |
| ADH1B ---tag | rs1159918 | G/G | 20 | 33,33 | 13 | 40,63 | 154 | 45,16 | 148 | 48,37 | 1.00 (.-.) | . | 0.91 (0.48-1.75) | 0.79 | 0.15 | 0.97 | 0.25 |
| ADH1B ---tag |  | G/T or T/T | 40 | 66,67 | 19 | 59,38 | 187 | 54,84 | 158 | 51,63 | 0.52 (0.23-1.18) | 0.12 | 0.90 (0.47-1.72) | 0.75 | . | . | . |
| ADH1B ---candidate literature | rs1229984 | G/G | 52 | 86,67 | 31 | 96,88 | 308 | 90,32 | 277 | 90,52 | 1.00 (.-.) | . | 1.20 (0.78-1.86) | 0.41 | 0.06 | 0.94 | 0.15 |
| ADH1B ---candidate literature |  | G/A or A/A | 8 | 13,33 | 1 | 3,13 | 33 | 9,68 | 29 | 9,48 | 0.22 (0.03-1.64) | 0.14 | 1.30 (0.71-2.39) | 0.39 | . | . | . |
| ADH1B ---tag | rs12507573 | C/C | 19 | 31,67 | 12 | 37,50 | 103 | 30,21 | 85 | 27,78 | 1.00 (.-.) | . | 1.43 (0.67-3.06) | 0.36 | 0.91 | 1.00 | 0.91 |
| ADH1B ---tag |  | C/A or A/A | 41 | 68,33 | 20 | 62,50 | 238 | 69,79 | 221 | 72,22 | 0.98 (0.42-2.32) | 0.97 | 1.33 (0.64-2.76) | 0.44 | . | . | . |
| ADH1B ---tag | rs1693457 | T/T | 40 | 66,67 | 19 | 59,38 | 232 | 68,04 | 224 | 73,20 | 1.00 (.-.) | . | 1.17 (0.69-1.98) | 0.57 | 0.43 | 0.97 | 0.53 |
| ADH1B ---tag |  | T/C or C/C | 20 | 33,33 | 13 | 40,63 | 109 | 31,96 | 82 | 26,80 | 0.62 (0.27-1.41) | 0.25 | 1.03 (0.58-1.80) | 0.93 | . | . | . |
| ADH1B ---tag | rs2066701 | C/C | 30 | 50,00 | 9 | 28,13 | 159 | 46,63 | 142 | 46,41 | 1.00 (.-.) | . | 2.62 (1.13-6.06) | 0.02 | 0.04 | 0.94 | 0.15 |
| ADH1B ---tag |  | C/T or T/T | 30 | 50,00 | 23 | 71,88 | 182 | 53,37 | 164 | 53,59 | 2.55 (1.01-6.46) | 0.05 | 2.52 (1.09-5.80) | 0.03 | . | . | . |
| ADH1C ---tag | rs11936869 | C/C | 30 | 50,00 | 12 | 37,50 | 176 | 51,61 | 170 | 55,56 | 1.00 (.-.) | . | 1.53 (0.81-2.90) | 0.19 | 0.62 | 0.97 | 0.77 |
| ADH1C ---tag |  | C/G or G/G | 30 | 50,00 | 20 | 62,50 | 165 | 48,39 | 136 | 44,44 | 1.18 (0.53-2.63) | 0.69 | 1.47 (0.77-2.78) | 0.24 | . | . | . |
| ADH1C ---tag | rs1229849 | T/T | 34 | 56,67 | 19 | 59,38 | 190 | 55,72 | 144 | 47,06 | 1.00 (.-.) | . | 1.22 (0.70-2.14) | 0.49 | 0.58 | 0.97 | 0.77 |
| ADH1C ---tag |  | T/A or A/A | 26 | 43,33 | 13 | 40,63 | 151 | 44,28 | 162 | 52,94 | 0.98 (0.43-2.20) | 0.95 | 1.51 (0.87-2.63) | 0.15 | . | . | . |
| ADH1C ---tag | rs1229863 | A/A | 44 | 73,33 | 22 | 68,75 | 247 | 72,43 | 226 | 73,86 | 1.00 (.-.) | . | 1.60 (0.97-2.65) | 0.07 | 0.20 | 0.97 | 0.77 |
| ADH1C ---tag |  | A/T or T/T | 16 | 26,67 | 10 | 31,25 | 94 | 27,57 | 80 | 26,14 | 1.71 (0.70-4.16) | 0.24 | 1.46 (0.84-2.51) | 0.18 | . | . | . |
| ADH1C ---tag | rs1229980 | C/C | 53 | 88,33 | 29 | 90,63 | 312 | 91,50 | 271 | 88,56 | 1.00 (.-.) | . | 1.40 (0.89-2.19) | 0.15 | 0.45 | 0.97 | 0.77 |
| ADH1C ---tag |  | C/G or G/G | 7 | 11,67 | 3 | 9,38 | 29 | 8,50 | 35 | 11,44 | 2.52 (0.58-11.00) | 0.22 | 1.87 (1.06-3.30) | 0.03 | . | . | . |
| ADH1C ---candidate | rs1693482 | C/C | 29 | 48,33 | 13 | 40,63 | 146 | 42,82 | 117 | 38,24 | 1.00 (.-.) | . | 1.47 (0.77-2.80) | 0.24 | 0.76 | 0.97 | 0.77 |
| ADH1C ---candidate |  | C/T or T/T | 31 | 51,67 | 19 | 59,38 | 195 | 57,18 | 189 | 61,76 | 1.20 (0.54-2.68) | 0.66 | 1.55 (0.82-2.91) | 0.18 | . | . | . |
| ADH1C ---tag | rs2173201 | C/C | 34 | 56,67 | 13 | 40,63 | 196 | 57,48 | 184 | 60,13 | 1.00 (.-.) | . | 1.93 (1.06-3.55) | 0.03 | 0.06 | 0.94 | 0.50 |
| ADH1C ---tag |  | C/A or A/A | 26 | 43,33 | 19 | 59,38 | 145 | 42,52 | 122 | 39,87 | 2.35 (1.06-5.24) | 0.04 | 2.00 (1.08-3.69) | 0.03 | . | . | . |
| ADH1C ---tag | rs2298753 | T/T | 51 | 85,00 | 25 | 78,13 | 272 | 79,77 | 257 | 83,99 | 1.00 (.-.) | . | 1.53 (0.95-2.47) | 0.08 | 0.27 | 0.97 | 0.77 |
| ADH1C ---tag |  | T/C or C/C | 9 | 15,00 | 7 | 21,88 | 69 | 20,23 | 49 | 16,01 | 1.38 (0.51-3.74) | 0.53 | 1.13 (0.64-1.99) | 0.69 | . | . | . |
| ADH1C ---tag | rs2866152 | G/G | 40 | 66,67 | 21 | 65,63 | 213 | 62,46 | 172 | 56,21 | 1.00 (.-.) | . | 1.26 (0.73-2.15) | 0.40 | 0.61 | 0.97 | 0.77 |
| ADH1C ---tag |  | G/C or C/C | 20 | 33,33 | 11 | 34,38 | 128 | 37,54 | 134 | 43,79 | 0.93 (0.41-2.14) | 0.87 | 1.47 (0.85-2.54) | 0.17 | . | . | . |
| ADH1C ---tag | rs904096 | T/T | 29 | 48,33 | 13 | 40,63 | 144 | 42,23 | 115 | 37,58 | 1.00 (.-.) | . | 1.46 (0.77-2.79) | 0.25 | 0.77 | 0.97 | 0.77 |
| ADH1C ---tag |  | T/G or G/G | 31 | 51,67 | 19 | 59,38 | 197 | 57,77 | 191 | 62,42 | 1.20 (0.54-2.68) | 0.66 | 1.55 (0.82-2.91) | 0.17 | . | . | . |
| BHMT ---tag | rs10944 | A/A | 16 | 26,67 | 7 | 21,88 | 72 | 21,11 | 78 | 25,49 | 1.00 (.-.) | . | 1.76 (0.69-4.50) | 0.24 | 0.54 | 0.97 | 0.55 |
| BHMT ---tag |  | A/C or C/C | 44 | 73,33 | 25 | 78,13 | 269 | 78,89 | 228 | 74,51 | 1.12 (0.41-3.03) | 0.83 | 1.43 (0.57-3.58) | 0.45 | . | . | . |
| BHMT ---tag | rs12655567 | C/C | 24 | 40,00 | 12 | 37,50 | 115 | 33,72 | 122 | 39,87 | 1.00 (.-.) | . | 2.02 (0.99-4.11) | 0.05 | 0.18 | 0.97 | 0.46 |
| BHMT ---tag |  | C/G or G/G | 36 | 60,00 | 20 | 62,50 | 226 | 66,28 | 184 | 60,13 | 1.23 (0.53-2.85) | 0.63 | 1.36 (0.68-2.74) | 0.39 | . | . | . |
| BHMT ---tag | rs1291041 | G/G | 28 | 46,67 | 14 | 43,75 | 131 | 38,42 | 143 | 46,73 | 1.00 (.-.) | . | 1.93 (0.98-3.79) | 0.06 | 0.20 | 0.97 | 0.46 |
| BHMT ---tag |  | G/T or T/T | 32 | 53,33 | 18 | 56,25 | 210 | 61,58 | 163 | 53,27 | 1.19 (0.52-2.73) | 0.67 | 1.31 (0.67-2.55) | 0.43 | . | . | . |
| BHMT ---tag | rs16876500 | C/C | 50 | 83,33 | 25 | 78,13 | 277 | 81,23 | 237 | 77,45 | 1.00 (.-.) | . | 1.30 (0.81-2.08) | 0.28 | 0.52 | 0.97 | 0.55 |
| BHMT ---tag |  | C/T or T/T | 10 | 16,67 | 7 | 21,88 | 64 | 18,77 | 69 | 22,55 | 0.88 (0.32-2.40) | 0.80 | 1.60 (0.93-2.74) | 0.09 | . | . | . |
| BHMT ---tag | rs492842 | A/A | 25 | 41,67 | 12 | 37,50 | 129 | 37,83 | 122 | 39,87 | 1.00 (.-.) | . | 2.00 (0.98-4.08) | 0.06 | 0.16 | 0.97 | 0.46 |
| BHMT ---tag |  | A/G or G/G | 35 | 58,33 | 20 | 62,50 | 212 | 62,17 | 184 | 60,13 | 1.67 (0.72-3.87) | 0.23 | 1.78 (0.88-3.59) | 0.11 | . | . | . |
| BHMT ---tag | rs558133 | T/T | 27 | 45,00 | 16 | 50,00 | 163 | 47,80 | 146 | 47,71 | 1.00 (.-.) | . | 1.56 (0.84-2.89) | 0.16 | 0.55 | 0.97 | 0.55 |
| BHMT ---tag |  | T/G or G/G | 33 | 55,00 | 16 | 50,00 | 178 | 52,20 | 160 | 52,29 | 1.24 (0.56-2.77) | 0.60 | 1.49 (0.81-2.76) | 0.20 | . | . | . |
| BHMT ---tag | rs9637824 | A/A | 23 | 38,33 | 11 | 34,38 | 130 | 38,12 | 117 | 38,24 | 1.00 (.-.) | . | 1.78 (0.87-3.64) | 0.11 | 0.34 | 0.97 | 0.55 |
| BHMT ---tag |  | A/G or G/G | 37 | 61,67 | 21 | 65,63 | 211 | 61,88 | 189 | 61,76 | 1.41 (0.61-3.27) | 0.43 | 1.64 (0.81-3.31) | 0.17 | . | . | . |
| BHMT2 ---tag | rs16876512 | C/C | 50 | 83,33 | 25 | 78,13 | 274 | 80,35 | 237 | 77,45 | 1.00 (.-.) | . | 1.30 (0.81-2.09) | 0.27 | 0.56 | 0.97 | 0.82 |
| BHMT2 ---tag |  | C/T or T/T | 10 | 16,67 | 7 | 21,88 | 67 | 19,65 | 69 | 22,55 | 0.88 (0.32-2.41) | 0.80 | 1.55 (0.91-2.67) | 0.11 | . | . | . |
| BHMT2 ---tag | rs2461248 | T/T | 16 | 26,67 | 6 | 18,75 | 71 | 20,82 | 78 | 25,49 | 1.00 (.-.) | . | 2.08 (0.74-5.87) | 0.17 | 0.35 | 0.97 | 0.71 |
| BHMT2 ---tag |  | T/A or A/A | 44 | 73,33 | 26 | 81,25 | 270 | 79,18 | 228 | 74,51 | 1.37 (0.46-4.06) | 0.57 | 1.69 (0.61-4.68) | 0.31 | . | . | . |
| BHMT2 ---tag | rs2909856 | T/T | 26 | 43,33 | 14 | 43,75 | 140 | 41,06 | 130 | 42,48 | 1.00 (.-.) | . | 1.78 (0.93-3.41) | 0.08 | 0.28 | 0.97 | 0.71 |
| BHMT2 ---tag |  | T/C or C/C | 34 | 56,67 | 18 | 56,25 | 201 | 58,94 | 176 | 57,52 | 1.41 (0.63-3.18) | 0.40 | 1.57 (0.83-2.97) | 0.17 | . | . | . |
| BHMT2 ---tag | rs476620 | A/A | 23 | 38,33 | 11 | 34,38 | 130 | 38,12 | 116 | 37,91 | 1.00 (.-.) | . | 1.77 (0.87-3.63) | 0.12 | 0.35 | 0.97 | 0.71 |
| BHMT2 ---tag |  | A/G or G/G | 37 | 61,67 | 21 | 65,63 | 211 | 61,88 | 190 | 62,09 | 1.41 (0.61-3.27) | 0.43 | 1.65 (0.81-3.33) | 0.17 | . | . | . |
| BHMT2 ---candidate literature | rs626105 | G/G | 33 | 55,00 | 23 | 71,88 | 214 | 62,76 | 196 | 64,05 | 1.00 (.-.) | . | 1.37 (0.82-2.29) | 0.22 | 0.95 | 1.00 | 0.95 |
| BHMT2 ---candidate literature |  | G/A or A/A | 27 | 45,00 | 9 | 28,13 | 127 | 37,24 | 110 | 35,95 | 0.78 (0.32-1.90) | 0.58 | 1.10 (0.65-1.88) | 0.71 | . | . | . |
| BHMT2 ---tag | rs631305 | G/G | 37 | 61,67 | 26 | 81,25 | 237 | 69,50 | 217 | 70,92 | 1.00 (.-.) | . | 1.32 (0.81-2.15) | 0.26 | 0.68 | 0.97 | 0.82 |
| BHMT2 ---tag |  | G/A or A/A | 23 | 38,33 | 6 | 18,75 | 104 | 30,50 | 89 | 29,08 | 0.65 (0.24-1.77) | 0.41 | 1.07 (0.64-1.80) | 0.79 | . | . | . |
| CBS ---tag | rs11701048 | C/C | 52 | 86,67 | 26 | 81,25 | 285 | 83,58 | 270 | 88,24 | 1.00 (.-.) | . | 1.53 (0.95-2.46) | 0.08 | 0.21 | 0.97 | 0.84 |
| CBS ---tag |  | C/T or T/T | 8 | 13,33 | 6 | 18,75 | 56 | 16,42 | 36 | 11,76 | 1.82 (0.67-4.91) | 0.24 | 1.36 (0.76-2.44) | 0.30 | . | . | . |
| CBS ---tag | rs234706 | G/G | 24 | 40,00 | 12 | 37,50 | 153 | 44,87 | 121 | 39,54 | 1.00 (.-.) | . | 1.06 (0.54-2.10) | 0.87 | 0.37 | 0.97 | 0.84 |
| CBS ---tag |  | G/A or A/A | 36 | 60,00 | 20 | 62,50 | 188 | 55,13 | 185 | 60,46 | 0.75 (0.33-1.70) | 0.50 | 1.19 (0.61-2.31) | 0.61 | . | . | . |
| CBS ---tag | rs234711 | C/C | 36 | 60,00 | 18 | 56,25 | 208 | 61,00 | 168 | 54,90 | 1.00 (.-.) | . | 1.25 (0.71-2.21) | 0.43 | 0.66 | 0.97 | 0.84 |
| CBS ---tag |  | C/A or A/A | 24 | 40,00 | 14 | 43,75 | 133 | 39,00 | 138 | 45,10 | 0.94 (0.41-2.13) | 0.88 | 1.43 (0.82-2.51) | 0.21 | . | . | . |
| CBS ---candidate literature | rs234713 | G/G | 30 | 50,00 | 16 | 50,00 | 172 | 50,44 | 142 | 46,41 | 1.00 (.-.) | . | 1.34 (0.73-2.45) | 0.35 | 0.93 | 1.00 | 0.95 |
| CBS ---candidate literature |  | G/A or A/A | 30 | 50,00 | 16 | 50,00 | 169 | 49,56 | 164 | 53,59 | 1.04 (0.47-2.32) | 0.92 | 1.45 (0.80-2.62) | 0.22 | . | . | . |
| CBS ---tag | rs2839623 | T/T | 48 | 80,00 | 29 | 90,63 | 279 | 81,82 | 254 | 83,01 | 1.00 (.-.) | . | 1.36 (0.86-2.16) | 0.19 | 0.95 | 1.00 | 0.95 |
| CBS ---tag |  | T/A or A/A | 12 | 20,00 | 3 | 9,38 | 62 | 18,18 | 52 | 16,99 | 1.01 (0.30-3.44) | 0.99 | 1.43 (0.83-2.46) | 0.19 | . | . | . |
| CBS ---tag | rs2839626 | C/C | 29 | 48,33 | 11 | 34,38 | 171 | 50,15 | 139 | 45,42 | 1.00 (.-.) | . | 1.58 (0.76-3.29) | 0.22 | 0.67 | 0.97 | 0.84 |
| CBS ---tag |  | C/T or T/T | 31 | 51,67 | 21 | 65,63 | 170 | 49,85 | 167 | 54,58 | 1.47 (0.62-3.46) | 0.38 | 1.92 (0.93-3.98) | 0.08 | . | . | . |
| CBS ---tag | rs422791 | T/T | 28 | 46,67 | 20 | 62,50 | 177 | 51,91 | 137 | 44,77 | 1.00 (.-.) | . | 1.06 (0.62-1.81) | 0.84 | 0.16 | 0.97 | 0.84 |
| CBS ---tag |  | T/C or C/C | 32 | 53,33 | 12 | 37,50 | 164 | 48,09 | 169 | 55,23 | 0.62 (0.26-1.46) | 0.28 | 1.22 (0.72-2.08) | 0.46 | . | . | . |
| CBS ---tag | rs706209 | C/C | 17 | 28,33 | 9 | 28,13 | 113 | 33,14 | 97 | 31,70 | 1.00 (.-.) | . | 1.13 (0.48-2.65) | 0.78 | 0.61 | 0.97 | 0.84 |
| CBS ---tag |  | C/T or T/T | 43 | 71,67 | 23 | 71,88 | 228 | 66,86 | 209 | 68,30 | 0.81 (0.32-2.07) | 0.66 | 1.19 (0.52-2.73) | 0.69 | . | . | . |
| CBS ---tag | rs719037 | A/A | 19 | 31,67 | 14 | 43,75 | 112 | 32,84 | 92 | 30,07 | 1.00 (.-.) | . | 1.19 (0.62-2.27) | 0.61 | 0.58 | 0.97 | 0.84 |
| CBS ---tag |  | A/G or G/G | 41 | 68,33 | 18 | 56,25 | 229 | 67,16 | 214 | 69,93 | 0.85 (0.38-1.91) | 0.70 | 1.29 (0.69-2.38) | 0.43 | . | . | . |
| CBS ---tag | rs719038 | T/T | 28 | 46,67 | 11 | 34,38 | 158 | 46,33 | 132 | 43,14 | 1.00 (.-.) | . | 1.59 (0.77-3.30) | 0.21 | 0.63 | 0.97 | 0.84 |
| CBS ---tag |  | T/C or C/C | 32 | 53,33 | 21 | 65,63 | 183 | 53,67 | 174 | 56,86 | 1.37 (0.58-3.23) | 0.47 | 1.75 (0.85-3.61) | 0.13 | . | . | . |
| DHFR ---tag | rs10474632 | G/G | 53 | 88,33 | 28 | 87,50 | 286 | 83,87 | 252 | 82,35 | 1.00 (.-.) | . | 1.32 (0.83-2.09) | 0.24 | 0.66 | 0.97 | 0.82 |
| DHFR ---tag |  | G/A or A/A | 7 | 11,67 | 4 | 12,50 | 55 | 16,13 | 54 | 17,65 | 0.82 (0.24-2.78) | 0.75 | 1.43 (0.84-2.44) | 0.19 | . | . | . |
| DHFR ---tag | rs11951910 | T/T | 48 | 80,00 | 25 | 78,13 | 277 | 81,23 | 249 | 81,37 | 1.00 (.-.) | . | 1.25 (0.76-2.06) | 0.37 | 0.38 | 0.97 | 0.76 |
| DHFR ---tag |  | T/C or C/C | 12 | 20,00 | 7 | 21,88 | 64 | 18,77 | 57 | 18,63 | 0.87 (0.34-2.23) | 0.77 | 1.70 (0.97-2.98) | 0.07 | . | . | . |
| DHFR ---tag | rs1643665 | T/T | 33 | 55,00 | 11 | 34,38 | 152 | 44,57 | 143 | 46,73 | 1.00 (.-.) | . | 1.78 (0.85-3.69) | 0.12 | 0.36 | 0.97 | 0.76 |
| DHFR ---tag |  | T/C or C/C | 27 | 45,00 | 21 | 65,63 | 189 | 55,43 | 163 | 53,27 | 1.47 (0.62-3.49) | 0.38 | 1.73 (0.83-3.59) | 0.14 | . | . | . |
| DHFR ---tag | rs1650717 | T/T | 26 | 43,33 | 17 | 53,13 | 190 | 55,72 | 165 | 53,92 | 1.00 (.-.) | . | 1.11 (0.60-2.05) | 0.74 | 0.31 | 0.97 | 0.76 |
| DHFR ---tag |  | T/G or G/G | 34 | 56,67 | 15 | 46,88 | 151 | 44,28 | 141 | 46,08 | 0.81 (0.37-1.80) | 0.61 | 1.39 (0.75-2.59) | 0.29 | . | . | . |
| DHFR ---tag | rs1805355 | G/G | 52 | 86,67 | 22 | 68,75 | 302 | 88,56 | 265 | 86,60 | 1.00 (.-.) | . | 1.86 (1.10-3.14) | 0.02 | 0.01 | 0.85 | 0.08 |
| DHFR ---tag |  | G/A or A/A | 8 | 13,33 | 10 | 31,25 | 39 | 11,44 | 41 | 13,40 | 4.37 (1.89-10.11) | 0.00 | 2.13 (1.15-3.95) | 0.02 | . | . | . |
| DHFR ---tag | rs6151617 | A/A | 27 | 45,00 | 9 | 28,13 | 118 | 34,60 | 108 | 35,29 | 1.00 (.-.) | . | 1.92 (0.83-4.47) | 0.13 | 0.32 | 0.97 | 0.76 |
| DHFR ---tag |  | A/G or G/G | 33 | 55,00 | 23 | 71,88 | 223 | 65,40 | 198 | 64,71 | 1.66 (0.65-4.23) | 0.29 | 1.98 (0.86-4.53) | 0.11 | . | . | . |
| DHFR ---tag | rs6864493 | T/T | 38 | 63,33 | 17 | 53,13 | 179 | 52,49 | 178 | 58,17 | 1.00 (.-.) | . | 1.22 (0.69-2.15) | 0.49 | 0.58 | 0.97 | 0.82 |
| DHFR ---tag |  | T/C or C/C | 22 | 36,67 | 15 | 46,88 | 162 | 47,51 | 128 | 41,83 | 0.67 (0.29-1.52) | 0.34 | 1.04 (0.58-1.85) | 0.90 | . | . | . |
| DHFR ---tag | rs836788 | G/G | 25 | 41,67 | 19 | 59,38 | 153 | 44,87 | 122 | 39,87 | 1.00 (.-.) | . | 1.37 (0.78-2.40) | 0.28 | 0.97 | 1.00 | 0.97 |
| DHFR ---tag |  | G/A or A/A | 35 | 58,33 | 13 | 40,63 | 188 | 55,13 | 184 | 60,13 | 1.08 (0.48-2.44) | 0.84 | 1.46 (0.84-2.52) | 0.18 | . | . | . |
| DHFR ---tag | rs836790 | A/A | 37 | 61,67 | 22 | 68,75 | 244 | 71,55 | 209 | 68,30 | 1.00 (.-.) | . | 1.23 (0.73-2.09) | 0.44 | 0.47 | 0.97 | 0.78 |
| DHFR ---tag |  | A/G or G/G | 23 | 38,33 | 10 | 31,25 | 97 | 28,45 | 97 | 31,70 | 0.85 (0.37-1.94) | 0.69 | 1.44 (0.83-2.51) | 0.20 | . | . | . |
| DHFR ---tag | rs836817 | G/G | 26 | 43,33 | 20 | 62,50 | 172 | 50,44 | 134 | 43,79 | 1.00 (.-.) | . | 1.38 (0.81-2.35) | 0.24 | 0.91 | 1.00 | 0.97 |
| DHFR ---tag |  | G/T or T/T | 34 | 56,67 | 12 | 37,50 | 169 | 49,56 | 172 | 56,21 | 1.12 (0.48-2.64) | 0.79 | 1.46 (0.87-2.47) | 0.16 | . | . | . |
| DNMT1 ---candidate | rs2228612 | A/A | 49 | 81,67 | 26 | 81,25 | 303 | 88,86 | 267 | 87,25 | 1.00 (.-.) | . | 1.34 (0.84-2.11) | 0.22 | 0.74 | 0.97 | 0.74 |
| DNMT1 ---candidate |  | A/G or G/G | 11 | 18,33 | 6 | 18,75 | 38 | 11,14 | 39 | 12,75 | 0.83 (0.25-2.79) | 0.76 | 1.37 (0.78-2.39) | 0.27 | . | . | . |
| DNMT3A ---tag | rs10460566 | A/A | 35 | 58,33 | 21 | 65,63 | 198 | 58,06 | 184 | 60,13 | 1.00 (.-.) | . | 1.47 (0.87-2.47) | 0.15 | 0.62 | 0.97 | 0.70 |
| DNMT3A ---tag |  | A/G or G/G | 25 | 41,67 | 11 | 34,38 | 143 | 41,94 | 122 | 39,87 | 1.28 (0.54-3.01) | 0.58 | 1.48 (0.87-2.53) | 0.15 | . | . | . |
| DNMT3A ---candidate literature | rs11695471 | T/T | 29 | 48,33 | 11 | 34,38 | 143 | 41,94 | 138 | 45,10 | 1.00 (.-.) | . | 1.18 (0.63-2.22) | 0.61 | 0.52 | 0.97 | 0.70 |
| DNMT3A ---candidate literature |  | T/A or A/A | 31 | 51,67 | 21 | 65,63 | 198 | 58,06 | 168 | 54,90 | 0.87 (0.39-1.95) | 0.74 | 1.36 (0.72-2.55) | 0.34 | . | . | . |
| DNMT3A ---tag | rs11887120 | C/C | 19 | 31,67 | 14 | 43,75 | 124 | 36,36 | 105 | 34,31 | 1.00 (.-.) | . | 1.16 (0.57-2.36) | 0.68 | 0.58 | 0.97 | 0.70 |
| DNMT3A ---tag |  | C/T or T/T | 41 | 68,33 | 18 | 56,25 | 217 | 63,64 | 201 | 65,69 | 0.78 (0.34-1.79) | 0.56 | 1.16 (0.58-2.32) | 0.67 | . | . | . |
| DNMT3A ---tag | rs12991495 | T/T | 32 | 53,33 | 12 | 37,50 | 150 | 43,99 | 150 | 49,02 | 1.00 (.-.) | . | 1.26 (0.67-2.37) | 0.48 | 0.70 | 0.97 | 0.70 |
| DNMT3A ---tag |  | T/C or C/C | 28 | 46,67 | 20 | 62,50 | 191 | 56,01 | 156 | 50,98 | 0.92 (0.41-2.06) | 0.83 | 1.36 (0.73-2.56) | 0.34 | . | . | . |
| DNMT3A ---tag | rs13401241 | A/A | 22 | 36,67 | 7 | 21,88 | 95 | 27,86 | 85 | 27,78 | 1.00 (.-.) | . | 2.77 (1.08-7.11) | 0.03 | 0.06 | 0.94 | 0.51 |
| DNMT3A ---tag |  | A/C or C/C | 38 | 63,33 | 25 | 78,13 | 246 | 72,14 | 221 | 72,22 | 2.03 (0.75-5.50) | 0.17 | 2.22 (0.89-5.58) | 0.09 | . | . | . |
| DNMT3A ---candidate literature | rs13420827 | C/C | 42 | 70,00 | 21 | 65,63 | 208 | 61,00 | 210 | 68,63 | 1.00 (.-.) | . | 1.66 (0.94-2.92) | 0.08 | 0.27 | 0.97 | 0.54 |
| DNMT3A ---candidate literature |  | C/G or G/G | 18 | 30,00 | 11 | 34,38 | 133 | 39,00 | 96 | 31,37 | 1.30 (0.58-2.93) | 0.53 | 1.32 (0.74-2.38) | 0.35 | . | . | . |
| DNMT3A ---tag | rs13428812 | A/A | 30 | 50,00 | 15 | 46,88 | 172 | 50,44 | 135 | 44,12 | 1.00 (.-.) | . | 1.80 (0.93-3.49) | 0.08 | 0.22 | 0.97 | 0.54 |
| DNMT3A ---tag |  | A/G or G/G | 30 | 50,00 | 17 | 53,13 | 169 | 49,56 | 171 | 55,88 | 1.96 (0.86-4.43) | 0.11 | 2.08 (1.08-4.03) | 0.03 | . | . | . |
| DNMT3A ---tag | rs4665287 | C/C | 43 | 71,67 | 21 | 65,63 | 213 | 62,46 | 215 | 70,26 | 1.00 (.-.) | . | 1.67 (0.95-2.94) | 0.08 | 0.25 | 0.97 | 0.54 |
| DNMT3A ---tag |  | C/T or T/T | 17 | 28,33 | 11 | 34,38 | 128 | 37,54 | 91 | 29,74 | 1.30 (0.58-2.92) | 0.53 | 1.30 (0.72-2.34) | 0.38 | . | . | . |
| DNMT3B ---tag | rs13045669 | A/A | 56 | 93,33 | 29 | 90,63 | 314 | 92,08 | 285 | 93,14 | 1.00 (.-.) | . | 1.38 (0.88-2.16) | 0.16 | 0.79 | 0.97 | 0.84 |
| DNMT3B ---tag |  | A/G or G/G | 4 | 6,67 | 3 | 9,38 | 27 | 7,92 | 21 | 6,86 | 0.81 (0.18-3.55) | 0.78 | 0.90 (0.44-1.83) | 0.77 | . | . | . |
| DNMT3B ---tag | rs17123673 | A/A | 54 | 90,00 | 29 | 90,63 | 313 | 91,79 | 282 | 92,16 | 1.00 (.-.) | . | 1.40 (0.89-2.21) | 0.15 | 0.80 | 0.97 | 0.84 |
| DNMT3B ---tag |  | A/G or G/G | 6 | 10,00 | 3 | 9,38 | 28 | 8,21 | 24 | 7,84 | 1.08 (0.32-3.67) | 0.90 | 1.27 (0.68-2.40) | 0.45 | . | . | . |
| DNMT3B ---tag | rs183603 | A/A | 30 | 50,00 | 20 | 62,50 | 181 | 53,08 | 166 | 54,25 | 1.00 (.-.) | . | 1.02 (0.59-1.78) | 0.94 | 0.16 | 0.97 | 0.84 |
| DNMT3B ---tag |  | A/G or G/G | 30 | 50,00 | 12 | 37,50 | 160 | 46,92 | 140 | 45,75 | 0.48 (0.21-1.09) | 0.08 | 0.91 (0.52-1.59) | 0.74 | . | . | . |
| DNMT3B ---tag | rs2235760 | C/C | 40 | 66,67 | 22 | 68,75 | 246 | 72,14 | 221 | 72,22 | 1.00 (.-.) | . | 1.48 (0.90-2.44) | 0.12 | 0.53 | 0.97 | 0.84 |
| DNMT3B ---tag |  | C/T or T/T | 20 | 33,33 | 10 | 31,25 | 95 | 27,86 | 85 | 27,78 | 1.27 (0.52-3.13) | 0.60 | 1.38 (0.80-2.38) | 0.24 | . | . | . |
| DNMT3B ---tag | rs2424908 | C/C | 38 | 63,33 | 22 | 68,75 | 212 | 62,17 | 196 | 64,05 | 1.00 (.-.) | . | 1.15 (0.69-1.94) | 0.59 | 0.32 | 0.97 | 0.84 |
| DNMT3B ---tag |  | C/T or T/T | 22 | 36,67 | 10 | 31,25 | 129 | 37,83 | 110 | 35,95 | 0.56 (0.24-1.31) | 0.18 | 1.01 (0.59-1.73) | 0.98 | . | . | . |
| DNMT3B ---candidate literature | rs2424909 | T/T | 19 | 31,67 | 12 | 37,50 | 128 | 37,54 | 129 | 42,16 | 1.00 (.-.) | . | 1.10 (0.58-2.08) | 0.76 | 0.42 | 0.97 | 0.84 |
| DNMT3B ---candidate literature |  | T/C or C/C | 41 | 68,33 | 20 | 62,50 | 213 | 62,46 | 177 | 57,84 | 0.59 (0.26-1.33) | 0.21 | 0.93 (0.49-1.74) | 0.82 | . | . | . |
| DNMT3B ---tag | rs4911108 | A/A | 20 | 33,33 | 13 | 40,63 | 138 | 40,47 | 135 | 44,12 | 1.00 (.-.) | . | 1.10 (0.58-2.08) | 0.77 | 0.42 | 0.97 | 0.84 |
| DNMT3B ---tag |  | A/G or G/G | 40 | 66,67 | 19 | 59,38 | 203 | 59,53 | 171 | 55,88 | 0.61 (0.27-1.36) | 0.22 | 0.95 (0.51-1.79) | 0.88 | . | . | . |
| DNMT3B ---tag | rs6058896 | C/C | 53 | 88,33 | 28 | 87,50 | 304 | 89,15 | 273 | 89,22 | 1.00 (.-.) | . | 1.37 (0.88-2.15) | 0.17 | 0.84 | 0.99 | 0.84 |
| DNMT3B ---tag |  | C/T or T/T | 7 | 11,67 | 4 | 12,50 | 37 | 10,85 | 33 | 10,78 | 1.53 (0.36-6.60) | 0.57 | 1.79 (1.01-3.19) | 0.05 | . | . | . |
| DNMT3B ---tag | rs6119954 | G/G | 36 | 60,00 | 24 | 75,00 | 242 | 70,97 | 220 | 71,90 | 1.00 (.-.) | . | 1.26 (0.78-2.06) | 0.35 | 0.52 | 0.97 | 0.84 |
| DNMT3B ---tag |  | G/A or A/A | 24 | 40,00 | 8 | 25,00 | 99 | 29,03 | 86 | 28,10 | 0.70 (0.27-1.78) | 0.45 | 1.21 (0.71-2.06) | 0.48 | . | . | . |
| DNMT3B ---tag | rs6579038 | A/A | 54 | 90,00 | 29 | 90,63 | 301 | 88,27 | 273 | 89,22 | 1.00 (.-.) | . | 1.39 (0.89-2.18) | 0.15 | 0.69 | 0.97 | 0.84 |
| DNMT3B ---tag |  | A/G or G/G | 6 | 10,00 | 3 | 9,38 | 40 | 11,73 | 33 | 10,78 | 1.65 (0.38-7.12) | 0.50 | 1.67 (0.94-2.98) | 0.08 | . | . | . |
| DPYD ---tag | rs1034215 | C/C | 38 | 63,33 | 19 | 59,38 | 194 | 56,89 | 188 | 61,44 | 1.00 (.-.) | . | 1.86 (1.03-3.38) | 0.04 | 0.10 | 0.97 | 0.61 |
| DPYD ---tag |  | C/T or T/T | 22 | 36,67 | 13 | 40,63 | 147 | 43,11 | 118 | 38,56 | 1.84 (0.81-4.14) | 0.14 | 1.66 (0.90-3.07) | 0.10 | . | . | . |
| DPYD ---tag | rs10783058 | T/T | 31 | 51,67 | 17 | 53,13 | 135 | 39,59 | 118 | 38,56 | 1.00 (.-.) | . | 1.13 (0.61-2.12) | 0.69 | 0.44 | 0.97 | 0.73 |
| DPYD ---tag |  | T/C or C/C | 29 | 48,33 | 15 | 46,88 | 206 | 60,41 | 188 | 61,44 | 0.89 (0.40-1.99) | 0.78 | 1.41 (0.76-2.60) | 0.27 | . | . | . |
| DPYD ---tag | rs10783070 | C/C | 43 | 71,67 | 27 | 84,38 | 252 | 73,90 | 205 | 66,99 | 1.00 (.-.) | . | 1.21 (0.74-1.98) | 0.44 | 0.39 | 0.97 | 0.69 |
| DPYD ---tag |  | C/T or T/T | 17 | 28,33 | 5 | 15,63 | 89 | 26,10 | 101 | 33,01 | 0.89 (0.33-2.42) | 0.83 | 1.68 (1.01-2.81) | 0.05 | . | . | . |
| DPYD ---tag | rs10875048 | G/G | 41 | 68,33 | 22 | 68,75 | 241 | 70,67 | 201 | 65,69 | 1.00 (.-.) | . | 1.23 (0.71-2.11) | 0.46 | 0.52 | 0.97 | 0.73 |
| DPYD ---tag |  | G/A or A/A | 19 | 31,67 | 10 | 31,25 | 100 | 29,33 | 105 | 34,31 | 0.80 (0.36-1.80) | 0.59 | 1.30 (0.74-2.27) | 0.37 | . | . | . |
| DPYD ---tag | rs10875055 | C/C | 30 | 50,00 | 7 | 21,88 | 96 | 28,15 | 64 | 20,92 | 1.00 (.-.) | . | 1.60 (0.72-3.57) | 0.25 | 0.51 | 0.97 | 0.73 |
| DPYD ---tag |  | C/T or T/T | 30 | 50,00 | 25 | 78,13 | 245 | 71,85 | 242 | 79,08 | 2.03 (0.84-4.90) | 0.12 | 2.38 (1.11-5.11) | 0.03 | . | . | . |
| DPYD ---tag | rs10875079 | A/A | 9 | 15,00 | 10 | 31,25 | 88 | 25,81 | 90 | 29,41 | 1.00 (.-.) | . | 1.43 (0.56-3.66) | 0.46 | 0.89 | 0.99 | 0.97 |
| DPYD ---tag |  | A/G or G/G | 51 | 85,00 | 22 | 68,75 | 253 | 74,19 | 216 | 70,59 | 0.84 (0.31-2.29) | 0.73 | 1.11 (0.44-2.80) | 0.82 | . | . | . |
| DPYD ---tag | rs10875085 | A/A | 44 | 73,33 | 19 | 59,38 | 253 | 74,19 | 192 | 62,75 | 1.00 (.-.) | . | 1.61 (0.92-2.83) | 0.10 | 0.34 | 0.97 | 0.69 |
| DPYD ---tag |  | A/T or T/T | 16 | 26,67 | 13 | 40,63 | 88 | 25,81 | 114 | 37,25 | 1.75 (0.78-3.95) | 0.18 | 1.85 (1.04-3.30) | 0.04 | . | . | . |
| DPYD ---tag | rs10875097 | G/G | 43 | 71,67 | 19 | 59,38 | 236 | 69,21 | 203 | 66,34 | 1.00 (.-.) | . | 1.93 (1.08-3.44) | 0.03 | 0.03 | 0.94 | 0.51 |
| DPYD ---tag |  | G/A or A/A | 17 | 28,33 | 13 | 40,63 | 105 | 30,79 | 103 | 33,66 | 2.67 (1.21-5.91) | 0.02 | 2.03 (1.11-3.71) | 0.02 | . | . | . |
| DPYD ---tag | rs11165781 | T/T | 41 | 68,33 | 24 | 75,00 | 244 | 71,55 | 197 | 64,38 | 1.00 (.-.) | . | 1.10 (0.69-1.74) | 0.68 | 0.04 | 0.94 | 0.51 |
| DPYD ---tag |  | T/C or C/C | 19 | 31,67 | 8 | 25,00 | 97 | 28,45 | 109 | 35,62 | 0.34 (0.08-1.45) | 0.15 | 1.37 (0.84-2.22) | 0.21 | . | . | . |
| DPYD ---tag | rs11165783 | T/T | 31 | 51,67 | 20 | 62,50 | 184 | 53,96 | 171 | 55,88 | 1.00 (.-.) | . | 1.54 (0.86-2.78) | 0.15 | 0.56 | 0.97 | 0.73 |
| DPYD ---tag |  | T/C or C/C | 29 | 48,33 | 12 | 37,50 | 157 | 46,04 | 135 | 44,12 | 1.18 (0.53-2.63) | 0.68 | 1.42 (0.79-2.57) | 0.25 | . | . | . |
| DPYD ---tag | rs11165873 | A/A | 12 | 20,00 | 9 | 28,13 | 93 | 27,27 | 94 | 30,72 | 1.00 (.-.) | . | 1.32 (0.59-2.93) | 0.50 | 0.92 | 1.00 | 0.97 |
| DPYD ---tag |  | A/T or T/T | 48 | 80,00 | 23 | 71,88 | 248 | 72,73 | 212 | 69,28 | 0.82 (0.33-2.02) | 0.67 | 1.13 (0.52-2.48) | 0.76 | . | . | . |
| DPYD ---tag | rs11165875 | T/T | 27 | 45,00 | 13 | 40,63 | 141 | 41,35 | 117 | 38,24 | 1.00 (.-.) | . | 1.65 (0.86-3.16) | 0.13 | 0.45 | 0.97 | 0.73 |
| DPYD ---tag |  | T/C or C/C | 33 | 55,00 | 19 | 59,38 | 200 | 58,65 | 189 | 61,76 | 1.30 (0.58-2.95) | 0.52 | 1.54 (0.82-2.90) | 0.18 | . | . | . |
| DPYD ---tag | rs11165881 | T/T | 24 | 40,00 | 15 | 46,88 | 112 | 32,84 | 107 | 34,97 | 1.00 (.-.) | . | 1.06 (0.54-2.09) | 0.86 | 0.35 | 0.97 | 0.69 |
| DPYD ---tag |  | T/C or C/C | 36 | 60,00 | 17 | 53,13 | 229 | 67,16 | 199 | 65,03 | 0.79 (0.34-1.79) | 0.57 | 1.28 (0.66-2.46) | 0.47 | . | . | . |
| DPYD ---tag | rs11587873 | C/C | 33 | 55,00 | 22 | 68,75 | 186 | 54,55 | 188 | 61,44 | 1.00 (.-.) | . | 1.08 (0.64-1.84) | 0.77 | 0.24 | 0.97 | 0.69 |
| DPYD ---tag |  | C/T or T/T | 27 | 45,00 | 10 | 31,25 | 155 | 45,45 | 118 | 38,56 | 0.52 (0.23-1.18) | 0.12 | 0.94 (0.54-1.63) | 0.83 | . | . | . |
| DPYD ---tag | rs12030174 | C/C | 46 | 76,67 | 20 | 62,50 | 266 | 78,01 | 205 | 66,99 | 1.00 (.-.) | . | 1.58 (0.92-2.73) | 0.10 | 0.35 | 0.97 | 0.69 |
| DPYD ---tag |  | C/T or T/T | 14 | 23,33 | 12 | 37,50 | 75 | 21,99 | 101 | 33,01 | 1.65 (0.73-3.74) | 0.23 | 1.72 (0.97-3.04) | 0.06 | . | . | . |
| DPYD ---tag | rs12046744 | A/A | 32 | 53,33 | 17 | 53,13 | 181 | 53,08 | 176 | 57,52 | 1.00 (.-.) | . | 1.80 (1.00-3.23) | 0.05 | 0.13 | 0.97 | 0.61 |
| DPYD ---tag |  | A/C or C/C | 28 | 46,67 | 15 | 46,88 | 160 | 46,92 | 130 | 42,48 | 1.78 (0.80-3.96) | 0.16 | 1.66 (0.91-3.01) | 0.10 | . | . | . |
| DPYD ---tag | rs12047910 | G/G | 44 | 73,33 | 23 | 71,88 | 258 | 75,66 | 223 | 72,88 | 1.00 (.-.) | . | 1.38 (0.82-2.35) | 0.23 | 0.97 | 1.00 | 0.97 |
| DPYD ---tag |  | G/A or A/A | 16 | 26,67 | 9 | 28,13 | 83 | 24,34 | 83 | 27,12 | 1.03 (0.45-2.37) | 0.94 | 1.40 (0.80-2.45) | 0.24 | . | . | . |
| DPYD ---tag | rs12073044 | T/T | 52 | 86,67 | 27 | 84,38 | 261 | 76,54 | 240 | 78,43 | 1.00 (.-.) | . | 1.42 (0.89-2.28) | 0.15 | 0.77 | 0.97 | 0.87 |
| DPYD ---tag |  | T/A or A/A | 8 | 13,33 | 5 | 15,63 | 80 | 23,46 | 66 | 21,57 | 1.08 (0.36-3.24) | 0.89 | 1.29 (0.76-2.20) | 0.34 | . | . | . |
| DPYD ---tag | rs12126093 | T/T | 23 | 38,33 | 14 | 43,75 | 174 | 51,03 | 164 | 53,59 | 1.00 (.-.) | . | 1.30 (0.69-2.46) | 0.41 | 0.86 | 0.99 | 0.96 |
| DPYD ---tag |  | T/C or C/C | 37 | 61,67 | 18 | 56,25 | 167 | 48,97 | 142 | 46,41 | 0.84 (0.37-1.87) | 0.66 | 1.18 (0.62-2.22) | 0.62 | . | . | . |
| DPYD ---tag | rs12134028 | C/C | 51 | 85,00 | 30 | 93,75 | 309 | 90,62 | 273 | 89,22 | 1.00 (.-.) | . | 1.09 (0.70-1.70) | 0.70 | 0.02 | 0.85 | 0.51 |
| DPYD ---tag |  | C/T or T/T | 9 | 15,00 | 2 | 6,25 | 32 | 9,38 | 33 | 10,78 | 0.13 (0.02-0.97) | 0.05 | 0.96 (0.54-1.72) | 0.89 | . | . | . |
| DPYD ---tag | rs12740796 | T/T | 41 | 68,33 | 24 | 75,00 | 253 | 74,19 | 235 | 76,80 | 1.00 (.-.) | . | 1.54 (0.94-2.55) | 0.09 | 0.33 | 0.97 | 0.69 |
| DPYD ---tag |  | T/C or C/C | 19 | 31,67 | 8 | 25,00 | 88 | 25,81 | 71 | 23,20 | 1.53 (0.63-3.73) | 0.35 | 1.45 (0.84-2.51) | 0.18 | . | . | . |
| DPYD ---tag | rs1333717 | A/A | 37 | 61,67 | 18 | 56,25 | 187 | 54,84 | 178 | 58,17 | 1.00 (.-.) | . | 1.84 (1.01-3.34) | 0.05 | 0.12 | 0.97 | 0.61 |
| DPYD ---tag |  | A/G or G/G | 23 | 38,33 | 14 | 43,75 | 154 | 45,16 | 128 | 41,83 | 1.80 (0.80-4.06) | 0.16 | 1.68 (0.92-3.08) | 0.09 | . | . | . |
| DPYD ---tag | rs1413228 | A/A | 49 | 81,67 | 28 | 87,50 | 275 | 80,65 | 237 | 77,45 | 1.00 (.-.) | . | 1.26 (0.80-1.98) | 0.31 | 0.31 | 0.97 | 0.69 |
| DPYD ---tag |  | A/G or G/G | 11 | 18,33 | 4 | 12,50 | 66 | 19,35 | 69 | 22,55 | 0.53 (0.12-2.29) | 0.40 | 1.36 (0.81-2.28) | 0.24 | . | . | . |
| DPYD ---tag | rs1415681 | G/G | 46 | 76,67 | 26 | 81,25 | 240 | 70,38 | 232 | 75,82 | 1.00 (.-.) | . | 1.27 (0.78-2.06) | 0.34 | 0.51 | 0.97 | 0.73 |
| DPYD ---tag |  | G/T or T/T | 14 | 23,33 | 6 | 18,75 | 101 | 29,62 | 74 | 24,18 | 0.74 (0.27-2.01) | 0.56 | 1.32 (0.78-2.24) | 0.30 | . | . | . |
| DPYD ---tag | rs1514495 | C/C | 31 | 51,67 | 19 | 59,38 | 217 | 63,64 | 179 | 58,50 | 1.00 (.-.) | . | 1.14 (0.68-1.93) | 0.61 | 0.26 | 0.97 | 0.69 |
| DPYD ---tag |  | C/T or T/T | 29 | 48,33 | 13 | 40,63 | 124 | 36,36 | 127 | 41,50 | 0.57 (0.23-1.38) | 0.21 | 1.09 (0.64-1.86) | 0.74 | . | . | . |
| DPYD ---tag | rs1520658 | A/A | 46 | 76,67 | 25 | 78,13 | 280 | 82,11 | 251 | 82,03 | 1.00 (.-.) | . | 1.51 (0.92-2.47) | 0.10 | 0.39 | 0.97 | 0.69 |
| DPYD ---tag |  | A/G or G/G | 14 | 23,33 | 7 | 21,88 | 61 | 17,89 | 55 | 17,97 | 1.47 (0.58-3.76) | 0.42 | 1.42 (0.82-2.46) | 0.21 | . | . | . |
| DPYD ---NA | rs17116806 | C/C | 36 | 60,00 | 19 | 59,38 | 228 | 66,86 | 193 | 63,07 | 1.00 (.-.) | . | 1.56 (0.87-2.80) | 0.14 | 0.54 | 0.97 | 0.73 |
| DPYD ---NA |  | C/A or A/A | 24 | 40,00 | 13 | 40,63 | 113 | 33,14 | 113 | 36,93 | 1.41 (0.64-3.13) | 0.40 | 1.69 (0.92-3.10) | 0.09 | . | . | . |
| DPYD ---tag | rs17431828 | G/G | 24 | 40,00 | 14 | 43,75 | 131 | 38,42 | 138 | 45,10 | 1.00 (.-.) | . | 1.21 (0.62-2.37) | 0.57 | 0.64 | 0.97 | 0.81 |
| DPYD ---tag |  | G/C or C/C | 36 | 60,00 | 18 | 56,25 | 210 | 61,58 | 168 | 54,90 | 0.84 (0.37-1.91) | 0.68 | 1.25 (0.64-2.44) | 0.51 | . | . | . |
| DPYD ---tag | rs17471640 | T/T | 24 | 40,00 | 11 | 34,38 | 141 | 41,35 | 152 | 49,67 | 1.00 (.-.) | . | 1.92 (0.92-4.00) | 0.08 | 0.22 | 0.97 | 0.69 |
| DPYD ---tag |  | T/C or C/C | 36 | 60,00 | 21 | 65,63 | 200 | 58,65 | 154 | 50,33 | 1.45 (0.61-3.42) | 0.40 | 1.59 (0.76-3.33) | 0.21 | . | . | . |
| DPYD ---tag | rs17702702 | G/G | 38 | 63,33 | 21 | 65,63 | 235 | 68,91 | 217 | 70,92 | 1.00 (.-.) | . | 1.86 (1.08-3.21) | 0.03 | 0.04 | 0.94 | 0.51 |
| DPYD ---tag |  | G/C or C/C | 22 | 36,67 | 11 | 34,38 | 106 | 31,09 | 89 | 29,08 | 2.25 (0.99-5.09) | 0.05 | 1.61 (0.91-2.84) | 0.10 | . | . | . |
| DPYD ---NA | rs1801265 | T/T | 34 | 56,67 | 22 | 68,75 | 197 | 57,77 | 177 | 57,84 | 1.00 (.-.) | . | 1.38 (0.82-2.32) | 0.23 | 0.97 | 1.00 | 0.97 |
| DPYD ---NA |  | T/C or C/C | 26 | 43,33 | 10 | 31,25 | 144 | 42,23 | 129 | 42,16 | 1.05 (0.45-2.48) | 0.91 | 1.42 (0.84-2.41) | 0.19 | . | . | . |
| DPYD ---tag | rs2039447 | T/T | 26 | 43,33 | 19 | 59,38 | 160 | 46,92 | 150 | 49,02 | 1.00 (.-.) | . | 1.50 (0.85-2.65) | 0.16 | 0.67 | 0.97 | 0.83 |
| DPYD ---tag |  | T/C or C/C | 34 | 56,67 | 13 | 40,63 | 181 | 53,08 | 156 | 50,98 | 1.09 (0.49-2.44) | 0.83 | 1.37 (0.77-2.41) | 0.28 | . | . | . |
| DPYD ---tag | rs2151567 | G/G | 53 | 88,33 | 30 | 93,75 | 312 | 91,50 | 274 | 89,54 | 1.00 (.-.) | . | 1.38 (0.88-2.16) | 0.16 | 0.95 | 1.00 | 0.97 |
| DPYD ---tag |  | G/A or A/A | 7 | 11,67 | 2 | 6,25 | 29 | 8,50 | 32 | 10,46 | 1.32 (0.31-5.70) | 0.71 | 1.75 (0.95-3.22) | 0.08 | . | . | . |
| DPYD ---tag | rs2152878 | A/A | 30 | 50,00 | 21 | 65,63 | 186 | 54,55 | 184 | 60,13 | 1.00 (.-.) | . | 1.17 (0.70-1.98) | 0.55 | 0.35 | 0.97 | 0.69 |
| DPYD ---tag |  | A/G or G/G | 30 | 50,00 | 11 | 34,38 | 155 | 45,45 | 122 | 39,87 | 0.65 (0.28-1.54) | 0.33 | 1.17 (0.68-2.01) | 0.57 | . | . | . |
| DPYD ---tag | rs2786505 | G/G | 46 | 76,67 | 27 | 84,38 | 259 | 75,95 | 221 | 72,22 | 1.00 (.-.) | . | 1.26 (0.77-2.05) | 0.35 | 0.52 | 0.97 | 0.73 |
| DPYD ---tag |  | G/T or T/T | 14 | 23,33 | 5 | 15,63 | 82 | 24,05 | 85 | 27,78 | 0.92 (0.34-2.48) | 0.86 | 1.61 (0.96-2.70) | 0.07 | . | . | . |
| DPYD ---tag | rs2786512 | G/G | 19 | 31,67 | 14 | 43,75 | 129 | 37,83 | 111 | 36,27 | 1.00 (.-.) | . | 1.05 (0.56-1.96) | 0.88 | 0.28 | 0.97 | 0.69 |
| DPYD ---tag |  | G/A or A/A | 41 | 68,33 | 18 | 56,25 | 212 | 62,17 | 195 | 63,73 | 0.70 (0.32-1.56) | 0.39 | 1.17 (0.64-2.15) | 0.60 | . | . | . |
| DPYD ---tag | rs2786519 | A/A | 39 | 65,00 | 17 | 53,13 | 213 | 62,46 | 176 | 57,52 | 1.00 (.-.) | . | 1.86 (1.01-3.41) | 0.05 | 0.10 | 0.97 | 0.61 |
| DPYD ---tag |  | A/G or G/G | 21 | 35,00 | 15 | 46,88 | 128 | 37,54 | 130 | 42,48 | 2.27 (1.01-5.07) | 0.05 | 2.06 (1.11-3.82) | 0.02 | . | . | . |
| DPYD ---tag | rs2811170 | A/A | 47 | 78,33 | 21 | 65,63 | 243 | 71,26 | 241 | 78,76 | 1.00 (.-.) | . | 1.63 (0.97-2.75) | 0.07 | 0.18 | 0.97 | 0.69 |
| DPYD ---tag |  | A/T or T/T | 13 | 21,67 | 11 | 34,38 | 98 | 28,74 | 65 | 21,24 | 1.35 (0.58-3.16) | 0.49 | 1.16 (0.65-2.08) | 0.61 | . | . | . |
| DPYD ---tag | rs2811199 | G/G | 44 | 73,33 | 27 | 84,38 | 254 | 74,49 | 212 | 69,28 | 1.00 (.-.) | . | 1.24 (0.76-2.02) | 0.38 | 0.47 | 0.97 | 0.73 |
| DPYD ---tag |  | G/A or A/A | 16 | 26,67 | 5 | 15,63 | 87 | 25,51 | 94 | 30,72 | 0.89 (0.33-2.42) | 0.83 | 1.61 (0.96-2.69) | 0.07 | . | . | . |
| DPYD ---tag | rs2811219 | T/T | 35 | 58,33 | 19 | 59,38 | 185 | 54,25 | 176 | 57,52 | 1.00 (.-.) | . | 1.45 (0.82-2.56) | 0.20 | 0.77 | 0.97 | 0.87 |
| DPYD ---tag |  | T/C or C/C | 25 | 41,67 | 13 | 40,63 | 156 | 45,75 | 130 | 42,48 | 1.05 (0.47-2.36) | 0.90 | 1.35 (0.76-2.39) | 0.31 | . | . | . |
| DPYD ---tag | rs4300257 | A/A | 43 | 71,67 | 17 | 53,13 | 235 | 68,91 | 181 | 59,15 | 1.00 (.-.) | . | 1.83 (1.02-3.27) | 0.04 | 0.09 | 0.97 | 0.61 |
| DPYD ---tag |  | A/C or C/C | 17 | 28,33 | 15 | 46,88 | 106 | 31,09 | 125 | 40,85 | 2.08 (0.93-4.64) | 0.07 | 1.81 (1.00-3.28) | 0.05 | . | . | . |
| DPYD ---tag | rs4379706 | T/T | 34 | 56,67 | 22 | 68,75 | 191 | 56,01 | 177 | 57,84 | 1.00 (.-.) | . | 1.39 (0.83-2.34) | 0.21 | 0.92 | 1.00 | 0.97 |
| DPYD ---tag |  | T/C or C/C | 26 | 43,33 | 10 | 31,25 | 150 | 43,99 | 129 | 42,16 | 1.05 (0.45-2.48) | 0.91 | 1.40 (0.82-2.38) | 0.21 | . | . | . |
| DPYD ---tag | rs4950021 | T/T | 22 | 36,67 | 11 | 34,38 | 106 | 31,09 | 90 | 29,41 | 1.00 (.-.) | . | 1.49 (0.77-2.89) | 0.24 | 0.73 | 0.97 | 0.87 |
| DPYD ---tag |  | T/G or G/G | 38 | 63,33 | 21 | 65,63 | 235 | 68,91 | 216 | 70,59 | 1.17 (0.53-2.61) | 0.70 | 1.50 (0.80-2.82) | 0.21 | . | . | . |
| DPYD ---tag | rs4950033 | T/T | 23 | 38,33 | 9 | 28,13 | 92 | 26,98 | 90 | 29,41 | 1.00 (.-.) | . | 1.81 (0.85-3.83) | 0.12 | 0.38 | 0.97 | 0.69 |
| DPYD ---tag |  | T/C or C/C | 37 | 61,67 | 23 | 71,88 | 249 | 73,02 | 216 | 70,59 | 1.27 (0.54-3.00) | 0.58 | 1.54 (0.75-3.19) | 0.24 | . | . | . |
| DPYD ---tag | rs495257 | T/T | 23 | 38,33 | 17 | 53,13 | 119 | 34,90 | 94 | 30,72 | 1.00 (.-.) | . | 1.19 (0.65-2.18) | 0.58 | 0.55 | 0.97 | 0.73 |
| DPYD ---tag |  | T/C or C/C | 37 | 61,67 | 15 | 46,88 | 222 | 65,10 | 212 | 69,28 | 0.85 (0.38-1.89) | 0.69 | 1.30 (0.73-2.34) | 0.37 | . | . | . |
| DPYD ---tag | rs552926 | A/A | 26 | 43,33 | 15 | 46,88 | 119 | 34,90 | 99 | 32,35 | 1.00 (.-.) | . | 1.08 (0.56-2.07) | 0.81 | 0.39 | 0.97 | 0.69 |
| DPYD ---tag |  | A/G or G/G | 34 | 56,67 | 17 | 53,13 | 222 | 65,10 | 207 | 67,65 | 0.85 (0.38-1.91) | 0.69 | 1.34 (0.72-2.53) | 0.36 | . | . | . |
| DPYD ---tag | rs628959 | A/A | 24 | 40,00 | 16 | 50,00 | 167 | 48,97 | 152 | 49,67 | 1.00 (.-.) | . | 1.07 (0.57-1.98) | 0.84 | 0.31 | 0.97 | 0.69 |
| DPYD ---tag |  | A/G or G/G | 36 | 60,00 | 16 | 50,00 | 174 | 51,03 | 154 | 50,33 | 0.65 (0.29-1.44) | 0.29 | 1.07 (0.57-1.99) | 0.84 | . | . | . |
| DPYD ---tag | rs6656660 | G/G | 45 | 75,00 | 28 | 87,50 | 266 | 78,01 | 230 | 75,16 | 1.00 (.-.) | . | 1.23 (0.78-1.94) | 0.36 | 0.22 | 0.97 | 0.69 |
| DPYD ---tag |  | G/T or T/T | 15 | 25,00 | 4 | 12,50 | 75 | 21,99 | 76 | 24,84 | 0.45 (0.10-1.94) | 0.28 | 1.30 (0.78-2.15) | 0.32 | . | . | . |
| DPYD ---tag | rs6663670 | A/A | 44 | 73,33 | 25 | 78,13 | 254 | 74,49 | 211 | 68,95 | 1.00 (.-.) | . | 1.30 (0.79-2.14) | 0.31 | 0.73 | 0.97 | 0.87 |
| DPYD ---tag |  | A/C or C/C | 16 | 26,67 | 7 | 21,88 | 87 | 25,51 | 95 | 31,05 | 1.10 (0.43-2.81) | 0.84 | 1.70 (1.00-2.87) | 0.05 | . | . | . |
| DPYD ---tag | rs6683883 | T/T | 30 | 50,00 | 10 | 31,25 | 132 | 38,71 | 102 | 33,33 | 1.00 (.-.) | . | 1.65 (0.75-3.62) | 0.21 | 0.57 | 0.97 | 0.73 |
| DPYD ---tag |  | T/C or C/C | 30 | 50,00 | 22 | 68,75 | 209 | 61,29 | 204 | 66,67 | 1.65 (0.68-4.03) | 0.27 | 2.08 (0.96-4.50) | 0.06 | . | . | . |
| DPYD ---tag | rs6686861 | C/C | 52 | 86,67 | 30 | 93,75 | 299 | 87,68 | 259 | 84,64 | 1.00 (.-.) | . | 1.12 (0.72-1.74) | 0.62 | 0.01 | 0.85 | 0.51 |
| DPYD ---tag |  | C/T or T/T | 8 | 13,33 | 2 | 6,25 | 42 | 12,32 | 47 | 15,36 | 0.16 (0.02-1.23) | 0.08 | 1.38 (0.81-2.35) | 0.24 | . | . | . |
| DPYD ---tag | rs7414210 | A/A | 42 | 70,00 | 27 | 84,38 | 235 | 68,91 | 231 | 75,49 | 1.00 (.-.) | . | 1.55 (0.98-2.46) | 0.06 | 0.36 | 0.97 | 0.69 |
| DPYD ---tag |  | A/C or C/C | 18 | 30,00 | 5 | 15,63 | 106 | 31,09 | 75 | 24,51 | 1.40 (0.40-4.86) | 0.59 | 1.15 (0.69-1.91) | 0.60 | . | . | . |
| DPYD ---tag | rs7530858 | A/A | 47 | 78,33 | 29 | 90,63 | 272 | 79,77 | 229 | 74,84 | 1.00 (.-.) | . | 1.23 (0.77-1.95) | 0.39 | 0.33 | 0.97 | 0.69 |
| DPYD ---tag |  | A/G or G/G | 13 | 21,67 | 3 | 9,38 | 69 | 20,23 | 77 | 25,16 | 0.71 (0.21-2.43) | 0.59 | 1.59 (0.96-2.64) | 0.07 | . | . | . |
| DPYD ---tag | rs7544128 | C/C | 34 | 56,67 | 14 | 43,75 | 201 | 58,94 | 158 | 51,63 | 1.00 (.-.) | . | 1.98 (1.05-3.73) | 0.04 | 0.07 | 0.97 | 0.61 |
| DPYD ---tag |  | C/G or G/G | 26 | 43,33 | 18 | 56,25 | 140 | 41,06 | 148 | 48,37 | 2.15 (0.96-4.84) | 0.06 | 1.95 (1.03-3.68) | 0.04 | . | . | . |
| DPYD ---tag | rs7545340 | G/G | 35 | 58,33 | 14 | 43,75 | 194 | 56,89 | 172 | 56,21 | 1.00 (.-.) | . | 1.82 (0.97-3.43) | 0.06 | 0.18 | 0.97 | 0.69 |
| DPYD ---tag |  | G/A or A/A | 25 | 41,67 | 18 | 56,25 | 147 | 43,11 | 134 | 43,79 | 1.72 (0.78-3.83) | 0.18 | 1.78 (0.94-3.37) | 0.08 | . | . | . |
| DPYD ---tag | rs828054 | C/C | 7 | 11,67 | 6 | 18,75 | 77 | 22,58 | 91 | 29,74 | 1.00 (.-.) | . | 0.95 (0.36-2.48) | 0.92 | 0.46 | 0.97 | 0.73 |
| DPYD ---tag |  | C/A or A/A | 53 | 88,33 | 26 | 81,25 | 264 | 77,42 | 215 | 70,26 | 0.56 (0.20-1.56) | 0.27 | 0.80 (0.31-2.07) | 0.65 | . | . | . |
| DPYD ---tag | rs885622 | G/G | 34 | 56,67 | 11 | 34,38 | 136 | 39,88 | 104 | 33,99 | 1.00 (.-.) | . | 1.80 (0.89-3.63) | 0.10 | 0.22 | 0.97 | 0.69 |
| DPYD ---tag |  | G/A or A/A | 26 | 43,33 | 21 | 65,63 | 205 | 60,12 | 202 | 66,01 | 2.23 (0.96-5.17) | 0.06 | 2.31 (1.17-4.58) | 0.02 | . | . | . |
| DPYD ---tag | rs9437663 | G/G | 40 | 66,67 | 23 | 71,88 | 225 | 65,98 | 187 | 61,11 | 1.00 (.-.) | . | 1.13 (0.70-1.80) | 0.62 | 0.11 | 0.97 | 0.61 |
| DPYD ---tag |  | G/A or A/A | 20 | 33,33 | 9 | 28,13 | 116 | 34,02 | 119 | 38,89 | 0.52 (0.15-1.76) | 0.29 | 1.48 (0.90-2.42) | 0.12 | . | . | . |
| DPYS ---tag | rs13249169 | A/A | 47 | 78,33 | 28 | 87,50 | 271 | 79,47 | 235 | 76,80 | 1.00 (.-.) | . | 1.23 (0.77-1.97) | 0.38 | 0.32 | 0.97 | 0.98 |
| DPYS ---tag |  | A/T or T/T | 13 | 21,67 | 4 | 12,50 | 70 | 20,53 | 71 | 23,20 | 0.65 (0.22-1.92) | 0.44 | 1.39 (0.82-2.34) | 0.22 | . | . | . |
| DPYS ---NA | rs13263121 | T/T | 25 | 41,67 | 16 | 50,00 | 153 | 44,87 | 139 | 45,42 | 1.00 (.-.) | . | 1.22 (0.65-2.30) | 0.53 | 0.63 | 0.97 | 0.98 |
| DPYS ---NA |  | T/A or A/A | 35 | 58,33 | 16 | 50,00 | 188 | 55,13 | 167 | 54,58 | 0.77 (0.34-1.71) | 0.51 | 1.15 (0.62-2.14) | 0.66 | . | . | . |
| DPYS ---tag | rs16871361 | T/T | 55 | 91,67 | 29 | 90,63 | 301 | 88,27 | 269 | 87,91 | 1.00 (.-.) | . | 1.37 (0.87-2.17) | 0.18 | 0.98 | 1.00 | 0.98 |
| DPYS ---tag |  | T/C or C/C | 5 | 8,33 | 3 | 9,38 | 40 | 11,73 | 37 | 12,09 | 0.96 (0.28-3.28) | 0.95 | 1.35 (0.76-2.38) | 0.31 | . | . | . |
| DPYS ---NA | rs17245950 | T/T | 49 | 81,67 | 25 | 78,13 | 259 | 75,95 | 237 | 77,45 | 1.00 (.-.) | . | 1.46 (0.90-2.36) | 0.12 | 0.58 | 0.97 | 0.98 |
| DPYS ---NA |  | T/A or A/A | 11 | 18,33 | 7 | 21,88 | 82 | 24,05 | 69 | 22,55 | 1.27 (0.46-3.46) | 0.65 | 1.36 (0.79-2.32) | 0.27 | . | . | . |
| DPYS ---NA | rs2253336 | A/A | 51 | 85,00 | 27 | 84,38 | 264 | 77,42 | 250 | 81,70 | 1.00 (.-.) | . | 1.47 (0.92-2.33) | 0.10 | 0.65 | 0.97 | 0.98 |
| DPYS ---NA |  | A/G or G/G | 9 | 15,00 | 5 | 15,63 | 77 | 22,58 | 56 | 18,30 | 1.02 (0.29-3.56) | 0.98 | 1.10 (0.64-1.88) | 0.73 | . | . | . |
| DPYS ---tag | rs2280010 | C/C | 36 | 60,00 | 11 | 34,38 | 206 | 60,41 | 169 | 55,23 | 1.00 (.-.) | . | 1.38 (0.71-2.69) | 0.34 | 0.88 | 0.99 | 0.98 |
| DPYS ---tag |  | C/T or T/T | 24 | 40,00 | 21 | 65,63 | 135 | 39,59 | 137 | 44,77 | 1.37 (0.60-3.09) | 0.45 | 2.02 (1.04-3.93) | 0.04 | . | . | . |
| DPYS ---tag | rs2333874 | T/T | 23 | 38,33 | 14 | 43,75 | 144 | 42,23 | 131 | 42,81 | 1.00 (.-.) | . | 1.79 (0.89-3.58) | 0.10 | 0.31 | 0.97 | 0.98 |
| DPYS ---tag |  | T/G or G/G | 37 | 61,67 | 18 | 56,25 | 197 | 57,77 | 175 | 57,19 | 1.40 (0.61-3.22) | 0.43 | 1.60 (0.80-3.19) | 0.19 | . | . | . |
| DPYS ---NA | rs2669429 | C/C | 15 | 25,00 | 8 | 25,00 | 113 | 33,14 | 87 | 28,43 | 1.00 (.-.) | . | 0.77 (0.35-1.73) | 0.53 | 0.12 | 0.97 | 0.98 |
| DPYS ---NA |  | C/T or T/T | 45 | 75,00 | 24 | 75,00 | 228 | 66,86 | 219 | 71,57 | 0.65 (0.27-1.58) | 0.34 | 1.09 (0.50-2.36) | 0.82 | . | . | . |
| DPYS ---tag | rs2669434 | C/C | 33 | 55,00 | 20 | 62,50 | 164 | 48,09 | 164 | 53,59 | 1.00 (.-.) | . | 1.35 (0.78-2.34) | 0.28 | 0.90 | 1.00 | 0.98 |
| DPYS ---tag |  | C/A or A/A | 27 | 45,00 | 12 | 37,50 | 177 | 51,91 | 142 | 46,41 | 0.81 (0.36-1.82) | 0.61 | 1.15 (0.66-2.02) | 0.62 | . | . | . |
| DPYS ---tag | rs2853142 | T/T | 28 | 46,67 | 14 | 43,75 | 125 | 36,66 | 127 | 41,50 | 1.00 (.-.) | . | 1.79 (0.94-3.41) | 0.08 | 0.30 | 0.97 | 0.98 |
| DPYS ---tag |  | T/C or C/C | 32 | 53,33 | 18 | 56,25 | 216 | 63,34 | 179 | 58,50 | 1.23 (0.55-2.78) | 0.61 | 1.40 (0.74-2.65) | 0.30 | . | . | . |
| DPYS ---NA | rs2853145 | A/A | 45 | 75,00 | 23 | 71,88 | 223 | 65,40 | 208 | 67,97 | 1.00 (.-.) | . | 1.55 (0.93-2.56) | 0.09 | 0.47 | 0.97 | 0.98 |
| DPYS ---NA |  | A/C or C/C | 15 | 25,00 | 9 | 28,13 | 118 | 34,60 | 98 | 32,03 | 1.13 (0.46-2.81) | 0.79 | 1.23 (0.72-2.08) | 0.45 | . | . | . |
| DPYS ---tag | rs2853149 | G/G | 15 | 25,00 | 7 | 21,88 | 89 | 26,10 | 85 | 27,78 | 1.00 (.-.) | . | 1.28 (0.51-3.22) | 0.60 | 0.88 | 0.99 | 0.98 |
| DPYS ---tag |  | G/A or A/A | 45 | 75,00 | 25 | 78,13 | 252 | 73,90 | 221 | 72,22 | 0.85 (0.32-2.30) | 0.76 | 1.18 (0.48-2.93) | 0.72 | . | . | . |
| DPYS ---tag | rs2853154 | T/T | 37 | 61,67 | 23 | 71,88 | 190 | 55,72 | 175 | 57,19 | 1.00 (.-.) | . | 1.46 (0.87-2.44) | 0.15 | 0.80 | 0.97 | 0.98 |
| DPYS ---tag |  | T/C or C/C | 23 | 38,33 | 9 | 28,13 | 151 | 44,28 | 131 | 42,81 | 1.00 (0.41-2.41) | 0.99 | 1.28 (0.76-2.15) | 0.35 | . | . | . |
| DPYS ---tag | rs2853161 | A/A | 20 | 33,33 | 3 | 9,38 | 96 | 28,15 | 77 | 25,16 | 1.00 (.-.) | . | 2.74 (0.84-8.90) | 0.09 | 0.16 | 0.97 | 0.98 |
| DPYS ---tag |  | A/G or G/G | 40 | 66,67 | 29 | 90,63 | 245 | 71,85 | 229 | 74,84 | 2.47 (0.73-8.38) | 0.15 | 2.94 (0.92-9.36) | 0.07 | . | . | . |
| DPYS ---NA | rs2959024 | T/T | 35 | 58,33 | 12 | 37,50 | 180 | 52,79 | 144 | 47,06 | 1.00 (.-.) | . | 1.19 (0.63-2.23) | 0.59 | 0.53 | 0.97 | 0.98 |
| DPYS ---NA |  | T/G or G/G | 25 | 41,67 | 20 | 62,50 | 161 | 47,21 | 162 | 52,94 | 1.04 (0.47-2.33) | 0.92 | 1.62 (0.87-3.03) | 0.13 | . | . | . |
| DPYS ---NA | rs2959025 | A/A | 26 | 43,33 | 12 | 37,50 | 148 | 43,40 | 119 | 38,89 | 1.00 (.-.) | . | 1.30 (0.69-2.47) | 0.41 | 0.86 | 0.99 | 0.98 |
| DPYS ---NA |  | A/G or G/G | 34 | 56,67 | 20 | 62,50 | 193 | 56,60 | 187 | 61,11 | 1.07 (0.48-2.39) | 0.87 | 1.50 (0.81-2.80) | 0.20 | . | . | . |
| DPYS ---tag | rs2959026 | G/G | 20 | 33,33 | 10 | 31,25 | 120 | 35,19 | 108 | 35,29 | 1.00 (.-.) | . | 1.36 (0.62-2.99) | 0.44 | 0.97 | 1.00 | 0.98 |
| DPYS ---tag |  | G/A or A/A | 40 | 66,67 | 22 | 68,75 | 221 | 64,81 | 198 | 64,71 | 1.01 (0.42-2.46) | 0.98 | 1.40 (0.65-3.03) | 0.39 | . | . | . |
| DPYS ---NA | rs3133278 | T/T | 31 | 51,67 | 19 | 59,38 | 176 | 51,61 | 153 | 50,00 | 1.00 (.-.) | . | 1.40 (0.78-2.51) | 0.26 | 0.96 | 1.00 | 0.98 |
| DPYS ---NA |  | T/C or C/C | 29 | 48,33 | 13 | 40,63 | 165 | 48,39 | 153 | 50,00 | 0.95 (0.43-2.11) | 0.90 | 1.30 (0.73-2.30) | 0.37 | . | . | . |
| DPYS ---tag | rs3750187 | G/G | 40 | 66,67 | 22 | 68,75 | 214 | 62,76 | 191 | 62,42 | 1.00 (.-.) | . | 1.39 (0.82-2.36) | 0.22 | 0.94 | 1.00 | 0.98 |
| DPYS ---tag |  | G/A or A/A | 20 | 33,33 | 10 | 31,25 | 127 | 37,24 | 115 | 37,58 | 1.01 (0.43-2.38) | 0.98 | 1.36 (0.79-2.32) | 0.27 | . | . | . |
| DPYS ---tag | rs3793357 | T/T | 53 | 88,33 | 29 | 90,63 | 299 | 87,68 | 277 | 90,52 | 1.00 (.-.) | . | 1.28 (0.81-2.01) | 0.29 | 0.48 | 0.97 | 0.98 |
| DPYS ---tag |  | T/G or G/G | 7 | 11,67 | 3 | 9,38 | 42 | 12,32 | 29 | 9,48 | 0.56 (0.16-1.95) | 0.36 | 1.15 (0.60-2.17) | 0.68 | . | . | . |
| DPYS ---tag | rs3793358 | G/G | 51 | 85,00 | 27 | 84,38 | 260 | 76,25 | 243 | 79,41 | 1.00 (.-.) | . | 1.51 (0.95-2.40) | 0.08 | 0.57 | 0.97 | 0.98 |
| DPYS ---tag |  | G/A or A/A | 9 | 15,00 | 5 | 15,63 | 81 | 23,75 | 63 | 20,59 | 1.07 (0.32-3.65) | 0.91 | 1.11 (0.66-1.86) | 0.70 | . | . | . |
| DPYS ---tag | rs6468924 | C/C | 39 | 65,00 | 22 | 68,75 | 206 | 60,41 | 190 | 62,09 | 1.00 (.-.) | . | 1.31 (0.78-2.20) | 0.31 | 0.75 | 0.97 | 0.98 |
| DPYS ---tag |  | C/T or T/T | 21 | 35,00 | 10 | 31,25 | 135 | 39,59 | 116 | 37,91 | 0.82 (0.35-1.94) | 0.66 | 1.25 (0.73-2.14) | 0.42 | . | . | . |
| DUT ---tag | rs8025164 | G/G | 45 | 75,00 | 21 | 65,63 | 221 | 64,81 | 223 | 72,88 | 1.00 (.-.) | . | 1.68 (1.00-2.80) | 0.05 | 0.12 | 0.97 | 0.12 |
| DUT ---tag |  | G/A or A/A | 15 | 25,00 | 11 | 34,38 | 120 | 35,19 | 83 | 27,12 | 1.56 (0.66-3.68) | 0.31 | 1.25 (0.72-2.17) | 0.44 | . | . | . |
| EHMT1 ---tag | rs10780190 | C/C | 56 | 93,33 | 28 | 87,50 | 304 | 89,15 | 274 | 89,54 | 1.00 (.-.) | . | 1.46 (0.93-2.28) | 0.10 | 0.16 | 0.97 | 0.31 |
| EHMT1 ---tag |  | C/T or T/T | 4 | 6,67 | 4 | 12,50 | 37 | 10,85 | 32 | 10,46 | 3.33 (0.72-15.40) | 0.12 | 1.30 (0.70-2.41) | 0.41 | . | . | . |
| EHMT1 ---tag | rs10867083 | G/G | 24 | 40,00 | 16 | 50,00 | 160 | 46,92 | 144 | 47,06 | 1.00 (.-.) | . | 0.95 (0.50-1.81) | 0.88 | 0.17 | 0.97 | 0.31 |
| EHMT1 ---tag |  | G/A or A/A | 36 | 60,00 | 16 | 50,00 | 181 | 53,08 | 162 | 52,94 | 0.56 (0.25-1.27) | 0.17 | 0.98 (0.52-1.84) | 0.95 | . | . | . |
| EHMT1 ---tag | rs11137190 | C/C | 33 | 55,00 | 16 | 50,00 | 177 | 51,91 | 157 | 51,31 | 1.00 (.-.) | . | 1.61 (0.89-2.90) | 0.12 | 0.35 | 0.97 | 0.50 |
| EHMT1 ---tag |  | C/G or G/G | 27 | 45,00 | 16 | 50,00 | 164 | 48,09 | 149 | 48,69 | 1.66 (0.74-3.71) | 0.22 | 1.77 (0.99-3.18) | 0.06 | . | . | . |
| EHMT1 ---tag | rs3123510 | G/G | 26 | 43,33 | 9 | 28,13 | 131 | 38,42 | 117 | 38,24 | 1.00 (.-.) | . | 2.23 (1.10-4.50) | 0.03 | 0.04 | 0.94 | 0.26 |
| EHMT1 ---tag |  | G/A or A/A | 34 | 56,67 | 23 | 71,88 | 210 | 61,58 | 189 | 61,76 | 2.32 (1.01-5.36) | 0.05 | 2.12 (1.07-4.21) | 0.03 | . | . | . |
| EHMT1 ---candidate literature | rs3125795 | G/G | 55 | 91,67 | 28 | 87,50 | 304 | 89,15 | 274 | 89,54 | 1.00 (.-.) | . | 1.46 (0.93-2.28) | 0.10 | 0.15 | 0.97 | 0.31 |
| EHMT1 ---candidate literature |  | G/T or T/T | 5 | 8,33 | 4 | 12,50 | 37 | 10,85 | 32 | 10,46 | 3.33 (0.72-15.39) | 0.12 | 1.29 (0.70-2.40) | 0.42 | . | . | . |
| EHMT1 ---tag | rs4573359 | G/G | 49 | 81,67 | 29 | 90,63 | 286 | 83,87 | 259 | 84,64 | 1.00 (.-.) | . | 1.34 (0.86-2.09) | 0.20 | 0.44 | 0.97 | 0.50 |
| EHMT1 ---tag |  | G/T or T/T | 11 | 18,33 | 3 | 9,38 | 55 | 16,13 | 47 | 15,36 | 0.41 (0.05-3.08) | 0.39 | 1.14 (0.68-1.94) | 0.62 | . | . | . |
| EHMT1 ---candidate literature | rs4634736 | G/G | 49 | 81,67 | 29 | 90,63 | 285 | 83,58 | 260 | 84,97 | 1.00 (.-.) | . | 1.34 (0.86-2.10) | 0.19 | 0.45 | 0.97 | 0.50 |
| EHMT1 ---candidate literature |  | G/A or A/A | 11 | 18,33 | 3 | 9,38 | 56 | 16,42 | 46 | 15,03 | 0.41 (0.05-3.08) | 0.39 | 1.12 (0.66-1.90) | 0.67 | . | . | . |
| EHMT1 ---tag | rs4876902 | C/C | 45 | 75,00 | 16 | 50,00 | 214 | 62,76 | 191 | 62,42 | 1.00 (.-.) | . | 1.94 (1.06-3.54) | 0.03 | 0.05 | 0.94 | 0.26 |
| EHMT1 ---tag |  | C/T or T/T | 15 | 25,00 | 16 | 50,00 | 127 | 37,24 | 115 | 37,58 | 2.70 (1.22-5.99) | 0.01 | 2.21 (1.20-4.10) | 0.01 | . | . | . |
| EHMT1 ---tag | rs4876904 | T/T | 20 | 33,33 | 11 | 34,38 | 95 | 27,86 | 94 | 30,72 | 1.00 (.-.) | . | 1.51 (0.71-3.20) | 0.28 | 0.76 | 0.97 | 0.76 |
| EHMT1 ---tag |  | T/G or G/G | 40 | 66,67 | 21 | 65,63 | 246 | 72,14 | 212 | 69,28 | 1.03 (0.43-2.43) | 0.95 | 1.35 (0.65-2.82) | 0.43 | . | . | . |
| EHMT1 ---tag | rs7390244 | A/A | 15 | 25,00 | 8 | 25,00 | 79 | 23,17 | 80 | 26,14 | 1.00 (.-.) | . | 1.81 (0.77-4.27) | 0.18 | 0.44 | 0.97 | 0.50 |
| EHMT1 ---tag |  | A/G or G/G | 45 | 75,00 | 24 | 75,00 | 262 | 76,83 | 226 | 73,86 | 1.43 (0.56-3.67) | 0.46 | 1.77 (0.77-4.10) | 0.18 | . | . | . |
| EHMT1 ---tag | rs9314635 | G/G | 30 | 50,00 | 12 | 37,50 | 143 | 41,94 | 121 | 39,54 | 1.00 (.-.) | . | 1.79 (0.94-3.39) | 0.08 | 0.17 | 0.97 | 0.31 |
| EHMT1 ---tag |  | G/T or T/T | 30 | 50,00 | 20 | 62,50 | 198 | 58,06 | 185 | 60,46 | 1.99 (0.89-4.44) | 0.09 | 1.96 (1.05-3.65) | 0.04 | . | . | . |
| EHMT2 ---candidate/tag | rs2736428 | G/G | 21 | 35,00 | 12 | 37,50 | 127 | 37,24 | 139 | 45,42 | 1.00 (.-.) | . | 1.28 (0.64-2.57) | 0.48 | 0.88 | 0.99 | 0.88 |
| EHMT2 ---candidate/tag |  | G/A or A/A | 39 | 65,00 | 20 | 62,50 | 214 | 62,76 | 167 | 54,58 | 0.73 (0.32-1.67) | 0.45 | 1.00 (0.50-1.98) | 0.99 | . | . | . |
| EHMT2 ---tag | rs9267649 | G/G | 47 | 78,33 | 24 | 75,00 | 246 | 72,14 | 219 | 71,57 | 1.00 (.-.) | . | 1.47 (0.89-2.43) | 0.13 | 0.46 | 0.97 | 0.88 |
| EHMT2 ---tag |  | G/A or A/A | 13 | 21,67 | 8 | 25,00 | 95 | 27,86 | 87 | 28,43 | 1.70 (0.70-4.14) | 0.24 | 1.74 (1.02-2.97) | 0.04 | . | . | . |
| FDXR ---NA | rs2070918 | T/T | 24 | 40,00 | 13 | 40,63 | 167 | 48,97 | 155 | 50,65 | 1.00 (.-.) | . | 1.65 (0.83-3.30) | 0.15 | 0.47 | 0.97 | 0.88 |
| FDXR ---NA |  | T/C or C/C | 36 | 60,00 | 19 | 59,38 | 174 | 51,03 | 151 | 49,35 | 1.66 (0.72-3.81) | 0.24 | 1.99 (1.00-3.96) | 0.05 | . | . | . |
| FDXR ---tag | rs509911 | A/A | 33 | 55,00 | 21 | 65,63 | 217 | 63,64 | 197 | 64,38 | 1.00 (.-.) | . | 1.38 (0.81-2.35) | 0.24 | 0.88 | 0.99 | 0.88 |
| FDXR ---tag |  | A/G or G/G | 27 | 45,00 | 11 | 34,38 | 124 | 36,36 | 109 | 35,62 | 1.43 (0.62-3.29) | 0.40 | 1.84 (1.07-3.19) | 0.03 | . | . | . |
| FDXR ---NA | rs689882 | G/G | 23 | 38,33 | 20 | 62,50 | 181 | 53,08 | 166 | 54,25 | 1.00 (.-.) | . | 1.08 (0.62-1.89) | 0.78 | 0.22 | 0.97 | 0.88 |
| FDXR ---NA |  | G/A or A/A | 37 | 61,67 | 12 | 37,50 | 160 | 46,92 | 140 | 45,75 | 0.68 (0.30-1.52) | 0.35 | 1.24 (0.71-2.17) | 0.45 | . | . | . |
| FDXR ---NA | rs689895 | G/G | 30 | 50,00 | 18 | 56,25 | 170 | 49,85 | 153 | 50,00 | 1.00 (.-.) | . | 1.46 (0.82-2.60) | 0.19 | 0.67 | 0.97 | 0.88 |
| FDXR ---NA |  | G/C or C/C | 30 | 50,00 | 14 | 43,75 | 171 | 50,15 | 153 | 50,00 | 1.29 (0.57-2.88) | 0.54 | 1.57 (0.89-2.75) | 0.12 | . | . | . |
| FOLH1 ---candidate literature | rs10839236 | T/T | 27 | 45,00 | 9 | 28,13 | 144 | 42,23 | 122 | 39,87 | 1.00 (.-.) | . | 1.75 (0.74-4.10) | 0.20 | 0.50 | 0.97 | 0.71 |
| FOLH1 ---candidate literature |  | T/C or C/C | 33 | 55,00 | 23 | 71,88 | 197 | 57,77 | 184 | 60,13 | 1.34 (0.53-3.42) | 0.54 | 1.68 (0.72-3.90) | 0.23 | . | . | . |
| FOLH1 ---tag | rs16906190 | A/A | 48 | 80,00 | 26 | 81,25 | 275 | 80,65 | 258 | 84,31 | 1.00 (.-.) | . | 1.44 (0.89-2.32) | 0.14 | 0.64 | 0.97 | 0.71 |
| FOLH1 ---tag |  | A/G or G/G | 12 | 20,00 | 6 | 18,75 | 66 | 19,35 | 48 | 15,69 | 1.06 (0.38-2.94) | 0.91 | 1.17 (0.66-2.07) | 0.59 | . | . | . |
| FOLH1 ---candidate | rs202676 | T/T | 38 | 63,33 | 18 | 56,25 | 213 | 62,46 | 192 | 62,75 | 1.00 (.-.) | . | 1.60 (0.86-2.96) | 0.14 | 0.47 | 0.97 | 0.71 |
| FOLH1 ---candidate |  | T/C or C/C | 22 | 36,67 | 14 | 43,75 | 128 | 37,54 | 114 | 37,25 | 1.29 (0.58-2.89) | 0.53 | 1.51 (0.80-2.84) | 0.20 | . | . | . |
| FOLH1 ---tag | rs202680 | A/A | 34 | 56,67 | 17 | 53,13 | 191 | 56,01 | 173 | 56,54 | 1.00 (.-.) | . | 1.68 (0.89-3.19) | 0.11 | 0.36 | 0.97 | 0.71 |
| FOLH1 ---tag |  | A/T or T/T | 26 | 43,33 | 15 | 46,88 | 150 | 43,99 | 133 | 43,46 | 1.37 (0.61-3.06) | 0.44 | 1.56 (0.81-2.98) | 0.18 | . | . | . |
| FOLH1 ---candidate literature | rs202720 | G/G | 37 | 61,67 | 18 | 56,25 | 214 | 62,76 | 192 | 62,75 | 1.00 (.-.) | . | 1.56 (0.84-2.88) | 0.16 | 0.55 | 0.97 | 0.71 |
| FOLH1 ---candidate literature |  | G/C or C/C | 23 | 38,33 | 14 | 43,75 | 127 | 37,24 | 114 | 37,25 | 1.23 (0.55-2.74) | 0.61 | 1.48 (0.79-2.78) | 0.22 | . | . | . |
| FOLH1 ---tag | rs2299650 | G/G | 26 | 43,33 | 9 | 28,13 | 142 | 41,64 | 122 | 39,87 | 1.00 (.-.) | . | 1.74 (0.74-4.09) | 0.20 | 0.50 | 0.97 | 0.71 |
| FOLH1 ---tag |  | G/T or T/T | 34 | 56,67 | 23 | 71,88 | 199 | 58,36 | 184 | 60,13 | 1.32 (0.52-3.37) | 0.56 | 1.66 (0.71-3.84) | 0.24 | . | . | . |
| FOLH1 ---tag | rs617528 | G/G | 48 | 80,00 | 22 | 68,75 | 269 | 78,89 | 243 | 79,41 | 1.00 (.-.) | . | 1.43 (0.87-2.34) | 0.16 | 0.76 | 0.97 | 0.76 |
| FOLH1 ---tag |  | G/A or A/A | 12 | 20,00 | 10 | 31,25 | 72 | 21,11 | 63 | 20,59 | 1.07 (0.44-2.62) | 0.87 | 1.32 (0.76-2.28) | 0.32 | . | . | . |
| FOLH1 ---tag | rs663877 | T/T | 50 | 83,33 | 22 | 68,75 | 273 | 80,06 | 233 | 76,14 | 1.00 (.-.) | . | 1.56 (0.90-2.73) | 0.12 | 0.50 | 0.97 | 0.71 |
| FOLH1 ---tag |  | T/G or G/G | 10 | 16,67 | 10 | 31,25 | 68 | 19,94 | 73 | 23,86 | 1.55 (0.67-3.58) | 0.30 | 1.78 (0.99-3.21) | 0.06 | . | . | . |
| FOLH1 ---tag | rs670776 | A/A | 38 | 63,33 | 18 | 56,25 | 213 | 62,46 | 192 | 62,75 | 1.00 (.-.) | . | 1.60 (0.86-2.96) | 0.14 | 0.47 | 0.97 | 0.71 |
| FOLH1 ---tag |  | A/T or T/T | 22 | 36,67 | 14 | 43,75 | 128 | 37,54 | 114 | 37,25 | 1.29 (0.58-2.89) | 0.53 | 1.51 (0.80-2.84) | 0.20 | . | . | . |
| FOLH1 ---tag | rs7124497 | G/G | 56 | 93,33 | 31 | 96,88 | 315 | 92,38 | 284 | 92,81 | 1.00 (.-.) | . | 1.40 (0.90-2.17) | 0.13 | 0.63 | 0.97 | 0.71 |
| FOLH1 ---tag |  | G/A or A/A | 4 | 6,67 | 1 | 3,13 | 26 | 7,62 | 22 | 7,19 | 1.61 (0.21-12.22) | 0.65 | 1.30 (0.68-2.46) | 0.43 | . | . | . |
| FOLR1 ---tag | rs651646 | T/T | 17 | 28,33 | 10 | 31,25 | 111 | 32,55 | 104 | 33,99 | 1.00 (.-.) | . | 1.76 (0.80-3.87) | 0.16 | 0.43 | 0.97 | 0.43 |
| FOLR1 ---tag |  | T/A or A/A | 43 | 71,67 | 22 | 68,75 | 230 | 67,45 | 202 | 66,01 | 1.24 (0.51-3.03) | 0.64 | 1.50 (0.69-3.26) | 0.30 | . | . | . |
| FPGS ---tag | rs10987746 | T/T | 17 | 28,33 | 7 | 21,88 | 108 | 31,67 | 81 | 26,47 | 1.00 (.-.) | . | 1.40 (0.60-3.28) | 0.44 | 0.96 | 1.00 | 0.96 |
| FPGS ---tag |  | T/C or C/C | 43 | 71,67 | 25 | 78,13 | 233 | 68,33 | 225 | 73,53 | 1.01 (0.40-2.57) | 0.98 | 1.38 (0.61-3.16) | 0.44 | . | . | . |
| FPGS ---tag | rs7033913 | T/T | 22 | 36,67 | 11 | 34,38 | 101 | 29,62 | 106 | 34,64 | 1.00 (.-.) | . | 1.56 (0.73-3.33) | 0.26 | 0.71 | 0.97 | 0.96 |
| FPGS ---tag |  | T/C or C/C | 38 | 63,33 | 21 | 65,63 | 240 | 70,38 | 200 | 65,36 | 1.00 (0.42-2.35) | 0.99 | 1.30 (0.62-2.72) | 0.48 | . | . | . |
| FPGS ---tag | rs7039798 | G/G | 19 | 31,67 | 7 | 21,88 | 123 | 36,07 | 86 | 28,10 | 1.00 (.-.) | . | 1.31 (0.56-3.06) | 0.53 | 0.88 | 0.99 | 0.96 |
| FPGS ---tag |  | G/A or A/A | 41 | 68,33 | 25 | 78,13 | 218 | 63,93 | 220 | 71,90 | 1.07 (0.42-2.70) | 0.89 | 1.51 (0.66-3.45) | 0.33 | . | . | . |
| GGH ---tag | rs10957264 | G/G | 41 | 68,33 | 25 | 78,13 | 232 | 68,04 | 220 | 71,90 | 1.00 (.-.) | . | 1.40 (0.85-2.29) | 0.19 | 0.91 | 1.00 | 0.96 |
| GGH ---tag |  | G/T or T/T | 19 | 31,67 | 7 | 21,88 | 109 | 31,96 | 86 | 28,10 | 0.97 (0.38-2.49) | 0.96 | 1.28 (0.75-2.18) | 0.36 | . | . | . |
| GGH ---candidate literature | rs11545076 | T/T | 31 | 51,67 | 16 | 50,00 | 167 | 48,97 | 149 | 48,69 | 1.00 (.-.) | . | 1.10 (0.58-2.08) | 0.77 | 0.38 | 0.97 | 0.96 |
| GGH ---candidate literature |  | T/G or G/G | 29 | 48,33 | 16 | 50,00 | 174 | 51,03 | 157 | 51,31 | 0.71 (0.31-1.60) | 0.40 | 1.14 (0.61-2.15) | 0.68 | . | . | . |
| GGH ---candidate | rs11545077 | G/G | 33 | 55,00 | 18 | 56,25 | 181 | 53,08 | 162 | 52,94 | 1.00 (.-.) | . | 1.11 (0.61-1.99) | 0.74 | 0.32 | 0.97 | 0.96 |
| GGH ---candidate |  | G/A or A/A | 27 | 45,00 | 14 | 43,75 | 160 | 46,92 | 144 | 47,06 | 0.68 (0.30-1.54) | 0.35 | 1.16 (0.64-2.09) | 0.63 | . | . | . |
| GGH ---candidate | rs11545078 | C/C | 50 | 83,33 | 28 | 87,50 | 280 | 82,11 | 251 | 82,03 | 1.00 (.-.) | . | 1.38 (0.87-2.19) | 0.17 | 0.96 | 1.00 | 0.96 |
| GGH ---candidate |  | C/T or T/T | 10 | 16,67 | 4 | 12,50 | 61 | 17,89 | 55 | 17,97 | 0.95 (0.28-3.22) | 0.94 | 1.27 (0.74-2.19) | 0.38 | . | . | . |
| GGH ---tag | rs11995525 | G/G | 29 | 48,33 | 16 | 50,00 | 189 | 55,43 | 162 | 52,94 | 1.00 (.-.) | . | 1.42 (0.77-2.62) | 0.26 | 0.89 | 0.99 | 0.96 |
| GGH ---tag |  | G/A or A/A | 31 | 51,67 | 16 | 50,00 | 152 | 44,57 | 144 | 47,06 | 1.14 (0.51-2.58) | 0.75 | 1.52 (0.83-2.81) | 0.18 | . | . | . |
| GGH ---tag | rs16930073 | G/G | 47 | 78,33 | 23 | 71,88 | 270 | 79,18 | 244 | 79,74 | 1.00 (.-.) | . | 1.40 (0.85-2.32) | 0.19 | 0.91 | 1.00 | 0.96 |
| GGH ---tag |  | G/A or A/A | 13 | 21,67 | 9 | 28,13 | 71 | 20,82 | 62 | 20,26 | 1.13 (0.47-2.76) | 0.78 | 1.50 (0.86-2.64) | 0.16 | . | . | . |
| GGH ---tag | rs17194931 | G/G | 50 | 83,33 | 28 | 87,50 | 280 | 82,11 | 251 | 82,03 | 1.00 (.-.) | . | 1.38 (0.87-2.19) | 0.17 | 0.96 | 1.00 | 0.96 |
| GGH ---tag |  | G/A or A/A | 10 | 16,67 | 4 | 12,50 | 61 | 17,89 | 55 | 17,97 | 0.95 (0.28-3.22) | 0.94 | 1.27 (0.74-2.19) | 0.38 | . | . | . |
| GGH ---candidate literature | rs1800909 | T/T | 31 | 51,67 | 16 | 50,00 | 166 | 48,68 | 149 | 48,69 | 1.00 (.-.) | . | 1.12 (0.59-2.11) | 0.73 | 0.42 | 0.97 | 0.96 |
| GGH ---candidate literature |  | T/C or C/C | 29 | 48,33 | 16 | 50,00 | 175 | 51,32 | 157 | 51,31 | 0.71 (0.31-1.60) | 0.40 | 1.12 (0.60-2.12) | 0.72 | . | . | . |
| GGH ---candidate literature | rs3758149 | C/C | 31 | 51,67 | 16 | 50,00 | 167 | 48,97 | 149 | 48,69 | 1.00 (.-.) | . | 1.10 (0.58-2.08) | 0.77 | 0.38 | 0.97 | 0.96 |
| GGH ---candidate literature |  | C/T or T/T | 29 | 48,33 | 16 | 50,00 | 174 | 51,03 | 157 | 51,31 | 0.71 (0.31-1.60) | 0.40 | 1.14 (0.61-2.15) | 0.68 | . | . | . |
| GGH ---tag | rs3780130 | A/A | 37 | 61,67 | 17 | 53,13 | 203 | 59,53 | 192 | 62,75 | 1.00 (.-.) | . | 1.56 (0.91-2.67) | 0.11 | 0.44 | 0.97 | 0.96 |
| GGH ---tag |  | A/T or T/T | 23 | 38,33 | 15 | 46,88 | 138 | 40,47 | 114 | 37,25 | 1.30 (0.57-2.98) | 0.54 | 1.43 (0.83-2.47) | 0.20 | . | . | . |
| GGH ---tag | rs4446729 | C/C | 38 | 63,33 | 18 | 56,25 | 183 | 53,67 | 163 | 53,27 | 1.00 (.-.) | . | 1.31 (0.75-2.27) | 0.34 | 0.78 | 0.97 | 0.96 |
| GGH ---tag |  | C/T or T/T | 22 | 36,67 | 14 | 43,75 | 158 | 46,33 | 143 | 46,73 | 0.86 (0.37-1.98) | 0.72 | 1.27 (0.73-2.22) | 0.40 | . | . | . |
| GGH ---tag | rs6472067 | C/C | 19 | 31,67 | 12 | 37,50 | 140 | 41,06 | 119 | 38,89 | 1.00 (.-.) | . | 1.52 (0.77-2.99) | 0.23 | 0.67 | 0.97 | 0.96 |
| GGH ---tag |  | C/G or G/G | 41 | 68,33 | 20 | 62,50 | 201 | 58,94 | 187 | 61,11 | 1.35 (0.59-3.10) | 0.48 | 1.69 (0.87-3.27) | 0.12 | . | . | . |
| GGH ---tag | rs7010484 | T/T | 34 | 56,67 | 17 | 53,13 | 163 | 47,80 | 140 | 45,75 | 1.00 (.-.) | . | 1.21 (0.67-2.18) | 0.54 | 0.55 | 0.97 | 0.96 |
| GGH ---tag |  | T/C or C/C | 26 | 43,33 | 15 | 46,88 | 178 | 52,20 | 166 | 54,25 | 0.78 (0.35-1.76) | 0.55 | 1.22 (0.68-2.21) | 0.51 | . | . | . |
| GNMT ---tag | rs1053538 | C/C | 21 | 35,00 | 10 | 31,25 | 84 | 24,63 | 80 | 26,14 | 1.00 (.-.) | . | 1.90 (0.81-4.44) | 0.14 | 0.35 | 0.97 | 0.99 |
| GNMT ---tag |  | C/G or G/G | 39 | 65,00 | 22 | 68,75 | 257 | 75,37 | 226 | 73,86 | 1.62 (0.64-4.09) | 0.31 | 1.94 (0.85-4.43) | 0.12 | . | . | . |
| GNMT ---tag | rs2296805 | G/G | 18 | 30,00 | 13 | 40,63 | 105 | 30,79 | 98 | 32,03 | 1.00 (.-.) | . | 1.45 (0.76-2.78) | 0.26 | 0.88 | 0.99 | 0.99 |
| GNMT ---tag |  | G/T or T/T | 42 | 70,00 | 19 | 59,38 | 236 | 69,21 | 208 | 67,97 | 0.92 (0.41-2.05) | 0.84 | 1.26 (0.67-2.35) | 0.47 | . | . | . |
| GNMT ---tag | rs6901782 | T/T | 40 | 66,67 | 21 | 65,63 | 264 | 77,42 | 241 | 78,76 | 1.00 (.-.) | . | 1.41 (0.84-2.39) | 0.20 | 0.99 | 1.00 | 0.99 |
| GNMT ---tag |  | T/C or C/C | 20 | 33,33 | 11 | 34,38 | 77 | 22,58 | 65 | 21,24 | 1.27 (0.55-2.90) | 0.58 | 1.80 (1.01-3.20) | 0.05 | . | . | . |
| GNMT ---tag | rs6927188 | A/A | 40 | 66,67 | 22 | 68,75 | 189 | 55,43 | 167 | 54,58 | 1.00 (.-.) | . | 1.45 (0.86-2.44) | 0.16 | 0.78 | 0.97 | 0.99 |
| GNMT ---tag |  | A/G or G/G | 20 | 33,33 | 10 | 31,25 | 152 | 44,57 | 139 | 45,42 | 1.01 (0.43-2.36) | 0.98 | 1.29 (0.76-2.19) | 0.35 | . | . | . |
| MAT1A ---tag | rs10887708 | G/G | 33 | 55,00 | 17 | 53,13 | 176 | 51,61 | 162 | 52,94 | 1.00 (.-.) | . | 1.65 (0.90-3.04) | 0.11 | 0.35 | 0.97 | 0.78 |
| MAT1A ---tag |  | G/A or A/A | 27 | 45,00 | 15 | 46,88 | 165 | 48,39 | 144 | 47,06 | 1.53 (0.68-3.43) | 0.30 | 1.69 (0.92-3.13) | 0.09 | . | . | . |
| MAT1A ---tag | rs10887718 | T/T | 18 | 30,00 | 9 | 28,13 | 97 | 28,45 | 82 | 26,80 | 1.00 (.-.) | . | 2.69 (0.96-7.58) | 0.06 | 0.11 | 0.97 | 0.78 |
| MAT1A ---tag |  | T/C or C/C | 42 | 70,00 | 23 | 71,88 | 244 | 71,55 | 224 | 73,20 | 2.48 (0.84-7.35) | 0.10 | 2.83 (1.02-7.82) | 0.05 | . | . | . |
| MAT1A ---tag | rs11202403 | C/C | 41 | 68,33 | 24 | 75,00 | 219 | 64,22 | 192 | 62,75 | 1.00 (.-.) | . | 1.42 (0.87-2.30) | 0.16 | 0.73 | 0.97 | 0.93 |
| MAT1A ---tag |  | C/T or T/T | 19 | 31,67 | 8 | 25,00 | 122 | 35,78 | 114 | 37,25 | 1.22 (0.45-3.31) | 0.70 | 1.43 (0.87-2.36) | 0.16 | . | . | . |
| MAT1A ---tag | rs1832683 | C/C | 39 | 65,00 | 23 | 71,88 | 226 | 66,28 | 215 | 70,26 | 1.00 (.-.) | . | 1.26 (0.78-2.05) | 0.35 | 0.44 | 0.97 | 0.78 |
| MAT1A ---tag |  | C/T or T/T | 21 | 35,00 | 9 | 28,13 | 115 | 33,72 | 91 | 29,74 | 0.52 (0.19-1.40) | 0.19 | 0.97 (0.58-1.63) | 0.91 | . | . | . |
| MAT1A ---tag | rs2236568 | C/C | 18 | 30,00 | 11 | 34,38 | 111 | 32,55 | 104 | 33,99 | 1.00 (.-.) | . | 1.30 (0.66-2.54) | 0.44 | 0.83 | 0.99 | 0.93 |
| MAT1A ---tag |  | C/A or A/A | 42 | 70,00 | 21 | 65,63 | 230 | 67,45 | 202 | 66,01 | 0.88 (0.39-1.99) | 0.76 | 1.26 (0.66-2.42) | 0.49 | . | . | . |
| MAT1A ---tag | rs2236569 | A/A | 24 | 40,00 | 15 | 46,88 | 161 | 47,21 | 128 | 41,83 | 1.00 (.-.) | . | 1.37 (0.68-2.79) | 0.38 | 0.93 | 1.00 | 0.93 |
| MAT1A ---tag |  | A/G or G/G | 36 | 60,00 | 17 | 53,13 | 180 | 52,79 | 178 | 58,17 | 1.20 (0.52-2.78) | 0.66 | 1.72 (0.85-3.47) | 0.13 | . | . | . |
| MAT1A ---tag | rs9421467 | G/G | 54 | 90,00 | 29 | 90,63 | 308 | 90,32 | 267 | 87,25 | 1.00 (.-.) | . | 1.46 (0.92-2.31) | 0.11 | 0.28 | 0.97 | 0.78 |
| MAT1A ---tag |  | G/C or C/C | 6 | 10,00 | 3 | 9,38 | 33 | 9,68 | 39 | 12,75 | 2.50 (0.72-8.70) | 0.15 | 1.69 (0.96-2.96) | 0.07 | . | . | . |
| MAT1A ---tag | rs998765 | A/A | 20 | 33,33 | 10 | 31,25 | 89 | 26,10 | 82 | 26,80 | 1.00 (.-.) | . | 1.98 (0.93-4.21) | 0.08 | 0.19 | 0.97 | 0.78 |
| MAT1A ---tag |  | A/T or T/T | 40 | 66,67 | 22 | 68,75 | 252 | 73,90 | 224 | 73,20 | 1.82 (0.77-4.31) | 0.17 | 1.97 (0.96-4.06) | 0.07 | . | . | . |
| MAT1A ---tag | rs998766 | C/C | 25 | 41,67 | 13 | 40,63 | 103 | 30,21 | 100 | 32,68 | 1.00 (.-.) | . | 1.60 (0.84-3.07) | 0.16 | 0.52 | 0.97 | 0.78 |
| MAT1A ---tag |  | C/G or G/G | 35 | 58,33 | 19 | 59,38 | 238 | 69,79 | 206 | 67,32 | 1.26 (0.56-2.84) | 0.57 | 1.53 (0.82-2.86) | 0.18 | . | . | . |
| MAT2B ---tag | rs12655857 | G/G | 24 | 40,00 | 21 | 65,63 | 189 | 55,43 | 170 | 55,56 | 1.00 (.-.) | . | 0.80 (0.47-1.36) | 0.42 | 0.01 | 0.85 | 0.03 |
| MAT2B ---tag |  | G/T or T/T | 36 | 60,00 | 11 | 34,38 | 152 | 44,57 | 136 | 44,44 | 0.33 (0.14-0.79) | 0.01 | 0.89 (0.52-1.52) | 0.67 | . | . | . |
| MAT2B ---tag | rs6869277 | C/C | 47 | 78,33 | 26 | 81,25 | 275 | 80,65 | 240 | 78,43 | 1.00 (.-.) | . | 1.32 (0.81-2.15) | 0.27 | 0.72 | 0.97 | 0.72 |
| MAT2B ---tag |  | C/T or T/T | 13 | 21,67 | 6 | 18,75 | 66 | 19,35 | 66 | 21,57 | 0.86 (0.34-2.19) | 0.76 | 1.36 (0.78-2.37) | 0.28 | . | . | . |
| MAT2B ---tag | rs6874065 | A/A | 21 | 35,00 | 8 | 25,00 | 95 | 27,86 | 87 | 28,43 | 1.00 (.-.) | . | 1.98 (0.90-4.36) | 0.09 | 0.24 | 0.97 | 0.30 |
| MAT2B ---tag |  | A/G or G/G | 39 | 65,00 | 24 | 75,00 | 246 | 72,14 | 219 | 71,57 | 1.61 (0.66-3.91) | 0.29 | 1.85 (0.86-3.98) | 0.12 | . | . | . |
| MAT2B ---tag | rs6882306 | T/T | 48 | 80,00 | 22 | 68,75 | 233 | 68,33 | 214 | 69,93 | 1.00 (.-.) | . | 1.66 (0.98-2.80) | 0.06 | 0.12 | 0.97 | 0.19 |
| MAT2B ---tag |  | T/C or C/C | 12 | 20,00 | 10 | 31,25 | 108 | 31,67 | 92 | 30,07 | 2.45 (1.04-5.74) | 0.04 | 1.93 (1.10-3.39) | 0.02 | . | . | . |
| MAT2B ---tag | rs7721639 | T/T | 45 | 75,00 | 22 | 68,75 | 246 | 72,14 | 220 | 71,90 | 1.00 (.-.) | . | 1.64 (0.99-2.74) | 0.06 | 0.11 | 0.97 | 0.19 |
| MAT2B ---tag |  | T/G or G/G | 15 | 25,00 | 10 | 31,25 | 95 | 27,86 | 86 | 28,10 | 2.31 (0.97-5.54) | 0.06 | 1.73 (1.00-3.00) | 0.05 | . | . | . |
| MTHFD1 ---tag | rs1256148 | G/G | 35 | 58,33 | 17 | 53,13 | 204 | 59,82 | 181 | 59,15 | 1.00 (.-.) | . | 1.64 (0.91-2.93) | 0.10 | 0.37 | 0.97 | 0.44 |
| MTHFD1 ---tag |  | G/A or A/A | 25 | 41,67 | 15 | 46,88 | 137 | 40,18 | 125 | 40,85 | 1.24 (0.55-2.78) | 0.61 | 1.37 (0.76-2.46) | 0.30 | . | . | . |
| MTHFD1 ---tag | rs13329053 | T/T | 15 | 25,00 | 6 | 18,75 | 109 | 31,96 | 105 | 34,31 | 1.00 (.-.) | . | 1.87 (0.75-4.66) | 0.18 | 0.44 | 0.97 | 0.44 |
| MTHFD1 ---tag |  | T/C or C/C | 45 | 75,00 | 26 | 81,25 | 232 | 68,04 | 201 | 65,69 | 1.65 (0.61-4.45) | 0.32 | 2.08 (0.84-5.12) | 0.11 | . | . | . |
| MTHFD1 ---candidate literature | rs2236224 | C/C | 21 | 35,00 | 7 | 21,88 | 132 | 38,71 | 128 | 41,83 | 1.00 (.-.) | . | 2.17 (0.94-4.97) | 0.07 | 0.17 | 0.97 | 0.44 |
| MTHFD1 ---candidate literature |  | C/T or T/T | 39 | 65,00 | 25 | 78,13 | 209 | 61,29 | 178 | 58,17 | 2.21 (0.87-5.63) | 0.10 | 2.50 (1.09-5.72) | 0.03 | . | . | . |
| MTHFD1 ---candidate | rs2236225 | C/C | 17 | 28,33 | 5 | 15,63 | 109 | 31,96 | 108 | 35,29 | 1.00 (.-.) | . | 2.09 (0.75-5.80) | 0.16 | 0.35 | 0.97 | 0.44 |
| MTHFD1 ---candidate |  | C/T or T/T | 43 | 71,67 | 27 | 84,38 | 232 | 68,04 | 198 | 64,71 | 1.79 (0.60-5.32) | 0.29 | 2.24 (0.82-6.16) | 0.12 | . | . | . |
| MTHFD1 ---tag | rs2281603 | A/A | 36 | 60,00 | 24 | 75,00 | 205 | 60,12 | 170 | 55,56 | 1.00 (.-.) | . | 1.23 (0.75-2.01) | 0.42 | 0.39 | 0.97 | 0.44 |
| MTHFD1 ---tag |  | A/G or G/G | 24 | 40,00 | 8 | 25,00 | 136 | 39,88 | 136 | 44,44 | 0.67 (0.26-1.70) | 0.40 | 1.24 (0.75-2.05) | 0.40 | . | . | . |
| MTHFD1 ---candidate literature | rs8003379 | A/A | 34 | 56,67 | 18 | 56,25 | 193 | 56,60 | 178 | 58,17 | 1.00 (.-.) | . | 1.18 (0.67-2.08) | 0.57 | 0.42 | 0.97 | 0.44 |
| MTHFD1 ---candidate literature |  | A/C or C/C | 26 | 43,33 | 14 | 43,75 | 148 | 43,40 | 128 | 41,83 | 0.87 (0.39-1.94) | 0.74 | 1.46 (0.83-2.58) | 0.19 | . | . | . |
| MTHFD2 ---tag | rs10177833 | A/A | 19 | 31,67 | 7 | 21,88 | 111 | 32,55 | 95 | 31,05 | 1.00 (.-.) | . | 1.75 (0.75-4.10) | 0.20 | 0.49 | 0.97 | 0.53 |
| MTHFD2 ---tag |  | A/C or C/C | 41 | 68,33 | 25 | 78,13 | 230 | 67,45 | 211 | 68,95 | 1.29 (0.50-3.31) | 0.60 | 1.60 (0.70-3.68) | 0.27 | . | . | . |
| MTHFD2 ---tag | rs702462 | T/T | 25 | 41,67 | 11 | 34,38 | 114 | 33,43 | 102 | 33,33 | 1.00 (.-.) | . | 1.70 (0.80-3.62) | 0.17 | 0.48 | 0.97 | 0.53 |
| MTHFD2 ---tag |  | T/A or A/A | 35 | 58,33 | 21 | 65,63 | 227 | 66,57 | 204 | 66,67 | 1.27 (0.53-3.03) | 0.59 | 1.56 (0.75-3.25) | 0.23 | . | . | . |
| MTHFD2 ---candidate literature | rs702465 | A/A | 17 | 28,33 | 11 | 34,38 | 100 | 29,33 | 78 | 25,49 | 1.00 (.-.) | . | 1.00 (0.48-2.08) | 1.00 | 0.33 | 0.97 | 0.53 |
| MTHFD2 ---candidate literature |  | A/T or T/T | 43 | 71,67 | 21 | 65,63 | 241 | 70,67 | 228 | 74,51 | 0.84 (0.36-1.96) | 0.69 | 1.32 (0.66-2.66) | 0.43 | . | . | . |
| MTHFD2 ---candidate literature | rs7571842 | A/A | 18 | 30,00 | 6 | 18,75 | 103 | 30,21 | 85 | 27,78 | 1.00 (.-.) | . | 1.85 (0.73-4.67) | 0.19 | 0.45 | 0.97 | 0.53 |
| MTHFD2 ---candidate literature |  | A/G or G/G | 42 | 70,00 | 26 | 81,25 | 238 | 69,79 | 221 | 72,22 | 1.35 (0.50-3.65) | 0.56 | 1.68 (0.68-4.15) | 0.26 | . | . | . |
| MTHFD2 ---tag | rs7587117 | T/T | 28 | 46,67 | 12 | 37,50 | 151 | 44,28 | 123 | 40,20 | 1.00 (.-.) | . | 1.74 (0.83-3.65) | 0.14 | 0.42 | 0.97 | 0.53 |
| MTHFD2 ---tag |  | T/C or C/C | 32 | 53,33 | 20 | 62,50 | 190 | 55,72 | 183 | 59,80 | 1.43 (0.60-3.40) | 0.42 | 1.71 (0.82-3.56) | 0.15 | . | . | . |
| MTHFD2 ---tag | rs828861 | C/C | 18 | 30,00 | 11 | 34,38 | 100 | 29,33 | 77 | 25,16 | 1.00 (.-.) | . | 1.12 (0.54-2.32) | 0.76 | 0.53 | 0.97 | 0.53 |
| MTHFD2 ---tag |  | C/G or G/G | 42 | 70,00 | 21 | 65,63 | 241 | 70,67 | 229 | 74,84 | 0.91 (0.39-2.12) | 0.83 | 1.37 (0.68-2.74) | 0.38 | . | . | . |
| MTHFD2 ---tag | rs828863 | G/G | 52 | 86,67 | 27 | 84,38 | 274 | 80,35 | 257 | 83,99 | 1.00 (.-.) | . | 1.46 (0.92-2.33) | 0.11 | 0.48 | 0.97 | 0.53 |
| MTHFD2 ---tag |  | G/A or A/A | 8 | 13,33 | 5 | 15,63 | 67 | 19,65 | 49 | 16,01 | 1.40 (0.47-4.14) | 0.54 | 1.33 (0.77-2.31) | 0.31 | . | . | . |
| MTHFR ---tag | rs1476413 | G/G | 32 | 53,33 | 17 | 53,13 | 175 | 51,32 | 167 | 54,58 | 1.00 (.-.) | . | 1.47 (0.80-2.68) | 0.21 | 0.75 | 0.97 | 0.99 |
| MTHFR ---tag |  | G/A or A/A | 28 | 46,67 | 15 | 46,88 | 166 | 48,68 | 139 | 45,42 | 1.15 (0.52-2.57) | 0.73 | 1.47 (0.80-2.70) | 0.21 | . | . | . |
| MTHFR ---tag | rs17376328 | G/G | 55 | 91,67 | 27 | 84,38 | 299 | 87,68 | 259 | 84,64 | 1.00 (.-.) | . | 1.45 (0.90-2.34) | 0.12 | 0.56 | 0.97 | 0.99 |
| MTHFR ---tag |  | G/A or A/A | 5 | 8,33 | 5 | 15,63 | 42 | 12,32 | 47 | 15,36 | 1.41 (0.51-3.88) | 0.51 | 1.47 (0.83-2.60) | 0.18 | . | . | . |
| MTHFR ---tag | rs17421462 | G/G | 51 | 85,00 | 26 | 81,25 | 293 | 85,92 | 261 | 85,29 | 1.00 (.-.) | . | 1.41 (0.87-2.29) | 0.17 | 0.88 | 0.99 | 0.99 |
| MTHFR ---tag |  | G/A or A/A | 9 | 15,00 | 6 | 18,75 | 48 | 14,08 | 45 | 14,71 | 1.23 (0.45-3.37) | 0.69 | 1.59 (0.89-2.85) | 0.12 | . | . | . |
| MTHFR ---candidate | rs1801131 | A/A | 26 | 43,33 | 15 | 46,88 | 155 | 45,45 | 147 | 48,04 | 1.00 (.-.) | . | 1.59 (0.83-3.08) | 0.17 | 0.54 | 0.97 | 0.99 |
| MTHFR ---candidate |  | A/C or C/C | 34 | 56,67 | 17 | 53,13 | 186 | 54,55 | 159 | 51,96 | 1.23 (0.54-2.79) | 0.62 | 1.51 (0.78-2.90) | 0.22 | . | . | . |
| MTHFR ---candidate | rs1801133 | C/C | 22 | 36,67 | 12 | 37,50 | 146 | 42,82 | 134 | 43,79 | 1.00 (.-.) | . | 1.14 (0.60-2.17) | 0.69 | 0.50 | 0.97 | 0.99 |
| MTHFR ---candidate |  | C/T or T/T | 38 | 63,33 | 20 | 62,50 | 195 | 57,18 | 172 | 56,21 | 0.57 (0.26-1.28) | 0.17 | 0.87 (0.46-1.65) | 0.68 | . | . | . |
| MTHFR ---tag | rs2066471 | G/G | 42 | 70,00 | 22 | 68,75 | 238 | 69,79 | 216 | 70,59 | 1.00 (.-.) | . | 1.41 (0.83-2.38) | 0.20 | 0.85 | 0.99 | 0.99 |
| MTHFR ---tag |  | G/A or A/A | 18 | 30,00 | 10 | 31,25 | 103 | 30,21 | 90 | 29,41 | 1.33 (0.57-3.06) | 0.51 | 1.71 (0.99-2.98) | 0.06 | . | . | . |
| MTHFR ---tag | rs4846047 | G/G | 30 | 50,00 | 15 | 46,88 | 171 | 50,15 | 155 | 50,65 | 1.00 (.-.) | . | 1.40 (0.72-2.72) | 0.33 | 0.99 | 1.00 | 0.99 |
| MTHFR ---tag |  | G/C or C/C | 30 | 50,00 | 17 | 53,13 | 170 | 49,85 | 151 | 49,35 | 1.16 (0.51-2.62) | 0.72 | 1.61 (0.83-3.12) | 0.16 | . | . | . |
| MTHFR ---tag | rs4846049 | G/G | 26 | 43,33 | 14 | 43,75 | 151 | 44,28 | 145 | 47,39 | 1.00 (.-.) | . | 1.64 (0.82-3.27) | 0.16 | 0.50 | 0.97 | 0.99 |
| MTHFR ---tag |  | G/T or T/T | 34 | 56,67 | 18 | 56,25 | 190 | 55,72 | 161 | 52,61 | 1.26 (0.55-2.90) | 0.59 | 1.54 (0.77-3.06) | 0.22 | . | . | . |
| MTHFR ---tag | rs7538516 | T/T | 23 | 38,33 | 12 | 37,50 | 121 | 35,48 | 112 | 36,60 | 1.00 (.-.) | . | 1.48 (0.71-3.10) | 0.30 | 0.80 | 0.97 | 0.99 |
| MTHFR ---tag |  | T/C or C/C | 37 | 61,67 | 20 | 62,50 | 220 | 64,52 | 194 | 63,40 | 1.17 (0.50-2.73) | 0.72 | 1.54 (0.75-3.18) | 0.24 | . | . | . |
| MTR ---tag | rs10733117 | A/A | 24 | 40,00 | 11 | 34,38 | 115 | 33,72 | 119 | 38,89 | 1.00 (.-.) | . | 0.99 (0.44-2.22) | 0.99 | 0.40 | 0.97 | 0.80 |
| MTR ---tag |  | A/G or G/G | 36 | 60,00 | 21 | 65,63 | 226 | 66,28 | 187 | 61,11 | 0.62 (0.25-1.50) | 0.29 | 0.92 (0.42-2.02) | 0.84 | . | . | . |
| MTR ---tag | rs12129440 | G/G | 35 | 58,33 | 21 | 65,63 | 179 | 52,49 | 183 | 59,80 | 1.00 (.-.) | . | 1.45 (0.85-2.48) | 0.17 | 0.78 | 0.97 | 0.80 |
| MTR ---tag |  | G/A or A/A | 25 | 41,67 | 11 | 34,38 | 162 | 47,51 | 123 | 40,20 | 0.98 (0.43-2.25) | 0.96 | 1.25 (0.73-2.16) | 0.42 | . | . | . |
| MTR ---candidate | rs1805087 | A/A | 43 | 71,67 | 23 | 71,88 | 229 | 67,16 | 208 | 67,97 | 1.00 (.-.) | . | 1.42 (0.87-2.33) | 0.16 | 0.80 | 0.97 | 0.80 |
| MTR ---candidate |  | A/G or G/G | 17 | 28,33 | 9 | 28,13 | 112 | 32,84 | 98 | 32,03 | 0.99 (0.39-2.52) | 0.99 | 1.25 (0.74-2.11) | 0.41 | . | . | . |
| MTR ---tag | rs3890786 | C/C | 25 | 41,67 | 12 | 37,50 | 120 | 35,19 | 102 | 33,33 | 1.00 (.-.) | . | 1.90 (0.94-3.85) | 0.07 | 0.15 | 0.97 | 0.75 |
| MTR ---tag |  | C/T or T/T | 35 | 58,33 | 20 | 62,50 | 221 | 64,81 | 204 | 66,67 | 2.19 (0.95-5.02) | 0.07 | 2.21 (1.11-4.38) | 0.02 | . | . | . |
| MTR ---tag | rs4659727 | A/A | 43 | 71,67 | 23 | 71,88 | 228 | 66,86 | 208 | 67,97 | 1.00 (.-.) | . | 1.43 (0.87-2.34) | 0.16 | 0.79 | 0.97 | 0.80 |
| MTR ---tag |  | A/G or G/G | 17 | 28,33 | 9 | 28,13 | 113 | 33,14 | 98 | 32,03 | 0.99 (0.39-2.52) | 0.99 | 1.24 (0.73-2.10) | 0.42 | . | . | . |
| MTRR ---candidate literature/tag | rs10380 | C/C | 50 | 83,33 | 26 | 81,25 | 290 | 85,04 | 255 | 83,33 | 1.00 (.-.) | . | 1.37 (0.86-2.21) | 0.19 | 0.95 | 1.00 | 0.99 |
| MTRR ---candidate literature/tag |  | C/T or T/T | 10 | 16,67 | 6 | 18,75 | 51 | 14,96 | 51 | 16,67 | 1.32 (0.46-3.77) | 0.61 | 1.75 (1.01-3.03) | 0.05 | . | . | . |
| MTRR ---tag | rs10475399 | G/G | 26 | 43,33 | 11 | 34,38 | 147 | 43,11 | 133 | 43,46 | 1.00 (.-.) | . | 1.41 (0.73-2.72) | 0.31 | 0.92 | 1.00 | 0.99 |
| MTRR ---tag |  | G/A or A/A | 34 | 56,67 | 21 | 65,63 | 194 | 56,89 | 173 | 56,54 | 1.11 (0.49-2.51) | 0.81 | 1.49 (0.78-2.87) | 0.23 | . | . | . |
| MTRR ---tag | rs11134265 | C/C | 23 | 38,33 | 12 | 37,50 | 153 | 44,87 | 137 | 44,77 | 1.00 (.-.) | . | 1.38 (0.72-2.67) | 0.34 | 0.99 | 1.00 | 0.99 |
| MTRR ---tag |  | C/T or T/T | 37 | 61,67 | 20 | 62,50 | 188 | 55,13 | 169 | 55,23 | 1.06 (0.47-2.42) | 0.88 | 1.46 (0.76-2.81) | 0.26 | . | . | . |
| MTRR ---tag | rs13181011 | T/T | 35 | 58,33 | 20 | 62,50 | 214 | 62,76 | 194 | 63,40 | 1.00 (.-.) | . | 1.27 (0.76-2.12) | 0.35 | 0.59 | 0.97 | 0.95 |
| MTRR ---tag |  | T/C or C/C | 25 | 41,67 | 12 | 37,50 | 127 | 37,24 | 112 | 36,60 | 0.74 (0.30-1.82) | 0.51 | 1.22 (0.72-2.06) | 0.46 | . | . | . |
| MTRR ---tag | rs161869 | C/C | 22 | 36,67 | 10 | 31,25 | 112 | 32,84 | 106 | 34,64 | 1.00 (.-.) | . | 1.06 (0.48-2.35) | 0.88 | 0.45 | 0.97 | 0.95 |
| MTRR ---tag |  | C/T or T/T | 38 | 63,33 | 22 | 68,75 | 229 | 67,16 | 200 | 65,36 | 0.83 (0.34-2.02) | 0.69 | 1.28 (0.59-2.77) | 0.53 | . | . | . |
| MTRR ---tagged by rs162039 | rs162036 | A/A | 49 | 81,67 | 24 | 75,00 | 275 | 80,65 | 244 | 79,74 | 1.00 (.-.) | . | 1.40 (0.87-2.28) | 0.17 | 0.78 | 0.97 | 0.95 |
| MTRR ---tagged by rs162039 |  | A/G or G/G | 11 | 18,33 | 8 | 25,00 | 66 | 19,35 | 62 | 20,26 | 1.52 (0.57-4.05) | 0.41 | 1.84 (1.07-3.16) | 0.03 | . | . | . |
| MTRR ---tag | rs162039 | C/C | 49 | 81,67 | 24 | 75,00 | 276 | 80,94 | 244 | 79,74 | 1.00 (.-.) | . | 1.40 (0.87-2.28) | 0.17 | 0.78 | 0.97 | 0.95 |
| MTRR ---tag |  | C/T or T/T | 11 | 18,33 | 8 | 25,00 | 65 | 19,06 | 62 | 20,26 | 1.52 (0.57-4.05) | 0.41 | 1.84 (1.07-3.16) | 0.03 | . | . | . |
| MTRR ---tag | rs162270 | G/G | 41 | 68,33 | 24 | 75,00 | 245 | 71,85 | 217 | 70,92 | 1.00 (.-.) | . | 1.24 (0.76-2.03) | 0.40 | 0.47 | 0.97 | 0.95 |
| MTRR ---tag |  | G/T or T/T | 19 | 31,67 | 8 | 25,00 | 96 | 28,15 | 89 | 29,08 | 0.66 (0.26-1.68) | 0.39 | 1.17 (0.68-2.00) | 0.57 | . | . | . |
| MTRR ---candidate | rs16879334 | C/C | 58 | 96,67 | 29 | 90,63 | 319 | 93,55 | 288 | 94,12 | 1.00 (.-.) | . | 1.49 (0.94-2.34) | 0.09 | 0.18 | 0.97 | 0.95 |
| MTRR ---candidate |  | C/G or G/G | 2 | 3,33 | 3 | 9,38 | 22 | 6,45 | 18 | 5,88 | 2.11 (0.62-7.23) | 0.23 | 1.17 (0.59-2.29) | 0.66 | . | . | . |
| MTRR ---singleton | rs1801394 | G/G | 19 | 31,67 | 10 | 31,25 | 112 | 32,84 | 87 | 28,43 | 1.00 (.-.) | . | 1.16 (0.55-2.44) | 0.70 | 0.59 | 0.97 | 0.95 |
| MTRR ---singleton |  | G/A or A/A | 41 | 68,33 | 22 | 68,75 | 229 | 67,16 | 219 | 71,57 | 0.91 (0.39-2.16) | 0.83 | 1.36 (0.66-2.80) | 0.41 | . | . | . |
| MTRR ---tag | rs1802059 | G/G | 26 | 43,33 | 17 | 53,13 | 131 | 38,42 | 118 | 38,56 | 1.00 (.-.) | . | 1.32 (0.74-2.36) | 0.35 | 0.77 | 0.97 | 0.95 |
| MTRR ---tag |  | G/A or A/A | 34 | 56,67 | 15 | 46,88 | 210 | 61,58 | 188 | 61,44 | 0.78 (0.35-1.73) | 0.54 | 1.16 (0.66-2.05) | 0.60 | . | . | . |
| MTRR ---tag | rs2077744 | T/T | 40 | 66,67 | 24 | 75,00 | 250 | 73,31 | 226 | 73,86 | 1.00 (.-.) | . | 1.23 (0.75-2.04) | 0.41 | 0.47 | 0.97 | 0.95 |
| MTRR ---tag |  | T/C or C/C | 20 | 33,33 | 8 | 25,00 | 91 | 26,69 | 80 | 26,14 | 0.71 (0.29-1.73) | 0.45 | 1.23 (0.71-2.14) | 0.46 | . | . | . |
| MTRR ---candidate | rs2287780 | C/C | 58 | 96,67 | 29 | 90,63 | 319 | 93,55 | 288 | 94,12 | 1.00 (.-.) | . | 1.49 (0.94-2.34) | 0.09 | 0.18 | 0.97 | 0.95 |
| MTRR ---candidate |  | C/T or T/T | 2 | 3,33 | 3 | 9,38 | 22 | 6,45 | 18 | 5,88 | 2.11 (0.62-7.23) | 0.23 | 1.17 (0.59-2.29) | 0.66 | . | . | . |
| MTRR ---candidate | rs2303080 | T/T | 58 | 96,67 | 29 | 90,63 | 319 | 93,55 | 289 | 94,44 | 1.00 (.-.) | . | 1.49 (0.95-2.35) | 0.09 | 0.17 | 0.97 | 0.95 |
| MTRR ---candidate |  | T/A or A/A | 2 | 3,33 | 3 | 9,38 | 22 | 6,45 | 17 | 5,56 | 2.11 (0.62-7.22) | 0.23 | 1.11 (0.56-2.22) | 0.76 | . | . | . |
| MTRR ---tag | rs7715062 | G/G | 21 | 35,00 | 13 | 40,63 | 114 | 33,43 | 104 | 33,99 | 1.00 (.-.) | . | 1.53 (0.80-2.93) | 0.20 | 0.72 | 0.97 | 0.95 |
| MTRR ---tag |  | G/T or T/T | 39 | 65,00 | 19 | 59,38 | 227 | 66,57 | 202 | 66,01 | 1.01 (0.45-2.25) | 0.99 | 1.31 (0.70-2.47) | 0.39 | . | . | . |
| MTRR ---tag | rs9282787 | T/T | 37 | 61,67 | 21 | 65,63 | 216 | 63,34 | 196 | 64,05 | 1.00 (.-.) | . | 1.31 (0.79-2.17) | 0.30 | 0.72 | 0.97 | 0.95 |
| MTRR ---tag |  | T/C or C/C | 23 | 38,33 | 11 | 34,38 | 125 | 36,66 | 110 | 35,95 | 0.83 (0.34-2.02) | 0.68 | 1.28 (0.76-2.17) | 0.35 | . | . | . |
| MTRR ---candidate literature | rs9332 | C/C | 49 | 81,67 | 24 | 75,00 | 276 | 80,94 | 244 | 79,74 | 1.00 (.-.) | . | 1.40 (0.87-2.28) | 0.17 | 0.78 | 0.97 | 0.95 |
| MTRR ---candidate literature |  | C/T or T/T | 11 | 18,33 | 8 | 25,00 | 65 | 19,06 | 62 | 20,26 | 1.52 (0.57-4.05) | 0.41 | 1.84 (1.07-3.16) | 0.03 | . | . | . |
| NFKB1 ---NA | rs1609798 | C/C | 19 | 31,67 | 17 | 53,13 | 163 | 47,80 | 141 | 46,08 | 1.00 (.-.) | . | 1.21 (0.70-2.09) | 0.51 | 0.50 | 0.97 | 0.50 |
| NFKB1 ---NA |  | C/T or T/T | 41 | 68,33 | 15 | 46,88 | 178 | 52,20 | 165 | 53,92 | 0.83 (0.37-1.90) | 0.67 | 1.34 (0.77-2.33) | 0.30 | . | . | . |
| NFKB1 ---tag | rs230540 | T/T | 17 | 28,33 | 15 | 46,88 | 139 | 40,76 | 130 | 42,48 | 1.00 (.-.) | . | 1.10 (0.62-1.96) | 0.74 | 0.30 | 0.97 | 0.47 |
| NFKB1 ---tag |  | T/C or C/C | 43 | 71,67 | 17 | 53,13 | 202 | 59,24 | 176 | 57,52 | 0.66 (0.30-1.49) | 0.32 | 1.14 (0.65-2.01) | 0.65 | . | . | . |
| NFKB1 ---tag | rs230541 | A/A | 14 | 23,33 | 11 | 34,38 | 111 | 32,55 | 106 | 34,64 | 1.00 (.-.) | . | 1.13 (0.58-2.20) | 0.72 | 0.48 | 0.97 | 0.50 |
| NFKB1 ---tag |  | A/G or G/G | 46 | 76,67 | 21 | 65,63 | 230 | 67,45 | 200 | 65,36 | 0.80 (0.36-1.82) | 0.60 | 1.24 (0.65-2.39) | 0.52 | . | . | . |
| NFKB1 ---NA | rs230547 | C/C | 52 | 86,67 | 26 | 81,25 | 267 | 78,30 | 251 | 82,03 | 1.00 (.-.) | . | 1.47 (0.91-2.37) | 0.11 | 0.48 | 0.97 | 0.50 |
| NFKB1 ---NA |  | C/T or T/T | 8 | 13,33 | 6 | 18,75 | 74 | 21,70 | 55 | 17,97 | 1.20 (0.44-3.25) | 0.72 | 1.19 (0.68-2.08) | 0.54 | . | . | . |
| NFKB1 ---tag | rs3774934 | G/G | 51 | 85,00 | 25 | 78,13 | 261 | 76,54 | 245 | 80,07 | 1.00 (.-.) | . | 1.48 (0.92-2.39) | 0.11 | 0.46 | 0.97 | 0.50 |
| NFKB1 ---tag |  | G/A or A/A | 9 | 15,00 | 7 | 21,88 | 80 | 23,46 | 61 | 19,93 | 1.20 (0.44-3.25) | 0.72 | 1.18 (0.68-2.04) | 0.55 | . | . | . |
| NFKB1 ---tag | rs3774968 | G/G | 29 | 48,33 | 10 | 31,25 | 108 | 31,67 | 91 | 29,74 | 1.00 (.-.) | . | 2.92 (1.25-6.82) | 0.01 | 0.02 | 0.85 | 0.23 |
| NFKB1 ---tag |  | G/A or A/A | 31 | 51,67 | 22 | 68,75 | 233 | 68,33 | 215 | 70,26 | 2.64 (1.04-6.71) | 0.04 | 2.51 (1.08-5.83) | 0.03 | . | . | . |
| NFKB1 ---NA | rs4648022 | C/C | 49 | 81,67 | 28 | 87,50 | 291 | 85,34 | 260 | 84,97 | 1.00 (.-.) | . | 1.29 (0.80-2.06) | 0.29 | 0.45 | 0.97 | 0.50 |
| NFKB1 ---NA |  | C/T or T/T | 11 | 18,33 | 4 | 12,50 | 50 | 14,66 | 46 | 15,03 | 0.99 (0.33-2.94) | 0.99 | 1.96 (1.13-3.41) | 0.02 | . | . | . |
| NFKB1 ---NA | rs4648090 | G/G | 43 | 71,67 | 27 | 84,38 | 254 | 74,49 | 228 | 74,51 | 1.00 (.-.) | . | 1.21 (0.75-1.95) | 0.43 | 0.30 | 0.97 | 0.47 |
| NFKB1 ---NA |  | G/A or A/A | 17 | 28,33 | 5 | 15,63 | 87 | 25,51 | 78 | 25,49 | 0.69 (0.24-2.04) | 0.51 | 1.47 (0.89-2.45) | 0.14 | . | . | . |
| NFKB1 ---tag | rs4648110 | T/T | 40 | 66,67 | 22 | 68,75 | 219 | 64,22 | 199 | 65,03 | 1.00 (.-.) | . | 1.08 (0.65-1.80) | 0.75 | 0.13 | 0.97 | 0.45 |
| NFKB1 ---tag |  | T/A or A/A | 20 | 33,33 | 10 | 31,25 | 122 | 35,78 | 107 | 34,97 | 0.49 (0.19-1.25) | 0.13 | 1.10 (0.65-1.85) | 0.73 | . | . | . |
| NFKB1 ---tag | rs4648141 | G/G | 44 | 73,33 | 22 | 68,75 | 243 | 71,26 | 219 | 71,57 | 1.00 (.-.) | . | 1.07 (0.64-1.78) | 0.80 | 0.11 | 0.97 | 0.45 |
| NFKB1 ---tag |  | G/A or A/A | 16 | 26,67 | 10 | 31,25 | 98 | 28,74 | 87 | 28,43 | 0.54 (0.22-1.32) | 0.18 | 1.20 (0.70-2.07) | 0.51 | . | . | . |
| NFKB1 ---tag | rs4698863 | C/C | 17 | 28,33 | 16 | 50,00 | 158 | 46,33 | 140 | 45,75 | 1.00 (.-.) | . | 1.08 (0.61-1.91) | 0.79 | 0.26 | 0.97 | 0.47 |
| NFKB1 ---tag |  | C/T or T/T | 43 | 71,67 | 16 | 50,00 | 183 | 53,67 | 166 | 54,25 | 0.67 (0.30-1.50) | 0.33 | 1.18 (0.66-2.08) | 0.58 | . | . | . |
| NFKB1 ---NA | rs7674640 | C/C | 9 | 15,00 | 7 | 21,88 | 75 | 21,99 | 76 | 24,84 | 1.00 (.-.) | . | 0.75 (0.33-1.67) | 0.48 | 0.12 | 0.97 | 0.45 |
| NFKB1 ---NA |  | C/T or T/T | 51 | 85,00 | 25 | 78,13 | 266 | 78,01 | 230 | 75,16 | 0.49 (0.20-1.18) | 0.11 | 0.79 (0.36-1.72) | 0.55 | . | . | . |
| NFKB1 ---tag | rs909332 | A/A | 56 | 93,33 | 27 | 84,38 | 306 | 89,74 | 280 | 91,50 | 1.00 (.-.) | . | 1.54 (0.96-2.47) | 0.08 | 0.23 | 0.97 | 0.47 |
| NFKB1 ---tag |  | A/T or T/T | 4 | 6,67 | 5 | 15,63 | 35 | 10,26 | 26 | 8,50 | 2.40 (0.88-6.49) | 0.09 | 1.82 (0.96-3.44) | 0.07 | . | . | . |
| NFKB1 ---tag | rs997476 | C/C | 56 | 93,33 | 27 | 84,38 | 303 | 88,86 | 276 | 90,20 | 1.00 (.-.) | . | 1.24 (0.79-1.96) | 0.35 | 0.30 | 0.97 | 0.47 |
| NFKB1 ---tag |  | C/A or A/A | 4 | 6,67 | 5 | 15,63 | 38 | 11,14 | 30 | 9,80 | 0.39 (0.09-1.68) | 0.20 | 1.01 (0.56-1.81) | 0.98 | . | . | . |
| NME1 ---NA | rs10514981 | T/T | 32 | 53,33 | 21 | 65,63 | 214 | 62,76 | 191 | 62,42 | 1.00 (.-.) | . | 1.13 (0.68-1.88) | 0.64 | 0.21 | 0.97 | 0.53 |
| NME1 ---NA |  | T/G or G/G | 28 | 46,67 | 11 | 34,38 | 127 | 37,24 | 115 | 37,58 | 0.57 (0.24-1.36) | 0.20 | 1.13 (0.66-1.91) | 0.66 | . | . | . |
| NME1 ---NA | rs11651252 | T/T | 49 | 81,67 | 26 | 81,25 | 310 | 90,91 | 270 | 88,24 | 1.00 (.-.) | . | 1.42 (0.90-2.24) | 0.14 | 0.65 | 0.97 | 0.92 |
| NME1 ---NA |  | T/C or C/C | 11 | 18,33 | 6 | 18,75 | 31 | 9,09 | 36 | 11,76 | 1.68 (0.50-5.66) | 0.41 | 1.74 (0.97-3.12) | 0.06 | . | . | . |
| NME1 ---tag | rs11652793 | T/T | 36 | 60,00 | 22 | 68,75 | 220 | 64,52 | 209 | 68,30 | 1.00 (.-.) | . | 1.12 (0.69-1.84) | 0.64 | 0.18 | 0.97 | 0.53 |
| NME1 ---tag |  | T/C or C/C | 24 | 40,00 | 10 | 31,25 | 121 | 35,48 | 97 | 31,70 | 0.51 (0.20-1.27) | 0.15 | 1.07 (0.63-1.82) | 0.79 | . | . | . |
| NME1 ---NA | rs11868380 | C/C | 36 | 60,00 | 18 | 56,25 | 211 | 61,88 | 191 | 62,42 | 1.00 (.-.) | . | 1.45 (0.77-2.73) | 0.24 | 0.84 | 0.99 | 0.92 |
| NME1 ---NA |  | C/G or G/G | 24 | 40,00 | 14 | 43,75 | 130 | 38,12 | 115 | 37,58 | 1.15 (0.51-2.56) | 0.74 | 1.52 (0.80-2.90) | 0.20 | . | . | . |
| NME1 ---NA | rs1558252 | T/T | 34 | 56,67 | 17 | 53,13 | 175 | 51,32 | 136 | 44,44 | 1.00 (.-.) | . | 1.25 (0.70-2.23) | 0.45 | 0.69 | 0.97 | 0.92 |
| NME1 ---NA |  | T/C or C/C | 26 | 43,33 | 15 | 46,88 | 166 | 48,68 | 170 | 55,56 | 1.03 (0.46-2.29) | 0.95 | 1.53 (0.86-2.70) | 0.15 | . | . | . |
| NME1 ---NA | rs1558253 | T/T | 57 | 95,00 | 30 | 93,75 | 304 | 89,15 | 266 | 86,93 | 1.00 (.-.) | . | 1.32 (0.84-2.07) | 0.23 | 0.59 | 0.97 | 0.92 |
| NME1 ---NA |  | T/G or G/G | 3 | 5,00 | 2 | 6,25 | 37 | 10,85 | 40 | 13,07 | 0.73 (0.17-3.20) | 0.68 | 1.44 (0.83-2.51) | 0.19 | . | . | . |
| NME1 ---tag | rs16949683 | C/C | 54 | 90,00 | 28 | 87,50 | 323 | 94,72 | 282 | 92,16 | 1.00 (.-.) | . | 1.52 (0.96-2.42) | 0.08 | 0.15 | 0.97 | 0.53 |
| NME1 ---tag |  | C/T or T/T | 6 | 10,00 | 4 | 12,50 | 18 | 5,28 | 24 | 7,84 | 3.79 (1.27-11.30) | 0.02 | 2.25 (1.19-4.27) | 0.01 | . | . | . |
| NME1 ---tag | rs2318784 | C/C | 40 | 66,67 | 22 | 68,75 | 270 | 79,18 | 227 | 74,18 | 1.00 (.-.) | . | 1.40 (0.84-2.32) | 0.20 | 0.88 | 0.99 | 0.92 |
| NME1 ---tag |  | C/T or T/T | 20 | 33,33 | 10 | 31,25 | 71 | 20,82 | 79 | 25,82 | 1.42 (0.58-3.48) | 0.44 | 1.84 (1.07-3.16) | 0.03 | . | . | . |
| NME1 ---NA | rs2318785 | G/G | 24 | 40,00 | 8 | 25,00 | 102 | 29,91 | 88 | 28,76 | 1.00 (.-.) | . | 3.00 (1.20-7.51) | 0.02 | 0.02 | 0.85 | 0.29 |
| NME1 ---NA |  | G/A or A/A | 36 | 60,00 | 24 | 75,00 | 239 | 70,09 | 218 | 71,24 | 2.69 (0.99-7.29) | 0.05 | 2.64 (1.07-6.49) | 0.04 | . | . | . |
| NME1 ---tag | rs3760469 | G/G | 23 | 38,33 | 9 | 28,13 | 89 | 26,10 | 69 | 22,55 | 1.00 (.-.) | . | 2.55 (1.08-6.01) | 0.03 | 0.06 | 0.94 | 0.29 |
| NME1 ---tag |  | G/T or T/T | 37 | 61,67 | 23 | 71,88 | 252 | 73,90 | 237 | 77,45 | 2.24 (0.87-5.78) | 0.09 | 2.29 (1.00-5.25) | 0.05 | . | . | . |
| NME1 ---NA | rs4605213 | G/G | 23 | 38,33 | 15 | 46,88 | 136 | 39,88 | 134 | 43,79 | 1.00 (.-.) | . | 1.32 (0.73-2.39) | 0.36 | 0.85 | 0.99 | 0.92 |
| NME1 ---NA |  | G/C or C/C | 37 | 61,67 | 17 | 53,13 | 205 | 60,12 | 172 | 56,21 | 0.91 (0.41-2.00) | 0.81 | 1.30 (0.72-2.33) | 0.38 | . | . | . |
| NME1 ---NA | rs7207090 | A/A | 14 | 23,33 | 11 | 34,38 | 86 | 25,22 | 74 | 24,18 | 1.00 (.-.) | . | 1.33 (0.63-2.77) | 0.46 | 0.88 | 0.99 | 0.92 |
| NME1 ---NA |  | A/T or T/T | 46 | 76,67 | 21 | 65,63 | 255 | 74,78 | 232 | 75,82 | 0.86 (0.37-1.99) | 0.73 | 1.23 (0.61-2.46) | 0.57 | . | . | . |
| NME1 ---tag | rs7222463 | A/A | 9 | 15,00 | 9 | 28,13 | 88 | 25,81 | 87 | 28,43 | 1.00 (.-.) | . | 1.15 (0.48-2.73) | 0.75 | 0.67 | 0.97 | 0.92 |
| NME1 ---tag |  | A/C or C/C | 51 | 85,00 | 23 | 71,88 | 253 | 74,19 | 219 | 71,57 | 0.72 (0.28-1.84) | 0.50 | 1.03 (0.44-2.39) | 0.94 | . | . | . |
| NME1 ---tag | rs7226059 | C/C | 29 | 48,33 | 11 | 34,38 | 147 | 43,11 | 131 | 42,81 | 1.00 (.-.) | . | 2.09 (1.08-4.07) | 0.03 | 0.05 | 0.94 | 0.29 |
| NME1 ---tag |  | C/T or T/T | 31 | 51,67 | 21 | 65,63 | 194 | 56,89 | 175 | 57,19 | 2.24 (0.99-5.05) | 0.05 | 2.03 (1.05-3.92) | 0.04 | . | . | . |
| NME1 ---NA | rs880178 | G/G | 13 | 21,67 | 10 | 31,25 | 89 | 26,10 | 71 | 23,20 | 1.00 (.-.) | . | 1.33 (0.62-2.89) | 0.47 | 0.92 | 1.00 | 0.92 |
| NME1 ---NA |  | G/T or T/T | 47 | 78,33 | 22 | 68,75 | 252 | 73,90 | 235 | 76,80 | 0.91 (0.39-2.14) | 0.82 | 1.27 (0.61-2.64) | 0.52 | . | . | . |
| NME2 ---tag | rs7220360 | C/C | 9 | 15,00 | 9 | 28,13 | 88 | 25,81 | 87 | 28,43 | 1.00 (.-.) | . | 1.15 (0.48-2.73) | 0.75 | 0.67 | 0.97 | 0.67 |
| NME2 ---tag |  | C/G or G/G | 51 | 85,00 | 23 | 71,88 | 253 | 74,19 | 219 | 71,57 | 0.72 (0.28-1.84) | 0.50 | 1.03 (0.44-2.39) | 0.94 | . | . | . |
| PON1 ---tag | rs2269829 | A/A | 34 | 56,67 | 18 | 56,25 | 176 | 51,61 | 152 | 49,67 | 1.00 (.-.) | . | 1.46 (0.82-2.59) | 0.20 | 0.78 | 0.97 | 0.99 |
| PON1 ---tag |  | A/G or G/G | 26 | 43,33 | 14 | 43,75 | 165 | 48,39 | 154 | 50,33 | 1.05 (0.47-2.37) | 0.90 | 1.35 (0.76-2.42) | 0.30 | . | . | . |
| PON1 ---tag | rs3917527 | A/A | 54 | 90,00 | 30 | 93,75 | 303 | 88,86 | 284 | 92,81 | 1.00 (.-.) | . | 1.45 (0.93-2.26) | 0.10 | 0.48 | 0.97 | 0.99 |
| PON1 ---tag |  | A/G or G/G | 6 | 10,00 | 2 | 6,25 | 38 | 11,14 | 22 | 7,19 | 1.10 (0.26-4.72) | 0.90 | 0.88 (0.48-1.65) | 0.70 | . | . | . |
| PON1 ---tag | rs3917538 | C/C | 38 | 63,33 | 19 | 59,38 | 201 | 58,94 | 162 | 52,94 | 1.00 (.-.) | . | 1.36 (0.78-2.39) | 0.28 | 0.99 | 1.00 | 0.99 |
| PON1 ---tag |  | C/T or T/T | 22 | 36,67 | 13 | 40,63 | 140 | 41,06 | 144 | 47,06 | 1.12 (0.49-2.55) | 0.79 | 1.53 (0.87-2.70) | 0.14 | . | . | . |
| PON1 ---tag | rs757158 | C/C | 20 | 33,33 | 11 | 34,38 | 139 | 40,76 | 107 | 34,97 | 1.00 (.-.) | . | 1.29 (0.63-2.62) | 0.48 | 0.83 | 0.99 | 0.99 |
| PON1 ---tag |  | C/T or T/T | 40 | 66,67 | 21 | 65,63 | 202 | 59,24 | 199 | 65,03 | 1.00 (0.43-2.29) | 0.99 | 1.41 (0.71-2.83) | 0.33 | . | . | . |
| PON1 ---candidate | rs854560 | A/A | 19 | 31,67 | 14 | 43,75 | 119 | 34,90 | 131 | 42,81 | 1.00 (.-.) | . | 1.26 (0.64-2.45) | 0.50 | 0.76 | 0.97 | 0.99 |
| PON1 ---candidate |  | A/T or T/T | 41 | 68,33 | 18 | 56,25 | 222 | 65,10 | 175 | 57,19 | 0.79 (0.35-1.80) | 0.58 | 1.14 (0.59-2.22) | 0.70 | . | . | . |
| PRDM2 ---tag | rs1015370 | C/C | 29 | 48,33 | 18 | 56,25 | 177 | 51,91 | 176 | 57,52 | 1.00 (.-.) | . | 1.57 (0.88-2.83) | 0.13 | 0.48 | 0.97 | 0.84 |
| PRDM2 ---tag |  | C/T or T/T | 31 | 51,67 | 14 | 43,75 | 164 | 48,09 | 130 | 42,48 | 1.16 (0.52-2.58) | 0.72 | 1.33 (0.74-2.42) | 0.34 | . | . | . |
| PRDM2 ---tag | rs1203634 | A/A | 38 | 63,33 | 23 | 71,88 | 213 | 62,46 | 189 | 61,76 | 1.00 (.-.) | . | 1.14 (0.70-1.87) | 0.59 | 0.20 | 0.97 | 0.84 |
| PRDM2 ---tag |  | A/G or G/G | 22 | 36,67 | 9 | 28,13 | 128 | 37,54 | 117 | 38,24 | 0.54 (0.21-1.36) | 0.19 | 1.13 (0.68-1.89) | 0.64 | . | . | . |
| PRDM2 ---tag | rs1203645 | A/A | 26 | 43,33 | 15 | 46,88 | 136 | 39,88 | 132 | 43,14 | 1.00 (.-.) | . | 1.29 (0.71-2.34) | 0.39 | 0.77 | 0.97 | 0.84 |
| PRDM2 ---tag |  | A/C or C/C | 34 | 56,67 | 17 | 53,13 | 205 | 60,12 | 174 | 56,86 | 0.84 (0.38-1.86) | 0.67 | 1.23 (0.68-2.21) | 0.49 | . | . | . |
| PRDM2 ---tag | rs1406416 | C/C | 34 | 56,67 | 14 | 43,75 | 183 | 53,67 | 151 | 49,35 | 1.00 (.-.) | . | 1.62 (0.80-3.28) | 0.18 | 0.59 | 0.97 | 0.84 |
| PRDM2 ---tag |  | C/T or T/T | 26 | 43,33 | 18 | 56,25 | 158 | 46,33 | 155 | 50,65 | 1.48 (0.64-3.42) | 0.36 | 1.89 (0.94-3.81) | 0.08 | . | . | . |
| PRDM2 ---candidate | rs17350795 | G/G | 59 | 98,33 | 31 | 96,88 | 324 | 95,01 | 291 | 95,10 | 1.00 (.-.) | . | 1.41 (0.91-2.19) | 0.12 | 0.76 | 0.97 | 0.84 |
| PRDM2 ---candidate |  | G/A or A/A | 1 | 1,67 | 1 | 3,13 | 17 | 4,99 | 15 | 4,90 | 0.83 (0.11-6.37) | 0.86 | 0.83 (0.39-1.77) | 0.63 | . | . | . |
| PRDM2 ---tag | rs1980472 | C/C | 37 | 61,67 | 20 | 62,50 | 191 | 56,01 | 171 | 55,88 | 1.00 (.-.) | . | 1.24 (0.74-2.10) | 0.41 | 0.52 | 0.97 | 0.84 |
| PRDM2 ---tag |  | C/G or G/G | 23 | 38,33 | 12 | 37,50 | 150 | 43,99 | 135 | 44,12 | 0.68 (0.29-1.60) | 0.38 | 1.13 (0.66-1.93) | 0.65 | . | . | . |
| PRDM2 ---tag | rs2235515 | G/G | 34 | 56,67 | 18 | 56,25 | 207 | 60,70 | 181 | 59,15 | 1.00 (.-.) | . | 1.48 (0.82-2.67) | 0.19 | 0.70 | 0.97 | 0.84 |
| PRDM2 ---tag |  | G/A or A/A | 26 | 43,33 | 14 | 43,75 | 134 | 39,30 | 125 | 40,85 | 1.11 (0.50-2.45) | 0.80 | 1.39 (0.76-2.53) | 0.28 | . | . | . |
| PRDM2 ---tag | rs2244634 | A/A | 35 | 58,33 | 22 | 68,75 | 227 | 66,57 | 201 | 65,69 | 1.00 (.-.) | . | 1.40 (0.85-2.31) | 0.19 | 0.89 | 0.99 | 0.89 |
| PRDM2 ---tag |  | A/C or C/C | 25 | 41,67 | 10 | 31,25 | 114 | 33,43 | 105 | 34,31 | 1.02 (0.41-2.54) | 0.96 | 1.34 (0.79-2.27) | 0.27 | . | . | . |
| PRDM2 ---tag | rs2245213 | G/G | 41 | 68,33 | 23 | 71,88 | 254 | 74,49 | 222 | 72,55 | 1.00 (.-.) | . | 1.43 (0.86-2.36) | 0.17 | 0.76 | 0.97 | 0.84 |
| PRDM2 ---tag |  | G/T or T/T | 19 | 31,67 | 9 | 28,13 | 87 | 25,51 | 84 | 27,45 | 1.13 (0.46-2.75) | 0.79 | 1.39 (0.81-2.39) | 0.23 | . | . | . |
| PRDM2 ---tag | rs2294484 | C/C | 49 | 81,67 | 25 | 78,13 | 283 | 82,99 | 255 | 83,33 | 1.00 (.-.) | . | 1.30 (0.82-2.07) | 0.27 | 0.55 | 0.97 | 0.84 |
| PRDM2 ---tag |  | C/G or G/G | 11 | 18,33 | 7 | 21,88 | 58 | 17,01 | 51 | 16,67 | 0.94 (0.32-2.78) | 0.91 | 1.72 (1.00-2.95) | 0.05 | . | . | . |
| PRDM2 ---tag | rs2744689 | G/G | 42 | 70,00 | 24 | 75,00 | 258 | 75,66 | 220 | 71,90 | 1.00 (.-.) | . | 1.44 (0.88-2.33) | 0.15 | 0.69 | 0.97 | 0.84 |
| PRDM2 ---tag |  | G/A or A/A | 18 | 30,00 | 8 | 25,00 | 83 | 24,34 | 86 | 28,10 | 1.21 (0.47-3.13) | 0.70 | 1.41 (0.83-2.38) | 0.20 | . | . | . |
| PRDM2 ---tag | rs6690270 | A/A | 25 | 41,67 | 11 | 34,38 | 136 | 39,88 | 112 | 36,60 | 1.00 (.-.) | . | 1.54 (0.73-3.24) | 0.26 | 0.73 | 0.97 | 0.84 |
| PRDM2 ---tag |  | A/G or G/G | 35 | 58,33 | 21 | 65,63 | 205 | 60,12 | 194 | 63,40 | 1.37 (0.58-3.22) | 0.47 | 1.80 (0.86-3.75) | 0.12 | . | . | . |
| RRM1 ---tag | rs10835601 | G/G | 32 | 53,33 | 15 | 46,88 | 174 | 51,03 | 143 | 46,73 | 1.00 (.-.) | . | 1.56 (0.85-2.86) | 0.16 | 0.48 | 0.97 | 0.97 |
| RRM1 ---tag |  | G/A or A/A | 28 | 46,67 | 17 | 53,13 | 167 | 48,97 | 163 | 53,27 | 1.65 (0.74-3.68) | 0.22 | 1.90 (1.04-3.49) | 0.04 | . | . | . |
| RRM1 ---tag | rs10835613 | C/C | 23 | 38,33 | 9 | 28,13 | 129 | 37,83 | 106 | 34,64 | 1.00 (.-.) | . | 1.66 (0.79-3.48) | 0.18 | 0.48 | 0.97 | 0.97 |
| RRM1 ---tag |  | C/G or G/G | 37 | 61,67 | 23 | 71,88 | 212 | 62,17 | 200 | 65,36 | 1.63 (0.69-3.82) | 0.26 | 1.96 (0.96-4.04) | 0.07 | . | . | . |
| RRM1 ---NA | rs10835677 | G/G | 53 | 88,33 | 27 | 84,38 | 277 | 81,23 | 249 | 81,37 | 1.00 (.-.) | . | 1.37 (0.86-2.17) | 0.18 | 0.97 | 1.00 | 0.97 |
| RRM1 ---NA |  | G/A or A/A | 7 | 11,67 | 5 | 15,63 | 64 | 18,77 | 57 | 18,63 | 0.99 (0.29-3.38) | 0.98 | 1.38 (0.82-2.34) | 0.22 | . | . | . |
| RRM1 ---tag | rs10835678 | A/A | 54 | 90,00 | 29 | 90,63 | 302 | 88,56 | 274 | 89,54 | 1.00 (.-.) | . | 1.41 (0.91-2.19) | 0.13 | 0.81 | 0.97 | 0.97 |
| RRM1 ---tag |  | A/G or G/G | 6 | 10,00 | 3 | 9,38 | 39 | 11,44 | 32 | 10,46 | 1.08 (0.14-8.27) | 0.94 | 1.16 (0.65-2.09) | 0.61 | . | . | . |
| RRM1 ---tag | rs12288551 | C/C | 56 | 93,33 | 30 | 93,75 | 316 | 92,67 | 269 | 87,91 | 1.00 (.-.) | . | 1.40 (0.89-2.18) | 0.14 | 0.58 | 0.97 | 0.97 |
| RRM1 ---tag |  | C/G or G/G | 4 | 6,67 | 2 | 6,25 | 25 | 7,33 | 37 | 12,09 | 2.03 (0.47-8.79) | 0.34 | 1.80 (1.01-3.23) | 0.05 | . | . | . |
| RRM1 ---NA | rs12806698 | C/C | 30 | 50,00 | 18 | 56,25 | 188 | 55,13 | 154 | 50,33 | 1.00 (.-.) | . | 1.08 (0.63-1.85) | 0.78 | 0.20 | 0.97 | 0.97 |
| RRM1 ---NA |  | C/A or A/A | 30 | 50,00 | 14 | 43,75 | 153 | 44,87 | 152 | 49,67 | 0.69 (0.30-1.59) | 0.39 | 1.32 (0.77-2.25) | 0.32 | . | . | . |
| RRM1 ---NA | rs1465952 | T/T | 47 | 78,33 | 25 | 78,13 | 280 | 82,11 | 255 | 83,33 | 1.00 (.-.) | . | 1.40 (0.88-2.24) | 0.16 | 0.87 | 0.99 | 0.97 |
| RRM1 ---NA |  | T/C or C/C | 13 | 21,67 | 7 | 21,88 | 61 | 17,89 | 51 | 16,67 | 0.91 (0.31-2.73) | 0.87 | 1.16 (0.67-2.01) | 0.59 | . | . | . |
| RRM1 ---tag | rs4910904 | A/A | 29 | 48,33 | 10 | 31,25 | 166 | 48,68 | 126 | 41,18 | 1.00 (.-.) | . | 1.61 (0.77-3.37) | 0.20 | 0.58 | 0.97 | 0.97 |
| RRM1 ---tag |  | A/G or G/G | 31 | 51,67 | 22 | 68,75 | 175 | 51,32 | 180 | 58,82 | 1.74 (0.74-4.08) | 0.21 | 2.18 (1.06-4.50) | 0.04 | . | . | . |
| RRM1 ---tag | rs7103860 | T/T | 44 | 73,33 | 22 | 68,75 | 264 | 77,42 | 245 | 80,07 | 1.00 (.-.) | . | 1.35 (0.83-2.21) | 0.23 | 0.92 | 1.00 | 0.97 |
| RRM1 ---tag |  | T/C or C/C | 16 | 26,67 | 10 | 31,25 | 77 | 22,58 | 61 | 19,93 | 0.87 (0.34-2.24) | 0.78 | 1.24 (0.72-2.16) | 0.44 | . | . | . |
| RRM1 ---tag | rs7115496 | C/C | 54 | 90,00 | 29 | 90,63 | 290 | 85,04 | 271 | 88,56 | 1.00 (.-.) | . | 1.42 (0.91-2.20) | 0.12 | 0.85 | 0.99 | 0.97 |
| RRM1 ---tag |  | C/T or T/T | 6 | 10,00 | 3 | 9,38 | 51 | 14,96 | 35 | 11,44 | 0.96 (0.13-7.34) | 0.97 | 1.11 (0.62-1.96) | 0.73 | . | . | . |
| RRM2 ---NA | rs1138729 | A/A | 43 | 71,67 | 20 | 62,50 | 247 | 72,43 | 225 | 73,53 | 1.00 (.-.) | . | 1.61 (0.92-2.84) | 0.10 | 0.36 | 0.97 | 0.66 |
| RRM2 ---NA |  | A/G or G/G | 17 | 28,33 | 12 | 37,50 | 94 | 27,57 | 81 | 26,47 | 1.51 (0.67-3.40) | 0.32 | 1.62 (0.89-2.94) | 0.11 | . | . | . |
| RRM2 ---tag | rs4668664 | G/G | 26 | 43,33 | 15 | 46,88 | 167 | 48,97 | 156 | 50,98 | 1.00 (.-.) | . | 1.48 (0.79-2.78) | 0.22 | 0.72 | 0.97 | 0.72 |
| RRM2 ---tag |  | G/A or A/A | 34 | 56,67 | 17 | 53,13 | 174 | 51,03 | 150 | 49,02 | 1.09 (0.49-2.45) | 0.83 | 1.39 (0.74-2.62) | 0.31 | . | . | . |
| RRM2 ---NA | rs6741290 | C/C | 17 | 28,33 | 8 | 25,00 | 102 | 29,91 | 105 | 34,31 | 1.00 (.-.) | . | 1.84 (0.74-4.59) | 0.19 | 0.44 | 0.97 | 0.66 |
| RRM2 ---NA |  | C/T or T/T | 43 | 71,67 | 24 | 75,00 | 239 | 70,09 | 201 | 65,69 | 1.33 (0.49-3.58) | 0.57 | 1.65 (0.67-4.06) | 0.28 | . | . | . |
| RRM2 ---tag | rs7574663 | C/C | 40 | 66,67 | 17 | 53,13 | 211 | 61,88 | 199 | 65,03 | 1.00 (.-.) | . | 1.56 (0.87-2.82) | 0.14 | 0.49 | 0.97 | 0.66 |
| RRM2 ---tag |  | C/G or G/G | 20 | 33,33 | 15 | 46,88 | 130 | 38,12 | 107 | 34,97 | 1.22 (0.55-2.72) | 0.62 | 1.42 (0.78-2.60) | 0.25 | . | . | . |
| SHMT1 ---candidate | rs1979277 | G/G | 30 | 50,00 | 20 | 62,50 | 151 | 44,28 | 150 | 49,02 | 1.00 (.-.) | . | 1.11 (0.66-1.89) | 0.69 | 0.22 | 0.97 | 0.22 |
| SHMT1 ---candidate |  | G/A or A/A | 30 | 50,00 | 12 | 37,50 | 190 | 55,72 | 156 | 50,98 | 0.47 (0.20-1.10) | 0.08 | 0.90 (0.53-1.54) | 0.71 | . | . | . |
| SHMT1 ---tag | rs2168781 | G/G | 21 | 35,00 | 18 | 56,25 | 102 | 29,91 | 113 | 36,93 | 1.00 (.-.) | . | 0.94 (0.53-1.64) | 0.82 | 0.06 | 0.94 | 0.12 |
| SHMT1 ---tag |  | G/C or C/C | 39 | 65,00 | 14 | 43,75 | 239 | 70,09 | 193 | 63,07 | 0.38 (0.16-0.88) | 0.02 | 0.80 (0.46-1.40) | 0.44 | . | . | . |
| SHMT1 ---tag | rs4924849 | C/C | 30 | 50,00 | 20 | 62,50 | 160 | 46,92 | 158 | 51,63 | 1.00 (.-.) | . | 1.10 (0.65-1.87) | 0.71 | 0.22 | 0.97 | 0.22 |
| SHMT1 ---tag |  | C/T or T/T | 30 | 50,00 | 12 | 37,50 | 181 | 53,08 | 148 | 48,37 | 0.47 (0.20-1.11) | 0.09 | 0.90 (0.53-1.55) | 0.71 | . | . | . |
| SHMT1 ---candidate literature | rs9909104 | T/T | 24 | 40,00 | 18 | 56,25 | 184 | 53,96 | 165 | 53,92 | 1.00 (.-.) | . | 0.84 (0.47-1.51) | 0.57 | 0.04 | 0.94 | 0.12 |
| SHMT1 ---candidate literature |  | T/C or C/C | 36 | 60,00 | 14 | 43,75 | 157 | 46,04 | 141 | 46,08 | 0.46 (0.21-1.02) | 0.06 | 0.93 (0.52-1.69) | 0.82 | . | . | . |
| SHMT2 ---tag | rs10876968 | G/G | 27 | 45,00 | 19 | 59,38 | 179 | 52,49 | 168 | 54,90 | 1.00 (.-.) | . | 1.19 (0.68-2.09) | 0.53 | 0.49 | 0.97 | 0.63 |
| SHMT2 ---tag |  | G/T or T/T | 33 | 55,00 | 13 | 40,63 | 162 | 47,51 | 138 | 45,10 | 0.69 (0.30-1.56) | 0.37 | 1.11 (0.63-1.96) | 0.72 | . | . | . |
| SHMT2 ---tag | rs1800165 | T/T | 27 | 45,00 | 17 | 53,13 | 161 | 47,21 | 152 | 49,67 | 1.00 (.-.) | . | 1.20 (0.69-2.10) | 0.52 | 0.50 | 0.97 | 0.63 |
| SHMT2 ---tag |  | T/C or C/C | 33 | 55,00 | 15 | 46,88 | 180 | 52,79 | 154 | 50,33 | 0.79 (0.35-1.79) | 0.58 | 1.28 (0.74-2.23) | 0.38 | . | . | . |
| SHMT2 ---tag | rs7133939 | T/T | 11 | 18,33 | 12 | 37,50 | 99 | 29,03 | 101 | 33,01 | 1.00 (.-.) | . | 1.22 (0.63-2.35) | 0.56 | 0.63 | 0.97 | 0.63 |
| SHMT2 ---tag |  | T/A or A/A | 49 | 81,67 | 20 | 62,50 | 242 | 70,97 | 205 | 66,99 | 0.73 (0.32-1.65) | 0.45 | 1.11 (0.59-2.09) | 0.76 | . | . | . |
| SHMT2 ---tag | rs7485577 | G/G | 30 | 50,00 | 20 | 62,50 | 181 | 53,08 | 166 | 54,25 | 1.00 (.-.) | . | 1.24 (0.72-2.13) | 0.43 | 0.58 | 0.97 | 0.63 |
| SHMT2 ---tag |  | G/A or A/A | 30 | 50,00 | 12 | 37,50 | 160 | 46,92 | 140 | 45,75 | 0.85 (0.37-1.96) | 0.71 | 1.35 (0.79-2.32) | 0.28 | . | . | . |
| SHMT2 ---tag | rs7489231 | T/T | 23 | 38,33 | 16 | 50,00 | 149 | 43,70 | 139 | 45,42 | 1.00 (.-.) | . | 1.15 (0.66-2.01) | 0.63 | 0.40 | 0.97 | 0.63 |
| SHMT2 ---tag |  | T/C or C/C | 37 | 61,67 | 16 | 50,00 | 192 | 56,30 | 167 | 54,58 | 0.83 (0.37-1.87) | 0.65 | 1.37 (0.79-2.38) | 0.27 | . | . | . |
| SLC19A1 ---candidate | rs1051266 | G/G | 19 | 31,67 | 8 | 25,00 | 110 | 32,26 | 86 | 28,10 | 1.00 (.-.) | . | 1.47 (0.66-3.30) | 0.35 | 0.85 | 0.99 | 0.86 |
| SLC19A1 ---candidate |  | G/A or A/A | 41 | 68,33 | 24 | 75,00 | 231 | 67,74 | 220 | 71,90 | 1.19 (0.49-2.91) | 0.70 | 1.61 (0.73-3.53) | 0.24 | . | . | . |
| SLC19A1 ---candidate literature | rs1131596 | T/T | 20 | 33,33 | 8 | 25,00 | 110 | 32,26 | 86 | 28,10 | 1.00 (.-.) | . | 1.54 (0.69-3.44) | 0.29 | 0.74 | 0.97 | 0.86 |
| SLC19A1 ---candidate literature |  | T/C or C/C | 40 | 66,67 | 24 | 75,00 | 231 | 67,74 | 220 | 71,90 | 1.28 (0.53-3.10) | 0.59 | 1.68 (0.77-3.69) | 0.19 | . | . | . |
| SLC19A1 ---tag | rs12483553 | G/G | 45 | 75,00 | 26 | 81,25 | 276 | 80,94 | 245 | 80,07 | 1.00 (.-.) | . | 1.32 (0.82-2.15) | 0.26 | 0.71 | 0.97 | 0.86 |
| SLC19A1 ---tag |  | G/A or A/A | 15 | 25,00 | 6 | 18,75 | 65 | 19,06 | 61 | 19,93 | 0.96 (0.38-2.45) | 0.94 | 1.53 (0.89-2.63) | 0.12 | . | . | . |
| SLC19A1 ---candidate literature | rs12659 | C/C | 19 | 31,67 | 8 | 25,00 | 115 | 33,72 | 91 | 29,74 | 1.00 (.-.) | . | 1.47 (0.66-3.28) | 0.35 | 0.86 | 0.99 | 0.86 |
| SLC19A1 ---candidate literature |  | C/T or T/T | 41 | 68,33 | 24 | 75,00 | 226 | 66,28 | 215 | 70,26 | 1.19 (0.49-2.91) | 0.70 | 1.61 (0.73-3.54) | 0.24 | . | . | . |
| SLC19A1 ---tag | rs3788190 | G/G | 18 | 30,00 | 8 | 25,00 | 109 | 31,96 | 88 | 28,76 | 1.00 (.-.) | . | 1.48 (0.66-3.29) | 0.34 | 0.83 | 0.99 | 0.86 |
| SLC19A1 ---tag |  | G/A or A/A | 42 | 70,00 | 24 | 75,00 | 232 | 68,04 | 218 | 71,24 | 1.14 (0.47-2.77) | 0.77 | 1.52 (0.70-3.34) | 0.29 | . | . | . |
| SLC19A1 ---tag | rs3788205 | C/C | 23 | 38,33 | 20 | 62,50 | 173 | 50,73 | 165 | 53,92 | 1.00 (.-.) | . | 1.14 (0.66-1.97) | 0.65 | 0.33 | 0.97 | 0.86 |
| SLC19A1 ---tag |  | C/T or T/T | 37 | 61,67 | 12 | 37,50 | 168 | 49,27 | 141 | 46,08 | 0.65 (0.29-1.47) | 0.30 | 1.13 (0.65-1.98) | 0.66 | . | . | . |
| SLC19A1 ---tag | rs7279664 | G/G | 21 | 35,00 | 14 | 43,75 | 135 | 39,59 | 127 | 41,50 | 1.00 (.-.) | . | 1.06 (0.56-2.04) | 0.85 | 0.36 | 0.97 | 0.86 |
| SLC19A1 ---tag |  | G/T or T/T | 39 | 65,00 | 18 | 56,25 | 206 | 60,41 | 179 | 58,50 | 0.63 (0.28-1.43) | 0.27 | 1.01 (0.53-1.92) | 0.97 | . | . | . |
| SLC29A1 ---NA | rs1057985 | C/C | 19 | 31,67 | 12 | 37,50 | 149 | 43,70 | 144 | 47,06 | 1.00 (.-.) | . | 1.48 (0.71-3.06) | 0.29 | 0.79 | 0.97 | 0.79 |
| SLC29A1 ---NA |  | C/T or T/T | 41 | 68,33 | 20 | 62,50 | 192 | 56,30 | 162 | 52,94 | 1.07 (0.46-2.51) | 0.87 | 1.41 (0.68-2.92) | 0.35 | . | . | . |
| SLC29A1 ---NA | rs6458375 | C/C | 37 | 61,67 | 16 | 50,00 | 179 | 52,49 | 173 | 56,54 | 1.00 (.-.) | . | 1.80 (0.97-3.32) | 0.06 | 0.19 | 0.97 | 0.31 |
| SLC29A1 ---NA |  | C/T or T/T | 23 | 38,33 | 16 | 50,00 | 162 | 47,51 | 133 | 43,46 | 1.45 (0.65-3.27) | 0.37 | 1.48 (0.79-2.75) | 0.22 | . | . | . |
| SLC29A1 ---NA | rs666462 | C/C | 18 | 30,00 | 7 | 21,88 | 83 | 24,34 | 92 | 30,07 | 1.00 (.-.) | . | 1.96 (0.84-4.59) | 0.12 | 0.30 | 0.97 | 0.36 |
| SLC29A1 ---NA |  | C/T or T/T | 42 | 70,00 | 25 | 78,13 | 258 | 75,66 | 214 | 69,93 | 1.50 (0.58-3.86) | 0.40 | 1.78 (0.78-4.08) | 0.17 | . | . | . |
| SLC29A1 ---NA | rs6905285 | A/A | 16 | 26,67 | 13 | 40,63 | 149 | 43,70 | 100 | 32,68 | 1.00 (.-.) | . | 0.66 (0.33-1.31) | 0.23 | 0.02 | 0.85 | 0.15 |
| SLC29A1 ---NA |  | A/T or T/T | 44 | 73,33 | 19 | 59,38 | 192 | 56,30 | 206 | 67,32 | 0.40 (0.18-0.91) | 0.03 | 0.76 (0.39-1.48) | 0.43 | . | . | . |
| SLC29A1 ---NA | rs693955 | G/G | 38 | 63,33 | 17 | 53,13 | 219 | 64,22 | 212 | 69,28 | 1.00 (.-.) | . | 1.70 (0.95-3.05) | 0.07 | 0.22 | 0.97 | 0.31 |
| SLC29A1 ---NA |  | G/T or T/T | 22 | 36,67 | 15 | 46,88 | 122 | 35,78 | 94 | 30,72 | 1.51 (0.68-3.37) | 0.31 | 1.51 (0.82-2.78) | 0.19 | . | . | . |
| SLC29A1 ---NA | rs747199 | C/C | 35 | 58,33 | 25 | 78,13 | 221 | 64,81 | 203 | 66,34 | 1.00 (.-.) | . | 1.14 (0.70-1.88) | 0.59 | 0.20 | 0.97 | 0.31 |
| SLC29A1 ---NA |  | C/G or G/G | 25 | 41,67 | 7 | 21,88 | 120 | 35,19 | 103 | 33,66 | 0.56 (0.22-1.43) | 0.23 | 1.19 (0.71-1.98) | 0.51 | . | . | . |
| SLC29A1 ---NA | rs9357436 | G/G | 37 | 61,67 | 26 | 81,25 | 248 | 72,73 | 221 | 72,22 | 1.00 (.-.) | . | 1.13 (0.69-1.84) | 0.64 | 0.16 | 0.97 | 0.31 |
| SLC29A1 ---NA |  | G/A or A/A | 23 | 38,33 | 6 | 18,75 | 93 | 27,27 | 85 | 27,78 | 0.56 (0.22-1.42) | 0.22 | 1.24 (0.74-2.09) | 0.42 | . | . | . |
| TCN2 ---tag | rs10418 | C/C | 32 | 53,33 | 17 | 53,13 | 208 | 61,00 | 166 | 54,25 | 1.00 (.-.) | . | 1.29 (0.71-2.33) | 0.40 | 0.74 | 0.97 | 0.74 |
| TCN2 ---tag |  | C/T or T/T | 28 | 46,67 | 15 | 46,88 | 133 | 39,00 | 140 | 45,75 | 0.97 (0.43-2.18) | 0.95 | 1.44 (0.80-2.62) | 0.23 | . | . | . |
| TCN2 ---candidate/singleton | rs1131603 | T/T | 57 | 95,00 | 27 | 84,38 | 300 | 87,98 | 269 | 87,91 | 1.00 (.-.) | . | 1.58 (0.99-2.52) | 0.06 | 0.09 | 0.97 | 0.20 |
| TCN2 ---candidate/singleton |  | T/C or C/C | 3 | 5,00 | 5 | 15,63 | 41 | 12,02 | 37 | 12,09 | 2.42 (0.82-7.18) | 0.11 | 1.26 (0.71-2.23) | 0.44 | . | . | . |
| TCN2 ---tag | rs1544468 | A/A | 21 | 35,00 | 9 | 28,13 | 96 | 28,15 | 71 | 23,20 | 1.00 (.-.) | . | 1.63 (0.73-3.63) | 0.24 | 0.61 | 0.97 | 0.66 |
| TCN2 ---tag |  | A/G or G/G | 39 | 65,00 | 23 | 71,88 | 245 | 71,85 | 235 | 76,80 | 1.26 (0.51-3.09) | 0.62 | 1.61 (0.73-3.51) | 0.24 | . | . | . |
| TCN2 ---candidate/tag | rs1801198 | C/C | 12 | 20,00 | 13 | 40,63 | 105 | 30,79 | 97 | 31,70 | 1.00 (.-.) | . | 0.68 (0.35-1.32) | 0.25 | 0.02 | 0.85 | 0.08 |
| TCN2 ---candidate/tag |  | C/G or G/G | 48 | 80,00 | 19 | 59,38 | 236 | 69,21 | 209 | 68,30 | 0.34 (0.15-0.78) | 0.01 | 0.65 (0.34-1.25) | 0.20 | . | . | . |
| TCN2 ---tag | rs4820872 | G/G | 14 | 23,33 | 13 | 40,63 | 131 | 38,42 | 115 | 37,58 | 1.00 (.-.) | . | 0.92 (0.48-1.77) | 0.80 | 0.16 | 0.97 | 0.25 |
| TCN2 ---tag |  | G/A or A/A | 46 | 76,67 | 19 | 59,38 | 210 | 61,58 | 191 | 62,42 | 0.53 (0.24-1.21) | 0.13 | 0.92 (0.48-1.76) | 0.80 | . | . | . |
| TCN2 ---tag | rs4820874 | A/A | 48 | 80,00 | 20 | 62,50 | 232 | 68,04 | 226 | 73,86 | 1.00 (.-.) | . | 1.72 (1.00-2.98) | 0.05 | 0.13 | 0.97 | 0.24 |
| TCN2 ---tag |  | A/G or G/G | 12 | 20,00 | 12 | 37,50 | 109 | 31,96 | 80 | 26,14 | 1.98 (0.86-4.53) | 0.11 | 1.71 (0.95-3.07) | 0.07 | . | . | . |
| TCN2 ---tag | rs4820886 | T/T | 54 | 90,00 | 25 | 78,13 | 264 | 77,42 | 256 | 83,66 | 1.00 (.-.) | . | 1.66 (1.03-2.67) | 0.04 | 0.02 | 0.85 | 0.08 |
| TCN2 ---tag |  | T/G or G/G | 6 | 10,00 | 7 | 21,88 | 77 | 22,58 | 50 | 16,34 | 3.34 (1.22-9.11) | 0.02 | 1.39 (0.80-2.43) | 0.24 | . | . | . |
| TCN2 ---candidate | rs4820889 | G/G | 55 | 91,67 | 31 | 96,88 | 312 | 91,50 | 287 | 93,79 | 1.00 (.-.) | . | 1.30 (0.84-2.01) | 0.24 | 0.38 | 0.97 | 0.54 |
| TCN2 ---candidate |  | G/A or A/A | 5 | 8,33 | 1 | 3,13 | 29 | 8,50 | 19 | 6,21 | 0.33 (0.04-2.46) | 0.28 | 0.99 (0.52-1.89) | 0.97 | . | . | . |
| TCN2 ---tag | rs5997711 | C/C | 14 | 23,33 | 14 | 43,75 | 116 | 34,02 | 107 | 34,97 | 1.00 (.-.) | . | 0.68 (0.36-1.28) | 0.23 | 0.01 | 0.85 | 0.08 |
| TCN2 ---tag |  | C/T or T/T | 46 | 76,67 | 18 | 56,25 | 225 | 65,98 | 199 | 65,03 | 0.34 (0.15-0.77) | 0.01 | 0.69 (0.37-1.28) | 0.24 | . | . | . |
| TCN2 ---tag | rs740234 | T/T | 44 | 73,33 | 21 | 65,63 | 222 | 65,10 | 197 | 64,38 | 1.00 (.-.) | . | 1.54 (0.89-2.67) | 0.12 | 0.48 | 0.97 | 0.62 |
| TCN2 ---tag |  | T/C or C/C | 16 | 26,67 | 11 | 34,38 | 119 | 34,90 | 109 | 35,62 | 1.35 (0.59-3.07) | 0.48 | 1.52 (0.85-2.69) | 0.16 | . | . | . |
| TCN2 ---tag | rs740235 | G/G | 24 | 40,00 | 13 | 40,63 | 125 | 36,66 | 91 | 29,74 | 1.00 (.-.) | . | 1.19 (0.60-2.35) | 0.62 | 0.60 | 0.97 | 0.66 |
| TCN2 ---tag |  | G/A or A/A | 36 | 60,00 | 19 | 59,38 | 216 | 63,34 | 215 | 70,26 | 0.83 (0.36-1.90) | 0.66 | 1.25 (0.64-2.44) | 0.52 | . | . | . |
| TCN2 ---candidate/singleton | rs9606756 | A/A | 53 | 88,33 | 23 | 71,88 | 264 | 77,42 | 252 | 82,35 | 1.00 (.-.) | . | 1.61 (0.99-2.62) | 0.06 | 0.09 | 0.97 | 0.20 |
| TCN2 ---candidate/singleton |  | A/G or G/G | 7 | 11,67 | 9 | 28,13 | 77 | 22,58 | 54 | 17,65 | 2.33 (0.90-6.02) | 0.08 | 1.48 (0.85-2.58) | 0.17 | . | . | . |
| TCN2 ---candidate | rs9621049 | C/C | 54 | 90,00 | 25 | 78,13 | 264 | 77,42 | 256 | 83,66 | 1.00 (.-.) | . | 1.66 (1.03-2.67) | 0.04 | 0.02 | 0.85 | 0.08 |
| TCN2 ---candidate |  | C/T or T/T | 6 | 10,00 | 7 | 21,88 | 77 | 22,58 | 50 | 16,34 | 3.34 (1.22-9.11) | 0.02 | 1.39 (0.80-2.43) | 0.24 | . | . | . |
| TK1 ---NA | rs1065769 | G/G | 32 | 53,33 | 11 | 34,38 | 161 | 47,21 | 146 | 47,71 | 1.00 (.-.) | . | 1.69 (0.85-3.35) | 0.14 | 0.44 | 0.97 | 0.66 |
| TK1 ---NA |  | G/A or A/A | 28 | 46,67 | 21 | 65,63 | 180 | 52,79 | 160 | 52,29 | 1.53 (0.67-3.50) | 0.32 | 1.84 (0.92-3.67) | 0.08 | . | . | . |
| TK1 ---NA | rs12232476 | G/G | 49 | 81,67 | 25 | 78,13 | 298 | 87,39 | 246 | 80,39 | 1.00 (.-.) | . | 1.26 (0.78-2.01) | 0.34 | 0.35 | 0.97 | 0.66 |
| TK1 ---NA |  | G/A or A/A | 11 | 18,33 | 7 | 21,88 | 43 | 12,61 | 60 | 19,61 | 0.71 (0.24-2.13) | 0.55 | 1.51 (0.87-2.62) | 0.14 | . | . | . |
| TK1 ---tag | rs16970907 | G/G | 50 | 83,33 | 27 | 84,38 | 307 | 90,03 | 266 | 86,93 | 1.00 (.-.) | . | 1.35 (0.86-2.11) | 0.19 | 1.00 | 1.00 | 1.00 |
| TK1 ---tag |  | G/C or C/C | 10 | 16,67 | 5 | 15,63 | 34 | 9,97 | 40 | 13,07 | 1.30 (0.30-5.63) | 0.72 | 1.77 (1.01-3.10) | 0.05 | . | . | . |
| TK1 ---tag | rs1811086 | C/C | 57 | 95,00 | 29 | 90,63 | 325 | 95,31 | 277 | 90,52 | 1.00 (.-.) | . | 1.40 (0.89-2.21) | 0.15 | 0.83 | 0.99 | 0.95 |
| TK1 ---tag |  | C/T or T/T | 3 | 5,00 | 3 | 9,38 | 16 | 4,69 | 29 | 9,48 | 1.81 (0.53-6.22) | 0.35 | 2.19 (1.19-4.06) | 0.01 | . | . | . |
| TK1 ---tag | rs2292235 | C/C | 22 | 36,67 | 9 | 28,13 | 118 | 34,60 | 97 | 31,70 | 1.00 (.-.) | . | 1.13 (0.56-2.28) | 0.73 | 0.50 | 0.97 | 0.66 |
| TK1 ---tag |  | C/A or A/A | 38 | 63,33 | 23 | 71,88 | 223 | 65,40 | 209 | 68,30 | 0.82 (0.35-1.88) | 0.64 | 1.26 (0.63-2.50) | 0.51 | . | . | . |
| TK1 ---tag | rs2854701 | A/A | 27 | 45,00 | 9 | 28,13 | 146 | 42,82 | 117 | 38,24 | 1.00 (.-.) | . | 1.99 (0.91-4.33) | 0.08 | 0.23 | 0.97 | 0.66 |
| TK1 ---tag |  | A/G or G/G | 33 | 55,00 | 23 | 71,88 | 195 | 57,18 | 189 | 61,76 | 2.05 (0.85-4.95) | 0.11 | 2.34 (1.08-5.06) | 0.03 | . | . | . |
| TK1 ---tag | rs2854702 | G/G | 46 | 76,67 | 22 | 68,75 | 284 | 83,28 | 215 | 70,26 | 1.00 (.-.) | . | 1.25 (0.76-2.08) | 0.38 | 0.39 | 0.97 | 0.66 |
| TK1 ---tag |  | G/A or A/A | 14 | 23,33 | 10 | 31,25 | 57 | 16,72 | 91 | 29,74 | 0.96 (0.39-2.34) | 0.92 | 1.80 (1.04-3.11) | 0.03 | . | . | . |
| TK1 ---tag | rs9897765 | G/G | 39 | 65,00 | 13 | 40,63 | 178 | 52,20 | 162 | 52,94 | 1.00 (.-.) | . | 1.70 (0.89-3.27) | 0.11 | 0.36 | 0.97 | 0.66 |
| TK1 ---tag |  | G/A or A/A | 21 | 35,00 | 19 | 59,38 | 163 | 47,80 | 144 | 47,06 | 1.50 (0.66-3.38) | 0.33 | 1.72 (0.88-3.34) | 0.11 | . | . | . |
| TYMP ---NA | rs131815 | G/G | 26 | 43,33 | 19 | 59,38 | 183 | 53,67 | 169 | 55,23 | 1.00 (.-.) | . | 1.23 (0.70-2.18) | 0.47 | 0.59 | 0.97 | 0.86 |
| TYMP ---NA |  | G/A or A/A | 34 | 56,67 | 13 | 40,63 | 158 | 46,33 | 137 | 44,77 | 0.89 (0.40-1.98) | 0.77 | 1.38 (0.78-2.43) | 0.27 | . | . | . |
| TYMP ---tag | rs131816 | A/A | 38 | 63,33 | 21 | 65,63 | 208 | 61,00 | 194 | 63,40 | 1.00 (.-.) | . | 1.49 (0.86-2.58) | 0.15 | 0.64 | 0.97 | 0.86 |
| TYMP ---tag |  | A/G or G/G | 22 | 36,67 | 11 | 34,38 | 133 | 39,00 | 112 | 36,60 | 0.97 (0.43-2.19) | 0.94 | 1.18 (0.67-2.07) | 0.57 | . | . | . |
| TYMP ---NA | rs131817 | C/C | 16 | 26,67 | 12 | 37,50 | 113 | 33,14 | 99 | 32,35 | 1.00 (.-.) | . | 1.49 (0.67-3.29) | 0.33 | 0.80 | 0.97 | 0.86 |
| TYMP ---NA |  | C/T or T/T | 44 | 73,33 | 20 | 62,50 | 228 | 66,86 | 207 | 67,65 | 1.25 (0.51-3.04) | 0.63 | 1.65 (0.76-3.58) | 0.20 | . | . | . |
| TYMP ---NA | rs140521 | T/T | 33 | 55,00 | 19 | 59,38 | 186 | 54,55 | 154 | 50,33 | 1.00 (.-.) | . | 1.43 (0.80-2.54) | 0.22 | 0.86 | 0.99 | 0.86 |
| TYMP ---NA |  | T/G or G/G | 27 | 45,00 | 13 | 40,63 | 155 | 45,45 | 152 | 49,67 | 0.97 (0.43-2.16) | 0.94 | 1.28 (0.72-2.28) | 0.39 | . | . | . |
| TYMP ---NA | rs140522 | G/G | 29 | 48,33 | 14 | 43,75 | 160 | 46,92 | 149 | 48,69 | 1.00 (.-.) | . | 1.64 (0.89-3.03) | 0.11 | 0.43 | 0.97 | 0.86 |
| TYMP ---NA |  | G/A or A/A | 31 | 51,67 | 18 | 56,25 | 181 | 53,08 | 157 | 51,31 | 1.13 (0.51-2.50) | 0.77 | 1.32 (0.71-2.42) | 0.38 | . | . | . |
| TYMP ---NA | rs140524 | G/G | 40 | 66,67 | 19 | 59,38 | 232 | 68,04 | 199 | 65,03 | 1.00 (.-.) | . | 1.48 (0.88-2.48) | 0.14 | 0.60 | 0.97 | 0.86 |
| TYMP ---NA |  | G/A or A/A | 20 | 33,33 | 13 | 40,63 | 109 | 31,96 | 107 | 34,97 | 1.28 (0.54-3.02) | 0.58 | 1.47 (0.85-2.55) | 0.17 | . | . | . |
| TYMS ---candidate literature | rs1001761 | C/C | 16 | 26,67 | 13 | 40,63 | 99 | 29,03 | 99 | 32,35 | 1.00 (.-.) | . | 1.37 (0.65-2.88) | 0.41 | 1.00 | 1.00 | 1.00 |
| TYMS ---candidate literature |  | C/T or T/T | 44 | 73,33 | 19 | 59,38 | 242 | 70,97 | 207 | 67,65 | 0.85 (0.36-1.99) | 0.70 | 1.16 (0.56-2.40) | 0.70 | . | . | . |
| TYMS ---candidate literature/tag | rs10502289 | T/T | 35 | 58,33 | 24 | 75,00 | 207 | 60,70 | 205 | 66,99 | 1.00 (.-.) | . | 1.31 (0.79-2.18) | 0.29 | 0.74 | 0.97 | 1.00 |
| TYMS ---candidate literature/tag |  | T/A or A/A | 25 | 41,67 | 8 | 25,00 | 134 | 39,30 | 101 | 33,01 | 0.69 (0.28-1.67) | 0.41 | 1.06 (0.62-1.80) | 0.84 | . | . | . |
| TYMS ---tag | rs15872 | C/C | 27 | 45,00 | 18 | 56,25 | 164 | 48,09 | 155 | 50,65 | 1.00 (.-.) | . | 1.08 (0.59-2.00) | 0.80 | 0.36 | 0.97 | 1.00 |
| TYMS ---tag |  | C/T or T/T | 33 | 55,00 | 14 | 43,75 | 177 | 51,91 | 151 | 49,35 | 0.60 (0.27-1.33) | 0.21 | 0.97 (0.52-1.78) | 0.91 | . | . | . |
| TYMS ---tag | rs2244500 | T/T | 16 | 26,67 | 13 | 40,63 | 99 | 29,03 | 99 | 32,35 | 1.00 (.-.) | . | 1.37 (0.65-2.88) | 0.41 | 1.00 | 1.00 | 1.00 |
| TYMS ---tag |  | T/C or C/C | 44 | 73,33 | 19 | 59,38 | 242 | 70,97 | 207 | 67,65 | 0.85 (0.36-1.99) | 0.70 | 1.16 (0.56-2.40) | 0.70 | . | . | . |
| TYMS ---tag | rs2741182 | G/G | 37 | 61,67 | 20 | 62,50 | 198 | 58,06 | 187 | 61,11 | 1.00 (.-.) | . | 1.46 (0.86-2.49) | 0.16 | 0.72 | 0.97 | 1.00 |
| TYMS ---tag |  | G/C or C/C | 23 | 38,33 | 12 | 37,50 | 143 | 41,94 | 119 | 38,89 | 1.01 (0.44-2.30) | 0.99 | 1.25 (0.72-2.18) | 0.42 | . | . | . |
| TYMS ---candidate literature | rs2847149 | G/G | 16 | 26,67 | 13 | 40,63 | 99 | 29,03 | 99 | 32,35 | 1.00 (.-.) | . | 1.37 (0.65-2.88) | 0.41 | 1.00 | 1.00 | 1.00 |
| TYMS ---candidate literature |  | G/A or A/A | 44 | 73,33 | 19 | 59,38 | 242 | 70,97 | 207 | 67,65 | 0.85 (0.36-1.99) | 0.70 | 1.16 (0.56-2.40) | 0.70 | . | . | . |
| TYMS ---candidate literature | rs2853533 | G/G | 45 | 75,00 | 24 | 75,00 | 256 | 75,07 | 228 | 74,51 | 1.00 (.-.) | . | 1.27 (0.77-2.10) | 0.36 | 0.58 | 0.97 | 1.00 |
| TYMS ---candidate literature |  | G/C or C/C | 15 | 25,00 | 8 | 25,00 | 85 | 24,93 | 78 | 25,49 | 0.76 (0.31-1.86) | 0.55 | 1.26 (0.73-2.16) | 0.41 | . | . | . |
| TYMS ---tag | rs495139 | C/C | 21 | 35,00 | 6 | 18,75 | 131 | 38,42 | 101 | 33,01 | 1.00 (.-.) | . | 2.33 (0.83-6.55) | 0.11 | 0.25 | 0.97 | 1.00 |
| TYMS ---tag |  | C/G or G/G | 39 | 65,00 | 26 | 81,25 | 210 | 61,58 | 205 | 66,99 | 2.39 (0.80-7.09) | 0.12 | 2.96 (1.07-8.19) | 0.04 | . | . | . |
| TYMS ---candidate literature | rs502396 | T/T | 16 | 26,67 | 15 | 46,88 | 97 | 28,45 | 102 | 33,33 | 1.00 (.-.) | . | 1.11 (0.57-2.20) | 0.75 | 0.47 | 0.97 | 1.00 |
| TYMS ---candidate literature |  | T/C or C/C | 44 | 73,33 | 17 | 53,13 | 244 | 71,55 | 204 | 66,67 | 0.65 (0.29-1.46) | 0.29 | 0.99 (0.51-1.92) | 0.99 | . | . | . |
| UMPH2 ---tag | rs2291028 | A/A | 18 | 30,00 | 13 | 40,63 | 149 | 43,70 | 131 | 42,81 | 1.00 (.-.) | . | 1.22 (0.60-2.46) | 0.59 | 0.67 | 0.97 | 0.94 |
| UMPH2 ---tag |  | A/G or G/G | 42 | 70,00 | 19 | 59,38 | 192 | 56,30 | 175 | 57,19 | 0.86 (0.37-1.99) | 0.72 | 1.26 (0.63-2.55) | 0.52 | . | . | . |
| UMPH2 ---NA | rs4789143 | A/A | 39 | 65,00 | 29 | 90,63 | 255 | 74,78 | 249 | 81,37 | 1.00 (.-.) | . | 1.39 (0.88-2.22) | 0.16 | 0.94 | 1.00 | 0.94 |
| UMPH2 ---NA |  | A/G or G/G | 21 | 35,00 | 3 | 9,38 | 86 | 25,22 | 57 | 18,63 | 0.69 (0.20-2.38) | 0.56 | 1.02 (0.60-1.73) | 0.95 | . | . | . |
| UMPH2 ---NA | rs750844 | G/G | 23 | 38,33 | 16 | 50,00 | 177 | 51,91 | 156 | 50,98 | 1.00 (.-.) | . | 1.16 (0.63-2.16) | 0.63 | 0.51 | 0.97 | 0.94 |
| UMPH2 ---NA |  | G/A or A/A | 37 | 61,67 | 16 | 50,00 | 164 | 48,09 | 150 | 49,02 | 0.71 (0.32-1.59) | 0.40 | 1.09 (0.58-2.04) | 0.78 | . | . | . |
| UMPK ---tag | rs11582877 | C/C | 45 | 75,00 | 24 | 75,00 | 254 | 74,49 | 220 | 71,90 | 1.00 (.-.) | . | 1.36 (0.82-2.24) | 0.23 | 0.93 | 1.00 | 0.93 |
| UMPK ---tag |  | C/T or T/T | 15 | 25,00 | 8 | 25,00 | 87 | 25,51 | 86 | 28,10 | 0.91 (0.37-2.22) | 0.84 | 1.29 (0.75-2.21) | 0.36 | . | . | . |
| UMPK ---tag | rs2622903 | A/A | 30 | 50,00 | 16 | 50,00 | 170 | 49,85 | 146 | 47,71 | 1.00 (.-.) | . | 1.29 (0.70-2.39) | 0.42 | 0.79 | 0.97 | 0.93 |
| UMPK ---tag |  | A/G or G/G | 30 | 50,00 | 16 | 50,00 | 171 | 50,15 | 160 | 52,29 | 0.85 (0.38-1.91) | 0.69 | 1.23 (0.66-2.28) | 0.52 | . | . | . |
| UMPK ---tag | rs2820989 | C/C | 19 | 31,67 | 11 | 34,38 | 108 | 31,67 | 92 | 30,07 | 1.00 (.-.) | . | 1.19 (0.56-2.52) | 0.65 | 0.66 | 0.97 | 0.93 |
| UMPK ---tag |  | C/G or G/G | 41 | 68,33 | 21 | 65,63 | 233 | 68,33 | 214 | 69,93 | 0.83 (0.35-1.96) | 0.66 | 1.21 (0.58-2.51) | 0.62 | . | . | . |
| UMPK ---tag | rs6660321 | A/A | 45 | 75,00 | 25 | 78,13 | 262 | 76,83 | 227 | 74,18 | 1.00 (.-.) | . | 1.29 (0.79-2.10) | 0.30 | 0.64 | 0.97 | 0.93 |
| UMPK ---tag |  | A/C or C/C | 15 | 25,00 | 7 | 21,88 | 79 | 23,17 | 79 | 25,82 | 0.75 (0.29-1.91) | 0.55 | 1.22 (0.72-2.08) | 0.46 | . | . | . |
| UMPK ---tag | rs6690084 | T/T | 54 | 90,00 | 31 | 96,88 | 300 | 87,98 | 262 | 85,62 | 1.00 (.-.) | . | 1.23 (0.80-1.90) | 0.34 | 0.03 | 0.85 | 0.12 |
| UMPK ---tag |  | T/C or C/C | 6 | 10,00 | 1 | 3,13 | 41 | 12,02 | 44 | 14,38 | 0.00 (0.00-3E226) | 0.96 | 1.35 (0.78-2.31) | 0.28 | . | . | . |
| UMPS ---NA | rs1162 | A/A | 32 | 53,33 | 15 | 46,88 | 175 | 51,32 | 132 | 43,14 | 1.00 (.-.) | . | 1.08 (0.58-2.02) | 0.81 | 0.29 | 0.97 | 0.58 |
| UMPS ---NA |  | A/G or G/G | 28 | 46,67 | 17 | 53,13 | 166 | 48,68 | 174 | 56,86 | 0.88 (0.39-1.96) | 0.75 | 1.51 (0.81-2.79) | 0.19 | . | . | . |
| UMPS ---tag | rs13146 | C/C | 44 | 73,33 | 21 | 65,63 | 243 | 71,26 | 209 | 68,30 | 1.00 (.-.) | . | 1.17 (0.70-1.97) | 0.55 | 0.28 | 0.97 | 0.58 |
| UMPS ---tag |  | C/T or T/T | 16 | 26,67 | 11 | 34,38 | 98 | 28,74 | 97 | 31,70 | 0.75 (0.32-1.77) | 0.51 | 1.43 (0.83-2.47) | 0.19 | . | . | . |
| UMPS ---tag | rs16835902 | C/C | 20 | 33,33 | 15 | 46,88 | 112 | 32,84 | 97 | 31,70 | 1.00 (.-.) | . | 1.17 (0.62-2.21) | 0.62 | 0.50 | 0.97 | 0.76 |
| UMPS ---tag |  | C/G or G/G | 40 | 66,67 | 17 | 53,13 | 229 | 67,16 | 209 | 68,30 | 0.73 (0.33-1.61) | 0.43 | 1.14 (0.62-2.08) | 0.68 | . | . | . |
| UMPS ---tag | rs17282057 | T/T | 48 | 80,00 | 26 | 81,25 | 272 | 79,77 | 218 | 71,24 | 1.00 (.-.) | . | 1.36 (0.84-2.19) | 0.21 | 0.98 | 1.00 | 0.98 |
| UMPS ---tag |  | T/C or C/C | 12 | 20,00 | 6 | 18,75 | 69 | 20,23 | 88 | 28,76 | 1.17 (0.43-3.17) | 0.77 | 1.56 (0.93-2.60) | 0.09 | . | . | . |
| UMPS ---tag | rs606552 | A/A | 28 | 46,67 | 19 | 59,38 | 167 | 48,97 | 176 | 57,52 | 1.00 (.-.) | . | 1.28 (0.73-2.24) | 0.39 | 0.70 | 0.97 | 0.83 |
| UMPS ---tag |  | A/G or G/G | 32 | 53,33 | 13 | 40,63 | 174 | 51,03 | 130 | 42,48 | 0.71 (0.32-1.60) | 0.41 | 1.08 (0.61-1.89) | 0.79 | . | . | . |
| UMPS ---tag | rs694897 | C/C | 27 | 45,00 | 14 | 43,75 | 136 | 39,88 | 114 | 37,25 | 1.00 (.-.) | . | 1.89 (1.00-3.58) | 0.05 | 0.16 | 0.97 | 0.58 |
| UMPS ---tag |  | C/G or G/G | 33 | 55,00 | 18 | 56,25 | 205 | 60,12 | 192 | 62,75 | 1.62 (0.72-3.63) | 0.24 | 1.65 (0.88-3.09) | 0.12 | . | . | . |
| UNG ---NA | rs1059262 | T/T | 42 | 70,00 | 20 | 62,50 | 231 | 67,74 | 211 | 68,95 | 1.00 (.-.) | . | 1.34 (0.79-2.27) | 0.28 | 0.89 | 0.99 | 0.91 |
| UNG ---NA |  | T/G or G/G | 18 | 30,00 | 12 | 37,50 | 110 | 32,26 | 95 | 31,05 | 0.92 (0.40-2.11) | 0.85 | 1.32 (0.75-2.30) | 0.33 | . | . | . |
| UNG ---tag | rs2160603 | T/T | 40 | 66,67 | 24 | 75,00 | 232 | 68,04 | 206 | 67,32 | 1.00 (.-.) | . | 1.14 (0.69-1.88) | 0.62 | 0.17 | 0.97 | 0.52 |
| UNG ---tag |  | T/C or C/C | 20 | 33,33 | 8 | 25,00 | 109 | 31,96 | 100 | 32,68 | 0.67 (0.26-1.73) | 0.41 | 1.48 (0.86-2.53) | 0.15 | . | . | . |
| UNG ---tag | rs246079 | A/A | 16 | 26,67 | 8 | 25,00 | 102 | 29,91 | 100 | 32,68 | 1.00 (.-.) | . | 1.42 (0.63-3.20) | 0.39 | 0.91 | 1.00 | 0.91 |
| UNG ---tag |  | A/G or G/G | 44 | 73,33 | 24 | 75,00 | 239 | 70,09 | 206 | 67,32 | 1.03 (0.42-2.52) | 0.95 | 1.39 (0.63-3.07) | 0.42 | . | . | . |
| UNG ---NA | rs246085 | T/T | 51 | 85,00 | 24 | 75,00 | 297 | 87,10 | 276 | 90,20 | 1.00 (.-.) | . | 1.48 (0.90-2.44) | 0.12 | 0.51 | 0.97 | 0.88 |
| UNG ---NA |  | T/C or C/C | 9 | 15,00 | 8 | 25,00 | 44 | 12,90 | 30 | 9,80 | 1.30 (0.53-3.18) | 0.57 | 1.37 (0.73-2.57) | 0.32 | . | . | . |
| UNG ---NA | rs2569987 | A/A | 48 | 80,00 | 23 | 71,88 | 226 | 66,28 | 222 | 72,55 | 1.00 (.-.) | . | 1.93 (1.14-3.27) | 0.01 | 0.00 | 0.85 | 0.02 |
| UNG ---NA |  | A/G or G/G | 12 | 20,00 | 9 | 28,13 | 115 | 33,72 | 84 | 27,45 | 3.61 (1.57-8.31) | 0.00 | 1.63 (0.93-2.87) | 0.09 | . | . | . |
| UNG ---tag | rs3219243 | T/T | 36 | 60,00 | 20 | 62,50 | 224 | 65,69 | 199 | 65,03 | 1.00 (.-.) | . | 1.49 (0.89-2.51) | 0.13 | 0.59 | 0.97 | 0.88 |
| UNG ---tag |  | T/C or C/C | 24 | 40,00 | 12 | 37,50 | 117 | 34,31 | 107 | 34,97 | 1.10 (0.46-2.61) | 0.83 | 1.27 (0.74-2.20) | 0.39 | . | . | . |

|  | | | **adchem_5fu = 0** | | | | **adchem_5fu = 1** | | | | **one reference** | | | | | | |
| --- | --- | --- | --- | --- | --- | --- | --- | --- | --- | --- | --- | --- | --- | --- | --- | --- | --- |
|  | | | **Ctrl** | | **Cases** | | **Ctrl** | | **Cases** | | **adchem_5fu = 0** | | **adchem_5fu = 1** | |  | | |
| **Gene** | **SNP** | **Genotype** | **N** | **%** | **N** | **%** | **N** | **%** | **N** | **%** | **HR (95%-CI)** | **p** | **HR (95%-CI)** | **p** | **LR_pTrend** | **FDR_pTrend** | **FDR(byGene)_pTrend** |
| AARS ---- tag | rs2070203 | T/T | 17 | 28,33 | 11 | 34,38 | 104 | 30,50 | 84 | 27,45 | 1.00 (.-.) | . | 0.92 (0.44-1.93) | 0.84 | 0.12 | 0.99 | 0.13 |
| AARS ---- tag |  | T/C | 27 | 45,00 | 14 | 43,75 | 176 | 51,61 | 152 | 49,67 | 0.80 (0.33-1.98) | 0.64 | 1.06 (0.52-2.16) | 0.87 | . | . | . |
| AARS ---- tag |  | C/C | 16 | 26,67 | 7 | 21,88 | 61 | 17,89 | 70 | 22,88 | 0.51 (0.17-1.59) | 0.25 | 1.21 (0.57-2.54) | 0.62 | . | . | . |
| AARS ---- tag | rs34087264 | G/G | 21 | 35,00 | 7 | 21,88 | 86 | 25,22 | 95 | 31,05 | 1.00 (.-.) | . | 2.80 (1.12-7.01) | 0.03 | 0.13 | 0.99 | 0.13 |
| AARS ---- tag |  | G/A | 23 | 38,33 | 17 | 53,13 | 174 | 51,03 | 145 | 47,39 | 2.13 (0.75-6.07) | 0.16 | 2.16 (0.87-5.38) | 0.10 | . | . | . |
| AARS ---- tag |  | A/A | 16 | 26,67 | 8 | 25,00 | 81 | 23,75 | 66 | 21,57 | 1.75 (0.52-5.92) | 0.37 | 1.95 (0.77-4.97) | 0.16 | . | . | . |
| ABCC4 ---- tag | rs10508023 | G/G | 49 | 81,67 | 25 | 78,13 | 267 | 78,30 | 250 | 81,70 | 1.00 (.-.) | . | 1.57 (0.97-2.52) | 0.06 | 0.34 | 0.99 | 1.00 |
| ABCC4 ---- tag |  | G/C | 10 | 16,67 | 7 | 21,88 | 71 | 20,82 | 49 | 16,01 | 1.89 (0.69-5.19) | 0.22 | 1.14 (0.66-1.97) | 0.65 | . | . | . |
| ABCC4 ---- tag |  | C/C | 1 | 1,67 | 0 | 0,00 | 3 | 0,88 | 7 | 2,29 |  | 0.97 | 1.98 (0.67-5.91) | 0.22 | . | . | . |
| ABCC4 ---- tag | rs1059751 | T/T | 20 | 33,33 | 8 | 25,00 | 81 | 23,75 | 78 | 25,49 | 1.00 (.-.) | . | 1.53 (0.69-3.39) | 0.30 | 0.66 | 0.99 | 1.00 |
| ABCC4 ---- tag |  | T/C | 33 | 55,00 | 20 | 62,50 | 191 | 56,01 | 161 | 52,61 | 1.16 (0.47-2.88) | 0.75 | 1.55 (0.72-3.37) | 0.27 | . | . | . |
| ABCC4 ---- tag |  | C/C | 7 | 11,67 | 4 | 12,50 | 69 | 20,23 | 67 | 21,90 | 1.48 (0.30-7.25) | 0.63 | 1.56 (0.70-3.48) | 0.27 | . | . | . |
| ABCC4 ---- tag | rs11568643 | A/A | 50 | 83,33 | 26 | 81,25 | 286 | 83,87 | 256 | 83,66 | 1.00 (.-.) | . | 1.46 (0.92-2.32) | 0.11 | 0.47 | 0.99 | 1.00 |
| ABCC4 ---- tag |  | A/G | 10 | 16,67 | 5 | 15,63 | 55 | 16,13 | 48 | 15,69 | 1.41 (0.42-4.82) | 0.58 | 1.20 (0.69-2.11) | 0.52 | . | . | . |
| ABCC4 ---- tag |  | G/G | 0 | 0,00 | 1 | 3,13 | 0 | 0,00 | 2 | 0,65 | 1.16 (0.15-9.03) | 0.89 | 2.88 (0.37-22.72) | 0.32 | . | . | . |
| ABCC4 ---- NA | rs11568658 | G/G | 56 | 93,33 | 31 | 96,88 | 321 | 94,13 | 293 | 95,75 | 1.00 (.-.) | . | 1.37 (0.89-2.13) | 0.16 | 0.96 | 1.00 | 1.00 |
| ABCC4 ---- NA |  | G/T | 4 | 6,67 | 1 | 3,13 | 20 | 5,87 | 13 | 4,25 | 1.00 (.-.) | . | 1.37 (0.89-2.13) | 0.16 | . | . | . |
| ABCC4 ---- NA |  | T/T | 0 | 0,00 | 0 | 0,00 | 0 | 0,00 | 0 | 0,00 | 1.15 (0.15-8.61) | 0.89 | 1.51 (0.73-3.13) | 0.27 | . | . | . |
| ABCC4 ---- tag | rs12864049 | T/T | 42 | 70,00 | 22 | 68,75 | 266 | 78,01 | 226 | 73,86 | 1.00 (.-.) | . | 1.29 (0.76-2.18) | 0.35 | 0.86 | 1.00 | 1.00 |
| ABCC4 ---- tag |  | T/C | 16 | 26,67 | 9 | 28,13 | 69 | 20,23 | 75 | 24,51 | 0.95 (0.40-2.24) | 0.91 | 1.68 (0.96-2.94) | 0.07 | . | . | . |
| ABCC4 ---- tag |  | C/C | 2 | 3,33 | 1 | 3,13 | 6 | 1,76 | 5 | 1,63 | 5.38 (0.69-42.18) | 0.11 | 1.88 (0.61-5.80) | 0.27 | . | . | . |
| ABCC4 ---- tag | rs1628382 | G/G | 38 | 63,33 | 23 | 71,88 | 209 | 61,29 | 189 | 61,76 | 1.00 (.-.) | . | 1.40 (0.84-2.33) | 0.20 | 0.77 | 0.99 | 1.00 |
| ABCC4 ---- tag |  | G/A | 20 | 33,33 | 8 | 25,00 | 116 | 34,02 | 104 | 33,99 | 0.99 (0.39-2.56) | 0.99 | 1.41 (0.83-2.41) | 0.21 | . | . | . |
| ABCC4 ---- tag |  | A/A | 2 | 3,33 | 1 | 3,13 | 16 | 4,69 | 13 | 4,25 | 2.25 (0.29-17.44) | 0.44 | 1.53 (0.70-3.34) | 0.28 | . | . | . |
| ABCC4 ---- tag | rs1678354 | C/C | 20 | 33,33 | 12 | 37,50 | 153 | 44,87 | 126 | 41,18 | 1.00 (.-.) | . | 1.22 (0.60-2.48) | 0.58 | 0.77 | 0.99 | 1.00 |
| ABCC4 ---- tag |  | C/G | 33 | 55,00 | 18 | 56,25 | 144 | 42,23 | 148 | 48,37 | 0.98 (0.42-2.29) | 0.96 | 1.72 (0.85-3.45) | 0.13 | . | . | . |
| ABCC4 ---- tag |  | G/G | 7 | 11,67 | 2 | 6,25 | 44 | 12,90 | 32 | 10,46 | 2.49 (0.52-11.88) | 0.25 | 1.20 (0.55-2.62) | 0.64 | . | . | . |
| ABCC4 ---- tag | rs1678383 | T/T | 48 | 80,00 | 27 | 84,38 | 282 | 82,70 | 249 | 81,37 | 1.00 (.-.) | . | 1.10 (0.69-1.75) | 0.69 | 0.04 | 0.99 | 0.76 |
| ABCC4 ---- tag |  | T/G | 11 | 18,33 | 5 | 15,63 | 54 | 15,84 | 54 | 17,65 | 0.35 (0.10-1.20) | 0.10 | 1.16 (0.68-1.99) | 0.58 | . | . | . |
| ABCC4 ---- tag |  | G/G | 1 | 1,67 | 0 | 0,00 | 5 | 1,47 | 3 | 0,98 | 1.96 (0.62-6.24) | 0.26 | 2.15 (0.63-7.31) | 0.22 | . | . | . |
| ABCC4 ---- tag | rs1678395 | G/G | 51 | 85,00 | 26 | 81,25 | 285 | 83,58 | 262 | 85,62 | 1.00 (.-.) | . | 1.51 (0.95-2.38) | 0.08 | 0.61 | 0.99 | 1.00 |
| ABCC4 ---- tag |  | G/A | 9 | 15,00 | 6 | 18,75 | 53 | 15,54 | 42 | 13,73 | 0.87 (0.26-2.98) | 0.83 | 0.93 (0.54-1.62) | 0.80 | . | . | . |
| ABCC4 ---- tag |  | A/A | 0 | 0,00 | 0 | 0,00 | 3 | 0,88 | 2 | 0,65 | 0.38 (0.05-2.84) | 0.34 | 0.57 (0.07-4.45) | 0.59 | . | . | . |
| ABCC4 ---- tag | rs1678405 | T/T | 24 | 40,00 | 21 | 65,63 | 156 | 45,75 | 132 | 43,14 | 1.00 (.-.) | . | 1.10 (0.65-1.88) | 0.72 | 0.41 | 0.99 | 1.00 |
| ABCC4 ---- tag |  | T/C | 32 | 53,33 | 10 | 31,25 | 157 | 46,04 | 140 | 45,75 | 0.66 (0.27-1.62) | 0.36 | 1.31 (0.77-2.24) | 0.33 | . | . | . |
| ABCC4 ---- tag |  | C/C | 4 | 6,67 | 1 | 3,13 | 28 | 8,21 | 34 | 11,11 | 4.69 (0.60-36.97) | 0.14 | 1.71 (0.92-3.16) | 0.09 | . | . | . |
| ABCC4 ---- tag | rs17189540 | A/A | 53 | 88,33 | 26 | 81,25 | 303 | 88,86 | 260 | 84,97 | 1.00 (.-.) | . | 1.42 (0.88-2.27) | 0.15 | 0.76 | 0.99 | 1.00 |
| ABCC4 ---- tag |  | A/G | 7 | 11,67 | 5 | 15,63 | 38 | 11,14 | 46 | 15,03 | 1.00 (.-.) | . | 1.42 (0.88-2.27) | 0.15 | . | . | . |
| ABCC4 ---- tag |  | G/G | 0 | 0,00 | 1 | 3,13 | 0 | 0,00 | 0 | 0,00 | 1.28 (0.46-3.52) | 0.63 | 1.52 (0.86-2.70) | 0.15 | . | . | . |
| ABCC4 ---- tag | rs17235152 | T/T | 41 | 68,33 | 24 | 75,00 | 247 | 72,43 | 226 | 73,86 | 1.00 (.-.) | . | 1.34 (0.80-2.26) | 0.27 | 0.81 | 0.99 | 1.00 |
| ABCC4 ---- tag |  | T/C | 17 | 28,33 | 8 | 25,00 | 86 | 25,22 | 75 | 24,51 | 1.00 (0.43-2.36) | 1.00 | 1.42 (0.80-2.51) | 0.24 | . | . | . |
| ABCC4 ---- tag |  | C/C | 2 | 3,33 | 0 | 0,00 | 8 | 2,35 | 5 | 1,63 | 0.00 (0.00-6E223) | 0.97 | 1.40 (0.50-3.95) | 0.52 | . | . | . |
| ABCC4 ---- tag | rs17268122 | G/G | 28 | 46,67 | 18 | 56,25 | 214 | 62,76 | 206 | 67,32 | 1.00 (.-.) | . | 1.13 (0.63-2.02) | 0.67 | 0.63 | 0.99 | 1.00 |
| ABCC4 ---- tag |  | G/T | 28 | 46,67 | 12 | 37,50 | 111 | 32,55 | 84 | 27,45 | 0.60 (0.25-1.44) | 0.25 | 0.99 (0.53-1.84) | 0.97 | . | . | . |
| ABCC4 ---- tag |  | T/T | 4 | 6,67 | 2 | 6,25 | 16 | 4,69 | 16 | 5,23 | 0.74 (0.17-3.31) | 0.69 | 0.84 (0.36-1.92) | 0.67 | . | . | . |
| ABCC4 ---- tag | rs17268170 | C/C | 47 | 78,33 | 24 | 75,00 | 284 | 83,28 | 246 | 80,39 | 1.00 (.-.) | . | 1.45 (0.88-2.37) | 0.15 | 0.22 | 0.99 | 1.00 |
| ABCC4 ---- tag |  | C/T | 13 | 21,67 | 4 | 12,50 | 56 | 16,42 | 58 | 18,95 | 0.82 (0.27-2.48) | 0.73 | 1.39 (0.80-2.43) | 0.25 | . | . | . |
| ABCC4 ---- tag |  | T/T | 0 | 0,00 | 4 | 12,50 | 1 | 0,29 | 2 | 0,65 | 4.59 (1.22-17.23) | 0.02 | 1.31 (0.17-10.16) | 0.80 | . | . | . |
| ABCC4 ---- tag | rs1729764 | A/A | 44 | 73,33 | 27 | 84,38 | 259 | 75,95 | 246 | 80,39 | 1.00 (.-.) | . | 1.33 (0.85-2.09) | 0.21 | 0.50 | 0.99 | 1.00 |
| ABCC4 ---- tag |  | A/G | 15 | 25,00 | 3 | 9,38 | 78 | 22,87 | 55 | 17,97 | 0.73 (0.17-3.15) | 0.68 | 1.18 (0.69-2.00) | 0.54 | . | . | . |
| ABCC4 ---- tag |  | G/G | 1 | 1,67 | 2 | 6,25 | 4 | 1,17 | 5 | 1,63 | 0.00 (0.00-I) | 0.98 | 3.40 (1.14-10.14) | 0.03 | . | . | . |
| ABCC4 ---- tag | rs1729767 | T/T | 29 | 48,33 | 14 | 43,75 | 183 | 53,67 | 156 | 50,98 | 1.00 (.-.) | . | 1.54 (0.82-2.88) | 0.18 | 0.95 | 1.00 | 1.00 |
| ABCC4 ---- tag |  | T/C | 24 | 40,00 | 18 | 56,25 | 140 | 41,06 | 132 | 43,14 | 1.64 (0.73-3.67) | 0.23 | 1.80 (0.96-3.39) | 0.07 | . | . | . |
| ABCC4 ---- tag |  | C/C | 7 | 11,67 | 0 | 0,00 | 18 | 5,28 | 18 | 5,88 | 0.00 (0.00-I) | 0.98 | 1.61 (0.71-3.62) | 0.25 | . | . | . |
| ABCC4 ---- tag | rs17300935 | C/C | 40 | 66,67 | 21 | 65,63 | 252 | 73,90 | 233 | 76,14 | 1.00 (.-.) | . | 1.34 (0.79-2.29) | 0.28 | 0.87 | 1.00 | 1.00 |
| ABCC4 ---- tag |  | C/G | 18 | 30,00 | 11 | 34,38 | 82 | 24,05 | 70 | 22,88 | 1.02 (0.44-2.35) | 0.97 | 1.47 (0.83-2.63) | 0.19 | . | . | . |
| ABCC4 ---- tag |  | G/G | 2 | 3,33 | 0 | 0,00 | 7 | 2,05 | 3 | 0,98 | 0.00 (0.00-7E223) | 0.97 | 0.93 (0.26-3.32) | 0.92 | . | . | . |
| ABCC4 ---- tag | rs1750190 | G/G | 18 | 30,00 | 8 | 25,00 | 91 | 26,69 | 72 | 23,53 | 1.00 (.-.) | . | 1.50 (0.63-3.55) | 0.36 | 0.67 | 0.99 | 1.00 |
| ABCC4 ---- tag |  | G/A | 29 | 48,33 | 17 | 53,13 | 166 | 48,68 | 158 | 51,63 | 1.56 (0.57-4.25) | 0.39 | 2.08 (0.90-4.79) | 0.09 | . | . | . |
| ABCC4 ---- tag |  | A/A | 13 | 21,67 | 7 | 21,88 | 84 | 24,63 | 76 | 24,84 | 1.53 (0.49-4.84) | 0.47 | 1.79 (0.76-4.21) | 0.18 | . | . | . |
| ABCC4 ---- tag | rs1750996 | A/A | 33 | 55,00 | 21 | 65,63 | 224 | 65,69 | 201 | 65,69 | 1.00 (.-.) | . | 1.31 (0.78-2.19) | 0.30 | 0.81 | 0.99 | 1.00 |
| ABCC4 ---- tag |  | A/G | 25 | 41,67 | 8 | 25,00 | 111 | 32,55 | 94 | 30,72 | 0.89 (0.36-2.18) | 0.80 | 1.42 (0.82-2.45) | 0.21 | . | . | . |
| ABCC4 ---- tag |  | G/G | 2 | 3,33 | 3 | 9,38 | 6 | 1,76 | 11 | 3,59 | 2.51 (0.33-19.27) | 0.38 | 2.55 (1.12-5.77) | 0.03 | . | . | . |
| ABCC4 ---- tag | rs1751025 | C/C | 27 | 45,00 | 18 | 56,25 | 167 | 48,97 | 134 | 43,79 | 1.00 (.-.) | . | 1.35 (0.76-2.39) | 0.30 | 0.41 | 0.99 | 1.00 |
| ABCC4 ---- tag |  | C/G | 28 | 46,67 | 8 | 25,00 | 146 | 42,82 | 134 | 43,79 | 0.73 (0.28-1.94) | 0.53 | 1.43 (0.81-2.55) | 0.22 | . | . | . |
| ABCC4 ---- tag |  | G/G | 5 | 8,33 | 6 | 18,75 | 28 | 8,21 | 38 | 12,42 | 3.27 (1.12-9.56) | 0.03 | 1.85 (0.97-3.53) | 0.06 | . | . | . |
| ABCC4 ---- tag | rs1751051 | T/T | 26 | 43,33 | 14 | 43,75 | 136 | 39,88 | 136 | 44,44 | 1.00 (.-.) | . | 1.40 (0.77-2.54) | 0.27 | 1.00 | 1.00 | 1.00 |
| ABCC4 ---- tag |  | T/A | 24 | 40,00 | 14 | 43,75 | 159 | 46,63 | 136 | 44,44 | 0.77 (0.32-1.85) | 0.55 | 1.05 (0.57-1.91) | 0.88 | . | . | . |
| ABCC4 ---- tag |  | A/A | 10 | 16,67 | 4 | 12,50 | 46 | 13,49 | 34 | 11,11 | 0.76 (0.21-2.79) | 0.68 | 1.10 (0.55-2.19) | 0.78 | . | . | . |
| ABCC4 ---- tag | rs1764416 | G/G | 53 | 88,33 | 31 | 96,88 | 292 | 85,63 | 265 | 86,60 | 1.00 (.-.) | . | 1.38 (0.89-2.15) | 0.15 | 0.93 | 1.00 | 1.00 |
| ABCC4 ---- tag |  | G/A | 7 | 11,67 | 1 | 3,13 | 49 | 14,37 | 40 | 13,07 | 1.00 (.-.) | . | 1.38 (0.89-2.15) | 0.15 | . | . | . |
| ABCC4 ---- tag |  | A/A | 0 | 0,00 | 0 | 0,00 | 0 | 0,00 | 1 | 0,33 | 0.79 (0.10-5.97) | 0.82 | 1.20 (0.67-2.13) | 0.55 | . | . | . |
| ABCC4 ---- tag | rs2274401 | T/T | 36 | 60,00 | 22 | 68,75 | 198 | 58,06 | 203 | 66,34 | 1.00 (.-.) | . | 1.33 (0.82-2.16) | 0.24 | 0.47 | 0.99 | 1.00 |
| ABCC4 ---- tag |  | T/C | 22 | 36,67 | 10 | 31,25 | 124 | 36,36 | 91 | 29,74 | 1.00 (0.37-2.68) | 0.99 | 1.12 (0.67-1.88) | 0.66 | . | . | . |
| ABCC4 ---- tag |  | C/C | 2 | 3,33 | 0 | 0,00 | 19 | 5,57 | 12 | 3,92 | 0.00 (0.00-I) | 0.97 | 1.07 (0.47-2.42) | 0.87 | . | . | . |
| ABCC4 ---- tag | rs2892716 | C/C | 28 | 46,67 | 15 | 46,88 | 117 | 34,31 | 114 | 37,25 | 1.00 (.-.) | . | 2.00 (1.07-3.73) | 0.03 | 0.15 | 0.99 | 1.00 |
| ABCC4 ---- tag |  | C/T | 29 | 48,33 | 16 | 50,00 | 164 | 48,09 | 152 | 49,67 | 2.56 (1.13-5.80) | 0.02 | 2.01 (1.08-3.75) | 0.03 | . | . | . |
| ABCC4 ---- tag |  | T/T | 3 | 5,00 | 1 | 3,13 | 60 | 17,60 | 40 | 13,07 | 0.00 (0.00-2E234) | 0.97 | 1.87 (0.95-3.69) | 0.07 | . | . | . |
| ABCC4 ---- tag | rs3782964 | C/C | 48 | 80,00 | 19 | 59,38 | 215 | 63,05 | 218 | 71,24 | 1.00 (.-.) | . | 1.75 (1.02-3.00) | 0.04 | 0.04 | 0.99 | 0.76 |
| ABCC4 ---- tag |  | C/T | 12 | 20,00 | 10 | 31,25 | 115 | 33,72 | 79 | 25,82 | 1.57 (0.68-3.62) | 0.29 | 1.36 (0.76-2.43) | 0.30 | . | . | . |
| ABCC4 ---- tag |  | T/T | 0 | 0,00 | 3 | 9,38 | 11 | 3,23 | 9 | 2,94 | 14.12 (1.63-122.0) | 0.02 | 1.54 (0.51-4.71) | 0.45 | . | . | . |
| ABCC4 ---- tag | rs3818494 | C/C | 30 | 50,00 | 21 | 65,63 | 158 | 46,33 | 123 | 40,20 | 1.00 (.-.) | . | 1.02 (0.59-1.75) | 0.94 | 0.29 | 0.99 | 1.00 |
| ABCC4 ---- tag |  | C/G | 24 | 40,00 | 10 | 31,25 | 146 | 42,82 | 140 | 45,75 | 0.84 (0.34-2.05) | 0.70 | 1.57 (0.92-2.70) | 0.10 | . | . | . |
| ABCC4 ---- tag |  | G/G | 6 | 10,00 | 1 | 3,13 | 37 | 10,85 | 43 | 14,05 | 1.09 (0.14-8.46) | 0.94 | 1.75 (0.96-3.20) | 0.07 | . | . | . |
| ABCC4 ---- tag | rs3864997 | G/G | 22 | 36,67 | 10 | 31,25 | 79 | 23,17 | 76 | 24,84 | 1.00 (.-.) | . | 2.04 (0.97-4.33) | 0.06 | 0.07 | 0.99 | 0.76 |
| ABCC4 ---- tag |  | G/T | 30 | 50,00 | 17 | 53,13 | 182 | 53,37 | 161 | 52,61 | 1.39 (0.57-3.40) | 0.47 | 1.86 (0.90-3.84) | 0.09 | . | . | . |
| ABCC4 ---- tag |  | T/T | 8 | 13,33 | 5 | 15,63 | 80 | 23,46 | 69 | 22,55 | 2.91 (0.86-9.84) | 0.09 | 1.62 (0.76-3.45) | 0.21 | . | . | . |
| ABCC4 ---- tag | rs4148421 | G/G | 15 | 25,00 | 4 | 12,50 | 105 | 30,79 | 91 | 29,74 | 1.00 (.-.) | . | 6.21 (0.85-45.10) | 0.07 | 0.62 | 0.99 | 1.00 |
| ABCC4 ---- tag |  | G/A | 33 | 55,00 | 21 | 65,63 | 170 | 49,85 | 161 | 52,61 | 5.83 (0.77-43.92) | 0.09 | 5.65 (0.78-40.97) | 0.09 | . | . | . |
| ABCC4 ---- tag |  | A/A | 12 | 20,00 | 7 | 21,88 | 66 | 19,35 | 54 | 17,65 | 2.93 (0.33-25.59) | 0.33 | 5.53 (0.75-40.60) | 0.09 | . | . | . |
| ABCC4 ---- tag | rs4148446 | G/G | 26 | 43,33 | 12 | 37,50 | 106 | 31,09 | 102 | 33,33 | 1.00 (.-.) | . | 1.92 (0.95-3.90) | 0.07 | 0.23 | 0.99 | 1.00 |
| ABCC4 ---- tag |  | G/A | 29 | 48,33 | 17 | 53,13 | 163 | 47,80 | 153 | 50,00 | 1.72 (0.74-4.00) | 0.21 | 1.91 (0.95-3.86) | 0.07 | . | . | . |
| ABCC4 ---- tag |  | A/A | 5 | 8,33 | 3 | 9,38 | 72 | 21,11 | 51 | 16,67 | 1.44 (0.18-11.55) | 0.73 | 1.69 (0.81-3.54) | 0.17 | . | . | . |
| ABCC4 ---- tag | rs4148455 | G/G | 43 | 71,67 | 27 | 84,38 | 261 | 76,54 | 226 | 73,86 | 1.00 (.-.) | . | 1.19 (0.73-1.92) | 0.48 | 0.51 | 0.99 | 1.00 |
| ABCC4 ---- tag |  | G/A | 16 | 26,67 | 4 | 12,50 | 72 | 21,11 | 77 | 25,16 | 0.71 (0.24-2.12) | 0.54 | 1.95 (1.15-3.32) | 0.01 | . | . | . |
| ABCC4 ---- tag |  | A/A | 1 | 1,67 | 1 | 3,13 | 8 | 2,35 | 3 | 0,98 | 3.11 (0.39-24.79) | 0.28 | 0.83 (0.24-2.88) | 0.77 | . | . | . |
| ABCC4 ---- tag | rs4148540 | C/C | 52 | 86,67 | 28 | 87,50 | 299 | 87,68 | 262 | 85,62 | 1.00 (.-.) | . | 1.43 (0.91-2.26) | 0.12 | 0.77 | 0.99 | 1.00 |
| ABCC4 ---- tag |  | C/T | 8 | 13,33 | 4 | 12,50 | 40 | 11,73 | 41 | 13,40 | 1.04 (0.30-3.56) | 0.95 | 1.18 (0.68-2.04) | 0.56 | . | . | . |
| ABCC4 ---- tag |  | T/T | 0 | 0,00 | 0 | 0,00 | 2 | 0,59 | 3 | 0,98 | 0.93 (0.28-3.03) | 0.90 | 1.33 (0.38-4.68) | 0.66 | . | . | . |
| ABCC4 ---- tag | rs4148542 | G/G | 11 | 18,33 | 4 | 12,50 | 94 | 27,57 | 79 | 25,82 | 1.00 (.-.) | . | 1.45 (0.35-6.01) | 0.61 | 0.98 | 1.00 | 1.00 |
| ABCC4 ---- tag |  | G/A | 30 | 50,00 | 21 | 65,63 | 179 | 52,49 | 160 | 52,29 | 1.19 (0.27-5.24) | 0.82 | 1.70 (0.41-6.98) | 0.46 | . | . | . |
| ABCC4 ---- tag |  | A/A | 19 | 31,67 | 7 | 21,88 | 68 | 19,94 | 67 | 21,90 | 1.20 (0.24-6.00) | 0.83 | 1.60 (0.38-6.67) | 0.52 | . | . | . |
| ABCC4 ---- tag | rs4148544 | G/G | 21 | 35,00 | 11 | 34,38 | 134 | 39,30 | 142 | 46,41 | 1.00 (.-.) | . | 1.62 (0.77-3.42) | 0.20 | 0.82 | 0.99 | 1.00 |
| ABCC4 ---- tag |  | G/A | 28 | 46,67 | 18 | 56,25 | 176 | 51,61 | 123 | 40,20 | 1.17 (0.49-2.80) | 0.73 | 1.30 (0.61-2.76) | 0.50 | . | . | . |
| ABCC4 ---- tag |  | A/A | 11 | 18,33 | 3 | 9,38 | 31 | 9,09 | 41 | 13,40 | 0.76 (0.16-3.62) | 0.73 | 1.46 (0.64-3.30) | 0.37 | . | . | . |
| ABCC4 ---- tag | rs4283094 | C/C | 10 | 16,67 | 5 | 15,63 | 93 | 27,27 | 70 | 22,88 | 1.00 (.-.) | . | 1.31 (0.39-4.40) | 0.66 | 0.42 | 0.99 | 1.00 |
| ABCC4 ---- tag |  | C/G | 32 | 53,33 | 17 | 53,13 | 174 | 51,03 | 167 | 54,58 | 1.29 (0.36-4.67) | 0.70 | 1.50 (0.45-4.95) | 0.51 | . | . | . |
| ABCC4 ---- tag |  | G/G | 18 | 30,00 | 10 | 31,25 | 74 | 21,70 | 69 | 22,55 | 0.77 (0.20-3.03) | 0.71 | 1.40 (0.42-4.69) | 0.58 | . | . | . |
| ABCC4 ---- tag | rs4636781 | A/A | 45 | 75,00 | 27 | 84,38 | 244 | 71,55 | 212 | 69,28 | 1.00 (.-.) | . | 1.23 (0.76-2.00) | 0.41 | 0.55 | 0.99 | 1.00 |
| ABCC4 ---- tag |  | A/G | 15 | 25,00 | 4 | 12,50 | 87 | 25,51 | 85 | 27,78 | 0.83 (0.28-2.46) | 0.73 | 1.73 (1.02-2.92) | 0.04 | . | . | . |
| ABCC4 ---- tag |  | G/G | 0 | 0,00 | 1 | 3,13 | 10 | 2,93 | 9 | 2,94 | 3.04 (0.38-24.23) | 0.29 | 2.80 (1.18-6.63) | 0.02 | . | . | . |
| ABCC4 ---- tag | rs4771910 | T/T | 32 | 53,33 | 14 | 43,75 | 161 | 47,21 | 146 | 47,71 | 1.00 (.-.) | . | 1.74 (0.90-3.37) | 0.10 | 0.42 | 0.99 | 1.00 |
| ABCC4 ---- tag |  | T/C | 24 | 40,00 | 16 | 50,00 | 148 | 43,40 | 133 | 43,46 | 1.36 (0.60-3.10) | 0.47 | 1.54 (0.79-3.00) | 0.20 | . | . | . |
| ABCC4 ---- tag |  | C/C | 4 | 6,67 | 2 | 6,25 | 32 | 9,38 | 27 | 8,82 | 0.88 (0.11-7.00) | 0.91 | 1.30 (0.60-2.81) | 0.50 | . | . | . |
| ABCC4 ---- tag | rs4773850 | T/T | 30 | 50,00 | 14 | 43,75 | 150 | 43,99 | 157 | 51,31 | 1.00 (.-.) | . | 1.82 (0.99-3.34) | 0.05 | 0.11 | 0.99 | 0.96 |
| ABCC4 ---- tag |  | T/G | 28 | 46,67 | 13 | 40,63 | 147 | 43,11 | 119 | 38,89 | 1.30 (0.55-3.04) | 0.55 | 1.54 (0.83-2.85) | 0.17 | . | . | . |
| ABCC4 ---- tag |  | G/G | 2 | 3,33 | 5 | 15,63 | 44 | 12,90 | 30 | 9,80 | 1.90 (0.50-7.28) | 0.35 | 1.09 (0.53-2.23) | 0.82 | . | . | . |
| ABCC4 ---- tag | rs7981095 | A/A | 39 | 65,00 | 20 | 62,50 | 223 | 65,40 | 196 | 64,05 | 1.00 (.-.) | . | 1.27 (0.76-2.12) | 0.37 | 0.73 | 0.99 | 1.00 |
| ABCC4 ---- tag |  | A/T | 20 | 33,33 | 8 | 25,00 | 108 | 31,67 | 99 | 32,35 | 0.65 (0.27-1.61) | 0.36 | 1.09 (0.63-1.88) | 0.76 | . | . | . |
| ABCC4 ---- tag |  | T/T | 1 | 1,67 | 4 | 12,50 | 10 | 2,93 | 11 | 3,59 | 1.28 (0.16-10.01) | 0.82 | 1.29 (0.53-3.18) | 0.57 | . | . | . |
| ABCC4 ---- tag | rs8001444 | C/C | 21 | 35,00 | 9 | 28,13 | 118 | 34,60 | 118 | 38,56 | 1.00 (.-.) | . | 1.53 (0.66-3.58) | 0.32 | 0.94 | 1.00 | 1.00 |
| ABCC4 ---- tag |  | C/T | 33 | 55,00 | 20 | 62,50 | 163 | 47,80 | 134 | 43,79 | 0.96 (0.37-2.48) | 0.93 | 1.13 (0.48-2.62) | 0.78 | . | . | . |
| ABCC4 ---- tag |  | T/T | 6 | 10,00 | 3 | 9,38 | 60 | 17,60 | 54 | 17,65 | 0.64 (0.12-3.24) | 0.59 | 1.26 (0.52-3.03) | 0.61 | . | . | . |
| ABCC4 ---- tag | rs931111 | T/T | 36 | 60,00 | 19 | 59,38 | 238 | 69,79 | 198 | 64,71 | 1.00 (.-.) | . | 1.49 (0.85-2.62) | 0.17 | 0.82 | 0.99 | 1.00 |
| ABCC4 ---- tag |  | T/C | 20 | 33,33 | 11 | 34,38 | 90 | 26,39 | 94 | 30,72 | 1.37 (0.58-3.23) | 0.47 | 1.58 (0.88-2.82) | 0.12 | . | . | . |
| ABCC4 ---- tag |  | C/C | 4 | 6,67 | 2 | 6,25 | 13 | 3,81 | 14 | 4,58 | 0.95 (0.21-4.31) | 0.94 | 1.46 (0.64-3.34) | 0.37 | . | . | . |
| ABCC4 ---- tag | rs943288 | T/T | 47 | 78,33 | 28 | 87,50 | 259 | 75,95 | 228 | 74,51 | 1.00 (.-.) | . | 1.20 (0.75-1.92) | 0.46 | 0.36 | 0.99 | 1.00 |
| ABCC4 ---- tag |  | T/A | 13 | 21,67 | 3 | 9,38 | 75 | 21,99 | 71 | 23,20 | 0.63 (0.19-2.15) | 0.46 | 1.69 (1.00-2.86) | 0.05 | . | . | . |
| ABCC4 ---- tag |  | A/A | 0 | 0,00 | 1 | 3,13 | 7 | 2,05 | 7 | 2,29 | 3.05 (0.38-24.27) | 0.29 | 3.04 (1.25-7.37) | 0.01 | . | . | . |
| ABCC4 ---- tag | rs943290 | A/A | 33 | 55,00 | 19 | 59,38 | 165 | 48,39 | 170 | 55,56 | 1.00 (.-.) | . | 1.52 (0.90-2.55) | 0.12 | 0.94 | 1.00 | 1.00 |
| ABCC4 ---- tag |  | A/G | 21 | 35,00 | 11 | 34,38 | 148 | 43,40 | 116 | 37,91 | 1.00 (0.41-2.46) | 1.00 | 1.09 (0.64-1.85) | 0.76 | . | . | . |
| ABCC4 ---- tag |  | G/G | 6 | 10,00 | 2 | 6,25 | 28 | 8,21 | 20 | 6,54 | 0.43 (0.05-3.35) | 0.42 | 1.16 (0.57-2.35) | 0.69 | . | . | . |
| ABCC4 ---- tag | rs9516530 | C/C | 32 | 53,33 | 22 | 68,75 | 193 | 56,60 | 168 | 54,90 | 1.00 (.-.) | . | 1.01 (0.60-1.71) | 0.96 | 0.07 | 0.99 | 0.76 |
| ABCC4 ---- tag |  | C/T | 26 | 43,33 | 8 | 25,00 | 116 | 34,02 | 112 | 36,60 | 0.51 (0.20-1.31) | 0.16 | 1.12 (0.66-1.90) | 0.68 | . | . | . |
| ABCC4 ---- tag |  | T/T | 2 | 3,33 | 2 | 6,25 | 32 | 9,38 | 26 | 8,50 | 0.50 (0.06-3.94) | 0.51 | 1.39 (0.73-2.66) | 0.32 | . | . | . |
| ABCC4 ---- tag | rs9516551 | C/C | 45 | 75,00 | 21 | 65,63 | 260 | 76,25 | 241 | 78,76 | 1.00 (.-.) | . | 1.60 (0.93-2.77) | 0.09 | 0.36 | 0.99 | 1.00 |
| ABCC4 ---- tag |  | C/A | 14 | 23,33 | 11 | 34,38 | 77 | 22,58 | 62 | 20,26 | 1.30 (0.58-2.92) | 0.53 | 1.26 (0.70-2.27) | 0.45 | . | . | . |
| ABCC4 ---- tag |  | A/A | 1 | 1,67 | 0 | 0,00 | 4 | 1,17 | 3 | 0,98 | 0.00 (0.00-I) | 0.98 | 1.03 (0.28-3.75) | 0.96 | . | . | . |
| ABCC4 ---- tag | rs9524822 | T/T | 48 | 80,00 | 18 | 56,25 | 213 | 62,46 | 200 | 65,36 | 1.00 (.-.) | . | 1.87 (1.05-3.33) | 0.03 | 0.01 | 0.99 | 0.75 |
| ABCC4 ---- tag |  | T/C | 12 | 20,00 | 10 | 31,25 | 112 | 32,84 | 94 | 30,72 | 1.39 (0.58-3.31) | 0.46 | 1.53 (0.84-2.79) | 0.16 | . | . | . |
| ABCC4 ---- tag |  | C/C | 0 | 0,00 | 4 | 12,50 | 16 | 4,69 | 12 | 3,92 | 6.84 (1.84-25.42) | 0.00 | 1.25 (0.47-3.35) | 0.66 | . | . | . |
| ABCC4 ---- tag | rs9524861 | G/G | 25 | 41,67 | 13 | 40,63 | 177 | 51,91 | 162 | 52,94 | 1.00 (.-.) | . | 1.21 (0.62-2.37) | 0.57 | 0.52 | 0.99 | 1.00 |
| ABCC4 ---- tag |  | G/C | 26 | 43,33 | 15 | 46,88 | 140 | 41,06 | 121 | 39,54 | 0.93 (0.40-2.19) | 0.87 | 1.13 (0.57-2.23) | 0.72 | . | . | . |
| ABCC4 ---- tag |  | C/C | 9 | 15,00 | 4 | 12,50 | 24 | 7,04 | 23 | 7,52 | 0.43 (0.11-1.60) | 0.21 | 0.86 (0.39-1.90) | 0.71 | . | . | . |
| ABCC4 ---- tag | rs9524902 | T/T | 17 | 28,33 | 6 | 18,75 | 88 | 25,81 | 103 | 33,66 | 1.00 (.-.) | . | 1.71 (0.62-4.74) | 0.30 | 0.43 | 0.99 | 1.00 |
| ABCC4 ---- tag |  | T/C | 32 | 53,33 | 15 | 46,88 | 177 | 51,91 | 141 | 46,08 | 0.91 (0.29-2.83) | 0.87 | 1.20 (0.44-3.33) | 0.72 | . | . | . |
| ABCC4 ---- tag |  | C/C | 11 | 18,33 | 11 | 34,38 | 76 | 22,29 | 62 | 20,26 | 1.23 (0.36-4.19) | 0.75 | 1.36 (0.48-3.84) | 0.56 | . | . | . |
| ABCC4 ---- tag | rs9556455 | G/G | 47 | 78,33 | 21 | 65,63 | 262 | 76,83 | 232 | 75,82 | 1.00 (.-.) | . | 1.58 (0.93-2.70) | 0.09 | 0.38 | 0.99 | 1.00 |
| ABCC4 ---- tag |  | G/A | 12 | 20,00 | 10 | 31,25 | 76 | 22,29 | 68 | 22,22 | 1.59 (0.67-3.81) | 0.29 | 1.63 (0.91-2.93) | 0.10 | . | . | . |
| ABCC4 ---- tag |  | A/A | 1 | 1,67 | 1 | 3,13 | 3 | 0,88 | 6 | 1,96 | 1.18 (0.15-9.30) | 0.87 | 0.94 (0.30-2.91) | 0.91 | . | . | . |
| ABCC4 ---- NA | rs9561778 | G/G | 37 | 61,67 | 23 | 71,88 | 214 | 62,76 | 211 | 68,95 | 1.00 (.-.) | . | 1.31 (0.82-2.11) | 0.26 | 0.41 | 0.99 | 1.00 |
| ABCC4 ---- NA |  | G/T | 21 | 35,00 | 9 | 28,13 | 112 | 32,84 | 86 | 28,10 | 0.97 (0.33-2.85) | 0.95 | 1.14 (0.68-1.91) | 0.63 | . | . | . |
| ABCC4 ---- NA |  | T/T | 2 | 3,33 | 0 | 0,00 | 15 | 4,40 | 9 | 2,94 | 0.00 (0.00-I) | 0.97 | 0.95 (0.39-2.29) | 0.91 | . | . | . |
| ABCC4 ---- tag | rs9561811 | C/C | 39 | 65,00 | 26 | 81,25 | 232 | 68,04 | 198 | 64,71 | 1.00 (.-.) | . | 1.36 (0.83-2.25) | 0.22 | 0.91 | 1.00 | 1.00 |
| ABCC4 ---- tag |  | C/T | 20 | 33,33 | 6 | 18,75 | 97 | 28,45 | 92 | 30,07 | 1.29 (0.50-3.34) | 0.60 | 1.55 (0.91-2.63) | 0.10 | . | . | . |
| ABCC4 ---- tag |  | T/T | 1 | 1,67 | 0 | 0,00 | 12 | 3,52 | 16 | 5,23 | 0.00 (0.00-I) | 0.98 | 2.08 (1.01-4.27) | 0.05 | . | . | . |
| ABCC4 ---- tag | rs9590183 | T/T | 54 | 90,00 | 30 | 93,75 | 293 | 85,92 | 264 | 86,27 | 1.00 (.-.) | . | 1.34 (0.86-2.10) | 0.19 | 0.67 | 0.99 | 1.00 |
| ABCC4 ---- tag |  | T/A | 6 | 10,00 | 2 | 6,25 | 46 | 13,49 | 42 | 13,73 | 0.54 (0.12-2.35) | 0.41 | 1.01 (0.57-1.78) | 0.98 | . | . | . |
| ABCC4 ---- tag |  | A/A | 0 | 0,00 | 0 | 0,00 | 2 | 0,59 | 0 | 0,00 | 0.00 (0.00-7E293) | 0.98 | 0.00 (0.00-1E294) | 0.98 | . | . | . |
| ABCC4 ---- tag | rs997777 | T/T | 27 | 45,00 | 15 | 46,88 | 166 | 48,68 | 153 | 50,00 | 1.00 (.-.) | . | 1.64 (0.90-3.00) | 0.11 | 0.62 | 0.99 | 1.00 |
| ABCC4 ---- tag |  | T/A | 24 | 40,00 | 14 | 43,75 | 149 | 43,70 | 128 | 41,83 | 1.69 (0.74-3.87) | 0.22 | 1.65 (0.90-3.04) | 0.11 | . | . | . |
| ABCC4 ---- tag |  | A/A | 9 | 15,00 | 3 | 9,38 | 26 | 7,62 | 25 | 8,17 | 0.61 (0.08-4.81) | 0.64 | 1.48 (0.71-3.08) | 0.30 | . | . | . |
| ADH1B ---- tag | rs1159918 | G/G | 20 | 33,33 | 13 | 40,63 | 154 | 45,16 | 148 | 48,37 | 1.00 (.-.) | . | 0.90 (0.47-1.73) | 0.76 | 0.06 | 0.99 | 0.11 |
| ADH1B ---- tag |  | G/T | 32 | 53,33 | 15 | 46,88 | 150 | 43,99 | 134 | 43,79 | 0.62 (0.27-1.42) | 0.26 | 0.86 (0.45-1.65) | 0.66 | . | . | . |
| ADH1B ---- tag |  | T/T | 8 | 13,33 | 4 | 12,50 | 37 | 10,85 | 24 | 7,84 | 0.26 (0.05-1.21) | 0.09 | 1.06 (0.49-2.30) | 0.87 | . | . | . |
| ADH1B ---- candidate literature | rs1229984 | G/G | 52 | 86,67 | 31 | 96,88 | 308 | 90,32 | 277 | 90,52 | 1.00 (.-.) | . | 1.21 (0.78-1.88) | 0.39 | 0.07 | 0.99 | 0.11 |
| ADH1B ---- candidate literature |  | G/A | 7 | 11,67 | 1 | 3,13 | 31 | 9,09 | 29 | 9,48 | 0.24 (0.03-1.79) | 0.16 | 1.39 (0.76-2.56) | 0.29 | . | . | . |
| ADH1B ---- candidate literature |  | A/A | 1 | 1,67 | 0 | 0,00 | 2 | 0,59 | 0 | 0,00 | 0.00 (0.00-I) | 0.99 | 0.00 (0.00-I) | 0.98 | . | . | . |
| ADH1B ---- tag | rs12507573 | C/C | 19 | 31,67 | 12 | 37,50 | 103 | 30,21 | 85 | 27,78 | 1.00 (.-.) | . | 1.43 (0.67-3.05) | 0.36 | 0.34 | 0.99 | 0.43 |
| ADH1B ---- tag |  | C/A | 26 | 43,33 | 19 | 59,38 | 164 | 48,09 | 146 | 47,71 | 1.19 (0.50-2.83) | 0.69 | 1.29 (0.61-2.70) | 0.51 | . | . | . |
| ADH1B ---- tag |  | A/A | 15 | 25,00 | 1 | 3,13 | 74 | 21,70 | 75 | 24,51 | 0.00 (0.00-9E292) | 0.97 | 1.44 (0.68-3.07) | 0.34 | . | . | . |
| ADH1B ---- tag | rs1693457 | T/T | 40 | 66,67 | 19 | 59,38 | 232 | 68,04 | 224 | 73,20 | 1.00 (.-.) | . | 1.17 (0.69-1.98) | 0.57 | 0.58 | 0.99 | 0.58 |
| ADH1B ---- tag |  | T/C | 19 | 31,67 | 11 | 34,38 | 97 | 28,45 | 74 | 24,18 | 0.57 (0.24-1.34) | 0.19 | 1.03 (0.58-1.82) | 0.92 | . | . | . |
| ADH1B ---- tag |  | C/C | 1 | 1,67 | 2 | 6,25 | 12 | 3,52 | 8 | 2,61 | 2.16 (0.28-16.96) | 0.46 | 1.02 (0.39-2.68) | 0.96 | . | . | . |
| ADH1B ---- tag | rs2066701 | C/C | 30 | 50,00 | 9 | 28,13 | 159 | 46,63 | 142 | 46,41 | 1.00 (.-.) | . | 2.64 (1.14-6.09) | 0.02 | 0.03 | 0.99 | 0.11 |
| ADH1B ---- tag |  | C/T | 28 | 46,67 | 21 | 65,63 | 147 | 43,11 | 131 | 42,81 | 2.39 (0.93-6.14) | 0.07 | 2.41 (1.04-5.57) | 0.04 | . | . | . |
| ADH1B ---- tag |  | T/T | 2 | 3,33 | 2 | 6,25 | 35 | 10,26 | 33 | 10,78 | 6.15 (1.21-31.29) | 0.03 | 3.19 (1.28-7.98) | 0.01 | . | . | . |
| ADH1C ---- tag | rs11936869 | C/C | 30 | 50,00 | 12 | 37,50 | 176 | 51,61 | 170 | 55,56 | 1.00 (.-.) | . | 1.52 (0.80-2.87) | 0.20 | 0.50 | 0.99 | 0.64 |
| ADH1C ---- tag |  | C/G | 25 | 41,67 | 15 | 46,88 | 135 | 39,59 | 110 | 35,95 | 1.07 (0.46-2.50) | 0.88 | 1.41 (0.74-2.70) | 0.30 | . | . | . |
| ADH1C ---- tag |  | G/G | 5 | 8,33 | 5 | 15,63 | 30 | 8,80 | 26 | 8,50 | 1.83 (0.49-6.81) | 0.37 | 1.64 (0.78-3.45) | 0.19 | . | . | . |
| ADH1C ---- tag | rs1229849 | T/T | 34 | 56,67 | 19 | 59,38 | 190 | 55,72 | 144 | 47,06 | 1.00 (.-.) | . | 1.23 (0.70-2.15) | 0.48 | 0.47 | 0.99 | 0.64 |
| ADH1C ---- tag |  | T/A | 24 | 40,00 | 11 | 34,38 | 135 | 39,59 | 139 | 45,42 | 0.98 (0.41-2.36) | 0.97 | 1.46 (0.83-2.55) | 0.19 | . | . | . |
| ADH1C ---- tag |  | A/A | 2 | 3,33 | 2 | 6,25 | 16 | 4,69 | 23 | 7,52 | 0.96 (0.21-4.34) | 0.95 | 2.04 (1.00-4.17) | 0.05 | . | . | . |
| ADH1C ---- tag | rs1229863 | A/A | 44 | 73,33 | 22 | 68,75 | 247 | 72,43 | 226 | 73,86 | 1.00 (.-.) | . | 1.62 (0.98-2.69) | 0.06 | 0.35 | 0.99 | 0.64 |
| ADH1C ---- tag |  | A/T | 16 | 26,67 | 7 | 21,88 | 88 | 25,81 | 70 | 22,88 | 1.87 (0.67-5.20) | 0.23 | 1.36 (0.78-2.38) | 0.28 | . | . | . |
| ADH1C ---- tag |  | T/T | 0 | 0,00 | 3 | 9,38 | 6 | 1,76 | 10 | 3,27 | 1.41 (0.31-6.53) | 0.66 | 2.63 (1.11-6.23) | 0.03 | . | . | . |
| ADH1C ---- tag | rs1229980 | C/C | 53 | 88,33 | 29 | 90,63 | 312 | 91,50 | 271 | 88,56 | 1.00 (.-.) | . | 1.40 (0.89-2.20) | 0.14 | 0.46 | 0.99 | 0.64 |
| ADH1C ---- tag |  | C/G | 7 | 11,67 | 3 | 9,38 | 29 | 8,50 | 33 | 10,78 | 2.52 (0.58-10.98) | 0.22 | 1.79 (1.01-3.19) | 0.05 | . | . | . |
| ADH1C ---- tag |  | G/G | 0 | 0,00 | 0 | 0,00 | 0 | 0,00 | 2 | 0,65 | 3.16 (0.74-13.55) | 0.12 | 4.43 (0.97-20.27) | 0.06 | . | . | . |
| ADH1C ---- candidate | rs1693482 | C/C | 29 | 48,33 | 13 | 40,63 | 146 | 42,82 | 117 | 38,24 | 1.00 (.-.) | . | 1.47 (0.77-2.81) | 0.24 | 0.91 | 1.00 | 0.92 |
| ADH1C ---- candidate |  | C/T | 26 | 43,33 | 13 | 40,63 | 152 | 44,57 | 141 | 46,08 | 1.19 (0.49-2.91) | 0.70 | 1.48 (0.78-2.81) | 0.23 | . | . | . |
| ADH1C ---- candidate |  | T/T | 5 | 8,33 | 6 | 18,75 | 43 | 12,61 | 48 | 15,69 | 1.24 (0.42-3.67) | 0.70 | 1.84 (0.91-3.71) | 0.09 | . | . | . |
| ADH1C ---- tag | rs2173201 | C/C | 34 | 56,67 | 13 | 40,63 | 196 | 57,48 | 184 | 60,13 | 1.00 (.-.) | . | 1.93 (1.05-3.53) | 0.03 | 0.07 | 0.99 | 0.64 |
| ADH1C ---- tag |  | C/A | 22 | 36,67 | 17 | 53,13 | 124 | 36,36 | 102 | 33,33 | 2.26 (0.98-5.21) | 0.06 | 1.92 (1.03-3.58) | 0.04 | . | . | . |
| ADH1C ---- tag |  | A/A | 4 | 6,67 | 2 | 6,25 | 21 | 6,16 | 20 | 6,54 | 2.95 (0.63-13.79) | 0.17 | 2.38 (1.11-5.12) | 0.03 | . | . | . |
| ADH1C ---- tag | rs2298753 | T/T | 51 | 85,00 | 25 | 78,13 | 272 | 79,77 | 257 | 83,99 | 1.00 (.-.) | . | 1.54 (0.95-2.48) | 0.08 | 0.26 | 0.99 | 0.64 |
| ADH1C ---- tag |  | T/C | 9 | 15,00 | 4 | 12,50 | 67 | 19,65 | 45 | 14,71 | 1.42 (0.41-4.92) | 0.58 | 1.08 (0.61-1.93) | 0.80 | . | . | . |
| ADH1C ---- tag |  | C/C | 0 | 0,00 | 3 | 9,38 | 2 | 0,59 | 4 | 1,31 | 1.34 (0.29-6.13) | 0.71 | 3.29 (0.69-15.67) | 0.13 | . | . | . |
| ADH1C ---- tag | rs2866152 | G/G | 40 | 66,67 | 21 | 65,63 | 213 | 62,46 | 172 | 56,21 | 1.00 (.-.) | . | 1.25 (0.73-2.15) | 0.41 | 0.41 | 0.99 | 0.64 |
| ADH1C ---- tag |  | G/C | 19 | 31,67 | 10 | 31,25 | 115 | 33,72 | 118 | 38,56 | 1.06 (0.45-2.52) | 0.89 | 1.45 (0.84-2.51) | 0.19 | . | . | . |
| ADH1C ---- tag |  | C/C | 1 | 1,67 | 1 | 3,13 | 13 | 3,81 | 16 | 5,23 | 0.48 (0.06-3.71) | 0.48 | 1.63 (0.75-3.53) | 0.22 | . | . | . |
| ADH1C ---- tag | rs904096 | T/T | 29 | 48,33 | 13 | 40,63 | 144 | 42,23 | 115 | 37,58 | 1.00 (.-.) | . | 1.47 (0.77-2.80) | 0.24 | 0.92 | 1.00 | 0.92 |
| ADH1C ---- tag |  | T/G | 26 | 43,33 | 13 | 40,63 | 154 | 45,16 | 143 | 46,73 | 1.19 (0.49-2.91) | 0.70 | 1.49 (0.79-2.81) | 0.22 | . | . | . |
| ADH1C ---- tag |  | G/G | 5 | 8,33 | 6 | 18,75 | 43 | 12,61 | 48 | 15,69 | 1.24 (0.42-3.67) | 0.70 | 1.84 (0.91-3.71) | 0.09 | . | . | . |
| BHMT ---- tag | rs10944 | A/A | 16 | 26,67 | 7 | 21,88 | 72 | 21,11 | 78 | 25,49 | 1.00 (.-.) | . | 1.73 (0.68-4.43) | 0.25 | 0.63 | 0.99 | 0.74 |
| BHMT ---- tag |  | A/C | 27 | 45,00 | 18 | 56,25 | 187 | 54,84 | 153 | 50,00 | 1.32 (0.47-3.69) | 0.60 | 1.26 (0.50-3.19) | 0.62 | . | . | . |
| BHMT ---- tag |  | C/C | 17 | 28,33 | 7 | 21,88 | 82 | 24,05 | 75 | 24,51 | 0.74 (0.21-2.63) | 0.65 | 1.75 (0.69-4.46) | 0.24 | . | . | . |
| BHMT ---- tag | rs12655567 | C/C | 24 | 40,00 | 12 | 37,50 | 115 | 33,72 | 122 | 39,87 | 1.00 (.-.) | . | 2.01 (0.99-4.10) | 0.05 | 0.28 | 0.99 | 0.74 |
| BHMT ---- tag |  | C/G | 30 | 50,00 | 15 | 46,88 | 176 | 51,61 | 144 | 47,06 | 1.22 (0.50-2.98) | 0.66 | 1.29 (0.64-2.62) | 0.48 | . | . | . |
| BHMT ---- tag |  | G/G | 6 | 10,00 | 5 | 15,63 | 50 | 14,66 | 40 | 13,07 | 1.28 (0.38-4.32) | 0.69 | 1.61 (0.75-3.45) | 0.22 | . | . | . |
| BHMT ---- tag | rs1291041 | G/G | 28 | 46,67 | 14 | 43,75 | 131 | 38,42 | 143 | 46,73 | 1.00 (.-.) | . | 1.95 (0.99-3.82) | 0.05 | 0.50 | 0.99 | 0.74 |
| BHMT ---- tag |  | G/T | 27 | 45,00 | 14 | 43,75 | 168 | 49,27 | 128 | 41,83 | 1.29 (0.54-3.06) | 0.57 | 1.19 (0.61-2.33) | 0.62 | . | . | . |
| BHMT ---- tag |  | T/T | 5 | 8,33 | 4 | 12,50 | 42 | 12,32 | 35 | 11,44 | 0.95 (0.25-3.57) | 0.94 | 1.98 (0.95-4.16) | 0.07 | . | . | . |
| BHMT ---- tag | rs16876500 | C/C | 50 | 83,33 | 25 | 78,13 | 277 | 81,23 | 237 | 77,45 | 1.00 (.-.) | . | 1.30 (0.81-2.08) | 0.28 | 0.35 | 0.99 | 0.74 |
| BHMT ---- tag |  | C/T | 9 | 15,00 | 6 | 18,75 | 61 | 17,89 | 64 | 20,92 | 1.04 (0.34-3.14) | 0.95 | 1.57 (0.91-2.70) | 0.11 | . | . | . |
| BHMT ---- tag |  | T/T | 1 | 1,67 | 1 | 3,13 | 3 | 0,88 | 5 | 1,63 | 0.54 (0.07-4.21) | 0.56 | 2.15 (0.61-7.60) | 0.24 | . | . | . |
| BHMT ---- tag | rs492842 | A/A | 25 | 41,67 | 12 | 37,50 | 129 | 37,83 | 122 | 39,87 | 1.00 (.-.) | . | 2.02 (0.99-4.12) | 0.05 | 0.43 | 0.99 | 0.74 |
| BHMT ---- tag |  | A/G | 23 | 38,33 | 18 | 56,25 | 168 | 49,27 | 141 | 46,08 | 1.91 (0.80-4.54) | 0.15 | 1.80 (0.89-3.66) | 0.10 | . | . | . |
| BHMT ---- tag |  | G/G | 12 | 20,00 | 2 | 6,25 | 44 | 12,90 | 43 | 14,05 | 0.92 (0.19-4.30) | 0.91 | 1.78 (0.83-3.80) | 0.14 | . | . | . |
| BHMT ---- tag | rs558133 | T/T | 27 | 45,00 | 16 | 50,00 | 163 | 47,80 | 146 | 47,71 | 1.00 (.-.) | . | 1.57 (0.85-2.91) | 0.15 | 0.96 | 1.00 | 0.96 |
| BHMT ---- tag |  | T/G | 19 | 31,67 | 13 | 40,63 | 142 | 41,64 | 129 | 42,16 | 1.52 (0.66-3.52) | 0.33 | 1.47 (0.79-2.74) | 0.22 | . | . | . |
| BHMT ---- tag |  | G/G | 14 | 23,33 | 3 | 9,38 | 36 | 10,56 | 31 | 10,13 | 0.62 (0.14-2.81) | 0.54 | 1.63 (0.80-3.30) | 0.18 | . | . | . |
| BHMT ---- tag | rs9637824 | A/A | 23 | 38,33 | 11 | 34,38 | 130 | 38,12 | 117 | 38,24 | 1.00 (.-.) | . | 1.78 (0.87-3.64) | 0.11 | 0.62 | 0.99 | 0.74 |
| BHMT ---- tag |  | A/G | 26 | 43,33 | 19 | 59,38 | 168 | 49,27 | 145 | 47,39 | 1.48 (0.62-3.52) | 0.37 | 1.58 (0.78-3.22) | 0.21 | . | . | . |
| BHMT ---- tag |  | G/G | 11 | 18,33 | 2 | 6,25 | 43 | 12,61 | 44 | 14,38 | 1.03 (0.22-4.87) | 0.97 | 1.84 (0.86-3.93) | 0.12 | . | . | . |
| BHMT2 ---- tag | rs16876512 | C/C | 50 | 83,33 | 25 | 78,13 | 274 | 80,35 | 237 | 77,45 | 1.00 (.-.) | . | 1.30 (0.81-2.09) | 0.27 | 0.39 | 0.99 | 0.78 |
| BHMT2 ---- tag |  | C/T | 9 | 15,00 | 6 | 18,75 | 64 | 18,77 | 63 | 20,59 | 1.04 (0.34-3.15) | 0.95 | 1.52 (0.88-2.63) | 0.13 | . | . | . |
| BHMT2 ---- tag |  | T/T | 1 | 1,67 | 1 | 3,13 | 3 | 0,88 | 6 | 1,96 | 0.54 (0.07-4.21) | 0.56 | 2.15 (0.61-7.59) | 0.24 | . | . | . |
| BHMT2 ---- tag | rs2461248 | T/T | 16 | 26,67 | 6 | 18,75 | 71 | 20,82 | 78 | 25,49 | 1.00 (.-.) | . | 2.05 (0.73-5.79) | 0.17 | 0.78 | 0.99 | 0.78 |
| BHMT2 ---- tag |  | T/A | 27 | 45,00 | 19 | 59,38 | 187 | 54,84 | 152 | 49,67 | 1.63 (0.53-4.95) | 0.39 | 1.50 (0.54-4.18) | 0.44 | . | . | . |
| BHMT2 ---- tag |  | A/A | 17 | 28,33 | 7 | 21,88 | 83 | 24,34 | 76 | 24,84 | 0.88 (0.23-3.37) | 0.86 | 2.05 (0.73-5.74) | 0.17 | . | . | . |
| BHMT2 ---- tag | rs2909856 | T/T | 26 | 43,33 | 14 | 43,75 | 140 | 41,06 | 130 | 42,48 | 1.00 (.-.) | . | 1.78 (0.93-3.41) | 0.08 | 0.53 | 0.99 | 0.78 |
| BHMT2 ---- tag |  | T/C | 24 | 40,00 | 16 | 50,00 | 161 | 47,21 | 136 | 44,44 | 1.52 (0.66-3.53) | 0.33 | 1.53 (0.80-2.91) | 0.20 | . | . | . |
| BHMT2 ---- tag |  | C/C | 10 | 16,67 | 2 | 6,25 | 40 | 11,73 | 40 | 13,07 | 0.99 (0.22-4.52) | 0.99 | 1.73 (0.85-3.52) | 0.13 | . | . | . |
| BHMT2 ---- tag | rs476620 | A/A | 23 | 38,33 | 11 | 34,38 | 130 | 38,12 | 116 | 37,91 | 1.00 (.-.) | . | 1.77 (0.87-3.63) | 0.12 | 0.63 | 0.99 | 0.78 |
| BHMT2 ---- tag |  | A/G | 26 | 43,33 | 19 | 59,38 | 168 | 49,27 | 146 | 47,71 | 1.48 (0.62-3.52) | 0.37 | 1.59 (0.78-3.23) | 0.20 | . | . | . |
| BHMT2 ---- tag |  | G/G | 11 | 18,33 | 2 | 6,25 | 43 | 12,61 | 44 | 14,38 | 1.04 (0.22-4.87) | 0.97 | 1.84 (0.86-3.93) | 0.12 | . | . | . |
| BHMT2 ---- candidate literature | rs626105 | G/G | 33 | 55,00 | 23 | 71,88 | 214 | 62,76 | 196 | 64,05 | 1.00 (.-.) | . | 1.39 (0.83-2.32) | 0.21 | 0.74 | 0.99 | 0.78 |
| BHMT2 ---- candidate literature |  | G/A | 24 | 40,00 | 9 | 28,13 | 118 | 34,60 | 101 | 33,01 | 0.84 (0.34-2.05) | 0.70 | 1.08 (0.63-1.85) | 0.77 | . | . | . |
| BHMT2 ---- candidate literature |  | A/A | 3 | 5,00 | 0 | 0,00 | 9 | 2,64 | 9 | 2,94 | 0.00 (0.00-4E251) | 0.97 | 1.55 (0.62-3.83) | 0.35 | . | . | . |
| BHMT2 ---- tag | rs631305 | G/G | 37 | 61,67 | 26 | 81,25 | 237 | 69,50 | 217 | 70,92 | 1.00 (.-.) | . | 1.33 (0.82-2.16) | 0.25 | 0.53 | 0.99 | 0.78 |
| BHMT2 ---- tag |  | G/A | 20 | 33,33 | 6 | 18,75 | 95 | 27,86 | 81 | 26,47 | 0.71 (0.26-1.94) | 0.51 | 1.06 (0.63-1.78) | 0.84 | . | . | . |
| BHMT2 ---- tag |  | A/A | 3 | 5,00 | 0 | 0,00 | 9 | 2,64 | 8 | 2,61 | 0.00 (0.00-9E251) | 0.97 | 1.38 (0.54-3.55) | 0.50 | . | . | . |
| CBS ---- tag | rs11701048 | C/C | 52 | 86,67 | 26 | 81,25 | 285 | 83,58 | 270 | 88,24 | 1.00 (.-.) | . | 1.53 (0.95-2.46) | 0.08 | 0.21 | 0.99 | 0.90 |
| CBS ---- tag |  | C/T | 8 | 13,33 | 6 | 18,75 | 55 | 16,13 | 35 | 11,44 | 1.82 (0.67-4.92) | 0.24 | 1.38 (0.77-2.47) | 0.28 | . | . | . |
| CBS ---- tag |  | T/T | 0 | 0,00 | 0 | 0,00 | 1 | 0,29 | 1 | 0,33 | 0.00 (0.00-5E287) | 0.98 | 0.00 (0.00-7E287) | 0.98 | . | . | . |
| CBS ---- tag | rs234706 | G/G | 24 | 40,00 | 12 | 37,50 | 153 | 44,87 | 121 | 39,54 | 1.00 (.-.) | . | 1.06 (0.54-2.11) | 0.86 | 0.29 | 0.99 | 0.90 |
| CBS ---- tag |  | G/A | 27 | 45,00 | 17 | 53,13 | 147 | 43,11 | 144 | 47,06 | 0.84 (0.36-1.95) | 0.69 | 1.23 (0.63-2.40) | 0.54 | . | . | . |
| CBS ---- tag |  | A/A | 9 | 15,00 | 3 | 9,38 | 41 | 12,02 | 41 | 13,40 | 0.45 (0.10-2.09) | 0.31 | 1.05 (0.50-2.20) | 0.89 | . | . | . |
| CBS ---- tag | rs234711 | C/C | 36 | 60,00 | 18 | 56,25 | 208 | 61,00 | 168 | 54,90 | 1.00 (.-.) | . | 1.26 (0.71-2.21) | 0.43 | 0.64 | 0.99 | 0.90 |
| CBS ---- tag |  | C/A | 20 | 33,33 | 13 | 40,63 | 112 | 32,84 | 119 | 38,89 | 0.97 (0.43-2.20) | 0.94 | 1.44 (0.82-2.54) | 0.20 | . | . | . |
| CBS ---- tag |  | A/A | 4 | 6,67 | 1 | 3,13 | 21 | 6,16 | 19 | 6,21 | 0.00 (0.00-I) | 0.98 | 1.34 (0.61-2.94) | 0.46 | . | . | . |
| CBS ---- candidate literature | rs234713 | G/G | 30 | 50,00 | 16 | 50,00 | 172 | 50,44 | 142 | 46,41 | 1.00 (.-.) | . | 1.34 (0.73-2.45) | 0.35 | 0.85 | 1.00 | 0.95 |
| CBS ---- candidate literature |  | G/A | 24 | 40,00 | 12 | 37,50 | 134 | 39,30 | 137 | 44,77 | 0.99 (0.43-2.29) | 0.98 | 1.49 (0.82-2.70) | 0.19 | . | . | . |
| CBS ---- candidate literature |  | A/A | 6 | 10,00 | 4 | 12,50 | 35 | 10,26 | 27 | 8,82 | 1.44 (0.32-6.48) | 0.64 | 1.27 (0.61-2.63) | 0.52 | . | . | . |
| CBS ---- tag | rs2839623 | T/T | 48 | 80,00 | 29 | 90,63 | 279 | 81,82 | 254 | 83,01 | 1.00 (.-.) | . | 1.36 (0.86-2.16) | 0.19 | 0.97 | 1.00 | 0.97 |
| CBS ---- tag |  | T/A | 12 | 20,00 | 3 | 9,38 | 58 | 17,01 | 51 | 16,67 | 1.01 (0.30-3.44) | 0.99 | 1.45 (0.84-2.49) | 0.18 | . | . | . |
| CBS ---- tag |  | A/A | 0 | 0,00 | 0 | 0,00 | 4 | 1,17 | 1 | 0,33 | 0.77 (0.11-5.56) | 0.80 | 1.05 (0.14-7.96) | 0.96 | . | . | . |
| CBS ---- tag | rs2839626 | C/C | 29 | 48,33 | 11 | 34,38 | 171 | 50,15 | 139 | 45,42 | 1.00 (.-.) | . | 1.58 (0.76-3.29) | 0.22 | 0.72 | 0.99 | 0.90 |
| CBS ---- tag |  | C/T | 26 | 43,33 | 16 | 50,00 | 140 | 41,06 | 136 | 44,44 | 1.46 (0.59-3.60) | 0.42 | 1.94 (0.93-4.05) | 0.08 | . | . | . |
| CBS ---- tag |  | T/T | 5 | 8,33 | 5 | 15,63 | 30 | 8,80 | 31 | 10,13 | 1.52 (0.45-5.14) | 0.50 | 1.83 (0.80-4.18) | 0.15 | . | . | . |
| CBS ---- tag | rs422791 | T/T | 28 | 46,67 | 20 | 62,50 | 177 | 51,91 | 137 | 44,77 | 1.00 (.-.) | . | 1.07 (0.62-1.83) | 0.82 | 0.19 | 0.99 | 0.90 |
| CBS ---- tag |  | T/C | 25 | 41,67 | 10 | 31,25 | 130 | 38,12 | 139 | 45,42 | 0.70 (0.28-1.74) | 0.45 | 1.31 (0.76-2.24) | 0.33 | . | . | . |
| CBS ---- tag |  | C/C | 7 | 11,67 | 2 | 6,25 | 34 | 9,97 | 30 | 9,80 | 0.36 (0.05-2.82) | 0.33 | 0.93 (0.48-1.82) | 0.84 | . | . | . |
| CBS ---- tag | rs706209 | C/C | 17 | 28,33 | 9 | 28,13 | 113 | 33,14 | 97 | 31,70 | 1.00 (.-.) | . | 1.13 (0.48-2.65) | 0.78 | 0.59 | 0.99 | 0.90 |
| CBS ---- tag |  | C/T | 32 | 53,33 | 12 | 37,50 | 171 | 50,15 | 149 | 48,69 | 0.77 (0.27-2.15) | 0.61 | 1.12 (0.48-2.58) | 0.80 | . | . | . |
| CBS ---- tag |  | T/T | 11 | 18,33 | 11 | 34,38 | 57 | 16,72 | 60 | 19,61 | 0.86 (0.30-2.46) | 0.77 | 1.44 (0.60-3.44) | 0.41 | . | . | . |
| CBS ---- tag | rs719037 | A/A | 19 | 31,67 | 14 | 43,75 | 112 | 32,84 | 92 | 30,07 | 1.00 (.-.) | . | 1.18 (0.62-2.26) | 0.61 | 0.36 | 0.99 | 0.90 |
| CBS ---- tag |  | A/G | 28 | 46,67 | 15 | 46,88 | 163 | 47,80 | 152 | 49,67 | 0.98 (0.42-2.26) | 0.96 | 1.27 (0.68-2.36) | 0.46 | . | . | . |
| CBS ---- tag |  | G/G | 13 | 21,67 | 3 | 9,38 | 66 | 19,35 | 62 | 20,26 | 0.50 (0.11-2.28) | 0.37 | 1.34 (0.68-2.61) | 0.40 | . | . | . |
| CBS ---- tag | rs719038 | T/T | 28 | 46,67 | 11 | 34,38 | 158 | 46,33 | 132 | 43,14 | 1.00 (.-.) | . | 1.59 (0.77-3.29) | 0.21 | 0.71 | 0.99 | 0.90 |
| CBS ---- tag |  | T/C | 26 | 43,33 | 15 | 46,88 | 147 | 43,11 | 136 | 44,44 | 1.30 (0.52-3.27) | 0.57 | 1.68 (0.81-3.49) | 0.16 | . | . | . |
| CBS ---- tag |  | C/C | 6 | 10,00 | 6 | 18,75 | 36 | 10,56 | 38 | 12,42 | 1.51 (0.49-4.69) | 0.47 | 2.05 (0.92-4.55) | 0.08 | . | . | . |
| DHFR ---- tag | rs10474632 | G/G | 53 | 88,33 | 28 | 87,50 | 286 | 83,87 | 252 | 82,35 | 1.00 (.-.) | . | 1.31 (0.83-2.08) | 0.24 | 0.75 | 0.99 | 0.81 |
| DHFR ---- tag |  | G/A | 7 | 11,67 | 4 | 12,50 | 51 | 14,96 | 54 | 17,65 | 0.82 (0.24-2.79) | 0.75 | 1.54 (0.90-2.62) | 0.12 | . | . | . |
| DHFR ---- tag |  | A/A | 0 | 0,00 | 0 | 0,00 | 4 | 1,17 | 0 | 0,00 | 0.00 (0.00-I) | 0.98 | 0.00 (0.00-I) | 0.98 | . | . | . |
| DHFR ---- tag | rs11951910 | T/T | 48 | 80,00 | 25 | 78,13 | 277 | 81,23 | 249 | 81,37 | 1.00 (.-.) | . | 1.25 (0.76-2.05) | 0.38 | 0.27 | 0.99 | 0.68 |
| DHFR ---- tag |  | T/C | 12 | 20,00 | 6 | 18,75 | 59 | 17,30 | 54 | 17,65 | 0.94 (0.34-2.57) | 0.90 | 1.68 (0.95-2.95) | 0.07 | . | . | . |
| DHFR ---- tag |  | C/C | 0 | 0,00 | 1 | 3,13 | 5 | 1,47 | 3 | 0,98 | 0.62 (0.08-4.93) | 0.66 | 2.13 (0.48-9.47) | 0.32 | . | . | . |
| DHFR ---- tag | rs1643665 | T/T | 33 | 55,00 | 11 | 34,38 | 152 | 44,57 | 143 | 46,73 | 1.00 (.-.) | . | 1.79 (0.86-3.71) | 0.12 | 0.33 | 0.99 | 0.68 |
| DHFR ---- tag |  | T/C | 21 | 35,00 | 15 | 46,88 | 145 | 42,52 | 143 | 46,73 | 1.55 (0.63-3.83) | 0.34 | 1.93 (0.93-4.02) | 0.08 | . | . | . |
| DHFR ---- tag |  | C/C | 6 | 10,00 | 6 | 18,75 | 44 | 12,90 | 20 | 6,54 | 1.35 (0.39-4.68) | 0.63 | 0.93 (0.37-2.30) | 0.87 | . | . | . |
| DHFR ---- tag | rs1650717 | T/T | 26 | 43,33 | 17 | 53,13 | 190 | 55,72 | 165 | 53,92 | 1.00 (.-.) | . | 1.10 (0.60-2.03) | 0.76 | 0.16 | 0.99 | 0.68 |
| DHFR ---- tag |  | T/G | 29 | 48,33 | 12 | 37,50 | 122 | 35,78 | 117 | 38,24 | 0.93 (0.40-2.17) | 0.86 | 1.34 (0.72-2.51) | 0.35 | . | . | . |
| DHFR ---- tag |  | G/G | 5 | 8,33 | 3 | 9,38 | 29 | 8,50 | 24 | 7,84 | 0.57 (0.16-2.06) | 0.39 | 1.67 (0.79-3.54) | 0.18 | . | . | . |
| DHFR ---- tag | rs1805355 | G/G | 52 | 86,67 | 22 | 68,75 | 302 | 88,56 | 265 | 86,60 | 1.00 (.-.) | . | 1.86 (1.10-3.14) | 0.02 | 0.01 | 0.99 | 0.07 |
| DHFR ---- tag |  | G/A | 8 | 13,33 | 10 | 31,25 | 38 | 11,14 | 40 | 13,07 | 4.37 (1.89-10.13) | 0.00 | 2.16 (1.16-4.01) | 0.02 | . | . | . |
| DHFR ---- tag |  | A/A | 0 | 0,00 | 0 | 0,00 | 1 | 0,29 | 1 | 0,33 | 0.00 (0.00-1E241) | 0.97 | 0.00 (0.00-2E241) | 0.97 | . | . | . |
| DHFR ---- tag | rs6151617 | A/A | 27 | 45,00 | 9 | 28,13 | 118 | 34,60 | 108 | 35,29 | 1.00 (.-.) | . | 1.93 (0.83-4.49) | 0.13 | 0.37 | 0.99 | 0.68 |
| DHFR ---- tag |  | A/G | 26 | 43,33 | 14 | 43,75 | 155 | 45,45 | 155 | 50,65 | 1.80 (0.67-4.82) | 0.24 | 2.20 (0.95-5.06) | 0.07 | . | . | . |
| DHFR ---- tag |  | G/G | 7 | 11,67 | 9 | 28,13 | 68 | 19,94 | 43 | 14,05 | 1.46 (0.45-4.66) | 0.53 | 1.45 (0.60-3.52) | 0.41 | . | . | . |
| DHFR ---- tag | rs6864493 | T/T | 38 | 63,33 | 17 | 53,13 | 179 | 52,49 | 178 | 58,17 | 1.00 (.-.) | . | 1.21 (0.69-2.14) | 0.51 | 0.69 | 0.99 | 0.81 |
| DHFR ---- tag |  | T/C | 19 | 31,67 | 12 | 37,50 | 135 | 39,59 | 115 | 37,58 | 0.69 (0.29-1.63) | 0.40 | 1.09 (0.61-1.95) | 0.77 | . | . | . |
| DHFR ---- tag |  | C/C | 3 | 5,00 | 3 | 9,38 | 27 | 7,92 | 13 | 4,25 | 0.59 (0.13-2.71) | 0.49 | 0.67 (0.28-1.64) | 0.38 | . | . | . |
| DHFR ---- tag | rs836788 | G/G | 25 | 41,67 | 19 | 59,38 | 153 | 44,87 | 122 | 39,87 | 1.00 (.-.) | . | 1.36 (0.77-2.40) | 0.28 | 0.81 | 0.99 | 0.81 |
| DHFR ---- tag |  | G/A | 28 | 46,67 | 10 | 31,25 | 143 | 41,94 | 142 | 46,41 | 1.04 (0.44-2.48) | 0.92 | 1.50 (0.86-2.61) | 0.16 | . | . | . |
| DHFR ---- tag |  | A/A | 7 | 11,67 | 3 | 9,38 | 45 | 13,20 | 42 | 13,73 | 1.27 (0.29-5.67) | 0.75 | 1.33 (0.71-2.49) | 0.38 | . | . | . |
| DHFR ---- tag | rs836790 | A/A | 37 | 61,67 | 22 | 68,75 | 244 | 71,55 | 209 | 68,30 | 1.00 (.-.) | . | 1.23 (0.73-2.09) | 0.44 | 0.41 | 0.99 | 0.68 |
| DHFR ---- tag |  | A/G | 21 | 35,00 | 10 | 31,25 | 88 | 25,81 | 89 | 29,08 | 0.88 (0.38-2.01) | 0.76 | 1.43 (0.82-2.50) | 0.21 | . | . | . |
| DHFR ---- tag |  | G/G | 2 | 3,33 | 0 | 0,00 | 9 | 2,64 | 8 | 2,61 | 0.00 (0.00-5E293) | 0.98 | 1.59 (0.61-4.16) | 0.34 | . | . | . |
| DHFR ---- tag | rs836817 | G/G | 26 | 43,33 | 20 | 62,50 | 172 | 50,44 | 134 | 43,79 | 1.00 (.-.) | . | 1.36 (0.79-2.32) | 0.26 | 0.68 | 0.99 | 0.81 |
| DHFR ---- tag |  | G/T | 28 | 46,67 | 10 | 31,25 | 130 | 38,12 | 145 | 47,39 | 1.12 (0.46-2.75) | 0.80 | 1.61 (0.95-2.72) | 0.08 | . | . | . |
| DHFR ---- tag |  | T/T | 6 | 10,00 | 2 | 6,25 | 39 | 11,44 | 27 | 8,82 | 1.03 (0.13-7.94) | 0.98 | 0.92 (0.47-1.78) | 0.80 | . | . | . |
| DNMT1 ---- candidate | rs2228612 | A/A | 49 | 81,67 | 26 | 81,25 | 303 | 88,86 | 267 | 87,25 | 1.00 (.-.) | . | 1.34 (0.85-2.11) | 0.21 | 0.77 | 0.99 | 0.77 |
| DNMT1 ---- candidate |  | A/G | 11 | 18,33 | 6 | 18,75 | 37 | 10,85 | 38 | 12,42 | 0.83 (0.25-2.80) | 0.76 | 1.40 (0.80-2.46) | 0.24 | . | . | . |
| DNMT1 ---- candidate |  | G/G | 0 | 0,00 | 0 | 0,00 | 1 | 0,29 | 1 | 0,33 | 0.55 (0.07-4.06) | 0.56 | 0.74 (0.10-5.67) | 0.77 | . | . | . |
| DNMT3A ---- tag | rs10460566 | A/A | 35 | 58,33 | 21 | 65,63 | 198 | 58,06 | 184 | 60,13 | 1.00 (.-.) | . | 1.46 (0.87-2.45) | 0.15 | 0.98 | 1.00 | 0.98 |
| DNMT3A ---- tag |  | A/G | 19 | 31,67 | 11 | 34,38 | 127 | 37,24 | 105 | 34,31 | 1.56 (0.66-3.69) | 0.31 | 1.50 (0.87-2.58) | 0.14 | . | . | . |
| DNMT3A ---- tag |  | G/G | 6 | 10,00 | 0 | 0,00 | 16 | 4,69 | 17 | 5,56 | 0.00 (0.00-5E289) | 0.97 | 1.33 (0.64-2.76) | 0.45 | . | . | . |
| DNMT3A ---- candidate literature | rs11695471 | T/T | 29 | 48,33 | 11 | 34,38 | 143 | 41,94 | 138 | 45,10 | 1.00 (.-.) | . | 1.18 (0.62-2.22) | 0.62 | 0.70 | 0.99 | 0.98 |
| DNMT3A ---- candidate literature |  | T/A | 24 | 40,00 | 15 | 46,88 | 152 | 44,57 | 131 | 42,81 | 0.84 (0.36-1.99) | 0.69 | 1.40 (0.74-2.64) | 0.30 | . | . | . |
| DNMT3A ---- candidate literature |  | A/A | 7 | 11,67 | 6 | 18,75 | 46 | 13,49 | 37 | 12,09 | 0.96 (0.26-3.51) | 0.95 | 1.23 (0.60-2.49) | 0.57 | . | . | . |
| DNMT3A ---- tag | rs11887120 | C/C | 19 | 31,67 | 14 | 43,75 | 124 | 36,36 | 105 | 34,31 | 1.00 (.-.) | . | 1.17 (0.58-2.37) | 0.67 | 0.73 | 0.99 | 0.98 |
| DNMT3A ---- tag |  | C/T | 32 | 53,33 | 14 | 43,75 | 164 | 48,09 | 143 | 46,73 | 0.78 (0.33-1.86) | 0.58 | 1.26 (0.62-2.53) | 0.52 | . | . | . |
| DNMT3A ---- tag |  | T/T | 9 | 15,00 | 4 | 12,50 | 53 | 15,54 | 58 | 18,95 | 0.77 (0.21-2.87) | 0.69 | 1.02 (0.49-2.11) | 0.96 | . | . | . |
| DNMT3A ---- tag | rs12991495 | T/T | 32 | 53,33 | 12 | 37,50 | 150 | 43,99 | 150 | 49,02 | 1.00 (.-.) | . | 1.26 (0.67-2.37) | 0.47 | 0.96 | 1.00 | 0.98 |
| DNMT3A ---- tag |  | T/C | 22 | 36,67 | 14 | 43,75 | 156 | 45,75 | 120 | 39,22 | 0.80 (0.33-1.92) | 0.61 | 1.31 (0.69-2.48) | 0.41 | . | . | . |
| DNMT3A ---- tag |  | C/C | 6 | 10,00 | 6 | 18,75 | 35 | 10,26 | 36 | 11,76 | 1.44 (0.44-4.67) | 0.54 | 1.58 (0.78-3.21) | 0.21 | . | . | . |
| DNMT3A ---- tag | rs13401241 | A/A | 22 | 36,67 | 7 | 21,88 | 95 | 27,86 | 85 | 27,78 | 1.00 (.-.) | . | 2.74 (1.07-7.04) | 0.04 | 0.69 | 0.99 | 0.98 |
| DNMT3A ---- tag |  | A/C | 24 | 40,00 | 19 | 59,38 | 172 | 50,44 | 164 | 53,59 | 2.67 (0.96-7.41) | 0.06 | 2.10 (0.83-5.29) | 0.12 | . | . | . |
| DNMT3A ---- tag |  | C/C | 14 | 23,33 | 6 | 18,75 | 74 | 21,70 | 57 | 18,63 | 1.00 (0.26-3.81) | 1.00 | 2.47 (0.95-6.42) | 0.06 | . | . | . |
| DNMT3A ---- candidate literature | rs13420827 | C/C | 42 | 70,00 | 21 | 65,63 | 208 | 61,00 | 210 | 68,63 | 1.00 (.-.) | . | 1.66 (0.94-2.92) | 0.08 | 0.40 | 0.99 | 0.98 |
| DNMT3A ---- candidate literature |  | C/G | 13 | 21,67 | 8 | 25,00 | 120 | 35,19 | 86 | 28,10 | 1.51 (0.62-3.68) | 0.37 | 1.35 (0.74-2.44) | 0.33 | . | . | . |
| DNMT3A ---- candidate literature |  | G/G | 5 | 8,33 | 3 | 9,38 | 13 | 3,81 | 10 | 3,27 | 0.92 (0.26-3.34) | 0.90 | 1.18 (0.50-2.80) | 0.71 | . | . | . |
| DNMT3A ---- tag | rs13428812 | A/A | 30 | 50,00 | 15 | 46,88 | 172 | 50,44 | 135 | 44,12 | 1.00 (.-.) | . | 1.81 (0.94-3.50) | 0.08 | 0.45 | 0.99 | 0.98 |
| DNMT3A ---- tag |  | A/G | 22 | 36,67 | 15 | 46,88 | 141 | 41,35 | 146 | 47,71 | 2.18 (0.95-5.02) | 0.07 | 2.14 (1.10-4.15) | 0.03 | . | . | . |
| DNMT3A ---- tag |  | G/G | 8 | 13,33 | 2 | 6,25 | 28 | 8,21 | 25 | 8,17 | 0.83 (0.10-6.55) | 0.86 | 1.85 (0.82-4.13) | 0.14 | . | . | . |
| DNMT3A ---- tag | rs4665287 | C/C | 43 | 71,67 | 21 | 65,63 | 213 | 62,46 | 215 | 70,26 | 1.00 (.-.) | . | 1.67 (0.95-2.94) | 0.08 | 0.39 | 0.99 | 0.98 |
| DNMT3A ---- tag |  | C/T | 12 | 20,00 | 8 | 25,00 | 114 | 33,43 | 81 | 26,47 | 1.56 (0.64-3.80) | 0.33 | 1.32 (0.73-2.40) | 0.36 | . | . | . |
| DNMT3A ---- tag |  | T/T | 5 | 8,33 | 3 | 9,38 | 14 | 4,11 | 10 | 3,27 | 0.87 (0.24-3.15) | 0.84 | 1.17 (0.49-2.76) | 0.73 | . | . | . |
| DNMT3B ---- tag | rs13045669 | A/A | 56 | 93,33 | 29 | 90,63 | 314 | 92,08 | 285 | 93,14 | 1.00 (.-.) | . | 1.38 (0.88-2.16) | 0.16 | 0.79 | 0.99 | 0.97 |
| DNMT3B ---- tag |  | A/G | 4 | 6,67 | 3 | 9,38 | 27 | 7,92 | 20 | 6,54 | 0.81 (0.18-3.55) | 0.78 | 0.90 (0.44-1.83) | 0.77 | . | . | . |
| DNMT3B ---- tag |  | G/G | 0 | 0,00 | 0 | 0,00 | 0 | 0,00 | 1 | 0,33 | 1.00 (.-.) | . | 1.38 (0.88-2.16) | 0.16 | . | . | . |
| DNMT3B ---- tag | rs17123673 | A/A | 54 | 90,00 | 29 | 90,63 | 313 | 91,79 | 282 | 92,16 | 1.00 (.-.) | . | 1.42 (0.90-2.24) | 0.14 | 0.89 | 1.00 | 0.97 |
| DNMT3B ---- tag |  | A/G | 6 | 10,00 | 3 | 9,38 | 28 | 8,21 | 22 | 7,19 | 1.11 (0.33-3.76) | 0.87 | 1.17 (0.61-2.24) | 0.64 | . | . | . |
| DNMT3B ---- tag |  | G/G | 0 | 0,00 | 0 | 0,00 | 0 | 0,00 | 2 | 0,65 | 3.43 (0.80-14.84) | 0.10 | 4.86 (1.05-22.57) | 0.04 | . | . | . |
| DNMT3B ---- tag | rs183603 | A/A | 30 | 50,00 | 20 | 62,50 | 181 | 53,08 | 166 | 54,25 | 1.00 (.-.) | . | 1.02 (0.58-1.77) | 0.95 | 0.33 | 0.99 | 0.74 |
| DNMT3B ---- tag |  | A/G | 23 | 38,33 | 10 | 31,25 | 132 | 38,71 | 125 | 40,85 | 0.46 (0.19-1.09) | 0.08 | 0.98 (0.56-1.72) | 0.96 | . | . | . |
| DNMT3B ---- tag |  | G/G | 7 | 11,67 | 2 | 6,25 | 28 | 8,21 | 15 | 4,90 | 0.60 (0.13-2.76) | 0.52 | 0.53 (0.23-1.20) | 0.13 | . | . | . |
| DNMT3B ---- tag | rs2235760 | C/C | 40 | 66,67 | 22 | 68,75 | 246 | 72,14 | 221 | 72,22 | 1.00 (.-.) | . | 1.49 (0.91-2.46) | 0.12 | 0.78 | 0.99 | 0.97 |
| DNMT3B ---- tag |  | C/T | 18 | 30,00 | 10 | 31,25 | 87 | 25,51 | 74 | 24,18 | 1.37 (0.56-3.37) | 0.49 | 1.29 (0.74-2.25) | 0.36 | . | . | . |
| DNMT3B ---- tag |  | T/T | 2 | 3,33 | 0 | 0,00 | 8 | 2,35 | 11 | 3,59 | 0.00 (0.00-7E271) | 0.97 | 2.22 (1.00-4.94) | 0.05 | . | . | . |
| DNMT3B ---- tag | rs2424908 | C/C | 38 | 63,33 | 22 | 68,75 | 212 | 62,17 | 196 | 64,05 | 1.00 (.-.) | . | 1.15 (0.68-1.93) | 0.60 | 0.37 | 0.99 | 0.74 |
| DNMT3B ---- tag |  | C/T | 18 | 30,00 | 10 | 31,25 | 109 | 31,96 | 101 | 33,01 | 0.56 (0.24-1.31) | 0.18 | 1.13 (0.65-1.94) | 0.67 | . | . | . |
| DNMT3B ---- tag |  | T/T | 4 | 6,67 | 0 | 0,00 | 20 | 5,87 | 9 | 2,94 | 0.00 (0.00-I) | 0.98 | 0.43 (0.17-1.10) | 0.08 | . | . | . |
| DNMT3B ---- candidate literature | rs2424909 | T/T | 19 | 31,67 | 12 | 37,50 | 128 | 37,54 | 129 | 42,16 | 1.00 (.-.) | . | 1.11 (0.59-2.10) | 0.74 | 0.11 | 0.99 | 0.65 |
| DNMT3B ---- candidate literature |  | T/C | 27 | 45,00 | 20 | 62,50 | 160 | 46,92 | 127 | 41,50 | 0.74 (0.33-1.67) | 0.47 | 0.93 (0.49-1.77) | 0.83 | . | . | . |
| DNMT3B ---- candidate literature |  | C/C | 14 | 23,33 | 0 | 0,00 | 53 | 15,54 | 50 | 16,34 | 0.00 (0.00-2E261) | 0.97 | 0.96 (0.48-1.90) | 0.91 | . | . | . |
| DNMT3B ---- tag | rs4911108 | A/A | 20 | 33,33 | 13 | 40,63 | 138 | 40,47 | 135 | 44,12 | 1.00 (.-.) | . | 1.11 (0.59-2.10) | 0.74 | 0.13 | 0.99 | 0.65 |
| DNMT3B ---- tag |  | A/G | 27 | 45,00 | 19 | 59,38 | 153 | 44,87 | 125 | 40,85 | 0.76 (0.33-1.71) | 0.50 | 0.98 (0.52-1.87) | 0.96 | . | . | . |
| DNMT3B ---- tag |  | G/G | 13 | 21,67 | 0 | 0,00 | 50 | 14,66 | 46 | 15,03 | 0.00 (0.00-2E267) | 0.97 | 0.91 (0.46-1.82) | 0.79 | . | . | . |
| DNMT3B ---- tag | rs6058896 | C/C | 53 | 88,33 | 28 | 87,50 | 304 | 89,15 | 273 | 89,22 | 1.00 (.-.) | . | 1.37 (0.87-2.15) | 0.17 | 0.97 | 1.00 | 0.97 |
| DNMT3B ---- tag |  | C/T | 6 | 10,00 | 3 | 9,38 | 36 | 10,56 | 32 | 10,46 | 1.65 (0.38-7.13) | 0.50 | 1.76 (0.98-3.14) | 0.06 | . | . | . |
| DNMT3B ---- tag |  | T/T | 1 | 1,67 | 1 | 3,13 | 1 | 0,29 | 1 | 0,33 | 0.00 (0.00-1E299) | 0.98 | 3.38 (0.44-26.26) | 0.24 | . | . | . |
| DNMT3B ---- tag | rs6119954 | G/G | 36 | 60,00 | 24 | 75,00 | 242 | 70,97 | 220 | 71,90 | 1.00 (.-.) | . | 1.27 (0.78-2.08) | 0.33 | 0.31 | 0.99 | 0.74 |
| DNMT3B ---- tag |  | G/A | 21 | 35,00 | 8 | 25,00 | 89 | 26,10 | 73 | 23,86 | 0.78 (0.31-1.99) | 0.61 | 1.11 (0.65-1.91) | 0.70 | . | . | . |
| DNMT3B ---- tag |  | A/A | 3 | 5,00 | 0 | 0,00 | 10 | 2,93 | 13 | 4,25 | 0.00 (0.00-I) | 0.98 | 2.04 (0.96-4.31) | 0.06 | . | . | . |
| DNMT3B ---- tag | rs6579038 | A/A | 54 | 90,00 | 29 | 90,63 | 301 | 88,27 | 273 | 89,22 | 1.00 (.-.) | . | 1.39 (0.89-2.17) | 0.15 | 0.70 | 0.99 | 0.97 |
| DNMT3B ---- tag |  | A/G | 6 | 10,00 | 3 | 9,38 | 39 | 11,44 | 32 | 10,46 | 1.65 (0.38-7.12) | 0.50 | 1.64 (0.91-2.93) | 0.10 | . | . | . |
| DNMT3B ---- tag |  | G/G | 0 | 0,00 | 0 | 0,00 | 1 | 0,29 | 1 | 0,33 | 2.45 (0.33-18.41) | 0.38 | 3.40 (0.44-26.35) | 0.24 | . | . | . |
| DPYD ---- tag | rs1034215 | C/C | 38 | 63,33 | 19 | 59,38 | 194 | 56,89 | 188 | 61,44 | 1.00 (.-.) | . | 1.87 (1.03-3.39) | 0.04 | 0.18 | 0.99 | 0.72 |
| DPYD ---- tag |  | C/T | 20 | 33,33 | 13 | 40,63 | 129 | 37,83 | 97 | 31,70 | 1.87 (0.83-4.22) | 0.13 | 1.58 (0.85-2.92) | 0.15 | . | . | . |
| DPYD ---- tag |  | T/T | 2 | 3,33 | 0 | 0,00 | 18 | 5,28 | 21 | 6,86 | 0.00 (0.00-9E226) | 0.97 | 2.41 (1.11-5.25) | 0.03 | . | . | . |
| DPYD ---- tag | rs10783058 | T/T | 31 | 51,67 | 17 | 53,13 | 135 | 39,59 | 118 | 38,56 | 1.00 (.-.) | . | 1.13 (0.61-2.12) | 0.69 | 0.50 | 0.99 | 0.77 |
| DPYD ---- tag |  | T/C | 22 | 36,67 | 11 | 34,38 | 165 | 48,39 | 147 | 48,04 | 0.89 (0.36-2.15) | 0.79 | 1.41 (0.76-2.63) | 0.28 | . | . | . |
| DPYD ---- tag |  | C/C | 7 | 11,67 | 4 | 12,50 | 41 | 12,02 | 41 | 13,40 | 0.90 (0.28-2.89) | 0.86 | 1.40 (0.72-2.74) | 0.32 | . | . | . |
| DPYD ---- tag | rs10783070 | C/C | 43 | 71,67 | 27 | 84,38 | 252 | 73,90 | 205 | 66,99 | 1.00 (.-.) | . | 1.20 (0.74-1.95) | 0.47 | 0.64 | 0.99 | 0.84 |
| DPYD ---- tag |  | C/T | 16 | 26,67 | 3 | 9,38 | 82 | 24,05 | 93 | 30,39 | 0.62 (0.18-2.13) | 0.45 | 1.64 (0.98-2.74) | 0.06 | . | . | . |
| DPYD ---- tag |  | T/T | 1 | 1,67 | 2 | 6,25 | 7 | 2,05 | 8 | 2,61 | 2.46 (0.55-10.92) | 0.24 | 2.07 (0.81-5.29) | 0.13 | . | . | . |
| DPYD ---- tag | rs10875048 | G/G | 41 | 68,33 | 22 | 68,75 | 241 | 70,67 | 201 | 65,69 | 1.00 (.-.) | . | 1.22 (0.71-2.10) | 0.47 | 0.20 | 0.99 | 0.72 |
| DPYD ---- tag |  | G/A | 14 | 23,33 | 9 | 28,13 | 94 | 27,57 | 96 | 31,37 | 0.99 (0.43-2.28) | 0.98 | 1.22 (0.69-2.15) | 0.49 | . | . | . |
| DPYD ---- tag |  | A/A | 5 | 8,33 | 1 | 3,13 | 6 | 1,76 | 9 | 2,94 | 0.29 (0.04-2.26) | 0.24 | 2.73 (1.14-6.55) | 0.03 | . | . | . |
| DPYD ---- tag | rs10875055 | C/C | 30 | 50,00 | 7 | 21,88 | 96 | 28,15 | 64 | 20,92 | 1.00 (.-.) | . | 1.60 (0.72-3.57) | 0.25 | 0.35 | 0.99 | 0.77 |
| DPYD ---- tag |  | C/T | 24 | 40,00 | 18 | 56,25 | 177 | 51,91 | 162 | 52,94 | 1.87 (0.74-4.73) | 0.18 | 2.32 (1.07-5.01) | 0.03 | . | . | . |
| DPYD ---- tag |  | T/T | 6 | 10,00 | 7 | 21,88 | 68 | 19,94 | 80 | 26,14 | 2.66 (0.81-8.68) | 0.11 | 2.54 (1.15-5.63) | 0.02 | . | . | . |
| DPYD ---- tag | rs10875079 | A/A | 9 | 15,00 | 10 | 31,25 | 88 | 25,81 | 90 | 29,41 | 1.00 (.-.) | . | 1.44 (0.56-3.70) | 0.45 | 0.88 | 1.00 | 0.93 |
| DPYD ---- tag |  | A/G | 38 | 63,33 | 16 | 50,00 | 164 | 48,09 | 150 | 49,02 | 0.96 (0.34-2.72) | 0.94 | 1.20 (0.47-3.04) | 0.70 | . | . | . |
| DPYD ---- tag |  | G/G | 13 | 21,67 | 6 | 18,75 | 89 | 26,10 | 66 | 21,57 | 0.62 (0.17-2.22) | 0.47 | 0.99 (0.38-2.56) | 0.99 | . | . | . |
| DPYD ---- tag | rs10875085 | A/A | 44 | 73,33 | 19 | 59,38 | 253 | 74,19 | 192 | 62,75 | 1.00 (.-.) | . | 1.60 (0.91-2.81) | 0.10 | 0.50 | 0.99 | 0.77 |
| DPYD ---- tag |  | A/T | 14 | 23,33 | 12 | 37,50 | 81 | 23,75 | 101 | 33,01 | 1.84 (0.82-4.15) | 0.14 | 1.78 (0.99-3.19) | 0.06 | . | . | . |
| DPYD ---- tag |  | T/T | 2 | 3,33 | 1 | 3,13 | 7 | 2,05 | 13 | 4,25 | 0.00 (0.00-I) | 0.98 | 2.42 (1.07-5.47) | 0.03 | . | . | . |
| DPYD ---- tag | rs10875097 | G/G | 43 | 71,67 | 19 | 59,38 | 236 | 69,21 | 203 | 66,34 | 1.00 (.-.) | . | 1.92 (1.07-3.43) | 0.03 | 0.03 | 0.99 | 0.55 |
| DPYD ---- tag |  | G/A | 17 | 28,33 | 12 | 37,50 | 93 | 27,27 | 92 | 30,07 | 2.57 (1.14-5.81) | 0.02 | 2.02 (1.10-3.71) | 0.02 | . | . | . |
| DPYD ---- tag |  | A/A | 0 | 0,00 | 1 | 3,13 | 12 | 3,52 | 11 | 3,59 | 4.48 (0.55-36.67) | 0.16 | 1.96 (0.79-4.85) | 0.14 | . | . | . |
| DPYD ---- tag | rs11165781 | T/T | 41 | 68,33 | 24 | 75,00 | 244 | 71,55 | 197 | 64,38 | 1.00 (.-.) | . | 1.10 (0.70-1.75) | 0.67 | 0.08 | 0.99 | 0.72 |
| DPYD ---- tag |  | T/C | 18 | 30,00 | 7 | 21,88 | 91 | 26,69 | 101 | 33,01 | 0.21 (0.03-1.56) | 0.13 | 1.35 (0.83-2.19) | 0.23 | . | . | . |
| DPYD ---- tag |  | C/C | 1 | 1,67 | 1 | 3,13 | 6 | 1,76 | 8 | 2,61 | 0.90 (0.12-6.87) | 0.92 | 2.01 (0.67-6.02) | 0.21 | . | . | . |
| DPYD ---- tag | rs11165783 | T/T | 31 | 51,67 | 20 | 62,50 | 184 | 53,96 | 171 | 55,88 | 1.00 (.-.) | . | 1.54 (0.86-2.78) | 0.15 | 0.53 | 0.99 | 0.77 |
| DPYD ---- tag |  | T/C | 25 | 41,67 | 11 | 34,38 | 130 | 38,12 | 107 | 34,97 | 1.16 (0.51-2.62) | 0.73 | 1.43 (0.78-2.60) | 0.25 | . | . | . |
| DPYD ---- tag |  | C/C | 4 | 6,67 | 1 | 3,13 | 27 | 7,92 | 28 | 9,15 | 1.60 (0.21-12.48) | 0.65 | 1.40 (0.69-2.85) | 0.35 | . | . | . |
| DPYD ---- tag | rs11165873 | A/A | 12 | 20,00 | 9 | 28,13 | 93 | 27,27 | 94 | 30,72 | 1.00 (.-.) | . | 1.31 (0.59-2.91) | 0.51 | 0.54 | 0.99 | 0.77 |
| DPYD ---- tag |  | A/T | 28 | 46,67 | 16 | 50,00 | 177 | 51,91 | 150 | 49,02 | 0.97 (0.37-2.54) | 0.96 | 1.09 (0.49-2.41) | 0.83 | . | . | . |
| DPYD ---- tag |  | T/T | 20 | 33,33 | 7 | 21,88 | 71 | 20,82 | 62 | 20,26 | 0.61 (0.20-1.88) | 0.39 | 1.21 (0.53-2.77) | 0.65 | . | . | . |
| DPYD ---- tag | rs11165875 | T/T | 27 | 45,00 | 13 | 40,63 | 141 | 41,35 | 117 | 38,24 | 1.00 (.-.) | . | 1.66 (0.87-3.17) | 0.13 | 0.43 | 0.99 | 0.77 |
| DPYD ---- tag |  | T/C | 29 | 48,33 | 12 | 37,50 | 160 | 46,92 | 141 | 46,08 | 1.17 (0.47-2.88) | 0.74 | 1.47 (0.78-2.77) | 0.24 | . | . | . |
| DPYD ---- tag |  | C/C | 4 | 6,67 | 7 | 21,88 | 40 | 11,73 | 48 | 15,69 | 1.75 (0.59-5.25) | 0.32 | 1.85 (0.92-3.71) | 0.08 | . | . | . |
| DPYD ---- tag | rs11165881 | T/T | 24 | 40,00 | 15 | 46,88 | 112 | 32,84 | 107 | 34,97 | 1.00 (.-.) | . | 1.06 (0.54-2.09) | 0.86 | 0.72 | 0.99 | 0.84 |
| DPYD ---- tag |  | T/C | 26 | 43,33 | 13 | 40,63 | 161 | 47,21 | 147 | 48,04 | 0.71 (0.29-1.73) | 0.45 | 1.33 (0.68-2.58) | 0.41 | . | . | . |
| DPYD ---- tag |  | C/C | 10 | 16,67 | 4 | 12,50 | 68 | 19,94 | 52 | 16,99 | 1.10 (0.33-3.62) | 0.88 | 1.17 (0.57-2.38) | 0.67 | . | . | . |
| DPYD ---- tag | rs11587873 | C/C | 33 | 55,00 | 22 | 68,75 | 186 | 54,55 | 188 | 61,44 | 1.00 (.-.) | . | 1.08 (0.64-1.85) | 0.77 | 0.41 | 0.99 | 0.77 |
| DPYD ---- tag |  | C/T | 23 | 38,33 | 9 | 28,13 | 124 | 36,36 | 110 | 35,95 | 0.48 (0.21-1.14) | 0.10 | 1.03 (0.59-1.79) | 0.93 | . | . | . |
| DPYD ---- tag |  | T/T | 4 | 6,67 | 1 | 3,13 | 31 | 9,09 | 8 | 2,61 | 0.99 (0.13-7.60) | 0.99 | 0.45 (0.17-1.17) | 0.10 | . | . | . |
| DPYD ---- tag | rs12030174 | C/C | 46 | 76,67 | 20 | 62,50 | 266 | 78,01 | 205 | 66,99 | 1.00 (.-.) | . | 1.57 (0.91-2.71) | 0.10 | 0.55 | 0.99 | 0.77 |
| DPYD ---- tag |  | C/T | 13 | 21,67 | 11 | 34,38 | 69 | 20,23 | 91 | 29,74 | 1.73 (0.76-3.94) | 0.19 | 1.64 (0.92-2.92) | 0.09 | . | . | . |
| DPYD ---- tag |  | T/T | 1 | 1,67 | 1 | 3,13 | 6 | 1,76 | 10 | 3,27 | 0.00 (0.00-I) | 0.98 | 2.61 (1.08-6.30) | 0.03 | . | . | . |
| DPYD ---- tag | rs12046744 | A/A | 32 | 53,33 | 17 | 53,13 | 181 | 53,08 | 176 | 57,52 | 1.00 (.-.) | . | 1.80 (1.01-3.23) | 0.05 | 0.21 | 0.99 | 0.72 |
| DPYD ---- tag |  | A/C | 24 | 40,00 | 12 | 37,50 | 129 | 37,83 | 110 | 35,95 | 1.95 (0.86-4.41) | 0.11 | 1.77 (0.97-3.24) | 0.07 | . | . | . |
| DPYD ---- tag |  | C/C | 4 | 6,67 | 3 | 9,38 | 31 | 9,09 | 20 | 6,54 | 0.91 (0.12-7.10) | 0.93 | 1.25 (0.59-2.68) | 0.56 | . | . | . |
| DPYD ---- tag | rs12047910 | G/G | 44 | 73,33 | 23 | 71,88 | 258 | 75,66 | 223 | 72,88 | 1.00 (.-.) | . | 1.38 (0.82-2.35) | 0.23 | 0.95 | 1.00 | 0.97 |
| DPYD ---- tag |  | G/A | 15 | 25,00 | 8 | 25,00 | 79 | 23,17 | 77 | 25,16 | 0.97 (0.41-2.31) | 0.95 | 1.33 (0.76-2.34) | 0.32 | . | . | . |
| DPYD ---- tag |  | A/A | 1 | 1,67 | 1 | 3,13 | 4 | 1,17 | 6 | 1,96 | 2.10 (0.27-16.40) | 0.48 | 3.07 (1.17-8.05) | 0.02 | . | . | . |
| DPYD ---- tag | rs12073044 | T/T | 52 | 86,67 | 27 | 84,38 | 261 | 76,54 | 240 | 78,43 | 1.00 (.-.) | . | 1.42 (0.89-2.28) | 0.15 | 0.75 | 0.99 | 0.84 |
| DPYD ---- tag |  | T/A | 8 | 13,33 | 5 | 15,63 | 76 | 22,29 | 65 | 21,24 | 1.08 (0.36-3.25) | 0.89 | 1.31 (0.77-2.23) | 0.31 | . | . | . |
| DPYD ---- tag |  | A/A | 0 | 0,00 | 0 | 0,00 | 4 | 1,17 | 1 | 0,33 | 0.00 (0.00-7E222) | 0.97 | 0.00 (0.00-1E223) | 0.97 | . | . | . |
| DPYD ---- tag | rs12126093 | T/T | 23 | 38,33 | 14 | 43,75 | 174 | 51,03 | 164 | 53,59 | 1.00 (.-.) | . | 1.30 (0.69-2.45) | 0.42 | 0.82 | 0.99 | 0.90 |
| DPYD ---- tag |  | T/C | 31 | 51,67 | 14 | 43,75 | 142 | 41,64 | 117 | 38,24 | 0.86 (0.37-2.02) | 0.73 | 1.17 (0.62-2.22) | 0.63 | . | . | . |
| DPYD ---- tag |  | C/C | 6 | 10,00 | 4 | 12,50 | 25 | 7,33 | 25 | 8,17 | 0.76 (0.20-2.80) | 0.67 | 1.19 (0.55-2.56) | 0.66 | . | . | . |
| DPYD ---- tag | rs12134028 | C/C | 51 | 85,00 | 30 | 93,75 | 309 | 90,62 | 273 | 89,22 | 1.00 (.-.) | . | 1.09 (0.70-1.70) | 0.70 | 0.02 | 0.99 | 0.47 |
| DPYD ---- tag |  | C/T | 9 | 15,00 | 1 | 3,13 | 32 | 9,38 | 32 | 10,46 | 0.13 (0.02-0.97) | 0.05 | 0.96 (0.53-1.72) | 0.88 | . | . | . |
| DPYD ---- tag |  | T/T | 0 | 0,00 | 1 | 3,13 | 0 | 0,00 | 1 | 0,33 | 0.98 (0.13-7.22) | 0.98 | 1.07 (0.14-8.23) | 0.95 | . | . | . |
| DPYD ---- tag | rs12740796 | T/T | 41 | 68,33 | 24 | 75,00 | 253 | 74,19 | 235 | 76,80 | 1.00 (.-.) | . | 1.55 (0.94-2.55) | 0.09 | 0.27 | 0.99 | 0.77 |
| DPYD ---- tag |  | T/C | 18 | 30,00 | 6 | 18,75 | 82 | 24,05 | 65 | 21,24 | 1.34 (0.49-3.69) | 0.57 | 1.43 (0.83-2.48) | 0.20 | . | . | . |
| DPYD ---- tag |  | C/C | 1 | 1,67 | 2 | 6,25 | 6 | 1,76 | 6 | 1,96 | 2.33 (0.53-10.26) | 0.26 | 1.80 (0.65-4.99) | 0.26 | . | . | . |
| DPYD ---- tag | rs1333717 | A/A | 37 | 61,67 | 18 | 56,25 | 187 | 54,84 | 178 | 58,17 | 1.00 (.-.) | . | 1.85 (1.02-3.36) | 0.04 | 0.20 | 0.99 | 0.72 |
| DPYD ---- tag |  | A/G | 20 | 33,33 | 14 | 43,75 | 135 | 39,59 | 106 | 34,64 | 1.84 (0.81-4.14) | 0.14 | 1.60 (0.87-2.96) | 0.13 | . | . | . |
| DPYD ---- tag |  | G/G | 3 | 5,00 | 0 | 0,00 | 19 | 5,57 | 22 | 7,19 | 0.00 (0.00-7E226) | 0.97 | 2.34 (1.09-5.04) | 0.03 | . | . | . |
| DPYD ---- tag | rs1413228 | A/A | 49 | 81,67 | 28 | 87,50 | 275 | 80,65 | 237 | 77,45 | 1.00 (.-.) | . | 1.26 (0.80-1.99) | 0.31 | 0.53 | 0.99 | 0.77 |
| DPYD ---- tag |  | A/G | 11 | 18,33 | 3 | 9,38 | 61 | 17,89 | 65 | 21,24 | 0.32 (0.04-2.44) | 0.27 | 1.38 (0.82-2.32) | 0.22 | . | . | . |
| DPYD ---- tag |  | G/G | 0 | 0,00 | 1 | 3,13 | 5 | 1,47 | 4 | 1,31 | 1.46 (0.19-11.39) | 0.72 | 1.00 (0.23-4.34) | 1.00 | . | . | . |
| DPYD ---- tag | rs1415681 | G/G | 46 | 76,67 | 26 | 81,25 | 240 | 70,38 | 232 | 75,82 | 1.00 (.-.) | . | 1.24 (0.76-2.02) | 0.39 | 0.42 | 0.99 | 0.77 |
| DPYD ---- tag |  | G/T | 13 | 21,67 | 6 | 18,75 | 94 | 27,57 | 64 | 20,92 | 0.73 (0.27-1.98) | 0.54 | 1.20 (0.70-2.05) | 0.52 | . | . | . |
| DPYD ---- tag |  | T/T | 1 | 1,67 | 0 | 0,00 | 7 | 2,05 | 10 | 3,27 | 1.94 (0.97-3.90) | 0.06 | 2.41 (1.09-5.35) | 0.03 | . | . | . |
| DPYD ---- tag | rs1514495 | C/C | 31 | 51,67 | 19 | 59,38 | 217 | 63,64 | 179 | 58,50 | 1.00 (.-.) | . | 1.14 (0.68-1.92) | 0.62 | 0.34 | 0.99 | 0.77 |
| DPYD ---- tag |  | C/T | 23 | 38,33 | 10 | 31,25 | 102 | 29,91 | 111 | 36,27 | 0.56 (0.21-1.53) | 0.26 | 1.11 (0.65-1.90) | 0.70 | . | . | . |
| DPYD ---- tag |  | T/T | 6 | 10,00 | 3 | 9,38 | 22 | 6,45 | 16 | 5,23 | 0.59 (0.13-2.62) | 0.48 | 0.95 (0.44-2.09) | 0.91 | . | . | . |
| DPYD ---- tag | rs1520658 | A/A | 46 | 76,67 | 25 | 78,13 | 280 | 82,11 | 251 | 82,03 | 1.00 (.-.) | . | 1.51 (0.92-2.47) | 0.10 | 0.21 | 0.99 | 0.72 |
| DPYD ---- tag |  | A/G | 14 | 23,33 | 6 | 18,75 | 55 | 16,13 | 52 | 16,99 | 1.27 (0.47-3.45) | 0.64 | 1.47 (0.85-2.55) | 0.17 | . | . | . |
| DPYD ---- tag |  | G/G | 0 | 0,00 | 1 | 3,13 | 6 | 1,76 | 3 | 0,98 | 8.92 (1.07-74.01) | 0.04 | 0.83 (0.19-3.66) | 0.81 | . | . | . |
| DPYD ---- NA | rs17116806 | C/C | 36 | 60,00 | 19 | 59,38 | 228 | 66,86 | 193 | 63,07 | 1.00 (.-.) | . | 1.55 (0.86-2.79) | 0.14 | 0.43 | 0.99 | 0.77 |
| DPYD ---- NA |  | C/A | 23 | 38,33 | 12 | 37,50 | 94 | 27,57 | 101 | 33,01 | 1.37 (0.61-3.10) | 0.45 | 1.75 (0.95-3.21) | 0.07 | . | . | . |
| DPYD ---- NA |  | A/A | 1 | 1,67 | 1 | 3,13 | 19 | 5,57 | 12 | 3,92 | 1.97 (0.25-15.39) | 0.52 | 1.33 (0.56-3.18) | 0.52 | . | . | . |
| DPYD ---- tag | rs17431828 | G/G | 24 | 40,00 | 14 | 43,75 | 131 | 38,42 | 138 | 45,10 | 1.00 (.-.) | . | 1.21 (0.62-2.37) | 0.58 | 0.73 | 0.99 | 0.84 |
| DPYD ---- tag |  | G/C | 28 | 46,67 | 13 | 40,63 | 162 | 47,51 | 133 | 43,46 | 0.70 (0.29-1.72) | 0.44 | 1.33 (0.68-2.61) | 0.41 | . | . | . |
| DPYD ---- tag |  | C/C | 8 | 13,33 | 5 | 15,63 | 48 | 14,08 | 35 | 11,44 | 1.40 (0.47-4.17) | 0.55 | 0.99 (0.46-2.13) | 0.98 | . | . | . |
| DPYD ---- tag | rs17471640 | T/T | 24 | 40,00 | 11 | 34,38 | 141 | 41,35 | 152 | 49,67 | 1.00 (.-.) | . | 1.92 (0.92-4.00) | 0.08 | 0.14 | 0.99 | 0.72 |
| DPYD ---- tag |  | T/C | 29 | 48,33 | 15 | 46,88 | 171 | 50,15 | 126 | 41,18 | 1.30 (0.52-3.23) | 0.58 | 1.57 (0.75-3.30) | 0.23 | . | . | . |
| DPYD ---- tag |  | C/C | 7 | 11,67 | 6 | 18,75 | 29 | 8,50 | 28 | 9,15 | 2.03 (0.65-6.33) | 0.22 | 1.71 (0.73-4.03) | 0.22 | . | . | . |
| DPYD ---- tag | rs17702702 | G/G | 38 | 63,33 | 21 | 65,63 | 235 | 68,91 | 217 | 70,92 | 1.00 (.-.) | . | 1.86 (1.08-3.22) | 0.03 | 0.07 | 0.99 | 0.72 |
| DPYD ---- tag |  | G/C | 19 | 31,67 | 9 | 28,13 | 97 | 28,45 | 78 | 25,49 | 2.30 (0.95-5.56) | 0.06 | 1.58 (0.89-2.80) | 0.12 | . | . | . |
| DPYD ---- tag |  | C/C | 3 | 5,00 | 2 | 6,25 | 9 | 2,64 | 11 | 3,59 | 2.06 (0.46-9.19) | 0.35 | 1.88 (0.83-4.29) | 0.13 | . | . | . |
| DPYD ---- NA | rs1801265 | T/T | 34 | 56,67 | 22 | 68,75 | 197 | 57,77 | 177 | 57,84 | 1.00 (.-.) | . | 1.38 (0.82-2.32) | 0.23 | 0.96 | 1.00 | 0.97 |
| DPYD ---- NA |  | T/C | 23 | 38,33 | 10 | 31,25 | 122 | 35,78 | 111 | 36,27 | 1.10 (0.47-2.59) | 0.82 | 1.44 (0.84-2.46) | 0.18 | . | . | . |
| DPYD ---- NA |  | C/C | 3 | 5,00 | 0 | 0,00 | 22 | 6,45 | 18 | 5,88 | 0.00 (0.00-4E261) | 0.97 | 1.31 (0.63-2.74) | 0.47 | . | . | . |
| DPYD ---- tag | rs2039447 | T/T | 26 | 43,33 | 19 | 59,38 | 160 | 46,92 | 150 | 49,02 | 1.00 (.-.) | . | 1.50 (0.85-2.65) | 0.16 | 0.59 | 0.99 | 0.80 |
| DPYD ---- tag |  | T/C | 24 | 40,00 | 8 | 25,00 | 147 | 43,11 | 132 | 43,14 | 1.07 (0.40-2.83) | 0.89 | 1.40 (0.79-2.48) | 0.25 | . | . | . |
| DPYD ---- tag |  | C/C | 10 | 16,67 | 5 | 15,63 | 34 | 9,97 | 24 | 7,84 | 1.12 (0.40-3.17) | 0.83 | 1.21 (0.59-2.49) | 0.61 | . | . | . |
| DPYD ---- tag | rs2151567 | G/G | 53 | 88,33 | 30 | 93,75 | 312 | 91,50 | 274 | 89,54 | 1.00 (.-.) | . | 1.38 (0.88-2.17) | 0.16 | 0.98 | 1.00 | 0.98 |
| DPYD ---- tag |  | G/A | 7 | 11,67 | 2 | 6,25 | 28 | 8,21 | 31 | 10,13 | 1.32 (0.31-5.67) | 0.71 | 1.69 (0.91-3.15) | 0.10 | . | . | . |
| DPYD ---- tag |  | A/A | 0 | 0,00 | 0 | 0,00 | 1 | 0,29 | 1 | 0,33 | 4.61 (0.61-34.78) | 0.14 | 6.38 (0.81-50.54) | 0.08 | . | . | . |
| DPYD ---- tag | rs2152878 | A/A | 30 | 50,00 | 21 | 65,63 | 186 | 54,55 | 184 | 60,13 | 1.00 (.-.) | . | 1.18 (0.70-1.99) | 0.54 | 0.46 | 0.99 | 0.77 |
| DPYD ---- tag |  | A/G | 27 | 45,00 | 6 | 18,75 | 132 | 38,71 | 105 | 34,31 | 0.59 (0.23-1.54) | 0.29 | 1.15 (0.67-2.00) | 0.61 | . | . | . |
| DPYD ---- tag |  | G/G | 3 | 5,00 | 5 | 15,63 | 23 | 6,74 | 17 | 5,56 | 0.93 (0.20-4.23) | 0.92 | 1.29 (0.62-2.71) | 0.50 | . | . | . |
| DPYD ---- tag | rs2786505 | G/G | 46 | 76,67 | 27 | 84,38 | 259 | 75,95 | 221 | 72,22 | 1.00 (.-.) | . | 1.26 (0.77-2.05) | 0.35 | 0.51 | 0.99 | 0.77 |
| DPYD ---- tag |  | G/T | 14 | 23,33 | 5 | 15,63 | 79 | 23,17 | 80 | 26,14 | 0.92 (0.34-2.48) | 0.87 | 1.57 (0.93-2.65) | 0.09 | . | . | . |
| DPYD ---- tag |  | T/T | 0 | 0,00 | 0 | 0,00 | 3 | 0,88 | 5 | 1,63 | 2.07 (0.75-5.66) | 0.16 | 2.60 (0.87-7.79) | 0.09 | . | . | . |
| DPYD ---- tag | rs2786512 | G/G | 19 | 31,67 | 14 | 43,75 | 129 | 37,83 | 111 | 36,27 | 1.00 (.-.) | . | 1.05 (0.57-1.97) | 0.87 | 0.10 | 0.99 | 0.72 |
| DPYD ---- tag |  | G/A | 26 | 43,33 | 15 | 46,88 | 150 | 43,99 | 144 | 47,06 | 1.02 (0.43-2.40) | 0.96 | 1.20 (0.65-2.21) | 0.57 | . | . | . |
| DPYD ---- tag |  | A/A | 15 | 25,00 | 3 | 9,38 | 62 | 18,18 | 51 | 16,67 | 0.34 (0.09-1.22) | 0.10 | 1.13 (0.58-2.20) | 0.71 | . | . | . |
| DPYD ---- tag | rs2786519 | A/A | 39 | 65,00 | 17 | 53,13 | 213 | 62,46 | 176 | 57,52 | 1.00 (.-.) | . | 1.86 (1.02-3.42) | 0.05 | 0.22 | 0.99 | 0.72 |
| DPYD ---- tag |  | A/G | 18 | 30,00 | 13 | 40,63 | 109 | 31,96 | 112 | 36,60 | 2.55 (1.10-5.92) | 0.03 | 2.14 (1.15-3.99) | 0.02 | . | . | . |
| DPYD ---- tag |  | G/G | 3 | 5,00 | 2 | 6,25 | 19 | 5,57 | 18 | 5,88 | 1.40 (0.30-6.39) | 0.67 | 1.66 (0.74-3.71) | 0.22 | . | . | . |
| DPYD ---- tag | rs2811170 | A/A | 47 | 78,33 | 21 | 65,63 | 243 | 71,26 | 241 | 78,76 | 1.00 (.-.) | . | 1.63 (0.97-2.75) | 0.07 | 0.18 | 0.99 | 0.72 |
| DPYD ---- tag |  | A/T | 13 | 21,67 | 11 | 34,38 | 93 | 27,27 | 63 | 20,59 | 1.35 (0.58-3.15) | 0.49 | 1.17 (0.65-2.09) | 0.60 | . | . | . |
| DPYD ---- tag |  | T/T | 0 | 0,00 | 0 | 0,00 | 5 | 1,47 | 2 | 0,65 | 0.58 (0.08-4.22) | 0.59 | 0.95 (0.12-7.28) | 0.96 | . | . | . |
| DPYD ---- tag | rs2811199 | G/G | 44 | 73,33 | 27 | 84,38 | 254 | 74,49 | 212 | 69,28 | 1.00 (.-.) | . | 1.24 (0.76-2.02) | 0.39 | 0.50 | 0.99 | 0.77 |
| DPYD ---- tag |  | G/A | 15 | 25,00 | 4 | 12,50 | 81 | 23,75 | 87 | 28,43 | 0.82 (0.28-2.45) | 0.72 | 1.57 (0.93-2.64) | 0.09 | . | . | . |
| DPYD ---- tag |  | A/A | 1 | 1,67 | 1 | 3,13 | 6 | 1,76 | 7 | 2,29 | 1.39 (0.18-10.78) | 0.75 | 2.32 (0.85-6.34) | 0.10 | . | . | . |
| DPYD ---- tag | rs2811219 | T/T | 35 | 58,33 | 19 | 59,38 | 185 | 54,25 | 176 | 57,52 | 1.00 (.-.) | . | 1.45 (0.82-2.56) | 0.20 | 0.75 | 0.99 | 0.84 |
| DPYD ---- tag |  | T/C | 24 | 40,00 | 12 | 37,50 | 135 | 39,59 | 114 | 37,25 | 1.03 (0.45-2.35) | 0.95 | 1.32 (0.74-2.36) | 0.35 | . | . | . |
| DPYD ---- tag |  | C/C | 1 | 1,67 | 1 | 3,13 | 21 | 6,16 | 16 | 5,23 | 1.43 (0.18-11.33) | 0.74 | 1.50 (0.68-3.30) | 0.31 | . | . | . |
| DPYD ---- tag | rs4300257 | A/A | 43 | 71,67 | 17 | 53,13 | 235 | 68,91 | 181 | 59,15 | 1.00 (.-.) | . | 1.83 (1.02-3.29) | 0.04 | 0.41 | 0.99 | 0.77 |
| DPYD ---- tag |  | A/C | 15 | 25,00 | 12 | 37,50 | 97 | 28,45 | 106 | 34,64 | 2.27 (1.00-5.17) | 0.05 | 1.59 (0.87-2.90) | 0.13 | . | . | . |
| DPYD ---- tag |  | C/C | 2 | 3,33 | 3 | 9,38 | 9 | 2,64 | 19 | 6,21 | 1.05 (0.14-8.22) | 0.96 | 4.52 (2.13-9.58) | <.001 | . | . | . |
| DPYD ---- tag | rs4379706 | T/T | 34 | 56,67 | 22 | 68,75 | 191 | 56,01 | 177 | 57,84 | 1.00 (.-.) | . | 1.39 (0.83-2.35) | 0.21 | 0.75 | 0.99 | 0.84 |
| DPYD ---- tag |  | T/C | 22 | 36,67 | 9 | 28,13 | 125 | 36,66 | 111 | 36,27 | 0.99 (0.41-2.43) | 0.99 | 1.44 (0.84-2.47) | 0.18 | . | . | . |
| DPYD ---- tag |  | C/C | 4 | 6,67 | 1 | 3,13 | 25 | 7,33 | 18 | 5,88 | 1.99 (0.25-15.93) | 0.52 | 1.20 (0.58-2.52) | 0.62 | . | . | . |
| DPYD ---- tag | rs4950021 | T/T | 22 | 36,67 | 11 | 34,38 | 106 | 31,09 | 90 | 29,41 | 1.00 (.-.) | . | 1.50 (0.77-2.90) | 0.23 | 0.47 | 0.99 | 0.77 |
| DPYD ---- tag |  | T/G | 25 | 41,67 | 14 | 43,75 | 161 | 47,21 | 143 | 46,73 | 1.05 (0.44-2.49) | 0.92 | 1.54 (0.81-2.92) | 0.19 | . | . | . |
| DPYD ---- tag |  | G/G | 13 | 21,67 | 7 | 21,88 | 74 | 21,70 | 73 | 23,86 | 1.67 (0.52-5.35) | 0.39 | 1.43 (0.73-2.79) | 0.30 | . | . | . |
| DPYD ---- tag | rs4950033 | T/T | 23 | 38,33 | 9 | 28,13 | 92 | 26,98 | 90 | 29,41 | 1.00 (.-.) | . | 1.81 (0.85-3.84) | 0.12 | 0.66 | 0.99 | 0.84 |
| DPYD ---- tag |  | T/C | 27 | 45,00 | 16 | 50,00 | 176 | 51,61 | 143 | 46,73 | 1.31 (0.53-3.25) | 0.56 | 1.45 (0.69-3.01) | 0.33 | . | . | . |
| DPYD ---- tag |  | C/C | 10 | 16,67 | 7 | 21,88 | 73 | 21,41 | 73 | 23,86 | 1.20 (0.38-3.81) | 0.75 | 1.76 (0.83-3.73) | 0.14 | . | . | . |
| DPYD ---- tag | rs495257 | T/T | 23 | 38,33 | 17 | 53,13 | 119 | 34,90 | 94 | 30,72 | 1.00 (.-.) | . | 1.19 (0.65-2.18) | 0.58 | 0.87 | 1.00 | 0.93 |
| DPYD ---- tag |  | T/C | 27 | 45,00 | 8 | 25,00 | 177 | 51,91 | 175 | 57,19 | 0.71 (0.26-1.88) | 0.49 | 1.33 (0.74-2.40) | 0.34 | . | . | . |
| DPYD ---- tag |  | C/C | 10 | 16,67 | 7 | 21,88 | 45 | 13,20 | 37 | 12,09 | 1.07 (0.40-2.88) | 0.89 | 1.20 (0.61-2.34) | 0.59 | . | . | . |
| DPYD ---- tag | rs552926 | A/A | 26 | 43,33 | 15 | 46,88 | 119 | 34,90 | 99 | 32,35 | 1.00 (.-.) | . | 1.09 (0.57-2.10) | 0.79 | 0.59 | 0.99 | 0.80 |
| DPYD ---- tag |  | A/G | 26 | 43,33 | 12 | 37,50 | 171 | 50,15 | 155 | 50,65 | 0.85 (0.35-2.04) | 0.71 | 1.45 (0.76-2.77) | 0.26 | . | . | . |
| DPYD ---- tag |  | G/G | 8 | 13,33 | 5 | 15,63 | 51 | 14,96 | 52 | 16,99 | 0.87 (0.27-2.83) | 0.82 | 1.17 (0.60-2.30) | 0.64 | . | . | . |
| DPYD ---- tag | rs628959 | A/A | 24 | 40,00 | 16 | 50,00 | 167 | 48,97 | 152 | 49,67 | 1.00 (.-.) | . | 1.07 (0.57-1.98) | 0.84 | 0.38 | 0.99 | 0.77 |
| DPYD ---- tag |  | A/G | 34 | 56,67 | 12 | 37,50 | 152 | 44,57 | 125 | 40,85 | 0.63 (0.28-1.46) | 0.29 | 1.06 (0.56-1.99) | 0.86 | . | . | . |
| DPYD ---- tag |  | G/G | 2 | 3,33 | 4 | 12,50 | 22 | 6,45 | 29 | 9,48 | 0.73 (0.16-3.39) | 0.68 | 1.10 (0.53-2.29) | 0.79 | . | . | . |
| DPYD ---- tag | rs6656660 | G/G | 45 | 75,00 | 28 | 87,50 | 266 | 78,01 | 230 | 75,16 | 1.00 (.-.) | . | 1.24 (0.79-1.95) | 0.36 | 0.38 | 0.99 | 0.77 |
| DPYD ---- tag |  | G/T | 14 | 23,33 | 3 | 9,38 | 67 | 19,65 | 66 | 21,57 | 0.27 (0.04-2.01) | 0.20 | 1.27 (0.76-2.13) | 0.37 | . | . | . |
| DPYD ---- tag |  | T/T | 1 | 1,67 | 1 | 3,13 | 8 | 2,35 | 10 | 3,27 | 1.39 (0.18-10.87) | 0.75 | 1.52 (0.66-3.53) | 0.33 | . | . | . |
| DPYD ---- tag | rs6663670 | A/A | 44 | 73,33 | 25 | 78,13 | 254 | 74,49 | 211 | 68,95 | 1.00 (.-.) | . | 1.30 (0.79-2.14) | 0.31 | 0.70 | 0.99 | 0.84 |
| DPYD ---- tag |  | A/C | 15 | 25,00 | 6 | 18,75 | 82 | 24,05 | 87 | 28,43 | 1.05 (0.39-2.88) | 0.92 | 1.65 (0.98-2.80) | 0.06 | . | . | . |
| DPYD ---- tag |  | C/C | 1 | 1,67 | 1 | 3,13 | 5 | 1,47 | 8 | 2,61 | 1.45 (0.19-11.28) | 0.72 | 2.64 (0.96-7.24) | 0.06 | . | . | . |
| DPYD ---- tag | rs6683883 | T/T | 30 | 50,00 | 10 | 31,25 | 132 | 38,71 | 102 | 33,33 | 1.00 (.-.) | . | 1.65 (0.75-3.62) | 0.21 | 0.28 | 0.99 | 0.77 |
| DPYD ---- tag |  | T/C | 28 | 46,67 | 17 | 53,13 | 163 | 47,80 | 145 | 47,39 | 1.47 (0.58-3.71) | 0.41 | 2.00 (0.92-4.35) | 0.08 | . | . | . |
| DPYD ---- tag |  | C/C | 2 | 3,33 | 5 | 15,63 | 46 | 13,49 | 59 | 19,28 | 3.27 (0.88-12.20) | 0.08 | 2.32 (1.03-5.23) | 0.04 | . | . | . |
| DPYD ---- tag | rs6686861 | C/C | 52 | 86,67 | 30 | 93,75 | 299 | 87,68 | 259 | 84,64 | 1.00 (.-.) | . | 1.12 (0.72-1.74) | 0.62 | 0.01 | 0.99 | 0.47 |
| DPYD ---- tag |  | C/T | 8 | 13,33 | 2 | 6,25 | 40 | 11,73 | 44 | 14,38 | 0.16 (0.02-1.23) | 0.08 | 1.42 (0.83-2.42) | 0.20 | . | . | . |
| DPYD ---- tag |  | T/T | 0 | 0,00 | 0 | 0,00 | 2 | 0,59 | 3 | 0,98 | 0.60 (0.08-4.37) | 0.61 | 0.67 (0.09-5.09) | 0.70 | . | . | . |
| DPYD ---- tag | rs7414210 | A/A | 42 | 70,00 | 27 | 84,38 | 235 | 68,91 | 231 | 75,49 | 1.00 (.-.) | . | 1.54 (0.97-2.45) | 0.07 | 0.48 | 0.99 | 0.77 |
| DPYD ---- tag |  | A/C | 16 | 26,67 | 5 | 15,63 | 93 | 27,27 | 70 | 22,88 | 1.62 (0.47-5.63) | 0.45 | 1.17 (0.70-1.96) | 0.54 | . | . | . |
| DPYD ---- tag |  | C/C | 2 | 3,33 | 0 | 0,00 | 13 | 3,81 | 5 | 1,63 | 0.00 (0.00-I) | 0.98 | 0.81 (0.24-2.76) | 0.73 | . | . | . |
| DPYD ---- tag | rs7530858 | A/A | 47 | 78,33 | 29 | 90,63 | 272 | 79,77 | 229 | 74,84 | 1.00 (.-.) | . | 1.23 (0.77-1.96) | 0.39 | 0.34 | 0.99 | 0.77 |
| DPYD ---- tag |  | A/G | 13 | 21,67 | 3 | 9,38 | 66 | 19,35 | 74 | 24,18 | 0.72 (0.21-2.43) | 0.59 | 1.58 (0.95-2.63) | 0.08 | . | . | . |
| DPYD ---- tag |  | G/G | 0 | 0,00 | 0 | 0,00 | 3 | 0,88 | 3 | 0,98 | 1.53 (0.37-6.31) | 0.56 | 1.87 (0.42-8.31) | 0.41 | . | . | . |
| DPYD ---- tag | rs7544128 | C/C | 34 | 56,67 | 14 | 43,75 | 201 | 58,94 | 158 | 51,63 | 1.00 (.-.) | . | 1.97 (1.05-3.72) | 0.04 | 0.22 | 0.99 | 0.72 |
| DPYD ---- tag |  | C/G | 22 | 36,67 | 14 | 43,75 | 122 | 35,78 | 121 | 39,54 | 2.21 (0.96-5.11) | 0.06 | 1.77 (0.93-3.37) | 0.08 | . | . | . |
| DPYD ---- tag |  | G/G | 4 | 6,67 | 4 | 12,50 | 18 | 5,28 | 27 | 8,82 | 1.98 (0.43-9.15) | 0.38 | 3.20 (1.53-6.72) | 0.00 | . | . | . |
| DPYD ---- tag | rs7545340 | G/G | 35 | 58,33 | 14 | 43,75 | 194 | 56,89 | 172 | 56,21 | 1.00 (.-.) | . | 1.80 (0.96-3.40) | 0.07 | 0.21 | 0.99 | 0.72 |
| DPYD ---- tag |  | G/A | 23 | 38,33 | 15 | 46,88 | 121 | 35,48 | 109 | 35,62 | 1.65 (0.72-3.80) | 0.24 | 1.69 (0.88-3.23) | 0.11 | . | . | . |
| DPYD ---- tag |  | A/A | 2 | 3,33 | 3 | 9,38 | 26 | 7,62 | 25 | 8,17 | 2.23 (0.46-10.87) | 0.32 | 2.10 (1.01-4.40) | 0.05 | . | . | . |
| DPYD ---- tag | rs828054 | C/C | 7 | 11,67 | 6 | 18,75 | 77 | 22,58 | 91 | 29,74 | 1.00 (.-.) | . | 0.94 (0.36-2.45) | 0.90 | 0.67 | 0.99 | 0.84 |
| DPYD ---- tag |  | C/A | 33 | 55,00 | 20 | 62,50 | 173 | 50,73 | 156 | 50,98 | 0.57 (0.20-1.64) | 0.30 | 0.90 (0.35-2.33) | 0.82 | . | . | . |
| DPYD ---- tag |  | A/A | 20 | 33,33 | 6 | 18,75 | 91 | 26,69 | 59 | 19,28 | 0.49 (0.14-1.77) | 0.28 | 0.61 (0.23-1.62) | 0.32 | . | . | . |
| DPYD ---- tag | rs885622 | G/G | 34 | 56,67 | 11 | 34,38 | 136 | 39,88 | 104 | 33,99 | 1.00 (.-.) | . | 1.80 (0.89-3.63) | 0.10 | 0.19 | 0.99 | 0.72 |
| DPYD ---- tag |  | G/A | 23 | 38,33 | 15 | 46,88 | 163 | 47,80 | 151 | 49,35 | 2.08 (0.86-5.02) | 0.11 | 2.24 (1.12-4.47) | 0.02 | . | . | . |
| DPYD ---- tag |  | A/A | 3 | 5,00 | 6 | 18,75 | 42 | 12,32 | 51 | 16,67 | 3.02 (0.87-10.44) | 0.08 | 2.56 (1.22-5.35) | 0.01 | . | . | . |
| DPYD ---- tag | rs9437663 | G/G | 40 | 66,67 | 23 | 71,88 | 225 | 65,98 | 187 | 61,11 | 1.00 (.-.) | . | 1.12 (0.70-1.79) | 0.63 | 0.23 | 0.99 | 0.72 |
| DPYD ---- tag |  | G/A | 18 | 30,00 | 6 | 18,75 | 105 | 30,79 | 99 | 32,35 | 0.23 (0.03-1.71) | 0.15 | 1.38 (0.84-2.28) | 0.21 | . | . | . |
| DPYD ---- tag |  | A/A | 2 | 3,33 | 3 | 9,38 | 11 | 3,23 | 20 | 6,54 | 1.41 (0.32-6.17) | 0.64 | 2.16 (1.08-4.29) | 0.03 | . | . | . |
| DPYS ---- tag | rs13249169 | A/A | 47 | 78,33 | 28 | 87,50 | 271 | 79,47 | 235 | 76,80 | 1.00 (.-.) | . | 1.23 (0.77-1.97) | 0.38 | 0.36 | 0.99 | 0.99 |
| DPYS ---- tag |  | A/T | 13 | 21,67 | 4 | 12,50 | 66 | 19,35 | 67 | 21,90 | 0.65 (0.22-1.92) | 0.44 | 1.43 (0.84-2.43) | 0.18 | . | . | . |
| DPYS ---- tag |  | T/T | 0 | 0,00 | 0 | 0,00 | 4 | 1,17 | 4 | 1,31 | 0.81 (0.30-2.23) | 0.69 | 1.00 (0.34-2.99) | 1.00 | . | . | . |
| DPYS ---- NA | rs13263121 | T/T | 25 | 41,67 | 16 | 50,00 | 153 | 44,87 | 139 | 45,42 | 1.00 (.-.) | . | 1.21 (0.64-2.28) | 0.55 | 0.99 | 1.00 | 0.99 |
| DPYS ---- NA |  | T/A | 32 | 53,33 | 14 | 43,75 | 143 | 41,94 | 135 | 44,12 | 0.69 (0.30-1.58) | 0.38 | 1.18 (0.63-2.20) | 0.60 | . | . | . |
| DPYS ---- NA |  | A/A | 3 | 5,00 | 2 | 6,25 | 45 | 13,20 | 32 | 10,46 | 2.01 (0.44-9.16) | 0.37 | 1.02 (0.50-2.08) | 0.95 | . | . | . |
| DPYS ---- tag | rs16871361 | T/T | 55 | 91,67 | 29 | 90,63 | 301 | 88,27 | 269 | 87,91 | 1.00 (.-.) | . | 1.37 (0.87-2.16) | 0.18 | 0.99 | 1.00 | 0.99 |
| DPYS ---- tag |  | T/C | 5 | 8,33 | 3 | 9,38 | 39 | 11,44 | 37 | 12,09 | 0.97 (0.28-3.29) | 0.96 | 1.35 (0.76-2.40) | 0.30 | . | . | . |
| DPYS ---- tag |  | C/C | 0 | 0,00 | 0 | 0,00 | 1 | 0,29 | 0 | 0,00 | 0.00 (0.00-4E305) | 0.98 | 0.00 (0.00-6E305) | 0.98 | . | . | . |
| DPYS ---- NA | rs17245950 | T/T | 49 | 81,67 | 25 | 78,13 | 259 | 75,95 | 237 | 77,45 | 1.00 (.-.) | . | 1.45 (0.90-2.35) | 0.13 | 0.66 | 0.99 | 0.99 |
| DPYS ---- NA |  | T/A | 9 | 15,00 | 7 | 21,88 | 76 | 22,29 | 66 | 21,57 | 1.35 (0.50-3.70) | 0.55 | 1.39 (0.81-2.38) | 0.24 | . | . | . |
| DPYS ---- NA |  | A/A | 2 | 3,33 | 0 | 0,00 | 6 | 1,76 | 3 | 0,98 | 0.00 (0.00-I) | 0.98 | 0.86 (0.19-3.80) | 0.84 | . | . | . |
| DPYS ---- NA | rs2253336 | A/A | 51 | 85,00 | 27 | 84,38 | 264 | 77,42 | 250 | 81,70 | 1.00 (.-.) | . | 1.47 (0.92-2.34) | 0.10 | 0.68 | 0.99 | 0.99 |
| DPYS ---- NA |  | A/G | 8 | 13,33 | 5 | 15,63 | 74 | 21,70 | 52 | 16,99 | 1.02 (0.29-3.56) | 0.98 | 1.09 (0.64-1.87) | 0.75 | . | . | . |
| DPYS ---- NA |  | G/G | 1 | 1,67 | 0 | 0,00 | 3 | 0,88 | 4 | 1,31 | 0.86 (0.21-3.54) | 0.84 | 1.27 (0.29-5.55) | 0.76 | . | . | . |
| DPYS ---- tag | rs2280010 | C/C | 36 | 60,00 | 11 | 34,38 | 206 | 60,41 | 169 | 55,23 | 1.00 (.-.) | . | 1.37 (0.71-2.67) | 0.35 | 0.42 | 0.99 | 0.99 |
| DPYS ---- tag |  | C/T | 20 | 33,33 | 18 | 56,25 | 124 | 36,36 | 119 | 38,89 | 1.60 (0.69-3.72) | 0.27 | 1.99 (1.02-3.89) | 0.04 | . | . | . |
| DPYS ---- tag |  | T/T | 4 | 6,67 | 3 | 9,38 | 11 | 3,23 | 18 | 5,88 | 0.68 (0.14-3.22) | 0.63 | 2.13 (0.92-4.97) | 0.08 | . | . | . |
| DPYS ---- tag | rs2333874 | T/T | 23 | 38,33 | 14 | 43,75 | 144 | 42,23 | 131 | 42,81 | 1.00 (.-.) | . | 1.80 (0.90-3.61) | 0.10 | 0.75 | 0.99 | 0.99 |
| DPYS ---- tag |  | T/G | 27 | 45,00 | 17 | 53,13 | 154 | 45,16 | 139 | 45,42 | 1.58 (0.68-3.67) | 0.29 | 1.56 (0.78-3.14) | 0.21 | . | . | . |
| DPYS ---- tag |  | G/G | 10 | 16,67 | 1 | 3,13 | 43 | 12,61 | 36 | 11,76 | 0.54 (0.07-4.30) | 0.56 | 1.80 (0.83-3.91) | 0.14 | . | . | . |
| DPYS ---- NA | rs2669429 | C/C | 15 | 25,00 | 8 | 25,00 | 113 | 33,14 | 87 | 28,43 | 1.00 (.-.) | . | 0.77 (0.35-1.73) | 0.53 | 0.11 | 0.99 | 0.99 |
| DPYS ---- NA |  | C/T | 30 | 50,00 | 16 | 50,00 | 167 | 48,97 | 163 | 53,27 | 0.71 (0.27-1.84) | 0.48 | 1.09 (0.50-2.37) | 0.83 | . | . | . |
| DPYS ---- NA |  | T/T | 15 | 25,00 | 8 | 25,00 | 61 | 17,89 | 56 | 18,30 | 0.58 (0.20-1.68) | 0.31 | 1.10 (0.49-2.50) | 0.81 | . | . | . |
| DPYS ---- tag | rs2669434 | C/C | 33 | 55,00 | 20 | 62,50 | 164 | 48,09 | 164 | 53,59 | 1.00 (.-.) | . | 1.36 (0.78-2.35) | 0.28 | 0.73 | 0.99 | 0.99 |
| DPYS ---- tag |  | C/A | 18 | 30,00 | 11 | 34,38 | 150 | 43,99 | 118 | 38,56 | 0.85 (0.37-1.97) | 0.71 | 1.13 (0.64-1.98) | 0.68 | . | . | . |
| DPYS ---- tag |  | A/A | 9 | 15,00 | 1 | 3,13 | 27 | 7,92 | 24 | 7,84 | 0.55 (0.07-4.20) | 0.56 | 1.33 (0.65-2.69) | 0.44 | . | . | . |
| DPYS ---- tag | rs2853142 | T/T | 28 | 46,67 | 14 | 43,75 | 125 | 36,66 | 127 | 41,50 | 1.00 (.-.) | . | 1.79 (0.94-3.41) | 0.08 | 0.50 | 0.99 | 0.99 |
| DPYS ---- tag |  | T/C | 24 | 40,00 | 16 | 50,00 | 174 | 51,03 | 141 | 46,08 | 1.30 (0.57-2.97) | 0.54 | 1.40 (0.74-2.66) | 0.30 | . | . | . |
| DPYS ---- tag |  | C/C | 8 | 13,33 | 2 | 6,25 | 42 | 12,32 | 38 | 12,42 | 0.75 (0.09-5.89) | 0.78 | 1.42 (0.69-2.90) | 0.34 | . | . | . |
| DPYS ---- NA | rs2853145 | A/A | 45 | 75,00 | 23 | 71,88 | 223 | 65,40 | 208 | 67,97 | 1.00 (.-.) | . | 1.55 (0.93-2.56) | 0.09 | 0.56 | 0.99 | 0.99 |
| DPYS ---- NA |  | A/C | 14 | 23,33 | 8 | 25,00 | 109 | 31,96 | 88 | 28,76 | 1.15 (0.46-2.85) | 0.77 | 1.21 (0.71-2.06) | 0.50 | . | . | . |
| DPYS ---- NA |  | C/C | 1 | 1,67 | 1 | 3,13 | 9 | 2,64 | 10 | 3,27 | 0.00 (0.00-I) | 0.98 | 1.46 (0.60-3.58) | 0.40 | . | . | . |
| DPYS ---- tag | rs2853149 | G/G | 15 | 25,00 | 7 | 21,88 | 89 | 26,10 | 85 | 27,78 | 1.00 (.-.) | . | 1.28 (0.51-3.23) | 0.60 | 0.49 | 0.99 | 0.99 |
| DPYS ---- tag |  | G/A | 26 | 43,33 | 21 | 65,63 | 176 | 51,61 | 149 | 48,69 | 0.98 (0.35-2.71) | 0.97 | 1.18 (0.47-2.93) | 0.73 | . | . | . |
| DPYS ---- tag |  | A/A | 19 | 31,67 | 4 | 12,50 | 76 | 22,29 | 72 | 23,53 | 0.57 (0.15-2.16) | 0.41 | 1.21 (0.47-3.09) | 0.70 | . | . | . |
| DPYS ---- tag | rs2853154 | T/T | 37 | 61,67 | 23 | 71,88 | 190 | 55,72 | 175 | 57,19 | 1.00 (.-.) | . | 1.45 (0.87-2.43) | 0.15 | 0.95 | 1.00 | 0.99 |
| DPYS ---- tag |  | T/C | 20 | 33,33 | 8 | 25,00 | 136 | 39,88 | 116 | 37,91 | 1.05 (0.43-2.55) | 0.91 | 1.28 (0.76-2.16) | 0.36 | . | . | . |
| DPYS ---- tag |  | C/C | 3 | 5,00 | 1 | 3,13 | 15 | 4,40 | 15 | 4,90 | 0.00 (0.00-I) | 0.98 | 1.28 (0.61-2.66) | 0.51 | . | . | . |
| DPYS ---- tag | rs2853161 | A/A | 20 | 33,33 | 3 | 9,38 | 96 | 28,15 | 77 | 25,16 | 1.00 (.-.) | . | 2.70 (0.83-8.79) | 0.10 | 0.78 | 0.99 | 0.99 |
| DPYS ---- tag |  | A/G | 31 | 51,67 | 24 | 75,00 | 169 | 49,56 | 167 | 54,58 | 2.83 (0.83-9.65) | 0.10 | 2.89 (0.91-9.24) | 0.07 | . | . | . |
| DPYS ---- tag |  | G/G | 9 | 15,00 | 5 | 15,63 | 76 | 22,29 | 62 | 20,26 | 1.03 (0.17-6.35) | 0.98 | 2.92 (0.89-9.52) | 0.08 | . | . | . |
| DPYS ---- NA | rs2959024 | T/T | 35 | 58,33 | 12 | 37,50 | 180 | 52,79 | 144 | 47,06 | 1.00 (.-.) | . | 1.17 (0.62-2.20) | 0.62 | 0.24 | 0.99 | 0.99 |
| DPYS ---- NA |  | T/G | 20 | 33,33 | 18 | 56,25 | 138 | 40,47 | 135 | 44,12 | 1.25 (0.55-2.84) | 0.60 | 1.61 (0.86-3.03) | 0.14 | . | . | . |
| DPYS ---- NA |  | G/G | 5 | 8,33 | 2 | 6,25 | 23 | 6,74 | 27 | 8,82 | 0.32 (0.04-2.60) | 0.29 | 1.63 (0.77-3.45) | 0.20 | . | . | . |
| DPYS ---- NA | rs2959025 | A/A | 26 | 43,33 | 12 | 37,50 | 148 | 43,40 | 119 | 38,89 | 1.00 (.-.) | . | 1.30 (0.69-2.46) | 0.42 | 0.56 | 0.99 | 0.99 |
| DPYS ---- NA |  | A/G | 27 | 45,00 | 16 | 50,00 | 152 | 44,57 | 139 | 45,42 | 1.24 (0.52-2.93) | 0.63 | 1.47 (0.78-2.76) | 0.23 | . | . | . |
| DPYS ---- NA |  | G/G | 7 | 11,67 | 4 | 12,50 | 41 | 12,02 | 48 | 15,69 | 0.72 (0.20-2.65) | 0.63 | 1.61 (0.81-3.19) | 0.17 | . | . | . |
| DPYS ---- tag | rs2959026 | G/G | 20 | 33,33 | 10 | 31,25 | 120 | 35,19 | 108 | 35,29 | 1.00 (.-.) | . | 1.37 (0.62-3.01) | 0.43 | 0.77 | 0.99 | 0.99 |
| DPYS ---- tag |  | G/A | 27 | 45,00 | 17 | 53,13 | 158 | 46,33 | 154 | 50,33 | 0.99 (0.39-2.54) | 0.99 | 1.51 (0.69-3.28) | 0.30 | . | . | . |
| DPYS ---- tag |  | A/A | 13 | 21,67 | 5 | 15,63 | 63 | 18,48 | 44 | 14,38 | 1.09 (0.34-3.49) | 0.89 | 1.11 (0.48-2.55) | 0.81 | . | . | . |
| DPYS ---- NA | rs3133278 | T/T | 31 | 51,67 | 19 | 59,38 | 176 | 51,61 | 153 | 50,00 | 1.00 (.-.) | . | 1.40 (0.78-2.51) | 0.26 | 0.92 | 1.00 | 0.99 |
| DPYS ---- NA |  | T/C | 26 | 43,33 | 13 | 40,63 | 141 | 41,35 | 133 | 43,46 | 0.99 (0.44-2.20) | 0.98 | 1.32 (0.74-2.36) | 0.35 | . | . | . |
| DPYS ---- NA |  | C/C | 3 | 5,00 | 0 | 0,00 | 24 | 7,04 | 20 | 6,54 | 0.00 (0.00-2E240) | 0.97 | 1.18 (0.57-2.44) | 0.65 | . | . | . |
| DPYS ---- tag | rs3750187 | G/G | 40 | 66,67 | 22 | 68,75 | 214 | 62,76 | 191 | 62,42 | 1.00 (.-.) | . | 1.39 (0.82-2.36) | 0.22 | 0.83 | 1.00 | 0.99 |
| DPYS ---- tag |  | G/A | 18 | 30,00 | 9 | 28,13 | 115 | 33,72 | 105 | 34,31 | 0.95 (0.39-2.33) | 0.91 | 1.35 (0.79-2.32) | 0.28 | . | . | . |
| DPYS ---- tag |  | A/A | 2 | 3,33 | 1 | 3,13 | 12 | 3,52 | 10 | 3,27 | 1.73 (0.22-13.38) | 0.60 | 1.42 (0.61-3.29) | 0.41 | . | . | . |
| DPYS ---- tag | rs3793357 | T/T | 53 | 88,33 | 29 | 90,63 | 299 | 87,68 | 277 | 90,52 | 1.00 (.-.) | . | 1.27 (0.81-2.00) | 0.30 | 0.42 | 0.99 | 0.99 |
| DPYS ---- tag |  | T/G | 6 | 10,00 | 3 | 9,38 | 41 | 12,02 | 27 | 8,82 | 0.59 (0.17-2.06) | 0.41 | 1.12 (0.58-2.14) | 0.74 | . | . | . |
| DPYS ---- tag |  | G/G | 1 | 1,67 | 0 | 0,00 | 1 | 0,29 | 2 | 0,65 | 0.00 (0.00-I) | 0.98 | 1.78 (0.23-13.84) | 0.58 | . | . | . |
| DPYS ---- tag | rs3793358 | G/G | 51 | 85,00 | 27 | 84,38 | 260 | 76,25 | 243 | 79,41 | 1.00 (.-.) | . | 1.51 (0.95-2.40) | 0.08 | 0.62 | 0.99 | 0.99 |
| DPYS ---- tag |  | G/A | 9 | 15,00 | 4 | 12,50 | 79 | 23,17 | 59 | 19,28 | 1.07 (0.32-3.65) | 0.91 | 1.09 (0.65-1.83) | 0.74 | . | . | . |
| DPYS ---- tag |  | A/A | 0 | 0,00 | 1 | 3,13 | 2 | 0,59 | 4 | 1,31 | 1.04 (0.32-3.33) | 0.95 | 1.56 (0.45-5.43) | 0.48 | . | . | . |
| DPYS ---- tag | rs6468924 | C/C | 39 | 65,00 | 22 | 68,75 | 206 | 60,41 | 190 | 62,09 | 1.00 (.-.) | . | 1.31 (0.78-2.20) | 0.31 | 0.71 | 0.99 | 0.99 |
| DPYS ---- tag |  | C/T | 16 | 26,67 | 9 | 28,13 | 118 | 34,60 | 103 | 33,66 | 0.86 (0.35-2.11) | 0.74 | 1.25 (0.72-2.16) | 0.42 | . | . | . |
| DPYS ---- tag |  | T/T | 5 | 8,33 | 1 | 3,13 | 17 | 4,99 | 13 | 4,25 | 0.64 (0.08-4.89) | 0.67 | 1.22 (0.53-2.81) | 0.64 | . | . | . |
| DUT ---- tag | rs8025164 | G/G | 45 | 75,00 | 21 | 65,63 | 221 | 64,81 | 223 | 72,88 | 1.00 (.-.) | . | 1.68 (1.01-2.81) | 0.05 | 0.10 | 0.99 | 0.10 |
| DUT ---- tag |  | G/A | 13 | 21,67 | 10 | 31,25 | 108 | 31,67 | 72 | 23,53 | 1.39 (0.57-3.41) | 0.47 | 1.16 (0.66-2.03) | 0.61 | . | . | . |
| DUT ---- tag |  | A/A | 2 | 3,33 | 1 | 3,13 | 12 | 3,52 | 11 | 3,59 | 9.31 (1.12-77.32) | 0.04 | 2.70 (1.12-6.51) | 0.03 | . | . | . |
| EHMT1 ---- tag | rs10780190 | C/C | 56 | 93,33 | 28 | 87,50 | 304 | 89,15 | 274 | 89,54 | 1.00 (.-.) | . | 1.46 (0.93-2.28) | 0.10 | 0.15 | 0.99 | 0.29 |
| EHMT1 ---- tag |  | C/T | 3 | 5,00 | 4 | 12,50 | 36 | 10,56 | 32 | 10,46 | 3.34 (0.72-15.42) | 0.12 | 1.32 (0.71-2.45) | 0.38 | . | . | . |
| EHMT1 ---- tag |  | T/T | 1 | 1,67 | 0 | 0,00 | 1 | 0,29 | 0 | 0,00 | 0.00 (0.00-I) | 0.98 | 0.00 (0.00-I) | 0.98 | . | . | . |
| EHMT1 ---- tag | rs10867083 | G/G | 24 | 40,00 | 16 | 50,00 | 160 | 46,92 | 144 | 47,06 | 1.00 (.-.) | . | 0.95 (0.50-1.80) | 0.87 | 0.34 | 0.99 | 0.53 |
| EHMT1 ---- tag |  | G/A | 32 | 53,33 | 13 | 40,63 | 152 | 44,57 | 134 | 43,79 | 0.53 (0.22-1.28) | 0.16 | 1.02 (0.54-1.92) | 0.96 | . | . | . |
| EHMT1 ---- tag |  | A/A | 4 | 6,67 | 3 | 9,38 | 29 | 8,50 | 28 | 9,15 | 0.67 (0.18-2.46) | 0.54 | 0.79 (0.37-1.70) | 0.55 | . | . | . |
| EHMT1 ---- tag | rs11137190 | C/C | 33 | 55,00 | 16 | 50,00 | 177 | 51,91 | 157 | 51,31 | 1.00 (.-.) | . | 1.60 (0.89-2.89) | 0.12 | 0.16 | 0.99 | 0.29 |
| EHMT1 ---- tag |  | C/G | 24 | 40,00 | 14 | 43,75 | 136 | 39,88 | 128 | 41,83 | 1.49 (0.64-3.46) | 0.36 | 1.91 (1.06-3.43) | 0.03 | . | . | . |
| EHMT1 ---- tag |  | G/G | 3 | 5,00 | 2 | 6,25 | 28 | 8,21 | 21 | 6,86 | 3.01 (0.67-13.61) | 0.15 | 1.12 (0.51-2.46) | 0.77 | . | . | . |
| EHMT1 ---- tag | rs3123510 | G/G | 26 | 43,33 | 9 | 28,13 | 131 | 38,42 | 117 | 38,24 | 1.00 (.-.) | . | 2.23 (1.10-4.50) | 0.03 | 0.03 | 0.99 | 0.18 |
| EHMT1 ---- tag |  | G/A | 28 | 46,67 | 17 | 53,13 | 162 | 47,51 | 146 | 47,71 | 2.06 (0.83-5.09) | 0.12 | 2.13 (1.07-4.25) | 0.03 | . | . | . |
| EHMT1 ---- tag |  | A/A | 6 | 10,00 | 6 | 18,75 | 48 | 14,08 | 43 | 14,05 | 3.13 (1.04-9.48) | 0.04 | 2.07 (0.97-4.44) | 0.06 | . | . | . |
| EHMT1 ---- candidate literature | rs3125795 | G/G | 55 | 91,67 | 28 | 87,50 | 304 | 89,15 | 274 | 89,54 | 1.00 (.-.) | . | 1.46 (0.93-2.28) | 0.10 | 0.15 | 0.99 | 0.29 |
| EHMT1 ---- candidate literature |  | G/T | 4 | 6,67 | 4 | 12,50 | 36 | 10,56 | 32 | 10,46 | 3.34 (0.72-15.42) | 0.12 | 1.31 (0.71-2.43) | 0.39 | . | . | . |
| EHMT1 ---- candidate literature |  | T/T | 1 | 1,67 | 0 | 0,00 | 1 | 0,29 | 0 | 0,00 | 0.00 (0.00-I) | 0.98 | 0.00 (0.00-I) | 0.98 | . | . | . |
| EHMT1 ---- tag | rs4573359 | G/G | 49 | 81,67 | 29 | 90,63 | 286 | 83,87 | 259 | 84,64 | 1.00 (.-.) | . | 1.34 (0.86-2.09) | 0.20 | 0.43 | 0.99 | 0.54 |
| EHMT1 ---- tag |  | G/T | 11 | 18,33 | 3 | 9,38 | 53 | 15,54 | 46 | 15,03 | 0.41 (0.05-3.08) | 0.39 | 1.14 (0.67-1.94) | 0.62 | . | . | . |
| EHMT1 ---- tag |  | T/T | 0 | 0,00 | 0 | 0,00 | 2 | 0,59 | 1 | 0,33 | 0.95 (0.13-6.89) | 0.96 | 1.27 (0.17-9.61) | 0.82 | . | . | . |
| EHMT1 ---- candidate literature | rs4634736 | G/G | 49 | 81,67 | 29 | 90,63 | 285 | 83,58 | 260 | 84,97 | 1.00 (.-.) | . | 1.34 (0.86-2.10) | 0.19 | 0.44 | 0.99 | 0.54 |
| EHMT1 ---- candidate literature |  | G/A | 11 | 18,33 | 3 | 9,38 | 54 | 15,84 | 45 | 14,71 | 0.41 (0.05-3.08) | 0.39 | 1.12 (0.66-1.90) | 0.69 | . | . | . |
| EHMT1 ---- candidate literature |  | A/A | 0 | 0,00 | 0 | 0,00 | 2 | 0,59 | 1 | 0,33 | 0.95 (0.13-6.88) | 0.96 | 1.28 (0.17-9.64) | 0.81 | . | . | . |
| EHMT1 ---- tag | rs4876902 | C/C | 45 | 75,00 | 16 | 50,00 | 214 | 62,76 | 191 | 62,42 | 1.00 (.-.) | . | 1.93 (1.06-3.52) | 0.03 | 0.03 | 0.99 | 0.18 |
| EHMT1 ---- tag |  | C/T | 14 | 23,33 | 15 | 46,88 | 113 | 33,14 | 102 | 33,33 | 2.61 (1.16-5.88) | 0.02 | 2.26 (1.22-4.20) | 0.01 | . | . | . |
| EHMT1 ---- tag |  | T/T | 1 | 1,67 | 1 | 3,13 | 14 | 4,11 | 13 | 4,25 | 4.49 (0.56-36.13) | 0.16 | 1.80 (0.72-4.48) | 0.21 | . | . | . |
| EHMT1 ---- tag | rs4876904 | T/T | 20 | 33,33 | 11 | 34,38 | 95 | 27,86 | 94 | 30,72 | 1.00 (.-.) | . | 1.49 (0.70-3.16) | 0.30 | 0.60 | 0.99 | 0.65 |
| EHMT1 ---- tag |  | T/G | 23 | 38,33 | 14 | 43,75 | 173 | 50,73 | 142 | 46,41 | 1.64 (0.65-4.15) | 0.29 | 1.40 (0.66-2.94) | 0.38 | . | . | . |
| EHMT1 ---- tag |  | G/G | 17 | 28,33 | 7 | 21,88 | 73 | 21,41 | 70 | 22,88 | 0.59 (0.20-1.75) | 0.34 | 1.24 (0.57-2.68) | 0.59 | . | . | . |
| EHMT1 ---- tag | rs7390244 | A/A | 15 | 25,00 | 8 | 25,00 | 79 | 23,17 | 80 | 26,14 | 1.00 (.-.) | . | 1.80 (0.76-4.24) | 0.18 | 0.65 | 0.99 | 0.65 |
| EHMT1 ---- tag |  | A/G | 31 | 51,67 | 17 | 53,13 | 173 | 50,73 | 140 | 45,75 | 1.55 (0.58-4.13) | 0.38 | 1.84 (0.79-4.28) | 0.16 | . | . | . |
| EHMT1 ---- tag |  | G/G | 14 | 23,33 | 7 | 21,88 | 89 | 26,10 | 86 | 28,10 | 1.16 (0.34-3.95) | 0.81 | 1.63 (0.69-3.89) | 0.27 | . | . | . |
| EHMT1 ---- tag | rs9314635 | G/G | 30 | 50,00 | 12 | 37,50 | 143 | 41,94 | 121 | 39,54 | 1.00 (.-.) | . | 1.77 (0.93-3.37) | 0.08 | 0.07 | 0.99 | 0.27 |
| EHMT1 ---- tag |  | G/T | 24 | 40,00 | 16 | 50,00 | 155 | 45,45 | 150 | 49,02 | 1.80 (0.77-4.19) | 0.18 | 2.06 (1.10-3.86) | 0.02 | . | . | . |
| EHMT1 ---- tag |  | T/T | 6 | 10,00 | 4 | 12,50 | 43 | 12,61 | 35 | 11,44 | 3.18 (0.85-11.84) | 0.09 | 1.51 (0.71-3.17) | 0.28 | . | . | . |
| EHMT2 ---- candidate/tag | rs2736428 | G/G | 21 | 35,00 | 12 | 37,50 | 127 | 37,24 | 139 | 45,42 | 1.00 (.-.) | . | 1.27 (0.64-2.55) | 0.49 | 0.56 | 0.99 | 0.61 |
| EHMT2 ---- candidate/tag |  | G/A | 29 | 48,33 | 15 | 46,88 | 168 | 49,27 | 133 | 43,46 | 0.88 (0.38-2.07) | 0.78 | 1.06 (0.53-2.13) | 0.86 | . | . | . |
| EHMT2 ---- candidate/tag |  | A/A | 10 | 16,67 | 5 | 15,63 | 46 | 13,49 | 34 | 11,11 | 0.31 (0.07-1.46) | 0.14 | 0.75 (0.35-1.63) | 0.47 | . | . | . |
| EHMT2 ---- tag | rs9267649 | G/G | 47 | 78,33 | 24 | 75,00 | 246 | 72,14 | 219 | 71,57 | 1.00 (.-.) | . | 1.47 (0.89-2.43) | 0.13 | 0.61 | 0.99 | 0.61 |
| EHMT2 ---- tag |  | G/A | 10 | 16,67 | 8 | 25,00 | 84 | 24,63 | 77 | 25,16 | 1.83 (0.75-4.46) | 0.19 | 1.71 (1.00-2.94) | 0.05 | . | . | . |
| EHMT2 ---- tag |  | A/A | 3 | 5,00 | 0 | 0,00 | 11 | 3,23 | 10 | 3,27 | 0.00 (0.00-6E286) | 0.98 | 2.01 (0.82-4.93) | 0.13 | . | . | . |
| FDXR ---- NA | rs2070918 | T/T | 24 | 40,00 | 13 | 40,63 | 167 | 48,97 | 155 | 50,65 | 1.00 (.-.) | . | 1.65 (0.83-3.29) | 0.15 | 0.64 | 0.99 | 0.88 |
| FDXR ---- NA |  | T/C | 28 | 46,67 | 16 | 50,00 | 139 | 40,76 | 116 | 37,91 | 1.72 (0.72-4.12) | 0.22 | 1.97 (0.99-3.94) | 0.05 | . | . | . |
| FDXR ---- NA |  | C/C | 8 | 13,33 | 3 | 9,38 | 35 | 10,26 | 35 | 11,44 | 1.43 (0.38-5.43) | 0.60 | 2.04 (0.95-4.38) | 0.07 | . | . | . |
| FDXR ---- tag | rs509911 | A/A | 33 | 55,00 | 21 | 65,63 | 217 | 63,64 | 197 | 64,38 | 1.00 (.-.) | . | 1.39 (0.81-2.37) | 0.23 | 0.88 | 1.00 | 0.88 |
| FDXR ---- tag |  | A/G | 21 | 35,00 | 10 | 31,25 | 105 | 30,79 | 99 | 32,35 | 1.53 (0.65-3.63) | 0.33 | 2.00 (1.15-3.48) | 0.01 | . | . | . |
| FDXR ---- tag |  | G/G | 6 | 10,00 | 1 | 3,13 | 19 | 5,57 | 10 | 3,27 | 0.96 (0.12-7.41) | 0.97 | 1.11 (0.48-2.55) | 0.81 | . | . | . |
| FDXR ---- NA | rs689882 | G/G | 23 | 38,33 | 20 | 62,50 | 181 | 53,08 | 166 | 54,25 | 1.00 (.-.) | . | 1.09 (0.62-1.90) | 0.76 | 0.86 | 1.00 | 0.88 |
| FDXR ---- NA |  | G/A | 30 | 50,00 | 8 | 25,00 | 134 | 39,30 | 125 | 40,85 | 0.48 (0.18-1.25) | 0.13 | 1.34 (0.76-2.36) | 0.31 | . | . | . |
| FDXR ---- NA |  | A/A | 7 | 11,67 | 4 | 12,50 | 26 | 7,62 | 15 | 4,90 | 1.69 (0.55-5.21) | 0.36 | 0.82 (0.39-1.73) | 0.60 | . | . | . |
| FDXR ---- NA | rs689895 | G/G | 30 | 50,00 | 18 | 56,25 | 170 | 49,85 | 153 | 50,00 | 1.00 (.-.) | . | 1.47 (0.82-2.60) | 0.19 | 0.14 | 0.99 | 0.57 |
| FDXR ---- NA |  | G/C | 26 | 43,33 | 10 | 31,25 | 139 | 40,76 | 138 | 45,10 | 0.95 (0.37-2.39) | 0.91 | 1.78 (1.01-3.14) | 0.05 | . | . | . |
| FDXR ---- NA |  | C/C | 4 | 6,67 | 4 | 12,50 | 32 | 9,38 | 15 | 4,90 | 3.11 (0.99-9.75) | 0.05 | 0.74 (0.34-1.63) | 0.45 | . | . | . |
| FOLH1 ---- candidate literature | rs10839236 | T/T | 27 | 45,00 | 9 | 28,13 | 144 | 42,23 | 122 | 39,87 | 1.00 (.-.) | . | 1.74 (0.74-4.09) | 0.20 | 0.40 | 0.99 | 0.73 |
| FOLH1 ---- candidate literature |  | T/C | 27 | 45,00 | 18 | 56,25 | 149 | 43,70 | 151 | 49,35 | 1.27 (0.48-3.38) | 0.63 | 1.71 (0.73-4.00) | 0.21 | . | . | . |
| FOLH1 ---- candidate literature |  | C/C | 6 | 10,00 | 5 | 15,63 | 48 | 14,08 | 33 | 10,78 | 1.58 (0.47-5.31) | 0.46 | 1.57 (0.64-3.86) | 0.33 | . | . | . |
| FOLH1 ---- tag | rs16906190 | A/A | 48 | 80,00 | 26 | 81,25 | 275 | 80,65 | 258 | 84,31 | 1.00 (.-.) | . | 1.43 (0.89-2.32) | 0.14 | 0.66 | 0.99 | 0.73 |
| FOLH1 ---- tag |  | A/G | 12 | 20,00 | 6 | 18,75 | 59 | 17,30 | 45 | 14,71 | 1.06 (0.38-2.95) | 0.90 | 1.18 (0.66-2.11) | 0.58 | . | . | . |
| FOLH1 ---- tag |  | G/G | 0 | 0,00 | 0 | 0,00 | 7 | 2,05 | 3 | 0,98 | 0.74 (0.23-2.35) | 0.61 | 1.06 (0.31-3.67) | 0.92 | . | . | . |
| FOLH1 ---- candidate | rs202676 | T/T | 38 | 63,33 | 18 | 56,25 | 213 | 62,46 | 192 | 62,75 | 1.00 (.-.) | . | 1.60 (0.86-2.96) | 0.14 | 0.43 | 0.99 | 0.73 |
| FOLH1 ---- candidate |  | T/C | 22 | 36,67 | 12 | 37,50 | 111 | 32,55 | 98 | 32,03 | 1.20 (0.52-2.76) | 0.67 | 1.41 (0.74-2.67) | 0.29 | . | . | . |
| FOLH1 ---- candidate |  | C/C | 0 | 0,00 | 2 | 6,25 | 17 | 4,99 | 16 | 5,23 | 2.33 (0.49-11.04) | 0.29 | 2.24 (1.02-4.89) | 0.04 | . | . | . |
| FOLH1 ---- tag | rs202680 | A/A | 34 | 56,67 | 17 | 53,13 | 191 | 56,01 | 173 | 56,54 | 1.00 (.-.) | . | 1.67 (0.88-3.17) | 0.12 | 0.34 | 0.99 | 0.73 |
| FOLH1 ---- tag |  | A/T | 26 | 43,33 | 13 | 40,63 | 130 | 38,12 | 114 | 37,25 | 1.27 (0.55-2.92) | 0.57 | 1.45 (0.75-2.79) | 0.27 | . | . | . |
| FOLH1 ---- tag |  | T/T | 0 | 0,00 | 2 | 6,25 | 20 | 5,87 | 19 | 6,21 | 2.41 (0.50-11.49) | 0.27 | 2.18 (1.01-4.70) | 0.05 | . | . | . |
| FOLH1 ---- candidate literature | rs202720 | G/G | 37 | 61,67 | 18 | 56,25 | 214 | 62,76 | 192 | 62,75 | 1.00 (.-.) | . | 1.56 (0.84-2.88) | 0.16 | 0.50 | 0.99 | 0.73 |
| FOLH1 ---- candidate literature |  | G/C | 23 | 38,33 | 12 | 37,50 | 110 | 32,26 | 98 | 32,03 | 1.14 (0.49-2.62) | 0.76 | 1.38 (0.73-2.62) | 0.32 | . | . | . |
| FOLH1 ---- candidate literature |  | C/C | 0 | 0,00 | 2 | 6,25 | 17 | 4,99 | 16 | 5,23 | 2.28 (0.48-10.78) | 0.30 | 2.19 (1.00-4.78) | 0.05 | . | . | . |
| FOLH1 ---- tag | rs2299650 | G/G | 26 | 43,33 | 9 | 28,13 | 142 | 41,64 | 122 | 39,87 | 1.00 (.-.) | . | 1.74 (0.74-4.08) | 0.20 | 0.40 | 0.99 | 0.73 |
| FOLH1 ---- tag |  | G/T | 28 | 46,67 | 18 | 56,25 | 151 | 44,28 | 151 | 49,35 | 1.25 (0.47-3.32) | 0.65 | 1.68 (0.72-3.92) | 0.23 | . | . | . |
| FOLH1 ---- tag |  | T/T | 6 | 10,00 | 5 | 15,63 | 48 | 14,08 | 33 | 10,78 | 1.56 (0.46-5.25) | 0.47 | 1.55 (0.63-3.82) | 0.34 | . | . | . |
| FOLH1 ---- tag | rs617528 | G/G | 48 | 80,00 | 22 | 68,75 | 269 | 78,89 | 243 | 79,41 | 1.00 (.-.) | . | 1.43 (0.87-2.34) | 0.16 | 0.86 | 1.00 | 0.86 |
| FOLH1 ---- tag |  | G/A | 10 | 16,67 | 9 | 28,13 | 67 | 19,65 | 61 | 19,93 | 1.29 (0.50-3.33) | 0.60 | 1.39 (0.80-2.40) | 0.24 | . | . | . |
| FOLH1 ---- tag |  | A/A | 2 | 3,33 | 1 | 3,13 | 5 | 1,47 | 2 | 0,65 | 0.54 (0.07-4.12) | 0.55 | 0.36 (0.05-2.73) | 0.32 | . | . | . |
| FOLH1 ---- tag | rs663877 | T/T | 50 | 83,33 | 22 | 68,75 | 273 | 80,06 | 233 | 76,14 | 1.00 (.-.) | . | 1.54 (0.89-2.69) | 0.13 | 0.54 | 0.99 | 0.73 |
| FOLH1 ---- tag |  | T/G | 10 | 16,67 | 10 | 31,25 | 64 | 18,77 | 68 | 22,22 | 1.53 (0.66-3.52) | 0.32 | 1.70 (0.93-3.08) | 0.08 | . | . | . |
| FOLH1 ---- tag |  | G/G | 0 | 0,00 | 0 | 0,00 | 4 | 1,17 | 5 | 1,63 | 1.96 (0.79-4.91) | 0.15 | 3.03 (1.08-8.47) | 0.03 | . | . | . |
| FOLH1 ---- tag | rs670776 | A/A | 38 | 63,33 | 18 | 56,25 | 213 | 62,46 | 192 | 62,75 | 1.00 (.-.) | . | 1.60 (0.86-2.96) | 0.14 | 0.43 | 0.99 | 0.73 |
| FOLH1 ---- tag |  | A/T | 22 | 36,67 | 12 | 37,50 | 111 | 32,55 | 98 | 32,03 | 1.20 (0.52-2.76) | 0.67 | 1.41 (0.74-2.67) | 0.29 | . | . | . |
| FOLH1 ---- tag |  | T/T | 0 | 0,00 | 2 | 6,25 | 17 | 4,99 | 16 | 5,23 | 2.33 (0.49-11.04) | 0.29 | 2.24 (1.02-4.89) | 0.04 | . | . | . |
| FOLH1 ---- tag | rs7124497 | G/G | 56 | 93,33 | 31 | 96,88 | 315 | 92,38 | 284 | 92,81 | 1.00 (.-.) | . | 1.40 (0.90-2.17) | 0.13 | 0.63 | 0.99 | 0.73 |
| FOLH1 ---- tag |  | G/A | 4 | 6,67 | 1 | 3,13 | 26 | 7,62 | 22 | 7,19 | 1.00 (.-.) | . | 1.40 (0.90-2.17) | 0.13 | . | . | . |
| FOLH1 ---- tag |  | A/A | 0 | 0,00 | 0 | 0,00 | 0 | 0,00 | 0 | 0,00 | 1.61 (0.21-12.22) | 0.65 | 1.30 (0.68-2.46) | 0.43 | . | . | . |
| FOLR1 ---- tag | rs651646 | T/T | 17 | 28,33 | 10 | 31,25 | 111 | 32,55 | 104 | 33,99 | 1.00 (.-.) | . | 1.76 (0.80-3.86) | 0.16 | 0.30 | 0.99 | 0.30 |
| FOLR1 ---- tag |  | T/A | 31 | 51,67 | 15 | 46,88 | 165 | 48,39 | 136 | 44,44 | 1.10 (0.42-2.92) | 0.84 | 1.51 (0.69-3.32) | 0.30 | . | . | . |
| FOLR1 ---- tag |  | A/A | 12 | 20,00 | 7 | 21,88 | 65 | 19,06 | 66 | 21,57 | 1.53 (0.52-4.55) | 0.44 | 1.46 (0.65-3.29) | 0.36 | . | . | . |
| FPGS ---- tag | rs10987746 | T/T | 17 | 28,33 | 7 | 21,88 | 108 | 31,67 | 81 | 26,47 | 1.00 (.-.) | . | 1.41 (0.60-3.30) | 0.43 | 0.35 | 0.99 | 0.52 |
| FPGS ---- tag |  | T/C | 26 | 43,33 | 18 | 56,25 | 167 | 48,97 | 150 | 49,02 | 1.23 (0.47-3.19) | 0.67 | 1.29 (0.56-2.97) | 0.55 | . | . | . |
| FPGS ---- tag |  | C/C | 17 | 28,33 | 7 | 21,88 | 66 | 19,35 | 75 | 24,51 | 0.59 (0.16-2.11) | 0.41 | 1.62 (0.69-3.82) | 0.27 | . | . | . |
| FPGS ---- tag | rs7033913 | T/T | 22 | 36,67 | 11 | 34,38 | 101 | 29,62 | 106 | 34,64 | 1.00 (.-.) | . | 1.57 (0.73-3.36) | 0.25 | 0.55 | 0.99 | 0.55 |
| FPGS ---- tag |  | T/C | 25 | 41,67 | 16 | 50,00 | 164 | 48,09 | 155 | 50,65 | 0.97 (0.39-2.38) | 0.95 | 1.39 (0.66-2.92) | 0.39 | . | . | . |
| FPGS ---- tag |  | C/C | 13 | 21,67 | 5 | 15,63 | 76 | 22,29 | 45 | 14,71 | 1.11 (0.32-3.80) | 0.87 | 1.12 (0.51-2.46) | 0.79 | . | . | . |
| FPGS ---- tag | rs7039798 | G/G | 19 | 31,67 | 7 | 21,88 | 123 | 36,07 | 86 | 28,10 | 1.00 (.-.) | . | 1.31 (0.56-3.06) | 0.53 | 0.27 | 0.99 | 0.52 |
| FPGS ---- tag |  | G/A | 25 | 41,67 | 19 | 59,38 | 163 | 47,80 | 158 | 51,63 | 1.31 (0.50-3.39) | 0.58 | 1.43 (0.62-3.29) | 0.40 | . | . | . |
| FPGS ---- tag |  | A/A | 16 | 26,67 | 6 | 18,75 | 55 | 16,13 | 62 | 20,26 | 0.61 (0.17-2.20) | 0.45 | 1.75 (0.74-4.13) | 0.20 | . | . | . |
| GGH ---- tag | rs10957264 | G/G | 41 | 68,33 | 25 | 78,13 | 232 | 68,04 | 220 | 71,90 | 1.00 (.-.) | . | 1.40 (0.85-2.29) | 0.18 | 0.87 | 1.00 | 0.97 |
| GGH ---- tag |  | G/T | 18 | 30,00 | 7 | 21,88 | 92 | 26,98 | 79 | 25,82 | 0.99 (0.39-2.53) | 0.99 | 1.38 (0.80-2.35) | 0.24 | . | . | . |
| GGH ---- tag |  | T/T | 1 | 1,67 | 0 | 0,00 | 17 | 4,99 | 7 | 2,29 | 0.00 (0.00-I) | 0.98 | 0.76 (0.30-1.93) | 0.56 | . | . | . |
| GGH ---- candidate literature | rs11545076 | T/T | 31 | 51,67 | 16 | 50,00 | 167 | 48,97 | 149 | 48,69 | 1.00 (.-.) | . | 1.11 (0.59-2.09) | 0.76 | 0.81 | 0.99 | 0.97 |
| GGH ---- candidate literature |  | T/G | 25 | 41,67 | 14 | 43,75 | 143 | 41,94 | 136 | 44,44 | 0.66 (0.28-1.55) | 0.34 | 1.27 (0.67-2.40) | 0.47 | . | . | . |
| GGH ---- candidate literature |  | G/G | 4 | 6,67 | 2 | 6,25 | 31 | 9,09 | 21 | 6,86 | 1.29 (0.28-5.89) | 0.74 | 0.70 (0.32-1.53) | 0.38 | . | . | . |
| GGH ---- candidate | rs11545077 | G/G | 33 | 55,00 | 18 | 56,25 | 181 | 53,08 | 162 | 52,94 | 1.00 (.-.) | . | 1.11 (0.62-1.99) | 0.73 | 0.74 | 0.99 | 0.97 |
| GGH ---- candidate |  | G/A | 23 | 38,33 | 12 | 37,50 | 132 | 38,71 | 130 | 42,48 | 0.62 (0.26-1.47) | 0.28 | 1.28 (0.70-2.33) | 0.42 | . | . | . |
| GGH ---- candidate |  | A/A | 4 | 6,67 | 2 | 6,25 | 28 | 8,21 | 14 | 4,58 | 1.29 (0.29-5.81) | 0.74 | 0.60 (0.26-1.36) | 0.22 | . | . | . |
| GGH ---- candidate | rs11545078 | C/C | 50 | 83,33 | 28 | 87,50 | 280 | 82,11 | 251 | 82,03 | 1.00 (.-.) | . | 1.39 (0.88-2.20) | 0.16 | 0.93 | 1.00 | 0.97 |
| GGH ---- candidate |  | C/T | 9 | 15,00 | 4 | 12,50 | 52 | 15,25 | 50 | 16,34 | 0.98 (0.29-3.32) | 0.98 | 1.42 (0.82-2.46) | 0.22 | . | . | . |
| GGH ---- candidate |  | T/T | 1 | 1,67 | 0 | 0,00 | 9 | 2,64 | 5 | 1,63 | 0.00 (0.00-I) | 0.98 | 0.66 (0.22-1.95) | 0.45 | . | . | . |
| GGH ---- tag | rs11995525 | G/G | 29 | 48,33 | 16 | 50,00 | 189 | 55,43 | 162 | 52,94 | 1.00 (.-.) | . | 1.42 (0.77-2.63) | 0.26 | 0.68 | 0.99 | 0.97 |
| GGH ---- tag |  | G/A | 26 | 43,33 | 12 | 37,50 | 137 | 40,18 | 121 | 39,54 | 0.96 (0.39-2.35) | 0.93 | 1.44 (0.77-2.67) | 0.25 | . | . | . |
| GGH ---- tag |  | A/A | 5 | 8,33 | 4 | 12,50 | 15 | 4,40 | 23 | 7,52 | 2.04 (0.63-6.61) | 0.23 | 2.18 (1.01-4.70) | 0.05 | . | . | . |
| GGH ---- tag | rs16930073 | G/G | 47 | 78,33 | 23 | 71,88 | 270 | 79,18 | 244 | 79,74 | 1.00 (.-.) | . | 1.41 (0.85-2.33) | 0.18 | 0.97 | 1.00 | 0.97 |
| GGH ---- tag |  | G/A | 12 | 20,00 | 9 | 28,13 | 63 | 18,48 | 61 | 19,93 | 1.20 (0.49-2.92) | 0.69 | 1.55 (0.88-2.73) | 0.13 | . | . | . |
| GGH ---- tag |  | A/A | 1 | 1,67 | 0 | 0,00 | 8 | 2,35 | 1 | 0,33 | 0.00 (0.00-5E257) | 0.97 | 0.65 (0.09-4.92) | 0.68 | . | . | . |
| GGH ---- tag | rs17194931 | G/G | 50 | 83,33 | 28 | 87,50 | 280 | 82,11 | 251 | 82,03 | 1.00 (.-.) | . | 1.39 (0.88-2.20) | 0.16 | 0.93 | 1.00 | 0.97 |
| GGH ---- tag |  | G/A | 9 | 15,00 | 4 | 12,50 | 52 | 15,25 | 50 | 16,34 | 0.98 (0.29-3.32) | 0.98 | 1.42 (0.82-2.46) | 0.22 | . | . | . |
| GGH ---- tag |  | A/A | 1 | 1,67 | 0 | 0,00 | 9 | 2,64 | 5 | 1,63 | 0.00 (0.00-I) | 0.98 | 0.66 (0.22-1.95) | 0.45 | . | . | . |
| GGH ---- candidate literature | rs1800909 | T/T | 31 | 51,67 | 16 | 50,00 | 166 | 48,68 | 149 | 48,69 | 1.00 (.-.) | . | 1.12 (0.60-2.12) | 0.72 | 0.85 | 1.00 | 0.97 |
| GGH ---- candidate literature |  | T/C | 25 | 41,67 | 14 | 43,75 | 145 | 42,52 | 136 | 44,44 | 0.66 (0.28-1.55) | 0.34 | 1.24 (0.66-2.36) | 0.51 | . | . | . |
| GGH ---- candidate literature |  | C/C | 4 | 6,67 | 2 | 6,25 | 30 | 8,80 | 21 | 6,86 | 1.29 (0.28-5.90) | 0.74 | 0.70 (0.32-1.53) | 0.37 | . | . | . |
| GGH ---- candidate literature | rs3758149 | C/C | 31 | 51,67 | 16 | 50,00 | 167 | 48,97 | 149 | 48,69 | 1.00 (.-.) | . | 1.11 (0.59-2.09) | 0.76 | 0.81 | 0.99 | 0.97 |
| GGH ---- candidate literature |  | C/T | 25 | 41,67 | 14 | 43,75 | 143 | 41,94 | 136 | 44,44 | 0.66 (0.28-1.55) | 0.34 | 1.27 (0.67-2.40) | 0.47 | . | . | . |
| GGH ---- candidate literature |  | T/T | 4 | 6,67 | 2 | 6,25 | 31 | 9,09 | 21 | 6,86 | 1.29 (0.28-5.89) | 0.74 | 0.70 (0.32-1.53) | 0.38 | . | . | . |
| GGH ---- tag | rs3780130 | A/A | 37 | 61,67 | 17 | 53,13 | 203 | 59,53 | 192 | 62,75 | 1.00 (.-.) | . | 1.56 (0.91-2.68) | 0.10 | 0.65 | 0.99 | 0.97 |
| GGH ---- tag |  | A/T | 19 | 31,67 | 14 | 43,75 | 120 | 35,19 | 105 | 34,31 | 1.41 (0.61-3.23) | 0.42 | 1.41 (0.81-2.44) | 0.23 | . | . | . |
| GGH ---- tag |  | T/T | 4 | 6,67 | 1 | 3,13 | 18 | 5,28 | 9 | 2,94 | 0.00 (0.00-3E240) | 0.97 | 1.74 (0.73-4.19) | 0.21 | . | . | . |
| GGH ---- tag | rs4446729 | C/C | 38 | 63,33 | 18 | 56,25 | 183 | 53,67 | 163 | 53,27 | 1.00 (.-.) | . | 1.31 (0.75-2.28) | 0.34 | 0.68 | 0.99 | 0.97 |
| GGH ---- tag |  | C/T | 19 | 31,67 | 11 | 34,38 | 139 | 40,76 | 119 | 38,89 | 0.90 (0.35-2.27) | 0.82 | 1.23 (0.70-2.17) | 0.47 | . | . | . |
| GGH ---- tag |  | T/T | 3 | 5,00 | 3 | 9,38 | 19 | 5,57 | 24 | 7,84 | 0.78 (0.21-2.82) | 0.70 | 1.53 (0.73-3.21) | 0.26 | . | . | . |
| GGH ---- tag | rs6472067 | C/C | 19 | 31,67 | 12 | 37,50 | 140 | 41,06 | 119 | 38,89 | 1.00 (.-.) | . | 1.50 (0.76-2.97) | 0.24 | 0.45 | 0.99 | 0.97 |
| GGH ---- tag |  | C/G | 29 | 48,33 | 13 | 40,63 | 155 | 45,45 | 141 | 46,08 | 1.16 (0.47-2.89) | 0.75 | 1.65 (0.85-3.22) | 0.14 | . | . | . |
| GGH ---- tag |  | G/G | 12 | 20,00 | 7 | 21,88 | 46 | 13,49 | 46 | 15,03 | 1.96 (0.64-5.95) | 0.24 | 1.75 (0.84-3.61) | 0.13 | . | . | . |
| GGH ---- tag | rs7010484 | T/T | 34 | 56,67 | 17 | 53,13 | 163 | 47,80 | 140 | 45,75 | 1.00 (.-.) | . | 1.20 (0.66-2.17) | 0.55 | 0.37 | 0.99 | 0.97 |
| GGH ---- tag |  | T/C | 20 | 33,33 | 11 | 34,38 | 140 | 41,06 | 126 | 41,18 | 0.92 (0.37-2.28) | 0.86 | 1.19 (0.65-2.17) | 0.58 | . | . | . |
| GGH ---- tag |  | C/C | 6 | 10,00 | 4 | 12,50 | 38 | 11,14 | 40 | 13,07 | 0.59 (0.19-1.89) | 0.38 | 1.32 (0.67-2.59) | 0.43 | . | . | . |
| GNMT ---- tag | rs1053538 | C/C | 21 | 35,00 | 10 | 31,25 | 84 | 24,63 | 80 | 26,14 | 1.00 (.-.) | . | 1.92 (0.82-4.50) | 0.13 | 0.78 | 0.99 | 0.88 |
| GNMT ---- tag |  | C/G | 27 | 45,00 | 17 | 53,13 | 173 | 50,73 | 153 | 50,00 | 1.82 (0.70-4.74) | 0.22 | 1.89 (0.82-4.33) | 0.13 | . | . | . |
| GNMT ---- tag |  | G/G | 12 | 20,00 | 5 | 15,63 | 84 | 24,63 | 73 | 23,86 | 1.08 (0.27-4.35) | 0.92 | 2.13 (0.90-5.03) | 0.09 | . | . | . |
| GNMT ---- tag | rs2296805 | G/G | 18 | 30,00 | 13 | 40,63 | 105 | 30,79 | 98 | 32,03 | 1.00 (.-.) | . | 1.45 (0.76-2.78) | 0.26 | 0.86 | 1.00 | 0.88 |
| GNMT ---- tag |  | G/T | 31 | 51,67 | 13 | 40,63 | 173 | 50,73 | 151 | 49,35 | 1.04 (0.45-2.39) | 0.93 | 1.28 (0.68-2.41) | 0.44 | . | . | . |
| GNMT ---- tag |  | T/T | 11 | 18,33 | 6 | 18,75 | 63 | 18,48 | 57 | 18,63 | 0.56 (0.12-2.58) | 0.46 | 1.17 (0.60-2.32) | 0.64 | . | . | . |
| GNMT ---- tag | rs6901782 | T/T | 40 | 66,67 | 21 | 65,63 | 264 | 77,42 | 241 | 78,76 | 1.00 (.-.) | . | 1.41 (0.84-2.38) | 0.20 | 0.88 | 1.00 | 0.88 |
| GNMT ---- tag |  | T/C | 18 | 30,00 | 11 | 34,38 | 73 | 21,41 | 59 | 19,28 | 1.38 (0.60-3.17) | 0.45 | 1.89 (1.05-3.38) | 0.03 | . | . | . |
| GNMT ---- tag |  | C/C | 2 | 3,33 | 0 | 0,00 | 4 | 1,17 | 6 | 1,96 | 0.00 (0.00-2E274) | 0.97 | 1.24 (0.45-3.46) | 0.68 | . | . | . |
| GNMT ---- tag | rs6927188 | A/A | 40 | 66,67 | 22 | 68,75 | 189 | 55,43 | 167 | 54,58 | 1.00 (.-.) | . | 1.46 (0.87-2.45) | 0.16 | 0.68 | 0.99 | 0.88 |
| GNMT ---- tag |  | A/G | 19 | 31,67 | 9 | 28,13 | 129 | 37,83 | 119 | 38,89 | 0.92 (0.38-2.24) | 0.85 | 1.25 (0.73-2.13) | 0.42 | . | . | . |
| GNMT ---- tag |  | G/G | 1 | 1,67 | 1 | 3,13 | 23 | 6,74 | 20 | 6,54 | 3.22 (0.42-24.91) | 0.26 | 1.67 (0.80-3.48) | 0.17 | . | . | . |
| MAT1A ---- tag | rs10887708 | G/G | 33 | 55,00 | 17 | 53,13 | 176 | 51,61 | 162 | 52,94 | 1.00 (.-.) | . | 1.64 (0.89-3.01) | 0.11 | 0.68 | 0.99 | 0.76 |
| MAT1A ---- tag |  | G/A | 23 | 38,33 | 11 | 34,38 | 129 | 37,83 | 117 | 38,24 | 1.80 (0.78-4.14) | 0.17 | 1.69 (0.91-3.14) | 0.10 | . | . | . |
| MAT1A ---- tag |  | A/A | 4 | 6,67 | 4 | 12,50 | 36 | 10,56 | 27 | 8,82 | 0.81 (0.17-3.79) | 0.79 | 1.62 (0.78-3.36) | 0.20 | . | . | . |
| MAT1A ---- tag | rs10887718 | T/T | 18 | 30,00 | 9 | 28,13 | 97 | 28,45 | 82 | 26,80 | 1.00 (.-.) | . | 2.64 (0.94-7.42) | 0.07 | 0.61 | 0.99 | 0.76 |
| MAT1A ---- tag |  | T/C | 27 | 45,00 | 14 | 43,75 | 182 | 53,37 | 151 | 49,35 | 2.70 (0.87-8.42) | 0.09 | 2.51 (0.90-6.98) | 0.08 | . | . | . |
| MAT1A ---- tag |  | C/C | 15 | 25,00 | 9 | 28,13 | 62 | 18,18 | 73 | 23,86 | 2.10 (0.62-7.16) | 0.24 | 3.56 (1.26-10.04) | 0.02 | . | . | . |
| MAT1A ---- tag | rs11202403 | C/C | 41 | 68,33 | 24 | 75,00 | 219 | 64,22 | 192 | 62,75 | 1.00 (.-.) | . | 1.41 (0.87-2.29) | 0.16 | 0.63 | 0.99 | 0.76 |
| MAT1A ---- tag |  | C/T | 16 | 26,67 | 7 | 21,88 | 104 | 30,50 | 104 | 33,99 | 1.21 (0.40-3.60) | 0.74 | 1.51 (0.92-2.50) | 0.11 | . | . | . |
| MAT1A ---- tag |  | T/T | 3 | 5,00 | 1 | 3,13 | 18 | 5,28 | 10 | 3,27 | 1.38 (0.18-10.48) | 0.76 | 0.97 (0.43-2.16) | 0.93 | . | . | . |
| MAT1A ---- tag | rs1832683 | C/C | 39 | 65,00 | 23 | 71,88 | 226 | 66,28 | 215 | 70,26 | 1.00 (.-.) | . | 1.26 (0.78-2.04) | 0.35 | 0.66 | 0.99 | 0.76 |
| MAT1A ---- tag |  | C/T | 18 | 30,00 | 6 | 18,75 | 104 | 30,50 | 82 | 26,80 | 0.44 (0.13-1.51) | 0.19 | 0.96 (0.57-1.62) | 0.88 | . | . | . |
| MAT1A ---- tag |  | T/T | 3 | 5,00 | 3 | 9,38 | 11 | 3,23 | 9 | 2,94 | 0.70 (0.16-3.16) | 0.65 | 1.07 (0.42-2.73) | 0.89 | . | . | . |
| MAT1A ---- tag | rs2236568 | C/C | 18 | 30,00 | 11 | 34,38 | 111 | 32,55 | 104 | 33,99 | 1.00 (.-.) | . | 1.28 (0.65-2.50) | 0.47 | 0.76 | 0.99 | 0.76 |
| MAT1A ---- tag |  | C/A | 33 | 55,00 | 13 | 40,63 | 150 | 43,99 | 154 | 50,33 | 0.86 (0.35-2.09) | 0.73 | 1.44 (0.75-2.77) | 0.28 | . | . | . |
| MAT1A ---- tag |  | A/A | 9 | 15,00 | 8 | 25,00 | 80 | 23,46 | 48 | 15,69 | 0.90 (0.30-2.69) | 0.86 | 0.82 (0.40-1.69) | 0.58 | . | . | . |
| MAT1A ---- tag | rs2236569 | A/A | 24 | 40,00 | 15 | 46,88 | 161 | 47,21 | 128 | 41,83 | 1.00 (.-.) | . | 1.37 (0.67-2.78) | 0.39 | 0.72 | 0.99 | 0.76 |
| MAT1A ---- tag |  | A/G | 29 | 48,33 | 10 | 31,25 | 147 | 43,11 | 140 | 45,75 | 1.26 (0.50-3.16) | 0.62 | 1.69 (0.83-3.44) | 0.15 | . | . | . |
| MAT1A ---- tag |  | G/G | 7 | 11,67 | 7 | 21,88 | 33 | 9,68 | 38 | 12,42 | 1.11 (0.38-3.19) | 0.85 | 1.81 (0.83-3.96) | 0.14 | . | . | . |
| MAT1A ---- tag | rs9421467 | G/G | 54 | 90,00 | 29 | 90,63 | 308 | 90,32 | 267 | 87,25 | 1.00 (.-.) | . | 1.46 (0.92-2.31) | 0.11 | 0.28 | 0.99 | 0.76 |
| MAT1A ---- tag |  | G/C | 5 | 8,33 | 3 | 9,38 | 32 | 9,38 | 38 | 12,42 | 2.50 (0.72-8.69) | 0.15 | 1.67 (0.95-2.95) | 0.07 | . | . | . |
| MAT1A ---- tag |  | C/C | 1 | 1,67 | 0 | 0,00 | 1 | 0,29 | 1 | 0,33 | 1.55 (0.21-11.58) | 0.67 | 2.27 (0.29-17.62) | 0.43 | . | . | . |
| MAT1A ---- tag | rs998765 | A/A | 20 | 33,33 | 10 | 31,25 | 89 | 26,10 | 82 | 26,80 | 1.00 (.-.) | . | 1.97 (0.93-4.20) | 0.08 | 0.36 | 0.99 | 0.76 |
| MAT1A ---- tag |  | A/T | 27 | 45,00 | 15 | 46,88 | 163 | 47,80 | 152 | 49,67 | 2.06 (0.83-5.15) | 0.12 | 2.04 (0.98-4.23) | 0.06 | . | . | . |
| MAT1A ---- tag |  | T/T | 13 | 21,67 | 7 | 21,88 | 89 | 26,10 | 72 | 23,53 | 1.41 (0.45-4.42) | 0.55 | 1.83 (0.86-3.91) | 0.12 | . | . | . |
| MAT1A ---- tag | rs998766 | C/C | 25 | 41,67 | 13 | 40,63 | 103 | 30,21 | 100 | 32,68 | 1.00 (.-.) | . | 1.60 (0.84-3.07) | 0.16 | 0.47 | 0.99 | 0.76 |
| MAT1A ---- tag |  | C/G | 24 | 40,00 | 12 | 37,50 | 167 | 48,97 | 157 | 51,31 | 1.32 (0.54-3.24) | 0.55 | 1.62 (0.87-3.05) | 0.13 | . | . | . |
| MAT1A ---- tag |  | G/G | 11 | 18,33 | 7 | 21,88 | 71 | 20,82 | 49 | 16,01 | 1.17 (0.40-3.44) | 0.78 | 1.27 (0.64-2.52) | 0.50 | . | . | . |
| MAT2B ---- tag | rs12655857 | G/G | 24 | 40,00 | 21 | 65,63 | 189 | 55,43 | 170 | 55,56 | 1.00 (.-.) | . | 0.80 (0.47-1.35) | 0.40 | 0.01 | 0.99 | 0.04 |
| MAT2B ---- tag |  | G/T | 31 | 51,67 | 9 | 28,13 | 128 | 37,54 | 119 | 38,89 | 0.36 (0.15-0.88) | 0.03 | 0.91 (0.53-1.55) | 0.72 | . | . | . |
| MAT2B ---- tag |  | T/T | 5 | 8,33 | 2 | 6,25 | 24 | 7,04 | 17 | 5,56 | 0.22 (0.03-1.67) | 0.14 | 0.78 (0.38-1.60) | 0.50 | . | . | . |
| MAT2B ---- tag | rs6869277 | C/C | 47 | 78,33 | 26 | 81,25 | 275 | 80,65 | 240 | 78,43 | 1.00 (.-.) | . | 1.32 (0.81-2.15) | 0.27 | 0.62 | 0.99 | 0.62 |
| MAT2B ---- tag |  | C/T | 12 | 20,00 | 6 | 18,75 | 61 | 17,89 | 61 | 19,93 | 0.88 (0.35-2.23) | 0.79 | 1.29 (0.73-2.27) | 0.39 | . | . | . |
| MAT2B ---- tag |  | T/T | 1 | 1,67 | 0 | 0,00 | 5 | 1,47 | 5 | 1,63 | 0.00 (0.00-5E300) | 0.98 | 2.44 (0.88-6.76) | 0.09 | . | . | . |
| MAT2B ---- tag | rs6874065 | A/A | 21 | 35,00 | 8 | 25,00 | 95 | 27,86 | 87 | 28,43 | 1.00 (.-.) | . | 2.00 (0.91-4.41) | 0.09 | 0.14 | 0.99 | 0.18 |
| MAT2B ---- tag |  | A/G | 29 | 48,33 | 15 | 46,88 | 160 | 46,92 | 144 | 47,06 | 1.45 (0.56-3.76) | 0.44 | 1.98 (0.91-4.32) | 0.09 | . | . | . |
| MAT2B ---- tag |  | G/G | 10 | 16,67 | 9 | 28,13 | 86 | 25,22 | 75 | 24,51 | 2.01 (0.66-6.06) | 0.22 | 1.67 (0.76-3.70) | 0.20 | . | . | . |
| MAT2B ---- tag | rs6882306 | T/T | 48 | 80,00 | 22 | 68,75 | 233 | 68,33 | 214 | 69,93 | 1.00 (.-.) | . | 1.67 (0.99-2.81) | 0.06 | 0.08 | 0.99 | 0.17 |
| MAT2B ---- tag |  | T/C | 12 | 20,00 | 9 | 28,13 | 102 | 29,91 | 81 | 26,47 | 2.23 (0.92-5.41) | 0.08 | 1.83 (1.03-3.23) | 0.04 | . | . | . |
| MAT2B ---- tag |  | C/C | 0 | 0,00 | 1 | 3,13 | 6 | 1,76 | 11 | 3,59 | 12.03 (1.49-97.17) | 0.02 | 3.63 (1.52-8.69) | 0.00 | . | . | . |
| MAT2B ---- tag | rs7721639 | T/T | 45 | 75,00 | 22 | 68,75 | 246 | 72,14 | 220 | 71,90 | 1.00 (.-.) | . | 1.65 (0.99-2.74) | 0.06 | 0.10 | 0.99 | 0.17 |
| MAT2B ---- tag |  | T/G | 14 | 23,33 | 9 | 28,13 | 82 | 24,05 | 80 | 26,14 | 2.22 (0.89-5.55) | 0.09 | 1.76 (1.01-3.07) | 0.05 | . | . | . |
| MAT2B ---- tag |  | G/G | 1 | 1,67 | 1 | 3,13 | 13 | 3,81 | 6 | 1,96 | 3.02 (0.39-23.67) | 0.29 | 1.38 (0.45-4.17) | 0.57 | . | . | . |
| MTHFD1 ---- tag | rs1256148 | G/G | 35 | 58,33 | 17 | 53,13 | 204 | 59,82 | 181 | 59,15 | 1.00 (.-.) | . | 1.64 (0.92-2.94) | 0.10 | 0.39 | 0.99 | 0.43 |
| MTHFD1 ---- tag |  | G/A | 24 | 40,00 | 12 | 37,50 | 122 | 35,78 | 107 | 34,97 | 1.12 (0.46-2.73) | 0.80 | 1.27 (0.70-2.30) | 0.43 | . | . | . |
| MTHFD1 ---- tag |  | A/A | 1 | 1,67 | 3 | 9,38 | 15 | 4,40 | 18 | 5,88 | 1.70 (0.47-6.16) | 0.42 | 2.22 (1.03-4.80) | 0.04 | . | . | . |
| MTHFD1 ---- tag | rs13329053 | T/T | 15 | 25,00 | 6 | 18,75 | 109 | 31,96 | 105 | 34,31 | 1.00 (.-.) | . | 1.88 (0.75-4.67) | 0.18 | 0.26 | 0.99 | 0.43 |
| MTHFD1 ---- tag |  | T/C | 32 | 53,33 | 16 | 50,00 | 165 | 48,39 | 141 | 46,08 | 1.38 (0.48-3.95) | 0.55 | 2.01 (0.81-4.98) | 0.13 | . | . | . |
| MTHFD1 ---- tag |  | C/C | 13 | 21,67 | 10 | 31,25 | 67 | 19,65 | 60 | 19,61 | 2.37 (0.75-7.48) | 0.14 | 2.27 (0.89-5.76) | 0.09 | . | . | . |
| MTHFD1 ---- candidate literature | rs2236224 | C/C | 21 | 35,00 | 7 | 21,88 | 132 | 38,71 | 128 | 41,83 | 1.00 (.-.) | . | 2.16 (0.94-4.96) | 0.07 | 0.08 | 0.99 | 0.43 |
| MTHFD1 ---- candidate literature |  | C/T | 32 | 53,33 | 16 | 50,00 | 154 | 45,16 | 133 | 43,46 | 1.77 (0.64-4.85) | 0.27 | 2.48 (1.08-5.70) | 0.03 | . | . | . |
| MTHFD1 ---- candidate literature |  | T/T | 7 | 11,67 | 9 | 28,13 | 55 | 16,13 | 45 | 14,71 | 3.41 (1.15-10.11) | 0.03 | 2.57 (1.07-6.16) | 0.03 | . | . | . |
| MTHFD1 ---- candidate | rs2236225 | C/C | 17 | 28,33 | 5 | 15,63 | 109 | 31,96 | 108 | 35,29 | 1.00 (.-.) | . | 2.08 (0.75-5.78) | 0.16 | 0.36 | 0.99 | 0.43 |
| MTHFD1 ---- candidate |  | C/T | 29 | 48,33 | 19 | 59,38 | 167 | 48,97 | 140 | 45,75 | 1.64 (0.53-5.08) | 0.39 | 2.15 (0.78-5.93) | 0.14 | . | . | . |
| MTHFD1 ---- candidate |  | T/T | 14 | 23,33 | 8 | 25,00 | 65 | 19,06 | 58 | 18,95 | 2.16 (0.62-7.59) | 0.23 | 2.47 (0.88-6.99) | 0.09 | . | . | . |
| MTHFD1 ---- tag | rs2281603 | A/A | 36 | 60,00 | 24 | 75,00 | 205 | 60,12 | 170 | 55,56 | 1.00 (.-.) | . | 1.23 (0.75-2.01) | 0.42 | 0.30 | 0.99 | 0.43 |
| MTHFD1 ---- tag |  | A/G | 22 | 36,67 | 6 | 18,75 | 121 | 35,48 | 117 | 38,24 | 0.73 (0.27-1.98) | 0.54 | 1.20 (0.72-1.99) | 0.48 | . | . | . |
| MTHFD1 ---- tag |  | G/G | 2 | 3,33 | 2 | 6,25 | 15 | 4,40 | 19 | 6,21 | 0.48 (0.06-3.70) | 0.48 | 1.58 (0.79-3.17) | 0.19 | . | . | . |
| MTHFD1 ---- candidate literature | rs8003379 | A/A | 34 | 56,67 | 18 | 56,25 | 193 | 56,60 | 178 | 58,17 | 1.00 (.-.) | . | 1.19 (0.67-2.09) | 0.56 | 0.43 | 0.99 | 0.43 |
| MTHFD1 ---- candidate literature |  | A/C | 22 | 36,67 | 13 | 40,63 | 127 | 37,24 | 102 | 33,33 | 0.86 (0.38-1.96) | 0.73 | 1.39 (0.78-2.48) | 0.27 | . | . | . |
| MTHFD1 ---- candidate literature |  | C/C | 4 | 6,67 | 1 | 3,13 | 21 | 6,16 | 26 | 8,50 | 1.03 (0.13-8.17) | 0.98 | 1.85 (0.93-3.69) | 0.08 | . | . | . |
| MTHFD2 ---- tag | rs10177833 | A/A | 19 | 31,67 | 7 | 21,88 | 111 | 32,55 | 95 | 31,05 | 1.00 (.-.) | . | 1.76 (0.75-4.13) | 0.19 | 0.19 | 0.99 | 0.54 |
| MTHFD2 ---- tag |  | A/C | 31 | 51,67 | 16 | 50,00 | 166 | 48,68 | 158 | 51,63 | 1.04 (0.38-2.90) | 0.94 | 1.64 (0.71-3.79) | 0.25 | . | . | . |
| MTHFD2 ---- tag |  | C/C | 10 | 16,67 | 9 | 28,13 | 64 | 18,77 | 53 | 17,32 | 1.91 (0.63-5.76) | 0.25 | 1.53 (0.64-3.69) | 0.34 | . | . | . |
| MTHFD2 ---- tag | rs702462 | T/T | 25 | 41,67 | 11 | 34,38 | 114 | 33,43 | 102 | 33,33 | 1.00 (.-.) | . | 1.68 (0.79-3.58) | 0.18 | 0.40 | 0.99 | 0.54 |
| MTHFD2 ---- tag |  | T/A | 25 | 41,67 | 14 | 43,75 | 164 | 48,09 | 155 | 50,65 | 1.24 (0.48-3.17) | 0.66 | 1.63 (0.78-3.41) | 0.19 | . | . | . |
| MTHFD2 ---- tag |  | A/A | 10 | 16,67 | 7 | 21,88 | 63 | 18,48 | 49 | 16,01 | 1.30 (0.44-3.90) | 0.64 | 1.32 (0.60-2.92) | 0.49 | . | . | . |
| MTHFD2 ---- candidate literature | rs702465 | A/A | 17 | 28,33 | 11 | 34,38 | 100 | 29,33 | 78 | 25,49 | 1.00 (.-.) | . | 1.00 (0.48-2.09) | 0.99 | 0.41 | 0.99 | 0.54 |
| MTHFD2 ---- candidate literature |  | A/T | 26 | 43,33 | 14 | 43,75 | 168 | 49,27 | 161 | 52,61 | 0.81 (0.32-2.05) | 0.66 | 1.29 (0.64-2.60) | 0.48 | . | . | . |
| MTHFD2 ---- candidate literature |  | T/T | 17 | 28,33 | 7 | 21,88 | 73 | 21,41 | 67 | 21,90 | 0.91 (0.31-2.64) | 0.86 | 1.43 (0.69-2.99) | 0.34 | . | . | . |
| MTHFD2 ---- candidate literature | rs7571842 | A/A | 18 | 30,00 | 6 | 18,75 | 103 | 30,21 | 85 | 27,78 | 1.00 (.-.) | . | 1.84 (0.73-4.66) | 0.20 | 0.44 | 0.99 | 0.54 |
| MTHFD2 ---- candidate literature |  | A/G | 28 | 46,67 | 17 | 53,13 | 169 | 49,56 | 164 | 53,59 | 1.32 (0.46-3.81) | 0.61 | 1.73 (0.70-4.28) | 0.24 | . | . | . |
| MTHFD2 ---- candidate literature |  | G/G | 14 | 23,33 | 9 | 28,13 | 69 | 20,23 | 57 | 18,63 | 1.37 (0.43-4.32) | 0.59 | 1.55 (0.60-3.98) | 0.36 | . | . | . |
| MTHFD2 ---- tag | rs7587117 | T/T | 28 | 46,67 | 12 | 37,50 | 151 | 44,28 | 123 | 40,20 | 1.00 (.-.) | . | 1.72 (0.82-3.62) | 0.15 | 0.34 | 0.99 | 0.54 |
| MTHFD2 ---- tag |  | T/C | 26 | 43,33 | 14 | 43,75 | 148 | 43,40 | 148 | 48,37 | 1.35 (0.54-3.38) | 0.52 | 1.77 (0.85-3.68) | 0.13 | . | . | . |
| MTHFD2 ---- tag |  | C/C | 6 | 10,00 | 6 | 18,75 | 42 | 12,32 | 35 | 11,44 | 1.60 (0.50-5.13) | 0.43 | 1.49 (0.66-3.36) | 0.34 | . | . | . |
| MTHFD2 ---- tag | rs828861 | C/C | 18 | 30,00 | 11 | 34,38 | 100 | 29,33 | 77 | 25,16 | 1.00 (.-.) | . | 1.12 (0.54-2.32) | 0.76 | 0.50 | 0.99 | 0.54 |
| MTHFD2 ---- tag |  | C/G | 26 | 43,33 | 15 | 46,88 | 166 | 48,68 | 165 | 53,92 | 0.95 (0.38-2.35) | 0.91 | 1.35 (0.67-2.72) | 0.41 | . | . | . |
| MTHFD2 ---- tag |  | G/G | 16 | 26,67 | 6 | 18,75 | 75 | 21,99 | 64 | 20,92 | 0.84 (0.27-2.59) | 0.76 | 1.41 (0.68-2.95) | 0.36 | . | . | . |
| MTHFD2 ---- tag | rs828863 | G/G | 52 | 86,67 | 27 | 84,38 | 274 | 80,35 | 257 | 83,99 | 1.00 (.-.) | . | 1.47 (0.92-2.34) | 0.11 | 0.54 | 0.99 | 0.54 |
| MTHFD2 ---- tag |  | G/A | 8 | 13,33 | 5 | 15,63 | 63 | 18,48 | 46 | 15,03 | 1.40 (0.47-4.15) | 0.54 | 1.27 (0.72-2.21) | 0.41 | . | . | . |
| MTHFD2 ---- tag |  | A/A | 0 | 0,00 | 0 | 0,00 | 4 | 1,17 | 3 | 0,98 | 2.58 (0.79-8.37) | 0.12 | 3.78 (1.07-13.30) | 0.04 | . | . | . |
| MTHFR ---- tag | rs1476413 | G/G | 32 | 53,33 | 17 | 53,13 | 175 | 51,32 | 167 | 54,58 | 1.00 (.-.) | . | 1.46 (0.80-2.67) | 0.22 | 0.36 | 0.99 | 0.75 |
| MTHFR ---- tag |  | G/A | 25 | 41,67 | 11 | 34,38 | 144 | 42,23 | 118 | 38,56 | 0.93 (0.39-2.25) | 0.87 | 1.47 (0.80-2.70) | 0.22 | . | . | . |
| MTHFR ---- tag |  | A/A | 3 | 5,00 | 4 | 12,50 | 22 | 6,45 | 21 | 6,86 | 2.46 (0.77-7.88) | 0.13 | 1.47 (0.69-3.15) | 0.32 | . | . | . |
| MTHFR ---- tag | rs17376328 | G/G | 55 | 91,67 | 27 | 84,38 | 299 | 87,68 | 259 | 84,64 | 1.00 (.-.) | . | 1.46 (0.91-2.35) | 0.12 | 0.50 | 0.99 | 0.75 |
| MTHFR ---- tag |  | G/A | 5 | 8,33 | 5 | 15,63 | 39 | 11,44 | 45 | 14,71 | 1.39 (0.50-3.83) | 0.52 | 1.56 (0.87-2.77) | 0.13 | . | . | . |
| MTHFR ---- tag |  | A/A | 0 | 0,00 | 0 | 0,00 | 3 | 0,88 | 2 | 0,65 | 0.54 (0.13-2.27) | 0.40 | 0.79 (0.18-3.51) | 0.76 | . | . | . |
| MTHFR ---- tag | rs17421462 | G/G | 51 | 85,00 | 26 | 81,25 | 293 | 85,92 | 261 | 85,29 | 1.00 (.-.) | . | 1.39 (0.86-2.26) | 0.18 | 0.99 | 1.00 | 0.99 |
| MTHFR ---- tag |  | G/A | 9 | 15,00 | 6 | 18,75 | 47 | 13,78 | 40 | 13,07 | 1.20 (0.44-3.29) | 0.72 | 1.41 (0.77-2.58) | 0.26 | . | . | . |
| MTHFR ---- tag |  | A/A | 0 | 0,00 | 0 | 0,00 | 1 | 0,29 | 5 | 1,63 | 3.40 (1.32-8.78) | 0.01 | 4.74 (1.69-13.29) | 0.00 | . | . | . |
| MTHFR ---- candidate | rs1801131 | A/A | 26 | 43,33 | 15 | 46,88 | 155 | 45,45 | 147 | 48,04 | 1.00 (.-.) | . | 1.59 (0.83-3.08) | 0.17 | 0.14 | 0.99 | 0.48 |
| MTHFR ---- candidate |  | A/C | 31 | 51,67 | 13 | 40,63 | 152 | 44,57 | 127 | 41,50 | 0.97 (0.41-2.33) | 0.95 | 1.50 (0.78-2.89) | 0.23 | . | . | . |
| MTHFR ---- candidate |  | C/C | 3 | 5,00 | 4 | 12,50 | 34 | 9,97 | 32 | 10,46 | 4.24 (1.30-13.79) | 0.02 | 1.54 (0.72-3.30) | 0.26 | . | . | . |
| MTHFR ---- candidate | rs1801133 | C/C | 22 | 36,67 | 12 | 37,50 | 146 | 42,82 | 134 | 43,79 | 1.00 (.-.) | . | 1.14 (0.60-2.18) | 0.69 | 0.48 | 0.99 | 0.75 |
| MTHFR ---- candidate |  | C/T | 34 | 56,67 | 18 | 56,25 | 155 | 45,45 | 140 | 45,75 | 0.56 (0.25-1.26) | 0.16 | 0.86 (0.45-1.63) | 0.64 | . | . | . |
| MTHFR ----candidate |  | T/T | 4 | 6,67 | 2 | 6,25 | 40 | 11,73 | 32 | 10,46 | 0.86 (0.11-6.74) | 0.89 | 0.96 (0.46-2.02) | 0.92 | . | . | . |
| MTHFR ---- tag | rs2066471 | G/G | 42 | 70,00 | 22 | 68,75 | 238 | 69,79 | 216 | 70,59 | 1.00 (.-.) | . | 1.41 (0.84-2.39) | 0.20 | 0.78 | 0.99 | 0.88 |
| MTHFR ---- tag |  | G/A | 17 | 28,33 | 9 | 28,13 | 97 | 28,45 | 82 | 26,80 | 1.27 (0.53-3.03) | 0.59 | 1.69 (0.97-2.96) | 0.06 | . | . | . |
| MTHFR ---- tag |  | A/A | 1 | 1,67 | 1 | 3,13 | 6 | 1,76 | 8 | 2,61 | 2.06 (0.27-15.90) | 0.49 | 1.98 (0.79-4.98) | 0.14 | . | . | . |
| MTHFR ---- tag | rs4846047 | G/G | 30 | 50,00 | 15 | 46,88 | 171 | 50,15 | 155 | 50,65 | 1.00 (.-.) | . | 1.39 (0.72-2.72) | 0.33 | 0.69 | 0.99 | 0.88 |
| MTHFR ---- tag |  | G/C | 26 | 43,33 | 15 | 46,88 | 150 | 43,99 | 128 | 41,83 | 1.05 (0.45-2.45) | 0.90 | 1.56 (0.80-3.04) | 0.19 | . | . | . |
| MTHFR ---- tag |  | C/C | 4 | 6,67 | 2 | 6,25 | 20 | 5,87 | 23 | 7,52 | 2.99 (0.64-13.93) | 0.16 | 1.92 (0.86-4.28) | 0.11 | . | . | . |
| MTHFR ---- tag | rs4846049 | G/G | 26 | 43,33 | 14 | 43,75 | 151 | 44,28 | 145 | 47,39 | 1.00 (.-.) | . | 1.62 (0.81-3.23) | 0.17 | 0.12 | 0.99 | 0.48 |
| MTHFR ---- tag |  | G/T | 31 | 51,67 | 13 | 40,63 | 156 | 45,75 | 129 | 42,16 | 0.98 (0.40-2.40) | 0.96 | 1.51 (0.75-3.00) | 0.25 | . | . | . |
| MTHFR ---- tag |  | T/T | 3 | 5,00 | 5 | 15,63 | 34 | 9,97 | 32 | 10,46 | 3.44 (1.12-10.58) | 0.03 | 1.61 (0.73-3.54) | 0.24 | . | . | . |
| MTHFR ---- tag | rs7538516 | T/T | 23 | 38,33 | 12 | 37,50 | 121 | 35,48 | 112 | 36,60 | 1.00 (.-.) | . | 1.46 (0.70-3.05) | 0.31 | 0.16 | 0.99 | 0.48 |
| MTHFR ---- tag |  | T/C | 30 | 50,00 | 13 | 40,63 | 177 | 51,91 | 150 | 49,02 | 0.80 (0.31-2.06) | 0.65 | 1.48 (0.72-3.07) | 0.29 | . | . | . |
| MTHFR ---- tag |  | C/C | 7 | 11,67 | 7 | 21,88 | 43 | 12,61 | 44 | 14,38 | 3.04 (1.09-8.49) | 0.03 | 1.69 (0.77-3.68) | 0.19 | . | . | . |
| MTR ---- tag | rs10733117 | A/A | 24 | 40,00 | 11 | 34,38 | 115 | 33,72 | 119 | 38,89 | 1.00 (.-.) | . | 0.99 (0.45-2.22) | 0.99 | 0.35 | 0.99 | 0.88 |
| MTR ---- tag |  | A/G | 24 | 40,00 | 13 | 40,63 | 175 | 51,32 | 139 | 45,42 | 0.69 (0.27-1.79) | 0.45 | 0.94 (0.42-2.06) | 0.87 | . | . | . |
| MTR ---- tag |  | G/G | 12 | 20,00 | 8 | 25,00 | 51 | 14,96 | 48 | 15,69 | 0.50 (0.16-1.53) | 0.23 | 0.88 (0.38-2.05) | 0.77 | . | . | . |
| MTR ---- tag | rs12129440 | G/G | 35 | 58,33 | 21 | 65,63 | 179 | 52,49 | 183 | 59,80 | 1.00 (.-.) | . | 1.46 (0.85-2.48) | 0.17 | 0.69 | 0.99 | 0.98 |
| MTR ---- tag |  | G/A | 21 | 35,00 | 10 | 31,25 | 141 | 41,35 | 105 | 34,31 | 0.95 (0.40-2.25) | 0.90 | 1.27 (0.74-2.21) | 0.39 | . | . | . |
| MTR ---- tag |  | A/A | 4 | 6,67 | 1 | 3,13 | 21 | 6,16 | 18 | 5,88 | 1.37 (0.18-10.60) | 0.76 | 1.11 (0.48-2.56) | 0.80 | . | . | . |
| MTR ---- candidate | rs1805087 | A/A | 43 | 71,67 | 23 | 71,88 | 229 | 67,16 | 208 | 67,97 | 1.00 (.-.) | . | 1.43 (0.87-2.33) | 0.16 | 0.98 | 1.00 | 0.98 |
| MTR ---- candidate |  | A/G | 13 | 21,67 | 8 | 25,00 | 98 | 28,74 | 87 | 28,43 | 1.13 (0.45-2.87) | 0.79 | 1.27 (0.75-2.16) | 0.38 | . | . | . |
| MTR ---- candidate |  | G/G | 4 | 6,67 | 1 | 3,13 | 14 | 4,11 | 11 | 3,59 | 0.00 (0.00-I) | 0.98 | 1.12 (0.49-2.56) | 0.78 | . | . | . |
| MTR ---- tag | rs3890786 | C/C | 25 | 41,67 | 12 | 37,50 | 120 | 35,19 | 102 | 33,33 | 1.00 (.-.) | . | 1.90 (0.94-3.85) | 0.07 | 0.25 | 0.99 | 0.88 |
| MTR ---- tag |  | C/T | 20 | 33,33 | 14 | 43,75 | 162 | 47,51 | 140 | 45,75 | 2.16 (0.88-5.30) | 0.09 | 2.13 (1.06-4.25) | 0.03 | . | . | . |
| MTR ---- tag |  | T/T | 15 | 25,00 | 6 | 18,75 | 59 | 17,30 | 64 | 20,92 | 2.23 (0.74-6.77) | 0.16 | 2.44 (1.18-5.05) | 0.02 | . | . | . |
| MTR ---- tag | rs4659727 | A/A | 43 | 71,67 | 23 | 71,88 | 228 | 66,86 | 208 | 67,97 | 1.00 (.-.) | . | 1.42 (0.87-2.33) | 0.16 | 0.97 | 1.00 | 0.98 |
| MTR ---- tag |  | A/G | 13 | 21,67 | 8 | 25,00 | 98 | 28,74 | 88 | 28,76 | 1.13 (0.45-2.88) | 0.79 | 1.29 (0.76-2.20) | 0.34 | . | . | . |
| MTR ---- tag |  | G/G | 4 | 6,67 | 1 | 3,13 | 15 | 4,40 | 10 | 3,27 | 0.00 (0.00-I) | 0.98 | 0.92 (0.39-2.19) | 0.85 | . | . | . |
| MTRR ---- candidate literature/tag | rs10380 | C/C | 50 | 83,33 | 26 | 81,25 | 290 | 85,04 | 255 | 83,33 | 1.00 (.-.) | . | 1.37 (0.86-2.21) | 0.19 | 1.00 | 1.00 | 1.00 |
| MTRR ---- candidate literature/tag |  | C/T | 10 | 16,67 | 6 | 18,75 | 51 | 14,96 | 50 | 16,34 | 1.31 (0.46-3.76) | 0.61 | 1.71 (0.99-2.97) | 0.06 | . | . | . |
| MTRR ---- candidate literature/tag |  | T/T | 0 | 0,00 | 0 | 0,00 | 0 | 0,00 | 1 | 0,33 | 36.63 (4.53-296.5) | 0.00 | 50.36 (5.97-424.9) | <.001 | . | . | . |
| MTRR ---- tag | rs10475399 | G/G | 26 | 43,33 | 11 | 34,38 | 147 | 43,11 | 133 | 43,46 | 1.00 (.-.) | . | 1.41 (0.73-2.72) | 0.31 | 0.83 | 0.99 | 1.00 |
| MTRR ---- tag |  | G/A | 25 | 41,67 | 17 | 53,13 | 153 | 44,87 | 137 | 44,77 | 1.09 (0.45-2.62) | 0.85 | 1.53 (0.79-2.96) | 0.21 | . | . | . |
| MTRR ---- tag |  | A/A | 9 | 15,00 | 4 | 12,50 | 41 | 12,02 | 36 | 11,76 | 1.17 (0.36-3.82) | 0.79 | 1.35 (0.65-2.82) | 0.43 | . | . | . |
| MTRR ---- tag | rs11134265 | C/C | 23 | 38,33 | 12 | 37,50 | 153 | 44,87 | 137 | 44,77 | 1.00 (.-.) | . | 1.38 (0.72-2.66) | 0.34 | 0.86 | 1.00 | 1.00 |
| MTRR ---- tag |  | C/T | 30 | 50,00 | 17 | 53,13 | 145 | 42,52 | 136 | 44,44 | 1.04 (0.44-2.49) | 0.92 | 1.51 (0.78-2.92) | 0.22 | . | . | . |
| MTRR ---- tag |  | T/T | 7 | 11,67 | 3 | 9,38 | 43 | 12,61 | 33 | 10,78 | 1.16 (0.31-4.29) | 0.83 | 1.28 (0.61-2.70) | 0.52 | . | . | . |
| MTRR ---- tag | rs13181011 | T/T | 35 | 58,33 | 20 | 62,50 | 214 | 62,76 | 194 | 63,40 | 1.00 (.-.) | . | 1.28 (0.77-2.13) | 0.34 | 0.98 | 1.00 | 1.00 |
| MTRR ---- tag |  | T/C | 23 | 38,33 | 10 | 31,25 | 117 | 34,31 | 100 | 32,68 | 0.67 (0.26-1.73) | 0.41 | 1.33 (0.78-2.26) | 0.29 | . | . | . |
| MTRR ---- tag |  | C/C | 2 | 3,33 | 2 | 6,25 | 10 | 2,93 | 12 | 3,92 | 4.49 (0.56-36.36) | 0.16 | 0.70 (0.31-1.59) | 0.39 | . | . | . |
| MTRR ---- tag | rs161869 | C/C | 22 | 36,67 | 10 | 31,25 | 112 | 32,84 | 106 | 34,64 | 1.00 (.-.) | . | 1.06 (0.48-2.35) | 0.88 | 0.82 | 0.99 | 1.00 |
| MTRR ---- tag |  | C/T | 28 | 46,67 | 14 | 43,75 | 178 | 52,20 | 148 | 48,37 | 0.69 (0.27-1.77) | 0.44 | 1.17 (0.54-2.55) | 0.69 | . | . | . |
| MTRR ---- tag |  | T/T | 10 | 16,67 | 8 | 25,00 | 51 | 14,96 | 52 | 16,99 | 1.37 (0.45-4.13) | 0.58 | 1.68 (0.74-3.79) | 0.22 | . | . | . |
| MTRR ---- tagged by rs162039 | rs162036 | A/A | 49 | 81,67 | 24 | 75,00 | 275 | 80,65 | 244 | 79,74 | 1.00 (.-.) | . | 1.41 (0.87-2.28) | 0.17 | 0.79 | 0.99 | 1.00 |
| MTRR ---- tagged by rs162039 |  | A/G | 11 | 18,33 | 8 | 25,00 | 64 | 18,77 | 59 | 19,28 | 1.52 (0.57-4.04) | 0.41 | 1.79 (1.04-3.09) | 0.04 | . | . | . |
| MTRR ---- tagged by rs162039 |  | G/G | 0 | 0,00 | 0 | 0,00 | 2 | 0,59 | 3 | 0,98 | 2.26 (0.70-7.28) | 0.17 | 3.17 (0.91-11.09) | 0.07 | . | . | . |
| MTRR ---- tag | rs162039 | C/C | 49 | 81,67 | 24 | 75,00 | 276 | 80,94 | 244 | 79,74 | 1.00 (.-.) | . | 1.41 (0.87-2.28) | 0.17 | 0.79 | 0.99 | 1.00 |
| MTRR ---- tag |  | C/T | 11 | 18,33 | 8 | 25,00 | 63 | 18,48 | 59 | 19,28 | 1.52 (0.57-4.04) | 0.41 | 1.79 (1.04-3.09) | 0.04 | . | . | . |
| MTRR ---- tag |  | T/T | 0 | 0,00 | 0 | 0,00 | 2 | 0,59 | 3 | 0,98 | 2.26 (0.70-7.28) | 0.17 | 3.17 (0.91-11.09) | 0.07 | . | . | . |
| MTRR ---- tag | rs162270 | G/G | 41 | 68,33 | 24 | 75,00 | 245 | 71,85 | 217 | 70,92 | 1.00 (.-.) | . | 1.24 (0.76-2.03) | 0.40 | 0.55 | 0.99 | 1.00 |
| MTRR ---- tag |  | G/T | 14 | 23,33 | 6 | 18,75 | 88 | 25,81 | 78 | 25,49 | 0.64 (0.21-1.91) | 0.42 | 1.16 (0.67-2.01) | 0.58 | . | . | . |
| MTRR ---- tag |  | T/T | 5 | 8,33 | 2 | 6,25 | 8 | 2,35 | 11 | 3,59 | 0.72 (0.16-3.14) | 0.66 | 1.18 (0.52-2.68) | 0.69 | . | . | . |
| MTRR ---- candidate | rs16879334 | C/C | 58 | 96,67 | 29 | 90,63 | 319 | 93,55 | 288 | 94,12 | 1.00 (.-.) | . | 1.49 (0.94-2.34) | 0.09 | 0.18 | 0.99 | 1.00 |
| MTRR ---- candidate |  | C/G | 2 | 3,33 | 3 | 9,38 | 22 | 6,45 | 18 | 5,88 | 1.00 (.-.) | . | 1.49 (0.94-2.34) | 0.09 | . | . | . |
| MTRR ---- candidate |  | G/G | . | . | . | . | . | . | . | . | 2.11 (0.62-7.23) | 0.23 | 1.17 (0.59-2.29) | 0.66 | . | . | . |
| MTRR ---- singleton | rs1801394 | G/G | 19 | 31,67 | 10 | 31,25 | 112 | 32,84 | 87 | 28,43 | 1.00 (.-.) | . | 1.16 (0.55-2.45) | 0.69 | 0.97 | 1.00 | 1.00 |
| MTRR ---- singleton |  | G/A | 27 | 45,00 | 17 | 53,13 | 157 | 46,04 | 169 | 55,23 | 0.88 (0.35-2.21) | 0.79 | 1.48 (0.71-3.06) | 0.30 | . | . | . |
| MTRR ---- singleton |  | A/A | 14 | 23,33 | 5 | 15,63 | 72 | 21,11 | 50 | 16,34 | 1.02 (0.33-3.16) | 0.98 | 1.08 (0.50-2.35) | 0.84 | . | . | . |
| MTRR ---- tag | rs1802059 | G/G | 26 | 43,33 | 17 | 53,13 | 131 | 38,42 | 118 | 38,56 | 1.00 (.-.) | . | 1.31 (0.73-2.36) | 0.36 | 0.99 | 1.00 | 1.00 |
| MTRR ---- tag |  | G/A | 26 | 43,33 | 12 | 37,50 | 161 | 47,21 | 143 | 46,73 | 0.72 (0.31-1.69) | 0.45 | 1.21 (0.68-2.15) | 0.51 | . | . | . |
| MTRR ---- tag |  | A/A | 8 | 13,33 | 3 | 9,38 | 49 | 14,37 | 45 | 14,71 | 1.15 (0.25-5.18) | 0.86 | 1.01 (0.53-1.93) | 0.98 | . | . | . |
| MTRR ---- tag | rs2077744 | T/T | 40 | 66,67 | 24 | 75,00 | 250 | 73,31 | 226 | 73,86 | 1.00 (.-.) | . | 1.23 (0.74-2.04) | 0.42 | 0.55 | 0.99 | 1.00 |
| MTRR ---- tag |  | T/C | 17 | 28,33 | 7 | 21,88 | 84 | 24,63 | 71 | 23,20 | 0.67 (0.26-1.72) | 0.40 | 1.20 (0.69-2.11) | 0.51 | . | . | . |
| MTRR ---- tag |  | C/C | 3 | 5,00 | 1 | 3,13 | 7 | 2,05 | 9 | 2,94 | 1.11 (0.15-8.46) | 0.92 | 1.44 (0.61-3.39) | 0.41 | . | . | . |
| MTRR ---- candidate | rs2287780 | C/C | 58 | 96,67 | 29 | 90,63 | 319 | 93,55 | 288 | 94,12 | 1.00 (.-.) | . | 1.49 (0.94-2.34) | 0.09 | 0.18 | 0.99 | 1.00 |
| MTRR ---- candidate |  | C/T | 2 | 3,33 | 3 | 9,38 | 22 | 6,45 | 18 | 5,88 | 1.00 (.-.) | . | 1.49 (0.94-2.34) | 0.09 | . | . | . |
| MTRR ---- candidate |  | T/T | . | . | . | . | . | . | . | . | 2.11 (0.62-7.23) | 0.23 | 1.17 (0.59-2.29) | 0.66 | . | . | . |
| MTRR ---- candidate | rs2303080 | T/T | 58 | 96,67 | 29 | 90,63 | 319 | 93,55 | 289 | 94,44 | 1.00 (.-.) | . | 1.49 (0.95-2.35) | 0.09 | 0.17 | 0.99 | 1.00 |
| MTRR ---- candidate |  | T/A | 2 | 3,33 | 3 | 9,38 | 22 | 6,45 | 17 | 5,56 | 1.00 (.-.) | . | 1.49 (0.95-2.35) | 0.09 | . | . | . |
| MTRR ---- candidate |  | A/A | . | . | . | . | . | . | . | . | 2.11 (0.62-7.22) | 0.23 | 1.11 (0.56-2.22) | 0.76 | . | . | . |
| MTRR ---- tag | rs7715062 | G/G | 21 | 35,00 | 13 | 40,63 | 114 | 33,43 | 104 | 33,99 | 1.00 (.-.) | . | 1.53 (0.80-2.93) | 0.20 | 0.75 | 0.99 | 1.00 |
| MTRR ---- tag |  | G/T | 28 | 46,67 | 16 | 50,00 | 171 | 50,15 | 148 | 48,37 | 1.00 (0.43-2.30) | 1.00 | 1.32 (0.70-2.49) | 0.39 | . | . | . |
| MTRR ---- tag |  | T/T | 11 | 18,33 | 3 | 9,38 | 56 | 16,42 | 54 | 17,65 | 1.06 (0.23-4.89) | 0.94 | 1.29 (0.65-2.57) | 0.46 | . | . | . |
| MTRR ---- tag | rs9282787 | T/T | 37 | 61,67 | 21 | 65,63 | 216 | 63,34 | 196 | 64,05 | 1.00 (.-.) | . | 1.31 (0.79-2.18) | 0.29 | 0.93 | 1.00 | 1.00 |
| MTRR ---- tag |  | T/C | 20 | 33,33 | 9 | 28,13 | 115 | 33,72 | 98 | 32,03 | 0.77 (0.30-1.97) | 0.59 | 1.41 (0.83-2.39) | 0.21 | . | . | . |
| MTRR ---- tag |  | C/C | 3 | 5,00 | 2 | 6,25 | 10 | 2,93 | 12 | 3,92 | 2.28 (0.28-18.34) | 0.44 | 0.72 (0.32-1.65) | 0.44 | . | . | . |
| MTRR ---- candidate literature | rs9332 | C/C | 49 | 81,67 | 24 | 75,00 | 276 | 80,94 | 244 | 79,74 | 1.00 (.-.) | . | 1.41 (0.87-2.28) | 0.17 | 0.79 | 0.99 | 1.00 |
| MTRR ---- candidate literature |  | C/T | 11 | 18,33 | 8 | 25,00 | 63 | 18,48 | 59 | 19,28 | 1.52 (0.57-4.04) | 0.41 | 1.79 (1.04-3.09) | 0.04 | . | . | . |
| MTRR ---- candidate literature |  | T/T | 0 | 0,00 | 0 | 0,00 | 2 | 0,59 | 3 | 0,98 | 2.26 (0.70-7.28) | 0.17 | 3.17 (0.91-11.09) | 0.07 | . | . | . |
| NFKB1 ---- NA | rs1609798 | C/C | 19 | 31,67 | 17 | 53,13 | 163 | 47,80 | 141 | 46,08 | 1.00 (.-.) | . | 1.20 (0.69-2.08) | 0.51 | 0.20 | 0.99 | 0.38 |
| NFKB1 ---- NA |  | C/T | 30 | 50,00 | 13 | 40,63 | 148 | 43,40 | 135 | 44,12 | 1.11 (0.47-2.58) | 0.82 | 1.32 (0.75-2.31) | 0.33 | . | . | . |
| NFKB1 ---- NA |  | T/T | 11 | 18,33 | 2 | 6,25 | 30 | 8,80 | 30 | 9,80 | 0.25 (0.03-1.96) | 0.19 | 1.42 (0.73-2.75) | 0.30 | . | . | . |
| NFKB1 ---- tag | rs230540 | T/T | 17 | 28,33 | 15 | 46,88 | 139 | 40,76 | 130 | 42,48 | 1.00 (.-.) | . | 1.11 (0.63-1.97) | 0.72 | 0.13 | 0.99 | 0.31 |
| NFKB1 ---- tag |  | T/C | 28 | 46,67 | 15 | 46,88 | 161 | 47,21 | 142 | 46,41 | 0.89 (0.39-2.04) | 0.79 | 1.20 (0.68-2.14) | 0.53 | . | . | . |
| NFKB1 ---- tag |  | C/C | 15 | 25,00 | 2 | 6,25 | 41 | 12,02 | 34 | 11,11 | 0.18 (0.02-1.37) | 0.10 | 0.96 (0.50-1.86) | 0.91 | . | . | . |
| NFKB1 ---- tag | rs230541 | A/A | 14 | 23,33 | 11 | 34,38 | 111 | 32,55 | 106 | 34,64 | 1.00 (.-.) | . | 1.14 (0.59-2.22) | 0.70 | 0.23 | 0.99 | 0.38 |
| NFKB1 ---- tag |  | A/G | 27 | 45,00 | 15 | 46,88 | 167 | 48,97 | 153 | 50,00 | 1.08 (0.45-2.57) | 0.87 | 1.29 (0.67-2.50) | 0.45 | . | . | . |
| NFKB1 ---- tag |  | G/G | 19 | 31,67 | 6 | 18,75 | 63 | 18,48 | 47 | 15,36 | 0.46 (0.14-1.51) | 0.20 | 1.15 (0.57-2.32) | 0.70 | . | . | . |
| NFKB1 ---- NA | rs230547 | C/C | 52 | 86,67 | 26 | 81,25 | 267 | 78,30 | 251 | 82,03 | 1.00 (.-.) | . | 1.47 (0.91-2.36) | 0.12 | 0.54 | 0.99 | 0.55 |
| NFKB1 ---- NA |  | C/T | 8 | 13,33 | 6 | 18,75 | 73 | 21,41 | 51 | 16,67 | 1.19 (0.44-3.23) | 0.73 | 1.14 (0.64-2.01) | 0.66 | . | . | . |
| NFKB1 ---- NA |  | T/T | 0 | 0,00 | 0 | 0,00 | 1 | 0,29 | 4 | 1,31 | 1.36 (0.48-3.85) | 0.56 | 2.00 (0.65-6.15) | 0.23 | . | . | . |
| NFKB1 ---- tag | rs3774934 | G/G | 51 | 85,00 | 25 | 78,13 | 261 | 76,54 | 245 | 80,07 | 1.00 (.-.) | . | 1.48 (0.92-2.39) | 0.11 | 0.55 | 0.99 | 0.55 |
| NFKB1 ---- tag |  | G/A | 9 | 15,00 | 7 | 21,88 | 78 | 22,87 | 55 | 17,97 | 1.20 (0.44-3.25) | 0.72 | 1.11 (0.63-1.94) | 0.72 | . | . | . |
| NFKB1 ---- tag |  | A/A | 0 | 0,00 | 0 | 0,00 | 2 | 0,59 | 6 | 1,96 | 1.42 (0.62-3.26) | 0.40 | 2.11 (0.82-5.40) | 0.12 | . | . | . |
| NFKB1 ---- tag | rs3774968 | G/G | 29 | 48,33 | 10 | 31,25 | 108 | 31,67 | 91 | 29,74 | 1.00 (.-.) | . | 2.91 (1.25-6.82) | 0.01 | 0.04 | 0.99 | 0.28 |
| NFKB1 ---- tag |  | G/A | 21 | 35,00 | 16 | 50,00 | 176 | 51,61 | 158 | 51,63 | 2.67 (0.99-7.17) | 0.05 | 2.53 (1.09-5.91) | 0.03 | . | . | . |
| NFKB1 ---- tag |  | A/A | 10 | 16,67 | 6 | 18,75 | 57 | 16,72 | 57 | 18,63 | 2.56 (0.82-8.04) | 0.11 | 2.46 (1.02-5.95) | 0.05 | . | . | . |
| NFKB1 ---- NA | rs4648022 | C/C | 49 | 81,67 | 28 | 87,50 | 291 | 85,34 | 260 | 84,97 | 1.00 (.-.) | . | 1.29 (0.80-2.06) | 0.29 | 0.47 | 0.99 | 0.55 |
| NFKB1 ---- NA |  | C/T | 11 | 18,33 | 4 | 12,50 | 49 | 14,37 | 43 | 14,05 | 0.99 (0.33-2.93) | 0.98 | 1.91 (1.09-3.36) | 0.02 | . | . | . |
| NFKB1 ---- NA |  | T/T | 0 | 0,00 | 0 | 0,00 | 1 | 0,29 | 3 | 0,98 | 2.18 (0.63-7.57) | 0.22 | 2.81 (0.75-10.47) | 0.12 | . | . | . |
| NFKB1 ---- NA | rs4648090 | G/G | 43 | 71,67 | 27 | 84,38 | 254 | 74,49 | 228 | 74,51 | 1.00 (.-.) | . | 1.21 (0.75-1.95) | 0.43 | 0.32 | 0.99 | 0.40 |
| NFKB1 ---- NA |  | G/A | 17 | 28,33 | 5 | 15,63 | 81 | 23,75 | 71 | 23,20 | 0.69 (0.24-2.04) | 0.51 | 1.46 (0.88-2.44) | 0.15 | . | . | . |
| NFKB1 ---- NA |  | A/A | 0 | 0,00 | 0 | 0,00 | 6 | 1,76 | 7 | 2,29 | 1.31 (0.52-3.30) | 0.57 | 1.58 (0.57-4.37) | 0.38 | . | . | . |
| NFKB1 ---- tag | rs4648110 | T/T | 40 | 66,67 | 22 | 68,75 | 219 | 64,22 | 199 | 65,03 | 1.00 (.-.) | . | 1.08 (0.65-1.80) | 0.75 | 0.10 | 0.99 | 0.28 |
| NFKB1 ---- tag |  | T/A | 19 | 31,67 | 10 | 31,25 | 110 | 32,26 | 95 | 31,05 | 0.50 (0.19-1.27) | 0.14 | 1.06 (0.63-1.79) | 0.83 | . | . | . |
| NFKB1 ---- tag |  | A/A | 1 | 1,67 | 0 | 0,00 | 12 | 3,52 | 12 | 3,92 | 0.00 (0.00-2E299) | 0.98 | 1.55 (0.70-3.46) | 0.28 | . | . | . |
| NFKB1 ---- tag | rs4648141 | G/G | 44 | 73,33 | 22 | 68,75 | 243 | 71,26 | 219 | 71,57 | 1.00 (.-.) | . | 1.06 (0.64-1.76) | 0.83 | 0.09 | 0.99 | 0.28 |
| NFKB1 ---- tag |  | G/A | 15 | 25,00 | 10 | 31,25 | 92 | 26,98 | 79 | 25,82 | 0.55 (0.22-1.34) | 0.19 | 1.13 (0.65-1.96) | 0.67 | . | . | . |
| NFKB1 ---- tag |  | A/A | 1 | 1,67 | 0 | 0,00 | 6 | 1,76 | 8 | 2,61 | 0.00 (0.00-1E299) | 0.98 | 2.06 (0.87-4.86) | 0.10 | . | . | . |
| NFKB1 ---- tag | rs4698863 | C/C | 17 | 28,33 | 16 | 50,00 | 158 | 46,33 | 140 | 45,75 | 1.00 (.-.) | . | 1.08 (0.61-1.92) | 0.79 | 0.08 | 0.99 | 0.28 |
| NFKB1 ---- tag |  | C/T | 30 | 50,00 | 14 | 43,75 | 153 | 44,87 | 135 | 44,12 | 0.87 (0.38-1.99) | 0.74 | 1.15 (0.65-2.05) | 0.63 | . | . | . |
| NFKB1 ---- tag |  | T/T | 13 | 21,67 | 2 | 6,25 | 30 | 8,80 | 31 | 10,13 | 0.20 (0.03-1.52) | 0.12 | 1.27 (0.64-2.48) | 0.49 | . | . | . |
| NFKB1 ---- NA | rs7674640 | C/C | 9 | 15,00 | 7 | 21,88 | 75 | 21,99 | 76 | 24,84 | 1.00 (.-.) | . | 0.76 (0.34-1.70) | 0.50 | 0.00 | 0.99 | 0.06 |
| NFKB1 ---- NA |  | C/T | 28 | 46,67 | 21 | 65,63 | 185 | 54,25 | 156 | 50,98 | 0.72 (0.29-1.75) | 0.46 | 0.78 (0.35-1.71) | 0.53 | . | . | . |
| NFKB1 ---- NA |  | T/T | 23 | 38,33 | 4 | 12,50 | 81 | 23,75 | 74 | 24,18 | 0.07 (0.01-0.62) | 0.02 | 0.83 (0.37-1.85) | 0.65 | . | . | . |
| NFKB1 ---- tag | rs909332 | A/A | 56 | 93,33 | 27 | 84,38 | 306 | 89,74 | 280 | 91,50 | 1.00 (.-.) | . | 1.54 (0.96-2.47) | 0.08 | 0.25 | 0.99 | 0.38 |
| NFKB1 ---- tag |  | A/T | 4 | 6,67 | 5 | 15,63 | 34 | 9,97 | 25 | 8,17 | 2.39 (0.88-6.47) | 0.09 | 1.76 (0.92-3.35) | 0.09 | . | . | . |
| NFKB1 ---- tag |  | T/T | 0 | 0,00 | 0 | 0,00 | 1 | 0,29 | 1 | 0,33 | 3.88 (0.50-29.96) | 0.19 | 5.97 (0.74-48.11) | 0.09 | . | . | . |
| NFKB1 ---- tag | rs997476 | C/C | 56 | 93,33 | 27 | 84,38 | 303 | 88,86 | 276 | 90,20 | 1.00 (.-.) | . | 1.24 (0.79-1.96) | 0.35 | 0.27 | 0.99 | 0.38 |
| NFKB1 ---- tag |  | C/A | 4 | 6,67 | 5 | 15,63 | 38 | 11,14 | 29 | 9,48 | 0.39 (0.09-1.69) | 0.21 | 0.98 (0.54-1.76) | 0.94 | . | . | . |
| NFKB1 ---- tag |  | A/A | 0 | 0,00 | 0 | 0,00 | 0 | 0,00 | 1 | 0,33 | 4.53 (0.59-35.03) | 0.15 | 5.63 (0.70-45.27) | 0.10 | . | . | . |
| NME1 ---- NA | rs10514981 | T/T | 32 | 53,33 | 21 | 65,63 | 214 | 62,76 | 191 | 62,42 | 1.00 (.-.) | . | 1.13 (0.68-1.88) | 0.64 | 0.34 | 0.99 | 0.91 |
| NME1 ---- NA |  | T/G | 26 | 43,33 | 9 | 28,13 | 113 | 33,14 | 105 | 34,31 | 0.51 (0.20-1.29) | 0.15 | 1.13 (0.66-1.93) | 0.66 | . | . | . |
| NME1 ---- NA |  | G/G | 2 | 3,33 | 2 | 6,25 | 14 | 4,11 | 10 | 3,27 | 1.75 (0.23-13.61) | 0.59 | 1.13 (0.46-2.76) | 0.80 | . | . | . |
| NME1 ---- NA | rs11651252 | T/T | 49 | 81,67 | 26 | 81,25 | 310 | 90,91 | 270 | 88,24 | 1.00 (.-.) | . | 1.42 (0.90-2.24) | 0.14 | 0.64 | 0.99 | 0.99 |
| NME1 ---- NA |  | T/C | 11 | 18,33 | 6 | 18,75 | 30 | 8,80 | 36 | 11,76 | 1.68 (0.50-5.66) | 0.41 | 1.75 (0.97-3.14) | 0.06 | . | . | . |
| NME1 ---- NA |  | C/C | 0 | 0,00 | 0 | 0,00 | 1 | 0,29 | 0 | 0,00 | 0.00 (0.00-I) | 0.98 | 0.00 (0.00-I) | 0.98 | . | . | . |
| NME1 ---- tag | rs11652793 | T/T | 36 | 60,00 | 22 | 68,75 | 220 | 64,52 | 209 | 68,30 | 1.00 (.-.) | . | 1.12 (0.69-1.84) | 0.64 | 0.17 | 0.99 | 0.82 |
| NME1 ---- tag |  | T/C | 24 | 40,00 | 9 | 28,13 | 113 | 33,14 | 88 | 28,76 | 0.51 (0.20-1.27) | 0.15 | 1.06 (0.63-1.81) | 0.82 | . | . | . |
| NME1 ---- tag |  | C/C | 0 | 0,00 | 1 | 3,13 | 8 | 2,35 | 9 | 2,94 | 1.06 (0.46-2.46) | 0.89 | 1.19 (0.46-3.08) | 0.72 | . | . | . |
| NME1 ---- NA | rs11868380 | C/C | 36 | 60,00 | 18 | 56,25 | 211 | 61,88 | 191 | 62,42 | 1.00 (.-.) | . | 1.46 (0.78-2.73) | 0.24 | 0.69 | 0.99 | 0.99 |
| NME1 ---- NA |  | C/G | 22 | 36,67 | 14 | 43,75 | 117 | 34,31 | 103 | 33,66 | 1.33 (0.60-2.99) | 0.48 | 1.52 (0.79-2.92) | 0.21 | . | . | . |
| NME1 ---- NA |  | G/G | 2 | 3,33 | 0 | 0,00 | 13 | 3,81 | 12 | 3,92 | 0.00 (0.00-1E305) | 0.97 | 1.48 (0.63-3.47) | 0.37 | . | . | . |
| NME1 ---- NA | rs1558252 | T/T | 34 | 56,67 | 17 | 53,13 | 175 | 51,32 | 136 | 44,44 | 1.00 (.-.) | . | 1.25 (0.70-2.23) | 0.45 | 0.94 | 1.00 | 1.00 |
| NME1 ---- NA |  | T/C | 24 | 40,00 | 10 | 31,25 | 131 | 38,42 | 141 | 46,08 | 0.92 (0.36-2.31) | 0.85 | 1.56 (0.88-2.78) | 0.13 | . | . | . |
| NME1 ---- NA |  | C/C | 2 | 3,33 | 5 | 15,63 | 35 | 10,26 | 29 | 9,48 | 1.29 (0.41-4.03) | 0.66 | 1.39 (0.70-2.75) | 0.35 | . | . | . |
| NME1 ---- NA | rs1558253 | T/T | 57 | 95,00 | 30 | 93,75 | 304 | 89,15 | 266 | 86,93 | 1.00 (.-.) | . | 1.32 (0.84-2.07) | 0.23 | 0.59 | 0.99 | 0.99 |
| NME1 ---- NA |  | T/G | 3 | 5,00 | 2 | 6,25 | 37 | 10,85 | 40 | 13,07 | 1.00 (.-.) | . | 1.32 (0.84-2.07) | 0.23 | . | . | . |
| NME1 ---- NA |  | G/G | 0 | 0,00 | 0 | 0,00 | 0 | 0,00 | 0 | 0,00 | 0.73 (0.17-3.20) | 0.68 | 1.44 (0.83-2.51) | 0.19 | . | . | . |
| NME1 ---- tag | rs16949683 | C/C | 54 | 90,00 | 28 | 87,50 | 323 | 94,72 | 282 | 92,16 | 1.00 (.-.) | . | 1.52 (0.96-2.42) | 0.08 | 0.13 | 0.99 | 0.82 |
| NME1 ---- tag |  | C/T | 6 | 10,00 | 4 | 12,50 | 17 | 4,99 | 23 | 7,52 | 3.79 (1.27-11.30) | 0.02 | 2.25 (1.18-4.29) | 0.01 | . | . | . |
| NME1 ---- tag |  | T/T | 0 | 0,00 | 0 | 0,00 | 1 | 0,29 | 1 | 0,33 | 1.58 (0.21-11.80) | 0.66 | 2.41 (0.31-18.86) | 0.40 | . | . | . |
| NME1 ---- tag | rs2318784 | C/C | 40 | 66,67 | 22 | 68,75 | 270 | 79,18 | 227 | 74,18 | 1.00 (.-.) | . | 1.39 (0.84-2.31) | 0.20 | 0.73 | 0.99 | 0.99 |
| NME1 ---- tag |  | C/T | 18 | 30,00 | 9 | 28,13 | 66 | 19,35 | 75 | 24,51 | 1.32 (0.52-3.39) | 0.56 | 1.87 (1.09-3.22) | 0.02 | . | . | . |
| NME1 ---- tag |  | T/T | 2 | 3,33 | 1 | 3,13 | 5 | 1,47 | 4 | 1,31 | 2.57 (0.33-20.05) | 0.37 | 1.35 (0.39-4.71) | 0.64 | . | . | . |
| NME1 ---- NA | rs2318785 | G/G | 24 | 40,00 | 8 | 25,00 | 102 | 29,91 | 88 | 28,76 | 1.00 (.-.) | . | 3.04 (1.21-7.62) | 0.02 | 0.06 | 0.99 | 0.82 |
| NME1 ---- NA |  | G/A | 28 | 46,67 | 20 | 62,50 | 171 | 50,15 | 170 | 55,56 | 2.85 (1.03-7.85) | 0.04 | 2.87 (1.16-7.10) | 0.02 | . | . | . |
| NME1 ---- NA |  | A/A | 8 | 13,33 | 4 | 12,50 | 68 | 19,94 | 48 | 15,69 | 2.17 (0.50-9.35) | 0.30 | 2.13 (0.83-5.48) | 0.12 | . | . | . |
| NME1 ---- tag | rs3760469 | G/G | 23 | 38,33 | 9 | 28,13 | 89 | 26,10 | 69 | 22,55 | 1.00 (.-.) | . | 2.58 (1.10-6.09) | 0.03 | 0.30 | 0.99 | 0.91 |
| NME1 ---- tag |  | G/T | 26 | 43,33 | 18 | 56,25 | 167 | 48,97 | 178 | 58,17 | 2.89 (1.09-7.67) | 0.03 | 2.46 (1.07-5.66) | 0.03 | . | . | . |
| NME1 ---- tag |  | T/T | 11 | 18,33 | 5 | 15,63 | 85 | 24,93 | 59 | 19,28 | 1.11 (0.27-4.48) | 0.89 | 1.98 (0.83-4.71) | 0.12 | . | . | . |
| NME1 ---- NA | rs4605213 | G/G | 23 | 38,33 | 15 | 46,88 | 136 | 39,88 | 134 | 43,79 | 1.00 (.-.) | . | 1.32 (0.73-2.38) | 0.36 | 1.00 | 1.00 | 1.00 |
| NME1 ---- NA |  | G/C | 29 | 48,33 | 12 | 37,50 | 167 | 48,97 | 137 | 44,77 | 0.80 (0.33-1.95) | 0.63 | 1.26 (0.70-2.28) | 0.45 | . | . | . |
| NME1 ---- NA |  | C/C | 8 | 13,33 | 5 | 15,63 | 38 | 11,14 | 35 | 11,44 | 1.21 (0.39-3.76) | 0.74 | 1.48 (0.74-2.94) | 0.26 | . | . | . |
| NME1 ---- NA | rs7207090 | A/A | 14 | 23,33 | 11 | 34,38 | 86 | 25,22 | 74 | 24,18 | 1.00 (.-.) | . | 1.33 (0.63-2.78) | 0.45 | 0.98 | 1.00 | 1.00 |
| NME1 ---- NA |  | A/T | 37 | 61,67 | 14 | 43,75 | 183 | 53,67 | 145 | 47,39 | 0.73 (0.29-1.84) | 0.51 | 1.13 (0.56-2.28) | 0.74 | . | . | . |
| NME1 ---- NA |  | T/T | 9 | 15,00 | 7 | 21,88 | 72 | 21,11 | 87 | 28,43 | 1.19 (0.41-3.42) | 0.75 | 1.44 (0.70-2.97) | 0.32 | . | . | . |
| NME1 ---- tag | rs7222463 | A/A | 9 | 15,00 | 9 | 28,13 | 88 | 25,81 | 87 | 28,43 | 1.00 (.-.) | . | 1.14 (0.48-2.71) | 0.76 | 0.36 | 0.99 | 0.91 |
| NME1 ---- tag |  | A/C | 39 | 65,00 | 16 | 50,00 | 179 | 52,49 | 151 | 49,35 | 0.78 (0.29-2.10) | 0.63 | 0.94 (0.40-2.19) | 0.88 | . | . | . |
| NME1 ---- tag |  | C/C | 12 | 20,00 | 7 | 21,88 | 74 | 21,70 | 68 | 22,22 | 0.61 (0.19-1.92) | 0.40 | 1.29 (0.54-3.08) | 0.57 | . | . | . |
| NME1 ---- tag | rs7226059 | C/C | 29 | 48,33 | 11 | 34,38 | 147 | 43,11 | 131 | 42,81 | 1.00 (.-.) | . | 2.09 (1.08-4.07) | 0.03 | 0.44 | 0.99 | 0.95 |
| NME1 ---- tag |  | C/T | 19 | 31,67 | 17 | 53,13 | 155 | 45,45 | 144 | 47,06 | 4.11 (1.74-9.71) | 0.00 | 2.13 (1.10-4.14) | 0.03 | . | . | . |
| NME1 ---- tag |  | T/T | 12 | 20,00 | 4 | 12,50 | 39 | 11,44 | 31 | 10,13 | 0.80 (0.22-2.93) | 0.73 | 1.67 (0.78-3.57) | 0.19 | . | . | . |
| NME1 ---- NA | rs880178 | G/G | 13 | 21,67 | 10 | 31,25 | 89 | 26,10 | 71 | 23,20 | 1.00 (.-.) | . | 1.33 (0.61-2.88) | 0.47 | 0.99 | 1.00 | 1.00 |
| NME1 ---- NA |  | G/T | 37 | 61,67 | 16 | 50,00 | 175 | 51,32 | 170 | 55,56 | 0.87 (0.35-2.16) | 0.76 | 1.26 (0.60-2.63) | 0.54 | . | . | . |
| NME1 ---- NA |  | T/T | 10 | 16,67 | 6 | 18,75 | 77 | 22,58 | 65 | 21,24 | 1.02 (0.33-3.15) | 0.98 | 1.30 (0.60-2.79) | 0.50 | . | . | . |
| NME2 ---- tag | rs7220360 | C/C | 9 | 15,00 | 9 | 28,13 | 88 | 25,81 | 87 | 28,43 | 1.00 (.-.) | . | 1.14 (0.48-2.71) | 0.76 | 0.36 | 0.99 | 0.36 |
| NME2 ---- tag |  | C/G | 39 | 65,00 | 16 | 50,00 | 179 | 52,49 | 151 | 49,35 | 0.78 (0.29-2.10) | 0.63 | 0.94 (0.40-2.19) | 0.88 | . | . | . |
| NME2 ---- tag |  | G/G | 12 | 20,00 | 7 | 21,88 | 74 | 21,70 | 68 | 22,22 | 0.61 (0.19-1.92) | 0.40 | 1.29 (0.54-3.08) | 0.57 | . | . | . |
| PON1 ---- tag | rs2269829 | A/A | 34 | 56,67 | 18 | 56,25 | 176 | 51,61 | 152 | 49,67 | 1.00 (.-.) | . | 1.47 (0.83-2.62) | 0.19 | 0.79 | 0.99 | 0.79 |
| PON1 ---- tag |  | A/G | 20 | 33,33 | 12 | 37,50 | 142 | 41,64 | 122 | 39,87 | 1.22 (0.53-2.82) | 0.64 | 1.29 (0.72-2.31) | 0.40 | . | . | . |
| PON1 ---- tag |  | G/G | 6 | 10,00 | 2 | 6,25 | 23 | 6,74 | 32 | 10,46 | 0.47 (0.06-3.65) | 0.47 | 1.73 (0.87-3.45) | 0.12 | . | . | . |
| PON1 ---- tag | rs3917527 | A/A | 54 | 90,00 | 30 | 93,75 | 303 | 88,86 | 284 | 92,81 | 1.00 (.-.) | . | 1.45 (0.93-2.26) | 0.10 | 0.52 | 0.99 | 0.79 |
| PON1 ---- tag |  | A/G | 6 | 10,00 | 2 | 6,25 | 36 | 10,56 | 21 | 6,86 | 1.10 (0.26-4.73) | 0.90 | 0.87 (0.46-1.63) | 0.66 | . | . | . |
| PON1 ---- tag |  | G/G | 0 | 0,00 | 0 | 0,00 | 2 | 0,59 | 1 | 0,33 | 0.96 (0.13-7.04) | 0.97 | 1.39 (0.18-10.64) | 0.75 | . | . | . |
| PON1 ---- tag | rs3917538 | C/C | 38 | 63,33 | 19 | 59,38 | 201 | 58,94 | 162 | 52,94 | 1.00 (.-.) | . | 1.39 (0.79-2.44) | 0.25 | 0.42 | 0.99 | 0.79 |
| PON1 ---- tag |  | C/T | 18 | 30,00 | 12 | 37,50 | 131 | 38,42 | 118 | 38,56 | 1.40 (0.61-3.19) | 0.43 | 1.42 (0.80-2.51) | 0.23 | . | . | . |
| PON1 ---- tag |  | T/T | 4 | 6,67 | 1 | 3,13 | 9 | 2,64 | 26 | 8,50 | 0.00 (0.00-I) | 0.98 | 2.60 (1.28-5.29) | 0.01 | . | . | . |
| PON1 ---- tag | rs757158 | C/C | 20 | 33,33 | 11 | 34,38 | 139 | 40,76 | 107 | 34,97 | 1.00 (.-.) | . | 1.30 (0.64-2.64) | 0.47 | 0.51 | 0.99 | 0.79 |
| PON1 ---- tag |  | C/T | 27 | 45,00 | 15 | 46,88 | 156 | 45,75 | 144 | 47,06 | 1.02 (0.42-2.45) | 0.97 | 1.29 (0.64-2.60) | 0.48 | . | . | . |
| PON1 ---- tag |  | T/T | 13 | 21,67 | 6 | 18,75 | 46 | 13,49 | 55 | 17,97 | 0.87 (0.26-2.92) | 0.82 | 1.99 (0.94-4.22) | 0.07 | . | . | . |
| PON1 ---- candidate | rs854560 | A/A | 19 | 31,67 | 14 | 43,75 | 119 | 34,90 | 131 | 42,81 | 1.00 (.-.) | . | 1.26 (0.65-2.47) | 0.49 | 0.65 | 0.99 | 0.79 |
| PON1 ---- candidate |  | A/T | 29 | 48,33 | 14 | 43,75 | 164 | 48,09 | 125 | 40,85 | 0.84 (0.35-2.01) | 0.69 | 1.11 (0.57-2.18) | 0.75 | . | . | . |
| PON1 ---- candidate |  | T/T | 12 | 20,00 | 4 | 12,50 | 58 | 17,01 | 50 | 16,34 | 0.72 (0.22-2.38) | 0.59 | 1.25 (0.60-2.62) | 0.55 | . | . | . |
| PRDM2 ---- tag | rs1015370 | C/C | 29 | 48,33 | 18 | 56,25 | 177 | 51,91 | 176 | 57,52 | 1.00 (.-.) | . | 1.57 (0.87-2.83) | 0.13 | 0.45 | 0.99 | 0.86 |
| PRDM2 ---- tag |  | C/T | 22 | 36,67 | 11 | 34,38 | 130 | 38,12 | 106 | 34,64 | 1.14 (0.48-2.71) | 0.77 | 1.37 (0.75-2.50) | 0.30 | . | . | . |
| PRDM2 ---- tag |  | T/T | 9 | 15,00 | 3 | 9,38 | 34 | 9,97 | 24 | 7,84 | 1.20 (0.33-4.31) | 0.78 | 1.18 (0.57-2.45) | 0.65 | . | . | . |
| PRDM2 ---- tag | rs1203634 | A/A | 38 | 63,33 | 23 | 71,88 | 213 | 62,46 | 189 | 61,76 | 1.00 (.-.) | . | 1.15 (0.70-1.88) | 0.59 | 0.29 | 0.99 | 0.86 |
| PRDM2 ---- tag |  | A/G | 18 | 30,00 | 6 | 18,75 | 111 | 32,55 | 93 | 30,39 | 0.48 (0.16-1.41) | 0.18 | 1.13 (0.67-1.92) | 0.64 | . | . | . |
| PRDM2 ---- tag |  | G/G | 4 | 6,67 | 3 | 9,38 | 17 | 4,99 | 24 | 7,84 | 0.72 (0.16-3.17) | 0.67 | 1.14 (0.60-2.18) | 0.69 | . | . | . |
| PRDM2 ---- tag | rs1203645 | A/A | 26 | 43,33 | 15 | 46,88 | 136 | 39,88 | 132 | 43,14 | 1.00 (.-.) | . | 1.29 (0.71-2.34) | 0.39 | 0.78 | 0.99 | 0.86 |
| PRDM2 ---- tag |  | A/C | 24 | 40,00 | 8 | 25,00 | 156 | 45,75 | 120 | 39,22 | 0.84 (0.31-2.25) | 0.73 | 1.22 (0.67-2.22) | 0.52 | . | . | . |
| PRDM2 ---- tag |  | C/C | 10 | 16,67 | 9 | 28,13 | 49 | 14,37 | 54 | 17,65 | 0.84 (0.31-2.27) | 0.73 | 1.26 (0.66-2.38) | 0.48 | . | . | . |
| PRDM2 ---- tag | rs1406416 | C/C | 34 | 56,67 | 14 | 43,75 | 183 | 53,67 | 151 | 49,35 | 1.00 (.-.) | . | 1.60 (0.79-3.23) | 0.19 | 0.79 | 0.99 | 0.86 |
| PRDM2 ---- tag |  | C/T | 24 | 40,00 | 12 | 37,50 | 130 | 38,12 | 122 | 39,87 | 1.41 (0.56-3.53) | 0.47 | 1.75 (0.86-3.58) | 0.12 | . | . | . |
| PRDM2 ---- tag |  | T/T | 2 | 3,33 | 6 | 18,75 | 28 | 8,21 | 33 | 10,78 | 1.55 (0.53-4.49) | 0.42 | 2.36 (1.09-5.13) | 0.03 | . | . | . |
| PRDM2 ---- candidate | rs17350795 | G/G | 59 | 98,33 | 31 | 96,88 | 324 | 95,01 | 291 | 95,10 | 1.00 (.-.) | . | 1.41 (0.91-2.19) | 0.12 | 0.76 | 0.99 | 0.86 |
| PRDM2 ---- candidate |  | G/A | 1 | 1,67 | 1 | 3,13 | 17 | 4,99 | 15 | 4,90 | 1.00 (.-.) | . | 1.41 (0.91-2.19) | 0.12 | . | . | . |
| PRDM2 ---- candidate |  | A/A | . | . | . | . | . | . | . | . | 0.83 (0.11-6.37) | 0.86 | 0.83 (0.39-1.77) | 0.63 | . | . | . |
| PRDM2 ---- tag | rs1980472 | C/C | 37 | 61,67 | 20 | 62,50 | 191 | 56,01 | 171 | 55,88 | 1.00 (.-.) | . | 1.24 (0.74-2.10) | 0.41 | 0.53 | 0.99 | 0.86 |
| PRDM2 ---- tag |  | C/G | 17 | 28,33 | 9 | 28,13 | 126 | 36,95 | 107 | 34,97 | 0.71 (0.28-1.84) | 0.48 | 1.15 (0.67-1.98) | 0.62 | . | . | . |
| PRDM2 ---- tag |  | G/G | 6 | 10,00 | 3 | 9,38 | 24 | 7,04 | 28 | 9,15 | 0.60 (0.14-2.66) | 0.50 | 1.07 (0.56-2.06) | 0.84 | . | . | . |
| PRDM2 ---- tag | rs2235515 | G/G | 34 | 56,67 | 18 | 56,25 | 207 | 60,70 | 181 | 59,15 | 1.00 (.-.) | . | 1.47 (0.82-2.65) | 0.20 | 0.81 | 0.99 | 0.86 |
| PRDM2 ---- tag |  | G/A | 24 | 40,00 | 14 | 43,75 | 118 | 34,60 | 108 | 35,29 | 1.15 (0.52-2.56) | 0.72 | 1.43 (0.78-2.61) | 0.25 | . | . | . |
| PRDM2 ---- tag |  | A/A | 2 | 3,33 | 0 | 0,00 | 16 | 4,69 | 17 | 5,56 | 0.00 (0.00-1E277) | 0.97 | 1.13 (0.51-2.51) | 0.77 | . | . | . |
| PRDM2 ---- tag | rs2244634 | A/A | 35 | 58,33 | 22 | 68,75 | 227 | 66,57 | 201 | 65,69 | 1.00 (.-.) | . | 1.40 (0.85-2.31) | 0.18 | 0.86 | 1.00 | 0.86 |
| PRDM2 ---- tag |  | A/C | 23 | 38,33 | 9 | 28,13 | 95 | 27,86 | 94 | 30,72 | 1.07 (0.43-2.66) | 0.88 | 1.46 (0.86-2.49) | 0.16 | . | . | . |
| PRDM2 ---- tag |  | C/C | 2 | 3,33 | 1 | 3,13 | 19 | 5,57 | 11 | 3,59 | 0.00 (0.00-3E305) | 0.98 | 0.82 (0.37-1.81) | 0.62 | . | . | . |
| PRDM2 ---- tag | rs2245213 | G/G | 41 | 68,33 | 23 | 71,88 | 254 | 74,49 | 222 | 72,55 | 1.00 (.-.) | . | 1.42 (0.86-2.35) | 0.17 | 0.60 | 0.99 | 0.86 |
| PRDM2 ---- tag |  | G/T | 19 | 31,67 | 9 | 28,13 | 77 | 22,58 | 79 | 25,82 | 1.13 (0.46-2.75) | 0.80 | 1.58 (0.92-2.72) | 0.10 | . | . | . |
| PRDM2 ---- tag |  | T/T | 0 | 0,00 | 0 | 0,00 | 10 | 2,93 | 5 | 1,63 | 0.32 (0.12-0.87) | 0.03 | 0.45 (0.15-1.36) | 0.16 | . | . | . |
| PRDM2 ---- tag | rs2294484 | C/C | 49 | 81,67 | 25 | 78,13 | 283 | 82,99 | 255 | 83,33 | 1.00 (.-.) | . | 1.31 (0.82-2.08) | 0.26 | 0.63 | 0.99 | 0.86 |
| PRDM2 ---- tag |  | C/G | 11 | 18,33 | 7 | 21,88 | 53 | 15,54 | 50 | 16,34 | 0.94 (0.32-2.77) | 0.90 | 1.78 (1.03-3.08) | 0.04 | . | . | . |
| PRDM2 ---- tag |  | G/G | 0 | 0,00 | 0 | 0,00 | 5 | 1,47 | 1 | 0,33 | 0.58 (0.08-4.18) | 0.59 | 0.75 (0.10-5.66) | 0.78 | . | . | . |
| PRDM2 ---- tag | rs2744689 | G/G | 42 | 70,00 | 24 | 75,00 | 258 | 75,66 | 220 | 71,90 | 1.00 (.-.) | . | 1.44 (0.88-2.33) | 0.14 | 0.54 | 0.99 | 0.86 |
| PRDM2 ---- tag |  | G/A | 18 | 30,00 | 8 | 25,00 | 72 | 21,11 | 80 | 26,14 | 1.22 (0.47-3.17) | 0.68 | 1.60 (0.95-2.72) | 0.08 | . | . | . |
| PRDM2 ---- tag |  | A/A | 0 | 0,00 | 0 | 0,00 | 11 | 3,23 | 6 | 1,96 | 0.37 (0.15-0.91) | 0.03 | 0.53 (0.19-1.44) | 0.21 | . | . | . |
| PRDM2 ---- tag | rs6690270 | A/A | 25 | 41,67 | 11 | 34,38 | 136 | 39,88 | 112 | 36,60 | 1.00 (.-.) | . | 1.53 (0.73-3.23) | 0.26 | 0.77 | 0.99 | 0.86 |
| PRDM2 ---- tag |  | A/G | 29 | 48,33 | 18 | 56,25 | 159 | 46,63 | 145 | 47,39 | 1.60 (0.66-3.85) | 0.30 | 1.78 (0.85-3.74) | 0.13 | . | . | . |
| PRDM2 ---- tag |  | G/G | 6 | 10,00 | 3 | 9,38 | 46 | 13,49 | 49 | 16,01 | 0.79 (0.20-3.07) | 0.73 | 1.83 (0.83-4.00) | 0.13 | . | . | . |
| RRM1 ---- tag | rs10835601 | G/G | 32 | 53,33 | 15 | 46,88 | 174 | 51,03 | 143 | 46,73 | 1.00 (.-.) | . | 1.56 (0.85-2.87) | 0.16 | 0.61 | 0.99 | 1.00 |
| RRM1 ---- tag |  | G/A | 22 | 36,67 | 14 | 43,75 | 137 | 40,18 | 133 | 43,46 | 1.68 (0.71-3.94) | 0.23 | 1.88 (1.02-3.47) | 0.04 | . | . | . |
| RRM1 ---- tag |  | A/A | 6 | 10,00 | 3 | 9,38 | 30 | 8,80 | 30 | 9,80 | 1.58 (0.44-5.71) | 0.49 | 2.01 (0.98-4.12) | 0.06 | . | . | . |
| RRM1 ---- tag | rs10835613 | C/C | 23 | 38,33 | 9 | 28,13 | 129 | 37,83 | 106 | 34,64 | 1.00 (.-.) | . | 1.66 (0.79-3.49) | 0.18 | 0.45 | 0.99 | 1.00 |
| RRM1 ---- tag |  | C/G | 28 | 46,67 | 16 | 50,00 | 156 | 45,75 | 149 | 48,69 | 1.54 (0.62-3.82) | 0.35 | 1.94 (0.94-4.01) | 0.07 | . | . | . |
| RRM1 ---- tag |  | G/G | 9 | 15,00 | 7 | 21,88 | 56 | 16,42 | 51 | 16,67 | 1.89 (0.61-5.87) | 0.27 | 2.04 (0.94-4.42) | 0.07 | . | . | . |
| RRM1 ---- NA | rs10835677 | G/G | 53 | 88,33 | 27 | 84,38 | 277 | 81,23 | 249 | 81,37 | 1.00 (.-.) | . | 1.37 (0.86-2.16) | 0.18 | 1.00 | 1.00 | 1.00 |
| RRM1 ---- NA |  | G/A | 7 | 11,67 | 5 | 15,63 | 60 | 17,60 | 53 | 17,32 | 0.99 (0.29-3.38) | 0.98 | 1.41 (0.83-2.40) | 0.20 | . | . | . |
| RRM1 ---- NA |  | A/A | 0 | 0,00 | 0 | 0,00 | 4 | 1,17 | 4 | 1,31 | 0.77 (0.24-2.45) | 0.66 | 1.05 (0.31-3.61) | 0.94 | . | . | . |
| RRM1 ---- tag | rs10835678 | A/A | 54 | 90,00 | 29 | 90,63 | 302 | 88,56 | 274 | 89,54 | 1.00 (.-.) | . | 1.41 (0.91-2.18) | 0.13 | 0.79 | 0.99 | 1.00 |
| RRM1 ---- tag |  | A/G | 6 | 10,00 | 3 | 9,38 | 37 | 10,85 | 32 | 10,46 | 1.08 (0.14-8.29) | 0.94 | 1.22 (0.68-2.20) | 0.50 | . | . | . |
| RRM1 ---- tag |  | G/G | 0 | 0,00 | 0 | 0,00 | 2 | 0,59 | 0 | 0,00 | 0.00 (0.00-5E235) | 0.97 | 0.00 (0.00-6E235) | 0.97 | . | . | . |
| RRM1 ---- tag | rs12288551 | C/C | 56 | 93,33 | 30 | 93,75 | 316 | 92,67 | 269 | 87,91 | 1.00 (.-.) | . | 1.40 (0.89-2.18) | 0.14 | 0.58 | 0.99 | 1.00 |
| RRM1 ---- tag |  | C/G | 4 | 6,67 | 2 | 6,25 | 25 | 7,33 | 36 | 11,76 | 1.00 (.-.) | . | 1.40 (0.89-2.18) | 0.14 | . | . | . |
| RRM1 ---- tag |  | G/G | 0 | 0,00 | 0 | 0,00 | 0 | 0,00 | 1 | 0,33 | 2.03 (0.47-8.79) | 0.34 | 1.80 (1.01-3.23) | 0.05 | . | . | . |
| RRM1 ---- NA | rs12806698 | C/C | 30 | 50,00 | 18 | 56,25 | 188 | 55,13 | 154 | 50,33 | 1.00 (.-.) | . | 1.08 (0.63-1.85) | 0.78 | 0.33 | 0.99 | 1.00 |
| RRM1 ---- NA |  | C/A | 23 | 38,33 | 11 | 34,38 | 126 | 36,95 | 128 | 41,83 | 0.59 (0.23-1.54) | 0.28 | 1.29 (0.75-2.22) | 0.36 | . | . | . |
| RRM1 ---- NA |  | A/A | 7 | 11,67 | 3 | 9,38 | 27 | 7,92 | 24 | 7,84 | 1.04 (0.30-3.65) | 0.95 | 1.48 (0.75-2.91) | 0.26 | . | . | . |
| RRM1 ---- NA | rs1465952 | T/T | 47 | 78,33 | 25 | 78,13 | 280 | 82,11 | 255 | 83,33 | 1.00 (.-.) | . | 1.41 (0.88-2.24) | 0.15 | 0.83 | 1.00 | 1.00 |
| RRM1 ---- NA |  | T/C | 13 | 21,67 | 7 | 21,88 | 58 | 17,01 | 50 | 16,34 | 0.92 (0.31-2.75) | 0.88 | 1.21 (0.70-2.09) | 0.50 | . | . | . |
| RRM1 ---- NA |  | C/C | 0 | 0,00 | 0 | 0,00 | 3 | 0,88 | 1 | 0,33 | 0.00 (0.00-I) | 0.97 | 0.00 (0.00-I) | 0.98 | . | . | . |
| RRM1 ---- tag | rs4910904 | A/A | 29 | 48,33 | 10 | 31,25 | 166 | 48,68 | 126 | 41,18 | 1.00 (.-.) | . | 1.62 (0.78-3.37) | 0.20 | 0.64 | 0.99 | 1.00 |
| RRM1 ---- tag |  | A/G | 22 | 36,67 | 17 | 53,13 | 139 | 40,76 | 144 | 47,06 | 1.69 (0.69-4.14) | 0.25 | 2.14 (1.03-4.43) | 0.04 | . | . | . |
| RRM1 ---- tag |  | G/G | 9 | 15,00 | 5 | 15,63 | 36 | 10,56 | 36 | 11,76 | 1.93 (0.57-6.54) | 0.29 | 2.40 (1.07-5.37) | 0.03 | . | . | . |
| RRM1 ---- tag | rs7103860 | T/T | 44 | 73,33 | 22 | 68,75 | 264 | 77,42 | 245 | 80,07 | 1.00 (.-.) | . | 1.35 (0.83-2.21) | 0.23 | 0.98 | 1.00 | 1.00 |
| RRM1 ---- tag |  | T/C | 16 | 26,67 | 10 | 31,25 | 71 | 20,82 | 60 | 19,61 | 0.88 (0.34-2.25) | 0.79 | 1.34 (0.77-2.33) | 0.29 | . | . | . |
| RRM1 ---- tag |  | C/C | 0 | 0,00 | 0 | 0,00 | 6 | 1,76 | 1 | 0,33 | 0.00 (0.00-I) | 0.97 | 0.00 (0.00-I) | 0.97 | . | . | . |
| RRM1 ---- tag | rs7115496 | C/C | 54 | 90,00 | 29 | 90,63 | 290 | 85,04 | 271 | 88,56 | 1.00 (.-.) | . | 1.41 (0.91-2.20) | 0.12 | 0.81 | 0.99 | 1.00 |
| RRM1 ---- tag |  | C/T | 6 | 10,00 | 3 | 9,38 | 47 | 13,78 | 35 | 11,44 | 0.97 (0.13-7.35) | 0.97 | 1.22 (0.69-2.16) | 0.50 | . | . | . |
| RRM1 ---- tag |  | T/T | 0 | 0,00 | 0 | 0,00 | 4 | 1,17 | 0 | 0,00 | 0.00 (0.00-2E252) | 0.97 | 0.00 (0.00-2E252) | 0.97 | . | . | . |
| RRM2 ---- NA | rs1138729 | A/A | 43 | 71,67 | 20 | 62,50 | 247 | 72,43 | 225 | 73,53 | 1.00 (.-.) | . | 1.60 (0.91-2.82) | 0.11 | 0.38 | 0.99 | 0.75 |
| RRM2 ---- NA |  | A/G | 16 | 26,67 | 11 | 34,38 | 87 | 25,51 | 71 | 23,20 | 1.46 (0.63-3.38) | 0.37 | 1.57 (0.86-2.89) | 0.14 | . | . | . |
| RRM2 ---- NA |  | G/G | 1 | 1,67 | 1 | 3,13 | 7 | 2,05 | 10 | 3,27 | 1.93 (0.25-15.22) | 0.53 | 1.94 (0.79-4.77) | 0.15 | . | . | . |
| RRM2 ---- tag | rs4668664 | G/G | 26 | 43,33 | 15 | 46,88 | 167 | 48,97 | 156 | 50,98 | 1.00 (.-.) | . | 1.48 (0.79-2.77) | 0.23 | 0.80 | 0.99 | 0.80 |
| RRM2 ---- tag |  | G/A | 29 | 48,33 | 13 | 40,63 | 135 | 39,59 | 124 | 40,52 | 1.13 (0.50-2.58) | 0.77 | 1.45 (0.76-2.74) | 0.26 | . | . | . |
| RRM2 ---- tag |  | A/A | 5 | 8,33 | 4 | 12,50 | 39 | 11,44 | 26 | 8,50 | 0.73 (0.09-5.75) | 0.76 | 1.15 (0.54-2.48) | 0.71 | . | . | . |
| RRM2 ---- NA | rs6741290 | C/C | 17 | 28,33 | 8 | 25,00 | 102 | 29,91 | 105 | 34,31 | 1.00 (.-.) | . | 1.84 (0.74-4.59) | 0.19 | 0.37 | 0.99 | 0.75 |
| RRM2 ---- NA |  | C/T | 28 | 46,67 | 14 | 43,75 | 165 | 48,39 | 135 | 44,12 | 1.22 (0.43-3.47) | 0.71 | 1.61 (0.65-3.99) | 0.31 | . | . | . |
| RRM2 ---- NA |  | T/T | 15 | 25,00 | 10 | 31,25 | 74 | 21,70 | 66 | 21,57 | 1.60 (0.50-5.13) | 0.43 | 1.73 (0.68-4.41) | 0.25 | . | . | . |
| RRM2 ---- tag | rs7574663 | C/C | 40 | 66,67 | 17 | 53,13 | 211 | 61,88 | 199 | 65,03 | 1.00 (.-.) | . | 1.55 (0.86-2.79) | 0.15 | 0.77 | 0.99 | 0.80 |
| RRM2 ---- tag |  | C/G | 14 | 23,33 | 14 | 43,75 | 120 | 35,19 | 90 | 29,41 | 1.28 (0.56-2.91) | 0.55 | 1.31 (0.71-2.43) | 0.39 | . | . | . |
| RRM2 ---- tag |  | G/G | 6 | 10,00 | 1 | 3,13 | 10 | 2,93 | 17 | 5,56 | 0.75 (0.10-5.83) | 0.79 | 2.13 (0.98-4.63) | 0.06 | . | . | . |
| SHMT1 ---- candidate | rs1979277 | G/G | 30 | 50,00 | 20 | 62,50 | 151 | 44,28 | 150 | 49,02 | 1.00 (.-.) | . | 1.11 (0.65-1.88) | 0.70 | 0.32 | 0.99 | 0.32 |
| SHMT1 ---- candidate |  | G/A | 23 | 38,33 | 9 | 28,13 | 157 | 46,04 | 130 | 42,48 | 0.42 (0.16-1.08) | 0.07 | 0.87 (0.51-1.50) | 0.63 | . | . | . |
| SHMT1 ---- candidate |  | A/A | 7 | 11,67 | 3 | 9,38 | 33 | 9,68 | 26 | 8,50 | 0.74 (0.17-3.24) | 0.69 | 1.06 (0.53-2.13) | 0.87 | . | . | . |
| SHMT1 ---- tag | rs2168781 | G/G | 21 | 35,00 | 18 | 56,25 | 102 | 29,91 | 113 | 36,93 | 1.00 (.-.) | . | 0.93 (0.53-1.63) | 0.80 | 0.17 | 0.99 | 0.32 |
| SHMT1 ---- tag |  | G/C | 29 | 48,33 | 9 | 28,13 | 184 | 53,96 | 141 | 46,08 | 0.29 (0.10-0.81) | 0.02 | 0.79 (0.45-1.38) | 0.40 | . | . | . |
| SHMT1 ---- tag |  | C/C | 10 | 16,67 | 5 | 15,63 | 55 | 16,13 | 52 | 16,99 | 0.60 (0.20-1.82) | 0.36 | 0.85 (0.45-1.58) | 0.60 | . | . | . |
| SHMT1 ---- tag | rs4924849 | C/C | 30 | 50,00 | 20 | 62,50 | 160 | 46,92 | 158 | 51,63 | 1.00 (.-.) | . | 1.10 (0.65-1.86) | 0.72 | 0.31 | 0.99 | 0.32 |
| SHMT1 ---- tag |  | C/T | 23 | 38,33 | 9 | 28,13 | 150 | 43,99 | 123 | 40,20 | 0.42 (0.16-1.08) | 0.07 | 0.87 (0.50-1.50) | 0.61 | . | . | . |
| SHMT1 ---- tag |  | T/T | 7 | 11,67 | 3 | 9,38 | 31 | 9,09 | 25 | 8,17 | 0.74 (0.17-3.25) | 0.69 | 1.11 (0.55-2.23) | 0.77 | . | . | . |
| SHMT1 ---- candidate literature | rs9909104 | T/T | 24 | 40,00 | 18 | 56,25 | 184 | 53,96 | 165 | 53,92 | 1.00 (.-.) | . | 0.84 (0.47-1.51) | 0.56 | 0.19 | 0.99 | 0.32 |
| SHMT1 ---- candidate literature |  | T/C | 31 | 51,67 | 10 | 31,25 | 136 | 39,88 | 120 | 39,22 | 0.38 (0.15-0.92) | 0.03 | 0.94 (0.52-1.71) | 0.84 | . | . | . |
| SHMT1 ---- candidate literature |  | C/C | 5 | 8,33 | 4 | 12,50 | 21 | 6,16 | 21 | 6,86 | 0.81 (0.26-2.55) | 0.72 | 0.84 (0.39-1.81) | 0.65 | . | . | . |
| SHMT2 ---- tag | rs10876968 | G/G | 27 | 45,00 | 19 | 59,38 | 179 | 52,49 | 168 | 54,90 | 1.00 (.-.) | . | 1.20 (0.69-2.10) | 0.51 | 0.48 | 0.99 | 0.92 |
| SHMT2 ---- tag |  | G/T | 27 | 45,00 | 12 | 37,50 | 130 | 38,12 | 115 | 37,58 | 0.73 (0.31-1.72) | 0.47 | 1.16 (0.65-2.06) | 0.62 | . | . | . |
| SHMT2 ---- tag |  | T/T | 6 | 10,00 | 1 | 3,13 | 32 | 9,38 | 23 | 7,52 | 0.45 (0.06-3.44) | 0.44 | 0.92 (0.45-1.89) | 0.83 | . | . | . |
| SHMT2 ---- tag | rs1800165 | T/T | 27 | 45,00 | 17 | 53,13 | 161 | 47,21 | 152 | 49,67 | 1.00 (.-.) | . | 1.20 (0.68-2.09) | 0.53 | 0.86 | 1.00 | 0.92 |
| SHMT2 ---- tag |  | T/C | 28 | 46,67 | 12 | 37,50 | 145 | 42,52 | 124 | 40,52 | 0.67 (0.28-1.60) | 0.36 | 1.25 (0.71-2.19) | 0.44 | . | . | . |
| SHMT2 ---- tag |  | C/C | 5 | 8,33 | 3 | 9,38 | 35 | 10,26 | 30 | 9,80 | 2.88 (0.64-12.88) | 0.17 | 1.41 (0.74-2.71) | 0.30 | . | . | . |
| SHMT2 ---- tag | rs7133939 | T/T | 11 | 18,33 | 12 | 37,50 | 99 | 29,03 | 101 | 33,01 | 1.00 (.-.) | . | 1.22 (0.63-2.36) | 0.55 | 0.30 | 0.99 | 0.92 |
| SHMT2 ---- tag |  | T/A | 33 | 55,00 | 17 | 53,13 | 162 | 47,51 | 145 | 47,39 | 1.00 (0.43-2.33) | 1.00 | 1.17 (0.62-2.24) | 0.62 | . | . | . |
| SHMT2 ---- tag |  | A/A | 16 | 26,67 | 3 | 9,38 | 80 | 23,46 | 60 | 19,61 | 0.29 (0.06-1.31) | 0.11 | 0.97 (0.49-1.91) | 0.93 | . | . | . |
| SHMT2 ---- tag | rs7485577 | G/G | 30 | 50,00 | 20 | 62,50 | 181 | 53,08 | 166 | 54,25 | 1.00 (.-.) | . | 1.24 (0.72-2.12) | 0.44 | 0.92 | 1.00 | 0.92 |
| SHMT2 ---- tag |  | G/A | 26 | 43,33 | 10 | 31,25 | 134 | 39,30 | 114 | 37,25 | 0.72 (0.29-1.76) | 0.47 | 1.31 (0.76-2.27) | 0.33 | . | . | . |
| SHMT2 ---- tag |  | A/A | 4 | 6,67 | 2 | 6,25 | 26 | 7,62 | 26 | 8,50 | 2.55 (0.57-11.35) | 0.22 | 1.52 (0.79-2.93) | 0.21 | . | . | . |
| SHMT2 ---- tag | rs7489231 | T/T | 23 | 38,33 | 16 | 50,00 | 149 | 43,70 | 139 | 45,42 | 1.00 (.-.) | . | 1.15 (0.65-2.01) | 0.63 | 0.75 | 0.99 | 0.92 |
| SHMT2 ---- tag |  | T/C | 32 | 53,33 | 13 | 40,63 | 152 | 44,57 | 129 | 42,16 | 0.71 (0.30-1.70) | 0.44 | 1.34 (0.76-2.34) | 0.31 | . | . | . |
| SHMT2 ---- tag |  | C/C | 5 | 8,33 | 3 | 9,38 | 40 | 11,73 | 38 | 12,42 | 2.50 (0.56-11.23) | 0.23 | 1.49 (0.79-2.80) | 0.22 | . | . | . |
| SLC19A1 ---- candidate | rs1051266 | G/G | 19 | 31,67 | 8 | 25,00 | 110 | 32,26 | 86 | 28,10 | 1.00 (.-.) | . | 1.47 (0.65-3.29) | 0.35 | 0.95 | 1.00 | 0.98 |
| SLC19A1 ---- candidate |  | G/A | 30 | 50,00 | 16 | 50,00 | 158 | 46,33 | 168 | 54,90 | 1.33 (0.52-3.43) | 0.55 | 1.64 (0.74-3.65) | 0.22 | . | . | . |
| SLC19A1 ---- candidate |  | A/A | 11 | 18,33 | 8 | 25,00 | 73 | 21,41 | 52 | 16,99 | 0.98 (0.32-3.02) | 0.98 | 1.49 (0.65-3.41) | 0.34 | . | . | . |
| SLC19A1 ---- candidate literature | rs1131596 | T/T | 20 | 33,33 | 8 | 25,00 | 110 | 32,26 | 86 | 28,10 | 1.00 (.-.) | . | 1.54 (0.69-3.43) | 0.30 | 0.98 | 1.00 | 0.98 |
| SLC19A1 ---- candidate literature |  | T/C | 29 | 48,33 | 16 | 50,00 | 158 | 46,33 | 168 | 54,90 | 1.45 (0.56-3.72) | 0.44 | 1.72 (0.78-3.80) | 0.18 | . | . | . |
| SLC19A1 ---- candidate literature |  | C/C | 11 | 18,33 | 8 | 25,00 | 73 | 21,41 | 52 | 16,99 | 1.03 (0.34-3.15) | 0.96 | 1.56 (0.68-3.55) | 0.29 | . | . | . |
| SLC19A1 ---- tag | rs12483553 | G/G | 45 | 75,00 | 26 | 81,25 | 276 | 80,94 | 245 | 80,07 | 1.00 (.-.) | . | 1.32 (0.81-2.14) | 0.26 | 0.78 | 0.99 | 0.98 |
| SLC19A1 ---- tag |  | G/A | 15 | 25,00 | 6 | 18,75 | 60 | 17,60 | 60 | 19,61 | 0.97 (0.38-2.46) | 0.94 | 1.58 (0.92-2.71) | 0.10 | . | . | . |
| SLC19A1 ---- tag |  | A/A | 0 | 0,00 | 0 | 0,00 | 5 | 1,47 | 1 | 0,33 | 0.45 (0.06-3.25) | 0.43 | 0.59 (0.08-4.53) | 0.61 | . | . | . |
| SLC19A1 ---- candidate literature | rs12659 | C/C | 19 | 31,67 | 8 | 25,00 | 115 | 33,72 | 91 | 29,74 | 1.00 (.-.) | . | 1.47 (0.66-3.30) | 0.35 | 0.94 | 1.00 | 0.98 |
| SLC19A1 ---- candidate literature |  | C/T | 32 | 53,33 | 16 | 50,00 | 151 | 44,28 | 165 | 53,92 | 1.28 (0.50-3.30) | 0.61 | 1.68 (0.76-3.74) | 0.20 | . | . | . |
| SLC19A1 ---- candidate literature |  | T/T | 9 | 15,00 | 8 | 25,00 | 75 | 21,99 | 50 | 16,34 | 1.06 (0.34-3.25) | 0.92 | 1.45 (0.63-3.31) | 0.38 | . | . | . |
| SLC19A1 ---- tag | rs3788190 | G/G | 18 | 30,00 | 8 | 25,00 | 109 | 31,96 | 88 | 28,76 | 1.00 (.-.) | . | 1.48 (0.67-3.31) | 0.34 | 0.81 | 0.99 | 0.98 |
| SLC19A1 ---- tag |  | G/A | 32 | 53,33 | 17 | 53,13 | 151 | 44,28 | 161 | 52,61 | 1.15 (0.45-2.92) | 0.77 | 1.58 (0.71-3.49) | 0.26 | . | . | . |
| SLC19A1 ---- tag |  | A/A | 10 | 16,67 | 7 | 21,88 | 81 | 23,75 | 57 | 18,63 | 1.12 (0.35-3.62) | 0.85 | 1.43 (0.63-3.23) | 0.40 | . | . | . |
| SLC19A1 ---- tag | rs3788205 | C/C | 23 | 38,33 | 20 | 62,50 | 173 | 50,73 | 165 | 53,92 | 1.00 (.-.) | . | 1.14 (0.66-1.96) | 0.65 | 0.51 | 0.99 | 0.98 |
| SLC19A1 ---- tag |  | C/T | 32 | 53,33 | 9 | 28,13 | 137 | 40,18 | 117 | 38,24 | 0.59 (0.24-1.46) | 0.25 | 1.14 (0.65-2.01) | 0.64 | . | . | . |
| SLC19A1 ---- tag |  | T/T | 5 | 8,33 | 3 | 9,38 | 31 | 9,09 | 24 | 7,84 | 0.88 (0.24-3.15) | 0.84 | 1.08 (0.55-2.15) | 0.82 | . | . | . |
| SLC19A1 ---- tag | rs7279664 | G/G | 21 | 35,00 | 14 | 43,75 | 135 | 39,59 | 127 | 41,50 | 1.00 (.-.) | . | 1.06 (0.55-2.02) | 0.87 | 0.18 | 0.99 | 0.98 |
| SLC19A1 ---- tag |  | G/T | 29 | 48,33 | 16 | 50,00 | 157 | 46,04 | 127 | 41,50 | 0.72 (0.31-1.68) | 0.45 | 0.98 (0.51-1.88) | 0.96 | . | . | . |
| SLC19A1 ---- tag |  | T/T | 10 | 16,67 | 2 | 6,25 | 49 | 14,37 | 52 | 16,99 | 0.35 (0.07-1.62) | 0.18 | 1.08 (0.54-2.16) | 0.83 | . | . | . |
| SLC29A1 ---- NA | rs1057985 | C/C | 19 | 31,67 | 12 | 37,50 | 149 | 43,70 | 144 | 47,06 | 1.00 (.-.) | . | 1.48 (0.71-3.06) | 0.29 | 0.74 | 0.99 | 0.85 |
| SLC29A1 ---- NA |  | C/T | 33 | 55,00 | 18 | 56,25 | 143 | 41,94 | 129 | 42,16 | 1.06 (0.45-2.53) | 0.89 | 1.45 (0.70-3.02) | 0.32 | . | . | . |
| SLC29A1 ---- NA |  | T/T | 8 | 13,33 | 2 | 6,25 | 49 | 14,37 | 33 | 10,78 | 1.14 (0.23-5.61) | 0.87 | 1.28 (0.57-2.88) | 0.55 | . | . | . |
| SLC29A1 ---- NA | rs6458375 | C/C | 37 | 61,67 | 16 | 50,00 | 179 | 52,49 | 173 | 56,54 | 1.00 (.-.) | . | 1.80 (0.97-3.34) | 0.06 | 0.16 | 0.99 | 0.39 |
| SLC29A1 ---- NA |  | C/T | 19 | 31,67 | 14 | 43,75 | 141 | 41,35 | 118 | 38,56 | 1.39 (0.60-3.23) | 0.44 | 1.50 (0.80-2.81) | 0.20 | . | . | . |
| SLC29A1 ---- NA |  | T/T | 4 | 6,67 | 2 | 6,25 | 21 | 6,16 | 15 | 4,90 | 1.95 (0.42-9.04) | 0.39 | 1.33 (0.56-3.13) | 0.52 | . | . | . |
| SLC29A1 ---- NA | rs666462 | C/C | 18 | 30,00 | 7 | 21,88 | 83 | 24,34 | 92 | 30,07 | 1.00 (.-.) | . | 1.95 (0.83-4.57) | 0.12 | 0.85 | 1.00 | 0.85 |
| SLC29A1 ---- NA |  | C/T | 26 | 43,33 | 16 | 50,00 | 178 | 52,20 | 150 | 49,02 | 1.79 (0.67-4.79) | 0.24 | 1.73 (0.75-4.00) | 0.20 | . | . | . |
| SLC29A1 ---- NA |  | T/T | 16 | 26,67 | 9 | 28,13 | 80 | 23,46 | 64 | 20,92 | 1.01 (0.30-3.42) | 0.99 | 1.86 (0.79-4.40) | 0.16 | . | . | . |
| SLC29A1 ---- NA | rs6905285 | A/A | 16 | 26,67 | 13 | 40,63 | 149 | 43,70 | 100 | 32,68 | 1.00 (.-.) | . | 0.65 (0.33-1.29) | 0.22 | 0.04 | 0.99 | 0.29 |
| SLC29A1 ---- NA |  | A/T | 35 | 58,33 | 16 | 50,00 | 149 | 43,70 | 149 | 48,69 | 0.37 (0.16-0.87) | 0.02 | 0.69 (0.35-1.36) | 0.29 | . | . | . |
| SLC29A1 ---- NA |  | T/T | 9 | 15,00 | 3 | 9,38 | 43 | 12,61 | 57 | 18,63 | 0.63 (0.14-2.90) | 0.55 | 0.92 (0.46-1.85) | 0.81 | . | . | . |
| SLC29A1 ---- NA | rs693955 | G/G | 38 | 63,33 | 17 | 53,13 | 219 | 64,22 | 212 | 69,28 | 1.00 (.-.) | . | 1.70 (0.95-3.05) | 0.07 | 0.28 | 0.99 | 0.39 |
| SLC29A1 ---- NA |  | G/T | 21 | 35,00 | 14 | 43,75 | 106 | 31,09 | 84 | 27,45 | 1.55 (0.69-3.45) | 0.29 | 1.50 (0.81-2.77) | 0.20 | . | . | . |
| SLC29A1 ---- NA |  | T/T | 1 | 1,67 | 1 | 3,13 | 16 | 4,69 | 10 | 3,27 | 0.00 (0.00-1E244) | 0.97 | 1.67 (0.62-4.50) | 0.31 | . | . | . |
| SLC29A1 ---- NA | rs747199 | C/C | 35 | 58,33 | 25 | 78,13 | 221 | 64,81 | 203 | 66,34 | 1.00 (.-.) | . | 1.14 (0.69-1.88) | 0.60 | 0.26 | 0.99 | 0.39 |
| SLC29A1 ---- NA |  | C/G | 23 | 38,33 | 6 | 18,75 | 107 | 31,38 | 90 | 29,41 | 0.53 (0.20-1.44) | 0.22 | 1.18 (0.71-1.98) | 0.53 | . | . | . |
| SLC29A1 ---- NA |  | G/G | 2 | 3,33 | 1 | 3,13 | 13 | 3,81 | 13 | 4,25 | 0.79 (0.10-6.21) | 0.82 | 1.19 (0.55-2.59) | 0.66 | . | . | . |
| SLC29A1 ---- NA | rs9357436 | G/G | 37 | 61,67 | 26 | 81,25 | 248 | 72,73 | 221 | 72,22 | 1.00 (.-.) | . | 1.12 (0.69-1.84) | 0.64 | 0.22 | 0.99 | 0.39 |
| SLC29A1 ---- NA |  | G/A | 21 | 35,00 | 5 | 15,63 | 84 | 24,63 | 74 | 24,18 | 0.53 (0.20-1.44) | 0.21 | 1.25 (0.74-2.13) | 0.40 | . | . | . |
| SLC29A1 ---- NA |  | A/A | 2 | 3,33 | 1 | 3,13 | 9 | 2,64 | 11 | 3,59 | 0.79 (0.10-6.20) | 0.82 | 1.14 (0.49-2.65) | 0.77 | . | . | . |
| TCN2 ---- tag | rs10418 | C/C | 32 | 53,33 | 17 | 53,13 | 208 | 61,00 | 166 | 54,25 | 1.00 (.-.) | . | 1.28 (0.71-2.32) | 0.41 | 0.91 | 1.00 | 0.98 |
| TCN2 ---- tag |  | C/T | 26 | 43,33 | 14 | 43,75 | 119 | 34,90 | 121 | 39,54 | 0.93 (0.41-2.13) | 0.87 | 1.44 (0.79-2.62) | 0.24 | . | . | . |
| TCN2 ---- tag |  | T/T | 2 | 3,33 | 1 | 3,13 | 14 | 4,11 | 19 | 6,21 | 1.81 (0.23-14.31) | 0.58 | 1.43 (0.66-3.11) | 0.36 | . | . | . |
| TCN2 ---- candidate/singleton | rs1131603 | T/T | 57 | 95,00 | 27 | 84,38 | 300 | 87,98 | 269 | 87,91 | 1.00 (.-.) | . | 1.58 (0.99-2.52) | 0.06 | 0.09 | 0.99 | 0.20 |
| TCN2 ---- candidate/singleton |  | T/C | 3 | 5,00 | 5 | 15,63 | 39 | 11,44 | 35 | 11,44 | 2.42 (0.82-7.17) | 0.11 | 1.30 (0.73-2.31) | 0.38 | . | . | . |
| TCN2 ---- candidate/singleton |  | C/C | 0 | 0,00 | 0 | 0,00 | 2 | 0,59 | 2 | 0,65 | 0.43 (0.06-3.17) | 0.41 | 0.69 (0.09-5.22) | 0.72 | . | . | . |
| TCN2 ---- tag | rs1544468 | A/A | 21 | 35,00 | 9 | 28,13 | 96 | 28,15 | 71 | 23,20 | 1.00 (.-.) | . | 1.62 (0.72-3.61) | 0.24 | 0.11 | 0.99 | 0.20 |
| TCN2 ---- tag |  | A/G | 28 | 46,67 | 11 | 34,38 | 156 | 45,75 | 158 | 51,63 | 0.81 (0.29-2.30) | 0.70 | 1.62 (0.73-3.57) | 0.23 | . | . | . |
| TCN2 ---- tag |  | G/G | 11 | 18,33 | 12 | 37,50 | 89 | 26,10 | 77 | 25,16 | 2.29 (0.84-6.24) | 0.11 | 1.58 (0.70-3.56) | 0.27 | . | . | . |
| TCN2 ---- candidate/tag | rs1801198 | C/C | 12 | 20,00 | 13 | 40,63 | 105 | 30,79 | 97 | 31,70 | 1.00 (.-.) | . | 0.68 (0.35-1.32) | 0.25 | 0.10 | 0.99 | 0.20 |
| TCN2 ---- candidate/tag |  | C/G | 35 | 58,33 | 11 | 34,38 | 157 | 46,04 | 148 | 48,37 | 0.29 (0.12-0.75) | 0.01 | 0.64 (0.33-1.24) | 0.19 | . | . | . |
| TCN2 ---- candidate/tag |  | G/G | 13 | 21,67 | 8 | 25,00 | 79 | 23,17 | 61 | 19,93 | 0.46 (0.16-1.29) | 0.14 | 0.68 (0.34-1.35) | 0.27 | . | . | . |
| TCN2 ---- tag | rs4820872 | G/G | 14 | 23,33 | 13 | 40,63 | 131 | 38,42 | 115 | 37,58 | 1.00 (.-.) | . | 0.92 (0.48-1.77) | 0.80 | 0.17 | 0.99 | 0.28 |
| TCN2 ---- tag |  | G/A | 32 | 53,33 | 11 | 34,38 | 144 | 42,23 | 135 | 44,12 | 0.57 (0.22-1.44) | 0.23 | 0.91 (0.47-1.76) | 0.79 | . | . | . |
| TCN2 ---- tag |  | A/A | 14 | 23,33 | 8 | 25,00 | 66 | 19,35 | 56 | 18,30 | 0.50 (0.18-1.39) | 0.18 | 0.94 (0.47-1.88) | 0.86 | . | . | . |
| TCN2 ---- tag | rs4820874 | A/A | 48 | 80,00 | 20 | 62,50 | 232 | 68,04 | 226 | 73,86 | 1.00 (.-.) | . | 1.73 (1.00-2.99) | 0.05 | 0.23 | 0.99 | 0.34 |
| TCN2 ---- tag |  | A/G | 11 | 18,33 | 11 | 34,38 | 97 | 28,45 | 72 | 23,53 | 2.10 (0.92-4.84) | 0.08 | 1.68 (0.93-3.05) | 0.09 | . | . | . |
| TCN2 ---- tag |  | G/G | 1 | 1,67 | 1 | 3,13 | 12 | 3,52 | 8 | 2,61 | 0.00 (0.00-I) | 0.98 | 2.08 (0.79-5.53) | 0.14 | . | . | . |
| TCN2 ---- tag | rs4820886 | T/T | 54 | 90,00 | 25 | 78,13 | 264 | 77,42 | 256 | 83,66 | 1.00 (.-.) | . | 1.65 (1.03-2.67) | 0.04 | 0.02 | 0.99 | 0.15 |
| TCN2 ---- tag |  | T/G | 6 | 10,00 | 6 | 18,75 | 73 | 21,41 | 47 | 15,36 | 3.33 (1.22-9.10) | 0.02 | 1.41 (0.80-2.45) | 0.23 | . | . | . |
| TCN2 ---- tag |  | G/G | 0 | 0,00 | 1 | 3,13 | 4 | 1,17 | 3 | 0,98 | 0.63 (0.09-4.60) | 0.65 | 1.05 (0.14-7.98) | 0.96 | . | . | . |
| TCN2 ---- candidate | rs4820889 | G/G | 55 | 91,67 | 31 | 96,88 | 312 | 91,50 | 287 | 93,79 | 1.00 (.-.) | . | 1.30 (0.84-2.01) | 0.24 | 0.38 | 0.99 | 0.49 |
| TCN2 ---- candidate |  | G/A | 5 | 8,33 | 1 | 3,13 | 29 | 8,50 | 19 | 6,21 | 1.00 (.-.) | . | 1.30 (0.84-2.01) | 0.24 | . | . | . |
| TCN2 ---- candidate |  | A/A | . | . | . | . | . | . | . | . | 0.33 (0.04-2.46) | 0.28 | 0.99 (0.52-1.89) | 0.97 | . | . | . |
| TCN2 ---- tag | rs5997711 | C/C | 14 | 23,33 | 14 | 43,75 | 116 | 34,02 | 107 | 34,97 | 1.00 (.-.) | . | 0.68 (0.36-1.28) | 0.23 | 0.10 | 0.99 | 0.20 |
| TCN2 ---- tag |  | C/T | 35 | 58,33 | 10 | 31,25 | 157 | 46,04 | 144 | 47,06 | 0.27 (0.10-0.70) | 0.01 | 0.68 (0.36-1.28) | 0.24 | . | . | . |
| TCN2 ---- tag |  | T/T | 11 | 18,33 | 8 | 25,00 | 68 | 19,94 | 55 | 17,97 | 0.51 (0.19-1.43) | 0.20 | 0.71 (0.36-1.38) | 0.31 | . | . | . |
| TCN2 ---- tag | rs740234 | T/T | 44 | 73,33 | 21 | 65,63 | 222 | 65,10 | 197 | 64,38 | 1.00 (.-.) | . | 1.53 (0.88-2.64) | 0.13 | 0.99 | 1.00 | 0.99 |
| TCN2 ---- tag |  | T/C | 13 | 21,67 | 11 | 34,38 | 107 | 31,38 | 97 | 31,70 | 1.77 (0.78-4.01) | 0.17 | 1.53 (0.86-2.73) | 0.15 | . | . | . |
| TCN2 ---- tag |  | C/C | 3 | 5,00 | 0 | 0,00 | 12 | 3,52 | 12 | 3,92 | 0.00 (0.00-8E262) | 0.97 | 1.23 (0.48-3.10) | 0.67 | . | . | . |
| TCN2 ---- tag | rs740235 | G/G | 24 | 40,00 | 13 | 40,63 | 125 | 36,66 | 91 | 29,74 | 1.00 (.-.) | . | 1.18 (0.60-2.34) | 0.63 | 0.73 | 0.99 | 0.87 |
| TCN2 ---- tag |  | G/A | 25 | 41,67 | 11 | 34,38 | 158 | 46,33 | 153 | 50,00 | 0.57 (0.21-1.53) | 0.27 | 1.23 (0.62-2.42) | 0.55 | . | . | . |
| TCN2 ---- tag |  | A/A | 11 | 18,33 | 8 | 25,00 | 58 | 17,01 | 62 | 20,26 | 1.42 (0.54-3.74) | 0.48 | 1.28 (0.63-2.62) | 0.49 | . | . | . |
| TCN2 ---- candidate/singleton | rs9606756 | A/A | 53 | 88,33 | 23 | 71,88 | 264 | 77,42 | 252 | 82,35 | 1.00 (.-.) | . | 1.61 (0.99-2.61) | 0.06 | 0.09 | 0.99 | 0.20 |
| TCN2 ---- candidate/singleton |  | A/G | 7 | 11,67 | 8 | 25,00 | 71 | 20,82 | 50 | 16,34 | 2.33 (0.90-6.01) | 0.08 | 1.49 (0.86-2.60) | 0.16 | . | . | . |
| TCN2 ---- candidate/singleton |  | G/G | 0 | 0,00 | 1 | 3,13 | 6 | 1,76 | 4 | 1,31 | 0.68 (0.09-4.94) | 0.70 | 1.09 (0.14-8.37) | 0.93 | . | . | . |
| TCN2 ---- candidate | rs9621049 | C/C | 54 | 90,00 | 25 | 78,13 | 264 | 77,42 | 256 | 83,66 | 1.00 (.-.) | . | 1.65 (1.03-2.67) | 0.04 | 0.02 | 0.99 | 0.15 |
| TCN2 ---- candidate |  | C/T | 6 | 10,00 | 6 | 18,75 | 73 | 21,41 | 47 | 15,36 | 3.33 (1.22-9.10) | 0.02 | 1.41 (0.80-2.45) | 0.23 | . | . | . |
| TCN2 ---- candidate |  | T/T | 0 | 0,00 | 1 | 3,13 | 4 | 1,17 | 3 | 0,98 | 0.63 (0.09-4.60) | 0.65 | 1.05 (0.14-7.98) | 0.96 | . | . | . |
| TK1 ---- NA | rs1065769 | G/G | 32 | 53,33 | 11 | 34,38 | 161 | 47,21 | 146 | 47,71 | 1.00 (.-.) | . | 1.70 (0.86-3.38) | 0.13 | 0.89 | 1.00 | 0.92 |
| TK1 ---- NA |  | G/A | 23 | 38,33 | 19 | 59,38 | 140 | 41,06 | 139 | 45,42 | 2.02 (0.86-4.71) | 0.11 | 2.09 (1.04-4.20) | 0.04 | . | . | . |
| TK1 ---- NA |  | A/A | 5 | 8,33 | 2 | 6,25 | 40 | 11,73 | 21 | 6,86 | 0.36 (0.04-2.85) | 0.33 | 1.12 (0.49-2.55) | 0.79 | . | . | . |
| TK1 ---- NA | rs12232476 | G/G | 49 | 81,67 | 25 | 78,13 | 298 | 87,39 | 246 | 80,39 | 1.00 (.-.) | . | 1.26 (0.79-2.02) | 0.33 | 0.33 | 0.99 | 0.92 |
| TK1 ---- NA |  | G/A | 11 | 18,33 | 7 | 21,88 | 42 | 12,32 | 56 | 18,30 | 0.72 (0.24-2.15) | 0.56 | 1.46 (0.84-2.56) | 0.18 | . | . | . |
| TK1 ---- NA |  | A/A | 0 | 0,00 | 0 | 0,00 | 1 | 0,29 | 4 | 1,31 | 2.37 (0.71-7.88) | 0.16 | 2.99 (0.83-10.85) | 0.10 | . | . | . |
| TK1 ---- tag | rs16970907 | G/G | 50 | 83,33 | 27 | 84,38 | 307 | 90,03 | 266 | 86,93 | 1.00 (.-.) | . | 1.37 (0.87-2.14) | 0.17 | 0.81 | 0.99 | 0.92 |
| TK1 ---- tag |  | G/C | 9 | 15,00 | 5 | 15,63 | 30 | 8,80 | 40 | 13,07 | 1.33 (0.31-5.74) | 0.70 | 2.12 (1.21-3.72) | 0.01 | . | . | . |
| TK1 ---- tag |  | C/C | 1 | 1,67 | 0 | 0,00 | 4 | 1,17 | 0 | 0,00 | 0.00 (0.00-I) | 0.97 | 0.00 (0.00-I) | 0.98 | . | . | . |
| TK1 ---- tag | rs1811086 | C/C | 57 | 95,00 | 29 | 90,63 | 325 | 95,31 | 277 | 90,52 | 1.00 (.-.) | . | 1.40 (0.89-2.21) | 0.15 | 0.81 | 0.99 | 0.92 |
| TK1 ---- tag |  | C/T | 3 | 5,00 | 3 | 9,38 | 16 | 4,69 | 28 | 9,15 | 1.81 (0.53-6.22) | 0.35 | 2.17 (1.17-4.03) | 0.02 | . | . | . |
| TK1 ---- tag |  | T/T | 0 | 0,00 | 0 | 0,00 | 0 | 0,00 | 1 | 0,33 | 2.16 (0.28-16.59) | 0.46 | 3.03 (0.38-24.12) | 0.30 | . | . | . |
| TK1 ---- tag | rs2292235 | C/C | 22 | 36,67 | 9 | 28,13 | 118 | 34,60 | 97 | 31,70 | 1.00 (.-.) | . | 1.14 (0.56-2.29) | 0.72 | 0.32 | 0.99 | 0.92 |
| TK1 ---- tag |  | C/A | 22 | 36,67 | 16 | 50,00 | 164 | 48,09 | 146 | 47,71 | 0.91 (0.36-2.30) | 0.84 | 1.20 (0.60-2.41) | 0.60 | . | . | . |
| TK1 ---- tag |  | A/A | 16 | 26,67 | 7 | 21,88 | 59 | 17,30 | 63 | 20,59 | 0.72 (0.25-2.04) | 0.53 | 1.44 (0.69-3.00) | 0.33 | . | . | . |
| TK1 ---- tag | rs2854701 | A/A | 27 | 45,00 | 9 | 28,13 | 146 | 42,82 | 117 | 38,24 | 1.00 (.-.) | . | 1.96 (0.90-4.27) | 0.09 | 0.69 | 0.99 | 0.92 |
| TK1 ---- tag |  | A/G | 25 | 41,67 | 21 | 65,63 | 143 | 41,94 | 156 | 50,98 | 2.55 (1.05-6.20) | 0.04 | 2.54 (1.17-5.51) | 0.02 | . | . | . |
| TK1 ---- tag |  | G/G | 8 | 13,33 | 2 | 6,25 | 52 | 15,25 | 33 | 10,78 | 0.43 (0.05-3.54) | 0.43 | 1.58 (0.67-3.74) | 0.30 | . | . | . |
| TK1 ---- tag | rs2854702 | G/G | 46 | 76,67 | 22 | 68,75 | 284 | 83,28 | 215 | 70,26 | 1.00 (.-.) | . | 1.26 (0.76-2.08) | 0.37 | 0.39 | 0.99 | 0.92 |
| TK1 ---- tag |  | G/A | 14 | 23,33 | 10 | 31,25 | 55 | 16,13 | 85 | 27,78 | 0.96 (0.40-2.35) | 0.94 | 1.74 (1.01-3.02) | 0.05 | . | . | . |
| TK1 ---- tag |  | A/A | 0 | 0,00 | 0 | 0,00 | 2 | 0,59 | 6 | 1,96 | 2.50 (1.00-6.25) | 0.05 | 3.15 (1.12-8.82) | 0.03 | . | . | . |
| TK1 ---- tag | rs9897765 | G/G | 39 | 65,00 | 13 | 40,63 | 178 | 52,20 | 162 | 52,94 | 1.00 (.-.) | . | 1.70 (0.89-3.26) | 0.11 | 0.92 | 1.00 | 0.92 |
| TK1 ---- tag |  | G/A | 16 | 26,67 | 18 | 56,25 | 129 | 37,83 | 126 | 41,18 | 1.99 (0.87-4.60) | 0.11 | 1.80 (0.92-3.51) | 0.09 | . | . | . |
| TK1 ---- tag |  | A/A | 5 | 8,33 | 1 | 3,13 | 34 | 9,97 | 18 | 5,88 | 0.35 (0.04-2.76) | 0.32 | 1.38 (0.60-3.13) | 0.45 | . | . | . |
| TYMP ---- NA | rs131815 | G/G | 26 | 43,33 | 19 | 59,38 | 183 | 53,67 | 169 | 55,23 | 1.00 (.-.) | . | 1.22 (0.69-2.15) | 0.50 | 0.86 | 1.00 | 1.00 |
| TYMP ---- NA |  | G/A | 31 | 51,67 | 10 | 31,25 | 127 | 37,24 | 118 | 38,56 | 0.71 (0.30-1.71) | 0.45 | 1.36 (0.77-2.41) | 0.29 | . | . | . |
| TYMP ---- NA |  | A/A | 3 | 5,00 | 3 | 9,38 | 31 | 9,09 | 19 | 6,21 | 2.84 (0.77-10.47) | 0.12 | 1.40 (0.69-2.84) | 0.35 | . | . | . |
| TYMP ---- tag | rs131816 | A/A | 38 | 63,33 | 21 | 65,63 | 208 | 61,00 | 194 | 63,40 | 1.00 (.-.) | . | 1.49 (0.86-2.58) | 0.16 | 0.58 | 0.99 | 0.86 |
| TYMP ---- tag |  | A/G | 19 | 31,67 | 10 | 31,25 | 112 | 32,84 | 94 | 30,72 | 0.93 (0.40-2.15) | 0.86 | 1.20 (0.67-2.13) | 0.54 | . | . | . |
| TYMP ---- tag |  | G/G | 3 | 5,00 | 1 | 3,13 | 21 | 6,16 | 18 | 5,88 | 1.62 (0.21-12.62) | 0.64 | 1.07 (0.50-2.28) | 0.86 | . | . | . |
| TYMP ---- NA | rs131817 | C/C | 16 | 26,67 | 12 | 37,50 | 113 | 33,14 | 99 | 32,35 | 1.00 (.-.) | . | 1.48 (0.67-3.27) | 0.33 | 1.00 | 1.00 | 1.00 |
| TYMP ---- NA |  | C/T | 30 | 50,00 | 12 | 37,50 | 160 | 46,92 | 159 | 51,96 | 1.27 (0.47-3.39) | 0.64 | 1.60 (0.73-3.49) | 0.24 | . | . | . |
| TYMP ---- NA |  | T/T | 14 | 23,33 | 8 | 25,00 | 68 | 19,94 | 48 | 15,69 | 1.21 (0.43-3.42) | 0.72 | 1.80 (0.79-4.08) | 0.16 | . | . | . |
| TYMP ---- NA | rs140521 | T/T | 33 | 55,00 | 19 | 59,38 | 186 | 54,55 | 154 | 50,33 | 1.00 (.-.) | . | 1.41 (0.79-2.50) | 0.24 | 0.51 | 0.99 | 0.86 |
| TYMP ---- NA |  | T/G | 26 | 43,33 | 9 | 28,13 | 127 | 37,24 | 127 | 41,50 | 0.75 (0.29-1.88) | 0.53 | 1.25 (0.70-2.23) | 0.46 | . | . | . |
| TYMP ---- NA |  | G/G | 1 | 1,67 | 4 | 12,50 | 28 | 8,21 | 25 | 8,17 | 1.88 (0.61-5.86) | 0.27 | 1.38 (0.69-2.75) | 0.36 | . | . | . |
| TYMP ---- NA | rs140522 | G/G | 29 | 48,33 | 14 | 43,75 | 160 | 46,92 | 149 | 48,69 | 1.00 (.-.) | . | 1.63 (0.88-3.00) | 0.12 | 0.16 | 0.99 | 0.86 |
| TYMP ---- NA |  | G/A | 27 | 45,00 | 15 | 46,88 | 147 | 43,11 | 128 | 41,83 | 0.94 (0.40-2.21) | 0.90 | 1.36 (0.73-2.52) | 0.33 | . | . | . |
| TYMP ---- NA |  | A/A | 4 | 6,67 | 3 | 9,38 | 34 | 9,97 | 29 | 9,48 | 2.98 (0.83-10.73) | 0.10 | 1.10 (0.53-2.31) | 0.80 | . | . | . |
| TYMP ---- NA | rs140524 | G/G | 40 | 66,67 | 19 | 59,38 | 232 | 68,04 | 199 | 65,03 | 1.00 (.-.) | . | 1.48 (0.88-2.48) | 0.14 | 0.54 | 0.99 | 0.86 |
| TYMP ---- NA |  | G/A | 19 | 31,67 | 13 | 40,63 | 99 | 29,03 | 101 | 33,01 | 1.29 (0.54-3.06) | 0.56 | 1.53 (0.88-2.66) | 0.13 | . | . | . |
| TYMP ---- NA |  | A/A | 1 | 1,67 | 0 | 0,00 | 10 | 2,93 | 6 | 1,96 | 0.63 (0.25-1.57) | 0.33 | 0.94 (0.34-2.61) | 0.90 | . | . | . |
| TYMS ---- candidate literature | rs1001761 | C/C | 16 | 26,67 | 13 | 40,63 | 99 | 29,03 | 99 | 32,35 | 1.00 (.-.) | . | 1.37 (0.65-2.88) | 0.41 | 0.75 | 0.99 | 0.85 |
| TYMS ---- candidate literature |  | C/T | 33 | 55,00 | 14 | 43,75 | 164 | 48,09 | 152 | 49,67 | 0.95 (0.39-2.29) | 0.91 | 1.20 (0.57-2.50) | 0.64 | . | . | . |
| TYMS ---- candidate literature |  | T/T | 11 | 18,33 | 5 | 15,63 | 78 | 22,87 | 55 | 17,97 | 0.57 (0.15-2.16) | 0.41 | 1.07 (0.49-2.31) | 0.87 | . | . | . |
| TYMS ---- candidate literature/tag | rs10502289 | T/T | 35 | 58,33 | 24 | 75,00 | 207 | 60,70 | 205 | 66,99 | 1.00 (.-.) | . | 1.31 (0.79-2.18) | 0.30 | 0.90 | 1.00 | 0.90 |
| TYMS ---- candidate literature/tag |  | T/A | 25 | 41,67 | 7 | 21,88 | 116 | 34,02 | 82 | 26,80 | 0.60 (0.23-1.53) | 0.28 | 1.02 (0.59-1.76) | 0.94 | . | . | . |
| TYMS ---- candidate literature/tag |  | A/A | 0 | 0,00 | 1 | 3,13 | 18 | 5,28 | 19 | 6,21 | 9.31 (1.16-74.44) | 0.04 | 1.26 (0.62-2.55) | 0.52 | . | . | . |
| TYMS ---- tag | rs15872 | C/C | 27 | 45,00 | 18 | 56,25 | 164 | 48,09 | 155 | 50,65 | 1.00 (.-.) | . | 1.08 (0.59-1.99) | 0.80 | 0.37 | 0.99 | 0.85 |
| TYMS ---- tag |  | C/T | 30 | 50,00 | 12 | 37,50 | 144 | 42,23 | 120 | 39,22 | 0.58 (0.26-1.31) | 0.19 | 0.92 (0.50-1.72) | 0.80 | . | . | . |
| TYMS ---- tag |  | T/T | 3 | 5,00 | 2 | 6,25 | 33 | 9,68 | 31 | 10,13 | 0.98 (0.13-7.65) | 0.99 | 1.15 (0.56-2.33) | 0.71 | . | . | . |
| TYMS ---- tag | rs2244500 | T/T | 16 | 26,67 | 13 | 40,63 | 99 | 29,03 | 99 | 32,35 | 1.00 (.-.) | . | 1.37 (0.65-2.88) | 0.41 | 0.76 | 0.99 | 0.85 |
| TYMS ---- tag |  | T/C | 33 | 55,00 | 14 | 43,75 | 163 | 47,80 | 152 | 49,67 | 0.95 (0.39-2.29) | 0.91 | 1.20 (0.57-2.51) | 0.63 | . | . | . |
| TYMS ---- tag |  | C/C | 11 | 18,33 | 5 | 15,63 | 79 | 23,17 | 55 | 17,97 | 0.57 (0.15-2.16) | 0.41 | 1.05 (0.49-2.28) | 0.90 | . | . | . |
| TYMS ---- tag | rs2741182 | G/G | 37 | 61,67 | 20 | 62,50 | 198 | 58,06 | 187 | 61,11 | 1.00 (.-.) | . | 1.46 (0.86-2.48) | 0.17 | 0.70 | 0.99 | 0.85 |
| TYMS ---- tag |  | G/C | 22 | 36,67 | 10 | 31,25 | 117 | 34,31 | 103 | 33,66 | 0.96 (0.41-2.27) | 0.94 | 1.22 (0.70-2.13) | 0.49 | . | . | . |
| TYMS ---- tag |  | C/C | 1 | 1,67 | 2 | 6,25 | 26 | 7,62 | 16 | 5,23 | 1.55 (0.19-12.52) | 0.68 | 1.44 (0.69-3.01) | 0.33 | . | . | . |
| TYMS ---- candidate literature | rs2847149 | G/G | 16 | 26,67 | 13 | 40,63 | 99 | 29,03 | 99 | 32,35 | 1.00 (.-.) | . | 1.37 (0.65-2.88) | 0.41 | 0.75 | 0.99 | 0.85 |
| TYMS ---- candidate literature |  | G/A | 33 | 55,00 | 14 | 43,75 | 164 | 48,09 | 152 | 49,67 | 0.95 (0.39-2.29) | 0.91 | 1.20 (0.57-2.50) | 0.64 | . | . | . |
| TYMS ---- candidate literature |  | A/A | 11 | 18,33 | 5 | 15,63 | 78 | 22,87 | 55 | 17,97 | 0.57 (0.15-2.16) | 0.41 | 1.07 (0.49-2.31) | 0.87 | . | . | . |
| TYMS ---- candidate literature | rs2853533 | G/G | 45 | 75,00 | 24 | 75,00 | 256 | 75,07 | 228 | 74,51 | 1.00 (.-.) | . | 1.27 (0.77-2.10) | 0.35 | 0.59 | 0.99 | 0.85 |
| TYMS ---- candidate literature |  | G/C | 15 | 25,00 | 8 | 25,00 | 74 | 21,70 | 73 | 23,86 | 0.76 (0.31-1.86) | 0.55 | 1.27 (0.73-2.19) | 0.40 | . | . | . |
| TYMS ---- candidate literature |  | C/C | 0 | 0,00 | 0 | 0,00 | 11 | 3,23 | 5 | 1,63 | 0.90 (0.33-2.47) | 0.84 | 1.14 (0.38-3.41) | 0.81 | . | . | . |
| TYMS ---- tag | rs495139 | C/C | 21 | 35,00 | 6 | 18,75 | 131 | 38,42 | 101 | 33,01 | 1.00 (.-.) | . | 2.32 (0.83-6.52) | 0.11 | 0.56 | 0.99 | 0.85 |
| TYMS ---- tag |  | C/G | 30 | 50,00 | 19 | 59,38 | 149 | 43,70 | 149 | 48,69 | 2.60 (0.86-7.84) | 0.09 | 3.05 (1.10-8.50) | 0.03 | . | . | . |
| TYMS ---- tag |  | G/G | 9 | 15,00 | 7 | 21,88 | 61 | 17,89 | 56 | 18,30 | 1.75 (0.42-7.26) | 0.44 | 2.72 (0.95-7.74) | 0.06 | . | . | . |
| TYMS ---- candidate literature | rs502396 | T/T | 16 | 26,67 | 15 | 46,88 | 97 | 28,45 | 102 | 33,33 | 1.00 (.-.) | . | 1.11 (0.56-2.20) | 0.76 | 0.32 | 0.99 | 0.85 |
| TYMS ---- candidate literature |  | T/C | 30 | 50,00 | 12 | 37,50 | 164 | 48,09 | 143 | 46,73 | 0.72 (0.31-1.68) | 0.45 | 0.99 (0.51-1.94) | 0.99 | . | . | . |
| TYMS ---- candidate literature |  | C/C | 14 | 23,33 | 5 | 15,63 | 80 | 23,46 | 61 | 19,93 | 0.46 (0.12-1.68) | 0.24 | 0.99 (0.49-2.00) | 0.98 | . | . | . |
| UMPH2 ---- tag | rs2291028 | A/A | 18 | 30,00 | 13 | 40,63 | 149 | 43,70 | 131 | 42,81 | 1.00 (.-.) | . | 1.21 (0.60-2.46) | 0.59 | 0.71 | 0.99 | 0.88 |
| UMPH2 ---- tag |  | A/G | 30 | 50,00 | 13 | 40,63 | 149 | 43,70 | 142 | 46,41 | 0.70 (0.28-1.74) | 0.45 | 1.28 (0.63-2.59) | 0.50 | . | . | . |
| UMPH2 ---- tag |  | G/G | 12 | 20,00 | 6 | 18,75 | 43 | 12,61 | 33 | 10,78 | 1.57 (0.52-4.79) | 0.43 | 1.17 (0.52-2.60) | 0.70 | . | . | . |
| UMPH2 ---- NA | rs4789143 | A/A | 39 | 65,00 | 29 | 90,63 | 255 | 74,78 | 249 | 81,37 | 1.00 (.-.) | . | 1.39 (0.87-2.21) | 0.16 | 0.65 | 0.99 | 0.88 |
| UMPH2 ---- NA |  | A/G | 19 | 31,67 | 2 | 6,25 | 81 | 23,75 | 55 | 17,97 | 0.47 (0.11-2.04) | 0.32 | 1.04 (0.61-1.77) | 0.89 | . | . | . |
| UMPH2 ---- NA |  | G/G | 2 | 3,33 | 1 | 3,13 | 5 | 1,47 | 2 | 0,65 | 8.86 (1.12-69.87) | 0.04 | 0.53 (0.07-3.96) | 0.53 | . | . | . |
| UMPH2 ---- NA | rs750844 | G/G | 23 | 38,33 | 16 | 50,00 | 177 | 51,91 | 156 | 50,98 | 1.00 (.-.) | . | 1.16 (0.63-2.16) | 0.63 | 0.88 | 1.00 | 0.88 |
| UMPH2 ---- NA |  | G/A | 30 | 50,00 | 12 | 37,50 | 137 | 40,18 | 127 | 41,50 | 0.58 (0.24-1.37) | 0.21 | 1.11 (0.59-2.09) | 0.74 | . | . | . |
| UMPH2 ---- NA |  | A/A | 7 | 11,67 | 4 | 12,50 | 27 | 7,92 | 23 | 7,52 | 2.62 (0.71-9.67) | 0.15 | 1.02 (0.47-2.21) | 0.96 | . | . | . |
| UMPK ---- tag | rs11582877 | C/C | 45 | 75,00 | 24 | 75,00 | 254 | 74,49 | 220 | 71,90 | 1.00 (.-.) | . | 1.36 (0.82-2.24) | 0.23 | 0.99 | 1.00 | 1.00 |
| UMPK ---- tag |  | C/T | 13 | 21,67 | 7 | 21,88 | 76 | 22,29 | 78 | 25,49 | 0.88 (0.34-2.26) | 0.79 | 1.29 (0.75-2.24) | 0.36 | . | . | . |
| UMPK ---- tag |  | T/T | 2 | 3,33 | 1 | 3,13 | 11 | 3,23 | 8 | 2,61 | 1.14 (0.15-8.67) | 0.90 | 1.22 (0.48-3.13) | 0.67 | . | . | . |
| UMPK ---- tag | rs2622903 | A/A | 30 | 50,00 | 16 | 50,00 | 170 | 49,85 | 146 | 47,71 | 1.00 (.-.) | . | 1.28 (0.69-2.38) | 0.43 | 0.99 | 1.00 | 1.00 |
| UMPK ---- tag |  | A/G | 25 | 41,67 | 12 | 37,50 | 135 | 39,59 | 134 | 43,79 | 0.80 (0.34-1.87) | 0.61 | 1.26 (0.67-2.35) | 0.47 | . | . | . |
| UMPK ---- tag |  | G/G | 5 | 8,33 | 4 | 12,50 | 36 | 10,56 | 26 | 8,50 | 1.19 (0.26-5.43) | 0.82 | 1.04 (0.48-2.23) | 0.93 | . | . | . |
| UMPK ---- tag | rs2820989 | C/C | 19 | 31,67 | 11 | 34,38 | 108 | 31,67 | 92 | 30,07 | 1.00 (.-.) | . | 1.18 (0.56-2.50) | 0.66 | 1.00 | 1.00 | 1.00 |
| UMPK ---- tag |  | C/G | 30 | 50,00 | 13 | 40,63 | 161 | 47,21 | 147 | 48,04 | 0.78 (0.31-1.99) | 0.61 | 1.30 (0.62-2.73) | 0.48 | . | . | . |
| UMPK ---- tag |  | G/G | 11 | 18,33 | 8 | 25,00 | 72 | 21,11 | 67 | 21,90 | 0.90 (0.30-2.68) | 0.85 | 0.99 (0.45-2.16) | 0.98 | . | . | . |
| UMPK ---- tag | rs6660321 | A/A | 45 | 75,00 | 25 | 78,13 | 262 | 76,83 | 227 | 74,18 | 1.00 (.-.) | . | 1.29 (0.79-2.10) | 0.30 | 0.75 | 0.99 | 1.00 |
| UMPK ---- tag |  | A/C | 13 | 21,67 | 6 | 18,75 | 72 | 21,11 | 73 | 23,86 | 0.70 (0.26-1.92) | 0.49 | 1.22 (0.71-2.10) | 0.46 | . | . | . |
| UMPK ---- tag |  | C/C | 2 | 3,33 | 1 | 3,13 | 7 | 2,05 | 6 | 1,96 | 1.09 (0.14-8.23) | 0.94 | 1.18 (0.43-3.22) | 0.74 | . | . | . |
| UMPK ---- tag | rs6690084 | T/T | 54 | 90,00 | 31 | 96,88 | 300 | 87,98 | 262 | 85,62 | 1.00 (.-.) | . | 1.23 (0.80-1.90) | 0.34 | 0.03 | 0.99 | 0.13 |
| UMPK ---- tag |  | T/C | 6 | 10,00 | 1 | 3,13 | 41 | 12,02 | 41 | 13,40 | 0.00 (0.00-3E226) | 0.96 | 1.35 (0.78-2.35) | 0.28 | . | . | . |
| UMPK ---- tag |  | C/C | 0 | 0,00 | 0 | 0,00 | 0 | 0,00 | 3 | 0,98 | 1.00 (0.24-4.21) | 1.00 | 1.24 (0.28-5.43) | 0.78 | . | . | . |
| UMPS ---- NA | rs1162 | A/A | 32 | 53,33 | 15 | 46,88 | 175 | 51,32 | 132 | 43,14 | 1.00 (.-.) | . | 1.08 (0.58-2.01) | 0.82 | 0.25 | 0.99 | 0.50 |
| UMPS ---- NA |  | A/G | 26 | 43,33 | 15 | 46,88 | 138 | 40,47 | 128 | 41,83 | 0.88 (0.38-2.05) | 0.77 | 1.39 (0.75-2.60) | 0.30 | . | . | . |
| UMPS ---- NA |  | G/G | 2 | 3,33 | 2 | 6,25 | 28 | 8,21 | 46 | 15,03 | 0.84 (0.18-3.91) | 0.83 | 1.93 (0.98-3.82) | 0.06 | . | . | . |
| UMPS ---- tag | rs13146 | C/C | 44 | 73,33 | 21 | 65,63 | 243 | 71,26 | 209 | 68,30 | 1.00 (.-.) | . | 1.17 (0.70-1.97) | 0.54 | 0.21 | 0.99 | 0.50 |
| UMPS ---- tag |  | C/T | 15 | 25,00 | 10 | 31,25 | 86 | 25,22 | 82 | 26,80 | 0.77 (0.33-1.82) | 0.56 | 1.33 (0.77-2.31) | 0.31 | . | . | . |
| UMPS ---- tag |  | T/T | 1 | 1,67 | 1 | 3,13 | 12 | 3,52 | 15 | 4,90 | 0.00 (0.00-I) | 0.98 | 2.24 (1.07-4.67) | 0.03 | . | . | . |
| UMPS ---- tag | rs16835902 | C/C | 20 | 33,33 | 15 | 46,88 | 112 | 32,84 | 97 | 31,70 | 1.00 (.-.) | . | 1.17 (0.62-2.21) | 0.63 | 0.74 | 0.99 | 0.85 |
| UMPS ---- tag |  | C/G | 27 | 45,00 | 13 | 40,63 | 159 | 46,63 | 161 | 52,61 | 0.66 (0.27-1.58) | 0.35 | 1.14 (0.62-2.11) | 0.67 | . | . | . |
| UMPS ---- tag |  | G/G | 13 | 21,67 | 4 | 12,50 | 70 | 20,53 | 48 | 15,69 | 0.94 (0.30-2.97) | 0.92 | 1.11 (0.57-2.17) | 0.76 | . | . | . |
| UMPS ---- tag | rs17282057 | T/T | 48 | 80,00 | 26 | 81,25 | 272 | 79,77 | 218 | 71,24 | 1.00 (.-.) | . | 1.36 (0.85-2.19) | 0.20 | 0.85 | 1.00 | 0.85 |
| UMPS ---- tag |  | T/C | 11 | 18,33 | 5 | 15,63 | 65 | 19,06 | 81 | 26,47 | 1.03 (0.34-3.10) | 0.95 | 1.56 (0.93-2.61) | 0.09 | . | . | . |
| UMPS ---- tag |  | C/C | 1 | 1,67 | 1 | 3,13 | 4 | 1,17 | 7 | 2,29 | 2.25 (0.29-17.23) | 0.43 | 1.62 (0.37-7.07) | 0.52 | . | . | . |
| UMPS ---- tag | rs606552 | A/A | 28 | 46,67 | 19 | 59,38 | 167 | 48,97 | 176 | 57,52 | 1.00 (.-.) | . | 1.28 (0.73-2.24) | 0.39 | 0.68 | 0.99 | 0.85 |
| UMPS ---- tag |  | A/G | 24 | 40,00 | 12 | 37,50 | 139 | 40,76 | 112 | 36,60 | 0.73 (0.32-1.68) | 0.46 | 1.10 (0.62-1.95) | 0.74 | . | . | . |
| UMPS ---- tag |  | G/G | 8 | 13,33 | 1 | 3,13 | 35 | 10,26 | 18 | 5,88 | 0.58 (0.07-4.52) | 0.60 | 0.96 (0.46-2.00) | 0.91 | . | . | . |
| UMPS ---- tag | rs694897 | C/C | 27 | 45,00 | 14 | 43,75 | 136 | 39,88 | 114 | 37,25 | 1.00 (.-.) | . | 1.89 (1.00-3.58) | 0.05 | 0.23 | 0.99 | 0.50 |
| UMPS ---- tag |  | C/G | 24 | 40,00 | 12 | 37,50 | 162 | 47,51 | 152 | 49,67 | 1.65 (0.69-3.96) | 0.26 | 1.64 (0.87-3.08) | 0.13 | . | . | . |
| UMPS ---- tag |  | G/G | 9 | 15,00 | 6 | 18,75 | 43 | 12,61 | 40 | 13,07 | 1.56 (0.49-5.01) | 0.46 | 1.71 (0.84-3.47) | 0.14 | . | . | . |
| UNG ---- NA | rs1059262 | T/T | 42 | 70,00 | 20 | 62,50 | 231 | 67,74 | 211 | 68,95 | 1.00 (.-.) | . | 1.34 (0.79-2.27) | 0.28 | 0.92 | 1.00 | 0.92 |
| UNG ---- NA |  | T/G | 16 | 26,67 | 11 | 34,38 | 96 | 28,15 | 88 | 28,76 | 0.90 (0.38-2.14) | 0.82 | 1.31 (0.75-2.29) | 0.35 | . | . | . |
| UNG ---- NA |  | G/G | 2 | 3,33 | 1 | 3,13 | 14 | 4,11 | 7 | 2,29 | 1.10 (0.14-8.43) | 0.93 | 1.43 (0.51-3.98) | 0.50 | . | . | . |
| UNG ---- tag | rs2160603 | T/T | 40 | 66,67 | 24 | 75,00 | 232 | 68,04 | 206 | 67,32 | 1.00 (.-.) | . | 1.14 (0.69-1.88) | 0.61 | 0.13 | 0.99 | 0.38 |
| UNG ---- tag |  | T/C | 17 | 28,33 | 7 | 21,88 | 97 | 28,45 | 85 | 27,78 | 0.74 (0.29-1.90) | 0.53 | 1.43 (0.83-2.48) | 0.20 | . | . | . |
| UNG ---- tag |  | C/C | 3 | 5,00 | 1 | 3,13 | 12 | 3,52 | 15 | 4,90 | 0.00 (0.00-3E224) | 0.97 | 1.75 (0.83-3.67) | 0.14 | . | . | . |
| UNG ---- tag | rs246079 | A/A | 16 | 26,67 | 8 | 25,00 | 102 | 29,91 | 100 | 32,68 | 1.00 (.-.) | . | 1.44 (0.64-3.24) | 0.37 | 0.80 | 0.99 | 0.92 |
| UNG ---- tag |  | A/G | 31 | 51,67 | 17 | 53,13 | 180 | 52,79 | 142 | 46,41 | 1.10 (0.43-2.81) | 0.85 | 1.32 (0.59-2.92) | 0.50 | . | . | . |
| UNG ---- tag |  | G/G | 13 | 21,67 | 7 | 21,88 | 59 | 17,30 | 64 | 20,92 | 0.92 (0.29-2.98) | 0.90 | 1.66 (0.72-3.83) | 0.24 | . | . | . |
| UNG ---- NA | rs246085 | T/T | 51 | 85,00 | 24 | 75,00 | 297 | 87,10 | 276 | 90,20 | 1.00 (.-.) | . | 1.47 (0.89-2.43) | 0.13 | 0.56 | 0.99 | 0.92 |
| UNG ---- NA |  | T/C | 9 | 15,00 | 8 | 25,00 | 44 | 12,90 | 29 | 9,48 | 1.29 (0.53-3.15) | 0.58 | 1.32 (0.70-2.49) | 0.38 | . | . | . |
| UNG ---- NA |  | C/C | 0 | 0,00 | 0 | 0,00 | 0 | 0,00 | 1 | 0,33 | 4.03 (0.51-32.01) | 0.19 | 5.94 (0.72-48.79) | 0.10 | . | . | . |
| UNG ---- NA | rs2569987 | A/A | 48 | 80,00 | 23 | 71,88 | 226 | 66,28 | 222 | 72,55 | 1.00 (.-.) | . | 1.93 (1.14-3.27) | 0.01 | 0.03 | 0.99 | 0.18 |
| UNG ---- NA |  | A/G | 9 | 15,00 | 9 | 28,13 | 99 | 29,03 | 76 | 24,84 | 4.53 (1.96-10.45) | <.001 | 1.65 (0.94-2.93) | 0.08 | . | . | . |
| UNG ---- NA |  | G/G | 3 | 5,00 | 0 | 0,00 | 16 | 4,69 | 8 | 2,61 | 0.00 (0.00-I) | 0.98 | 1.52 (0.61-3.79) | 0.37 | . | . | . |
| UNG ---- tag | rs3219243 | T/T | 36 | 60,00 | 20 | 62,50 | 224 | 65,69 | 199 | 65,03 | 1.00 (.-.) | . | 1.49 (0.89-2.50) | 0.13 | 0.69 | 0.99 | 0.92 |
| UNG ---- tag |  | T/C | 22 | 36,67 | 11 | 34,38 | 108 | 31,67 | 89 | 29,08 | 1.07 (0.43-2.65) | 0.88 | 1.17 (0.67-2.03) | 0.59 | . | . | . |
| UNG ---- tag |  | C/C | 2 | 3,33 | 1 | 3,13 | 9 | 2,64 | 18 | 5,88 | 1.35 (0.17-10.87) | 0.78 | 2.28 (1.05-4.92) | 0.04 | . | . | . |

|  | | | **adchem_5fu = 0** | | | | **adchem_5fu = 1** | | | | **one reference** | | | | | | |
| --- | --- | --- | --- | --- | --- | --- | --- | --- | --- | --- | --- | --- | --- | --- | --- | --- | --- |
|  | | | **Ctrl** | | **Cases** | | **Ctrl** | | **Cases** | | **adchem_5fu = 0** | | **adchem_5fu = 1** | |  | | |
| **Gene** | **SNP** | **Genotype** | **N** | **%** | **N** | **%** | **N** | **%** | **N** | **%** | **HR (95%-CI)** | **p** | **HR (95%-CI)** | **p** | **LR_pInt** | **FDR_pInt** | **FDR(byGene)_pInt** |
| AARS ---- tag | rs2070203 | 0 | 17 | 28,33 | 11 | 34,38 | 104 | 30,50 | 84 | 27,45 | 1.00 (.-.) | . | 0.89 (0.46-1.74) | 0.74 | 0.12 | 0.99 | 0.13 |
| AARS ---- tag |  | 1 | 27 | 45,00 | 14 | 43,75 | 176 | 51,61 | 152 | 49,67 | 0.73 (0.42-1.25) | 0.25 | 1.02 (0.54-1.94) | 0.95 | . | . | . |
| AARS ---- tag |  | 2 | 16 | 26,67 | 7 | 21,88 | 61 | 17,89 | 70 | 22,88 | 0.53 (0.18-1.56) | 0.25 | 1.17 (0.60-2.29) | 0.65 | . | . | . |
| AARS ---- tag | rs34087264 | 0 | 21 | 35,00 | 7 | 21,88 | 86 | 25,22 | 95 | 31,05 | 1.00 (.-.) | . | 2.10 (1.02-4.31) | 0.04 | 0.13 | 0.99 | 0.13 |
| AARS ---- tag |  | 1 | 23 | 38,33 | 17 | 53,13 | 174 | 51,03 | 145 | 47,39 | 1.30 (0.75-2.24) | 0.35 | 1.74 (0.86-3.50) | 0.12 | . | . | . |
| AARS ---- tag |  | 2 | 16 | 26,67 | 8 | 25,00 | 81 | 23,75 | 66 | 21,57 | 1.68 (0.57-5.00) | 0.35 | 1.44 (0.69-2.98) | 0.33 | . | . | . |
| ABCC4 ---- tag | rs10508023 | 0 | 49 | 81,67 | 25 | 78,13 | 267 | 78,30 | 250 | 81,70 | 1.00 (.-.) | . | 1.50 (0.94-2.40) | 0.09 | 0.34 | 0.99 | 1.00 |
| ABCC4 ---- tag |  | 1 | 10 | 16,67 | 7 | 21,88 | 71 | 20,82 | 49 | 16,01 | 1.31 (0.55-3.12) | 0.55 | 1.22 (0.72-2.05) | 0.46 | . | . | . |
| ABCC4 ---- tag |  | 2 | 1 | 1,67 | 0 | 0,00 | 3 | 0,88 | 7 | 2,29 | 1.71 (0.30-9.73) | 0.55 | 0.99 (0.48-2.02) | 0.97 | . | . | . |
| ABCC4 ---- tag | rs1059751 | 0 | 20 | 33,33 | 8 | 25,00 | 81 | 23,75 | 78 | 25,49 | 1.00 (.-.) | . | 1.56 (0.75-3.24) | 0.24 | 0.66 | 0.99 | 1.00 |
| ABCC4 ---- tag |  | 1 | 33 | 55,00 | 20 | 62,50 | 191 | 56,01 | 161 | 52,61 | 1.19 (0.59-2.43) | 0.63 | 1.57 (0.77-3.20) | 0.21 | . | . | . |
| ABCC4 ---- tag |  | 2 | 7 | 11,67 | 4 | 12,50 | 69 | 20,23 | 67 | 21,90 | 1.42 (0.34-5.89) | 0.63 | 1.59 (0.76-3.32) | 0.22 | . | . | . |
| ABCC4 ---- tag | rs11568643 | 0 | 50 | 83,33 | 26 | 81,25 | 286 | 83,87 | 256 | 83,66 | 1.00 (.-.) | . | 1.45 (0.91-2.30) | 0.12 | 0.47 | 0.99 | 1.00 |
| ABCC4 ---- tag |  | 1 | 10 | 16,67 | 5 | 15,63 | 55 | 16,13 | 48 | 15,69 | 1.19 (0.54-2.62) | 0.66 | 1.24 (0.71-2.16) | 0.45 | . | . | . |
| ABCC4 ---- tag |  | 2 | 0 | 0,00 | 1 | 3,13 | 0 | 0,00 | 2 | 0,65 | 1.42 (0.29-6.85) | 0.66 | 1.06 (0.47-2.38) | 0.88 | . | . | . |
| ABCC4 ---- NA | rs11568658 | 0 | 56 | 93,33 | 31 | 96,88 | 321 | 94,13 | 293 | 95,75 | 1.00 (.-.) | . | 1.37 (0.89-2.13) | 0.16 | 0.96 | 1.00 | 1.00 |
| ABCC4 ---- NA |  | 1 | 4 | 6,67 | 1 | 3,13 | 20 | 5,87 | 13 | 4,25 | 1.15 (0.15-8.61) | 0.89 | 1.51 (0.73-3.13) | 0.27 | . | . | . |
| ABCC4 ---- NA |  | 2 | 0 | 0,00 | 0 | 0,00 | 0 | 0,00 | 0 | 0,00 | 1.32 (0.02-74.18) | 0.89 | 1.65 (0.46-5.93) | 0.44 | . | . | . |
| ABCC4 ---- tag | rs12864049 | 0 | 42 | 70,00 | 22 | 68,75 | 266 | 78,01 | 226 | 73,86 | 1.00 (.-.) | . | 1.36 (0.80-2.31) | 0.26 | 0.86 | 1.00 | 1.00 |
| ABCC4 ---- tag |  | 1 | 16 | 26,67 | 9 | 28,13 | 69 | 20,23 | 75 | 24,51 | 1.19 (0.55-2.58) | 0.66 | 1.74 (1.00-3.04) | 0.05 | . | . | . |
| ABCC4 ---- tag |  | 2 | 2 | 3,33 | 1 | 3,13 | 6 | 1,76 | 5 | 1,63 | 1.42 (0.30-6.68) | 0.66 | 2.23 (1.12-4.45) | 0.02 | . | . | . |
| ABCC4 ---- tag | rs1628382 | 0 | 38 | 63,33 | 23 | 71,88 | 209 | 61,29 | 189 | 61,76 | 1.00 (.-.) | . | 1.42 (0.86-2.37) | 0.17 | 0.77 | 0.99 | 1.00 |
| ABCC4 ---- tag |  | 1 | 20 | 33,33 | 8 | 25,00 | 116 | 34,02 | 104 | 33,99 | 1.16 (0.53-2.53) | 0.71 | 1.46 (0.87-2.46) | 0.15 | . | . | . |
| ABCC4 ---- tag |  | 2 | 2 | 3,33 | 1 | 3,13 | 16 | 4,69 | 13 | 4,25 | 1.35 (0.28-6.39) | 0.71 | 1.50 (0.81-2.78) | 0.20 | . | . | . |
| ABCC4 ---- tag | rs1678354 | 0 | 20 | 33,33 | 12 | 37,50 | 153 | 44,87 | 126 | 41,18 | 1.00 (.-.) | . | 1.48 (0.74-3.00) | 0.27 | 0.77 | 0.99 | 1.00 |
| ABCC4 ---- tag |  | 1 | 33 | 55,00 | 18 | 56,25 | 144 | 42,23 | 148 | 48,37 | 1.23 (0.59-2.57) | 0.59 | 1.63 (0.82-3.24) | 0.17 | . | . | . |
| ABCC4 ---- tag |  | 2 | 7 | 11,67 | 2 | 6,25 | 44 | 12,90 | 32 | 10,46 | 1.51 (0.34-6.61) | 0.59 | 1.79 (0.86-3.69) | 0.12 | . | . | . |
| ABCC4 ---- tag | rs1678383 | 0 | 48 | 80,00 | 27 | 84,38 | 282 | 82,70 | 249 | 81,37 | 1.00 (.-.) | . | 1.10 (0.69-1.74) | 0.70 | 0.04 | 0.99 | 0.76 |
| ABCC4 ---- tag |  | 1 | 11 | 18,33 | 5 | 15,63 | 54 | 15,84 | 54 | 17,65 | 0.35 (0.10-1.20) | 0.10 | 1.23 (0.73-2.07) | 0.43 | . | . | . |
| ABCC4 ---- tag |  | 2 | 1 | 1,67 | 0 | 0,00 | 5 | 1,47 | 3 | 0,98 | 0.12 (0.01-1.44) | 0.10 | 1.38 (0.68-2.83) | 0.38 | . | . | . |
| ABCC4 ---- tag | rs1678395 | 0 | 51 | 85,00 | 26 | 81,25 | 285 | 83,58 | 262 | 85,62 | 1.00 (.-.) | . | 1.51 (0.95-2.38) | 0.08 | 0.61 | 0.99 | 1.00 |
| ABCC4 ---- tag |  | 1 | 9 | 15,00 | 6 | 18,75 | 53 | 15,54 | 42 | 13,73 | 0.87 (0.26-2.98) | 0.83 | 0.93 (0.54-1.60) | 0.80 | . | . | . |
| ABCC4 ---- tag |  | 2 | 0 | 0,00 | 0 | 0,00 | 3 | 0,88 | 2 | 0,65 | 0.76 (0.07-8.87) | 0.83 | 0.58 (0.26-1.27) | 0.17 | . | . | . |
| ABCC4 ---- tag | rs1678405 | 0 | 24 | 40,00 | 21 | 65,63 | 156 | 45,75 | 132 | 43,14 | 1.00 (.-.) | . | 1.15 (0.67-1.97) | 0.62 | 0.41 | 0.99 | 1.00 |
| ABCC4 ---- tag |  | 1 | 32 | 53,33 | 10 | 31,25 | 157 | 46,04 | 140 | 45,75 | 0.86 (0.38-1.96) | 0.73 | 1.41 (0.83-2.38) | 0.21 | . | . | . |
| ABCC4 ---- tag |  | 2 | 4 | 6,67 | 1 | 3,13 | 28 | 8,21 | 34 | 11,11 | 0.75 (0.14-3.86) | 0.73 | 1.72 (0.96-3.09) | 0.07 | . | . | . |
| ABCC4 ---- tag | rs17189540 | 0 | 53 | 88,33 | 26 | 81,25 | 303 | 88,86 | 260 | 84,97 | 1.00 (.-.) | . | 1.42 (0.88-2.27) | 0.15 | 0.76 | 0.99 | 1.00 |
| ABCC4 ---- tag |  | 1 | 7 | 11,67 | 5 | 15,63 | 38 | 11,14 | 46 | 15,03 | 1.28 (0.46-3.52) | 0.63 | 1.52 (0.86-2.70) | 0.15 | . | . | . |
| ABCC4 ---- tag |  | 2 | 0 | 0,00 | 1 | 3,13 | 0 | 0,00 | 0 | 0,00 | 1.64 (0.22-12.42) | 0.63 | 1.64 (0.70-3.83) | 0.25 | . | . | . |
| ABCC4 ---- tag | rs17235152 | 0 | 41 | 68,33 | 24 | 75,00 | 247 | 72,43 | 226 | 73,86 | 1.00 (.-.) | . | 1.33 (0.80-2.22) | 0.28 | 0.81 | 0.99 | 1.00 |
| ABCC4 ---- tag |  | 1 | 17 | 28,33 | 8 | 25,00 | 86 | 25,22 | 75 | 24,51 | 0.94 (0.41-2.15) | 0.88 | 1.39 (0.80-2.41) | 0.24 | . | . | . |
| ABCC4 ---- tag |  | 2 | 2 | 3,33 | 0 | 0,00 | 8 | 2,35 | 5 | 1,63 | 0.88 (0.17-4.61) | 0.88 | 1.45 (0.72-2.93) | 0.30 | . | . | . |
| ABCC4 ---- tag | rs17268122 | 0 | 28 | 46,67 | 18 | 56,25 | 214 | 62,76 | 206 | 67,32 | 1.00 (.-.) | . | 1.20 (0.68-2.12) | 0.54 | 0.63 | 0.99 | 1.00 |
| ABCC4 ---- tag |  | 1 | 28 | 46,67 | 12 | 37,50 | 111 | 32,55 | 84 | 27,45 | 0.73 (0.37-1.42) | 0.36 | 1.04 (0.58-1.86) | 0.91 | . | . | . |
| ABCC4 ---- tag |  | 2 | 4 | 6,67 | 2 | 6,25 | 16 | 4,69 | 16 | 5,23 | 0.53 (0.14-2.03) | 0.36 | 0.90 (0.45-1.79) | 0.76 | . | . | . |
| ABCC4 ---- tag | rs17268170 | 0 | 47 | 78,33 | 24 | 75,00 | 284 | 83,28 | 246 | 80,39 | 1.00 (.-.) | . | 1.58 (0.96-2.62) | 0.07 | 0.22 | 0.99 | 1.00 |
| ABCC4 ---- tag |  | 1 | 13 | 21,67 | 4 | 12,50 | 56 | 16,42 | 58 | 18,95 | 1.56 (0.79-3.07) | 0.20 | 1.52 (0.87-2.65) | 0.14 | . | . | . |
| ABCC4 ---- tag |  | 2 | 0 | 0,00 | 4 | 12,50 | 1 | 0,29 | 2 | 0,65 | 2.44 (0.63-9.42) | 0.20 | 1.46 (0.68-3.10) | 0.33 | . | . | . |
| ABCC4 ---- tag | rs1729764 | 0 | 44 | 73,33 | 27 | 84,38 | 259 | 75,95 | 246 | 80,39 | 1.00 (.-.) | . | 1.31 (0.84-2.06) | 0.24 | 0.50 | 0.99 | 1.00 |
| ABCC4 ---- tag |  | 1 | 15 | 25,00 | 3 | 9,38 | 78 | 22,87 | 55 | 17,97 | 0.64 (0.16-2.52) | 0.53 | 1.31 (0.79-2.18) | 0.30 | . | . | . |
| ABCC4 ---- tag |  | 2 | 1 | 1,67 | 2 | 6,25 | 4 | 1,17 | 5 | 1,63 | 0.41 (0.03-6.36) | 0.53 | 1.31 (0.65-2.67) | 0.45 | . | . | . |
| ABCC4 ---- tag | rs1729767 | 0 | 29 | 48,33 | 14 | 43,75 | 183 | 53,67 | 156 | 50,98 | 1.00 (.-.) | . | 1.39 (0.78-2.47) | 0.26 | 0.95 | 1.00 | 1.00 |
| ABCC4 ---- tag |  | 1 | 24 | 40,00 | 18 | 56,25 | 140 | 41,06 | 132 | 43,14 | 1.12 (0.58-2.17) | 0.73 | 1.53 (0.86-2.71) | 0.15 | . | . | . |
| ABCC4 ---- tag |  | 2 | 7 | 11,67 | 0 | 0,00 | 18 | 5,28 | 18 | 5,88 | 1.26 (0.34-4.72) | 0.73 | 1.68 (0.88-3.19) | 0.11 | . | . | . |
| ABCC4 ---- tag | rs17300935 | 0 | 40 | 66,67 | 21 | 65,63 | 252 | 73,90 | 233 | 76,14 | 1.00 (.-.) | . | 1.34 (0.79-2.27) | 0.27 | 0.87 | 1.00 | 1.00 |
| ABCC4 ---- tag |  | 1 | 18 | 30,00 | 11 | 34,38 | 82 | 24,05 | 70 | 22,88 | 0.95 (0.43-2.14) | 0.91 | 1.37 (0.78-2.41) | 0.27 | . | . | . |
| ABCC4 ---- tag |  | 2 | 2 | 3,33 | 0 | 0,00 | 7 | 2,05 | 3 | 0,98 | 0.91 (0.18-4.56) | 0.91 | 1.41 (0.69-2.88) | 0.35 | . | . | . |
| ABCC4 ---- tag | rs1750190 | 0 | 18 | 30,00 | 8 | 25,00 | 91 | 26,69 | 72 | 23,53 | 1.00 (.-.) | . | 1.53 (0.75-3.12) | 0.25 | 0.67 | 0.99 | 1.00 |
| ABCC4 ---- tag |  | 1 | 29 | 48,33 | 17 | 53,13 | 166 | 48,68 | 158 | 51,63 | 1.23 (0.71-2.13) | 0.46 | 1.66 (0.83-3.31) | 0.15 | . | . | . |
| ABCC4 ---- tag |  | 2 | 13 | 21,67 | 7 | 21,88 | 84 | 24,63 | 76 | 24,84 | 1.51 (0.51-4.52) | 0.46 | 1.80 (0.89-3.66) | 0.10 | . | . | . |
| ABCC4 ---- tag | rs1750996 | 0 | 33 | 55,00 | 21 | 65,63 | 224 | 65,69 | 201 | 65,69 | 1.00 (.-.) | . | 1.33 (0.79-2.22) | 0.28 | 0.81 | 0.99 | 1.00 |
| ABCC4 ---- tag |  | 1 | 25 | 41,67 | 8 | 25,00 | 111 | 32,55 | 94 | 30,72 | 1.08 (0.50-2.33) | 0.85 | 1.58 (0.93-2.69) | 0.09 | . | . | . |
| ABCC4 ---- tag |  | 2 | 2 | 3,33 | 3 | 9,38 | 6 | 1,76 | 11 | 3,59 | 1.16 (0.25-5.43) | 0.85 | 1.88 (0.98-3.59) | 0.06 | . | . | . |
| ABCC4 ---- tag | rs1751025 | 0 | 27 | 45,00 | 18 | 56,25 | 167 | 48,97 | 134 | 43,79 | 1.00 (.-.) | . | 1.56 (0.87-2.78) | 0.14 | 0.41 | 0.99 | 1.00 |
| ABCC4 ---- tag |  | 1 | 28 | 46,67 | 8 | 25,00 | 146 | 42,82 | 134 | 43,79 | 1.48 (0.82-2.69) | 0.19 | 1.77 (1.00-3.13) | 0.05 | . | . | . |
| ABCC4 ---- tag |  | 2 | 5 | 8,33 | 6 | 18,75 | 28 | 8,21 | 38 | 12,42 | 2.20 (0.67-7.22) | 0.19 | 2.02 (1.09-3.74) | 0.03 | . | . | . |
| ABCC4 ---- tag | rs1751051 | 0 | 26 | 43,33 | 14 | 43,75 | 136 | 39,88 | 136 | 44,44 | 1.00 (.-.) | . | 1.38 (0.77-2.45) | 0.28 | 1.00 | 1.00 | 1.00 |
| ABCC4 ---- tag |  | 1 | 24 | 40,00 | 14 | 43,75 | 159 | 46,63 | 136 | 44,44 | 0.84 (0.46-1.52) | 0.56 | 1.15 (0.65-2.03) | 0.63 | . | . | . |
| ABCC4 ---- tag |  | 2 | 10 | 16,67 | 4 | 12,50 | 46 | 13,49 | 34 | 11,11 | 0.70 (0.21-2.31) | 0.56 | 0.96 (0.51-1.81) | 0.91 | . | . | . |
| ABCC4 ---- tag | rs1764416 | 0 | 53 | 88,33 | 31 | 96,88 | 292 | 85,63 | 265 | 86,60 | 1.00 (.-.) | . | 1.38 (0.89-2.15) | 0.15 | 0.93 | 1.00 | 1.00 |
| ABCC4 ---- tag |  | 1 | 7 | 11,67 | 1 | 3,13 | 49 | 14,37 | 40 | 13,07 | 0.79 (0.10-5.97) | 0.82 | 1.20 (0.67-2.13) | 0.55 | . | . | . |
| ABCC4 ---- tag |  | 2 | 0 | 0,00 | 0 | 0,00 | 0 | 0,00 | 1 | 0,33 | 0.63 (0.01-35.60) | 0.82 | 1.03 (0.42-2.57) | 0.94 | . | . | . |
| ABCC4 ---- tag | rs2274401 | 0 | 36 | 60,00 | 22 | 68,75 | 198 | 58,06 | 203 | 66,34 | 1.00 (.-.) | . | 1.28 (0.80-2.04) | 0.30 | 0.47 | 0.99 | 1.00 |
| ABCC4 ---- tag |  | 1 | 22 | 36,67 | 10 | 31,25 | 124 | 36,36 | 91 | 29,74 | 0.64 (0.28-1.46) | 0.29 | 1.10 (0.68-1.80) | 0.69 | . | . | . |
| ABCC4 ---- tag |  | 2 | 2 | 3,33 | 0 | 0,00 | 19 | 5,57 | 12 | 3,92 | 0.40 (0.08-2.14) | 0.29 | 0.95 (0.52-1.76) | 0.88 | . | . | . |
| ABCC4 ---- tag | rs2892716 | 0 | 28 | 46,67 | 15 | 46,88 | 117 | 34,31 | 114 | 37,25 | 1.00 (.-.) | . | 1.75 (0.99-3.10) | 0.05 | 0.15 | 0.99 | 1.00 |
| ABCC4 ---- tag |  | 1 | 29 | 48,33 | 16 | 50,00 | 164 | 48,09 | 152 | 49,67 | 1.66 (0.86-3.21) | 0.13 | 1.72 (0.99-2.98) | 0.06 | . | . | . |
| ABCC4 ---- tag |  | 2 | 3 | 5,00 | 1 | 3,13 | 60 | 17,60 | 40 | 13,07 | 2.75 (0.73-10.33) | 0.13 | 1.68 (0.92-3.06) | 0.09 | . | . | . |
| ABCC4 ---- tag | rs3782964 | 0 | 48 | 80,00 | 19 | 59,38 | 215 | 63,05 | 218 | 71,24 | 1.00 (.-.) | . | 1.84 (1.07-3.18) | 0.03 | 0.04 | 0.99 | 0.76 |
| ABCC4 ---- tag |  | 1 | 12 | 20,00 | 10 | 31,25 | 115 | 33,72 | 79 | 25,82 | 1.92 (0.89-4.14) | 0.10 | 1.49 (0.84-2.65) | 0.17 | . | . | . |
| ABCC4 ---- tag |  | 2 | 0 | 0,00 | 3 | 9,38 | 11 | 3,23 | 9 | 2,94 | 3.69 (0.80-17.11) | 0.10 | 1.21 (0.59-2.46) | 0.60 | . | . | . |
| ABCC4 ---- tag | rs3818494 | 0 | 30 | 50,00 | 21 | 65,63 | 158 | 46,33 | 123 | 40,20 | 1.00 (.-.) | . | 1.08 (0.63-1.83) | 0.78 | 0.29 | 0.99 | 1.00 |
| ABCC4 ---- tag |  | 1 | 24 | 40,00 | 10 | 31,25 | 146 | 42,82 | 140 | 45,75 | 0.90 (0.43-1.91) | 0.79 | 1.46 (0.87-2.45) | 0.15 | . | . | . |
| ABCC4 ---- tag |  | 2 | 6 | 10,00 | 1 | 3,13 | 37 | 10,85 | 43 | 14,05 | 0.82 (0.18-3.64) | 0.79 | 1.98 (1.13-3.48) | 0.02 | . | . | . |
| ABCC4 ---- tag | rs3864997 | 0 | 22 | 36,67 | 10 | 31,25 | 79 | 23,17 | 76 | 24,84 | 1.00 (.-.) | . | 2.22 (1.10-4.46) | 0.03 | 0.07 | 0.99 | 0.76 |
| ABCC4 ---- tag |  | 1 | 30 | 50,00 | 17 | 53,13 | 182 | 53,37 | 161 | 52,61 | 1.64 (0.88-3.05) | 0.12 | 1.98 (1.01-3.88) | 0.05 | . | . | . |
| ABCC4 ---- tag |  | 2 | 8 | 13,33 | 5 | 15,63 | 80 | 23,46 | 69 | 22,55 | 2.68 (0.77-9.28) | 0.12 | 1.76 (0.88-3.55) | 0.11 | . | . | . |
| ABCC4 ---- tag | rs4148421 | 0 | 15 | 25,00 | 4 | 12,50 | 105 | 30,79 | 91 | 29,74 | 1.00 (.-.) | . | 1.63 (0.70-3.78) | 0.26 | 0.62 | 0.99 | 1.00 |
| ABCC4 ---- tag |  | 1 | 33 | 55,00 | 21 | 65,63 | 170 | 49,85 | 161 | 52,61 | 1.10 (0.60-2.05) | 0.75 | 1.53 (0.67-3.49) | 0.32 | . | . | . |
| ABCC4 ---- tag |  | 2 | 12 | 20,00 | 7 | 21,88 | 66 | 19,35 | 54 | 17,65 | 1.22 (0.36-4.18) | 0.75 | 1.43 (0.61-3.37) | 0.41 | . | . | . |
| ABCC4 ---- tag | rs4148446 | 0 | 26 | 43,33 | 12 | 37,50 | 106 | 31,09 | 102 | 33,33 | 1.00 (.-.) | . | 1.82 (0.95-3.50) | 0.07 | 0.23 | 0.99 | 1.00 |
| ABCC4 ---- tag |  | 1 | 29 | 48,33 | 17 | 53,13 | 163 | 47,80 | 153 | 50,00 | 1.47 (0.74-2.91) | 0.27 | 1.73 (0.92-3.25) | 0.09 | . | . | . |
| ABCC4 ---- tag |  | 2 | 5 | 8,33 | 3 | 9,38 | 72 | 21,11 | 51 | 16,67 | 2.16 (0.55-8.45) | 0.27 | 1.64 (0.84-3.19) | 0.15 | . | . | . |
| ABCC4 ---- tag | rs4148455 | 0 | 43 | 71,67 | 27 | 84,38 | 261 | 76,54 | 226 | 73,86 | 1.00 (.-.) | . | 1.29 (0.79-2.09) | 0.31 | 0.51 | 0.99 | 1.00 |
| ABCC4 ---- tag |  | 1 | 16 | 26,67 | 4 | 12,50 | 72 | 21,11 | 77 | 25,16 | 1.00 (0.42-2.38) | 1.00 | 1.73 (1.03-2.90) | 0.04 | . | . | . |
| ABCC4 ---- tag |  | 2 | 1 | 1,67 | 1 | 3,13 | 8 | 2,35 | 3 | 0,98 | 1.00 (0.18-5.69) | 1.00 | 2.31 (1.19-4.47) | 0.01 | . | . | . |
| ABCC4 ---- tag | rs4148540 | 0 | 52 | 86,67 | 28 | 87,50 | 299 | 87,68 | 262 | 85,62 | 1.00 (.-.) | . | 1.42 (0.90-2.25) | 0.13 | 0.77 | 0.99 | 1.00 |
| ABCC4 ---- tag |  | 1 | 8 | 13,33 | 4 | 12,50 | 40 | 11,73 | 41 | 13,40 | 1.04 (0.30-3.56) | 0.95 | 1.22 (0.72-2.06) | 0.46 | . | . | . |
| ABCC4 ---- tag |  | 2 | 0 | 0,00 | 0 | 0,00 | 2 | 0,59 | 3 | 0,98 | 1.08 (0.09-12.67) | 0.95 | 1.05 (0.50-2.21) | 0.91 | . | . | . |
| ABCC4 ---- tag | rs4148542 | 0 | 11 | 18,33 | 4 | 12,50 | 94 | 27,57 | 79 | 25,82 | 1.00 (.-.) | . | 1.40 (0.54-3.63) | 0.49 | 0.98 | 1.00 | 1.00 |
| ABCC4 ---- tag |  | 1 | 30 | 50,00 | 21 | 65,63 | 179 | 52,49 | 160 | 52,29 | 1.06 (0.52-2.17) | 0.86 | 1.48 (0.58-3.77) | 0.41 | . | . | . |
| ABCC4 ---- tag |  | 2 | 19 | 31,67 | 7 | 21,88 | 68 | 19,94 | 67 | 21,90 | 1.13 (0.27-4.70) | 0.86 | 1.56 (0.60-4.06) | 0.36 | . | . | . |
| ABCC4 ---- tag | rs4148544 | 0 | 21 | 35,00 | 11 | 34,38 | 134 | 39,30 | 142 | 46,41 | 1.00 (.-.) | . | 1.44 (0.74-2.80) | 0.29 | 0.82 | 0.99 | 1.00 |
| ABCC4 ---- tag |  | 1 | 28 | 46,67 | 18 | 56,25 | 176 | 51,61 | 123 | 40,20 | 0.97 (0.51-1.83) | 0.93 | 1.29 (0.67-2.50) | 0.45 | . | . | . |
| ABCC4 ---- tag |  | 2 | 11 | 18,33 | 3 | 9,38 | 31 | 9,09 | 41 | 13,40 | 0.94 (0.26-3.35) | 0.93 | 1.16 (0.57-2.36) | 0.69 | . | . | . |
| ABCC4 ---- tag | rs4283094 | 0 | 10 | 16,67 | 5 | 15,63 | 93 | 27,27 | 70 | 22,88 | 1.00 (.-.) | . | 1.01 (0.43-2.36) | 0.99 | 0.42 | 0.99 | 1.00 |
| ABCC4 ---- tag |  | 1 | 32 | 53,33 | 17 | 53,13 | 174 | 51,03 | 167 | 54,58 | 0.80 (0.45-1.44) | 0.46 | 1.04 (0.45-2.39) | 0.92 | . | . | . |
| ABCC4 ---- tag |  | 2 | 18 | 30,00 | 10 | 31,25 | 74 | 21,70 | 69 | 22,55 | 0.64 (0.20-2.06) | 0.46 | 1.08 (0.46-2.52) | 0.86 | . | . | . |
| ABCC4 ---- tag | rs4636781 | 0 | 45 | 75,00 | 27 | 84,38 | 244 | 71,55 | 212 | 69,28 | 1.00 (.-.) | . | 1.27 (0.78-2.06) | 0.33 | 0.55 | 0.99 | 1.00 |
| ABCC4 ---- tag |  | 1 | 15 | 25,00 | 4 | 12,50 | 87 | 25,51 | 85 | 27,78 | 1.12 (0.48-2.63) | 0.80 | 1.84 (1.11-3.06) | 0.02 | . | . | . |
| ABCC4 ---- tag |  | 2 | 0 | 0,00 | 1 | 3,13 | 10 | 2,93 | 9 | 2,94 | 1.25 (0.23-6.91) | 0.80 | 2.67 (1.42-5.04) | 0.00 | . | . | . |
| ABCC4 ---- tag | rs4771910 | 0 | 32 | 53,33 | 14 | 43,75 | 161 | 47,21 | 146 | 47,71 | 1.00 (.-.) | . | 1.65 (0.89-3.04) | 0.11 | 0.42 | 0.99 | 1.00 |
| ABCC4 ---- tag |  | 1 | 24 | 40,00 | 16 | 50,00 | 148 | 43,40 | 133 | 43,46 | 1.16 (0.60-2.25) | 0.66 | 1.44 (0.78-2.63) | 0.24 | . | . | . |
| ABCC4 ---- tag |  | 2 | 4 | 6,67 | 2 | 6,25 | 32 | 9,38 | 27 | 8,82 | 1.35 (0.36-5.07) | 0.66 | 1.25 (0.64-2.43) | 0.51 | . | . | . |
| ABCC4 ---- tag | rs4773850 | 0 | 30 | 50,00 | 14 | 43,75 | 150 | 43,99 | 157 | 51,31 | 1.00 (.-.) | . | 1.87 (1.05-3.34) | 0.03 | 0.11 | 0.99 | 0.96 |
| ABCC4 ---- tag |  | 1 | 28 | 46,67 | 13 | 40,63 | 147 | 43,11 | 119 | 38,89 | 1.35 (0.74-2.45) | 0.32 | 1.50 (0.85-2.66) | 0.17 | . | . | . |
| ABCC4 ---- tag |  | 2 | 2 | 3,33 | 5 | 15,63 | 44 | 12,90 | 30 | 9,80 | 1.82 (0.55-6.01) | 0.32 | 1.20 (0.64-2.25) | 0.57 | . | . | . |
| ABCC4 ---- tag | rs7981095 | 0 | 39 | 65,00 | 20 | 62,50 | 223 | 65,40 | 196 | 64,05 | 1.00 (.-.) | . | 1.31 (0.78-2.19) | 0.31 | 0.73 | 0.99 | 1.00 |
| ABCC4 ---- tag |  | 1 | 20 | 33,33 | 8 | 25,00 | 108 | 31,67 | 99 | 32,35 | 0.78 (0.36-1.70) | 0.54 | 1.18 (0.69-2.01) | 0.54 | . | . | . |
| ABCC4 ---- tag |  | 2 | 1 | 1,67 | 4 | 12,50 | 10 | 2,93 | 11 | 3,59 | 0.62 (0.13-2.90) | 0.54 | 1.07 (0.56-2.04) | 0.85 | . | . | . |
| ABCC4 ---- tag | rs8001444 | 0 | 21 | 35,00 | 9 | 28,13 | 118 | 34,60 | 118 | 38,56 | 1.00 (.-.) | . | 1.33 (0.64-2.80) | 0.44 | 0.94 | 1.00 | 1.00 |
| ABCC4 ---- tag |  | 1 | 33 | 55,00 | 20 | 62,50 | 163 | 47,80 | 134 | 43,79 | 0.85 (0.42-1.69) | 0.64 | 1.16 (0.56-2.40) | 0.69 | . | . | . |
| ABCC4 ---- tag |  | 2 | 6 | 10,00 | 3 | 9,38 | 60 | 17,60 | 54 | 17,65 | 0.72 (0.18-2.87) | 0.64 | 1.01 (0.47-2.16) | 0.98 | . | . | . |
| ABCC4 ---- tag | rs931111 | 0 | 36 | 60,00 | 19 | 59,38 | 238 | 69,79 | 198 | 64,71 | 1.00 (.-.) | . | 1.42 (0.84-2.42) | 0.19 | 0.82 | 0.99 | 1.00 |
| ABCC4 ---- tag |  | 1 | 20 | 33,33 | 11 | 34,38 | 90 | 26,39 | 94 | 30,72 | 1.11 (0.62-1.99) | 0.72 | 1.47 (0.86-2.51) | 0.16 | . | . | . |
| ABCC4 ---- tag |  | 2 | 4 | 6,67 | 2 | 6,25 | 13 | 3,81 | 14 | 4,58 | 1.23 (0.38-3.97) | 0.72 | 1.52 (0.81-2.84) | 0.20 | . | . | . |
| ABCC4 ---- tag | rs943288 | 0 | 47 | 78,33 | 28 | 87,50 | 259 | 75,95 | 228 | 74,51 | 1.00 (.-.) | . | 1.24 (0.77-2.00) | 0.37 | 0.36 | 0.99 | 1.00 |
| ABCC4 ---- tag |  | 1 | 13 | 21,67 | 3 | 9,38 | 75 | 21,99 | 71 | 23,20 | 0.97 (0.39-2.43) | 0.95 | 1.84 (1.11-3.05) | 0.02 | . | . | . |
| ABCC4 ---- tag |  | 2 | 0 | 0,00 | 1 | 3,13 | 7 | 2,05 | 7 | 2,29 | 0.94 (0.15-5.90) | 0.95 | 2.73 (1.43-5.21) | 0.00 | . | . | . |
| ABCC4 ---- tag | rs943290 | 0 | 33 | 55,00 | 19 | 59,38 | 165 | 48,39 | 170 | 55,56 | 1.00 (.-.) | . | 1.43 (0.87-2.37) | 0.16 | 0.94 | 1.00 | 1.00 |
| ABCC4 ---- tag |  | 1 | 21 | 35,00 | 11 | 34,38 | 148 | 43,40 | 116 | 37,91 | 0.81 (0.41-1.59) | 0.54 | 1.13 (0.68-1.87) | 0.64 | . | . | . |
| ABCC4 ---- tag |  | 2 | 6 | 10,00 | 2 | 6,25 | 28 | 8,21 | 20 | 6,54 | 0.66 (0.17-2.52) | 0.54 | 0.89 (0.49-1.61) | 0.70 | . | . | . |
| ABCC4 ---- tag | rs9516530 | 0 | 32 | 53,33 | 22 | 68,75 | 193 | 56,60 | 168 | 54,90 | 1.00 (.-.) | . | 1.02 (0.61-1.71) | 0.94 | 0.07 | 0.99 | 0.76 |
| ABCC4 ---- tag |  | 1 | 26 | 43,33 | 8 | 25,00 | 116 | 34,02 | 112 | 36,60 | 0.58 (0.27-1.25) | 0.16 | 1.17 (0.70-1.95) | 0.55 | . | . | . |
| ABCC4 ---- tag |  | 2 | 2 | 3,33 | 2 | 6,25 | 32 | 9,38 | 26 | 8,50 | 0.34 (0.07-1.55) | 0.16 | 1.34 (0.75-2.39) | 0.33 | . | . | . |
| ABCC4 ---- tag | rs9516551 | 0 | 45 | 75,00 | 21 | 65,63 | 260 | 76,25 | 241 | 78,76 | 1.00 (.-.) | . | 1.56 (0.91-2.67) | 0.10 | 0.36 | 0.99 | 1.00 |
| ABCC4 ---- tag |  | 1 | 14 | 23,33 | 11 | 34,38 | 77 | 22,58 | 62 | 20,26 | 1.17 (0.54-2.50) | 0.69 | 1.23 (0.70-2.19) | 0.47 | . | . | . |
| ABCC4 ---- tag |  | 2 | 1 | 1,67 | 0 | 0,00 | 4 | 1,17 | 3 | 0,98 | 1.36 (0.30-6.27) | 0.69 | 0.98 (0.47-2.02) | 0.95 | . | . | . |
| ABCC4 ---- tag | rs9524822 | 0 | 48 | 80,00 | 18 | 56,25 | 213 | 62,46 | 200 | 65,36 | 1.00 (.-.) | . | 2.08 (1.16-3.72) | 0.01 | 0.01 | 0.99 | 0.75 |
| ABCC4 ---- tag |  | 1 | 12 | 20,00 | 10 | 31,25 | 112 | 32,84 | 94 | 30,72 | 2.03 (1.05-3.92) | 0.04 | 1.70 (0.94-3.09) | 0.08 | . | . | . |
| ABCC4 ---- tag |  | 2 | 0 | 0,00 | 4 | 12,50 | 16 | 4,69 | 12 | 3,92 | 4.12 (1.11-15.34) | 0.04 | 1.40 (0.69-2.81) | 0.35 | . | . | . |
| ABCC4 ---- tag | rs9524861 | 0 | 25 | 41,67 | 13 | 40,63 | 177 | 51,91 | 162 | 52,94 | 1.00 (.-.) | . | 1.13 (0.61-2.09) | 0.69 | 0.52 | 0.99 | 1.00 |
| ABCC4 ---- tag |  | 1 | 26 | 43,33 | 15 | 46,88 | 140 | 41,06 | 121 | 39,54 | 0.72 (0.41-1.26) | 0.25 | 0.99 (0.54-1.82) | 0.98 | . | . | . |
| ABCC4 ---- tag |  | 2 | 9 | 15,00 | 4 | 12,50 | 24 | 7,04 | 23 | 7,52 | 0.52 (0.17-1.59) | 0.25 | 0.87 (0.45-1.69) | 0.68 | . | . | . |
| ABCC4 ---- tag | rs9524902 | 0 | 17 | 28,33 | 6 | 18,75 | 88 | 25,81 | 103 | 33,66 | 1.00 (.-.) | . | 1.79 (0.76-4.18) | 0.18 | 0.43 | 0.99 | 1.00 |
| ABCC4 ---- tag |  | 1 | 32 | 53,33 | 15 | 46,88 | 177 | 51,91 | 141 | 46,08 | 1.13 (0.60-2.12) | 0.70 | 1.56 (0.68-3.58) | 0.30 | . | . | . |
| ABCC4 ---- tag |  | 2 | 11 | 18,33 | 11 | 34,38 | 76 | 22,29 | 62 | 20,26 | 1.28 (0.37-4.47) | 0.70 | 1.35 (0.57-3.19) | 0.49 | . | . | . |
| ABCC4 ---- tag | rs9556455 | 0 | 47 | 78,33 | 21 | 65,63 | 262 | 76,83 | 232 | 75,82 | 1.00 (.-.) | . | 1.54 (0.92-2.59) | 0.10 | 0.38 | 0.99 | 1.00 |
| ABCC4 ---- tag |  | 1 | 12 | 20,00 | 10 | 31,25 | 76 | 22,29 | 68 | 22,22 | 1.32 (0.68-2.60) | 0.41 | 1.46 (0.84-2.54) | 0.18 | . | . | . |
| ABCC4 ---- tag |  | 2 | 1 | 1,67 | 1 | 3,13 | 3 | 0,88 | 6 | 1,96 | 1.76 (0.46-6.74) | 0.41 | 1.39 (0.69-2.77) | 0.36 | . | . | . |
| ABCC4 ---- NA | rs9561778 | 0 | 37 | 61,67 | 23 | 71,88 | 214 | 62,76 | 211 | 68,95 | 1.00 (.-.) | . | 1.27 (0.80-2.02) | 0.31 | 0.41 | 0.99 | 1.00 |
| ABCC4 ---- NA |  | 1 | 21 | 35,00 | 9 | 28,13 | 112 | 32,84 | 86 | 28,10 | 0.59 (0.24-1.47) | 0.26 | 1.09 (0.67-1.79) | 0.72 | . | . | . |
| ABCC4 ---- NA |  | 2 | 2 | 3,33 | 0 | 0,00 | 15 | 4,40 | 9 | 2,94 | 0.35 (0.06-2.15) | 0.26 | 0.94 (0.50-1.75) | 0.84 | . | . | . |
| ABCC4 ---- tag | rs9561811 | 0 | 39 | 65,00 | 26 | 81,25 | 232 | 68,04 | 198 | 64,71 | 1.00 (.-.) | . | 1.35 (0.82-2.22) | 0.23 | 0.91 | 1.00 | 1.00 |
| ABCC4 ---- tag |  | 1 | 20 | 33,33 | 6 | 18,75 | 97 | 28,45 | 92 | 30,07 | 1.25 (0.49-3.17) | 0.64 | 1.60 (0.97-2.64) | 0.07 | . | . | . |
| ABCC4 ---- tag |  | 2 | 1 | 1,67 | 0 | 0,00 | 12 | 3,52 | 16 | 5,23 | 1.56 (0.24-10.08) | 0.64 | 1.89 (1.04-3.43) | 0.04 | . | . | . |
| ABCC4 ---- tag | rs9590183 | 0 | 54 | 90,00 | 30 | 93,75 | 293 | 85,92 | 264 | 86,27 | 1.00 (.-.) | . | 1.35 (0.86-2.10) | 0.19 | 0.67 | 0.99 | 1.00 |
| ABCC4 ---- tag |  | 1 | 6 | 10,00 | 2 | 6,25 | 46 | 13,49 | 42 | 13,73 | 0.54 (0.12-2.34) | 0.41 | 1.00 (0.57-1.76) | 1.00 | . | . | . |
| ABCC4 ---- tag |  | 2 | 0 | 0,00 | 0 | 0,00 | 2 | 0,59 | 0 | 0,00 | 0.29 (0.02-5.50) | 0.41 | 0.74 (0.31-1.78) | 0.50 | . | . | . |
| ABCC4 ---- tag | rs997777 | 0 | 27 | 45,00 | 15 | 46,88 | 166 | 48,68 | 153 | 50,00 | 1.00 (.-.) | . | 1.50 (0.86-2.61) | 0.15 | 0.62 | 0.99 | 1.00 |
| ABCC4 ---- tag |  | 1 | 24 | 40,00 | 14 | 43,75 | 149 | 43,70 | 128 | 41,83 | 1.15 (0.62-2.11) | 0.66 | 1.46 (0.84-2.53) | 0.18 | . | . | . |
| ABCC4 ---- tag |  | 2 | 9 | 15,00 | 3 | 9,38 | 26 | 7,62 | 25 | 8,17 | 1.32 (0.39-4.44) | 0.66 | 1.42 (0.77-2.62) | 0.27 | . | . | . |
| ADH1B ---- tag | rs1159918 | 0 | 20 | 33,33 | 13 | 40,63 | 154 | 45,16 | 148 | 48,37 | 1.00 (.-.) | . | 0.85 (0.46-1.56) | 0.59 | 0.06 | 0.99 | 0.11 |
| ADH1B ---- tag |  | 1 | 32 | 53,33 | 15 | 46,88 | 150 | 43,99 | 134 | 43,79 | 0.55 (0.29-1.02) | 0.06 | 0.87 (0.48-1.59) | 0.65 | . | . | . |
| ADH1B ---- tag |  | 2 | 8 | 13,33 | 4 | 12,50 | 37 | 10,85 | 24 | 7,84 | 0.30 (0.09-1.05) | 0.06 | 0.89 (0.46-1.74) | 0.74 | . | . | . |
| ADH1B ---- candidate literature | rs1229984 | 0 | 52 | 86,67 | 31 | 96,88 | 308 | 90,32 | 277 | 90,52 | 1.00 (.-.) | . | 1.21 (0.78-1.87) | 0.40 | 0.07 | 0.99 | 0.11 |
| ADH1B ---- candidate literature |  | 1 | 7 | 11,67 | 1 | 3,13 | 31 | 9,09 | 29 | 9,48 | 0.23 (0.03-1.65) | 0.14 | 1.23 (0.68-2.22) | 0.49 | . | . | . |
| ADH1B ---- candidate literature |  | 2 | 1 | 1,67 | 0 | 0,00 | 2 | 0,59 | 0 | 0,00 | 0.05 (0.00-2.71) | 0.14 | 1.26 (0.49-3.22) | 0.64 | . | . | . |
| ADH1B ---- tag | rs12507573 | 0 | 19 | 31,67 | 12 | 37,50 | 103 | 30,21 | 85 | 27,78 | 1.00 (.-.) | . | 1.08 (0.56-2.07) | 0.83 | 0.34 | 0.99 | 0.43 |
| ADH1B ---- tag |  | 1 | 26 | 43,33 | 19 | 59,38 | 164 | 48,09 | 146 | 47,71 | 0.72 (0.37-1.39) | 0.33 | 1.08 (0.58-2.00) | 0.81 | . | . | . |
| ADH1B ---- tag |  | 2 | 15 | 25,00 | 1 | 3,13 | 74 | 21,70 | 75 | 24,51 | 0.52 (0.14-1.92) | 0.33 | 1.08 (0.57-2.06) | 0.81 | . | . | . |
| ADH1B ---- tag | rs1693457 | 0 | 40 | 66,67 | 19 | 59,38 | 232 | 68,04 | 224 | 73,20 | 1.00 (.-.) | . | 1.22 (0.71-2.09) | 0.46 | 0.58 | 0.99 | 0.58 |
| ADH1B ---- tag |  | 1 | 19 | 31,67 | 11 | 34,38 | 97 | 28,45 | 74 | 24,18 | 0.71 (0.33-1.56) | 0.40 | 1.10 (0.63-1.92) | 0.74 | . | . | . |
| ADH1B ---- tag |  | 2 | 1 | 1,67 | 2 | 6,25 | 12 | 3,52 | 8 | 2,61 | 0.51 (0.11-2.43) | 0.40 | 0.99 (0.50-1.97) | 0.97 | . | . | . |
| ADH1B ---- tag | rs2066701 | 0 | 30 | 50,00 | 9 | 28,13 | 159 | 46,63 | 142 | 46,41 | 1.00 (.-.) | . | 2.56 (1.19-5.50) | 0.02 | 0.03 | 0.99 | 0.11 |
| ADH1B ---- tag |  | 1 | 28 | 46,67 | 21 | 65,63 | 147 | 43,11 | 131 | 42,81 | 2.44 (1.16-5.12) | 0.02 | 2.62 (1.23-5.60) | 0.01 | . | . | . |
| ADH1B ---- tag |  | 2 | 2 | 3,33 | 2 | 6,25 | 35 | 10,26 | 33 | 10,78 | 5.96 (1.35-26.25) | 0.02 | 2.69 (1.20-6.02) | 0.02 | . | . | . |
| ADH1C ---- tag | rs11936869 | 0 | 30 | 50,00 | 12 | 37,50 | 176 | 51,61 | 170 | 55,56 | 1.00 (.-.) | . | 1.58 (0.86-2.91) | 0.14 | 0.50 | 0.99 | 0.64 |
| ADH1C ---- tag |  | 1 | 25 | 41,67 | 15 | 46,88 | 135 | 39,59 | 110 | 35,95 | 1.25 (0.67-2.32) | 0.48 | 1.58 (0.86-2.88) | 0.14 | . | . | . |
| ADH1C ---- tag |  | 2 | 5 | 8,33 | 5 | 15,63 | 30 | 8,80 | 26 | 8,50 | 1.56 (0.45-5.37) | 0.48 | 1.57 (0.81-3.05) | 0.19 | . | . | . |
| ADH1C ---- tag | rs1229849 | 0 | 34 | 56,67 | 19 | 59,38 | 190 | 55,72 | 144 | 47,06 | 1.00 (.-.) | . | 1.20 (0.70-2.07) | 0.51 | 0.47 | 0.99 | 0.64 |
| ADH1C ---- tag |  | 1 | 24 | 40,00 | 11 | 34,38 | 135 | 39,59 | 139 | 45,42 | 0.98 (0.52-1.82) | 0.94 | 1.49 (0.87-2.55) | 0.14 | . | . | . |
| ADH1C ---- tag |  | 2 | 2 | 3,33 | 2 | 6,25 | 16 | 4,69 | 23 | 7,52 | 0.96 (0.28-3.32) | 0.94 | 1.85 (1.01-3.40) | 0.05 | . | . | . |
| ADH1C ---- tag | rs1229863 | 0 | 44 | 73,33 | 22 | 68,75 | 247 | 72,43 | 226 | 73,86 | 1.00 (.-.) | . | 1.51 (0.93-2.46) | 0.10 | 0.35 | 0.99 | 0.64 |
| ADH1C ---- tag |  | 1 | 16 | 26,67 | 7 | 21,88 | 88 | 25,81 | 70 | 22,88 | 1.37 (0.74-2.52) | 0.32 | 1.49 (0.89-2.49) | 0.13 | . | . | . |
| ADH1C ---- tag |  | 2 | 0 | 0,00 | 3 | 9,38 | 6 | 1,76 | 10 | 3,27 | 1.86 (0.55-6.36) | 0.32 | 1.47 (0.76-2.84) | 0.26 | . | . | . |
| ADH1C ---- tag | rs1229980 | 0 | 53 | 88,33 | 29 | 90,63 | 312 | 91,50 | 271 | 88,56 | 1.00 (.-.) | . | 1.39 (0.89-2.18) | 0.15 | 0.46 | 0.99 | 0.64 |
| ADH1C ---- tag |  | 1 | 7 | 11,67 | 3 | 9,38 | 29 | 8,50 | 33 | 10,78 | 2.52 (0.58-10.98) | 0.22 | 1.90 (1.10-3.30) | 0.02 | . | . | . |
| ADH1C ---- tag |  | 2 | 0 | 0,00 | 0 | 0,00 | 0 | 0,00 | 2 | 0,65 | 6.34 (0.33-120.7) | 0.22 | 2.60 (1.14-5.96) | 0.02 | . | . | . |
| ADH1C ---- candidate | rs1693482 | 0 | 29 | 48,33 | 13 | 40,63 | 146 | 42,82 | 117 | 38,24 | 1.00 (.-.) | . | 1.40 (0.77-2.57) | 0.27 | 0.91 | 1.00 | 0.92 |
| ADH1C ---- candidate |  | 1 | 26 | 43,33 | 13 | 40,63 | 152 | 44,57 | 141 | 46,08 | 1.12 (0.67-1.89) | 0.66 | 1.53 (0.85-2.75) | 0.16 | . | . | . |
| ADH1C ---- candidate |  | 2 | 5 | 8,33 | 6 | 18,75 | 43 | 12,61 | 48 | 15,69 | 1.26 (0.45-3.56) | 0.66 | 1.66 (0.88-3.13) | 0.12 | . | . | . |
| ADH1C ---- tag | rs2173201 | 0 | 34 | 56,67 | 13 | 40,63 | 196 | 57,48 | 184 | 60,13 | 1.00 (.-.) | . | 1.81 (1.03-3.17) | 0.04 | 0.07 | 0.99 | 0.64 |
| ADH1C ---- tag |  | 1 | 22 | 36,67 | 17 | 53,13 | 124 | 36,36 | 102 | 33,33 | 1.93 (1.06-3.54) | 0.03 | 1.91 (1.09-3.36) | 0.03 | . | . | . |
| ADH1C ---- tag |  | 2 | 4 | 6,67 | 2 | 6,25 | 21 | 6,16 | 20 | 6,54 | 3.73 (1.11-12.50) | 0.03 | 2.02 (1.06-3.83) | 0.03 | . | . | . |
| ADH1C ---- tag | rs2298753 | 0 | 51 | 85,00 | 25 | 78,13 | 272 | 79,77 | 257 | 83,99 | 1.00 (.-.) | . | 1.51 (0.94-2.41) | 0.09 | 0.26 | 0.99 | 0.64 |
| ADH1C ---- tag |  | 1 | 9 | 15,00 | 4 | 12,50 | 67 | 19,65 | 45 | 14,71 | 1.22 (0.63-2.35) | 0.56 | 1.17 (0.67-2.03) | 0.58 | . | . | . |
| ADH1C ---- tag |  | 2 | 0 | 0,00 | 3 | 9,38 | 2 | 0,59 | 4 | 1,31 | 1.48 (0.40-5.52) | 0.56 | 0.91 (0.40-2.03) | 0.81 | . | . | . |
| ADH1C ---- tag | rs2866152 | 0 | 40 | 66,67 | 21 | 65,63 | 213 | 62,46 | 172 | 56,21 | 1.00 (.-.) | . | 1.21 (0.72-2.04) | 0.46 | 0.41 | 0.99 | 0.64 |
| ADH1C ---- tag |  | 1 | 19 | 31,67 | 10 | 31,25 | 115 | 33,72 | 118 | 38,56 | 0.86 (0.45-1.67) | 0.66 | 1.39 (0.83-2.35) | 0.21 | . | . | . |
| ADH1C ---- tag |  | 2 | 1 | 1,67 | 1 | 3,13 | 13 | 3,81 | 16 | 5,23 | 0.75 (0.20-2.78) | 0.66 | 1.60 (0.87-2.95) | 0.13 | . | . | . |
| ADH1C ---- tag | rs904096 | 0 | 29 | 48,33 | 13 | 40,63 | 144 | 42,23 | 115 | 37,58 | 1.00 (.-.) | . | 1.40 (0.77-2.56) | 0.27 | 0.92 | 1.00 | 0.92 |
| ADH1C ---- tag |  | 1 | 26 | 43,33 | 13 | 40,63 | 154 | 45,16 | 143 | 46,73 | 1.12 (0.67-1.89) | 0.66 | 1.53 (0.85-2.75) | 0.16 | . | . | . |
| ADH1C ---- tag |  | 2 | 5 | 8,33 | 6 | 18,75 | 43 | 12,61 | 48 | 15,69 | 1.26 (0.45-3.56) | 0.66 | 1.67 (0.88-3.14) | 0.11 | . | . | . |
| BHMT ---- tag | rs10944 | 0 | 16 | 26,67 | 7 | 21,88 | 72 | 21,11 | 78 | 25,49 | 1.00 (.-.) | . | 1.18 (0.56-2.49) | 0.67 | 0.63 | 0.99 | 0.74 |
| BHMT ---- tag |  | 1 | 27 | 45,00 | 18 | 56,25 | 187 | 54,84 | 153 | 50,00 | 0.87 (0.50-1.54) | 0.64 | 1.19 (0.58-2.44) | 0.63 | . | . | . |
| BHMT ---- tag |  | 2 | 17 | 28,33 | 7 | 21,88 | 82 | 24,05 | 75 | 24,51 | 0.76 (0.25-2.37) | 0.64 | 1.20 (0.57-2.52) | 0.62 | . | . | . |
| BHMT ---- tag | rs12655567 | 0 | 24 | 40,00 | 12 | 37,50 | 115 | 33,72 | 122 | 39,87 | 1.00 (.-.) | . | 1.79 (0.94-3.42) | 0.08 | 0.28 | 0.99 | 0.74 |
| BHMT ---- tag |  | 1 | 30 | 50,00 | 15 | 46,88 | 176 | 51,61 | 144 | 47,06 | 1.14 (0.64-2.02) | 0.66 | 1.46 (0.78-2.72) | 0.24 | . | . | . |
| BHMT ---- tag |  | 2 | 6 | 10,00 | 5 | 15,63 | 50 | 14,66 | 40 | 13,07 | 1.30 (0.41-4.09) | 0.66 | 1.18 (0.60-2.32) | 0.63 | . | . | . |
| BHMT ---- tag | rs1291041 | 0 | 28 | 46,67 | 14 | 43,75 | 131 | 38,42 | 143 | 46,73 | 1.00 (.-.) | . | 1.60 (0.87-2.96) | 0.13 | 0.50 | 0.99 | 0.74 |
| BHMT ---- tag |  | 1 | 27 | 45,00 | 14 | 43,75 | 168 | 49,27 | 128 | 41,83 | 1.05 (0.59-1.87) | 0.86 | 1.36 (0.75-2.47) | 0.31 | . | . | . |
| BHMT ---- tag |  | 2 | 5 | 8,33 | 4 | 12,50 | 42 | 12,32 | 35 | 11,44 | 1.11 (0.35-3.51) | 0.86 | 1.15 (0.60-2.22) | 0.67 | . | . | . |
| BHMT ---- tag | rs16876500 | 0 | 50 | 83,33 | 25 | 78,13 | 277 | 81,23 | 237 | 77,45 | 1.00 (.-.) | . | 1.27 (0.80-2.03) | 0.31 | 0.35 | 0.99 | 0.74 |
| BHMT ---- tag |  | 1 | 9 | 15,00 | 6 | 18,75 | 61 | 17,89 | 64 | 20,92 | 0.85 (0.40-1.79) | 0.66 | 1.56 (0.93-2.63) | 0.09 | . | . | . |
| BHMT ---- tag |  | 2 | 1 | 1,67 | 1 | 3,13 | 3 | 0,88 | 5 | 1,63 | 0.71 (0.16-3.20) | 0.66 | 1.92 (0.94-3.90) | 0.07 | . | . | . |
| BHMT ---- tag | rs492842 | 0 | 25 | 41,67 | 12 | 37,50 | 129 | 37,83 | 122 | 39,87 | 1.00 (.-.) | . | 1.64 (0.89-3.03) | 0.12 | 0.43 | 0.99 | 0.74 |
| BHMT ---- tag |  | 1 | 23 | 38,33 | 18 | 56,25 | 168 | 49,27 | 141 | 46,08 | 1.19 (0.67-2.10) | 0.55 | 1.52 (0.84-2.77) | 0.17 | . | . | . |
| BHMT ---- tag |  | 2 | 12 | 20,00 | 2 | 6,25 | 44 | 12,90 | 43 | 14,05 | 1.42 (0.45-4.42) | 0.55 | 1.41 (0.74-2.69) | 0.29 | . | . | . |
| BHMT ---- tag | rs558133 | 0 | 27 | 45,00 | 16 | 50,00 | 163 | 47,80 | 146 | 47,71 | 1.00 (.-.) | . | 1.36 (0.78-2.38) | 0.28 | 0.96 | 1.00 | 0.96 |
| BHMT ---- tag |  | 1 | 19 | 31,67 | 13 | 40,63 | 142 | 41,64 | 129 | 42,16 | 0.98 (0.56-1.70) | 0.94 | 1.35 (0.78-2.34) | 0.28 | . | . | . |
| BHMT ---- tag |  | 2 | 14 | 23,33 | 3 | 9,38 | 36 | 10,56 | 31 | 10,13 | 0.96 (0.32-2.90) | 0.94 | 1.34 (0.73-2.47) | 0.35 | . | . | . |
| BHMT ---- tag | rs9637824 | 0 | 23 | 38,33 | 11 | 34,38 | 130 | 38,12 | 117 | 38,24 | 1.00 (.-.) | . | 1.54 (0.82-2.92) | 0.18 | 0.62 | 0.99 | 0.74 |
| BHMT ---- tag |  | 1 | 26 | 43,33 | 19 | 59,38 | 168 | 49,27 | 145 | 47,39 | 1.16 (0.63-2.15) | 0.63 | 1.52 (0.82-2.84) | 0.19 | . | . | . |
| BHMT ---- tag |  | 2 | 11 | 18,33 | 2 | 6,25 | 43 | 12,61 | 44 | 14,38 | 1.36 (0.40-4.60) | 0.63 | 1.50 (0.77-2.93) | 0.23 | . | . | . |
| BHMT2 ---- tag | rs16876512 | 0 | 50 | 83,33 | 25 | 78,13 | 274 | 80,35 | 237 | 77,45 | 1.00 (.-.) | . | 1.28 (0.80-2.04) | 0.30 | 0.39 | 0.99 | 0.78 |
| BHMT2 ---- tag |  | 1 | 9 | 15,00 | 6 | 18,75 | 64 | 18,77 | 63 | 20,59 | 0.85 (0.40-1.79) | 0.66 | 1.53 (0.91-2.57) | 0.11 | . | . | . |
| BHMT2 ---- tag |  | 2 | 1 | 1,67 | 1 | 3,13 | 3 | 0,88 | 6 | 1,96 | 0.72 (0.16-3.21) | 0.66 | 1.83 (0.90-3.72) | 0.10 | . | . | . |
| BHMT2 ---- tag | rs2461248 | 0 | 16 | 26,67 | 6 | 18,75 | 71 | 20,82 | 78 | 25,49 | 1.00 (.-.) | . | 1.25 (0.58-2.71) | 0.57 | 0.78 | 0.99 | 0.78 |
| BHMT2 ---- tag |  | 1 | 27 | 45,00 | 19 | 59,38 | 187 | 54,84 | 152 | 49,67 | 0.92 (0.52-1.64) | 0.78 | 1.26 (0.60-2.64) | 0.55 | . | . | . |
| BHMT2 ---- tag |  | 2 | 17 | 28,33 | 7 | 21,88 | 83 | 24,34 | 76 | 24,84 | 0.85 (0.27-2.69) | 0.78 | 1.26 (0.59-2.71) | 0.55 | . | . | . |
| BHMT2 ---- tag | rs2909856 | 0 | 26 | 43,33 | 14 | 43,75 | 140 | 41,06 | 130 | 42,48 | 1.00 (.-.) | . | 1.56 (0.87-2.83) | 0.14 | 0.53 | 0.99 | 0.78 |
| BHMT2 ---- tag |  | 1 | 24 | 40,00 | 16 | 50,00 | 161 | 47,21 | 136 | 44,44 | 1.16 (0.65-2.08) | 0.62 | 1.49 (0.84-2.65) | 0.18 | . | . | . |
| BHMT2 ---- tag |  | 2 | 10 | 16,67 | 2 | 6,25 | 40 | 11,73 | 40 | 13,07 | 1.35 (0.42-4.34) | 0.62 | 1.41 (0.76-2.64) | 0.28 | . | . | . |
| BHMT2 ---- tag | rs476620 | 0 | 23 | 38,33 | 11 | 34,38 | 130 | 38,12 | 116 | 37,91 | 1.00 (.-.) | . | 1.54 (0.81-2.91) | 0.19 | 0.63 | 0.99 | 0.78 |
| BHMT2 ---- tag |  | 1 | 26 | 43,33 | 19 | 59,38 | 168 | 49,27 | 146 | 47,71 | 1.16 (0.63-2.15) | 0.63 | 1.53 (0.82-2.85) | 0.18 | . | . | . |
| BHMT2 ---- tag |  | 2 | 11 | 18,33 | 2 | 6,25 | 43 | 12,61 | 44 | 14,38 | 1.36 (0.40-4.60) | 0.63 | 1.51 (0.78-2.95) | 0.22 | . | . | . |
| BHMT2 ---- candidate literature | rs626105 | 0 | 33 | 55,00 | 23 | 71,88 | 214 | 62,76 | 196 | 64,05 | 1.00 (.-.) | . | 1.32 (0.80-2.19) | 0.27 | 0.74 | 0.99 | 0.78 |
| BHMT2 ---- candidate literature |  | 1 | 24 | 40,00 | 9 | 28,13 | 118 | 34,60 | 101 | 33,01 | 0.74 (0.32-1.70) | 0.47 | 1.13 (0.67-1.89) | 0.65 | . | . | . |
| BHMT2 ---- candidate literature |  | 2 | 3 | 5,00 | 0 | 0,00 | 9 | 2,64 | 9 | 2,94 | 0.54 (0.10-2.90) | 0.47 | 0.96 (0.51-1.82) | 0.90 | . | . | . |
| BHMT2 ---- tag | rs631305 | 0 | 37 | 61,67 | 26 | 81,25 | 237 | 69,50 | 217 | 70,92 | 1.00 (.-.) | . | 1.29 (0.80-2.08) | 0.30 | 0.53 | 0.99 | 0.78 |
| BHMT2 ---- tag |  | 1 | 20 | 33,33 | 6 | 18,75 | 95 | 27,86 | 81 | 26,47 | 0.63 (0.25-1.62) | 0.34 | 1.10 (0.67-1.82) | 0.71 | . | . | . |
| BHMT2 ---- tag |  | 2 | 3 | 5,00 | 0 | 0,00 | 9 | 2,64 | 8 | 2,61 | 0.40 (0.06-2.61) | 0.34 | 0.94 (0.49-1.79) | 0.85 | . | . | . |
| CBS ---- tag | rs11701048 | 0 | 52 | 86,67 | 26 | 81,25 | 285 | 83,58 | 270 | 88,24 | 1.00 (.-.) | . | 1.53 (0.95-2.46) | 0.08 | 0.21 | 0.99 | 0.90 |
| CBS ---- tag |  | 1 | 8 | 13,33 | 6 | 18,75 | 55 | 16,13 | 35 | 11,44 | 1.82 (0.67-4.91) | 0.24 | 1.35 (0.75-2.41) | 0.32 | . | . | . |
| CBS ---- tag |  | 2 | 0 | 0,00 | 0 | 0,00 | 1 | 0,29 | 1 | 0,33 | 3.30 (0.45-24.12) | 0.24 | 1.18 (0.49-2.84) | 0.71 | . | . | . |
| CBS ---- tag | rs234706 | 0 | 24 | 40,00 | 12 | 37,50 | 153 | 44,87 | 121 | 39,54 | 1.00 (.-.) | . | 1.05 (0.56-1.98) | 0.87 | 0.29 | 0.99 | 0.90 |
| CBS ---- tag |  | 1 | 27 | 45,00 | 17 | 53,13 | 147 | 43,11 | 144 | 47,06 | 0.74 (0.40-1.36) | 0.33 | 1.10 (0.60-2.02) | 0.77 | . | . | . |
| CBS ---- tag |  | 2 | 9 | 15,00 | 3 | 9,38 | 41 | 12,02 | 41 | 13,40 | 0.54 (0.16-1.86) | 0.33 | 1.14 (0.60-2.18) | 0.69 | . | . | . |
| CBS ---- tag | rs234711 | 0 | 36 | 60,00 | 18 | 56,25 | 208 | 61,00 | 168 | 54,90 | 1.00 (.-.) | . | 1.25 (0.72-2.18) | 0.43 | 0.64 | 0.99 | 0.90 |
| CBS ---- tag |  | 1 | 20 | 33,33 | 13 | 40,63 | 112 | 32,84 | 119 | 38,89 | 0.90 (0.41-1.97) | 0.80 | 1.37 (0.79-2.37) | 0.26 | . | . | . |
| CBS ---- tag |  | 2 | 4 | 6,67 | 1 | 3,13 | 21 | 6,16 | 19 | 6,21 | 0.81 (0.17-3.90) | 0.80 | 1.50 (0.81-2.80) | 0.20 | . | . | . |
| CBS ---- candidate literature | rs234713 | 0 | 30 | 50,00 | 16 | 50,00 | 172 | 50,44 | 142 | 46,41 | 1.00 (.-.) | . | 1.42 (0.79-2.55) | 0.24 | 0.85 | 1.00 | 0.95 |
| CBS ---- candidate literature |  | 1 | 24 | 40,00 | 12 | 37,50 | 134 | 39,30 | 137 | 44,77 | 1.10 (0.58-2.10) | 0.78 | 1.46 (0.83-2.58) | 0.19 | . | . | . |
| CBS ---- candidate literature |  | 2 | 6 | 10,00 | 4 | 12,50 | 35 | 10,26 | 27 | 8,82 | 1.21 (0.33-4.39) | 0.78 | 1.50 (0.80-2.81) | 0.20 | . | . | . |
| CBS ---- tag | rs2839623 | 0 | 48 | 80,00 | 29 | 90,63 | 279 | 81,82 | 254 | 83,01 | 1.00 (.-.) | . | 1.37 (0.86-2.16) | 0.18 | 0.97 | 1.00 | 0.97 |
| CBS ---- tag |  | 1 | 12 | 20,00 | 3 | 9,38 | 58 | 17,01 | 51 | 16,67 | 1.01 (0.30-3.44) | 0.99 | 1.42 (0.83-2.42) | 0.20 | . | . | . |
| CBS ---- tag |  | 2 | 0 | 0,00 | 0 | 0,00 | 4 | 1,17 | 1 | 0,33 | 1.02 (0.09-11.86) | 0.99 | 1.47 (0.69-3.14) | 0.32 | . | . | . |
| CBS ---- tag | rs2839626 | 0 | 29 | 48,33 | 11 | 34,38 | 171 | 50,15 | 139 | 45,42 | 1.00 (.-.) | . | 1.53 (0.80-2.93) | 0.20 | 0.72 | 0.99 | 0.90 |
| CBS ---- tag |  | 1 | 26 | 43,33 | 16 | 50,00 | 140 | 41,06 | 136 | 44,44 | 1.26 (0.71-2.23) | 0.42 | 1.73 (0.91-3.29) | 0.10 | . | . | . |
| CBS ---- tag |  | 2 | 5 | 8,33 | 5 | 15,63 | 30 | 8,80 | 31 | 10,13 | 1.59 (0.51-4.95) | 0.42 | 1.95 (0.98-3.90) | 0.06 | . | . | . |
| CBS ---- tag | rs422791 | 0 | 28 | 46,67 | 20 | 62,50 | 177 | 51,91 | 137 | 44,77 | 1.00 (.-.) | . | 1.10 (0.66-1.86) | 0.71 | 0.19 | 0.99 | 0.90 |
| CBS ---- tag |  | 1 | 25 | 41,67 | 10 | 31,25 | 130 | 38,12 | 139 | 45,42 | 0.65 (0.32-1.31) | 0.23 | 1.15 (0.69-1.91) | 0.60 | . | . | . |
| CBS ---- tag |  | 2 | 7 | 11,67 | 2 | 6,25 | 34 | 9,97 | 30 | 9,80 | 0.42 (0.10-1.71) | 0.23 | 1.19 (0.68-2.11) | 0.54 | . | . | . |
| CBS ---- tag | rs706209 | 0 | 17 | 28,33 | 9 | 28,13 | 113 | 33,14 | 97 | 31,70 | 1.00 (.-.) | . | 1.18 (0.55-2.52) | 0.67 | 0.59 | 0.99 | 0.90 |
| CBS ---- tag |  | 1 | 32 | 53,33 | 12 | 37,50 | 171 | 50,15 | 149 | 48,69 | 0.94 (0.55-1.63) | 0.83 | 1.31 (0.63-2.74) | 0.48 | . | . | . |
| CBS ---- tag |  | 2 | 11 | 18,33 | 11 | 34,38 | 57 | 16,72 | 60 | 19,61 | 0.89 (0.30-2.64) | 0.83 | 1.45 (0.67-3.12) | 0.34 | . | . | . |
| CBS ---- tag | rs719037 | 0 | 19 | 31,67 | 14 | 43,75 | 112 | 32,84 | 92 | 30,07 | 1.00 (.-.) | . | 1.12 (0.61-2.03) | 0.72 | 0.36 | 0.99 | 0.90 |
| CBS ---- tag |  | 1 | 28 | 46,67 | 15 | 46,88 | 163 | 47,80 | 152 | 49,67 | 0.80 (0.44-1.45) | 0.46 | 1.19 (0.67-2.10) | 0.55 | . | . | . |
| CBS ---- tag |  | 2 | 13 | 21,67 | 3 | 9,38 | 66 | 19,35 | 62 | 20,26 | 0.64 (0.19-2.10) | 0.46 | 1.26 (0.69-2.32) | 0.45 | . | . | . |
| CBS ---- tag | rs719038 | 0 | 28 | 46,67 | 11 | 34,38 | 158 | 46,33 | 132 | 43,14 | 1.00 (.-.) | . | 1.53 (0.80-2.94) | 0.20 | 0.71 | 0.99 | 0.90 |
| CBS ---- tag |  | 1 | 26 | 43,33 | 15 | 46,88 | 147 | 43,11 | 136 | 44,44 | 1.24 (0.71-2.15) | 0.45 | 1.70 (0.89-3.23) | 0.11 | . | . | . |
| CBS ---- tag |  | 2 | 6 | 10,00 | 6 | 18,75 | 36 | 10,56 | 38 | 12,42 | 1.54 (0.51-4.63) | 0.45 | 1.88 (0.94-3.76) | 0.07 | . | . | . |
| DHFR ---- tag | rs10474632 | 0 | 53 | 88,33 | 28 | 87,50 | 286 | 83,87 | 252 | 82,35 | 1.00 (.-.) | . | 1.34 (0.85-2.12) | 0.21 | 0.75 | 0.99 | 0.81 |
| DHFR ---- tag |  | 1 | 7 | 11,67 | 4 | 12,50 | 51 | 14,96 | 54 | 17,65 | 0.82 (0.24-2.78) | 0.74 | 1.33 (0.79-2.25) | 0.29 | . | . | . |
| DHFR ---- tag |  | 2 | 0 | 0,00 | 0 | 0,00 | 4 | 1,17 | 0 | 0,00 | 0.66 (0.06-7.71) | 0.74 | 1.32 (0.64-2.76) | 0.45 | . | . | . |
| DHFR ---- tag | rs11951910 | 0 | 48 | 80,00 | 25 | 78,13 | 277 | 81,23 | 249 | 81,37 | 1.00 (.-.) | . | 1.24 (0.76-2.02) | 0.39 | 0.27 | 0.99 | 0.68 |
| DHFR ---- tag |  | 1 | 12 | 20,00 | 6 | 18,75 | 59 | 17,30 | 54 | 17,65 | 0.86 (0.41-1.80) | 0.69 | 1.65 (0.96-2.85) | 0.07 | . | . | . |
| DHFR ---- tag |  | 2 | 0 | 0,00 | 1 | 3,13 | 5 | 1,47 | 3 | 0,98 | 0.74 (0.17-3.26) | 0.69 | 2.21 (1.04-4.67) | 0.04 | . | . | . |
| DHFR ---- tag | rs1643665 | 0 | 33 | 55,00 | 11 | 34,38 | 152 | 44,57 | 143 | 46,73 | 1.00 (.-.) | . | 1.70 (0.90-3.24) | 0.10 | 0.33 | 0.99 | 0.68 |
| DHFR ---- tag |  | 1 | 21 | 35,00 | 15 | 46,88 | 145 | 42,52 | 143 | 46,73 | 1.20 (0.68-2.10) | 0.53 | 1.51 (0.80-2.84) | 0.21 | . | . | . |
| DHFR ---- tag |  | 2 | 6 | 10,00 | 6 | 18,75 | 44 | 12,90 | 20 | 6,54 | 1.43 (0.46-4.40) | 0.53 | 1.33 (0.67-2.66) | 0.42 | . | . | . |
| DHFR ---- tag | rs1650717 | 0 | 26 | 43,33 | 17 | 53,13 | 190 | 55,72 | 165 | 53,92 | 1.00 (.-.) | . | 1.06 (0.59-1.88) | 0.85 | 0.16 | 0.99 | 0.68 |
| DHFR ---- tag |  | 1 | 29 | 48,33 | 12 | 37,50 | 122 | 35,78 | 117 | 38,24 | 0.80 (0.46-1.40) | 0.43 | 1.30 (0.73-2.30) | 0.38 | . | . | . |
| DHFR ---- tag |  | 2 | 5 | 8,33 | 3 | 9,38 | 29 | 8,50 | 24 | 7,84 | 0.64 (0.21-1.95) | 0.43 | 1.59 (0.84-3.02) | 0.16 | . | . | . |
| DHFR ---- tag | rs1805355 | 0 | 52 | 86,67 | 22 | 68,75 | 302 | 88,56 | 265 | 86,60 | 1.00 (.-.) | . | 1.86 (1.10-3.15) | 0.02 | 0.01 | 0.99 | 0.07 |
| DHFR ---- tag |  | 1 | 8 | 13,33 | 10 | 31,25 | 38 | 11,14 | 40 | 13,07 | 4.37 (1.89-10.12) | 0.00 | 2.10 (1.13-3.88) | 0.02 | . | . | . |
| DHFR ---- tag |  | 2 | 0 | 0,00 | 0 | 0,00 | 1 | 0,29 | 1 | 0,33 | 19.10 (3.56-102.4) | 0.00 | 2.36 (0.98-5.71) | 0.06 | . | . | . |
| DHFR ---- tag | rs6151617 | 0 | 27 | 45,00 | 9 | 28,13 | 118 | 34,60 | 108 | 35,29 | 1.00 (.-.) | . | 1.73 (0.87-3.47) | 0.12 | 0.37 | 0.99 | 0.68 |
| DHFR ---- tag |  | 1 | 26 | 43,33 | 14 | 43,75 | 155 | 45,45 | 155 | 50,65 | 1.20 (0.70-2.05) | 0.50 | 1.60 (0.81-3.15) | 0.17 | . | . | . |
| DHFR ---- tag |  | 2 | 7 | 11,67 | 9 | 28,13 | 68 | 19,94 | 43 | 14,05 | 1.44 (0.49-4.19) | 0.50 | 1.48 (0.73-3.02) | 0.28 | . | . | . |
| DHFR ---- tag | rs6864493 | 0 | 38 | 63,33 | 17 | 53,13 | 179 | 52,49 | 178 | 58,17 | 1.00 (.-.) | . | 1.25 (0.72-2.18) | 0.43 | 0.69 | 0.99 | 0.81 |
| DHFR ---- tag |  | 1 | 19 | 31,67 | 12 | 37,50 | 135 | 39,59 | 115 | 37,58 | 0.73 (0.38-1.40) | 0.34 | 1.05 (0.60-1.83) | 0.87 | . | . | . |
| DHFR ---- tag |  | 2 | 3 | 5,00 | 3 | 9,38 | 27 | 7,92 | 13 | 4,25 | 0.53 (0.15-1.95) | 0.34 | 0.88 (0.46-1.67) | 0.69 | . | . | . |
| DHFR ---- tag | rs836788 | 0 | 25 | 41,67 | 19 | 59,38 | 153 | 44,87 | 122 | 39,87 | 1.00 (.-.) | . | 1.42 (0.82-2.46) | 0.21 | 0.81 | 0.99 | 0.81 |
| DHFR ---- tag |  | 1 | 28 | 46,67 | 10 | 31,25 | 143 | 41,94 | 142 | 46,41 | 1.10 (0.58-2.06) | 0.78 | 1.44 (0.85-2.44) | 0.18 | . | . | . |
| DHFR ---- tag |  | 2 | 7 | 11,67 | 3 | 9,38 | 45 | 13,20 | 42 | 13,73 | 1.20 (0.34-4.23) | 0.78 | 1.45 (0.82-2.59) | 0.21 | . | . | . |
| DHFR ---- tag | rs836790 | 0 | 37 | 61,67 | 22 | 68,75 | 244 | 71,55 | 209 | 68,30 | 1.00 (.-.) | . | 1.22 (0.72-2.05) | 0.47 | 0.41 | 0.99 | 0.68 |
| DHFR ---- tag |  | 1 | 21 | 35,00 | 10 | 31,25 | 88 | 25,81 | 89 | 29,08 | 0.82 (0.37-1.81) | 0.62 | 1.40 (0.81-2.41) | 0.22 | . | . | . |
| DHFR ---- tag |  | 2 | 2 | 3,33 | 0 | 0,00 | 9 | 2,64 | 8 | 2,61 | 0.66 (0.14-3.26) | 0.62 | 1.61 (0.83-3.12) | 0.16 | . | . | . |
| DHFR ---- tag | rs836817 | 0 | 26 | 43,33 | 20 | 62,50 | 172 | 50,44 | 134 | 43,79 | 1.00 (.-.) | . | 1.48 (0.88-2.49) | 0.14 | 0.68 | 0.99 | 0.81 |
| DHFR ---- tag |  | 1 | 28 | 46,67 | 10 | 31,25 | 130 | 38,12 | 145 | 47,39 | 1.09 (0.54-2.21) | 0.82 | 1.38 (0.83-2.30) | 0.21 | . | . | . |
| DHFR ---- tag |  | 2 | 6 | 10,00 | 2 | 6,25 | 39 | 11,44 | 27 | 8,82 | 1.18 (0.29-4.88) | 0.82 | 1.29 (0.73-2.27) | 0.39 | . | . | . |
| DNMT1 ---- candidate | rs2228612 | 0 | 49 | 81,67 | 26 | 81,25 | 303 | 88,86 | 267 | 87,25 | 1.00 (.-.) | . | 1.34 (0.85-2.12) | 0.21 | 0.77 | 0.99 | 0.77 |
| DNMT1 ---- candidate |  | 1 | 11 | 18,33 | 6 | 18,75 | 37 | 10,85 | 38 | 12,42 | 0.83 (0.25-2.79) | 0.76 | 1.33 (0.77-2.31) | 0.30 | . | . | . |
| DNMT1 ---- candidate |  | 2 | 0 | 0,00 | 0 | 0,00 | 1 | 0,29 | 1 | 0,33 | 0.68 (0.06-7.80) | 0.76 | 1.33 (0.60-2.94) | 0.48 | . | . | . |
| DNMT3A ---- tag | rs10460566 | 0 | 35 | 58,33 | 21 | 65,63 | 198 | 58,06 | 184 | 60,13 | 1.00 (.-.) | . | 1.38 (0.84-2.27) | 0.21 | 0.98 | 1.00 | 0.98 |
| DNMT3A ---- tag |  | 1 | 19 | 31,67 | 11 | 34,38 | 127 | 37,24 | 105 | 34,31 | 1.00 (0.49-2.04) | 1.00 | 1.37 (0.83-2.26) | 0.22 | . | . | . |
| DNMT3A ---- tag |  | 2 | 6 | 10,00 | 0 | 0,00 | 16 | 4,69 | 17 | 5,56 | 1.00 (0.24-4.17) | 1.00 | 1.36 (0.75-2.45) | 0.31 | . | . | . |
| DNMT3A ---- candidate literature | rs11695471 | 0 | 29 | 48,33 | 11 | 34,38 | 143 | 41,94 | 138 | 45,10 | 1.00 (.-.) | . | 1.26 (0.69-2.31) | 0.45 | 0.70 | 0.99 | 0.98 |
| DNMT3A ---- candidate literature |  | 1 | 24 | 40,00 | 15 | 46,88 | 152 | 44,57 | 131 | 42,81 | 0.94 (0.51-1.72) | 0.84 | 1.34 (0.74-2.44) | 0.34 | . | . | . |
| DNMT3A ---- candidate literature |  | 2 | 7 | 11,67 | 6 | 18,75 | 46 | 13,49 | 37 | 12,09 | 0.88 (0.26-2.96) | 0.84 | 1.43 (0.75-2.71) | 0.28 | . | . | . |
| DNMT3A ---- tag | rs11887120 | 0 | 19 | 31,67 | 14 | 43,75 | 124 | 36,36 | 105 | 34,31 | 1.00 (.-.) | . | 1.26 (0.65-2.45) | 0.49 | 0.73 | 0.99 | 0.98 |
| DNMT3A ---- tag |  | 1 | 32 | 53,33 | 14 | 43,75 | 164 | 48,09 | 143 | 46,73 | 0.85 (0.45-1.58) | 0.60 | 1.20 (0.63-2.28) | 0.59 | . | . | . |
| DNMT3A ---- tag |  | 2 | 9 | 15,00 | 4 | 12,50 | 53 | 15,54 | 58 | 18,95 | 0.71 (0.20-2.51) | 0.60 | 1.13 (0.58-2.22) | 0.72 | . | . | . |
| DNMT3A ---- tag | rs12991495 | 0 | 32 | 53,33 | 12 | 37,50 | 150 | 43,99 | 150 | 49,02 | 1.00 (.-.) | . | 1.37 (0.74-2.54) | 0.32 | 0.96 | 1.00 | 0.98 |
| DNMT3A ---- tag |  | 1 | 22 | 36,67 | 14 | 43,75 | 156 | 45,75 | 120 | 39,22 | 1.08 (0.59-1.97) | 0.81 | 1.50 (0.81-2.76) | 0.20 | . | . | . |
| DNMT3A ---- tag |  | 2 | 6 | 10,00 | 6 | 18,75 | 35 | 10,26 | 36 | 11,76 | 1.16 (0.35-3.86) | 0.81 | 1.64 (0.85-3.17) | 0.14 | . | . | . |
| DNMT3A ---- tag | rs13401241 | 0 | 22 | 36,67 | 7 | 21,88 | 95 | 27,86 | 85 | 27,78 | 1.00 (.-.) | . | 1.53 (0.76-3.10) | 0.24 | 0.69 | 0.99 | 0.98 |
| DNMT3A ---- tag |  | 1 | 24 | 40,00 | 19 | 59,38 | 172 | 50,44 | 164 | 53,59 | 1.05 (0.62-1.77) | 0.86 | 1.43 (0.73-2.83) | 0.30 | . | . | . |
| DNMT3A ---- tag |  | 2 | 14 | 23,33 | 6 | 18,75 | 74 | 21,70 | 57 | 18,63 | 1.10 (0.38-3.15) | 0.86 | 1.34 (0.66-2.74) | 0.42 | . | . | . |
| DNMT3A ---- candidate literature | rs13420827 | 0 | 42 | 70,00 | 21 | 65,63 | 208 | 61,00 | 210 | 68,63 | 1.00 (.-.) | . | 1.55 (0.91-2.64) | 0.11 | 0.40 | 0.99 | 0.98 |
| DNMT3A ---- candidate literature |  | 1 | 13 | 21,67 | 8 | 25,00 | 120 | 35,19 | 86 | 28,10 | 1.07 (0.62-1.84) | 0.81 | 1.28 (0.74-2.20) | 0.38 | . | . | . |
| DNMT3A ---- candidate literature |  | 2 | 5 | 8,33 | 3 | 9,38 | 13 | 3,81 | 10 | 3,27 | 1.14 (0.39-3.38) | 0.81 | 1.05 (0.55-2.03) | 0.88 | . | . | . |
| DNMT3A ---- tag | rs13428812 | 0 | 30 | 50,00 | 15 | 46,88 | 172 | 50,44 | 135 | 44,12 | 1.00 (.-.) | . | 1.58 (0.88-2.82) | 0.13 | 0.45 | 0.99 | 0.98 |
| DNMT3A ---- tag |  | 1 | 22 | 36,67 | 15 | 46,88 | 141 | 41,35 | 146 | 47,71 | 1.39 (0.76-2.52) | 0.29 | 1.71 (0.96-3.04) | 0.07 | . | . | . |
| DNMT3A ---- tag |  | 2 | 8 | 13,33 | 2 | 6,25 | 28 | 8,21 | 25 | 8,17 | 1.92 (0.58-6.37) | 0.29 | 1.85 (0.97-3.53) | 0.06 | . | . | . |
| DNMT3A ---- tag | rs4665287 | 0 | 43 | 71,67 | 21 | 65,63 | 213 | 62,46 | 215 | 70,26 | 1.00 (.-.) | . | 1.55 (0.91-2.63) | 0.11 | 0.39 | 0.99 | 0.98 |
| DNMT3A ---- tag |  | 1 | 12 | 20,00 | 8 | 25,00 | 114 | 33,43 | 81 | 26,47 | 1.05 (0.61-1.80) | 0.86 | 1.25 (0.73-2.16) | 0.42 | . | . | . |
| DNMT3A ---- tag |  | 2 | 5 | 8,33 | 3 | 9,38 | 14 | 4,11 | 10 | 3,27 | 1.10 (0.38-3.23) | 0.86 | 1.01 (0.52-1.95) | 0.97 | . | . | . |
| DNMT3B ---- tag | rs13045669 | 0 | 56 | 93,33 | 29 | 90,63 | 314 | 92,08 | 285 | 93,14 | 1.00 (.-.) | . | 1.38 (0.88-2.16) | 0.16 | 0.79 | 0.99 | 0.97 |
| DNMT3B ---- tag |  | 1 | 4 | 6,67 | 3 | 9,38 | 27 | 7,92 | 20 | 6,54 | 0.81 (0.18-3.55) | 0.78 | 0.90 (0.44-1.83) | 0.77 | . | . | . |
| DNMT3B ---- tag |  | 2 | 0 | 0,00 | 0 | 0,00 | 0 | 0,00 | 1 | 0,33 | 0.65 (0.03-12.62) | 0.78 | 0.59 (0.18-1.96) | 0.39 | . | . | . |
| DNMT3B ---- tag | rs17123673 | 0 | 54 | 90,00 | 29 | 90,63 | 313 | 91,79 | 282 | 92,16 | 1.00 (.-.) | . | 1.39 (0.88-2.19) | 0.16 | 0.89 | 1.00 | 0.97 |
| DNMT3B ---- tag |  | 1 | 6 | 10,00 | 3 | 9,38 | 28 | 8,21 | 22 | 7,19 | 1.08 (0.32-3.67) | 0.90 | 1.37 (0.75-2.52) | 0.31 | . | . | . |
| DNMT3B ---- tag |  | 2 | 0 | 0,00 | 0 | 0,00 | 0 | 0,00 | 2 | 0,65 | 1.17 (0.10-13.46) | 0.90 | 1.36 (0.52-3.57) | 0.54 | . | . | . |
| DNMT3B ---- tag | rs183603 | 0 | 30 | 50,00 | 20 | 62,50 | 181 | 53,08 | 166 | 54,25 | 1.00 (.-.) | . | 1.13 (0.65-1.96) | 0.67 | 0.33 | 0.99 | 0.74 |
| DNMT3B ---- tag |  | 1 | 23 | 38,33 | 10 | 31,25 | 132 | 38,71 | 125 | 40,85 | 0.60 (0.30-1.19) | 0.14 | 0.95 (0.55-1.66) | 0.87 | . | . | . |
| DNMT3B ---- tag |  | 2 | 7 | 11,67 | 2 | 6,25 | 28 | 8,21 | 15 | 4,90 | 0.36 (0.09-1.41) | 0.14 | 0.81 (0.43-1.52) | 0.51 | . | . | . |
| DNMT3B ---- tag | rs2235760 | 0 | 40 | 66,67 | 22 | 68,75 | 246 | 72,14 | 221 | 72,22 | 1.00 (.-.) | . | 1.42 (0.87-2.31) | 0.16 | 0.78 | 0.99 | 0.97 |
| DNMT3B ---- tag |  | 1 | 18 | 30,00 | 10 | 31,25 | 87 | 25,51 | 74 | 24,18 | 1.13 (0.50-2.57) | 0.77 | 1.42 (0.85-2.38) | 0.18 | . | . | . |
| DNMT3B ---- tag |  | 2 | 2 | 3,33 | 0 | 0,00 | 8 | 2,35 | 11 | 3,59 | 1.28 (0.25-6.62) | 0.77 | 1.42 (0.74-2.72) | 0.29 | . | . | . |
| DNMT3B ---- tag | rs2424908 | 0 | 38 | 63,33 | 22 | 68,75 | 212 | 62,17 | 196 | 64,05 | 1.00 (.-.) | . | 1.19 (0.71-1.99) | 0.51 | 0.37 | 0.99 | 0.74 |
| DNMT3B ---- tag |  | 1 | 18 | 30,00 | 10 | 31,25 | 109 | 31,96 | 101 | 33,01 | 0.55 (0.24-1.29) | 0.17 | 0.97 (0.57-1.65) | 0.92 | . | . | . |
| DNMT3B ---- tag |  | 2 | 4 | 6,67 | 0 | 0,00 | 20 | 5,87 | 9 | 2,94 | 0.30 (0.06-1.65) | 0.17 | 0.79 (0.42-1.49) | 0.47 | . | . | . |
| DNMT3B ---- candidate literature | rs2424909 | 0 | 19 | 31,67 | 12 | 37,50 | 128 | 37,54 | 129 | 42,16 | 1.00 (.-.) | . | 0.96 (0.54-1.71) | 0.88 | 0.11 | 0.99 | 0.65 |
| DNMT3B ---- candidate literature |  | 1 | 27 | 45,00 | 20 | 62,50 | 160 | 46,92 | 127 | 41,50 | 0.52 (0.27-1.02) | 0.06 | 0.87 (0.49-1.53) | 0.63 | . | . | . |
| DNMT3B ---- candidate literature |  | 2 | 14 | 23,33 | 0 | 0,00 | 53 | 15,54 | 50 | 16,34 | 0.27 (0.07-1.04) | 0.06 | 0.79 (0.43-1.46) | 0.45 | . | . | . |
| DNMT3B ---- tag | rs4911108 | 0 | 20 | 33,33 | 13 | 40,63 | 138 | 40,47 | 135 | 44,12 | 1.00 (.-.) | . | 0.98 (0.55-1.74) | 0.93 | 0.13 | 0.99 | 0.65 |
| DNMT3B ---- tag |  | 1 | 27 | 45,00 | 19 | 59,38 | 153 | 44,87 | 125 | 40,85 | 0.53 (0.27-1.04) | 0.07 | 0.88 (0.50-1.55) | 0.65 | . | . | . |
| DNMT3B ---- tag |  | 2 | 13 | 21,67 | 0 | 0,00 | 50 | 14,66 | 46 | 15,03 | 0.28 (0.07-1.08) | 0.07 | 0.79 (0.43-1.46) | 0.45 | . | . | . |
| DNMT3B ---- tag | rs6058896 | 0 | 53 | 88,33 | 28 | 87,50 | 304 | 89,15 | 273 | 89,22 | 1.00 (.-.) | . | 1.36 (0.87-2.12) | 0.18 | 0.97 | 1.00 | 0.97 |
| DNMT3B ---- tag |  | 1 | 6 | 10,00 | 3 | 9,38 | 36 | 10,56 | 32 | 10,46 | 1.35 (0.35-5.12) | 0.66 | 1.79 (1.02-3.13) | 0.04 | . | . | . |
| DNMT3B ---- tag |  | 2 | 1 | 1,67 | 1 | 3,13 | 1 | 0,29 | 1 | 0,33 | 1.81 (0.13-26.25) | 0.66 | 2.34 (0.99-5.56) | 0.05 | . | . | . |
| DNMT3B ---- tag | rs6119954 | 0 | 36 | 60,00 | 24 | 75,00 | 242 | 70,97 | 220 | 71,90 | 1.00 (.-.) | . | 1.21 (0.75-1.96) | 0.43 | 0.31 | 0.99 | 0.74 |
| DNMT3B ---- tag |  | 1 | 21 | 35,00 | 8 | 25,00 | 89 | 26,10 | 73 | 23,86 | 0.66 (0.28-1.58) | 0.36 | 1.26 (0.76-2.09) | 0.37 | . | . | . |
| DNMT3B ---- tag |  | 2 | 3 | 5,00 | 0 | 0,00 | 10 | 2,93 | 13 | 4,25 | 0.44 (0.08-2.50) | 0.36 | 1.32 (0.70-2.47) | 0.39 | . | . | . |
| DNMT3B ---- tag | rs6579038 | 0 | 54 | 90,00 | 29 | 90,63 | 301 | 88,27 | 273 | 89,22 | 1.00 (.-.) | . | 1.39 (0.89-2.17) | 0.15 | 0.70 | 0.99 | 0.97 |
| DNMT3B ---- tag |  | 1 | 6 | 10,00 | 3 | 9,38 | 39 | 11,44 | 32 | 10,46 | 1.65 (0.38-7.13) | 0.50 | 1.69 (0.96-2.98) | 0.07 | . | . | . |
| DNMT3B ---- tag |  | 2 | 0 | 0,00 | 0 | 0,00 | 1 | 0,29 | 1 | 0,33 | 2.72 (0.15-50.79) | 0.50 | 2.06 (0.86-4.91) | 0.10 | . | . | . |
| DPYD ---- tag | rs1034215 | 0 | 38 | 63,33 | 19 | 59,38 | 194 | 56,89 | 188 | 61,44 | 1.00 (.-.) | . | 1.75 (0.98-3.13) | 0.06 | 0.18 | 0.99 | 0.72 |
| DPYD ---- tag |  | 1 | 20 | 33,33 | 13 | 40,63 | 129 | 37,83 | 97 | 31,70 | 1.70 (0.79-3.67) | 0.18 | 1.70 (0.95-3.06) | 0.08 | . | . | . |
| DPYD ---- tag |  | 2 | 2 | 3,33 | 0 | 0,00 | 18 | 5,28 | 21 | 6,86 | 2.90 (0.62-13.50) | 0.18 | 1.65 (0.84-3.25) | 0.14 | . | . | . |
| DPYD ---- tag | rs10783058 | 0 | 31 | 51,67 | 17 | 53,13 | 135 | 39,59 | 118 | 38,56 | 1.00 (.-.) | . | 1.19 (0.66-2.14) | 0.57 | 0.50 | 0.99 | 0.77 |
| DPYD ---- tag |  | 1 | 22 | 36,67 | 11 | 34,38 | 165 | 48,39 | 147 | 48,04 | 0.93 (0.53-1.62) | 0.80 | 1.35 (0.76-2.40) | 0.31 | . | . | . |
| DPYD ---- tag |  | 2 | 7 | 11,67 | 4 | 12,50 | 41 | 12,02 | 41 | 13,40 | 0.87 (0.29-2.64) | 0.80 | 1.53 (0.83-2.84) | 0.18 | . | . | . |
| DPYD ---- tag | rs10783070 | 0 | 43 | 71,67 | 27 | 84,38 | 252 | 73,90 | 205 | 66,99 | 1.00 (.-.) | . | 1.29 (0.79-2.10) | 0.31 | 0.64 | 0.99 | 0.84 |
| DPYD ---- tag |  | 1 | 16 | 26,67 | 3 | 9,38 | 82 | 24,05 | 93 | 30,39 | 1.12 (0.53-2.37) | 0.77 | 1.74 (1.05-2.88) | 0.03 | . | . | . |
| DPYD ---- tag |  | 2 | 1 | 1,67 | 2 | 6,25 | 7 | 2,05 | 8 | 2,61 | 1.25 (0.28-5.61) | 0.77 | 2.34 (1.25-4.37) | 0.01 | . | . | . |
| DPYD ---- tag | rs10875048 | 0 | 41 | 68,33 | 22 | 68,75 | 241 | 70,67 | 201 | 65,69 | 1.00 (.-.) | . | 1.12 (0.67-1.88) | 0.66 | 0.20 | 0.99 | 0.72 |
| DPYD ---- tag |  | 1 | 14 | 23,33 | 9 | 28,13 | 94 | 27,57 | 96 | 31,37 | 0.73 (0.39-1.37) | 0.33 | 1.27 (0.75-2.15) | 0.38 | . | . | . |
| DPYD ---- tag |  | 2 | 5 | 8,33 | 1 | 3,13 | 6 | 1,76 | 9 | 2,94 | 0.53 (0.15-1.88) | 0.33 | 1.43 (0.75-2.73) | 0.28 | . | . | . |
| DPYD ---- tag | rs10875055 | 0 | 30 | 50,00 | 7 | 21,88 | 96 | 28,15 | 64 | 20,92 | 1.00 (.-.) | . | 1.64 (0.82-3.27) | 0.16 | 0.35 | 0.99 | 0.77 |
| DPYD ---- tag |  | 1 | 24 | 40,00 | 18 | 56,25 | 177 | 51,91 | 162 | 52,94 | 1.66 (0.94-2.92) | 0.08 | 2.05 (1.05-3.99) | 0.04 | . | . | . |
| DPYD ---- tag |  | 2 | 6 | 10,00 | 7 | 21,88 | 68 | 19,94 | 80 | 26,14 | 2.75 (0.89-8.54) | 0.08 | 2.56 (1.28-5.12) | 0.01 | . | . | . |
| DPYD ---- tag | rs10875079 | 0 | 9 | 15,00 | 10 | 31,25 | 88 | 25,81 | 90 | 29,41 | 1.00 (.-.) | . | 1.29 (0.60-2.77) | 0.52 | 0.88 | 1.00 | 0.93 |
| DPYD ---- tag |  | 1 | 38 | 63,33 | 16 | 50,00 | 164 | 48,09 | 150 | 49,02 | 0.79 (0.43-1.44) | 0.44 | 1.07 (0.51-2.25) | 0.87 | . | . | . |
| DPYD ---- tag |  | 2 | 13 | 21,67 | 6 | 18,75 | 89 | 26,10 | 66 | 21,57 | 0.62 (0.19-2.08) | 0.44 | 0.88 (0.41-1.91) | 0.75 | . | . | . |
| DPYD ---- tag | rs10875085 | 0 | 44 | 73,33 | 19 | 59,38 | 253 | 74,19 | 192 | 62,75 | 1.00 (.-.) | . | 1.52 (0.88-2.61) | 0.13 | 0.50 | 0.99 | 0.77 |
| DPYD ---- tag |  | 1 | 14 | 23,33 | 12 | 37,50 | 81 | 23,75 | 101 | 33,01 | 1.51 (0.73-3.13) | 0.27 | 1.76 (1.01-3.05) | 0.05 | . | . | . |
| DPYD ---- tag |  | 2 | 2 | 3,33 | 1 | 3,13 | 7 | 2,05 | 13 | 4,25 | 2.28 (0.53-9.82) | 0.27 | 2.03 (1.07-3.87) | 0.03 | . | . | . |
| DPYD ---- tag | rs10875097 | 0 | 43 | 71,67 | 19 | 59,38 | 236 | 69,21 | 203 | 66,34 | 1.00 (.-.) | . | 1.88 (1.08-3.27) | 0.03 | 0.03 | 0.99 | 0.55 |
| DPYD ---- tag |  | 1 | 17 | 28,33 | 12 | 37,50 | 93 | 27,27 | 92 | 30,07 | 2.39 (1.22-4.68) | 0.01 | 1.95 (1.11-3.44) | 0.02 | . | . | . |
| DPYD ---- tag |  | 2 | 0 | 0,00 | 1 | 3,13 | 12 | 3,52 | 11 | 3,59 | 5.70 (1.48-21.93) | 0.01 | 2.02 (1.03-3.95) | 0.04 | . | . | . |
| DPYD ---- tag | rs11165781 | 0 | 41 | 68,33 | 24 | 75,00 | 244 | 71,55 | 197 | 64,38 | 1.00 (.-.) | . | 1.13 (0.71-1.80) | 0.59 | 0.08 | 0.99 | 0.72 |
| DPYD ---- tag |  | 1 | 18 | 30,00 | 7 | 21,88 | 91 | 26,69 | 101 | 33,01 | 0.52 (0.17-1.57) | 0.25 | 1.41 (0.87-2.29) | 0.16 | . | . | . |
| DPYD ---- tag |  | 2 | 1 | 1,67 | 1 | 3,13 | 6 | 1,76 | 8 | 2,61 | 0.27 (0.03-2.47) | 0.25 | 1.76 (0.95-3.26) | 0.07 | . | . | . |
| DPYD ---- tag | rs11165783 | 0 | 31 | 51,67 | 20 | 62,50 | 184 | 53,96 | 171 | 55,88 | 1.00 (.-.) | . | 1.55 (0.87-2.76) | 0.14 | 0.53 | 0.99 | 0.77 |
| DPYD ---- tag |  | 1 | 25 | 41,67 | 11 | 34,38 | 130 | 38,12 | 107 | 34,97 | 1.19 (0.59-2.41) | 0.62 | 1.46 (0.82-2.59) | 0.20 | . | . | . |
| DPYD ---- tag |  | 2 | 4 | 6,67 | 1 | 3,13 | 27 | 7,92 | 28 | 9,15 | 1.43 (0.35-5.83) | 0.62 | 1.38 (0.73-2.60) | 0.33 | . | . | . |
| DPYD ---- tag | rs11165873 | 0 | 12 | 20,00 | 9 | 28,13 | 93 | 27,27 | 94 | 30,72 | 1.00 (.-.) | . | 1.13 (0.56-2.27) | 0.73 | 0.54 | 0.99 | 0.77 |
| DPYD ---- tag |  | 1 | 28 | 46,67 | 16 | 50,00 | 177 | 51,91 | 150 | 49,02 | 0.79 (0.46-1.35) | 0.39 | 1.07 (0.54-2.10) | 0.85 | . | . | . |
| DPYD ---- tag |  | 2 | 20 | 33,33 | 7 | 21,88 | 71 | 20,82 | 62 | 20,26 | 0.62 (0.21-1.83) | 0.39 | 1.01 (0.49-2.06) | 0.98 | . | . | . |
| DPYD ---- tag | rs11165875 | 0 | 27 | 45,00 | 13 | 40,63 | 141 | 41,35 | 117 | 38,24 | 1.00 (.-.) | . | 1.61 (0.88-2.96) | 0.13 | 0.43 | 0.99 | 0.77 |
| DPYD ---- tag |  | 1 | 29 | 48,33 | 12 | 37,50 | 160 | 46,92 | 141 | 46,08 | 1.29 (0.75-2.22) | 0.37 | 1.63 (0.91-2.95) | 0.10 | . | . | . |
| DPYD ---- tag |  | 2 | 4 | 6,67 | 7 | 21,88 | 40 | 11,73 | 48 | 15,69 | 1.66 (0.56-4.93) | 0.37 | 1.66 (0.88-3.13) | 0.12 | . | . | . |
| DPYD ---- tag | rs11165881 | 0 | 24 | 40,00 | 15 | 46,88 | 112 | 32,84 | 107 | 34,97 | 1.00 (.-.) | . | 1.25 (0.65-2.41) | 0.50 | 0.72 | 0.99 | 0.84 |
| DPYD ---- tag |  | 1 | 26 | 43,33 | 13 | 40,63 | 161 | 47,21 | 147 | 48,04 | 0.95 (0.51-1.77) | 0.88 | 1.35 (0.72-2.55) | 0.35 | . | . | . |
| DPYD ---- tag |  | 2 | 10 | 16,67 | 4 | 12,50 | 68 | 19,94 | 52 | 16,99 | 0.91 (0.27-3.13) | 0.88 | 1.45 (0.74-2.84) | 0.27 | . | . | . |
| DPYD ---- tag | rs11587873 | 0 | 33 | 55,00 | 22 | 68,75 | 186 | 54,55 | 188 | 61,44 | 1.00 (.-.) | . | 1.16 (0.68-1.98) | 0.58 | 0.41 | 0.99 | 0.77 |
| DPYD ---- tag |  | 1 | 23 | 38,33 | 9 | 28,13 | 124 | 36,36 | 110 | 35,95 | 0.59 (0.28-1.27) | 0.18 | 0.96 (0.55-1.66) | 0.88 | . | . | . |
| DPYD ---- tag |  | 2 | 4 | 6,67 | 1 | 3,13 | 31 | 9,09 | 8 | 2,61 | 0.35 (0.08-1.62) | 0.18 | 0.79 (0.41-1.51) | 0.47 | . | . | . |
| DPYD ---- tag | rs12030174 | 0 | 46 | 76,67 | 20 | 62,50 | 266 | 78,01 | 205 | 66,99 | 1.00 (.-.) | . | 1.50 (0.88-2.54) | 0.13 | 0.55 | 0.99 | 0.77 |
| DPYD ---- tag |  | 1 | 13 | 21,67 | 11 | 34,38 | 69 | 20,23 | 91 | 29,74 | 1.43 (0.68-3.00) | 0.34 | 1.68 (0.97-2.89) | 0.06 | . | . | . |
| DPYD ---- tag |  | 2 | 1 | 1,67 | 1 | 3,13 | 6 | 1,76 | 10 | 3,27 | 2.04 (0.47-8.98) | 0.34 | 1.88 (0.97-3.64) | 0.06 | . | . | . |
| DPYD ---- tag | rs12046744 | 0 | 32 | 53,33 | 17 | 53,13 | 181 | 53,08 | 176 | 57,52 | 1.00 (.-.) | . | 1.68 (0.97-2.89) | 0.06 | 0.21 | 0.99 | 0.72 |
| DPYD ---- tag |  | 1 | 24 | 40,00 | 12 | 37,50 | 129 | 37,83 | 110 | 35,95 | 1.38 (0.75-2.54) | 0.31 | 1.50 (0.87-2.60) | 0.14 | . | . | . |
| DPYD ---- tag |  | 2 | 4 | 6,67 | 3 | 9,38 | 31 | 9,09 | 20 | 6,54 | 1.89 (0.56-6.44) | 0.31 | 1.35 (0.72-2.52) | 0.35 | . | . | . |
| DPYD ---- tag | rs12047910 | 0 | 44 | 73,33 | 23 | 71,88 | 258 | 75,66 | 223 | 72,88 | 1.00 (.-.) | . | 1.39 (0.82-2.36) | 0.22 | 0.95 | 1.00 | 0.97 |
| DPYD ---- tag |  | 1 | 15 | 25,00 | 8 | 25,00 | 79 | 23,17 | 77 | 25,16 | 1.10 (0.52-2.32) | 0.80 | 1.50 (0.86-2.59) | 0.15 | . | . | . |
| DPYD ---- tag |  | 2 | 1 | 1,67 | 1 | 3,13 | 4 | 1,17 | 6 | 1,96 | 1.22 (0.27-5.40) | 0.80 | 1.61 (0.81-3.18) | 0.17 | . | . | . |
| DPYD ---- tag | rs12073044 | 0 | 52 | 86,67 | 27 | 84,38 | 261 | 76,54 | 240 | 78,43 | 1.00 (.-.) | . | 1.43 (0.89-2.29) | 0.14 | 0.75 | 0.99 | 0.84 |
| DPYD ---- tag |  | 1 | 8 | 13,33 | 5 | 15,63 | 76 | 22,29 | 65 | 21,24 | 1.08 (0.36-3.24) | 0.89 | 1.28 (0.75-2.17) | 0.36 | . | . | . |
| DPYD ---- tag |  | 2 | 0 | 0,00 | 0 | 0,00 | 4 | 1,17 | 1 | 0,33 | 1.17 (0.13-10.51) | 0.89 | 1.14 (0.55-2.36) | 0.72 | . | . | . |
| DPYD ---- tag | rs12126093 | 0 | 23 | 38,33 | 14 | 43,75 | 174 | 51,03 | 164 | 53,59 | 1.00 (.-.) | . | 1.29 (0.70-2.36) | 0.41 | 0.82 | 0.99 | 0.90 |
| DPYD ---- tag |  | 1 | 31 | 51,67 | 14 | 43,75 | 142 | 41,64 | 117 | 38,24 | 0.87 (0.48-1.57) | 0.63 | 1.20 (0.66-2.19) | 0.55 | . | . | . |
| DPYD ---- tag |  | 2 | 6 | 10,00 | 4 | 12,50 | 25 | 7,33 | 25 | 8,17 | 0.75 (0.23-2.46) | 0.63 | 1.12 (0.58-2.17) | 0.74 | . | . | . |
| DPYD ---- tag | rs12134028 | 0 | 51 | 85,00 | 30 | 93,75 | 309 | 90,62 | 273 | 89,22 | 1.00 (.-.) | . | 1.09 (0.70-1.70) | 0.71 | 0.02 | 0.99 | 0.47 |
| DPYD ---- tag |  | 1 | 9 | 15,00 | 1 | 3,13 | 32 | 9,38 | 32 | 10,46 | 0.13 (0.02-0.97) | 0.05 | 0.97 (0.55-1.72) | 0.92 | . | . | . |
| DPYD ---- tag |  | 2 | 0 | 0,00 | 1 | 3,13 | 0 | 0,00 | 1 | 0,33 | 0.02 (0.00-0.94) | 0.05 | 0.87 (0.36-2.08) | 0.75 | . | . | . |
| DPYD ---- tag | rs12740796 | 0 | 41 | 68,33 | 24 | 75,00 | 253 | 74,19 | 235 | 76,80 | 1.00 (.-.) | . | 1.55 (0.95-2.54) | 0.08 | 0.27 | 0.99 | 0.77 |
| DPYD ---- tag |  | 1 | 18 | 30,00 | 6 | 18,75 | 82 | 24,05 | 65 | 21,24 | 1.46 (0.76-2.79) | 0.26 | 1.50 (0.88-2.54) | 0.13 | . | . | . |
| DPYD ---- tag |  | 2 | 1 | 1,67 | 2 | 6,25 | 6 | 1,76 | 6 | 1,96 | 2.12 (0.58-7.78) | 0.26 | 1.45 (0.74-2.85) | 0.28 | . | . | . |
| DPYD ---- tag | rs1333717 | 0 | 37 | 61,67 | 18 | 56,25 | 187 | 54,84 | 178 | 58,17 | 1.00 (.-.) | . | 1.73 (0.97-3.10) | 0.07 | 0.20 | 0.99 | 0.72 |
| DPYD ---- tag |  | 1 | 20 | 33,33 | 14 | 43,75 | 135 | 39,59 | 106 | 34,64 | 1.67 (0.77-3.61) | 0.19 | 1.71 (0.95-3.06) | 0.07 | . | . | . |
| DPYD ---- tag |  | 2 | 3 | 5,00 | 0 | 0,00 | 19 | 5,57 | 22 | 7,19 | 2.80 (0.60-13.07) | 0.19 | 1.68 (0.86-3.27) | 0.13 | . | . | . |
| DPYD ---- tag | rs1413228 | 0 | 49 | 81,67 | 28 | 87,50 | 275 | 80,65 | 237 | 77,45 | 1.00 (.-.) | . | 1.30 (0.83-2.05) | 0.25 | 0.53 | 0.99 | 0.77 |
| DPYD ---- tag |  | 1 | 11 | 18,33 | 3 | 9,38 | 61 | 17,89 | 65 | 21,24 | 0.75 (0.26-2.20) | 0.60 | 1.37 (0.83-2.28) | 0.22 | . | . | . |
| DPYD ---- tag |  | 2 | 0 | 0,00 | 1 | 3,13 | 5 | 1,47 | 4 | 1,31 | 0.57 (0.07-4.84) | 0.60 | 1.45 (0.72-2.89) | 0.30 | . | . | . |
| DPYD ---- tag | rs1415681 | 0 | 46 | 76,67 | 26 | 81,25 | 240 | 70,38 | 232 | 75,82 | 1.00 (.-.) | . | 1.24 (0.76-2.02) | 0.39 | 0.42 | 0.99 | 0.77 |
| DPYD ---- tag |  | 1 | 13 | 21,67 | 6 | 18,75 | 94 | 27,57 | 64 | 20,92 | 0.74 (0.27-2.00) | 0.55 | 1.38 (0.83-2.29) | 0.22 | . | . | . |
| DPYD ---- tag |  | 2 | 1 | 1,67 | 0 | 0,00 | 7 | 2,05 | 10 | 3,27 | 0.54 (0.07-4.01) | 0.55 | 1.53 (0.80-2.92) | 0.20 | . | . | . |
| DPYD ---- tag | rs1514495 | 0 | 31 | 51,67 | 19 | 59,38 | 217 | 63,64 | 179 | 58,50 | 1.00 (.-.) | . | 1.18 (0.71-1.98) | 0.53 | 0.34 | 0.99 | 0.77 |
| DPYD ---- tag |  | 1 | 23 | 38,33 | 10 | 31,25 | 102 | 29,91 | 111 | 36,27 | 0.68 (0.35-1.33) | 0.26 | 1.12 (0.67-1.88) | 0.67 | . | . | . |
| DPYD ---- tag |  | 2 | 6 | 10,00 | 3 | 9,38 | 22 | 6,45 | 16 | 5,23 | 0.46 (0.12-1.77) | 0.26 | 1.06 (0.58-1.95) | 0.85 | . | . | . |
| DPYD ---- tag | rs1520658 | 0 | 46 | 76,67 | 25 | 78,13 | 280 | 82,11 | 251 | 82,03 | 1.00 (.-.) | . | 1.58 (0.96-2.59) | 0.07 | 0.21 | 0.99 | 0.72 |
| DPYD ---- tag |  | 1 | 14 | 23,33 | 6 | 18,75 | 55 | 16,13 | 52 | 16,99 | 1.68 (0.72-3.91) | 0.23 | 1.45 (0.85-2.50) | 0.18 | . | . | . |
| DPYD ---- tag |  | 2 | 0 | 0,00 | 1 | 3,13 | 6 | 1,76 | 3 | 0,98 | 2.83 (0.52-15.28) | 0.23 | 1.34 (0.65-2.75) | 0.43 | . | . | . |
| DPYD ---- NA | rs17116806 | 0 | 36 | 60,00 | 19 | 59,38 | 228 | 66,86 | 193 | 63,07 | 1.00 (.-.) | . | 1.58 (0.90-2.79) | 0.11 | 0.43 | 0.99 | 0.77 |
| DPYD ---- NA |  | 1 | 23 | 38,33 | 12 | 37,50 | 94 | 27,57 | 101 | 33,01 | 1.39 (0.69-2.77) | 0.36 | 1.64 (0.92-2.92) | 0.10 | . | . | . |
| DPYD ---- NA |  | 2 | 1 | 1,67 | 1 | 3,13 | 19 | 5,57 | 12 | 3,92 | 1.92 (0.48-7.67) | 0.36 | 1.69 (0.87-3.30) | 0.12 | . | . | . |
| DPYD ---- tag | rs17431828 | 0 | 24 | 40,00 | 14 | 43,75 | 131 | 38,42 | 138 | 45,10 | 1.00 (.-.) | . | 1.49 (0.77-2.87) | 0.23 | 0.73 | 0.99 | 0.84 |
| DPYD ---- tag |  | 1 | 28 | 46,67 | 13 | 40,63 | 162 | 47,51 | 133 | 43,46 | 1.08 (0.59-1.96) | 0.81 | 1.44 (0.75-2.75) | 0.28 | . | . | . |
| DPYD ---- tag |  | 2 | 8 | 13,33 | 5 | 15,63 | 48 | 14,08 | 35 | 11,44 | 1.16 (0.35-3.84) | 0.81 | 1.39 (0.69-2.79) | 0.36 | . | . | . |
| DPYD ---- tag | rs17471640 | 0 | 24 | 40,00 | 11 | 34,38 | 141 | 41,35 | 152 | 49,67 | 1.00 (.-.) | . | 1.94 (0.99-3.81) | 0.05 | 0.14 | 0.99 | 0.72 |
| DPYD ---- tag |  | 1 | 29 | 48,33 | 15 | 46,88 | 171 | 50,15 | 126 | 41,18 | 1.41 (0.79-2.50) | 0.24 | 1.72 (0.88-3.35) | 0.11 | . | . | . |
| DPYD ---- tag |  | 2 | 7 | 11,67 | 6 | 18,75 | 29 | 8,50 | 28 | 9,15 | 1.98 (0.63-6.25) | 0.24 | 1.52 (0.73-3.15) | 0.26 | . | . | . |
| DPYD ---- tag | rs17702702 | 0 | 38 | 63,33 | 21 | 65,63 | 235 | 68,91 | 217 | 70,92 | 1.00 (.-.) | . | 1.73 (1.03-2.92) | 0.04 | 0.07 | 0.99 | 0.72 |
| DPYD ---- tag |  | 1 | 19 | 31,67 | 9 | 28,13 | 97 | 28,45 | 78 | 25,49 | 1.67 (0.94-2.98) | 0.08 | 1.58 (0.93-2.68) | 0.09 | . | . | . |
| DPYD ---- tag |  | 2 | 3 | 5,00 | 2 | 6,25 | 9 | 2,64 | 11 | 3,59 | 2.80 (0.88-8.88) | 0.08 | 1.43 (0.75-2.71) | 0.27 | . | . | . |
| DPYD ---- NA | rs1801265 | 0 | 34 | 56,67 | 22 | 68,75 | 197 | 57,77 | 177 | 57,84 | 1.00 (.-.) | . | 1.36 (0.81-2.27) | 0.24 | 0.96 | 1.00 | 0.97 |
| DPYD ---- NA |  | 1 | 23 | 38,33 | 10 | 31,25 | 122 | 35,78 | 111 | 36,27 | 0.99 (0.44-2.22) | 0.98 | 1.38 (0.82-2.30) | 0.22 | . | . | . |
[truncated: 129,268 more chars]
